# Stage-specific coexpression network analysis of Myc in cohorts of renal cancer

**DOI:** 10.1038/s41598-023-38681-x

**Published:** 2023-07-22

**Authors:** Jyotsna Priyam, Urmila Saxena

**Affiliations:** Department of Biotechnology, National Institute of Technology Warangal, Warangal, 506004 Telangana India

**Keywords:** Cancer, Cancer genomics

## Abstract

The present study investigates the molecular dynamics of Myc in normal precursors and in different stages (I/II/III/IV) of cohorts of renal cancer using two distinct yet complementary approaches: gene expression and gene coexpression. We also analysed the variation of coexpression networks of Myc through the stage-wise progression of renal cancer cohorts. Myc expression is significantly higher in stage I compared to normal tissue but changed inconsistently across stages of renal cancer. We identified that Myc consistently coexpressed with fourteen genes in the KIPAN [Pan-kidney cohort (KICH + KIRC + KIRP)] and eight in the KIRC (Kidney renal clear cell carcinoma) across all stages, providing potential prognostic and diagnostic biomarkers. Coexpression network complexity decreased from normal precursor tissues to associated tumour stage I in KIPAN and KIRC but was inconsistent after that. In the process of cancer development, there is generally lower cross-tissue cancer network homology observed among coexpressed genes with Myc during the normal to the stage I compared to the stage-wise progression of cancer. Overall, this research provides novel perceptions of the molecular causes of kidney cancer. It also highlights potential genes and pathways crucial for diagnosing and treating this disease.

## Introduction

Renal cell carcinoma (RCC) refers to a broad range of cancers with distinct histological characteristics originating from renal tubular cells^1^. They exhibit various gene mutations, epigenetic alterations, clinical dynamics, and therapeutic responses^2^. Clear cell renal cell carcinoma (ccRCC), papillary renal cell carcinoma (pRCC) and chromophobe renal cell carcinoma (chrRCC) are the three primary histopathological cohorts of kidney cancer^3^. The distinct functions played by the Myc gene in renal cancer cohorts and its varying expression and gene interactions have been demonstrated in prior research. Understanding kidney cancer causes and potential treatments is aided by the correlation between Myc overexpression, unfavourable clinical outcomes, and immune infiltration^4^. Although Myc's function in RCC is known, minimal studies have focused on its role in stagewise renal cancer progression. Analysing stage-specific progression can provide a more profound knowledge of renal cancer.

Additionally, the dysregulation of numerous genes plays a vital role in the aetiology of cancer. Therefore, it is crucial to investigate Myc-related coexpressed genes, how their dynamics change from progression towards one stage to another and the molecular mechanism by which they may cause RCC. Investigating how Myc functions differently in diverse RCC cohorts is also critical. Thus, a more accurate understanding of the disease could be achieved by knowing how it progresses in terms of molecular and genetic factors.

This work is focused on detecting Myc-interacting partners across different stages of renal cancer. We used TCGA (The cancer genome atlas) data to examine stage-specific Myc expression and coexpression status in two cohorts of renal cancer, KIPAN [Pan-kidney cohort (KICH + KIRC + KIRP)] and KIRC (Kidney renal clear cell carcinoma). We also performed a detailed analysis of subgroups; KICH (Kidney chromophobe) and KIRP (Kidney renal papillary cell carcinoma). Additionally, we examined consistent coexpressed genes with Myc within stages, their diagnostic and prognostic outcome and stage-specific Myc-associated DNA methylation in KIPAN and KIRC. Our findings suggest that Myc and its crucial coexpressed genes could be potential therapeutic targets for improving patient survival and accurate diagnosis in renal cancer cohorts. Likewise, we discovered that Myc behaves differently depending on the stage and type of kidney cancer. Addressing this unsteady behaviour could be crucial in characterising contemporary molecular targets and developing efficient therapeutic approaches.

## Results

### Stage-specific expression analysis of Myc in cohorts of renal cancer

**Figure 1:**
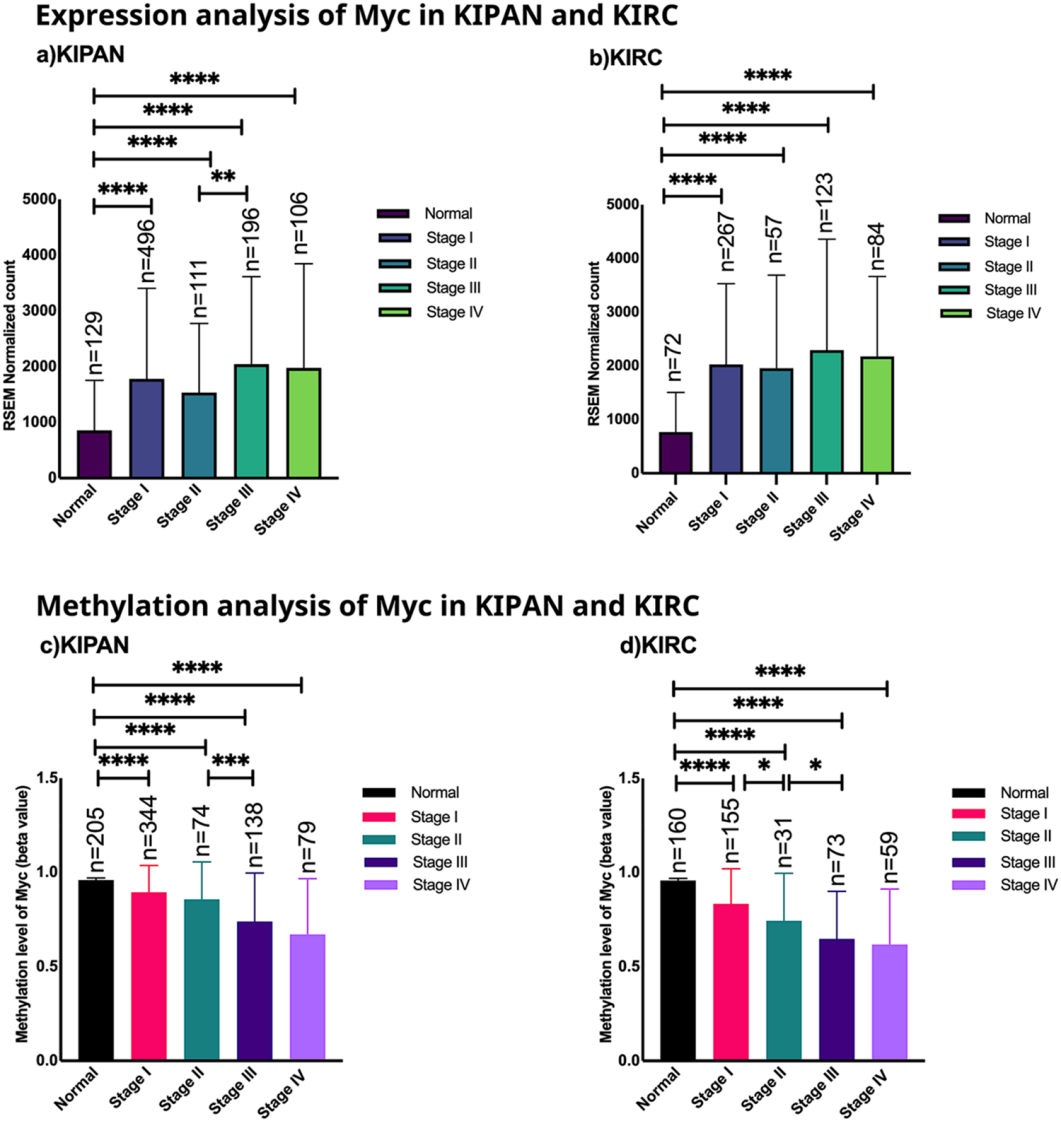
(**a**) Stagewise expression analysis of Myc mRNA in (**a**) KIPAN and (**b**) KIRC patient. The graph's error bars show the study's standard deviation. Promoter methylation analysis of Myc across normal versus stages and among stages in (**c**) KIPAN (KICH + KIRC + KIRP) and (**d**) KIRC. Lower levels of promoter methylation of Myc were found across stages in tumour samples in comparison to normal and among themselves, as indicated in the bar graph (****p < 0.0001, ***p < 0.001, *p < 0.05; Mann–Whitney U test). The Y-axis reflects the level of mRNA expression of Myc (**a**,**b**) and DNA methylation (**c**,**d**), which varies from 0 (unmethylated) to 1 (fully methylated). [*KIPAN* Pan-kidney cohort (KICH + KIRC + KIRP), *KIRC* Kidney renal clear cell carcinoma and *n* number of samples].

Stage-wise transcriptional expression state of Myc is analyzed in KIPAN and KIRC among normal and tumour samples and across different tumour stages. Myc expression was significantly higher in stages I, II, III, and IV in KIPAN and KIRC compared to normal samples (Fig. 1a,b). There was a significant increase in the expression of Myc as cancer progressed from stage II to stage III in KIPAN only. Otherwise, no change in expression value as cancer progresses from stage I to stage II or stage III to stage IV in KIPAN (Fig. 1a). Furthermore, in KIRC, no significant change in the expression level of Myc was found among stages I/II/III/IV (Fig. 1b). Our result reflects that Myc mRNA (Messenger ribonucleic acid) expression significantly increases from normal to the cancerous stage in KIPAN and KIRC. However, there is an inconsistent change in Myc expression as cancer progresses through different stages in both KIPAN and KIRC. In addition, we assessed stage-specific expression analysis of Myc in the datasets KICH and KIRP. Myc expression significantly increased in KIRP with the transition from normal to cancer, although it did not differ between stages (I–IV). In contrast, the KICH dataset's expression analysis reveals a statistically significant increase of Myc in normal compared to tumour tissue (Supplementary file, Fig. S1).

### Promoter methylation analysis of Myc across stages in datasets of renal cancers

In epigenetics, the genome undergoes transient functional modifications unrelated to DNA sequence differences^5^. DNA methylation is a significant epigenetic alteration that affects cellular characteristics^6^. Stage-specific Myc DNA methylation in renal carcinoma revealed decreased promoter methylation in stage I of KIPAN and KIRC compared to normal (Fig. 1c,d). We also found a significant decrease in methylation levels in stages II/III of KIPAN (Fig. 1c) and stages I/II/III of KIRC (Fig. 1d), reflecting variations from established theory. We speculate that hypomethylation of Myc’s promoter may contribute to its upregulation in KIPAN but not KIRC.

Additionally, we also performed promoter methylation analysis of subgroups KICH and KIRP. Myc showed lower promoter methylation in KIRP stages I to IV than normal but no significant variation within stages. These findings persist with the earlier hypothesis that hypomethylation is proportional to gene overexpression^7^. Furthermore, the KICH normal sample data were unavailable, and we did not find statistically significant data for stages I–IV either (Supplementary file, Fig. S2).

### Analysis of common, consistent coexpressed genes with Myc through stages I–IV in KIPAN and KIRC, revealing their diagnostic and prognostic outcomes and assessing Jaccard similarity of Myc-coexpressed genes

Genes work together to carry out intricate biological functions. We analyzed coexpressed genes with Myc in normal and stage-specific samples. Cancer samples from the same stage often have stage-specific coexpressed genes, which execute stage-specific biological functions^8^.

For correlation analysis, stage-specific cancer patient samples from KIPAN and KIRC having higher and lower Myc expression were collected. The correlation coefficient of Myc for several other genes expressed in KIPAN and KIRC is calculated in normal samples and stages I–IV. Table 1 below lists the coexpressed genes with Myc in normal and stage-specific samples from KIPAN and KIRC.

**Table 1:** List of coexpressed genes with Myc in normal and stage-specific samples of KIPAN and KIRC.

	Normal	Stage I	Stage II	Stage III	Stage IV
KIPAN	2674	511	2268	356	465
KIRC	3357	280	1173	475	532

Highly expressed genes tend to be more coexpressed^9^. The number of coexpressed genes associated with Myc is higher in normal KIPAN and KIRC samples than in the tumour stages. Stage I of KIPAN and KIRC reveals a significant decline, followed by a stage II increase, a stage III decrease, and a stage IV increase. We also investigated genes that coexpressed with Myc in normal and stage-specific samples of the renal cancer cohorts KICH and KIRP. Compared to tumour stages, normal samples from KICH and KIRP contain more coexpressed genes with Myc. Stage I sees a drop in coexpression which rises in stage II in both KICH and KIRP. Again stage III has a decline in coexpression in KICH but not in KIRP. Finally, at stage IV, KIRP exhibits an increase, whereas KICH demonstrates a decline in coexpressed genes with Myc (Supplementary file Table S6, S7). These results indicate that Myc coexpression networks are highly altered through different stages of renal cancer.

**Figure 2:**
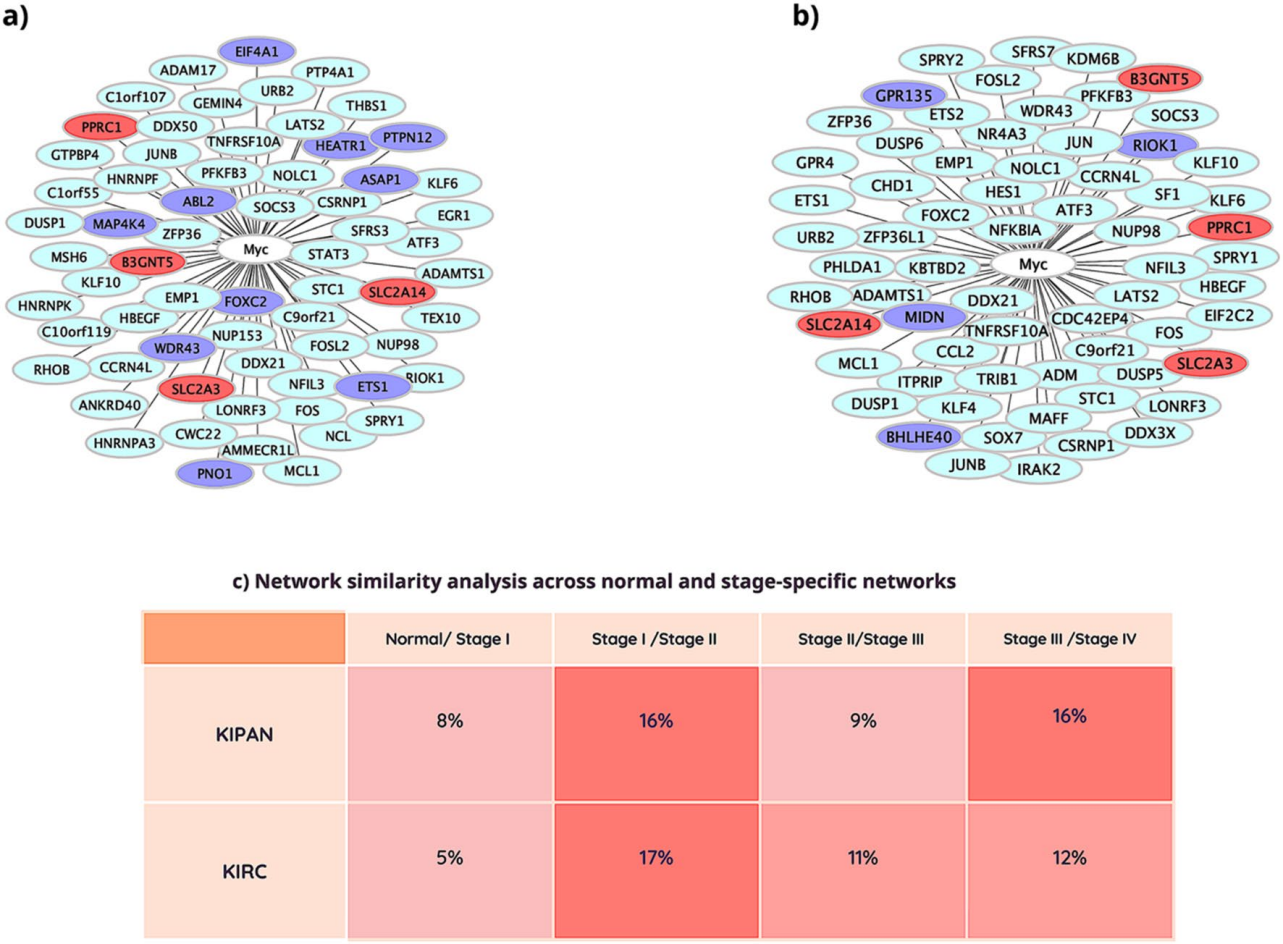
Network of consistent genes across stages with Myc in KIPAN (**a**) and KIRC (**b**). Cyan colour nodes are coexpressed genes with Myc (consistent through stages; r > 0.3 and P < 0.05). Purple colour nodes are consistent coexpressed genes having diagnostic and prognostic importance in KIPAN and KIRC. Orange colour is common, consistent coexpressed genes in KIPAN and KIRC, showing diagnostic and prognostic relevance, and the central white node is Myc (**c**) Cross-tissue Jaccard similarity analysis of coexpressed genes with Myc in KIPAN and KIRC. We compared the stage-wise cancer networks with their associated normal precursor networks. The numbers in the matrix are Jaccard Similarity Index percentage. Orange (dark) is a higher similarity between networks, Orange (in-between) is an intermediate similarity between networks and Orange (light) is a lower similarity between networks.

Furthermore, Jaccard similarity calculates how similar two networks are; when they were created from overlapping, non-identical subsets. A higher Jaccard coefficient value denotes self-consistent networks and vital cluster conservation between two sets of genes^10^. Jaccard similarity analysis was performed to check the similarity between several modules of coexpressed genes with Myc in KIPAN and KIRC (Fig. 2c). The highest similarity was found between stage I/II and stage III/IV of KIPAN and stage I/II of KIRC (Fig. 2c). In addition, stage II/III and stage III/IV of KIRC also display considerable network similarity (greater than 10%; Fig. 2c). Jaccard similarity analysis was carried out for the KICH and KIRP datasets to evaluate the similarity between several modules of coexpressed genes with Myc. Stage I/II of KICH and stage III/IV of KIRP were found to be the most similar. In addition, the normal/stage I of KICH also showed a remarkable network similarity of 10% (Supplementary file Fig. S3).

**Figure 3:**
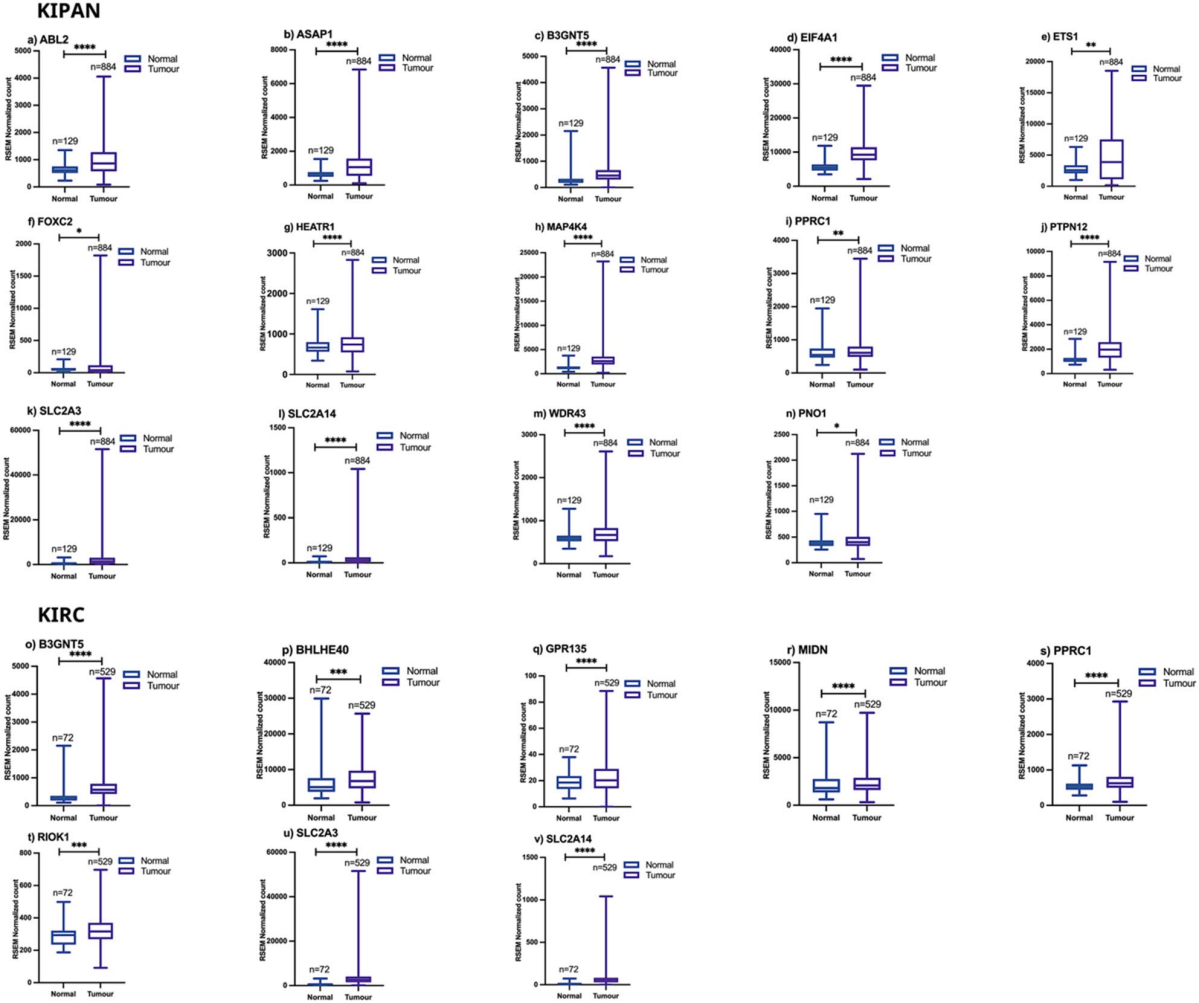
Box plot showing the expression value of Myc mRNA in coexpressed genes with  Myc across stages (I–IV) of (**a**) KIPAN and (**b**) KIRC normal and cancerous tissue of the patient. The graph's error bars display the standard deviation for each study. [*KIPAN* Pan-kidney cohort (KICH + KIRC + KIRP), *KIRC* Kidney renal clear cell carcinoma and *n* number of samples)].

**Figure 4:**
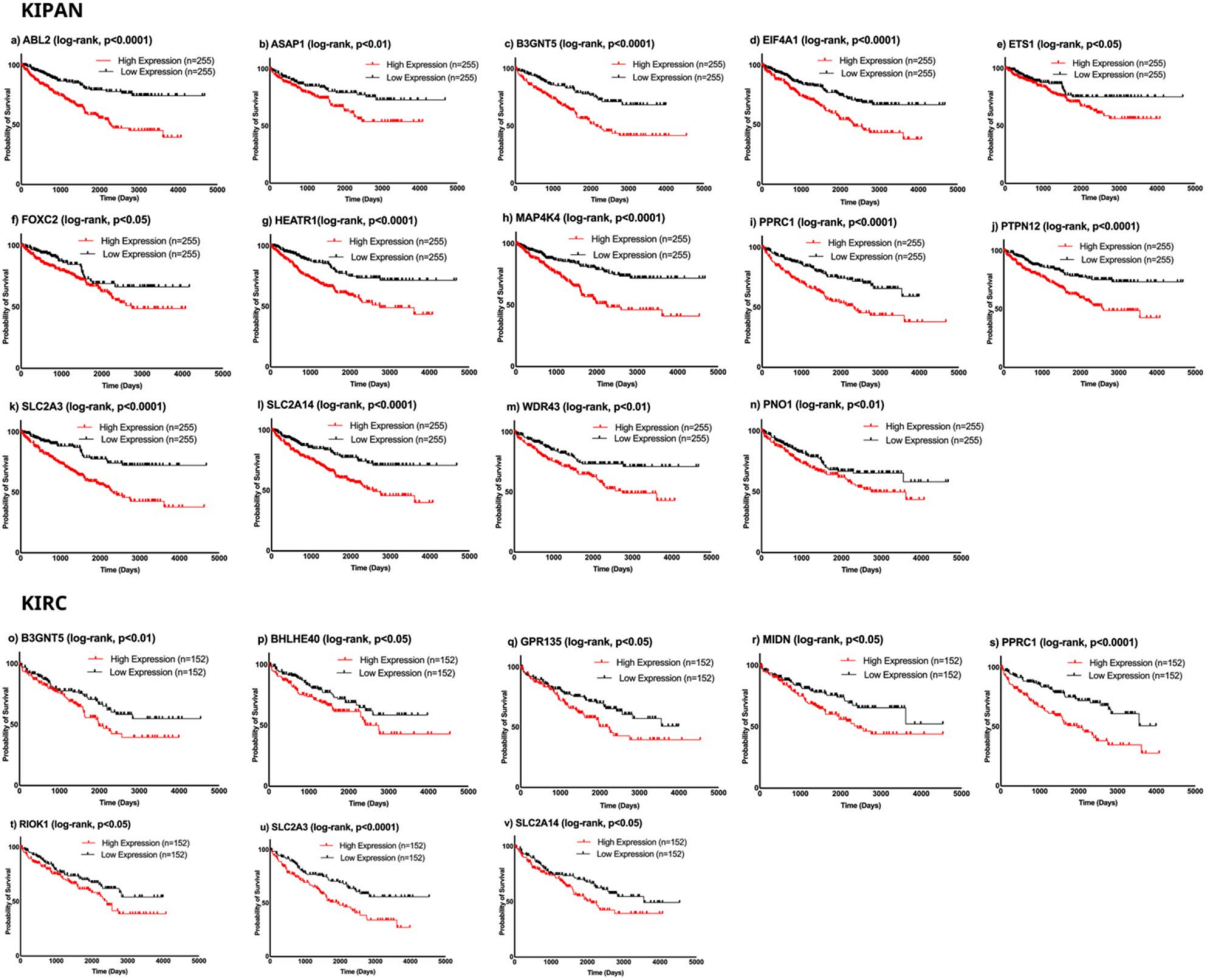
Comparison of the Kaplan–Meier curves for coexpressed genes with Myc having prognostic relevance across stages (I–IV) in KIPAN and KIRC for higher expression (> 75% of expression values) and lower expression (< 25% of expression values). The higher and lower expression categories in KIPAN and KIRC have statistically different overall survival rates, as shown by the Log-rank (Mantel-Cox) test. [*KIPAN* Pan-kidney cohort (KICH + KIRC + KIRP), *KIRC* Kidney renal clear cell carcinoma and *n* number of samples].

Of all coexpressed genes with Myc (Table 1), 65 genes in KIPAN and 66 genes in KIRC are consistently coexpressed with Myc across all stages of cancer (Fig. 2a,b). Expression and survival analyses of these consistent coexpressed genes with Myc in KIPAN and KIRC were conducted to select the genes with high diagnostic and prognostic value. Fourteen genes in KIPAN and eight genes in KIRC (Figs. 3 and 4) are overexpressed in tumour samples relative to normal samples. Also, their increased expression is related to the poor survival of cancer patients. In the subgroup datasets KICH and KIRP also, we examined the consistency of genes that coexpress with Myc throughout stages (Supplementary file Table S1). Three genes in KICH and nineteen in KIRP consistently show coexpression across all stages with Myc. Expression and survival studies discovered two overexpressed genes in KIRP with poor prognosis (Supplementary file Fig. S4), while no genes in KICH matched these criteria. The supplementary file Table S2 also lists the clinical and pathological characteristics of the patients in the KIPAN, KIRC, and subgroups (KICH, KIRP).

### Regardless of expression, gene loss or gain causes complexity changes in renal cancer networks and enrichment analysis of lost, conserved, acquired genes in renal cancer cohorts

**Figure 5:**
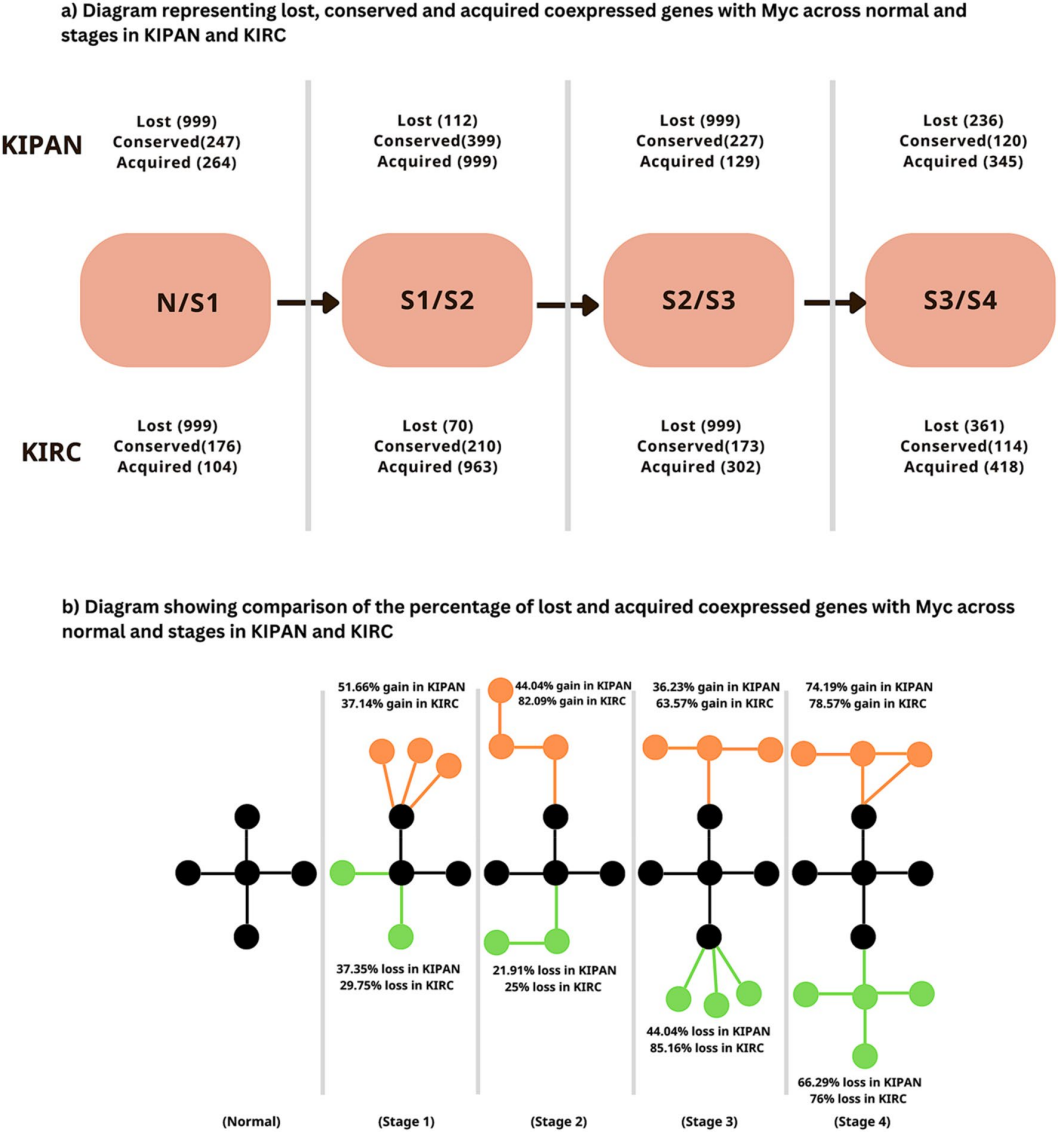
(**a**) Illustration representing coexpressed genes with Myc throughout normal and stages in KIPAN and KIRC: lost, conserved, and acquired; *N* Normal, *S1* Stage I, *S2* Stage II, *S3* Stage III, *S4* Stage IV and (**b**) Relation of the percentage of lost, conserved, and acquired genes along with Myc in KIPAN and KIRC all through normal and stages advancement; black colour represents conserved genes, green colour denotes lost genes and orange colour depicts acquired genes, [*KIPAN* Pan-kidney cohort (KICH + KIRC + KIRP), *KIRC* Kidney renal clear cell carcinoma and *n* number of samples].

According to published research, most changes in how genes interact in cancer networks are triggered by the loss or addition of a node^11^. We compared the gene coexpression of Myc in normal samples and different stages of KIPAN and KIRC. Figure 5a shows the changes in several coexpressed genes with Myc as cancer advances stagewise in KIPAN and KIRC. We found that maximum loss (66.29%) and gain (74.19%) of genes occurred during stages III/IV in KIPAN. We also found maximum loss during stage II/III (85.16%) and gain during stage I/II (82.09%) in KIRC. Our result validates that the comparative degree of network connection loss and gain differed between cancer types out of all coexpressed genes with Myc. Figure 5b shows the relative degree of network loss and gain in KIPAN and KIRC.

Interestingly, there is still a 16% similarity in the network for KIPAN (stage III/IV), despite the highest percentage of lost and gained nodes. Similarly, in KIRC (stage I/II), after acquiring the highest number of nodes, the network has a 17% similarity. Furthermore, the subgroup (KICH and KIRP) analysis demonstrates the highest rate of gene loss (97.25%) and gain (92.54%) in stages III/IV of KICH. In addition, we found that the highest gene gain (91.25%) was recorded during stage I/II in KIRP. In contrast, the most significant gene loss (97.82%) was reported during the conversion from normal to stage I (Supplementary file, Table S3). An intriguing finding is that there is still a 1.9% network similarity despite the highest node loss and gain in KICH (stage III/IV). In KIRP (stage I/II), despite acquiring the highest number of nodes, there is a 6.7% network similarity and a 1.8% network similarity after the most significant percentage of gene loss (normal/stage I). In conclusion, despite dynamic changes, consistent underlying similarities point to regulatory or functional connections across cancer stages.

Analysis of biological pathways and processes using specified gene sets that take changes in connectivity and gene expression into account is an effort to understand better the molecular basis of disease aetiology^12^. Thus, functional enrichment analysis of lost, conserved and acquired genes, coexpressing with Myc, was done to check their contribution to the pathogenesis of the disease. The highest enriched pathway for lost, conserved, and acquired genes coexpressed with Myc in KIPAN and KIRC was "signalling by interleukins". Interleukins are essential for controlling immunological responses, particularly inflammatory responses^13^.

**Figure 6:**
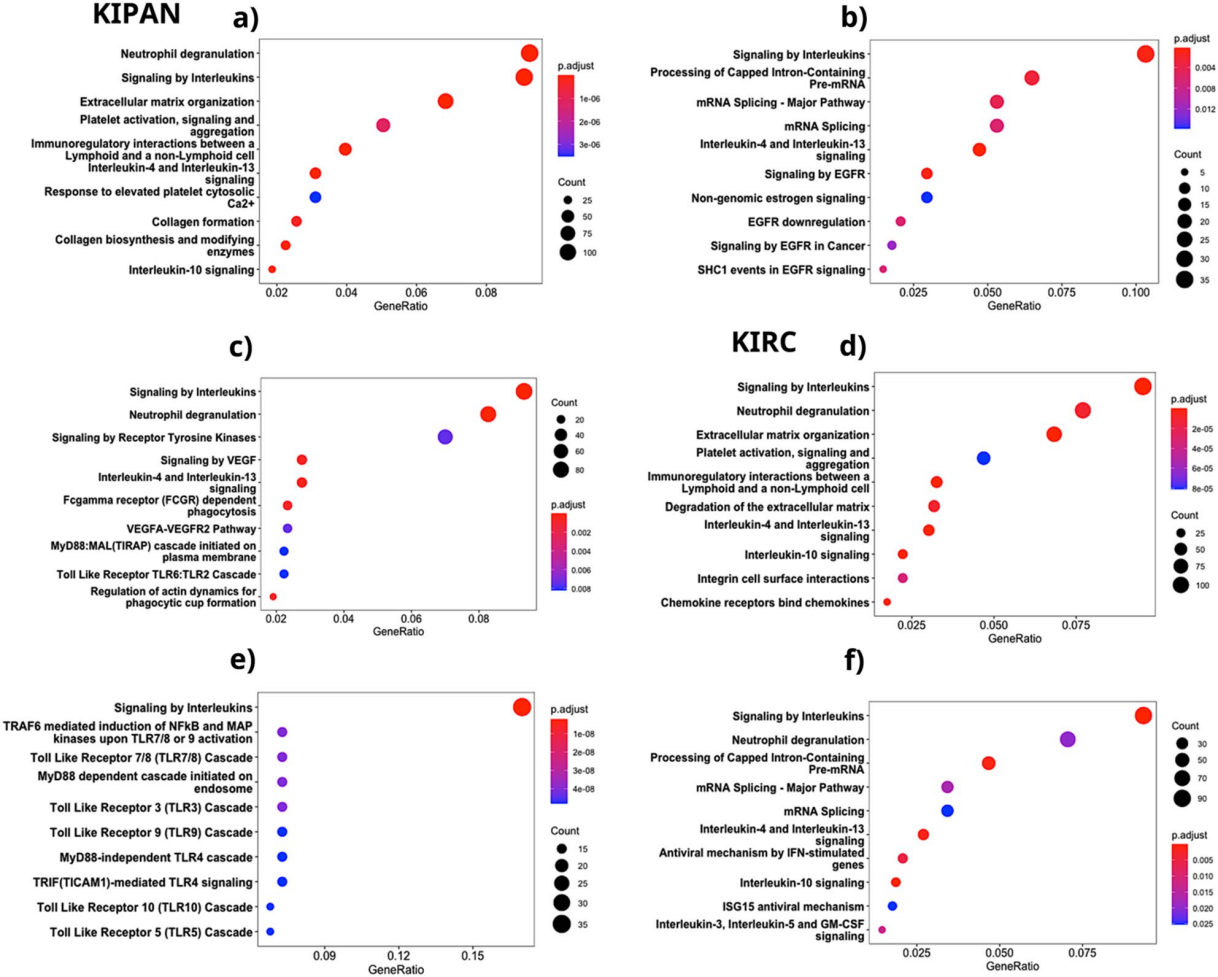
Functional enrichment analysis of lost (**a**), conserved (**b**) and acquired (**c**) genes; in KIPAN and lost (**d**), conserved (**e**) and acquired genes (**f**); in KIRC. A dot plot graph displays pathways associated with genes. The X-axis shows the gene ratio, and the Y-axis represents the pathway's name. P < 0.05 is the threshold for a significant P value. The P value is indicated by colour, whereas the dot size indicates the number of genes.

Additionally, our research found that the interleukin-4 and interleukin-13 signalling pathways, which affect immune cell activity and are related to inflammation and kidney cancer, are enriched^14,15^. Other noteworthy pathways in our research included EGFR (Epidermal growth factor receptor) signalling, which is linked to the advancement of kidney cancer^16^, and neutrophil degranulation, which is implicated in innate immunity^17,18^. In cohorts KICH and KIRP, immunoregulatory interactions between lymphoid and non-lymphoid cells and extracellular matrix organisation were also enriched pathways (Supplementary file Fig. S5). Figure 6 shows a schematic representation of the top ten enriched pathways in the lost, conserved, and acquired gene lists in KIPAN and KIRC. Our findings demonstrate that coexpressed genes with Myc are crucial in renal cancer development in both KIPAN and KIRC. It confirms our theory that Myc's influence on the development of renal carcinoma is influenced by its interactions with coexpressed genes, despite uneven stage-specific expression.

### Differential expression analysis of acquired genes in stage I of renal cancer datasets

We also conducted an expression analysis of all acquired genes (coexpressed genes with Myc) in stage I of KIPAN and KIRC because gene interactions rather than individual gene expression play a vital role in explaining the mechanisms underlying kidney cancer progression. Out of 264 acquired genes in stage I of KIPAN, only 125 (47.34%) were differentially expressed and showed significantly higher expression in tumours than normal. A list of differentially expressed genes is given in the supplementary file, Fig. S6. Similarly, among 104 acquired genes in stage I of KIRC, only 40 (38.46%) were differentially expressed and showed significantly higher expression in tumours than normal (Supplementary file; Fig. S7). Additionally, only 77 (8.86%) of the 869 and 84 (19.95%) of 421 acquired genes showed significantly higher expression in stage I of KICH and KIRP, respectively (Supplementary file Figs. S8 and S9).

## Discussion

In this study, we hypothesised that the connectivity of coexpressed genes with Myc in normal and stage-specific cancer networks could identify driver genes in cohorts of renal cancer. We observed considerably greater Myc expression in stage I KIPAN and KIRC, with no following stage-wise increases, which is consistent with earlier research on the renal cell carcinoma biomarkers DCLK1 (Doublecortin-like kinase1) and SAA1 (Serum Amyloid A1)^19,20^. The stage-specific methylation state of Myc in cohorts of renal cancer reveals that promoter hypomethylation is related to increased Myc expression in KIPAN only.

To better understand the pathology of renal cancer, we examined coexpressed genes with Myc in KIPAN and KIRC across normal and stage-specific samples. Coexpressed genes with Myc, in terms of number, are nearly five and twelve times higher in normal than stage I of KIPAN and KIRC, respectively. This reveals that more physiological pathways are involved in normal precursor cells than in stage I of renal cancer. Interestingly, there was a noticeable decrease in coexpressed genes from normal to the stage I and an inconsistent pattern across other stages in KIPAN and KIRC. At the same time, more substantial network similarity is created between the genes within stages compared to a healthy state. Our findings reveal that overall, there is heterogeneity in the number of coexpressed genes throughout stages, and Myc coexpression networks substantially alter in kidney cancer cohorts. These results give insight into the relevance of potential coexpressed genes associated with Myc in diagnosing renal cancer.

We also assessed the diagnostic and prognostic role of consistent and common, consistent coexpressed genes with Myc across stages in KIPAN and KIRC. We identified SLC2A3 (Solute carrier family 2 member 3), SLC2A14 (Solute carrier family 2 members 14), B3GNT5 (Beta-1,3-N-acetylglucosaminyltransferase 5), and PPRC1 [Peroxisome proliferator-activated receptor γ (PPARG) related coactivator 1] are common persistent coexpressed genes with Myc (Fig. 2a,b). They may act as a linking factor, possible predictive biomarkers, and therapeutic targets for KIPAN and KIRC. We hypothesise that the highlighted genes aid in the search for new drugs to treat kidney cancer. Regardless of the stage-specific expression of Myc, up-regulation of SLC2A3^21^, PPRC1, EIF4A1(Eukaryotic translation initiation factor 4A1)^22^, and ETS1 (ETS proto-oncogene 1, transcription factor)^23^ is associated with advanced renal carcinoma.

Furthermore, a succession of RCC is correlated with HIF (Hypoxia-inducible transcription factor), NICI (Non-coding intergenic co-induced transcript), and SLC2A3^24–26^. Since HIF2 (an isoform of HIFs) promotes the activity of c-Myc on target genes to increase cell cycle development in ccRCC^27,28^. Hence, it validates that Myc is part of the HIF/NICI/SLC2A3 paradigm and related to the PPARC1 gene, whose up-regulation cause RCC. Our study validates that PPRC1, SLC2A3, SLC2A14 and B3GNT5 should be considered connecting links between KIPAN and KIRC.

Under 50% of the genes in stage I of KIPAN and KIRC showed significantly higher expression in tumours, according to our differential gene expression analysis. Myc coexpression networks changed as cancer advanced, with losses and gains in KIPAN (37.35% loss, 51.66% gain) and KIRC (29.75% loss, 37.14% gain). Overall, network complexity was highest in advanced stages in both cohorts of renal cancer, which is consistent with earlier studies^29^. Between normal/stage I and stage I/ II, the network similarity ratio increased by a factor of two (KIPAN) and more than three (KIRC). Compared to healthy precursor cells, cancer cells relapse to undifferentiated states with more progenitor markers and fewer differentiation markers^30,31^.

Our stage-specific analyses, including KIPAN stage III/IV, demonstrate an overall increase in cross-tissue network similarity of 7%. However, there are differences in the patterns of network similarity, which decrease in KIPAN stage II/III and KIRC stages II/III and III/IV. Previous research has emphasised the various network topologies and gene participation in cancer-related pathways^32,33^. The development of kidney cancer is influenced by genes preserved in both normal and tumour networks via gene-network interactions.

Our findings support a considerable enrichment of pathways involved in immunological responses, such as EGFR signalling and interleukin-4 and interleukin-13 signalling. JUNB (JunB proto-oncogene, AP-1 transcription factor subunit), MCL1 (MCL1 apoptosis regulator, BCL2 family member), SOCS3 (Suppressor of cytokine signalling 3), CCL2 (C–C motif chemokine ligand 2), ICAM1 (Intercellular adhesion molecule 1), LIF (LIF interleukin 6 family cytokine), STAT3 (Signal transducer and activator of transcription 3), VIM (Vimentin), FOS (Fos proto-oncogene, AP-1 transcription factor subunit), IL6R (Interleukin 6 receptor), VEGFA (Vascular endothelial growth factor A), S1PR1 (Sphingosine-1-phosphate receptor 1), ZEB1 (Zinc finger E-box binding homeobox 1), CEBPD (CCAAT enhancer binding protein delta), BCL6 (B-cell lymphoma 6), IL1B (Interleukin 1 beta), and MyD88 (Myeloid differentiation primary response 88) are important genes associated with these pathways. JUNB regulates the genes involved in immunological responses^34^, and MyD88 is a critical signal transducer^35^. These findings point to possible kidney cancer treatment targets. Prior research links genes involved in enriched pathways to prognosis and Myc's function in renal cancer, demonstrating the relevance of these genes on the growth of the disease and their potential as therapeutic targets^36^.

The combined analysis of KICH, KIRC, and KIRP as KIPAN enables a thorough comprehension of shared biological mechanisms in kidney cancer. Besides, common, consistent coexpressed genes, which could be a connecting link between different cohorts of renal cancer, were only found when the larger KIPAN and KIRC datasets were compared, not when the individual KICH, KIRC, and KIRP subgroups were examined. While the present study is limited in identifying the various genetic and epigenetic aspects impacting disease progression and the inability to monitor long-term changes within specific patients, it opens up opportunities for further investigation to help us understand these issues better and overcome these challenges.

## Materials and methods

### RNA-seq data extraction and expression analysis of Myc in cohorts of renal cancer

**Figure 7:**
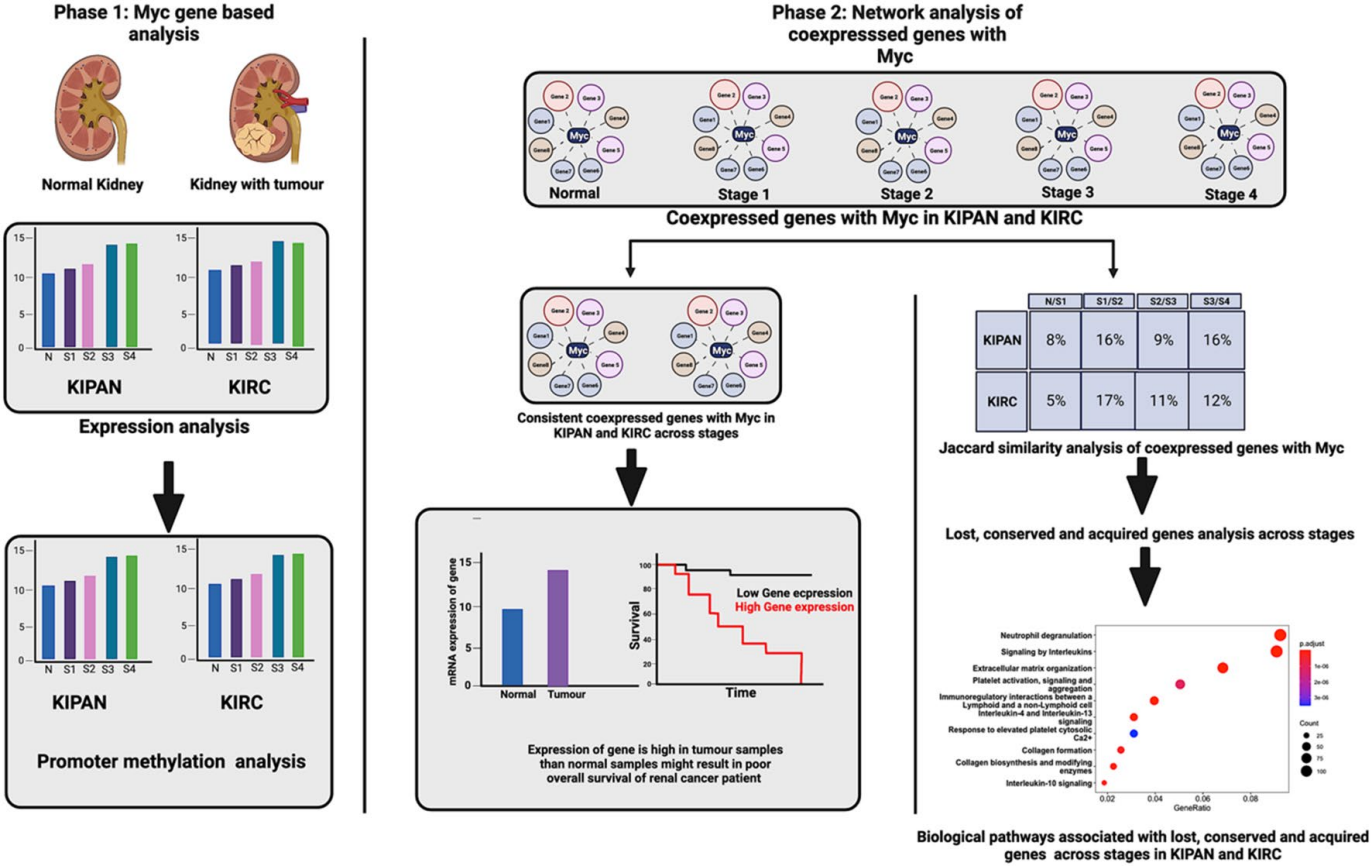
An illustration of the computational workflow used in this paper. Left is Myc gene-based analysis, and right is network analysis. *N* Normal, *S1* Stage I, *S2* Stage II, *S3* Stage III, *S4* Stage IV; [*KIPAN* Pan-kidney cohort (KICH + KIRC + KIRP), *KIRC* Kidney renal clear cell carcinoma and *n* number of samples].

We collected patient-specific RNA sequencing (TCGA) data of renal cancer datasets from Broad GDAC Firehose (http://gdac.broadinstitute.org) 'Illumina hiseq RNase-qv2-level 3 RSEM genes normalized' data used for getting expression value of Myc and gdac.broad institute.org_KIPAN.Clinical_Pick_Tier1.Level 4 folder data provide information about the stage-specific categorisation of patient samples (link available in Table 2). Combining these two data sources provides information about normal and stage-specific renal cancer patient samples and their respective expression values of Myc. The non-parametric Mann–Whitney test was used to examine the differential expression of Myc across normal and all four stages of tumour samples (stages I/II/III/IV) and within individual stages. P < 0.05 is the threshold selected for the significant difference in the expression. For statistical analysis, we applied GraphPad Prism 9.1.1. The overall procedure is shown in Fig. 7.

**Table 2:** Links for the accession of gene expression, DNA methylation and corresponding clinical data of datasets KIPAN, KIRC, KICH and KIRP from Broad GDAC Firehose, [*KIPAN* Pan-kidney cohort (KICH + KIRC + KIRP), *KIRC* Kidney Renal Clear Cell Carcinoma, *KICH* Kidney chromophobe, *KIRP* Kidney renal papillary cell carcinoma, *RSEM* RNA-Seq by Expectation Maximization].

	Gene expression data	DNA methylation data	Clinical data
KIPAN	https://gdac.broadinstitute.org/runs/stddata__2016_01_28/data/KIPAN/20160128/gdac.broadinstitute.org_KIPAN.Merge_rnaseqv2__illuminahiseq_rnaseqv2__unc_edu__Level_3__RSEM_genes_normalized__data.Level_3.2016012800.0.0.tar.gz	https://gdac.broadinstitute.org/runs/stddata__2016_01_28/data/KIPAN/20160128/gdac.broadinstitute.org_KIPAN.Methylation_Preprocess.Level_3.2016012800.0.0.tar.gz	https://gdac.broadinstitute.org/runs/stddata__2016_01_28/data/KIPAN/20160128/gdac.broadinstitute.org_KIPAN.Clinical_Pick_Tier1.Level_4.2016012800.0.0.tar.gz
KIRC	https://gdac.broadinstitute.org/runs/stddata__2016_01_28/data/KIRC/20160128/gdac.broadinstitute.org_KIRC.Merge_rnaseqv2__illuminahiseq_rnaseqv2__unc_edu__Level_3__RSEM_genes_normalized__data.Level_3.2016012800.0.0.tar.gz	https://gdac.broadinstitute.org/runs/stddata__2016_01_28/data/KIRC/20160128/gdac.broadinstitute.org_KIRC.Methylation_Preprocess.Level_3.2016012800.0.0.tar.gz	https://gdac.broadinstitute.org/runs/stddata__2016_01_28/data/KIRC/20160128/gdac.broadinstitute.org_KIRC.Clinical_Pick_Tier1.Level_4.2016012800.0.0.tar.gz
KICH	https://gdac.broadinstitute.org/runs/stddata__2016_01_28/data/KICH/20160128/gdac.broadinstitute.org_KICH.Merge_rnaseqv2__illuminahiseq_rnaseqv2__unc_edu__Level_3__RSEM_genes_normalized__data.Level_3.2016012800.0.0.tar.gz	https://gdac.broadinstitute.org/runs/stddata__2016_01_28/data/KICH/20160128/gdac.broadinstitute.org_KICH.Methylation_Preprocess.Level_3.2016012800.0.0.tar.gz	https://gdac.broadinstitute.org/runs/stddata__2016_01_28/data/KICH/20160128/gdac.broadinstitute.org_KICH.Clinical_Pick_Tier1.Level_4.2016012800.0.0.tar.gz
KIRP	https://gdac.broadinstitute.org/runs/stddata__2016_01_28/data/KIRP/20160128/gdac.broadinstitute.org_KIRP.Merge_rnaseqv2__illuminahiseq_rnaseqv2__unc_edu__Level_3__RSEM_genes_normalized__data.Level_3.2016012800.0.0.tar.gz	https://gdac.broadinstitute.org/runs/stddata__2016_01_28/data/KIRP/20160128/gdac.broadinstitute.org_KIRP.Methylation_Preprocess.Level_3.2016012800.0.0.tar.gz	https://gdac.broadinstitute.org/runs/stddata__2016_01_28/data/KIRP/20160128/gdac.broadinstitute.org_KIRP.Clinical_Pick_Tier1.Level_4.2016012800.0.0.tar.gz

### Analysis of promoter methylation

The Broad Institute GDAC Firehose (http://gdac.broadinstitute.org) gathers gene methylation data. Meth.by_min_expr_corr.data is used for methylation study (link available in Table 2), and data is divided into normal and stages I/II/III/IV. As revealed by DNA methylation data, the proportion of methylated to unmethylated allele intensities is the β-value. The non-parametric Mann–Whitney *U* test was used with GraphPad Prism 9.1.1 to evaluate the promoter methylation rates among the normal and stagewise tumour groups (stages I, II, III, and IV). P < 0.05 was the threshold for statistically significant methylation levels (β values).

### Assessment of genes coexpressed with Myc in renal cancer datasets

In the renal cancer datasets, we employed Spearman's rank correlation coefficient to evaluate the level of coexpression among Myc and other genes. We used pre-processed datasets for coexpression analysis, and the correlation coefficient (rs) and corresponding P-value were estimated in R (Version 3.6.3) using the Hmisc package. In addition, we also applied the packages 'psych' and 'dplyr'. Genes are considered to be significantly coexpressed with Myc if their correlation coefficient (rs) is between 0.3 and 1 and P < 0.05. Further, data were divided into normal/stage I/II/III/IV using default packages in R. Coexpressed genes associated with Myc were identified in normal and stage-specific samples, as reported in supplementary file Tables S4–S7.

### Expression analysis of coexpressed genes with Myc in renal cancers

We collected transcriptional expression level data of coexpressed genes with Myc from 'Illumina hiseq RNase-qv2-level 3 RSEM genes normalized' from Broad GDAC Firehose (link available in Table 2). We used this data for expression analysis of consistent coexpressed genes with Myc in KIPAN and KIRC among normal and tumour patient samples. The non-parametric Mann–Whitney test was employed to assess the expression of Myc in normal and tumour samples and P < 0.05 was the cutoff for a significant difference in the expression. For statistical analysis, we utilised GraphPad Prism 9.1.1.

### Survival analysis of consistent coexpressed genes with Myc across stages in cohorts of renal cancers

For survival analysis, gdac.broadinstitute.org_KIPAN.Clinical_Pick_Tier1.Level_4 and gdac.broadinstitute.org_KIRC.Clinical_Pick_Tier1.Level_4, data of KIPAN and KIRC datasets, respectively, obtained from Broad GDAC Firehose (link available in Table 2). We separated patient samples into two groups based on the gene's mRNA expression values in the KIPAN and KIRC datasets. Similarly, we performed survival analysis of consistent coexpressed genes with Myc in subgroup KIRP, also using the same procedure (link available in Table 2). Patient samples with expression values greater than 75% were categorised as having higher expression, whereas samples with expression values lower than 25% were categorised as having lower expression. Moreover, the Kaplan–Meier algorithm and Log-rank testing examine the impact of low and high gene expressions on a patient's overall survival^37^. We used GraphPad Prism 9.1.1 application and R programming to create and analyse survival curves using R packages survminer, survival and dplyr.

### Jaccard similarity analysis and lost, conserved, acquired genes analysis in datasets of renal cancers

The Jaccard index is the intersection of two gene modules divided by the size of their union^38^. Jaccard similarity coefficient calculated using the formula J(A, B) = |A ∩ B|/|A ∪ B|; Where; J = Jaccard distance, A = Genes set1, and B = Genes set 2, using dist() function in R. Coexpressed genes directly associated with Myc selected in normal and stages I–IV of KIPAN and KIRC, as well as subgroups, KICH and KIRP, for Jaccard similarity analysis to check network similarity between them. We transformed gene symbols to Entrez ID (Identifier) using org.Hs.eg.db and default packages in R. Jaccard similarity coefficient analysis in four phases: normal/stage I; stage I/II; stage II/III, and stage III/IV. Set diff and intersect functions in R were used to identify lost, conserved, and acquired genes associated with Myc.

### Biological pathway enrichment analysis

Pathway enrichment analysis was conducted using R programming and various packages to find the metabolic pathways and cellular components connected to the gene list. From KIPAN and KIRC datasets, including subgroup analysis of KICH and KIRP, lost, conserved, and acquired coexpressed genes with Myc were chosen in normal and stage-specific cancer (Supplementary file Tables S8–S11). ClusterProfiler, enrichplot, org.Hs.eg.db, ReactomePA, and stringr packages were used for the enrichment analysis. Stringr package was used to wrap text in the plot labels. We used dot plots and R scripts to visualise the enrichment process. The p-value cutoff of 0.05 was used in this study. Using the Benjamini–Hochberg method (pAdjustMethod = "BH") for multiple testing correction, the q-value, which stands for the false discovery rate (FDR), was regulated^39–41^.

## Supplementary Information

Supplementary Information.

## Data Availability

RNA sequencing data from the Cancer Genome Atlas (TCGA) was extracted from Broad GDAC Firehose (http://gdac.broadinstitute.org/). Renal cancer datasets [KIPAN and KIRC] were used in the current study.
