# Supplementary material for: Stage-specific coexpression network analysis of Myc in cohorts of renal cancer
Source: Sci Rep. 2023 Jul 22;13:11848. doi: 10.1038/s41598-023-38681-x (PMC10363146; doi:10.1038/s41598-023-38681-x)
Supplement: Supplementary file 1 — Supplementary Information. [file 41598_2023_38681_MOESM1_ESM.pdf]

Stage-Specific Co-expression Network Analysis of Myc in Cohorts of Renal Cancer

Jyotsna Priyam, Urmila Saxena*

Department of Biotechnology, National Institute of Technology Warangal, Warangal 506004, Telangana, India

***Corresponding author:**

Dr Urmila Saxena,

Associate Professor,

Department of Biotechnology, National Institute of Technology Warangal India

E-mail addresses: urmila@nitw.ac.in, Phone: 8332969442

Figure S1: Expression analysis of Myc in KICH and KIRP [(a) Stagewise expression analysis of Myc mRNA in a) KICH and b) KIRP patients. The graph's error bars show the study's standard deviation. The Y-axis reflects Myc's mRNA expression level (1a,1b). (****$p < 0.0001$, ***$p < 0.001$, *$p < 0.05$; Mann-Whitney U test)). (KICH= Kidney chromophobe, KIRP= Kidney renal papillary cell carcinoma and n=number of samples)]

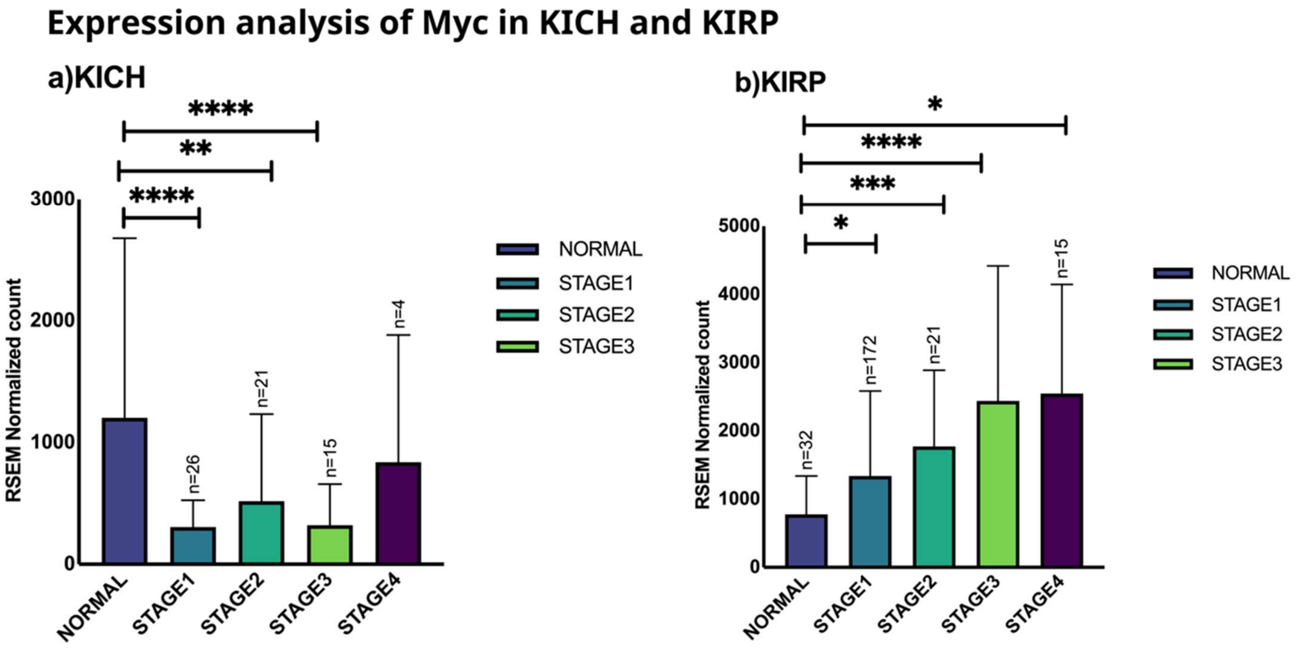

Figure S2: Methylation analysis of Myc in KICH and KIRP [Promoter methylation analysis of Myc across normal versus stages and among stages in c) KICH and d) KIRP. Lower levels of promoter methylation of Myc were found across stages in tumour samples in comparison to normal and among themselves, as indicated in the bar graph (****$p < 0.0001$, ***$p < 0.001$, *$p < 0.05$; Mann-Whitney U test). The Y-axis reflects the DNA methylation (2a,2b), which varies from 0 (unmethylated) to 1 (fully methylated). (KICH= Kidney chromophobe, KIRP= Kidney renal papillary cell carcinoma and n=number of samples)]

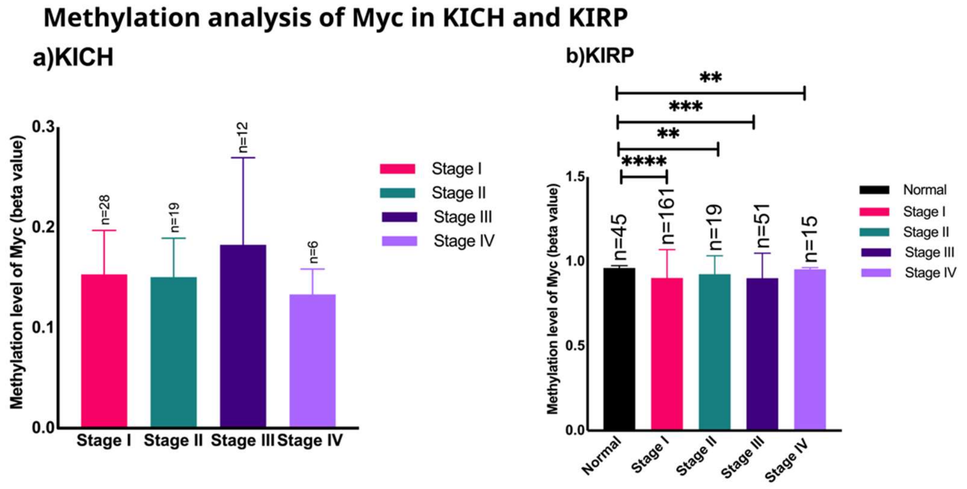

Figure S3: Network similarity analysis of KICH and KIRP **[**Cross-tissue Jaccard similarity analysis of coexpressed genes with Myc in KICH and KIRP. We compared the stage-wise cancer networks with their associated normal precursor networks. The numbers in the matrix are Jaccard Similarity Index percentage (KICH= Kidney chromophobe, KIRP= Kidney renal papillary cell carcinoma and n=number of samples)]

a) Network similarity analysis across normal and stage-specific networks

	Normal/ Stage I	Stage I /Stage II	Stage II/Stage III	Stage III /Stage IV
KICH	10%	13%	7%	1.9%
KIRP	1.8%	6.7%	6.7%	12.8%

Figure S4: Expression and survival analysis of consistent coexpressed genes in KIRP **[**Bar graph showing the expression value of RAN (4a) and KPNB1 (4b) mRNA in coexpressed genes with Myc across stages (I-IV) of normal and cancerous tissue of the KIRP patients. The graph's error bars display the standard deviation for each study. Comparison of the Kaplan-Meier curves for RAN (4c) and KPNB1 (4d) having prognostic relevance across stages (I-IV) in KIRP for higher expression (>75% of expression values) and lower expression (< 25% of expression values). The higher and lower expression categories in KIRP have statistically different overall survival rates, as shown by the Log-rank (Mantel-Cox) test (KIRP= Kidney renal papillary cell carcinoma and n=number of samples)]

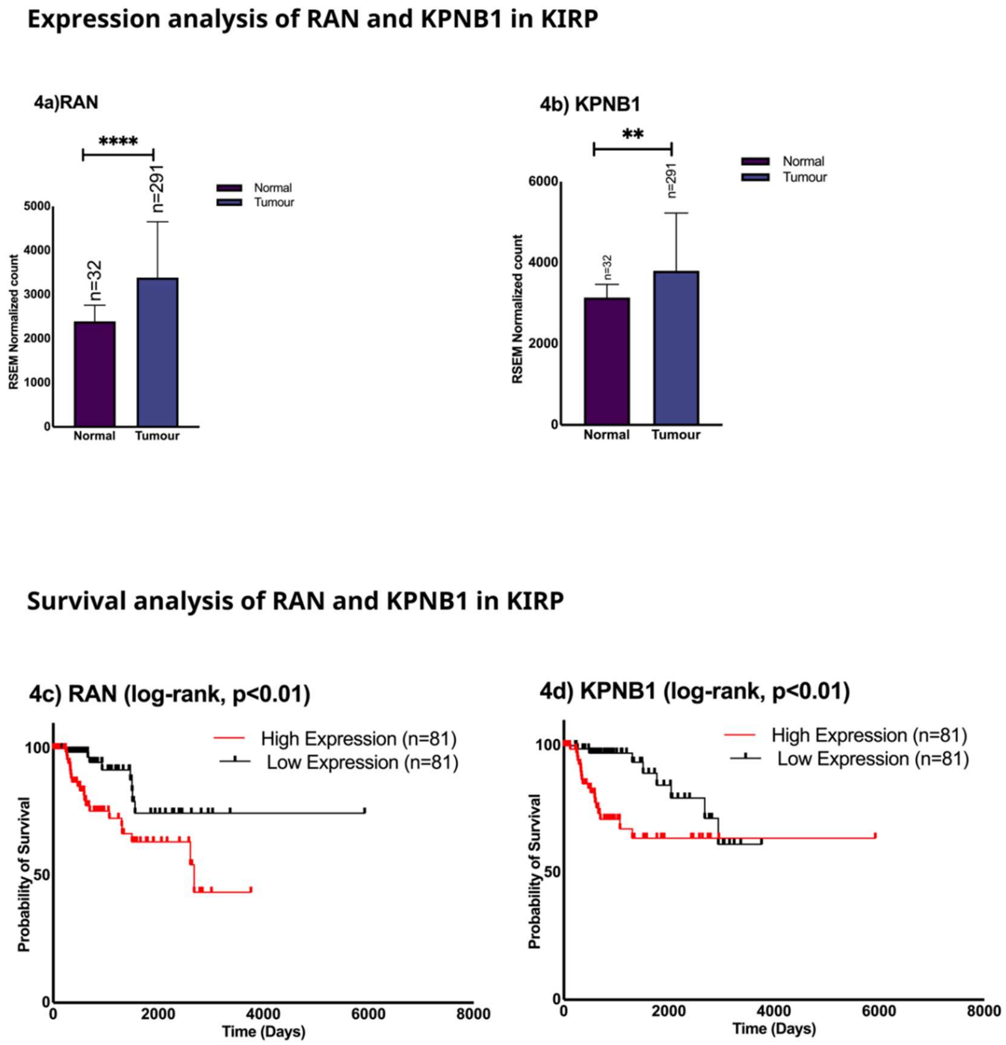

Figure S5: Functional enrichment analysis of lost (a), conserved (b) and acquired (c) genes; in KICH and lost (d), conserved (e) and acquired genes (f); in KIRP. A dot plot graph displays pathways associated with genes. The X-axis shows the gene ratio, and the Y-axis represents the pathway's name. P<0:05 is the threshold for a significant P value. The P value is indicated by colour, whereas the dot size indicates the number of genes. [KICH= Kidney chromophobe, KIRP= Kidney renal papillary cell carcinoma and n=number of samples]

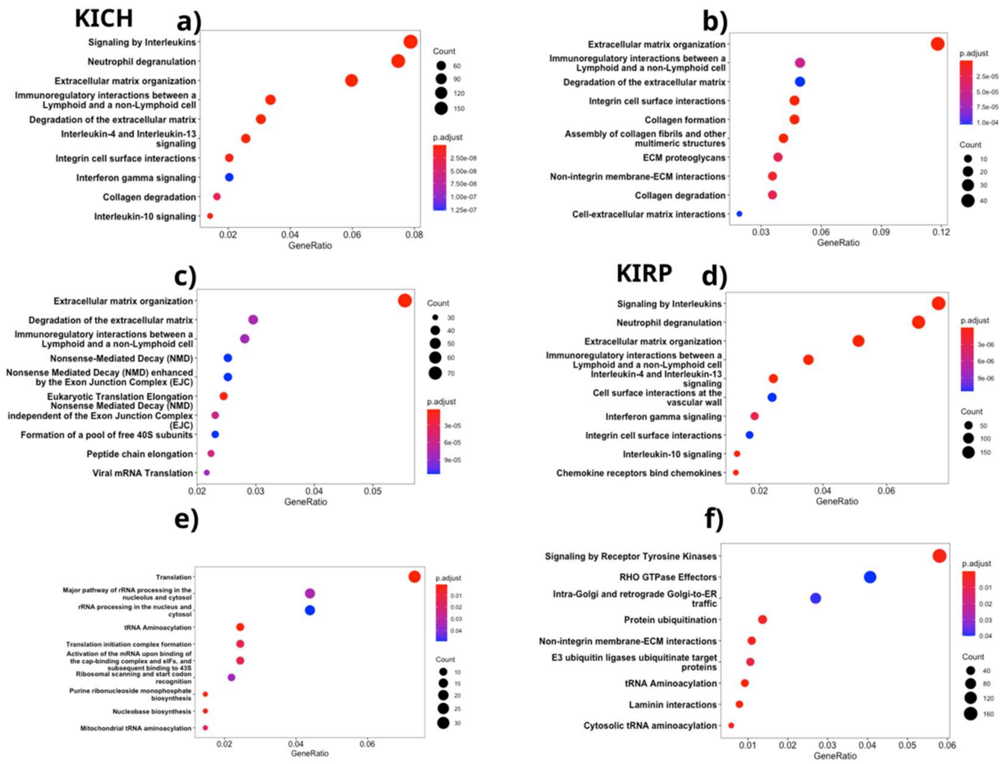

Table S1: Consistent coexpressed genes with Myc in datasets KIPAN, KIRC, KICH and KIRP, [KIPAN= Pan-kidney cohort (KICH+KIRC+KIRP), KIRC=Kidney Renal Clear Cell Carcinoma, KICH= Kidney chromophobe , KIRP= Kidney renal papillary cell carcinoma]

**KIPAN**	NFIL3	ZFP36	CCRN4L	JUNB	MCL1	KLF10	RIOK1	NOLC1	DDX21	PPRC1	CSRNP1
	KLF6	EIF4A1	WDR43	DUSP1	SLC2A14	RHOB	SLC2A3	SOCS3	THBS1	C9orf21	EMP1
	B3GNT5	LATS2	GTPBP4	STC1	URB2	SPRY1	ETS1	TNFRSF10A	HBEGF	C1orf107	FOS
	PFKFB3	ASAP1	NUP153	C10orf119	ABL2	FOXC2	NCL	TEX10	C1orf55	ATF3	HNRNPK
	ANKRD40	DDX50	CWC22	AMMECR1L	FOSL2	ADAM17	PTPN12	PNO1	STAT3	SFRS3	NUP98
	MSH6	EGR1	MAP4K4	GEMIN4	HNRNPA3	LONRF3	ADAMTS1	HEATR1	PTP4A1	HNRNPF	
**KIRC**	NFIL3	KDM6B	ZFP36	MIDN	CCRN4L	JUNB	PPRC1	SOCS3	KLF4	MCL1	CSRNP1
	MAFF	ITPRIP	KLF10	DUSP5	RIOK1	ADAMTS1	HBEGF	KLF6	DUSP6	BHLHE40	PFKFB3
	CCL2	SOX7	EMP1	DDX21	IRAK2	NOLC1	SLC2A14	HES1	URB2	ATF3	SPRY2
	FOSL2	TRIB1	ETS2	SLC2A3	JUN	DUSP1	FOS	DDX3X	B3GNT5	WDR43	SFRS7
	PHLDA1	TNFRSF10A	KBTBD2	RHOB	SF1	STC1	SPRY1	NR4A3	GPR4	LATS2	CHD1
	ZFP36L1	ADM	CDC42EP4	NFKBIA	EIF2C2	GPR135	LONRF3	C9orf21	ETS1	FOXC2	NUP98
**KICH**	FCRL3	MFI2	FAM49B								
**KIRP**	NUAK2	RAN	FXR1	PLK2	KPNB1	MRPL3	C1orf107	TEX10	KIAA0020	IARS2	RPL7L1
	NCRNA00120	DARS2	GPN1	IARS	EIF2S3	GMPS	VDAC2	HAUS6			

Table S2: Clinical and pathological characteristics of patients in datasets KIPAN, KIRC and KIRP **[**The size of a primary tumour is described by its T stage. Different phases or classifications of tumour growth or extent are represented by the numbers 1, 2, and 3. A higher T stage typically denotes a larger tumour size or broader dissemination of the disease (KIPAN= Pan-kidney cohort (KICH+KIRC+KIRP), KIRC=Kidney Renal Clear Cell Carcinoma, KIRP= Kidney renal papillary cell carcinoma)]

a)KIPAN

Patient_Barcode	Pathologic_stage	Pathology_T_stage	Gender	Gene name	Percentile of gene expression
TCGA-KN-8427-01A-11R-2315-07	stage iii	t3a	female	PPRC1	>75 percentile
TCGA-CJ-4878-01A-01R-1305-07	stage iii	t3a	male		
TCGA-CW-6093-01A-11R-1672-07	stage i	t1b	female		
TCGA-B0-5104-01A-01R-1420-07	stage iv	t2	female		
TCGA-B0-4701-01A-01R-1277-07	stage iii	t3a	male		
TCGA-B8-A7U6-01A-12R-A37O-07	stage i	t1b	male		
TCGA-B0-5098-01A-01R-1420-07	stage iii	t3a	male		
TCGA-BP-4774-01A-01R-1289-07	stage i	t1a	male		
TCGA-B3-4104-01A-02R-1351-07	stage i	t1	male		
TCGA-B0-5698-01A-11R-1672-07	stage i	t1a	male		
TCGA-AL-3468-01A-02R-1351-07	stage iii	t3a	male		
TCGA-A4-8098-01A-11R-2404-07	stage i	t1a	male		
TCGA-B0-4698-01A-01R-1503-07	stage iv	t4	male		
TCGA-B8-A8YJ-01A-13R-A39I-07	stage i	t1b	male		
TCGA-BP-4992-01A-01R-1334-07	stage i	t1a	male		
TCGA-BP-5200-01A-01R-1426-07	stage i	t1b	female		
TCGA-B0-4696-01A-01R-1277-07	stage iv	t4	male		
TCGA-CZ-5982-01A-11R-1672-07	stage ii	t2	male		
TCGA-B0-4849-01A-01R-1277-07	stage i	t1a	female		
TCGA-B8-4148-01A-02R-1325-07	stage iii	t3a	male		
TCGA-G7-A8LD-01A-11R-A36F-07	stage iv	t4	male		
TCGA-DW-7834-01A-11R-2139-07	stage i	t1b	male		

TCGA-A3-A6NJ-01A-12R-A33J-07	stage i	t1a	male		
TCGA-BQ-5894-01A-11R-1592-07	stage i	t1b	male		
TCGA-F9-A4JJ-01A-11R-A24Z-07	stage i	t1a	female		
TCGA-CJ-4904-01A-02R-1426-07	stage i	t1a	male		
TCGA-B4-5377-01A-01R-1503-07	stage iii	t3b	male		
TCGA-B0-5693-01A-11R-1541-07	stage i	t1b	female		
TCGA-BP-4169-01A-02R-1289-07	stage ii	t2	male		
TCGA-DV-5569-01A-01R-1541-07	stage i	t1a	male		
TCGA-DV-A4VZ-01A-11R-A266-07	stage i	t1	female		
TCGA-GK-A6C7-01A-11R-A33J-07	stage ii	t2a	male		
TCGA-BP-5202-01A-02R-1426-07	stage i	t1b	male		
TCGA-BP-4983-01A-01R-1334-07	stage i	t1b	female		
TCGA-BP-4352-01A-01R-1289-07	stage iii	t3a	female		
TCGA-AK-3440-01A-02R-1277-07	stage i	t1b	female		
TCGA-AK-3428-01A-02R-1277-07	stage iii	t3b	male		
TCGA-SX-A7SM-01A-11R-A355-07	stage i	t1b	female		
TCGA-B0-5084-01A-01R-1334-07	stage iv	t1a	female		
TCGA-A3-3373-01A-02R-1420-07	stage i	t1a	male		
TCGA-BP-5201-01A-01R-1426-07	stage ii	t2	male		
TCGA-B0-5097-01A-01R-1420-07	stage iii	t3b	female		
TCGA-BP-5006-01A-01R-1334-07	stage i	t1a	male		
TCGA-2Z-A9JI-01A-11R-A42S-07	stage i	t1a	male		
TCGA-HE-7130-01A-11R-1965-07	stage i	t1a	male		
TCGA-BP-5004-01A-01R-1334-07	stage i	t1a	male		
TCGA-BP-4326-01A-01R-1289-07	stage iii	t3a	female		
TCGA-DV-5565-01A-01R-1541-07	stage i	t1a	female		
TCGA-KL-8324-01A-11R-2315-07	stage iii	t3a	male		
TCGA-CW-6087-01A-11R-1672-07	stage i	t1b	male		
TCGA-BP-5008-01A-01R-1334-07	stage i	t1b	male		

TCGA-B0-5700-01A-11R-1541-07	stage i	t1b	male		
TCGA-B8-A54J-01A-11R-A33J-07	stage i	t1b	female		
TCGA-BP-4974-01A-01R-1334-07	stage i	t1b	male		
TCGA-A4-7287-01A-11R-2139-07	stage iii	t3a	male		
TCGA-B0-4710-01A-01R-1503-07	stage iv	t3b	male		
TCGA-B0-4838-01A-01R-1305-07	stage iv	t2	male		
TCGA-GL-7966-01A-11R-2204-07	stage i	t1b	male		
TCGA-CZ-4858-01A-01R-1305-07	stage iv	t4	male		
TCGA-CJ-4876-01A-01R-1305-07	stage iii	t3a	male		
TCGA-DV-A4W0-01A-11R-A266-07	stage i	t1a	female		
TCGA-CW-6097-01A-11R-1672-07	stage iv	t3a	male		
TCGA-SX-A7SU-01A-11R-A36F-07	stage i	t1a	male		
TCGA-A3-3307-01A-01R-0864-07	stage i	t1b	male		
TCGA-BQ-5879-01A-11R-1592-07	stage i	t1a	male		
TCGA-BP-4765-01A-01R-1289-07	stage i	t1a	female		
TCGA-DW-7838-01A-11R-2139-07	stage i	t1b	male		
TCGA-BP-4347-01A-01R-1289-07	stage iii	t3a	female		
TCGA-A3-3317-01A-02R-1325-07	stage i	t1b	female		
TCGA-UZ-A9PN-01A-11R-A38C-07	stage iii	t2	male		
TCGA-Y8-A894-01A-11R-A36F-07	stage i	t1a	female		
TCGA-B0-5109-01A-02R-1420-07	stage iii	t3a	female		
TCGA-A3-3313-01A-02R-1325-07	stage ii	t2	male		
TCGA-CJ-4908-01A-01R-1426-07	stage iii	t3a	female		
TCGA-CZ-5467-01A-01R-1503-07	stage ii	t2	male		
TCGA-BQ-5887-01A-11R-1965-07	stage iii	t3a	male		
TCGA-J7-A8I2-01A-12R-A36F-07	stage i	t1a	male		
TCGA-BP-4760-01A-02R-1420-07	stage i	t1a	female		
TCGA-2Z-A9J7-01A-11R-A38C-07	stage i	t1a	male		
TCGA-F9-A8NY-01A-11R-A36F-07	stage iv	t3a	female		

TCGA-CW-5580-01A-01R-1672-07	stage i	t1a	female
TCGA-BQ-7049-01A-11R-1965-07	stage iii	t3b	female
TCGA-CJ-4900-01A-01R-1334-07	stage iii	t3a	male
TCGA-G7-6793-01A-11R-1965-07	stage i	t1a	male
TCGA-CZ-4860-01A-01R-1305-07	stage iii	t3b	female
TCGA-BP-5169-01A-01R-1426-07	stage i	t1a	male
TCGA-A3-3365-01A-01R-0864-07	stage i	t1b	female
TCGA-BP-5198-01A-01R-1426-07	stage iii	t3a	male
TCGA-BP-4981-01A-01R-1334-07	stage iii	t3a	female
TCGA-B0-5108-01A-01R-1420-07	stage i	t1a	female
TCGA-BQ-5877-01A-11R-1592-07	stage iii	t3b	female
TCGA-PJ-A5Z8-01A-11R-A28H-07	stage i	t1a	female
TCGA-P4-A5EA-01A-11R-A28H-07	stage i	t1a	male
TCGA-A3-A8CQ-01A-11R-A37O-07	stage i	t1a	male
TCGA-KO-8413-01A-11R-2315-07	stage i	t1	female
TCGA-BP-4989-01A-01R-1334-07	stage i	t1b	male
TCGA-BP-4993-01A-02R-1420-07	stage i	t1a	male
TCGA-B0-4846-01A-01R-1277-07	stage iii	t3b	male
TCGA-B0-4852-01A-01R-1503-07	stage iii	t3a	female
TCGA-B0-4819-01A-01R-1277-07	stage ii	t2	male
TCGA-P4-A5E8-01A-11R-A28H-07	stage i	t1b	male
TCGA-BP-4173-01A-02R-1289-07	stage i	t1b	male
TCGA-BP-4170-01A-02R-1289-07	stage ii	t2	male
TCGA-A3-3347-01A-02R-1325-07	stage ii	t2a	male
TCGA-CZ-5463-01A-01R-1503-07	stage iii	t3b	female
TCGA-B0-4841-01A-01R-1277-07	stage iii	t3a	male
TCGA-B8-5551-01A-01R-1541-07	stage i	t1b	female
TCGA-G7-6796-01A-11R-1965-07	stage iii	t3a	female
TCGA-A3-A8OX-01A-11R-A37O-07	stage i	t1a	female

TCGA-B0-5110-01A-01R-1420-07	stage iv	t2	male		
TCGA-AK-3461-01A-02R-1277-07	stage i	t1a	male		
TCGA-DZ-6131-01A-11R-1965-07	stage i	t1a	female		
TCGA-DV-5576-01A-01R-1541-07	stage i	t1b	female		
TCGA-EV-5901-01A-11R-1592-07	stage i	t1a	male		
TCGA-2Z-A9JJ-01A-11R-A42S-07	stage i	t1a	male		
TCGA-BP-4771-01A-01R-1289-07	stage i	t1a	male		
TCGA-BP-4349-01A-01R-1289-07	stage iv	t3b	female		
TCGA-KV-A6GD-01A-11R-A31O-07	stage i	t1a	male		
TCGA-B4-5835-01A-11R-1672-07	stage iv	t3	male		
TCGA-G7-A4TM-01A-11R-A31O-07	stage i	t1a	male		
TCGA-BQ-5889-01A-11R-1592-07	stage iii	t3b	female		
TCGA-IZ-8196-01A-11R-2404-07	stage i	t1	male		
TCGA-B0-5107-01A-01R-1420-07	stage iii	t3b	male		
TCGA-BP-4162-01A-02R-1325-07	stage i	t1b	female		
TCGA-SX-A71S-01A-11R-A33Z-07	stage i	t1	male		
TCGA-O9-A75Z-01A-11R-A33Z-07	stage i	t1b	female		
TCGA-G7-6789-01A-11R-1965-07	stage i	t1b	male		
TCGA-CJ-6027-01A-11R-1672-07	stage i	t1a	male		
TCGA-B0-4693-01A-01R-1277-07	stage iii	t3a	male		
TCGA-B0-4815-01A-01R-1503-07	stage i	t1a	female		
TCGA-UZ-A9PL-01A-11R-A38C-07	stage iii	t3	male		
TCGA-B8-4620-01A-02R-1325-07	stage iii	t3a	male		
TCGA-DZ-6132-01A-11R-1965-07	stage i	t1a	male		
TCGA-A4-A57E-01A-11R-A26U-07	stage ii	t2a	female		
TCGA-CJ-6032-01A-11R-1672-07	stage iv	t3a	female		
TCGA-CJ-4920-01A-01R-1426-07	stage i	t1a	male		
TCGA-UZ-A9PR-01A-11R-A42S-07	stage i	t1b	male		
TCGA-BP-4355-01A-01R-1289-07	stage i	t1a	male		

TCGA-KL-8333-01A-11R-2315-07	stage iii	t3a	male		
TCGA-BQ-7059-01A-11R-1965-07	stage i	t1b	female		
TCGA-BQ-5893-01A-11R-1592-07	stage iii	t3a	male		
TCGA-GL-7773-01A-11R-A32Z-07	stage i	t1b	male		
TCGA-BP-4331-01A-01R-1289-07	stage i	t1a	female		
TCGA-DV-5568-01A-01R-1541-07	stage i	t1a	male		
TCGA-A3-3372-01A-02R-1325-07	stage i	t1b	female		
TCGA-KM-8442-01A-11R-2315-07	stage i	t1a	female		
TCGA-CJ-4899-01A-01R-1334-07	stage iii	t3b	male		
TCGA-P4-A5ED-01A-11R-A28H-07	stage iii	t3b	male		
TCGA-KO-8410-01A-11R-2315-07	stage ii	t2	female		
TCGA-B8-5162-01A-01R-1420-07	stage i	t1a	male		
TCGA-B0-4699-01A-01R-1277-07	stage iv	t3a	female		
TCGA-B0-5096-01A-01R-1420-07	stage i	t1	female		
TCGA-B4-5378-01A-01R-1503-07	stage i	t1	male		
TCGA-A4-8311-01A-11R-2404-07	stage i	t1a	male		
TCGA-CZ-5987-01A-11R-1672-07	stage ii	t2	male		
TCGA-J7-8537-01A-11R-2404-07	stage i	t1b	male		
TCGA-BP-4971-01A-01R-1334-07	stage iii	t3a	male		
TCGA-B0-4945-01A-01R-1420-07	stage i	t1a	male		
TCGA-B0-4833-01A-01R-1305-07	stage iv	t3b	male		
TCGA-CZ-5466-01A-01R-1503-07	stage iv	t3b	male		
TCGA-DV-5575-01A-01R-1541-07	stage i	t1b	male		
TCGA-B0-5713-01A-11R-1672-07	stage i	t1b	male		
TCGA-BP-4354-01A-02R-1289-07	stage i	t1b	female		
TCGA-BQ-7048-01A-11R-1965-07	stage ii	t2	male		
TCGA-B0-4690-01A-01R-1277-07	stage iii	t3a	female		
TCGA-DV-5567-01A-01R-1541-07	stage i	t1a	female		
TCGA-DW-7836-01A-11R-2139-07	stage i	t1b	male		

TCGA-B0-4821-01A-01R-1503-07	stage i	t1a	male		
TCGA-AL-A5DJ-01A-11R-A26U-07	stage i	t1b	female		
TCGA-A4-7734-01A-11R-A32Z-07	stage ii	t2	male		
TCGA-V9-A7HT-01A-11R-A33Z-07	stage i	t1a	female		
TCGA-UZ-A9PK-01A-11R-A38C-07	stage ii	t2	male		
TCGA-BP-4781-01A-01R-1305-07	stage iv	t3a	female		
TCGA-B0-4816-01A-01R-1503-07	stage ii	t2	female		
TCGA-CJ-4882-01A-02R-1426-07	stage iv	t3a	male		
TCGA-CJ-4888-01A-01R-1305-07	stage iv	t3a	male		
TCGA-KO-8405-01A-11R-2315-07	stage ii	t2	male		
TCGA-A3-3308-01A-02R-1325-07	stage i	t1b	male		
TCGA-DW-5560-01A-01R-1592-07	stage i	t1	male		
TCGA-KN-8426-01A-11R-2315-07	stage ii	t2	male		
TCGA-BQ-7050-01A-11R-1965-07	stage iii	t3	female		
TCGA-B0-4718-01A-01R-1277-07	stage iii	t3a	male		
TCGA-A3-3387-01A-01R-1541-07	stage i	t1a	female		
TCGA-G6-A8L6-01A-11R-A37O-07	stage i	t1a	female		
TCGA-G7-6790-01A-11R-1965-07	stage iv	t3a	female		
TCGA-BQ-7044-01A-11R-1965-07	stage iii	t3a	male		
TCGA-2Z-A9J9-01A-11R-A42S-07	stage i	t1a	female		
TCGA-CZ-4859-01A-02R-1426-07	stage ii	t2	male		
TCGA-BP-4770-01A-01R-1503-07	stage i	t1a	female		
TCGA-CJ-4901-01A-01R-1426-07	stage i	t1b	male		
TCGA-CJ-4643-01A-02R-1325-07	stage iv	t3a	male		
TCGA-B1-5398-01A-02R-1592-07	stage i	t1a	male		
TCGA-BP-4177-01A-02R-1420-07	stage i	t1b	female		
TCGA-B0-4688-01A-01R-1277-07	stage iv	t2	male		
TCGA-BP-4986-01A-01R-1334-07	stage i	t1a	male		
TCGA-B2-5636-01A-02R-1541-07	stage i	t1a	male		

TCGA-BP-4776-01A-01R-1289-07	stage i	t1a	male		
TCGA-2Z-A9J3-01A-12R-A38C-07	stage i	t1a	male		
TCGA-BP-4798-01A-01R-1305-07	stage iii	t3a	male		
TCGA-B0-4828-01A-01R-1277-07	stage i	t1a	male		
TCGA-CJ-4894-01A-01R-1305-07	stage i	t1b	male		
TCGA-B9-A69E-01A-11R-A31O-07	stage i	t1b	male		
TCGA-B0-4691-01A-01R-1277-07	stage iii	t3b	male		
TCGA-BQ-5888-01A-11R-1592-07	stage i	t1a	male		
TCGA-2Z-A9JL-01A-11R-A42S-07	stage i	t1a	male		
TCGA-B0-4697-01A-01R-1277-07	stage iv	t4	male		
TCGA-B0-4823-01A-02R-1420-07	stage iii	t3b	female		
TCGA-CZ-4854-01A-01R-1305-07	stage i	t1b	male		
TCGA-CJ-4916-01A-01R-1426-07	stage i	t1b	female		
TCGA-2Z-A9JS-01A-21R-A42S-07	stage iii	t3a	male		
TCGA-B4-5844-01A-11R-1672-07	stage i	t1b	female		
TCGA-IZ-A6M9-01A-11R-A31O-07	stage iii	t3	female		<25 Percentile
TCGA-B9-A8YH-01A-11R-A37K-07	stage iii	t3a	female		
TCGA-BP-4959-01A-01R-1334-07	stage i	t1a	male		
TCGA-KL-8336-01A-11R-2315-07	stage iii	t3a	male		
TCGA-CJ-4635-01A-02R-1305-07	stage iv	t3a	female		
TCGA-BP-4972-01A-01R-1334-07	stage iv	t3a	male		
TCGA-BP-5186-01A-01R-1426-07	stage i	t1a	male		
TCGA-P4-AAVK-01A-11R-A42S-07	stage i	t1a	male		
TCGA-BP-5000-01A-01R-1334-07	stage i	t1a	male		
TCGA-B0-4824-01A-01R-1277-07	stage iv	t2	male		
TCGA-A3-3383-01A-02R-1325-07	stage i	t1a	male		
TCGA-BP-4975-01A-01R-1334-07	stage iii	t3a	female		
TCGA-HE-A5NL-01A-11R-A26U-07	stage i	t1a	female		
TCGA-BP-4804-01A-02R-1305-07	stage ii	t2	male		

TCGA-UZ-A9PJ-01A-11R-A38C-07	stage ii	t2	male
TCGA-KN-8436-01A-11R-2315-07	stage i	t1a	male
TCGA-BP-5174-01A-01R-1426-07	stage i	t1a	female
TCGA-BP-4964-01A-01R-1334-07	stage i	t1b	male
TCGA-BP-4164-01A-02R-1325-07	stage i	t1a	male
TCGA-BP-5173-01A-01R-1426-07	stage i	t1a	male
TCGA-A4-7732-01A-11R-2139-07	stage ii	t2b	female
TCGA-BQ-7058-01A-11R-1965-07	stage i	t1a	male
TCGA-A4-8310-01A-11R-2404-07	stage i	t1	male
TCGA-SX-A71V-01A-11R-A33Z-07	stage iv	t3a	male
TCGA-CW-5589-01A-01R-1541-07	stage iv	t3a	male
TCGA-B0-5116-01A-02R-1420-07	stage i	t1b	female
TCGA-B8-A54F-01A-11R-A266-07	stage i	t1b	male
TCGA-BP-4338-01A-01R-1289-07	stage iii	t3a	male
TCGA-B0-5120-01A-01R-1420-07	stage i	t1b	male
TCGA-SX-A7SL-01A-11R-A355-07	stage i	t1b	male
TCGA-B9-A44B-01A-11R-A24Z-07	stage iii	t3a	male
TCGA-BP-4789-01A-01R-1305-07	stage iii	t3b	male
TCGA-2Z-A9JG-01A-11R-A42S-07	stage iii	t3a	male
TCGA-B3-3925-01A-02R-1351-07	stage i	t1a	male
TCGA-2Z-A9J5-01A-21R-A38C-07	stage iv	t2	male
TCGA-A4-A772-01A-11R-A33Z-07	stage iv	t3b	male
TCGA-AK-3429-01A-02R-1325-07	stage ii	t2	female
TCGA-BP-4174-01A-02R-1289-07	stage i	t1a	male
TCGA-CJ-4907-01A-01R-1426-07	stage ii	t2	male
TCGA-KO-8416-01A-11R-2315-07	stage i	t1a	male
TCGA-A3-3316-01A-01R-0864-07	stage i	t1b	male
TCGA-KN-8418-01A-11R-2315-07	stage ii	t2	female
TCGA-BP-5184-01A-01R-1426-07	stage i	t1a	female

TCGA-BP-5192-01A-01R-1426-07	stage i	t1a	male		
TCGA-CJ-4644-01A-02R-1325-07	stage iii	t2	male		
TCGA-BP-4158-01A-02R-1289-07	stage iii	t3a	female		
TCGA-B0-5117-01A-01R-1420-07	stage i	t1a	female		
TCGA-CJ-4639-01A-02R-1325-07	stage iv	t3a	female		
TCGA-KO-8411-01A-11R-2315-07	stage i	t1b	female		
TCGA-BP-4759-01A-01R-1289-07	stage i	t1a	female		
TCGA-BP-4803-01A-01R-1305-07	stage i	t1b	male		
TCGA-B0-5711-01A-11R-1672-07	stage iii	t3b	female		
TCGA-BP-4766-01A-01R-1289-07	stage i	t1a	male		
TCGA-GL-8500-01A-11R-2404-07	stage iii	t3c	male		
TCGA-KN-8422-01A-11R-2315-07	stage i	t1b	female		
TCGA-MH-A55Z-01A-11R-A26U-07	stage i	t1a	male		
TCGA-A3-3349-01A-01R-1188-07	stage iii	t3a	male		
TCGA-B8-5165-01A-01R-1420-07	stage i	t1b	female		
TCGA-B0-5701-01A-11R-1541-07	stage i	t1b	male		
TCGA-UZ-A9PZ-01A-11R-A42S-07	stage i	t1a	male		
TCGA-B0-4712-01A-01R-1503-07	stage i	t1b	male		
TCGA-A3-A8OV-01A-11R-A37O-07	stage iii	t3a	male		
TCGA-BQ-5875-01A-11R-1592-07	stage iv	t3a	male		
TCGA-WN-A9G9-01A-12R-A37K-07	stage i	t1a	female		
TCGA-B2-3923-01A-02R-1325-07	stage i	t1b	female		
TCGA-CW-5581-01A-02R-1541-07	stage iii	t3b	male		
TCGA-BP-4166-01A-02R-1289-07	stage ii	t2	female		
TCGA-B0-5699-01A-11R-1541-07	stage iii	t3b	male		
TCGA-B0-5102-01A-01R-1420-07	stage i	t1a	male		
TCGA-SX-A71R-01A-12R-A33Z-07	stage i	t1a	male		
TCGA-BQ-5892-01A-11R-1592-07	stage i	t1a	female		
TCGA-SX-A7SS-01A-11R-A36F-07	stage i	t1b	female		

TCGA-KN-8419-01A-11R-2315-07	stage i	t1a	female		
TCGA-BQ-5881-01A-11R-1592-07	stage i	t1a	female		
TCGA-G7-A8LB-01A-11R-A36F-07	stage iii	t3a	male		
TCGA-A3-3385-01A-02R-1420-07	stage i	t1a	male		
TCGA-B0-4837-01A-01R-1305-07	stage i	t1b	female		
TCGA-BP-4982-01A-01R-1334-07	stage iii	t3a	male		
TCGA-BP-5185-01A-01R-1426-07	stage i	t1a	male		
TCGA-A4-A5DU-01A-11R-A28H-07	stage i	t1b	female		
TCGA-CJ-6031-01A-11R-1672-07	stage iv	t3a	female		
TCGA-KL-8334-01A-11R-2315-07	stage iv	t3b	female		
TCGA-CZ-5985-01A-11R-1672-07	stage iv	t3b	male		
TCGA-B9-4617-01A-01R-1193-07	stage ii	t2	male		
TCGA-A4-7288-01A-11R-A32Z-07	stage i	t1a	female		
TCGA-MH-A562-01A-11R-A26U-07	stage i	t1a	male		
TCGA-AK-3465-01A-02R-1325-07	stage i	t1a	male		
TCGA-KM-8477-01A-11R-2315-07	stage i	t1b	male		
TCGA-AK-3434-01A-02R-1277-07	stage i	t1a	male		
TCGA-CJ-5678-01A-11R-1541-07	stage iv	t3a	female		
TCGA-BP-4976-01A-01R-1334-07	stage i	t1a	male		
TCGA-A4-8312-01A-11R-2404-07	stage iii	t3a	male		
TCGA-B4-5834-01A-11R-1672-07	stage i	t1b	female		
TCGA-B8-A54E-01A-11R-A266-07	stage i	t1a	male		
TCGA-J7-6720-01A-11R-2139-07	stage i	t1b	male		
TCGA-CW-5588-01A-01R-1541-07	stage iv	t3a	male		
TCGA-EU-5907-01A-11R-1672-07	stage iv	t2a	male		
TCGA-CJ-4887-01A-01R-1305-07	stage i	t1a	female		
TCGA-G7-7501-01A-11R-2204-07	stage i	t1a	male		
TCGA-KO-8415-01A-11R-2315-07	stage i	t1	female		
TCGA-BP-4968-01A-01R-1334-07	stage iii	t1a	male		

TCGA-A4-7286-01A-11R-A32Z-07	stage i	t1	male		
TCGA-F9-A7VF-01A-11R-A33Z-07	stage iii	t3	male		
TCGA-2Z-A9JP-01A-11R-A42S-07	stage i	t1a	female		
TCGA-A3-3357-01A-02R-1420-07	stage ii	t2	male		
TCGA-B0-5706-01A-11R-1541-07	stage iii	t3a	female		
TCGA-CJ-4885-01A-01R-1305-07	stage iv	t3a	male		
TCGA-B0-4713-01A-01R-1277-07	stage iii	t3a	male		
TCGA-KL-8339-01A-11R-2315-07	stage i	t1b	female		
TCGA-B2-4099-01A-02R-1188-07	stage i	t1b	male		
TCGA-B8-A54D-01A-21R-A266-07	stage i	t1a	female		
TCGA-IA-A40U-01A-11R-A24Z-07	stage iii	t3a	female		
TCGA-CJ-4638-01A-02R-1325-07	stage iii	t3a	male		
TCGA-A4-A48D-01A-11R-A24Z-07	stage iv	t2a	male		
TCGA-CW-5585-01A-01R-1541-07	stage i	t1a	female		
TCGA-KL-8343-01A-11R-2315-07	stage iii	t3a	male		
TCGA-2Z-A9J8-01A-11R-A42S-07	stage i	t1a	male		
TCGA-MH-A55W-01A-11R-A26U-07	stage i	t1a	male		
TCGA-B0-4836-01A-01R-1305-07	stage i	t1b	female		
TCGA-KL-8335-01A-11R-2315-07	stage ii	t2	male		
TCGA-KO-8408-01A-11R-2315-07	stage i	t1b	female		
TCGA-B0-5119-01A-02R-1420-07	stage i	t1b	male		
TCGA-CJ-4875-01A-01R-1305-07	stage iii	t3a	female		
TCGA-MH-A560-01A-11R-A26U-07	stage i	t1a	male		
TCGA-BQ-5876-01A-11R-1592-07	stage iii	t3a	female		
TCGA-GL-A9DE-01A-11R-A37K-07	stage ii	t2	male		
TCGA-KM-8440-01A-11R-2315-07	stage i	t1	male		
TCGA-B0-5402-01A-01R-1503-07	stage iii	t3b	female		
TCGA-B0-5696-01A-11R-1541-07	stage i	t1	male		
TCGA-B0-4817-01A-01R-1277-07	stage iv	t3b	female		

TCGA-CW-5583-01A-02R-1541-07	stage iv	t3b	male		
TCGA-KL-8342-01A-11R-2315-07	stage iii	t3a	male		
TCGA-CZ-5468-01A-01R-1503-07	stage ii	t2	female		
TCGA-KN-8430-01A-11R-2315-07	stage iii	t3a	male		
TCGA-A3-3331-01A-02R-1325-07	stage i	t1b	male		
TCGA-DV-A4VX-01A-11R-A266-07	stage i	t1	female		
TCGA-BP-4807-01A-01R-1305-07	stage i	t1a	male		
TCGA-CZ-5458-01A-01R-1503-07	stage iv	t3b	male		
TCGA-CJ-4893-01A-01R-1305-07	stage iii	t3a	female		
TCGA-CZ-4866-01A-01R-1503-07	stage ii	t2	male		
TCGA-BP-4353-01A-02R-1289-07	stage i	t1a	male		
TCGA-BQ-7046-01A-11R-1965-07	stage i	t1a	male		
TCGA-KM-8476-01A-11R-2315-07	stage i	t1a	male		
TCGA-B1-A657-01A-11R-A31O-07	stage iii	t3a	male		
TCGA-P4-A5EB-01A-11R-A28H-07	stage iii	t3a	male		
TCGA-AK-3455-01A-01R-0864-07	stage iv	t3a	male		
TCGA-KO-8409-01A-11R-2315-07	stage i	t1	male		
TCGA-CZ-5470-01A-01R-1503-07	stage i	t1b	male		
TCGA-BP-4762-01A-02R-1289-07	stage i	t1a	female		
TCGA-KL-8346-01A-11R-2315-07	stage iii	t3a	male		
TCGA-A3-3328-01A-01R-0864-07	stage i	t1	female		
TCGA-KL-8345-01A-11R-2315-07	stage ii	t2	female		
TCGA-B0-4814-01A-01R-1277-07	stage iii	t3c	male		
TCGA-PJ-A5Z9-01A-11R-A28H-07	stage i	t1b	male		
TCGA-A3-3363-01A-01R-0864-07	stage i	t1b	male		
TCGA-B8-5549-01A-01R-1541-07	stage i	t1b	female		
TCGA-B0-5083-01A-02R-1420-07	stage iii	t3a	female		
TCGA-A3-3323-01A-02R-1325-07	stage i	t1a	male		
TCGA-BP-4167-01A-02R-1325-07	stage i	t1b	female		

TCGA-BQ-5886-01A-11R-1592-07	stage i	t1b	male		
TCGA-BP-5194-01A-02R-1426-07	stage i	t1a	male		
TCGA-BP-5181-01A-01R-1426-07	stage iii	t3a	male		
TCGA-A4-8515-01A-11R-2404-07	stage i	t1a	male		
TCGA-BQ-5880-01A-11R-1592-07	stage iii	t3b	male		
TCGA-G6-A5PC-01A-11R-A33J-07	stage i	t1b	female		
TCGA-MH-A855-01A-11R-A355-07	stage i	t1a	female		
TCGA-UZ-A9Q0-01A-12R-A42S-07	stage ii	t2	male		
TCGA-AK-3436-01A-02R-1325-07	stage ii	t2	male		
TCGA-B0-4839-01A-01R-1305-07	stage iii	t3a	female		
TCGA-BP-5191-01A-01R-1426-07	stage iv	t3b	male		
TCGA-B4-5838-01A-11R-1672-07	stage ii	t2	female		
TCGA-A3-3352-01A-01R-0864-07	stage i	t1a	female		
TCGA-DZ-6133-01A-11R-1965-07	stage i	t1	female		
TCGA-AK-3427-01A-01R-0864-07	stage ii	t2	female		
TCGA-BP-4962-01A-01R-1334-07	stage i	t1a	female		
TCGA-F9-A97G-01A-11R-A38C-07	stage i	t1a	male		
TCGA-B0-4694-01A-01R-1277-07	stage iv	t3b	female		
TCGA-CJ-4897-01A-03R-1426-07	stage iv	t4	female		
TCGA-AK-3443-01A-02R-1325-07	stage iii	t3a	male		
TCGA-SX-A7SR-01A-12R-A36F-07	stage i	t1	male		
TCGA-KL-8341-01A-11R-2315-07	stage iii	t3	male		
TCGA-CJ-4874-01A-01R-1305-07	stage ii	t2b	male		
TCGA-BP-5190-01A-01R-1426-07	stage i	t1a	male		
TCGA-IZ-A6M8-01A-11R-A31O-07	stage i	t1a	male		
TCGA-Y8-A895-01A-11R-A36F-07	stage i	t1	male		
TCGA-B0-5702-01A-11R-1541-07	stage i	t1	female		
TCGA-BP-4329-01A-02R-1289-07	stage iii	t3a	male		
TCGA-CZ-4853-01A-01R-1426-07	stage ii	t2	male		

TCGA-CZ-5451-01A-01R-1503-07	stage ii	t2	male		
TCGA-2Z-A9J1-01A-11R-A38C-07	stage ii	t2	male		
TCGA-IA-A83S-01A-11R-A355-07	stage i	t1b	male		
TCGA-A4-7585-01A-11R-2139-07	stage i	t1a	female		
TCGA-G7-A8LC-01A-11R-A36F-07	stage i	t1a	male		
TCGA-KL-8325-01A-11R-2315-07	stage i	t1b	female		
TCGA-BP-5180-01A-01R-1426-07	stage iii	t3a	male		
TCGA-CZ-4856-01A-02R-1426-07	stage i	t1a	female		
TCGA-B8-4621-01A-01R-1503-07	stage ii	t2a	female		
TCGA-B8-4154-01A-01R-1188-07	stage iii	t3a	female		
TCGA-UZ-A9PS-01A-11R-A42S-07	stage i	t1	male		
TCGA-A4-8518-01A-11R-2404-07	stage i	t1b	male		
TCGA-UZ-A9Q1-01A-11R-A42S-07	stage i	t1b	male		
TCGA-BP-4763-01A-01R-1289-07	stage i	t1a	female		
TCGA-A4-A5XZ-01A-11R-A31O-07	stage i	t1a	male		
TCGA-BP-4343-01A-02R-1289-07	stage iii	t3b	male		
TCGA-IZ-8195-01A-31R-2404-07	stage i	t1a	male		
TCGA-AL-3473-01A-01R-1193-07	stage i	t1a	male		
TCGA-AK-3451-01A-02R-1188-07	stage ii	t2	male		
TCGA-CZ-5989-01A-11R-1672-07	stage i	t1a	male		
TCGA-AK-3458-01A-01R-1503-07	stage i	t1a	male		
TCGA-AK-3447-01A-01R-1766-07	stage ii	t2	female		
TCGA-B4-5832-01A-11R-1672-07	stage i	t1	female		
TCGA-4A-A93X-01A-11R-A37K-07	stage iv	t3a	male	EIF4A1	>75 percentile
TCGA-B0-5109-01A-02R-1420-07	stage iii	t3b	male		
TCGA-BP-4983-01A-01R-1334-07	stage iii	t3a	female		
TCGA-KN-8427-01A-11R-2315-07	stage iv	t4	male		
TCGA-AL-A5DJ-01A-11R-A26U-07	stage iii	t3a	female		
TCGA-B0-4698-01A-01R-1503-07	stage iv	t4	male		

TCGA-B0-4696-01A-01R-1277-07	stage iii	t3a	male		
TCGA-GL-A59T-01A-21R-A28H-07	stage i	t1b	male		
TCGA-BP-4338-01A-01R-1289-07	stage i	t1b	male		
TCGA-B0-5702-01A-11R-1541-07	stage i	t1b	male		
TCGA-A4-A5Y0-01A-11R-A31O-07	stage i	t1b	female		
TCGA-B0-5098-01A-01R-1420-07	stage i	t1	female		
TCGA-B8-A7U6-01A-12R-A37O-07	stage i	t1a	female		
TCGA-BQ-5893-01A-11R-1592-07	stage iv	t3a	male		
TCGA-B0-4701-01A-01R-1277-07	stage iv	t3a	female		
TCGA-B0-5097-01A-01R-1420-07	stage iii	t3b	female		
TCGA-B0-4688-01A-01R-1277-07	stage iv	t4	male		
TCGA-DW-7838-01A-11R-2139-07	stage i	t1b	male		
TCGA-Q2-A5QZ-01A-11R-A28H-07	stage iii	t3a	female		
TCGA-2Z-A9J7-01A-11R-A38C-07	stage iv	t2	male		
TCGA-B0-4712-01A-01R-1503-07	stage iv	t3a	male		
TCGA-2Z-A9J8-01A-11R-A42S-07	stage i	t1b	male		
TCGA-BQ-5891-01A-11R-1592-07	stage iii	t3b	female		
TCGA-2Z-A9JT-01A-11R-A42S-07	stage i	t1a	male		
TCGA-DV-A4VZ-01A-11R-A266-07	stage i	t1a	male		
TCGA-B2-4099-01A-02R-1188-07	stage i	t1a	male		
TCGA-P4-AAVO-01A-11R-A42S-07	stage i	t1b	male		
TCGA-Y8-A898-01A-11R-A355-07	stage i	t1a	male		
TCGA-F9-A4JJ-01A-11R-A24Z-07	stage iii	t3a	female		
TCGA-BP-4326-01A-01R-1289-07	stage i	t1b	female		
TCGA-G7-6790-01A-11R-1965-07	stage i	t1a	male		
TCGA-Y8-A8RZ-01A-11R-A37K-07	stage i	t1	male		
TCGA-CW-6090-01A-11R-1672-07	stage i	t1b	male		
TCGA-BP-5178-01A-01R-1426-07	stage iv	t3a	male		
TCGA-CZ-4861-01A-01R-1305-07	stage ii	t2	male		

TCGA-SX-A7SM-01A-11R-A355-07	stage iv	t3a	male		
TCGA-DV-5569-01A-01R-1541-07	stage i	t1a	female		
TCGA-CJ-4878-01A-01R-1305-07	stage iii	t3a	female		
TCGA-B0-5707-01A-11R-1541-07	stage i	t1a	female		
TCGA-BQ-7046-01A-11R-1965-07	stage i	t1a	male		
TCGA-CJ-6027-01A-11R-1672-07	stage i	t1a	male		
TCGA-B3-4104-01A-02R-1351-07	stage ii	t2	male		
TCGA-CZ-5982-01A-11R-1672-07	stage i	t1a	female		
TCGA-BP-5169-01A-01R-1426-07	stage i	t1b	male		
TCGA-BQ-5894-01A-11R-1592-07	stage iv	t3b	male		
TCGA-B0-5107-01A-01R-1420-07	stage iv	t2	female		
TCGA-IA-A40Y-01A-11R-A24Z-07	stage iii	t3a	female		
TCGA-B0-4700-01A-02R-1541-07	stage iv	t4	male		
TCGA-KO-8404-01A-11R-2315-07	stage iv	t4	male		
TCGA-BP-4985-01A-01R-1334-07	stage iii	t3a	male		
TCGA-BP-5006-01A-01R-1334-07	stage i	t1a	male		
TCGA-F9-A7VF-01A-11R-A33Z-07	stage i	t1a	female		
TCGA-B8-5162-01A-01R-1420-07	stage ii	t2a	male		
TCGA-CJ-5679-01A-11R-1541-07	stage iii	t3b	male		
TCGA-B0-5700-01A-11R-1541-07	stage i	t1a	male		
TCGA-B8-4620-01A-02R-1325-07	stage iii	t3a	female		
TCGA-KV-A74V-01A-11R-A33Z-07	stage i	t1a	male		
TCGA-CZ-4857-01A-01R-1305-07	stage iv	t3a	male		
TCGA-BP-4343-01A-02R-1289-07	stage iii	t3a	male		
TCGA-UZ-A9PM-01A-21R-A38C-07	stage ii	t2	male		
TCGA-AK-3431-01A-02R-1277-07	stage ii	t2	female		
TCGA-AK-3428-01A-02R-1277-07	stage iii	t3b	male		
TCGA-CJ-4904-01A-02R-1426-07	stage iv	t3a	female		
TCGA-DW-7834-01A-11R-2139-07	stage i	t1	male		

TCGA-2Z-A9J2-01A-11R-A38C-07	stage i	t1a	female		
TCGA-2Z-A9JQ-01A-11R-A42S-07	stage i	t1a	male		
TCGA-BP-4770-01A-01R-1503-07	stage iv	t4	female		
TCGA-A4-8310-01A-11R-2404-07	stage iii	t3a	male		
TCGA-P4-A5EA-01A-11R-A28H-07	stage iii	t3a	female		
TCGA-DV-5566-01A-01R-1541-07	stage i	t1a	female		
TCGA-CZ-5454-01A-01R-1503-07	stage iv	t2	male		
TCGA-B8-5551-01A-01R-1541-07	stage i	t1b	female		
TCGA-B0-4844-01A-01R-1277-07	stage iv	t3a	male		
TCGA-P4-A5E8-01A-11R-A28H-07	stage iii	t2a	male		
TCGA-BP-5202-01A-02R-1426-07	stage iii	t3a	male		
TCGA-DV-5568-01A-01R-1541-07	stage i	t1a	male		
TCGA-B0-4690-01A-01R-1277-07	stage iv	t4	male		
TCGA-B0-5116-01A-02R-1420-07	stage iii	t3b	male		
TCGA-CW-6093-01A-11R-1672-07	stage i	t1a	male		
TCGA-B0-5084-01A-01R-1334-07	stage iv	t3a	male		
TCGA-B8-4143-01A-01R-1188-07	stage iv	t3a	female		
TCGA-IZ-A6M8-01A-11R-A31O-07	stage i	t1a	male		
TCGA-2Z-A9JE-01A-11R-A42S-07	stage i	t1a	male		
TCGA-BP-4774-01A-01R-1289-07	stage i	t1a	female		
TCGA-B9-4115-01A-01R-1193-07	stage i	t1a	male		
TCGA-HE-A5NF-01A-11R-A26U-07	stage i	t1a	male		
TCGA-2Z-A9JP-01A-11R-A42S-07	stage i	t1a	male		
TCGA-UZ-A9PS-01A-11R-A42S-07	stage ii	t2	female		
TCGA-B0-5104-01A-01R-1420-07	stage i	t1	female		
TCGA-CJ-6033-01A-11R-1672-07	stage iv	t3a	female		
TCGA-B8-5158-01A-01R-1420-07	stage iii	t3a	male		
TCGA-BP-5173-01A-01R-1426-07	stage i	t1a	male		
TCGA-2Z-A9J3-01A-12R-A38C-07	stage ii	t2	male		

TCGA-BQ-7044-01A-11R-1965-07	stage iii	t3a	male		
TCGA-GL-A4EM-01A-11R-A24Z-07	stage i	t1b	male		
TCGA-B0-5693-01A-11R-1541-07	stage i	t1b	female		
TCGA-A3-3365-01A-01R-0864-07	stage i	t1a	male		
TCGA-BQ-5882-01A-11R-1592-07	stage iii	t3b	male		
TCGA-CZ-5465-01A-01R-1503-07	stage iii	t3b	female		
TCGA-CJ-4901-01A-01R-1426-07	stage iii	t3b	male		
TCGA-CJ-5678-01A-11R-1541-07	stage iv	t2b	male		
TCGA-A3-3383-01A-02R-1325-07	stage i	t1	male		
TCGA-BP-5200-01A-01R-1426-07	stage ii	t2	male		
TCGA-BP-4329-01A-02R-1289-07	stage iii	t3a	male		
TCGA-CW-6097-01A-11R-1672-07	stage iii	t3a	male		
TCGA-A3-3307-01A-01R-0864-07	stage iii	t3b	male		
TCGA-B0-5698-01A-11R-1672-07	stage i	t1b	male		
TCGA-F9-A8NY-01A-11R-A36F-07	stage iv	t4	female		
TCGA-A3-3335-01A-01R-0864-07	stage ii	t2a	male		
TCGA-CZ-4860-01A-01R-1305-07	stage iv	t4	male		
TCGA-AK-3461-01A-02R-1277-07	stage i	t1a	male		
TCGA-B0-5703-01A-11R-1541-07	stage i	t1b	male		
TCGA-CJ-4891-01A-01R-1305-07	stage iii	t3c	female		
TCGA-CZ-5466-01A-01R-1503-07	stage iii	t3a	male		
TCGA-B0-5697-01A-11R-1541-07	stage i	t1a	male		
TCGA-CZ-5467-01A-01R-1503-07	stage iii	t3a	female		
TCGA-GL-A9DC-01A-11R-A37K-07	stage i	t1b	female		
TCGA-B4-5838-01A-11R-1672-07	stage iv	t3	male		
TCGA-BP-4354-01A-02R-1289-07	stage iv	t4	male		
TCGA-B1-5398-01A-02R-1592-07	stage iii	t3b	male		
TCGA-B2-3924-01A-02R-1325-07	stage i	t1b	male		
TCGA-B0-5696-01A-11R-1541-07	stage iii	t3a	male		

TCGA-BP-4761-01A-01R-1289-07	stage iii	t3a	male		
TCGA-CZ-5469-01A-01R-1503-07	stage ii	t2	male		
TCGA-B0-5695-01A-11R-1541-07	stage i	t1b	female		
TCGA-IA-A83V-01A-11R-A355-07	stage i	t1b	male		
TCGA-BP-4759-01A-01R-1289-07	stage i	t1a	male		
TCGA-BQ-5879-01A-11R-1592-07	stage iii	t3b	female		
TCGA-CJ-4890-01A-01R-1305-07	stage iv	t3a	male		
TCGA-A3-3363-01A-01R-0864-07	stage ii	t2	male		
TCGA-CJ-4900-01A-01R-1334-07	stage iv	t4	female		
TCGA-BQ-5881-01A-11R-1592-07	stage i	t1a	male		
TCGA-A3-3313-01A-02R-1325-07	stage i	t1b	male		
TCGA-BQ-5890-01A-11R-1592-07	stage iii	t3a	male		
TCGA-B1-A47M-01A-11R-A24Z-07	stage iii	t3a	male		
TCGA-BP-4781-01A-01R-1305-07	stage i	t1a	male		
TCGA-A3-3325-01A-01R-0864-07	stage i	t1a	male		
TCGA-EU-5907-01A-11R-1672-07	stage iii	t3a	male		
TCGA-BP-5198-01A-01R-1426-07	stage iii	t3b	male		
TCGA-B1-7332-01A-11R-A32Z-07	stage ii	t2a	female		
TCGA-B1-A656-01A-11R-A31O-07	stage i	t1a	male		
TCGA-BP-4345-01A-01R-1289-07	stage iii	t3b	male		
TCGA-BP-5170-01A-01R-1426-07	stage i	t1a	male		
TCGA-AK-3451-01A-02R-1188-07	stage ii	t2	male		
TCGA-BP-4975-01A-01R-1334-07	stage i	t1b	male		
TCGA-CW-5590-01A-01R-1541-07	stage iv	t3a	male		
TCGA-MH-A561-01A-11R-A26U-07	stage i	t1a	male		
TCGA-A3-A6NJ-01A-12R-A33J-07	stage i	t1a	female		
TCGA-B0-5094-01A-01R-1420-07	stage iv	t3b	male		
TCGA-A4-8518-01A-11R-2404-07	stage i	t1a	male		
TCGA-B9-A44B-01A-11R-A24Z-07	stage iii	t3b	male		

TCGA-GL-7966-01A-11R-2204-07	stage iii	t3	female			
TCGA-2Z-A9J5-01A-21R-A38C-07	stage ii	t2	male			
TCGA-MH-A854-01A-11R-A355-07	stage i	t1b	female			
TCGA-B0-4849-01A-01R-1277-07	stage iii	t3a	male			
TCGA-KV-A6GD-01A-11R-A31O-07	stage i	t1b	male			
TCGA-2K-A9WE-01A-11R-A38C-07	stage ii	t2b	male			
TCGA-CZ-5986-01A-11R-1672-07	stage i	t1	male			
TCGA-B0-4691-01A-01R-1277-07	stage iv	t2	male			
TCGA-J7-6720-01A-11R-2139-07	stage i	t1	male			
TCGA-CZ-5461-01A-01R-1503-07	stage iv	t1b	male			
TCGA-AK-3425-01A-02R-1277-07	stage i	t1	male			
TCGA-BQ-7051-01A-12R-1965-07	stage ii	t2	male			
TCGA-B0-5096-01A-01R-1420-07	stage iii	t3a	female			
TCGA-CJ-4923-01A-01R-1426-07	stage iv	t3a	female			
TCGA-G7-6795-01A-11R-1965-07	stage i	t1a	male			
TCGA-B0-4836-01A-01R-1305-07	stage iv	t3b	male			
TCGA-BQ-7045-01A-31R-1965-07	stage i	t1b	male			
TCGA-CZ-5455-01A-01R-1503-07	stage iv	t3b	male			
TCGA-DV-5565-01A-01R-1541-07	stage i	t1a	male			
TCGA-CJ-4882-01A-02R-1426-07	stage iii	t3a	male			
TCGA-CW-5580-01A-01R-1672-07	stage iv	t3a	female			
TCGA-AK-3436-01A-02R-1325-07	stage iv	t2	male			
TCGA-SX-A7SP-01A-11R-A355-07	stage i	t1b	female			
TCGA-CZ-5470-01A-01R-1503-07	stage ii	t2	female			
TCGA-CZ-5457-01A-01R-1503-07	stage iii	t3a	male			
TCGA-BP-4160-01A-02R-1289-07	stage iii	t3a	male			
TCGA-CW-6087-01A-11R-1672-07	stage iv	t3a	male			
TCGA-B2-4098-01A-02R-1325-07	stage i	t1b	female			
TCGA-B8-4154-01A-01R-1188-07	stage i	t1a	female			

TCGA-P4-A5E7-01A-31R-A28H-07	stage i	t1b	female		
TCGA-CJ-4868-01A-01R-1305-07	stage iv	t3a	male		
TCGA-BP-5175-01A-01R-1426-07	stage i	t1a	male		
TCGA-BP-5201-01A-01R-1426-07	stage iv	t3b	male		
TCGA-B1-A654-01A-11R-A31O-07	stage i	t1a	female		
TCGA-G7-6796-01A-11R-1965-07	stage i	t1a	male		
TCGA-2Z-A9JD-01A-11R-A42S-07	stage i	t1a	male		
TCGA-V9-A7HT-01A-11R-A33Z-07	stage ii	t2	male		
TCGA-B4-5377-01A-01R-1503-07	stage iv	t3	female		
TCGA-2Z-A9JM-01A-12R-A42S-07	stage i	t1a	male		
TCGA-B9-A5W9-01A-11R-A28H-07	stage i	t1b	male		
TCGA-BP-4986-01A-01R-1334-07	stage i	t1a	male		
TCGA-MH-A560-01A-11R-A26U-07	stage i	t1a	male		
TCGA-UZ-A9PK-01A-11R-A38C-07	stage i	t1	male		
TCGA-A3-3387-01A-01R-1541-07	stage i	t1a	male		
TCGA-CJ-4918-01A-01R-1426-07	stage iv	t3a	male		
TCGA-A3-3306-01A-01R-0864-07	stage i	t1b	male		
TCGA-AL-3468-01A-02R-1351-07	stage ii	t2	male		
TCGA-SX-A71V-01A-11R-A33Z-07	stage i	t1	male		
TCGA-B9-A8YI-01A-21R-A37K-07	stage i	t1b	male		
TCGA-G7-7501-01A-11R-2204-07	stage iii	t3a	female		
TCGA-BP-4765-01A-01R-1289-07	stage i	t1a	male		
TCGA-CZ-5456-01A-01R-1503-07	stage ii	t2	male		
TCGA-AK-3458-01A-01R-1503-07	stage i	t1b	male		
TCGA-A4-8098-01A-11R-2404-07	stage i	t1a	male		
TCGA-A3-3329-01A-01R-0864-07	stage i	t1b	male		
TCGA-CJ-5671-01A-11R-1541-07	stage i	t1a	male		
TCGA-B8-4151-01A-01R-1188-07	stage iii	t3a	female		
TCGA-Y8-A8S1-01A-11R-A37K-07	stage i	t1a	male		

TCGA-BP-4170-01A-02R-1289-07	stage i	t1b	female		
TCGA-B0-4845-01A-01R-1277-07	stage iv	t3a	male		
TCGA-BP-4159-01A-02R-1289-07	stage i	t1b	male		
TCGA-BP-4169-01A-02R-1289-07	stage ii	t2	female		
TCGA-GK-A6C7-01A-11R-A33J-07	stage i	t1a	female		
TCGA-A4-7915-01A-11R-2204-07	stage ii	t2b	female		
TCGA-IA-A83W-01A-11R-A355-07	stage i	t1	male		
TCGA-CJ-4875-01A-01R-1305-07	stage iv	t3a	male		
TCGA-A3-3376-01A-02R-1420-07	stage i	t1a	male		
TCGA-DW-5561-01A-01R-1592-07	stage i	t1a	male		
TCGA-B1-A655-01A-11R-A31O-07	stage i	t1a	female		
TCGA-BP-4158-01A-02R-1289-07	stage i	t1b	male		
TCGA-G7-7502-01A-11R-2204-07	stage i	t1b	male		
TCGA-BP-4341-01A-01R-1289-07	stage iii	t3a	male		
TCGA-BQ-7056-01A-11R-1965-07	stage iii	t3b	female		
TCGA-A3-3308-01A-02R-1325-07	stage iii	t3b	female		
TCGA-BP-4330-01A-01R-1289-07	stage iii	t3a	female		<25 Percentile
TCGA-B0-4713-01A-01R-1277-07	stage iii	t3b	female		
TCGA-B0-5692-01A-11R-1541-07	stage iii	t3b	female		
TCGA-BP-4352-01A-01R-1289-07	stage iv	t3b	female		
TCGA-2Z-A9JK-01A-11R-A42S-07	stage iii	t3a	male		
TCGA-UZ-A9PQ-01A-11R-A42S-07	stage iii	t2	male		
TCGA-B2-4102-01A-02R-1325-07	stage i	t1b	male		
TCGA-UZ-A9PX-01A-11R-A42S-07	stage i	t1	male		
TCGA-A3-3349-01A-01R-1188-07	stage i	t1b	female		
TCGA-Y8-A8S0-01A-11R-A37K-07	stage i	t1a	male		
TCGA-AK-3427-01A-01R-0864-07	stage i	t1a	male		
TCGA-EU-5904-01A-11R-1672-07	stage i	t1	female		
TCGA-CJ-5682-01A-11R-1541-07	stage iv	t3a	male		

TCGA-A3-3378-01A-02R-1325-07	stage i	t1	male			
TCGA-CJ-4639-01A-02R-1325-07	stage ii	t2	female			
TCGA-BP-4982-01A-01R-1334-07	stage i	t1b	male			
TCGA-B8-A54G-01A-11R-A266-07	stage i	t1a	male			
TCGA-BP-4355-01A-01R-1289-07	stage iii	t3a	female			
TCGA-A3-3320-01A-02R-1325-07	stage i	t1b	female			
TCGA-AL-3466-01A-02R-1351-07	stage iv	t3b	male			
TCGA-B4-5834-01A-11R-1672-07	stage i	t1	male			
TCGA-B0-5085-01A-01R-1334-07	stage iii	t3a	female			
TCGA-A4-A4ZT-01A-11R-A26U-07	stage i	t1b	female			
TCGA-CJ-4889-01A-01R-1305-07	stage i	t1a	female			
TCGA-Y8-A8RY-01A-11R-A37K-07	stage i	t1b	male			
TCGA-5P-A9KF-01A-11R-A42S-07	NA	t1a	male			
TCGA-G6-A8L8-01A-21R-A37O-07	stage i	t1b	female			
TCGA-DW-7842-01A-11R-A32Z-07	stage i	t1a	male			
TCGA-CZ-5451-01A-01R-1503-07	stage ii	t2	male			
TCGA-B0-4707-01A-01R-1277-07	stage iii	t3a	male			
TCGA-AT-A5NU-01A-11R-A28H-07	stage i	t1a	male			
TCGA-CZ-4864-01A-01R-1503-07	stage ii	t2	male			
TCGA-B0-4811-01A-01R-1503-07	stage iii	t3a	male			
TCGA-CJ-4635-01A-02R-1305-07	stage i	t1b	male			
TCGA-SX-A7SO-01A-11R-A355-07	stage i	t1b	male			
TCGA-BP-4795-01A-02R-1420-07	stage i	t1a	female			
TCGA-BP-4991-01A-01R-1334-07	stage i	t1a	male			
TCGA-CZ-4862-01A-01R-1305-07	stage i	t1b	male			
TCGA-A4-7584-01A-11R-2139-07	stage i	t1a	male			
TCGA-BP-4987-01A-01R-1334-07	stage i	t1b	female			
TCGA-BP-5007-01A-01R-1334-07	stage ii	t2	male			
TCGA-B9-4117-01A-02R-1351-07	NA	t2	female			

TCGA-A3-3323-01A-02R-1325-07	stage i	t1b	male		
TCGA-SX-A71R-01A-12R-A33Z-07	stage i	t1a	male		
TCGA-BP-4790-01A-01R-1305-07	stage i	t1a	male		
TCGA-WN-A9G9-01A-12R-A37K-07	stage i	t1b	male		
TCGA-B0-5710-01A-11R-1672-07	stage i	t1b	male		
TCGA-G6-A8L7-01A-11R-A37O-07	stage i	t1b	female		
TCGA-Y8-A896-01A-11R-A36F-07	stage iii	t3a	male		
TCGA-CJ-4634-01A-02R-1325-07	stage i	t1b	female		
TCGA-BP-4346-01A-01R-1289-07	stage iii	t3b	male		
TCGA-Y8-A897-01A-11R-A36F-07	stage i	t1a	female		
TCGA-CJ-4892-01A-01R-1305-07	stage i	t1b	female		
TCGA-BP-4967-01A-01R-1334-07	stage iii	t3a	male		
TCGA-DZ-6135-01A-11R-1965-07	stage i	t1a	male		
TCGA-A4-A57E-01A-11R-A26U-07	stage iv	t2a	male		
TCGA-B9-4113-01A-01R-1193-07	stage i	t1	male		
TCGA-BP-4971-01A-01R-1334-07	stage iii	t3a	male		
TCGA-BP-5181-01A-01R-1426-07	stage i	t1b	female		
TCGA-B0-4824-01A-01R-1277-07	stage i	t1a	female		
TCGA-DZ-6133-01A-11R-1965-07	stage i	t1a	female		
TCGA-G7-6797-01A-11R-1965-07	stage iii	t1a	male		
TCGA-CJ-5680-01A-11R-1541-07	stage iv	t3a	female		
TCGA-BP-4327-01A-01R-1289-07	stage ii	t2	female		
TCGA-A3-3351-01A-02R-1325-07	stage ii	t2a	male		
TCGA-MH-A55W-01A-11R-A26U-07	stage i	t1b	male		
TCGA-A3-A8OW-01A-11R-A37O-07	stage iii	t3a	male		
TCGA-CW-5583-01A-02R-1541-07	stage i	t1a	female		
TCGA-UZ-A9Q1-01A-11R-A42S-07	stage i	t1b	female		
TCGA-DV-5567-01A-01R-1541-07	stage i	t1a	female		
TCGA-B8-A54E-01A-11R-A266-07	stage i	t1b	female		

TCGA-SX-A7SS-01A-11R-A36F-07	stage i	t1	male			
TCGA-HE-7130-01A-11R-1965-07	stage iii	t3	female			
TCGA-2Z-A9J9-01A-11R-A42S-07	stage i	t1a	male			
TCGA-AK-3453-01A-02R-1277-07	stage ii	t2	female			
TCGA-BP-4769-01A-01R-1289-07	stage i	t1a	male			
TCGA-B0-4714-01A-01R-1277-07	stage iv	t3b	male			
TCGA-B0-5100-01A-01R-1420-07	stage iii	t3a	male			
TCGA-B8-5159-01A-01R-1420-07	stage i	t1a	female			
TCGA-BQ-7050-01A-11R-1965-07	stage i	t1a	female			
TCGA-J7-A8I2-01A-12R-A36F-07	stage i	t1b	male			
TCGA-SX-A7SR-01A-12R-A36F-07	stage ii	t2a	male			
TCGA-KO-8405-01A-11R-2315-07	stage iii	t3a	male			
TCGA-P4-A5ED-01A-11R-A28H-07	stage i	t1a	male			
TCGA-B2-5636-01A-02R-1541-07	stage i	t1a	male			
TCGA-BP-4969-01A-01R-1334-07	stage i	t1a	female			
TCGA-A3-3382-01A-02R-1325-07	stage i	t1b	male			
TCGA-A4-A5DU-01A-11R-A28H-07	stage i	t1a	female			
TCGA-B8-A54F-01A-11R-A266-07	stage i	t1a	female			
TCGA-BP-5001-01A-01R-1334-07	stage i	t1b	female			
TCGA-BP-4988-01A-01R-1334-07	stage i	t1a	male			
TCGA-BP-4995-01A-01R-1334-07	stage i	t1b	male			
TCGA-B0-4813-01A-01R-1277-07	stage iii	t3b	male			
TCGA-B0-5691-01A-11R-1541-07	stage i	t1a	female			
TCGA-CJ-4638-01A-02R-1325-07	stage iv	t3a	female			
TCGA-P4-AAVL-01A-11R-A42S-07	stage iii	t3b	male			
TCGA-G7-6793-01A-11R-1965-07	stage iv	t3a	female			
TCGA-6D-AA2E-01A-11R-A37O-07	stage i	t1b	female			
TCGA-KL-8344-01A-11R-2315-07	stage iii	t3a	male			
TCGA-Y8-A894-01A-11R-A36F-07	stage i	t1a	female			

TCGA-AK-3456-01A-02R-1325-07	stage ii	t2	male		
TCGA-2Z-A9JN-01A-21R-A42S-07	stage i	t1a	female		
TCGA-AS-3777-01A-01R-0864-07	stage i	t1a	male		
TCGA-BQ-7058-01A-11R-1965-07	stage iii	t3	male		
TCGA-BQ-7055-01A-11R-1965-07	stage i	t1a	male		
TCGA-DV-A4VX-01A-11R-A266-07	stage iv	t3b	male		
TCGA-B2-A4SR-01A-11R-A266-07	stage ii	t2a	male		
TCGA-AL-3471-01A-02R-1351-07	stage i	t1b	male		
TCGA-G7-A8LB-01A-11R-A36F-07	stage iv	t2a	male		
TCGA-BQ-5887-01A-11R-1965-07	stage iii	t3a	male		
TCGA-B0-5083-01A-02R-1420-07	stage i	t1a	male		
TCGA-CW-5585-01A-01R-1541-07	stage iv	t3b	male		
TCGA-BP-4351-01A-01R-1289-07	stage iii	t3a	female		
TCGA-G7-A8LD-01A-11R-A36F-07	stage iii	t3a	male		
TCGA-EV-5902-01A-11R-1592-07	stage i	t1	male		
TCGA-B8-A54D-01A-21R-A266-07	stage iii	t3a	male		
TCGA-A3-3328-01A-01R-0864-07	stage i	t1b	male		
TCGA-B0-4834-01A-01R-1305-07	stage i	t1a	male		
TCGA-BQ-5884-01A-11R-1592-07	stage i	t1a	female		
TCGA-KO-8413-01A-11R-2315-07	stage i	t1	male		
TCGA-T7-A92I-01A-11R-A37O-07	stage i	t1a	female		
TCGA-KN-8428-01A-11R-2315-07	stage ii	t2	male		
TCGA-B8-A54K-01A-11R-A33J-07	stage i	t1a	male		
TCGA-B8-5546-01A-01R-1541-07	stage i	t1b	female		
TCGA-AK-3443-01A-02R-1325-07	stage ii	t2	male		
TCGA-WN-AB4C-01A-11R-A42S-07	stage i	t1a	female		
TCGA-B8-4619-01A-02R-1325-07	stage i	t1a	male		
TCGA-B1-A47N-01A-11R-A24Z-07	stage i	t1a	male		
TCGA-A4-8312-01A-11R-2404-07	stage i	t1	male		

TCGA-MH-A857-01A-11R-A355-07	stage i	t1a	male		
TCGA-BP-4994-01A-01R-1334-07	stage i	t1a	male		
TCGA-BQ-5883-01A-11R-1592-07	stage i	t1a	female		
TCGA-KL-8336-01A-11R-2315-07	stage iv	t3b	female		
TCGA-KM-8441-01A-11R-2315-07	stage i	t1b	female		
TCGA-KL-8343-01A-11R-2315-07	stage iii	t3	male		
TCGA-B0-4817-01A-01R-1277-07	stage iii	t3c	male		
TCGA-KL-8326-01A-11R-2315-07	stage iii	t3a	male		
TCGA-AK-3440-01A-02R-1277-07	stage i	t1a	male		
TCGA-KM-8639-01A-11R-2403-07	stage i	t1a	male		
TCGA-KO-8415-01A-11R-2315-07	stage i	t1	female		
TCGA-KL-8332-01A-11R-2315-07	stage i	t1b	male		
TCGA-KO-8410-01A-11R-2315-07	stage i	t1b	female		
TCGA-KO-8403-01A-11R-2315-07	stage i	t1a	male		
TCGA-KM-8477-01A-11R-2315-07	stage i	t1a	male		
TCGA-KN-8419-01A-11R-2315-07	stage ii	t2	male		
TCGA-KL-8329-01A-11R-2315-07	stage i	t1b	female		
TCGA-AK-3465-01A-02R-1325-07	stage i	t1b	female		
TCGA-KN-8426-01A-11R-2315-07	stage iv	t3a	male		
TCGA-KL-8324-01A-11R-2315-07	stage ii	t2	female		
TCGA-KN-8430-01A-11R-2315-07	stage i	t1b	male		
TCGA-B0-5117-01A-01R-1420-07	stage i	t1b	male		
TCGA-KN-8424-01A-11R-2315-07	stage i	t1b	female		
TCGA-AK-3447-01A-01R-1766-07	stage ii	t2	male		
TCGA-B2-3923-01A-02R-1325-07	stage ii	t2	male		
TCGA-KN-8433-01A-11R-2315-07	stage iii	t3a	female		
TCGA-B4-5832-01A-11R-1672-07	stage iii	t3b	male		
TCGA-KL-8338-01A-11R-2315-07	stage iii	t3a	male		
TCGA-KO-8408-01A-11R-2315-07	stage iii	t3a	male		

TCGA-KM-8476-01A-11R-2315-07	stage i	t1	male			
TCGA-KN-8437-01A-11R-2315-07	stage i	t1a	female			
TCGA-KM-8442-01A-11R-2315-07	stage ii	t2	male			
TCGA-KL-8335-01A-11R-2315-07	stage iii	t3a	male			
TCGA-KL-8342-01A-11R-2315-07	stage ii	t2b	female			
TCGA-KN-8425-01A-11R-2315-07	stage i	t1b	male			
TCGA-KL-8334-01A-11R-2315-07	stage iii	t3a	female			
TCGA-KL-8339-01A-11R-2315-07	#N/A	#N/A	#N/A			
TCGA-KO-8411-01A-11R-2315-07	stage i	t1b	male			
TCGA-KL-8337-01A-11R-2315-07	stage ii	t2	male			
TCGA-KM-8439-01A-11R-2315-07	stage i	t1b	male			
TCGA-AK-3433-01A-02R-1277-07	stage ii	t2	female			
TCGA-KN-8434-01A-11R-2315-07	stage ii	t2	female			
TCGA-KL-8341-01A-11R-2315-07	stage iv	t3b	male			
TCGA-KL-8323-01A-21R-2315-07	stage iii	t3b	female			
TCGA-KM-8438-01A-11R-2315-07	stage ii	t2	female			
TCGA-KO-8407-01A-11R-2315-07	stage ii	t2	male			
TCGA-KO-8416-01A-11R-2315-07	stage iii	t3a	male			
TCGA-KN-8436-01A-11R-2315-07	stage ii	t2	male			
TCGA-KN-8423-01A-11R-2315-07	stage i	t1b	male			
TCGA-CZ-5989-01A-11R-1672-07	stage ii	t2	male			
TCGA-KL-8327-01A-11R-2315-07	stage i	t1b	female			
TCGA-KO-8414-01A-11R-2315-07	stage ii	t2	female			
TCGA-KL-8331-01A-11R-2315-07	stage ii	t2	female			
TCGA-KN-8429-01A-11R-2315-07	stage iii	t3a	female			
TCGA-KO-8406-01A-11R-2315-07	stage i	t1	female			
TCGA-KL-8346-01A-11R-2315-07	stage ii	t2b	male			
TCGA-KN-8418-01A-11R-2315-07	stage ii	t2	female			
TCGA-KL-8340-01A-11R-2315-07	stage ii	t2	male			

TCGA-KN-8435-01A-11R-2315-07	stage ii	t2	male		
TCGA-BP-4334-01A-01R-1289-07	stage iii	t3a	male		
TCGA-KO-8417-01A-11R-2315-07	stage i	t1	female		
TCGA-KN-8421-01A-11R-2315-07	stage ii	t2	female		
TCGA-KO-8409-01A-11R-2315-07	stage ii	t2b	male		
TCGA-KL-8345-01A-11R-2315-07	stage iii	t3a	male		
TCGA-KM-8443-01A-11R-2315-07	stage ii	t2	male		
TCGA-KN-8431-01A-11R-2315-07	stage ii	t2	female		
TCGA-KN-8432-01A-11R-2315-07	stage ii	t2b	female		
TCGA-KL-8330-01A-11R-2315-07	stage ii	t2b	female		
TCGA-KL-8325-01A-11R-2315-07	stage ii	t2	female		
TCGA-KM-8440-01A-11R-2315-07	stage iii	t3a	male		
TCGA-KN-8422-01A-11R-2315-07	stage i	t1a	female		
TCGA-KN-8427-01A-11R-2315-07	stage iii	t3	female		
TCGA-GL-7966-01A-11R-2204-07	stage i	t1a	female		
TCGA-CZ-5982-01A-11R-1672-07	stage iv	t4	male		
TCGA-CZ-4860-01A-01R-1305-07	stage iii	t3a	female		
TCGA-BP-4983-01A-01R-1334-07	stage iv	t4	male		
TCGA-B0-4698-01A-01R-1503-07	stage iii	t3a	male		
TCGA-G7-A8LD-01A-11R-A36F-07	stage iv	t3a	male		
TCGA-BQ-5893-01A-11R-1592-07	stage ii	t2	female	WDR43	>75 percentile
TCGA-CJ-6032-01A-11R-1672-07	stage iii	t3b	male		
TCGA-B4-5832-01A-11R-1672-07	stage ii	t2	male		
TCGA-B3-4104-01A-02R-1351-07	stage i	t1b	male		
TCGA-A3-3313-01A-02R-1325-07	stage iii	t3	female		
TCGA-J7-8537-01A-11R-2404-07	stage iv	t4	male		
TCGA-BP-4354-01A-02R-1289-07	stage i	t1a	male		
TCGA-CW-6093-01A-11R-1672-07	stage iv	t3b	male		
TCGA-BQ-5894-01A-11R-1592-07	stage ii	t2	male		

TCGA-CZ-5989-01A-11R-1672-07	stage i	t1a	male		
TCGA-BQ-5876-01A-11R-1592-07	stage iii	t3a	female		
TCGA-CJ-4878-01A-01R-1305-07	stage iii	t3b	male		
TCGA-BP-4799-01A-01R-1305-07	stage iv	t3a	female		
TCGA-CJ-4904-01A-02R-1426-07	stage iii	t3b	male		
TCGA-AK-3428-01A-02R-1277-07	stage iii	t3	female		
TCGA-HE-7130-01A-11R-1965-07	stage i	t1	female		
TCGA-CZ-4859-01A-02R-1426-07	stage iii	t3a	female		
TCGA-IA-A40Y-01A-11R-A24Z-07	stage i	t1b	female		
TCGA-A4-A5Y0-01A-11R-A31O-07	stage iii	t3a	male		
TCGA-DZ-6131-01A-11R-1965-07	stage iii	t3b	male		
TCGA-B0-5109-01A-02R-1420-07	stage iv	t4	male		
TCGA-B0-4688-01A-01R-1277-07	stage iv	t3a	female		
TCGA-B0-4701-01A-01R-1277-07	stage iii	t3b	male		
TCGA-BP-5198-01A-01R-1426-07	stage i	t1a	male		
TCGA-DV-5565-01A-01R-1541-07	stage iii	t3a	female		
TCGA-F9-A4JJ-01A-11R-A24Z-07	stage i	t1	female		
TCGA-B0-5104-01A-01R-1420-07	stage iv	t3	female		
TCGA-B4-5377-01A-01R-1503-07	stage ii	t2	male		
TCGA-A3-3317-01A-02R-1325-07	stage iv	t4	female		
TCGA-F9-A8NY-01A-11R-A36F-07	stage ii	t2	male		
TCGA-KL-8333-01A-11R-2315-07	stage iii	t3a	male		
TCGA-CZ-5457-01A-01R-1503-07	stage iii	t3a	male		
TCGA-2Z-A9JI-01A-11R-A42S-07	stage iii	t3b	male		
TCGA-BQ-5882-01A-11R-1592-07	stage i	t1	female		
TCGA-B0-5098-01A-01R-1420-07	stage ii	t2	male		
TCGA-V9-A7HT-01A-11R-A33Z-07	stage iv	t3a	male		
TCGA-B0-5084-01A-01R-1334-07	stage ii	t2	male		
TCGA-CZ-5463-01A-01R-1503-07	stage i	t1b	male		

TCGA-A3-3306-01A-01R-0864-07	stage i	t1b	female		
TCGA-KO-8410-01A-11R-2315-07	stage iii	t3b	female		
TCGA-B0-5099-01A-01R-1420-07	stage iii	t3a	female		
TCGA-BP-4164-01A-02R-1325-07	stage ii	t2	female		
TCGA-B0-4852-01A-01R-1503-07	stage i	t1b	female		
TCGA-B0-5693-01A-11R-1541-07	stage i	t1b	male		
TCGA-DW-7838-01A-11R-2139-07	stage iii	t3a	male		
TCGA-CW-6097-01A-11R-1672-07	stage i	t1b	female		
TCGA-A3-3373-01A-02R-1420-07	stage iii	t3b	female		
TCGA-B0-5097-01A-01R-1420-07	stage iv	t3b	male		
TCGA-AL-3466-01A-02R-1351-07	stage i	t1	female		
TCGA-A4-7287-01A-11R-2139-07	stage i	t1	female		
TCGA-B4-5835-01A-11R-1672-07	stage i	t1a	male		
TCGA-BP-4765-01A-01R-1289-07	stage i	t1b	male		
TCGA-A3-3382-01A-02R-1325-07	stage i	t1a	female		
TCGA-DV-5569-01A-01R-1541-07	stage iv	t4	male		
TCGA-B0-4690-01A-01R-1277-07	stage i	t1b	male		
TCGA-BP-4338-01A-01R-1289-07	stage ii	t2	male		
TCGA-BP-5200-01A-01R-1426-07	stage iii	t3a	male		
TCGA-BP-5202-01A-02R-1426-07	stage i	t1b	male		
TCGA-5P-A9K9-01A-11R-A42S-07	stage iii	t3a	female		
TCGA-AL-A5DJ-01A-11R-A26U-07	stage iii	t3a	female		
TCGA-B8-4620-01A-02R-1325-07	stage i	t1	male		
TCGA-B4-5843-01A-11R-1672-07	stage i	t1a	male		
TCGA-G7-6790-01A-11R-1965-07	stage i	t1	male		
TCGA-A3-3311-01A-02R-1325-07	stage iii	t3a	male		
TCGA-B0-4696-01A-01R-1277-07	stage iii	t3a	male		
TCGA-B8-5550-01A-01R-1541-07	stage i	t1a	male		
TCGA-A3-3365-01A-01R-0864-07	stage i	t1a	female		

TCGA-B8-4148-01A-02R-1325-07	stage iii	t3a	male		
TCGA-BP-4160-01A-02R-1289-07	stage iii	t3b	male		
TCGA-BP-4347-01A-01R-1289-07	stage iii	t3a	male		
TCGA-EU-5907-01A-11R-1672-07	stage i	t1a	female		
TCGA-BP-4763-01A-01R-1289-07	stage iii	t3a	male		
TCGA-BP-4343-01A-02R-1289-07	stage iii	t3a	female		
TCGA-G7-7501-01A-11R-2204-07	stage i	t1a	female		
TCGA-BP-4775-01A-01R-1289-07	stage i	t1a	male		
TCGA-CJ-6027-01A-11R-1672-07	stage iv	t1b	male		
TCGA-CZ-5461-01A-01R-1503-07	stage i	t1a	male		
TCGA-AK-3461-01A-02R-1277-07	stage iii	t3b	male		
TCGA-A3-3307-01A-01R-0864-07	stage i	t1b	female		
TCGA-B8-A8YJ-01A-13R-A39I-07	stage i	t1a	male		
TCGA-A4-8098-01A-11R-2404-07	stage i	t1b	male		
TCGA-B0-5703-01A-11R-1541-07	stage iv	t3a	female		
TCGA-CW-5580-01A-01R-1672-07	stage iii	t3b	female		
TCGA-BQ-5879-01A-11R-1592-07	stage i	t1a	female		
TCGA-BP-4768-01A-01R-1289-07	stage iii	t3b	male		
TCGA-B0-4813-01A-01R-1277-07	stage i	t1a	male		
TCGA-BP-5006-01A-01R-1334-07	stage i	t1b	male		
TCGA-B9-4116-01A-02R-1351-07	stage i	t1b	female		
TCGA-AK-3444-01A-02R-1325-07	stage iii	t3a	female		
TCGA-CZ-5467-01A-01R-1503-07	stage iii	t3b	male		
TCGA-BP-4797-01A-01R-1305-07	stage i	t1a	female		
TCGA-B8-A7U6-01A-12R-A37O-07	stage iii	t3b	female		
TCGA-CZ-5465-01A-01R-1503-07	stage i	t1a	female		
TCGA-A3-A6NJ-01A-12R-A33J-07	stage iii	t3a	female		
TCGA-B0-5096-01A-01R-1420-07	stage i	t1a	male		
TCGA-BP-5004-01A-01R-1334-07	stage i	t1b	female		

TCGA-CJ-4893-01A-01R-1305-07	stage ii	t2a	male		
TCGA-B8-5162-01A-01R-1420-07	stage i	t1b	female		
TCGA-BP-4170-01A-02R-1289-07	stage iii	t3a	female		
TCGA-B0-5113-01A-01R-1420-07	stage i	t1a	male		
TCGA-BP-5008-01A-01R-1334-07	stage i	t1b	female		
TCGA-BP-4162-01A-02R-1325-07	stage iii	t3a	male		
TCGA-B0-4849-01A-01R-1277-07	stage i	t1	male		
TCGA-BP-4353-01A-02R-1289-07	stage ii	t2	male		
TCGA-CZ-4861-01A-01R-1305-07	stage i	t1b	male		
TCGA-DV-A4W0-01A-11R-A266-07	stage i	t1b	female		
TCGA-B2-4098-01A-02R-1325-07	stage iv	t3a	male		
TCGA-CW-6087-01A-11R-1672-07	stage iii	t3b	female		
TCGA-A3-3308-01A-02R-1325-07	stage i	t1a	male		
TCGA-BP-4760-01A-02R-1420-07	stage iii	t3a	male		
TCGA-BP-4985-01A-01R-1334-07	stage i	t1	male		
TCGA-SX-A71V-01A-11R-A33Z-07	stage i	t1b	male		
TCGA-CZ-4854-01A-01R-1305-07	stage iv	t3a	male		
TCGA-B0-4846-01A-01R-1277-07	stage i	t1a	male		
TCGA-B0-4823-01A-02R-1420-07	stage i	t1b	male		
TCGA-B2-3924-01A-02R-1325-07	stage i	t1b	female		
TCGA-B0-4838-01A-01R-1305-07	stage i	t1b	male		
TCGA-BP-4959-01A-01R-1334-07	stage ii	t2	male		
TCGA-BP-4174-01A-02R-1289-07	stage iv	t3	male		
TCGA-B4-5838-01A-11R-1672-07	stage i	t1	male		
TCGA-A3-3380-01A-01R-0864-07	stage ii	t2b	male		
TCGA-CJ-4876-01A-01R-1305-07	stage iv	t3a	male		
TCGA-SX-A7SM-01A-11R-A355-07	stage iii	t3a	male		
TCGA-BP-4989-01A-01R-1334-07	stage iii	t3a	female		
TCGA-Q2-A5QZ-01A-11R-A28H-07	stage i	t1a	female		

TCGA-A3-A8CQ-01A-11R-A37O-07	stage i	t1a	male			
TCGA-BP-4781-01A-01R-1305-07	stage iv	t3a	male			
TCGA-CJ-4890-01A-01R-1305-07	stage iii	t3b	male			
TCGA-B0-5116-01A-02R-1420-07	stage i	t1b	male			
TCGA-CJ-4903-01A-01R-1426-07	stage iii	t3c	female			
TCGA-CJ-4891-01A-01R-1305-07	stage i	t1a	male			
TCGA-BP-4961-01A-01R-1334-07	stage i	t1a	male			
TCGA-B0-4834-01A-01R-1305-07	stage i	t1b	female			
TCGA-BP-4340-01A-01R-1289-07	stage ii	t2	male			
TCGA-AK-3456-01A-02R-1325-07	stage i	t1b	male			
TCGA-A3-3324-01A-02R-1325-07	stage iii	t3	male			
TCGA-A3-3372-01A-02R-1325-07	stage i	t1a	male			
TCGA-CJ-4908-01A-01R-1426-07	stage iii	t3b	female			
TCGA-CW-5587-01A-01R-1541-07	stage i	t1a	female			
TCGA-BP-4774-01A-01R-1289-07	stage iii	t3a	female			
TCGA-BP-4163-01A-02R-1325-07	stage iii	t1a	male			
TCGA-BP-4970-01A-01R-1334-07	stage iii	t1b	female			
TCGA-A3-3347-01A-02R-1325-07	stage i	t1	male			
TCGA-CZ-5986-01A-11R-1672-07	stage ii	t2	female			
TCGA-AK-3431-01A-02R-1277-07	stage iv	t3a	male			
TCGA-CW-5590-01A-01R-1541-07	stage i	t1a	female			
TCGA-PJ-A5Z8-01A-11R-A28H-07	stage i	t1b	female			
TCGA-BP-4325-01A-02R-1289-07	stage i	t1b	male			
TCGA-B0-5812-01A-11R-1672-07	stage i	t1a	male			
TCGA-CJ-6030-01A-11R-1672-07	stage iv	t3b	male			
TCGA-CZ-5455-01A-01R-1503-07	stage ii	t2	male			
TCGA-A3-3316-01A-01R-0864-07	stage iii	t3b	male			
TCGA-CJ-4901-01A-01R-1426-07	stage iv	t4	female			
TCGA-BP-4770-01A-01R-1503-07	stage ii	t2	male			

TCGA-A3-3357-01A-02R-1420-07	stage i	t1a	male		
TCGA-DV-A4VZ-01A-11R-A266-07	stage ii	t2	female		
TCGA-AK-3429-01A-02R-1325-07	stage ii	t2	male		
TCGA-CZ-4858-01A-01R-1305-07	stage i	t1a	female		
TCGA-BQ-7050-01A-11R-1965-07	stage i	t1a	female		
TCGA-B0-5110-01A-01R-1420-07	stage i	t1	male		
TCGA-B4-5378-01A-01R-1503-07	stage i	t1	male		
TCGA-AK-3425-01A-02R-1277-07	stage iii	t3a	male		
TCGA-CJ-4894-01A-01R-1305-07	stage i	t1b	male		
TCGA-BP-4176-01A-02R-1289-07	stage i	t1a	female		
TCGA-B0-4945-01A-01R-1420-07	stage iii	t3a	male		
TCGA-BP-4332-01A-01R-1289-07	stage i	t1a	male		
TCGA-A3-3387-01A-01R-1541-07	stage iv	t3b	male		
TCGA-BP-5201-01A-01R-1426-07	stage i	t1a	male		
TCGA-BP-5187-01A-01R-1426-07	stage i	t1b	male		
TCGA-CJ-4899-01A-01R-1334-07	stage iii	t3a	female		
TCGA-B0-4693-01A-01R-1277-07	stage i	t1b	female		
TCGA-KM-8441-01A-11R-2315-07	stage ii	t2	female		
TCGA-BP-4169-01A-02R-1289-07	stage iii	t3a	male		
TCGA-B0-4815-01A-01R-1503-07	stage i	t1b	female		
TCGA-B8-5551-01A-01R-1541-07	stage i	t1b	male		
TCGA-A3-3319-01A-02R-1325-07	stage i	t1a	female		
TCGA-DV-5575-01A-01R-1541-07	stage i	t1a	male		
TCGA-BP-4777-01A-01R-1289-07	stage i	t1b	female		
TCGA-B8-5546-01A-01R-1541-07	stage i	t1a	female		
TCGA-A3-3385-01A-02R-1420-07	stage iv	t3a	male		
TCGA-CJ-4918-01A-01R-1426-07	stage i	t1a	female		
TCGA-CW-5588-01A-01R-1541-07	stage i	t1b	male		
TCGA-CW-6088-01A-11R-1672-07	stage i	t1b	male		

TCGA-2Z-A9J8-01A-11R-A42S-07	stage iii	t3a	male		
TCGA-B0-5108-01A-01R-1420-07	stage i	t1b	male		
TCGA-BP-5189-01A-02R-1426-07	stage i	t1a	female		
TCGA-CJ-4889-01A-01R-1305-07	stage i	t1a	female		
TCGA-DV-5566-01A-01R-1541-07	stage i	t1a	female		
TCGA-WN-AB4C-01A-11R-A42S-07	stage i	t1a	female		
TCGA-B8-5159-01A-01R-1420-07	stage i	t1a	male		
TCGA-BP-5194-01A-02R-1426-07	stage i	t1a	male		
TCGA-BP-4986-01A-01R-1334-07	stage i	t1b	female		
TCGA-BP-4756-01A-01R-1289-07	stage i	t1	male		
TCGA-DW-7834-01A-11R-2139-07	stage iv	t3a	male		
TCGA-BP-4974-01A-01R-1334-07	stage iii	t3a	female		
TCGA-B0-4710-01A-01R-1503-07	stage i	t1a	male		
TCGA-BP-4991-01A-01R-1334-07	stage i	t1a	female		
TCGA-BP-4784-01A-01R-1305-07	stage i	t1b	male		
TCGA-CW-6090-01A-11R-1672-07	stage iv	t3a	male		
TCGA-BQ-5877-01A-11R-1592-07	stage i	t1b	male		
TCGA-CZ-4862-01A-01R-1305-07	stage ii	t2	female		
TCGA-B0-4818-01A-01R-1503-07	stage iii	t3a	male		
TCGA-CZ-5466-01A-01R-1503-07	stage i	t1a	male		
TCGA-AK-3427-01A-01R-0864-07	stage iii	t3a	female		
TCGA-B8-5163-01A-01R-1420-07	stage i	t1a	male		
TCGA-BP-5173-01A-01R-1426-07	stage iii	t3a	female		
TCGA-B0-5075-01A-01R-1334-07	stage i	t1a	male		
TCGA-B8-5545-01A-01R-1672-07	stage i	t1b	male		
TCGA-CZ-5988-01A-11R-1672-07	stage iii	t3a	female		
TCGA-B8-4151-01A-01R-1188-07	stage i	t1a	male		
TCGA-A3-3322-01A-02R-1325-07	stage i	t1b	female		
TCGA-5P-A9K2-01A-11R-A42S-07	stage iii	t3b	female		

TCGA-BP-4337-01A-01R-1289-07	stage iii	t3a	male		
TCGA-B9-5155-01A-01R-1592-07	stage i	t1a	male		
TCGA-B9-A5W7-01A-11R-A31O-07	stage iv	t3a	male		
TCGA-B0-4844-01A-01R-1277-07	stage i	t1	female		
TCGA-B0-5102-01A-01R-1420-07	stage i	t1b	male		
TCGA-KN-8423-01A-11R-2315-07	stage i	t1b	male		
TCGA-SX-A7SN-01A-11R-A355-07	stage i	t1b	female		
TCGA-KL-8329-01A-11R-2315-07	stage iii	t3a	male		
TCGA-P4-AAVK-01A-11R-A42S-07	stage iv	t3b	male		
TCGA-CW-5585-01A-01R-1541-07	stage i	t1	male		
TCGA-IA-A83W-01A-11R-A355-07	stage iii	t3b	male		
TCGA-B9-A44B-01A-11R-A24Z-07	stage ii	t2	male		
TCGA-KN-8435-01A-11R-2315-07	stage iv	t3b	male		
TCGA-BQ-5889-01A-11R-1592-07	stage i	t1a	male		
TCGA-B0-5083-01A-02R-1420-07	stage i	t1a	male		
TCGA-UZ-A9PP-01A-11R-A42S-07	stage i	t1a	male		
TCGA-MH-A856-01A-11R-A355-07	stage i	t1a	male		
TCGA-B8-A54G-01A-11R-A266-07	stage i	t1a	male		
TCGA-DZ-6135-01A-11R-1965-07	stage iii	t3b	female		
TCGA-BQ-7056-01A-11R-1965-07	stage iii	t3c	male		
TCGA-GL-A59R-01A-11R-A26U-07	stage i	t1b	male		
TCGA-BQ-7045-01A-31R-1965-07	stage i	t1a	female		
TCGA-MM-A84U-01A-11R-A37O-07	stage iii	t1a	male		
TCGA-G7-6797-01A-11R-1965-07	stage i	t1b	male		
TCGA-DW-7841-01A-11R-A32Z-07	stage i	t1b	male		
TCGA-B8-4621-01A-01R-1503-07	stage i	t1b	male		
TCGA-SX-A71W-01A-12R-A355-07	stage i	t1b	male		
TCGA-5P-A9K0-01A-11R-A42S-07	stage i	t1b	female		
TCGA-BQ-7061-01A-11R-1965-07	stage i	t1a	male		

TCGA-BP-4758-01A-01R-1289-07	stage i	t1b	male		
TCGA-BP-5000-01A-01R-1334-07	stage iv	t3	female		
TCGA-HE-A5NK-01A-11R-A26U-07	stage i	t1	male		
TCGA-A4-8312-01A-11R-2404-07	stage iii	t3a	male		
TCGA-BP-4341-01A-01R-1289-07	stage iii	t2	male		
TCGA-CJ-4869-01A-02R-1426-07	stage i	t1a	male		
TCGA-GL-7773-01A-11R-A32Z-07	stage iii	t3a	male		
TCGA-AK-3426-01A-02R-1325-07	stage i	t1a	male		
TCGA-B9-A8YH-01A-11R-A37K-07	stage i	t1b	female		
TCGA-KN-8424-01A-11R-2315-07	stage i	t1	male		
TCGA-UZ-A9PV-01A-11R-A42S-07	stage iv	t1a	female		
TCGA-B0-5092-01A-01R-1420-07	stage iii	t3a	female		
TCGA-B0-5085-01A-01R-1334-07	stage ii	t2b	female		
TCGA-MH-A855-01A-11R-A355-07	stage i	t1	male		
TCGA-UZ-A9PX-01A-11R-A42S-07	stage i	t1a	male		
TCGA-DW-7842-01A-11R-A32Z-07	stage iii	t3a	male		
TCGA-BQ-5880-01A-11R-1592-07	stage i	t1a	male		
TCGA-Y8-A8S0-01A-11R-A37K-07	stage ii	t2	male		
TCGA-KN-8419-01A-11R-2315-07	stage iv	t3a	male		
TCGA-CJ-4887-01A-01R-1305-07	stage ii	t2	male		
TCGA-AL-3473-01A-01R-1193-07	stage i	t1a	male		
TCGA-AS-3777-01A-01R-0864-07	stage iii	t3	male		
TCGA-BQ-5885-01A-11R-1592-07	stage i	t1b	male		
TCGA-GL-A9DE-01A-11R-A37K-07	stage i	t1b	male		
TCGA-KN-8425-01A-11R-2315-07	stage ii	t2	male		
TCGA-UZ-A9PM-01A-21R-A38C-07	stage i	t1	male		
TCGA-J7-6720-01A-11R-2139-07	stage i	t1a	male		
TCGA-A4-8517-01A-11R-2404-07	stage ii	t2	male		
TCGA-B0-4822-01A-01R-1277-07	stage i	t1a	male		

TCGA-BP-5190-01A-01R-1426-07	stage iii	t3	female		
TCGA-BQ-7053-01A-11R-1965-07	stage i	t1a	male		
TCGA-B9-4115-01A-01R-1193-07	stage i	t1b	female		
TCGA-5P-A9KF-01A-11R-A42S-07	stage i	t1a	male		
TCGA-A4-7584-01A-11R-2139-07	stage iii	t3a	male		
TCGA-CJ-4882-01A-02R-1426-07	stage i	t1a	male		
TCGA-A4-8515-01A-11R-2404-07	stage iii	t3a	male		
TCGA-KO-8405-01A-11R-2315-07	stage i	t1b	male		
TCGA-KN-8430-01A-11R-2315-07	stage i	t1a	male		
TCGA-5P-A9K4-01A-11R-A42S-07	stage i	t1a	female		
TCGA-BQ-7049-01A-11R-1965-07	stage ii	t2a	male		
TCGA-5P-A9JW-01A-11R-A42S-07	stage iii	t3b	male		
TCGA-B1-5398-01A-02R-1592-07	stage i	t1a	male		
TCGA-BP-4776-01A-01R-1289-07	stage i	t1a	female		
TCGA-F9-A7VF-01A-11R-A33Z-07	stage iii	t3a	male		
TCGA-KL-8345-01A-11R-2315-07	stage iii	t3a	male		
TCGA-A4-8516-01A-11R-2404-07	stage ii	t2	female		
TCGA-5P-A9KA-01A-11R-A42S-07	stage iii	t3a	male		
TCGA-2Z-A9JK-01A-11R-A42S-07	stage iii	t3a	male		
TCGA-Y8-A896-01A-11R-A36F-07	stage i	t1a	male		
TCGA-2Z-A9JT-01A-11R-A42S-07	stage iv	t3b	female		
TCGA-KL-8336-01A-11R-2315-07	stage iii	t3	male		
TCGA-BQ-7058-01A-11R-1965-07	stage iv	t2	female		
TCGA-B0-5712-01A-11R-1672-07	stage i	t1a	male		
TCGA-MH-A560-01A-11R-A26U-07	stage i	t1a	female		
TCGA-A4-A5DU-01A-11R-A28H-07	stage i	t1a	male		
TCGA-BQ-7060-01A-11R-1965-07	stage iii	t3a	male		
TCGA-A4-7732-01A-11R-2139-07	stage iii	t3a	male		
TCGA-KL-8326-01A-11R-2315-07	stage i	t1b	male		

TCGA-B0-5702-01A-11R-1541-07	stage i	t1b	female		
TCGA-BP-4165-01A-02R-1289-07	stage i	t1a	male		
TCGA-UZ-A9PJ-01A-11R-A38C-07	stage iv	t3a	male		
TCGA-B0-4847-01A-01R-1277-07	stage i	t1a	male		
TCGA-BQ-7062-01A-11R-1965-07	stage i	t1a	female		
TCGA-5P-A9KE-01A-11R-A42S-07	stage iii	t3a	female		
TCGA-CJ-4873-01A-01R-1305-07	stage iv	t3b	male		
TCGA-KL-8341-01A-11R-2315-07	stage iii	t3b	female		
TCGA-KL-8323-01A-21R-2315-07	stage iv	t2a	male		
TCGA-A4-A57E-01A-11R-A26U-07	stage i	t1a	male		
TCGA-G7-A8LE-01A-11R-A36F-07	stage i	t1a	male		
TCGA-HE-A5NL-01A-11R-A26U-07	stage iv	t3b	male		
TCGA-B0-4714-01A-01R-1277-07	stage i	t1b	male		
TCGA-KM-8439-01A-11R-2315-07	stage i	t1b	male		
TCGA-B1-A657-01A-11R-A31O-07	stage iii	t3a	male		
TCGA-BP-4167-01A-02R-1325-07	stage i	t1b	female		
TCGA-MH-A854-01A-11R-A355-07	stage i	t1a	male		
TCGA-5P-A9KH-01A-11R-A42S-07	stage iii	t3a	male		
TCGA-BP-5191-01A-01R-1426-07	stage iv	t2a	male		
TCGA-G7-A8LB-01A-11R-A36F-07	stage i	t1b	male		
TCGA-IA-A83V-01A-11R-A355-07	stage i	t1b	male		
TCGA-KO-8411-01A-11R-2315-07	stage i	t1a	male		
TCGA-2Z-A9JE-01A-11R-A42S-07	stage i	t1	male		
TCGA-A3-3383-01A-02R-1325-07	stage iv	t3a	male		
TCGA-A4-7286-01A-11R-A32Z-07	stage i	t1	male		
TCGA-KO-8413-01A-11R-2315-07	stage iii	t3a	male		
TCGA-BQ-5886-01A-11R-1592-07	stage ii	t2	male		
TCGA-KM-8443-01A-11R-2315-07	stage ii	t2a	male		
TCGA-5P-A9K8-01A-11R-A42S-07	stage i	t1a	female		

TCGA-4A-A93W-01A-11R-A37K-07	stage iii	t3b	female		
TCGA-B0-4713-01A-01R-1277-07	stage i	t1b	male		
TCGA-Y8-A895-01A-11R-A36F-07	stage ii	t2	female		
TCGA-KN-8418-01A-11R-2315-07	stage ii	t2	female		
TCGA-KL-8325-01A-11R-2315-07	stage i	t1	male		
TCGA-UZ-A9Q0-01A-12R-A42S-07	stage iii	t3a	male		
TCGA-KM-8440-01A-11R-2315-07	stage i	t1b	male		
TCGA-5P-A9JY-01A-11R-A42S-07	stage iii	t3b	female		
TCGA-B0-5692-01A-11R-1541-07	stage i	t1a	male		
TCGA-A4-8518-01A-11R-2404-07	stage i	t1a	female		
TCGA-Y8-A897-01A-11R-A36F-07	stage i	t1b	female		
TCGA-A3-3374-01A-02R-1325-07	stage i	t1a	male		
TCGA-KM-8639-01A-11R-2403-07	stage iii	t3a	female		
TCGA-B0-4842-01A-02R-1420-07	stage i	t1a	male		
TCGA-2Z-A9JR-01A-12R-A42S-07	stage iii	t1b	male		
TCGA-P4-A5E6-01A-11R-A28H-07	stage i	t1b	female		
TCGA-B0-4839-01A-01R-1305-07	stage i	t1b	female		
TCGA-PJ-A5Z9-01A-11R-A28H-07	stage i	t1b	male		
TCGA-Y8-A8RY-01A-11R-A37K-07	#N/A	#N/A	#N/A		
TCGA-KL-8328-01A-11R-2315-07	stage ii	t2	male		
TCGA-UZ-A9PL-01A-11R-A38C-07	stage i	t1a	male		
TCGA-IZ-A6M9-01A-11R-A31O-07	stage i	t1a	male		
TCGA-BQ-5881-01A-11R-1592-07	stage i	t1	female		
TCGA-KO-8406-01A-11R-2315-07	stage ii	t2	male		
TCGA-KL-8337-01A-11R-2315-07	stage i	t1a	male		
TCGA-DW-7836-01A-11R-2139-07	stage i	t1	female		
TCGA-KO-8417-01A-11R-2315-07	stage i	t1a	male		
TCGA-G7-6795-01A-11R-1965-07	stage iii	t3b	female		
TCGA-B0-5400-01A-01R-1503-07	stage i	t1b	male		

TCGA-A4-A772-01A-11R-A33Z-07	stage i	t1b	male			
TCGA-A3-3328-01A-01R-0864-07	stage i	t1b	male			
TCGA-DW-7840-01A-11R-A32Z-07	stage i	t1a	male			
TCGA-G7-6796-01A-11R-1965-07	stage i	t1a	male			
TCGA-2Z-A9J1-01A-11R-A38C-07	stage i	t1a	male			
TCGA-2Z-A9J6-01A-11R-A38C-07	stage ii	t2b	male			
TCGA-B9-A5W8-01A-11R-A28H-07	stage i	t1b	male			
TCGA-KL-8332-01A-11R-2315-07	stage ii	t2b	female			
TCGA-KL-8330-01A-11R-2315-07	NA	t2	male			
TCGA-5P-A9K3-01A-11R-A42S-07	stage ii	t2	female			
TCGA-KL-8331-01A-11R-2315-07	stage i	t1b	female			
TCGA-B1-A47O-01A-11R-A24Z-07	stage ii	t2	male			
TCGA-KO-8407-01A-11R-2315-07	stage iii	t3a	female			
TCGA-BQ-5878-01A-11R-1592-07	stage i	t1a	male			
TCGA-B1-A47N-01A-11R-A24Z-07	stage iii	t3b	male			
TCGA-P4-AAVL-01A-11R-A42S-07	stage i	t1b	male			
TCGA-WN-A9G9-01A-12R-A37K-07	stage ii	t2	male			
TCGA-KL-8340-01A-11R-2315-07	stage ii	t2	female			
TCGA-KM-8438-01A-11R-2315-07	NA	tx	male			
TCGA-IA-A83S-01A-11R-A355-07	stage i	t1	male			
TCGA-SX-A7SS-01A-11R-A36F-07	stage i	t1b	female			
TCGA-IA-A83T-01A-11R-A355-07	NA	t2	male			
TCGA-B9-4114-01A-01R-1193-07	stage iii	t3a	male			
TCGA-BP-4761-01A-01R-1289-07	stage i	t1a	male			
TCGA-2Z-A9JP-01A-11R-A42S-07	stage i	t1a	female			
TCGA-BQ-5888-01A-11R-1592-07	stage ii	t2	female			
TCGA-KN-8434-01A-11R-2315-07	stage ii	t2b	female			
TCGA-KL-8342-01A-11R-2315-07	stage iii	t3a	male			
TCGA-B1-A47M-01A-11R-A24Z-07	NA	t1a	male			

TCGA-B9-4617-01A-01R-1193-07	stage i	t1a	female		
TCGA-B1-A654-01A-11R-A31O-07	stage iii	t3a	male		
TCGA-KL-8335-01A-11R-2315-07	stage i	t1a	male		
TCGA-BP-5175-01A-01R-1426-07	stage i	t1	female		
TCGA-KO-8415-01A-11R-2315-07	stage i	t1a	male		
TCGA-SX-A71R-01A-12R-A33Z-07	stage i	t1b	female		
TCGA-DW-5560-01A-01R-1592-07	stage i	t1a	male		
TCGA-2Z-A9JQ-01A-11R-A42S-07	stage i	t1b	male		
TCGA-AL-3472-01A-01R-1193-07	stage ii	t2	female		
TCGA-KO-8414-01A-11R-2315-07	stage i	t1a	male		
TCGA-B1-A656-01A-11R-A31O-07	stage i	t1a	male		
TCGA-A4-A6HP-01A-11R-A31O-07	stage i	t1	male		
TCGA-KM-8476-01A-11R-2315-07	stage i	t1a	male		
TCGA-IZ-A6M8-01A-11R-A31O-07	stage ii	t2	female		
TCGA-KL-8324-01A-11R-2315-07	stage iv	t3a	female		
TCGA-CJ-4638-01A-02R-1325-07	stage i	t1a	male		
TCGA-P4-A5ED-01A-11R-A28H-07	stage iii	t3a	male		
TCGA-KO-8416-01A-11R-2315-07	stage ii	t2	female		
TCGA-KN-8431-01A-11R-2315-07	stage iii	t3a	female		
TCGA-KN-8433-01A-11R-2315-07	stage ii	t2b	male		
TCGA-KL-8346-01A-11R-2315-07	stage iii	t3a	male		
TCGA-KL-8338-01A-11R-2315-07	stage iii	t3a	male		
TCGA-BP-4334-01A-01R-1289-07	stage i	t1b	male		
TCGA-MH-A55Z-01A-11R-A26U-07	stage ii	t2b	male		
TCGA-KO-8409-01A-11R-2315-07	stage i	t1b	female		
TCGA-KL-8327-01A-11R-2315-07	stage i	t1a	male		
TCGA-Y8-A8S1-01A-11R-A37K-07	stage i	t1a	male		
TCGA-MH-A562-01A-11R-A26U-07	stage iii	t3a	male		
TCGA-KO-8408-01A-11R-2315-07	stage ii	t2	male		

TCGA-BP-4326-01A-01R-1289-07	stage i	t2	male		
TCGA-CZ-4858-01A-01R-1305-07	stage ii	t2	male		
TCGA-B0-5706-01A-11R-1541-07	stage ii	t2	male		
TCGA-CZ-5456-01A-01R-1503-07	stage ii	t2	female		
TCGA-BP-4169-01A-02R-1289-07	stage ii	t1a	male		
TCGA-CJ-5671-01A-11R-1541-07	stage i	t3a	female		
TCGA-B8-4620-01A-02R-1325-07	stage iii	t3b	male		
TCGA-CJ-4901-01A-01R-1426-07	stage iii	t1a	female		
TCGA-A4-7828-01A-11R-2139-07	stage i	t3a	female		
TCGA-B0-4710-01A-01R-1503-07	stage iii	t4	male		
TCGA-B0-4698-01A-01R-1503-07	stage iv	t1	female		
TCGA-EU-5904-01A-11R-1672-07	stage i	t2	male		
TCGA-BP-5200-01A-01R-1426-07	stage ii	t3b	female		
TCGA-B0-5097-01A-01R-1420-07	stage iii	t1a	female		
TCGA-GK-A6C7-01A-11R-A33J-07	stage i	t3a	male	SLC2A14	>75 percentile
TCGA-CJ-4882-01A-02R-1426-07	stage iii	t2a	male		
TCGA-A3-3335-01A-01R-0864-07	stage ii	t1a	male		
TCGA-BP-4781-01A-01R-1305-07	stage i	t1	female		
TCGA-B0-5098-01A-01R-1420-07	stage i	t2	male		
TCGA-BP-4960-01A-01R-1334-07	stage ii	t2	male		
TCGA-CZ-4861-01A-01R-1305-07	stage ii	t1b	female		
TCGA-BP-4340-01A-01R-1289-07	stage i	t2	male		
TCGA-AK-3436-01A-02R-1325-07	stage iv	t3a	male		
TCGA-CJ-4868-01A-01R-1305-07	stage iv	t3b	male		
TCGA-B0-5116-01A-02R-1420-07	stage iii	t3b	female		
TCGA-B0-4821-01A-01R-1503-07	stage iii	t3b	male		
TCGA-BP-4799-01A-01R-1305-07	stage iii	t3a	male		
TCGA-BP-4974-01A-01R-1334-07	stage iv	t4	female		
TCGA-BP-4770-01A-01R-1503-07	stage iv	t1b	female		

TCGA-B8-A8YJ-01A-13R-A39I-07	stage i	t3a	male		
TCGA-EU-5907-01A-11R-1672-07	stage iii	t3a	female		
TCGA-BP-4355-01A-01R-1289-07	stage iii	t3a	male		
TCGA-BP-5178-01A-01R-1426-07	stage iv	t1a	male		
TCGA-BP-5008-01A-01R-1334-07	stage i	t3a	male		
TCGA-CZ-4857-01A-01R-1305-07	stage iv	t4	male		
TCGA-B0-4690-01A-01R-1277-07	stage iv	t3b	male		
TCGA-CZ-5459-01A-01R-1503-07	stage iii	t1a	female		
TCGA-DV-5569-01A-01R-1541-07	stage i	t3a	female		
TCGA-BP-4335-01A-01R-1289-07	stage iv	t3a	male		
TCGA-B0-4718-01A-01R-1277-07	stage iii	t1b	male		
TCGA-B0-5698-01A-11R-1672-07	stage i	t1	male		
TCGA-B4-5378-01A-01R-1503-07	stage i	t1b	male		
TCGA-CZ-5461-01A-01R-1503-07	stage iv	t1	male		
TCGA-BP-4353-01A-02R-1289-07	stage i	t1a	female		
TCGA-DV-5575-01A-01R-1541-07	stage i	t1b	male		
TCGA-CZ-5988-01A-11R-1672-07	stage i	t1b	male		
TCGA-EU-5906-01A-11R-1672-07	stage i	t3b	male		
TCGA-BP-4345-01A-01R-1289-07	stage iii	t2	male		
TCGA-B0-4822-01A-01R-1277-07	stage ii	t1a	male		
TCGA-BP-5187-01A-01R-1426-07	stage i	t3a	male		
TCGA-B0-5108-01A-01R-1420-07	stage iii	t1b	male		
TCGA-CW-6090-01A-11R-1672-07	stage i	t1b	female		
TCGA-BP-4170-01A-02R-1289-07	stage i	t2	female		
TCGA-B0-4852-01A-01R-1503-07	stage ii	t1b	male		
TCGA-BP-4338-01A-01R-1289-07	stage i	t3a	male		
TCGA-CJ-4885-01A-01R-1305-07	stage iv	t1a	male		
TCGA-B0-5077-01A-01R-1334-07	stage i	t3b	female		
TCGA-B0-5099-01A-01R-1420-07	stage iii	t4	male		

TCGA-B0-4814-01A-01R-1277-07	stage iv	t1b	female		
TCGA-CJ-4920-01A-01R-1426-07	stage i	t1a	male		
TCGA-BP-4998-01A-01R-1334-07	stage i	t3a	male		
TCGA-BP-4343-01A-02R-1289-07	stage iii	t4	male		
TCGA-CZ-4860-01A-01R-1305-07	stage iv	t1b	male		
TCGA-B8-5549-01A-01R-1541-07	stage i	t1a	male		
TCGA-B8-5545-01A-01R-1672-07	stage i	t1a	male		
TCGA-BP-4988-01A-01R-1334-07	stage i	t3a	male		
TCGA-BP-4973-01A-01R-1334-07	stage iii	t3a	female		
TCGA-B8-4143-01A-01R-1188-07	stage iv	t3a	female		
TCGA-BP-4983-01A-01R-1334-07	stage iii	t1	female		
TCGA-EU-5905-01A-11R-1672-07	stage i	t3b	male		
TCGA-B0-5094-01A-01R-1420-07	stage iv	t3a	female		
TCGA-CJ-4878-01A-01R-1305-07	stage iii	t1b	male		
TCGA-B0-5703-01A-11R-1541-07	stage i	t1a	female		
TCGA-BP-4768-01A-01R-1289-07	stage i	t3b	female		
TCGA-B0-4819-01A-01R-1277-07	stage iv	t1b	female		
TCGA-B0-5695-01A-11R-1541-07	stage i	t1	male		
TCGA-CZ-5986-01A-11R-1672-07	stage i	t1b	male		
TCGA-A3-3382-01A-02R-1325-07	stage i	t1b	male		
TCGA-AK-3454-01A-02R-1277-07	stage i	t1a	male		
TCGA-B0-5700-01A-11R-1541-07	stage i	t1	male		
TCGA-A3-3378-01A-02R-1325-07	stage i	t1b	male		
TCGA-BP-4176-01A-02R-1289-07	stage i	t3a	male		
TCGA-CJ-4918-01A-01R-1426-07	stage iv	t1a	female		
TCGA-B8-A7U6-01A-12R-A37O-07	stage i	t3a	male		
TCGA-B0-4815-01A-01R-1503-07	stage iii	t3b	female		
TCGA-BP-4352-01A-01R-1289-07	stage iv	t1b	male		
TCGA-B0-4837-01A-01R-1305-07	stage i	t1a	female		

TCGA-B8-A54F-01A-11R-A266-07	stage i	t1b	male		
TCGA-CJ-5689-01A-11R-1541-07	stage i	t3a	female		
TCGA-BP-4787-01A-01R-1305-07	stage iv	t3b	male		
TCGA-CZ-5460-01A-01R-1503-07	stage iv	t1a	male		
TCGA-BP-4993-01A-02R-1420-07	stage i	t1b	male		
TCGA-A3-3346-01A-01R-1766-07	stage i	t1b	male		
TCGA-DV-A4W0-01A-11R-A266-07	stage i	t1b	male		
TCGA-CZ-5984-01A-11R-1672-07	stage i	t1b	male		
TCGA-CZ-4854-01A-01R-1305-07	stage i	t2	male		
TCGA-KL-8333-01A-11R-2315-07	stage ii	t3a	male		
TCGA-BP-4803-01A-01R-1305-07	stage iii	t2	male		
TCGA-BP-5199-01A-01R-1426-07	stage ii	t1a	female		
TCGA-DV-5576-01A-01R-1541-07	stage i	t3a	male		
TCGA-CJ-6028-01A-11R-1672-07	stage iv	t4	male		
TCGA-B0-4700-01A-02R-1541-07	stage iv	t3a	male		
TCGA-B0-5080-01A-01R-1503-07	stage iv	t1b	female		
TCGA-A3-3347-01A-02R-1325-07	stage iii	t2	male		
TCGA-BP-4342-01A-01R-1289-07	stage ii	t3a	male		
TCGA-B0-4712-01A-01R-1503-07	stage iv	t3a	female		
TCGA-CJ-4904-01A-02R-1426-07	stage iv	t3a	male		
TCGA-BP-4971-01A-01R-1334-07	stage iii	t1a	male		
TCGA-B0-4823-01A-02R-1420-07	stage i	t1a	female		
TCGA-A3-A8OU-01A-11R-A37O-07	stage i	t3b	male		
TCGA-CZ-5464-01A-01R-1503-07	stage iv	t3	male		
TCGA-A3-3372-01A-02R-1325-07	stage iii	t3a	male		
TCGA-B8-5158-01A-01R-1420-07	stage iii	t1b	male		
TCGA-BP-4977-01A-01R-1334-07	stage i	t3a	female		
TCGA-B8-5163-01A-01R-1420-07	stage iii	t2	female		
TCGA-CJ-6032-01A-11R-1672-07	stage ii	t1a	male		

TCGA-CJ-6030-01A-11R-1672-07	stage i	t1a	female		
TCGA-BQ-7049-01A-11R-1965-07	stage i	t3a	male		
TCGA-B0-5696-01A-11R-1541-07	stage iii	t3a	male		
TCGA-BP-4329-01A-02R-1289-07	stage iii	t1a	male		
TCGA-AS-3778-01A-01R-A32Z-07	stage i	t3a	female		
TCGA-BP-4972-01A-01R-1334-07	stage iii	t1a	male		
TCGA-GL-7773-01A-11R-A32Z-07	stage i	t3a	male		
TCGA-B0-5694-01A-11R-1541-07	stage iii	t3a	male		
TCGA-CJ-4902-01A-01R-1426-07	stage iii	t1b	female		
TCGA-B8-5551-01A-01R-1541-07	stage i	t2	male		
TCGA-BP-4174-01A-02R-1289-07	stage ii	t1a	female		
TCGA-BP-4763-01A-01R-1289-07	stage i	t1a	male		
TCGA-BP-4986-01A-01R-1334-07	stage i	t3a	female		
TCGA-BP-4351-01A-01R-1289-07	stage iii	t3a	female		
TCGA-B0-4693-01A-01R-1277-07	stage iii	t1	male		
TCGA-AK-3425-01A-02R-1277-07	stage i	t3a	male		
TCGA-CJ-4890-01A-01R-1305-07	stage iv	t3a	female		
TCGA-B0-4701-01A-01R-1277-07	stage iv	t1a	female		
TCGA-DV-5567-01A-01R-1541-07	stage i	t1a	male		
TCGA-BP-4807-01A-01R-1305-07	stage i	t1a	male		
TCGA-BP-4769-01A-01R-1289-07	stage i	t2b	female		
TCGA-CJ-4643-01A-02R-1325-07	stage ii	t1b	male		
TCGA-CJ-4872-01A-01R-1305-07	stage i	t3a	male		
TCGA-CJ-4871-01A-01R-1305-07	stage iv	t4	male		
TCGA-B0-4699-01A-01R-1277-07	stage iv	t1a	female		
TCGA-A3-3358-01A-01R-1541-07	stage i	t1a	male		
TCGA-BP-4760-01A-02R-1420-07	stage i	t1a	male		
TCGA-DV-5565-01A-01R-1541-07	stage i	t3b	male		
TCGA-CZ-5987-01A-11R-1672-07	stage iv	t2	female		

TCGA-BP-4327-01A-01R-1289-07	stage ii	t1a	male		
TCGA-BP-4999-01A-01R-1334-07	stage i	t3a	female		
TCGA-CJ-4870-01A-01R-1305-07	stage iii	t3a	male		
TCGA-BP-4160-01A-02R-1289-07	stage iii	t4	female		
TCGA-CJ-4900-01A-01R-1334-07	stage iv	t3b	female		
TCGA-A3-3308-01A-02R-1325-07	stage iii	t1	female		
TCGA-B0-5104-01A-01R-1420-07	stage i	t1a	female		
TCGA-BP-4766-01A-01R-1289-07	stage i	t3b	male		
TCGA-B0-4836-01A-01R-1305-07	stage iv	t3a	male		
TCGA-B0-4703-01A-01R-1277-07	stage iv	t3b	male		
TCGA-CJ-5676-01A-11R-1541-07	stage iii	t1a	male		
TCGA-CW-6093-01A-11R-1672-07	stage i	t2	male		
TCGA-BP-4173-01A-02R-1289-07	stage ii	t1b	male		
TCGA-BP-5189-01A-02R-1426-07	stage i	t1b	male		
TCGA-B0-5121-01A-02R-1420-07	stage i	t1a	male		
TCGA-5P-A9JV-01A-12R-A42S-07	NA	t3a	male		
TCGA-BP-4985-01A-01R-1334-07	stage iii	t1a	male		
TCGA-A3-3365-01A-01R-0864-07	stage i	t3b	male		
TCGA-BP-4347-01A-01R-1289-07	stage iii	t1a	male		
TCGA-BP-5168-01A-01R-1420-07	stage i	t1b	female		
TCGA-A3-A6NL-01A-11R-A33J-07	stage i	t1a	female		
TCGA-BP-4344-01A-01R-1289-07	stage i	t2	male		
TCGA-B0-5115-01A-01R-1420-07	stage iv	t3a	female		
TCGA-CJ-6033-01A-11R-1672-07	stage iv	t3b	male		
TCGA-B0-4694-01A-01R-1277-07	stage iii	t1a	male		
TCGA-CJ-6027-01A-11R-1672-07	stage i	t1b	male		
TCGA-BP-4963-01A-01R-1334-07	stage i	t2	male		
TCGA-CZ-5453-01A-01R-1503-07	stage ii	t1b	male		
TCGA-CJ-6031-01A-11R-1672-07	stage i	t1a	male		

TCGA-BP-5180-01A-01R-1426-07	stage i	t3a	male		
TCGA-B0-4843-01A-01R-1277-07	stage iii	t1b	male		
TCGA-BP-4975-01A-01R-1334-07	stage i	t1b	male		
TCGA-A3-3319-01A-02R-1325-07	stage i	t1b	male		
TCGA-BP-5009-01A-01R-1334-07	stage i	t1b	male		
TCGA-B0-5088-01A-01R-1334-07	stage i	t1b	male		
TCGA-A3-3324-01A-02R-1325-07	stage i	t1a	male		
TCGA-BP-4776-01A-01R-1289-07	stage i	t1b	female		
TCGA-B0-5693-01A-11R-1541-07	stage i	t1a	male		
TCGA-BP-4765-01A-01R-1289-07	stage i	t3b	female		
TCGA-CZ-5465-01A-01R-1503-07	stage iii	t1a	male		
TCGA-B0-5106-01A-01R-1420-07	stage i	t1a	male		
TCGA-DV-5574-01A-01R-1541-07	stage i	t3a	female		
TCGA-B0-5113-01A-01R-1420-07	stage iii	t1a	male		
TCGA-BP-4801-01A-02R-1420-07	stage i	t3b	female		
TCGA-B0-5713-01A-11R-1672-07	stage iii	t3a	female		
TCGA-CZ-5467-01A-01R-1503-07	stage iii	t1a	female		
TCGA-B0-5120-01A-01R-1420-07	stage i	t3a	female		
TCGA-B0-5096-01A-01R-1420-07	stage iii	t1a	male		
TCGA-BP-4991-01A-01R-1334-07	stage i	t1a	male		
TCGA-BP-4789-01A-01R-1305-07	stage i	t3b	male		
TCGA-AK-3428-01A-02R-1277-07	stage iii	t1a	female		
TCGA-BP-4964-01A-01R-1334-07	stage i	t2a	male		
TCGA-B8-5162-01A-01R-1420-07	stage ii	t1b	male		
TCGA-CJ-5683-01A-11R-1541-07	stage i	t1b	female		
TCGA-BP-4162-01A-02R-1325-07	stage i	t3b	male		
TCGA-CJ-5679-01A-11R-1541-07	stage iii	t4	male		
TCGA-BP-4354-01A-02R-1289-07	stage iv	t1a	male		
TCGA-BP-5170-01A-01R-1426-07	stage i	t2a	male		

TCGA-IZ-8195-01A-31R-2404-07	stage ii	t2	female		
TCGA-AK-3429-01A-02R-1325-07	stage ii	t1b	female		
TCGA-B0-5690-01A-11R-1541-07	stage i	t1b	male		
TCGA-BP-4158-01A-02R-1289-07	stage i	t1b	male		
TCGA-CZ-5462-01A-01R-1503-07	stage iv	t1a	male		
TCGA-BP-5194-01A-02R-1426-07	stage i	t4	male		
TCGA-B0-4688-01A-01R-1277-07	stage iv	t1b	male		
TCGA-DW-7838-01A-11R-2139-07	stage i	t2	male		
TCGA-CZ-5454-01A-01R-1503-07	stage iv	t2	male		
TCGA-KN-8419-01A-11R-2315-07	stage ii	t1a	male		
TCGA-BP-5004-01A-01R-1334-07	stage i	t3a	male		
TCGA-B0-5095-01A-01R-1420-07	stage iii	t3a	male		
TCGA-B8-4622-01A-02R-1277-07	stage iv	t3a	male		
TCGA-B0-4810-01A-01R-1503-07	stage iii	t3a	male		
TCGA-CW-5590-01A-01R-1541-07	stage iv	t3a	female		
TCGA-CJ-4897-01A-03R-1426-07	stage iii	t1b	female		
TCGA-A3-3373-01A-02R-1420-07	stage i	t2b	male		
TCGA-DW-7840-01A-11R-A32Z-07	stage i	t3a	female		<25 Percentile
TCGA-KN-8429-01A-11R-2315-07	stage iii	t1a	male		
TCGA-A4-8098-01A-11R-2404-07	stage i	t3	male		
TCGA-UZ-A9PN-01A-11R-A38C-07	stage iii	t1b	male		
TCGA-A4-A5Y1-01A-11R-A28H-07	stage iii	t3b	female		
TCGA-KL-8323-01A-21R-2315-07	stage iii	t1	male		
TCGA-UZ-A9Q0-01A-12R-A42S-07	stage i	t3a	male		
TCGA-B9-5155-01A-01R-1592-07	stage iii	t1b	male		
TCGA-KL-8332-01A-11R-2315-07	stage i	t2	male		
TCGA-KO-8407-01A-11R-2315-07	stage ii	t1b	male		
TCGA-KM-8439-01A-11R-2315-07	stage i	t2	female		
TCGA-KN-8431-01A-11R-2315-07	stage ii	t1a	male		

TCGA-B9-A8YH-01A-11R-A37K-07	stage i	t3a	male		
TCGA-KL-8338-01A-11R-2315-07	stage iii	#N/A	#N/A		
TCGA-KL-8339-01A-11R-2315-07	#N/A	t2	male		
TCGA-KN-8436-01A-11R-2315-07	stage ii	t3b	male		
TCGA-B1-5398-01A-02R-1592-07	stage iii	t1a	male		
TCGA-BP-5190-01A-01R-1426-07	stage i	t1a	male		
TCGA-G7-6796-01A-11R-1965-07	stage i	t3a	female		
TCGA-CJ-5680-01A-11R-1541-07	stage iv	t1b	female		
TCGA-A3-3370-01A-02R-1420-07	stage i	t1a	male		
TCGA-B1-A47M-01A-11R-A24Z-07	stage iii	t1a	male		
TCGA-B0-4834-01A-01R-1305-07	stage i	t3a	female		
TCGA-CJ-5681-01A-11R-1541-07	stage iv	t1b	male		
TCGA-A3-3313-01A-02R-1325-07	stage i	t3a	male		
TCGA-KO-8416-01A-11R-2315-07	stage iii	t3b	male		
TCGA-B4-5832-01A-11R-1672-07	stage iii	t3a	male		
TCGA-A4-8516-01A-11R-2404-07	stage iii	t3a	male		
TCGA-UZ-A9PZ-01A-11R-A42S-07	stage iii	t1a	male		
TCGA-KM-8639-01A-11R-2403-07	stage i	t1b	female		
TCGA-PJ-A5Z9-01A-11R-A28H-07	stage i	t2	male		
TCGA-B9-7268-01A-11R-A32Z-07	stage ii	t1b	male		
TCGA-KN-8422-01A-11R-2315-07	stage i	t1b	male		
TCGA-2Z-A9J8-01A-11R-A42S-07	stage i	t3a	male		
TCGA-G7-A8LD-01A-11R-A36F-07	stage iii	t1a	male		
TCGA-G7-A4TM-01A-11R-A31O-07	stage i	t1b	male		
TCGA-DW-7837-01A-11R-2139-07	stage i	t2	male		
TCGA-B2-3923-01A-02R-1325-07	stage ii	t3a	male		
TCGA-A4-7286-01A-11R-A32Z-07	NA	t1a	male		
TCGA-A4-8311-01A-11R-2404-07	stage i	t1a	male		
TCGA-UZ-A9PO-01A-11R-A38C-07	stage i	t1a	female		

TCGA-BQ-5876-01A-11R-1592-07	stage i	t3b	female		
TCGA-BQ-7056-01A-11R-1965-07	stage iii	t2	male		
TCGA-KL-8337-01A-11R-2315-07	stage ii	t2	female		
TCGA-J7-8537-01A-11R-2404-07	stage iii	t1	female		
TCGA-A4-7287-01A-11R-2139-07	stage i	t1a	male		
TCGA-Y8-A8S0-01A-11R-A37K-07	stage i	t1a	male		
TCGA-B8-A54G-01A-11R-A266-07	stage i	t3b	male		
TCGA-BQ-5894-01A-11R-1592-07	stage iv	t1b	male		
TCGA-GL-A4EM-01A-11R-A24Z-07	stage i	t2a	male		
TCGA-KN-8432-01A-11R-2315-07	stage ii	t2	male		
TCGA-KN-8435-01A-11R-2315-07	stage ii	t1a	male		
TCGA-2Z-A9JM-01A-12R-A42S-07	stage i	t1a	male		
TCGA-2Z-A9JL-01A-11R-A42S-07	stage i	t1b	female		
TCGA-BP-4756-01A-01R-1289-07	stage i	t2	male		
TCGA-B3-4104-01A-02R-1351-07	stage ii	t1	male		
TCGA-EV-5902-01A-11R-1592-07	stage i	t1	male		
TCGA-J7-6720-01A-11R-2139-07	stage i	t3a	male		
TCGA-B9-A69E-01A-11R-A31O-07	stage iii	t1a	male		
TCGA-A4-A6HP-01A-11R-A31O-07	stage i	t3a	male		
TCGA-DZ-6131-01A-11R-1965-07	stage iii	t1b	male		
TCGA-BQ-5885-01A-11R-1592-07	stage iii	t1b	male		
TCGA-MH-A55Z-01A-11R-A26U-07	stage i	t1b	male		
TCGA-J7-A8I2-01A-12R-A36F-07	stage i	t3a	female		
TCGA-F9-A4JJ-01A-11R-A24Z-07	stage iii	t2	female		
TCGA-B0-5107-01A-01R-1420-07	stage iv	t3	male		
TCGA-BQ-7058-01A-11R-1965-07	stage iii	t2a	female		
TCGA-BQ-5893-01A-11R-1592-07	stage iv	t2	male		
TCGA-HE-7129-01A-11R-1965-07	stage ii	t2	male		
TCGA-AL-3473-01A-01R-1193-07	stage ii	t2	female		

TCGA-AK-3433-01A-02R-1277-07	stage ii	t1	female		
TCGA-B4-5835-01A-11R-1672-07	stage i	t1b	male		
TCGA-A3-3328-01A-01R-0864-07	stage i	t1a	male		
TCGA-B9-4115-01A-01R-1193-07	stage i	t1a	male		
TCGA-IZ-8196-01A-11R-2404-07	stage i	t1b	male		
TCGA-B0-5117-01A-01R-1420-07	stage i	t1a	male		
TCGA-2Z-A9JR-01A-12R-A42S-07	stage i	t1a	male		
TCGA-4A-A93X-01A-11R-A37K-07	stage iv	t1a	male		
TCGA-O9-A75Z-01A-11R-A33Z-07	stage i	t1a	male		
TCGA-2Z-A9JS-01A-21R-A42S-07	stage i	t1b	female		
TCGA-B8-5546-01A-01R-1541-07	stage i	t3b	male		
TCGA-KL-8341-01A-11R-2315-07	stage iv	t1a	male		
TCGA-G7-6795-01A-11R-1965-07	stage i	t1b	male		
TCGA-AL-3471-01A-02R-1351-07	stage i	t1a	male		
TCGA-DW-7836-01A-11R-2139-07	stage i	t1a	male		
TCGA-HE-A5NH-01A-11R-A26U-07	stage i	t1a	male		
TCGA-G7-6797-01A-11R-1965-07	stage iii	t1a	female		
TCGA-T7-A92I-01A-11R-A37O-07	stage i	t1	male		
TCGA-IA-A83W-01A-11R-A355-07	stage i	t1	male		
TCGA-A4-8312-01A-11R-2404-07	stage i	t3a	female		
TCGA-HE-A5NF-01A-11R-A26U-07	stage i	t1b	female		
TCGA-A4-A4ZT-01A-11R-A26U-07	stage i	t3a	female		
TCGA-KN-8433-01A-11R-2315-07	stage iii	t2	male		
TCGA-KL-8340-01A-11R-2315-07	stage ii	t2a	male		
TCGA-A4-A57E-01A-11R-A26U-07	stage iv	t3a	male		
TCGA-KL-8345-01A-11R-2315-07	stage iii	t3a	male		
TCGA-B3-3925-01A-02R-1351-07	stage iii	t2	female		
TCGA-KN-8418-01A-11R-2315-07	stage ii	t1a	female		
TCGA-4A-A93W-01A-11R-A37K-07	stage i	t2	male		

TCGA-A3-3363-01A-01R-0864-07	stage ii	t1b	male		
TCGA-B9-A8YI-01A-21R-A37K-07	stage i	t1b	male		
TCGA-KN-8423-01A-11R-2315-07	stage i	t3a	female		
TCGA-BQ-5878-01A-11R-1592-07	stage iii	t3	female		
TCGA-BQ-7053-01A-11R-1965-07	stage iii	t1a	male		
TCGA-DW-5561-01A-01R-1592-07	stage i	t1b	female		
TCGA-B8-A54E-01A-11R-A266-07	stage i	t1b	male		
TCGA-GL-A9DE-01A-11R-A37K-07	stage i	t1a	male		
TCGA-2Z-A9JP-01A-11R-A42S-07	stage i	t3a	female		
TCGA-P4-A5EA-01A-11R-A28H-07	stage iii	t3c	male		
TCGA-GL-A59R-01A-11R-A26U-07	stage iii	t3b	female		
TCGA-BQ-5891-01A-11R-1592-07	stage iii	t1a	female		
TCGA-F9-A7VF-01A-11R-A33Z-07	stage i	t1a	male		
TCGA-DZ-6135-01A-11R-1965-07	stage i	t1a	male		
TCGA-AS-3777-01A-01R-0864-07	stage i	t1	male		
TCGA-SX-A7SQ-01A-12R-A36F-07	stage i	t2	male		
TCGA-KN-8428-01A-11R-2315-07	stage ii	t2	male		
TCGA-AK-3447-01A-01R-1766-07	stage ii	t1a	female		
TCGA-KN-8437-01A-11R-2315-07	stage i	t3a	male		
TCGA-CW-5591-01A-01R-1541-07	stage iv	t1b	female		
TCGA-KL-8329-01A-11R-2315-07	stage i	t1a	male		
TCGA-BQ-7055-01A-11R-1965-07	stage i	t2b	female		
TCGA-KL-8330-01A-11R-2315-07	stage ii	t1a	female		
TCGA-B1-A655-01A-11R-A31O-07	stage i	t3a	male		
TCGA-KN-8426-01A-11R-2315-07	stage iv	t2a	male		
TCGA-P4-A5E8-01A-11R-A28H-07	stage iii	t3a	female		
TCGA-B0-4842-01A-02R-1420-07	stage iii	t2	male		
TCGA-UZ-A9PQ-01A-11R-A42S-07	stage iii	t2	female		
TCGA-KL-8324-01A-11R-2315-07	stage ii	t2	male		

TCGA-2Z-A9J7-01A-11R-A38C-07	stage iv	t3b	female		
TCGA-KL-8336-01A-11R-2315-07	stage iv	t1b	male		
TCGA-UZ-A9PR-01A-11R-A42S-07	stage i	t1a	female		
TCGA-BQ-5883-01A-11R-1592-07	stage i	t3a	male		
TCGA-B8-A54D-01A-21R-A266-07	stage iii	t1a	male		
TCGA-AK-3427-01A-01R-0864-07	stage i	t3	female		
TCGA-HE-7130-01A-11R-1965-07	stage iii	t1	female		
TCGA-B3-3926-01A-02R-1351-07	stage i	t3a	male		
TCGA-KL-8326-01A-11R-2315-07	stage iii	t1a	female		
TCGA-2Z-A9JN-01A-21R-A42S-07	stage i	t1b	female		
TCGA-AK-3465-01A-02R-1325-07	stage i	t1b	female		
TCGA-MH-A854-01A-11R-A355-07	stage i	t1b	female		
TCGA-KL-8327-01A-11R-2315-07	stage i	t1a	female		
TCGA-BQ-5884-01A-11R-1592-07	stage i	t1	female		
TCGA-KO-8406-01A-11R-2315-07	stage i	t3a	female		
TCGA-BQ-5875-01A-11R-1592-07	stage iii	t3a	male		
TCGA-BP-4761-01A-01R-1289-07	stage iii	t1a	male		
TCGA-Y8-A898-01A-11R-A355-07	stage i	t1b	male		
TCGA-KN-8425-01A-11R-2315-07	stage i	t1	male		
TCGA-KM-8476-01A-11R-2315-07	stage i	t2b	male		
TCGA-KL-8346-01A-11R-2315-07	stage ii	t2	male		
TCGA-G7-7501-01A-11R-2204-07	stage iii	t1a	male		
TCGA-P4-A5ED-01A-11R-A28H-07	stage i	t2	female		
TCGA-KO-8414-01A-11R-2315-07	stage ii	t2	male		
TCGA-UZ-A9PL-01A-11R-A38C-07	stage ii	t1	female		
TCGA-KO-8417-01A-11R-2315-07	stage i	t1a	male		
TCGA-SX-A71R-01A-12R-A33Z-07	stage i	t1a	male		
TCGA-IZ-A6M9-01A-11R-A31O-07	stage i	t3a	female		
TCGA-KL-8334-01A-11R-2315-07	stage iii	t2	male		

TCGA-2Z-A9J3-01A-12R-A38C-07	stage ii	t1a	male		
TCGA-AT-A5NU-01A-11R-A28H-07	stage i	t1a	female		
TCGA-IA-A40X-01A-11R-A24Z-07	stage i	t3	male		
TCGA-KL-8343-01A-11R-2315-07	stage iii	t1b	male		
TCGA-P4-AAVO-01A-11R-A42S-07	stage i	t1a	male		
TCGA-B0-5083-01A-02R-1420-07	stage i	t3a	male		
TCGA-KM-8440-01A-11R-2315-07	stage iii	t2	male		
TCGA-AK-3451-01A-02R-1188-07	stage ii	t1b	female		
TCGA-A4-A5Y0-01A-11R-A31O-07	stage i	t1a	female		
TCGA-2Z-A9J2-01A-11R-A38C-07	stage i	t1b	male		
TCGA-B9-A5W9-01A-11R-A28H-07	stage i	t1b	male		
TCGA-F9-A8NY-01A-11R-A36F-07	stage iv	t1a	female		
TCGA-BQ-7050-01A-11R-1965-07	stage i	t1b	female		
TCGA-UN-AAZ9-01A-11R-A38C-07	stage i	t2b	male		
TCGA-KO-8409-01A-11R-2315-07	stage ii	t1b	female		
TCGA-DW-5560-01A-01R-1592-07	stage i	t1b	female		
TCGA-F9-A7Q0-01A-11R-A36F-07	stage i	t2	female		
TCGA-AK-3453-01A-02R-1277-07	stage ii	t2	female		
TCGA-KL-8325-01A-11R-2315-07	stage ii	t2	male		
TCGA-AK-3443-01A-02R-1325-07	stage ii	t1b	female		
TCGA-DW-7839-01A-11R-2139-07	stage i	t1b	male		
TCGA-SX-A7SO-01A-11R-A355-07	stage i	t3a	male		
TCGA-BP-4334-01A-01R-1289-07	stage iii	t1a	female		
TCGA-Y8-A894-01A-11R-A36F-07	stage i	t3a	male		
TCGA-KO-8408-01A-11R-2315-07	stage iii	t1b	male		
TCGA-KO-8411-01A-11R-2315-07	stage i	t1b	female		
TCGA-G6-A5PC-01A-11R-A33J-07	stage iv	t1a	male		
TCGA-2Z-A9JT-01A-11R-A42S-07	stage i	t1b	female		
TCGA-2Z-A9JO-01A-11R-A42S-07	stage i	t1a	male		

TCGA-2Z-A9JQ-01A-11R-A42S-07	stage i	t1	male		
TCGA-Y8-A8RZ-01A-11R-A37K-07	stage i	t2	female		
TCGA-KL-8331-01A-11R-2315-07	stage ii	t1a	male		
TCGA-MH-A560-01A-11R-A26U-07	stage i	t1a	male		
TCGA-SX-A7SL-01A-11R-A355-07	stage i	t3a	female		
TCGA-AL-A5DJ-01A-11R-A26U-07	stage iii	t1a	male		
TCGA-G7-A8LC-01A-11R-A36F-07	stage i	t1a	male		
TCGA-2Z-A9JE-01A-11R-A42S-07	stage i	t1a	male		
TCGA-MH-A857-01A-11R-A355-07	stage i	t1b	female		
TCGA-KM-8443-01A-11R-2315-07	stage ii	t2	female		
TCGA-KN-8421-01A-11R-2315-07	stage ii	t1b	male		
TCGA-B0-5702-01A-11R-1541-07	stage i	t1b	female		
TCGA-B1-A654-01A-11R-A31O-07	stage i	t1a	female		
TCGA-BQ-5888-01A-11R-1592-07	stage i	t1a	male		
TCGA-MH-A562-01A-11R-A26U-07	stage i	t3a	female		
TCGA-Q2-A5QZ-01A-11R-A28H-07	stage iii	t1a	female		
TCGA-WN-AB4C-01A-11R-A42S-07	stage i	#N/A	#N/A		
TCGA-BP-4326-01A-01R-1289-07	stage i	t1b	female	SLC2A3	>75 percentile
TCGA-B0-5706-01A-11R-1541-07	stage ii	t2	male		
TCGA-CZ-4858-01A-01R-1305-07	stage ii	t2	male		
TCGA-A4-7828-01A-11R-2139-07	stage i	t1a	female		
TCGA-B8-4620-01A-02R-1325-07	stage iii	t3a	female		
TCGA-BP-4770-01A-01R-1503-07	stage iv	t4	female		
TCGA-BP-4169-01A-02R-1289-07	stage ii	t2	female		
TCGA-CZ-5456-01A-01R-1503-07	stage ii	t2	male		
TCGA-B0-5098-01A-01R-1420-07	stage i	t1	female		
TCGA-CJ-4901-01A-01R-1426-07	stage iii	t3b	male		
TCGA-EU-5904-01A-11R-1672-07	stage i	t1	female		
TCGA-B0-5097-01A-01R-1420-07	stage iii	t3b	female		

TCGA-CJ-4882-01A-02R-1426-07	stage iii	t3a	male		
TCGA-B0-4698-01A-01R-1503-07	stage iv	t4	male		
TCGA-B0-4718-01A-01R-1277-07	stage iii	t3a	male		
TCGA-BP-4960-01A-01R-1334-07	stage ii	t2	male		
TCGA-BP-4799-01A-01R-1305-07	stage iii	t3b	male		
TCGA-B0-4710-01A-01R-1503-07	stage iii	t3a	female		
TCGA-CJ-5671-01A-11R-1541-07	stage i	t1a	male		
TCGA-BP-5200-01A-01R-1426-07	stage ii	t2	male		
TCGA-CZ-4861-01A-01R-1305-07	stage ii	t2	male		
TCGA-CZ-5988-01A-11R-1672-07	stage i	t1b	male		
TCGA-BP-4340-01A-01R-1289-07	stage i	t1b	female		
TCGA-B0-4821-01A-01R-1503-07	stage iii	t3b	female		
TCGA-B0-5116-01A-02R-1420-07	stage iii	t3b	male		
TCGA-BP-4974-01A-01R-1334-07	stage iv	t3a	male		
TCGA-GK-A6C7-01A-11R-A33J-07	stage i	t1a	female		
TCGA-CJ-4868-01A-01R-1305-07	stage iv	t3a	male		
TCGA-BP-5008-01A-01R-1334-07	stage i	t1a	male		
TCGA-BP-4355-01A-01R-1289-07	stage iii	t3a	female		
TCGA-BP-4335-01A-01R-1289-07	stage iv	t3a	female		
TCGA-DV-5575-01A-01R-1541-07	stage i	t1a	female		
TCGA-BP-4345-01A-01R-1289-07	stage iii	t3b	male		
TCGA-CZ-5459-01A-01R-1503-07	stage iii	t3b	male		
TCGA-A3-3335-01A-01R-0864-07	stage ii	t2a	male		
TCGA-AK-3436-01A-02R-1325-07	stage iv	t2	male		
TCGA-B0-4690-01A-01R-1277-07	stage iv	t4	male		
TCGA-CW-6090-01A-11R-1672-07	stage i	t1b	male		
TCGA-BP-4353-01A-02R-1289-07	stage i	t1	male		
TCGA-B0-4852-01A-01R-1503-07	stage ii	t2	female		
TCGA-CZ-4860-01A-01R-1305-07	stage iv	t4	male		

TCGA-B0-5108-01A-01R-1420-07	stage iii	t3a	male		
TCGA-CZ-5461-01A-01R-1503-07	stage iv	t1b	male		
TCGA-B4-5378-01A-01R-1503-07	stage i	t1	male		
TCGA-CJ-4878-01A-01R-1305-07	stage iii	t3a	female		
TCGA-B0-4814-01A-01R-1277-07	stage iv	t4	male		
TCGA-CJ-4885-01A-01R-1305-07	stage iv	t3a	male		
TCGA-DV-5569-01A-01R-1541-07	stage i	t1a	female		
TCGA-BP-4170-01A-02R-1289-07	stage i	t1b	female		
TCGA-BP-4781-01A-01R-1305-07	stage i	t1a	male		
TCGA-BP-4352-01A-01R-1289-07	stage iv	t3b	female		
TCGA-DV-A4W0-01A-11R-A266-07	stage i	t1b	male		
TCGA-BP-4176-01A-02R-1289-07	stage i	t1b	male		
TCGA-B0-4822-01A-01R-1277-07	stage ii	t2	male		
TCGA-AK-3454-01A-02R-1277-07	stage i	t1b	male		
TCGA-BP-4769-01A-01R-1289-07	stage i	t1a	male		
TCGA-BP-4988-01A-01R-1334-07	stage i	t1a	male		
TCGA-B0-5077-01A-01R-1334-07	stage i	t1a	male		
TCGA-BP-5199-01A-01R-1426-07	stage ii	t2	male		
TCGA-B0-5099-01A-01R-1420-07	stage iii	t3b	female		
TCGA-B0-4819-01A-01R-1277-07	stage iv	t3b	female		
TCGA-CZ-5986-01A-11R-1672-07	stage i	t1	male		
TCGA-B8-4143-01A-01R-1188-07	stage iv	t3a	female		
TCGA-B8-A54F-01A-11R-A266-07	stage i	t1a	female		
TCGA-B0-5700-01A-11R-1541-07	stage i	t1a	male		
TCGA-B0-4712-01A-01R-1503-07	stage iv	t3a	male		
TCGA-B0-4823-01A-02R-1420-07	stage i	t1a	male		
TCGA-A3-3372-01A-02R-1325-07	stage iii	t3	male		
TCGA-DV-5576-01A-01R-1541-07	stage i	t1a	female		
TCGA-B0-4837-01A-01R-1305-07	stage i	t1b	male		

TCGA-CZ-5460-01A-01R-1503-07	stage iv	t3b	male		
TCGA-B0-4811-01A-01R-1503-07	stage iii	t3a	male		
TCGA-BP-5170-01A-01R-1426-07	stage i	t1a	male		
TCGA-CJ-4876-01A-01R-1305-07	stage ii	t2b	male		
TCGA-BP-4776-01A-01R-1289-07	stage i	t1a	male		
TCGA-B8-A8YJ-01A-13R-A39I-07	stage i	t1b	female		
TCGA-BP-4971-01A-01R-1334-07	stage iii	t3a	male		
TCGA-EU-5907-01A-11R-1672-07	stage iii	t3a	male		
TCGA-CJ-6030-01A-11R-1672-07	stage i	t1a	male		
TCGA-CZ-4857-01A-01R-1305-07	stage iv	t3a	male		
TCGA-B8-5545-01A-01R-1672-07	stage i	t1a	male		
TCGA-AK-3428-01A-02R-1277-07	stage iii	t3b	male		
TCGA-B0-5695-01A-11R-1541-07	stage i	t1b	female		
TCGA-BP-5168-01A-01R-1420-07	stage i	t1a	male		
TCGA-BP-4162-01A-02R-1325-07	stage i	t1b	female		
TCGA-CJ-4920-01A-01R-1426-07	stage i	t1b	female		
TCGA-BP-4993-01A-02R-1420-07	stage i	t1a	male		
TCGA-CJ-6028-01A-11R-1672-07	stage iv	t3a	male		
TCGA-B0-5698-01A-11R-1672-07	stage i	t1b	male		
TCGA-CZ-5467-01A-01R-1503-07	stage iii	t3a	female		
TCGA-CJ-4918-01A-01R-1426-07	stage iv	t3a	male		
TCGA-B8-5163-01A-01R-1420-07	stage iii	t3a	female		
TCGA-BP-5187-01A-01R-1426-07	stage i	t1a	male		
TCGA-KO-8404-01A-11R-2315-07	stage iv	t4	male		
TCGA-B0-5080-01A-01R-1503-07	stage iv	t3a	male		
TCGA-B0-4693-01A-01R-1277-07	stage iii	t3a	female		
TCGA-CJ-4900-01A-01R-1334-07	stage iv	t4	female		
TCGA-BP-4998-01A-01R-1334-07	stage i	t1a	male		
TCGA-BP-4760-01A-02R-1420-07	stage i	t1a	male		

TCGA-CJ-6032-01A-11R-1672-07	stage ii	t2	female		
TCGA-B0-5115-01A-01R-1420-07	stage iv	t2	male		
TCGA-CZ-5453-01A-01R-1503-07	stage ii	t2	male		
TCGA-CZ-4854-01A-01R-1305-07	stage i	t1b	male		
TCGA-B0-5096-01A-01R-1420-07	stage iii	t3a	female		
TCGA-BP-4329-01A-02R-1289-07	stage iii	t3a	male		
TCGA-B0-4700-01A-02R-1541-07	stage iv	t4	male		
TCGA-BP-4985-01A-01R-1334-07	stage iii	t3a	male		
TCGA-B8-A7U6-01A-12R-A37O-07	stage i	t1a	female		
TCGA-CZ-5465-01A-01R-1503-07	stage iii	t3b	female		
TCGA-CJ-5689-01A-11R-1541-07	stage i	t1b	male		
TCGA-CW-6093-01A-11R-1672-07	stage i	t1a	male		
TCGA-B8-5549-01A-01R-1541-07	stage i	t1b	male		
TCGA-BP-4973-01A-01R-1334-07	stage iii	t3a	male		
TCGA-A3-3365-01A-01R-0864-07	stage i	t1a	male		
TCGA-BP-4787-01A-01R-1305-07	stage iv	t3a	female		
TCGA-B0-5094-01A-01R-1420-07	stage iv	t3b	male		
TCGA-A3-A8OU-01A-11R-A37O-07	stage i	t1a	female		
TCGA-CJ-4904-01A-02R-1426-07	stage iv	t3a	female		
TCGA-B0-4815-01A-01R-1503-07	stage iii	t3a	male		
TCGA-KL-8333-01A-11R-2315-07	stage ii	t2	male		
TCGA-A3-3316-01A-01R-0864-07	stage ii	t2	male		
TCGA-CZ-5987-01A-11R-1672-07	stage iv	t3b	male		
TCGA-BP-4807-01A-01R-1305-07	stage i	t1a	male		
TCGA-BP-4983-01A-01R-1334-07	stage iii	t3a	female		
TCGA-EU-5906-01A-11R-1672-07	stage i	t1b	male		
TCGA-BP-4344-01A-01R-1289-07	stage i	t1a	female		
TCGA-CJ-4902-01A-01R-1426-07	stage iii	t3a	male		
TCGA-BP-4351-01A-01R-1289-07	stage iii	t3a	female		

TCGA-CJ-6033-01A-11R-1672-07	stage iv	t3a	female			
TCGA-B0-4813-01A-01R-1277-07	stage iii	t3b	male			
TCGA-DV-5565-01A-01R-1541-07	stage i	t1a	male			
TCGA-B0-4699-01A-01R-1277-07	stage iv	t4	male			
TCGA-AK-3429-01A-02R-1325-07	stage ii	t2	female			
TCGA-CJ-4890-01A-01R-1305-07	stage iv	t3a	male			
TCGA-CJ-6027-01A-11R-1672-07	stage i	t1a	male			
TCGA-BP-4768-01A-01R-1289-07	stage i	t1a	female			
TCGA-BP-4158-01A-02R-1289-07	stage i	t1b	male			
TCGA-CJ-4872-01A-01R-1305-07	stage i	t1b	male			
TCGA-B0-5095-01A-01R-1420-07	stage iii	t3a	male			
TCGA-B0-4846-01A-01R-1277-07	stage iv	t3a	male			
TCGA-BP-4173-01A-02R-1289-07	stage ii	t2	male			
TCGA-BP-5178-01A-01R-1426-07	stage iv	t3a	male			
TCGA-B0-4843-01A-01R-1277-07	stage iii	t3a	male			
TCGA-B0-5104-01A-01R-1420-07	stage i	t1	female			
TCGA-BP-5010-01A-02R-1420-07	stage iii	t3a	male			
TCGA-B0-5694-01A-11R-1541-07	stage iii	t3a	male			
TCGA-B0-5102-01A-01R-1420-07	stage i	t1	female			
TCGA-CJ-4891-01A-01R-1305-07	stage iii	t3c	female			
TCGA-B0-5106-01A-01R-1420-07	stage i	t1a	male			
TCGA-A3-3378-01A-02R-1325-07	stage i	t1	male			
TCGA-BP-4174-01A-02R-1289-07	stage ii	t2	male			
TCGA-B0-4701-01A-01R-1277-07	stage iv	t3a	female			
TCGA-BP-4972-01A-01R-1334-07	stage iii	t3a	female			
TCGA-B0-4810-01A-01R-1503-07	stage iii	t3a	male			
TCGA-BP-4977-01A-01R-1334-07	stage i	t1b	male			
TCGA-B0-5088-01A-01R-1334-07	stage i	t1b	male			
TCGA-CZ-5454-01A-01R-1503-07	stage iv	t2	male			

TCGA-A3-3382-01A-02R-1325-07	stage i	t1b	male		
TCGA-BP-4342-01A-01R-1289-07	stage ii	t2	male		
TCGA-BP-4343-01A-02R-1289-07	stage iii	t3a	male		
TCGA-B8-5162-01A-01R-1420-07	stage ii	t2a	male		
TCGA-BP-4327-01A-01R-1289-07	stage ii	t2	female		
TCGA-B0-5713-01A-11R-1672-07	stage iii	t3b	female		
TCGA-A3-3346-01A-01R-1766-07	stage i	t1b	male		
TCGA-B8-5158-01A-01R-1420-07	stage iii	t3a	male		
TCGA-BP-4347-01A-01R-1289-07	stage iii	t3b	male		
TCGA-BP-4338-01A-01R-1289-07	stage i	t1b	male		
TCGA-CZ-5464-01A-01R-1503-07	stage iv	t3b	male		
TCGA-BP-4790-01A-01R-1305-07	stage i	t1a	male		
TCGA-GL-7773-01A-11R-A32Z-07	stage i	t1a	male		
TCGA-B0-5120-01A-01R-1420-07	stage i	t1a	female		
TCGA-B0-5113-01A-01R-1420-07	stage iii	t3a	female		
TCGA-CZ-5463-01A-01R-1503-07	stage ii	t2	male		
TCGA-DV-5574-01A-01R-1541-07	stage i	t1a	male		
TCGA-BP-4999-01A-01R-1334-07	stage i	t1a	male		
TCGA-B0-4945-01A-01R-1420-07	stage i	t1a	female		
TCGA-BP-4964-01A-01R-1334-07	stage i	t1a	female		
TCGA-BP-5180-01A-01R-1426-07	stage i	t1a	male		
TCGA-CJ-4871-01A-01R-1305-07	stage iv	t3a	male		
TCGA-CJ-6031-01A-11R-1672-07	stage i	t1b	male		
TCGA-BP-4164-01A-02R-1325-07	stage iii	t3a	female		
TCGA-BP-4803-01A-01R-1305-07	stage iii	t3a	male		
TCGA-BP-5196-01A-01R-1426-07	stage i	t1a	male		
TCGA-CJ-5676-01A-11R-1541-07	stage iii	t3b	male		
TCGA-B0-5703-01A-11R-1541-07	stage i	t1b	male		
TCGA-B0-4836-01A-01R-1305-07	stage iv	t3b	male		

TCGA-A3-3308-01A-02R-1325-07	stage iii	t3b	female		
TCGA-B0-5696-01A-11R-1541-07	stage iii	t3a	male		
TCGA-CJ-4870-01A-01R-1305-07	stage iii	t3a	female		
TCGA-B0-5121-01A-02R-1420-07	stage i	t1b	male		
TCGA-B0-5109-01A-02R-1420-07	stage iii	t3b	male		
TCGA-2K-A9WE-01A-11R-A38C-07	stage ii	t2b	male		
TCGA-BP-4986-01A-01R-1334-07	stage i	t1a	male		
TCGA-B0-5400-01A-01R-1503-07	stage iii	t3b	female		
TCGA-DV-5567-01A-01R-1541-07	stage i	t1a	female		
TCGA-CJ-4908-01A-01R-1426-07	stage i	t1a	male		
TCGA-AK-3431-01A-02R-1277-07	stage ii	t2	female		
TCGA-BP-5169-01A-01R-1426-07	stage i	t1b	male		
TCGA-CJ-4903-01A-01R-1426-07	stage i	t1b	male		
TCGA-AK-3425-01A-02R-1277-07	stage i	t1	male		
TCGA-CJ-4643-01A-02R-1325-07	stage ii	t2b	female		
TCGA-BQ-5889-01A-11R-1592-07	stage iv	t3b	male		
TCGA-B0-4703-01A-01R-1277-07	stage iv	t3a	male		
TCGA-BP-5173-01A-01R-1426-07	stage i	t1a	male		
TCGA-CJ-4895-01A-01R-1305-07	stage iv	t3a	male		
TCGA-CJ-4886-01A-01R-1305-07	stage i	t1a	female		
TCGA-BP-5009-01A-01R-1334-07	stage i	t1b	male		
TCGA-5P-A9JV-01A-12R-A42S-07	NA	t1a	male		
TCGA-B0-5690-01A-11R-1541-07	stage i	t1b	female		
TCGA-BP-4354-01A-02R-1289-07	stage iv	t4	male		
TCGA-BP-4775-01A-01R-1289-07	stage i	t1a	female		
TCGA-BP-4766-01A-01R-1289-07	stage i	t1a	female		
TCGA-B0-5693-01A-11R-1541-07	stage i	t1b	female		
TCGA-CZ-5468-01A-01R-1503-07	stage iv	t3b	male		
TCGA-BP-4968-01A-01R-1334-07	stage i	t1b	male		

TCGA-A3-3358-01A-01R-1541-07	stage i	t1a	female		
TCGA-CJ-4635-01A-02R-1305-07	stage i	t1b	male		
TCGA-BP-4991-01A-01R-1334-07	stage i	t1a	male		
TCGA-CZ-5457-01A-01R-1503-07	stage iii	t3a	male		
TCGA-EU-5905-01A-11R-1672-07	stage i	t1	female		
TCGA-2Z-A9JG-01A-11R-A42S-07	stage i	t1a	male		
TCGA-A4-7996-01A-11R-2204-07	stage i	t1a	female		
TCGA-A3-3328-01A-01R-0864-07	stage i	t1b	male		
TCGA-KM-8439-01A-11R-2315-07	stage i	t1b	male		
TCGA-P4-A5E6-01A-11R-A28H-07	stage iii	t1b	male		
TCGA-B9-4117-01A-02R-1351-07	NA	t2	female		
TCGA-B1-A657-01A-11R-A31O-07	stage i	t1b	male		
TCGA-F9-A7VF-01A-11R-A33Z-07	stage i	t1a	female		
TCGA-G7-6790-01A-11R-1965-07	stage i	t1a	male		
TCGA-KN-8435-01A-11R-2315-07	stage ii	t2	male		
TCGA-5P-A9K8-01A-11R-A42S-07	NA	t3a	female		
TCGA-A4-8630-01A-11R-2404-07	stage i	t1b	female		<25 Percentile
TCGA-B9-5155-01A-01R-1592-07	stage iii	t3a	male		
TCGA-UZ-A9PX-01A-11R-A42S-07	stage i	t1	male		
TCGA-BQ-7044-01A-11R-1965-07	stage iii	t3a	male		
TCGA-AK-3465-01A-02R-1325-07	stage i	t1b	female		
TCGA-KN-8436-01A-11R-2315-07	stage ii	t2	male		
TCGA-2Z-A9JR-01A-12R-A42S-07	stage i	t1a	male		
TCGA-KN-8426-01A-11R-2315-07	stage iv	t3a	male		
TCGA-A4-A57E-01A-11R-A26U-07	stage iv	t2a	male		
TCGA-V9-A7HT-01A-11R-A33Z-07	stage ii	t2	male		
TCGA-A4-8098-01A-11R-2404-07	stage i	t1a	male		
TCGA-B3-4103-01A-02R-1351-07	stage i	t1a	male		
TCGA-BQ-5883-01A-11R-1592-07	stage i	t1a	female		

TCGA-KO-8416-01A-11R-2315-07	stage iii	t3a	male		
TCGA-B4-5835-01A-11R-1672-07	stage i	t1	female		
TCGA-A4-7583-01A-11R-A32Z-07	stage i	t1	male		
TCGA-A4-A6HP-01A-11R-A31O-07	stage i	t1a	male		
TCGA-B9-4113-01A-01R-1193-07	stage i	t1	male		
TCGA-B9-4115-01A-01R-1193-07	stage i	t1a	male		
TCGA-A4-A772-01A-11R-A33Z-07	stage i	t1b	male		
TCGA-F9-A4JJ-01A-11R-A24Z-07	stage iii	t3a	female		
TCGA-G7-A8LD-01A-11R-A36F-07	stage iii	t3a	male		
TCGA-B3-3926-01A-02R-1351-07	stage i	t1	female		
TCGA-A4-A5XZ-01A-11R-A31O-07	stage ii	t2a	female		
TCGA-BQ-7055-01A-11R-1965-07	stage i	t1a	male		
TCGA-GL-A59T-01A-21R-A28H-07	stage i	t1b	male		
TCGA-KL-8334-01A-11R-2315-07	stage iii	t3a	female		
TCGA-MH-A55W-01A-11R-A26U-07	stage i	t1b	male		
TCGA-AL-3473-01A-01R-1193-07	stage ii	t2	male		
TCGA-KO-8408-01A-11R-2315-07	stage iii	t3a	male		
TCGA-CW-5591-01A-01R-1541-07	stage iv	t3a	male		
TCGA-B0-5107-01A-01R-1420-07	stage iv	t2	female		
TCGA-KN-8437-01A-11R-2315-07	stage i	t1a	female		
TCGA-WN-A9G9-01A-12R-A37K-07	stage i	t1b	male		
TCGA-KN-8428-01A-11R-2315-07	stage ii	t2	male		
TCGA-B8-4621-01A-01R-1503-07	stage i	t1b	male		
TCGA-J7-6720-01A-11R-2139-07	stage i	t1	male		
TCGA-A4-8312-01A-11R-2404-07	stage i	t1	male		
TCGA-BQ-5893-01A-11R-1592-07	stage iv	t3a	male		
TCGA-IZ-8196-01A-11R-2404-07	stage i	t1a	male		
TCGA-SX-A7SS-01A-11R-A36F-07	stage i	t1	male		
TCGA-G7-A4TM-01A-11R-A31O-07	stage i	t1a	male		

TCGA-KL-8330-01A-11R-2315-07	stage ii	t2b	female		
TCGA-UZ-A9PZ-01A-11R-A42S-07	stage iii	t3a	male		
TCGA-KL-8329-01A-11R-2315-07	stage i	t1b	female		
TCGA-KN-8433-01A-11R-2315-07	stage iii	t3a	female		
TCGA-HE-A5NH-01A-11R-A26U-07	stage i	t1a	male		
TCGA-AK-3433-01A-02R-1277-07	stage ii	t2	female		
TCGA-G6-A5PC-01A-11R-A33J-07	stage iv	t1b	female		
TCGA-O9-A75Z-01A-11R-A33Z-07	stage i	t1a	male		
TCGA-F9-A7Q0-01A-11R-A36F-07	stage i	t1b	female		
TCGA-KL-8341-01A-11R-2315-07	stage iv	t3b	male		
TCGA-A4-8516-01A-11R-2404-07	stage iii	t3a	male		
TCGA-T7-A92I-01A-11R-A37O-07	stage i	t1a	female		
TCGA-GL-A9DE-01A-11R-A37K-07	stage i	t1b	male		
TCGA-SX-A71U-01A-12R-A33Z-07	stage i	t1a	male		
TCGA-B8-A54E-01A-11R-A266-07	stage i	t1b	female		
TCGA-UZ-A9PO-01A-11R-A38C-07	stage i	t1a	male		
TCGA-DZ-6135-01A-11R-1965-07	stage i	t1a	male		
TCGA-BP-4761-01A-01R-1289-07	stage iii	t3a	male		
TCGA-KO-8414-01A-11R-2315-07	stage ii	t2	female		
TCGA-KN-8425-01A-11R-2315-07	stage i	t1b	male		
TCGA-KM-8476-01A-11R-2315-07	stage i	t1	male		
TCGA-SX-A7SR-01A-12R-A36F-07	stage ii	t2a	male		
TCGA-2Z-A9JM-01A-12R-A42S-07	stage i	t1a	male		
TCGA-SX-A71W-01A-12R-A355-07	stage i	t1b	male		
TCGA-PJ-A5Z9-01A-11R-A28H-07	stage i	t1b	female		
TCGA-BQ-7061-01A-11R-1965-07	stage i	t1b	female		
TCGA-G7-6795-01A-11R-1965-07	stage i	t1a	male		
TCGA-IA-A40X-01A-11R-A24Z-07	stage i	t1a	female		
TCGA-AT-A5NU-01A-11R-A28H-07	stage i	t1a	male		

TCGA-SX-A7SM-01A-11R-A355-07	stage iv	t3a	male		
TCGA-EV-5902-01A-11R-1592-07	stage i	t1	male		
TCGA-KL-8336-01A-11R-2315-07	stage iv	t3b	female		
TCGA-HE-7130-01A-11R-1965-07	stage iii	t3	female		
TCGA-KL-8324-01A-11R-2315-07	stage ii	t2	female		
TCGA-SX-A7SP-01A-11R-A355-07	stage i	t1b	female		
TCGA-KL-8345-01A-11R-2315-07	stage iii	t3a	male		
TCGA-KN-8432-01A-11R-2315-07	stage ii	t2b	female		
TCGA-B3-3925-01A-02R-1351-07	stage iii	t3a	male		
TCGA-SX-A7SQ-01A-12R-A36F-07	stage i	t1	male		
TCGA-KL-8340-01A-11R-2315-07	stage ii	t2	male		
TCGA-2Z-A9JN-01A-21R-A42S-07	stage i	t1a	female		
TCGA-BQ-5885-01A-11R-1592-07	stage iii	t3	male		
TCGA-KN-8423-01A-11R-2315-07	stage i	t1b	male		
TCGA-SX-A71R-01A-12R-A33Z-07	stage i	t1a	male		
TCGA-A3-3363-01A-01R-0864-07	stage ii	t2	male		
TCGA-UN-AAZ9-01A-11R-A38C-07	stage i	t1b	female		
TCGA-B1-A655-01A-11R-A31O-07	stage i	t1a	female		
TCGA-GL-A59R-01A-11R-A26U-07	stage iii	t3c	male		
TCGA-AK-3447-01A-01R-1766-07	stage ii	t2	male		
TCGA-IA-A83W-01A-11R-A355-07	stage i	t1	male		
TCGA-2Z-A9JT-01A-11R-A42S-07	stage i	t1a	male		
TCGA-DW-5560-01A-01R-1592-07	stage i	t1b	female		
TCGA-AS-3777-01A-01R-0864-07	stage i	t1a	male		
TCGA-KN-8418-01A-11R-2315-07	stage ii	t2	female		
TCGA-AK-3427-01A-01R-0864-07	stage i	t1a	male		
TCGA-BQ-5875-01A-11R-1592-07	stage iii	t3a	female		
TCGA-DW-5561-01A-01R-1592-07	stage i	t1a	male		
TCGA-BQ-7058-01A-11R-1965-07	stage iii	t3	male		

TCGA-Y8-A8S0-01A-11R-A37K-07	stage i	t1a	male		
TCGA-A4-A4ZT-01A-11R-A26U-07	stage i	t1b	female		
TCGA-AL-3471-01A-02R-1351-07	stage i	t1b	male		
TCGA-B8-5546-01A-01R-1541-07	stage i	t1b	female		
TCGA-HE-A5NF-01A-11R-A26U-07	stage i	t1a	male		
TCGA-F9-A8NY-01A-11R-A36F-07	stage iv	t4	female		
TCGA-4A-A93X-01A-11R-A37K-07	stage iv	t3a	male		
TCGA-KL-8326-01A-11R-2315-07	stage iii	t3a	male		
TCGA-AK-3443-01A-02R-1325-07	stage ii	t2	male		
TCGA-KO-8409-01A-11R-2315-07	stage ii	t2b	male		
TCGA-Y8-A8RY-01A-11R-A37K-07	stage i	t1b	male		
TCGA-AK-3453-01A-02R-1277-07	stage ii	t2	female		
TCGA-BQ-7056-01A-11R-1965-07	stage iii	t3b	female		
TCGA-2Z-A9JE-01A-11R-A42S-07	stage i	t1a	male		
TCGA-BQ-7051-01A-12R-1965-07	stage ii	t2	male		
TCGA-BQ-5884-01A-11R-1592-07	stage i	t1a	female		
TCGA-G7-7501-01A-11R-2204-07	stage iii	t3a	female		
TCGA-KL-8346-01A-11R-2315-07	stage ii	t2b	male		
TCGA-B1-A654-01A-11R-A31O-07	stage i	t1a	female		
TCGA-MH-A55Z-01A-11R-A26U-07	stage i	t1b	male		
TCGA-2Z-A9JL-01A-11R-A42S-07	stage i	t1a	male		
TCGA-KO-8406-01A-11R-2315-07	stage i	t1	female		
TCGA-4A-A93W-01A-11R-A37K-07	stage i	t1a	female		
TCGA-MH-A857-01A-11R-A355-07	stage i	t1a	male		
TCGA-2Z-A9JO-01A-11R-A42S-07	stage i	t1b	female		
TCGA-KL-8343-01A-11R-2315-07	stage iii	t3	male		
TCGA-B3-A6W5-01A-12R-A33Z-07	stage i	t1	male		
TCGA-BQ-7053-01A-11R-1965-07	stage iii	t3	female		
TCGA-A4-A5DU-01A-11R-A28H-07	stage i	t1a	female		

TCGA-AL-A5DJ-01A-11R-A26U-07	stage iii	t3a	female		
TCGA-Q2-A5QZ-01A-11R-A28H-07	stage iii	t3a	female		
TCGA-KL-8331-01A-11R-2315-07	stage ii	t2	female		
TCGA-BQ-5888-01A-11R-1592-07	stage i	t1a	female		
TCGA-GL-8500-01A-11R-2404-07	stage i	t1b	male		
TCGA-BQ-5886-01A-11R-1592-07	stage iii	t3a	male		
TCGA-BQ-7060-01A-11R-1965-07	stage i	t1a	male		
TCGA-A4-8311-01A-11R-2404-07	stage i	t1a	male		
TCGA-GL-6846-01A-11R-1965-07	stage iv	t4	male		
TCGA-A4-8518-01A-11R-2404-07	stage i	t1a	male		
TCGA-B9-A8YH-01A-11R-A37K-07	stage i	t1a	male		
TCGA-UZ-A9PV-01A-11R-A42S-07	stage i	t1	male		
TCGA-UZ-A9PR-01A-11R-A42S-07	stage i	t1b	male		
TCGA-B1-A47N-01A-11R-A24Z-07	stage i	t1a	male		
TCGA-A4-A5Y1-01A-11R-A28H-07	stage iii	t1b	male		
TCGA-KO-8417-01A-11R-2315-07	stage i	t1	female		
TCGA-2Z-A9J3-01A-12R-A38C-07	stage ii	t2	male		
TCGA-2Z-A9J7-01A-11R-A38C-07	stage iv	t2	male		
TCGA-KM-8440-01A-11R-2315-07	stage iii	t3a	male		
TCGA-AK-3451-01A-02R-1188-07	stage ii	t2	male		
TCGA-A4-7584-01A-11R-2139-07	stage i	t1a	male		
TCGA-SX-A71V-01A-11R-A33Z-07	stage i	t1	male		
TCGA-SX-A7SO-01A-11R-A355-07	stage i	t1b	male		
TCGA-5P-A9JU-01A-11R-A42S-07	stage iii	t3a	male		
TCGA-G7-A8LB-01A-11R-A36F-07	stage iv	t2a	male		
TCGA-2Z-A9JS-01A-21R-A42S-07	stage i	t1a	male		
TCGA-P4-AAVK-01A-11R-A42S-07	stage iii	t3a	male		
TCGA-G7-A8LC-01A-11R-A36F-07	stage i	t1a	male		
TCGA-WN-AB4C-01A-11R-A42S-07	stage i	t1a	female		

TCGA-HE-7129-01A-11R-1965-07	stage ii	t2	male		
TCGA-BQ-5878-01A-11R-1592-07	stage iii	t3a	female		
TCGA-2Z-A9JP-01A-11R-A42S-07	stage i	t1a	male		
TCGA-MH-A562-01A-11R-A26U-07	stage i	t1a	male		
TCGA-UZ-A9Q1-01A-11R-A42S-07	stage i	t1b	female		
TCGA-KM-8443-01A-11R-2315-07	stage ii	t2	male		
TCGA-B9-7268-01A-11R-A32Z-07	stage ii	t2	male		
TCGA-2Z-A9J8-01A-11R-A42S-07	stage i	t1b	male		
TCGA-KV-A74V-01A-11R-A33Z-07	stage i	t1a	male		
TCGA-2Z-A9J2-01A-11R-A38C-07	stage i	t1a	female		
TCGA-KO-8411-01A-11R-2315-07	stage i	t1b	male		
TCGA-BQ-5891-01A-11R-1592-07	stage iii	t3b	female		
TCGA-A4-A48D-01A-11R-A24Z-07	stage i	t1b	male		
TCGA-UZ-A9PQ-01A-11R-A42S-07	stage iii	t2	male		
TCGA-UZ-A9PL-01A-11R-A38C-07	stage ii	t2	male		
TCGA-MH-A560-01A-11R-A26U-07	stage i	t1a	male		
TCGA-A4-8310-01A-11R-2404-07	stage iii	t3a	male		
TCGA-KL-8325-01A-11R-2315-07	stage ii	t2	female		
TCGA-P4-A5EB-01A-11R-A28H-07	stage i	t1b	male		
TCGA-B9-A8YI-01A-21R-A37K-07	stage i	t1b	male		
TCGA-BQ-7050-01A-11R-1965-07	stage i	t1a	female		
TCGA-G7-6797-01A-11R-1965-07	stage iii	t1a	male		
TCGA-A4-8515-01A-11R-2404-07	stage i	t1a	male		
TCGA-KN-8421-01A-11R-2315-07	stage ii	t2	female		
TCGA-DW-7839-01A-11R-2139-07	stage i	t1b	female		
TCGA-Y8-A8RZ-01A-11R-A37K-07	stage i	t1	male		
TCGA-Y8-A894-01A-11R-A36F-07	stage i	t1a	female		
TCGA-P4-AAVO-01A-11R-A42S-07	stage i	t1b	male		
TCGA-P4-AAVM-01A-11R-A42S-07	stage i	t1a	male		

TCGA-DZ-6133-01A-11R-1965-07	stage i	t1a	female		
TCGA-A4-A5Y0-01A-11R-A31O-07	stage i	t1b	female		
TCGA-B9-A5W9-01A-11R-A28H-07	stage i	t1b	male		
TCGA-SX-A7SL-01A-11R-A355-07	stage i	t1a	male		
TCGA-BP-4983-01A-01R-1334-07	stage iii	t3a	female	B3GTT5	>75 percentile
TCGA-B0-5097-01A-01R-1420-07	stage iii	t3b	female		
TCGA-B0-4698-01A-01R-1503-07	stage iv	t4	male		
TCGA-BP-4799-01A-01R-1305-07	stage iii	t3b	male		
TCGA-BP-4326-01A-01R-1289-07	stage i	t1b	female		
TCGA-CZ-5989-01A-11R-1672-07	stage ii	t2	male		
TCGA-B8-4620-01A-02R-1325-07	stage iii	t3a	female		
TCGA-B4-5832-01A-11R-1672-07	stage iii	t3b	male		
TCGA-EU-5904-01A-11R-1672-07	stage i	t1	female		
TCGA-B0-5690-01A-11R-1541-07	stage i	t1b	female		
TCGA-A4-7915-01A-11R-2204-07	stage ii	t2b	female		
TCGA-B0-5698-01A-11R-1672-07	stage i	t1b	male		
TCGA-BP-5200-01A-01R-1426-07	stage ii	t2	male		
TCGA-BP-4985-01A-01R-1334-07	stage iii	t3a	male		
TCGA-AK-3454-01A-02R-1277-07	stage i	t1b	male		
TCGA-DV-5565-01A-01R-1541-07	stage i	t1a	male		
TCGA-BP-4781-01A-01R-1305-07	stage i	t1a	male		
TCGA-BP-4354-01A-02R-1289-07	stage iv	t4	male		
TCGA-B4-5835-01A-11R-1672-07	stage i	t1	female		
TCGA-BP-4176-01A-02R-1289-07	stage i	t1b	male		
TCGA-B0-4712-01A-01R-1503-07	stage iv	t3a	male		
TCGA-B0-5099-01A-01R-1420-07	stage iii	t3b	female		
TCGA-CZ-5468-01A-01R-1503-07	stage iv	t3b	male		
TCGA-B0-4693-01A-01R-1277-07	stage iii	t3a	female		
TCGA-BP-4162-01A-02R-1325-07	stage i	t1b	female		

TCGA-BP-4343-01A-02R-1289-07	stage iii	t3a	male		
TCGA-BP-4352-01A-01R-1289-07	stage iv	t3b	female		
TCGA-KN-8427-01A-11R-2315-07	stage iv	t4	male		
TCGA-B0-4841-01A-01R-1277-07	stage iv	t2	male		
TCGA-CJ-4868-01A-01R-1305-07	stage iv	t3a	male		
TCGA-BP-5198-01A-01R-1426-07	stage iii	t3b	male		
TCGA-CJ-4920-01A-01R-1426-07	stage i	t1b	female		
TCGA-BP-4335-01A-01R-1289-07	stage iv	t3a	female		
TCGA-DV-5569-01A-01R-1541-07	stage i	t1a	female		
TCGA-B0-4690-01A-01R-1277-07	stage iv	t4	male		
TCGA-CZ-5988-01A-11R-1672-07	stage i	t1b	male		
TCGA-CJ-4878-01A-01R-1305-07	stage iii	t3a	female		
TCGA-B0-5711-01A-11R-1672-07	stage iii	t3b	male		
TCGA-BP-4776-01A-01R-1289-07	stage i	t1a	male		
TCGA-BP-5168-01A-01R-1420-07	stage i	t1a	male		
TCGA-B0-5098-01A-01R-1420-07	stage i	t1	female		
TCGA-CJ-4871-01A-01R-1305-07	stage iv	t3a	male		
TCGA-B0-4813-01A-01R-1277-07	stage iii	t3b	male		
TCGA-B0-5710-01A-11R-1672-07	stage i	t1b	male		
TCGA-CZ-5461-01A-01R-1503-07	stage iv	t1b	male		
TCGA-A3-3331-01A-02R-1325-07	stage i	t1	female		
TCGA-BP-4774-01A-01R-1289-07	stage i	t1a	female		
TCGA-BP-5008-01A-01R-1334-07	stage i	t1a	male		
TCGA-BP-4169-01A-02R-1289-07	stage ii	t2	female		
TCGA-CJ-4872-01A-01R-1305-07	stage i	t1b	male		
TCGA-BP-4345-01A-01R-1289-07	stage iii	t3b	male		
TCGA-BP-4775-01A-01R-1289-07	stage i	t1a	female		
TCGA-BP-5009-01A-01R-1334-07	stage i	t1b	male		
TCGA-CJ-4902-01A-01R-1426-07	stage iii	t3a	male		

TCGA-B0-4846-01A-01R-1277-07	stage iv	t3a	male		
TCGA-BP-4329-01A-02R-1289-07	stage iii	t3a	male		
TCGA-B0-5108-01A-01R-1420-07	stage iii	t3a	male		
TCGA-AK-3428-01A-02R-1277-07	stage iii	t3b	male		
TCGA-AL-3466-01A-02R-1351-07	stage iv	t3b	male		
TCGA-B0-4849-01A-01R-1277-07	stage iii	t3a	male		
TCGA-B0-5080-01A-01R-1503-07	stage iv	t3a	male		
TCGA-A3-3320-01A-02R-1325-07	stage i	t1b	female		
TCGA-BQ-5876-01A-11R-1592-07	stage i	t1a	male		
TCGA-CJ-6028-01A-11R-1672-07	stage iv	t3a	male		
TCGA-B0-5706-01A-11R-1541-07	stage ii	t2	male		
TCGA-G6-A8L6-01A-11R-A37O-07	stage iv	t2a	male		
TCGA-BP-4972-01A-01R-1334-07	stage iii	t3a	female		
TCGA-A3-3387-01A-01R-1541-07	stage i	t1a	male		
TCGA-CZ-5467-01A-01R-1503-07	stage iii	t3a	female		
TCGA-A3-A6NL-01A-11R-A33J-07	stage i	t1b	female		
TCGA-CW-5580-01A-01R-1672-07	stage iv	t3a	female		
TCGA-DV-5568-01A-01R-1541-07	stage i	t1a	male		
TCGA-B0-4845-01A-01R-1277-07	stage iv	t3a	male		
TCGA-CJ-4907-01A-01R-1426-07	stage iii	t3b	male		
TCGA-A4-7287-01A-11R-2139-07	stage i	t1	female		
TCGA-B0-4718-01A-01R-1277-07	stage iii	t3a	male		
TCGA-B0-5812-01A-11R-1672-07	stage i	t1b	male		
TCGA-CJ-6033-01A-11R-1672-07	stage iv	t3a	female		
TCGA-A4-7828-01A-11R-2139-07	stage i	t1a	female		
TCGA-B8-A7U6-01A-12R-A37O-07	stage i	t1a	female		
TCGA-CZ-4861-01A-01R-1305-07	stage ii	t2	male		
TCGA-B2-4101-01A-02R-1277-07	stage ii	t2a	male		
TCGA-BP-4967-01A-01R-1334-07	stage iii	t3a	male		

TCGA-DW-7834-01A-11R-2139-07	stage i	t1	male			
TCGA-BP-4986-01A-01R-1334-07	stage i	t1a	male			
TCGA-B4-5844-01A-11R-1672-07	stage ii	t2	female			
TCGA-B0-5110-01A-01R-1420-07	stage i	t1a	female			
TCGA-BP-4160-01A-02R-1289-07	stage iii	t3a	male			
TCGA-CJ-4639-01A-02R-1325-07	stage ii	t2	female			
TCGA-A3-3347-01A-02R-1325-07	stage iii	t1b	female			
TCGA-CZ-5465-01A-01R-1503-07	stage iii	t3b	female			
TCGA-BP-5004-01A-01R-1334-07	stage i	t1a	male			
TCGA-B0-5694-01A-11R-1541-07	stage iii	t3a	male			
TCGA-BP-5189-01A-02R-1426-07	stage i	t1b	male			
TCGA-B0-4821-01A-01R-1503-07	stage iii	t3b	female			
TCGA-AK-3431-01A-02R-1277-07	stage ii	t2	female			
TCGA-BP-4991-01A-01R-1334-07	stage i	t1a	male			
TCGA-BP-4975-01A-01R-1334-07	stage i	t1b	male			
TCGA-A3-3316-01A-01R-0864-07	stage ii	t2	male			
TCGA-B0-4852-01A-01R-1503-07	stage ii	t2	female			
TCGA-A3-3373-01A-02R-1420-07	stage i	t1b	female			
TCGA-CZ-4858-01A-01R-1305-07	stage ii	t2	male			
TCGA-BP-4995-01A-01R-1334-07	stage i	t1b	male			
TCGA-B0-4819-01A-01R-1277-07	stage iv	t3b	female			
TCGA-B8-4143-01A-01R-1188-07	stage iv	t3a	female			
TCGA-BP-4166-01A-02R-1289-07	stage iii	t3a	male			
TCGA-B0-4700-01A-02R-1541-07	stage iv	t4	male			
TCGA-G7-6793-01A-11R-1965-07	stage iv	t3a	female			
TCGA-CZ-4857-01A-01R-1305-07	stage iv	t3a	male			
TCGA-BQ-5885-01A-11R-1592-07	stage iii	t3	male			
TCGA-CJ-4885-01A-01R-1305-07	stage iv	t3a	male			
TCGA-B0-5106-01A-01R-1420-07	stage i	t1a	male			

TCGA-AK-3440-01A-02R-1277-07	stage i	t1a	male		
TCGA-A3-3325-01A-01R-0864-07	stage i	t1a	male		
TCGA-B8-5163-01A-01R-1420-07	stage iii	t3a	female		
TCGA-B8-A8YJ-01A-13R-A39I-07	stage i	t1b	female		
TCGA-BP-4960-01A-01R-1334-07	stage ii	t2	male		
TCGA-UZ-A9PS-01A-11R-A42S-07	stage ii	t2	female		
TCGA-BP-5201-01A-01R-1426-07	stage iv	t3b	male		
TCGA-B0-4836-01A-01R-1305-07	stage iv	t3b	male		
TCGA-CZ-5454-01A-01R-1503-07	stage iv	t2	male		
TCGA-BP-5006-01A-01R-1334-07	stage i	t1a	male		
TCGA-B8-4622-01A-02R-1277-07	stage iv	t3a	male		
TCGA-B0-5096-01A-01R-1420-07	stage iii	t3a	female		
TCGA-AK-3436-01A-02R-1325-07	stage iv	t2	male		
TCGA-B0-4843-01A-01R-1277-07	stage iii	t3a	male		
TCGA-CJ-4881-01A-01R-1305-07	stage iii	t3a	male		
TCGA-CZ-5456-01A-01R-1503-07	stage ii	t2	male		
TCGA-B8-5549-01A-01R-1541-07	stage i	t1b	male		
TCGA-A3-3358-01A-01R-1541-07	stage i	t1a	female		
TCGA-CJ-6032-01A-11R-1672-07	stage ii	t2	female		
TCGA-CJ-4905-01A-02R-1426-07	stage i	t1a	female		
TCGA-P4-A5E8-01A-11R-A28H-07	stage iii	t2a	male		
TCGA-A3-3329-01A-01R-0864-07	stage i	t1b	male		
TCGA-B0-5402-01A-01R-1503-07	stage iv	t4	male		
TCGA-A3-3343-01A-01R-0864-07	stage ii	t2	male		
TCGA-BP-4963-01A-01R-1334-07	stage i	t1b	male		
TCGA-5P-A9KA-01A-11R-A42S-07	NA	t1a	male		
TCGA-BP-4341-01A-01R-1289-07	stage iii	t3a	male		
TCGA-BP-4994-01A-01R-1334-07	stage i	t1a	male		
TCGA-B0-5700-01A-11R-1541-07	stage i	t1a	male		

TCGA-A3-3365-01A-01R-0864-07	stage i	t1a	male			
TCGA-B0-4701-01A-01R-1277-07	stage iv	t3a	female			
TCGA-A3-3352-01A-01R-0864-07	stage iii	t3a	male			
TCGA-A3-3335-01A-01R-0864-07	stage ii	t2a	male			
TCGA-DW-7838-01A-11R-2139-07	stage i	t1b	male			
TCGA-A3-3351-01A-02R-1325-07	stage ii	t2a	male			
TCGA-CJ-6030-01A-11R-1672-07	stage i	t1a	male			
TCGA-AK-3426-01A-02R-1325-07	stage iii	t3a	male			
TCGA-G7-7501-01A-11R-2204-07	stage iii	t3a	female			
TCGA-BP-5177-01A-01R-1426-07	stage i	t1a	female			
TCGA-HE-7130-01A-11R-1965-07	stage iii	t3	female			
TCGA-CZ-5982-01A-11R-1672-07	stage i	t1a	female			
TCGA-CZ-5463-01A-01R-1503-07	stage ii	t2	male			
TCGA-BP-4964-01A-01R-1334-07	stage i	t1a	female			
TCGA-BP-4973-01A-01R-1334-07	stage iii	t3a	male			
TCGA-CZ-5455-01A-01R-1503-07	stage iv	t3b	male			
TCGA-B0-5116-01A-02R-1420-07	stage iii	t3b	male			
TCGA-B0-4839-01A-01R-1305-07	stage i	t1b	female			
TCGA-CJ-4901-01A-01R-1426-07	stage iii	t3b	male			
TCGA-B0-4816-01A-01R-1503-07	stage ii	t2	male			
TCGA-B0-5084-01A-01R-1334-07	stage iv	t3a	male			
TCGA-CJ-4893-01A-01R-1305-07	stage i	t1b	female			
TCGA-CZ-5986-01A-11R-1672-07	stage i	t1	male			
TCGA-G6-A8L8-01A-21R-A37O-07	stage i	t1b	female			
TCGA-CJ-4918-01A-01R-1426-07	stage iv	t3a	male			
TCGA-BP-4790-01A-01R-1305-07	stage i	t1a	male			
TCGA-B0-4699-01A-01R-1277-07	stage iv	t4	male			
TCGA-B0-5104-01A-01R-1420-07	stage i	t1	female			
TCGA-BP-4807-01A-01R-1305-07	stage i	t1a	male			

TCGA-BP-4770-01A-01R-1503-07	stage iv	t4	female		
TCGA-BP-4777-01A-01R-1289-07	stage i	t1a	male		
TCGA-B0-5088-01A-01R-1334-07	stage i	t1b	male		
TCGA-KO-8404-01A-11R-2315-07	stage iv	t4	male		
TCGA-Y8-A895-01A-11R-A36F-07	stage i	t1b	male		
TCGA-B0-4838-01A-01R-1305-07	stage i	t1b	female		
TCGA-CZ-4854-01A-01R-1305-07	stage i	t1b	male		
TCGA-CZ-4860-01A-01R-1305-07	stage iv	t4	male		
TCGA-B0-5120-01A-01R-1420-07	stage i	t1a	female		
TCGA-BP-5175-01A-01R-1426-07	stage i	t1a	male		
TCGA-B0-5115-01A-01R-1420-07	stage iv	t2	male		
TCGA-CJ-4888-01A-01R-1305-07	stage iv	t3a	male		
TCGA-B8-4154-01A-01R-1188-07	stage i	t1a	female		
TCGA-KN-8419-01A-11R-2315-07	stage ii	t2	male		
TCGA-KO-8405-01A-11R-2315-07	stage iii	t3a	male		
TCGA-B0-5113-01A-01R-1420-07	stage iii	t3a	female		
TCGA-CJ-4886-01A-01R-1305-07	stage i	t1a	female		
TCGA-CZ-5469-01A-01R-1503-07	stage ii	t2	male		
TCGA-BP-4982-01A-01R-1334-07	stage i	t1b	male		
TCGA-CJ-5676-01A-11R-1541-07	stage iii	t3b	male		
TCGA-B0-5081-01A-01R-1334-07	stage iii	t3b	female		
TCGA-CW-6093-01A-11R-1672-07	stage i	t1a	male		
TCGA-BP-4334-01A-01R-1289-07	stage iii	t3a	male		
TCGA-BP-4173-01A-02R-1289-07	stage ii	t2	male		
TCGA-BP-4327-01A-01R-1289-07	stage ii	t2	female		
TCGA-KL-8333-01A-11R-2315-07	stage ii	t2	male		
TCGA-B8-5551-01A-01R-1541-07	stage i	t1b	female		
TCGA-AK-3460-01A-02R-1277-07	stage i	t1a	male		
TCGA-BP-4974-01A-01R-1334-07	stage iv	t3a	male		

TCGA-B0-5119-01A-02R-1420-07	stage i	t1b	female		
TCGA-CJ-4884-01A-01R-1305-07	stage iii	t3a	female		
TCGA-CW-6090-01A-11R-1672-07	stage i	t1b	male		
TCGA-DV-5566-01A-01R-1541-07	stage i	t1a	female		
TCGA-BP-4762-01A-02R-1289-07	stage i	t1a	male		
TCGA-B0-4714-01A-01R-1277-07	stage iv	t3b	male		
TCGA-A3-3346-01A-01R-1766-07	stage i	t1b	male		
TCGA-CJ-4916-01A-01R-1426-07	stage iii	t3a	female		
TCGA-B4-5834-01A-11R-1672-07	stage i	t1	male		
TCGA-BP-4787-01A-01R-1305-07	stage iv	t3a	female		
TCGA-BP-4981-01A-01R-1334-07	stage iii	t3a	female		
TCGA-BP-4797-01A-01R-1305-07	stage iii	t3b	male		
TCGA-CJ-4900-01A-01R-1334-07	stage iv	t4	female		
TCGA-B0-5695-01A-11R-1541-07	stage i	t1b	female		
TCGA-CJ-4891-01A-01R-1305-07	stage iii	t3c	female		
TCGA-GK-A6C7-01A-11R-A33J-07	stage i	t1a	female		
TCGA-A3-3378-01A-02R-1325-07	stage i	t1	male		
TCGA-B0-4848-01A-01R-1277-07	stage iii	t3b	male		
TCGA-BP-4332-01A-01R-1289-07	stage iii	t3a	male		
TCGA-A3-3363-01A-01R-0864-07	stage ii	t2	male		<25 Percentile
TCGA-AL-7173-01A-11R-2139-07	stage iv	t3	female		
TCGA-2Z-A9JL-01A-11R-A42S-07	stage i	t1a	male		
TCGA-B3-8121-01A-21R-2404-07	stage i	t1	female		
TCGA-B9-A69E-01A-11R-A31O-07	stage iii	t3a	male		
TCGA-G7-6797-01A-11R-1965-07	stage iii	t1a	male		
TCGA-A3-3326-01A-01R-0864-07	stage i	t1a	male		
TCGA-A4-A5Y0-01A-11R-A31O-07	stage i	t1b	female		
TCGA-BP-4346-01A-01R-1289-07	stage iii	t3b	male		
TCGA-DZ-6134-01A-11R-1965-07	stage i	t1a	male		

TCGA-BP-4761-01A-01R-1289-07	stage iii	t3a	male		
TCGA-B0-5712-01A-11R-1672-07	stage iv	t2	female		
TCGA-KL-8338-01A-11R-2315-07	stage iii	t3a	male		
TCGA-SX-A71R-01A-12R-A33Z-07	stage i	t1a	male		
TCGA-A3-3349-01A-01R-1188-07	stage i	t1b	female		
TCGA-KN-8422-01A-11R-2315-07	stage i	t1a	female		
TCGA-G7-6789-01A-11R-1965-07	stage iv	t3a	female		
TCGA-KM-8440-01A-11R-2315-07	stage iii	t3a	male		
TCGA-CZ-5459-01A-01R-1503-07	stage iii	t3b	male		
TCGA-B4-5836-01A-11R-1672-07	stage i	t1b	female		
TCGA-BQ-7051-01A-12R-1965-07	stage ii	t2	male		
TCGA-CW-5587-01A-01R-1541-07	stage iii	t3b	female		
TCGA-B1-A47M-01A-11R-A24Z-07	stage iii	t3a	male		
TCGA-B8-A54G-01A-11R-A266-07	stage i	t1a	male		
TCGA-AK-3427-01A-01R-0864-07	stage i	t1a	male		
TCGA-B1-A47N-01A-11R-A24Z-07	stage i	t1a	male		
TCGA-UZ-A9PO-01A-11R-A38C-07	stage i	t1a	male		
TCGA-A4-A772-01A-11R-A33Z-07	stage i	t1b	male		
TCGA-KO-8415-01A-11R-2315-07	stage i	t1	female		
TCGA-BP-4969-01A-01R-1334-07	stage i	t1a	female		
TCGA-J7-6720-01A-11R-2139-07	stage i	t1	male		
TCGA-DZ-6132-01A-11R-1965-07	stage i	t1a	male		
TCGA-UZ-A9PM-01A-21R-A38C-07	stage ii	t2	male		
TCGA-KL-8345-01A-11R-2315-07	stage iii	t3a	male		
TCGA-MW-A4EC-01A-11R-A266-07	stage i	t1a	female		
TCGA-BP-4782-01A-02R-1420-07	stage i	t1a	female		
TCGA-EV-5903-01A-11R-1592-07	stage i	t1a	male		
TCGA-CJ-4634-01A-02R-1325-07	stage i	t1b	female		
TCGA-Y8-A8S0-01A-11R-A37K-07	stage i	t1a	male		

TCGA-KL-8326-01A-11R-2315-07	stage iii	t3a	male		
TCGA-B9-A44B-01A-11R-A24Z-07	stage iii	t3b	male		
TCGA-B8-5165-01A-01R-1420-07	stage i	t1a	male		
TCGA-A4-8630-01A-11R-2404-07	stage i	t1b	female		
TCGA-KL-8332-01A-11R-2315-07	stage i	t1b	male		
TCGA-KN-8433-01A-11R-2315-07	stage iii	t3a	female		
TCGA-B0-5107-01A-01R-1420-07	stage iv	t2	female		
TCGA-BQ-7048-01A-11R-1965-07	stage iii	t3a	male		
TCGA-UZ-A9PX-01A-11R-A42S-07	stage i	t1	male		
TCGA-A3-A8OV-01A-11R-A37O-07	stage i	t1a	male		
TCGA-BQ-7055-01A-11R-1965-07	stage i	t1a	male		
TCGA-CZ-5457-01A-01R-1503-07	stage iii	t3a	male		
TCGA-B0-4707-01A-01R-1277-07	stage iii	t3a	male		
TCGA-B8-A54H-01A-11R-A33J-07	stage ii	t2a	female		
TCGA-6D-AA2E-01A-11R-A37O-07	stage i	t1b	female		
TCGA-UZ-A9PZ-01A-11R-A42S-07	stage iii	t3a	male		
TCGA-BP-5190-01A-01R-1426-07	stage i	t1a	male		
TCGA-2Z-A9JG-01A-11R-A42S-07	stage i	t1a	male		
TCGA-KL-8334-01A-11R-2315-07	stage iii	t3a	female		
TCGA-B8-5159-01A-01R-1420-07	stage i	t1a	female		
TCGA-A4-8515-01A-11R-2404-07	stage i	t1a	male		
TCGA-BP-5186-01A-01R-1426-07	stage i	t1a	female		
TCGA-B9-4113-01A-01R-1193-07	stage i	t1	male		
TCGA-KO-8411-01A-11R-2315-07	stage i	t1b	male		
TCGA-4A-A93W-01A-11R-A37K-07	stage i	t1a	female		
TCGA-2Z-A9JR-01A-12R-A42S-07	stage i	t1a	male		
TCGA-2Z-A9J1-01A-11R-A38C-07	stage i	t1a	male		
TCGA-B3-3926-01A-02R-1351-07	stage i	t1	female		
TCGA-KN-8426-01A-11R-2315-07	stage iv	t3a	male		

TCGA-IZ-A6M9-01A-11R-A31O-07	stage i	t1a	male			
TCGA-GL-A59R-01A-11R-A26U-07	stage iii	t3c	male			
TCGA-IA-A40U-01A-11R-A24Z-07	stage iii	t3b	male			
TCGA-B0-4706-01A-01R-1503-07	stage iii	t3a	male			
TCGA-A4-7584-01A-11R-2139-07	stage i	t1a	male			
TCGA-Y8-A8RY-01A-11R-A37K-07	stage i	t1b	male			
TCGA-BP-5185-01A-01R-1426-07	stage i	t1a	male			
TCGA-BQ-5887-01A-11R-1965-07	stage iii	t3a	male			
TCGA-A3-3362-01A-02R-1325-07	stage i	t1a	female			
TCGA-CZ-5452-01A-01R-1503-07	stage ii	t2	male			
TCGA-BP-4976-01A-01R-1334-07	stage i	t1a	male			
TCGA-F9-A7VF-01A-11R-A33Z-07	stage i	t1a	female			
TCGA-SX-A71V-01A-11R-A33Z-07	stage i	t1	male			
TCGA-B9-A5W9-01A-11R-A28H-07	stage i	t1b	male			
TCGA-F9-A7Q0-01A-11R-A36F-07	stage i	t1b	female			
TCGA-MM-A84U-01A-11R-A37O-07	stage i	t1a	female			
TCGA-KN-8428-01A-11R-2315-07	stage ii	t2	male			
TCGA-KO-8409-01A-11R-2315-07	stage ii	t2b	male			
TCGA-CJ-4638-01A-02R-1325-07	stage iv	t3a	female			
TCGA-GL-6846-01A-11R-1965-07	stage iv	t4	male			
TCGA-P4-AAVL-01A-11R-A42S-07	stage iii	t3b	male			
TCGA-2Z-A9J3-01A-12R-A38C-07	stage ii	t2	male			
TCGA-J7-A8I2-01A-12R-A36F-07	stage i	t1b	male			
TCGA-G7-A8LB-01A-11R-A36F-07	stage iv	t2a	male			
TCGA-KM-8477-01A-11R-2315-07	stage i	t1a	male			
TCGA-A4-8516-01A-11R-2404-07	stage iii	t3a	male			
TCGA-BQ-5891-01A-11R-1592-07	stage iii	t3b	female			
TCGA-BQ-5882-01A-11R-1592-07	stage iii	t3b	male			
TCGA-2Z-A9J5-01A-21R-A38C-07	stage ii	t2	male			

TCGA-P4-A5E6-01A-11R-A28H-07	stage iii	t1b	male		
TCGA-UZ-A9PQ-01A-11R-A42S-07	stage iii	t2	male		
TCGA-B3-A6W5-01A-12R-A33Z-07	stage i	t1	male		
TCGA-A3-A8OW-01A-11R-A37O-07	stage iii	t3a	male		
TCGA-A3-A8CQ-01A-11R-A37O-07	stage i	t1a	female		
TCGA-2Z-A9J9-01A-11R-A42S-07	stage i	t1a	male		
TCGA-A4-7583-01A-11R-A32Z-07	stage i	t1	male		
TCGA-HE-A5NH-01A-11R-A26U-07	stage i	t1a	male		
TCGA-KM-8476-01A-11R-2315-07	stage i	t1	male		
TCGA-2Z-A9J8-01A-11R-A42S-07	stage i	t1b	male		
TCGA-UZ-A9PL-01A-11R-A38C-07	stage ii	t2	male		
TCGA-SX-A7SS-01A-11R-A36F-07	stage i	t1	male		
TCGA-BQ-5881-01A-11R-1592-07	stage i	t1a	male		
TCGA-P4-AAVM-01A-11R-A42S-07	stage i	t1a	male		
TCGA-KL-8344-01A-11R-2315-07	stage iii	t3a	male		
TCGA-PJ-A5Z9-01A-11R-A28H-07	stage i	t1b	female		
TCGA-B8-A54E-01A-11R-A266-07	stage i	t1b	female		
TCGA-SX-A7SL-01A-11R-A355-07	stage i	t1a	male		
TCGA-Y8-A897-01A-11R-A36F-07	stage i	t1a	female		
TCGA-KL-8337-01A-11R-2315-07	stage ii	t2	male		
TCGA-BP-5174-01A-01R-1426-07	stage i	t1a	female		
TCGA-EV-5902-01A-11R-1592-07	stage i	t1	male		
TCGA-B2-5636-01A-02R-1541-07	stage i	t1a	male		
TCGA-HE-7129-01A-11R-1965-07	stage ii	t2	male		
TCGA-BQ-7058-01A-11R-1965-07	stage iii	t3	male		
TCGA-AS-3777-01A-01R-0864-07	stage i	t1a	male		
TCGA-KL-8330-01A-11R-2315-07	stage ii	t2b	female		
TCGA-BQ-5875-01A-11R-1592-07	stage iii	t3a	female		
TCGA-IA-A83V-01A-11R-A355-07	stage i	t1b	male		

TCGA-G7-6795-01A-11R-1965-07	stage i	t1a	male		
TCGA-KN-8418-01A-11R-2315-07	stage ii	t2	female		
TCGA-HE-A5NF-01A-11R-A26U-07	stage i	t1a	male		
TCGA-BQ-5878-01A-11R-1592-07	stage iii	t3a	female		
TCGA-GL-A9DC-01A-11R-A37K-07	stage i	t1b	female		
TCGA-BP-4756-01A-01R-1289-07	stage i	t1b	female		
TCGA-B0-4834-01A-01R-1305-07	stage i	t1a	male		
TCGA-UN-AAZ9-01A-11R-A38C-07	stage i	t1b	female		
TCGA-F9-A97G-01A-11R-A38C-07	stage iii	t3	male		
TCGA-KN-8432-01A-11R-2315-07	stage ii	t2b	female		
TCGA-BQ-5884-01A-11R-1592-07	stage i	t1a	female		
TCGA-KO-8414-01A-11R-2315-07	stage ii	t2	female		
TCGA-Y8-A894-01A-11R-A36F-07	stage i	t1a	female		
TCGA-B1-7332-01A-11R-A32Z-07	stage ii	t2a	female		
TCGA-4A-A93X-01A-11R-A37K-07	stage iv	t3a	male		
TCGA-T7-A92I-01A-11R-A37O-07	stage i	t1a	female		
TCGA-A4-7585-01A-11R-2139-07	stage iii	t3a	male		
TCGA-SX-A71W-01A-12R-A355-07	stage i	t1b	male		
TCGA-KL-8341-01A-11R-2315-07	stage iv	t3b	male		
TCGA-2Z-A9JS-01A-21R-A42S-07	stage i	t1a	male		
TCGA-BQ-7044-01A-11R-1965-07	stage iii	t3a	male		
TCGA-KL-8343-01A-11R-2315-07	stage iii	t3	male		
TCGA-GL-A59T-01A-21R-A28H-07	stage i	t1b	male		
TCGA-KN-8437-01A-11R-2315-07	stage i	t1a	female		
TCGA-A4-8310-01A-11R-2404-07	stage iii	t3a	male		
TCGA-Q2-A5QZ-01A-11R-A28H-07	stage iii	t3a	female		
TCGA-WN-A9G9-01A-12R-A37K-07	stage i	t1b	male		
TCGA-UZ-A9PP-01A-11R-A42S-07	stage i	t1a	male		
TCGA-BQ-7050-01A-11R-1965-07	stage i	t1a	female		

TCGA-2Z-A9JN-01A-21R-A42S-07	stage i	t1a	female		
TCGA-2Z-A9JO-01A-11R-A42S-07	stage i	t1b	female		
TCGA-F9-A8NY-01A-11R-A36F-07	stage iv	t4	female		
TCGA-B1-A656-01A-11R-A31O-07	stage i	t1a	male		
TCGA-MH-A855-01A-11R-A355-07	stage ii	t2b	female		
TCGA-KM-8439-01A-11R-2315-07	stage i	t1b	male		
TCGA-BQ-7053-01A-11R-1965-07	stage iii	t3	female		
TCGA-BQ-5880-01A-11R-1592-07	stage iii	t3a	male		
TCGA-AT-A5NU-01A-11R-A28H-07	stage i	t1a	male		
TCGA-2Z-A9JP-01A-11R-A42S-07	stage i	t1a	male		
TCGA-B1-A654-01A-11R-A31O-07	stage i	t1a	female		
TCGA-IZ-A6M8-01A-11R-A31O-07	stage i	t1a	male		
TCGA-MH-A854-01A-11R-A355-07	stage i	t1b	female		
TCGA-GL-A9DE-01A-11R-A37K-07	stage i	t1b	male		
TCGA-A4-8312-01A-11R-2404-07	stage i	t1	male		
TCGA-SX-A7SO-01A-11R-A355-07	stage i	t1b	male		
TCGA-BQ-5883-01A-11R-1592-07	stage i	t1a	female		
TCGA-BQ-7056-01A-11R-1965-07	stage iii	t3b	female		
TCGA-2Z-A9J7-01A-11R-A38C-07	stage iv	t2	male		
TCGA-G6-A5PC-01A-11R-A33J-07	stage iv	t1b	female		
TCGA-B8-5546-01A-01R-1541-07	stage i	t1b	female		
TCGA-2Z-A9JE-01A-11R-A42S-07	stage i	t1a	male		
TCGA-GL-8500-01A-11R-2404-07	stage i	t1b	male		
TCGA-A4-8098-01A-11R-2404-07	stage i	t1a	male		
TCGA-KN-8423-01A-11R-2315-07	stage i	t1b	male		
TCGA-IZ-8195-01A-31R-2404-07	stage ii	t2a	male		
TCGA-A4-A6HP-01A-11R-A31O-07	stage i	t1a	male		
TCGA-B9-A8YI-01A-21R-A37K-07	stage i	t1b	male		
TCGA-P4-A5EB-01A-11R-A28H-07	stage i	t1b	male		

TCGA-A4-A48D-01A-11R-A24Z-07	stage i	t1b	male		
TCGA-P4-AAVK-01A-11R-A42S-07	stage iii	t3a	male		
TCGA-DW-7839-01A-11R-2139-07	stage i	t1b	female		
TCGA-MH-A857-01A-11R-A355-07	stage i	t1a	male		
TCGA-2Z-A9J2-01A-11R-A38C-07	stage i	t1a	female		
TCGA-KN-8436-01A-11R-2315-07	stage ii	t2	male		
TCGA-IA-A40X-01A-11R-A24Z-07	stage i	t1a	female		
TCGA-MH-A55W-01A-11R-A26U-07	stage i	t1b	male		
TCGA-2Z-A9JQ-01A-11R-A42S-07	stage i	t1a	male		
TCGA-MH-A562-01A-11R-A26U-07	stage i	t1a	male		
TCGA-GL-A4EM-01A-11R-A24Z-07	stage i	t1b	male		
TCGA-KM-8639-01A-11R-2403-07	stage i	t1a	male		
TCGA-2Z-A9JT-01A-11R-A42S-07	stage i	t1a	male		
TCGA-P4-AAVO-01A-11R-A42S-07	stage i	t1b	male		
TCGA-B1-5398-01A-02R-1592-07	stage iii	t3b	male		
TCGA-Y8-A8S1-01A-11R-A37K-07	stage i	t1a	male		
TCGA-MH-A560-01A-11R-A26U-07	stage i	t1a	male		
TCGA-AK-3453-01A-02R-1277-07	stage ii	t2	female		
TCGA-B0-4696-01A-01R-1277-07	stage iii	t3a	male		
TCGA-KN-8421-01A-11R-2315-07	stage ii	t2	female		
TCGA-B0-5702-01A-11R-1541-07	stage i	t1b	male		
TCGA-A3-3376-01A-02R-1420-07	stage i	t1a	male	ETS1	>75 percentile
TCGA-CW-6093-01A-11R-1672-07	stage i	t1a	male		
TCGA-BP-5200-01A-01R-1426-07	stage ii	t2	male		
TCGA-CZ-5989-01A-11R-1672-07	stage ii	t2	male		
TCGA-BP-4974-01A-01R-1334-07	stage iv	t3a	male		
TCGA-BP-4961-01A-01R-1334-07	stage i	t1a	male		
TCGA-CJ-4904-01A-02R-1426-07	stage iv	t3a	female		
TCGA-CJ-4905-01A-02R-1426-07	stage i	t1a	female		

TCGA-BP-4789-01A-01R-1305-07	stage i	t1a	male		
TCGA-BP-4991-01A-01R-1334-07	stage i	t1a	male		
TCGA-B0-5097-01A-01R-1420-07	stage iii	t3b	female		
TCGA-CJ-4908-01A-01R-1426-07	stage i	t1a	male		
TCGA-CJ-6032-01A-11R-1672-07	stage ii	t2	female		
TCGA-CZ-5982-01A-11R-1672-07	stage i	t1a	female		
TCGA-B0-5690-01A-11R-1541-07	stage i	t1b	female		
TCGA-B8-A8YJ-01A-13R-A39I-07	stage i	t1b	female		
TCGA-BP-4999-01A-01R-1334-07	stage i	t1a	male		
TCGA-B0-5710-01A-11R-1672-07	stage i	t1b	male		
TCGA-BP-4344-01A-01R-1289-07	stage i	t1a	female		
TCGA-B2-5636-01A-02R-1541-07	stage i	t1a	male		
TCGA-CJ-4899-01A-01R-1334-07	stage i	t1b	male		
TCGA-B8-5165-01A-01R-1420-07	stage i	t1a	male		
TCGA-BP-4353-01A-02R-1289-07	stage i	t1	male		
TCGA-B4-5844-01A-11R-1672-07	stage ii	t2	female		
TCGA-B0-5110-01A-01R-1420-07	stage i	t1a	female		
TCGA-B0-5812-01A-11R-1672-07	stage i	t1b	male		
TCGA-CJ-4918-01A-01R-1426-07	stage iv	t3a	male		
TCGA-BP-4993-01A-02R-1420-07	stage i	t1a	male		
TCGA-BP-4982-01A-01R-1334-07	stage i	t1b	male		
TCGA-BP-4765-01A-01R-1289-07	stage i	t1a	male		
TCGA-DV-5569-01A-01R-1541-07	stage i	t1a	female		
TCGA-BP-5008-01A-01R-1334-07	stage i	t1a	male		
TCGA-AK-3461-01A-02R-1277-07	stage i	t1a	male		
TCGA-BP-4965-01A-01R-1334-07	stage i	t1a	male		
TCGA-GK-A6C7-01A-11R-A33J-07	stage i	t1a	female		
TCGA-B4-5377-01A-01R-1503-07	stage iv	t3	female		
TCGA-B8-5163-01A-01R-1420-07	stage iii	t3a	female		

TCGA-BP-5006-01A-01R-1334-07	stage i	t1a	male		
TCGA-CJ-4878-01A-01R-1305-07	stage iii	t3a	female		
TCGA-A3-3365-01A-01R-0864-07	stage i	t1a	male		
TCGA-CW-6088-01A-11R-1672-07	stage i	t1b	male		
TCGA-BP-5194-01A-02R-1426-07	stage i	t1a	male		
TCGA-B8-4148-01A-02R-1325-07	stage i	t1a	female		
TCGA-BP-4345-01A-01R-1289-07	stage iii	t3b	male		
TCGA-A3-3349-01A-01R-1188-07	stage i	t1b	female		
TCGA-B8-A54J-01A-11R-A33J-07	stage ii	t2a	male		
TCGA-DV-5574-01A-01R-1541-07	stage i	t1a	male		
TCGA-DV-5575-01A-01R-1541-07	stage i	t1a	female		
TCGA-BP-4762-01A-02R-1289-07	stage i	t1a	male		
TCGA-BP-4795-01A-02R-1420-07	stage i	t1a	female		
TCGA-B0-5698-01A-11R-1672-07	stage i	t1b	male		
TCGA-B4-5378-01A-01R-1503-07	stage i	t1	male		
TCGA-AK-3431-01A-02R-1277-07	stage ii	t2	female		
TCGA-B4-5834-01A-11R-1672-07	stage i	t1	male		
TCGA-CZ-4856-01A-02R-1426-07	stage i	t1b	female		
TCGA-A3-A8OW-01A-11R-A37O-07	stage iii	t3a	male		
TCGA-BP-5189-01A-02R-1426-07	stage i	t1b	male		
TCGA-B8-5553-01A-01R-1541-07	stage i	t1b	female		
TCGA-B2-A4SR-01A-11R-A266-07	stage ii	t2a	male		
TCGA-EU-5906-01A-11R-1672-07	stage i	t1b	male		
TCGA-BP-4355-01A-01R-1289-07	stage iii	t3a	female		
TCGA-BP-5187-01A-01R-1426-07	stage i	t1a	male		
TCGA-BP-4347-01A-01R-1289-07	stage iii	t3b	male		
TCGA-B0-5104-01A-01R-1420-07	stage i	t1	female		
TCGA-B8-A7U6-01A-12R-A37O-07	stage i	t1a	female		
TCGA-CJ-4886-01A-01R-1305-07	stage i	t1a	female		

TCGA-B0-5119-01A-02R-1420-07	stage i	t1b	female		
TCGA-B8-5162-01A-01R-1420-07	stage ii	t2a	male		
TCGA-B0-5693-01A-11R-1541-07	stage i	t1b	female		
TCGA-BP-5195-01A-02R-1426-07	stage i	t1a	male		
TCGA-B0-5121-01A-02R-1420-07	stage i	t1b	male		
TCGA-BP-4763-01A-01R-1289-07	stage i	t1a	female		
TCGA-A3-A8CQ-01A-11R-A37O-07	stage i	t1a	female		
TCGA-BP-4970-01A-01R-1334-07	stage iii	t1a	male		
TCGA-CJ-4643-01A-02R-1325-07	stage ii	t2b	female		
TCGA-EU-5904-01A-11R-1672-07	stage i	t1	female		
TCGA-CZ-5467-01A-01R-1503-07	stage iii	t3a	female		
TCGA-B0-5080-01A-01R-1503-07	stage iv	t3a	male		
TCGA-B0-4945-01A-01R-1420-07	stage i	t1a	female		
TCGA-DV-5567-01A-01R-1541-07	stage i	t1a	female		
TCGA-CJ-4893-01A-01R-1305-07	stage i	t1b	female		
TCGA-BP-4162-01A-02R-1325-07	stage i	t1b	female		
TCGA-BP-4975-01A-01R-1334-07	stage i	t1b	male		
TCGA-BP-4774-01A-01R-1289-07	stage i	t1a	female		
TCGA-BP-5184-01A-01R-1426-07	stage i	t1a	male		
TCGA-BP-4987-01A-01R-1334-07	stage i	t1b	female		
TCGA-B0-4838-01A-01R-1305-07	stage i	t1b	female		
TCGA-BP-4169-01A-02R-1289-07	stage ii	t2	female		
TCGA-BP-4170-01A-02R-1289-07	stage i	t1b	female		
TCGA-AK-3428-01A-02R-1277-07	stage iii	t3b	male		
TCGA-DV-5565-01A-01R-1541-07	stage i	t1a	male		
TCGA-A3-3307-01A-01R-0864-07	stage iii	t3b	male		
TCGA-CW-5583-01A-02R-1541-07	stage i	t1a	female		
TCGA-CZ-5455-01A-01R-1503-07	stage iv	t3b	male		
TCGA-CJ-4870-01A-01R-1305-07	stage iii	t3a	female		

TCGA-A3-3370-01A-02R-1420-07	stage i	t1b	female			
TCGA-CJ-4907-01A-01R-1426-07	stage iii	t3b	male			
TCGA-CJ-4872-01A-01R-1305-07	stage i	t1b	male			
TCGA-B0-5108-01A-01R-1420-07	stage iii	t3a	male			
TCGA-A3-A6NJ-01A-12R-A33J-07	stage i	t1a	female			
TCGA-BP-4160-01A-02R-1289-07	stage iii	t3a	male			
TCGA-CJ-4639-01A-02R-1325-07	stage ii	t2	female			
TCGA-CJ-4874-01A-01R-1305-07	stage i	t1b	female			
TCGA-A4-7828-01A-11R-2139-07	stage i	t1a	female			
TCGA-BP-4166-01A-02R-1289-07	stage iii	t3a	male			
TCGA-BP-4799-01A-01R-1305-07	stage iii	t3b	male			
TCGA-B0-5711-01A-11R-1672-07	stage iii	t3b	male			
TCGA-BP-5201-01A-01R-1426-07	stage iv	t3b	male			
TCGA-CJ-4888-01A-01R-1305-07	stage iv	t3a	male			
TCGA-A3-3373-01A-02R-1420-07	stage i	t1b	female			
TCGA-CJ-4890-01A-01R-1305-07	stage iv	t3a	male			
TCGA-B0-5695-01A-11R-1541-07	stage i	t1b	female			
TCGA-BP-4784-01A-01R-1305-07	stage i	t1a	female			
TCGA-B8-5545-01A-01R-1672-07	stage i	t1a	male			
TCGA-B8-A54H-01A-11R-A33J-07	stage ii	t2a	female			
TCGA-B0-4849-01A-01R-1277-07	stage iii	t3a	male			
TCGA-B4-5832-01A-11R-1672-07	stage iii	t3b	male			
TCGA-B0-5713-01A-11R-1672-07	stage iii	t3b	female			
TCGA-B0-4846-01A-01R-1277-07	stage iv	t3a	male			
TCGA-B0-5120-01A-01R-1420-07	stage i	t1a	female			
TCGA-B0-4701-01A-01R-1277-07	stage iv	t3a	female			
TCGA-B0-5700-01A-11R-1541-07	stage i	t1a	male			
TCGA-BP-4790-01A-01R-1305-07	stage i	t1a	male			
TCGA-CJ-4634-01A-02R-1325-07	stage i	t1b	female			

TCGA-BP-5186-01A-01R-1426-07	stage i	t1a	female		
TCGA-BP-4766-01A-01R-1289-07	stage i	t1a	female		
TCGA-B4-5836-01A-11R-1672-07	stage i	t1b	female		
TCGA-CJ-5671-01A-11R-1541-07	stage i	t1a	male		
TCGA-BP-4173-01A-02R-1289-07	stage ii	t2	male		
TCGA-B2-4101-01A-02R-1277-07	stage ii	t2a	male		
TCGA-B0-4823-01A-02R-1420-07	stage i	t1a	male		
TCGA-A3-3372-01A-02R-1325-07	stage iii	t3	male		
TCGA-AK-3454-01A-02R-1277-07	stage i	t1b	male		
TCGA-BP-4326-01A-01R-1289-07	stage i	t1b	female		
TCGA-CJ-6028-01A-11R-1672-07	stage iv	t3a	male		
TCGA-B2-5633-01A-01R-1541-07	stage i	t1b	male		
TCGA-BP-5177-01A-01R-1426-07	stage i	t1a	female		
TCGA-DV-5576-01A-01R-1541-07	stage i	t1a	female		
TCGA-BP-4801-01A-02R-1420-07	stage i	t1a	male		
TCGA-CZ-5988-01A-11R-1672-07	stage i	t1b	male		
TCGA-A3-A6NL-01A-11R-A33J-07	stage i	t1b	female		
TCGA-BP-5199-01A-01R-1426-07	stage ii	t2	male		
TCGA-A3-3326-01A-01R-0864-07	stage i	t1a	male		
TCGA-BP-5168-01A-01R-1420-07	stage i	t1a	male		
TCGA-CW-5588-01A-01R-1541-07	stage i	t1a	female		
TCGA-BP-5198-01A-01R-1426-07	stage iii	t3b	male		
TCGA-CZ-5457-01A-01R-1503-07	stage iii	t3a	male		
TCGA-A3-3320-01A-02R-1325-07	stage i	t1b	female		
TCGA-BP-4964-01A-01R-1334-07	stage i	t1a	female		
TCGA-AK-3460-01A-02R-1277-07	stage i	t1a	male		
TCGA-CJ-4920-01A-01R-1426-07	stage i	t1b	female		
TCGA-BP-4329-01A-02R-1289-07	stage iii	t3a	male		
TCGA-A3-3359-01A-01R-0864-07	stage i	t1a	female		

TCGA-BP-4159-01A-02R-1289-07	stage i	t1b	male		
TCGA-CJ-4902-01A-01R-1426-07	stage iii	t3a	male		
TCGA-BP-4988-01A-01R-1334-07	stage i	t1a	male		
TCGA-CW-5589-01A-01R-1541-07	stage i	t1a	male		
TCGA-B0-5113-01A-01R-1420-07	stage iii	t3a	female		
TCGA-BP-4777-01A-01R-1289-07	stage i	t1a	male		
TCGA-B0-4852-01A-01R-1503-07	stage ii	t2	female		
TCGA-CZ-5458-01A-01R-1503-07	stage iii	t3a	male		
TCGA-CZ-4862-01A-01R-1305-07	stage i	t1b	male		
TCGA-B8-5549-01A-01R-1541-07	stage i	t1b	male		
TCGA-B0-5402-01A-01R-1503-07	stage iv	t4	male		
TCGA-BP-4771-01A-01R-1289-07	stage iv	t3a	male		
TCGA-BP-4782-01A-02R-1420-07	stage i	t1a	female		
TCGA-A3-3380-01A-01R-0864-07	stage i	t1	male		
TCGA-B0-5095-01A-01R-1420-07	stage iii	t3a	male		
TCGA-BP-4332-01A-01R-1289-07	stage iii	t3a	male		
TCGA-B0-5115-01A-01R-1420-07	stage iv	t2	male		
TCGA-CW-6097-01A-11R-1672-07	stage iii	t3a	male		
TCGA-BP-5202-01A-02R-1426-07	stage iii	t3a	male		
TCGA-CJ-5684-01A-11R-1541-07	stage iii	t3a	male		
TCGA-B0-4845-01A-01R-1277-07	stage iv	t3a	male		
TCGA-CW-5580-01A-01R-1672-07	stage iv	t3a	female		
TCGA-BP-4759-01A-01R-1289-07	stage i	t1a	male		
TCGA-A3-A6NN-01A-12R-A33J-07	stage i	t1a	male		
TCGA-CZ-4863-01A-01R-1503-07	stage iii	t3b	female		
TCGA-B4-5843-01A-11R-1672-07	stage i	t1	male		
TCGA-CZ-5986-01A-11R-1672-07	stage i	t1	male		
TCGA-B0-4693-01A-01R-1277-07	stage iii	t3a	female		
TCGA-B0-4816-01A-01R-1503-07	stage ii	t2	male		

TCGA-CJ-4889-01A-01R-1305-07	stage i	t1a	female		
TCGA-B0-5081-01A-01R-1334-07	stage iii	t3b	female		
TCGA-DV-A4W0-01A-11R-A266-07	stage i	t1b	male		
TCGA-BP-4330-01A-01R-1289-07	stage iii	t3a	female		
TCGA-BP-4758-01A-01R-1289-07	stage i	t1a	male		
TCGA-A3-3331-01A-02R-1325-07	stage i	t1	female		
TCGA-CJ-4868-01A-01R-1305-07	stage iv	t3a	male		
TCGA-B0-4818-01A-01R-1503-07	stage ii	t2	female		
TCGA-B0-5116-01A-02R-1420-07	stage iii	t3b	male		
TCGA-CZ-5461-01A-01R-1503-07	stage iv	t1b	male		
TCGA-B0-4819-01A-01R-1277-07	stage iv	t3b	female		
TCGA-CJ-5689-01A-11R-1541-07	stage i	t1b	male		
TCGA-BP-5176-01A-01R-1426-07	stage i	t1a	female		
TCGA-BP-4976-01A-01R-1334-07	stage i	t1a	male		
TCGA-BP-4354-01A-02R-1289-07	stage iv	t4	male		
TCGA-CZ-4865-01A-02R-1503-07	stage i	t1a	female		
TCGA-BP-4803-01A-01R-1305-07	stage iii	t3a	male		
TCGA-BP-4349-01A-01R-1289-07	stage i	t1a	female		
TCGA-CJ-4901-01A-01R-1426-07	stage iii	t3b	male		
TCGA-A3-3308-01A-02R-1325-07	stage iii	t3b	female		
TCGA-A3-3323-01A-02R-1325-07	stage i	t1b	male		
TCGA-CZ-5451-01A-01R-1503-07	stage ii	t2	male		
TCGA-CZ-4859-01A-02R-1426-07	stage i	t1	female		
TCGA-B0-5691-01A-11R-1541-07	stage i	t1a	female		
TCGA-BP-4989-01A-01R-1334-07	stage iii	t3a	male		
TCGA-A3-3316-01A-01R-0864-07	stage ii	t2	male		
TCGA-CW-5590-01A-01R-1541-07	stage iv	t3a	male		
TCGA-CJ-4916-01A-01R-1426-07	stage iii	t3a	female		
TCGA-CJ-4897-01A-03R-1426-07	stage iii	t3a	female		

TCGA-BP-5004-01A-01R-1334-07	stage i	t1a	male		
TCGA-CJ-4871-01A-01R-1305-07	stage iv	t3a	male		
TCGA-BP-4158-01A-02R-1289-07	stage i	t1b	male		
TCGA-CJ-6031-01A-11R-1672-07	stage i	t1b	male		
TCGA-AK-3434-01A-02R-1277-07	stage i	t1b	male		
TCGA-BP-4760-01A-02R-1420-07	stage i	t1a	male		
TCGA-CW-5587-01A-01R-1541-07	stage iii	t3b	female		
TCGA-B0-5099-01A-01R-1420-07	stage iii	t3b	female		
TCGA-BP-4998-01A-01R-1334-07	stage i	t1a	male		
TCGA-UZ-A9PK-01A-11R-A38C-07	stage i	t1	male		
TCGA-A3-3363-01A-01R-0864-07	stage ii	t2	male		
TCGA-BQ-5878-01A-11R-1592-07	stage iii	t3a	female		
TCGA-B2-4098-01A-02R-1325-07	stage i	t1b	female		
TCGA-KO-8416-01A-11R-2315-07	stage iii	t3a	male		
TCGA-BQ-5877-01A-11R-1592-07	stage iv	t3a	male		
TCGA-AL-3466-01A-02R-1351-07	stage iv	t3b	male		
TCGA-B9-A44B-01A-11R-A24Z-07	stage iii	t3b	male		
TCGA-KL-8336-01A-11R-2315-07	stage iv	t3b	female		
TCGA-B0-5702-01A-11R-1541-07	stage i	t1b	male		<25 Percentile
TCGA-5P-A9JU-01A-11R-A42S-07	stage iii	t3a	male		
TCGA-2Z-A9JG-01A-11R-A42S-07	stage i	t1a	male		
TCGA-A4-A7UZ-01A-12R-A355-07	stage iii	t3a	male		
TCGA-GL-A9DD-01A-11R-A37K-07	stage i	t1a	male		
TCGA-GL-A9DC-01A-11R-A37K-07	stage i	t1b	female		
TCGA-B8-4621-01A-01R-1503-07	stage i	t1b	male		
TCGA-BP-4352-01A-01R-1289-07	stage iv	t3b	female		
TCGA-B9-4113-01A-01R-1193-07	stage i	t1	male		
TCGA-MH-A55W-01A-11R-A26U-07	stage i	t1b	male		
TCGA-A4-8098-01A-11R-2404-07	stage i	t1a	male		

TCGA-B9-A5W7-01A-11R-A31O-07	stage i	t1a	male		
TCGA-F9-A97G-01A-11R-A38C-07	stage iii	t3	male		
TCGA-IA-A83V-01A-11R-A355-07	stage i	t1b	male		
TCGA-J7-6720-01A-11R-2139-07	stage i	t1	male		
TCGA-DW-7840-01A-11R-A32Z-07	stage i	t1b	male		
TCGA-UZ-A9PO-01A-11R-A38C-07	stage i	t1a	male		
TCGA-KL-8323-01A-21R-2315-07	stage iii	t3b	female		
TCGA-GL-A4EM-01A-11R-A24Z-07	stage i	t1b	male		
TCGA-B0-5707-01A-11R-1541-07	stage i	t1a	female		
TCGA-G7-6790-01A-11R-1965-07	stage i	t1a	male		
TCGA-KL-8327-01A-11R-2315-07	stage i	t1b	female		
TCGA-A4-A4ZT-01A-11R-A26U-07	stage i	t1b	female		
TCGA-2Z-A9JI-01A-11R-A42S-07	stage iii	t3a	male		
TCGA-2Z-A9JL-01A-11R-A42S-07	stage i	t1a	male		
TCGA-KM-8476-01A-11R-2315-07	stage i	t1	male		
TCGA-V9-A7HT-01A-11R-A33Z-07	stage ii	t2	male		
TCGA-SX-A7SU-01A-11R-A36F-07	stage i	t1	male		
TCGA-F9-A7Q0-01A-11R-A36F-07	stage i	t1b	female		
TCGA-SX-A7SN-01A-11R-A355-07	stage i	t1b	male		
TCGA-KN-8433-01A-11R-2315-07	stage iii	t3a	female		
TCGA-AK-3440-01A-02R-1277-07	stage i	t1a	male		
TCGA-KN-8426-01A-11R-2315-07	stage iv	t3a	male		
TCGA-KO-8417-01A-11R-2315-07	stage i	t1	female		
TCGA-HE-A5NL-01A-11R-A26U-07	stage i	t1a	male		
TCGA-B1-A655-01A-11R-A31O-07	stage i	t1a	female		
TCGA-A4-A48D-01A-11R-A24Z-07	stage i	t1b	male		
TCGA-KN-8435-01A-11R-2315-07	stage ii	t2	male		
TCGA-KM-8442-01A-11R-2315-07	stage ii	t2	male		
TCGA-B9-A5W9-01A-11R-A28H-07	stage i	t1b	male		

TCGA-Y8-A897-01A-11R-A36F-07	stage i	t1a	female		
TCGA-G7-7502-01A-11R-2204-07	stage i	t1b	male		
TCGA-KL-8334-01A-11R-2315-07	stage iii	t3a	female		
TCGA-HE-A5NH-01A-11R-A26U-07	stage i	t1a	male		
TCGA-DZ-6131-01A-11R-1965-07	stage iii	t3a	male		
TCGA-UZ-A9PM-01A-21R-A38C-07	stage ii	t2	male		
TCGA-IA-A40U-01A-11R-A24Z-07	stage iii	t3b	male		
TCGA-BQ-7050-01A-11R-1965-07	stage i	t1a	female		
TCGA-A4-A57E-01A-11R-A26U-07	stage iv	t2a	male		
TCGA-A4-A5Y1-01A-11R-A28H-07	stage iii	t1b	male		
TCGA-A4-8516-01A-11R-2404-07	stage iii	t3a	male		
TCGA-B8-A54E-01A-11R-A266-07	stage i	t1b	female		
TCGA-AK-3447-01A-01R-1766-07	stage ii	t2	male		
TCGA-A4-7584-01A-11R-2139-07	stage i	t1a	male		
TCGA-Y8-A8RY-01A-11R-A37K-07	stage i	t1b	male		
TCGA-4A-A93X-01A-11R-A37K-07	stage iv	t3a	male		
TCGA-A4-8517-01A-11R-2404-07	stage i	t1a	male		
TCGA-EV-5902-01A-11R-1592-07	stage i	t1	male		
TCGA-B3-4103-01A-02R-1351-07	stage i	t1a	male		
TCGA-KV-A6GE-01A-11R-A31O-07	stage i	t1a	male		
TCGA-B1-A657-01A-11R-A31O-07	stage i	t1b	male		
TCGA-KN-8436-01A-11R-2315-07	stage ii	t2	male		
TCGA-KN-8429-01A-11R-2315-07	stage iii	t3a	female		
TCGA-BQ-7046-01A-11R-1965-07	stage i	t1a	male		
TCGA-A4-8312-01A-11R-2404-07	stage i	t1	male		
TCGA-B9-7268-01A-11R-A32Z-07	stage ii	t2	male		
TCGA-HE-A5NF-01A-11R-A26U-07	stage i	t1a	male		
TCGA-P4-AAVK-01A-11R-A42S-07	stage iii	t3a	male		
TCGA-HE-A5NI-01A-11R-A26U-07	stage i	t1a	male		

TCGA-MH-A55Z-01A-11R-A26U-07	stage i	t1b	male		
TCGA-BQ-5881-01A-11R-1592-07	stage i	t1a	male		
TCGA-2Z-A9J1-01A-11R-A38C-07	stage i	t1a	male		
TCGA-2Z-A9JD-01A-11R-A42S-07	stage i	t1a	male		
TCGA-AL-3471-01A-02R-1351-07	stage i	t1b	male		
TCGA-2Z-A9JR-01A-12R-A42S-07	stage i	t1a	male		
TCGA-2Z-A9JS-01A-21R-A42S-07	stage i	t1a	male		
TCGA-SX-A7SS-01A-11R-A36F-07	stage i	t1	male		
TCGA-BQ-5888-01A-11R-1592-07	stage i	t1a	female		
TCGA-SX-A7SQ-01A-12R-A36F-07	stage i	t1	male		
TCGA-KN-8422-01A-11R-2315-07	stage i	t1a	female		
TCGA-B3-A6W5-01A-12R-A33Z-07	stage i	t1	male		
TCGA-DZ-6133-01A-11R-1965-07	stage i	t1a	female		
TCGA-B1-7332-01A-11R-A32Z-07	stage ii	t2a	female		
TCGA-G7-A8LC-01A-11R-A36F-07	stage i	t1a	male		
TCGA-GL-8500-01A-11R-2404-07	stage i	t1b	male		
TCGA-BQ-5890-01A-11R-1592-07	stage iii	t3a	male		
TCGA-UZ-A9PX-01A-11R-A42S-07	stage i	t1	male		
TCGA-2Z-A9JO-01A-11R-A42S-07	stage i	t1b	female		
TCGA-MH-A854-01A-11R-A355-07	stage i	t1b	female		
TCGA-SX-A71W-01A-12R-A355-07	stage i	t1b	male		
TCGA-DW-5560-01A-01R-1592-07	stage i	t1b	female		
TCGA-UZ-A9PQ-01A-11R-A42S-07	stage iii	t2	male		
TCGA-A4-A5XZ-01A-11R-A31O-07	stage ii	t2a	female		
TCGA-SX-A7SO-01A-11R-A355-07	stage i	t1b	male		
TCGA-A4-A5DU-01A-11R-A28H-07	stage i	t1a	female		
TCGA-A4-8311-01A-11R-2404-07	stage i	t1a	male		
TCGA-2Z-A9JE-01A-11R-A42S-07	stage i	t1a	male		
TCGA-AT-A5NU-01A-11R-A28H-07	stage i	t1a	male		

TCGA-2Z-A9J6-01A-11R-A38C-07	stage i	t1a	male		
TCGA-2Z-A9J8-01A-11R-A42S-07	stage i	t1b	male		
TCGA-A4-A772-01A-11R-A33Z-07	stage i	t1b	male		
TCGA-B1-A654-01A-11R-A31O-07	stage i	t1a	female		
TCGA-KO-8408-01A-11R-2315-07	stage iii	t3a	male		
TCGA-KV-A74V-01A-11R-A33Z-07	stage i	t1a	male		
TCGA-KL-8346-01A-11R-2315-07	stage ii	t2b	male		
TCGA-KO-8415-01A-11R-2315-07	stage i	t1	female		
TCGA-SX-A7SR-01A-12R-A36F-07	stage ii	t2a	male		
TCGA-P4-A5EB-01A-11R-A28H-07	stage i	t1b	male		
TCGA-BQ-7060-01A-11R-1965-07	stage i	t1a	male		
TCGA-KL-8335-01A-11R-2315-07	stage iii	t3a	male		
TCGA-GL-A59R-01A-11R-A26U-07	stage iii	t3c	male		
TCGA-B1-A47O-01A-11R-A24Z-07	stage i	t1b	female		
TCGA-AK-3427-01A-01R-0864-07	stage i	t1a	male		
TCGA-Y8-A8S0-01A-11R-A37K-07	stage i	t1a	male		
TCGA-KL-8338-01A-11R-2315-07	stage iii	t3a	male		
TCGA-SX-A7SP-01A-11R-A355-07	stage i	t1b	female		
TCGA-SX-A71U-01A-12R-A33Z-07	stage i	t1a	male		
TCGA-KO-8409-01A-11R-2315-07	stage ii	t2b	male		
TCGA-UZ-A9PU-01A-11R-A42S-07	stage i	t1b	male		
TCGA-Y8-A895-01A-11R-A36F-07	stage i	t1b	male		
TCGA-KN-8418-01A-11R-2315-07	stage ii	t2	female		
TCGA-A4-8518-01A-11R-2404-07	stage i	t1a	male		
TCGA-SX-A71R-01A-12R-A33Z-07	stage i	t1a	male		
TCGA-KL-8326-01A-11R-2315-07	stage iii	t3a	male		
TCGA-P4-A5E6-01A-11R-A28H-07	stage iii	t1b	male		
TCGA-KL-8341-01A-11R-2315-07	stage iv	t3b	male		
TCGA-IZ-8195-01A-31R-2404-07	stage ii	t2a	male		

TCGA-G7-6797-01A-11R-1965-07	stage iii	t1a	male		
TCGA-A4-8630-01A-11R-2404-07	stage i	t1b	female		
TCGA-BQ-5875-01A-11R-1592-07	stage iii	t3a	female		
TCGA-B1-A47N-01A-11R-A24Z-07	stage i	t1a	male		
TCGA-KO-8414-01A-11R-2315-07	stage ii	t2	female		
TCGA-KN-8428-01A-11R-2315-07	stage ii	t2	male		
TCGA-B9-A8YI-01A-21R-A37K-07	stage i	t1b	male		
TCGA-A4-A5Y0-01A-11R-A31O-07	stage i	t1b	female		
TCGA-KM-8440-01A-11R-2315-07	stage iii	t3a	male		
TCGA-UZ-A9Q1-01A-11R-A42S-07	stage i	t1b	female		
TCGA-G7-A8LD-01A-11R-A36F-07	stage iii	t3a	male		
TCGA-UZ-A9PS-01A-11R-A42S-07	stage ii	t2	female		
TCGA-B9-4115-01A-01R-1193-07	stage i	t1a	male		
TCGA-MH-A562-01A-11R-A26U-07	stage i	t1a	male		
TCGA-2Z-A9J5-01A-21R-A38C-07	stage ii	t2	male		
TCGA-DW-7839-01A-11R-2139-07	stage i	t1b	female		
TCGA-A4-8310-01A-11R-2404-07	stage iii	t3a	male		
TCGA-WN-AB4C-01A-11R-A42S-07	stage i	t1a	female		
TCGA-SX-A71V-01A-11R-A33Z-07	stage i	t1	male		
TCGA-KL-8337-01A-11R-2315-07	stage ii	t2	male		
TCGA-A4-A6HP-01A-11R-A31O-07	stage i	t1a	male		
TCGA-Y8-A898-01A-11R-A355-07	stage i	t1a	male		
TCGA-UZ-A9PL-01A-11R-A38C-07	stage ii	t2	male		
TCGA-KL-8331-01A-11R-2315-07	stage ii	t2	female		
TCGA-A4-8515-01A-11R-2404-07	stage i	t1a	male		
TCGA-A3-3328-01A-01R-0864-07	stage i	t1b	male		
TCGA-2Z-A9JT-01A-11R-A42S-07	stage i	t1a	male		
TCGA-BP-4334-01A-01R-1289-07	stage iii	t3a	male		
TCGA-IA-A40Y-01A-11R-A24Z-07	stage iii	t3a	female		

TCGA-BQ-5891-01A-11R-1592-07	stage iii	t3b	female		
TCGA-Y8-A8RZ-01A-11R-A37K-07	stage i	t1	male		
TCGA-2Z-A9JP-01A-11R-A42S-07	stage i	t1a	male		
TCGA-UZ-A9PZ-01A-11R-A42S-07	stage iii	t3a	male		
TCGA-2Z-A9J3-01A-12R-A38C-07	stage ii	t2	male		
TCGA-UZ-A9PV-01A-11R-A42S-07	stage i	t1	male		
TCGA-PJ-A5Z9-01A-11R-A28H-07	stage i	t1b	female		
TCGA-UZ-A9Q0-01A-12R-A42S-07	stage i	t1	male		
TCGA-G7-6795-01A-11R-1965-07	stage i	t1a	male		
TCGA-GL-A59T-01A-21R-A28H-07	stage i	t1b	male		
TCGA-F9-A7VF-01A-11R-A33Z-07	stage i	t1a	female		
TCGA-5P-A9JW-01A-11R-A42S-07	NA	t1a	male		
TCGA-KL-8325-01A-11R-2315-07	stage ii	t2	female		
TCGA-KL-8330-01A-11R-2315-07	stage ii	t2b	female		
TCGA-SX-A7SL-01A-11R-A355-07	stage i	t1a	male		
TCGA-2Z-A9JQ-01A-11R-A42S-07	stage i	t1a	male		
TCGA-5P-A9KE-01A-11R-A42S-07	NA	t1b	male		
TCGA-B1-A47M-01A-11R-A24Z-07	stage iii	t3a	male		
TCGA-5P-A9JY-01A-11R-A42S-07	NA	t2	male		
TCGA-AL-A5DJ-01A-11R-A26U-07	stage iii	t3a	female		
TCGA-HE-7129-01A-11R-1965-07	stage ii	t2	male		
TCGA-BQ-7051-01A-12R-1965-07	stage ii	t2	male		
TCGA-BQ-7044-01A-11R-1965-07	stage iii	t3a	male		
TCGA-2Z-A9J7-01A-11R-A38C-07	stage iv	t2	male		
TCGA-KM-8443-01A-11R-2315-07	stage ii	t2	male		
TCGA-G7-A8LB-01A-11R-A36F-07	stage iv	t2a	male		
TCGA-BQ-5886-01A-11R-1592-07	stage iii	t3a	male		
TCGA-IZ-A6M8-01A-11R-A31O-07	stage i	t1a	male		
TCGA-B9-5155-01A-01R-1592-07	stage iii	t3a	male		

TCGA-P4-AAVM-01A-11R-A42S-07	stage i	t1a	male		
TCGA-MH-A560-01A-11R-A26U-07	stage i	t1a	male		
TCGA-2Z-A9J2-01A-11R-A38C-07	stage i	t1a	female		
TCGA-Q2-A5QZ-01A-11R-A28H-07	stage iii	t3a	female		
TCGA-IZ-A6M9-01A-11R-A31O-07	stage i	t1a	male		
TCGA-P4-AAVO-01A-11R-A42S-07	stage i	t1b	male		
TCGA-5P-A9K2-01A-11R-A42S-07	NA	t1b	male		
TCGA-KN-8421-01A-11R-2315-07	stage ii	t2	female		
TCGA-B0-5097-01A-01R-1420-07	stage iii	t3b	female	ABL2	>75 percentile
TCGA-KN-8427-01A-11R-2315-07	stage iv	t4	male		
TCGA-CZ-5988-01A-11R-1672-07	stage i	t1b	male		
TCGA-B0-5096-01A-01R-1420-07	stage iii	t3a	female		
TCGA-B8-A8YJ-01A-13R-A39I-07	stage i	t1b	female		
TCGA-B8-4620-01A-02R-1325-07	stage iii	t3a	female		
TCGA-B0-4841-01A-01R-1277-07	stage iv	t2	male		
TCGA-A3-3382-01A-02R-1325-07	stage i	t1b	male		
TCGA-CJ-4908-01A-01R-1426-07	stage i	t1a	male		
TCGA-BP-4961-01A-01R-1334-07	stage i	t1a	male		
TCGA-BP-4354-01A-02R-1289-07	stage iv	t4	male		
TCGA-BP-4986-01A-01R-1334-07	stage i	t1a	male		
TCGA-B0-5108-01A-01R-1420-07	stage iii	t3a	male		
TCGA-BP-4326-01A-01R-1289-07	stage i	t1b	female		
TCGA-BP-4352-01A-01R-1289-07	stage iv	t3b	female		
TCGA-DV-5565-01A-01R-1541-07	stage i	t1a	male		
TCGA-BP-4974-01A-01R-1334-07	stage iv	t3a	male		
TCGA-B8-5162-01A-01R-1420-07	stage ii	t2a	male		
TCGA-BP-5189-01A-02R-1426-07	stage i	t1b	male		
TCGA-BP-4176-01A-02R-1289-07	stage i	t1b	male		
TCGA-CZ-5982-01A-11R-1672-07	stage i	t1a	female		

TCGA-BP-4169-01A-02R-1289-07	stage ii	t2	female		
TCGA-BP-4781-01A-01R-1305-07	stage i	t1a	male		
TCGA-DV-5574-01A-01R-1541-07	stage i	t1a	male		
TCGA-CZ-5467-01A-01R-1503-07	stage iii	t3a	female		
TCGA-BP-5200-01A-01R-1426-07	stage ii	t2	male		
TCGA-A3-3316-01A-01R-0864-07	stage ii	t2	male		
TCGA-B8-5163-01A-01R-1420-07	stage iii	t3a	female		
TCGA-CW-6090-01A-11R-1672-07	stage i	t1b	male		
TCGA-CJ-4904-01A-02R-1426-07	stage iv	t3a	female		
TCGA-CJ-4891-01A-01R-1305-07	stage iii	t3c	female		
TCGA-BP-5008-01A-01R-1334-07	stage i	t1a	male		
TCGA-B0-5710-01A-11R-1672-07	stage i	t1b	male		
TCGA-BP-4991-01A-01R-1334-07	stage i	t1a	male		
TCGA-A3-3373-01A-02R-1420-07	stage i	t1b	female		
TCGA-B0-5110-01A-01R-1420-07	stage i	t1a	female		
TCGA-CW-5580-01A-01R-1672-07	stage iv	t3a	female		
TCGA-BP-4803-01A-01R-1305-07	stage iii	t3a	male		
TCGA-CJ-4868-01A-01R-1305-07	stage iv	t3a	male		
TCGA-B0-5080-01A-01R-1503-07	stage iv	t3a	male		
TCGA-CJ-4918-01A-01R-1426-07	stage iv	t3a	male		
TCGA-BP-4345-01A-01R-1289-07	stage iii	t3b	male		
TCGA-CJ-4902-01A-01R-1426-07	stage iii	t3a	male		
TCGA-A3-3308-01A-02R-1325-07	stage iii	t3b	female		
TCGA-BP-4355-01A-01R-1289-07	stage iii	t3a	female		
TCGA-CJ-4635-01A-02R-1305-07	stage i	t1b	male		
TCGA-BP-4985-01A-01R-1334-07	stage iii	t3a	male		
TCGA-BP-4353-01A-02R-1289-07	stage i	t1	male		
TCGA-B8-5545-01A-01R-1672-07	stage i	t1a	male		
TCGA-BP-5195-01A-02R-1426-07	stage i	t1a	male		

TCGA-A3-3323-01A-02R-1325-07	stage i	t1b	male			
TCGA-CW-6097-01A-11R-1672-07	stage iii	t3a	male			
TCGA-BP-4993-01A-02R-1420-07	stage i	t1a	male			
TCGA-B0-5690-01A-11R-1541-07	stage i	t1b	female			
TCGA-GL-7966-01A-11R-2204-07	stage iii	t3	female			
TCGA-BP-5198-01A-01R-1426-07	stage iii	t3b	male			
TCGA-B4-5844-01A-11R-1672-07	stage ii	t2	female			
TCGA-B0-4819-01A-01R-1277-07	stage iv	t3b	female			
TCGA-AK-3431-01A-02R-1277-07	stage ii	t2	female			
TCGA-B2-5633-01A-01R-1541-07	stage i	t1b	male			
TCGA-CJ-5676-01A-11R-1541-07	stage iii	t3b	male			
TCGA-CJ-4905-01A-02R-1426-07	stage i	t1a	female			
TCGA-BQ-5879-01A-11R-1592-07	stage iii	t3b	female			
TCGA-B0-4703-01A-01R-1277-07	stage iv	t3a	male			
TCGA-CW-6093-01A-11R-1672-07	stage i	t1a	male			
TCGA-AL-3466-01A-02R-1351-07	stage iv	t3b	male			
TCGA-B0-4688-01A-01R-1277-07	stage iv	t4	male			
TCGA-B0-5698-01A-11R-1672-07	stage i	t1b	male			
TCGA-A3-3376-01A-02R-1420-07	stage i	t1a	male			
TCGA-B0-4822-01A-01R-1277-07	stage ii	t2	male			
TCGA-B0-5109-01A-02R-1420-07	stage iii	t3b	male			
TCGA-B0-5095-01A-01R-1420-07	stage iii	t3a	male			
TCGA-BP-5175-01A-01R-1426-07	stage i	t1a	male			
TCGA-BP-5196-01A-01R-1426-07	stage i	t1a	male			
TCGA-A3-3347-01A-02R-1325-07	stage iii	t1b	female			
TCGA-BP-4342-01A-01R-1289-07	stage ii	t2	male			
TCGA-GK-A6C7-01A-11R-A33J-07	stage i	t1a	female			
TCGA-B4-5832-01A-11R-1672-07	stage iii	t3b	male			
TCGA-CJ-5671-01A-11R-1541-07	stage i	t1a	male			

TCGA-BP-4963-01A-01R-1334-07	stage i	t1b	male		
TCGA-BP-4967-01A-01R-1334-07	stage iii	t3a	male		
TCGA-CJ-4890-01A-01R-1305-07	stage iv	t3a	male		
TCGA-BP-4998-01A-01R-1334-07	stage i	t1a	male		
TCGA-CW-5590-01A-01R-1541-07	stage iv	t3a	male		
TCGA-BP-4784-01A-01R-1305-07	stage i	t1a	female		
TCGA-A3-3372-01A-02R-1325-07	stage iii	t3	male		
TCGA-BP-5004-01A-01R-1334-07	stage i	t1a	male		
TCGA-BP-4770-01A-01R-1503-07	stage iv	t4	female		
TCGA-DV-5568-01A-01R-1541-07	stage i	t1a	male		
TCGA-CZ-4857-01A-01R-1305-07	stage iv	t3a	male		
TCGA-BP-4960-01A-01R-1334-07	stage ii	t2	male		
TCGA-BP-4988-01A-01R-1334-07	stage i	t1a	male		
TCGA-B0-4690-01A-01R-1277-07	stage iv	t4	male		
TCGA-CJ-5689-01A-11R-1541-07	stage i	t1b	male		
TCGA-B0-5121-01A-02R-1420-07	stage i	t1b	male		
TCGA-B0-5116-01A-02R-1420-07	stage iii	t3b	male		
TCGA-KL-8333-01A-11R-2315-07	stage ii	t2	male		
TCGA-CJ-4899-01A-01R-1334-07	stage i	t1b	male		
TCGA-CZ-4860-01A-01R-1305-07	stage iv	t4	male		
TCGA-BP-4787-01A-01R-1305-07	stage iv	t3a	female		
TCGA-CZ-5454-01A-01R-1503-07	stage iv	t2	male		
TCGA-B0-4816-01A-01R-1503-07	stage ii	t2	male		
TCGA-BP-5010-01A-02R-1420-07	stage iii	t3a	male		
TCGA-CJ-4888-01A-01R-1305-07	stage iv	t3a	male		
TCGA-BP-5170-01A-01R-1426-07	stage i	t1a	male		
TCGA-CJ-5679-01A-11R-1541-07	stage iii	t3b	male		
TCGA-BP-4349-01A-01R-1289-07	stage i	t1a	female		
TCGA-BP-4982-01A-01R-1334-07	stage i	t1b	male		

TCGA-BP-4789-01A-01R-1305-07	stage i	t1a	male		
TCGA-CJ-4637-01A-02R-1325-07	stage iv	t2b	female		
TCGA-BP-4999-01A-01R-1334-07	stage i	t1a	male		
TCGA-BP-4170-01A-02R-1289-07	stage i	t1b	female		
TCGA-B0-5106-01A-01R-1420-07	stage i	t1a	male		
TCGA-B0-4718-01A-01R-1277-07	stage iii	t3a	male		
TCGA-B9-4116-01A-02R-1351-07	NA	t2a	female		
TCGA-B8-5549-01A-01R-1541-07	stage i	t1b	male		
TCGA-CJ-6033-01A-11R-1672-07	stage iv	t3a	female		
TCGA-CZ-5989-01A-11R-1672-07	stage ii	t2	male		
TCGA-B8-4143-01A-01R-1188-07	stage iv	t3a	female		
TCGA-BP-4799-01A-01R-1305-07	stage iii	t3b	male		
TCGA-BP-4797-01A-01R-1305-07	stage iii	t3b	male		
TCGA-CZ-4861-01A-01R-1305-07	stage ii	t2	male		
TCGA-B0-5812-01A-11R-1672-07	stage i	t1b	male		
TCGA-BP-5178-01A-01R-1426-07	stage iv	t3a	male		
TCGA-B2-5636-01A-02R-1541-07	stage i	t1a	male		
TCGA-B8-A54F-01A-11R-A266-07	stage i	t1a	female		
TCGA-B0-5402-01A-01R-1503-07	stage iv	t4	male		
TCGA-A3-A6NN-01A-12R-A33J-07	stage i	t1a	male		
TCGA-BP-4771-01A-01R-1289-07	stage iv	t3a	male		
TCGA-G7-A8LD-01A-11R-A36F-07	stage iii	t3a	male		
TCGA-B0-4698-01A-01R-1503-07	stage iv	t4	male		
TCGA-B0-5104-01A-01R-1420-07	stage i	t1	female		
TCGA-CZ-4854-01A-01R-1305-07	stage i	t1b	male		
TCGA-AK-3461-01A-02R-1277-07	stage i	t1a	male		
TCGA-A3-3317-01A-02R-1325-07	stage ii	t2	male		
TCGA-CJ-4881-01A-01R-1305-07	stage iii	t3a	male		
TCGA-CJ-6030-01A-11R-1672-07	stage i	t1a	male		

TCGA-B8-5158-01A-01R-1420-07	stage iii	t3a	male		
TCGA-B8-A7U6-01A-12R-A37O-07	stage i	t1a	female		
TCGA-BP-5187-01A-01R-1426-07	stage i	t1a	male		
TCGA-B8-4622-01A-02R-1277-07	stage iv	t3a	male		
TCGA-CJ-6027-01A-11R-1672-07	stage i	t1a	male		
TCGA-B0-5113-01A-01R-1420-07	stage iii	t3a	female		
TCGA-CJ-6032-01A-11R-1672-07	stage ii	t2	female		
TCGA-B2-4101-01A-02R-1277-07	stage ii	t2a	male		
TCGA-B0-5706-01A-11R-1541-07	stage ii	t2	male		
TCGA-CJ-4634-01A-02R-1325-07	stage i	t1b	female		
TCGA-CJ-4907-01A-01R-1426-07	stage iii	t3b	male		
TCGA-B0-5693-01A-11R-1541-07	stage i	t1b	female		
TCGA-CJ-6028-01A-11R-1672-07	stage iv	t3a	male		
TCGA-A3-A6NL-01A-11R-A33J-07	stage i	t1b	female		
TCGA-EU-5904-01A-11R-1672-07	stage i	t1	female		
TCGA-CZ-5456-01A-01R-1503-07	stage ii	t2	male		
TCGA-B0-5092-01A-01R-1420-07	stage iv	t1a	female		
TCGA-DV-5569-01A-01R-1541-07	stage i	t1a	female		
TCGA-CZ-5984-01A-11R-1672-07	stage i	t1b	male		
TCGA-BP-4166-01A-02R-1289-07	stage iii	t3a	male		
TCGA-B8-5551-01A-01R-1541-07	stage i	t1b	female		
TCGA-A4-A5Y1-01A-11R-A28H-07	stage iii	t1b	male		
TCGA-B0-4815-01A-01R-1503-07	stage iii	t3a	male		
TCGA-BP-4983-01A-01R-1334-07	stage iii	t3a	female		
TCGA-A3-3378-01A-02R-1325-07	stage i	t1	male		
TCGA-CJ-5686-01A-11R-1672-07	stage i	t1b	female		
TCGA-CZ-4862-01A-01R-1305-07	stage i	t1b	male		
TCGA-BP-4763-01A-01R-1289-07	stage i	t1a	female		
TCGA-BP-4790-01A-01R-1305-07	stage i	t1a	male		

TCGA-B0-4836-01A-01R-1305-07	stage iv	t3b	male		
TCGA-DV-5567-01A-01R-1541-07	stage i	t1a	female		
TCGA-B0-5115-01A-01R-1420-07	stage iv	t2	male		
TCGA-CW-6087-01A-11R-1672-07	stage iv	t3a	male		
TCGA-DV-5576-01A-01R-1541-07	stage i	t1a	female		
TCGA-B4-5834-01A-11R-1672-07	stage i	t1	male		
TCGA-CJ-4871-01A-01R-1305-07	stage iv	t3a	male		
TCGA-B8-5165-01A-01R-1420-07	stage i	t1a	male		
TCGA-BQ-5877-01A-11R-1592-07	stage iv	t3a	male		
TCGA-EU-5906-01A-11R-1672-07	stage i	t1b	male		
TCGA-AK-3454-01A-02R-1277-07	stage i	t1b	male		
TCGA-BP-4965-01A-01R-1334-07	stage i	t1a	male		
TCGA-B8-5550-01A-01R-1541-07	stage iii	t3a	male		
TCGA-BP-4162-01A-02R-1325-07	stage i	t1b	female		
TCGA-CJ-4912-01A-01R-1426-07	stage ii	t2	male		
TCGA-CJ-4643-01A-02R-1325-07	stage ii	t2b	female		
TCGA-A4-7915-01A-11R-2204-07	stage ii	t2b	female		
TCGA-B8-5553-01A-01R-1541-07	stage i	t1b	female		
TCGA-CJ-4640-01A-02R-1325-07	stage iii	t3a	male		
TCGA-B0-4697-01A-01R-1277-07	stage iv	t3b	female		
TCGA-B0-4821-01A-01R-1503-07	stage iii	t3b	female		
TCGA-B0-4945-01A-01R-1420-07	stage i	t1a	female		
TCGA-CJ-4886-01A-01R-1305-07	stage i	t1a	female		
TCGA-BP-5199-01A-01R-1426-07	stage ii	t2	male		
TCGA-B0-4700-01A-02R-1541-07	stage iv	t4	male		
TCGA-BP-4970-01A-01R-1334-07	stage iii	t1a	male		
TCGA-B8-A54J-01A-11R-A33J-07	stage ii	t2a	male		
TCGA-A3-3320-01A-02R-1325-07	stage i	t1b	female		
TCGA-BP-5009-01A-01R-1334-07	stage i	t1b	male		

TCGA-A3-3387-01A-01R-1541-07	stage i	t1a	male		
TCGA-DV-A4VZ-01A-11R-A266-07	stage i	t1a	male		
TCGA-CW-5588-01A-01R-1541-07	stage i	t1a	female		
TCGA-B0-4837-01A-01R-1305-07	stage i	t1b	male		
TCGA-B0-5120-01A-01R-1420-07	stage i	t1a	female		
TCGA-DW-7838-01A-11R-2139-07	stage i	t1b	male		
TCGA-EU-5907-01A-11R-1672-07	stage iii	t3a	male		
TCGA-CZ-5458-01A-01R-1503-07	stage iii	t3a	male		
TCGA-BP-5194-01A-02R-1426-07	stage i	t1a	male		
TCGA-CJ-4920-01A-01R-1426-07	stage i	t1b	female		
TCGA-B4-5377-01A-01R-1503-07	stage iv	t3	female		
TCGA-G7-6793-01A-11R-1965-07	stage iv	t3a	female		
TCGA-DV-A4W0-01A-11R-A266-07	stage i	t1b	male		
TCGA-EU-5905-01A-11R-1672-07	stage i	t1	female		
TCGA-B4-5378-01A-01R-1503-07	stage i	t1	male		
TCGA-BP-4782-01A-02R-1420-07	stage i	t1a	female		
TCGA-CJ-4903-01A-01R-1426-07	stage i	t1b	male		
TCGA-A4-7828-01A-11R-2139-07	stage i	t1a	female		
TCGA-B2-A4SR-01A-11R-A266-07	stage ii	t2a	male		
TCGA-CZ-5468-01A-01R-1503-07	stage iv	t3b	male		
TCGA-BP-4160-01A-02R-1289-07	stage iii	t3a	male		
TCGA-CZ-5986-01A-11R-1672-07	stage i	t1	male		
TCGA-BQ-5893-01A-11R-1592-07	stage iv	t3a	male		
TCGA-BP-4173-01A-02R-1289-07	stage ii	t2	male		
TCGA-A3-3319-01A-02R-1325-07	stage i	t1b	male		
TCGA-BQ-7053-01A-11R-1965-07	stage iii	t3	female		
TCGA-DZ-6133-01A-11R-1965-07	stage i	t1a	female		
TCGA-6D-AA2E-01A-11R-A37O-07	stage i	t1b	female		
TCGA-5P-A9KC-01A-11R-A42S-07	NA	t1b	female		

TCGA-KN-8419-01A-11R-2315-07	stage ii	t2	male		
TCGA-KM-8441-01A-11R-2315-07	stage i	t1b	female		
TCGA-UZ-A9PP-01A-11R-A42S-07	stage i	t1a	male		
TCGA-B0-4824-01A-01R-1277-07	stage i	t1a	female		
TCGA-P4-AAVL-01A-11R-A42S-07	stage iii	t3b	male		
TCGA-GL-A59R-01A-11R-A26U-07	stage iii	t3c	male		
TCGA-AK-3455-01A-01R-0864-07	stage iii	t3b	female		
TCGA-BQ-7046-01A-11R-1965-07	stage i	t1a	male		
TCGA-UZ-A9PM-01A-21R-A38C-07	stage ii	t2	male		
TCGA-AL-3472-01A-01R-1193-07	NA	t2	male		
TCGA-G7-6796-01A-11R-1965-07	stage i	t1a	male		
TCGA-Y8-A8S1-01A-11R-A37K-07	stage i	t1a	male		
TCGA-A4-8630-01A-11R-2404-07	stage i	t1b	female		
TCGA-HE-A5NI-01A-11R-A26U-07	stage i	t1a	male		
TCGA-UZ-A9PK-01A-11R-A38C-07	stage i	t1	male		
TCGA-A4-8517-01A-11R-2404-07	stage i	t1a	male		
TCGA-B0-4847-01A-01R-1277-07	stage iv	t3a	male		
TCGA-WN-A9G9-01A-12R-A37K-07	stage i	t1b	male		
TCGA-BQ-5881-01A-11R-1592-07	stage i	t1a	male		
TCGA-UN-AAZ9-01A-11R-A38C-07	stage i	t1b	female		
TCGA-G7-6790-01A-11R-1965-07	stage i	t1a	male		
TCGA-A4-7584-01A-11R-2139-07	stage i	t1a	male		
TCGA-SX-A71V-01A-11R-A33Z-07	stage i	t1	male		
TCGA-SX-A71W-01A-12R-A355-07	stage i	t1b	male		
TCGA-DW-5561-01A-01R-1592-07	stage i	t1a	male		
TCGA-BQ-7050-01A-11R-1965-07	stage i	t1a	female		
TCGA-IA-A83V-01A-11R-A355-07	stage i	t1b	male		
TCGA-BQ-7056-01A-11R-1965-07	stage iii	t3b	female		
TCGA-B0-5692-01A-11R-1541-07	stage iii	t3b	female		

TCGA-KO-8403-01A-11R-2315-07	stage i	t1a	male			
TCGA-UZ-A9Q0-01A-12R-A42S-07	stage i	t1	male			
TCGA-2Z-A9JM-01A-12R-A42S-07	stage i	t1a	male			
TCGA-4A-A93W-01A-11R-A37K-07	stage i	t1a	female			
TCGA-KM-8442-01A-11R-2315-07	stage ii	t2	male			
TCGA-AL-A5DJ-01A-11R-A26U-07	stage iii	t3a	female			
TCGA-B9-7268-01A-11R-A32Z-07	stage ii	t2	male			
TCGA-KN-8434-01A-11R-2315-07	stage ii	t2	female			
TCGA-Y8-A8RZ-01A-11R-A37K-07	stage i	t1	male			
TCGA-5P-A9K3-01A-11R-A42S-07	NA	t2	male			
TCGA-Y8-A895-01A-11R-A36F-07	stage i	t1b	male			
TCGA-MH-A855-01A-11R-A355-07	stage ii	t2b	female			
TCGA-GL-A4EM-01A-11R-A24Z-07	stage i	t1b	male			
TCGA-WN-AB4C-01A-11R-A42S-07	stage i	t1a	female			
TCGA-B3-A6W5-01A-12R-A33Z-07	stage i	t1	male			
TCGA-AL-3473-01A-01R-1193-07	stage ii	t2	male			
TCGA-A3-A8OV-01A-11R-A37O-07	stage i	t1a	male			
TCGA-KO-8410-01A-11R-2315-07	stage i	t1b	female			
TCGA-AK-3433-01A-02R-1277-07	stage ii	t2	female			
TCGA-SX-A7SL-01A-11R-A355-07	stage i	t1a	male			
TCGA-Q2-A5QZ-01A-11R-A28H-07	stage iii	t3a	female			
TCGA-IA-A40U-01A-11R-A24Z-07	stage iii	t3b	male			
TCGA-5P-A9K0-01A-11R-A42S-07	NA	t1a	male			
TCGA-A4-7287-01A-11R-2139-07	stage i	t1	female			
TCGA-P4-A5EB-01A-11R-A28H-07	stage i	t1b	male			
TCGA-BP-5174-01A-01R-1426-07	stage i	t1a	female			
TCGA-B8-4151-01A-01R-1188-07	stage iii	t3a	female			
TCGA-BP-5185-01A-01R-1426-07	stage i	t1a	male			
TCGA-A4-A57E-01A-11R-A26U-07	stage iv	t2a	male			

TCGA-SX-A7SN-01A-11R-A355-07	stage i	t1b	male		
TCGA-5P-A9KE-01A-11R-A42S-07	NA	t1b	male		
TCGA-B9-4115-01A-01R-1193-07	stage i	t1a	male		
TCGA-G6-A5PC-01A-11R-A33J-07	stage iv	t1b	female		
TCGA-B9-A5W8-01A-11R-A28H-07	stage ii	t2b	male		
TCGA-A4-A5DU-01A-11R-A28H-07	stage i	t1a	female		
TCGA-2Z-A9JQ-01A-11R-A42S-07	stage i	t1a	male		
TCGA-P4-A5E6-01A-11R-A28H-07	stage iii	t1b	male		
TCGA-F9-A97G-01A-11R-A38C-07	stage iii	t3	male		
TCGA-A3-3363-01A-01R-0864-07	stage ii	t2	male		
TCGA-5P-A9JW-01A-11R-A42S-07	NA	t1a	male		
TCGA-G7-6795-01A-11R-1965-07	stage i	t1a	male		
TCGA-AK-3443-01A-02R-1325-07	stage ii	t2	male		
TCGA-MH-A561-01A-11R-A26U-07	stage i	t1a	male		
TCGA-A4-7583-01A-11R-A32Z-07	stage i	t1	male		
TCGA-AK-3429-01A-02R-1325-07	stage ii	t2	female		
TCGA-F9-A7Q0-01A-11R-A36F-07	stage i	t1b	female		
TCGA-5P-A9K4-01A-11R-A42S-07	NA	t1b	male		
TCGA-DW-7840-01A-11R-A32Z-07	stage i	t1b	male		
TCGA-F9-A7VF-01A-11R-A33Z-07	stage i	t1a	female		
TCGA-B2-3923-01A-02R-1325-07	stage ii	t2	male		
TCGA-KN-8424-01A-11R-2315-07	stage i	t1b	female		
TCGA-AT-A5NU-01A-11R-A28H-07	stage i	t1a	male		
TCGA-5P-A9JY-01A-11R-A42S-07	NA	t2	male		
TCGA-A3-3383-01A-02R-1325-07	stage i	t1	male		
TCGA-B9-4114-01A-01R-1193-07	NA	t2	male		
TCGA-AK-3465-01A-02R-1325-07	stage i	t1b	female		
TCGA-2Z-A9JN-01A-21R-A42S-07	stage i	t1a	female		
TCGA-SX-A7SQ-01A-12R-A36F-07	stage i	t1	male		

TCGA-IA-A40X-01A-11R-A24Z-07	stage i	t1a	female		
TCGA-B9-A5W9-01A-11R-A28H-07	stage i	t1b	male		
TCGA-DW-7836-01A-11R-2139-07	stage i	t1a	male		
TCGA-A4-A5XZ-01A-11R-A31O-07	stage ii	t2a	female		
TCGA-2Z-A9JG-01A-11R-A42S-07	stage i	t1a	male		
TCGA-J7-A8I2-01A-12R-A36F-07	stage i	t1b	male		
TCGA-5P-A9K8-01A-11R-A42S-07	NA	t3a	female		
TCGA-SX-A7SO-01A-11R-A355-07	stage i	t1b	male		
TCGA-G7-A8LB-01A-11R-A36F-07	stage iv	t2a	male		
TCGA-BQ-7048-01A-11R-1965-07	stage iii	t3a	male		
TCGA-UZ-A9PL-01A-11R-A38C-07	stage ii	t2	male		
TCGA-GL-A59T-01A-21R-A28H-07	stage i	t1b	male		
TCGA-A4-A6HP-01A-11R-A31O-07	stage i	t1a	male		
TCGA-DV-A4VX-01A-11R-A266-07	stage iv	t3b	male		
TCGA-KV-A6GE-01A-11R-A31O-07	stage i	t1a	male		
TCGA-KM-8438-01A-11R-2315-07	stage ii	t2	female		
TCGA-IA-A83S-01A-11R-A355-07	NA	tx	male		
TCGA-GL-A9DE-01A-11R-A37K-07	stage i	t1b	male		
TCGA-HE-A5NF-01A-11R-A26U-07	stage i	t1a	male		
TCGA-A4-8310-01A-11R-2404-07	stage iii	t3a	male		
TCGA-UZ-A9PO-01A-11R-A38C-07	stage i	t1a	male		
TCGA-MH-A854-01A-11R-A355-07	stage i	t1b	female		
TCGA-2Z-A9J1-01A-11R-A38C-07	stage i	t1a	male		
TCGA-2Z-A9J3-01A-12R-A38C-07	stage ii	t2	male		
TCGA-KM-8439-01A-11R-2315-07	stage i	t1b	male		
TCGA-KL-8332-01A-11R-2315-07	stage i	t1b	male		
TCGA-2Z-A9J5-01A-21R-A38C-07	stage ii	t2	male		
TCGA-CJ-4638-01A-02R-1325-07	stage iv	t3a	female		
TCGA-Y8-A898-01A-11R-A355-07	stage i	t1a	male		

TCGA-SX-A7SS-01A-11R-A36F-07	stage i	t1	male		
TCGA-KO-8413-01A-11R-2315-07	stage i	t1	male		
TCGA-G7-A8LC-01A-11R-A36F-07	stage i	t1a	male		
TCGA-KN-8437-01A-11R-2315-07	stage i	t1a	female		
TCGA-2Z-A9JO-01A-11R-A42S-07	stage i	t1b	female		
TCGA-A4-A48D-01A-11R-A24Z-07	stage i	t1b	male		
TCGA-KN-8426-01A-11R-2315-07	stage iv	t3a	male		
TCGA-P4-AAVO-01A-11R-A42S-07	stage i	t1b	male		
TCGA-BQ-5880-01A-11R-1592-07	stage iii	t3a	male		
TCGA-MH-A562-01A-11R-A26U-07	stage i	t1a	male		
TCGA-B0-5107-01A-01R-1420-07	stage iv	t2	female		
TCGA-IZ-8195-01A-31R-2404-07	stage ii	t2a	male		
TCGA-A4-8312-01A-11R-2404-07	stage i	t1	male		
TCGA-KO-8406-01A-11R-2315-07	stage i	t1	female		
TCGA-B0-4696-01A-01R-1277-07	stage iii	t3a	male		
TCGA-KN-8429-01A-11R-2315-07	stage iii	t3a	female		
TCGA-KN-8435-01A-11R-2315-07	stage ii	t2	male		
TCGA-MH-A55Z-01A-11R-A26U-07	stage i	t1b	male		
TCGA-KM-8477-01A-11R-2315-07	stage i	t1a	male		
TCGA-UZ-A9Q1-01A-11R-A42S-07	stage i	t1b	female		
TCGA-MH-A55W-01A-11R-A26U-07	stage i	t1b	male		
TCGA-B1-A47M-01A-11R-A24Z-07	stage iii	t3a	male		
TCGA-KL-8327-01A-11R-2315-07	stage i	t1b	female		
TCGA-B1-7332-01A-11R-A32Z-07	stage ii	t2a	female		
TCGA-4A-A93X-01A-11R-A37K-07	stage iv	t3a	male		
TCGA-2Z-A9J7-01A-11R-A38C-07	stage iv	t2	male		
TCGA-B1-A654-01A-11R-A31O-07	stage i	t1a	female		
TCGA-BQ-5886-01A-11R-1592-07	stage iii	t3a	male		
TCGA-BP-4334-01A-01R-1289-07	stage iii	t3a	male		

TCGA-KO-8407-01A-11R-2315-07	stage ii	t2	male		
TCGA-B0-5117-01A-01R-1420-07	stage i	t1b	male		
TCGA-P4-AAVK-01A-11R-A42S-07	stage iii	t3a	male		
TCGA-B1-A655-01A-11R-A31O-07	stage i	t1a	female		
TCGA-KL-8340-01A-11R-2315-07	stage ii	t2	male		
TCGA-2Z-A9JE-01A-11R-A42S-07	stage i	t1a	male		
TCGA-B9-A8YI-01A-21R-A37K-07	stage i	t1b	male		
TCGA-5P-A9KH-01A-11R-A42S-07	NA	t1a	male		
TCGA-B9-A44B-01A-11R-A24Z-07	stage iii	t3b	male		
TCGA-B2-4099-01A-02R-1188-07	stage i	t1a	male		
TCGA-BQ-7058-01A-11R-1965-07	stage iii	t3	male		
TCGA-KN-8428-01A-11R-2315-07	stage ii	t2	male		
TCGA-PJ-A5Z9-01A-11R-A28H-07	stage i	t1b	female		
TCGA-KV-A74V-01A-11R-A33Z-07	stage i	t1a	male		
TCGA-KL-8336-01A-11R-2315-07	stage iv	t3b	female		
TCGA-KL-8323-01A-21R-2315-07	stage iii	t3b	female		
TCGA-2Z-A9JP-01A-11R-A42S-07	stage i	t1a	male		
TCGA-5P-A9K6-01A-11R-A42S-07	NA	t1a	male		
TCGA-2Z-A9JT-01A-11R-A42S-07	stage i	t1a	male		
TCGA-KO-8416-01A-11R-2315-07	stage iii	t3a	male		
TCGA-P4-AAVM-01A-11R-A42S-07	stage i	t1a	male		
TCGA-KL-8325-01A-11R-2315-07	stage ii	t2	female		
TCGA-MH-A857-01A-11R-A355-07	stage i	t1a	male		
TCGA-KN-8436-01A-11R-2315-07	stage ii	t2	male		
TCGA-IZ-A6M8-01A-11R-A31O-07	stage i	t1a	male		
TCGA-2Z-A9J2-01A-11R-A38C-07	stage i	t1a	female		
TCGA-B8-4154-01A-01R-1188-07	stage i	t1a	female		
TCGA-A4-8518-01A-11R-2404-07	stage i	t1a	male		
TCGA-KN-8425-01A-11R-2315-07	stage i	t1b	male		

TCGA-B8-A54E-01A-11R-A266-07	stage i	t1b	female		
TCGA-KM-8443-01A-11R-2315-07	stage ii	t2	male		
TCGA-KL-8326-01A-11R-2315-07	stage iii	t3a	male		
TCGA-KO-8417-01A-11R-2315-07	stage i	t1	female		
TCGA-KL-8345-01A-11R-2315-07	stage iii	t3a	male		
TCGA-KL-8329-01A-11R-2315-07	stage i	t1b	female		
TCGA-AS-3777-01A-01R-0864-07	stage i	t1a	male		
TCGA-AK-3453-01A-02R-1277-07	stage ii	t2	female		
TCGA-KL-8341-01A-11R-2315-07	stage iv	t3b	male		
TCGA-KL-8342-01A-11R-2315-07	stage ii	t2b	female		
TCGA-KM-8639-01A-11R-2403-07	stage i	t1a	male		
TCGA-KL-8337-01A-11R-2315-07	stage ii	t2	male		
TCGA-AK-3458-01A-01R-1503-07	stage i	t1b	male		
TCGA-A4-7286-01A-11R-A32Z-07	NA	t3a	male		
TCGA-KL-8343-01A-11R-2315-07	stage iii	t3	male		
TCGA-MH-A560-01A-11R-A26U-07	stage i	t1a	male		
TCGA-KL-8334-01A-11R-2315-07	stage iii	t3a	female		
TCGA-KN-8431-01A-11R-2315-07	stage ii	t2	female		
TCGA-KN-8418-01A-11R-2315-07	stage ii	t2	female		
TCGA-KL-8338-01A-11R-2315-07	stage iii	t3a	male		
TCGA-GL-8500-01A-11R-2404-07	stage i	t1b	male		
TCGA-KO-8408-01A-11R-2315-07	stage iii	t3a	male		
TCGA-KL-8324-01A-11R-2315-07	stage ii	t2	female		
TCGA-KL-8335-01A-11R-2315-07	stage iii	t3a	male		
TCGA-KM-8440-01A-11R-2315-07	stage iii	t3a	male		
TCGA-KL-8331-01A-11R-2315-07	stage ii	t2	female		
TCGA-A3-3328-01A-01R-0864-07	stage i	t1b	male		
TCGA-KL-8330-01A-11R-2315-07	stage ii	t2b	female		
TCGA-KO-8415-01A-11R-2315-07	stage i	t1	female		

TCGA-KO-8414-01A-11R-2315-07	stage ii	t2	female		
TCGA-KL-8346-01A-11R-2315-07	stage ii	t2b	male		
TCGA-KM-8476-01A-11R-2315-07	stage i	t1	male		
TCGA-KN-8433-01A-11R-2315-07	stage iii	t3a	female		
TCGA-KO-8409-01A-11R-2315-07	stage ii	t2b	male		
TCGA-KO-8411-01A-11R-2315-07	stage i	t1b	male		
TCGA-KN-8421-01A-11R-2315-07	stage ii	t2	female		
TCGA-AK-3451-01A-02R-1188-07	stage ii	t2	male		
TCGA-KN-8422-01A-11R-2315-07	stage i	t1a	female		
TCGA-AK-3427-01A-01R-0864-07	stage i	t1a	male		
TCGA-AK-3447-01A-01R-1766-07	stage ii	t2	male		
TCGA-KN-8427-01A-11R-2315-07	stage iv	t4	male	ASAP1	>75 percentile
TCGA-BP-4983-01A-01R-1334-07	stage iii	t3a	female		
TCGA-BP-4974-01A-01R-1334-07	stage iv	t3a	male		
TCGA-B0-4688-01A-01R-1277-07	stage iv	t4	male		
TCGA-B8-A8YJ-01A-13R-A39I-07	stage i	t1b	female		
TCGA-B0-4698-01A-01R-1503-07	stage iv	t4	male		
TCGA-A3-3376-01A-02R-1420-07	stage i	t1a	male		
TCGA-BP-4961-01A-01R-1334-07	stage i	t1a	male		
TCGA-CJ-4899-01A-01R-1334-07	stage i	t1b	male		
TCGA-CZ-5989-01A-11R-1672-07	stage ii	t2	male		
TCGA-DV-5574-01A-01R-1541-07	stage i	t1a	male		
TCGA-B2-5636-01A-02R-1541-07	stage i	t1a	male		
TCGA-BP-4789-01A-01R-1305-07	stage i	t1a	male		
TCGA-BP-4353-01A-02R-1289-07	stage i	t1	male		
TCGA-B0-5690-01A-11R-1541-07	stage i	t1b	female		
TCGA-BP-4999-01A-01R-1334-07	stage i	t1a	male		
TCGA-BP-4991-01A-01R-1334-07	stage i	t1a	male		
TCGA-CJ-4918-01A-01R-1426-07	stage iv	t3a	male		

TCGA-CW-6088-01A-11R-1672-07	stage i	t1b	male		
TCGA-BP-5187-01A-01R-1426-07	stage i	t1a	male		
TCGA-BP-4169-01A-02R-1289-07	stage ii	t2	female		
TCGA-B8-5165-01A-01R-1420-07	stage i	t1a	male		
TCGA-BP-4347-01A-01R-1289-07	stage iii	t3b	male		
TCGA-A3-3349-01A-01R-1188-07	stage i	t1b	female		
TCGA-CJ-4908-01A-01R-1426-07	stage i	t1a	male		
TCGA-B0-5710-01A-11R-1672-07	stage i	t1b	male		
TCGA-B0-5110-01A-01R-1420-07	stage i	t1a	female		
TCGA-BP-4354-01A-02R-1289-07	stage iv	t4	male		
TCGA-CJ-4901-01A-01R-1426-07	stage iii	t3b	male		
TCGA-CW-6093-01A-11R-1672-07	stage i	t1a	male		
TCGA-B4-5832-01A-11R-1672-07	stage iii	t3b	male		
TCGA-A3-3308-01A-02R-1325-07	stage iii	t3b	female		
TCGA-B0-5097-01A-01R-1420-07	stage iii	t3b	female		
TCGA-B8-5553-01A-01R-1541-07	stage i	t1b	female		
TCGA-BP-4344-01A-01R-1289-07	stage i	t1a	female		
TCGA-B8-5163-01A-01R-1420-07	stage iii	t3a	female		
TCGA-A3-3316-01A-01R-0864-07	stage ii	t2	male		
TCGA-B0-5693-01A-11R-1541-07	stage i	t1b	female		
TCGA-BP-4965-01A-01R-1334-07	stage i	t1a	male		
TCGA-B0-4699-01A-01R-1277-07	stage iv	t4	male		
TCGA-B4-5378-01A-01R-1503-07	stage i	t1	male		
TCGA-BP-4982-01A-01R-1334-07	stage i	t1b	male		
TCGA-CJ-4905-01A-02R-1426-07	stage i	t1a	female		
TCGA-B4-5834-01A-11R-1672-07	stage i	t1	male		
TCGA-B0-5098-01A-01R-1420-07	stage i	t1	female		
TCGA-CW-6097-01A-11R-1672-07	stage iii	t3a	male		
TCGA-EU-5906-01A-11R-1672-07	stage i	t1b	male		

TCGA-BP-4970-01A-01R-1334-07	stage iii	t1a	male		
TCGA-BP-4988-01A-01R-1334-07	stage i	t1a	male		
TCGA-B0-4703-01A-01R-1277-07	stage iv	t3a	male		
TCGA-B0-5095-01A-01R-1420-07	stage iii	t3a	male		
TCGA-B4-5844-01A-11R-1672-07	stage ii	t2	female		
TCGA-BP-4986-01A-01R-1334-07	stage i	t1a	male		
TCGA-B2-5633-01A-01R-1541-07	stage i	t1b	male		
TCGA-A3-A8CQ-01A-11R-A37O-07	stage i	t1a	female		
TCGA-AK-3461-01A-02R-1277-07	stage i	t1a	male		
TCGA-CJ-4890-01A-01R-1305-07	stage iv	t3a	male		
TCGA-BP-4342-01A-01R-1289-07	stage ii	t2	male		
TCGA-BP-4345-01A-01R-1289-07	stage iii	t3b	male		
TCGA-BP-5008-01A-01R-1334-07	stage i	t1a	male		
TCGA-A3-3323-01A-02R-1325-07	stage i	t1b	male		
TCGA-B2-A4SR-01A-11R-A266-07	stage ii	t2a	male		
TCGA-BP-5189-01A-02R-1426-07	stage i	t1b	male		
TCGA-CZ-5455-01A-01R-1503-07	stage iv	t3b	male		
TCGA-F9-A4JJ-01A-11R-A24Z-07	stage iii	t3a	female		
TCGA-A3-3378-01A-02R-1325-07	stage i	t1	male		
TCGA-CJ-5671-01A-11R-1541-07	stage i	t1a	male		
TCGA-BP-4993-01A-02R-1420-07	stage i	t1a	male		
TCGA-BP-4998-01A-01R-1334-07	stage i	t1a	male		
TCGA-BP-4971-01A-01R-1334-07	stage iii	t3a	male		
TCGA-B8-A54J-01A-11R-A33J-07	stage ii	t2a	male		
TCGA-A3-3317-01A-02R-1325-07	stage ii	t2	male		
TCGA-A3-A8OW-01A-11R-A37O-07	stage iii	t3a	male		
TCGA-BP-4330-01A-01R-1289-07	stage iii	t3a	female		
TCGA-BP-4795-01A-02R-1420-07	stage i	t1a	female		
TCGA-BP-5200-01A-01R-1426-07	stage ii	t2	male		

TCGA-GK-A6C7-01A-11R-A33J-07	stage i	t1a	female		
TCGA-BP-4326-01A-01R-1289-07	stage i	t1b	female		
TCGA-BP-4355-01A-01R-1289-07	stage iii	t3a	female		
TCGA-BP-4763-01A-01R-1289-07	stage i	t1a	female		
TCGA-B0-5812-01A-11R-1672-07	stage i	t1b	male		
TCGA-CJ-4902-01A-01R-1426-07	stage iii	t3a	male		
TCGA-B2-4101-01A-02R-1277-07	stage ii	t2a	male		
TCGA-CJ-4904-01A-02R-1426-07	stage iv	t3a	female		
TCGA-CJ-4634-01A-02R-1325-07	stage i	t1b	female		
TCGA-DV-5565-01A-01R-1541-07	stage i	t1a	male		
TCGA-A3-3365-01A-01R-0864-07	stage i	t1a	male		
TCGA-CJ-4923-01A-01R-1426-07	stage iv	t3a	female		
TCGA-BP-5195-01A-02R-1426-07	stage i	t1a	male		
TCGA-B0-5120-01A-01R-1420-07	stage i	t1a	female		
TCGA-B2-4102-01A-02R-1325-07	stage i	t1b	male		
TCGA-BP-4972-01A-01R-1334-07	stage iii	t3a	female		
TCGA-BP-4341-01A-01R-1289-07	stage iii	t3a	male		
TCGA-BP-4801-01A-02R-1420-07	stage i	t1a	male		
TCGA-CJ-4872-01A-01R-1305-07	stage i	t1b	male		
TCGA-BP-4798-01A-01R-1305-07	NA	t3b	male		
TCGA-CJ-4643-01A-02R-1325-07	stage ii	t2b	female		
TCGA-BP-4987-01A-01R-1334-07	stage i	t1b	female		
TCGA-B8-5158-01A-01R-1420-07	stage iii	t3a	male		
TCGA-BP-5184-01A-01R-1426-07	stage i	t1a	male		
TCGA-B0-5115-01A-01R-1420-07	stage iv	t2	male		
TCGA-EU-5904-01A-11R-1672-07	stage i	t1	female		
TCGA-B4-5836-01A-11R-1672-07	stage i	t1b	female		
TCGA-B0-5081-01A-01R-1334-07	stage iii	t3b	female		
TCGA-GL-7966-01A-11R-2204-07	stage iii	t3	female		

TCGA-B0-5121-01A-02R-1420-07	stage i	t1b	male		
TCGA-B8-5162-01A-01R-1420-07	stage ii	t2a	male		
TCGA-BP-4803-01A-01R-1305-07	stage iii	t3a	male		
TCGA-AK-3454-01A-02R-1277-07	stage i	t1b	male		
TCGA-B0-5402-01A-01R-1503-07	stage iv	t4	male		
TCGA-CW-5583-01A-02R-1541-07	stage i	t1a	female		
TCGA-BP-4770-01A-01R-1503-07	stage iv	t4	female		
TCGA-B0-4845-01A-01R-1277-07	stage iv	t3a	male		
TCGA-B0-5108-01A-01R-1420-07	stage iii	t3a	male		
TCGA-AL-3466-01A-02R-1351-07	stage iv	t3b	male		
TCGA-BP-4332-01A-01R-1289-07	stage iii	t3a	male		
TCGA-CZ-5982-01A-11R-1672-07	stage i	t1a	female		
TCGA-BP-4799-01A-01R-1305-07	stage iii	t3b	male		
TCGA-CZ-5988-01A-11R-1672-07	stage i	t1b	male		
TCGA-CJ-5689-01A-11R-1541-07	stage i	t1b	male		
TCGA-BP-5202-01A-02R-1426-07	stage iii	t3a	male		
TCGA-BP-4766-01A-01R-1289-07	stage i	t1a	female		
TCGA-A3-3380-01A-01R-0864-07	stage i	t1	male		
TCGA-BP-5182-01A-01R-1426-07	stage i	t1a	male		
TCGA-CJ-4868-01A-01R-1305-07	stage iv	t3a	male		
TCGA-BP-4349-01A-01R-1289-07	stage i	t1a	female		
TCGA-BP-4352-01A-01R-1289-07	stage iv	t3b	female		
TCGA-CW-6087-01A-11R-1672-07	stage iv	t3a	male		
TCGA-DV-5575-01A-01R-1541-07	stage i	t1a	female		
TCGA-A3-3372-01A-02R-1325-07	stage iii	t3	male		
TCGA-CJ-4891-01A-01R-1305-07	stage iii	t3c	female		
TCGA-B8-4143-01A-01R-1188-07	stage iv	t3a	female		
TCGA-CJ-4912-01A-01R-1426-07	stage ii	t2	male		
TCGA-A3-3320-01A-02R-1325-07	stage i	t1b	female		

TCGA-BP-4765-01A-01R-1289-07	stage i	t1a	male		
TCGA-B0-5711-01A-11R-1672-07	stage iii	t3b	male		
TCGA-BP-5177-01A-01R-1426-07	stage i	t1a	female		
TCGA-MW-A4EC-01A-11R-A266-07	stage i	t1a	female		
TCGA-BP-4782-01A-02R-1420-07	stage i	t1a	female		
TCGA-B4-5843-01A-11R-1672-07	stage i	t1	male		
TCGA-A3-A6NL-01A-11R-A33J-07	stage i	t1b	female		
TCGA-A3-3370-01A-02R-1420-07	stage i	t1b	female		
TCGA-BP-4963-01A-01R-1334-07	stage i	t1b	male		
TCGA-BP-4774-01A-01R-1289-07	stage i	t1a	female		
TCGA-A3-3311-01A-02R-1325-07	stage i	t1	male		
TCGA-B8-5551-01A-01R-1541-07	stage i	t1b	female		
TCGA-BP-5194-01A-02R-1426-07	stage i	t1a	male		
TCGA-B0-5701-01A-11R-1541-07	stage iii	t3b	male		
TCGA-CJ-4888-01A-01R-1305-07	stage iv	t3a	male		
TCGA-B0-5119-01A-02R-1420-07	stage i	t1b	female		
TCGA-CW-5580-01A-01R-1672-07	stage iv	t3a	female		
TCGA-4A-A93Y-01A-11R-A37K-07	stage ii	t2b	female		
TCGA-CJ-4907-01A-01R-1426-07	stage iii	t3b	male		
TCGA-CZ-5451-01A-01R-1503-07	stage ii	t2	male		
TCGA-CZ-4856-01A-02R-1426-07	stage i	t1b	female		
TCGA-AK-3445-01A-02R-1277-07	stage iii	t3a	male		
TCGA-A3-3351-01A-02R-1325-07	stage ii	t2a	male		
TCGA-B8-5549-01A-01R-1541-07	stage i	t1b	male		
TCGA-AK-3434-01A-02R-1277-07	stage i	t1b	male		
TCGA-BP-4976-01A-01R-1334-07	stage i	t1a	male		
TCGA-CJ-4882-01A-02R-1426-07	stage iii	t3a	male		
TCGA-CZ-4860-01A-01R-1305-07	stage iv	t4	male		
TCGA-CJ-4886-01A-01R-1305-07	stage i	t1a	female		

TCGA-B0-5709-01A-11R-1541-07	stage iii	t3a	female		
TCGA-B0-5691-01A-11R-1541-07	stage i	t1a	female		
TCGA-BP-4790-01A-01R-1305-07	stage i	t1a	male		
TCGA-AK-3431-01A-02R-1277-07	stage ii	t2	female		
TCGA-BP-4162-01A-02R-1325-07	stage i	t1b	female		
TCGA-BP-4771-01A-01R-1289-07	stage iv	t3a	male		
TCGA-B0-4815-01A-01R-1503-07	stage iii	t3a	male		
TCGA-B8-4148-01A-02R-1325-07	stage i	t1a	female		
TCGA-A3-3347-01A-02R-1325-07	stage iii	t1b	female		
TCGA-BP-4173-01A-02R-1289-07	stage ii	t2	male		
TCGA-B8-5545-01A-01R-1672-07	stage i	t1a	male		
TCGA-B8-A7U6-01A-12R-A37O-07	stage i	t1a	female		
TCGA-BP-4769-01A-01R-1289-07	stage i	t1a	male		
TCGA-BP-4158-01A-02R-1289-07	stage i	t1b	male		
TCGA-B0-5080-01A-01R-1503-07	stage iv	t3a	male		
TCGA-B8-A54F-01A-11R-A266-07	stage i	t1a	female		
TCGA-A4-7828-01A-11R-2139-07	stage i	t1a	female		
TCGA-BP-5004-01A-01R-1334-07	stage i	t1a	male		
TCGA-BP-5186-01A-01R-1426-07	stage i	t1a	female		
TCGA-B8-A54H-01A-11R-A33J-07	stage ii	t2a	female		
TCGA-B4-5838-01A-11R-1672-07	stage iv	t3	male		
TCGA-B2-4098-01A-02R-1325-07	stage i	t1b	female		
TCGA-B0-4843-01A-01R-1277-07	stage iii	t3a	male		
TCGA-CZ-5466-01A-01R-1503-07	stage iii	t3a	male		
TCGA-CJ-5686-01A-11R-1672-07	stage i	t1b	female		
TCGA-CJ-4889-01A-01R-1305-07	stage i	t1a	female		
TCGA-A3-3362-01A-02R-1325-07	stage i	t1a	female		
TCGA-B0-4813-01A-01R-1277-07	stage iii	t3b	male		
TCGA-BP-4760-01A-02R-1420-07	stage i	t1a	male		

TCGA-B8-5550-01A-01R-1541-07	stage iii	t3a	male		
TCGA-BP-4776-01A-01R-1289-07	stage i	t1a	male		
TCGA-CJ-4639-01A-02R-1325-07	stage ii	t2	female		
TCGA-CW-5587-01A-01R-1541-07	stage iii	t3b	female		
TCGA-BP-4170-01A-02R-1289-07	stage i	t1b	female		
TCGA-CJ-4893-01A-01R-1305-07	stage i	t1b	female		
TCGA-B0-4846-01A-01R-1277-07	stage iv	t3a	male		
TCGA-CJ-4920-01A-01R-1426-07	stage i	t1b	female		
TCGA-BP-4975-01A-01R-1334-07	stage i	t1b	male		
TCGA-BP-5198-01A-01R-1426-07	stage iii	t3b	male		
TCGA-BP-5006-01A-01R-1334-07	stage i	t1a	male		
TCGA-CJ-4894-01A-01R-1305-07	stage iii	t3a	male		
TCGA-DV-5567-01A-01R-1541-07	stage i	t1a	female		
TCGA-A3-3326-01A-01R-0864-07	stage i	t1a	male		
TCGA-BP-5009-01A-01R-1334-07	stage i	t1b	male		
TCGA-CJ-4878-01A-01R-1305-07	stage iii	t3a	female		
TCGA-A3-3313-01A-02R-1325-07	stage i	t1b	male		
TCGA-CZ-4862-01A-01R-1305-07	stage i	t1b	male		
TCGA-B0-4823-01A-02R-1420-07	stage i	t1a	male		
TCGA-A3-3331-01A-02R-1325-07	stage i	t1	female		
TCGA-B0-4945-01A-01R-1420-07	stage i	t1a	female		
TCGA-CJ-4897-01A-03R-1426-07	stage iii	t3a	female		
TCGA-B8-5159-01A-01R-1420-07	stage i	t1a	female		
TCGA-BP-5007-01A-01R-1334-07	stage ii	t2	male		
TCGA-BP-5199-01A-01R-1426-07	stage ii	t2	male		
TCGA-B0-5077-01A-01R-1334-07	stage i	t1a	male		
TCGA-KL-8333-01A-11R-2315-07	stage ii	t2	male		
TCGA-B0-4710-01A-01R-1503-07	stage iii	t3a	female		
TCGA-B9-4113-01A-01R-1193-07	stage i	t1	male		<25 Percentile

TCGA-A4-A7UZ-01A-12R-A355-07	stage iii	t3a	male		
TCGA-A4-A5Y1-01A-11R-A28H-07	stage iii	t1b	male		
TCGA-MH-A856-01A-11R-A355-07	stage i	t1a	male		
TCGA-AL-3471-01A-02R-1351-07	stage i	t1b	male		
TCGA-AK-3433-01A-02R-1277-07	stage ii	t2	female		
TCGA-A4-A48D-01A-11R-A24Z-07	stage i	t1b	male		
TCGA-2Z-A9JM-01A-12R-A42S-07	stage i	t1a	male		
TCGA-B3-4103-01A-02R-1351-07	stage i	t1a	male		
TCGA-KM-8442-01A-11R-2315-07	stage ii	t2	male		
TCGA-BQ-7062-01A-11R-1965-07	stage i	t1a	male		
TCGA-G7-A8LE-01A-11R-A36F-07	stage i	t1a	male		
TCGA-A4-8310-01A-11R-2404-07	stage iii	t3a	male		
TCGA-A3-3383-01A-02R-1325-07	stage i	t1	male		
TCGA-B9-A5W9-01A-11R-A28H-07	stage i	t1b	male		
TCGA-KN-8425-01A-11R-2315-07	stage i	t1b	male		
TCGA-BQ-5888-01A-11R-1592-07	stage i	t1a	female		
TCGA-F9-A7Q0-01A-11R-A36F-07	stage i	t1b	female		
TCGA-AL-3473-01A-01R-1193-07	stage ii	t2	male		
TCGA-B1-A656-01A-11R-A31O-07	stage i	t1a	male		
TCGA-UN-AAZ9-01A-11R-A38C-07	stage i	t1b	female		
TCGA-CJ-4876-01A-01R-1305-07	stage ii	t2b	male		
TCGA-KN-8419-01A-11R-2315-07	stage ii	t2	male		
TCGA-HE-A5NJ-01A-11R-A26U-07	NA	t1b	male		
TCGA-DW-7841-01A-11R-A32Z-07	stage i	t1b	male		
TCGA-CJ-5678-01A-11R-1541-07	stage iv	t2b	male		
TCGA-2Z-A9J8-01A-11R-A42S-07	stage i	t1b	male		
TCGA-5P-A9KE-01A-11R-A42S-07	NA	t1b	male		
TCGA-A4-7584-01A-11R-2139-07	stage i	t1a	male		
TCGA-MH-A55W-01A-11R-A26U-07	stage i	t1b	male		

TCGA-UZ-A9Q1-01A-11R-A42S-07	stage i	t1b	female		
TCGA-AK-3453-01A-02R-1277-07	stage ii	t2	female		
TCGA-MH-A855-01A-11R-A355-07	stage ii	t2b	female		
TCGA-KL-8336-01A-11R-2315-07	stage iv	t3b	female		
TCGA-P4-AAVK-01A-11R-A42S-07	stage iii	t3a	male		
TCGA-5P-A9K0-01A-11R-A42S-07	NA	t1a	male		
TCGA-DZ-6133-01A-11R-1965-07	stage i	t1a	female		
TCGA-IA-A40U-01A-11R-A24Z-07	stage iii	t3b	male		
TCGA-BQ-7050-01A-11R-1965-07	stage i	t1a	female		
TCGA-HE-A5NI-01A-11R-A26U-07	stage i	t1a	male		
TCGA-BQ-5891-01A-11R-1592-07	stage iii	t3b	female		
TCGA-KN-8435-01A-11R-2315-07	stage ii	t2	male		
TCGA-GL-8500-01A-11R-2404-07	stage i	t1b	male		
TCGA-KO-8406-01A-11R-2315-07	stage i	t1	female		
TCGA-B9-A44B-01A-11R-A24Z-07	stage iii	t3b	male		
TCGA-KL-8340-01A-11R-2315-07	stage ii	t2	male		
TCGA-GL-6846-01A-11R-1965-07	stage iv	t4	male		
TCGA-UZ-A9PM-01A-21R-A38C-07	stage ii	t2	male		
TCGA-KL-8331-01A-11R-2315-07	stage ii	t2	female		
TCGA-A3-3328-01A-01R-0864-07	stage i	t1b	male		
TCGA-SX-A7SO-01A-11R-A355-07	stage i	t1b	male		
TCGA-DZ-6135-01A-11R-1965-07	stage i	t1a	male		
TCGA-SX-A7SM-01A-11R-A355-07	stage iv	t3a	male		
TCGA-GL-A9DC-01A-11R-A37K-07	stage i	t1b	female		
TCGA-WN-A9G9-01A-12R-A37K-07	stage i	t1b	male		
TCGA-KL-8345-01A-11R-2315-07	stage iii	t3a	male		
TCGA-AK-3443-01A-02R-1325-07	stage ii	t2	male		
TCGA-KL-8325-01A-11R-2315-07	stage ii	t2	female		
TCGA-4A-A93X-01A-11R-A37K-07	stage iv	t3a	male		

TCGA-5P-A9JZ-01A-11R-A42S-07	NA	t1b	female		
TCGA-DW-5560-01A-01R-1592-07	stage i	t1b	female		
TCGA-A4-8312-01A-11R-2404-07	stage i	t1	male		
TCGA-B1-A657-01A-11R-A31O-07	stage i	t1b	male		
TCGA-KL-8339-01A-11R-2315-07	#N/A	#N/A	#N/A		
TCGA-KL-8329-01A-11R-2315-07	stage i	t1b	female		
TCGA-A4-7288-01A-11R-A32Z-07	NA	t1a	female		
TCGA-B0-5707-01A-11R-1541-07	stage i	t1a	female		
TCGA-Y8-A898-01A-11R-A355-07	stage i	t1a	male		
TCGA-5P-A9K6-01A-11R-A42S-07	NA	t1a	male		
TCGA-SX-A7SN-01A-11R-A355-07	stage i	t1b	male		
TCGA-KL-8338-01A-11R-2315-07	stage iii	t3a	male		
TCGA-KL-8341-01A-11R-2315-07	stage iv	t3b	male		
TCGA-Y8-A897-01A-11R-A36F-07	stage i	t1a	female		
TCGA-AL-3468-01A-02R-1351-07	stage ii	t2	male		
TCGA-B3-A6W5-01A-12R-A33Z-07	stage i	t1	male		
TCGA-IA-A83S-01A-11R-A355-07	NA	tx	male		
TCGA-KN-8429-01A-11R-2315-07	stage iii	t3a	female		
TCGA-B9-A8YI-01A-21R-A37K-07	stage i	t1b	male		
TCGA-CJ-4638-01A-02R-1325-07	stage iv	t3a	female		
TCGA-G7-A4TM-01A-11R-A31O-07	stage i	t1a	male		
TCGA-Y8-A8RY-01A-11R-A37K-07	stage i	t1b	male		
TCGA-J7-6720-01A-11R-2139-07	stage i	t1	male		
TCGA-SX-A7SQ-01A-12R-A36F-07	stage i	t1	male		
TCGA-GL-A59R-01A-11R-A26U-07	stage iii	t3c	male		
TCGA-P4-AAVL-01A-11R-A42S-07	stage iii	t3b	male		
TCGA-5P-A9K8-01A-11R-A42S-07	NA	t3a	female		
TCGA-2Z-A9JL-01A-11R-A42S-07	stage i	t1a	male		
TCGA-KL-8324-01A-11R-2315-07	stage ii	t2	female		

TCGA-HE-A5NH-01A-11R-A26U-07	stage i	t1a	male		
TCGA-KO-8415-01A-11R-2315-07	stage i	t1	female		
TCGA-AL-7173-01A-11R-2139-07	stage iv	t3	female		
TCGA-DW-7840-01A-11R-A32Z-07	stage i	t1b	male		
TCGA-KM-8477-01A-11R-2315-07	stage i	t1a	male		
TCGA-G7-6790-01A-11R-1965-07	stage i	t1a	male		
TCGA-KN-8422-01A-11R-2315-07	stage i	t1a	female		
TCGA-AS-3777-01A-01R-0864-07	stage i	t1a	male		
TCGA-2Z-A9JS-01A-21R-A42S-07	stage i	t1a	male		
TCGA-GL-A59T-01A-21R-A28H-07	stage i	t1b	male		
TCGA-AK-3451-01A-02R-1188-07	stage ii	t2	male		
TCGA-KN-8421-01A-11R-2315-07	stage ii	t2	female		
TCGA-KN-8433-01A-11R-2315-07	stage iii	t3a	female		
TCGA-G6-A5PC-01A-11R-A33J-07	stage iv	t1b	female		
TCGA-KO-8411-01A-11R-2315-07	stage i	t1b	male		
TCGA-5P-A9K4-01A-11R-A42S-07	NA	t1b	male		
TCGA-KL-8346-01A-11R-2315-07	stage ii	t2b	male		
TCGA-DW-5561-01A-01R-1592-07	stage i	t1a	male		
TCGA-KM-8439-01A-11R-2315-07	stage i	t1b	male		
TCGA-A4-7732-01A-11R-2139-07	NA	t2a	male		
TCGA-DW-7834-01A-11R-2139-07	stage i	t1	male		
TCGA-G7-A8LC-01A-11R-A36F-07	stage i	t1a	male		
TCGA-UZ-A9PX-01A-11R-A42S-07	stage i	t1	male		
TCGA-UZ-A9PJ-01A-11R-A38C-07	stage i	t1a	male		
TCGA-A4-8311-01A-11R-2404-07	stage i	t1a	male		
TCGA-B9-A5W7-01A-11R-A31O-07	stage i	t1a	male		
TCGA-KN-8418-01A-11R-2315-07	stage ii	t2	female		
TCGA-SX-A71W-01A-12R-A355-07	stage i	t1b	male		
TCGA-2Z-A9JD-01A-11R-A42S-07	stage i	t1a	male		

TCGA-MH-A857-01A-11R-A355-07	stage i	t1a	male		
TCGA-2Z-A9J5-01A-21R-A38C-07	stage ii	t2	male		
TCGA-A4-A5DU-01A-11R-A28H-07	stage i	t1a	female		
TCGA-AK-3427-01A-01R-0864-07	stage i	t1a	male		
TCGA-KL-8330-01A-11R-2315-07	stage ii	t2b	female		
TCGA-UZ-A9PP-01A-11R-A42S-07	stage i	t1a	male		
TCGA-KL-8327-01A-11R-2315-07	stage i	t1b	female		
TCGA-A3-3363-01A-01R-0864-07	stage ii	t2	male		
TCGA-UZ-A9Q0-01A-12R-A42S-07	stage i	t1	male		
TCGA-Y8-A895-01A-11R-A36F-07	stage i	t1b	male		
TCGA-SX-A7SU-01A-11R-A36F-07	stage i	t1	male		
TCGA-KL-8335-01A-11R-2315-07	stage iii	t3a	male		
TCGA-BQ-7045-01A-31R-1965-07	stage i	t1b	male		
TCGA-2Z-A9JR-01A-12R-A42S-07	stage i	t1a	male		
TCGA-5P-A9JW-01A-11R-A42S-07	NA	t1a	male		
TCGA-O9-A75Z-01A-11R-A33Z-07	stage i	t1a	male		
TCGA-G7-6796-01A-11R-1965-07	stage i	t1a	male		
TCGA-2Z-A9J6-01A-11R-A38C-07	stage i	t1a	male		
TCGA-DW-7837-01A-11R-2139-07	stage i	t1b	male		
TCGA-B1-7332-01A-11R-A32Z-07	stage ii	t2a	female		
TCGA-P4-A5EB-01A-11R-A28H-07	stage i	t1b	male		
TCGA-2Z-A9JP-01A-11R-A42S-07	stage i	t1a	male		
TCGA-HE-7129-01A-11R-1965-07	stage ii	t2	male		
TCGA-SX-A71S-01A-11R-A33Z-07	stage i	t1	female		
TCGA-MH-A560-01A-11R-A26U-07	stage i	t1a	male		
TCGA-AK-3440-01A-02R-1277-07	stage i	t1a	male		
TCGA-KM-8639-01A-11R-2403-07	stage i	t1a	male		
TCGA-UZ-A9PV-01A-11R-A42S-07	stage i	t1	male		
TCGA-SX-A7SS-01A-11R-A36F-07	stage i	t1	male		

TCGA-DW-7842-01A-11R-A32Z-07	stage i	t1a	male			
TCGA-KO-8409-01A-11R-2315-07	stage ii	t2b	male			
TCGA-KN-8432-01A-11R-2315-07	stage ii	t2b	female			
TCGA-4A-A93W-01A-11R-A37K-07	stage i	t1a	female			
TCGA-BQ-5881-01A-11R-1592-07	stage i	t1a	male			
TCGA-KM-8440-01A-11R-2315-07	stage iii	t3a	male			
TCGA-A4-A772-01A-11R-A33Z-07	stage i	t1b	male			
TCGA-2Z-A9J2-01A-11R-A38C-07	stage i	t1a	female			
TCGA-UZ-A9PU-01A-11R-A42S-07	stage i	t1b	male			
TCGA-B9-4115-01A-01R-1193-07	stage i	t1a	male			
TCGA-P4-AAVO-01A-11R-A42S-07	stage i	t1b	male			
TCGA-EV-5902-01A-11R-1592-07	stage i	t1	male			
TCGA-KO-8417-01A-11R-2315-07	stage i	t1	female			
TCGA-MH-A561-01A-11R-A26U-07	stage i	t1a	male			
TCGA-IZ-A6M8-01A-11R-A31O-07	stage i	t1a	male			
TCGA-G7-6795-01A-11R-1965-07	stage i	t1a	male			
TCGA-GL-A9DE-01A-11R-A37K-07	stage i	t1b	male			
TCGA-2Z-A9J1-01A-11R-A38C-07	stage i	t1a	male			
TCGA-B9-7268-01A-11R-A32Z-07	stage ii	t2	male			
TCGA-B9-5155-01A-01R-1592-07	stage iii	t3a	male			
TCGA-KO-8416-01A-11R-2315-07	stage iii	t3a	male			
TCGA-B1-A47M-01A-11R-A24Z-07	stage iii	t3a	male			
TCGA-BQ-5890-01A-11R-1592-07	stage iii	t3a	male			
TCGA-DW-7836-01A-11R-2139-07	stage i	t1a	male			
TCGA-AT-A5NU-01A-11R-A28H-07	stage i	t1a	male			
TCGA-KL-8326-01A-11R-2315-07	stage iii	t3a	male			
TCGA-Q2-A5QZ-01A-11R-A28H-07	stage iii	t3a	female			
TCGA-KM-8443-01A-11R-2315-07	stage ii	t2	male			
TCGA-MH-A55Z-01A-11R-A26U-07	stage i	t1b	male			

TCGA-DW-7839-01A-11R-2139-07	stage i	t1b	female		
TCGA-B9-4114-01A-01R-1193-07	NA	t2	male		
TCGA-SX-A71R-01A-12R-A33Z-07	stage i	t1a	male		
TCGA-BQ-7058-01A-11R-1965-07	stage iii	t3	male		
TCGA-BQ-5886-01A-11R-1592-07	stage iii	t3a	male		
TCGA-UZ-A9PR-01A-11R-A42S-07	stage i	t1b	male		
TCGA-KM-8476-01A-11R-2315-07	stage i	t1	male		
TCGA-5P-A9KF-01A-11R-A42S-07	NA	t1a	male		
TCGA-UZ-A9PZ-01A-11R-A42S-07	stage iii	t3a	male		
TCGA-Y8-A8S0-01A-11R-A37K-07	stage i	t1a	male		
TCGA-UZ-A9PK-01A-11R-A38C-07	stage i	t1	male		
TCGA-P4-AAVM-01A-11R-A42S-07	stage i	t1a	male		
TCGA-UZ-A9PL-01A-11R-A38C-07	stage ii	t2	male		
TCGA-2Z-A9JT-01A-11R-A42S-07	stage i	t1a	male		
TCGA-5P-A9K2-01A-11R-A42S-07	NA	t1b	male		
TCGA-BP-4334-01A-01R-1289-07	stage iii	t3a	male		
TCGA-Y8-A894-01A-11R-A36F-07	stage i	t1a	female		
TCGA-2Z-A9J7-01A-11R-A38C-07	stage iv	t2	male		
TCGA-2Z-A9J3-01A-12R-A38C-07	stage ii	t2	male		
TCGA-G7-A8LB-01A-11R-A36F-07	stage iv	t2a	male		
TCGA-B9-4617-01A-01R-1193-07	NA	t1a	male		
TCGA-SX-A7SL-01A-11R-A355-07	stage i	t1a	male		
TCGA-KV-A74V-01A-11R-A33Z-07	stage i	t1a	male		
TCGA-KV-A6GE-01A-11R-A31O-07	stage i	t1a	male		
TCGA-J7-A8I2-01A-12R-A36F-07	stage i	t1b	male		
TCGA-A4-8516-01A-11R-2404-07	stage iii	t3a	male		
TCGA-P4-A5E6-01A-11R-A28H-07	stage iii	t1b	male		
TCGA-AL-3472-01A-01R-1193-07	NA	t2	male		
TCGA-KO-8414-01A-11R-2315-07	stage ii	t2	female		

TCGA-PJ-A5Z9-01A-11R-A28H-07	stage i	t1b	female		
TCGA-B1-A47N-01A-11R-A24Z-07	stage i	t1a	male		
TCGA-A4-8515-01A-11R-2404-07	stage i	t1a	male		
TCGA-KL-8337-01A-11R-2315-07	stage ii	t2	male		
TCGA-2Z-A9JE-01A-11R-A42S-07	stage i	t1a	male		
TCGA-AK-3447-01A-01R-1766-07	stage ii	t2	male		
TCGA-MH-A854-01A-11R-A355-07	stage i	t1b	female		
TCGA-MH-A562-01A-11R-A26U-07	stage i	t1a	male		
TCGA-IA-A40X-01A-11R-A24Z-07	stage i	t1a	female		
TCGA-A4-A6HP-01A-11R-A31O-07	stage i	t1a	male		
TCGA-IZ-A6M9-01A-11R-A31O-07	stage i	t1a	male		
TCGA-B0-5702-01A-11R-1541-07	stage i	t1b	male		
TCGA-5P-A9JY-01A-11R-A42S-07	NA	t2	male		
TCGA-2Z-A9JQ-01A-11R-A42S-07	stage i	t1a	male		
TCGA-B1-A654-01A-11R-A31O-07	stage i	t1a	female		
TCGA-F9-A7VF-01A-11R-A33Z-07	stage i	t1a	female		
TCGA-GK-A6C7-01A-11R-A33J-07	stage iii	t3a	female	FOXC2	>75 percentile
TCGA-B8-4620-01A-02R-1325-07	stage ii	t2	male		
TCGA-BP-5200-01A-01R-1426-07	stage i	t1b	female		
TCGA-B8-A8YJ-01A-13R-A39I-07	stage ii	t2b	male		
TCGA-CJ-4876-01A-01R-1305-07	stage i	t1a	male		
TCGA-BP-5006-01A-01R-1334-07	stage i	t1a	male		
TCGA-BP-5187-01A-01R-1426-07	stage i	t1b	male		
TCGA-CJ-4899-01A-01R-1334-07	stage i	t1	female		
TCGA-CZ-4859-01A-02R-1426-07	stage ii	t2	female		
TCGA-CJ-6032-01A-11R-1672-07	stage ii	t2a	male		
TCGA-B8-A54J-01A-11R-A33J-07	stage ii	t2	male		
TCGA-BP-4342-01A-01R-1289-07	stage i	t1a	female		
TCGA-A3-A6NJ-01A-12R-A33J-07	stage i	t1a	male		

TCGA-BP-5168-01A-01R-1420-07	stage iv	t3a	female		
TCGA-CJ-4904-01A-02R-1426-07	stage i	t1a	male		
TCGA-BP-5192-01A-01R-1426-07	stage i	t1a	male		
TCGA-BP-4999-01A-01R-1334-07	stage i	t1b	female		
TCGA-CJ-4920-01A-01R-1426-07	stage i	t1a	male		
TCGA-BP-4993-01A-02R-1420-07	stage i	t1a	male		
TCGA-CW-6093-01A-11R-1672-07	stage i	t1a	female		
TCGA-MW-A4EC-01A-11R-A266-07	stage i	t1b	female		
TCGA-A3-A6NL-01A-11R-A33J-07	stage i	t1a	male		
TCGA-BP-4769-01A-01R-1289-07	stage i	t1a	male		
TCGA-BP-5008-01A-01R-1334-07	stage iii	t3a	female		
TCGA-BP-4355-01A-01R-1289-07	stage i	t1b	female		
TCGA-G6-A8L7-01A-11R-A37O-07	stage iv	t3a	female		
TCGA-B0-4701-01A-01R-1277-07	stage i	t1a	male		
TCGA-B0-4823-01A-02R-1420-07	stage ii	t2b	female		
TCGA-KN-8432-01A-11R-2315-07	stage iii	t3a	female		
TCGA-B0-4710-01A-01R-1503-07	stage iv	t3a	male		
TCGA-CZ-4857-01A-01R-1305-07	stage iii	t3a	female		
TCGA-B0-5113-01A-01R-1420-07	stage ii	t2	female		
TCGA-AK-3429-01A-02R-1325-07	stage i	t1a	male		
TCGA-CJ-5671-01A-11R-1541-07	stage iii	t3b	male		
TCGA-BP-4345-01A-01R-1289-07	stage iii	t3a	male		
TCGA-B0-5108-01A-01R-1420-07	stage iii	t3b	male		
TCGA-A3-3307-01A-01R-0864-07	stage i	t1b	female		
TCGA-A3-3373-01A-02R-1420-07	stage i	t1b	male		
TCGA-CZ-5984-01A-11R-1672-07	stage iv	t1b	male		
TCGA-CZ-5462-01A-01R-1503-07	stage i	t1b	male		
TCGA-AK-3454-01A-02R-1277-07	stage iii	t3a	male		
TCGA-BP-4971-01A-01R-1334-07	stage i	t1b	female		

TCGA-CZ-4856-01A-02R-1426-07	stage iii	t3b	male		
TCGA-BP-4799-01A-01R-1305-07	stage i	t1b	male		
TCGA-B0-5703-01A-11R-1541-07	stage i	t1	female		
TCGA-EU-5904-01A-11R-1672-07	stage iii	t3a	male		
TCGA-B0-5095-01A-01R-1420-07	stage i	t1	female		
TCGA-EU-5905-01A-11R-1672-07	stage i	t1a	female		
TCGA-BP-5177-01A-01R-1426-07	stage iii	t3a	male		
TCGA-B0-4849-01A-01R-1277-07	stage ii	t2b	female		
TCGA-CJ-4643-01A-02R-1325-07	stage iii	t3a	male		
TCGA-CJ-4882-01A-02R-1426-07	stage i	t1a	male		
TCGA-AK-3461-01A-02R-1277-07	stage i	t1a	female		
TCGA-BP-4784-01A-01R-1305-07	stage i	t1a	male		
TCGA-BP-4986-01A-01R-1334-07	stage i	t1a	female		
TCGA-BP-4349-01A-01R-1289-07	stage i	t1a	female		
TCGA-A4-7828-01A-11R-2139-07	stage i	t1a	male		
TCGA-3Z-A93Z-01A-11R-A37O-07	stage i	t1b	female		
TCGA-BP-4340-01A-01R-1289-07	stage ii	t2	female		
TCGA-AK-3431-01A-02R-1277-07	stage iii	t3b	male		
TCGA-CJ-4901-01A-01R-1426-07	stage iii	t3a	male		
TCGA-B0-4706-01A-01R-1503-07	stage iv	t3a	male		
TCGA-CW-5590-01A-01R-1541-07	stage iii	t3a	male		
TCGA-BP-4160-01A-02R-1289-07	stage i	t1b	female		
TCGA-CJ-4874-01A-01R-1305-07	stage iii	t3a	female		
TCGA-BP-4351-01A-01R-1289-07	stage iv	t3a	male		
TCGA-B0-4703-01A-01R-1277-07	stage iv	t3b	male		
TCGA-BP-5201-01A-01R-1426-07	stage i	t1a	male		
TCGA-BP-4776-01A-01R-1289-07	stage i	t1a	female		
TCGA-B8-A7U6-01A-12R-A37O-07	stage i	t1a	female		
TCGA-B8-4148-01A-02R-1325-07	stage iii	t3b	female		

TCGA-B0-5099-01A-01R-1420-07	stage i	t1a	male		
TCGA-CJ-4908-01A-01R-1426-07	stage i	t1a	female		
TCGA-BP-4795-01A-02R-1420-07	stage i	t1a	male		
TCGA-BP-4789-01A-01R-1305-07	stage iii	t3a	male		
TCGA-B0-4810-01A-01R-1503-07	stage i	t1	male		
TCGA-B4-5834-01A-11R-1672-07	stage i	t1a	female		
TCGA-DV-5575-01A-01R-1541-07	stage i	t1a	male		
TCGA-BP-5195-01A-02R-1426-07	stage i	t1a	female		
TCGA-DV-5566-01A-01R-1541-07	stage i	t1a	male		
TCGA-BP-4998-01A-01R-1334-07	stage iii	t3b	male		
TCGA-CW-5584-01A-01R-1541-07	stage iii	t3b	female		
TCGA-B0-5713-01A-11R-1672-07	stage i	t1	male		
TCGA-B4-5378-01A-01R-1503-07	stage iii	t3b	female		
TCGA-B0-5081-01A-01R-1334-07	stage i	t1a	female		
TCGA-BP-4964-01A-01R-1334-07	stage ii	t2	female		
TCGA-BP-4169-01A-02R-1289-07	stage i	t1b	male		
TCGA-B0-5710-01A-11R-1672-07	stage iii	t3b	male		
TCGA-BP-4347-01A-01R-1289-07	stage iii	t3a	male		
TCGA-B0-5100-01A-01R-1420-07	stage ii	t2	male		
TCGA-CZ-5456-01A-01R-1503-07	stage i	t1a	male		
TCGA-BP-4781-01A-01R-1305-07	stage i	t1b	male		
TCGA-A3-3324-01A-02R-1325-07	stage iv	t3a	female		
TCGA-CJ-6033-01A-11R-1672-07	stage i	t1a	female		
TCGA-A3-A8OU-01A-11R-A37O-07	stage iii	t3a	female		
TCGA-B8-5163-01A-01R-1420-07	stage iii	t3a	female		
TCGA-CJ-4870-01A-01R-1305-07	stage iv	t3a	male		
TCGA-B0-4846-01A-01R-1277-07	stage i	t1a	male		
TCGA-A3-3376-01A-02R-1420-07	stage i	t1a	male		
TCGA-BP-4965-01A-01R-1334-07	stage i	t1a	male		

TCGA-BP-5004-01A-01R-1334-07	stage i	t1a	male		
TCGA-BP-4331-01A-01R-1289-07	stage i	t1b	male		
TCGA-A3-3382-01A-02R-1325-07	stage ii	t2a	male		
TCGA-B2-4101-01A-02R-1277-07	stage iii	t3a	male		
TCGA-BP-4166-01A-02R-1289-07	stage i	t1a	male		
TCGA-BP-4988-01A-01R-1334-07	stage i	t1b	male		
TCGA-B2-5633-01A-01R-1541-07	stage i	t1a	male		
TCGA-B0-5106-01A-01R-1420-07	stage iii	t3a	male		
TCGA-AK-3445-01A-02R-1277-07	stage iv	t3a	male		
TCGA-B0-5080-01A-01R-1503-07	stage iv	t3a	male		
TCGA-CJ-4890-01A-01R-1305-07	stage iv	t3a	male		
TCGA-B8-4622-01A-02R-1277-07	stage iii	t3a	male		
TCGA-CJ-4894-01A-01R-1305-07	stage i	t1a	female		
TCGA-CJ-4886-01A-01R-1305-07	stage i	t1a	female		
TCGA-BP-4344-01A-01R-1289-07	stage i	t1a	male		
TCGA-CW-5589-01A-01R-1541-07	stage i	t1	male		
TCGA-A3-3380-01A-01R-0864-07	stage i	t1a	female		
TCGA-DV-5569-01A-01R-1541-07	stage iv	t3a	female		
TCGA-BP-4335-01A-01R-1289-07	stage i	t1a	male		
TCGA-BP-4762-01A-02R-1289-07	stage i	t1a	male		
TCGA-DV-5565-01A-01R-1541-07	stage i	t1a	male		
TCGA-AK-3460-01A-02R-1277-07	stage i	t1b	male		
TCGA-BP-4176-01A-02R-1289-07	stage i	t1a	male		
TCGA-BP-4765-01A-01R-1289-07	stage i	t1b	male		
TCGA-BP-4804-01A-02R-1305-07	stage iv	t3	female		
TCGA-B4-5377-01A-01R-1503-07	stage i	t1b	female		
TCGA-BP-4170-01A-02R-1289-07	stage i	t1a	male		
TCGA-A3-3387-01A-01R-1541-07	stage i	t1a	male		
TCGA-BP-4759-01A-01R-1289-07	stage iv	t3a	female		

TCGA-CW-5580-01A-01R-1672-07	stage iv	t3b	male		
TCGA-B0-4836-01A-01R-1305-07	stage iv	t2	male		
TCGA-B0-4828-01A-01R-1277-07	stage i	t1a	female		
TCGA-B0-4945-01A-01R-1420-07	stage iii	t3a	female		
TCGA-CJ-4878-01A-01R-1305-07	stage i	t1	male		
TCGA-BP-4353-01A-02R-1289-07	stage iii	t3a	male		
TCGA-A3-A8OW-01A-11R-A37O-07	stage i	t1a	female		
TCGA-A3-3358-01A-01R-1541-07	stage iii	t3b	female		
TCGA-B0-5097-01A-01R-1420-07	stage iii	t3a	female		
TCGA-BP-4983-01A-01R-1334-07	stage i	t1b	male		
TCGA-CJ-5689-01A-11R-1541-07	stage i	t1	male		
TCGA-EV-5902-01A-11R-1592-07	stage iii	t3a	male		
TCGA-BP-4985-01A-01R-1334-07	stage i	t1b	male		
TCGA-CJ-4872-01A-01R-1305-07	stage iii	t3b	male		
TCGA-CJ-4907-01A-01R-1426-07	stage i	t1a	male		
TCGA-BP-4961-01A-01R-1334-07	stage i	t1b	male		
TCGA-B2-3924-01A-02R-1325-07	stage i	t1a	male		
TCGA-BP-5196-01A-01R-1426-07	stage i	t1a	male		
TCGA-BP-4801-01A-02R-1420-07	stage i	t1b	female		
TCGA-A3-3349-01A-01R-1188-07	stage i	t1a	female		
TCGA-BP-4763-01A-01R-1289-07	stage ii	t2	male		
TCGA-BP-5199-01A-01R-1426-07	stage iii	t3a	male		
TCGA-CJ-4902-01A-01R-1426-07	stage i	t1b	male		
TCGA-B0-5121-01A-02R-1420-07	stage i	t1a	female		
TCGA-BP-4774-01A-01R-1289-07	stage iv	t4	male		
TCGA-B0-4688-01A-01R-1277-07	stage iii	t3b	male		
TCGA-BP-5198-01A-01R-1426-07	stage i	t1b	male		
TCGA-BP-5189-01A-02R-1426-07	stage iv	t2a	male		
TCGA-G6-A8L6-01A-11R-A37O-07	stage iii	t3a	male		

TCGA-BP-4329-01A-02R-1289-07	stage iv	t2	male		
TCGA-B0-5115-01A-01R-1420-07	stage iv	t3a	male		
TCGA-BP-4974-01A-01R-1334-07	stage iii	t3b	male		
TCGA-B0-5711-01A-11R-1672-07	stage i	t1b	female		
TCGA-BP-4326-01A-01R-1289-07	stage iii	t3b	male		
TCGA-CJ-5679-01A-11R-1541-07	stage i	t1a	female		
TCGA-B8-A54F-01A-11R-A266-07	stage i	t1a	female		
TCGA-B0-5120-01A-01R-1420-07	stage iv	t3a	male		
TCGA-CJ-4868-01A-01R-1305-07	stage i	t1a	female		
TCGA-A3-A8CQ-01A-11R-A37O-07	stage i	t1a	male		
TCGA-BP-5182-01A-01R-1426-07	stage iii	t3a	female		
TCGA-B0-5709-01A-11R-1541-07	stage i	t1a	male		
TCGA-BP-4807-01A-01R-1305-07	stage i	t1b	female		
TCGA-BP-4165-01A-02R-1289-07	stage ii	t2	male		
TCGA-BP-4960-01A-01R-1334-07	stage i	t1b	male		
TCGA-BP-4982-01A-01R-1334-07	stage i	t1a	female		
TCGA-CJ-4905-01A-02R-1426-07	stage i	t1b	male		
TCGA-B0-5698-01A-11R-1672-07	stage iii	t1b	female		
TCGA-A3-3347-01A-02R-1325-07	stage iii	t3	female		
TCGA-GL-7966-01A-11R-2204-07	stage i	t1a	male		
TCGA-BP-5173-01A-01R-1426-07	stage i	t1b	male		
TCGA-EU-5906-01A-11R-1672-07	stage iii	t3	male		
TCGA-A3-3372-01A-02R-1325-07	stage ii	t2a	male		
TCGA-B8-5162-01A-01R-1420-07	stage iii	t3a	male		
TCGA-A3-3352-01A-01R-0864-07	stage i	t1b	male		
TCGA-BP-5169-01A-01R-1426-07	stage iv	t3a	male		
TCGA-CJ-4918-01A-01R-1426-07	stage i	t1b	male		
TCGA-CJ-6031-01A-11R-1672-07	stage iii	t3a	male		
TCGA-B0-5694-01A-11R-1541-07	stage i	t1b	male		

TCGA-BP-4159-01A-02R-1289-07	stage iii	t3a	male			
TCGA-CZ-5458-01A-01R-1503-07	stage i	t1b	female			
TCGA-B0-5690-01A-11R-1541-07	stage i	t1b	male			
TCGA-BP-4975-01A-01R-1334-07	stage ii	t2	female			
TCGA-B0-4818-01A-01R-1503-07	stage iv	t1b	male			
TCGA-CZ-5461-01A-01R-1503-07	stage iii	t1a	male			
TCGA-BP-4970-01A-01R-1334-07	stage i	t1	male			
TCGA-B4-5843-01A-11R-1672-07	stage i	t1a	male			
TCGA-A3-A6NN-01A-12R-A33J-07	stage i	t1b	male			
TCGA-B8-A54I-01A-21R-A33J-07	stage iii	t3a	male			
TCGA-BP-4167-01A-02R-1325-07	stage iii	t3a	male			
TCGA-BQ-5887-01A-11R-1965-07	stage iv	t3a	male			
TCGA-BP-5178-01A-01R-1426-07	stage iii	t3b	male			
TCGA-CJ-5676-01A-11R-1541-07	stage ii	t2	male			
TCGA-BP-4173-01A-02R-1289-07	stage ii	t2	male			
TCGA-BP-5007-01A-01R-1334-07	stage i	t1b	female			
TCGA-B0-5693-01A-11R-1541-07	stage i	t1b	male			
TCGA-BP-4158-01A-02R-1289-07	stage i	t1a	male			
TCGA-CJ-5672-01A-11R-1541-07	stage ii	t2	male			
TCGA-B0-4816-01A-01R-1503-07	stage i	t1	female			
TCGA-B0-5104-01A-01R-1420-07	stage i	t1b	male			
TCGA-BP-5009-01A-01R-1334-07	stage iii	t3a	male			
TCGA-B8-5550-01A-01R-1541-07	stage i	t1	male			
TCGA-CZ-5986-01A-11R-1672-07	stage i	t1a	male			
TCGA-A3-3322-01A-02R-1325-07	stage iv	t3a	male			
TCGA-B0-4712-01A-01R-1503-07	stage i	t1a	male			
TCGA-BP-4760-01A-02R-1420-07	stage ii	t2a	female			
TCGA-B8-A54H-01A-11R-A33J-07	stage iii	t3a	male			
TCGA-EU-5907-01A-11R-1672-07	stage iv	t3a	male			

TCGA-CJ-5682-01A-11R-1541-07	stage iii	t3a	male			
TCGA-CJ-5684-01A-11R-1541-07	stage i	t1b	female			
TCGA-B0-5119-01A-02R-1420-07	stage i	t1a	female			
TCGA-CW-5583-01A-02R-1541-07	stage iii	t3a	male			
TCGA-AK-3427-01A-01R-0864-07	stage i	t1a	male			
TCGA-B3-4103-01A-02R-1351-07	stage i	t1a	male			
TCGA-MH-A561-01A-11R-A26U-07	stage i	t1a	female			
TCGA-KN-8437-01A-11R-2315-07	stage i	t1a	male			
TCGA-AS-3777-01A-01R-0864-07	NA	t2	male			
TCGA-B9-4114-01A-01R-1193-07	stage iv	t2a	male			
TCGA-A4-A57E-01A-11R-A26U-07	stage i	t1b	male			
TCGA-WN-A9G9-01A-12R-A37K-07	stage i	t1	male			
TCGA-B3-A6W5-01A-12R-A33Z-07	stage ii	t2	male			
TCGA-A3-3357-01A-02R-1420-07	stage i	t1b	male			
TCGA-DW-7837-01A-11R-2139-07	stage i	t1	male			
TCGA-J7-6720-01A-11R-2139-07	stage ii	t2	female			
TCGA-KN-8418-01A-11R-2315-07	NA	t1b	male			
TCGA-HE-A5NJ-01A-11R-A26U-07	stage i	t1b	female			
TCGA-GL-A9DC-01A-11R-A37K-07	stage iii	t3b	female			
TCGA-BQ-7056-01A-11R-1965-07	stage i	t1a	male			
TCGA-2Z-A9JM-01A-12R-A42S-07	stage i	t1	male			
TCGA-UZ-A9PX-01A-11R-A42S-07	NA	t1a	female			
TCGA-A4-7288-01A-11R-A32Z-07	stage i	t1a	male			
TCGA-GL-A9DD-01A-11R-A37K-07	stage i	t1a	male			
TCGA-2Z-A9JL-01A-11R-A42S-07	stage i	t1a	male			
TCGA-BQ-5876-01A-11R-1592-07	stage i	t1a	male			
TCGA-2Z-A9JR-01A-12R-A42S-07	stage i	t1a	male			
TCGA-G7-6790-01A-11R-1965-07	stage ii	t2b	female			
TCGA-MH-A855-01A-11R-A355-07	stage i	t1a	male			<25 Percentile

TCGA-AT-A5NU-01A-11R-A28H-07	stage ii	t2	female		
TCGA-KO-8414-01A-11R-2315-07	stage ii	t2	male		
TCGA-UZ-A9PL-01A-11R-A38C-07	#N/A	#N/A	#N/A		
TCGA-KL-8339-01A-11R-2315-07	stage ii	t2	male		
TCGA-UZ-A9PM-01A-21R-A38C-07	stage i	t1	male		
TCGA-A4-8312-01A-11R-2404-07	stage iii	t3	female		
TCGA-HE-7130-01A-11R-1965-07	stage iii	t3a	male		
TCGA-B1-A47M-01A-11R-A24Z-07	stage iv	t3b	female		
TCGA-KL-8336-01A-11R-2315-07	stage i	t1a	male		
TCGA-UZ-A9PO-01A-11R-A38C-07	stage iv	t3b	male		
TCGA-KL-8341-01A-11R-2315-07	stage iii	t3b	male		
TCGA-B4-5832-01A-11R-1672-07	stage i	t1	male		
TCGA-HE-7128-01A-11R-1965-07	stage i	t1	female		
TCGA-B4-5835-01A-11R-1672-07	stage iii	t1b	male		
TCGA-A4-A5Y1-01A-11R-A28H-07	NA	t1a	male		
TCGA-B9-5156-01A-01R-1592-07	stage i	t1	female		
TCGA-KO-8406-01A-11R-2315-07	stage i	t1b	male		
TCGA-SX-A71W-01A-12R-A355-07	stage iii	t3	male		
TCGA-BQ-7058-01A-11R-1965-07	stage i	t1	female		
TCGA-B0-5098-01A-01R-1420-07	stage i	t1b	female		
TCGA-2Z-A9JO-01A-11R-A42S-07	stage i	t1a	male		
TCGA-GL-7773-01A-11R-A32Z-07	stage i	t1b	male		
TCGA-KO-8411-01A-11R-2315-07	stage iii	t3	male		
TCGA-BQ-5885-01A-11R-1592-07	stage i	t1b	female		
TCGA-B8-5546-01A-01R-1541-07	stage i	t1b	male		
TCGA-B1-A657-01A-11R-A31O-07	stage iii	t3a	female		
TCGA-G7-7501-01A-11R-2204-07	stage i	t1a	male		
TCGA-2Z-A9J1-01A-11R-A38C-07	stage i	t1a	male		
TCGA-B1-A656-01A-11R-A31O-07	stage iii	t3a	male		

TCGA-Y8-A896-01A-11R-A36F-07	stage iii	t3a	male		
TCGA-P4-AAVK-01A-11R-A42S-07	stage ii	t2b	female		
TCGA-KL-8330-01A-11R-2315-07	stage i	t1b	male		
TCGA-BQ-7045-01A-31R-1965-07	stage i	t1b	male		
TCGA-UZ-A9PU-01A-11R-A42S-07	stage i	t1b	female		
TCGA-SX-A7SP-01A-11R-A355-07	stage ii	t2	female		
TCGA-AK-3433-01A-02R-1277-07	stage i	t1	female		
TCGA-CZ-4866-01A-01R-1503-07	stage iii	t3b	male		
TCGA-B1-5398-01A-02R-1592-07	stage i	t1b	male		
TCGA-A4-7997-01A-11R-2204-07	NA	t2a	male		
TCGA-A4-7732-01A-11R-2139-07	stage i	t1a	male		
TCGA-HE-A5NI-01A-11R-A26U-07	stage i	t1b	male		
TCGA-A4-A772-01A-11R-A33Z-07	stage iii	t3b	female		
TCGA-BQ-5891-01A-11R-1592-07	stage ii	t2	male		
TCGA-BQ-7051-01A-12R-1965-07	stage i	t1a	male		
TCGA-A4-8098-01A-11R-2404-07	NA	t1a	male		
TCGA-5P-A9K0-01A-11R-A42S-07	stage i	t1a	male		
TCGA-B9-A5W7-01A-11R-A31O-07	stage i	t1a	male		
TCGA-UZ-A9PJ-01A-11R-A38C-07	stage i	t1b	male		
TCGA-G7-6792-01A-21R-1965-07	stage i	t1a	female		
TCGA-2Z-A9JN-01A-21R-A42S-07	stage i	t1a	female		
TCGA-Y8-A897-01A-11R-A36F-07	stage i	t1b	female		
TCGA-B1-A47O-01A-11R-A24Z-07	stage i	t1a	male		
TCGA-HE-A5NL-01A-11R-A26U-07	stage i	t1b	female		
TCGA-DW-7839-01A-11R-2139-07	stage iii	t3a	male		
TCGA-A4-8516-01A-11R-2404-07	stage i	t1b	male		
TCGA-B9-A5W9-01A-11R-A28H-07	stage i	t1b	male		
TCGA-Y8-A8RY-01A-11R-A37K-07	stage iv	t4	male		
TCGA-BP-4354-01A-02R-1289-07	stage i	t1a	female		

TCGA-Y8-A894-01A-11R-A36F-07	stage i	t1a	female		
TCGA-T7-A92I-01A-11R-A37O-07	stage iii	t3a	female		
TCGA-BQ-5875-01A-11R-1592-07	stage i	t1b	male		
TCGA-KN-8423-01A-11R-2315-07	stage i	t1b	female		
TCGA-KL-8327-01A-11R-2315-07	stage i	t1b	male		
TCGA-DW-7840-01A-11R-A32Z-07	stage iii	t3a	male		
TCGA-UZ-A9PZ-01A-11R-A42S-07	NA	t3a	male		
TCGA-A4-7286-01A-11R-A32Z-07	stage i	t1a	female		
TCGA-B1-A654-01A-11R-A31O-07	stage i	t1a	male		
TCGA-2Z-A9JS-01A-21R-A42S-07	stage i	t1b	male		
TCGA-P4-A5EB-01A-11R-A28H-07	stage iii	t3a	male		
TCGA-G7-A8LD-01A-11R-A36F-07	stage iii	t3a	male		
TCGA-BQ-7044-01A-11R-1965-07	stage i	t1b	male		
TCGA-SX-A7SN-01A-11R-A355-07	stage iv	t3a	male		
TCGA-4A-A93X-01A-11R-A37K-07	stage i	t1a	female		
TCGA-DZ-6133-01A-11R-1965-07	stage iv	t4	male		
TCGA-GL-6846-01A-11R-1965-07	NA	tx	male		
TCGA-IA-A83S-01A-11R-A355-07	stage iii	t3a	female		
TCGA-Q2-A5QZ-01A-11R-A28H-07	stage i	t1b	male		
TCGA-G7-7502-01A-11R-2204-07	stage i	t1a	male		
TCGA-A4-8517-01A-11R-2404-07	stage i	t1a	female		
TCGA-BQ-5884-01A-11R-1592-07	stage i	t1a	female		
TCGA-B1-A655-01A-11R-A31O-07	stage ii	t2b	male		
TCGA-KO-8409-01A-11R-2315-07	stage i	t1a	male		
TCGA-B9-A8YH-01A-11R-A37K-07	NA	t1b	female		
TCGA-5P-A9JZ-01A-11R-A42S-07	stage i	t1	male		
TCGA-B9-4113-01A-01R-1193-07	#N/A	#N/A	#N/A		
TCGA-KL-8328-01A-11R-2315-07	stage i	t1b	female		
TCGA-F9-A7Q0-01A-11R-A36F-07	stage i	t1a	male		

TCGA-2Z-A9J6-01A-11R-A38C-07	stage i	t1a	male			
TCGA-BQ-7060-01A-11R-1965-07	stage i	t1b	male			
TCGA-GL-A9DE-01A-11R-A37K-07	stage i	t1b	male			
TCGA-A4-A48D-01A-11R-A24Z-07	stage i	t1a	male			
TCGA-MH-A857-01A-11R-A355-07	stage ii	t2	female			
TCGA-UZ-A9PS-01A-11R-A42S-07	stage iii	t3a	male			
TCGA-BQ-5890-01A-11R-1592-07	stage i	t1b	male			
TCGA-AL-3471-01A-02R-1351-07	stage iii	t2a	male			
TCGA-P4-A5E8-01A-11R-A28H-07	stage i	t1	male			
TCGA-SX-A71V-01A-11R-A33Z-07	stage iii	t3b	female			
TCGA-KL-8323-01A-21R-2315-07	stage i	t1a	male			
TCGA-SX-A71U-01A-12R-A33Z-07	stage i	t1b	male			
TCGA-SX-A7SO-01A-11R-A355-07	stage i	t1a	female			
TCGA-A4-7996-01A-11R-2204-07	stage i	t1a	male			
TCGA-KM-8639-01A-11R-2403-07	stage ii	t2	female			
TCGA-KN-8421-01A-11R-2315-07	stage i	t1a	male			
TCGA-KV-A74V-01A-11R-A33Z-07	stage i	t1a	female			
TCGA-IA-A40X-01A-11R-A24Z-07	stage i	t1a	male			
TCGA-B1-A47N-01A-11R-A24Z-07	stage i	t1b	male			
TCGA-B8-4621-01A-01R-1503-07	stage ii	t2	male			
TCGA-2Z-A9J3-01A-12R-A38C-07	stage i	t1b	female			
TCGA-PJ-A5Z9-01A-11R-A28H-07	stage i	t1a	male			
TCGA-Y8-A8S0-01A-11R-A37K-07	stage i	t1b	male			
TCGA-B9-A8YI-01A-21R-A37K-07	stage iii	t3a	female			
TCGA-B0-4842-01A-02R-1420-07	stage ii	t2	male			
TCGA-AL-3468-01A-02R-1351-07	stage iii	t1b	male			
TCGA-P4-A5E6-01A-11R-A28H-07	stage i	t1a	male			
TCGA-DW-5561-01A-01R-1592-07	stage ii	t2	male			
TCGA-B9-7268-01A-11R-A32Z-07	NA	t2	male			

TCGA-5P-A9K3-01A-11R-A42S-07	stage i	t1a	male		
TCGA-BQ-7046-01A-11R-1965-07	stage i	t1a	male		
TCGA-Y8-A898-01A-11R-A355-07	stage i	t1b	female		
TCGA-UN-AAZ9-01A-11R-A38C-07	NA	t1b	male		
TCGA-5P-A9KE-01A-11R-A42S-07	stage i	t1b	male		
TCGA-Y8-A895-01A-11R-A36F-07	NA	t1a	male		
TCGA-5P-A9KH-01A-11R-A42S-07	stage iii	t3a	male		
TCGA-BP-4334-01A-01R-1289-07	stage iii	t3a	male		
TCGA-B0-4696-01A-01R-1277-07	stage i	t1a	male		
TCGA-MH-A560-01A-11R-A26U-07	NA	t1a	male		
TCGA-5P-A9KF-01A-11R-A42S-07	stage i	t1a	male		
TCGA-SX-A71R-01A-12R-A33Z-07	stage i	t1b	male		
TCGA-MH-A55W-01A-11R-A26U-07	stage i	t1a	female		
TCGA-A4-A5DU-01A-11R-A28H-07	stage i	t1	male		
TCGA-UZ-A9PK-01A-11R-A38C-07	stage ii	t2b	male		
TCGA-B9-A5W8-01A-11R-A28H-07	stage ii	t2a	female		
TCGA-A4-A5XZ-01A-11R-A31O-07	stage i	t1a	male		
TCGA-KV-A6GE-01A-11R-A31O-07	stage iv	t1b	female		
TCGA-G6-A5PC-01A-11R-A33J-07	stage i	t1	female		
TCGA-B3-3926-01A-02R-1351-07	stage i	t1a	female		
TCGA-BQ-7050-01A-11R-1965-07	NA	t2	male		
TCGA-AL-3472-01A-01R-1193-07	stage i	t1a	male		
TCGA-A4-8311-01A-11R-2404-07	stage iv	t2	male		
TCGA-2Z-A9J7-01A-11R-A38C-07	stage i	t1b	male		
TCGA-GL-A59T-01A-21R-A28H-07	stage i	t1b	male		
TCGA-GL-8500-01A-11R-2404-07	stage ii	t2	female		
TCGA-KL-8331-01A-11R-2315-07	stage i	t1a	male		
TCGA-IZ-A6M8-01A-11R-A31O-07	stage ii	t2	female		
TCGA-KL-8325-01A-11R-2315-07	stage ii	t2a	male		

TCGA-SX-A7SR-01A-12R-A36F-07	stage i	t1a	male		
TCGA-A4-A6HP-01A-11R-A31O-07	stage iii	t3a	female		
TCGA-BQ-5878-01A-11R-1592-07	stage iii	t3a	male		
TCGA-KM-8440-01A-11R-2315-07	stage i	t1a	female		
TCGA-KN-8422-01A-11R-2315-07	NA	t1a	male		
TCGA-B9-4617-01A-01R-1193-07	stage i	t1a	male		
TCGA-P4-AAVM-01A-11R-A42S-07	stage i	t1a	male		
TCGA-A4-7584-01A-11R-2139-07	stage i	t1b	male		
TCGA-UZ-A9PR-01A-11R-A42S-07	stage i	t1	male		
TCGA-UZ-A9PV-01A-11R-A42S-07	stage i	t1a	male		
TCGA-B9-4115-01A-01R-1193-07	stage i	t1b	female		
TCGA-A4-8630-01A-11R-2404-07	stage ii	t2	male		
TCGA-2Z-A9J5-01A-21R-A38C-07	stage i	t1b	male		
TCGA-J7-A8I2-01A-12R-A36F-07	stage i	t1	male		
TCGA-Y8-A8RZ-01A-11R-A37K-07	stage i	t1a	male		
TCGA-2Z-A9JT-01A-11R-A42S-07	stage ii	t2	male		
TCGA-KM-8443-01A-11R-2315-07	stage ii	t2a	male		
TCGA-IZ-8195-01A-31R-2404-07	stage iii	t3a	male		
TCGA-BQ-5886-01A-11R-1592-07	stage i	t1a	male		
TCGA-2Z-A9JP-01A-11R-A42S-07	stage i	t1a	male		
TCGA-A4-8515-01A-11R-2404-07	NA	t1b	male		
TCGA-5P-A9K2-01A-11R-A42S-07	stage iii	t3a	male		
TCGA-A4-8310-01A-11R-2404-07	stage i	t1b	female		
TCGA-B8-A54E-01A-11R-A266-07	stage i	t1a	male		
TCGA-G7-6795-01A-11R-1965-07	stage i	t1a	female		
TCGA-BQ-5888-01A-11R-1592-07	stage i	t1b	female		
TCGA-A4-A5Y0-01A-11R-A31O-07	stage i	t1b	male		
TCGA-2Z-A9J8-01A-11R-A42S-07	stage i	t1a	male		
TCGA-IZ-A6M9-01A-11R-A31O-07	stage i	t1a	male		

TCGA-2Z-A9JE-01A-11R-A42S-07	stage ii	t2	male		
TCGA-HE-7129-01A-11R-1965-07	stage i	t1b	male		
TCGA-P4-AAVO-01A-11R-A42S-07	stage iv	t2	female		
TCGA-B0-5107-01A-01R-1420-07	stage i	t1b	female		
TCGA-DW-5560-01A-01R-1592-07	stage iii	t3a	female		
TCGA-AL-A5DJ-01A-11R-A26U-07	NA	t2	male		
TCGA-5P-A9JY-01A-11R-A42S-07	NA	t1a	male		
TCGA-5P-A9JW-01A-11R-A42S-07	stage i	t1b	male		
TCGA-MH-A55Z-01A-11R-A26U-07	stage ii	t2	male		
TCGA-CZ-5989-01A-11R-1672-07	stage i	t1b	female		
TCGA-UZ-A9Q1-01A-11R-A42S-07	stage i	t1b	male		
TCGA-A3-3328-01A-01R-0864-07	stage ii	t2	male		
TCGA-AK-3447-01A-01R-1766-07	stage i	t1a	female		
TCGA-2Z-A9J2-01A-11R-A38C-07	stage i	t1a	male		
TCGA-2Z-A9JQ-01A-11R-A42S-07	stage i	t1a	female		
TCGA-F9-A7VF-01A-11R-A33Z-07	stage iv	t2a	male		
TCGA-G7-A8LB-01A-11R-A36F-07	stage i	t1a	male		
TCGA-HE-A5NH-01A-11R-A26U-07	stage i	t1a	male		
TCGA-MH-A562-01A-11R-A26U-07	stage i	t1a	male		
TCGA-P4-A5ED-01A-11R-A28H-07	stage i	t1	male		
TCGA-UZ-A9Q0-01A-12R-A42S-07	stage i	t1a	female		
TCGA-KN-8427-01A-11R-2315-07	stage iv	t4	male		
TCGA-B0-5096-01A-01R-1420-07	stage iii	t3a	female		
TCGA-GL-7966-01A-11R-2204-07	stage iii	t3	female		
TCGA-CW-6097-01A-11R-1672-07	stage iii	t3a	male		
TCGA-B0-5098-01A-01R-1420-07	stage i	t1	female		
TCGA-CZ-4860-01A-01R-1305-07	stage iv	t4	male		
TCGA-BQ-5891-01A-11R-1592-07	stage iii	t3b	female		
TCGA-A3-3382-01A-02R-1325-07	stage i	t1b	male	HEATR1	>75 percentile

TCGA-AL-3466-01A-02R-1351-07	stage iv	t3b	male			
TCGA-G7-A8LD-01A-11R-A36F-07	stage iii	t3a	male			
TCGA-B8-5551-01A-01R-1541-07	stage i	t1b	female			
TCGA-IA-A40Y-01A-11R-A24Z-07	stage iii	t3a	female			
TCGA-B0-5109-01A-02R-1420-07	stage iii	t3b	male			
TCGA-CZ-5982-01A-11R-1672-07	stage i	t1a	female			
TCGA-J7-8537-01A-11R-2404-07	stage iii	t3	female			
TCGA-B0-5084-01A-01R-1334-07	stage iv	t3a	male			
TCGA-BQ-5876-01A-11R-1592-07	stage i	t1a	male			
TCGA-CW-6087-01A-11R-1672-07	stage iv	t3a	male			
TCGA-A3-3313-01A-02R-1325-07	stage i	t1b	male			
TCGA-B0-4690-01A-01R-1277-07	stage iv	t4	male			
TCGA-F9-A4JJ-01A-11R-A24Z-07	stage iii	t3a	female			
TCGA-B0-5097-01A-01R-1420-07	stage iii	t3b	female			
TCGA-A4-7287-01A-11R-2139-07	stage i	t1	female			
TCGA-CZ-4857-01A-01R-1305-07	stage iv	t3a	male			
TCGA-B0-5701-01A-11R-1541-07	stage iii	t3b	male			
TCGA-A3-3316-01A-01R-0864-07	stage ii	t2	male			
TCGA-2Z-A9JI-01A-11R-A42S-07	stage iii	t3a	male			
TCGA-BQ-5879-01A-11R-1592-07	stage iii	t3b	female			
TCGA-B4-5843-01A-11R-1672-07	stage i	t1	male			
TCGA-BQ-5882-01A-11R-1592-07	stage iii	t3b	male			
TCGA-CZ-4861-01A-01R-1305-07	stage ii	t2	male			
TCGA-5P-A9K9-01A-11R-A42S-07	NA	t3a	female			
TCGA-HE-7130-01A-11R-1965-07	stage iii	t3	female			
TCGA-B0-4688-01A-01R-1277-07	stage iv	t4	male			
TCGA-CW-5587-01A-01R-1541-07	stage iii	t3b	female			
TCGA-B8-4620-01A-02R-1325-07	stage iii	t3a	female			
TCGA-BP-4161-01A-02R-1325-07	stage i	t1b	male			

TCGA-B3-4104-01A-02R-1351-07	stage ii	t2	male		
TCGA-B8-5158-01A-01R-1420-07	stage iii	t3a	male		
TCGA-CW-5580-01A-01R-1672-07	stage iv	t3a	female		
TCGA-BP-4174-01A-02R-1289-07	stage ii	t2	male		
TCGA-A4-7915-01A-11R-2204-07	stage ii	t2b	female		
TCGA-BP-4781-01A-01R-1305-07	stage i	t1a	male		
TCGA-BP-4354-01A-02R-1289-07	stage iv	t4	male		
TCGA-BP-4983-01A-01R-1334-07	stage iii	t3a	female		
TCGA-B4-5835-01A-11R-1672-07	stage i	t1	female		
TCGA-BP-4343-01A-02R-1289-07	stage iii	t3a	male		
TCGA-CJ-6032-01A-11R-1672-07	stage ii	t2	female		
TCGA-CZ-4858-01A-01R-1305-07	stage ii	t2	male		
TCGA-B0-4815-01A-01R-1503-07	stage iii	t3a	male		
TCGA-CJ-6028-01A-11R-1672-07	stage iv	t3a	male		
TCGA-CJ-6027-01A-11R-1672-07	stage i	t1a	male		
TCGA-B0-5115-01A-01R-1420-07	stage iv	t2	male		
TCGA-A3-3347-01A-02R-1325-07	stage iii	t1b	female		
TCGA-B0-4701-01A-01R-1277-07	stage iv	t3a	female		
TCGA-BP-5198-01A-01R-1426-07	stage iii	t3b	male		
TCGA-BP-4176-01A-02R-1289-07	stage i	t1b	male		
TCGA-BP-5189-01A-02R-1426-07	stage i	t1b	male		
TCGA-B0-4698-01A-01R-1503-07	stage iv	t4	male		
TCGA-CJ-4637-01A-02R-1325-07	stage iv	t2b	female		
TCGA-DV-5565-01A-01R-1541-07	stage i	t1a	male		
TCGA-CW-6093-01A-11R-1672-07	stage i	t1a	male		
TCGA-CJ-4635-01A-02R-1305-07	stage i	t1b	male		
TCGA-B0-4848-01A-01R-1277-07	stage iii	t3b	male		
TCGA-A3-3308-01A-02R-1325-07	stage iii	t3b	female		
TCGA-A3-3317-01A-02R-1325-07	stage ii	t2	male		

TCGA-B0-5116-01A-02R-1420-07	stage iii	t3b	male			
TCGA-B2-5633-01A-01R-1541-07	stage i	t1b	male			
TCGA-B0-5402-01A-01R-1503-07	stage iv	t4	male			
TCGA-BQ-5887-01A-11R-1965-07	stage iii	t3a	male			
TCGA-CZ-5463-01A-01R-1503-07	stage ii	t2	male			
TCGA-BP-4986-01A-01R-1334-07	stage i	t1a	male			
TCGA-BP-4797-01A-01R-1305-07	stage iii	t3b	male			
TCGA-DZ-6131-01A-11R-1965-07	stage iii	t3a	male			
TCGA-BQ-5893-01A-11R-1592-07	stage iv	t3a	male			
TCGA-B2-5641-01A-01R-1541-07	stage i	t1a	male			
TCGA-CZ-5465-01A-01R-1503-07	stage iii	t3b	female			
TCGA-A3-3372-01A-02R-1325-07	stage iii	t3	male			
TCGA-CZ-5986-01A-11R-1672-07	stage i	t1	male			
TCGA-CW-5591-01A-01R-1541-07	stage iv	t3a	male			
TCGA-CJ-5675-01A-11R-1541-07	stage ii	t2a	male			
TCGA-BP-5004-01A-01R-1334-07	stage i	t1a	male			
TCGA-AK-3450-01A-02R-1277-07	stage i	t1a	female			
TCGA-CZ-5455-01A-01R-1503-07	stage iv	t3b	male			
TCGA-B8-4143-01A-01R-1188-07	stage iv	t3a	female			
TCGA-G7-7501-01A-11R-2204-07	stage iii	t3a	female			
TCGA-BQ-5877-01A-11R-1592-07	stage iv	t3a	male			
TCGA-BP-4963-01A-01R-1334-07	stage i	t1b	male			
TCGA-CZ-5985-01A-11R-1672-07	stage ii	t2	male			
TCGA-B8-5164-01A-01R-1420-07	stage iii	t3a	male			
TCGA-CZ-5461-01A-01R-1503-07	stage iv	t1b	male			
TCGA-B8-5165-01A-01R-1420-07	stage i	t1a	male			
TCGA-EU-5907-01A-11R-1672-07	stage iii	t3a	male			
TCGA-CW-5590-01A-01R-1541-07	stage iv	t3a	male			
TCGA-DV-5569-01A-01R-1541-07	stage i	t1a	female			

TCGA-BP-4770-01A-01R-1503-07	stage iv	t4	female		
TCGA-BP-4163-01A-02R-1325-07	stage iii	t3a	female		
TCGA-G7-6789-01A-11R-1965-07	stage iv	t3a	female		
TCGA-BP-4985-01A-01R-1334-07	stage iii	t3a	male		
TCGA-BP-5187-01A-01R-1426-07	stage i	t1a	male		
TCGA-CZ-5984-01A-11R-1672-07	stage i	t1b	male		
TCGA-CZ-5989-01A-11R-1672-07	stage ii	t2	male		
TCGA-BP-4342-01A-01R-1289-07	stage ii	t2	male		
TCGA-CZ-5988-01A-11R-1672-07	stage i	t1b	male		
TCGA-BP-4798-01A-01R-1305-07	NA	t3b	male		
TCGA-EU-5906-01A-11R-1672-07	stage i	t1b	male		
TCGA-CZ-5454-01A-01R-1503-07	stage iv	t2	male		
TCGA-A4-A5Y1-01A-11R-A28H-07	stage iii	t1b	male		
TCGA-A3-3319-01A-02R-1325-07	stage i	t1b	male		
TCGA-CW-5581-01A-02R-1541-07	stage i	t1b	male		
TCGA-BQ-5890-01A-11R-1592-07	stage iii	t3a	male		
TCGA-A3-3367-01A-02R-1420-07	stage i	t1b	male		
TCGA-B2-5636-01A-02R-1541-07	stage i	t1a	male		
TCGA-A3-3306-01A-01R-0864-07	stage i	t1b	male		
TCGA-A3-3311-01A-02R-1325-07	stage i	t1	male		
TCGA-B4-5838-01A-11R-1672-07	stage iv	t3	male		
TCGA-CJ-5679-01A-11R-1541-07	stage iii	t3b	male		
TCGA-B0-5104-01A-01R-1420-07	stage i	t1	female		
TCGA-G7-6793-01A-11R-1965-07	stage iv	t3a	female		
TCGA-CJ-4904-01A-02R-1426-07	stage iv	t3a	female		
TCGA-B0-5709-01A-11R-1541-07	stage iii	t3a	female		
TCGA-A3-3378-01A-02R-1325-07	stage i	t1	male		
TCGA-A3-3385-01A-02R-1420-07	stage i	t1a	female		
TCGA-B0-5113-01A-01R-1420-07	stage iii	t3a	female		

TCGA-B8-5550-01A-01R-1541-07	stage iii	t3a	male		
TCGA-CW-6088-01A-11R-1672-07	stage i	t1b	male		
TCGA-CW-5583-01A-02R-1541-07	stage i	t1a	female		
TCGA-A3-3323-01A-02R-1325-07	stage i	t1b	male		
TCGA-BP-5194-01A-02R-1426-07	stage i	t1a	male		
TCGA-2Z-A9JJ-01A-11R-A42S-07	stage i	t1a	female		
TCGA-B8-5162-01A-01R-1420-07	stage ii	t2a	male		
TCGA-BP-4169-01A-02R-1289-07	stage ii	t2	female		
TCGA-BP-4803-01A-01R-1305-07	stage iii	t3a	male		
TCGA-A3-3351-01A-02R-1325-07	stage ii	t2a	male		
TCGA-CJ-4643-01A-02R-1325-07	stage ii	t2b	female		
TCGA-CZ-5456-01A-01R-1503-07	stage ii	t2	male		
TCGA-BP-4973-01A-01R-1334-07	stage iii	t3a	male		
TCGA-A3-3324-01A-02R-1325-07	stage i	t1b	male		
TCGA-BP-4974-01A-01R-1334-07	stage iv	t3a	male		
TCGA-A3-3343-01A-01R-0864-07	stage ii	t2	male		
TCGA-BP-4998-01A-01R-1334-07	stage i	t1a	male		
TCGA-B3-8121-01A-21R-2404-07	stage i	t1	female		
TCGA-B0-4827-01A-02R-1420-07	stage iii	t3b	female		
TCGA-DV-5574-01A-01R-1541-07	stage i	t1a	male		
TCGA-5P-A9K3-01A-11R-A42S-07	NA	t2	male		
TCGA-CJ-4905-01A-02R-1426-07	stage i	t1a	female		
TCGA-B8-5159-01A-01R-1420-07	stage i	t1a	female		
TCGA-A3-3352-01A-01R-0864-07	stage iii	t3a	male		
TCGA-B8-5163-01A-01R-1420-07	stage iii	t3a	female		
TCGA-2Z-A9J8-01A-11R-A42S-07	stage i	t1b	male		
TCGA-B0-4852-01A-01R-1503-07	stage ii	t2	female		
TCGA-A3-3387-01A-01R-1541-07	stage i	t1a	male		
TCGA-CJ-4923-01A-01R-1426-07	stage iv	t3a	female		

TCGA-BP-4164-01A-02R-1325-07	stage iii	t3a	female		
TCGA-B0-5108-01A-01R-1420-07	stage iii	t3a	male		
TCGA-BP-4771-01A-01R-1289-07	stage iv	t3a	male		
TCGA-BP-4961-01A-01R-1334-07	stage i	t1a	male		
TCGA-B4-5378-01A-01R-1503-07	stage i	t1	male		
TCGA-A3-3373-01A-02R-1420-07	stage i	t1b	female		
TCGA-B0-5693-01A-11R-1541-07	stage i	t1b	female		
TCGA-BQ-7051-01A-12R-1965-07	stage ii	t2	male		
TCGA-A3-3322-01A-02R-1325-07	stage i	t1a	male		
TCGA-CJ-5686-01A-11R-1672-07	stage i	t1b	female		
TCGA-CJ-4641-01A-02R-1325-07	stage iv	t3a	female		
TCGA-BP-4967-01A-01R-1334-07	stage iii	t3a	male		
TCGA-BP-5202-01A-02R-1426-07	stage iii	t3a	male		
TCGA-BP-4992-01A-01R-1334-07	stage i	t1b	male		
TCGA-B0-4836-01A-01R-1305-07	stage iv	t3b	male		
TCGA-CW-6090-01A-11R-1672-07	stage i	t1b	male		
TCGA-BP-5173-01A-01R-1426-07	stage i	t1a	male		
TCGA-B0-4712-01A-01R-1503-07	stage iv	t3a	male		
TCGA-AK-3456-01A-02R-1325-07	stage ii	t2	male		
TCGA-AK-3455-01A-01R-0864-07	stage iii	t3b	female		
TCGA-B0-5110-01A-01R-1420-07	stage i	t1a	female		
TCGA-CJ-4920-01A-01R-1426-07	stage i	t1b	female		
TCGA-B4-5836-01A-11R-1672-07	stage i	t1b	female		
TCGA-B0-5706-01A-11R-1541-07	stage ii	t2	male		
TCGA-B0-4703-01A-01R-1277-07	stage iv	t3a	male		
TCGA-B0-4838-01A-01R-1305-07	stage i	t1b	female		
TCGA-B0-5711-01A-11R-1672-07	stage iii	t3b	male		
TCGA-BP-4160-01A-02R-1289-07	stage iii	t3a	male		
TCGA-BP-4766-01A-01R-1289-07	stage i	t1a	female		

TCGA-CZ-5467-01A-01R-1503-07	stage iii	t3a	female		
TCGA-BP-5183-01A-01R-1426-07	stage iii	t3a	male		
TCGA-DV-5567-01A-01R-1541-07	stage i	t1a	female		
TCGA-CJ-4881-01A-01R-1305-07	stage iii	t3a	male		
TCGA-CZ-5459-01A-01R-1503-07	stage iii	t3b	male		
TCGA-B0-5710-01A-11R-1672-07	stage i	t1b	male		
TCGA-AK-3461-01A-02R-1277-07	stage i	t1a	male		
TCGA-BQ-7061-01A-11R-1965-07	stage i	t1b	female		
TCGA-B2-4102-01A-02R-1325-07	stage i	t1b	male		
TCGA-A3-3346-01A-01R-1766-07	stage i	t1b	male		
TCGA-BP-4353-01A-02R-1289-07	stage i	t1	male		
TCGA-BP-4965-01A-01R-1334-07	stage i	t1a	male		
TCGA-BP-4787-01A-01R-1305-07	stage iv	t3a	female		
TCGA-P4-A5E8-01A-11R-A28H-07	stage iii	t2a	male		
TCGA-CJ-4640-01A-02R-1325-07	stage iii	t3a	male		
TCGA-CJ-5680-01A-11R-1541-07	stage iv	t3a	female		
TCGA-CJ-4908-01A-01R-1426-07	stage i	t1a	male		
TCGA-B0-4814-01A-01R-1277-07	stage iv	t4	male		
TCGA-BP-4763-01A-01R-1289-07	stage i	t1a	female		
TCGA-BQ-5878-01A-11R-1592-07	stage iii	t3a	female		
TCGA-CJ-5671-01A-11R-1541-07	stage i	t1a	male		
TCGA-CZ-4862-01A-01R-1305-07	stage i	t1b	male		
TCGA-B0-4710-01A-01R-1503-07	stage iii	t3a	female		
TCGA-BP-5200-01A-01R-1426-07	stage ii	t2	male		
TCGA-A3-3320-01A-02R-1325-07	stage i	t1b	female		
TCGA-A4-7997-01A-11R-2204-07	stage i	t1b	male		
TCGA-SX-A7SM-01A-11R-A355-07	stage iv	t3a	male		
TCGA-CJ-4872-01A-01R-1305-07	stage i	t1b	male		
TCGA-BP-5169-01A-01R-1426-07	stage i	t1b	male		

TCGA-CJ-4634-01A-02R-1325-07	stage i	t1b	female		
TCGA-B8-5549-01A-01R-1541-07	stage i	t1b	male		
TCGA-B2-5639-01A-01R-1541-07	stage iv	t3	male		
TCGA-CJ-4895-01A-01R-1305-07	stage iv	t3a	male		
TCGA-BP-4789-01A-01R-1305-07	stage i	t1a	male		
TCGA-B8-5545-01A-01R-1672-07	stage i	t1a	male		
TCGA-A4-8311-01A-11R-2404-07	stage i	t1a	male		
TCGA-B8-A54H-01A-11R-A33J-07	stage ii	t2a	female		
TCGA-CJ-6030-01A-11R-1672-07	stage i	t1a	male		
TCGA-A4-8312-01A-11R-2404-07	stage i	t1	male		
TCGA-BP-4165-01A-02R-1289-07	stage i	t1b	female		
TCGA-SX-A7SS-01A-11R-A36F-07	stage i	t1	male		
TCGA-KM-8441-01A-11R-2315-07	stage i	t1b	female		
TCGA-AT-A5NU-01A-11R-A28H-07	stage i	t1a	male		
TCGA-A4-7732-01A-11R-2139-07	NA	t2a	male		
TCGA-KO-8404-01A-11R-2315-07	stage iv	t4	male		
TCGA-DV-A4VX-01A-11R-A266-07	stage iv	t3b	male		
TCGA-KN-8428-01A-11R-2315-07	stage ii	t2	male		
TCGA-KL-8325-01A-11R-2315-07	stage ii	t2	female		
TCGA-GL-A9DD-01A-11R-A37K-07	stage i	t1a	male		
TCGA-B0-4691-01A-01R-1277-07	stage iv	t2	male		
TCGA-B0-5696-01A-11R-1541-07	stage iii	t3a	male		
TCGA-BP-4758-01A-01R-1289-07	stage i	t1a	male		
TCGA-5P-A9KE-01A-11R-A42S-07	NA	t1b	male		
TCGA-A4-A5XZ-01A-11R-A31O-07	stage ii	t2a	female		
TCGA-BP-5191-01A-01R-1426-07	stage iii	t3a	male		
TCGA-SX-A71R-01A-12R-A33Z-07	stage i	t1a	male		
TCGA-P4-A5E7-01A-31R-A28H-07	stage i	t1b	female		
TCGA-CJ-5678-01A-11R-1541-07	stage iv	t2b	male		<25 Percentile

TCGA-KN-8424-01A-11R-2315-07	stage i	t1b	female			
TCGA-MW-A4EC-01A-11R-A266-07	stage i	t1a	female			
TCGA-AL-3471-01A-02R-1351-07	stage i	t1b	male			
TCGA-UZ-A9PX-01A-11R-A42S-07	stage i	t1	male			
TCGA-KL-8345-01A-11R-2315-07	stage iii	t3a	male			
TCGA-P4-AAVO-01A-11R-A42S-07	stage i	t1b	male			
TCGA-5P-A9KA-01A-11R-A42S-07	NA	t1a	male			
TCGA-UZ-A9PP-01A-11R-A42S-07	stage i	t1a	male			
TCGA-SX-A7SP-01A-11R-A355-07	stage i	t1b	female			
TCGA-UZ-A9PZ-01A-11R-A42S-07	stage iii	t3a	male			
TCGA-KL-8326-01A-11R-2315-07	stage iii	t3a	male			
TCGA-IA-A40X-01A-11R-A24Z-07	stage i	t1a	female			
TCGA-CJ-4638-01A-02R-1325-07	stage iv	t3a	female			
TCGA-B3-A6W5-01A-12R-A33Z-07	stage i	t1	male			
TCGA-SX-A71U-01A-12R-A33Z-07	stage i	t1a	male			
TCGA-G6-A5PC-01A-11R-A33J-07	stage iv	t1b	female			
TCGA-B0-5712-01A-11R-1672-07	stage iv	t2	female			
TCGA-P4-A5EB-01A-11R-A28H-07	stage i	t1b	male			
TCGA-KL-8344-01A-11R-2315-07	stage iii	t3a	male			
TCGA-UZ-A9PL-01A-11R-A38C-07	stage ii	t2	male			
TCGA-SX-A7SU-01A-11R-A36F-07	stage i	t1	male			
TCGA-DW-7839-01A-11R-2139-07	stage i	t1b	female			
TCGA-A3-3380-01A-01R-0864-07	stage i	t1	male			
TCGA-B0-4817-01A-01R-1277-07	stage iii	t3c	male			
TCGA-KM-8442-01A-11R-2315-07	stage ii	t2	male			
TCGA-Y8-A8RY-01A-11R-A37K-07	stage i	t1b	male			
TCGA-AK-3440-01A-02R-1277-07	stage i	t1a	male			
TCGA-B9-4115-01A-01R-1193-07	stage i	t1a	male			
TCGA-KO-8406-01A-11R-2315-07	stage i	t1	female			

TCGA-B0-5100-01A-01R-1420-07	stage iii	t3a	male		
TCGA-B0-5400-01A-01R-1503-07	stage iii	t3b	female		
TCGA-KL-8330-01A-11R-2315-07	stage ii	t2b	female		
TCGA-5P-A9K6-01A-11R-A42S-07	NA	t1a	male		
TCGA-5P-A9KF-01A-11R-A42S-07	NA	t1a	male		
TCGA-KO-8417-01A-11R-2315-07	stage i	t1	female		
TCGA-B0-4841-01A-01R-1277-07	stage iv	t2	male		
TCGA-AL-3473-01A-01R-1193-07	stage ii	t2	male		
TCGA-B0-5085-01A-01R-1334-07	stage iii	t3a	female		
TCGA-KL-8343-01A-11R-2315-07	stage iii	t3	male		
TCGA-B9-A5W7-01A-11R-A31O-07	stage i	t1a	male		
TCGA-IZ-8195-01A-31R-2404-07	stage ii	t2a	male		
TCGA-5P-A9JY-01A-11R-A42S-07	NA	t2	male		
TCGA-BP-4167-01A-02R-1325-07	stage iii	t3a	male		
TCGA-DW-5561-01A-01R-1592-07	stage i	t1a	male		
TCGA-AK-3433-01A-02R-1277-07	stage ii	t2	female		
TCGA-KL-8336-01A-11R-2315-07	stage iv	t3b	female		
TCGA-A4-8516-01A-11R-2404-07	stage iii	t3a	male		
TCGA-BQ-7045-01A-31R-1965-07	stage i	t1b	male		
TCGA-KN-8435-01A-11R-2315-07	stage ii	t2	male		
TCGA-MH-A857-01A-11R-A355-07	stage i	t1a	male		
TCGA-UZ-A9PM-01A-21R-A38C-07	stage ii	t2	male		
TCGA-KM-8440-01A-11R-2315-07	stage iii	t3a	male		
TCGA-A3-3374-01A-02R-1325-07	stage i	t1b	female		
TCGA-UZ-A9Q0-01A-12R-A42S-07	stage i	t1	male		
TCGA-A4-A48D-01A-11R-A24Z-07	stage i	t1b	male		
TCGA-DW-7837-01A-11R-2139-07	stage i	t1b	male		
TCGA-BQ-7062-01A-11R-1965-07	stage i	t1a	male		
TCGA-Y8-A897-01A-11R-A36F-07	stage i	t1a	female		

TCGA-B0-4839-01A-01R-1305-07	stage i	t1b	female		
TCGA-B1-A657-01A-11R-A31O-07	stage i	t1b	male		
TCGA-UN-AAZ9-01A-11R-A38C-07	stage i	t1b	female		
TCGA-AK-3453-01A-02R-1277-07	stage ii	t2	female		
TCGA-KM-8443-01A-11R-2315-07	stage ii	t2	male		
TCGA-IA-A83V-01A-11R-A355-07	stage i	t1b	male		
TCGA-KL-8329-01A-11R-2315-07	stage i	t1b	female		
TCGA-B9-4113-01A-01R-1193-07	stage i	t1	male		
TCGA-P4-A5EA-01A-11R-A28H-07	stage iii	t3a	female		
TCGA-AL-7173-01A-11R-2139-07	stage iv	t3	female		
TCGA-Q2-A5QZ-01A-11R-A28H-07	stage iii	t3a	female		
TCGA-PJ-A5Z8-01A-11R-A28H-07	stage i	t1a	female		
TCGA-KL-8331-01A-11R-2315-07	stage ii	t2	female		
TCGA-J7-6720-01A-11R-2139-07	stage i	t1	male		
TCGA-4A-A93W-01A-11R-A37K-07	stage i	t1a	female		
TCGA-2Z-A9JR-01A-12R-A42S-07	stage i	t1a	male		
TCGA-KN-8425-01A-11R-2315-07	stage i	t1b	male		
TCGA-AS-3777-01A-01R-0864-07	stage i	t1a	male		
TCGA-5P-A9K8-01A-11R-A42S-07	NA	t3a	female		
TCGA-G7-A8LB-01A-11R-A36F-07	stage iv	t2a	male		
TCGA-6D-AA2E-01A-11R-A37O-07	stage i	t1b	female		
TCGA-KM-8639-01A-11R-2403-07	stage i	t1a	male		
TCGA-BQ-5888-01A-11R-1592-07	stage i	t1a	female		
TCGA-G6-A8L6-01A-11R-A37O-07	stage iv	t2a	male		
TCGA-P4-A5E6-01A-11R-A28H-07	stage iii	t1b	male		
TCGA-B0-5700-01A-11R-1541-07	stage i	t1a	male		
TCGA-G7-6796-01A-11R-1965-07	stage i	t1a	male		
TCGA-Y8-A895-01A-11R-A36F-07	stage i	t1b	male		
TCGA-3Z-A93Z-01A-11R-A37O-07	stage i	t1a	male		

TCGA-IA-A83S-01A-11R-A355-07	NA	tx	male		
TCGA-B0-4842-01A-02R-1420-07	stage iii	t3a	female		
TCGA-BP-5185-01A-01R-1426-07	stage i	t1a	male		
TCGA-2Z-A9J6-01A-11R-A38C-07	stage i	t1a	male		
TCGA-A4-A5DU-01A-11R-A28H-07	stage i	t1a	female		
TCGA-BP-4968-01A-01R-1334-07	stage i	t1b	male		
TCGA-KN-8434-01A-11R-2315-07	stage ii	t2	female		
TCGA-BP-4329-01A-02R-1289-07	stage iii	t3a	male		
TCGA-HE-A5NI-01A-11R-A26U-07	stage i	t1a	male		
TCGA-G7-A4TM-01A-11R-A31O-07	stage i	t1a	male		
TCGA-B1-A655-01A-11R-A31O-07	stage i	t1a	female		
TCGA-KO-8403-01A-11R-2315-07	stage i	t1a	male		
TCGA-KL-8332-01A-11R-2315-07	stage i	t1b	male		
TCGA-HE-A5NL-01A-11R-A26U-07	stage i	t1a	male		
TCGA-KN-8418-01A-11R-2315-07	stage ii	t2	female		
TCGA-5P-A9K2-01A-11R-A42S-07	NA	t1b	male		
TCGA-G7-7502-01A-11R-2204-07	stage i	t1b	male		
TCGA-F9-A7Q0-01A-11R-A36F-07	stage i	t1b	female		
TCGA-BP-4776-01A-01R-1289-07	stage i	t1a	male		
TCGA-UZ-A9PK-01A-11R-A38C-07	stage i	t1	male		
TCGA-B1-A47O-01A-11R-A24Z-07	stage i	t1b	female		
TCGA-GL-A59R-01A-11R-A26U-07	stage iii	t3c	male		
TCGA-B0-5102-01A-01R-1420-07	stage i	t1	female		
TCGA-DW-7840-01A-11R-A32Z-07	stage i	t1b	male		
TCGA-P4-A5ED-01A-11R-A28H-07	stage i	t1a	male		
TCGA-DW-7836-01A-11R-2139-07	stage i	t1a	male		
TCGA-KN-8426-01A-11R-2315-07	stage iv	t3a	male		
TCGA-KL-8340-01A-11R-2315-07	stage ii	t2	male		
TCGA-KO-8407-01A-11R-2315-07	stage ii	t2	male		

TCGA-BP-4761-01A-01R-1289-07	stage iii	t3a	male		
TCGA-KO-8411-01A-11R-2315-07	stage i	t1b	male		
TCGA-CW-5585-01A-01R-1541-07	stage iv	t3b	male		
TCGA-GL-A59T-01A-21R-A28H-07	stage i	t1b	male		
TCGA-BQ-5886-01A-11R-1592-07	stage iii	t3a	male		
TCGA-DW-7834-01A-11R-2139-07	stage i	t1	male		
TCGA-KM-8439-01A-11R-2315-07	stage i	t1b	male		
TCGA-A4-A772-01A-11R-A33Z-07	stage i	t1b	male		
TCGA-KV-A6GD-01A-11R-A31O-07	stage i	t1b	male		
TCGA-BQ-7058-01A-11R-1965-07	stage iii	t3	male		
TCGA-B9-A5W8-01A-11R-A28H-07	stage ii	t2b	male		
TCGA-KL-8341-01A-11R-2315-07	stage iv	t3b	male		
TCGA-IA-A40U-01A-11R-A24Z-07	stage iii	t3b	male		
TCGA-B1-A47M-01A-11R-A24Z-07	stage iii	t3a	male		
TCGA-SX-A7SR-01A-12R-A36F-07	stage ii	t2a	male		
TCGA-KN-8419-01A-11R-2315-07	stage ii	t2	male		
TCGA-KM-8438-01A-11R-2315-07	stage ii	t2	female		
TCGA-KV-A74V-01A-11R-A33Z-07	stage i	t1a	male		
TCGA-KL-8342-01A-11R-2315-07	stage ii	t2b	female		
TCGA-B0-5117-01A-01R-1420-07	stage i	t1b	male		
TCGA-BQ-5880-01A-11R-1592-07	stage iii	t3a	male		
TCGA-KO-8416-01A-11R-2315-07	stage iii	t3a	male		
TCGA-A4-7583-01A-11R-A32Z-07	stage i	t1	male		
TCGA-G6-A8L8-01A-21R-A37O-07	stage i	t1b	female		
TCGA-KO-8405-01A-11R-2315-07	stage iii	t3a	male		
TCGA-IA-A83T-01A-11R-A355-07	NA	tx	male		
TCGA-KL-8337-01A-11R-2315-07	stage ii	t2	male		
TCGA-KL-8338-01A-11R-2315-07	stage iii	t3a	male		
TCGA-G7-A8LC-01A-11R-A36F-07	stage i	t1a	male		

TCGA-A3-3383-01A-02R-1325-07	stage i	t1	male		
TCGA-B9-4617-01A-01R-1193-07	NA	t1a	male		
TCGA-AL-3472-01A-01R-1193-07	NA	t2	male		
TCGA-2Z-A9JE-01A-11R-A42S-07	stage i	t1a	male		
TCGA-KL-8346-01A-11R-2315-07	stage ii	t2b	male		
TCGA-KL-8335-01A-11R-2315-07	stage iii	t3a	male		
TCGA-B1-A47N-01A-11R-A24Z-07	stage i	t1a	male		
TCGA-B1-7332-01A-11R-A32Z-07	stage ii	t2a	female		
TCGA-HE-A5NK-01A-11R-A26U-07	NA	t1b	male		
TCGA-MH-A560-01A-11R-A26U-07	stage i	t1a	male		
TCGA-KO-8413-01A-11R-2315-07	stage i	t1	male		
TCGA-B9-4114-01A-01R-1193-07	NA	t2	male		
TCGA-Y8-A898-01A-11R-A355-07	stage i	t1a	male		
TCGA-KV-A6GE-01A-11R-A31O-07	stage i	t1a	male		
TCGA-A4-7286-01A-11R-A32Z-07	NA	t3a	male		
TCGA-A3-A8OV-01A-11R-A37O-07	stage i	t1a	male		
TCGA-MH-A561-01A-11R-A26U-07	stage i	t1a	male		
TCGA-KO-8408-01A-11R-2315-07	stage iii	t3a	male		
TCGA-KO-8414-01A-11R-2315-07	stage ii	t2	female		
TCGA-KN-8422-01A-11R-2315-07	stage i	t1a	female		
TCGA-B1-A656-01A-11R-A31O-07	stage i	t1a	male		
TCGA-KN-8433-01A-11R-2315-07	stage iii	t3a	female		
TCGA-PJ-A5Z9-01A-11R-A28H-07	stage i	t1b	female		
TCGA-2Z-A9JP-01A-11R-A42S-07	stage i	t1a	male		
TCGA-WN-AB4C-01A-11R-A42S-07	stage i	t1a	female		
TCGA-2Z-A9J1-01A-11R-A38C-07	stage i	t1a	male		
TCGA-AK-3451-01A-02R-1188-07	stage ii	t2	male		
TCGA-KO-8415-01A-11R-2315-07	stage i	t1	female		
TCGA-KN-8421-01A-11R-2315-07	stage ii	t2	female		

TCGA-A3-3328-01A-01R-0864-07	stage i	t1b	male		
TCGA-AK-3447-01A-01R-1766-07	stage ii	t2	male		
TCGA-KL-8328-01A-11R-2315-07	#N/A	#N/A	#N/A		
TCGA-KN-8431-01A-11R-2315-07	stage ii	t2	female		
TCGA-G7-6795-01A-11R-1965-07	stage i	t1a	male		
TCGA-KO-8409-01A-11R-2315-07	stage ii	t2b	male		
TCGA-2Z-A9J5-01A-21R-A38C-07	stage ii	t2	male		
TCGA-BQ-5881-01A-11R-1592-07	stage i	t1a	male		
TCGA-KL-8324-01A-11R-2315-07	stage ii	t2	female		
TCGA-KL-8327-01A-11R-2315-07	stage i	t1b	female		
TCGA-Y8-A8S1-01A-11R-A37K-07	stage i	t1a	male		
TCGA-BP-4334-01A-01R-1289-07	stage iii	t3a	male		
TCGA-MH-A854-01A-11R-A355-07	stage i	t1b	female		
TCGA-MH-A55Z-01A-11R-A26U-07	stage i	t1b	male		
TCGA-KM-8476-01A-11R-2315-07	stage i	t1	male		
TCGA-AK-3427-01A-01R-0864-07	stage i	t1a	male		
TCGA-F9-A7VF-01A-11R-A33Z-07	stage i	t1a	female		
TCGA-GL-8500-01A-11R-2404-07	stage i	t1b	male		
TCGA-IZ-A6M9-01A-11R-A31O-07	stage i	t1a	male		
TCGA-A4-A6HP-01A-11R-A31O-07	stage i	t1a	male		
TCGA-MH-A562-01A-11R-A26U-07	stage i	t1a	male		
TCGA-IZ-A6M8-01A-11R-A31O-07	stage i	t1a	male		
TCGA-2Z-A9JT-01A-11R-A42S-07	stage i	t1a	male		
TCGA-B1-A654-01A-11R-A31O-07	stage i	t1a	female		
TCGA-P4-AAVL-01A-11R-A42S-07	stage iii	t3b	male		
TCGA-2Z-A9JQ-01A-11R-A42S-07	stage i	t1a	male		
TCGA-B0-5702-01A-11R-1541-07	stage i	t1b	male		
TCGA-KN-8427-01A-11R-2315-07	stage iv	t4	male		
TCGA-CZ-5456-01A-01R-1503-07	stage ii	t2	male	MAP4K4	>75 percentile

TCGA-B0-4698-01A-01R-1503-07	stage iv	t4	male			
TCGA-BQ-5894-01A-11R-1592-07	stage iv	t3b	male			
TCGA-BP-4789-01A-01R-1305-07	stage i	t1a	male			
TCGA-B0-5690-01A-11R-1541-07	stage i	t1b	female			
TCGA-A3-3376-01A-02R-1420-07	stage i	t1a	male			
TCGA-BP-4974-01A-01R-1334-07	stage iv	t3a	male			
TCGA-CW-6093-01A-11R-1672-07	stage i	t1a	male			
TCGA-CJ-4905-01A-02R-1426-07	stage i	t1a	female			
TCGA-B0-5097-01A-01R-1420-07	stage iii	t3b	female			
TCGA-BP-4345-01A-01R-1289-07	stage iii	t3b	male			
TCGA-CJ-4908-01A-01R-1426-07	stage i	t1a	male			
TCGA-B0-5080-01A-01R-1503-07	stage iv	t3a	male			
TCGA-BP-4353-01A-02R-1289-07	stage i	t1	male			
TCGA-B8-5163-01A-01R-1420-07	stage iii	t3a	female			
TCGA-BP-4991-01A-01R-1334-07	stage i	t1a	male			
TCGA-GL-7966-01A-11R-2204-07	stage iii	t3	female			
TCGA-A3-3316-01A-01R-0864-07	stage ii	t2	male			
TCGA-B0-5095-01A-01R-1420-07	stage iii	t3a	male			
TCGA-CJ-5671-01A-11R-1541-07	stage i	t1a	male			
TCGA-BP-5198-01A-01R-1426-07	stage iii	t3b	male			
TCGA-B0-4703-01A-01R-1277-07	stage iv	t3a	male			
TCGA-BP-4961-01A-01R-1334-07	stage i	t1a	male			
TCGA-B0-4836-01A-01R-1305-07	stage iv	t3b	male			
TCGA-B0-4813-01A-01R-1277-07	stage iii	t3b	male			
TCGA-B4-5832-01A-11R-1672-07	stage iii	t3b	male			
TCGA-BP-5195-01A-02R-1426-07	stage i	t1a	male			
TCGA-BQ-5879-01A-11R-1592-07	stage iii	t3b	female			
TCGA-BP-4344-01A-01R-1289-07	stage i	t1a	female			
TCGA-BP-4770-01A-01R-1503-07	stage iv	t4	female			

TCGA-B2-5636-01A-02R-1541-07	stage i	t1a	male		
TCGA-DV-A4VZ-01A-11R-A266-07	stage i	t1a	male		
TCGA-BP-5196-01A-01R-1426-07	stage i	t1a	male		
TCGA-BP-4999-01A-01R-1334-07	stage i	t1a	male		
TCGA-G7-6789-01A-11R-1965-07	stage iv	t3a	female		
TCGA-B0-5710-01A-11R-1672-07	stage i	t1b	male		
TCGA-B0-5108-01A-01R-1420-07	stage iii	t3a	male		
TCGA-DV-5574-01A-01R-1541-07	stage i	t1a	male		
TCGA-BP-4983-01A-01R-1334-07	stage iii	t3a	female		
TCGA-CJ-4918-01A-01R-1426-07	stage iv	t3a	male		
TCGA-B0-4843-01A-01R-1277-07	stage iii	t3a	male		
TCGA-A3-A8CQ-01A-11R-A37O-07	stage i	t1a	female		
TCGA-DV-5565-01A-01R-1541-07	stage i	t1a	male		
TCGA-BP-5189-01A-02R-1426-07	stage i	t1b	male		
TCGA-CJ-4902-01A-01R-1426-07	stage iii	t3a	male		
TCGA-BP-5194-01A-02R-1426-07	stage i	t1a	male		
TCGA-BP-4986-01A-01R-1334-07	stage i	t1a	male		
TCGA-B8-5158-01A-01R-1420-07	stage iii	t3a	male		
TCGA-BP-5199-01A-01R-1426-07	stage ii	t2	male		
TCGA-B0-4688-01A-01R-1277-07	stage iv	t4	male		
TCGA-A3-3349-01A-01R-1188-07	stage i	t1b	female		
TCGA-CZ-5988-01A-11R-1672-07	stage i	t1b	male		
TCGA-B0-4700-01A-02R-1541-07	stage iv	t4	male		
TCGA-BP-5178-01A-01R-1426-07	stage iv	t3a	male		
TCGA-CW-5588-01A-01R-1541-07	stage i	t1a	female		
TCGA-B8-5545-01A-01R-1672-07	stage i	t1a	male		
TCGA-B8-A8YJ-01A-13R-A39I-07	stage i	t1b	female		
TCGA-B2-5633-01A-01R-1541-07	stage i	t1b	male		
TCGA-CJ-4907-01A-01R-1426-07	stage iii	t3b	male		

TCGA-BP-4763-01A-01R-1289-07	stage i	t1a	female		
TCGA-B0-5098-01A-01R-1420-07	stage i	t1	female		
TCGA-CJ-4634-01A-02R-1325-07	stage i	t1b	female		
TCGA-A3-3308-01A-02R-1325-07	stage iii	t3b	female		
TCGA-A3-A6NL-01A-11R-A33J-07	stage i	t1b	female		
TCGA-BP-4988-01A-01R-1334-07	stage i	t1a	male		
TCGA-B8-5165-01A-01R-1420-07	stage i	t1a	male		
TCGA-BP-4352-01A-01R-1289-07	stage iv	t3b	female		
TCGA-BP-4166-01A-02R-1289-07	stage iii	t3a	male		
TCGA-BP-5200-01A-01R-1426-07	stage ii	t2	male		
TCGA-BP-5010-01A-02R-1420-07	stage iii	t3a	male		
TCGA-CJ-4891-01A-01R-1305-07	stage iii	t3c	female		
TCGA-B8-5162-01A-01R-1420-07	stage ii	t2a	male		
TCGA-A3-3347-01A-02R-1325-07	stage iii	t1b	female		
TCGA-BP-4982-01A-01R-1334-07	stage i	t1b	male		
TCGA-B4-5844-01A-11R-1672-07	stage ii	t2	female		
TCGA-GK-A6C7-01A-11R-A33J-07	stage i	t1a	female		
TCGA-B0-5110-01A-01R-1420-07	stage i	t1a	female		
TCGA-DV-5568-01A-01R-1541-07	stage i	t1a	male		
TCGA-B2-4101-01A-02R-1277-07	stage ii	t2a	male		
TCGA-BP-4326-01A-01R-1289-07	stage i	t1b	female		
TCGA-B8-4620-01A-02R-1325-07	stage iii	t3a	female		
TCGA-B0-4837-01A-01R-1305-07	stage i	t1b	male		
TCGA-EU-5905-01A-11R-1672-07	stage i	t1	female		
TCGA-CJ-4920-01A-01R-1426-07	stage i	t1b	female		
TCGA-BP-4347-01A-01R-1289-07	stage iii	t3b	male		
TCGA-B0-4690-01A-01R-1277-07	stage iv	t4	male		
TCGA-B0-4822-01A-01R-1277-07	stage ii	t2	male		
TCGA-CJ-5676-01A-11R-1541-07	stage iii	t3b	male		

TCGA-BP-4354-01A-02R-1289-07	stage iv	t4	male		
TCGA-CW-6088-01A-11R-1672-07	stage i	t1b	male		
TCGA-A4-7915-01A-11R-2204-07	stage ii	t2b	female		
TCGA-A3-3382-01A-02R-1325-07	stage i	t1b	male		
TCGA-CJ-4899-01A-01R-1334-07	stage i	t1b	male		
TCGA-CJ-4923-01A-01R-1426-07	stage iv	t3a	female		
TCGA-EU-5906-01A-11R-1672-07	stage i	t1b	male		
TCGA-B0-5116-01A-02R-1420-07	stage iii	t3b	male		
TCGA-A4-7828-01A-11R-2139-07	stage i	t1a	female		
TCGA-2Z-A9JJ-01A-11R-A42S-07	stage i	t1a	female		
TCGA-BP-4169-01A-02R-1289-07	stage ii	t2	female		
TCGA-BP-4759-01A-01R-1289-07	stage i	t1a	male		
TCGA-BP-5008-01A-01R-1334-07	stage i	t1a	male		
TCGA-B0-5121-01A-02R-1420-07	stage i	t1b	male		
TCGA-BP-4985-01A-01R-1334-07	stage iii	t3a	male		
TCGA-B0-5109-01A-02R-1420-07	stage iii	t3b	male		
TCGA-B0-5812-01A-11R-1672-07	stage i	t1b	male		
TCGA-BP-4342-01A-01R-1289-07	stage ii	t2	male		
TCGA-CZ-4857-01A-01R-1305-07	stage iv	t3a	male		
TCGA-CJ-4895-01A-01R-1305-07	stage iv	t3a	male		
TCGA-CZ-5469-01A-01R-1503-07	stage ii	t2	male		
TCGA-CZ-5461-01A-01R-1503-07	stage iv	t1b	male		
TCGA-CZ-5989-01A-11R-1672-07	stage ii	t2	male		
TCGA-B4-5834-01A-11R-1672-07	stage i	t1	male		
TCGA-BP-4799-01A-01R-1305-07	stage iii	t3b	male		
TCGA-CJ-4904-01A-02R-1426-07	stage iv	t3a	female		
TCGA-CW-6097-01A-11R-1672-07	stage iii	t3a	male		
TCGA-BP-4782-01A-02R-1420-07	stage i	t1a	female		
TCGA-B8-4143-01A-01R-1188-07	stage iv	t3a	female		

TCGA-KO-8404-01A-11R-2315-07	stage iv	t4	male		
TCGA-AK-3445-01A-02R-1277-07	stage iii	t3a	male		
TCGA-B9-4117-01A-02R-1351-07	NA	t2	female		
TCGA-BP-5177-01A-01R-1426-07	stage i	t1a	female		
TCGA-A3-A8OW-01A-11R-A37O-07	stage iii	t3a	male		
TCGA-CW-5590-01A-01R-1541-07	stage iv	t3a	male		
TCGA-BP-4787-01A-01R-1305-07	stage iv	t3a	female		
TCGA-B8-A54H-01A-11R-A33J-07	stage ii	t2a	female		
TCGA-BP-4965-01A-01R-1334-07	stage i	t1a	male		
TCGA-CJ-4868-01A-01R-1305-07	stage iv	t3a	male		
TCGA-CJ-4886-01A-01R-1305-07	stage i	t1a	female		
TCGA-CJ-6028-01A-11R-1672-07	stage iv	t3a	male		
TCGA-CW-6090-01A-11R-1672-07	stage i	t1b	male		
TCGA-B0-4712-01A-01R-1503-07	stage iv	t3a	male		
TCGA-A3-3372-01A-02R-1325-07	stage iii	t3	male		
TCGA-BP-4340-01A-01R-1289-07	stage i	t1b	female		
TCGA-G7-6793-01A-11R-1965-07	stage iv	t3a	female		
TCGA-CJ-5689-01A-11R-1541-07	stage i	t1b	male		
TCGA-CZ-5982-01A-11R-1672-07	stage i	t1a	female		
TCGA-BP-4803-01A-01R-1305-07	stage iii	t3a	male		
TCGA-B0-5115-01A-01R-1420-07	stage iv	t2	male		
TCGA-B8-A7U6-01A-12R-A37O-07	stage i	t1a	female		
TCGA-J7-8537-01A-11R-2404-07	stage iii	t3	female		
TCGA-B0-5113-01A-01R-1420-07	stage iii	t3a	female		
TCGA-B0-4821-01A-01R-1503-07	stage iii	t3b	female		
TCGA-BP-4960-01A-01R-1334-07	stage ii	t2	male		
TCGA-CZ-5468-01A-01R-1503-07	stage iv	t3b	male		
TCGA-AK-3431-01A-02R-1277-07	stage ii	t2	female		
TCGA-A3-3323-01A-02R-1325-07	stage i	t1b	male		

TCGA-CJ-4901-01A-01R-1426-07	stage iii	t3b	male		
TCGA-B0-5693-01A-11R-1541-07	stage i	t1b	female		
TCGA-BP-4975-01A-01R-1334-07	stage i	t1b	male		
TCGA-B2-A4SR-01A-11R-A266-07	stage ii	t2a	male		
TCGA-CJ-4635-01A-02R-1305-07	stage i	t1b	male		
TCGA-BQ-5893-01A-11R-1592-07	stage iv	t3a	male		
TCGA-BP-4987-01A-01R-1334-07	stage i	t1b	female		
TCGA-CJ-6031-01A-11R-1672-07	stage i	t1b	male		
TCGA-BP-5176-01A-01R-1426-07	stage i	t1a	female		
TCGA-CJ-4890-01A-01R-1305-07	stage iv	t3a	male		
TCGA-BP-4993-01A-02R-1420-07	stage i	t1a	male		
TCGA-CJ-6033-01A-11R-1672-07	stage iv	t3a	female		
TCGA-DV-5569-01A-01R-1541-07	stage i	t1a	female		
TCGA-BP-4963-01A-01R-1334-07	stage i	t1b	male		
TCGA-CW-5580-01A-01R-1672-07	stage iv	t3a	female		
TCGA-BP-4766-01A-01R-1289-07	stage i	t1a	female		
TCGA-B0-5709-01A-11R-1541-07	stage iii	t3a	female		
TCGA-B0-4699-01A-01R-1277-07	stage iv	t4	male		
TCGA-CJ-4889-01A-01R-1305-07	stage i	t1a	female		
TCGA-B8-5550-01A-01R-1541-07	stage iii	t3a	male		
TCGA-CJ-5683-01A-11R-1541-07	stage i	t1b	male		
TCGA-BP-4162-01A-02R-1325-07	stage i	t1b	female		
TCGA-BP-4784-01A-01R-1305-07	stage i	t1a	female		
TCGA-BP-4795-01A-02R-1420-07	stage i	t1a	female		
TCGA-CW-5581-01A-02R-1541-07	stage i	t1b	male		
TCGA-BP-4355-01A-01R-1289-07	stage iii	t3a	female		
TCGA-BP-5169-01A-01R-1426-07	stage i	t1b	male		
TCGA-BP-4159-01A-02R-1289-07	stage i	t1b	male		
TCGA-CZ-4854-01A-01R-1305-07	stage i	t1b	male		

TCGA-CZ-4865-01A-02R-1503-07	stage i	t1a	female		
TCGA-CJ-6032-01A-11R-1672-07	stage ii	t2	female		
TCGA-MM-A564-01A-11R-A266-07	stage ii	t2a	male		
TCGA-A3-3311-01A-02R-1325-07	stage i	t1	male		
TCGA-A3-3313-01A-02R-1325-07	stage i	t1b	male		
TCGA-BP-4964-01A-01R-1334-07	stage i	t1a	female		
TCGA-BP-4998-01A-01R-1334-07	stage i	t1a	male		
TCGA-B4-5843-01A-11R-1672-07	stage i	t1	male		
TCGA-KL-8344-01A-11R-2315-07	stage iii	t3a	male		
TCGA-CJ-4888-01A-01R-1305-07	stage iv	t3a	male		
TCGA-B0-5711-01A-11R-1672-07	stage iii	t3b	male		
TCGA-BP-4970-01A-01R-1334-07	stage iii	t1a	male		
TCGA-BP-5187-01A-01R-1426-07	stage i	t1a	male		
TCGA-BP-4765-01A-01R-1289-07	stage i	t1a	male		
TCGA-CJ-4643-01A-02R-1325-07	stage ii	t2b	female		
TCGA-BP-4771-01A-01R-1289-07	stage iv	t3a	male		
TCGA-B0-5713-01A-11R-1672-07	stage iii	t3b	female		
TCGA-AL-3466-01A-02R-1351-07	stage iv	t3b	male		
TCGA-BP-4976-01A-01R-1334-07	stage i	t1a	male		
TCGA-B0-5106-01A-01R-1420-07	stage i	t1a	male		
TCGA-CJ-5684-01A-11R-1541-07	stage iii	t3a	male		
TCGA-BP-5175-01A-01R-1426-07	stage i	t1a	male		
TCGA-5P-A9K9-01A-11R-A42S-07	NA	t3a	female		
TCGA-CJ-4872-01A-01R-1305-07	stage i	t1b	male		
TCGA-CJ-6027-01A-11R-1672-07	stage i	t1a	male		
TCGA-BP-4332-01A-01R-1289-07	stage iii	t3a	male		
TCGA-B8-5549-01A-01R-1541-07	stage i	t1b	male		
TCGA-DV-5567-01A-01R-1541-07	stage i	t1a	female		
TCGA-A3-A8OX-01A-11R-A37O-07	stage i	t1a	female		

TCGA-B0-4841-01A-01R-1277-07	stage iv	t2	male		
TCGA-P4-A5E8-01A-11R-A28H-07	stage iii	t2a	male		
TCGA-B8-5553-01A-01R-1541-07	stage i	t1b	female		
TCGA-B0-5081-01A-01R-1334-07	stage iii	t3b	female		
TCGA-A3-3359-01A-01R-0864-07	stage i	t1a	female		
TCGA-BP-4790-01A-01R-1305-07	stage i	t1a	male		
TCGA-CJ-4882-01A-02R-1426-07	stage iii	t3a	male		
TCGA-CJ-4639-01A-02R-1325-07	stage ii	t2	female		
TCGA-B0-5100-01A-01R-1420-07	stage iii	t3a	male		
TCGA-B0-4845-01A-01R-1277-07	stage iv	t3a	male		
TCGA-BP-4781-01A-01R-1305-07	stage i	t1a	male		
TCGA-B0-4697-01A-01R-1277-07	stage iv	t3b	female		
TCGA-B0-5084-01A-01R-1334-07	stage iv	t3a	male		
TCGA-A3-3387-01A-01R-1541-07	stage i	t1a	male		
TCGA-A3-3378-01A-02R-1325-07	stage i	t1	male		
TCGA-BP-5170-01A-01R-1426-07	stage i	t1a	male		
TCGA-F9-A97G-01A-11R-A38C-07	stage iii	t3	male		
TCGA-AL-3472-01A-01R-1193-07	NA	t2	male		
TCGA-Y8-A898-01A-11R-A355-07	stage i	t1a	male		
TCGA-BP-5190-01A-01R-1426-07	stage i	t1a	male		
TCGA-BQ-7060-01A-11R-1965-07	stage i	t1a	male		
TCGA-P4-AAVM-01A-11R-A42S-07	stage i	t1a	male		
TCGA-B2-4099-01A-02R-1188-07	stage i	t1a	male		
TCGA-BP-4761-01A-01R-1289-07	stage iii	t3a	male		
TCGA-P4-A5E7-01A-31R-A28H-07	stage i	t1b	female		
TCGA-HE-A5NK-01A-11R-A26U-07	NA	t1b	male		
TCGA-B9-5155-01A-01R-1592-07	stage iii	t3a	male		
TCGA-T7-A92I-01A-11R-A37O-07	stage i	t1a	female		
TCGA-DW-7837-01A-11R-2139-07	stage i	t1b	male		<25 Percentile

TCGA-GL-7773-01A-11R-A32Z-07	stage i	t1a	male		
TCGA-B0-4847-01A-01R-1277-07	stage iv	t3a	male		
TCGA-KN-8422-01A-11R-2315-07	stage i	t1a	female		
TCGA-EV-5902-01A-11R-1592-07	stage i	t1	male		
TCGA-BQ-7059-01A-11R-1965-07	stage i	t1b	male		
TCGA-SX-A7SR-01A-12R-A36F-07	stage ii	t2a	male		
TCGA-Y8-A8RZ-01A-11R-A37K-07	stage i	t1	male		
TCGA-O9-A75Z-01A-11R-A33Z-07	stage i	t1a	male		
TCGA-DW-7838-01A-11R-2139-07	stage i	t1b	male		
TCGA-BQ-5883-01A-11R-1592-07	stage i	t1a	female		
TCGA-HE-7130-01A-11R-1965-07	stage iii	t3	female		
TCGA-CJ-5675-01A-11R-1541-07	stage ii	t2a	male		
TCGA-B8-5546-01A-01R-1541-07	stage i	t1b	female		
TCGA-BQ-5886-01A-11R-1592-07	stage iii	t3a	male		
TCGA-B9-4116-01A-02R-1351-07	NA	t2a	female		
TCGA-DW-5560-01A-01R-1592-07	stage i	t1b	female		
TCGA-5P-A9JU-01A-11R-A42S-07	stage iii	t3a	male		
TCGA-B9-5156-01A-01R-1592-07	NA	t1a	male		
TCGA-DW-7836-01A-11R-2139-07	stage i	t1a	male		
TCGA-B0-5107-01A-01R-1420-07	stage iv	t2	female		
TCGA-B8-A54G-01A-11R-A266-07	stage i	t1a	male		
TCGA-KV-A6GE-01A-11R-A31O-07	stage i	t1a	male		
TCGA-G6-A5PC-01A-11R-A33J-07	stage iv	t1b	female		
TCGA-G6-A8L8-01A-21R-A37O-07	stage i	t1b	female		
TCGA-P4-AAVK-01A-11R-A42S-07	stage iii	t3a	male		
TCGA-A3-A8OV-01A-11R-A37O-07	stage i	t1a	male		
TCGA-DW-7841-01A-11R-A32Z-07	stage i	t1b	male		
TCGA-SX-A71R-01A-12R-A33Z-07	stage i	t1a	male		
TCGA-B9-4113-01A-01R-1193-07	stage i	t1	male		

TCGA-5P-A9JY-01A-11R-A42S-07	NA	t2	male			
TCGA-5P-A9K4-01A-11R-A42S-07	NA	t1b	male			
TCGA-UZ-A9PZ-01A-11R-A42S-07	stage iii	t3a	male			
TCGA-UZ-A9PN-01A-11R-A38C-07	stage iii	t3	male			
TCGA-5P-A9KE-01A-11R-A42S-07	NA	t1b	male			
TCGA-DV-A4VX-01A-11R-A266-07	stage iv	t3b	male			
TCGA-GL-A59R-01A-11R-A26U-07	stage iii	t3c	male			
TCGA-A4-A57E-01A-11R-A26U-07	stage iv	t2a	male			
TCGA-5P-A9K6-01A-11R-A42S-07	NA	t1a	male			
TCGA-A4-A48D-01A-11R-A24Z-07	stage i	t1b	male			
TCGA-5P-A9K2-01A-11R-A42S-07	NA	t1b	male			
TCGA-GL-A9DE-01A-11R-A37K-07	stage i	t1b	male			
TCGA-BQ-5887-01A-11R-1965-07	stage iii	t3a	male			
TCGA-UZ-A9PU-01A-11R-A42S-07	stage i	t1b	male			
TCGA-P4-A5ED-01A-11R-A28H-07	stage i	t1a	male			
TCGA-GL-A9DD-01A-11R-A37K-07	stage i	t1a	male			
TCGA-2Z-A9JE-01A-11R-A42S-07	stage i	t1a	male			
TCGA-F9-A7Q0-01A-11R-A36F-07	stage i	t1b	female			
TCGA-SX-A7SS-01A-11R-A36F-07	stage i	t1	male			
TCGA-A4-8517-01A-11R-2404-07	stage i	t1a	male			
TCGA-A4-8516-01A-11R-2404-07	stage iii	t3a	male			
TCGA-B9-A5W8-01A-11R-A28H-07	stage ii	t2b	male			
TCGA-B1-A47M-01A-11R-A24Z-07	stage iii	t3a	male			
TCGA-F9-A7VF-01A-11R-A33Z-07	stage i	t1a	female			
TCGA-HE-A5NL-01A-11R-A26U-07	stage i	t1a	male			
TCGA-WN-AB4C-01A-11R-A42S-07	stage i	t1a	female			
TCGA-AL-A5DJ-01A-11R-A26U-07	stage iii	t3a	female			
TCGA-B9-7268-01A-11R-A32Z-07	stage ii	t2	male			
TCGA-5P-A9JZ-01A-11R-A42S-07	NA	t1b	female			

TCGA-AK-3429-01A-02R-1325-07	stage ii	t2	female			
TCGA-A4-8098-01A-11R-2404-07	stage i	t1a	male			
TCGA-2K-A9WE-01A-11R-A38C-07	stage ii	t2b	male			
TCGA-KN-8437-01A-11R-2315-07	stage i	t1a	female			
TCGA-2Z-A9JP-01A-11R-A42S-07	stage i	t1a	male			
TCGA-Y8-A8RY-01A-11R-A37K-07	stage i	t1b	male			
TCGA-B0-4713-01A-01R-1277-07	stage iii	t3b	female			
TCGA-UZ-A9PO-01A-11R-A38C-07	stage i	t1a	male			
TCGA-B8-4154-01A-01R-1188-07	stage i	t1a	female			
TCGA-BP-4962-01A-01R-1334-07	stage ii	t2	male			
TCGA-SX-A7SU-01A-11R-A36F-07	stage i	t1	male			
TCGA-KL-8332-01A-11R-2315-07	stage i	t1b	male			
TCGA-A3-3363-01A-01R-0864-07	stage ii	t2	male			
TCGA-Q2-A5QZ-01A-11R-A28H-07	stage iii	t3a	female			
TCGA-B1-A47N-01A-11R-A24Z-07	stage i	t1a	male			
TCGA-P4-AAVL-01A-11R-A42S-07	stage iii	t3b	male			
TCGA-KV-A6GD-01A-11R-A31O-07	stage i	t1b	male			
TCGA-KV-A74V-01A-11R-A33Z-07	stage i	t1a	male			
TCGA-B9-A5W7-01A-11R-A31O-07	stage i	t1a	male			
TCGA-KL-8324-01A-11R-2315-07	stage ii	t2	female			
TCGA-B0-5692-01A-11R-1541-07	stage iii	t3b	female			
TCGA-2Z-A9J5-01A-21R-A38C-07	stage ii	t2	male			
TCGA-UZ-A9PM-01A-21R-A38C-07	stage ii	t2	male			
TCGA-CJ-4638-01A-02R-1325-07	stage iv	t3a	female			
TCGA-B0-5083-01A-02R-1420-07	stage i	t1a	male			
TCGA-B8-A54D-01A-21R-A266-07	stage iii	t3a	male			
TCGA-5P-A9K0-01A-11R-A42S-07	NA	t1a	male			
TCGA-SX-A7SO-01A-11R-A355-07	stage i	t1b	male			
TCGA-AK-3465-01A-02R-1325-07	stage i	t1b	female			

TCGA-2Z-A9JQ-01A-11R-A42S-07	stage i	t1a	male		
TCGA-UZ-A9Q0-01A-12R-A42S-07	stage i	t1	male		
TCGA-IA-A83T-01A-11R-A355-07	NA	tx	male		
TCGA-B3-4104-01A-02R-1351-07	stage ii	t2	male		
TCGA-BQ-5876-01A-11R-1592-07	stage i	t1a	male		
TCGA-MH-A854-01A-11R-A355-07	stage i	t1b	female		
TCGA-KN-8423-01A-11R-2315-07	stage i	t1b	male		
TCGA-SX-A71W-01A-12R-A355-07	stage i	t1b	male		
TCGA-HE-7129-01A-11R-1965-07	stage ii	t2	male		
TCGA-A3-3383-01A-02R-1325-07	stage i	t1	male		
TCGA-IZ-A6M8-01A-11R-A31O-07	stage i	t1a	male		
TCGA-P4-AAVO-01A-11R-A42S-07	stage i	t1b	male		
TCGA-KL-8323-01A-21R-2315-07	stage iii	t3b	female		
TCGA-2Z-A9J1-01A-11R-A38C-07	stage i	t1a	male		
TCGA-KO-8405-01A-11R-2315-07	stage iii	t3a	male		
TCGA-A4-A772-01A-11R-A33Z-07	stage i	t1b	male		
TCGA-B9-4115-01A-01R-1193-07	stage i	t1a	male		
TCGA-B0-5117-01A-01R-1420-07	stage i	t1b	male		
TCGA-HE-A5NJ-01A-11R-A26U-07	NA	t1b	male		
TCGA-SX-A7SL-01A-11R-A355-07	stage i	t1a	male		
TCGA-GL-8500-01A-11R-2404-07	stage i	t1b	male		
TCGA-DW-7840-01A-11R-A32Z-07	stage i	t1b	male		
TCGA-KN-8419-01A-11R-2315-07	stage ii	t2	male		
TCGA-KM-8477-01A-11R-2315-07	stage i	t1a	male		
TCGA-WN-A9G9-01A-12R-A37K-07	stage i	t1b	male		
TCGA-IZ-A6M9-01A-11R-A31O-07	stage i	t1a	male		
TCGA-G7-A8LD-01A-11R-A36F-07	stage iii	t3a	male		
TCGA-B8-4151-01A-01R-1188-07	stage iii	t3a	female		
TCGA-G7-A8LB-01A-11R-A36F-07	stage iv	t2a	male		

TCGA-KN-8434-01A-11R-2315-07	stage ii	t2	female			
TCGA-2Z-A9JT-01A-11R-A42S-07	stage i	t1a	male			
TCGA-MH-A55Z-01A-11R-A26U-07	stage i	t1b	male			
TCGA-5P-A9KC-01A-11R-A42S-07	NA	t1b	female			
TCGA-KM-8438-01A-11R-2315-07	stage ii	t2	female			
TCGA-AL-3467-01A-02R-1351-07	NA	t1b	female			
TCGA-B0-5075-01A-01R-1334-07	stage iii	t3a	female			
TCGA-MH-A562-01A-11R-A26U-07	stage i	t1a	male			
TCGA-B0-5712-01A-11R-1672-07	stage iv	t2	female			
TCGA-B1-A47O-01A-11R-A24Z-07	stage i	t1b	female			
TCGA-KN-8425-01A-11R-2315-07	stage i	t1b	male			
TCGA-2Z-A9J6-01A-11R-A38C-07	stage i	t1a	male			
TCGA-MH-A560-01A-11R-A26U-07	stage i	t1a	male			
TCGA-2Z-A9J3-01A-12R-A38C-07	stage ii	t2	male			
TCGA-AK-3455-01A-01R-0864-07	stage iii	t3b	female			
TCGA-KO-8413-01A-11R-2315-07	stage i	t1	male			
TCGA-B1-A656-01A-11R-A31O-07	stage i	t1a	male			
TCGA-5P-A9K3-01A-11R-A42S-07	NA	t2	male			
TCGA-KO-8407-01A-11R-2315-07	stage ii	t2	male			
TCGA-A4-A6HP-01A-11R-A31O-07	stage i	t1a	male			
TCGA-CJ-4912-01A-01R-1426-07	stage ii	t2	male			
TCGA-KO-8403-01A-11R-2315-07	stage i	t1a	male			
TCGA-KM-8639-01A-11R-2403-07	stage i	t1a	male			
TCGA-J7-A8I2-01A-12R-A36F-07	stage i	t1b	male			
TCGA-BQ-5885-01A-11R-1592-07	stage iii	t3	male			
TCGA-KL-8342-01A-11R-2315-07	stage ii	t2b	female			
TCGA-BQ-5888-01A-11R-1592-07	stage i	t1a	female			
TCGA-5P-A9K8-01A-11R-A42S-07	NA	t3a	female			
TCGA-KN-8424-01A-11R-2315-07	stage i	t1b	female			

TCGA-B2-3923-01A-02R-1325-07	stage ii	t2	male		
TCGA-KL-8341-01A-11R-2315-07	stage iv	t3b	male		
TCGA-A4-8312-01A-11R-2404-07	stage i	t1	male		
TCGA-UZ-A9PL-01A-11R-A38C-07	stage ii	t2	male		
TCGA-MH-A857-01A-11R-A355-07	stage i	t1a	male		
TCGA-B9-4114-01A-01R-1193-07	NA	t2	male		
TCGA-AK-3433-01A-02R-1277-07	stage ii	t2	female		
TCGA-KL-8336-01A-11R-2315-07	stage iv	t3b	female		
TCGA-2Z-A9J7-01A-11R-A38C-07	stage iv	t2	male		
TCGA-KO-8406-01A-11R-2315-07	stage i	t1	female		
TCGA-KL-8343-01A-11R-2315-07	stage iii	t3	male		
TCGA-KM-8442-01A-11R-2315-07	stage ii	t2	male		
TCGA-5P-A9KH-01A-11R-A42S-07	NA	t1a	male		
TCGA-KN-8435-01A-11R-2315-07	stage ii	t2	male		
TCGA-KL-8326-01A-11R-2315-07	stage iii	t3a	male		
TCGA-KO-8415-01A-11R-2315-07	stage i	t1	female		
TCGA-KN-8431-01A-11R-2315-07	stage ii	t2	female		
TCGA-KL-8340-01A-11R-2315-07	stage ii	t2	male		
TCGA-AK-3453-01A-02R-1277-07	stage ii	t2	female		
TCGA-2Z-A9J2-01A-11R-A38C-07	stage i	t1a	female		
TCGA-AK-3451-01A-02R-1188-07	stage ii	t2	male		
TCGA-2Z-A9JD-01A-11R-A42S-07	stage i	t1a	male		
TCGA-KN-8429-01A-11R-2315-07	stage iii	t3a	female		
TCGA-AK-3458-01A-01R-1503-07	stage i	t1b	male		
TCGA-KN-8436-01A-11R-2315-07	stage ii	t2	male		
TCGA-KM-8439-01A-11R-2315-07	stage i	t1b	male		
TCGA-KN-8421-01A-11R-2315-07	stage ii	t2	female		
TCGA-AL-3473-01A-01R-1193-07	stage ii	t2	male		
TCGA-KL-8345-01A-11R-2315-07	stage iii	t3a	male		

TCGA-4A-A93X-01A-11R-A37K-07	stage iv	t3a	male		
TCGA-KL-8334-01A-11R-2315-07	stage iii	t3a	female		
TCGA-KL-8327-01A-11R-2315-07	stage i	t1b	female		
TCGA-KN-8426-01A-11R-2315-07	stage iv	t3a	male		
TCGA-BP-4334-01A-01R-1289-07	stage iii	t3a	male		
TCGA-KO-8411-01A-11R-2315-07	stage i	t1b	male		
TCGA-KO-8416-01A-11R-2315-07	stage iii	t3a	male		
TCGA-KL-8335-01A-11R-2315-07	stage iii	t3a	male		
TCGA-AK-3443-01A-02R-1325-07	stage ii	t2	male		
TCGA-KL-8346-01A-11R-2315-07	stage ii	t2b	male		
TCGA-KL-8329-01A-11R-2315-07	stage i	t1b	female		
TCGA-KO-8417-01A-11R-2315-07	stage i	t1	female		
TCGA-KL-8331-01A-11R-2315-07	stage ii	t2	female		
TCGA-KO-8408-01A-11R-2315-07	stage iii	t3a	male		
TCGA-KN-8432-01A-11R-2315-07	stage ii	t2b	female		
TCGA-KN-8433-01A-11R-2315-07	stage iii	t3a	female		
TCGA-KL-8325-01A-11R-2315-07	stage ii	t2	female		
TCGA-KO-8409-01A-11R-2315-07	stage ii	t2b	male		
TCGA-AK-3440-01A-02R-1277-07	stage i	t1a	male		
TCGA-KM-8443-01A-11R-2315-07	stage ii	t2	male		
TCGA-A3-3374-01A-02R-1325-07	stage i	t1b	female		
TCGA-A3-3328-01A-01R-0864-07	stage i	t1b	male		
TCGA-KN-8418-01A-11R-2315-07	stage ii	t2	female		
TCGA-KO-8414-01A-11R-2315-07	stage ii	t2	female		
TCGA-AK-3427-01A-01R-0864-07	stage i	t1a	male		
TCGA-KL-8338-01A-11R-2315-07	stage iii	t3a	male		
TCGA-KL-8339-01A-11R-2315-07	#N/A	#N/A	#N/A		
TCGA-KM-8476-01A-11R-2315-07	stage i	t1	male		
TCGA-KL-8330-01A-11R-2315-07	stage ii	t2b	female		

TCGA-KN-8428-01A-11R-2315-07	stage ii	t2	male		
TCGA-KM-8440-01A-11R-2315-07	stage iii	t3a	male		
TCGA-KL-8337-01A-11R-2315-07	stage ii	t2	male		
TCGA-AK-3447-01A-01R-1766-07	stage ii	t2	male		
TCGA-CZ-4860-01A-01R-1305-07	stage iv	t4	male		
TCGA-AK-3427-01A-01R-0864-07	stage i	t1a	male		
TCGA-CZ-5982-01A-11R-1672-07	stage i	t1a	female		
TCGA-KN-8427-01A-11R-2315-07	stage iv	t4	male		
TCGA-BQ-5876-01A-11R-1592-07	stage i	t1a	male		
TCGA-DV-5565-01A-01R-1541-07	stage i	t1a	male		
TCGA-F9-A4JJ-01A-11R-A24Z-07	stage iii	t3a	female		
TCGA-B4-5832-01A-11R-1672-07	stage iii	t3b	male		
TCGA-BP-4347-01A-01R-1289-07	stage iii	t3b	male		
TCGA-G7-A8LD-01A-11R-A36F-07	stage iii	t3a	male		
TCGA-BP-4174-01A-02R-1289-07	stage ii	t2	male		
TCGA-CJ-6032-01A-11R-1672-07	stage ii	t2	female		
TCGA-CZ-4859-01A-02R-1426-07	stage i	t1	female		
TCGA-B8-5550-01A-01R-1541-07	stage iii	t3a	male		
TCGA-A3-3346-01A-01R-1766-07	stage i	t1b	male		
TCGA-AK-3428-01A-02R-1277-07	stage iii	t3b	male		
TCGA-BQ-5893-01A-11R-1592-07	stage iv	t3a	male		
TCGA-A3-3306-01A-01R-0864-07	stage i	t1b	male		
TCGA-CJ-4878-01A-01R-1305-07	stage iii	t3a	female		
TCGA-CW-6093-01A-11R-1672-07	stage i	t1a	male		
TCGA-CJ-4904-01A-02R-1426-07	stage iv	t3a	female		
TCGA-AK-3429-01A-02R-1325-07	stage ii	t2	female		
TCGA-CZ-5463-01A-01R-1503-07	stage ii	t2	male		
TCGA-BP-4338-01A-01R-1289-07	stage i	t1b	male		
TCGA-GL-7966-01A-11R-2204-07	stage iii	t3	female	PNO1	>75 percentile

TCGA-B2-4099-01A-02R-1188-07	stage i	t1a	male		
TCGA-B4-5838-01A-11R-1672-07	stage iv	t3	male		
TCGA-B0-5099-01A-01R-1420-07	stage iii	t3b	female		
TCGA-CJ-4876-01A-01R-1305-07	stage ii	t2b	male		
TCGA-B0-4701-01A-01R-1277-07	stage iv	t3a	female		
TCGA-IA-A40Y-01A-11R-A24Z-07	stage iii	t3a	female		
TCGA-B0-5698-01A-11R-1672-07	stage i	t1b	male		
TCGA-GL-A59T-01A-21R-A28H-07	stage i	t1b	male		
TCGA-BQ-5894-01A-11R-1592-07	stage iv	t3b	male		
TCGA-B8-A7U6-01A-12R-A37O-07	stage i	t1a	female		
TCGA-B0-5098-01A-01R-1420-07	stage i	t1	female		
TCGA-B1-7332-01A-11R-A32Z-07	stage ii	t2a	female		
TCGA-BP-4799-01A-01R-1305-07	stage iii	t3b	male		
TCGA-CJ-4893-01A-01R-1305-07	stage i	t1b	female		
TCGA-CZ-5457-01A-01R-1503-07	stage iii	t3a	male		
TCGA-AK-3451-01A-02R-1188-07	stage ii	t2	male		
TCGA-B4-5377-01A-01R-1503-07	stage iv	t3	female		
TCGA-B0-4698-01A-01R-1503-07	stage iv	t4	male		
TCGA-BP-5006-01A-01R-1334-07	stage i	t1a	male		
TCGA-B8-4620-01A-02R-1325-07	stage iii	t3a	female		
TCGA-2Z-A9JI-01A-11R-A42S-07	stage iii	t3a	male		
TCGA-5P-A9K9-01A-11R-A42S-07	NA	t3a	female		
TCGA-A3-A6NJ-01A-12R-A33J-07	stage i	t1a	female		
TCGA-BP-4160-01A-02R-1289-07	stage iii	t3a	male		
TCGA-BP-5202-01A-02R-1426-07	stage iii	t3a	male		
TCGA-CZ-5468-01A-01R-1503-07	stage iv	t3b	male		
TCGA-B0-4712-01A-01R-1503-07	stage iv	t3a	male		
TCGA-B0-5109-01A-02R-1420-07	stage iii	t3b	male		
TCGA-B8-4151-01A-01R-1188-07	stage iii	t3a	female		

TCGA-KL-8333-01A-11R-2315-07	stage ii	t2	male		
TCGA-BP-5200-01A-01R-1426-07	stage ii	t2	male		
TCGA-CJ-4640-01A-02R-1325-07	stage iii	t3a	male		
TCGA-B0-4852-01A-01R-1503-07	stage ii	t2	female		
TCGA-AK-3458-01A-01R-1503-07	stage i	t1b	male		
TCGA-DW-7838-01A-11R-2139-07	stage i	t1b	male		
TCGA-AK-3444-01A-02R-1325-07	stage i	t1b	female		
TCGA-KO-8410-01A-11R-2315-07	stage i	t1b	female		
TCGA-A4-A5Y1-01A-11R-A28H-07	stage iii	t1b	male		
TCGA-CZ-5453-01A-01R-1503-07	stage ii	t2	male		
TCGA-B0-4690-01A-01R-1277-07	stage iv	t4	male		
TCGA-A4-7996-01A-11R-2204-07	stage i	t1a	female		
TCGA-A3-3382-01A-02R-1325-07	stage i	t1b	male		
TCGA-B0-5693-01A-11R-1541-07	stage i	t1b	female		
TCGA-BP-4164-01A-02R-1325-07	stage iii	t3a	female		
TCGA-BP-5173-01A-01R-1426-07	stage i	t1a	male		
TCGA-SX-A71V-01A-11R-A33Z-07	stage i	t1	male		
TCGA-B2-3924-01A-02R-1325-07	stage i	t1b	male		
TCGA-B0-5104-01A-01R-1420-07	stage i	t1	female		
TCGA-BP-5004-01A-01R-1334-07	stage i	t1a	male		
TCGA-B3-A6W5-01A-12R-A33Z-07	stage i	t1	male		
TCGA-B0-5096-01A-01R-1420-07	stage iii	t3a	female		
TCGA-BP-4354-01A-02R-1289-07	stage iv	t4	male		
TCGA-AL-3466-01A-02R-1351-07	stage iv	t3b	male		
TCGA-DV-5569-01A-01R-1541-07	stage i	t1a	female		
TCGA-A3-3365-01A-01R-0864-07	stage i	t1a	male		
TCGA-AL-A5DJ-01A-11R-A26U-07	stage iii	t3a	female		
TCGA-2Z-A9J8-01A-11R-A42S-07	stage i	t1b	male		
TCGA-EU-5907-01A-11R-1672-07	stage iii	t3a	male		

TCGA-B8-4154-01A-01R-1188-07	stage i	t1a	female		
TCGA-MH-A55W-01A-11R-A26U-07	stage i	t1b	male		
TCGA-V9-A7HT-01A-11R-A33Z-07	stage ii	t2	male		
TCGA-BP-4775-01A-01R-1289-07	stage i	t1a	female		
TCGA-BP-4763-01A-01R-1289-07	stage i	t1a	female		
TCGA-B9-4115-01A-01R-1193-07	stage i	t1a	male		
TCGA-BP-5198-01A-01R-1426-07	stage iii	t3b	male		
TCGA-BP-4770-01A-01R-1503-07	stage iv	t4	female		
TCGA-BP-4985-01A-01R-1334-07	stage iii	t3a	male		
TCGA-BP-4983-01A-01R-1334-07	stage iii	t3a	female		
TCGA-A4-A5XZ-01A-11R-A31O-07	stage ii	t2a	female		
TCGA-A3-3373-01A-02R-1420-07	stage i	t1b	female		
TCGA-AK-3455-01A-01R-0864-07	stage iii	t3b	female		
TCGA-B0-4688-01A-01R-1277-07	stage iv	t4	male		
TCGA-J7-8537-01A-11R-2404-07	stage iii	t3	female		
TCGA-A3-3307-01A-01R-0864-07	stage iii	t3b	male		
TCGA-BP-4343-01A-02R-1289-07	stage iii	t3a	male		
TCGA-HE-A5NF-01A-11R-A26U-07	stage i	t1a	male		
TCGA-G7-6790-01A-11R-1965-07	stage i	t1a	male		
TCGA-AK-3447-01A-01R-1766-07	stage ii	t2	male		
TCGA-CJ-4870-01A-01R-1305-07	stage iii	t3a	female		
TCGA-B0-5084-01A-01R-1334-07	stage iv	t3a	male		
TCGA-B9-5155-01A-01R-1592-07	stage iii	t3a	male		
TCGA-CZ-5461-01A-01R-1503-07	stage iv	t1b	male		
TCGA-CW-5587-01A-01R-1541-07	stage iii	t3b	female		
TCGA-CZ-5462-01A-01R-1503-07	stage iv	t1b	male		
TCGA-B0-5097-01A-01R-1420-07	stage iii	t3b	female		
TCGA-CJ-5679-01A-11R-1541-07	stage iii	t3b	male		
TCGA-A3-3363-01A-01R-0864-07	stage ii	t2	male		

TCGA-CZ-5467-01A-01R-1503-07	stage iii	t3a	female		
TCGA-B4-5843-01A-11R-1672-07	stage i	t1	male		
TCGA-CJ-4891-01A-01R-1305-07	stage iii	t3c	female		
TCGA-CZ-5989-01A-11R-1672-07	stage ii	t2	male		
TCGA-BP-4765-01A-01R-1289-07	stage i	t1a	male		
TCGA-CZ-4854-01A-01R-1305-07	stage i	t1b	male		
TCGA-PJ-A5Z8-01A-11R-A28H-07	stage i	t1a	female		
TCGA-5P-A9KC-01A-11R-A42S-07	NA	t1b	female		
TCGA-B8-A54E-01A-11R-A266-07	stage i	t1b	female		
TCGA-BP-4797-01A-01R-1305-07	stage iii	t3b	male		
TCGA-BP-4959-01A-01R-1334-07	stage i	t1b	male		
TCGA-DW-7834-01A-11R-2139-07	stage i	t1	male		
TCGA-AL-3473-01A-01R-1193-07	stage ii	t2	male		
TCGA-AK-3431-01A-02R-1277-07	stage ii	t2	female		
TCGA-B9-A8YI-01A-21R-A37K-07	stage i	t1b	male		
TCGA-B0-5712-01A-11R-1672-07	stage iv	t2	female		
TCGA-AK-3461-01A-02R-1277-07	stage i	t1a	male		
TCGA-BP-5201-01A-01R-1426-07	stage iv	t3b	male		
TCGA-A3-3335-01A-01R-0864-07	stage ii	t2a	male		
TCGA-B0-5700-01A-11R-1541-07	stage i	t1a	male		
TCGA-A3-A8CQ-01A-11R-A37O-07	stage i	t1a	female		
TCGA-A4-A4ZT-01A-11R-A26U-07	stage i	t1b	female		
TCGA-UN-AAZ9-01A-11R-A38C-07	stage i	t1b	female		
TCGA-BP-4162-01A-02R-1325-07	stage i	t1b	female		
TCGA-CZ-5455-01A-01R-1503-07	stage iv	t3b	male		
TCGA-BP-4170-01A-02R-1289-07	stage i	t1b	female		
TCGA-G7-7501-01A-11R-2204-07	stage iii	t3a	female		
TCGA-BP-4166-01A-02R-1289-07	stage iii	t3a	male		
TCGA-B8-5162-01A-01R-1420-07	stage ii	t2a	male		

TCGA-A4-8098-01A-11R-2404-07	stage i	t1a	male			
TCGA-BP-4756-01A-01R-1289-07	stage i	t1b	female			
TCGA-BQ-5882-01A-11R-1592-07	stage iii	t3b	male			
TCGA-A3-A8OX-01A-11R-A37O-07	stage i	t1a	female			
TCGA-BQ-5884-01A-11R-1592-07	stage i	t1a	female			
TCGA-CJ-4903-01A-01R-1426-07	stage i	t1b	male			
TCGA-CZ-4858-01A-01R-1305-07	stage ii	t2	male			
TCGA-BP-4774-01A-01R-1289-07	stage i	t1a	female			
TCGA-GL-8500-01A-11R-2404-07	stage i	t1b	male			
TCGA-B0-4838-01A-01R-1305-07	stage i	t1b	female			
TCGA-T7-A92I-01A-11R-A37O-07	stage i	t1a	female			
TCGA-G7-6795-01A-11R-1965-07	stage i	t1a	male			
TCGA-B3-4104-01A-02R-1351-07	stage ii	t2	male			
TCGA-KV-A6GD-01A-11R-A31O-07	stage i	t1b	male			
TCGA-B0-4849-01A-01R-1277-07	stage iii	t3a	male			
TCGA-B9-4116-01A-02R-1351-07	NA	t2a	female			
TCGA-KL-8343-01A-11R-2315-07	stage iii	t3	male			
TCGA-2Z-A9JO-01A-11R-A42S-07	stage i	t1b	female			
TCGA-BQ-7048-01A-11R-1965-07	stage iii	t3a	male			
TCGA-CJ-6033-01A-11R-1672-07	stage iv	t3a	female			
TCGA-CJ-5677-01A-11R-1541-07	stage iv	t3a	female			
TCGA-A3-3316-01A-01R-0864-07	stage ii	t2	male			
TCGA-A4-7287-01A-11R-2139-07	stage i	t1	female			
TCGA-3Z-A93Z-01A-11R-A37O-07	stage i	t1a	male			
TCGA-BP-4176-01A-02R-1289-07	stage i	t1b	male			
TCGA-MW-A4EC-01A-11R-A266-07	stage i	t1a	female			
TCGA-B8-5159-01A-01R-1420-07	stage i	t1a	female			
TCGA-AK-3425-01A-02R-1277-07	stage i	t1	male			
TCGA-J7-6720-01A-11R-2139-07	stage i	t1	male			

TCGA-F9-A8NY-01A-11R-A36F-07	stage iv	t4	female		
TCGA-BP-5178-01A-01R-1426-07	stage iv	t3a	male		
TCGA-CZ-5465-01A-01R-1503-07	stage iii	t3b	female		
TCGA-DV-5568-01A-01R-1541-07	stage i	t1a	male		
TCGA-B0-4846-01A-01R-1277-07	stage iv	t3a	male		
TCGA-KM-8441-01A-11R-2315-07	stage i	t1b	female		
TCGA-P4-AAVO-01A-11R-A42S-07	stage i	t1b	male		
TCGA-CW-6097-01A-11R-1672-07	stage iii	t3a	male		
TCGA-A3-3311-01A-02R-1325-07	stage i	t1	male		
TCGA-B0-5110-01A-01R-1420-07	stage i	t1a	female		
TCGA-B0-5703-01A-11R-1541-07	stage i	t1b	male		
TCGA-2Z-A9J2-01A-11R-A38C-07	stage i	t1a	female		
TCGA-UZ-A9PK-01A-11R-A38C-07	stage i	t1	male		
TCGA-CJ-4871-01A-01R-1305-07	stage iv	t3a	male		
TCGA-P4-A5E7-01A-31R-A28H-07	stage i	t1b	female		
TCGA-F9-A97G-01A-11R-A38C-07	stage iii	t3	male		
TCGA-AT-A5NU-01A-11R-A28H-07	stage i	t1a	male		
TCGA-AK-3450-01A-02R-1277-07	stage i	t1a	female		
TCGA-CJ-6027-01A-11R-1672-07	stage i	t1a	male		
TCGA-BP-4777-01A-01R-1289-07	stage i	t1a	male		
TCGA-A3-3317-01A-02R-1325-07	stage ii	t2	male		
TCGA-B8-4148-01A-02R-1325-07	stage i	t1a	female		
TCGA-B8-5551-01A-01R-1541-07	stage i	t1b	female		
TCGA-AK-3460-01A-02R-1277-07	stage i	t1a	male		
TCGA-CJ-4644-01A-02R-1325-07	stage iv	t3a	female		
TCGA-B0-4696-01A-01R-1277-07	stage iii	t3a	male		
TCGA-DV-A4W0-01A-11R-A266-07	stage i	t1b	male		
TCGA-A3-3387-01A-01R-1541-07	stage i	t1a	male		
TCGA-BP-4989-01A-01R-1334-07	stage iii	t3a	male		

TCGA-BQ-7046-01A-11R-1965-07	stage i	t1a	male		
TCGA-B0-4945-01A-01R-1420-07	stage i	t1a	female		
TCGA-BP-4169-01A-02R-1289-07	stage ii	t2	female		
TCGA-BQ-5879-01A-11R-1592-07	stage iii	t3b	female		
TCGA-UZ-A9PN-01A-11R-A38C-07	stage iii	t3	male		
TCGA-B8-A8YJ-01A-13R-A39I-07	stage i	t1b	female		
TCGA-BP-4986-01A-01R-1334-07	stage i	t1a	male		
TCGA-A3-3359-01A-01R-0864-07	stage i	t1a	female		
TCGA-B0-4691-01A-01R-1277-07	stage iv	t2	male		
TCGA-BP-4781-01A-01R-1305-07	stage i	t1a	male		
TCGA-HE-7130-01A-11R-1965-07	stage iii	t3	female		
TCGA-B0-5697-01A-11R-1541-07	stage i	t1a	male		
TCGA-B1-A655-01A-11R-A31O-07	stage i	t1a	female		
TCGA-B3-3926-01A-02R-1351-07	stage i	t1	female		
TCGA-CZ-5984-01A-11R-1672-07	stage i	t1b	male		
TCGA-BP-5174-01A-01R-1426-07	stage i	t1a	female		
TCGA-BP-5183-01A-01R-1426-07	stage iii	t3a	male		
TCGA-GK-A6C7-01A-11R-A33J-07	stage i	t1a	female		
TCGA-B9-A69E-01A-11R-A31O-07	stage iii	t3a	male		
TCGA-DV-5566-01A-01R-1541-07	stage i	t1a	female		
TCGA-BP-4326-01A-01R-1289-07	stage i	t1b	female		
TCGA-B0-5707-01A-11R-1541-07	stage i	t1a	female		
TCGA-BP-4165-01A-02R-1289-07	stage i	t1b	female		
TCGA-DW-7840-01A-11R-A32Z-07	stage i	t1b	male		
TCGA-B8-4622-01A-02R-1277-07	stage iv	t3a	male		
TCGA-IA-A83T-01A-11R-A355-07	NA	tx	male		
TCGA-BP-5000-01A-01R-1334-07	stage i	t1b	male		
TCGA-B0-5120-01A-01R-1420-07	stage i	t1a	female		
TCGA-BQ-5889-01A-11R-1592-07	stage iv	t3b	male		<25 Percentile

TCGA-A4-7732-01A-11R-2139-07	NA	t2a	male		
TCGA-B8-A54F-01A-11R-A266-07	stage i	t1a	female		
TCGA-CJ-4892-01A-01R-1305-07	stage i	t1b	female		
TCGA-BP-5186-01A-01R-1426-07	stage i	t1a	female		
TCGA-BP-4327-01A-01R-1289-07	stage ii	t2	female		
TCGA-B9-4113-01A-01R-1193-07	stage i	t1	male		
TCGA-B0-4824-01A-01R-1277-07	stage i	t1a	female		
TCGA-BP-5175-01A-01R-1426-07	stage i	t1a	male		
TCGA-BP-4776-01A-01R-1289-07	stage i	t1a	male		
TCGA-B0-5710-01A-11R-1672-07	stage i	t1b	male		
TCGA-DZ-6135-01A-11R-1965-07	stage i	t1a	male		
TCGA-BP-4994-01A-01R-1334-07	stage i	t1a	male		
TCGA-CJ-6031-01A-11R-1672-07	stage i	t1b	male		
TCGA-B8-A54G-01A-11R-A266-07	stage i	t1a	male		
TCGA-BQ-7059-01A-11R-1965-07	stage i	t1b	male		
TCGA-5P-A9JV-01A-12R-A42S-07	NA	t1a	male		
TCGA-B2-4101-01A-02R-1277-07	stage ii	t2a	male		
TCGA-B0-4713-01A-01R-1277-07	stage iii	t3b	female		
TCGA-B0-4843-01A-01R-1277-07	stage iii	t3a	male		
TCGA-5P-A9KF-01A-11R-A42S-07	NA	t1a	male		
TCGA-A3-A6NL-01A-11R-A33J-07	stage i	t1b	female		
TCGA-BP-4967-01A-01R-1334-07	stage iii	t3a	male		
TCGA-KM-8477-01A-11R-2315-07	stage i	t1a	male		
TCGA-CJ-4634-01A-02R-1325-07	stage i	t1b	female		
TCGA-BQ-7061-01A-11R-1965-07	stage i	t1b	female		
TCGA-BP-5185-01A-01R-1426-07	stage i	t1a	male		
TCGA-KL-8328-01A-11R-2315-07	#N/A	#N/A	#N/A		
TCGA-B1-A657-01A-11R-A31O-07	stage i	t1b	male		
TCGA-DW-7841-01A-11R-A32Z-07	stage i	t1b	male		

TCGA-MM-A563-01A-11R-A266-07	NA	t3	male			
TCGA-BQ-5888-01A-11R-1592-07	stage i	t1a	female			
TCGA-BP-4353-01A-02R-1289-07	stage i	t1	male			
TCGA-KL-8332-01A-11R-2315-07	stage i	t1b	male			
TCGA-BQ-7056-01A-11R-1965-07	stage iii	t3b	female			
TCGA-BP-5007-01A-01R-1334-07	stage ii	t2	male			
TCGA-KL-8338-01A-11R-2315-07	stage iii	t3a	male			
TCGA-B2-5636-01A-02R-1541-07	stage i	t1a	male			
TCGA-GL-7773-01A-11R-A32Z-07	stage i	t1a	male			
TCGA-BP-4963-01A-01R-1334-07	stage i	t1b	male			
TCGA-B0-5117-01A-01R-1420-07	stage i	t1b	male			
TCGA-BP-4987-01A-01R-1334-07	stage i	t1b	female			
TCGA-B0-4821-01A-01R-1503-07	stage iii	t3b	female			
TCGA-AS-3778-01A-01R-A32Z-07	stage i	t1a	male			
TCGA-AK-3434-01A-02R-1277-07	stage i	t1b	male			
TCGA-KO-8415-01A-11R-2315-07	stage i	t1	female			
TCGA-KM-8440-01A-11R-2315-07	stage iii	t3a	male			
TCGA-B0-5106-01A-01R-1420-07	stage i	t1a	male			
TCGA-BP-5010-01A-02R-1420-07	stage iii	t3a	male			
TCGA-5P-A9K4-01A-11R-A42S-07	NA	t1b	male			
TCGA-IZ-8195-01A-31R-2404-07	stage ii	t2a	male			
TCGA-CZ-5458-01A-01R-1503-07	stage iii	t3a	male			
TCGA-A4-A772-01A-11R-A33Z-07	stage i	t1b	male			
TCGA-UZ-A9PP-01A-11R-A42S-07	stage i	t1a	male			
TCGA-CJ-4887-01A-01R-1305-07	stage iv	t3a	male			
TCGA-B0-5081-01A-01R-1334-07	stage iii	t3b	female			
TCGA-UZ-A9Q1-01A-11R-A42S-07	stage i	t1b	female			
TCGA-BP-5181-01A-01R-1426-07	stage i	t1b	female			
TCGA-CJ-4636-01A-02R-1325-07	stage iii	t3a	male			

TCGA-CJ-5684-01A-11R-1541-07	stage iii	t3a	male		
TCGA-KN-8419-01A-11R-2315-07	stage ii	t2	male		
TCGA-F9-A7Q0-01A-11R-A36F-07	stage i	t1b	female		
TCGA-KL-8329-01A-11R-2315-07	stage i	t1b	female		
TCGA-BQ-7062-01A-11R-1965-07	stage i	t1a	male		
TCGA-CJ-4882-01A-02R-1426-07	stage iii	t3a	male		
TCGA-B0-4817-01A-01R-1277-07	stage iii	t3c	male		
TCGA-CJ-4635-01A-02R-1305-07	stage i	t1b	male		
TCGA-B0-5691-01A-11R-1541-07	stage i	t1a	female		
TCGA-EU-5904-01A-11R-1672-07	stage i	t1	female		
TCGA-KO-8403-01A-11R-2315-07	stage i	t1a	male		
TCGA-CJ-4875-01A-01R-1305-07	stage iv	t3a	male		
TCGA-HE-A5NL-01A-11R-A26U-07	stage i	t1a	male		
TCGA-BP-4999-01A-01R-1334-07	stage i	t1a	male		
TCGA-2Z-A9JP-01A-11R-A42S-07	stage i	t1a	male		
TCGA-UZ-A9PJ-01A-11R-A38C-07	stage i	t1a	male		
TCGA-A3-3370-01A-02R-1420-07	stage i	t1b	female		
TCGA-CZ-5451-01A-01R-1503-07	stage ii	t2	male		
TCGA-B0-5694-01A-11R-1541-07	stage iii	t3a	male		
TCGA-B4-5834-01A-11R-1672-07	stage i	t1	male		
TCGA-BP-4341-01A-01R-1289-07	stage iii	t3a	male		
TCGA-2Z-A9JL-01A-11R-A42S-07	stage i	t1a	male		
TCGA-IA-A83V-01A-11R-A355-07	stage i	t1b	male		
TCGA-BP-4798-01A-01R-1305-07	NA	t3b	male		
TCGA-G6-A5PC-01A-11R-A33J-07	stage iv	t1b	female		
TCGA-BP-4991-01A-01R-1334-07	stage i	t1a	male		
TCGA-EV-5902-01A-11R-1592-07	stage i	t1	male		
TCGA-KO-8404-01A-11R-2315-07	stage iv	t4	male		
TCGA-WN-AB4C-01A-11R-A42S-07	stage i	t1a	female		

TCGA-KL-8323-01A-21R-2315-07	stage iii	t3b	female		
TCGA-Y8-A8S1-01A-11R-A37K-07	stage i	t1a	male		
TCGA-B9-4617-01A-01R-1193-07	NA	t1a	male		
TCGA-KN-8426-01A-11R-2315-07	stage iv	t3a	male		
TCGA-2Z-A9J1-01A-11R-A38C-07	stage i	t1a	male		
TCGA-J7-A8I2-01A-12R-A36F-07	stage i	t1b	male		
TCGA-BP-4973-01A-01R-1334-07	stage iii	t3a	male		
TCGA-AK-3426-01A-02R-1325-07	stage iii	t3a	male		
TCGA-B8-A54D-01A-21R-A266-07	stage iii	t3a	male		
TCGA-A3-A8OW-01A-11R-A37O-07	stage iii	t3a	male		
TCGA-UZ-A9PL-01A-11R-A38C-07	stage ii	t2	male		
TCGA-B2-A4SR-01A-11R-A266-07	stage ii	t2a	male		
TCGA-KN-8418-01A-11R-2315-07	stage ii	t2	female		
TCGA-KM-8438-01A-11R-2315-07	stage ii	t2	female		
TCGA-4A-A93W-01A-11R-A37K-07	stage i	t1a	female		
TCGA-BP-4761-01A-01R-1289-07	stage iii	t3a	male		
TCGA-BP-4992-01A-01R-1334-07	stage i	t1b	male		
TCGA-HE-A5NJ-01A-11R-A26U-07	NA	t1b	male		
TCGA-KL-8340-01A-11R-2315-07	stage ii	t2	male		
TCGA-B9-A8YH-01A-11R-A37K-07	stage i	t1a	male		
TCGA-BQ-7045-01A-31R-1965-07	stage i	t1b	male		
TCGA-SX-A71U-01A-12R-A33Z-07	stage i	t1a	male		
TCGA-Y8-A896-01A-11R-A36F-07	stage iii	t3a	male		
TCGA-G7-A8LE-01A-11R-A36F-07	stage i	t1a	male		
TCGA-A3-3376-01A-02R-1420-07	stage i	t1a	male		
TCGA-A3-3326-01A-01R-0864-07	stage i	t1a	male		
TCGA-KN-8429-01A-11R-2315-07	stage iii	t3a	female		
TCGA-KL-8325-01A-11R-2315-07	stage ii	t2	female		
TCGA-BP-4346-01A-01R-1289-07	stage iii	t3b	male		

TCGA-KO-8408-01A-11R-2315-07	stage iii	t3a	male		
TCGA-B0-5690-01A-11R-1541-07	stage i	t1b	female		
TCGA-BP-4790-01A-01R-1305-07	stage i	t1a	male		
TCGA-B3-3925-01A-02R-1351-07	stage iii	t3a	male		
TCGA-B0-4697-01A-01R-1277-07	stage iv	t3b	female		
TCGA-B0-4811-01A-01R-1503-07	stage iii	t3a	male		
TCGA-KN-8430-01A-11R-2315-07	stage i	t1b	male		
TCGA-5P-A9JY-01A-11R-A42S-07	NA	t2	male		
TCGA-BP-5001-01A-01R-1334-07	stage i	t1b	female		
TCGA-KL-8337-01A-11R-2315-07	stage ii	t2	male		
TCGA-HE-A5NI-01A-11R-A26U-07	stage i	t1a	male		
TCGA-KN-8434-01A-11R-2315-07	stage ii	t2	female		
TCGA-KN-8423-01A-11R-2315-07	stage i	t1b	male		
TCGA-CZ-4853-01A-01R-1426-07	stage i	t1a	male		
TCGA-A3-3349-01A-01R-1188-07	stage i	t1b	female		
TCGA-KN-8436-01A-11R-2315-07	stage ii	t2	male		
TCGA-A3-3367-01A-02R-1420-07	stage i	t1b	male		
TCGA-5P-A9KA-01A-11R-A42S-07	NA	t1a	male		
TCGA-A3-3351-01A-02R-1325-07	stage ii	t2a	male		
TCGA-2Z-A9J6-01A-11R-A38C-07	stage i	t1a	male		
TCGA-P4-A5E6-01A-11R-A28H-07	stage iii	t1b	male		
TCGA-KL-8335-01A-11R-2315-07	stage iii	t3a	male		
TCGA-UZ-A9PZ-01A-11R-A42S-07	stage iii	t3a	male		
TCGA-KO-8417-01A-11R-2315-07	stage i	t1	female		
TCGA-B0-4706-01A-01R-1503-07	stage iii	t3a	male		
TCGA-KN-8433-01A-11R-2315-07	stage iii	t3a	female		
TCGA-A4-A57E-01A-11R-A26U-07	stage iv	t2a	male		
TCGA-KO-8414-01A-11R-2315-07	stage ii	t2	female		
TCGA-BP-5191-01A-01R-1426-07	stage iii	t3a	male		

TCGA-B0-5699-01A-11R-1541-07	stage i	t1	male		
TCGA-B0-5088-01A-01R-1334-07	stage i	t1b	male		
TCGA-2Z-A9JT-01A-11R-A42S-07	stage i	t1a	male		
TCGA-MM-A564-01A-11R-A266-07	stage ii	t2a	male		
TCGA-BQ-5885-01A-11R-1592-07	stage iii	t3	male		
TCGA-KL-8331-01A-11R-2315-07	stage ii	t2	female		
TCGA-Y8-A8RY-01A-11R-A37K-07	stage i	t1b	male		
TCGA-A3-3383-01A-02R-1325-07	stage i	t1	male		
TCGA-BP-4351-01A-01R-1289-07	stage iii	t3a	female		
TCGA-CJ-4638-01A-02R-1325-07	stage iv	t3a	female		
TCGA-BP-4977-01A-01R-1334-07	stage i	t1b	male		
TCGA-BP-4982-01A-01R-1334-07	stage i	t1b	male		
TCGA-A4-A6HP-01A-11R-A31O-07	stage i	t1a	male		
TCGA-CJ-4873-01A-01R-1305-07	stage iii	t3a	female		
TCGA-BP-4971-01A-01R-1334-07	stage iii	t3a	male		
TCGA-UZ-A9PQ-01A-11R-A42S-07	stage iii	t2	male		
TCGA-BP-4330-01A-01R-1289-07	stage iii	t3a	female		
TCGA-B8-A54K-01A-11R-A33J-07	stage i	t1a	male		
TCGA-B0-5100-01A-01R-1420-07	stage iii	t3a	male		
TCGA-AL-3471-01A-02R-1351-07	stage i	t1b	male		
TCGA-SX-A7SS-01A-11R-A36F-07	stage i	t1	male		
TCGA-MH-A55Z-01A-11R-A26U-07	stage i	t1b	male		
TCGA-KO-8407-01A-11R-2315-07	stage ii	t2	male		
TCGA-KO-8416-01A-11R-2315-07	stage iii	t3a	male		
TCGA-A3-3323-01A-02R-1325-07	stage i	t1b	male		
TCGA-KO-8406-01A-11R-2315-07	stage i	t1	female		
TCGA-Y8-A895-01A-11R-A36F-07	stage i	t1b	male		
TCGA-B0-4847-01A-01R-1277-07	stage iv	t3a	male		
TCGA-F9-A7VF-01A-11R-A33Z-07	stage i	t1a	female		

TCGA-B0-4822-01A-01R-1277-07	stage ii	t2	male		
TCGA-G7-6797-01A-11R-1965-07	stage iii	t1a	male		
TCGA-B1-A654-01A-11R-A31O-07	stage i	t1a	female		
TCGA-B0-4714-01A-01R-1277-07	stage iv	t3b	male		
TCGA-KL-8342-01A-11R-2315-07	stage ii	t2b	female		
TCGA-CW-5585-01A-01R-1541-07	stage iv	t3b	male		
TCGA-B0-4814-01A-01R-1277-07	stage iv	t4	male		
TCGA-KN-8432-01A-11R-2315-07	stage ii	t2b	female		
TCGA-SX-A7SR-01A-12R-A36F-07	stage ii	t2a	male		
TCGA-MH-A856-01A-11R-A355-07	stage i	t1a	male		
TCGA-IA-A83W-01A-11R-A355-07	stage i	t1	male		
TCGA-B1-A656-01A-11R-A31O-07	stage i	t1a	male		
TCGA-2Z-A9JQ-01A-11R-A42S-07	stage i	t1a	male		
TCGA-P4-AAVL-01A-11R-A42S-07	stage iii	t3b	male		
TCGA-KN-8431-01A-11R-2315-07	stage ii	t2	female		
TCGA-KL-8346-01A-11R-2315-07	stage ii	t2b	male		
TCGA-P4-A5ED-01A-11R-A28H-07	stage i	t1a	male		
TCGA-AL-3472-01A-01R-1193-07	NA	t2	male		
TCGA-KO-8409-01A-11R-2315-07	stage ii	t2b	male		
TCGA-KL-8330-01A-11R-2315-07	stage ii	t2b	female		
TCGA-UZ-A9Q0-01A-12R-A42S-07	stage i	t1	male		
TCGA-MH-A562-01A-11R-A26U-07	stage i	t1a	male		
TCGA-B0-5085-01A-01R-1334-07	stage iii	t3a	female		
TCGA-KL-8324-01A-11R-2315-07	stage ii	t2	female		
TCGA-WN-A9G9-01A-12R-A37K-07	stage i	t1b	male		
TCGA-BP-4988-01A-01R-1334-07	stage i	t1a	male		
TCGA-DW-5560-01A-01R-1592-07	stage i	t1b	female		
TCGA-5P-A9K2-01A-11R-A42S-07	NA	t1b	male		
TCGA-IZ-A6M9-01A-11R-A31O-07	stage i	t1a	male		

TCGA-G7-6789-01A-11R-1965-07	stage iv	t3a	female			
TCGA-SX-A71R-01A-12R-A33Z-07	stage i	t1a	male			
TCGA-CJ-5681-01A-11R-1541-07	stage iv	t3a	female			
TCGA-BP-4334-01A-01R-1289-07	stage iii	t3a	male			
TCGA-KM-8476-01A-11R-2315-07	stage i	t1	male			
TCGA-B1-A47O-01A-11R-A24Z-07	stage i	t1b	female			
TCGA-G7-6792-01A-21R-1965-07	stage i	t1b	male			
TCGA-G7-A8LB-01A-11R-A36F-07	stage iv	t2a	male			
TCGA-B1-A47N-01A-11R-A24Z-07	stage i	t1a	male			
TCGA-KL-8327-01A-11R-2315-07	stage i	t1b	female			
TCGA-B0-5702-01A-11R-1541-07	stage i	t1b	male			
TCGA-CZ-5989-01A-11R-1672-07	stage ii	t2	male			
TCGA-B4-5832-01A-11R-1672-07	stage iii	t3b	male			
TCGA-CW-6097-01A-11R-1672-07	stage iii	t3a	male			
TCGA-B0-5710-01A-11R-1672-07	stage i	t1b	male			
TCGA-CJ-4899-01A-01R-1334-07	stage i	t1b	male			
TCGA-BP-4799-01A-01R-1305-07	stage iii	t3b	male			
TCGA-BP-4354-01A-02R-1289-07	stage iv	t4	male			
TCGA-BP-5187-01A-01R-1426-07	stage i	t1a	male			
TCGA-BP-4353-01A-02R-1289-07	stage i	t1	male			
TCGA-B8-4620-01A-02R-1325-07	stage iii	t3a	female			
TCGA-B0-4718-01A-01R-1277-07	stage iii	t3a	male			
TCGA-CZ-5454-01A-01R-1503-07	stage iv	t2	male			
TCGA-B0-4703-01A-01R-1277-07	stage iv	t3a	male			
TCGA-B0-5113-01A-01R-1420-07	stage iii	t3a	female			
TCGA-CJ-4912-01A-01R-1426-07	stage ii	t2	male			
TCGA-CZ-4860-01A-01R-1305-07	stage iv	t4	male			
TCGA-BP-4998-01A-01R-1334-07	stage i	t1a	male			
TCGA-EU-5904-01A-11R-1672-07	stage i	t1	female	PTPN12	>75 percentile	

TCGA-CW-6090-01A-11R-1672-07	stage i	t1b	male		
TCGA-B0-5812-01A-11R-1672-07	stage i	t1b	male		
TCGA-CJ-4908-01A-01R-1426-07	stage i	t1a	male		
TCGA-CW-6093-01A-11R-1672-07	stage i	t1a	male		
TCGA-BP-4763-01A-01R-1289-07	stage i	t1a	female		
TCGA-AK-3461-01A-02R-1277-07	stage i	t1a	male		
TCGA-BP-4983-01A-01R-1334-07	stage iii	t3a	female		
TCGA-BP-4338-01A-01R-1289-07	stage i	t1b	male		
TCGA-A3-3320-01A-02R-1325-07	stage i	t1b	female		
TCGA-B0-5095-01A-01R-1420-07	stage iii	t3a	male		
TCGA-DV-5574-01A-01R-1541-07	stage i	t1a	male		
TCGA-B0-4852-01A-01R-1503-07	stage ii	t2	female		
TCGA-B0-5690-01A-11R-1541-07	stage i	t1b	female		
TCGA-BP-4159-01A-02R-1289-07	stage i	t1b	male		
TCGA-BP-4974-01A-01R-1334-07	stage iv	t3a	male		
TCGA-BP-4991-01A-01R-1334-07	stage i	t1a	male		
TCGA-BP-4781-01A-01R-1305-07	stage i	t1a	male		
TCGA-B0-5108-01A-01R-1420-07	stage iii	t3a	male		
TCGA-A3-3365-01A-01R-0864-07	stage i	t1a	male		
TCGA-BP-4803-01A-01R-1305-07	stage iii	t3a	male		
TCGA-BP-4332-01A-01R-1289-07	stage iii	t3a	male		
TCGA-BP-4343-01A-02R-1289-07	stage iii	t3a	male		
TCGA-B0-5080-01A-01R-1503-07	stage iv	t3a	male		
TCGA-BP-4770-01A-01R-1503-07	stage iv	t4	female		
TCGA-BP-4344-01A-01R-1289-07	stage i	t1a	female		
TCGA-BP-4985-01A-01R-1334-07	stage iii	t3a	male		
TCGA-AK-3431-01A-02R-1277-07	stage ii	t2	female		
TCGA-CZ-4858-01A-01R-1305-07	stage ii	t2	male		
TCGA-A3-3376-01A-02R-1420-07	stage i	t1a	male		

TCGA-CZ-4857-01A-01R-1305-07	stage iv	t3a	male		
TCGA-B0-5110-01A-01R-1420-07	stage i	t1a	female		
TCGA-CJ-4643-01A-02R-1325-07	stage ii	t2b	female		
TCGA-CJ-4639-01A-02R-1325-07	stage ii	t2	female		
TCGA-BP-4986-01A-01R-1334-07	stage i	t1a	male		
TCGA-KN-8427-01A-11R-2315-07	stage iv	t4	male		
TCGA-CZ-5982-01A-11R-1672-07	stage i	t1a	female		
TCGA-CZ-5467-01A-01R-1503-07	stage iii	t3a	female		
TCGA-CZ-5988-01A-11R-1672-07	stage i	t1b	male		
TCGA-B0-5098-01A-01R-1420-07	stage i	t1	female		
TCGA-CJ-4872-01A-01R-1305-07	stage i	t1b	male		
TCGA-B2-5633-01A-01R-1541-07	stage i	t1b	male		
TCGA-BP-4158-01A-02R-1289-07	stage i	t1b	male		
TCGA-A3-3349-01A-01R-1188-07	stage i	t1b	female		
TCGA-B2-A4SR-01A-11R-A266-07	stage ii	t2a	male		
TCGA-B0-4690-01A-01R-1277-07	stage iv	t4	male		
TCGA-CJ-4636-01A-02R-1325-07	stage iii	t3a	male		
TCGA-A3-3382-01A-02R-1325-07	stage i	t1b	male		
TCGA-CJ-5671-01A-11R-1541-07	stage i	t1a	male		
TCGA-CJ-6028-01A-11R-1672-07	stage iv	t3a	male		
TCGA-BP-4961-01A-01R-1334-07	stage i	t1a	male		
TCGA-BP-4993-01A-02R-1420-07	stage i	t1a	male		
TCGA-B0-5120-01A-01R-1420-07	stage i	t1a	female		
TCGA-CZ-4861-01A-01R-1305-07	stage ii	t2	male		
TCGA-B0-5116-01A-02R-1420-07	stage iii	t3b	male		
TCGA-BP-4789-01A-01R-1305-07	stage i	t1a	male		
TCGA-B4-5843-01A-11R-1672-07	stage i	t1	male		
TCGA-MM-A564-01A-11R-A266-07	stage ii	t2a	male		
TCGA-B0-5402-01A-01R-1503-07	stage iv	t4	male		

TCGA-CJ-4920-01A-01R-1426-07	stage i	t1b	female		
TCGA-BP-4329-01A-02R-1289-07	stage iii	t3a	male		
TCGA-CZ-5468-01A-01R-1503-07	stage iv	t3b	male		
TCGA-BP-4973-01A-01R-1334-07	stage iii	t3a	male		
TCGA-BP-4999-01A-01R-1334-07	stage i	t1a	male		
TCGA-BP-5177-01A-01R-1426-07	stage i	t1a	female		
TCGA-BP-4355-01A-01R-1289-07	stage iii	t3a	female		
TCGA-CJ-4918-01A-01R-1426-07	stage iv	t3a	male		
TCGA-BP-4967-01A-01R-1334-07	stage iii	t3a	male		
TCGA-CJ-4875-01A-01R-1305-07	stage iv	t3a	male		
TCGA-CZ-5460-01A-01R-1503-07	stage iv	t3b	male		
TCGA-B0-5693-01A-11R-1541-07	stage i	t1b	female		
TCGA-BP-4335-01A-01R-1289-07	stage iv	t3a	female		
TCGA-B4-5844-01A-11R-1672-07	stage ii	t2	female		
TCGA-A3-3316-01A-01R-0864-07	stage ii	t2	male		
TCGA-DV-5565-01A-01R-1541-07	stage i	t1a	male		
TCGA-A3-3380-01A-01R-0864-07	stage i	t1	male		
TCGA-BP-4759-01A-01R-1289-07	stage i	t1a	male		
TCGA-B2-4102-01A-02R-1325-07	stage i	t1b	male		
TCGA-B0-4849-01A-01R-1277-07	stage iii	t3a	male		
TCGA-B4-5834-01A-11R-1672-07	stage i	t1	male		
TCGA-BP-4987-01A-01R-1334-07	stage i	t1b	female		
TCGA-GL-7966-01A-11R-2204-07	stage iii	t3	female		
TCGA-CW-5580-01A-01R-1672-07	stage iv	t3a	female		
TCGA-AK-3428-01A-02R-1277-07	stage iii	t3b	male		
TCGA-BP-4790-01A-01R-1305-07	stage i	t1a	male		
TCGA-CJ-4904-01A-02R-1426-07	stage iv	t3a	female		
TCGA-B8-5162-01A-01R-1420-07	stage ii	t2a	male		
TCGA-B8-A8YJ-01A-13R-A39I-07	stage i	t1b	female		

TCGA-A3-3387-01A-01R-1541-07	stage i	t1a	male		
TCGA-CJ-4878-01A-01R-1305-07	stage iii	t3a	female		
TCGA-BP-4170-01A-02R-1289-07	stage i	t1b	female		
TCGA-A3-3372-01A-02R-1325-07	stage iii	t3	male		
TCGA-CJ-5686-01A-11R-1672-07	stage i	t1b	female		
TCGA-BP-4163-01A-02R-1325-07	stage iii	t3a	female		
TCGA-BP-4349-01A-01R-1289-07	stage i	t1a	female		
TCGA-CJ-6030-01A-11R-1672-07	stage i	t1a	male		
TCGA-GK-A6C7-01A-11R-A33J-07	stage i	t1a	female		
TCGA-BP-5007-01A-01R-1334-07	stage ii	t2	male		
TCGA-BP-4169-01A-02R-1289-07	stage ii	t2	female		
TCGA-B0-5711-01A-11R-1672-07	stage iii	t3b	male		
TCGA-CZ-5457-01A-01R-1503-07	stage iii	t3a	male		
TCGA-BP-4982-01A-01R-1334-07	stage i	t1b	male		
TCGA-B0-5694-01A-11R-1541-07	stage iii	t3a	male		
TCGA-B8-5549-01A-01R-1541-07	stage i	t1b	male		
TCGA-BP-4345-01A-01R-1289-07	stage iii	t3b	male		
TCGA-CJ-4635-01A-02R-1305-07	stage i	t1b	male		
TCGA-B8-A54F-01A-11R-A266-07	stage i	t1a	female		
TCGA-BP-4176-01A-02R-1289-07	stage i	t1b	male		
TCGA-A3-3362-01A-02R-1325-07	stage i	t1a	female		
TCGA-CJ-5683-01A-11R-1541-07	stage i	t1b	male		
TCGA-A3-3331-01A-02R-1325-07	stage i	t1	female		
TCGA-BP-4326-01A-01R-1289-07	stage i	t1b	female		
TCGA-AK-3460-01A-02R-1277-07	stage i	t1a	male		
TCGA-B0-4816-01A-01R-1503-07	stage ii	t2	male		
TCGA-B4-5836-01A-11R-1672-07	stage i	t1b	female		
TCGA-CJ-4634-01A-02R-1325-07	stage i	t1b	female		
TCGA-B2-4101-01A-02R-1277-07	stage ii	t2a	male		

TCGA-BP-4352-01A-01R-1289-07	stage iv	t3b	female		
TCGA-B8-4622-01A-02R-1277-07	stage iv	t3a	male		
TCGA-A3-3351-01A-02R-1325-07	stage ii	t2a	male		
TCGA-CJ-6033-01A-11R-1672-07	stage iv	t3a	female		
TCGA-B0-5106-01A-01R-1420-07	stage i	t1a	male		
TCGA-BP-4965-01A-01R-1334-07	stage i	t1a	male		
TCGA-B0-5097-01A-01R-1420-07	stage iii	t3b	female		
TCGA-BP-4162-01A-02R-1325-07	stage i	t1b	female		
TCGA-BP-5194-01A-02R-1426-07	stage i	t1a	male		
TCGA-BP-4963-01A-01R-1334-07	stage i	t1b	male		
TCGA-BP-5009-01A-01R-1334-07	stage i	t1b	male		
TCGA-BP-4325-01A-02R-1289-07	stage i	t1b	female		
TCGA-IZ-8196-01A-11R-2404-07	stage i	t1a	male		
TCGA-CJ-4901-01A-01R-1426-07	stage iii	t3b	male		
TCGA-BP-4988-01A-01R-1334-07	stage i	t1a	male		
TCGA-BQ-5879-01A-11R-1592-07	stage iii	t3b	female		
TCGA-BP-5200-01A-01R-1426-07	stage ii	t2	male		
TCGA-A3-3323-01A-02R-1325-07	stage i	t1b	male		
TCGA-DW-7842-01A-11R-A32Z-07	stage i	t1a	male		
TCGA-B0-4945-01A-01R-1420-07	stage i	t1a	female		
TCGA-B0-5077-01A-01R-1334-07	stage i	t1a	male		
TCGA-B8-A7U6-01A-12R-A37O-07	stage i	t1a	female		
TCGA-B9-4113-01A-01R-1193-07	stage i	t1	male		
TCGA-CZ-5455-01A-01R-1503-07	stage iv	t3b	male		
TCGA-A3-3324-01A-02R-1325-07	stage i	t1b	male		
TCGA-CZ-4865-01A-02R-1503-07	stage i	t1a	female		
TCGA-A3-3325-01A-01R-0864-07	stage i	t1a	male		
TCGA-EU-5906-01A-11R-1672-07	stage i	t1b	male		
TCGA-CJ-4868-01A-01R-1305-07	stage iv	t3a	male		

TCGA-A3-3347-01A-02R-1325-07	stage iii	t1b	female			
TCGA-BP-4776-01A-01R-1289-07	stage i	t1a	male			
TCGA-A3-3317-01A-02R-1325-07	stage ii	t2	male			
TCGA-B8-5165-01A-01R-1420-07	stage i	t1a	male			
TCGA-BP-5195-01A-02R-1426-07	stage i	t1a	male			
TCGA-BP-4989-01A-01R-1334-07	stage iii	t3a	male			
TCGA-B0-4841-01A-01R-1277-07	stage iv	t2	male			
TCGA-CW-5583-01A-02R-1541-07	stage i	t1a	female			
TCGA-B0-4706-01A-01R-1503-07	stage iii	t3a	male			
TCGA-BP-4798-01A-01R-1305-07	NA	t3b	male			
TCGA-BP-4970-01A-01R-1334-07	stage iii	t1a	male			
TCGA-B0-5094-01A-01R-1420-07	stage iv	t3b	male			
TCGA-BP-5170-01A-01R-1426-07	stage i	t1a	male			
TCGA-CZ-5461-01A-01R-1503-07	stage iv	t1b	male			
TCGA-A3-A6NL-01A-11R-A33J-07	stage i	t1b	female			
TCGA-DV-5569-01A-01R-1541-07	stage i	t1a	female			
TCGA-CJ-4905-01A-02R-1426-07	stage i	t1a	female			
TCGA-B0-5121-01A-02R-1420-07	stage i	t1b	male			
TCGA-BP-5196-01A-01R-1426-07	stage i	t1a	male			
TCGA-CJ-4907-01A-01R-1426-07	stage iii	t3b	male			
TCGA-CJ-4897-01A-03R-1426-07	stage iii	t3a	female			
TCGA-CJ-4902-01A-01R-1426-07	stage iii	t3a	male			
TCGA-BP-4797-01A-01R-1305-07	stage iii	t3b	male			
TCGA-BP-5173-01A-01R-1426-07	stage i	t1a	male			
TCGA-B8-5550-01A-01R-1541-07	stage iii	t3a	male			
TCGA-B0-4688-01A-01R-1277-07	stage iv	t4	male			
TCGA-B0-4823-01A-02R-1420-07	stage i	t1a	male			
TCGA-CJ-4885-01A-01R-1305-07	stage iv	t3a	male			
TCGA-B4-5378-01A-01R-1503-07	stage i	t1	male			

TCGA-BP-5168-01A-01R-1420-07	stage i	t1a	male		
TCGA-A3-3322-01A-02R-1325-07	stage i	t1a	male		
TCGA-CJ-5684-01A-11R-1541-07	stage iii	t3a	male		
TCGA-G6-A8L6-01A-11R-A37O-07	stage iv	t2a	male		
TCGA-BP-4964-01A-01R-1334-07	stage i	t1a	female		
TCGA-B0-4698-01A-01R-1503-07	stage iv	t4	male		
TCGA-A3-3359-01A-01R-0864-07	stage i	t1a	female		
TCGA-B8-5163-01A-01R-1420-07	stage iii	t3a	female		
TCGA-BQ-5894-01A-11R-1592-07	stage iv	t3b	male		
TCGA-SX-A71S-01A-11R-A33Z-07	stage i	t1	female		
TCGA-B0-4818-01A-01R-1503-07	stage ii	t2	female		
TCGA-BP-4784-01A-01R-1305-07	stage i	t1a	female		
TCGA-BP-4972-01A-01R-1334-07	stage iii	t3a	female		
TCGA-B0-5115-01A-01R-1420-07	stage iv	t2	male		
TCGA-CZ-4854-01A-01R-1305-07	stage i	t1b	male		
TCGA-BP-4330-01A-01R-1289-07	stage iii	t3a	female		
TCGA-G7-7502-01A-11R-2204-07	stage i	t1b	male		
TCGA-AK-3454-01A-02R-1277-07	stage i	t1b	male		
TCGA-CW-6088-01A-11R-1672-07	stage i	t1b	male		
TCGA-BP-4768-01A-01R-1289-07	stage i	t1a	female		
TCGA-CJ-5679-01A-11R-1541-07	stage iii	t3b	male		
TCGA-CJ-4882-01A-02R-1426-07	stage iii	t3a	male		
TCGA-B0-5701-01A-11R-1541-07	stage iii	t3b	male		
TCGA-CJ-4895-01A-01R-1305-07	stage iv	t3a	male		
TCGA-B8-A54H-01A-11R-A33J-07	stage ii	t2a	female		
TCGA-CZ-5465-01A-01R-1503-07	stage iii	t3b	female		
TCGA-CJ-4903-01A-01R-1426-07	stage i	t1b	male		
TCGA-CZ-4866-01A-01R-1503-07	stage i	t1	female		
TCGA-F9-A97G-01A-11R-A38C-07	stage iii	t3	male		<25 Percentile

TCGA-A4-7288-01A-11R-A32Z-07	NA	t1a	female			
TCGA-BQ-7051-01A-12R-1965-07	stage ii	t2	male			
TCGA-G7-A8LC-01A-11R-A36F-07	stage i	t1a	male			
TCGA-B1-A655-01A-11R-A31O-07	stage i	t1a	female			
TCGA-CJ-4638-01A-02R-1325-07	stage iv	t3a	female			
TCGA-UZ-A9Q1-01A-11R-A42S-07	stage i	t1b	female			
TCGA-G7-6795-01A-11R-1965-07	stage i	t1a	male			
TCGA-2Z-A9JD-01A-11R-A42S-07	stage i	t1a	male			
TCGA-G7-6793-01A-11R-1965-07	stage iv	t3a	female			
TCGA-2Z-A9JQ-01A-11R-A42S-07	stage i	t1a	male			
TCGA-BP-5190-01A-01R-1426-07	stage i	t1a	male			
TCGA-B9-5156-01A-01R-1592-07	NA	t1a	male			
TCGA-AL-3473-01A-01R-1193-07	stage ii	t2	male			
TCGA-B9-A5W8-01A-11R-A28H-07	stage ii	t2b	male			
TCGA-5P-A9K0-01A-11R-A42S-07	NA	t1a	male			
TCGA-IZ-8195-01A-31R-2404-07	stage ii	t2a	male			
TCGA-2Z-A9JI-01A-11R-A42S-07	stage iii	t3a	male			
TCGA-BQ-5891-01A-11R-1592-07	stage iii	t3b	female			
TCGA-AK-3427-01A-01R-0864-07	stage i	t1a	male			
TCGA-BP-5169-01A-01R-1426-07	stage i	t1b	male			
TCGA-KV-A6GE-01A-11R-A31O-07	stage i	t1a	male			
TCGA-CZ-4859-01A-02R-1426-07	stage i	t1	female			
TCGA-B0-5707-01A-11R-1541-07	stage i	t1a	female			
TCGA-BQ-5875-01A-11R-1592-07	stage iii	t3a	female			
TCGA-B8-A54E-01A-11R-A266-07	stage i	t1b	female			
TCGA-MH-A561-01A-11R-A26U-07	stage i	t1a	male			
TCGA-BQ-7060-01A-11R-1965-07	stage i	t1a	male			
TCGA-P4-AAVL-01A-11R-A42S-07	stage iii	t3b	male			
TCGA-A4-8516-01A-11R-2404-07	stage iii	t3a	male			

TCGA-P4-AAVO-01A-11R-A42S-07	stage i	t1b	male		
TCGA-5P-A9JY-01A-11R-A42S-07	NA	t2	male		
TCGA-KV-A6GD-01A-11R-A31O-07	stage i	t1b	male		
TCGA-BP-5185-01A-01R-1426-07	stage i	t1a	male		
TCGA-B9-7268-01A-11R-A32Z-07	stage ii	t2	male		
TCGA-A4-7585-01A-11R-2139-07	stage iii	t3a	male		
TCGA-Y8-A897-01A-11R-A36F-07	stage i	t1a	female		
TCGA-SX-A7SP-01A-11R-A355-07	stage i	t1b	female		
TCGA-SX-A71V-01A-11R-A33Z-07	stage i	t1	male		
TCGA-2Z-A9JL-01A-11R-A42S-07	stage i	t1a	male		
TCGA-BQ-5890-01A-11R-1592-07	stage iii	t3a	male		
TCGA-A4-8517-01A-11R-2404-07	stage i	t1a	male		
TCGA-F9-A7Q0-01A-11R-A36F-07	stage i	t1b	female		
TCGA-BQ-5883-01A-11R-1592-07	stage i	t1a	female		
TCGA-MH-A855-01A-11R-A355-07	stage ii	t2b	female		
TCGA-UZ-A9PP-01A-11R-A42S-07	stage i	t1a	male		
TCGA-EV-5902-01A-11R-1592-07	stage i	t1	male		
TCGA-P4-AAVK-01A-11R-A42S-07	stage iii	t3a	male		
TCGA-5P-A9KC-01A-11R-A42S-07	NA	t1b	female		
TCGA-BQ-5888-01A-11R-1592-07	stage i	t1a	female		
TCGA-UZ-A9PO-01A-11R-A38C-07	stage i	t1a	male		
TCGA-BQ-7062-01A-11R-1965-07	stage i	t1a	male		
TCGA-BQ-7048-01A-11R-1965-07	stage iii	t3a	male		
TCGA-G6-A8L7-01A-11R-A37O-07	stage i	t1b	female		
TCGA-AT-A5NU-01A-11R-A28H-07	stage i	t1a	male		
TCGA-BQ-5887-01A-11R-1965-07	stage iii	t3a	male		
TCGA-KO-8413-01A-11R-2315-07	stage i	t1	male		
TCGA-5P-A9KE-01A-11R-A42S-07	NA	t1b	male		
TCGA-A4-8310-01A-11R-2404-07	stage iii	t3a	male		

TCGA-B3-4104-01A-02R-1351-07	stage ii	t2	male		
TCGA-G7-6790-01A-11R-1965-07	stage i	t1a	male		
TCGA-2Z-A9JN-01A-21R-A42S-07	stage i	t1a	female		
TCGA-A4-A57E-01A-11R-A26U-07	stage iv	t2a	male		
TCGA-B0-4707-01A-01R-1277-07	stage iii	t3a	male		
TCGA-G6-A5PC-01A-11R-A33J-07	stage iv	t1b	female		
TCGA-BQ-5886-01A-11R-1592-07	stage iii	t3a	male		
TCGA-BQ-5882-01A-11R-1592-07	stage iii	t3b	male		
TCGA-KN-8429-01A-11R-2315-07	stage iii	t3a	female		
TCGA-BQ-7053-01A-11R-1965-07	stage iii	t3	female		
TCGA-DZ-6134-01A-11R-1965-07	stage i	t1a	male		
TCGA-BQ-7046-01A-11R-1965-07	stage i	t1a	male		
TCGA-B9-A5W9-01A-11R-A28H-07	stage i	t1b	male		
TCGA-DW-7841-01A-11R-A32Z-07	stage i	t1b	male		
TCGA-BQ-7055-01A-11R-1965-07	stage i	t1a	male		
TCGA-IZ-A6M9-01A-11R-A31O-07	stage i	t1a	male		
TCGA-T7-A92I-01A-11R-A37O-07	stage i	t1a	female		
TCGA-A4-A5Y0-01A-11R-A31O-07	stage i	t1b	female		
TCGA-B0-5083-01A-02R-1420-07	stage i	t1a	male		
TCGA-CJ-5681-01A-11R-1541-07	stage iv	t3a	female		
TCGA-P4-A5EA-01A-11R-A28H-07	stage iii	t3a	female		
TCGA-B1-7332-01A-11R-A32Z-07	stage ii	t2a	female		
TCGA-2Z-A9J6-01A-11R-A38C-07	stage i	t1a	male		
TCGA-UZ-A9Q0-01A-12R-A42S-07	stage i	t1	male		
TCGA-WN-AB4C-01A-11R-A42S-07	stage i	t1a	female		
TCGA-BP-5191-01A-01R-1426-07	stage iii	t3a	male		
TCGA-HE-A5NF-01A-11R-A26U-07	stage i	t1a	male		
TCGA-A4-A5Y1-01A-11R-A28H-07	stage iii	t1b	male		
TCGA-BQ-7058-01A-11R-1965-07	stage iii	t3	male		

TCGA-BP-4756-01A-01R-1289-07	stage i	t1b	female		
TCGA-SX-A7SL-01A-11R-A355-07	stage i	t1a	male		
TCGA-WN-A9G9-01A-12R-A37K-07	stage i	t1b	male		
TCGA-B8-5546-01A-01R-1541-07	stage i	t1b	female		
TCGA-IA-A83V-01A-11R-A355-07	stage i	t1b	male		
TCGA-BQ-7050-01A-11R-1965-07	stage i	t1a	female		
TCGA-IA-A40X-01A-11R-A24Z-07	stage i	t1a	female		
TCGA-SX-A7SO-01A-11R-A355-07	stage i	t1b	male		
TCGA-5P-A9JW-01A-11R-A42S-07	NA	t1a	male		
TCGA-2Z-A9J1-01A-11R-A38C-07	stage i	t1a	male		
TCGA-KN-8430-01A-11R-2315-07	stage i	t1b	male		
TCGA-B0-4842-01A-02R-1420-07	stage iii	t3a	female		
TCGA-KN-8423-01A-11R-2315-07	stage i	t1b	male		
TCGA-B9-A69E-01A-11R-A31O-07	stage iii	t3a	male		
TCGA-2Z-A9JP-01A-11R-A42S-07	stage i	t1a	male		
TCGA-P4-A5ED-01A-11R-A28H-07	stage i	t1a	male		
TCGA-BP-5175-01A-01R-1426-07	stage i	t1a	male		
TCGA-SX-A71R-01A-12R-A33Z-07	stage i	t1a	male		
TCGA-GL-A9DE-01A-11R-A37K-07	stage i	t1b	male		
TCGA-AK-3465-01A-02R-1325-07	stage i	t1b	female		
TCGA-KM-8438-01A-11R-2315-07	stage ii	t2	female		
TCGA-B1-A654-01A-11R-A31O-07	stage i	t1a	female		
TCGA-B2-3923-01A-02R-1325-07	stage ii	t2	male		
TCGA-BQ-5881-01A-11R-1592-07	stage i	t1a	male		
TCGA-5P-A9K8-01A-11R-A42S-07	NA	t3a	female		
TCGA-P4-AAVM-01A-11R-A42S-07	stage i	t1a	male		
TCGA-IA-A83S-01A-11R-A355-07	NA	tx	male		
TCGA-MH-A562-01A-11R-A26U-07	stage i	t1a	male		
TCGA-5P-A9JZ-01A-11R-A42S-07	NA	t1b	female		

TCGA-GL-A59T-01A-21R-A28H-07	stage i	t1b	male		
TCGA-KO-8403-01A-11R-2315-07	stage i	t1a	male		
TCGA-IA-A40U-01A-11R-A24Z-07	stage iii	t3b	male		
TCGA-6D-AA2E-01A-11R-A37O-07	stage i	t1b	female		
TCGA-BQ-5880-01A-11R-1592-07	stage iii	t3a	male		
TCGA-B9-A44B-01A-11R-A24Z-07	stage iii	t3b	male		
TCGA-5P-A9K3-01A-11R-A42S-07	NA	t2	male		
TCGA-B0-5117-01A-01R-1420-07	stage i	t1b	male		
TCGA-KM-8441-01A-11R-2315-07	stage i	t1b	female		
TCGA-AK-3433-01A-02R-1277-07	stage ii	t2	female		
TCGA-4A-A93X-01A-11R-A37K-07	stage iv	t3a	male		
TCGA-KN-8437-01A-11R-2315-07	stage i	t1a	female		
TCGA-B1-A656-01A-11R-A31O-07	stage i	t1a	male		
TCGA-P4-A5EB-01A-11R-A28H-07	stage i	t1b	male		
TCGA-G7-6796-01A-11R-1965-07	stage i	t1a	male		
TCGA-UZ-A9PL-01A-11R-A38C-07	stage ii	t2	male		
TCGA-KL-8341-01A-11R-2315-07	stage iv	t3b	male		
TCGA-KO-8407-01A-11R-2315-07	stage ii	t2	male		
TCGA-B1-5398-01A-02R-1592-07	stage iii	t3b	male		
TCGA-DZ-6131-01A-11R-1965-07	stage iii	t3a	male		
TCGA-PJ-A5Z9-01A-11R-A28H-07	stage i	t1b	female		
TCGA-GL-A4EM-01A-11R-A24Z-07	stage i	t1b	male		
TCGA-2Z-A9J5-01A-21R-A38C-07	stage ii	t2	male		
TCGA-KN-8419-01A-11R-2315-07	stage ii	t2	male		
TCGA-KN-8434-01A-11R-2315-07	stage ii	t2	female		
TCGA-KO-8415-01A-11R-2315-07	stage i	t1	female		
TCGA-KL-8332-01A-11R-2315-07	stage i	t1b	male		
TCGA-5P-A9KH-01A-11R-A42S-07	NA	t1a	male		
TCGA-KN-8432-01A-11R-2315-07	stage ii	t2b	female		

TCGA-B1-A47O-01A-11R-A24Z-07	stage i	t1b	female		
TCGA-KN-8426-01A-11R-2315-07	stage iv	t3a	male		
TCGA-KM-8442-01A-11R-2315-07	stage ii	t2	male		
TCGA-KM-8477-01A-11R-2315-07	stage i	t1a	male		
TCGA-KN-8431-01A-11R-2315-07	stage ii	t2	female		
TCGA-AK-3443-01A-02R-1325-07	stage ii	t2	male		
TCGA-B9-A8YI-01A-21R-A37K-07	stage i	t1b	male		
TCGA-2Z-A9JO-01A-11R-A42S-07	stage i	t1b	female		
TCGA-F9-A7VF-01A-11R-A33Z-07	stage i	t1a	female		
TCGA-KV-A74V-01A-11R-A33Z-07	stage i	t1a	male		
TCGA-P4-A5E6-01A-11R-A28H-07	stage iii	t1b	male		
TCGA-J7-A8I2-01A-12R-A36F-07	stage i	t1b	male		
TCGA-IA-A83T-01A-11R-A355-07	NA	tx	male		
TCGA-AS-3777-01A-01R-0864-07	stage i	t1a	male		
TCGA-KO-8416-01A-11R-2315-07	stage iii	t3a	male		
TCGA-MH-A560-01A-11R-A26U-07	stage i	t1a	male		
TCGA-KN-8424-01A-11R-2315-07	stage i	t1b	female		
TCGA-A4-8312-01A-11R-2404-07	stage i	t1	male		
TCGA-KL-8343-01A-11R-2315-07	stage iii	t3	male		
TCGA-KN-8421-01A-11R-2315-07	stage ii	t2	female		
TCGA-KN-8433-01A-11R-2315-07	stage iii	t3a	female		
TCGA-A4-7286-01A-11R-A32Z-07	NA	t3a	male		
TCGA-KL-8342-01A-11R-2315-07	stage ii	t2b	female		
TCGA-KL-8340-01A-11R-2315-07	stage ii	t2	male		
TCGA-KL-8324-01A-11R-2315-07	stage ii	t2	female		
TCGA-BP-4761-01A-01R-1289-07	stage iii	t3a	male		
TCGA-MH-A857-01A-11R-A355-07	stage i	t1a	male		
TCGA-A4-A48D-01A-11R-A24Z-07	stage i	t1b	male		
TCGA-MH-A854-01A-11R-A355-07	stage i	t1b	female		

TCGA-KL-8325-01A-11R-2315-07	stage ii	t2	female		
TCGA-B0-5702-01A-11R-1541-07	stage i	t1b	male		
TCGA-KN-8425-01A-11R-2315-07	stage i	t1b	male		
TCGA-AK-3440-01A-02R-1277-07	stage i	t1a	male		
TCGA-KL-8323-01A-21R-2315-07	stage iii	t3b	female		
TCGA-KL-8336-01A-11R-2315-07	stage iv	t3b	female		
TCGA-KN-8428-01A-11R-2315-07	stage ii	t2	male		
TCGA-KO-8410-01A-11R-2315-07	stage i	t1b	female		
TCGA-IZ-A6M8-01A-11R-A31O-07	stage i	t1a	male		
TCGA-2Z-A9JT-01A-11R-A42S-07	stage i	t1a	male		
TCGA-A3-3328-01A-01R-0864-07	stage i	t1b	male		
TCGA-KO-8411-01A-11R-2315-07	stage i	t1b	male		
TCGA-KN-8418-01A-11R-2315-07	stage ii	t2	female		
TCGA-KL-8329-01A-11R-2315-07	stage i	t1b	female		
TCGA-KL-8335-01A-11R-2315-07	stage iii	t3a	male		
TCGA-KO-8409-01A-11R-2315-07	stage ii	t2b	male		
TCGA-KO-8408-01A-11R-2315-07	stage iii	t3a	male		
TCGA-KL-8346-01A-11R-2315-07	stage ii	t2b	male		
TCGA-A4-8518-01A-11R-2404-07	stage i	t1a	male		
TCGA-KL-8327-01A-11R-2315-07	stage i	t1b	female		
TCGA-KO-8406-01A-11R-2315-07	stage i	t1	female		
TCGA-KN-8435-01A-11R-2315-07	stage ii	t2	male		
TCGA-KO-8417-01A-11R-2315-07	stage i	t1	female		
TCGA-KN-8422-01A-11R-2315-07	stage i	t1a	female		
TCGA-KL-8339-01A-11R-2315-07	#N/A	#N/A	#N/A		
TCGA-A3-3374-01A-02R-1325-07	stage i	t1b	female		
TCGA-KM-8639-01A-11R-2403-07	stage i	t1a	male		
TCGA-KM-8439-01A-11R-2315-07	stage i	t1b	male		
TCGA-KL-8330-01A-11R-2315-07	stage ii	t2b	female		

TCGA-BP-4334-01A-01R-1289-07	stage iii	t3a	male		
TCGA-KL-8338-01A-11R-2315-07	stage iii	t3a	male		
TCGA-KM-8440-01A-11R-2315-07	stage iii	t3a	male		
TCGA-KO-8414-01A-11R-2315-07	stage ii	t2	female		
TCGA-AL-A5DJ-01A-11R-A26U-07	stage iii	t3a	female		
TCGA-2Z-A9J2-01A-11R-A38C-07	stage i	t1a	female		
TCGA-KM-8443-01A-11R-2315-07	stage ii	t2	male		
TCGA-KM-8476-01A-11R-2315-07	stage i	t1	male		
TCGA-KL-8345-01A-11R-2315-07	stage iii	t3a	male		
TCGA-KL-8331-01A-11R-2315-07	stage ii	t2	female		
TCGA-AK-3447-01A-01R-1766-07	stage ii	t2	male		
TCGA-Y8-A8S1-01A-11R-A37K-07	stage i	t1a	male		
TCGA-KL-8326-01A-11R-2315-07	stage iii	t3a	male		
TCGA-KL-8337-01A-11R-2315-07	stage ii	t2	male		
TCGA-KL-8334-01A-11R-2315-07	stage iii	t3a	female		
TCGA-Q2-A5QZ-01A-11R-A28H-07	stage iii	t3a	female		
B) KIRC					
TCGA-B0-5700-01A-11R-1541-07	stage i	t1a	male		
TCGA-B8-A7U6-01A-12R-A37O-07	stage i	t1a	female		
TCGA-BP-5200-01A-01R-1426-07	stage i	t1	male		
TCGA-A3-A6NJ-01A-12R-A33J-07	stage i	t1a	female		
TCGA-CJ-4876-01A-01R-1305-07	stage i	t1b	male		
TCGA-BP-4774-01A-01R-1289-07	stage iii	t3b	male		
TCGA-B0-5099-01A-01R-1420-07	stage i	t1a	female		
TCGA-B0-4710-01A-01R-1503-07	stage i	t1b	male		
TCGA-BP-4326-01A-01R-1289-07	stage iii	t3a	male		
TCGA-BP-4776-01A-01R-1289-07	stage iv	t4	male		
TCGA-GK-A6C7-01A-11R-A33J-07	stage iii	t3a	male	MIDN	>75 Percentile

TCGA-DV-A4W0-01A-11R-A266-07	stage i	t1a	male			
TCGA-CZ-5453-01A-01R-1503-07	stage iii	t3a	female			
TCGA-B0-5693-01A-11R-1541-07	stage i	t1b	male			
TCGA-BP-5008-01A-01R-1334-07	stage iii	t3a	male			
TCGA-B8-A8YJ-01A-13R-A39I-07	stage i	t1a	female			
TCGA-BP-5201-01A-01R-1426-07	stage i	t1a	male			
TCGA-BP-5202-01A-02R-1426-07	stage i	t1a	male			
TCGA-CW-6093-01A-11R-1672-07	stage i	t1a	male			
TCGA-B8-A54J-01A-11R-A33J-07	stage i	t1a	male			
TCGA-BP-5006-01A-01R-1334-07	stage i	t1	male			
TCGA-BP-4169-01A-02R-1289-07	stage ii	t2	female			
TCGA-A3-A8OU-01A-11R-A37O-07	stage i	t1a	male			
TCGA-BP-4781-01A-01R-1305-07	stage iii	t3c	male			
TCGA-B0-5707-01A-11R-1541-07	stage i	t1b	male			
TCGA-DV-5569-01A-01R-1541-07	stage iii	t3c	female			
TCGA-B0-5698-01A-11R-1672-07	stage i	t1a	female			
TCGA-DV-5566-01A-01R-1541-07	stage iv	t3b	male			
TCGA-A3-3373-01A-02R-1420-07	stage ii	t2	male			
TCGA-B0-4822-01A-01R-1277-07	stage ii	t2b	male			
TCGA-B8-4148-01A-02R-1325-07	stage i	t1b	male			
TCGA-B0-4813-01A-01R-1277-07	stage iii	t3a	male			
TCGA-B0-4841-01A-01R-1277-07	stage i	t1b	male			
TCGA-B0-5695-01A-11R-1541-07	stage ii	t2	male			
TCGA-CJ-4920-01A-01R-1426-07	stage i	t1a	male			
TCGA-BP-4974-01A-01R-1334-07	stage iii	t3a	male			
TCGA-B0-4718-01A-01R-1277-07	stage iii	t3a	male			
TCGA-G6-A8L7-01A-11R-A37O-07	stage i	t1a	male			
TCGA-CJ-4882-01A-02R-1426-07	stage i	t1a	male			
TCGA-A3-3365-01A-01R-0864-07	NA	t1a	female			

TCGA-B0-5104-01A-01R-1420-07	stage iii	t3a	male		
TCGA-BP-4999-01A-01R-1334-07	stage i	t1a	female		
TCGA-B0-5702-01A-11R-1541-07	stage i	t1a	male		
TCGA-CJ-4878-01A-01R-1305-07	stage i	t1a	male		
TCGA-AK-3454-01A-02R-1277-07	stage iii	t3a	male		
TCGA-CZ-4861-01A-01R-1305-07	stage iii	t3a	male		
TCGA-DV-5565-01A-01R-1541-07	stage ii	t2	male		
TCGA-B0-5690-01A-11R-1541-07	stage ii	t2a	male		
TCGA-EU-5904-01A-11R-1672-07	stage i	t1b	male		
TCGA-DV-5575-01A-01R-1541-07	stage i	t1b	male		
TCGA-CZ-5467-01A-01R-1503-07	stage iv	t3a	male		
TCGA-B0-5706-01A-11R-1541-07	stage i	t1a	female		
TCGA-B0-4693-01A-01R-1277-07	stage i	t1a	male		
TCGA-B0-4846-01A-01R-1277-07	stage i	t1	male		
TCGA-B0-4849-01A-01R-1277-07	stage ii	t2	male		
TCGA-B0-5085-01A-01R-1334-07	stage iii	t3a	female		
TCGA-B8-4151-01A-01R-1188-07	stage iii	t1b	female		
TCGA-AK-3440-01A-02R-1277-07	stage i	t1a	male		
TCGA-BP-4981-01A-01R-1334-07	stage iii	t3a	female		
TCGA-BP-5010-01A-02R-1420-07	stage i	t1	female		
TCGA-B0-5119-01A-02R-1420-07	stage i	t1	female		
TCGA-CW-5580-01A-01R-1672-07	stage iii	t3a	female		
TCGA-MW-A4EC-01A-11R-A266-07	stage iii	t1a	male		
TCGA-AK-3460-01A-02R-1277-07	stage i	t1b	female		
TCGA-B0-5077-01A-01R-1334-07	stage i	t1b	female		
TCGA-BP-4162-01A-02R-1325-07	stage iii	t3a	male		
TCGA-BP-4355-01A-01R-1289-07	stage iv	t4	male		
TCGA-B0-4852-01A-01R-1503-07	stage ii	t2	female		
TCGA-B0-4823-01A-02R-1420-07	stage ii	t2	female		

TCGA-3Z-A93Z-01A-11R-A37O-07	stage i	t1b	female		
TCGA-AK-3461-01A-02R-1277-07	stage i	t1a	female		
TCGA-CZ-5456-01A-01R-1503-07	stage iii	t3a	female		
TCGA-B8-4620-01A-02R-1325-07	stage ii	t2	male		
TCGA-B0-5713-01A-11R-1672-07	stage iii	t3a	female		
TCGA-BP-4173-01A-02R-1289-07	stage i	t1b	male		
TCGA-CZ-5465-01A-01R-1503-07	stage i	t1a	male		
TCGA-CJ-5676-01A-11R-1541-07	stage i	t1b	female		
TCGA-BP-5185-01A-01R-1426-07	stage i	t1a	female		
TCGA-DV-5568-01A-01R-1541-07	stage ii	t2	male		
TCGA-BP-4993-01A-02R-1420-07	stage i	t1a	male		
TCGA-B0-4945-01A-01R-1420-07	stage i	t1a	female		
TCGA-B0-5110-01A-01R-1420-07	stage i	t1b	female		
TCGA-B0-5116-01A-02R-1420-07	stage i	t1	female		
TCGA-BP-4765-01A-01R-1289-07	stage iii	t3a	female		
TCGA-BP-4340-01A-01R-1289-07	stage i	t1b	female		
TCGA-CZ-5986-01A-11R-1672-07	stage i	t1a	male		
TCGA-A3-A8CQ-01A-11R-A37O-07	stage i	t1a	female		
TCGA-DV-A4VZ-01A-11R-A266-07	stage iii	t3a	male		
TCGA-DV-A4VX-01A-11R-A266-07	stage i	t1a	male		
TCGA-G6-A8L6-01A-11R-A37O-07	stage i	t1b	male		
TCGA-CJ-4901-01A-01R-1426-07	stage iii	t3a	female		
TCGA-BP-4170-01A-02R-1289-07	stage ii	t2	female		
TCGA-BP-5199-01A-01R-1426-07	stage i	t1a	male		
TCGA-BP-4341-01A-01R-1289-07	stage i	t1a	male		
TCGA-B0-5097-01A-01R-1420-07	stage ii	t2	male		
TCGA-EU-5906-01A-11R-1672-07	stage ii	t2	male		
TCGA-BP-4967-01A-01R-1334-07	stage iii	t3a	female		
TCGA-BP-4960-01A-01R-1334-07	stage iv	t3b	male		

TCGA-BP-5004-01A-01R-1334-07	stage i	t1	female		
TCGA-B8-5164-01A-01R-1420-07	stage i	t1b	female		
TCGA-A3-3372-01A-02R-1325-07	stage iii	t1b	male		
TCGA-G6-A8L8-01A-21R-A37O-07	stage i	t1a	female		
TCGA-BP-5178-01A-01R-1426-07	stage i	t1a	female		
TCGA-B0-4811-01A-01R-1503-07	stage ii	t2	male		
TCGA-AK-3428-01A-02R-1277-07	stage i	t1	male		
TCGA-BP-4992-01A-01R-1334-07	stage iv	t1a	female		
TCGA-B0-4843-01A-01R-1277-07	stage i	t1a	female		
TCGA-BP-5198-01A-01R-1426-07	stage i	t1a	male		
TCGA-BP-4766-01A-01R-1289-07	stage iii	t3b	female		
TCGA-B0-4838-01A-01R-1305-07	stage i	t1b	male		
TCGA-CZ-5987-01A-11R-1672-07	stage i	t1a	male		
TCGA-B0-4701-01A-01R-1277-07	stage i	t1b	female		
TCGA-CJ-6032-01A-11R-1672-07	stage i	t1b	male		
TCGA-A3-3331-01A-02R-1325-07	stage i	t1a	female		
TCGA-BP-4769-01A-01R-1289-07	stage iii	t3a	male		
TCGA-B0-5088-01A-01R-1334-07	stage i	t1a	male		
TCGA-B0-4816-01A-01R-1503-07	stage iv	t3b	male		
TCGA-B0-4847-01A-01R-1277-07	stage i	t1a	female		
TCGA-BP-4761-01A-01R-1289-07	stage iv	t3a	male		
TCGA-B0-5107-01A-01R-1420-07	stage ii	t2	male		
TCGA-BP-4799-01A-01R-1305-07	stage ii	t2a	female		
TCGA-BP-5168-01A-01R-1420-07	stage i	t1a	male		
TCGA-CJ-4904-01A-02R-1426-07	stage i	t1b	male		
TCGA-B0-5096-01A-01R-1420-07	stage ii	t2	female		
TCGA-CZ-5460-01A-01R-1503-07	stage iii	t3a	male		
TCGA-B0-5697-01A-11R-1541-07	stage i	t1b	female		
TCGA-B0-4828-01A-01R-1277-07	stage i	t1	male		

TCGA-CJ-4923-01A-01R-1426-07	stage i	t1a	male		
TCGA-BP-4342-01A-01R-1289-07	stage i	t1b	female		
TCGA-A3-3359-01A-01R-0864-07	stage i	t1a	male		
TCGA-A3-3351-01A-02R-1325-07	stage i	t1a	male		
TCGA-CJ-4875-01A-01R-1305-07	stage i	t1b	male		
TCGA-CJ-4636-01A-02R-1325-07	stage i	t1a	male		
TCGA-A3-3316-01A-01R-0864-07	stage i	t1a	male		
TCGA-BP-4995-01A-01R-1334-07	stage ii	t2	male		
TCGA-CW-5583-01A-02R-1541-07	stage iii	t3a	male		
TCGA-CJ-4893-01A-01R-1305-07	stage i	t1	male		
TCGA-B8-5163-01A-01R-1420-07	stage i	t1b	male		
TCGA-B4-5836-01A-11R-1672-07	stage i	t1a	male		
TCGA-CW-6087-01A-11R-1672-07	stage i	t1b	female		
TCGA-B0-5399-01A-01R-1503-07	stage ii	t2a	female		
TCGA-CZ-5468-01A-01R-1503-07	stage i	t1a	female		
TCGA-A3-3347-01A-02R-1325-07	stage i	t1a	female		
TCGA-CJ-4890-01A-01R-1305-07	stage i	t1b	female		
TCGA-BP-4163-01A-02R-1325-07	stage i	t1a	male		
TCGA-B0-4814-01A-01R-1277-07	stage ii	t2	male		
TCGA-BP-4972-01A-01R-1334-07	stage iv	t3a	male		
TCGA-B0-5705-01A-11R-1541-07	stage i	t1b	male		
TCGA-CJ-4638-01A-02R-1325-07	stage i	t1	female		
TCGA-BP-5180-01A-01R-1426-07	stage iv	t2	male		
TCGA-B8-5551-01A-01R-1541-07	stage i	t1	male		
TCGA-A3-3378-01A-02R-1325-07	stage i	t1b	female		
TCGA-BP-4803-01A-01R-1305-07	stage iv	t2	male		
TCGA-CZ-5984-01A-11R-1672-07	stage i	t1a	female		
TCGA-BP-4988-01A-01R-1334-07	stage ii	t2	female		
TCGA-AK-3458-01A-01R-1503-07	stage i	t1a	female		<25 Percentile

TCGA-A3-3358-01A-01R-1541-07	stage i	t1a	female			
TCGA-B0-5084-01A-01R-1334-07	stage ii	t2	male			
TCGA-CJ-5680-01A-11R-1541-07	stage i	t1b	female			
TCGA-A3-3387-01A-01R-1541-07	stage i	t1a	male			
TCGA-CW-5581-01A-02R-1541-07	stage i	t1b	female			
TCGA-B0-5710-01A-11R-1672-07	stage i	t1b	female			
TCGA-BP-4964-01A-01R-1334-07	stage iv	t2	male			
TCGA-A3-3352-01A-01R-0864-07	stage i	t1a	male			
TCGA-BP-5183-01A-01R-1426-07	stage i	t1b	male			
TCGA-BP-5174-01A-01R-1426-07	stage iii	t3a	male			
TCGA-BP-4330-01A-01R-1289-07	stage ii	t2	male			
TCGA-B0-4690-01A-01R-1277-07	stage iii	t3a	male			
TCGA-B0-5117-01A-01R-1420-07	stage iii	t3a	male			
TCGA-BP-4770-01A-01R-1503-07	stage iii	t3a	male			
TCGA-CZ-4862-01A-01R-1305-07	stage i	t1b	male			
TCGA-CZ-5451-01A-01R-1503-07	stage iii	t3b	male			
TCGA-CZ-4858-01A-01R-1305-07	stage iii	t3b	female			
TCGA-B0-4836-01A-01R-1305-07	stage i	t1b	male			
TCGA-CJ-4907-01A-01R-1426-07	stage iii	t3a	female			
TCGA-CZ-5452-01A-01R-1503-07	stage i	t1a	female			
TCGA-CJ-4639-01A-02R-1325-07	stage ii	t2	male			
TCGA-BP-5181-01A-01R-1426-07	stage i	t1a	female			
TCGA-A3-3306-01A-01R-0864-07	stage i	t1a	female			
TCGA-B8-A54G-01A-11R-A266-07	stage i	t1a	female			
TCGA-B2-4101-01A-02R-1277-07	stage ii	t2a	male			
TCGA-B0-4714-01A-01R-1277-07	stage iii	t3a	female			
TCGA-BP-5189-01A-02R-1426-07	stage i	t1a	female			
TCGA-B2-5639-01A-01R-1541-07	stage i	t1b	male			
TCGA-BP-5191-01A-01R-1426-07	stage iii	t3b	male			

TCGA-BP-4962-01A-01R-1334-07	stage i	t1b	female		
TCGA-BP-4959-01A-01R-1334-07	stage i	t1a	male		
TCGA-CW-5588-01A-01R-1541-07	stage ii	t2	male		
TCGA-BP-4973-01A-01R-1334-07	stage iii	t3b	male		
TCGA-AK-3456-01A-02R-1325-07	stage i	t1a	male		
TCGA-AK-3436-01A-02R-1325-07	stage i	t1b	male		
TCGA-B0-5691-01A-11R-1541-07	stage i	t1b	male		
TCGA-B0-4694-01A-01R-1277-07	stage i	t1	male		
TCGA-CJ-4641-01A-02R-1325-07	stage iii	t3a	female		
TCGA-B8-5158-01A-01R-1420-07	stage ii	t2	male		
TCGA-A3-3324-01A-02R-1325-07	stage i	t1b	male		
TCGA-BP-5190-01A-01R-1426-07	stage iii	t3b	female		
TCGA-B8-4154-01A-01R-1188-07	stage ii	t2a	male		
TCGA-B0-5711-01A-11R-1672-07	stage ii	t2	female		
TCGA-CZ-4860-01A-01R-1305-07	stage i	t1b	female		
TCGA-CJ-6027-01A-11R-1672-07	stage i	t1a	male		
TCGA-B4-5834-01A-11R-1672-07	stage i	t1b	male		
TCGA-B8-5165-01A-01R-1420-07	stage iii	t3	male		
TCGA-A3-3385-01A-02R-1420-07	stage iii	t3b	male		
TCGA-B4-5843-01A-11R-1672-07	stage i	t1b	male		
TCGA-A3-3326-01A-01R-0864-07	stage i	t1b	female		
TCGA-AK-3445-01A-02R-1277-07	stage i	t1a	male		
TCGA-B8-A54K-01A-11R-A33J-07	stage iii	t3b	female		
TCGA-CJ-5672-01A-11R-1541-07	stage i	t1b	male		
TCGA-B2-5633-01A-01R-1541-07	stage i	t1a	male		
TCGA-EU-5905-01A-11R-1672-07	stage i	t1b	male		
TCGA-A3-3357-01A-02R-1420-07	stage i	t1a	male		
TCGA-AK-3425-01A-02R-1277-07	stage i	t1b	male		
TCGA-BP-4762-01A-02R-1289-07	stage i	t1b	male		

TCGA-CJ-4644-01A-02R-1325-07	stage iii	t3b	male			
TCGA-CJ-4874-01A-01R-1305-07	stage ii	t2a	male			
TCGA-B4-5838-01A-11R-1672-07	stage i	t1b	male			
TCGA-CJ-4884-01A-01R-1305-07	stage ii	t2a	male			
TCGA-CZ-5463-01A-01R-1503-07	stage i	t1a	female			
TCGA-AK-3444-01A-02R-1325-07	stage i	t1b	male			
TCGA-B8-5159-01A-01R-1420-07	stage i	t1a	male			
TCGA-BP-4976-01A-01R-1334-07	stage iii	t3a	male			
TCGA-B2-4102-01A-02R-1325-07	stage i	t1b	female			
TCGA-BP-5194-01A-02R-1426-07	stage i	t1b	female			
TCGA-B8-5550-01A-01R-1541-07	stage i	t1	male			
TCGA-BP-4158-01A-02R-1289-07	stage iii	t3a	male			
TCGA-BP-4965-01A-01R-1334-07	stage i	t1b	male			
TCGA-B2-3923-01A-02R-1325-07	stage i	t1a	female			
TCGA-B2-5641-01A-01R-1541-07	stage iii	t3b	female			
TCGA-B0-5701-01A-11R-1541-07	stage i	t1a	female			
TCGA-CZ-5985-01A-11R-1672-07	stage iv	t3a	female			
TCGA-BP-4353-01A-02R-1289-07	stage i	t1b	female			
TCGA-CW-5587-01A-01R-1541-07	stage i	t1b	female			
TCGA-B0-4696-01A-01R-1277-07	stage iii	t3b	female			
TCGA-CW-5585-01A-01R-1541-07	stage ii	t2	female			
TCGA-A3-3328-01A-01R-0864-07	stage i	t1b	male			
TCGA-BP-4354-01A-02R-1289-07	stage iv	t3b	female			
TCGA-BP-4756-01A-01R-1289-07	stage iv	t4	male			
TCGA-B0-5083-01A-02R-1420-07	stage iv	t4	male			
TCGA-A3-3323-01A-02R-1325-07	stage i	t1a	female			
TCGA-CJ-4897-01A-03R-1426-07	stage iv	t3a	female			
TCGA-AK-3447-01A-01R-1766-07	stage i	t1	male			
TCGA-AS-3777-01A-01R-0864-07	stage i	t1b	male			

TCGA-B8-A54E-01A-11R-A266-07	stage i	t1a	male		
TCGA-AK-3433-01A-02R-1277-07	stage i	t1b	female		
TCGA-BP-4763-01A-01R-1289-07	stage iii	t3a	male		
TCGA-CZ-4853-01A-01R-1426-07	stage iii	t3a	male		
TCGA-A3-3367-01A-02R-1420-07	stage i	t1a	male		
TCGA-B0-4834-01A-01R-1305-07	stage i	t1a	male		
TCGA-CZ-5458-01A-01R-1503-07	stage i	t1a	male		
TCGA-B8-5546-01A-01R-1541-07	stage i	t1b	female		
TCGA-CZ-4866-01A-01R-1503-07	stage iii	t3b	male		
TCGA-AK-3451-01A-02R-1188-07	stage i	t1a	male		
TCGA-B8-4619-01A-02R-1325-07	stage iii	t3a	male		
TCGA-CJ-5681-01A-11R-1541-07	stage i	t1a	female		
TCGA-AK-3453-01A-02R-1277-07	stage ii	t2b	female		
TCGA-A3-3313-01A-02R-1325-07	stage i	t1a	female		
TCGA-BP-4343-01A-02R-1289-07	stage i	t1a	male		
TCGA-B4-5832-01A-11R-1672-07	stage ii	t2	male		
TCGA-T7-A92I-01A-11R-A37O-07	stage iii	t3a	male		
TCGA-CZ-5989-01A-11R-1672-07	NA	t3b	male		
TCGA-AK-3427-01A-01R-0864-07	stage i	t1a	male		
TCGA-CJ-4878-01A-01R-1305-07	stage i	t1a	male		
TCGA-CW-6093-01A-11R-1672-07	stage i	t1a	male		
TCGA-B0-5104-01A-01R-1420-07	stage iii	t3a	male		
TCGA-B0-4701-01A-01R-1277-07	stage i	t1b	female		
TCGA-B8-A7U6-01A-12R-A37O-07	stage i	t1a	female		
TCGA-B0-5098-01A-01R-1420-07	stage ii	t2	male		
TCGA-BP-4774-01A-01R-1289-07	stage iii	t3b	male		
TCGA-B0-5698-01A-11R-1672-07	stage i	t1a	female		
TCGA-B0-4698-01A-01R-1503-07	stage ii	t2	female		
TCGA-B8-A8YJ-01A-13R-A39I-07	stage i	t1a	female	PPRC1	>75 Percentile

TCGA-BP-4992-01A-01R-1334-07	stage iv	t1a	female		
TCGA-BP-5200-01A-01R-1426-07	stage i	t1	male		
TCGA-B0-4696-01A-01R-1277-07	stage iii	t3b	female		
TCGA-CZ-5982-01A-11R-1672-07	stage i	t1a	male		
TCGA-B0-4849-01A-01R-1277-07	stage ii	t2	male		
TCGA-B8-4148-01A-02R-1325-07	stage i	t1b	male		
TCGA-A3-A6NJ-01A-12R-A33J-07	stage i	t1a	female		
TCGA-CJ-4904-01A-02R-1426-07	stage i	t1b	male		
TCGA-B4-5377-01A-01R-1503-07	stage i	t1b	male		
TCGA-B0-5693-01A-11R-1541-07	stage i	t1b	male		
TCGA-BP-4169-01A-02R-1289-07	stage ii	t2	female		
TCGA-DV-5569-01A-01R-1541-07	stage iii	t3c	female		
TCGA-DV-A4VZ-01A-11R-A266-07	stage iii	t3a	male		
TCGA-GK-A6C7-01A-11R-A33J-07	stage iii	t3a	male		
TCGA-BP-5202-01A-02R-1426-07	stage i	t1a	male		
TCGA-BP-4983-01A-01R-1334-07	stage iv	t3a	male		
TCGA-BP-4352-01A-01R-1289-07	stage iii	t3a	male		
TCGA-AK-3440-01A-02R-1277-07	stage i	t1a	male		
TCGA-AK-3428-01A-02R-1277-07	stage i	t1	male		
TCGA-B0-5084-01A-01R-1334-07	stage ii	t2	male		
TCGA-A3-3373-01A-02R-1420-07	stage ii	t2	male		
TCGA-BP-5201-01A-01R-1426-07	stage i	t1a	male		
TCGA-B0-5097-01A-01R-1420-07	stage ii	t2	male		
TCGA-BP-5006-01A-01R-1334-07	stage i	t1	male		
TCGA-BP-5004-01A-01R-1334-07	stage i	t1	female		
TCGA-BP-4326-01A-01R-1289-07	stage iii	t3a	male		
TCGA-DV-5565-01A-01R-1541-07	stage ii	t2	male		
TCGA-CW-6087-01A-11R-1672-07	stage i	t1b	female		
TCGA-BP-5008-01A-01R-1334-07	stage iii	t3a	male		

TCGA-B0-5700-01A-11R-1541-07	stage i	t1a	male			
TCGA-B8-A54J-01A-11R-A33J-07	stage i	t1a	male			
TCGA-BP-4974-01A-01R-1334-07	stage iii	t3a	male			
TCGA-B0-4710-01A-01R-1503-07	stage i	t1b	male			
TCGA-B0-4838-01A-01R-1305-07	stage i	t1b	male			
TCGA-CZ-4858-01A-01R-1305-07	stage iii	t3b	female			
TCGA-CJ-4876-01A-01R-1305-07	stage i	t1b	male			
TCGA-DV-A4W0-01A-11R-A266-07	stage i	t1a	male			
TCGA-CW-6097-01A-11R-1672-07	stage iii	t3a	male			
TCGA-BP-4765-01A-01R-1289-07	stage iii	t3a	female			
TCGA-BP-4347-01A-01R-1289-07	stage iv	t2	male			
TCGA-A3-3317-01A-02R-1325-07	stage i	t1a	female			
TCGA-B0-5109-01A-02R-1420-07	stage ii	t2b	male			
TCGA-A3-3313-01A-02R-1325-07	stage i	t1a	female			
TCGA-CJ-4908-01A-01R-1426-07	stage iv	t3a	male			
TCGA-CZ-5467-01A-01R-1503-07	stage iv	t3a	male			
TCGA-BP-4760-01A-02R-1420-07	stage i	t1b	male			
TCGA-CW-5580-01A-01R-1672-07	stage iii	t3a	female			
TCGA-CJ-4900-01A-01R-1334-07	stage i	t1a	female			
TCGA-CZ-4860-01A-01R-1305-07	stage i	t1b	female			
TCGA-BP-5169-01A-01R-1426-07	stage i	t1	female			
TCGA-A3-3365-01A-01R-0864-07	NA	t1a	female			
TCGA-BP-5198-01A-01R-1426-07	stage i	t1a	male			
TCGA-BP-4981-01A-01R-1334-07	stage iii	t3a	female			
TCGA-B0-5108-01A-01R-1420-07	stage iii	t3a	male			
TCGA-A3-A8CQ-01A-11R-A37O-07	stage i	t1a	female			
TCGA-BP-4989-01A-01R-1334-07	stage iv	t3a	male			
TCGA-BP-4993-01A-02R-1420-07	stage i	t1a	male			
TCGA-B0-4846-01A-01R-1277-07	stage i	t1	male			

TCGA-B0-4852-01A-01R-1503-07	stage ii	t2	female			
TCGA-B0-4819-01A-01R-1277-07	stage iii	t3a	male			
TCGA-BP-4173-01A-02R-1289-07	stage i	t1b	male			
TCGA-BP-4170-01A-02R-1289-07	stage ii	t2	female			
TCGA-A3-3347-01A-02R-1325-07	stage i	t1a	female			
TCGA-CZ-5463-01A-01R-1503-07	stage i	t1a	female			
TCGA-B0-4841-01A-01R-1277-07	stage i	t1b	male			
TCGA-B8-5551-01A-01R-1541-07	stage i	t1	male			
TCGA-A3-A8OX-01A-11R-A37O-07	stage i	t1b	male			
TCGA-B0-5110-01A-01R-1420-07	stage i	t1b	female			
TCGA-AK-3461-01A-02R-1277-07	stage i	t1a	female			
TCGA-DV-5576-01A-01R-1541-07	stage i	t1a	male			
TCGA-BP-4771-01A-01R-1289-07	stage iii	t3a	male			
TCGA-BP-4349-01A-01R-1289-07	stage iii	t3a	female			
TCGA-B4-5835-01A-11R-1672-07	stage i	t1b	female			
TCGA-B0-5107-01A-01R-1420-07	stage ii	t2	male			
TCGA-BP-4162-01A-02R-1325-07	stage iii	t3a	male			
TCGA-CJ-6027-01A-11R-1672-07	stage i	t1a	male			
TCGA-B0-4693-01A-01R-1277-07	stage i	t1a	male			
TCGA-B0-4815-01A-01R-1503-07	stage iii	t3b	male			
TCGA-B8-4620-01A-02R-1325-07	stage ii	t2	male			
TCGA-CJ-6032-01A-11R-1672-07	stage i	t1b	male			
TCGA-CJ-4920-01A-01R-1426-07	stage i	t1a	male			
TCGA-BP-4355-01A-01R-1289-07	stage iv	t4	male			
TCGA-BP-4331-01A-01R-1289-07	stage i	t1a	female			
TCGA-DV-5568-01A-01R-1541-07	stage ii	t2	male			
TCGA-A3-3372-01A-02R-1325-07	stage iii	t1b	male			
TCGA-CJ-4899-01A-01R-1334-07	stage i	t1b	female			
TCGA-B8-5162-01A-01R-1420-07	stage i	t1a	female			

TCGA-B0-4699-01A-01R-1277-07	stage iii	t3a	male		
TCGA-B0-5096-01A-01R-1420-07	stage ii	t2	female		
TCGA-B4-5378-01A-01R-1503-07	stage ii	t2	male		
TCGA-CZ-5987-01A-11R-1672-07	stage i	t1a	male		
TCGA-BP-4971-01A-01R-1334-07	stage iv	t3a	male		
TCGA-B0-4945-01A-01R-1420-07	stage i	t1a	female		
TCGA-B0-4833-01A-01R-1305-07	stage i	t1a	male		
TCGA-CZ-5466-01A-01R-1503-07	stage iv	t4	female		
TCGA-DV-5575-01A-01R-1541-07	stage i	t1b	male		
TCGA-B0-5713-01A-11R-1672-07	stage iii	t3a	female		
TCGA-BP-4354-01A-02R-1289-07	stage iv	t3b	female		
TCGA-B0-4690-01A-01R-1277-07	stage iii	t3a	male		
TCGA-DV-5567-01A-01R-1541-07	stage iii	t3a	male		
TCGA-B0-4821-01A-01R-1503-07	stage iii	t3a	male		
TCGA-BP-4781-01A-01R-1305-07	stage iii	t3c	male		
TCGA-B0-4816-01A-01R-1503-07	stage iv	t3b	male		
TCGA-CJ-4882-01A-02R-1426-07	stage i	t1a	male		
TCGA-CJ-4888-01A-01R-1305-07	stage i	t1	male		
TCGA-A3-3308-01A-02R-1325-07	stage i	t1a	male		
TCGA-B0-4718-01A-01R-1277-07	stage iii	t3a	male		
TCGA-A3-3387-01A-01R-1541-07	stage i	t1a	male		
TCGA-G6-A8L6-01A-11R-A37O-07	stage i	t1b	male		
TCGA-CZ-4859-01A-02R-1426-07	stage i	t1b	male		
TCGA-BP-4770-01A-01R-1503-07	stage iii	t3a	male		
TCGA-CJ-4901-01A-01R-1426-07	stage iii	t3a	female		
TCGA-CJ-4643-01A-02R-1325-07	stage i	t1b	male		
TCGA-BP-4177-01A-02R-1420-07	stage ii	t2	male		
TCGA-B0-4688-01A-01R-1277-07	stage i	t1b	male		
TCGA-BP-4986-01A-01R-1334-07	stage iii	t3a	female		

TCGA-B2-5636-01A-02R-1541-07	stage i	t1b	female		
TCGA-BP-4776-01A-01R-1289-07	stage iv	t4	male		
TCGA-BP-4798-01A-01R-1305-07	stage i	t1a	female		
TCGA-B0-4828-01A-01R-1277-07	stage i	t1	male		
TCGA-CJ-4894-01A-01R-1305-07	stage i	t1a	male		
TCGA-CJ-5681-01A-11R-1541-07	stage i	t1a	female		
TCGA-B2-5641-01A-01R-1541-07	stage iii	t3b	female		
TCGA-B0-4842-01A-02R-1420-07	stage i	t1a	female		
TCGA-B8-A54G-01A-11R-A266-07	stage i	t1a	female		
TCGA-A3-3367-01A-02R-1420-07	stage i	t1a	male		
TCGA-BP-4965-01A-01R-1334-07	stage i	t1b	male		
TCGA-BP-4784-01A-01R-1305-07	stage ii	t2	female		
TCGA-BP-5176-01A-01R-1426-07	stage i	t1a	female		
TCGA-CJ-5676-01A-11R-1541-07	stage i	t1b	female		
TCGA-BP-5007-01A-01R-1334-07	stage iii	t3b	female		
TCGA-BP-4959-01A-01R-1334-07	stage i	t1a	male		
TCGA-CJ-4635-01A-02R-1305-07	stage i	t1b	male		
TCGA-BP-4972-01A-01R-1334-07	stage iv	t3a	male		
TCGA-BP-5186-01A-01R-1426-07	stage i	t1b	male		
TCGA-BP-5000-01A-01R-1334-07	stage iii	t3a	female		
TCGA-B0-4824-01A-01R-1277-07	stage iii	t3a	male		
TCGA-A3-3383-01A-02R-1325-07	stage iii	t3a	male		
TCGA-BP-4975-01A-01R-1334-07	stage ii	t2	female		
TCGA-BP-4804-01A-02R-1305-07	stage i	t1b	female		
TCGA-BP-5174-01A-01R-1426-07	stage iii	t3a	male		
TCGA-BP-4964-01A-01R-1334-07	stage iv	t2	male		
TCGA-BP-4164-01A-02R-1325-07	stage iii	t3a	male		
TCGA-BP-5173-01A-01R-1426-07	stage iv	t2	female		
TCGA-CW-5589-01A-01R-1541-07	stage ii	t2	male		<25 Percentile

TCGA-B0-5116-01A-02R-1420-07	stage i	t1	female		
TCGA-B8-A54F-01A-11R-A266-07	stage i	t1a	male		
TCGA-BP-4338-01A-01R-1289-07	stage i	t1a	male		
TCGA-B0-5120-01A-01R-1420-07	stage i	t1a	male		
TCGA-BP-4789-01A-01R-1305-07	stage iii	t3b	female		
TCGA-AK-3429-01A-02R-1325-07	stage i	t1a	male		
TCGA-BP-4174-01A-02R-1289-07	stage iv	t2	male		
TCGA-CJ-4907-01A-01R-1426-07	stage iii	t3a	female		
TCGA-A3-3316-01A-01R-0864-07	stage i	t1a	male		
TCGA-BP-5184-01A-01R-1426-07	stage i	t1b	female		
TCGA-BP-5192-01A-01R-1426-07	stage iv	t4	male		
TCGA-CJ-4644-01A-02R-1325-07	stage iii	t3b	male		
TCGA-BP-4158-01A-02R-1289-07	stage iii	t3a	male		
TCGA-B0-5117-01A-01R-1420-07	stage iii	t3a	male		
TCGA-CJ-4639-01A-02R-1325-07	stage ii	t2	male		
TCGA-BP-4759-01A-01R-1289-07	stage iv	t3a	female		
TCGA-BP-4803-01A-01R-1305-07	stage iv	t2	male		
TCGA-B0-5711-01A-11R-1672-07	stage ii	t2	female		
TCGA-BP-4766-01A-01R-1289-07	stage iii	t3b	female		
TCGA-A3-3349-01A-01R-1188-07	stage i	t1a	male		
TCGA-B8-5165-01A-01R-1420-07	stage iii	t3	male		
TCGA-B0-5701-01A-11R-1541-07	stage i	t1a	female		
TCGA-B0-4712-01A-01R-1503-07	stage iv	t1b	female		
TCGA-A3-A8OV-01A-11R-A37O-07	stage iii	t3b	female		
TCGA-B2-3923-01A-02R-1325-07	stage i	t1a	female		
TCGA-CW-5581-01A-02R-1541-07	stage i	t1b	female		
TCGA-BP-4166-01A-02R-1289-07	stage ii	t2	female		
TCGA-B0-5699-01A-11R-1541-07	stage i	t1b	female		
TCGA-B0-5102-01A-01R-1420-07	stage iv	t4	male		

TCGA-A3-3385-01A-02R-1420-07	stage iii	t3b	male		
TCGA-B0-4837-01A-01R-1305-07	stage iii	t3	male		
TCGA-BP-4982-01A-01R-1334-07	stage i	t1a	male		
TCGA-BP-5185-01A-01R-1426-07	stage i	t1a	female		
TCGA-CJ-6031-01A-11R-1672-07	stage i	t1b	male		
TCGA-CZ-5985-01A-11R-1672-07	stage iv	t3a	female		
TCGA-AK-3465-01A-02R-1325-07	stage i	t1b	male		
TCGA-AK-3434-01A-02R-1277-07	stage i	t1b	male		
TCGA-CJ-5678-01A-11R-1541-07	stage i	t1b	female		
TCGA-BP-4976-01A-01R-1334-07	stage iii	t3a	male		
TCGA-B4-5834-01A-11R-1672-07	stage i	t1b	male		
TCGA-B8-A54E-01A-11R-A266-07	stage i	t1a	male		
TCGA-CW-5588-01A-01R-1541-07	stage ii	t2	male		
TCGA-EU-5907-01A-11R-1672-07	stage i	t1a	male		
TCGA-CJ-4887-01A-01R-1305-07	stage iii	t3b	male		
TCGA-BP-4968-01A-01R-1334-07	stage iii	t3a	male		
TCGA-A3-3357-01A-02R-1420-07	stage i	t1a	male		
TCGA-B0-5706-01A-11R-1541-07	stage i	t1a	female		
TCGA-CJ-4885-01A-01R-1305-07	stage iv	t3	female		
TCGA-B0-4713-01A-01R-1277-07	stage ii	t2	male		
TCGA-B2-4099-01A-02R-1188-07	stage i	t1a	male		
TCGA-B8-A54D-01A-21R-A266-07	stage i	t1a	female		
TCGA-CJ-4638-01A-02R-1325-07	stage i	t1	female		
TCGA-CW-5585-01A-01R-1541-07	stage ii	t2	female		
TCGA-B0-4836-01A-01R-1305-07	stage i	t1b	male		
TCGA-B0-5119-01A-02R-1420-07	stage i	t1	female		
TCGA-CJ-4875-01A-01R-1305-07	stage i	t1b	male		
TCGA-B0-5402-01A-01R-1503-07	stage i	t1b	female		
TCGA-B0-5696-01A-11R-1541-07	stage iii	t3a	male		

TCGA-B0-4817-01A-01R-1277-07	stage ii	t2b	female		
TCGA-CW-5583-01A-02R-1541-07	stage iii	t3a	male		
TCGA-CZ-5468-01A-01R-1503-07	stage i	t1a	female		
TCGA-A3-3331-01A-02R-1325-07	stage i	t1a	female		
TCGA-DV-A4VX-01A-11R-A266-07	stage i	t1a	male		
TCGA-BP-4807-01A-01R-1305-07	stage i	t1a	male		
TCGA-CZ-5458-01A-01R-1503-07	stage i	t1a	male		
TCGA-CJ-4893-01A-01R-1305-07	stage i	t1	male		
TCGA-CZ-4866-01A-01R-1503-07	stage iii	t3b	male		
TCGA-BP-4353-01A-02R-1289-07	stage i	t1b	female		
TCGA-AK-3455-01A-01R-0864-07	stage i	t1b	female		
TCGA-CZ-5470-01A-01R-1503-07	stage i	t1a	male		
TCGA-BP-4762-01A-02R-1289-07	stage i	t1b	male		
TCGA-A3-3328-01A-01R-0864-07	stage i	t1b	male		
TCGA-B0-4814-01A-01R-1277-07	stage ii	t2	male		
TCGA-A3-3363-01A-01R-0864-07	NA	t1a	male		
TCGA-B8-5549-01A-01R-1541-07	stage i	t1a	male		
TCGA-B0-5083-01A-02R-1420-07	stage iv	t4	male		
TCGA-A3-3323-01A-02R-1325-07	stage i	t1a	female		
TCGA-BP-4167-01A-02R-1325-07	stage iii	t3b	male		
TCGA-BP-5194-01A-02R-1426-07	stage i	t1b	female		
TCGA-BP-5181-01A-01R-1426-07	stage i	t1a	female		
TCGA-G6-A5PC-01A-11R-A33J-07	stage ii	t2	male		
TCGA-AK-3436-01A-02R-1325-07	stage i	t1b	male		
TCGA-B0-4839-01A-01R-1305-07	stage ii	t2a	male		
TCGA-BP-5191-01A-01R-1426-07	stage iii	t3b	male		
TCGA-B4-5838-01A-11R-1672-07	stage i	t1b	male		
TCGA-A3-3352-01A-01R-0864-07	stage i	t1a	male		
TCGA-AK-3427-01A-01R-0864-07	stage i	t1a	male		

TCGA-BP-4962-01A-01R-1334-07	stage i	t1b	female		
TCGA-B0-4694-01A-01R-1277-07	stage i	t1	male		
TCGA-CJ-4897-01A-03R-1426-07	stage iv	t3a	female		
TCGA-AK-3443-01A-02R-1325-07	stage i	t1a	male		
TCGA-CJ-4874-01A-01R-1305-07	stage ii	t2a	male		
TCGA-BP-5190-01A-01R-1426-07	stage iii	t3b	female		
TCGA-B0-5702-01A-11R-1541-07	stage i	t1a	male		
TCGA-BP-4329-01A-02R-1289-07	stage ii	t2	male		
TCGA-CZ-4853-01A-01R-1426-07	stage iii	t3a	male		
TCGA-CZ-5451-01A-01R-1503-07	stage iii	t3b	male		
TCGA-BP-5180-01A-01R-1426-07	stage iv	t2	male		
TCGA-CZ-4856-01A-02R-1426-07	stage i	t1b	female		
TCGA-B8-4621-01A-01R-1503-07	stage i	t1	male		
TCGA-B8-4154-01A-01R-1188-07	stage ii	t2a	male		
TCGA-BP-4763-01A-01R-1289-07	stage iii	t3a	male		
TCGA-BP-4343-01A-02R-1289-07	stage i	t1a	male		
TCGA-AK-3451-01A-02R-1188-07	stage i	t1a	male		
TCGA-CZ-5989-01A-11R-1672-07	NA	t3b	male		
TCGA-AK-3458-01A-01R-1503-07	stage i	t1a	female		
TCGA-AK-3447-01A-01R-1766-07	stage i	t1	male		
TCGA-B4-5832-01A-11R-1672-07	stage ii	t2	male		
TCGA-B0-5098-01A-01R-1420-07	stage ii	t2	male		
TCGA-CW-6093-01A-11R-1672-07	stage i	t1a	male		
TCGA-CZ-5982-01A-11R-1672-07	stage i	t1a	male		
TCGA-CJ-4878-01A-01R-1305-07	stage i	t1a	male		
TCGA-BP-4770-01A-01R-1503-07	stage iii	t3a	male		
TCGA-DV-5565-01A-01R-1541-07	stage ii	t2	male		
TCGA-BP-4799-01A-01R-1305-07	stage ii	t2a	female		
TCGA-B8-A7U6-01A-12R-A37O-07	stage i	t1a	female	RIOK1	>75 Percentile

TCGA-B0-4698-01A-01R-1503-07	stage ii	t2	female		
TCGA-BP-4169-01A-02R-1289-07	stage ii	t2	female		
TCGA-A3-3365-01A-01R-0864-07	NA	t1a	female		
TCGA-B0-5693-01A-11R-1541-07	stage i	t1b	male		
TCGA-B0-4701-01A-01R-1277-07	stage i	t1b	female		
TCGA-CZ-5989-01A-11R-1672-07	NA	t3b	male		
TCGA-B0-5109-01A-02R-1420-07	stage ii	t2b	male		
TCGA-BP-5006-01A-01R-1334-07	stage i	t1	male		
TCGA-B0-4852-01A-01R-1503-07	stage ii	t2	female		
TCGA-BP-4774-01A-01R-1289-07	stage iii	t3b	male		
TCGA-B0-4849-01A-01R-1277-07	stage ii	t2	male		
TCGA-BP-5202-01A-02R-1426-07	stage i	t1a	male		
TCGA-BP-4765-01A-01R-1289-07	stage iii	t3a	female		
TCGA-B0-5104-01A-01R-1420-07	stage iii	t3a	male		
TCGA-DV-5574-01A-01R-1541-07	stage iv	t3a	female		
TCGA-B0-5097-01A-01R-1420-07	stage ii	t2	male		
TCGA-B8-A8YJ-01A-13R-A39I-07	stage i	t1a	female		
TCGA-CJ-4908-01A-01R-1426-07	stage iv	t3a	male		
TCGA-CJ-4904-01A-02R-1426-07	stage i	t1b	male		
TCGA-AK-3431-01A-02R-1277-07	stage i	t1b	male		
TCGA-AK-3461-01A-02R-1277-07	stage i	t1a	female		
TCGA-B0-4846-01A-01R-1277-07	stage i	t1	male		
TCGA-B4-5832-01A-11R-1672-07	stage ii	t2	male		
TCGA-BP-4170-01A-02R-1289-07	stage ii	t2	female		
TCGA-BP-5177-01A-01R-1426-07	stage iii	t3a	female		
TCGA-BP-4769-01A-01R-1289-07	stage iii	t3a	male		
TCGA-BP-4974-01A-01R-1334-07	stage iii	t3a	male		
TCGA-BP-5198-01A-01R-1426-07	stage i	t1a	male		
TCGA-AK-3428-01A-02R-1277-07	stage i	t1	male		

TCGA-DV-A4W0-01A-11R-A266-07	stage i	t1a	male		
TCGA-CW-6090-01A-11R-1672-07	stage iii	t3a	male		
TCGA-CZ-4860-01A-01R-1305-07	stage i	t1b	female		
TCGA-CJ-6030-01A-11R-1672-07	stage i	t1b	male		
TCGA-AK-3460-01A-02R-1277-07	stage i	t1b	female		
TCGA-B8-A54J-01A-11R-A33J-07	stage i	t1a	male		
TCGA-B0-4815-01A-01R-1503-07	stage iii	t3b	male		
TCGA-CZ-5457-01A-01R-1503-07	stage i	t1b	female		
TCGA-BP-4763-01A-01R-1289-07	stage iii	t3a	male		
TCGA-A3-A6NJ-01A-12R-A33J-07	stage i	t1a	female		
TCGA-MW-A4EC-01A-11R-A266-07	stage iii	t1a	male		
TCGA-BP-4326-01A-01R-1289-07	stage iii	t3a	male		
TCGA-DV-5575-01A-01R-1541-07	stage i	t1b	male		
TCGA-B0-5812-01A-11R-1672-07	stage i	t1a	female		
TCGA-CJ-4905-01A-02R-1426-07	stage i	t1a	male		
TCGA-CZ-5454-01A-01R-1503-07	stage iv	t3b	female		
TCGA-BP-4344-01A-01R-1289-07	stage iii	t3a	male		
TCGA-B0-5115-01A-01R-1420-07	stage ii	t2	female		
TCGA-BP-4345-01A-01R-1289-07	stage iv	t4	male		
TCGA-BP-5195-01A-02R-1426-07	stage iii	t3b	female		
TCGA-B2-3924-01A-02R-1325-07	stage iii	t3a	male		
TCGA-B8-4148-01A-02R-1325-07	stage i	t1b	male		
TCGA-CZ-5453-01A-01R-1503-07	stage iii	t3a	female		
TCGA-B0-4688-01A-01R-1277-07	stage i	t1b	male		
TCGA-CJ-4890-01A-01R-1305-07	stage i	t1b	female		
TCGA-B0-4811-01A-01R-1503-07	stage ii	t2	male		
TCGA-B0-5110-01A-01R-1420-07	stage i	t1b	female		
TCGA-BP-4353-01A-02R-1289-07	stage i	t1b	female		
TCGA-BP-4784-01A-01R-1305-07	stage ii	t2	female		

TCGA-B0-5113-01A-01R-1420-07	stage i	t1	male		
TCGA-B0-5713-01A-11R-1672-07	stage iii	t3a	female		
TCGA-B0-5703-01A-11R-1541-07	stage i	t1a	male		
TCGA-B8-5162-01A-01R-1420-07	stage i	t1a	female		
TCGA-CJ-4899-01A-01R-1334-07	stage i	t1b	female		
TCGA-GK-A6C7-01A-11R-A33J-07	stage iii	t3a	male		
TCGA-BP-4354-01A-02R-1289-07	stage iv	t3b	female		
TCGA-BP-4347-01A-01R-1289-07	stage iv	t2	male		
TCGA-BP-5196-01A-01R-1426-07	stage iii	t3a	male		
TCGA-BP-4343-01A-02R-1289-07	stage i	t1a	male		
TCGA-BP-4789-01A-01R-1305-07	stage iii	t3b	female		
TCGA-DV-5569-01A-01R-1541-07	stage iii	t3c	female		
TCGA-CJ-6033-01A-11R-1672-07	stage i	t1b	female		
TCGA-MM-A564-01A-11R-A266-07	stage i	t1a	female		
TCGA-CZ-5460-01A-01R-1503-07	stage iii	t3a	male		
TCGA-CW-6097-01A-11R-1672-07	stage iii	t3a	male		
TCGA-B0-5698-01A-11R-1672-07	stage i	t1a	female		
TCGA-B4-5835-01A-11R-1672-07	stage i	t1b	female		
TCGA-CJ-4872-01A-01R-1305-07	stage i	t1b	female		
TCGA-BP-4329-01A-02R-1289-07	stage ii	t2	male		
TCGA-B0-4945-01A-01R-1420-07	stage i	t1a	female		
TCGA-CJ-4885-01A-01R-1305-07	stage iv	t3	female		
TCGA-CZ-5455-01A-01R-1503-07	stage iv	t4	male		
TCGA-B0-4816-01A-01R-1503-07	stage iv	t3b	male		
TCGA-CJ-4920-01A-01R-1426-07	stage i	t1a	male		
TCGA-CJ-4876-01A-01R-1305-07	stage i	t1b	male		
TCGA-B2-4099-01A-02R-1188-07	stage i	t1a	male		
TCGA-B0-4690-01A-01R-1277-07	stage iii	t3a	male		
TCGA-BP-4993-01A-02R-1420-07	stage i	t1a	male		

TCGA-BP-5008-01A-01R-1334-07	stage iii	t3a	male		
TCGA-A3-A8CQ-01A-11R-A37O-07	stage i	t1a	female		
TCGA-CZ-5463-01A-01R-1503-07	stage i	t1a	female		
TCGA-B0-4838-01A-01R-1305-07	stage i	t1b	male		
TCGA-CZ-5461-01A-01R-1503-07	stage i	t1a	female		
TCGA-BP-4961-01A-01R-1334-07	stage i	t1b	male		
TCGA-CJ-4918-01A-01R-1426-07	stage iii	t3a	male		
TCGA-B0-4819-01A-01R-1277-07	stage iii	t3a	male		
TCGA-B4-5834-01A-11R-1672-07	stage i	t1b	male		
TCGA-B0-4845-01A-01R-1277-07	stage i	t1a	female		
TCGA-CZ-5988-01A-11R-1672-07	stage iii	t3b	male		
TCGA-BP-5200-01A-01R-1426-07	stage i	t1	male		
TCGA-B4-5378-01A-01R-1503-07	stage ii	t2	male		
TCGA-CW-6087-01A-11R-1672-07	stage i	t1b	female		
TCGA-CW-6088-01A-11R-1672-07	stage i	t1b	female		
TCGA-A3-3313-01A-02R-1325-07	stage i	t1a	female		
TCGA-CJ-4643-01A-02R-1325-07	stage i	t1b	male		
TCGA-BP-5004-01A-01R-1334-07	stage i	t1	female		
TCGA-BP-4798-01A-01R-1305-07	stage i	t1a	female		
TCGA-AK-3425-01A-02R-1277-07	stage i	t1b	male		
TCGA-3Z-A93Z-01A-11R-A37O-07	stage i	t1b	female		
TCGA-B8-5163-01A-01R-1420-07	stage i	t1b	male		
TCGA-BP-5010-01A-02R-1420-07	stage i	t1	female		
TCGA-BP-4998-01A-01R-1334-07	stage iii	t3a	male		
TCGA-CJ-4903-01A-01R-1426-07	stage i	t1a	female		
TCGA-BP-5194-01A-02R-1426-07	stage i	t1b	female		
TCGA-B0-4696-01A-01R-1277-07	stage iii	t3b	female		
TCGA-B8-4620-01A-02R-1325-07	stage ii	t2	male		
TCGA-CJ-4894-01A-01R-1305-07	stage i	t1a	male		

TCGA-CZ-4865-01A-02R-1503-07	stage iii	t3a	male		
TCGA-A3-3347-01A-02R-1325-07	stage i	t1a	female		
TCGA-DV-5566-01A-01R-1541-07	stage iv	t3b	male		
TCGA-G6-A8L6-01A-11R-A37O-07	stage i	t1b	male		
TCGA-BP-5187-01A-01R-1426-07	stage i	t1b	male		
TCGA-BP-4162-01A-02R-1325-07	stage iii	t3a	male		
TCGA-A3-3323-01A-02R-1325-07	stage i	t1a	female		
TCGA-CW-5587-01A-01R-1541-07	stage i	t1b	female		
TCGA-MM-A563-01A-11R-A266-07	stage i	t1b	male		
TCGA-B0-5697-01A-11R-1541-07	stage i	t1b	female		
TCGA-B8-5551-01A-01R-1541-07	stage i	t1	male		
TCGA-BP-4992-01A-01R-1334-07	stage iv	t1a	female		
TCGA-CZ-5468-01A-01R-1503-07	stage i	t1a	female		
TCGA-CZ-5470-01A-01R-1503-07	stage i	t1a	male		
TCGA-B8-5550-01A-01R-1541-07	stage i	t1	male		
TCGA-B8-5553-01A-01R-1541-07	stage i	t1	male		
TCGA-A3-3378-01A-02R-1325-07	stage i	t1b	female		
TCGA-B2-5633-01A-01R-1541-07	stage i	t1a	male		
TCGA-B8-4143-01A-01R-1188-07	stage i	t1	female		
TCGA-BP-4972-01A-01R-1334-07	stage iv	t3a	male		
TCGA-B0-5692-01A-11R-1541-07	stage ii	t2b	female		
TCGA-6D-AA2E-01A-11R-A37O-07	stage i	t1b	male		
TCGA-B0-5094-01A-01R-1420-07	stage ii	t2b	female		
TCGA-BP-5174-01A-01R-1426-07	stage iii	t3a	male		
TCGA-B0-5107-01A-01R-1420-07	stage ii	t2	male		
TCGA-A3-3385-01A-02R-1420-07	stage iii	t3b	male		
TCGA-B8-A54E-01A-11R-A266-07	stage i	t1a	male		
TCGA-BP-4994-01A-01R-1334-07	stage iv	t3b	male		
TCGA-B0-4821-01A-01R-1503-07	stage iii	t3a	male		<25 Percentile

TCGA-AK-3444-01A-02R-1325-07	stage i	t1b	male			
TCGA-CZ-5451-01A-01R-1503-07	stage iii	t3b	male			
TCGA-BP-4342-01A-01R-1289-07	stage i	t1b	female			
TCGA-BP-4352-01A-01R-1289-07	stage iii	t3a	male			
TCGA-DV-5568-01A-01R-1541-07	stage ii	t2	male			
TCGA-B2-5641-01A-01R-1541-07	stage iii	t3b	female			
TCGA-CZ-5459-01A-01R-1503-07	stage i	t1a	male			
TCGA-B0-4713-01A-01R-1277-07	stage ii	t2	male			
TCGA-CZ-5985-01A-11R-1672-07	stage iv	t3a	female			
TCGA-B0-5083-01A-02R-1420-07	stage iv	t4	male			
TCGA-AK-3440-01A-02R-1277-07	stage i	t1a	male			
TCGA-BP-4969-01A-01R-1334-07	stage iv	t3a	male			
TCGA-A3-3383-01A-02R-1325-07	stage iii	t3a	male			
TCGA-B2-4098-01A-02R-1325-07	stage ii	t2	male			
TCGA-BP-4790-01A-01R-1305-07	stage ii	t2	male			
TCGA-BP-4756-01A-01R-1289-07	stage iv	t4	male			
TCGA-CJ-4635-01A-02R-1305-07	stage i	t1b	male			
TCGA-B0-5696-01A-11R-1541-07	stage iii	t3a	male			
TCGA-AK-3433-01A-02R-1277-07	stage i	t1b	female			
TCGA-T7-A92I-01A-11R-A37O-07	stage iii	t3a	male			
TCGA-BP-4797-01A-01R-1305-07	stage i	t1a	male			
TCGA-A3-3346-01A-01R-1766-07	stage i	t1a	male			
TCGA-B0-4827-01A-02R-1420-07	stage ii	t2	male			
TCGA-B8-A54K-01A-11R-A33J-07	stage iii	t3b	female			
TCGA-BP-4967-01A-01R-1334-07	stage iii	t3a	female			
TCGA-BP-4787-01A-01R-1305-07	stage iv	t3b	female			
TCGA-B0-4844-01A-01R-1277-07	stage i	t1a	female			
TCGA-CZ-5469-01A-01R-1503-07	stage i	t1a	male			
TCGA-B0-5699-01A-11R-1541-07	stage i	t1b	female			

TCGA-MM-A84U-01A-11R-A37O-07	stage i	t1b	male		
TCGA-B2-5635-01A-01R-1541-07	stage i	t1a	female		
TCGA-A3-3382-01A-02R-1325-07	stage i	t1	male		
TCGA-CW-5583-01A-02R-1541-07	stage iii	t3a	male		
TCGA-CJ-5681-01A-11R-1541-07	stage i	t1a	female		
TCGA-B0-5701-01A-11R-1541-07	stage i	t1a	female		
TCGA-BP-5000-01A-01R-1334-07	stage iii	t3a	female		
TCGA-BP-5181-01A-01R-1426-07	stage i	t1a	female		
TCGA-CJ-4873-01A-01R-1305-07	stage i	t1a	male		
TCGA-AS-3778-01A-01R-A32Z-07	stage i	t1a	male		
TCGA-BP-4976-01A-01R-1334-07	stage iii	t3a	male		
TCGA-B0-5100-01A-01R-1420-07	stage i	t1a	male		
TCGA-CJ-4887-01A-01R-1305-07	stage iii	t3b	male		
TCGA-B8-5549-01A-01R-1541-07	stage i	t1a	male		
TCGA-BP-4351-01A-01R-1289-07	stage iii	t3b	male		
TCGA-B0-5077-01A-01R-1334-07	stage i	t1b	female		
TCGA-B8-A54I-01A-21R-A33J-07	stage i	t1b	female		
TCGA-CJ-4869-01A-02R-1426-07	stage iii	t3b	female		
TCGA-CJ-4897-01A-03R-1426-07	stage iv	t3a	female		
TCGA-B8-5158-01A-01R-1420-07	stage ii	t2	male		
TCGA-CW-5581-01A-02R-1541-07	stage i	t1b	female		
TCGA-BP-4977-01A-01R-1334-07	stage i	t1a	female		
TCGA-CW-5585-01A-01R-1541-07	stage ii	t2	female		
TCGA-BP-4327-01A-01R-1289-07	stage i	t1b	male		
TCGA-B0-5075-01A-01R-1334-07	stage i	t1b	male		
TCGA-A3-3306-01A-01R-0864-07	stage i	t1a	female		
TCGA-BP-4807-01A-01R-1305-07	stage i	t1a	male		
TCGA-EU-5907-01A-11R-1672-07	stage i	t1a	male		
TCGA-BP-5001-01A-01R-1334-07	stage iii	t3b	female		

TCGA-AK-3456-01A-02R-1325-07	stage i	t1a	male		
TCGA-AK-3451-01A-02R-1188-07	stage i	t1a	male		
TCGA-CJ-5680-01A-11R-1541-07	stage i	t1b	female		
TCGA-CZ-5984-01A-11R-1672-07	stage i	t1a	female		
TCGA-DV-A4VX-01A-11R-A266-07	stage i	t1a	male		
TCGA-CJ-4638-01A-02R-1325-07	stage i	t1	female		
TCGA-B4-5838-01A-11R-1672-07	stage i	t1b	male		
TCGA-BP-5191-01A-01R-1426-07	stage iii	t3b	male		
TCGA-BP-5180-01A-01R-1426-07	stage iv	t2	male		
TCGA-CW-5588-01A-01R-1541-07	stage ii	t2	male		
TCGA-BP-4985-01A-01R-1334-07	stage iii	t3b	female		
TCGA-B0-4839-01A-01R-1305-07	stage ii	t2a	male		
TCGA-CZ-5987-01A-11R-1672-07	stage i	t1a	male		
TCGA-CW-5591-01A-01R-1541-07	stage i	t1a	male		
TCGA-B0-4836-01A-01R-1305-07	stage i	t1b	male		
TCGA-CJ-5679-01A-11R-1541-07	stage iii	t3a	male		
TCGA-CJ-5672-01A-11R-1541-07	stage i	t1b	male		
TCGA-B0-5400-01A-01R-1503-07	stage i	t1a	female		
TCGA-CZ-5456-01A-01R-1503-07	stage iii	t3a	female		
TCGA-B0-5707-01A-11R-1541-07	stage i	t1b	male		
TCGA-AK-3434-01A-02R-1277-07	stage i	t1b	male		
TCGA-EU-5905-01A-11R-1672-07	stage i	t1b	male		
TCGA-BP-4775-01A-01R-1289-07	stage i	t1a	male		
TCGA-B8-5546-01A-01R-1541-07	stage i	t1b	female		
TCGA-BP-4959-01A-01R-1334-07	stage i	t1a	male		
TCGA-B2-3923-01A-02R-1325-07	stage i	t1a	female		
TCGA-AK-3453-01A-02R-1277-07	stage ii	t2b	female		
TCGA-A3-3335-01A-01R-0864-07	stage i	t1a	male		
TCGA-AK-3465-01A-02R-1325-07	stage i	t1b	male		

TCGA-B0-5102-01A-01R-1420-07	stage iv	t4	male		
TCGA-B8-4621-01A-01R-1503-07	stage i	t1	male		
TCGA-B0-4694-01A-01R-1277-07	stage i	t1	male		
TCGA-B0-5117-01A-01R-1420-07	stage iii	t3a	male		
TCGA-B0-4817-01A-01R-1277-07	stage ii	t2b	female		
TCGA-BP-5175-01A-01R-1426-07	stage iii	t3b	male		
TCGA-AK-3426-01A-02R-1325-07	stage i	t1b	female		
TCGA-AK-3436-01A-02R-1325-07	stage i	t1b	male		
TCGA-A3-3319-01A-02R-1325-07	stage i	t1a	male		
TCGA-B0-4842-01A-02R-1420-07	stage i	t1a	female		
TCGA-BP-5185-01A-01R-1426-07	stage i	t1a	female		
TCGA-G6-A5PC-01A-11R-A33J-07	stage ii	t2	male		
TCGA-CZ-4853-01A-01R-1426-07	stage iii	t3a	male		
TCGA-BP-4962-01A-01R-1334-07	stage i	t1b	female		
TCGA-G6-A8L7-01A-11R-A37O-07	stage i	t1a	male		
TCGA-B0-4707-01A-01R-1277-07	stage ii	t2	male		
TCGA-AS-3777-01A-01R-0864-07	stage i	t1b	male		
TCGA-BP-4334-01A-01R-1289-07	stage ii	t2	male		
TCGA-B0-5702-01A-11R-1541-07	stage i	t1a	male		
TCGA-AK-3447-01A-01R-1766-07	stage i	t1	male		
TCGA-A3-3374-01A-02R-1325-07	stage iii	t3a	female		
TCGA-BP-4761-01A-01R-1289-07	stage iv	t3a	male		
TCGA-6D-AA2E-01A-11R-A37O-07	stage i	t1b	male		
TCGA-BP-5200-01A-01R-1426-07	stage i	t1	male		
TCGA-B0-4699-01A-01R-1277-07	stage iii	t3a	male		
TCGA-DV-A4W0-01A-11R-A266-07	stage i	t1a	male		
TCGA-B0-5099-01A-01R-1420-07	stage i	t1a	female		
TCGA-BP-5008-01A-01R-1334-07	stage iii	t3a	male		
TCGA-BP-4326-01A-01R-1289-07	stage iii	t3a	male	BHLHE40	>75 Percentile

TCGA-B0-5077-01A-01R-1334-07	stage i	t1b	female		
TCGA-BP-4960-01A-01R-1334-07	stage iv	t3b	male		
TCGA-BP-4169-01A-02R-1289-07	stage ii	t2	female		
TCGA-CZ-5988-01A-11R-1672-07	stage iii	t3b	male		
TCGA-EU-5904-01A-11R-1672-07	stage i	t1b	male		
TCGA-BP-4355-01A-01R-1289-07	stage iv	t4	male		
TCGA-B0-5085-01A-01R-1334-07	stage iii	t3a	female		
TCGA-B8-A7U6-01A-12R-A37O-07	stage i	t1a	female		
TCGA-BP-4770-01A-01R-1503-07	stage iii	t3a	male		
TCGA-CJ-4901-01A-01R-1426-07	stage iii	t3a	female		
TCGA-B8-A8YJ-01A-13R-A39I-07	stage i	t1a	female		
TCGA-BP-4769-01A-01R-1289-07	stage iii	t3a	male		
TCGA-B0-4710-01A-01R-1503-07	stage i	t1b	male		
TCGA-AK-3453-01A-02R-1277-07	stage ii	t2b	female		
TCGA-AK-3440-01A-02R-1277-07	stage i	t1a	male		
TCGA-BP-4335-01A-01R-1289-07	stage i	t1b	male		
TCGA-DV-5567-01A-01R-1541-07	stage iii	t3a	male		
TCGA-B0-4846-01A-01R-1277-07	stage i	t1	male		
TCGA-BP-4974-01A-01R-1334-07	stage iii	t3a	male		
TCGA-B8-5545-01A-01R-1672-07	stage i	t1b	female		
TCGA-BP-4994-01A-01R-1334-07	stage iv	t3b	male		
TCGA-CJ-4882-01A-02R-1426-07	stage i	t1a	male		
TCGA-CW-6093-01A-11R-1672-07	stage i	t1a	male		
TCGA-BP-4760-01A-02R-1420-07	stage i	t1b	male		
TCGA-CJ-4920-01A-01R-1426-07	stage i	t1a	male		
TCGA-BP-4162-01A-02R-1325-07	stage iii	t3a	male		
TCGA-BP-4340-01A-01R-1289-07	stage i	t1b	female		
TCGA-BP-4776-01A-01R-1289-07	stage iv	t4	male		
TCGA-CZ-5986-01A-11R-1672-07	stage i	t1a	male		

TCGA-B0-5700-01A-11R-1541-07	stage i	t1a	male		
TCGA-BP-4352-01A-01R-1289-07	stage iii	t3a	male		
TCGA-B0-5117-01A-01R-1420-07	stage iii	t3a	male		
TCGA-BP-4795-01A-02R-1420-07	stage ii	t2a	male		
TCGA-B0-5088-01A-01R-1334-07	stage i	t1a	male		
TCGA-BP-4799-01A-01R-1305-07	stage ii	t2a	female		
TCGA-B0-5115-01A-01R-1420-07	stage ii	t2	female		
TCGA-B0-4822-01A-01R-1277-07	stage ii	t2b	male		
TCGA-CZ-5459-01A-01R-1503-07	stage i	t1a	male		
TCGA-B0-4852-01A-01R-1503-07	stage ii	t2	female		
TCGA-CJ-5671-01A-11R-1541-07	stage i	t1b	female		
TCGA-CJ-4868-01A-01R-1305-07	stage iv	t2	female		
TCGA-B0-4718-01A-01R-1277-07	stage iii	t3a	male		
TCGA-BP-4170-01A-02R-1289-07	stage ii	t2	female		
TCGA-A3-3328-01A-01R-0864-07	stage i	t1b	male		
TCGA-DV-5576-01A-01R-1541-07	stage i	t1a	male		
TCGA-B0-4813-01A-01R-1277-07	stage iii	t3a	male		
TCGA-B0-5116-01A-02R-1420-07	stage i	t1	female		
TCGA-BP-4971-01A-01R-1334-07	stage iv	t3a	male		
TCGA-BP-4995-01A-01R-1334-07	stage ii	t2	male		
TCGA-DV-5575-01A-01R-1541-07	stage i	t1b	male		
TCGA-BP-5187-01A-01R-1426-07	stage i	t1b	male		
TCGA-B0-5109-01A-02R-1420-07	stage ii	t2b	male		
TCGA-CJ-4878-01A-01R-1305-07	stage i	t1a	male		
TCGA-A3-3313-01A-02R-1325-07	stage i	t1a	female		
TCGA-B0-4814-01A-01R-1277-07	stage ii	t2	male		
TCGA-DV-A4VZ-01A-11R-A266-07	stage iii	t3a	male		
TCGA-BP-5170-01A-01R-1426-07	stage i	t1a	male		
TCGA-BP-5201-01A-01R-1426-07	stage i	t1a	male		

TCGA-CJ-4876-01A-01R-1305-07	stage i	t1b	male		
TCGA-BP-4993-01A-02R-1420-07	stage i	t1a	male		
TCGA-CW-6090-01A-11R-1672-07	stage iii	t3a	male		
TCGA-B0-5098-01A-01R-1420-07	stage ii	t2	male		
TCGA-B0-5713-01A-11R-1672-07	stage iii	t3a	female		
TCGA-DV-5569-01A-01R-1541-07	stage iii	t3c	female		
TCGA-B0-5080-01A-01R-1503-07	stage i	t1b	male		
TCGA-B0-5108-01A-01R-1420-07	stage iii	t3a	male		
TCGA-B0-4823-01A-02R-1420-07	stage ii	t2	female		
TCGA-B0-5102-01A-01R-1420-07	stage iv	t4	male		
TCGA-GK-A6C7-01A-11R-A33J-07	stage iii	t3a	male		
TCGA-B4-5378-01A-01R-1503-07	stage ii	t2	male		
TCGA-BP-4973-01A-01R-1334-07	stage iii	t3b	male		
TCGA-B8-A54J-01A-11R-A33J-07	stage i	t1a	male		
TCGA-B0-4841-01A-01R-1277-07	stage i	t1b	male		
TCGA-B0-4833-01A-01R-1305-07	stage i	t1a	male		
TCGA-BP-4173-01A-02R-1289-07	stage i	t1b	male		
TCGA-CZ-5468-01A-01R-1503-07	stage i	t1a	female		
TCGA-B0-5693-01A-11R-1541-07	stage i	t1b	male		
TCGA-BP-5199-01A-01R-1426-07	stage i	t1a	male		
TCGA-BP-4968-01A-01R-1334-07	stage iii	t3a	male		
TCGA-B0-4811-01A-01R-1503-07	stage ii	t2	male		
TCGA-B0-4819-01A-01R-1277-07	stage iii	t3a	male		
TCGA-BP-4774-01A-01R-1289-07	stage iii	t3b	male		
TCGA-BP-4345-01A-01R-1289-07	stage iv	t4	male		
TCGA-CJ-6030-01A-11R-1672-07	stage i	t1b	male		
TCGA-CJ-4635-01A-02R-1305-07	stage i	t1b	male		
TCGA-BP-4765-01A-01R-1289-07	stage iii	t3a	female		
TCGA-B0-5104-01A-01R-1420-07	stage iii	t3a	male		

TCGA-CZ-5467-01A-01R-1503-07	stage iv	t3a	male		
TCGA-BP-4334-01A-01R-1289-07	stage ii	t2	male		
TCGA-B0-5106-01A-01R-1420-07	stage i	t1	female		
TCGA-BP-4756-01A-01R-1289-07	stage iv	t4	male		
TCGA-CZ-4860-01A-01R-1305-07	stage i	t1b	female		
TCGA-CJ-4904-01A-02R-1426-07	stage i	t1b	male		
TCGA-BP-4998-01A-01R-1334-07	stage iii	t3a	male		
TCGA-CJ-4903-01A-01R-1426-07	stage i	t1a	female		
TCGA-BP-5010-01A-02R-1420-07	stage i	t1	female		
TCGA-BP-4332-01A-01R-1289-07	stage ii	t2	female		
TCGA-BP-4983-01A-01R-1334-07	stage iv	t3a	male		
TCGA-BP-5196-01A-01R-1426-07	stage iii	t3a	male		
TCGA-CJ-4912-01A-01R-1426-07	stage iii	t3a	male		
TCGA-CZ-5456-01A-01R-1503-07	stage iii	t3a	female		
TCGA-CJ-4899-01A-01R-1334-07	stage i	t1b	female		
TCGA-B0-4690-01A-01R-1277-07	stage iii	t3a	male		
TCGA-BP-4775-01A-01R-1289-07	stage i	t1a	male		
TCGA-B0-5698-01A-11R-1672-07	stage i	t1a	female		
TCGA-B8-5546-01A-01R-1541-07	stage i	t1b	female		
TCGA-BP-5006-01A-01R-1334-07	stage i	t1	male		
TCGA-BP-5168-01A-01R-1420-07	stage i	t1a	male		
TCGA-CJ-6032-01A-11R-1672-07	stage i	t1b	male		
TCGA-B0-5097-01A-01R-1420-07	stage ii	t2	male		
TCGA-DV-5566-01A-01R-1541-07	stage iv	t3b	male		
TCGA-A3-3331-01A-02R-1325-07	stage i	t1a	female		
TCGA-BP-4174-01A-02R-1289-07	stage iv	t2	male		
TCGA-A3-A6NJ-01A-12R-A33J-07	stage i	t1a	female		
TCGA-B0-4843-01A-01R-1277-07	stage i	t1a	female		
TCGA-CZ-5453-01A-01R-1503-07	stage iii	t3a	female		

TCGA-B0-5113-01A-01R-1420-07	stage i	t1	male		
TCGA-BP-5192-01A-01R-1426-07	stage iv	t4	male		
TCGA-BP-4341-01A-01R-1289-07	stage i	t1a	male		
TCGA-BP-4763-01A-01R-1289-07	stage iii	t3a	male		
TCGA-B0-4849-01A-01R-1277-07	stage ii	t2	male		
TCGA-EU-5906-01A-11R-1672-07	stage ii	t2	male		
TCGA-BP-4784-01A-01R-1305-07	stage ii	t2	female		
TCGA-BP-5173-01A-01R-1426-07	stage iv	t2	female		
TCGA-B0-4706-01A-01R-1503-07	stage ii	t2	female		
TCGA-AK-3465-01A-02R-1325-07	stage i	t1b	male		
TCGA-A3-A6NL-01A-11R-A33J-07	stage i	t1b	male		
TCGA-B0-5092-01A-01R-1420-07	stage ii	t2	female		
TCGA-CW-5589-01A-01R-1541-07	stage ii	t2	male		
TCGA-AK-3425-01A-02R-1277-07	stage i	t1b	male		
TCGA-CZ-4856-01A-02R-1426-07	stage i	t1b	female		
TCGA-B0-4827-01A-02R-1420-07	stage ii	t2	male		
TCGA-CJ-4873-01A-01R-1305-07	stage i	t1a	male		
TCGA-CW-5587-01A-01R-1541-07	stage i	t1b	female		
TCGA-B8-5165-01A-01R-1420-07	stage iii	t3	male		
TCGA-BP-4787-01A-01R-1305-07	stage iv	t3b	female		
TCGA-B8-4151-01A-01R-1188-07	stage iii	t1b	female		
TCGA-B8-4622-01A-02R-1277-07	stage i	t1a	female		
TCGA-B0-5696-01A-11R-1541-07	stage iii	t3a	male		
TCGA-B8-A54E-01A-11R-A266-07	stage i	t1a	male		
TCGA-CW-6087-01A-11R-1672-07	stage i	t1b	female		
TCGA-3Z-A93Z-01A-11R-A37O-07	stage i	t1b	female		
TCGA-B8-4143-01A-01R-1188-07	stage i	t1	female		
TCGA-CJ-4892-01A-01R-1305-07	stage iv	t3	male		
TCGA-CJ-4874-01A-01R-1305-07	stage ii	t2a	male		<25 Percentile

TCGA-A3-3385-01A-02R-1420-07	stage iii	t3b	male		
TCGA-CJ-5682-01A-11R-1541-07	stage i	t1a	male		
TCGA-BP-4963-01A-01R-1334-07	stage i	t1b	female		
TCGA-CZ-5464-01A-01R-1503-07	stage i	t1a	female		
TCGA-AK-3460-01A-02R-1277-07	stage i	t1b	female		
TCGA-A3-A6NI-01A-11R-A33J-07	stage iii	t3a	male		
TCGA-B2-3924-01A-02R-1325-07	stage iii	t3a	male		
TCGA-B2-5639-01A-01R-1541-07	stage i	t1b	male		
TCGA-B0-4713-01A-01R-1277-07	stage ii	t2	male		
TCGA-BP-4342-01A-01R-1289-07	stage i	t1b	female		
TCGA-B0-4836-01A-01R-1305-07	stage i	t1b	male		
TCGA-A3-3378-01A-02R-1325-07	stage i	t1b	female		
TCGA-B4-5843-01A-11R-1672-07	stage i	t1b	male		
TCGA-CZ-5461-01A-01R-1503-07	stage i	t1a	female		
TCGA-AK-3444-01A-02R-1325-07	stage i	t1b	male		
TCGA-BP-4991-01A-01R-1334-07	stage iii	t3a	female		
TCGA-B4-5834-01A-11R-1672-07	stage i	t1b	male		
TCGA-BP-4999-01A-01R-1334-07	stage i	t1a	female		
TCGA-CW-5585-01A-01R-1541-07	stage ii	t2	female		
TCGA-CZ-5984-01A-11R-1672-07	stage i	t1a	female		
TCGA-BP-4962-01A-01R-1334-07	stage i	t1b	female		
TCGA-CJ-4887-01A-01R-1305-07	stage iii	t3b	male		
TCGA-BP-4330-01A-01R-1289-07	stage ii	t2	male		
TCGA-CJ-5680-01A-11R-1541-07	stage i	t1b	female		
TCGA-CZ-5466-01A-01R-1503-07	stage iv	t4	female		
TCGA-BP-4762-01A-02R-1289-07	stage i	t1b	male		
TCGA-CZ-5985-01A-11R-1672-07	stage iv	t3a	female		
TCGA-B8-5553-01A-01R-1541-07	stage i	t1	male		
TCGA-MM-A84U-01A-11R-A37O-07	stage i	t1b	male		

TCGA-CJ-4644-01A-02R-1325-07	stage iii	t3b	male		
TCGA-BP-5183-01A-01R-1426-07	stage i	t1b	male		
TCGA-BP-5000-01A-01R-1334-07	stage iii	t3a	female		
TCGA-MM-A564-01A-11R-A266-07	stage i	t1a	female		
TCGA-CJ-5672-01A-11R-1541-07	stage i	t1b	male		
TCGA-BP-4759-01A-01R-1289-07	stage iv	t3a	female		
TCGA-B0-5691-01A-11R-1541-07	stage i	t1b	male		
TCGA-AK-3450-01A-02R-1277-07	stage i	t1b	male		
TCGA-B0-4848-01A-01R-1277-07	stage ii	t2	female		
TCGA-AS-3777-01A-01R-0864-07	stage i	t1b	male		
TCGA-BP-4159-01A-02R-1289-07	stage i	t1a	male		
TCGA-BP-4343-01A-02R-1289-07	stage i	t1a	male		
TCGA-BP-4166-01A-02R-1289-07	stage ii	t2	female		
TCGA-BP-5004-01A-01R-1334-07	stage i	t1	female		
TCGA-B8-A54G-01A-11R-A266-07	stage i	t1a	female		
TCGA-DV-A4VX-01A-11R-A266-07	stage i	t1a	male		
TCGA-B2-4102-01A-02R-1325-07	stage i	t1b	female		
TCGA-A3-3367-01A-02R-1420-07	stage i	t1a	male		
TCGA-CZ-4866-01A-01R-1503-07	stage iii	t3b	male		
TCGA-BP-5178-01A-01R-1426-07	stage i	t1a	female		
TCGA-CZ-5989-01A-11R-1672-07	NA	t3b	male		
TCGA-B8-A54D-01A-21R-A266-07	stage i	t1a	female		
TCGA-CW-5584-01A-01R-1541-07	stage iii	t3a	male		
TCGA-AK-3434-01A-02R-1277-07	stage i	t1b	male		
TCGA-AK-3447-01A-01R-1766-07	stage i	t1	male		
TCGA-B2-A4SR-01A-11R-A266-07	stage i	t1	male		
TCGA-B0-5083-01A-02R-1420-07	stage iv	t4	male		
TCGA-BP-5189-01A-02R-1426-07	stage i	t1a	female		
TCGA-A3-A8OW-01A-11R-A37O-07	stage iii	t3	male		

TCGA-B0-5084-01A-01R-1334-07	stage ii	t2	male		
TCGA-A3-3323-01A-02R-1325-07	stage i	t1a	female		
TCGA-B0-5710-01A-11R-1672-07	stage i	t1b	female		
TCGA-B4-5836-01A-11R-1672-07	stage i	t1a	male		
TCGA-BP-4349-01A-01R-1289-07	stage iii	t3a	female		
TCGA-B0-4842-01A-02R-1420-07	stage i	t1a	female		
TCGA-B0-5402-01A-01R-1503-07	stage i	t1b	female		
TCGA-BP-4961-01A-01R-1334-07	stage i	t1b	male		
TCGA-CW-5590-01A-01R-1541-07	stage i	t1b	male		
TCGA-B4-5835-01A-11R-1672-07	stage i	t1b	female		
TCGA-A3-3387-01A-01R-1541-07	stage i	t1a	male		
TCGA-A3-3343-01A-01R-0864-07	stage i	t1a	female		
TCGA-AK-3455-01A-01R-0864-07	stage i	t1b	female		
TCGA-BP-5191-01A-01R-1426-07	stage iii	t3b	male		
TCGA-CZ-5452-01A-01R-1503-07	stage i	t1a	female		
TCGA-G6-A5PC-01A-11R-A33J-07	stage ii	t2	male		
TCGA-BP-4782-01A-02R-1420-07	stage i	t1b	male		
TCGA-BP-5169-01A-01R-1426-07	stage i	t1	female		
TCGA-CJ-4888-01A-01R-1305-07	stage i	t1	male		
TCGA-A3-3347-01A-02R-1325-07	stage i	t1a	female		
TCGA-B2-5633-01A-01R-1541-07	stage i	t1a	male		
TCGA-B0-5107-01A-01R-1420-07	stage ii	t2	male		
TCGA-B8-4621-01A-01R-1503-07	stage i	t1	male		
TCGA-T7-A92I-01A-11R-A37O-07	stage iii	t3a	male		
TCGA-A3-3370-01A-02R-1420-07	stage i	t1a	female		
TCGA-A3-3382-01A-02R-1325-07	stage i	t1	male		
TCGA-A3-3306-01A-01R-0864-07	stage i	t1a	female		
TCGA-CW-5591-01A-01R-1541-07	stage i	t1a	male		
TCGA-A3-3380-01A-01R-0864-07	stage i	t1b	male		

TCGA-AK-3445-01A-02R-1277-07	stage i	t1a	male		
TCGA-A3-3326-01A-01R-0864-07	stage i	t1b	female		
TCGA-B2-4101-01A-02R-1277-07	stage ii	t2a	male		
TCGA-CZ-5451-01A-01R-1503-07	stage iii	t3b	male		
TCGA-BP-4798-01A-01R-1305-07	stage i	t1a	female		
TCGA-B8-4619-01A-02R-1325-07	stage iii	t3a	male		
TCGA-AK-3426-01A-02R-1325-07	stage i	t1b	female		
TCGA-AK-3433-01A-02R-1277-07	stage i	t1b	female		
TCGA-AK-3451-01A-02R-1188-07	stage i	t1a	male		
TCGA-BP-5007-01A-01R-1334-07	stage iii	t3b	female		
TCGA-B8-5159-01A-01R-1420-07	stage i	t1a	male		
TCGA-G6-A8L7-01A-11R-A37O-07	stage i	t1a	male		
TCGA-BP-5190-01A-01R-1426-07	stage iii	t3b	female		
TCGA-B0-5702-01A-11R-1541-07	stage i	t1a	male		
TCGA-B4-5838-01A-11R-1672-07	stage i	t1b	male		
TCGA-AK-3427-01A-01R-0864-07	stage i	t1a	male		
TCGA-AK-3436-01A-02R-1325-07	stage i	t1b	male		
TCGA-AK-3443-01A-02R-1325-07	stage i	t1a	male		
TCGA-CZ-5458-01A-01R-1503-07	stage i	t1a	male		
TCGA-AK-3458-01A-01R-1503-07	stage i	t1a	female		
TCGA-B0-5701-01A-11R-1541-07	stage i	t1a	female		
TCGA-B4-5832-01A-11R-1672-07	stage ii	t2	male		
TCGA-EU-5905-01A-11R-1672-07	stage i	t1b	male		
TCGA-BP-4326-01A-01R-1289-07	stage iii	t3a	male		
TCGA-CZ-4858-01A-01R-1305-07	stage iii	t3b	female		
TCGA-B0-5706-01A-11R-1541-07	stage i	t1a	female		
TCGA-CZ-5456-01A-01R-1503-07	stage iii	t3a	female		
TCGA-BP-4169-01A-02R-1289-07	stage ii	t2	female		
TCGA-CJ-5671-01A-11R-1541-07	stage i	t1b	female	SLC2A14	>75 Percentile

TCGA-B8-4620-01A-02R-1325-07	stage ii	t2	male		
TCGA-CJ-4901-01A-01R-1426-07	stage iii	t3a	female		
TCGA-B0-4710-01A-01R-1503-07	stage i	t1b	male		
TCGA-B0-4698-01A-01R-1503-07	stage ii	t2	female		
TCGA-EU-5904-01A-11R-1672-07	stage i	t1b	male		
TCGA-BP-5200-01A-01R-1426-07	stage i	t1	male		
TCGA-B0-5097-01A-01R-1420-07	stage ii	t2	male		
TCGA-GK-A6C7-01A-11R-A33J-07	stage iii	t3a	male		
TCGA-CJ-4882-01A-02R-1426-07	stage i	t1a	male		
TCGA-A3-3335-01A-01R-0864-07	stage i	t1a	male		
TCGA-BP-4781-01A-01R-1305-07	stage iii	t3c	male		
TCGA-B0-5098-01A-01R-1420-07	stage ii	t2	male		
TCGA-BP-4960-01A-01R-1334-07	stage iv	t3b	male		
TCGA-CZ-4861-01A-01R-1305-07	stage iii	t3a	male		
TCGA-BP-4340-01A-01R-1289-07	stage i	t1b	female		
TCGA-AK-3436-01A-02R-1325-07	stage i	t1b	male		
TCGA-CJ-4868-01A-01R-1305-07	stage iv	t2	female		
TCGA-B0-5116-01A-02R-1420-07	stage i	t1	female		
TCGA-B0-4821-01A-01R-1503-07	stage iii	t3a	male		
TCGA-BP-4799-01A-01R-1305-07	stage ii	t2a	female		
TCGA-BP-4974-01A-01R-1334-07	stage iii	t3a	male		
TCGA-BP-4770-01A-01R-1503-07	stage iii	t3a	male		
TCGA-B8-A8YJ-01A-13R-A39I-07	stage i	t1a	female		
TCGA-EU-5907-01A-11R-1672-07	stage i	t1a	male		
TCGA-BP-4355-01A-01R-1289-07	stage iv	t4	male		
TCGA-BP-5178-01A-01R-1426-07	stage i	t1a	female		
TCGA-BP-5008-01A-01R-1334-07	stage iii	t3a	male		
TCGA-CZ-4857-01A-01R-1305-07	stage iv	t3a	female		
TCGA-B0-4690-01A-01R-1277-07	stage iii	t3a	male		

TCGA-CZ-5459-01A-01R-1503-07	stage i	t1a	male		
TCGA-DV-5569-01A-01R-1541-07	stage iii	t3c	female		
TCGA-BP-4335-01A-01R-1289-07	stage i	t1b	male		
TCGA-B0-4718-01A-01R-1277-07	stage iii	t3a	male		
TCGA-B0-5698-01A-11R-1672-07	stage i	t1a	female		
TCGA-B4-5378-01A-01R-1503-07	stage ii	t2	male		
TCGA-CZ-5461-01A-01R-1503-07	stage i	t1a	female		
TCGA-BP-4353-01A-02R-1289-07	stage i	t1b	female		
TCGA-DV-5575-01A-01R-1541-07	stage i	t1b	male		
TCGA-CZ-5988-01A-11R-1672-07	stage iii	t3b	male		
TCGA-EU-5906-01A-11R-1672-07	stage ii	t2	male		
TCGA-BP-4345-01A-01R-1289-07	stage iv	t4	male		
TCGA-B0-4822-01A-01R-1277-07	stage ii	t2b	male		
TCGA-BP-5187-01A-01R-1426-07	stage i	t1b	male		
TCGA-B0-5108-01A-01R-1420-07	stage iii	t3a	male		
TCGA-CW-6090-01A-11R-1672-07	stage iii	t3a	male		
TCGA-BP-4170-01A-02R-1289-07	stage ii	t2	female		
TCGA-B0-4852-01A-01R-1503-07	stage ii	t2	female		
TCGA-BP-4338-01A-01R-1289-07	stage i	t1a	male		
TCGA-CJ-4885-01A-01R-1305-07	stage iv	t3	female		
TCGA-B0-5077-01A-01R-1334-07	stage i	t1b	female		
TCGA-B0-5099-01A-01R-1420-07	stage i	t1a	female		
TCGA-B0-4814-01A-01R-1277-07	stage ii	t2	male		
TCGA-CJ-4920-01A-01R-1426-07	stage i	t1a	male		
TCGA-BP-4998-01A-01R-1334-07	stage iii	t3a	male		
TCGA-BP-4343-01A-02R-1289-07	stage i	t1a	male		
TCGA-CZ-4860-01A-01R-1305-07	stage i	t1b	female		
TCGA-B8-5549-01A-01R-1541-07	stage i	t1a	male		
TCGA-B8-5545-01A-01R-1672-07	stage i	t1b	female		

TCGA-BP-4988-01A-01R-1334-07	stage ii	t2	female		
TCGA-BP-4973-01A-01R-1334-07	stage iii	t3b	male		
TCGA-B8-4143-01A-01R-1188-07	stage i	t1	female		
TCGA-BP-4983-01A-01R-1334-07	stage iv	t3a	male		
TCGA-EU-5905-01A-11R-1672-07	stage i	t1b	male		
TCGA-B0-5094-01A-01R-1420-07	stage ii	t2b	female		
TCGA-CJ-4878-01A-01R-1305-07	stage i	t1a	male		
TCGA-B0-5703-01A-11R-1541-07	stage i	t1a	male		
TCGA-BP-4768-01A-01R-1289-07	stage iv	t3b	male		
TCGA-B0-4819-01A-01R-1277-07	stage iii	t3a	male		
TCGA-B0-5695-01A-11R-1541-07	stage ii	t2	male		
TCGA-CZ-5986-01A-11R-1672-07	stage i	t1a	male		
TCGA-A3-3382-01A-02R-1325-07	stage i	t1	male		
TCGA-AK-3454-01A-02R-1277-07	stage iii	t3a	male		
TCGA-B0-5700-01A-11R-1541-07	stage i	t1a	male		
TCGA-A3-3378-01A-02R-1325-07	stage i	t1b	female		
TCGA-BP-4176-01A-02R-1289-07	stage i	t1a	male		
TCGA-CJ-4918-01A-01R-1426-07	stage iii	t3a	male		
TCGA-B8-A7U6-01A-12R-A37O-07	stage i	t1a	female		
TCGA-B0-4815-01A-01R-1503-07	stage iii	t3b	male		
TCGA-BP-4352-01A-01R-1289-07	stage iii	t3a	male		
TCGA-B0-4837-01A-01R-1305-07	stage iii	t3	male		
TCGA-B8-A54F-01A-11R-A266-07	stage i	t1a	male		
TCGA-CJ-5689-01A-11R-1541-07	stage i	t1a	male		
TCGA-BP-4787-01A-01R-1305-07	stage iv	t3b	female		
TCGA-CZ-5460-01A-01R-1503-07	stage iii	t3a	male		
TCGA-BP-4993-01A-02R-1420-07	stage i	t1a	male		
TCGA-A3-3346-01A-01R-1766-07	stage i	t1a	male		
TCGA-DV-A4W0-01A-11R-A266-07	stage i	t1a	male		

TCGA-CZ-5984-01A-11R-1672-07	stage i	t1a	female		
TCGA-CZ-4854-01A-01R-1305-07	stage iv	t3a	male		
TCGA-BP-4803-01A-01R-1305-07	stage iv	t2	male		
TCGA-BP-5199-01A-01R-1426-07	stage i	t1a	male		
TCGA-DV-5576-01A-01R-1541-07	stage i	t1a	male		
TCGA-CJ-6028-01A-11R-1672-07	stage i	t1b	female		
TCGA-B0-4700-01A-02R-1541-07	stage i	t1b	female		
TCGA-B0-5080-01A-01R-1503-07	stage i	t1b	male		
TCGA-A3-3347-01A-02R-1325-07	stage i	t1a	female		
TCGA-BP-4342-01A-01R-1289-07	stage i	t1b	female		
TCGA-B0-4712-01A-01R-1503-07	stage iv	t1b	female		
TCGA-CJ-4904-01A-02R-1426-07	stage i	t1b	male		
TCGA-BP-4971-01A-01R-1334-07	stage iv	t3a	male		
TCGA-B0-4823-01A-02R-1420-07	stage ii	t2	female		
TCGA-A3-A8OU-01A-11R-A37O-07	stage i	t1a	male		
TCGA-CZ-5464-01A-01R-1503-07	stage i	t1a	female		
TCGA-A3-3372-01A-02R-1325-07	stage iii	t1b	male		
TCGA-B8-5158-01A-01R-1420-07	stage ii	t2	male		
TCGA-BP-4977-01A-01R-1334-07	stage i	t1a	female		
TCGA-B8-5163-01A-01R-1420-07	stage i	t1b	male		
TCGA-CJ-6032-01A-11R-1672-07	stage i	t1b	male		
TCGA-CJ-6030-01A-11R-1672-07	stage i	t1b	male		
TCGA-B0-5696-01A-11R-1541-07	stage iii	t3a	male		
TCGA-BP-4329-01A-02R-1289-07	stage ii	t2	male		
TCGA-AS-3778-01A-01R-A32Z-07	stage i	t1a	male		
TCGA-BP-4972-01A-01R-1334-07	stage iv	t3a	male		
TCGA-B0-5694-01A-11R-1541-07	stage i	t1	female		
TCGA-CJ-4902-01A-01R-1426-07	stage iii	t3a	male		
TCGA-B8-5551-01A-01R-1541-07	stage i	t1	male		

TCGA-BP-4174-01A-02R-1289-07	stage iv	t2	male		
TCGA-BP-4763-01A-01R-1289-07	stage iii	t3a	male		
TCGA-BP-4986-01A-01R-1334-07	stage iii	t3a	female		
TCGA-BP-4351-01A-01R-1289-07	stage iii	t3b	male		
TCGA-B0-4693-01A-01R-1277-07	stage i	t1a	male		
TCGA-AK-3425-01A-02R-1277-07	stage i	t1b	male		
TCGA-CJ-4890-01A-01R-1305-07	stage i	t1b	female		
TCGA-B0-4701-01A-01R-1277-07	stage i	t1b	female		
TCGA-DV-5567-01A-01R-1541-07	stage iii	t3a	male		
TCGA-BP-4807-01A-01R-1305-07	stage i	t1a	male		
TCGA-B2-5639-01A-01R-1541-07	stage i	t1b	male		
TCGA-BP-4346-01A-01R-1289-07	stage iv	t4	male		
TCGA-BP-5202-01A-02R-1426-07	stage i	t1a	male		
TCGA-G6-A8L8-01A-21R-A37O-07	stage i	t1a	female		
TCGA-CZ-4863-01A-01R-1503-07	stage iii	t3a	male		
TCGA-CJ-4641-01A-02R-1325-07	stage iii	t3a	female		
TCGA-B0-4707-01A-01R-1277-07	stage ii	t2	male		
TCGA-B0-4818-01A-01R-1503-07	stage iii	t3	male		
TCGA-B0-5084-01A-01R-1334-07	stage ii	t2	male		
TCGA-BP-5184-01A-01R-1426-07	stage i	t1b	female		
TCGA-BP-4349-01A-01R-1289-07	stage iii	t3a	female		
TCGA-B8-4154-01A-01R-1188-07	stage ii	t2a	male		
TCGA-B0-5711-01A-11R-1672-07	stage ii	t2	female		
TCGA-B0-5697-01A-11R-1541-07	stage i	t1b	female		
TCGA-B2-4101-01A-02R-1277-07	stage ii	t2a	male		
TCGA-MM-A84U-01A-11R-A37O-07	stage i	t1b	male		
TCGA-A3-3367-01A-02R-1420-07	stage i	t1a	male		
TCGA-BP-4771-01A-01R-1289-07	stage iii	t3a	male		
TCGA-B2-4102-01A-02R-1325-07	stage i	t1b	female		<25 Percentile

TCGA-CJ-4873-01A-01R-1305-07	stage i	t1a	male			
TCGA-BP-4331-01A-01R-1289-07	stage i	t1a	female			
TCGA-BP-5183-01A-01R-1426-07	stage i	t1b	male			
TCGA-B0-5119-01A-02R-1420-07	stage i	t1	female			
TCGA-B4-5377-01A-01R-1503-07	stage i	t1b	male			
TCGA-B0-5692-01A-11R-1541-07	stage ii	t2b	female			
TCGA-BP-4989-01A-01R-1334-07	stage iv	t3a	male			
TCGA-CZ-4865-01A-02R-1503-07	stage iii	t3a	male			
TCGA-CJ-4894-01A-01R-1305-07	stage i	t1a	male			
TCGA-CZ-4853-01A-01R-1426-07	stage iii	t3a	male			
TCGA-G6-A8L6-01A-11R-A37O-07	stage i	t1b	male			
TCGA-CJ-5677-01A-11R-1541-07	stage i	t1b	female			
TCGA-AK-3440-01A-02R-1277-07	stage i	t1a	male			
TCGA-B0-4706-01A-01R-1503-07	stage ii	t2	female			
TCGA-B0-4714-01A-01R-1277-07	stage iii	t3a	female			
TCGA-B8-5550-01A-01R-1541-07	stage i	t1	male			
TCGA-B2-4099-01A-02R-1188-07	stage i	t1a	male			
TCGA-DV-5573-01A-01R-1541-07	stage iii	t3a	male			
TCGA-A3-3317-01A-02R-1325-07	stage i	t1a	female			
TCGA-BP-4992-01A-01R-1334-07	stage iv	t1a	female			
TCGA-DV-A4VX-01A-11R-A266-07	stage i	t1a	male			
TCGA-A3-3357-01A-02R-1420-07	stage i	t1a	male			
TCGA-CJ-4875-01A-01R-1305-07	stage i	t1b	male			
TCGA-B0-5399-01A-01R-1503-07	stage ii	t2a	female			
TCGA-CW-5584-01A-01R-1541-07	stage iii	t3a	male			
TCGA-B0-4697-01A-01R-1277-07	stage ii	t2	female			
TCGA-B0-5699-01A-11R-1541-07	stage i	t1b	female			
TCGA-A3-3383-01A-02R-1325-07	stage iii	t3a	male			
TCGA-BP-4159-01A-02R-1289-07	stage i	t1a	male			

TCGA-B0-4841-01A-01R-1277-07	stage i	t1b	male		
TCGA-B0-4713-01A-01R-1277-07	stage ii	t2	male		
TCGA-CJ-5672-01A-11R-1541-07	stage i	t1b	male		
TCGA-B8-5159-01A-01R-1420-07	stage i	t1a	male		
TCGA-B0-4838-01A-01R-1305-07	stage i	t1b	male		
TCGA-BP-4163-01A-02R-1325-07	stage i	t1a	male		
TCGA-BP-4969-01A-01R-1334-07	stage iv	t3a	male		
TCGA-B8-4148-01A-02R-1325-07	stage i	t1b	male		
TCGA-BP-5001-01A-01R-1334-07	stage iii	t3b	female		
TCGA-MM-A564-01A-11R-A266-07	stage i	t1a	female		
TCGA-A3-3326-01A-01R-0864-07	stage i	t1b	female		
TCGA-B8-4621-01A-01R-1503-07	stage i	t1	male		
TCGA-CJ-5675-01A-11R-1541-07	stage iii	t3a	male		
TCGA-A3-3385-01A-02R-1420-07	stage iii	t3b	male		
TCGA-CJ-4887-01A-01R-1305-07	stage iii	t3b	male		
TCGA-BP-4962-01A-01R-1334-07	stage i	t1b	female		
TCGA-6D-AA2E-01A-11R-A37O-07	stage i	t1b	male		
TCGA-BP-5191-01A-01R-1426-07	stage iii	t3b	male		
TCGA-A3-A8OW-01A-11R-A37O-07	stage iii	t3	male		
TCGA-CJ-4884-01A-01R-1305-07	stage ii	t2a	male		
TCGA-CZ-5470-01A-01R-1503-07	stage i	t1a	male		
TCGA-BP-4167-01A-02R-1325-07	stage iii	t3b	male		
TCGA-CZ-5455-01A-01R-1503-07	stage iv	t4	male		
TCGA-B0-4847-01A-01R-1277-07	stage i	t1a	female		
TCGA-CJ-4637-01A-02R-1325-07	stage i	t1b	male		
TCGA-B0-5092-01A-01R-1420-07	stage ii	t2	female		
TCGA-B8-4619-01A-02R-1325-07	stage iii	t3a	male		
TCGA-B8-4151-01A-01R-1188-07	stage iii	t1b	female		
TCGA-B0-5705-01A-11R-1541-07	stage i	t1b	male		

TCGA-CJ-4638-01A-02R-1325-07	stage i	t1	female		
TCGA-B0-4696-01A-01R-1277-07	stage iii	t3b	female		
TCGA-A3-3374-01A-02R-1325-07	stage iii	t3a	female		
TCGA-B0-5707-01A-11R-1541-07	stage i	t1b	male		
TCGA-CZ-4866-01A-01R-1503-07	stage iii	t3b	male		
TCGA-BP-4325-01A-02R-1289-07	stage i	t1b	female		
TCGA-BP-4959-01A-01R-1334-07	stage i	t1a	male		
TCGA-B0-4824-01A-01R-1277-07	stage iii	t3a	male		
TCGA-CZ-5458-01A-01R-1503-07	stage i	t1a	male		
TCGA-AK-3455-01A-01R-0864-07	stage i	t1b	female		
TCGA-BP-5185-01A-01R-1426-07	stage i	t1a	female		
TCGA-AK-3426-01A-02R-1325-07	stage i	t1b	female		
TCGA-AK-3458-01A-01R-1503-07	stage i	t1a	female		
TCGA-BP-5174-01A-01R-1426-07	stage iii	t3a	male		
TCGA-B0-5100-01A-01R-1420-07	stage i	t1a	male		
TCGA-AK-3456-01A-02R-1325-07	stage i	t1a	male		
TCGA-G6-A8L7-01A-11R-A37O-07	stage i	t1a	male		
TCGA-B0-5712-01A-11R-1672-07	stage i	t1a	male		
TCGA-CZ-5452-01A-01R-1503-07	stage i	t1a	female		
TCGA-BP-5190-01A-01R-1426-07	stage iii	t3b	female		
TCGA-CJ-5680-01A-11R-1541-07	stage i	t1b	female		
TCGA-A3-3370-01A-02R-1420-07	stage i	t1a	female		
TCGA-B0-4834-01A-01R-1305-07	stage i	t1a	male		
TCGA-CJ-5681-01A-11R-1541-07	stage i	t1a	female		
TCGA-A3-3313-01A-02R-1325-07	stage i	t1a	female		
TCGA-B4-5832-01A-11R-1672-07	stage ii	t2	male		
TCGA-B2-3923-01A-02R-1325-07	stage i	t1a	female		
TCGA-B8-A54G-01A-11R-A266-07	stage i	t1a	female		
TCGA-BP-4756-01A-01R-1289-07	stage iv	t4	male		

TCGA-B0-5107-01A-01R-1420-07	stage ii	t2	male		
TCGA-AK-3433-01A-02R-1277-07	stage i	t1b	female		
TCGA-B4-5835-01A-11R-1672-07	stage i	t1b	female		
TCGA-A3-3328-01A-01R-0864-07	stage i	t1b	male		
TCGA-B0-5117-01A-01R-1420-07	stage iii	t3a	male		
TCGA-B8-5546-01A-01R-1541-07	stage i	t1b	female		
TCGA-T7-A92I-01A-11R-A37O-07	stage iii	t3a	male		
TCGA-A3-3363-01A-01R-0864-07	NA	t1a	male		
TCGA-B8-A54E-01A-11R-A266-07	stage i	t1a	male		
TCGA-AS-3777-01A-01R-0864-07	stage i	t1b	male		
TCGA-AK-3447-01A-01R-1766-07	stage i	t1	male		
TCGA-CW-5591-01A-01R-1541-07	stage i	t1a	male		
TCGA-B0-4842-01A-02R-1420-07	stage i	t1a	female		
TCGA-B8-A54D-01A-21R-A266-07	stage i	t1a	female		
TCGA-AK-3427-01A-01R-0864-07	stage i	t1a	male		
TCGA-AK-3465-01A-02R-1325-07	stage i	t1b	male		
TCGA-BP-4761-01A-01R-1289-07	stage iv	t3a	male		
TCGA-B0-5083-01A-02R-1420-07	stage iv	t4	male		
TCGA-AK-3451-01A-02R-1188-07	stage i	t1a	male		
TCGA-AK-3453-01A-02R-1277-07	stage ii	t2b	female		
TCGA-AK-3443-01A-02R-1325-07	stage i	t1a	male		
TCGA-BP-4334-01A-01R-1289-07	stage ii	t2	male		
TCGA-G6-A5PC-01A-11R-A33J-07	stage ii	t2	male		
TCGA-B0-5702-01A-11R-1541-07	stage i	t1a	male		
TCGA-BP-4326-01A-01R-1289-07	stage iii	t3a	male		
TCGA-B0-5706-01A-11R-1541-07	stage i	t1a	female		
TCGA-CZ-4858-01A-01R-1305-07	stage iii	t3b	female		
TCGA-B8-4620-01A-02R-1325-07	stage ii	t2	male		
TCGA-BP-4770-01A-01R-1503-07	stage iii	t3a	male	SLC2A3	>75 Percentile

TCGA-BP-4169-01A-02R-1289-07	stage ii	t2	female			
TCGA-CZ-5456-01A-01R-1503-07	stage iii	t3a	female			
TCGA-B0-5098-01A-01R-1420-07	stage ii	t2	male			
TCGA-CJ-4901-01A-01R-1426-07	stage iii	t3a	female			
TCGA-EU-5904-01A-11R-1672-07	stage i	t1b	male			
TCGA-B0-5097-01A-01R-1420-07	stage ii	t2	male			
TCGA-CJ-4882-01A-02R-1426-07	stage i	t1a	male			
TCGA-B0-4698-01A-01R-1503-07	stage ii	t2	female			
TCGA-B0-4718-01A-01R-1277-07	stage iii	t3a	male			
TCGA-BP-4960-01A-01R-1334-07	stage iv	t3b	male			
TCGA-BP-4799-01A-01R-1305-07	stage ii	t2a	female			
TCGA-B0-4710-01A-01R-1503-07	stage i	t1b	male			
TCGA-CJ-5671-01A-11R-1541-07	stage i	t1b	female			
TCGA-BP-5200-01A-01R-1426-07	stage i	t1	male			
TCGA-CZ-4861-01A-01R-1305-07	stage iii	t3a	male			
TCGA-CZ-5988-01A-11R-1672-07	stage iii	t3b	male			
TCGA-BP-4340-01A-01R-1289-07	stage i	t1b	female			
TCGA-B0-4821-01A-01R-1503-07	stage iii	t3a	male			
TCGA-B0-5116-01A-02R-1420-07	stage i	t1	female			
TCGA-BP-4974-01A-01R-1334-07	stage iii	t3a	male			
TCGA-GK-A6C7-01A-11R-A33J-07	stage iii	t3a	male			
TCGA-CJ-4868-01A-01R-1305-07	stage iv	t2	female			
TCGA-BP-5008-01A-01R-1334-07	stage iii	t3a	male			
TCGA-BP-4355-01A-01R-1289-07	stage iv	t4	male			
TCGA-BP-4335-01A-01R-1289-07	stage i	t1b	male			
TCGA-DV-5575-01A-01R-1541-07	stage i	t1b	male			
TCGA-BP-4345-01A-01R-1289-07	stage iv	t4	male			
TCGA-CZ-5459-01A-01R-1503-07	stage i	t1a	male			
TCGA-A3-3335-01A-01R-0864-07	stage i	t1a	male			

TCGA-AK-3436-01A-02R-1325-07	stage i	t1b	male			
TCGA-B0-4690-01A-01R-1277-07	stage iii	t3a	male			
TCGA-CW-6090-01A-11R-1672-07	stage iii	t3a	male			
TCGA-BP-4353-01A-02R-1289-07	stage i	t1b	female			
TCGA-B0-4852-01A-01R-1503-07	stage ii	t2	female			
TCGA-CZ-4860-01A-01R-1305-07	stage i	t1b	female			
TCGA-B0-5108-01A-01R-1420-07	stage iii	t3a	male			
TCGA-CZ-5461-01A-01R-1503-07	stage i	t1a	female			
TCGA-B4-5378-01A-01R-1503-07	stage ii	t2	male			
TCGA-CJ-4878-01A-01R-1305-07	stage i	t1a	male			
TCGA-B0-4814-01A-01R-1277-07	stage ii	t2	male			
TCGA-CJ-4885-01A-01R-1305-07	stage iv	t3	female			
TCGA-DV-5569-01A-01R-1541-07	stage iii	t3c	female			
TCGA-BP-4170-01A-02R-1289-07	stage ii	t2	female			
TCGA-BP-4781-01A-01R-1305-07	stage iii	t3c	male			
TCGA-BP-4352-01A-01R-1289-07	stage iii	t3a	male			
TCGA-DV-A4W0-01A-11R-A266-07	stage i	t1a	male			
TCGA-BP-4176-01A-02R-1289-07	stage i	t1a	male			
TCGA-B0-4822-01A-01R-1277-07	stage ii	t2b	male			
TCGA-AK-3454-01A-02R-1277-07	stage iii	t3a	male			
TCGA-BP-4769-01A-01R-1289-07	stage iii	t3a	male			
TCGA-BP-4988-01A-01R-1334-07	stage ii	t2	female			
TCGA-B0-5077-01A-01R-1334-07	stage i	t1b	female			
TCGA-BP-5199-01A-01R-1426-07	stage i	t1a	male			
TCGA-B0-5099-01A-01R-1420-07	stage i	t1a	female			
TCGA-B0-4819-01A-01R-1277-07	stage iii	t3a	male			
TCGA-CZ-5986-01A-11R-1672-07	stage i	t1a	male			
TCGA-B8-4143-01A-01R-1188-07	stage i	t1	female			
TCGA-B8-A54F-01A-11R-A266-07	stage i	t1a	male			

TCGA-B0-5700-01A-11R-1541-07	stage i	t1a	male		
TCGA-B0-4712-01A-01R-1503-07	stage iv	t1b	female		
TCGA-B0-4823-01A-02R-1420-07	stage ii	t2	female		
TCGA-A3-3372-01A-02R-1325-07	stage iii	t1b	male		
TCGA-DV-5576-01A-01R-1541-07	stage i	t1a	male		
TCGA-B0-4837-01A-01R-1305-07	stage iii	t3	male		
TCGA-CZ-5460-01A-01R-1503-07	stage iii	t3a	male		
TCGA-B0-4811-01A-01R-1503-07	stage ii	t2	male		
TCGA-BP-5170-01A-01R-1426-07	stage i	t1a	male		
TCGA-CJ-4876-01A-01R-1305-07	stage i	t1b	male		
TCGA-BP-4776-01A-01R-1289-07	stage iv	t4	male		
TCGA-B8-A8YJ-01A-13R-A39I-07	stage i	t1a	female		
TCGA-BP-4971-01A-01R-1334-07	stage iv	t3a	male		
TCGA-EU-5907-01A-11R-1672-07	stage i	t1a	male		
TCGA-CJ-6030-01A-11R-1672-07	stage i	t1b	male		
TCGA-CZ-4857-01A-01R-1305-07	stage iv	t3a	female		
TCGA-B8-5545-01A-01R-1672-07	stage i	t1b	female		
TCGA-AK-3428-01A-02R-1277-07	stage i	t1	male		
TCGA-B0-5695-01A-11R-1541-07	stage ii	t2	male		
TCGA-BP-5168-01A-01R-1420-07	stage i	t1a	male		
TCGA-BP-4162-01A-02R-1325-07	stage iii	t3a	male		
TCGA-CJ-4920-01A-01R-1426-07	stage i	t1a	male		
TCGA-BP-4993-01A-02R-1420-07	stage i	t1a	male		
TCGA-CJ-6028-01A-11R-1672-07	stage i	t1b	female		
TCGA-B0-5698-01A-11R-1672-07	stage i	t1a	female		
TCGA-CZ-5467-01A-01R-1503-07	stage iv	t3a	male		
TCGA-CJ-4918-01A-01R-1426-07	stage iii	t3a	male		
TCGA-B8-5163-01A-01R-1420-07	stage i	t1b	male		
TCGA-BP-5187-01A-01R-1426-07	stage i	t1b	male		

TCGA-B0-5080-01A-01R-1503-07	stage i	t1b	male			
TCGA-B0-4693-01A-01R-1277-07	stage i	t1a	male			
TCGA-CJ-4900-01A-01R-1334-07	stage i	t1a	female			
TCGA-BP-4998-01A-01R-1334-07	stage iii	t3a	male			
TCGA-BP-4760-01A-02R-1420-07	stage i	t1b	male			
TCGA-CJ-6032-01A-11R-1672-07	stage i	t1b	male			
TCGA-B0-5115-01A-01R-1420-07	stage ii	t2	female			
TCGA-CZ-5453-01A-01R-1503-07	stage iii	t3a	female			
TCGA-CZ-4854-01A-01R-1305-07	stage iv	t3a	male			
TCGA-B0-5096-01A-01R-1420-07	stage ii	t2	female			
TCGA-BP-4329-01A-02R-1289-07	stage ii	t2	male			
TCGA-B0-4700-01A-02R-1541-07	stage i	t1b	female			
TCGA-BP-4985-01A-01R-1334-07	stage iii	t3b	female			
TCGA-B8-A7U6-01A-12R-A37O-07	stage i	t1a	female			
TCGA-CZ-5465-01A-01R-1503-07	stage i	t1a	male			
TCGA-CJ-5689-01A-11R-1541-07	stage i	t1a	male			
TCGA-CW-6093-01A-11R-1672-07	stage i	t1a	male			
TCGA-B8-5549-01A-01R-1541-07	stage i	t1a	male			
TCGA-BP-4973-01A-01R-1334-07	stage iii	t3b	male			
TCGA-BP-4787-01A-01R-1305-07	stage iv	t3b	female			
TCGA-B0-5094-01A-01R-1420-07	stage ii	t2b	female			
TCGA-A3-A8OU-01A-11R-A37O-07	stage i	t1a	male			
TCGA-CJ-4904-01A-02R-1426-07	stage i	t1b	male			
TCGA-B0-4815-01A-01R-1503-07	stage iii	t3b	male			
TCGA-A3-3316-01A-01R-0864-07	stage i	t1a	male			
TCGA-CZ-5987-01A-11R-1672-07	stage i	t1a	male			
TCGA-BP-4807-01A-01R-1305-07	stage i	t1a	male			
TCGA-BP-4983-01A-01R-1334-07	stage iv	t3a	male			
TCGA-EU-5906-01A-11R-1672-07	stage ii	t2	male			

TCGA-BP-4344-01A-01R-1289-07	stage iii	t3a	male		
TCGA-CJ-4902-01A-01R-1426-07	stage iii	t3a	male		
TCGA-BP-4351-01A-01R-1289-07	stage iii	t3b	male		
TCGA-CJ-6033-01A-11R-1672-07	stage i	t1b	female		
TCGA-B0-4813-01A-01R-1277-07	stage iii	t3a	male		
TCGA-DV-5565-01A-01R-1541-07	stage ii	t2	male		
TCGA-B0-4699-01A-01R-1277-07	stage iii	t3a	male		
TCGA-AK-3429-01A-02R-1325-07	stage i	t1a	male		
TCGA-CJ-4890-01A-01R-1305-07	stage i	t1b	female		
TCGA-CJ-6027-01A-11R-1672-07	stage i	t1a	male		
TCGA-CJ-4892-01A-01R-1305-07	stage iv	t3	male		
TCGA-B8-5553-01A-01R-1541-07	stage i	t1	male		
TCGA-BP-4995-01A-01R-1334-07	stage ii	t2	male		
TCGA-BP-4976-01A-01R-1334-07	stage iii	t3a	male		
TCGA-B0-5119-01A-02R-1420-07	stage i	t1	female		
TCGA-A3-3343-01A-01R-0864-07	stage i	t1a	female		
TCGA-A3-A6NJ-01A-12R-A33J-07	stage i	t1a	female		
TCGA-A3-3367-01A-02R-1420-07	stage i	t1a	male		
TCGA-BP-4989-01A-01R-1334-07	stage iv	t3a	male		
TCGA-G6-A8L7-01A-11R-A37O-07	stage i	t1a	male		
TCGA-CZ-5985-01A-11R-1672-07	stage iv	t3a	female		
TCGA-CJ-4893-01A-01R-1305-07	stage i	t1	male		
TCGA-B2-3924-01A-02R-1325-07	stage iii	t3a	male		
TCGA-B0-4706-01A-01R-1503-07	stage ii	t2	female		
TCGA-B4-5838-01A-11R-1672-07	stage i	t1b	male		
TCGA-B0-4707-01A-01R-1277-07	stage ii	t2	male		
TCGA-CJ-5678-01A-11R-1541-07	stage i	t1b	female		
TCGA-CJ-4873-01A-01R-1305-07	stage i	t1a	male		
TCGA-BP-4759-01A-01R-1289-07	stage iv	t3a	female		<25 Percentile

TCGA-DV-5573-01A-01R-1541-07	stage iii	t3a	male		
TCGA-B2-4099-01A-02R-1188-07	stage i	t1a	male		
TCGA-BP-4782-01A-02R-1420-07	stage i	t1b	male		
TCGA-A3-3306-01A-01R-0864-07	stage i	t1a	female		
TCGA-B0-5711-01A-11R-1672-07	stage ii	t2	female		
TCGA-CJ-4887-01A-01R-1305-07	stage iii	t3b	male		
TCGA-CW-5587-01A-01R-1541-07	stage i	t1b	female		
TCGA-BP-4349-01A-01R-1289-07	stage iii	t3a	female		
TCGA-BP-4771-01A-01R-1289-07	stage iii	t3a	male		
TCGA-A3-3383-01A-02R-1325-07	stage iii	t3a	male		
TCGA-BP-5182-01A-01R-1426-07	stage iii	t3b	male		
TCGA-CW-5585-01A-01R-1541-07	stage ii	t2	female		
TCGA-BP-4167-01A-02R-1325-07	stage iii	t3b	male		
TCGA-AK-3458-01A-01R-1503-07	stage i	t1a	female		
TCGA-B0-5697-01A-11R-1541-07	stage i	t1b	female		
TCGA-B2-5641-01A-01R-1541-07	stage iii	t3b	female		
TCGA-CZ-5455-01A-01R-1503-07	stage iv	t4	male		
TCGA-A3-3374-01A-02R-1325-07	stage iii	t3a	female		
TCGA-BP-4994-01A-01R-1334-07	stage iv	t3b	male		
TCGA-B0-5702-01A-11R-1541-07	stage i	t1a	male		
TCGA-B0-5692-01A-11R-1541-07	stage ii	t2b	female		
TCGA-A3-3387-01A-01R-1541-07	stage i	t1a	male		
TCGA-B0-4713-01A-01R-1277-07	stage ii	t2	male		
TCGA-CW-5581-01A-02R-1541-07	stage i	t1b	female		
TCGA-CZ-4853-01A-01R-1426-07	stage iii	t3a	male		
TCGA-B2-5635-01A-01R-1541-07	stage i	t1a	female		
TCGA-CJ-5677-01A-11R-1541-07	stage i	t1b	female		
TCGA-A3-3357-01A-02R-1420-07	stage i	t1a	male		
TCGA-CJ-4637-01A-02R-1325-07	stage i	t1b	male		

TCGA-B8-4154-01A-01R-1188-07	stage ii	t2a	male		
TCGA-B0-4824-01A-01R-1277-07	stage iii	t3a	male		
TCGA-CZ-4863-01A-01R-1503-07	stage iii	t3a	male		
TCGA-B0-5092-01A-01R-1420-07	stage ii	t2	female		
TCGA-B2-4101-01A-02R-1277-07	stage ii	t2a	male		
TCGA-B0-5701-01A-11R-1541-07	stage i	t1a	female		
TCGA-BP-4346-01A-01R-1289-07	stage iv	t4	male		
TCGA-B4-5377-01A-01R-1503-07	stage i	t1b	male		
TCGA-BP-4331-01A-01R-1289-07	stage i	t1a	female		
TCGA-B2-4102-01A-02R-1325-07	stage i	t1b	female		
TCGA-AK-3426-01A-02R-1325-07	stage i	t1b	female		
TCGA-CJ-4641-01A-02R-1325-07	stage iii	t3a	female		
TCGA-A3-3317-01A-02R-1325-07	stage i	t1a	female		
TCGA-BP-5202-01A-02R-1426-07	stage i	t1a	male		
TCGA-B0-4847-01A-01R-1277-07	stage i	t1a	female		
TCGA-BP-4969-01A-01R-1334-07	stage iv	t3a	male		
TCGA-B8-5159-01A-01R-1420-07	stage i	t1a	male		
TCGA-BP-5183-01A-01R-1426-07	stage i	t1b	male		
TCGA-AK-3455-01A-01R-0864-07	stage i	t1b	female		
TCGA-AK-3440-01A-02R-1277-07	stage i	t1a	male		
TCGA-BP-4962-01A-01R-1334-07	stage i	t1b	female		
TCGA-B0-4838-01A-01R-1305-07	stage i	t1b	male		
TCGA-B2-5639-01A-01R-1541-07	stage i	t1b	male		
TCGA-B0-5699-01A-11R-1541-07	stage i	t1b	female		
TCGA-6D-AA2E-01A-11R-A37O-07	stage i	t1b	male		
TCGA-CW-5584-01A-01R-1541-07	stage iii	t3a	male		
TCGA-B8-4151-01A-01R-1188-07	stage iii	t1b	female		
TCGA-B0-4696-01A-01R-1277-07	stage iii	t3b	female		
TCGA-CZ-5470-01A-01R-1503-07	stage i	t1a	male		

TCGA-B0-4842-01A-02R-1420-07	stage i	t1a	female		
TCGA-BP-4325-01A-02R-1289-07	stage i	t1b	female		
TCGA-B8-5550-01A-01R-1541-07	stage i	t1	male		
TCGA-BP-4163-01A-02R-1325-07	stage i	t1a	male		
TCGA-CJ-4884-01A-01R-1305-07	stage ii	t2a	male		
TCGA-CJ-5675-01A-11R-1541-07	stage iii	t3a	male		
TCGA-CJ-5672-01A-11R-1541-07	stage i	t1b	male		
TCGA-B0-5712-01A-11R-1672-07	stage i	t1a	male		
TCGA-A3-3326-01A-01R-0864-07	stage i	t1b	female		
TCGA-B8-4619-01A-02R-1325-07	stage iii	t3a	male		
TCGA-CZ-5458-01A-01R-1503-07	stage i	t1a	male		
TCGA-B0-5705-01A-11R-1541-07	stage i	t1b	male		
TCGA-B8-4148-01A-02R-1325-07	stage i	t1b	male		
TCGA-CZ-5452-01A-01R-1503-07	stage i	t1a	female		
TCGA-B0-4834-01A-01R-1305-07	stage i	t1a	male		
TCGA-A3-A8OW-01A-11R-A37O-07	stage iii	t3	male		
TCGA-A3-3385-01A-02R-1420-07	stage iii	t3b	male		
TCGA-CZ-4866-01A-01R-1503-07	stage iii	t3b	male		
TCGA-BP-4959-01A-01R-1334-07	stage i	t1a	male		
TCGA-AK-3456-01A-02R-1325-07	stage i	t1a	male		
TCGA-A3-3370-01A-02R-1420-07	stage i	t1a	female		
TCGA-BP-4334-01A-01R-1289-07	stage ii	t2	male		
TCGA-B0-5707-01A-11R-1541-07	stage i	t1b	male		
TCGA-BP-5174-01A-01R-1426-07	stage iii	t3a	male		
TCGA-BP-5190-01A-01R-1426-07	stage iii	t3b	female		
TCGA-B2-3923-01A-02R-1325-07	stage i	t1a	female		
TCGA-CJ-5680-01A-11R-1541-07	stage i	t1b	female		
TCGA-CJ-4638-01A-02R-1325-07	stage i	t1	female		
TCGA-A3-3313-01A-02R-1325-07	stage i	t1a	female		

TCGA-BP-4756-01A-01R-1289-07	stage iv	t4	male		
TCGA-B0-5083-01A-02R-1420-07	stage iv	t4	male		
TCGA-CJ-5681-01A-11R-1541-07	stage i	t1a	female		
TCGA-B8-A54G-01A-11R-A266-07	stage i	t1a	female		
TCGA-B8-A54D-01A-21R-A266-07	stage i	t1a	female		
TCGA-B0-5117-01A-01R-1420-07	stage iii	t3a	male		
TCGA-A3-3328-01A-01R-0864-07	stage i	t1b	male		
TCGA-AK-3465-01A-02R-1325-07	stage i	t1b	male		
TCGA-B4-5835-01A-11R-1672-07	stage i	t1b	female		
TCGA-CW-5591-01A-01R-1541-07	stage i	t1a	male		
TCGA-B0-5107-01A-01R-1420-07	stage ii	t2	male		
TCGA-B8-4621-01A-01R-1503-07	stage i	t1	male		
TCGA-AK-3433-01A-02R-1277-07	stage i	t1b	female		
TCGA-G6-A5PC-01A-11R-A33J-07	stage ii	t2	male		
TCGA-T7-A92I-01A-11R-A37O-07	stage iii	t3a	male		
TCGA-B8-A54E-01A-11R-A266-07	stage i	t1a	male		
TCGA-BP-4761-01A-01R-1289-07	stage iv	t3a	male		
TCGA-AK-3447-01A-01R-1766-07	stage i	t1	male		
TCGA-AS-3777-01A-01R-0864-07	stage i	t1b	male		
TCGA-AK-3427-01A-01R-0864-07	stage i	t1a	male		
TCGA-B8-5546-01A-01R-1541-07	stage i	t1b	female		
TCGA-AK-3443-01A-02R-1325-07	stage i	t1a	male		
TCGA-AK-3453-01A-02R-1277-07	stage ii	t2b	female		
TCGA-AK-3451-01A-02R-1188-07	stage i	t1a	male		
TCGA-BP-4983-01A-01R-1334-07	stage iv	t3a	male		
TCGA-B0-5097-01A-01R-1420-07	stage ii	t2	male		
TCGA-B0-4698-01A-01R-1503-07	stage ii	t2	female		
TCGA-BP-4799-01A-01R-1305-07	stage ii	t2a	female		
TCGA-BP-4326-01A-01R-1289-07	stage iii	t3a	male	B3GNT5	>75 Percentile

TCGA-B8-4620-01A-02R-1325-07	stage ii	t2	male		
TCGA-B4-5832-01A-11R-1672-07	stage ii	t2	male		
TCGA-EU-5904-01A-11R-1672-07	stage i	t1b	male		
TCGA-B0-5690-01A-11R-1541-07	stage ii	t2a	male		
TCGA-B0-5698-01A-11R-1672-07	stage i	t1a	female		
TCGA-BP-5200-01A-01R-1426-07	stage i	t1	male		
TCGA-BP-4985-01A-01R-1334-07	stage iii	t3b	female		
TCGA-AK-3454-01A-02R-1277-07	stage iii	t3a	male		
TCGA-DV-5565-01A-01R-1541-07	stage ii	t2	male		
TCGA-BP-4781-01A-01R-1305-07	stage iii	t3c	male		
TCGA-BP-4354-01A-02R-1289-07	stage iv	t3b	female		
TCGA-B4-5835-01A-11R-1672-07	stage i	t1b	female		
TCGA-BP-4176-01A-02R-1289-07	stage i	t1a	male		
TCGA-B0-4712-01A-01R-1503-07	stage iv	t1b	female		
TCGA-B0-5099-01A-01R-1420-07	stage i	t1a	female		
TCGA-CZ-5468-01A-01R-1503-07	stage i	t1a	female		
TCGA-B0-4693-01A-01R-1277-07	stage i	t1a	male		
TCGA-BP-4162-01A-02R-1325-07	stage iii	t3a	male		
TCGA-BP-4343-01A-02R-1289-07	stage i	t1a	male		
TCGA-BP-4352-01A-01R-1289-07	stage iii	t3a	male		
TCGA-B0-4841-01A-01R-1277-07	stage i	t1b	male		
TCGA-CJ-4868-01A-01R-1305-07	stage iv	t2	female		
TCGA-BP-5198-01A-01R-1426-07	stage i	t1a	male		
TCGA-CJ-4920-01A-01R-1426-07	stage i	t1a	male		
TCGA-BP-4335-01A-01R-1289-07	stage i	t1b	male		
TCGA-DV-5569-01A-01R-1541-07	stage iii	t3c	female		
TCGA-B0-4690-01A-01R-1277-07	stage iii	t3a	male		
TCGA-CZ-5988-01A-11R-1672-07	stage iii	t3b	male		
TCGA-CJ-4878-01A-01R-1305-07	stage i	t1a	male		

TCGA-B0-5711-01A-11R-1672-07	stage ii	t2	female			
TCGA-BP-4776-01A-01R-1289-07	stage iv	t4	male			
TCGA-BP-5168-01A-01R-1420-07	stage i	t1a	male			
TCGA-B0-5098-01A-01R-1420-07	stage ii	t2	male			
TCGA-CJ-4871-01A-01R-1305-07	stage i	t1b	male			
TCGA-B0-4813-01A-01R-1277-07	stage iii	t3a	male			
TCGA-B0-5710-01A-11R-1672-07	stage i	t1b	female			
TCGA-CZ-5461-01A-01R-1503-07	stage i	t1a	female			
TCGA-A3-3331-01A-02R-1325-07	stage i	t1a	female			
TCGA-BP-4774-01A-01R-1289-07	stage iii	t3b	male			
TCGA-BP-5008-01A-01R-1334-07	stage iii	t3a	male			
TCGA-BP-4169-01A-02R-1289-07	stage ii	t2	female			
TCGA-CJ-4872-01A-01R-1305-07	stage i	t1b	female			
TCGA-BP-4345-01A-01R-1289-07	stage iv	t4	male			
TCGA-BP-4775-01A-01R-1289-07	stage i	t1a	male			
TCGA-BP-5009-01A-01R-1334-07	stage i	t1a	male			
TCGA-CJ-4902-01A-01R-1426-07	stage iii	t3a	male			
TCGA-B0-4846-01A-01R-1277-07	stage i	t1	male			
TCGA-BP-4329-01A-02R-1289-07	stage ii	t2	male			
TCGA-B0-5108-01A-01R-1420-07	stage iii	t3a	male			
TCGA-AK-3428-01A-02R-1277-07	stage i	t1	male			
TCGA-B0-4849-01A-01R-1277-07	stage ii	t2	male			
TCGA-B0-5080-01A-01R-1503-07	stage i	t1b	male			
TCGA-A3-3320-01A-02R-1325-07	stage i	t1a	male			
TCGA-CJ-6028-01A-11R-1672-07	stage i	t1b	female			
TCGA-B0-5706-01A-11R-1541-07	stage i	t1a	female			
TCGA-G6-A8L6-01A-11R-A37O-07	stage i	t1b	male			
TCGA-BP-4972-01A-01R-1334-07	stage iv	t3a	male			
TCGA-A3-3387-01A-01R-1541-07	stage i	t1a	male			

TCGA-CZ-5467-01A-01R-1503-07	stage iv	t3a	male		
TCGA-A3-A6NL-01A-11R-A33J-07	stage i	t1b	male		
TCGA-CW-5580-01A-01R-1672-07	stage iii	t3a	female		
TCGA-DV-5568-01A-01R-1541-07	stage ii	t2	male		
TCGA-B0-4845-01A-01R-1277-07	stage i	t1a	female		
TCGA-CJ-4907-01A-01R-1426-07	stage iii	t3a	female		
TCGA-B0-4718-01A-01R-1277-07	stage iii	t3a	male		
TCGA-B0-5812-01A-11R-1672-07	stage i	t1a	female		
TCGA-CJ-6033-01A-11R-1672-07	stage i	t1b	female		
TCGA-B8-A7U6-01A-12R-A37O-07	stage i	t1a	female		
TCGA-CZ-4861-01A-01R-1305-07	stage iii	t3a	male		
TCGA-B2-4101-01A-02R-1277-07	stage ii	t2a	male		
TCGA-BP-4967-01A-01R-1334-07	stage iii	t3a	female		
TCGA-BP-4986-01A-01R-1334-07	stage iii	t3a	female		
TCGA-B4-5844-01A-11R-1672-07	stage i	t1a	male		
TCGA-B0-5110-01A-01R-1420-07	stage i	t1b	female		
TCGA-BP-4160-01A-02R-1289-07	stage iv	t3a	male		
TCGA-CJ-4639-01A-02R-1325-07	stage ii	t2	male		
TCGA-A3-3347-01A-02R-1325-07	stage i	t1a	female		
TCGA-CZ-5465-01A-01R-1503-07	stage i	t1a	male		
TCGA-BP-5004-01A-01R-1334-07	stage i	t1	female		
TCGA-B0-5694-01A-11R-1541-07	stage i	t1	female		
TCGA-BP-5189-01A-02R-1426-07	stage i	t1a	female		
TCGA-B0-4821-01A-01R-1503-07	stage iii	t3a	male		
TCGA-AK-3431-01A-02R-1277-07	stage i	t1b	male		
TCGA-BP-4991-01A-01R-1334-07	stage iii	t3a	female		
TCGA-BP-4975-01A-01R-1334-07	stage ii	t2	female		
TCGA-A3-3316-01A-01R-0864-07	stage i	t1a	male		
TCGA-B0-4852-01A-01R-1503-07	stage ii	t2	female		

TCGA-A3-3373-01A-02R-1420-07	stage ii	t2	male		
TCGA-CZ-4858-01A-01R-1305-07	stage iii	t3b	female		
TCGA-BP-4995-01A-01R-1334-07	stage ii	t2	male		
TCGA-B0-4819-01A-01R-1277-07	stage iii	t3a	male		
TCGA-B8-4143-01A-01R-1188-07	stage i	t1	female		
TCGA-BP-4166-01A-02R-1289-07	stage ii	t2	female		
TCGA-B0-4700-01A-02R-1541-07	stage i	t1b	female		
TCGA-CZ-4857-01A-01R-1305-07	stage iv	t3a	female		
TCGA-CJ-4885-01A-01R-1305-07	stage iv	t3	female		
TCGA-B0-5106-01A-01R-1420-07	stage i	t1	female		
TCGA-AK-3440-01A-02R-1277-07	stage i	t1a	male		
TCGA-B8-5163-01A-01R-1420-07	stage i	t1b	male		
TCGA-B8-A8YJ-01A-13R-A39I-07	stage i	t1a	female		
TCGA-BP-4960-01A-01R-1334-07	stage iv	t3b	male		
TCGA-BP-5201-01A-01R-1426-07	stage i	t1a	male		
TCGA-B0-4836-01A-01R-1305-07	stage i	t1b	male		
TCGA-CZ-5454-01A-01R-1503-07	stage iv	t3b	female		
TCGA-BP-5006-01A-01R-1334-07	stage i	t1	male		
TCGA-B8-4622-01A-02R-1277-07	stage i	t1a	female		
TCGA-B0-5096-01A-01R-1420-07	stage ii	t2	female		
TCGA-AK-3436-01A-02R-1325-07	stage i	t1b	male		
TCGA-B0-4843-01A-01R-1277-07	stage i	t1a	female		
TCGA-CJ-4881-01A-01R-1305-07	stage iv	t3	male		
TCGA-CZ-5456-01A-01R-1503-07	stage iii	t3a	female		
TCGA-B8-5549-01A-01R-1541-07	stage i	t1a	male		
TCGA-A3-3358-01A-01R-1541-07	stage i	t1a	female		
TCGA-CJ-6032-01A-11R-1672-07	stage i	t1b	male		
TCGA-CJ-4905-01A-02R-1426-07	stage i	t1a	male		
TCGA-A3-3329-01A-01R-0864-07	stage i	t1b	female		

TCGA-B0-5402-01A-01R-1503-07	stage i	t1b	female		
TCGA-A3-3343-01A-01R-0864-07	stage i	t1a	female		
TCGA-BP-4963-01A-01R-1334-07	stage i	t1b	female		
TCGA-BP-4341-01A-01R-1289-07	stage i	t1a	male		
TCGA-BP-4994-01A-01R-1334-07	stage iv	t3b	male		
TCGA-B0-5700-01A-11R-1541-07	stage i	t1a	male		
TCGA-B0-4701-01A-01R-1277-07	stage i	t1b	female		
TCGA-A3-3352-01A-01R-0864-07	stage i	t1a	male		
TCGA-CJ-5682-01A-11R-1541-07	stage i	t1a	male		
TCGA-BP-5180-01A-01R-1426-07	stage iv	t2	male		
TCGA-B0-5709-01A-11R-1541-07	stage i	t1b	female		
TCGA-A3-3319-01A-02R-1325-07	stage i	t1a	male		
TCGA-A3-3313-01A-02R-1325-07	stage i	t1a	female		
TCGA-B2-4098-01A-02R-1325-07	stage ii	t2	male		
TCGA-B0-4847-01A-01R-1277-07	stage i	t1a	female		
TCGA-B0-5083-01A-02R-1420-07	stage iv	t4	male		
TCGA-B0-5077-01A-01R-1334-07	stage i	t1b	female		
TCGA-B0-5121-01A-02R-1420-07	stage i	t1a	female		
TCGA-BP-4801-01A-02R-1420-07	stage iii	t3b	female		
TCGA-CW-5585-01A-01R-1541-07	stage ii	t2	female		
TCGA-B2-3923-01A-02R-1325-07	stage i	t1a	female		
TCGA-CJ-5675-01A-11R-1541-07	stage iii	t3a	male		
TCGA-CJ-4641-01A-02R-1325-07	stage iii	t3a	female		
TCGA-BP-5001-01A-01R-1334-07	stage iii	t3b	female		
TCGA-B0-5691-01A-11R-1541-07	stage i	t1b	male		
TCGA-A3-3328-01A-01R-0864-07	stage i	t1b	male		
TCGA-BP-5196-01A-01R-1426-07	stage iii	t3a	male		
TCGA-A3-A6NJ-01A-12R-A33J-07	stage i	t1a	female		
TCGA-B8-5553-01A-01R-1541-07	stage i	t1	male		<25 Percentile

TCGA-G6-A8L7-01A-11R-A37O-07	stage i	t1a	male		
TCGA-B8-4619-01A-02R-1325-07	stage iii	t3a	male		
TCGA-AK-3458-01A-01R-1503-07	stage i	t1a	female		
TCGA-AK-3455-01A-01R-0864-07	stage i	t1b	female		
TCGA-BP-4798-01A-01R-1305-07	stage i	t1a	female		
TCGA-B4-5843-01A-11R-1672-07	stage i	t1b	male		
TCGA-EU-5907-01A-11R-1672-07	stage i	t1a	male		
TCGA-AK-3451-01A-02R-1188-07	stage i	t1a	male		
TCGA-BP-5183-01A-01R-1426-07	stage i	t1b	male		
TCGA-CJ-4897-01A-03R-1426-07	stage iv	t3a	female		
TCGA-A3-A6NN-01A-12R-A33J-07	stage i	t1a	male		
TCGA-A3-3374-01A-02R-1325-07	stage iii	t3a	female		
TCGA-CJ-5681-01A-11R-1541-07	stage i	t1a	female		
TCGA-DV-5573-01A-01R-1541-07	stage iii	t3a	male		
TCGA-BP-4177-01A-02R-1420-07	stage ii	t2	male		
TCGA-BP-4970-01A-01R-1334-07	stage iv	t3a	male		
TCGA-B0-5117-01A-01R-1420-07	stage iii	t3a	male		
TCGA-A3-A6NI-01A-11R-A33J-07	stage iii	t3a	male		
TCGA-AK-3443-01A-02R-1325-07	stage i	t1a	male		
TCGA-BP-4971-01A-01R-1334-07	stage iv	t3a	male		
TCGA-DV-5567-01A-01R-1541-07	stage iii	t3a	male		
TCGA-CJ-4873-01A-01R-1305-07	stage i	t1a	male		
TCGA-B0-4814-01A-01R-1277-07	stage ii	t2	male		
TCGA-B0-5705-01A-11R-1541-07	stage i	t1b	male		
TCGA-BP-5184-01A-01R-1426-07	stage i	t1b	female		
TCGA-BP-4167-01A-02R-1325-07	stage iii	t3b	male		
TCGA-CJ-5677-01A-11R-1541-07	stage i	t1b	female		
TCGA-B0-4713-01A-01R-1277-07	stage ii	t2	male		
TCGA-B0-5092-01A-01R-1420-07	stage ii	t2	female		

TCGA-BP-5191-01A-01R-1426-07	stage iii	t3b	male		
TCGA-BP-4987-01A-01R-1334-07	stage i	t1a	male		
TCGA-B2-5635-01A-01R-1541-07	stage i	t1a	female		
TCGA-BP-4988-01A-01R-1334-07	stage ii	t2	female		
TCGA-A3-3376-01A-02R-1420-07	stage iii	t3a	male		
TCGA-B2-A4SR-01A-11R-A266-07	stage i	t1	male		
TCGA-CZ-4866-01A-01R-1503-07	stage iii	t3b	male		
TCGA-A3-3380-01A-01R-0864-07	stage i	t1b	male		
TCGA-BP-4795-01A-02R-1420-07	stage ii	t2a	male		
TCGA-3Z-A93Z-01A-11R-A37O-07	stage i	t1b	female		
TCGA-B0-5707-01A-11R-1541-07	stage i	t1b	male		
TCGA-AK-3433-01A-02R-1277-07	stage i	t1b	female		
TCGA-A3-3370-01A-02R-1420-07	stage i	t1a	female		
TCGA-BP-4962-01A-01R-1334-07	stage i	t1b	female		
TCGA-A3-A8OU-01A-11R-A37O-07	stage i	t1a	male		
TCGA-B8-A54J-01A-11R-A33J-07	stage i	t1a	male		
TCGA-BP-4771-01A-01R-1289-07	stage iii	t3a	male		
TCGA-CZ-4856-01A-02R-1426-07	stage i	t1b	female		
TCGA-DV-5576-01A-01R-1541-07	stage i	t1a	male		
TCGA-B8-A54K-01A-11R-A33J-07	stage iii	t3b	female		
TCGA-CJ-5680-01A-11R-1541-07	stage i	t1b	female		
TCGA-BP-4163-01A-02R-1325-07	stage i	t1a	male		
TCGA-BP-4961-01A-01R-1334-07	stage i	t1b	male		
TCGA-BP-5176-01A-01R-1426-07	stage i	t1a	female		
TCGA-CJ-5672-01A-11R-1541-07	stage i	t1b	male		
TCGA-CJ-4899-01A-01R-1334-07	stage i	t1b	female		
TCGA-BP-4337-01A-01R-1289-07	stage i	t1a	male		
TCGA-BP-4347-01A-01R-1289-07	stage iv	t2	male		
TCGA-CJ-4889-01A-01R-1305-07	stage i	t1	female		

TCGA-AK-3456-01A-02R-1325-07	stage i	t1a	male		
TCGA-BP-4993-01A-02R-1420-07	stage i	t1a	male		
TCGA-B2-5641-01A-01R-1541-07	stage iii	t3b	female		
TCGA-CZ-4863-01A-01R-1503-07	stage iii	t3a	male		
TCGA-B0-4842-01A-02R-1420-07	stage i	t1a	female		
TCGA-B8-A54F-01A-11R-A266-07	stage i	t1a	male		
TCGA-B0-5100-01A-01R-1420-07	stage i	t1a	male		
TCGA-A3-3363-01A-01R-0864-07	NA	t1a	male		
TCGA-A3-3326-01A-01R-0864-07	stage i	t1b	female		
TCGA-BP-4346-01A-01R-1289-07	stage iv	t4	male		
TCGA-BP-4761-01A-01R-1289-07	stage iv	t3a	male		
TCGA-B0-5712-01A-11R-1672-07	stage i	t1a	male		
TCGA-A3-3349-01A-01R-1188-07	stage i	t1a	male		
TCGA-CZ-5459-01A-01R-1503-07	stage i	t1a	male		
TCGA-B4-5836-01A-11R-1672-07	stage i	t1a	male		
TCGA-CW-5587-01A-01R-1541-07	stage i	t1b	female		
TCGA-B8-A54G-01A-11R-A266-07	stage i	t1a	female		
TCGA-AK-3427-01A-01R-0864-07	stage i	t1a	male		
TCGA-BP-4969-01A-01R-1334-07	stage iv	t3a	male		
TCGA-MW-A4EC-01A-11R-A266-07	stage iii	t1a	male		
TCGA-BP-4782-01A-02R-1420-07	stage i	t1b	male		
TCGA-CJ-4634-01A-02R-1325-07	stage iii	t3b	male		
TCGA-B8-5165-01A-01R-1420-07	stage iii	t3	male		
TCGA-B0-5107-01A-01R-1420-07	stage ii	t2	male		
TCGA-A3-A8OV-01A-11R-A37O-07	stage iii	t3b	female		
TCGA-CZ-5457-01A-01R-1503-07	stage i	t1b	female		
TCGA-B0-4707-01A-01R-1277-07	stage ii	t2	male		
TCGA-B8-A54H-01A-11R-A33J-07	stage i	t1b	male		
TCGA-6D-AA2E-01A-11R-A37O-07	stage i	t1b	male		

TCGA-BP-5190-01A-01R-1426-07	stage iii	t3b	female		
TCGA-B8-5159-01A-01R-1420-07	stage i	t1a	male		
TCGA-BP-5186-01A-01R-1426-07	stage i	t1b	male		
TCGA-B0-4706-01A-01R-1503-07	stage ii	t2	female		
TCGA-BP-5185-01A-01R-1426-07	stage i	t1a	female		
TCGA-A3-3362-01A-02R-1325-07	stage iii	t3a	male		
TCGA-CZ-5452-01A-01R-1503-07	stage i	t1a	female		
TCGA-BP-4976-01A-01R-1334-07	stage iii	t3a	male		
TCGA-MM-A84U-01A-11R-A37O-07	stage i	t1b	male		
TCGA-CJ-4638-01A-02R-1325-07	stage i	t1	female		
TCGA-A3-A8OW-01A-11R-A37O-07	stage iii	t3	male		
TCGA-A3-A8CQ-01A-11R-A37O-07	stage i	t1a	female		
TCGA-B8-A54E-01A-11R-A266-07	stage i	t1a	male		
TCGA-BP-5174-01A-01R-1426-07	stage iii	t3a	male		
TCGA-B2-5636-01A-02R-1541-07	stage i	t1b	female		
TCGA-AS-3777-01A-01R-0864-07	stage i	t1b	male		
TCGA-BP-4756-01A-01R-1289-07	stage iv	t4	male		
TCGA-B0-4834-01A-01R-1305-07	stage i	t1a	male		
TCGA-T7-A92I-01A-11R-A37O-07	stage iii	t3a	male		
TCGA-G6-A5PC-01A-11R-A33J-07	stage ii	t2	male		
TCGA-B8-5546-01A-01R-1541-07	stage i	t1b	female		
TCGA-AK-3453-01A-02R-1277-07	stage ii	t2b	female		
TCGA-B0-4696-01A-01R-1277-07	stage iii	t3b	female		
TCGA-B0-5702-01A-11R-1541-07	stage i	t1a	male		
TCGA-B0-5104-01A-01R-1420-07	stage iii	t3a	male		
TCGA-B0-4710-01A-01R-1503-07	stage i	t1b	male		
TCGA-BP-4985-01A-01R-1334-07	stage iii	t3b	female		
TCGA-BP-4351-01A-01R-1289-07	stage iii	t3b	male		
TCGA-B4-5377-01A-01R-1503-07	stage i	t1b	male	GPR135	>75 Percentile

TCGA-BP-4170-01A-02R-1289-07	stage ii	t2	female		
TCGA-B0-4701-01A-01R-1277-07	stage i	t1b	female		
TCGA-B2-4098-01A-02R-1325-07	stage ii	t2	male		
TCGA-B0-4837-01A-01R-1305-07	stage iii	t3	male		
TCGA-CJ-4635-01A-02R-1305-07	stage i	t1b	male		
TCGA-BP-4756-01A-01R-1289-07	stage iv	t4	male		
TCGA-BP-5187-01A-01R-1426-07	stage i	t1b	male		
TCGA-B8-4620-01A-02R-1325-07	stage ii	t2	male		
TCGA-BP-4352-01A-01R-1289-07	stage iii	t3a	male		
TCGA-BP-4766-01A-01R-1289-07	stage iii	t3b	female		
TCGA-CJ-4870-01A-01R-1305-07	stage ii	t2	male		
TCGA-B4-5378-01A-01R-1503-07	stage ii	t2	male		
TCGA-BP-4781-01A-01R-1305-07	stage iii	t3c	male		
TCGA-BP-4325-01A-02R-1289-07	stage i	t1b	female		
TCGA-BP-4987-01A-01R-1334-07	stage i	t1a	male		
TCGA-B0-4821-01A-01R-1503-07	stage iii	t3a	male		
TCGA-BP-4992-01A-01R-1334-07	stage iv	t1a	female		
TCGA-BP-4993-01A-02R-1420-07	stage i	t1a	male		
TCGA-B8-A54J-01A-11R-A33J-07	stage i	t1a	male		
TCGA-DV-5565-01A-01R-1541-07	stage ii	t2	male		
TCGA-B0-5119-01A-02R-1420-07	stage i	t1	female		
TCGA-A3-A6NJ-01A-12R-A33J-07	stage i	t1a	female		
TCGA-BP-4774-01A-01R-1289-07	stage iii	t3b	male		
TCGA-B8-A54H-01A-11R-A33J-07	stage i	t1b	male		
TCGA-B8-A54F-01A-11R-A266-07	stage i	t1a	male		
TCGA-DV-5567-01A-01R-1541-07	stage iii	t3a	male		
TCGA-AK-3445-01A-02R-1277-07	stage i	t1a	male		
TCGA-CZ-4859-01A-02R-1426-07	stage i	t1b	male		
TCGA-B0-4822-01A-01R-1277-07	stage ii	t2b	male		

TCGA-CZ-4857-01A-01R-1305-07	stage iv	t3a	female		
TCGA-CJ-4634-01A-02R-1325-07	stage iii	t3b	male		
TCGA-BP-4355-01A-01R-1289-07	stage iv	t4	male		
TCGA-B0-4696-01A-01R-1277-07	stage iii	t3b	female		
TCGA-BP-4349-01A-01R-1289-07	stage iii	t3a	female		
TCGA-MM-A564-01A-11R-A266-07	stage i	t1a	female		
TCGA-BP-4988-01A-01R-1334-07	stage ii	t2	female		
TCGA-G6-A8L8-01A-21R-A37O-07	stage i	t1a	female		
TCGA-BP-4165-01A-02R-1289-07	stage iii	t3b	male		
TCGA-B0-4846-01A-01R-1277-07	stage i	t1	male		
TCGA-BP-4335-01A-01R-1289-07	stage i	t1b	male		
TCGA-AK-3425-01A-02R-1277-07	stage i	t1b	male		
TCGA-BP-4169-01A-02R-1289-07	stage ii	t2	female		
TCGA-GK-A6C7-01A-11R-A33J-07	stage iii	t3a	male		
TCGA-BP-4971-01A-01R-1334-07	stage iv	t3a	male		
TCGA-B8-A54K-01A-11R-A33J-07	stage iii	t3b	female		
TCGA-B0-4697-01A-01R-1277-07	stage ii	t2	female		
TCGA-CJ-4882-01A-02R-1426-07	stage i	t1a	male		
TCGA-CJ-4876-01A-01R-1305-07	stage i	t1b	male		
TCGA-CJ-4643-01A-02R-1325-07	stage i	t1b	male		
TCGA-AK-3431-01A-02R-1277-07	stage i	t1b	male		
TCGA-CJ-4895-01A-01R-1305-07	stage ii	t2	female		
TCGA-B0-5100-01A-01R-1420-07	stage i	t1a	male		
TCGA-BP-5201-01A-01R-1426-07	stage i	t1a	male		
TCGA-CJ-4891-01A-01R-1305-07	stage i	t1a	male		
TCGA-BP-4158-01A-02R-1289-07	stage iii	t3a	male		
TCGA-B0-5113-01A-01R-1420-07	stage i	t1	male		
TCGA-CJ-4878-01A-01R-1305-07	stage i	t1a	male		
TCGA-B0-5693-01A-11R-1541-07	stage i	t1b	male		

TCGA-B0-5695-01A-11R-1541-07	stage ii	t2	male		
TCGA-A3-3322-01A-02R-1325-07	stage i	t1a	female		
TCGA-CZ-5982-01A-11R-1672-07	stage i	t1a	male		
TCGA-B0-5107-01A-01R-1420-07	stage ii	t2	male		
TCGA-CJ-4874-01A-01R-1305-07	stage ii	t2a	male		
TCGA-A3-3320-01A-02R-1325-07	stage i	t1a	male		
TCGA-CZ-5456-01A-01R-1503-07	stage iii	t3a	female		
TCGA-CZ-5461-01A-01R-1503-07	stage i	t1a	female		
TCGA-A3-3372-01A-02R-1325-07	stage iii	t1b	male		
TCGA-CZ-4860-01A-01R-1305-07	stage i	t1b	female		
TCGA-B0-4718-01A-01R-1277-07	stage iii	t3a	male		
TCGA-CZ-4854-01A-01R-1305-07	stage iv	t3a	male		
TCGA-BP-4340-01A-01R-1289-07	stage i	t1b	female		
TCGA-B0-4845-01A-01R-1277-07	stage i	t1a	female		
TCGA-CZ-5466-01A-01R-1503-07	stage iv	t4	female		
TCGA-CZ-4862-01A-01R-1305-07	stage i	t1b	male		
TCGA-CJ-4920-01A-01R-1426-07	stage i	t1a	male		
TCGA-B0-5095-01A-01R-1420-07	stage iii	t3a	female		
TCGA-AS-3778-01A-01R-A32Z-07	stage i	t1a	male		
TCGA-BP-4775-01A-01R-1289-07	stage i	t1a	male		
TCGA-CZ-5469-01A-01R-1503-07	stage i	t1a	male		
TCGA-BP-4969-01A-01R-1334-07	stage iv	t3a	male		
TCGA-BP-4344-01A-01R-1289-07	stage iii	t3a	male		
TCGA-A3-A8OU-01A-11R-A37O-07	stage i	t1a	male		
TCGA-BP-4964-01A-01R-1334-07	stage iv	t2	male		
TCGA-CZ-4853-01A-01R-1426-07	stage iii	t3a	male		
TCGA-B0-5705-01A-11R-1541-07	stage i	t1b	male		
TCGA-BP-4960-01A-01R-1334-07	stage iv	t3b	male		
TCGA-B8-4622-01A-02R-1277-07	stage i	t1a	female		

TCGA-BP-4329-01A-02R-1289-07	stage ii	t2	male		
TCGA-B0-4714-01A-01R-1277-07	stage iii	t3a	female		
TCGA-BP-4164-01A-02R-1325-07	stage iii	t3a	male		
TCGA-A3-3313-01A-02R-1325-07	stage i	t1a	female		
TCGA-BP-5006-01A-01R-1334-07	stage i	t1	male		
TCGA-CJ-4923-01A-01R-1426-07	stage i	t1a	male		
TCGA-B0-4827-01A-02R-1420-07	stage ii	t2	male		
TCGA-B0-5121-01A-02R-1420-07	stage i	t1a	female		
TCGA-A3-A8OW-01A-11R-A37O-07	stage iii	t3	male		
TCGA-CJ-4889-01A-01R-1305-07	stage i	t1	female		
TCGA-B2-5636-01A-02R-1541-07	stage i	t1b	female		
TCGA-B0-5698-01A-11R-1672-07	stage i	t1a	female		
TCGA-G6-A8L6-01A-11R-A37O-07	stage i	t1b	male		
TCGA-B0-4706-01A-01R-1503-07	stage ii	t2	female		
TCGA-BP-4769-01A-01R-1289-07	stage iii	t3a	male		
TCGA-B0-4852-01A-01R-1503-07	stage ii	t2	female		
TCGA-CZ-5459-01A-01R-1503-07	stage i	t1a	male		
TCGA-MM-A84U-01A-11R-A37O-07	stage i	t1b	male		
TCGA-B0-4703-01A-01R-1277-07	stage ii	t2b	female		
TCGA-BP-4999-01A-01R-1334-07	stage i	t1a	female		
TCGA-BP-4768-01A-01R-1289-07	stage iv	t3b	male		
TCGA-A3-A8CQ-01A-11R-A37O-07	stage i	t1a	female		
TCGA-BP-4337-01A-01R-1289-07	stage i	t1a	male		
TCGA-B0-4849-01A-01R-1277-07	stage ii	t2	male		
TCGA-CZ-5462-01A-01R-1503-07	stage i	t1a	male		
TCGA-BP-4801-01A-02R-1420-07	stage iii	t3b	female		
TCGA-DV-5566-01A-01R-1541-07	stage iv	t3b	male		
TCGA-BP-4799-01A-01R-1305-07	stage ii	t2a	female		
TCGA-B8-A7U6-01A-12R-A37O-07	stage i	t1a	female		

TCGA-B0-5120-01A-01R-1420-07	stage i	t1a	male		
TCGA-DV-5575-01A-01R-1541-07	stage i	t1b	male		
TCGA-BP-5185-01A-01R-1426-07	stage i	t1a	female		
TCGA-A3-3331-01A-02R-1325-07	stage i	t1a	female		
TCGA-BP-4342-01A-01R-1289-07	stage i	t1b	female		
TCGA-CJ-4908-01A-01R-1426-07	stage iv	t3a	male		
TCGA-BP-4341-01A-01R-1289-07	stage i	t1a	male		
TCGA-B0-4688-01A-01R-1277-07	stage i	t1b	male		
TCGA-B0-4841-01A-01R-1277-07	stage i	t1b	male		
TCGA-A3-3362-01A-02R-1325-07	stage iii	t3a	male		
TCGA-B0-5703-01A-11R-1541-07	stage i	t1a	male		
TCGA-CJ-5681-01A-11R-1541-07	stage i	t1a	female		
TCGA-CJ-4912-01A-01R-1426-07	stage iii	t3a	male		
TCGA-AK-3434-01A-02R-1277-07	stage i	t1b	male		
TCGA-AK-3454-01A-02R-1277-07	stage iii	t3a	male		
TCGA-B0-5697-01A-11R-1541-07	stage i	t1b	female		
TCGA-AK-3444-01A-02R-1325-07	stage i	t1b	male		
TCGA-BP-4961-01A-01R-1334-07	stage i	t1b	male		
TCGA-B0-4817-01A-01R-1277-07	stage ii	t2b	female		
TCGA-AK-3458-01A-01R-1503-07	stage i	t1a	female		
TCGA-AK-3436-01A-02R-1325-07	stage i	t1b	male		
TCGA-BP-5190-01A-01R-1426-07	stage iii	t3b	female		
TCGA-BP-5189-01A-02R-1426-07	stage i	t1a	female		
TCGA-BP-4965-01A-01R-1334-07	stage i	t1b	male		
TCGA-B0-4699-01A-01R-1277-07	stage iii	t3a	male		
TCGA-CJ-5680-01A-11R-1541-07	stage i	t1b	female		
TCGA-CZ-4858-01A-01R-1305-07	stage iii	t3b	female		
TCGA-CZ-5464-01A-01R-1503-07	stage i	t1a	female		
TCGA-CZ-4866-01A-01R-1503-07	stage iii	t3b	male		<25 Percentile

TCGA-BP-4763-01A-01R-1289-07	stage iii	t3a	male		
TCGA-B4-5844-01A-11R-1672-07	stage i	t1a	male		
TCGA-B0-4698-01A-01R-1503-07	stage ii	t2	female		
TCGA-B0-5692-01A-11R-1541-07	stage ii	t2b	female		
TCGA-B4-5843-01A-11R-1672-07	stage i	t1b	male		
TCGA-BP-5169-01A-01R-1426-07	stage i	t1	female		
TCGA-CJ-4905-01A-02R-1426-07	stage i	t1a	male		
TCGA-BP-5009-01A-01R-1334-07	stage i	t1a	male		
TCGA-CW-5587-01A-01R-1541-07	stage i	t1b	female		
TCGA-B0-5106-01A-01R-1420-07	stage i	t1	female		
TCGA-BP-5175-01A-01R-1426-07	stage iii	t3b	male		
TCGA-BP-4986-01A-01R-1334-07	stage iii	t3a	female		
TCGA-BP-4347-01A-01R-1289-07	stage iv	t2	male		
TCGA-AK-3460-01A-02R-1277-07	stage i	t1b	female		
TCGA-DV-5574-01A-01R-1541-07	stage iv	t3a	female		
TCGA-A3-3346-01A-01R-1766-07	stage i	t1a	male		
TCGA-B0-5699-01A-11R-1541-07	stage i	t1b	female		
TCGA-B8-5163-01A-01R-1420-07	stage i	t1b	male		
TCGA-B0-4823-01A-02R-1420-07	stage ii	t2	female		
TCGA-CJ-5678-01A-11R-1541-07	stage i	t1b	female		
TCGA-DV-A4VX-01A-11R-A266-07	stage i	t1a	male		
TCGA-B4-5838-01A-11R-1672-07	stage i	t1b	male		
TCGA-B0-5690-01A-11R-1541-07	stage ii	t2a	male		
TCGA-B0-5108-01A-01R-1420-07	stage iii	t3a	male		
TCGA-CJ-6033-01A-11R-1672-07	stage i	t1b	female		
TCGA-CZ-5467-01A-01R-1503-07	stage iv	t3a	male		
TCGA-B0-5712-01A-11R-1672-07	stage i	t1a	male		
TCGA-CJ-5671-01A-11R-1541-07	stage i	t1b	female		
TCGA-CZ-5452-01A-01R-1503-07	stage i	t1a	female		

TCGA-BP-4334-01A-01R-1289-07	stage ii	t2	male			
TCGA-BP-4968-01A-01R-1334-07	stage iii	t3a	male			
TCGA-AK-3428-01A-02R-1277-07	stage i	t1	male			
TCGA-B0-5109-01A-02R-1420-07	stage ii	t2b	male			
TCGA-BP-4983-01A-01R-1334-07	stage iv	t3a	male			
TCGA-B8-5158-01A-01R-1420-07	stage ii	t2	male			
TCGA-CW-5591-01A-01R-1541-07	stage i	t1a	male			
TCGA-AK-3450-01A-02R-1277-07	stage i	t1b	male			
TCGA-CJ-5684-01A-11R-1541-07	stage ii	t2a	male			
TCGA-BP-4343-01A-02R-1289-07	stage i	t1a	male			
TCGA-BP-4760-01A-02R-1420-07	stage i	t1b	male			
TCGA-B2-3924-01A-02R-1325-07	stage iii	t3a	male			
TCGA-A3-3380-01A-01R-0864-07	stage i	t1b	male			
TCGA-BP-4761-01A-01R-1289-07	stage iv	t3a	male			
TCGA-B0-5400-01A-01R-1503-07	stage i	t1a	female			
TCGA-A3-3349-01A-01R-1188-07	stage i	t1a	male			
TCGA-CZ-5463-01A-01R-1503-07	stage i	t1a	female			
TCGA-CW-5589-01A-01R-1541-07	stage ii	t2	male			
TCGA-CJ-5677-01A-11R-1541-07	stage i	t1b	female			
TCGA-B8-5164-01A-01R-1420-07	stage i	t1b	female			
TCGA-A3-3328-01A-01R-0864-07	stage i	t1b	male			
TCGA-DV-5573-01A-01R-1541-07	stage iii	t3a	male			
TCGA-B8-4621-01A-01R-1503-07	stage i	t1	male			
TCGA-B2-4102-01A-02R-1325-07	stage i	t1b	female			
TCGA-BP-4777-01A-01R-1289-07	stage ii	t2	male			
TCGA-B0-5711-01A-11R-1672-07	stage ii	t2	female			
TCGA-CZ-5985-01A-11R-1672-07	stage iv	t3a	female			
TCGA-BP-5182-01A-01R-1426-07	stage iii	t3b	male			
TCGA-BP-4795-01A-02R-1420-07	stage ii	t2a	male			

TCGA-B0-5081-01A-01R-1334-07	stage iv	t3a	male		
TCGA-B8-4619-01A-02R-1325-07	stage iii	t3a	male		
TCGA-A3-3335-01A-01R-0864-07	stage i	t1a	male		
TCGA-BP-4962-01A-01R-1334-07	stage i	t1b	female		
TCGA-CW-5581-01A-02R-1541-07	stage i	t1b	female		
TCGA-G6-A8L7-01A-11R-A37O-07	stage i	t1a	male		
TCGA-BP-5191-01A-01R-1426-07	stage iii	t3b	male		
TCGA-BP-4758-01A-01R-1289-07	stage iv	t4	male		
TCGA-B8-5162-01A-01R-1420-07	stage i	t1a	female		
TCGA-BP-4981-01A-01R-1334-07	stage iii	t3a	female		
TCGA-B0-5812-01A-11R-1672-07	stage i	t1a	female		
TCGA-CW-6090-01A-11R-1672-07	stage iii	t3a	male		
TCGA-BP-5174-01A-01R-1426-07	stage iii	t3a	male		
TCGA-AK-3453-01A-02R-1277-07	stage ii	t2b	female		
TCGA-B0-4712-01A-01R-1503-07	stage iv	t1b	female		
TCGA-A3-A8OX-01A-11R-A37O-07	stage i	t1b	male		
TCGA-CZ-5455-01A-01R-1503-07	stage iv	t4	male		
TCGA-BP-4976-01A-01R-1334-07	stage iii	t3a	male		
TCGA-BP-5170-01A-01R-1426-07	stage i	t1a	male		
TCGA-CJ-5675-01A-11R-1541-07	stage iii	t3a	male		
TCGA-AK-3465-01A-02R-1325-07	stage i	t1b	male		
TCGA-AK-3455-01A-01R-0864-07	stage i	t1b	female		
TCGA-B0-5083-01A-02R-1420-07	stage iv	t4	male		
TCGA-CZ-5468-01A-01R-1503-07	stage i	t1a	female		
TCGA-CW-5588-01A-01R-1541-07	stage ii	t2	male		
TCGA-B4-5834-01A-11R-1672-07	stage i	t1b	male		
TCGA-CJ-4875-01A-01R-1305-07	stage i	t1b	male		
TCGA-B2-5639-01A-01R-1541-07	stage i	t1b	male		
TCGA-BP-4174-01A-02R-1289-07	stage iv	t2	male		

TCGA-CW-6087-01A-11R-1672-07	stage i	t1b	female		
TCGA-B0-4842-01A-02R-1420-07	stage i	t1a	female		
TCGA-B0-5117-01A-01R-1420-07	stage iii	t3a	male		
TCGA-B0-4834-01A-01R-1305-07	stage i	t1a	male		
TCGA-B0-5702-01A-11R-1541-07	stage i	t1a	male		
TCGA-B0-5701-01A-11R-1541-07	stage i	t1a	female		
TCGA-B0-4694-01A-01R-1277-07	stage i	t1	male		
TCGA-B0-5710-01A-11R-1672-07	stage i	t1b	female		
TCGA-BP-5173-01A-01R-1426-07	stage iv	t2	female		
TCGA-B2-5641-01A-01R-1541-07	stage iii	t3b	female		
TCGA-BP-4959-01A-01R-1334-07	stage i	t1a	male		
TCGA-B0-4839-01A-01R-1305-07	stage ii	t2a	male		
TCGA-B0-4945-01A-01R-1420-07	stage i	t1a	female		
TCGA-AK-3427-01A-01R-0864-07	stage i	t1a	male		
TCGA-CZ-5988-01A-11R-1672-07	stage iii	t3b	male		
TCGA-CZ-5454-01A-01R-1503-07	stage iv	t3b	female		
TCGA-AK-3443-01A-02R-1325-07	stage i	t1a	male		
TCGA-AK-3451-01A-02R-1188-07	stage i	t1a	male		
TCGA-B4-5832-01A-11R-1672-07	stage ii	t2	male		
TCGA-AS-3777-01A-01R-0864-07	stage i	t1b	male		
TCGA-AK-3433-01A-02R-1277-07	stage i	t1b	female		
TCGA-AK-3440-01A-02R-1277-07	stage i	t1a	male		
TCGA-B2-3923-01A-02R-1325-07	stage i	t1a	female		
TCGA-AK-3447-01A-01R-1766-07	stage i	t1	male		
C)KIRP					
TCGA-F9-A4JJ-01A-11R-A24Z-07	stage iii	t3a	female		
TCGA-A4-7915-01A-11R-2204-07	stage ii	t2b	female		
TCGA-BQ-5875-01A-11R-1592-07	stage iii	t3a	female	RAN	>75 Percentile

TCGA-P4-A5E8-01A-11R-A28H-07	stage iii	t2a	male		
TCGA-BQ-5879-01A-11R-1592-07	stage iii	t3b	female		
TCGA-GL-7966-01A-11R-2204-07	stage iii	t3	female		
TCGA-BQ-5876-01A-11R-1592-07	stage i	t1a	male		
TCGA-B3-4104-01A-02R-1351-07	stage ii	t2	male		
TCGA-BQ-5893-01A-11R-1592-07	stage iv	t3a	male		
TCGA-BQ-5894-01A-11R-1592-07	stage iv	t3b	male		
TCGA-F9-A8NY-01A-11R-A36F-07	stage iv	t4	female		
TCGA-J7-8537-01A-11R-2404-07	stage iii	t3	female		
TCGA-UZ-A9PS-01A-11R-A42S-07	stage ii	t2	female		
TCGA-G7-A8LD-01A-11R-A36F-07	stage iii	t3a	male		
TCGA-BQ-5882-01A-11R-1592-07	stage iii	t3b	male		
TCGA-2Z-A9JK-01A-11R-A42S-07	stage iii	t3a	male		
TCGA-B3-4103-01A-02R-1351-07	stage i	t1a	male		
TCGA-A4-7583-01A-11R-A32Z-07	stage i	t1	male		
TCGA-B9-A44B-01A-11R-A24Z-07	stage iii	t3b	male		
TCGA-PJ-A5Z8-01A-11R-A28H-07	stage i	t1a	female		
TCGA-UZ-A9Q1-01A-11R-A42S-07	stage i	t1b	female		
TCGA-A4-7997-01A-11R-2204-07	stage i	t1b	male		
TCGA-Y8-A8RZ-01A-11R-A37K-07	stage i	t1	male		
TCGA-G7-6797-01A-11R-1965-07	stage iii	t1a	male		
TCGA-5P-A9JU-01A-11R-A42S-07	stage iii	t3a	male		
TCGA-A4-7996-01A-11R-2204-07	stage i	t1a	female		
TCGA-AL-3468-01A-02R-1351-07	stage ii	t2	male		
TCGA-AL-3473-01A-01R-1193-07	stage ii	t2	male		
TCGA-A4-A5Y0-01A-11R-A31O-07	stage i	t1b	female		
TCGA-B3-3925-01A-02R-1351-07	stage iii	t3a	male		
TCGA-B9-5155-01A-01R-1592-07	stage iii	t3a	male		
TCGA-A4-8098-01A-11R-2404-07	stage i	t1a	male		

TCGA-AL-3466-01A-02R-1351-07	stage iv	t3b	male		
TCGA-A4-8311-01A-11R-2404-07	stage i	t1a	male		
TCGA-P4-AAVM-01A-11R-A42S-07	stage i	t1a	male		
TCGA-A4-7287-01A-11R-2139-07	stage i	t1	female		
TCGA-A4-8630-01A-11R-2404-07	stage i	t1b	female		
TCGA-2Z-A9J8-01A-11R-A42S-07	stage i	t1b	male		
TCGA-IZ-8195-01A-31R-2404-07	stage ii	t2a	male		
TCGA-DW-7838-01A-11R-2139-07	stage i	t1b	male		
TCGA-V9-A7HT-01A-11R-A33Z-07	stage ii	t2	male		
TCGA-SX-A7SL-01A-11R-A355-07	stage i	t1a	male		
TCGA-A4-7585-01A-11R-2139-07	stage iii	t3a	male		
TCGA-P4-A5E7-01A-31R-A28H-07	stage i	t1b	female		
TCGA-BQ-5891-01A-11R-1592-07	stage iii	t3b	female		
TCGA-B1-A655-01A-11R-A31O-07	stage i	t1a	female		
TCGA-Y8-A8S0-01A-11R-A37K-07	stage i	t1a	male		
TCGA-AL-A5DJ-01A-11R-A26U-07	stage iii	t3a	female		
TCGA-GL-8500-01A-11R-2404-07	stage i	t1b	male		
TCGA-B3-8121-01A-21R-2404-07	stage i	t1	female		
TCGA-B9-7268-01A-11R-A32Z-07	stage ii	t2	male		
TCGA-BQ-7060-01A-11R-1965-07	stage i	t1a	male		
TCGA-GL-6846-01A-11R-1965-07	stage iv	t4	male		
TCGA-BQ-7045-01A-31R-1965-07	stage i	t1b	male		
TCGA-BQ-7044-01A-11R-1965-07	stage iii	t3a	male		
TCGA-UZ-A9PS-05A-11R-A42S-07	stage ii	t2	female		
TCGA-2Z-A9JI-01A-11R-A42S-07	stage iii	t3a	male		
TCGA-BQ-7046-01A-11R-1965-07	stage i	t1a	male		
TCGA-BQ-7061-01A-11R-1965-07	stage i	t1b	female		
TCGA-BQ-5886-01A-11R-1592-07	stage iii	t3a	male		
TCGA-J7-A8I2-01A-12R-A36F-07	stage i	t1b	male		

TCGA-A4-8310-01A-11R-2404-07	stage iii	t3a	male			
TCGA-BQ-7051-01A-12R-1965-07	stage ii	t2	male			
TCGA-UZ-A9PO-01A-11R-A38C-07	stage i	t1a	male			
TCGA-SX-A71V-01A-11R-A33Z-07	stage i	t1	male			
TCGA-A4-7584-01A-11R-2139-07	stage i	t1a	male			
TCGA-MH-A55Z-01A-11R-A26U-07	stage i	t1b	male			
TCGA-G7-6793-01A-11R-1965-07	stage iv	t3a	female			
TCGA-A4-8312-01A-11R-2404-07	stage i	t1	male			
TCGA-Y8-A898-01A-11R-A355-07	stage i	t1a	male			
TCGA-G7-A8LC-01A-11R-A36F-07	stage i	t1a	male			
TCGA-B9-4115-11A-01R-1758-07	stage i	t1a	male			
TCGA-KV-A6GE-01A-11R-A31O-07	stage i	t1a	male			
TCGA-IA-A40Y-01A-11R-A24Z-07	stage iii	t3a	female			
TCGA-BQ-7045-11A-01R-1965-07	stage i	t1b	male			
TCGA-BQ-5890-11A-01R-1592-07	stage iii	t3a	male			
TCGA-F9-A7Q0-01A-11R-A36F-07	stage i	t1b	female			
TCGA-UZ-A9PQ-01A-11R-A42S-07	stage iii	t2	male			
TCGA-F9-A7VF-01A-11R-A33Z-07	stage i	t1a	female			
TCGA-BQ-7055-11A-01R-1965-07	stage i	t1a	male			
TCGA-IZ-A6M9-01A-11R-A31O-07	stage i	t1a	male			
TCGA-2Z-A9JO-01A-11R-A42S-07	stage i	t1b	female			
TCGA-BQ-5881-01A-11R-1592-07	stage i	t1a	male			
TCGA-BQ-5888-11A-01R-1592-07	stage i	t1a	female			
TCGA-BQ-5878-11A-01R-1592-07	stage iii	t3a	female			
TCGA-SX-A7SO-01A-11R-A355-07	stage i	t1b	male			
TCGA-BQ-7059-01A-11R-1965-07	stage i	t1b	male			
TCGA-BQ-5880-01A-11R-1592-07	stage iii	t3a	male			
TCGA-KV-A6GD-01A-11R-A31O-07	stage i	t1b	male			
TCGA-HE-7130-01A-11R-1965-07	stage iii	t3	female			<25 Percentile

TCGA-DZ-6133-11A-01R-1965-07	stage i	t1a	female			
TCGA-BQ-5891-11A-01R-1592-07	stage iii	t3b	female			
TCGA-BQ-5889-01A-11R-1592-07	stage iv	t3b	male			
TCGA-A4-A48D-01A-11R-A24Z-07	stage i	t1b	male			
TCGA-P4-A5ED-01A-11R-A28H-07	stage i	t1a	male			
TCGA-BQ-5879-11A-01R-1592-07	stage iii	t3b	female			
TCGA-BQ-7044-11A-01R-1965-07	stage iii	t3a	male			
TCGA-BQ-7046-11A-01R-1965-07	stage i	t1a	male			
TCGA-2Z-A9JJ-01A-11R-A42S-07	stage i	t1a	female			
TCGA-GL-A4EM-01A-11R-A24Z-07	stage i	t1b	male			
TCGA-2Z-A9JP-01A-11R-A42S-07	stage i	t1a	male			
TCGA-BQ-7050-01A-11R-1965-07	stage i	t1a	female			
TCGA-DZ-6131-01A-11R-1965-07	stage iii	t3a	male			
TCGA-BQ-5877-11A-01R-1592-07	stage iv	t3a	male			
TCGA-DZ-6131-11A-01R-1965-07	stage iii	t3a	male			
TCGA-GL-A9DE-11A-11R-A37K-07	stage i	t1b	male			
TCGA-MH-A854-01A-11R-A355-07	stage i	t1b	female			
TCGA-A4-A57E-11A-11R-A26U-07	stage iv	t2a	male			
TCGA-MH-A857-01A-11R-A355-07	stage i	t1a	male			
TCGA-B1-5398-01A-02R-1592-07	stage iii	t3b	male			
TCGA-A4-A5Y1-01A-11R-A28H-07	stage iii	t1b	male			
TCGA-WN-AB4C-01A-11R-A42S-07	stage i	t1a	female			
TCGA-2Z-A9JQ-01A-11R-A42S-07	stage i	t1a	male			
TCGA-GL-A59R-11A-11R-A26U-07	stage iii	t3c	male			
TCGA-MH-A561-01A-11R-A26U-07	stage i	t1a	male			
TCGA-G7-6789-01A-11R-1965-07	stage iv	t3a	female			
TCGA-BQ-5882-11A-01R-1592-07	stage iii	t3b	male			
TCGA-DZ-6134-11A-01R-1965-07	stage i	t1a	male			
TCGA-BQ-5887-01A-11R-1965-07	stage iii	t3a	male			

TCGA-IA-A40X-01A-11R-A24Z-07	stage i	t1a	female		
TCGA-BQ-7051-11A-02R-1965-07	stage ii	t2	male		
TCGA-BQ-5888-01A-11R-1592-07	stage i	t1a	female		
TCGA-IA-A83V-01A-11R-A355-07	stage i	t1b	male		
TCGA-DW-7840-01A-11R-A32Z-07	stage i	t1b	male		
TCGA-Q2-A5QZ-01A-11R-A28H-07	stage iii	t3a	female		
TCGA-P4-A5E8-11A-12R-A28H-07	stage iii	t2a	male		
TCGA-BQ-5887-11A-01R-1965-07	stage iii	t3a	male		
TCGA-BQ-7059-11A-01R-1965-07	stage i	t1b	male		
TCGA-KV-A74V-01A-11R-A33Z-07	stage i	t1a	male		
TCGA-2Z-A9JT-01A-11R-A42S-07	stage i	t1a	male		
TCGA-B1-A654-01A-11R-A31O-07	stage i	t1a	female		
TCGA-BQ-5894-11A-01R-1592-07	stage iv	t3b	male		
TCGA-BQ-5884-11A-01R-1592-07	stage i	t1a	female		
TCGA-UZ-A9PM-01A-21R-A38C-07	stage ii	t2	male		
TCGA-IZ-A6M8-01A-11R-A31O-07	stage i	t1a	male		
TCGA-G7-6795-01A-11R-1965-07	stage i	t1a	male		
TCGA-B9-A69E-01A-11R-A31O-07	stage iii	t3a	male		
TCGA-MH-A562-01A-11R-A26U-07	stage i	t1a	male		
TCGA-B1-A656-01A-11R-A31O-07	stage i	t1a	male		
TCGA-GL-6846-11A-01R-1965-07	stage iv	t4	male		
TCGA-A4-A6HP-01A-11R-A31O-07	stage i	t1a	male		
TCGA-P4-AAVL-01A-11R-A42S-07	stage iii	t3b	male		
TCGA-G7-6796-01A-11R-1965-07	stage i	t1a	male		
TCGA-BQ-7056-01A-11R-1965-07	stage iii	t3b	female		
TCGA-Y8-A8S1-01A-11R-A37K-07	stage i	t1a	male		
TCGA-GL-7966-01A-11R-2204-07	stage iii	t3	female		
TCGA-J7-8537-01A-11R-2404-07	stage iii	t3	female		
TCGA-G7-A8LD-01A-11R-A36F-07	stage iii	t3a	male	KPNB1	>75 Percentile

TCGA-A4-7915-01A-11R-2204-07	stage ii	t2b	female		
TCGA-2Z-A9JI-01A-11R-A42S-07	stage iii	t3a	male		
TCGA-BQ-5875-01A-11R-1592-07	stage iii	t3a	female		
TCGA-G7-6789-01A-11R-1965-07	stage iv	t3a	female		
TCGA-BQ-5891-01A-11R-1592-07	stage iii	t3b	female		
TCGA-4A-A93X-01A-11R-A37K-07	stage iv	t3a	male		
TCGA-BQ-5893-01A-11R-1592-07	stage iv	t3a	male		
TCGA-BQ-5876-01A-11R-1592-07	stage i	t1a	male		
TCGA-AL-3466-01A-02R-1351-07	stage iv	t3b	male		
TCGA-BQ-5894-01A-11R-1592-07	stage iv	t3b	male		
TCGA-A4-A5Y0-01A-11R-A31O-07	stage i	t1b	female		
TCGA-G7-7501-01A-11R-2204-07	stage iii	t3a	female		
TCGA-A4-8098-01A-11R-2404-07	stage i	t1a	male		
TCGA-IA-A40Y-01A-11R-A24Z-07	stage iii	t3a	female		
TCGA-BQ-5890-01A-11R-1592-07	stage iii	t3a	male		
TCGA-B1-A655-01A-11R-A31O-07	stage i	t1a	female		
TCGA-BQ-7061-01A-11R-1965-07	stage i	t1b	female		
TCGA-G7-6793-01A-11R-1965-07	stage iv	t3a	female		
TCGA-HE-A5NF-01A-11R-A26U-07	stage i	t1a	male		
TCGA-BQ-5892-01A-11R-1592-07	stage i	t1b	male		
TCGA-A4-A57E-01A-11R-A26U-07	stage iv	t2a	male		
TCGA-P4-A5E8-01A-11R-A28H-07	stage iii	t2a	male		
TCGA-F9-A4JJ-01A-11R-A24Z-07	stage iii	t3a	female		
TCGA-BQ-7051-01A-12R-1965-07	stage ii	t2	male		
TCGA-BQ-5879-01A-11R-1592-07	stage iii	t3b	female		
TCGA-BQ-5877-01A-11R-1592-07	stage iv	t3a	male		
TCGA-HE-7130-01A-11R-1965-07	stage iii	t3	female		
TCGA-BQ-5887-01A-11R-1965-07	stage iii	t3a	male		
TCGA-B3-3925-01A-02R-1351-07	stage iii	t3a	male		

TCGA-B9-A5W9-01A-11R-A28H-07	stage i	t1b	male		
TCGA-BQ-5882-01A-11R-1592-07	stage iii	t3b	male		
TCGA-G7-6790-01A-11R-1965-07	stage i	t1a	male		
TCGA-A4-8310-01A-11R-2404-07	stage iii	t3a	male		
TCGA-BQ-7053-01A-11R-1965-07	stage iii	t3	female		
TCGA-BQ-7058-01A-11R-1965-07	stage iii	t3	male		
TCGA-A4-7287-01A-11R-2139-07	stage i	t1	female		
TCGA-B3-8121-01A-21R-2404-07	stage i	t1	female		
TCGA-F9-A97G-01A-11R-A38C-07	stage iii	t3	male		
TCGA-A4-8518-01A-11R-2404-07	stage i	t1a	male		
TCGA-5P-A9JU-01A-11R-A42S-07	stage iii	t3a	male		
TCGA-BQ-5884-01A-11R-1592-07	stage i	t1a	female		
TCGA-DZ-6131-01A-11R-1965-07	stage iii	t3a	male		
TCGA-A4-7996-01A-11R-2204-07	stage i	t1a	female		
TCGA-GL-A59T-01A-21R-A28H-07	stage i	t1b	male		
TCGA-2Z-A9J8-01A-11R-A42S-07	stage i	t1b	male		
TCGA-GL-6846-01A-11R-1965-07	stage iv	t4	male		
TCGA-DZ-6134-01A-11R-1965-07	stage i	t1a	male		
TCGA-A4-7734-01A-11R-A32Z-07	stage i	t1a	female		
TCGA-B3-4104-01A-02R-1351-07	stage ii	t2	male		
TCGA-B1-A47O-01A-11R-A24Z-07	stage i	t1b	female		
TCGA-BQ-7046-01A-11R-1965-07	stage i	t1a	male		
TCGA-GL-A9DC-01A-11R-A37K-07	stage i	t1b	female		
TCGA-B9-A8YI-01A-21R-A37K-07	stage i	t1b	male		
TCGA-Q2-A5QZ-01A-11R-A28H-07	stage iii	t3a	female		
TCGA-IZ-8195-01A-31R-2404-07	stage ii	t2a	male		
TCGA-DZ-6132-01A-11R-1965-07	stage i	t1a	male		
TCGA-V9-A7HT-01A-11R-A33Z-07	stage ii	t2	male		
TCGA-B3-A6W5-01A-12R-A33Z-07	stage i	t1	male		

TCGA-SX-A7SM-01A-11R-A355-07	stage iv	t3a	male		
TCGA-2Z-A9JM-01A-12R-A42S-07	stage i	t1a	male		
TCGA-UZ-A9PO-01A-11R-A38C-07	stage i	t1a	male		
TCGA-A4-7585-01A-11R-2139-07	stage iii	t3a	male		
TCGA-SX-A71V-01A-11R-A33Z-07	stage i	t1	male		
TCGA-IZ-8196-01A-11R-2404-07	stage i	t1a	male		
TCGA-EV-5901-01A-11R-1592-07	stage i	t1	female		
TCGA-A4-7584-01A-11R-2139-07	stage i	t1a	male		
TCGA-GL-A4EM-01A-11R-A24Z-07	stage i	t1b	male		
TCGA-GL-A9DE-01A-11R-A37K-07	stage i	t1b	male		
TCGA-A4-A7UZ-01A-12R-A355-07	stage iii	t3a	male		
TCGA-Y8-A894-01A-11R-A36F-07	stage i	t1a	female		
TCGA-SX-A7SU-01A-11R-A36F-07	stage i	t1	male		
TCGA-G7-7502-01A-11R-2204-07	stage i	t1b	male		
TCGA-BQ-7059-11A-01R-1965-07	stage i	t1b	male		
TCGA-GL-7773-01A-11R-A32Z-07	stage i	t1a	male		
TCGA-BQ-5888-11A-01R-1592-07	stage i	t1a	female		
TCGA-A4-8312-01A-11R-2404-07	stage i	t1	male		
TCGA-MH-A856-01A-11R-A355-07	stage i	t1a	male		
TCGA-A4-A4ZT-11A-11R-A26U-07	stage i	t1b	female		
TCGA-2Z-A9J3-01A-12R-A38C-07	stage ii	t2	male		
TCGA-B9-A5W8-01A-11R-A28H-07	stage ii	t2b	male		
TCGA-HE-A5NL-01A-11R-A26U-07	stage i	t1a	male		
TCGA-GL-A59R-11A-11R-A26U-07	stage iii	t3c	male		
TCGA-2Z-A9JL-01A-11R-A42S-07	stage i	t1a	male		
TCGA-Y8-A897-01A-11R-A36F-07	stage i	t1a	female		
TCGA-BQ-7061-11A-01R-1965-07	stage i	t1b	female		
TCGA-Y8-A898-01A-11R-A355-07	stage i	t1a	male		
TCGA-B1-A47M-01A-11R-A24Z-07	stage iii	t3a	male		<25 Percentile

TCGA-G7-A8LC-01A-11R-A36F-07	stage i	t1a	male			
TCGA-B9-4115-01A-01R-1193-07	stage i	t1a	male			
TCGA-DW-7836-01A-11R-2139-07	stage i	t1a	male			
TCGA-2Z-A9J5-01A-21R-A38C-07	stage ii	t2	male			
TCGA-2Z-A9J1-01A-11R-A38C-07	stage i	t1a	male			
TCGA-IA-A83V-01A-11R-A355-07	stage i	t1b	male			
TCGA-DW-7840-01A-11R-A32Z-07	stage i	t1b	male			
TCGA-A4-A5DU-01A-11R-A28H-07	stage i	t1a	female			
TCGA-PJ-A5Z9-01A-11R-A28H-07	stage i	t1b	female			
TCGA-P4-A5E8-11A-12R-A28H-07	stage iii	t2a	male			
TCGA-AL-7173-01A-11R-2139-07	stage iv	t3	female			
TCGA-G7-6792-01A-21R-1965-07	stage i	t1b	male			
TCGA-A4-A57E-11A-11R-A26U-07	stage iv	t2a	male			
TCGA-IA-A40X-01A-11R-A24Z-07	stage i	t1a	female			
TCGA-BQ-5885-01A-11R-1592-07	stage iii	t3	male			
TCGA-IA-A83W-01A-11R-A355-07	stage i	t1	male			
TCGA-BQ-7044-11A-01R-1965-07	stage iii	t3a	male			
TCGA-BQ-5881-01A-11R-1592-07	stage i	t1a	male			
TCGA-B1-A656-01A-11R-A31O-07	stage i	t1a	male			
TCGA-A4-7828-01A-11R-2139-07	stage i	t1a	female			
TCGA-G7-A8LB-01A-11R-A36F-07	stage iv	t2a	male			
TCGA-B9-A44B-01A-11R-A24Z-07	stage iii	t3b	male			
TCGA-GL-8500-01A-11R-2404-07	stage i	t1b	male			
TCGA-AT-A5NU-01A-11R-A28H-07	stage i	t1a	male			
TCGA-B1-A654-01A-11R-A31O-07	stage i	t1a	female			
TCGA-SX-A7SL-01A-11R-A355-07	stage i	t1a	male			
TCGA-HE-A5NI-01A-11R-A26U-07	stage i	t1a	male			
TCGA-SX-A7SS-01A-11R-A36F-07	stage i	t1	male			
TCGA-G7-6796-01A-11R-1965-07	stage i	t1a	male			

TCGA-IZ-A6M8-01A-11R-A31O-07	stage i	t1a	male		
TCGA-MH-A562-01A-11R-A26U-07	stage i	t1a	male		
TCGA-P4-AAVL-01A-11R-A42S-07	stage iii	t3b	male		
TCGA-UZ-A9Q0-01A-12R-A42S-07	stage i	t1	male		
TCGA-A4-A48D-01A-11R-A24Z-07	stage i	t1b	male		
TCGA-F9-A7Q0-01A-11R-A36F-07	stage i	t1b	female		
TCGA-AL-3473-01A-01R-1193-07	stage ii	t2	male		
TCGA-MH-A55Z-01A-11R-A26U-07	stage i	t1b	male		
TCGA-2Z-A9JT-01A-11R-A42S-07	stage i	t1a	male		
TCGA-UZ-A9PL-01A-11R-A38C-07	stage ii	t2	male		
TCGA-P4-A5E6-01A-11R-A28H-07	stage iii	t1b	male		
TCGA-KV-A6GE-01A-11R-A31O-07	stage i	t1a	male		
TCGA-Y8-A8S1-01A-11R-A37K-07	stage i	t1a	male		
TCGA-SX-A71R-01A-12R-A33Z-07	stage i	t1a	male		
TCGA-A4-A6HP-01A-11R-A31O-07	stage i	t1a	male		
TCGA-F9-A7VF-01A-11R-A33Z-07	stage i	t1a	female		
TCGA-DW-5560-01A-01R-1592-07	stage i	t1b	female		
TCGA-2Z-A9JQ-01A-11R-A42S-07	stage i	t1a	male		
TCGA-G7-6795-01A-11R-1965-07	stage i	t1a	male		
TCGA-B1-A47N-01A-11R-A24Z-07	stage i	t1a	male		
TCGA-IZ-A6M9-01A-11R-A31O-07	stage i	t1a	male		
TCGA-MH-A854-01A-11R-A355-07	stage i	t1b	female		
TCGA-P4-A5ED-01A-11R-A28H-07	stage i	t1a	male		

Table S3: A) Percentage loss and gain in transformation from normal to stages (I-IV), [KICH= Kidney chromophobe , KIRP= Kidney renal papillary cell carcinoma]

	N/S1	S1/S2	S2/S3	S3/S4
**KICH**	85.80 % loss from normal<br>74.52 % gain in S1	74.87 % loss from S1<br>77.61 % gain in S2	89.83 % loss from S2<br>83.37 % gain in S3	97.25 % loss from S3<br>92.54 % gain in S4
**KIRP**	97.82 % loss from normal<br>87.70 % gain in S1	76.87 % loss from S1<br>91.25 % gain in S2	87.47 % loss from S2<br>87.45 % gain in S3	55.40 % loss from S3<br>85.07 % gain in S4

B) List of lost, conserved and acquired genes across stages in KIPAN, [KIPAN= Pan-kidney cohort (KICH+KIRC+KIRP), KIRC=Kidney Renal Clear Cell Carcinoma, KICH= Kidney chromophobe , KIRP= Kidney renal papillary cell carcinoma]

**Lost from normal**	CEBPB	TUBA1A	SERPINH1	FSTL3	TMEM217	ZSWIM4	GDF5	HAPLN3	CXCL1	CDC42EP1	CITED4
	SPHK1	TM4SF4	COL8A1	COL16A1	IL6	ARL4C	TUBB3	PLAUR	COL1A1	IGFN1	SPSB1
	NLRP3	FJX1	MEX3D	SPON2	COL5A1	KRT86	ICAM4	PDLIM1	C2orf56	PLEKHO2	WNT2B
	ANGPTL7	PDLIM7	CCIN	CSF1	THBS2	TNFRSF1A	COL1A2	CMTM3	C3orf36	NUMBL	CXCL6
	SFRP4	SELE	SYNC	ASCL1	ELN	BAI2	COL11A1	MMP14	EMILIN2	PCOLCE	PODNL1
	FLJ16779	FSCN1	ZYX	HS3ST1	AKAP12	CXCL3	TIMP1	UGCG	TRH	IFFO2	KRTAP1-1
	C6orf174	CCDC80	GPRIN1	RCN3	DLGAP1	HK3	MDK	PRIC285	TYMP	TNFSF9	HCN1
	GBP1	PTPN1	WIF1	RRAD	ADAMTS2	MAP7D1	BCL2A1	FNDC1	ATP1A3	TWIST2	COL3A1
	LRRC59	HAS1	CD276	COMP	GLIPR2	DLGAP4	C1QTNF6	CCL4	SYCE1L	PVR	MFAP5

	MAGED4B	RELT	TNFAIP2	C1R	IFITM3	PLEKHA4	LOH3CR2A	RHOG	GHRH	HTN3	KRTAP9-9
	LOC255025	TRIM43	TRIM48	PLA2G2A	KLF16	B4GALT5	EMP3	TGFB3	PXDN	NINJ2	SAMD4A
	OSCAR	C5AR1	LOXL1	RND3	MAGED4	C1orf38	KLK8	C8orf71	ANXA1	KCNA6	MEGF6
	CCKBR	RARA	SCARF2	LOC100126784	CXCL16	COL6A2	CNTNAP1	HNRNPCL1	LILRA6	TPM1	PTX3
	EXT1	CD177	MAP7D3	TFE3	CFP	ANKRD1	RBM24	FPR1	IL4R	DNAJB11	COL5A2
	TWIST1	PLEKHO1	TSKS	BMP1	LILRB2	HLA-DQA2	OSR1	TNXB	FSTL1	PLD2	TAC1
	IL18BP	SOX9	TMPRSS11D	LRRC25	AEN	MTA2	OLFML2B	CYP21A2	PPP1R2P9	HRH1	KIAA0802
	MEX3A	FUT4	COL4A2	CADM3	GALNT5	S100A16	PODN	RAP2B	CHRM1	ODF3B	SCG2
	SLCO5A1	C1S	RRS1	C1QTNF1	ARSI	SERPINB9	CABYR	CDH22	DMRT1	TGFB1I1	MDFI
	MICAL2	MMP11	TREM1	FHL3	SBNO2	LY6H	PLSCR3	CREB5	LGALS9	GRAMD1A	CDV3
	RGS1	TP53BP2	NID2	NPW	TAGLN2	CLCF1	ASPHD1	SCXB	MAP3K8	MLKL	TICAM1
	FGFR1	GATA6	SCGN	SERPINF1	NFKBIE	AEBP1	RIN1	KDELC1	ARL 9.00	RXFP2	UPP1
	MAMLD1	DARC	PRR7	GDPD5	IRF8	RIPK2	MKX	UBE2S	MAP3K12	BTBD19	NKAIN4
	GPR84	IER5	SDS	TUBB6	SULT1C4	C1orf198	ADAM19	UBE2I	MMP19	IFI16	GPR37
	PROCR	SH2D5	CAMK2N2	OBFC2A	RHOU	STAC3	PRAM1	NFKB2	CD74	SLC38A8	IRF7
	CTXN1	LCP2	FGR	TNFAIP8L3	VCAM1	CLEC12A	EFHD2	TAP1	SLC35C1	CRP	ADAMTSL4
	ADAMTS12	SFRP2	GPC4	LRRN1	MARCKSL1	SEMA7A	TLE1	FCAR	ZNF365	GZMB	WISP1
	DAPK3	MEIS3	MMP2	NFIX	OXTR	RASSF2	NFKBIZ	C11orf9	HSPB8	FADS3	TMEM173
	CNKSR2	GNAI2	CD300LB	NNMT	PDGFRL	KRT80	ARHGAP17	GBP2	KCNT2	IL1R1	THBS4
	OR2B11	NGFR	AHNAK2	CSNK1E	CD72	FCN1	TMSB10	LRRN4	RHBDF2	TNFRSF1B	TWF2
	SIGLEC1	GNL2	PIM1	RBM46	CD300LF	HLA-DRB6	C1QA	IGF1	PML	LGALS9C	C17orf60
	ANKRD13B	RASA3	TTLL7	CNIH2	CPA4	SAMD14	DTX3L	CARS	PRICKLE1	HEYL	RAB32
	KIF3C	MATN3	SHISA5	S1PR2	CXCR2P1	CD248	TRPV2	NHEDC2	MED17	ABCB4	SOD2
	PAQR4	SGK223	TNFRSF10B	NT5DC3	UNC5A	ISLR	PTRH2	LOC606724	ZNF469	C8orf84	MYOZ3
	SERPING1	GUCY1A2	PLB1	C1orf127	IFITM2	EFEMP2	ZDHHC18	SACS	PRR24	PLAC8	HNRNPAB
	CNN2	ATP8B2	VASP	KRT33B	NLGN2	C11orf24	NRBP1	TPRG1	RFTN1	CEBPD	BATF
	TYROBP	DOK2	CKAP4	TNFSF13B	C13orf18	PLEK	LOC100130776	PRKCDBP	CFB	GNA15	ODF3L1
	KIAA1045	CLEC11A	CD300C	MGP	SLC27A3	WAS	YWHAH	CCL18	ITGAX	ROPN1L	LEPRE1

	DNM1	COL4A1	S100A3	PHF19	IRAK2	CD300A	LOXL3	TMEM43	CCL4L2	CLIC1	LIMD2
	MN1	GRASP	LSP1	TSPAN18	NFAM1	INHBA	CCDC109B	LILRB4	CD86	MMP17	GJA1
	AKIRIN2	ADAM12	VSTM1	LILRB3	CSDA	HCK	CTSZ	FERMT3	CRYGD	RASSF5	SPI1
	PRRX2	CD80	MYO1F	CX3CL1	SFRS13B	NR0B1	PYCR1	FCGR1C	IFNAR2	LGALS1	PVRL2
	ARF6	SH3BGRL3	IFNGR2	RAB35	C2orf89	IFITM1	KRT8	KIF7	CTGF	SLC15A3	HCLS1
	HOMER3	MT1A	ITK	LILRA4	PCDHB14	RIMS4	NCF2	C1orf162	RAC2	HLA-DPB1	MXRA8
	BEAN	GLIPR1	C13orf36	LDLRAD3	CILP2	C12orf68	PIWIL4	C1QB	IL4I1	PDIA4	HCST
	DPPA2	C20orf103	DOK1	NAV3	FBLIM1	ARMC4	LTBP2	BCL3	LST1	CDKN3	RETN
	PLTP	ULBP2	CFH	KLF2	BBC3	MAN2B1	CSF3R	NAMPT	PTCHD2	ADAMTS3	CDH6
	GPR97	PRRX1	CEACAM21	PIGR	TUBA1B	SELPLG	GPR171	IGFBP6	EPSTI1	PHF17	SDC3
	PSTPIP2	MSC	DDX5	MT2A	GMIP	GLI1	PARVG	FMNL1	MEX3C	C1QTNF2	GDF1
	MYD88	MTHFD2	F12	LRAT	CENPW	CORO7	PTRF	MANF	GMFG	MAP1LC3C	FEZ1
	MICAL1	CCRL2	NLRP12	FBN1	C1RL	FCGR1A	MEGF11	MESP2	HTR3A	TUBA1C	FCER1G
	SH3PXD2B	CDH23	ALDH1L2	NLRC5	UTF1	COL6A1	BMPER	CD69	FBLN1	CD40LG	INMT
	DEGS1	DACT1	PYCARD	ISG20	SERPINA3	FGF21	PAPLN	LILRA2	CD14	CASP4	SLC2A10
	FCGR1B	HKDC1	TNFSF8	SRPX	DPYSL4	MX2	VIPR2	BASP1	PTP4A3	ZMYND15	CCDC50
	CD44	CTTNBP2NL	CCL3	SFN	AMICA1	ITGB3	MATN4	CD300E	EMILIN1	KCTD4	DTX2
	INPP1	NOP2	PPFIBP1	IL7R	SERPINE2	C1QC	DKK 2.00	PTCRA	AGFG1	LBR	YWHAG
	DCLK1	KCNQ3	GPR56	TMEM233	KRT18	HLA-DOA	CRISPLD2	MAPK7	KCNK6	ZFPM2	SIGLEC9
	HABP2	FKBP1A	MMP23B	CD151	RASAL3	LILRA5	PSG1	DUSP10	SHF	EPB41L2	OLFM4
	WDR69	FZD1	CD3E	IRF9	BAX	LOC148145	CDH24	CSF2RA	NOP56	CD63	FPR2
	SLA	C10orf26	TIMP2	PTGIR	RELA	RGS19	TBXAS1	FAM5B	EHBP1L1	TNFSF10	LILRB1
	CST7	PTCH2	CSPG5	ADAMTS7	CNKSR3	TCF7	UTP14A	GLI2	NBL1	GAB2	HLA-B
	KRTAP2-1	PFN1	COL18A1	THBS3	PMP22	MFGE8	GPSM3	GRK6	MC1R	HIVEP3	TMEM119
	CFL1	JAK3	DPEP2	CDCA4	MMP16	PSTPIP1	SIX3	FGD2	ANTXR1	SCGB1D4	ARHGAP15
	DYRK2	HAVCR1	HTRA3	RRAS	C19orf38	CFTR	SRM	MIDN	MFRP	ALOX5	STXBP1
	KLF7	SLIT3	FAM83G	RHOH	RSPO1	FAM180B	CD6	LRFN5	ELF4	DUSP8	PTGFRN
	HNRNPA0	MYBL1	ADCY7	HLA-DQB2	CD5	HM13	C3AR1	ITGBL1	C10orf105	NCF1	CDON

	LRRC8E	SHANK1	MEF2D	ADAM8	FUT7	SMOX	GBGT1	NMT2	PSMB9	NRP2	HSPA5
	PALLD	LAIR1	PIK3R5	HLA-E	UBAP1	CNR1	GFPT2	TREML3	NKIRAS2	CHPF2	STS
	C19orf22	GOLGA7B	SDC4	LY96	LRP1	TEAD3	LIMK1	TXLNB	TTC39C	LAT2	TNFRSF9
	PMS2L11	LRRC41	LAPTM5	C13orf39	ITGB2	ARHGAP30	EFEMP1	TRAT1	DDAH2	LEPREL2	ARL 14.00
	IFI35	TLR2	TNFRSF12A	KRT23	CASP1	KRT75	APBB1IP	OMD	CCL5	SEL1L3	ICOS
	RAB31	CYP2C9	CD28	C12orf70	LRIT2	KRT33A	DPYSL3	NCF4	WNT6	RGS4	KIAA1274
	HLA-DMA	KCTD5	SLC24A4	NPDC1	ALOX5AP	IL21R	ENAH	CLDN1	APOBEC3F	NAPSB	GPR172A
	LY86	PARP9	RELL1	MIR155HG	SEC24D	LYZ	CD4	CD7	HSP90B1	TRIM38	ACTN4
	AOAH	CD200	SH2D2A	BIRC3	POU2F2	RIN3	RASSF1	RCVRN	EDN2	HUS1B	TPM3
	RBP1	CDKN2A	ETV6	GZMM	COL15A1	JUND	CORO1A	LILRA1	IL10RA	PMM2	MYO9B
	FAS	CSF2RB	IL32	TRPM2	CARHSP1	BTK	TSC22D2	LSR	C14orf80	KCTD10	CMTM7
	IRF1	GTF2E2	RPIA	TP53	DTNA	CHI3L2	FCGR2A	FAM109A	TEAD4	MUC12	WNT5B
	TRIM21	OSMR	CCDC46	GRIA1	HP	ELK3	MYO1G	AXL	PRSS23	KLHL29	SERPINB1
	NUDT11	SMAP2	PCDHGC3	PSMB10	DNAJC2	PLAC9	LOC100233209	GGT5	LPGAT1	CD101	SSTR4
	TNNT3	FHL1	TIGIT	CAMSAP1	PPIB	ARRB2	KPNA2	ADPRHL2	LCP1	SYT11	MyD99
	PKDCC	EPN2	SULF1	SLAMF8	SORBS2	ITGAL	HERPUD2	SIPA1L2	GLDN	GNL3	KCTD11
	WISP2	CYP19A1	LIX1L	FFAR2	HIVEP1	RRP12	TMEM39B	FKBP10	HLA-A	CCNK	VAV1
	MYH9	IMPDH2	SAMHD1	ANXA2	BEST1	RARRES3	NLGN4X	C5orf58	HAS2	CNGB1	PCDHGA5
	CD3D	NAALADL1	GLT25D1	FBXL12	KAAG1	C22orf9	LEFTY2	GRID1	DAPL1	ARNTL2	TES
	CTLA4	SLAMF1	WIPF1	RGL4	GXYLT2	CHSY3	PATE4	TSHZ3	SLAMF6	CBFB	ARL8A
	ARL4A	ARHGDIA	CACNB3	MMP25	FAM26F	BMP15	FAM64A	SETD8	IL1B	TRIM47	CD96
	CA5BP	CXCL11	NME1-NME2	PRC1	UCN2	DEF6	B3GNT7	HAMP	PYHIN1	CDHR1	DOCK2
	VEGFC	PHC2	GAB3	GTPBP2	MED26	EFCAB4B	PRG4	BEND6	KIAA1199	FPR3	CSTA
	CSF3	EEPD1	SMURF1	AGAP2	TRANK1	LOC100270710	WASF1	S1PR4	LYPD1	CRYBB1	ADCY2
	SVIL	NECAP2	SOX4	ALPK2	IL12RB1	CEACAM3	FBLN7	HAVCR2	RAB42	CDKN2BAS	STAB1
	NMNAT2	PLA2G7	YPEL4	TGFB2	SH3RF3	MGC87042	EPHA7	IFNGR1	C10orf54		
**Conserved in normal and S1**	NFIL3	ZFP36	CCRN4L	CYR61	BYSL	MYADM	ADAMTS4	JUNB	MCL1	FOSB	KLF10
	C6orf145	ITPRIP	PPP1R15B	NOLC1	DDX21	POLR3D	PPRC1	DCUN1D3	CSRNP1	KLF6	EIF4A1

	LYAR	WDR43	DUSP1	DNAJB5	SOX7	SLC2A14	ISG20L2	SRF	JOSD1	JDP2	RHOB
	SLC2A3	XIRP1	F2RL3	SERTAD1	SNAI1	SOCS3	CCL2	THBS1	C9orf21	ETS2	TNFAIP3
	EMP1	ERF	FOSL1	PNRC1	B3GNT5	LATS2	APOLD1	GTPBP4	CSGALNACT2	KDM6B	NDEL1
	SEMA4C	GEM	URB2	SPRY1	DDX3X	WTAP	ARID5A	ETS1	TNFRSF10A	PLK2	PDGFB
	AG2	PPP1R15A	RNF19B	HBEGF	ICAM1	SEC23A	SERPINE1	ITGA5	DENND5A	NFKBIA	SH3BP5
	TAF5L	SPRY4	TAL1	SRGN	PLK3	DLC1	RNF122	BAZ1A	MAFF	RAI14	SERPINB8
	MESDC1	AREG	KLF4	CHSY1	PLEKHG2	ASAP1	LRRC8A	PTPRE	NFKB1	SLC25A32	LMNA
	PELO	SH2B3	TRA2B	ABL2	FOXC2	MSN	CALD1	GRK5	LIF	JMJD6	POLR2D
	BMP2	SPRY2	SLFN11	PHLDA1	DGKD	PYGL	TEX10	RBMXL1	GNA13	KCTD20	FRMD8
	EPHA2	LMCD1	NRAS	UCK2	NEDD9	PLEKHG1	MKI67IP	NID1	RIPK1	DUSP14	PEA15
	FILIP1L	PHF13	PANX1	INHBB	KIAA1949	HIVEP2	CWC22	CORO1C	AMMECR1L	FOSL2	THBD
	CD97	PDE4B	DYRK3	ACTN1	SLC10A6	PITPNB	C13orf33	ITPKC	SMTN	SHC1	ADAM17
	SH2D3C	CXorf36	NRP1	KIAA0020	CD93	KBTBD2	MAP3K14	LRRC32	PTPN12	TMEM2	NFATC1
	HIC1	PRR16	APOBEC3A	CACNA1C	STEAP4	RBMS1	BATF3	GNB1	GPR176	STAT3	C6orf150
	CNN3	CLEC1A	C10orf10	CHD1	FAM102B	CSPG4	ROBO4	AFAP1	RELB	SAP30	STK10
	GRRP1	FAM70B	STX11	PRDM1	MAP4K4	RAPGEF5	VIM	ZNF267	SDPR	NFATC2	PER2
	MCAM	TBCCD1	PDGFRB	BTN2A1	GNAI3	SPRED2	C3orf59	TNFRSF4	GIMAP5	FAM110C	PIP4K2A
	CDC42SE1	ARID5B	ADAMTS1	C9orf25	POLR1E	LHFP	SPARC	PVT1	GPR183	CHIC2	PAK1IP1
	ZNF496	LZTS1	RNF19A	SYNCRIP	BCL9L	COL6A3	SGK1	BHLHE40	NR4A3	ELL2	FCHSD2
	FLNA	NUP62	SYDE1	CEP170	RNF145	EBF1	HEPH	TRPC4	CXCL2	JAM3	RALGDS
	RBM7	TMEM22	ACBD3	RSU1	ADA						
	RIOK1	AATK	JUN	STC1	GPR4	C1orf183	TNFRSF10D	C1orf107	RGL1	BCL6B	PELI1
	ETF1	KCNE4	FOS	PFKFB3	C10orf108	SPG20	MAT2A	ADM	PPP2R2A	ABCE1	NUP153
	SEH1L	ACLY	SHANK3	CFLAR	YRDC	C10orf119	EIF2S1	S1PR1	NCL	HBB	STARD13
**Acquired in S1**	DNTTIP2	KCTD15	TAF4B	NEDD4	NUPL1	LRRC70	DLL4	EGLN3	CDK17	ZNF295	CXCR7
	SLC35F1	CA9	SERTAD2	PCBP1	C9orf30	C1orf55	UBC	NOP14	RBM8A	NOTCH4	ATF3
	LDB2	ANGPTL4	P2RY8	EPAS1	VEGFA	STC2	AK3L1	HNRNPK	EGR3	FAM38A	PDLIM2
	ELOVL5	ANKRD40	COL5A3	TSR1	BNIP3L	CD36	EIF2C2	CDKN2B	ENPP3	DDX50	RLF

	NOS3	COL25A1	FLT1	PLXND1	RALB	BARX2	KCNAB1	HSPA14	CNST	S1PR3	HDX
	C15orf39	ARRDC3	EPC1	QSOX2	ZCCHC2	PRPS1	P4HA1	RBM12	SLC16A1	EIF3J	SCARF1
	USP13	GEMIN5	PRPF4	WAC	KPNB1	DPYSL2	KCNK3	TCP1	FLT4	DNAJA1	NONO
	HTR1F	TNFAIP8L1	TIE1	YME1L1	C7orf68	SKIL	ADCY4	RSL1D1	HNRNPU	RPS6KA5	RBM15
	RRAGA	CD34	GDI2	CDH5	CDK18	NAA15	GJA4	DOCK6	WWTR1	MPI	HYAL2
	ERG	SEC14L1	EHBP1	PNO1	KCNE3	APLN	GJC1	SHROOM4	SIK1	DCLRE1B	NOVA2
	SLC15A4	NDRG1	PITPNC1	ZNF395	F2R	SFRS3	PLEKHA2	LOC154761	SCARB1	SHMT2	OPTN
	PGM3	FAM115C	FAM57A	PLVAP	PFKP	ARAP3	MAPT	PEAR1	PLXNA2	TYMS	PTPRM
	TMEM194B	ZNF281	BTG1	SFRS7	NUP98	TMIGD1	KLF9	FYN	RHOJ	C13orf15	GPR3
	DYSF	PGK 1.00	DAXX	C22orf45	ELTD1	SGCB	TMEM66	KLHL20	MSH6	PPAP2B	EGR1
	UBA2	CCDC102B	CALCOCO2	FDPS	TIMP3	ERRFI1	ENPEP	PRPF38A	RASGRP3	SOX17	SIRT1
	NAF1	INTS6	MMRN2	AFARP1	RAP2A	KIAA0146	IL6R	PWP2	FRMD3	GEMIN4	PCDH12
	HNRNPA3	NETO2	LONRF3	ZBTB2	HAT1	SWAP70	MYCT1	ARHGAP42	ADRA1B	GPR135	ALKBH5
	ESAM	HEATR1	CYB5A	S100A12	FAM43A	NUAK2	BNIP3	EGOT	FHL5	PAIP2	PRMT5
	ZEB1	ETV3	SLC25A43	DEDD	PABPC1	TCF4	COL21A1	SASH1	UBE2J1	RERGL	EXOC3L
	WDR75	PGAM4	NOTCH3	ARHGEF7	BHMT2	PDK1	PTP4A1	CASP7	GPATCH4	SLC35D2	GART
	INSR	CAMK4	PPM1F	GALC	LEPROTL1	DAB2	ZEB2	HNRNPF	KHDRBS1	WDR3	MAPK12
	POLR3D	ERF	PPP1R15A	MAFF	AREG	C10orf108	ADM	LRRC8A	PPP2R2A	ABCE1	SLC25A32
	PELO	SEH1L	YRDC	EIF2S1	HBB	KCTD15	TAF4B	BMP2	DLL4	PHLDA1	CDK17
	ZNF295	SLC35F1	CA9	NOP14	LDB2	STC2	COL5A3	CD36	EIF2C2	CDKN2B	NOS3
	COL25A1	FLT1	BARX2	KCNAB1	RBM12	EIF3J	SCARF1	PRPF4	PITPNB	WAC	FLT4
	DNAJA1	HTR1F	SMTN	SH2D3C	RSL1D1	KIAA0020	RPS6KA5	NAA15	HIC1	DOCK6	HYAL2
**Lost from S1**	KCNE3	APLN	SIK1	NOVA2	CNN3	SCARB1	OPTN	PLVAP	MAPT	PLXNA2	TMIGD1
	KLF9	C13orf15	GPR3	PGK 1.00	ELTD1	SGCB	TMEM66	PPAP2B	UBA2	FDPS	TIMP3
	SIRT1	SDPR	NAF1	INTS6	PWP2	FRMD3	SPRED2	NETO2	TNFRSF4	MYCT1	C9orf25
	ALKBH5	ESAM	CYB5A	NUAK2	PVT1	PAIP2	PRMT5	PABPC1	COL21A1	BHLHE40	RERGL
	EXOC3L	PGAM4	ARHGEF7	BHMT2	GPATCH4	EBF1	INSR	LEPROTL1	JAM3	RBM7	WDR3
	ADA	MAPK12									

	ZFP36	SOCS3	MCL1	FOSL2	CYR61	JOSD1	NFIL3	ICAM1	THBS1	ADAMTS1	HBEGF
	STAT3	KLF6	ABL2	EIF4A1	PLK3	JUNB	EGR3	DNAJB5	FLNA	CORO1C	PFKFB3
	PYGL	PHF13	SGK1	C9orf30	MYADM	WTAP	MSN	NDEL1	SNAI1	SRGN	WWTR1
	DPYSL2	RHOB	CSGALNACT2	SERPINB8	SERTAD2	CXCL2	JUN	GPR135	RNF19B	AFAP1	CCL2
	DCLRE1B	DUSP1	DUSP14	KIAA1949	LATS2	ADAM17	NUPL1	S100A12	SLC15A4	ACTN1	ARID5B
	PPP1R15B	RIOK1	EMP1	SEC23A	ARID5A	CHSY1	DLC1	MAP4K4	ADAMTS4	SEMA4C	LMNA
	PRPF38A	POLR2D	SPRY1	NRP1	VIM	ZEB2	GPR183	KCTD20	ASAP1	AMMECR1L	MSH6
	GNA13	PTPRE	CFLAR	EPHA2	TNFAIP3	APOBEC3A	GNB1	KLF10	BAZ1A	JDP2	NR4A3
	FILIP1L	KLF4	CD97	BYSL	HIVEP2	STEAP4	ISG20L2	GNAI3	DCUN1D3	CEP170	HEATR1
	ETS1	STK10	RAI14	PDE4B	PER2	PELI1	PLXND1	P2RY8	SLFN11	FAM110C	C10orf10
	PIP4K2A	FOS	CAMK4	C6orf145	PRPS1	SRF	SLC2A14	PGM3	NCL	RLF	SFRS7
	SH2B3	C1orf107	TNFRSF10A	ETV3	STARD13	BCL9L	PPRC1	SLC2A3	UCK2	LMCD1	KPNB1
	SHC1	PLK2	RALB	MAT2A	WDR43	SLC25A43	AK3L1	CCRN4L	ZNF281	PANX1	DENND5A
**Conserved in S1 and S2**	FOSB	RBM8A	C6orf150	RGL1	ITPRIP	SH3BP5	TRPC4	TMEM194B	ARRDC3	SKIL	S1PR3
	C3orf59	C9orf21	LYAR	HNRNPA3	FAM38A	SHROOM4	DDX21	CASP7	GPR176	FAM102B	EGR1
	PCBP1	DYRK3	C10orf119	ACBD3	CALD1	TNFRSF10D	DDX3X	KCNK3	DAXX	AG2	LIF
	GTPBP4	TSR1	PDK1	TAF5L	EPC1	HNRNPU	RBMS1	NFKBIA	RIPK1	CHIC2	DEDD
	HSPA14	MESDC1	FCHSD2	PTP4A1	ELL2	JMJD6	ZNF267	STC1	NONO	ZNF496	C1orf183
	UBC	ETS2	NOLC1	SYDE1	GALC	B3GNT5	LRRC32	ACLY	MAP3K14	ITGA5	GPR4
	RSU1	SAP30	PLEKHA2	FOXC2	IL6R	BTN2A1	KDM6B	CCDC102B	WDR75	PNRC1	PLEKHG2
	TMEM2	URB2	C13orf33	SPRY4	SLC16A1	NRAS	HAT1	ZBTB2	NFATC1	EHBP1	SOX7
	FOSL1	CALCOCO2	FYN	F2R	CHD1	DAB2	APOLD1	GRK5	SPARC	GRRP1	RELB
	TRA2B	C15orf39	RNF19A	BCL6B	DDX50	HDX	YME1L1	TMEM22	EPAS1	LONRF3	RBMXL1
	PLEKHG1	TYMS	DYSF	SWAP70	MCAM	EGLN3	STX11	PDGFB	LZTS1	KHDRBS1	ZCCHC2
	SERTAD1	PRDM1	UBE2J1	KIAA0146	RNF145	CD93	FAM57A	PTPRM	ITPKC	PEA15	RAP2A
	C1orf55	KCNE4	GIMAP5	TBCCD1	BNIP3L	ENPEP	PDGFRB	NUP153	COL6A3	NID1	ERRFI1
	SEC14L1	KLHL20	POLR1E	PTPN12	CXCR7	GEMIN4	FAM70B	NUP62	TNFAIP8L1	PDLIM2	AFARP1
	RBM15	NFKB1	CDC42SE1	TAL1	ELOVL5	PEAR1	ZNF395	SPRY2	KBTBD2	C7orf68	SHMT2

	RHOJ	ANKRD40	HEPH	SFRS3	CSRNP1	ERG	NEDD9	ENPP3	PCDH12	XIRP1	BATF3
	RRAGA	VEGFA	NEDD4	RNF122	PPM1F	SERPINE1	SHANK3	NOTCH3	GEMIN5	PFKP	PNO1
	GJC1	ANGPTL4	FRMD8	DNTTIP2	PITPNC1	FAM43A	LHFP	GDI2	AATK	TEX10	CWC22
	ATF3	P4HA1	CSPG4	ROBO4	CNST	F2RL3	S1PR1	PRR16	TCF4	LOC154761	SLC10A6
	MPI	USP13	CACNA1C	GJA4	TCP1	RAPGEF5	PAK1IP1	RALGDS	GART	LRRC70	HNRNPK
	SASH1	CDK18	MMRN2	DGKD	ADCY4	ADRA1B	SYNCRIP	ETF1	ZEB1	SLC35D2	ARAP3
	NUP98	QSOX2	RASGRP3	GEM	ARHGAP42	SOX17	INHBB	MKI67IP	NDRG1	C22orf45	BNIP3
	NOTCH4	FAM115C	EGOT	NFATC2	HNRNPF	BTG1	CD34	FHL5	CLEC1A	TIE1	CXorf36
	CDH5	THBD	SPG20								
	BCL6	IL4R	RND3	IL8	KIRREL	F13A1	ZFP36L1	FAM49A	CD300E	FPR2	TMEM49
	PADI4	TNFSF18	CD209	MLKL	IFFO2	POLD3	S100A9	EHBP1L1	SMARCAL1	FPR1	EPB41L2
	AMPD2	SAP130	UTP6	OSMR	RASSF3	MYD88	C5AR1	PSMD11	PLEKHO2	RASSF2	WDR47
	MYOF	C1orf216	IFNAR2	TCP11L1	RAD51L3	LRRC59	SP110	PHLDB1	CAP1	MRC1	PLAUR
	NRP2	CEP170L	MPZL1	S100A8	ARNT	RNF217	EGFLAM	ADPRH	CRISPLD2	PPM1D	IL1B
	FHL2	KALRN	TPM3	MTERFD2	CHST11	CRLF3	PHACTR1	CTTNBP2NL	ZRANB3	MCTP1	TRAM2
	NFKBID	TUBA1A	COTL1	ATP8B2	ARL4C	GPATCH2	CASP10	PLEKHG5	RNASE2	IL2RA	C10orf54
	STAT4	TET3	DDIT4	GMEB1	CCDC46	TNFRSF1A	PIK3R1	USP37	RILPL2	CFH	HTR6
	BCL3	NR1H4	GON4L	FCGR2A	FTO	EIF2C1	LOC284441	PMP22	SFPQ	PIP5K1A	LILRB2
**Acquired in S2**	NFKB2	GNAI2	ELMO1	IRAK3	PRICKLE2	CASP5	CCDC88A	ODF2	LDLRAD3	TNFRSF1B	OSM
	LHX8	IL7R	TP53BP2	SLC2A1	LILRA5	CSF2RB	CSDAP1	SIRPB1	SUPT7L	CMIP	LIPN
	ABCB6	USP18	NLRP3	PDIA6	KCMF1	SERPINB9	CEBPD	SELL	EHD2	DYNC1I1	IFI16
	CCL4L2	TUBGCP3	CLEC4D	DISC1	PROS1	RGS9	BTBD19	TRIP12	MSL1	MTCP1	C1S
	PHC2	ARRB1	ATF7	BGN	AOAH	EMR1	FCGR3B	H6PD	TRIM9	ARPC2	IL10
	ENO1	SF3A3	S100PBP	WDFY2	EIF4A3	HCLS1	JUB	CLEC4C	ZNF643	C1R	SUPT6H
	SLC13A5	TTC27	TUBB	CACNA2D1	BHLHE41	WDR26	TAF12	SAMSN1	TRIM5	INVS	CBLB
	ALDH18A1	MMP14	A2M	ARHGAP29	VSTM1	NRBP1	SLC16A3	WEE1	C13orf18	AGTPBP1	DUSP12
	YEATS2	SMAP2	IPO9	ULBP2	OTUD3	ITGB1	CEACAM3	APOBEC3C	SH3PXD2B	ADAP2	MTF1
	SRGAP2	GLIPR1	LOC643837	CASP1	LYN	EXOSC10	HEATR5B	LCP2	UGGT1	LRRC42	ELK3

	ZC3H12A	LRP1	DNAJC10	CASP4	FBXO42	FPR3	CXorf21	GLIPR2	NOTCH2	SULT1B1	RPS6KC1
	YY1AP1	ZCCHC24	ATP2B4	PDIA5	CXCL3	MAP7D2	IPMK	IL13RA2	RNASE1	MKNK1	SLC11A1
	NNMT	GLT25D1	SFRS4	CLEC4A	WDFY1	PPT1	TEAD4	TTLL4	FAM55C	ARHGDIB	SIRPA
	TMCO7	SIRPB2	EMILIN2	CXCR2	COL12A1	KCTD11	CSF1R	NBPF10	MID1	PLEK	MALT1
	LRRC37B2	TFPI	FLJ36031	ANXA5	MYLC2	IRF2BP2	MS4A6E	TTC28	CCNYL1	CD86	RFX3
	GBP1	CLASP1	HK3	PCDHGC3	SAMHD1	PDLIM1	C5orf58	PBX2	LBR	FUT11	C2orf86
	SF3B1	CD53	ATP13A3	GPLD1	IRS4	MNDA	FGD5	MSRB3	TPM4	FOLR2	SFXN3
	ZHX2	KIF3C	EHD3	THRAP3	EML4	LINGO1	HNRNPH1	ADCY3	PPP1R3B	FSTL3	C19orf38
	STAB1	KRT75	MFSD11	TLR6	NECAP2	TTLL7	ENTPD7	SSR3	FADS1	BAT2L2	REL
	ADAM19	GPR161	CFHR1	MMP8	ZNF462	SLC6A5	VEZT	FCN1	FADS3	OAS3	CCNL1
	LCP1	TYMP	SP100	PUS7	SPEN	TTC13	AATF	NOS2	LIX1L	CCL8	BICD1
	CD33	SLC24A4	TRPV2	LILRB1	EMR2	TRNAU1AP	RAD51L1	AXL	EMR3	RIMKLA	ABCF1
	CD14	RASGRP2	TAF11	EIF2C3	SGK269	MYO1B	ZYX	MYLK	HELB	FAM20A	CSTT
	CD300C	UGDH	LEPRE1	KCTD3	ZNF597	TRAF1	ZNF830	S100A7	ELK4	LIPI	EIF2S3
	CASP8	SKI	THOC5	KLRG1	RFFL	CD226	KLHL33	HDGF	LAIR1	MAP1D	NES
	FAM119B	CEACAM4	SACS	DCBLD1	MPEG1	C3orf64	PREX1	CD69	GFRAL	DAAM2	TNKS1BP1
	KCNJ8	ACTR3	YBX1	PTRF	PDCD1LG2	MAP2K3	PLEKHM3	SLC30A1	MAF	MS4A6A	FAM129A
	WDR33	IFNGR2	NAMPT	BARD1	TRIM38	CD68	TMEM217	GUCY1A2	APLF	ZSWIM2	HNRNPC
	BMP2K	VSIG4	CCL7	C13orf29	CD200	DENND3	GPR84	PLXDC2	GIT2	ACTN3	NAV1
	BLMH	SLC43A3	CREM	FUT4	TRDMT1	SEL1L3	P2RX1	C6orf114	IL4I1	CXCR4	KIF2A
	IL1R1	BBS9	TNFSF11	HIP1	DHX9	LOC100134259	MT1X	SPSB1	IRAK2	KDM3A	AGAP1
	NLRC4	IL24	MYO1G	TNFAIP1	SLCO2B1	COL27A1	CD4	TTC4	USP31	HNRNPR	ITPRIPL1
	KIAA0090	SRBD1	FAM26E	CDH11	CHSY3	ST3GAL2	MDC 1.00	GPN1	PARP1	C1RL	KDM5B
	CAPZA1	PAG1	DHX57	SDCCAG8	SLC1A4	SIGLEC5	FHL3	ATF2	R3HDM1	CR1	RUNX2
	LOC283663	NIN	PGM2L1	RASGRP4	PRKCB	LOC100131551	WASF2	SMC6	DYNC1I2	DSCC1	STK17B
	LPGAT1	DDX5	IRF9	SLC25A37	LY6G6C	GTPBP2	FSTL1	EIF2B4	CLEC7A	NRIP1	ARL5B
	HMOX1	ADAMTS2	ENAH	TNFSF13B	CSDA	MBD5	RNF24	C12orf68	TMEM106A	JAG1	RPH3A
	AVEN	PLEKHM2	OLFML2B	TXNDC12	COL4A2	WIPF2	YWHAG	TTC21B	UBE2Z	CDC42EP3	PDE3A

	UBTD2	BMP1	NCKAP1L	GUCY1B3	INSIG2	SLC4A1AP	RFTN1	CD5	OSBPL11	RASAL2	PPP4R1L
	LOH3CR2A	LILRA1	RGS2	TARDBP	GPCPD1	CASC3	C3	GYPC	HCK	CDC42EP4	FZD5
	LOC282997	SIGLEC9	POLR3C	FKBP7	TLR1	MAP3K2	CXCR1	TGFB1	PCDHAC2	CEP135	IDH1
	APOL3	ZMYM4	VANGL1	TAF13	IER3	MS4A4A	FNDC3B	ARHGEF10	BCORL1	STAMBP	CDK12
	PDCD11	NUP85	ARHGAP22	RPS6KB1	CCL19	CDK2	LRRC33	SNRNP40	RNASE4	C14orf149	WDR35
	FHOD1	CHST15	RASSF5	KATNAL1	STK38	GYPE	ALS2	XPNPEP1	PPP3R1	RASAL3	LHFPL2
	BNIP2	TULP3	TMEM173	MAML2	IER5	SIGLEC10	VAMP3	NOL3	SOCS5	BCL2A1	MAP3K12
	CCR1	RCSD1	DOCK2	ZNF318	CHEK2	MTHFR	AIF1	ZNF207	FRMD4B	RPIA	AMDHD1
	CSDE1	EHD4	P4HA2	TTYH3	CYBB	PARP14	DHX8	SYNJ2	PDLIM3	SOD2	ARHGAP30
	XRCC5	PANK2	IL1RAP	RQCD1	HNRNPA3P1	KLF2	CD300LB	KRT32	C17orf107	C2orf48	SIGLEC12
	NBPF9	PNMA2	SAMD8	CD1D	CYP7A1	C3AR1	PML	GAS2L3	ETV6	NFYA	C6orf204
	RECQL	OGFRL1	SLIT3	SP140L	TTYH2	VCAM1	FBXL7	MCTP2	ARNTL2	ARHGEF2	KSR1
	KLF8	CEP164	TRIM34	SQRDL	ECD	TMEM67	CGREF1	ENTPD1	ZNF438	FAM38B	ERCC3
	CD163	SLFN5	POLR1B	AGFG1	NFAM1	HPS3	FKBP1A	P2RY6	FFAR3	NBPF14	CLEC2B
	TMEM218	MYO1F	ANTXR2	ARL13B	C1orf198	RPA1	TRIM6	FEZ1	CSF3R	UIMC1	DCHS1
	C2orf96	APAF1	RPGRIP1L	IL13	DKK 3.00	DLX5	ZNHIT6	FOXP3	FKBP10	PUS10	THADA
	LAPTM5	RHO	ACTR2	LAMC1	EFTUD2	GRAP2	NEK6	THG1L	DNAJC18	METTL13	TLR8
	SMOX	ZC3HAV1L	AP4B1	PTPN18	DIP2B	ME1	CNRIP1	SSTR2	FMNL1	HEG1	KBTBD11
	TNFSF8	PLXNC1	MSH2	LDHAL6B	ARHGAP10	C2orf29	IRF1	PALMD	KIAA1462	VASH1	PARVA
	DUSP11	SP3	PLOD1	DBH	RXFP1	GNL1	NPHP3	C10orf128	METAP2	RNF128	APBB1IP
	INPP5B	FLI1	CENPJ	FCGR1C	INPP5D	HPCAL1	C1QC	OBFC2A	MED8	MYOCD	DOK2
	ABLIM3	KIF21B	ORC2L	HES1	SH3KBP1	EDEM1	GEN1	RPGR	GAB3	C21orf63	RAB9B
	C9orf47	C1QB	TICAM1	ZCCHC11	CDKN1A	MGAT2	DAPP1	BIRC3	ADRBK2	HK2	PLOD2
	CWC25	CRK	PPP1R16B	SH3GL1	ACSM4	CLIC2	TUBB6	RBM17	RASA3	LYVE1	LOC145783
	LDHA	DOCK1	IWS1	CDC27	DDX26B	C1orf38	C1orf112	HECW2	VAV1	NUFIP2	DZIP1L
	FES	NBPF15	HSPB8	UGCG	MS4A1	SPOP	RSC1A1	PPP1R9B	RAB3IL1	ARSB	RNASE6
	TMEM71	IGSF21	LRRFIP1	APOBEC3F	NAP1L1	NFE2	CTGF	ANKRD6	SEMA5B	CSF1	FCGR1B
	CEBPB	COL4A1	FUBP1	CD72	SDC3	HOXA4	DSE	RCN1	FAM104A	C17orf87	RAB20

	TMEM200A	EBI3	ZNF608	GGT5	EEF2K	TWIST2	ILF2	RARA	FCGR3A	CCL23	YWHAQ
	CXCL12	BRE	CACNA2D4	RNFT1	CEP110	EPHA3	RRM2	GBGT1	GGCX	FCAR	CD6
	APLNR	LOC84856	PAK2	IL18R1	RNF112	C1QA	RAB39	PIK3CD	IL21R	CNR2	MAP7D3
	PAPOLG	RAB23	MSTO2P	DHX33	TMEM26	GPR132	PPPDE1	NFATC3	PHC1	KPNA2	SFT2D2
	COL5A2	PRKD3	PMS1	KCNA6	C19orf59	IFITM3	AP3S1	SLC39A10	CREG1	PHACTR4	IKZF1
	HVCN1	CYFIP1	C2orf49	ATXN1	PEX13	NOL9	CALHM2	OR5V1	FRMD4A	HEYL	COL15A1
	DUSP22	ARHGAP25	PSMB2	HELZ	P2RX7	ADORA2A	HDAC3	SLAMF1	PLSCR1	CDH2	AFAP1L2
	LOC644538	NTF3	FN1	DYRK2	ZNF641	C9orf96	KCNJ5	CSRNP2	CCDC88C	RNF125	GPR141
	FCGR1A	PIK3CG	NAIP	STX2	IL10RB	CD80	TESK2	KERA	WSB1	FMO3	GIMAP4
	GRASP	AHDC1	IFI44	SLC26A10	MIDN	IL10RA	DAPK1	MAPK14	C4orf47	PMEPA1	PTENP1
	TSPAN18	EVI2B	VRK2	FAIM3	HPS5	C1orf162	SLC7A5	C17orf85	LAMC3	CLSTN3	ALPK2
	RABGAP1L	USP48	WAS	CMKLR1	ASCC1	LTA4H	HNRNPH3	THUMPD2	ITGAM	SLAMF8	MDM2
	PIK3C2B	TGFB3	SETDB1	C1orf113	PLSCR4	MTF2	FCER1G	SLFN12	ANKZF1	PHKA2	LIG3
	GAL3ST4	COPA	CTNNB1	SYNE1	ABI3	TNFAIP8	NID2	BCL10	FGD6	PRIMA1	IL6ST
	STX4	MCM6	DDB2	TLN1	HLA-DRA	WDR46	ANTXR1	CD28	WARS	ENG	CEP68
	S100A16	SERBP1	MFGE8	RHBDF2	DCDC1	PCDHB3	TMEM14E	AMICA1	PKD1L1		
	JOSD1	PLK3	IL4R	DNAJB5	FLNA	CORO1C	PYGL	PHF13	SGK1	IL8	MYADM
	KIRREL	MSN	NDEL1	F13A1	SNAI1	WWTR1	DPYSL2	CSGALNACT2	SERPINB8	SERTAD2	CXCL2
	CD300E	JUN	FPR2	GPR135	RNF19B	AFAP1	CCL2	DCLRE1B	DUSP14	KIAA1949	PADI4
	TNFSF18	CD209	MLKL	NUPL1	IFFO2	POLD3	S100A12	S100A9	EHBP1L1	SLC15A4	SMARCAL1
	FPR1	ACTN1	EPB41L2	AMPD2	SAP130	UTP6	SEC23A	ARID5A	RASSF3	MYD88	DLC1
**Lost from S2**	C5AR1	SEMA4C	LMNA	PRPF38A	PSMD11	NRP1	VIM	PLEKHO2	ZEB2	GPR183	KCTD20
	RASSF2	WDR47	PTPRE	MYOF	CFLAR	EPHA2	APOBEC3A	C1orf216	GNB1	IFNAR2	RAD51L3
	LRRC59	SP110	PHLDB1	JDP2	CAP1	NR4A3	MRC1	PLAUR	FILIP1L	NRP2	CEP170L
	KLF4	CD97	BYSL	MPZL1	S100A8	ARNT	HIVEP2	RNF217	ISG20L2	GNAI3	EGFLAM
	ADPRH	CRISPLD2	DCUN1D3	CEP170	STK10	RAI14	KALRN	TPM3	PDE4B	CHST11	CRLF3
	PER2	PHACTR1	CTTNBP2NL	MCTP1	TRAM2	NFKBID	PELI1	TUBA1A	COTL1	PLXND1	P2RY8
	ATP8B2	ARL4C	GPATCH2	CASP10	PLEKHG5	RNASE2	IL2RA	C10orf54	STAT4	TET3	FAM110C

	DDIT4	C10orf10	GMEB1	CCDC46	PIP4K2A	TNFRSF1A	PIK3R1	CAMK4	C6orf145	SRF	RILPL2
	CFH	HTR6	BCL3	NR1H4	GON4L	FCGR2A	RLF	FTO	EIF2C1	LOC284441	PMP22
	SFPQ	SH2B3	PIP5K1A	LILRB2	NFKB2	GNAI2	ELMO1	IRAK3	PRICKLE2	CASP5	ETV3
	STARD13	BCL9L	CCDC88A	ODF2	TNFRSF1B	OSM	LHX8	IL7R	TP53BP2	UCK2	LMCD1
	SLC2A1	LILRA5	KPNB1	CSF2RB	SIRPB1	SUPT7L	CMIP	LIPN	ABCB6	USP18	SHC1
	NLRP3	KCMF1	SERPINB9	RALB	MAT2A	SELL	EHD2	SLC25A43	DYNC1I1	IFI16	CCL4L2
	TUBGCP3	CLEC4D	AK3L1	DISC1	PROS1	RGS9	BTBD19	TRIP12	MSL1	MTCP1	DENND5A
	C1S	FOSB	RBM8A	PHC2	C6orf150	ARRB1	ATF7	BGN	RGL1	AOAH	ITPRIP
	EMR1	FCGR3B	TRPC4	H6PD	TRIM9	ARPC2	IL10	ENO1	SF3A3	ARRDC3	S100PBP
	SKIL	C3orf59	LYAR	WDFY2	HCLS1	CLEC4C	ZNF643	C1R	SUPT6H	SLC13A5	FAM38A
	TUBB	CACNA2D1	BHLHE41	WDR26	SHROOM4	TAF12	SAMSN1	TRIM5	CASP7	GPR176	INVS
	CBLB	FAM102B	ALDH18A1	MMP14	A2M	ARHGAP29	VSTM1	NRBP1	SLC16A3	C13orf18	AGTPBP1
	PCBP1	DUSP12	DYRK3	YEATS2	SMAP2	IPO9	ACBD3	ULBP2	OTUD3	ITGB1	CALD1
	CEACAM3	APOBEC3C	SH3PXD2B	ADAP2	MTF1	SRGAP2	GLIPR1	LOC643837	CASP1	LYN	EXOSC10
	TNFRSF10D	DDX3X	KCNK3	DAXX	HEATR5B	AG2	LIF	LCP2	UGGT1	LRRC42	ELK3
	ZC3H12A	PDK1	LRP1	DNAJC10	TAF5L	CASP4	EPC1	FBXO42	FPR3	CXorf21	GLIPR2
	NOTCH2	HNRNPU	RPS6KC1	YY1AP1	ZCCHC24	RIPK1	ATP2B4	PDIA5	CHIC2	DEDD	MAP7D2
	IPMK	IL13RA2	RNASE1	MKNK1	SLC11A1	NNMT	GLT25D1	SFRS4	CLEC4A	MESDC1	FCHSD2
	WDFY1	PPT1	TEAD4	TTLL4	FAM55C	ARHGDIB	SIRPA	ELL2	TMCO7	SIRPB2	EMILIN2
	CXCR2	JMJD6	COL12A1	ZNF267	KCTD11	CSF1R	NBPF10	MID1	PLEK	LRRC37B2	NONO
	ANXA5	ZNF496	IRF2BP2	MS4A6E	C1orf183	TTC28	CCNYL1	CD86	RFX3	GBP1	CLASP1
	HK3	PCDHGC3	SAMHD1	PDLIM1	C5orf58	PBX2	LBR	UBC	FUT11	CD53	GPLD1
	IRS4	MNDA	FGD5	MSRB3	TPM4	FOLR2	SFXN3	SYDE1	KIF3C	EHD3	THRAP3
	EML4	LINGO1	HNRNPH1	ADCY3	FSTL3	C19orf38	STAB1	LRRC32	ACLY	KRT75	MFSD11
	TLR6	NECAP2	TTLL7	ENTPD7	SSR3	FADS1	BAT2L2	REL	MAP3K14	ITGA5	ADAM19
	GPR161	CFHR1	MMP8	ZNF462	SLC6A5	VEZT	FCN1	FADS3	OAS3	CCNL1	LCP1
	TYMP	GPR4	SP100	SPEN	RSU1	TTC13	AATF	NOS2	LIX1L	CCL8	BICD1
	CD33	SLC24A4	TRPV2	LILRB1	EMR2	TRNAU1AP	RAD51L1	AXL	EMR3	RIMKLA	ABCF1

	CD14	RASGRP2	TAF11	EIF2C3	SGK269	SAP30	MYO1B	ZYX	MYLK	HELB	FAM20A
	CSTT	CD300C	UGDH	LEPRE1	KCTD3	ZNF597	TRAF1	S100A7	ELK4	LIPI	PLEKHA2
	CASP8	SKI	THOC5	KLRG1	RFFL	CD226	KLHL33	HDGF	LAIR1	MAP1D	NES
	IL6R	CEACAM4	SACS	BTN2A1	DCBLD1	KDM6B	MPEG1	C3orf64	PREX1	CD69	CCDC102B
	GFRAL	DAAM2	TNKS1BP1	ACTR3	YBX1	PTRF	PLEKHG2	TMEM2	PDCD1LG2	MAP2K3	C13orf33
	PLEKHM3	SLC30A1	MAF	MS4A6A	FAM129A	WDR33	SPRY4	IFNGR2	SLC16A1	NRAS	BARD1
	TRIM38	CD68	TMEM217	GUCY1A2	APLF	ZSWIM2	BMP2K	VSIG4	CCL7	C13orf29	CD200
	DENND3	GPR84	PLXDC2	NFATC1	GIT2	ACTN3	EHBP1	NAV1	BLMH	FUT4	TRDMT1
	SEL1L3	P2RX1	C6orf114	IL4I1	CXCR4	KIF2A	IL1R1	BBS9	TNFSF11	SOX7	HIP1
	CALCOCO2	DHX9	FYN	LOC100134259	F2R	SPSB1	IRAK2	KDM3A	AGAP1	NLRC4	IL24
	MYO1G	TNFAIP1	SLCO2B1	COL27A1	CD4	TTC4	CHD1	USP31	ITPRIPL1	KIAA0090	SRBD1
	FAM26E	CDH11	CHSY3	ST3GAL2	MDC 1.00	PARP1	DAB2	C1RL	KDM5B	APOLD1	CAPZA1
	PAG1	GRK5	DHX57	SDCCAG8	SPARC	SLC1A4	SIGLEC5	FHL3	R3HDM1	CR1	RUNX2
	LOC283663	NIN	PGM2L1	RASGRP4	PRKCB	WASF2	SMC6	DSCC1	STK17B	LPGAT1	GRRP1
	DDX5	RELB	IRF9	SLC25A37	LY6G6C	FSTL1	EIF2B4	TRA2B	CLEC7A	NRIP1	HMOX1
	C15orf39	ADAMTS2	ENAH	RNF19A	TNFSF13B	CSDA	BCL6B	MBD5	HDX	RNF24	YME1L1
	C12orf68	TMEM106A	TMEM22	JAG1	RPH3A	AVEN	PLEKHM2	OLFML2B	TXNDC12	COL4A2	WIPF2
	YWHAG	TTC21B	UBE2Z	CDC42EP3	PDE3A	UBTD2	BMP1	EPAS1	NCKAP1L	GUCY1B3	INSIG2
	SLC4A1AP	RFTN1	CD5	OSBPL11	RASAL2	PPP4R1L	LOH3CR2A	LILRA1	RGS2	TARDBP	GPCPD1
	CASC3	PLEKHG1	C3	GYPC	HCK	CDC42EP4	TYMS	FZD5	LOC282997	SIGLEC9	POLR3C
	FKBP7	TLR1	MAP3K2	CXCR1	TGFB1	PCDHAC2	CEP135	IDH1	APOL3	ZMYM4	DYSF
	VANGL1	SWAP70	TAF13	MCAM	IER3	EGLN3	MS4A4A	FNDC3B	ARHGEF10	BCORL1	STAMBP
	CDK12	NUP85	ARHGAP22	CCL19	CDK2	LRRC33	STX11	SNRNP40	C14orf149	WDR35	FHOD1
	CHST15	PDGFB	RASSF5	KATNAL1	STK38	GYPE	ALS2	XPNPEP1	PPP3R1	RASAL3	LHFPL2
	BNIP2	TULP3	TMEM173	MAML2	IER5	SIGLEC10	VAMP3	NOL3	SOCS5	BCL2A1	MAP3K12
	CCR1	LZTS1	RCSD1	DOCK2	KHDRBS1	CHEK2	MTHFR	AIF1	FRMD4B	RPIA	AMDHD1
	CSDE1	EHD4	P4HA2	ZCCHC2	TTYH3	CYBB	PARP14	DHX8	SYNJ2	PDLIM3	SOD2
	ARHGAP30	PRDM1	UBE2J1	PANK2	RQCD1	HNRNPA3P1	KLF2	CD300LB	KRT32	C17orf107	C2orf48

	SIGLEC12	NBPF9	PNMA2	SAMD8	CD1D	CYP7A1	C3AR1	PML	GAS2L3	ETV6	C6orf204
	RECQL	OGFRL1	SLIT3	SP140L	TTYH2	RNF145	VCAM1	FBXL7	MCTP2	ARNTL2	ARHGEF2
	KSR1	KLF8	CEP164	TRIM34	SQRDL	CD93	CGREF1	ENTPD1	FAM57A	ZNF438	FAM38B
	ERCC3	PTPRM	CD163	SLFN5	AGFG1	NFAM1	FKBP1A	P2RY6	FFAR3	NBPF14	ITPKC
	CLEC2B	TMEM218	MYO1F	ANTXR2	ARL13B	C1orf198	RPA1	PEA15	TRIM6	FEZ1	CSF3R
	UIMC1	RAP2A	DCHS1	CSL21	APAF1	RPGRIP1L	IL13	DKK 3.00	DLX5	FOXP3	FKBP10
	KCNE4	PUS10	THADA	LAPTM5	GIMAP5	RHO	ACTR2	LAMC1	EFTUD2	TBCCD1	GRAP2
	NEK6	THG1L	DNAJC18	BNIP3L	METTL13	TLR8	SMOX	ZC3HAV1L	AP4B1	PTPN18	ENPEP
	DIP2B	ME1	CNRIP1	SSTR2	FMNL1	HEG1	KBTBD11	TNFSF8	PLXNC1	MSH2	ARHGAP10
	C2orf29	IRF1	PDGFRB	PALMD	KIAA1462	COL6A3	VASH1	PARVA	PLOD1	DBH	RXFP1
	GNL1	NPHP3	C10orf128	NID1	RNF128	APBB1IP	INPP5B	FLI1	CENPJ	FCGR1C	INPP5D
	HPCAL1	ERRFI1	C1QC	MED8	SEC14L1	MYOCD	DOK2	ABLIM3	KIF21B	KLHL20	SH3KBP1
	EDEM1	GEN1	RPGR	GAB3	C21orf63	RAB9B	C9orf47	POLR1E	C1QB	TICAM1	ZCCHC11
	CDKN1A	MGAT2	DAPP1	BIRC3	ADRBK2	HK2	PLOD2	CWC25	CRK	CXCR7	PPP1R16B
	SH3GL1	ACSM4	CLIC2	TUBB6	RBM17	RASA3	LYVE1	LOC145783	LDHA	DOCK1	IWS1
	CDC27	DDX26B	C1orf38	C1orf112	HECW2	VAV1	NUFIP2	DZIP1L	FES	NBPF15	HSPB8
	MS4A1	SPOP	FAM70B	RSC1A1	PPP1R9B	RAB3IL1	ARSB	RNASE6	TMEM71	IGSF21	NUP62
	LRRFIP1	APOBEC3F	NAP1L1	NFE2	CTGF	ANKRD6	SEMA5B	CSF1	FCGR1B	CEBPB	COL4A1
	FUBP1	CD72	SDC3	HOXA4	DSE	RCN1	FAM104A	C17orf87	RAB20	TMEM200A	EBI3
	TNFAIP8L1	ZNF608	GGT5	EEF2K	TWIST2	ILF2	RARA	FCGR3A	PDLIM2	CCL23	CXCL12
	BRE	CACNA2D4	RNFT1	CEP110	EPHA3	RRM2	GBGT1	GGCX	FCAR	CD6	APLNR
	AFARP1	LOC84856	PAK2	IL18R1	RNF112	C1QA	RAB39	PIK3CD	IL21R	CNR2	MAP7D3
	PAPOLG	NFKB1	RAB23	MSTO2P	DHX33	TMEM26	GPR132	PPPDE1	NFATC3		
**Conserved in S2 and S3**	NFIL3	PNRC1	ZFP36	WDR43	PPRC1	KLF6	C10orf119	SLC2A14	SNAPC1	SLC2A3	DDX21
	MCL1	UGCG	SOCS3	ADAMTS4	NAMPT	POLR1B	RIOK1	PLK2	KIAA0146	NOLC1	PTP4A1
	PUS7	JUNB	ZFP36L1	FLJ36031	FOS	LDLRAD3	EGR1	PPM1D	PFKFB3	NCL	BAZ1A
	ELOVL5	DUSP1	ZNF143	IL1RAP	STEAP4	NUP98	NFYA	PNO1	RHOB	PRPS1	ASNSD1
	HNRNPF	CCRN4L	DDX18	C9orf21	RBMS1	JUB	EEF1B2	TEX10	ARL6IP6	ZNF207	HAT1

	TMEM49	USP37	NOC3L	STAT3	PANX1	WTAP	C7orf68	GNA13	PDIA6	RNASE4	EIF4A3
	SERPINE1	C1orf107	HSPA14	POLR2D	CEBPD	PPP1R15B	MSH6	EIF2S3	DDX50	ARL5B	TMEM67
	ZRANB3	RNF149	ADAM17	ICAM1	TIAL1	KCNJ8	KLF10	SLC16A2	ADAMTS1	CYR61	AMMECR1L
	NFKBIA	TGFA	HNRNPA3	SFRS3	CSDAP1	WDR75	SFRS13B	B3GNT5	MMADHC	MALT1	ATF2
	SPRY1	HSPA5	ANKRD40	TNFRSF10A	GPN1	ATP13A3	SYNCRIP	TNFAIP3	ZHX2	TTC27	OSMR
	FOSL1	LDHAL6B	C2orf49	SF3B1	URB2	YWHAQ	XRCC5	GALC	PAK1IP1	FOSL2	RND3
	NSMAF	37500	WDR12	CDC5L	PTPN12	DYNC1I2	CEBPZ	EIF4A1	CCL20	METAP2	ATF3
	TCP11L1	FOXC2	ANG	TXLNG	TFPI	PRPF40A	HNRNPC	NUP153	GTPBP2	ASAP1	SSRP1
	EGR3	ZNF318	SP3	WEE1	TMEM194B	LOC100131551	STC1	S1PR3	SLFN11	GDI2	HPS3
	GTPBP4	CXCL3	FAM49A	ZBTB2	EPC2	BCL6	DUSP11	TSR1	ABCC9	RBM15	SLC39A10
	HSD17B7	ORC2L	FARSB	HES1	CREM	STAG1	PLSCR4	GEM	HEATR1	SERTAD1	CSRNP1
	LATS2	RBMXL1	ETS2	BZW1	SFRS7	LONRF3	ARID5B	GYM65	SRGN	FHL2	GEMIN4
	PPP1R3B	ZNHIT6	FAM119B	C1orf55	OBFC2A	C9orf30	THBS1	ABI2	GART	EMP1	MKI67IP
	PDCD11	ABL2	HNRNPK	DARS	P2RY14	MAP4K4	IL7	CWC22	MTERFD2	MT1X	ETS1
	SULT1B1	SDCBP	SH3BP5	IL1B	HNRNPR	SNORA8	RPS6KB1	NHEDC2	ZNF830	PGM3	PPBP
	CHSY1	C2orf86	GTF2H1	HBEGF	ZNF281	ECD	SLC43A3				
	NUDCD1	NUP35	EDIL3	RNF139	SMNDC1	RPL7	TMEM185B	EIF3E	ZC3H15	MIPOL1	TAF4B
	ADM	YRDC	EREG	TCEA1	EIF3J	MED30	DKFZP434K028	ZFAND1	CALCR	WDYHV1	PATL1
	TAF1D	TRIB1	APEX1	PABPC3	GTF3C3	SIRT1	RPS12	PABPC1	NCRNA00188	SEH1L	SNORD1C
	PCDHGB2	NT5E	E2F5	RBM12	EIF2C2	UTP18	UBA2	LOC100133669	NFE2L2	MIR17HG	CCT4
	NCOA7	TIMM9	LOC100127888	NOP58	RPS13	EIF3H	ORM1	NETO2	RBM7	YWHAZ	C20orf199
**Acquired in S3**	ABCE1	FASTKD2	SLC35A1	ADAMTS9	TMEM209	CCT2	OLA1	RPL10A	EIF2S1	CA1	HMGN4
	SLC22A3	UBXN4	TTC9C	RPL7L1	PLAC8	WIT1	RPS3A	SUV39H2	PM20D2	AZIN1	ERO1L
	KIN	RBMX	MTDH	HSP90AB1	SLC25A32	ARMC10	EXT2	CSE1L	TIMP4	ALS2CR4	NMD3
	USP49	LOC728190	YY1	CNIH	MORF4L2	MLLT10	MMRN1	SLC39A6	CLDND1	EEF1A1P9	DIO3OS
	RPL31	ACTL6A	C12orf11	PKNOX1	MID2	NUAK2	C6orf72	MTHFD2	H3F3B	POLH	BTF3
	HSP90AB2P	TMEM39A	RPS6KA5	MAD2L1BP	ZNF410	NKX3-1	C10orf18	PRMT5	EXOC6	DKFZP434H168	UNC50
	NXT1	DERL1	MTPAP	TOMM20	IFITM5	CD2AP	DPAGT1	SLC35F5			

	PNRC1	SNAPC1	NUDCD1	ADAMTS4	NAMPT	PLK2	KIAA0146	PUS7	NUP35	EDIL3	RNF139
	FLJ36031	RPL7	TMEM185B	PPM1D	EIF3E	BAZ1A	ELOVL5	MIPOL1	STEAP4	PRPS1	ASNSD1
	YRDC	RBMS1	EREG	JUB	EEF1B2	ZNF207	HAT1	TMEM49	TCEA1	EIF3J	MED30
	DKFZP434K028	ZFAND1	CALCR	PANX1	WDYHV1	WTAP	PATL1	C7orf68	GNA13	PDIA6	RNASE4
	SERPINE1	TAF1D	TRIB1	HSPA14	POLR2D	APEX1	CEBPD	PABPC3	GTF3C3	PPP1R15B	RPS12
	PABPC1	EIF2S3	ARL5B	NCRNA00188	TMEM67	SEH1L	RNF149	ICAM1	KCNJ8	PCDHGB2	SLC16A2
	CYR61	NT5E	E2F5	NFKBIA	RBM12	WDR75	LOC100133669	SFRS13B	NFE2L2	MMADHC	MALT1
	ATF2	MIR17HG	HSPA5	CCT4	NCOA7	ATP13A3	TIMM9	SYNCRIP	LOC100127888	TNFAIP3	ZHX2
	RPS13	OSMR	EIF3H	ORM1	FOSL1	LDHAL6B	C2orf49	SF3B1	YWHAQ	XRCC5	GALC
	PAK1IP1	RND3	NETO2	NSMAF	RBM7	WDR12	CDC5L	YWHAZ	CCL20	C20orf199	ABCE1
**Lost from S3**	FASTKD2	TCP11L1	SLC35A1	ADAMTS9	ANG	TMEM209	CCT2	TFPI	OLA1	RPL10A	HNRNPC
	GTPBP2	SSRP1	EGR3	ZNF318	EIF2S1	CA1	TMEM194B	LOC100131551	HMGN4	S1PR3	SLFN11
	GDI2	HPS3	CXCL3	FAM49A	ZBTB2	SLC22A3	UBXN4	BCL6	DUSP11	TSR1	TTC9C
	RPL7L1	PLAC8	WIT1	ABCC9	RBM15	SLC39A10	RPS3A	HSD17B7	SUV39H2	FARSB	PM20D2
	STAG1	AZIN1	ERO1L	KIN	PLSCR4	MTDH	GEM	HSP90AB1	SLC25A32	ARMC10	SERTAD1
	RBMXL1	ETS2	EXT2	CSE1L	TIMP4	ALS2CR4	NMD3	SFRS7	USP49	ARID5B	SRGN
	FHL2	LOC728190	YY1	CNIH	MORF4L2	PPP1R3B	MMRN1	SLC39A6	OBFC2A	C9orf30	GART
	CLDND1	MKI67IP	PDCD11	EEF1A1P9	DIO3OS	DARS	RPL31	ACTL6A	C12orf11	P2RY14	PKNOX1
	MID2	NUAK2	C6orf72	MTHFD2	POLH	BTF3	HSP90AB2P	TMEM39A	MAD2L1BP	ZNF410	NKX3-1
	C10orf18	PRMT5	EXOC6	MT1X	SULT1B1	SDCBP	DKFZP434H168	SH3BP5	IL1B	SNORA8	UNC50
	NXT1	NHEDC2	ZNF830	PGM3	PPBP	DERL1	CHSY1	MTPAP	TOMM20	GTF2H1	IFITM5
	CD2AP	ZNF281	DPAGT1	SLC43A3	SLC35F5						
	KLF6	NFIL3	FOSL2	JUNB	MCL1	PFKFB3	WDR43	MTERFD2	C10orf119	DUSP1	DDX21
	NOLC1	ZFP36	TAF4B	ANKRD40	PPRC1	HNRNPA3	NOC3L	B3GNT5	CCRN4L	ECD	AMMECR1L
**Conserved in S3 and S4**	EIF4A3	RPS6KA5	ABI2	EMP1	STC1	PTP4A1	EPC2	SMNDC1	C9orf21	ABL2	SPRY1
	C1orf107	RIOK1	GTPBP4	PTPN12	SOCS3	ETS1	POLR1B	ORC2L	TIAL1	URB2	TXLNG
	MLLT10	LDLRAD3	SIRT1	ZRANB3	ZFP36L1	GEMIN4	SP3	RBMX	UGCG	HNRNPK	SLC2A14
	METAP2	ARL6IP6	HBEGF	RPS6KB1	CWC22	EIF2C2	C1orf55	FAM119B	NUP153	ZNF143	FOXC2

	DYNC1I2	IL1RAP	UTP18	C2orf86	SLC2A3	LATS2	DDX18	ASAP1	ADAM17	DDX50	NCL
	KLF10	TNFRSF10A	GPN1	HNRNPF	LONRF3	H3F3B	IL7	ZC3H15	HEATR1	PRPF40A	CREM
	CEBPZ	STAT3	TGFA	CSRNP1	NUP98	UBA2	C2orf90	ADAMTS1	BZW1	CSDAP1	ATF3
	THBS1	NOP58	RHOB	TEX10	PNO1	ADM	USP37	NFYA	MSH6	HNRNPR	MAP4K4
	HES1	ZNHIT6	WEE1	EIF4A1	EGR1	SFRS3	SNORD1C	TTC27	FOS	C2orf76	
	KLF4	HNRNPH3	DUSP6	ELK3	ARRDC3	SPRED2	CAMSAP1	TMC7	B3GALT1	SEMA4C	ZNF462
	ITPRIP	INVS	PEAR1	EPAS1	STARD13	DUSP5	SPRED3	ISG20L2	TUBGCP3	SPAG9	EPHA2
	FAM110C	EXOSC2	JMJD6	NAB1	HNRPDL	RNF19B	PHF3	NPDC1	LRRFIP1	MYO1B	DOCK5
	RFX3	C16orf72	HNRNPU	ADCY3	WAC	SH2B3	CNBP	GPR135	FOSB	RUFY2	EFNB2
	ERLIN1	WDR47	CDC27	ZNF496	ARHGAP29	PPP3R1	TM4SF1	CALCOCO2	LOC100302650	SOX7	SPRY4
	LOC344595	FUBP1	CEP68	GPATCH2	TGFBR2	NDEL1	BHLHE40	DNTTIP2	DDX3X	CHD1	UPF2
	DIAPH2	TNFAIP1	USP31	SERTAD2	NRP1	ZCCHC2	SCN4B	SAMD8	ZEB1	LOC440354	LRRC42
	OTUD4	CPSF6	DDX42	TTLL11	CSGALNACT2	SLFN5	JOSD1	ZNF25	YME1L1	MFSD11	GRIK3
	ASPH	TAF5L	SMC3	RAPGEF5	CCDC46	KIAA0355	F2RL3	THRAP3	DDX5	SLCO2A1	PSMD11
	THUMPD2	CDKN1A	SRGAP2	COIL	PCDHAC2	FAM38B	CDK17	NCRNA00120	EBF3	MBD5	HNRNPA3P1
	CDNF	NEK5	SMURF2	LMNA	LOC641367	JMJD1C	BYSL	EPC1	NAA25	GRB10	PGM2L1
**Acquired in S4**	RALB	HTR7P1	PNP	CRK	RGS9	SPRY2	KCNE4	PGAP1	BRSK2	HSPG2	DGKD
	OR56B4	ZNF343	C2orf65	YEATS2	ROCK2	USP13	DHX33	SEC14L1	S1PR1	KHDRBS1	HEATR5B
	CREB1	HERC4	KLF7	DEFB118	CNST	TARDBP	NOTCH1	SHROOM4	SLC26A10	ANKRD27	C20orf112
	GPATCH8	SWAP70	MCFD2	NR4A2	VPS24	ZNF584	RPGR	CCDC55	SPEN	TCTE1	STAM
	MAPK3	LIG3	LRIG2	MPP3	PCDH17	LOC286094	ETV3	SERINC3	CLIC2	CDKN2B	PCSK5
	RAB3GAP1	PMS1	MCTP1	WDFY1	PRPF38A	CST11	C17orf46	OTUD3	RSL1D1	AGTPBP1	KCTD20
	C9orf150	ACLY	HIVEP2	ALS2	TMEM22	APOLD1	DOCK9	CHMP1B	PRDM10	ZNF146	ITGB1
	PLEKHG1	VPS26A	WWTR1	MAPK8	DCUN1D3	SFN	TASP1	BMS1	SMC6	FBXO11	TCF23
	ANO2	PAPL	SRP68	MAFG	MAP1D	JAG1	UTP6	LRRC70	RAPH1	IRF2BP2	RND1
	NCK1	DLL4	VEZF1	PELI1	PPP2R2A	IL4R	ARHGAP17	MDGA2	PIBF1	GPR161	LPHN2
	ANKMY1	RASA2	SNRK	DDIT4	FRZB	TNFAIP8L1	DOCK1	C3orf64	GPR4	SKIL	MYADM
	MOBP	PPIG	RARA	KCNE3	IL1RAPL1	C20orf117	FBXO45	WDR3	C10orf10	ADAM15	RBM8A

	GMCL1	TAF5	ZC3HAV1L	UBC	CCDC88A	RIF1	B4GALNT1	CHML	FBXO48	ABCG2	APLF
	SV2C	PLXND1	ARAP3	FEZ2	PPP2R2C	PEG10	AKT3	AP3M1	PPARGC1B	ZNF507	DLC1
	H3F3C	WAPAL	GABRB3	PFKP	USP36	CDC73	THBD	BMX	MASTL	SGK1	QSOX2
	FOXD2	GMEB1	TMCO7	PCDHA7	LDB2	PLEKHA1	ADAMTS5	ATP11B	ANKAR	TACR1	RGS5
	MTAP	CCNYL1	EFTUD2	ATAD2B	KBTBD2	ING1	C3orf36	UBE2Z	GDAP1	TRDMT1	CDK12
	STAMBP	LOC399815	DNMBP	OR2W3	CEP170	JUN	HOXD10	IL6R	CXCR7	FNDC3B	TNKS2
	DOCK6	RPE	RPE54	HNRNPM	FLT1	PITPNC1	PHOSPHO2	ATG12	GRK5	SLC10A6	MSL1
	ZNF500	RQCD1	VEZT	GJC1	RBMS3	C10orf78	BCL6B	TUBD1	TGFB1	UIMC1	PHLDA1
	ANAPC1	C18orf54	CLEC1A	PIK3C2B							

C) List of lost, conserved and acquired genes across stages in KIRC:

**Lost from normal**	CEBPB	SPHK1	AHNAK2	NUMBL	TUBA1A	CITED4	KIAA1949	CXCL6	ANXA1	CSF1	BLK
	ARL4C	PDLIM1	PODNL1	SYNC	RAC2	C1QTNF1	PEA15	FSTL3	AKAP12	RBM24	SERPINH1
	PLEKHO2	MYCL22	HRH1	COL8A1	MMP14	PLEKHO1	DARC	BAI2	MTHFD2	HAPLN3	POU2F2
	TM4SF4	CREB5	ZSWIM4	MAP3K12	SPSB1	CXCL1	MMP19	CD276	NLGN2	DLGAP1	PTPN1
	MAGED4	PLD2	MMP2	LOXL1	TGFB1I1	SLCO5A1	FUT4	IER5	C1R	TXLNB	IL4R
	LOC100126784	MAGED4B	EXT1	DLGAP4	HEYL	PDLIM7	GPR37	WNT2B	MICAL1	FILIP1L	TTLL7
	KCNT2	SERPINB9	PHF17	TIMP1	DTNA	MAP7D1	SYT11	SGK223	CPA4	C1S	KDELC1
	BIRC3	GRASP	SPON2	UGCG	MICAL2	LIMD2	B4GALT5	NBL1	EHBP1L1	C6orf174	COL16A1
	SLC27A3	IFFO2	CNTNAP1	ADAM8	C1orf38	CD40LG	CORO1C	LAT	NFIX	CMTM3	CRLF2
	TNFAIP2	PLK2	TLE1	RIN1	CCIN	RRAS	SCARF2	CHI3L2	BTN2A1	MEIS3	BMPER
	CD97	CYP19A1	PXDN	LZTS1	ZDHHC18	CD44	VIM	BATF3	CFH	IGFBP6	RHOG
	KRT86	MAMLD1	FADS3	RASA3	TMEM43	GDF5	CD69	IGFN1	TMC8	TGFB3	CDV3
	TUBB3	CNR1	COL6A2	RELT	LOXL3	RCN3	PIGR	AIM2	PLEKHA4	THBS3	TMEM233
	TNFAIP8L3	LSP1	LMCD1	MAP1LC3C	CACNA1I	FSTL1	VCAM1	ITGAX	CCL21	SRPX	IFI16
	MICALL2	S1PR2	DOK1	RNF175	CXCL16	GNAI2	FSCN1	SH2B3	THBS2	TFE3	MEI1
	DUSP10	ATP8B2	NGFR	GATA6	KIF7	SORBS2	GAB3	RHBDF2	RASGRP2	PLXNB3	LY6H
	LOC100270710	RGS1	LRRN1	C1QTNF6	SMAP2	PIK3R6	CD3E	SERPINF1	LIX1L	TWIST1	GPR183
	FEZ1	SLC35C1	STEAP4	C11orf24	NLRP3	GOLGA7B	ITK	EMILIN2	GLIPR2	AEBP1	STAC3
	KLF2	IFITM3	GALNT5	C19orf30	C17orf60	FLJ41941	PVR	LRRC59	CD72	CCDC80	WAS
	SBNO2	BATF	KIAA1045	CLEC4F	ICAM4	FAIM3	SDK1	MAP4K1	MTA2	COLQ	MAP7D3
	IFITM2	TTC39C	PSTPIP1	DNAJB11	SFRP2	C6	RNF112	ARHGAP22	EPB41L2	FLJ16779	TSKS
	CD37	SLAMF6	YWHAH	HSPB8	WNT1	PCOLCE	YPEL4	LY9	C1orf162	IL7R	PLSCR3
	C10orf10	HSPA7	C6orf150	CADPS	CXCR4	MLKL	LHFP	ACAP1	CTGF	FJX1	CD6
	RAB31	PRR24	ARHGAP1	FLNC	C9orf25	BTBD19	MIA	BEAN	ELN	KCTD4	LDLRAD3
	PCDHB14	BTN2A2	DGKA	C13orf18	SHC1	GLDN	GDPD5	SEMA7A	ITGB3	RFTN1	C2CD4A
	DOK2	HCST	PSTPIP2	TNFRSF1B	STX11	LGALS1	OSR1	FHL3	ANKRD35	CDH22	NINJ2

	SACS	PTRF	VSTM2A	CD74	KRTAP5-1	PTGIR	CNKSR2	LOC100130776	BCL2A1	SDC3	SLA
	TCF7	MC1R	SERPING1	LOC338651	PIWIL4	CD248	MED17	FAM129A	CORO6	HCLS1	EMP3
	RHOU	MARVELD1	GRAMD1A	TMEM156	ACTN1	TMEM173	PRAM1	C3orf36	CADM3	CTSG	MAP4K4
	TMEM2	FUT7	SV2A	MDK	ADAM19	VENTX	ENAH	RXFP2	FERMT3	CARS	ISG20
	CCR7	PARVG	ARHGAP15	RASSF2	EFEMP1	MXRA8	LILRB3	HSPB6	SAMD4A	GGTA1	RELL1
	C11orf9	HP	OXTR	IL1R1	TPM1	OLFML2B	CCDC50	NLGN4X	NKX6-1	PDIA4	C1RL
	CD5	ANKRD13B	PLAUR	OBFC2A	GFI1	REEP2	LCP2	SH3PXD2B	CNTN6	C2orf18	NFAM1
	CXCL3	RASSF5	DAPK3	UNC5A	FLNA	MEX3C	DEF6	C1QA	LRRN4	CCL4	ZAK
	MT1A	ANGPTL7	FAM113B	COMP	CCDC88B	C2orf89	CCL14	COL1A1	PAPLN	C21orf7	GPC4
	C8orf84	LBR	SLAMF1	DYRK2	NHEDC2	NLRP1	IL18BP	CYP21A2	NRP1	ITGA7	CD207
	P2RX6	OSCAR	ZNF831	INMT	TWF2	TRAT1	MEX3A	LRRC25	ENTHD1	NT5DC3	RHOH
	POU2AF1	COL6A1	CACNB1	BDNF	FSHR	DTX3L	HTR7	B3GNT7	CCL4L2	SH3BGRL3	COL1A2
	SLITRK5	FGR	GPR55	DTX2	GFPT2	AGAP2	EDN1	CD27	SULT1C4	TAGLN2	TREM1
	PML	GPR171	KIF3C	GMIP	NEXN	IFNAR2	KCNH8	SELPLG	NPDC1	ZIC4	CSPG4
	C1QTNF2	C1orf198	FAM198B	CCDC97	CFP	MAP1A	ARHGAP30	CORO7	AKIRIN2	FZD1	GRK5
	CMA1	MMP11	SAMD14	CAP2	SLC24A4	ARMC4	TNXB	ALDH1L2	CCDC109B	KIAA0802	CORO1A
	C4orf55	PLAC9	MEF2D	FBLN7	BTK	CDH6	KIAA0748	PYGM	SEMA3A	UBE2I	HKDC1
	PODN	BEST1	LAX1	TNIP3	SH2D5	IL1B	GPSM3	FIBIN	ANKRD53	RGS19	MFGE8
	KLF7	CLEC10A	TBC1D10C	PTRH2	TNFSF13B	DACT1	CCL22	ATP2B4	KIAA1274	AMICA1	TIGIT
	DPYSL3	DPEP2	PLB1	ODF3B	EBF1	IGFBP7	HAND2	NFKBIE	MCAM	HSP90B1	GPR176
	RASAL3	BRSK1	KCNK7	PCDHGA5	PRICKLE1	C19orf35	JAK3	MAP1B	ITGAL	PALLD	SAMD3
	ACAN	TIMP2	IL1F10	APOBEC3D	GPRIN1	PDZD4	MAP3K8	LOH3CR2A	TNFSF8	GNG8	CNRIP1
	FMNL1	PLTP	SVIL	IKZF1	LRG1	TYMP	RIPK2	APBB1IP	TRPV2	TXNDC5	ETV6
	DUSP8	FAM92B	FPR1	C2orf85	PRIC285	SCGN	ADAMTSL4	NELL2	NAV3	SETD8	SNAI3
	CD3G	CLEC1A	ABCB4	ADAMTS2	SCG2	LAIR1	CCRL2	PITPNB	EFHD2	UTP6	FAM102B
	HSPB3	DCBLD2	FAM46A	ISLR	FNDC4	GRRP1	IFNGR1	WNT5B	CAMK2N2	CKAP4	SMARCD3
	IL31RA	PTP4A3	SLC18A1	NT5E	TNFRSF13C	CYTIP	COL18A1	NFKBIZ	SHOX2	FAM150B	CELF2
	SPEG	LILRB2	C16orf54	IL21R	KCTD10	CLEC2B	MAN2B1	PTX3	PRRX1	ASPHD1	TXNDC3

	MSN	COL5A1	CSTA	NAPSB	SIRPG	CEACAM4	KCTD5	SFRP4	ZCCHC5	SPI1	CDH24
	LEPRE1	HAVCR1	LRRN3	NAALADL1	IL10RA	TSPAN18	SAP30	WNT10B	PCDHGB7	ZBP1	BTLA
	KCNA5	PTCH2	NID2	CMTM7	CLIC1	IFITM1	ARL4A	IDUA	FBXL12	STK10	FCHO1
	MYBL1	SASH3	GAP43	PTPRCAP	CD300LF	RNASE6	IL34	MDFI	DOK3	DNM1	GNA15
	CABYR	CLDND1	ZNF365	FHL1	CENPW	TAGAP	CACNA1A	CCR6	CTTNBP2NL	STXBP1	MSC
	SMTN	PTPN12	TLN1	FHOD1	BIRC7	PHF19	PMP22	PLEKHN1	SOD2	MYO9B	RAB33A
	APOBEC3F	YWHAG	ANTXR1	ARSI	PIK3R5	VAV1	LGALS9C	LAT2	STAT4	TCEAL7	C20orf103
	MMP23B	CCDC140	AXL	KLF8	PLAC8	LONRF1	TNFSF9	CAV1	FXYD5	CHST7	LOC541471
	JPH2	PTPRE	ZFPM2	RAB35	LEPREL2	CSF2RA	CD3D	GBP2	LILRA4	CDH23	KIF21B
	TAGLN	PDGFRB	GMFG	STS	PCDHB18	BEND6	SPARC	ADCY7	TLR9	ZNF193	MYH9
	NLRC5	CEACAM21	S100A16	CD300LB	IRF8	SGTB	SH2D2A	CEBPD	RRS1	ASB2	GPR84
	KLHL6	GFRA1	LOC283050	GLIPR1	MEGF11	MIR155HG	FGFR1	OSMR	MED26	CD2	BBC3
	ARL 14.00	COL4A2	C12orf70	SEMA6A	ARHGEF1	PRKCDBP	NCF1	PLEK	CD151	NNMT	NRP2
	ELK3	MGP	RRN3P2	EPN2	C10orf105	WIPF1	SMURF1	WDR82	CCL23	TNFSF18	SEL1L3
	TP53BP2	C10orf54	GRK6	RASSF3	ARRDC5	MYOZ3	PTGFRN	KIAA1199	UBASH3A	PLCH1	MYO1F
	KCNA6	FCRLA	MN1	PGBD1	DCLK1	ARID5B	CD7	TREML2	RGL4	RD3	SAMHD1
	CALU	FCHSD2	CHFR	LIMK1	HTRA3	GBP1	DGKG	PRDM1	CCKBR	ITGB8	SLC24A3
	PTGDR	GLI1	RGS20	PPP1R14A	KRTAP1-1	EIF1AD	CST2	STK17B	COL6A3	ITGA3	MSRB3
	DISC1	CSF2RB	ROBO1	CCDC141	CLEC11A	UTP14A	CCDC46	LTBP2	FAIM2	ROM1	LOC400891
	CD300A	GRAP2	NRG3	CALD1	RNF145	C10orf128	CD96	LRRC15	VASP	KLHDC7B	FCRL2
	TRPC4	FAM126A	SPIB	TYROBP	NALCN	AFAP1	ARHGDIB	CXCR3	HABP2	PRRX2	CHSY3
	SDC4	CLEC4E	TPSB2	CMTM5	HECA	SHISA5	GRID1	HLA-E	LOC283070	LTA	CD1E
	CFHR3	IL16	PROCR	LRIG1	SFRS13B	FIGF	NPR 2.00	NUDT11	SHF	SLC1A4	FAS
	IL17D	PLA2G5	FLT3LG	UBE2S	MYLK	LPXN	TMEM119	LILRA6	ARVCF	KIAA0922	RBM46
	TMEM158	GUCY1B3	FBLIM1	NRBP1	KCNK6	IRAK3	CCDC81	SLIT3	CRISPLD1	SLAMF8	CLEC12A
	FAM5B	NAMPT	CCL5	LOC100134259	OMD	FRMD5	CNIH2	SLC2A10	ACTA2	TMEM196	NCF4
	TPM2	CALHM2	ARRDC2	TTYH2	CSRP1	CD63	TMEM39B	KAZALD1	RAB32	PRR16	CACNA1C
	AGFG1	TBX1	PECAM1	IL6	STX1A	NTRK3	TLR2	C9orf91	PIP4K2A	IGDCC4	HUS1B

	MMP17	STIM1	SYNPO2	SIGLECP3	MAP3K6	FKBP11	CD4	PACS1	INPP5D	WISP1	CRTAM
	IL17B	LOC283663	TMSB10	SIGLEC1	TPSAB1	PRND	LGALS9	GAB2	TNFAIP8L2	ADCY2	CSMD2
	HLA-DPB1	DACT3	TPRG1	MAP1S	LY86	FOXG1	CST7	CFB	LOC401093	CRLF3	EBF2
	SPARCL1	SIAH1	DCP1A	SH2D1A	BMP1	SLFN11	A2M	MEGF6	LPGAT1	RLTPR	PAQR4
	KRT75	ADAMTS12	GJA1	PTCRA	OLFM4	ARF6	TEAD3	FLI1	WISP2	CD48	ITGBL1
	FXYD1	FGD2	DEGS1	LOC100233209	LTBP4	CX3CL1	FLRT2	RCSD1	JPH3	NLRP12	GIMAP5
	GAPT	IL12RB1	SGCE	MATN3	SIX3	ADAM33	ADARB1	BASP1	AMOTL1	GRM7	ROR1
	APOBEC3C	LY96	C11orf87	GZMM	FCGR1B	HS3ST1	LST1	C4orf7	HAS1	CD52	CYTH4
	PSG1	KCNA3	CD86	VSTM2L	FGF13	LOC158696	MGC87042	EML3	RHOA		
**Conserved in normal and S1**	NFIL3	KDM6B	ZFP36	MIDN	PLK3	FOSL1	CCRN4L	NDEL1	MYADM	PPRC1	SOCS3
	EPHA2	RARA	KLF4	MCL1	C6orf145	ADAMTS4	JMJD6	CSRNP1	ERF	PPP1R15A	BYSL
	ITPRIP	PHF13	KLF10	DUSP5	SERTAD1	JOSD1	XIRP1	TICAM1	CYR61	ADAMTS1	ZC3H12A
	AREG	EIF4A1	DNAJB5	JDP2	KLF16	CLCF1	HBEGF	CDKN1A	POLR3D	BCL3	NFKB1
	LMNA	KLF6	SNAI1	DDX5	AG2	CCL2	CXCL2	SOX7	DDX21	IRAK2	SLC2A14
	ARID5A	DCUN1D3	URB2	TAF5L	BAZ1A	SPRY2	PPP1R15B	LYAR	FOSL2	ETS2	SLC2A3
	DUSP1	F2RL3	LIF	AMMECR1L	NAB2	ZYX	NFATC1	BMP2	DDX3X	GEM	TNFAIP3
	FRMD8	ISG20L2	B3GNT5	WDR43	PHLDA1	RELB	TNFRSF10A	KBTBD2	RHOB	RELA	CSNK1E
	MESDC1	SOX9	MAPK7	SEMA4C	SPATA2L	ZNF295	RND3	RIPK1	CHSY1	PHC2	SERPINE1
	RALGDS	APOLD1	TMEM217	KCTD11	C12orf68	NFKBID	THBS1	RFX2	SPRY1	AEN	RRAD
	CSGALNACT2	CHIC2	FERMT2	GPR4	NFKB2	LATS2	STAT3	CHD1	TNFRSF1A	PIM1	NIP7
	CDC42EP4	DUSP14	TUBB6	NFKBIA	LRRC8A	THBD	TSC22D2	CYTH2	NFE2L2	BRD1	HIVEP2
	HDC	PRKD2	MEX3D	CDC42SE1	PER1	TAL1	EIF2S1	WTAP	MAP3K14	PNRC1	TNFRSF10B
	C9orf21	ICAM1	SPRY4	HIVEP1	PLEKHG2	CDC42EP1	PDGFB	ETS1	ZNF830	RCE1	CCNL1
	RASSF1	BCL9L	GNL2	RNF122	SLC25A32	SNORA1	IPO4	LRRC42	RRP12	ADAMTS9	SERTAD3
	SERPINB8	C5AR1	ARHGAP17	DLC1	GTPBP2	CCDC9	ITGA5	TNFAIP1	C3orf59	RAB20	RBMS1
**Acquired in S1**	JUNB	FOSB	MAFF	MAP2K3	RIOK1	ITPKC	SFPQ	DUSP6	BHLHE40	PFKFB3	SRF
	EMP1	NOLC1	HES1	ATF3	SFRS15	PHLDA2	TRIB1	GTPBP4	YRDC	JUN	STK40
	FOS	IER3	MAT2A	PNP	PWP2	IL1RL1	SFRS7	MAFK	SIK1	GPR3	SF1

	TRA2B	ZBED4	STC1	NR4A1	IDI2	NR4A3	AATK	ING1	EIF4A3	ODF2	DOT1L
	ZFP36L1	ADM	SNORA67	ELL	EIF2C2	GPR135	EGR3	FAM107A	BCL6B	MAFG	FAM160A2
	PELI1	SPEN	LONRF3	PRPF4	TAF13	C10orf108	TGIF2	ATXN7L3	TRIM40	TDG	C15orf39
	INTS6	AOC2	SFRS3	FOXC2	ETF1	POLR1C	HNRNPU	NUP98	C9orf30	GADD45B	SAFB
	NUP153	DMWD	SOX21	TCEB3	USP42	NUAK2	TFRC	HYMAI	DNAJA1	RGS2	ZBTB7B
	LRCH1	C1orf183	MAK16	TNFRSF10D	FAM43A	GFOD2	NOP16	SFRS2	KBTBD5	KCNE4	NCL
	PIP5K1A	AKIRIN1	PTBP1	ARC	C17orf85						
**Lost from S1**	TICAM1	YRDC	F2RL3	BMP2	IER3	PNP	GEM	PWP2	IL1RL1	SOX9	MAPK7
	SPATA2L	ZNF295	SERPINE1	RFX2	AEN	AATK	FERMT2	ING1	NIP7	SNORA67	LRRC8A
	CYTH2	BRD1	PRKD2	MEX3D	MAFG	TAL1	FAM160A2	EIF2S1	MAP3K14	PNRC1	TAF13
	TNFRSF10B	C10orf108	TGIF2	CDC42EP1	ZNF830	RCE1	TRIM40	INTS6	GNL2	AOC2	SFRS3
	SLC25A32	SNORA1	POLR1C	HNRNPU	RRP12	ADAMTS9	SERTAD3	SAFB	NUP153	DMWD	TCEB3
	USP42	NUAK2	HYMAI	DNAJA1	ZBTB7B	LRCH1	MAK16	NOP16	GTPBP2	KBTBD5	CCDC9
	KCNE4	ARC	RAB20	C17orf85							
**Conserved in S1 and S2**	FOSL2	JOSD1	JUNB	SOCS3	PLK3	ZFP36	ADAMTS1	HBEGF	ADAMTS4	THBD	CYR61
	PPRC1	EGR3	NDEL1	THBS1	SNAI1	C5AR1	ICAM1	TNFAIP3	MCL1	RNF122	MYADM
	NR4A3	STAT3	KLF6	JUN	ITPKC	SPRY1	IRAK2	DLC1	EMP1	PHF13	NFIL3
	BHLHE40	SOX7	DNAJB5	DUSP1	C15orf39	CCRN4L	CXCL2	SRF	CSRNP1	ELL	EIF4A1
	RND3	CCL2	KLF10	SFPQ	TSC22D2	KDM6B	DUSP6	AMMECR1L	LATS2	EPHA2	CHSY1
	PFKFB3	RELA	CSGALNACT2	JDP2	MIDN	WTAP	LMNA	PHC2	MAFF	CCNL1	ZFP36L1
	MAP2K3	FOS	NFKBID	ATF3	ITPRIP	KLF4	SERPINB8	BCL9L	ARID5A	NCL	TDG
	CDC42SE1	AG2	TAF5L	ZYX	BCL3	NFE2L2	URB2	SLC2A14	FOSB	TUBB6	BAZ1A
	C9orf30	ETS1	PPP1R15B	SLC2A3	PIM1	SEMA4C	DDX3X	PPP1R15A	PIP5K1A	ODF2	RIOK1
	HIVEP2	TNFRSF1A	SFRS7	DDX5	SF1	STK40	DUSP14	RGS2	FOXC2	RHOB	ZC3H12A
	ISG20L2	MESDC1	RELB	GPR4	LYAR	GTPBP4	GPR135	NFKB1	SERTAD1	KCTD11	C12orf68
	SPEN	NUP98	BYSL	EIF2C2	JMJD6	NOLC1	PRPF4	ZBED4	TNFRSF10D	RALGDS	FRMD8
	FOSL1	KBTBD2	BCL6B	SFRS2	RIPK1	CLCF1	ETF1	SIK1	DCUN1D3	CSNK1E	DDX21
	STC1	IPO4	ADM	PELI1	GADD45B	SPRY4	MAT2A	WDR43	NFKBIA	ETS2	HIVEP1

	GFOD2	APOLD1	PHLDA1	RARA	DUSP5	POLR3D	SOX21	ERF	TNFAIP1	SPRY2	NR4A1
	XIRP1	HES1	RBMS1	CHD1	ARHGAP17	CDC42EP4	AKIRIN1	LIF	ITGA5	TRA2B	PHLDA2
	ATXN7L3	PTBP1	IDI2	NFKB2	RASSF1	SFRS15	PER1	TNFRSF10A	CDKN1A	C6orf145	NFATC1
	TRIB1	C9orf21	PLEKHG2	MAFK	C1orf183	HDC	CHIC2	RRAD	TFRC	KLF16	TMEM217
	PDGFB	FAM43A	LONRF3	DOT1L	B3GNT5	EIF4A3	GPR3	C3orf59	FAM107A	NAB2	LRRC42
	AREG										
**Acquired in S2**	PADI4	IL1RN	OSM	TREML2	GPR183	IL1B	FPR2	CH25H	CD300E	IL8	FLNA
	CXCR2	KCTD10	F13A1	APOBEC3A	S100A12	UGT1A7	ABL2	CCL8	CSF3	PLAUR	SKIL
	TNFSF18	S100A9	BCL6	LHX8	GMEB1	C14orf165	CXCR1	GPR97	WWTR1	PLEKHO2	NUPL1
	CSDAP1	MMP10	FCGR3B	CD209	CCL3	TRAF3	IL4R	CORO1C	GLT1D1	FGF10	CFHR1
	SELE	SRGN	HTR6	ETV3	CXCL3	CRISPLD2	STEAP4	LOC84740	SGK1	FPR1	IRS4
	FCRL1	KRT75	GNA12	FAM49A	KIRREL	LIPN	PLEKHG5	PTPRE	MMP8	FAM157B	WEE1
	NRP2	ST3GAL2	ATOH1	CSTT	OPN1LW	OR5V1	RNU4ATAC	SPZ1	TMEM2	TREML3	CYP2W1
	SLC13A5	MX2	TFE3	C14orf43	SLC6A5	RND1	PAGE4	CCL4L2	SH3D20	FAM53C	EGFLAM
	C1orf216	CLEC4D	SSRP1	SCNN1B	MXD1	NAMPT	FADS1	CCL3L1	CD83	MSN	S100A8
	CFHR4	PDE4B	P2RY8	SH2B3	ZSWIM2	LILRA5	EHBP1L1	MS4A6E	AADACL4	LIPI	S100A7
	MRC1	CEACAM4	IL2RA	HNRNPM	ADAM17	ARL4C	EIF4G1	ACTN3	SELL	CASP5	BCORL1
	WDR76	PLXND1	CNR2	RASSF2	PER2	EGR4	C10orf10	GPR27	RQCD1	DCLRE1B	EMILIN2
	DENND5A	MMP12	HMX3	RELT	RASSF3	TMEM39A	PANX1	FCGR2A	BCL2A1	TAC4	ACSM4
	DPYSL2	GFRAL	ODF3L1	RGS9	NIPAL4	GRRP1	HK3	NUP188	FHL2	TREM1	CSRNP2
	PYGL	POLR2D	SCNN1G	TNKS1BP1	SAP130	KIAA1949	YY2	PLEKHM2	IL13RA2	C10orf54	TNFRSF1B
	OTUD3	SLC25A25	CLEC4C	CCL7	LY6G6C	QTRTD1	C13orf33	MAP7D1	IL10	MTCP1	BARD1
	KCTD5	SERTAD2	LOC440563	BGN	CEACAM3	EMR3	CR1	MS4A1	SSH1	SP110	CDK2
	GABRG3	TNFSF11	REL	ELF4	ASAP1	TET3	BICD1	FFAR3	ZNFX1	NFKBIZ	NLRP3
	DNMT1	ULBP2	RNF19B	SEC23A	FILIP1L	LINGO1	RPH3A	MCTP1	TMPRSS4	C19orf75	VNN 3.00
	BTBD19	NOS2	MSH6	INS-IGF2	IL13	HSD3B1	GUCY1A2	CSF2RB	HCK	OAS3	VEZT
	POLD3	CD14	C17orf46	S1PR3	LILRB2	ADAM8	FAM110C	CEP170L	KIF3C	LOC284441	SIGLEC12
	PRPS1	CD97	MYD88	SLFN11	SLC15A4	ZNF597	CEBPD	CMIP	CAMK4	EGR1	CYP2A6

	SHC1	ELMO1	KDM5C	CD69	FOLR2	AFAP1	RNASE2	PLA2G2A	MOBKL2C	VSTM1	FRMD6
	HTR7P1	CYP7A1	EHD3	MLXIP	LRRC33	ARID5B	PPAN-P2RY11	DYNC1I1	SLC25A43	EPHB1	TMEM173
	C13orf18	KIAA1644	SBNO2	DKC1	TRAM2	C11orf24	MLKL	LOC595101	CCL21	CCL19	LOC100126784
	VSIG8	TGM3	PLEKHM1	ACTA1	FBN2	RAI14	FAM20A	STK10	IL7R	LRRFIP1	GYPE
	TRPC4	SLC11A1	ZFPM2	LRRC59	MAP7D2	EHD4	CRKL	FLJ36031	CREG2	ACVR1	GPR176
	SIRPB2	IFFO2	TMEM184B	ACTN1	PHACTR1	ASB10	ARHGEF6	IRAK3	MAML1	MMP25	HK1
	TGFB3	INSL4	CACNA2D1	P2RX1	CCL4	KIAA1244	ELK3	SULF1	RLF	ALOX15	RILPL2
	PTPN1	SLC10A1	GNAI2	MSRB3	FCER2	OSMR	PRIC285	KRT32	MT1B	IGSF21	DCBLD1
	PREX1	SULT1B1	IFNA1	POTEE	CHN1	SMAP2	GNA15	SLC2A1	LOC284688	ACTB	MAPK6
	CDC42EP2	KIAA0146	KALRN	PSMD11	KCNK3	LOC644936	C9orf25	PLB1	CEBPB	ARL5B	CCR7
	IL1R1	DUOXA1	VSX2	FPR3	MT1X	TAS2R40	LOC340017	MAP4K4	KCTD20	EHD2	NONO
	CLSPN	SCN3A	AOAH	MURC	ZEB2	SKI	ARRB1	ADPRH	CENPI	LRRC32	USP36
	SEC24D	KCNA6	TMEM84	TUBGCP3	C6orf150	CSF3R	C5orf58	FIGLA	IL17RA	MYLC2	CLEC4A
	AMPD2	UGT1A8	TUBA1A	TNFRSF13C	DDIT4	H3F3C	TSHZ3	KCNJ5	GORASP2	ARRDC3	HEATR1
	ABCF1	CLASP1	A2M	BLK	HSP90B3P	DAAM2	HCLS1	PIP4K2A	KHNYN	CBWD6	AHDC1
	GBP1	EPB41L2	RNF217	SERPINB9	HNRNPR	UIMC1	TTYH2	VIM	TBC1D25	SH3BP5	SRL
	KERA	FAM38A	CSF1R	TWIST2	PCDHAC2	FCN1	EMR1	MKNK1	PLEK	DLGAP5	CFH
	ARID3B	KDM3A	ZRANB3	CHRM3	CTGF	STAB1	KHSRP	CDV3	CASP7	NKX6-3	SLCO1B1
	CCL13	ARHGEF2	TP53BP2	ADORA2A	NFAM1	ISY1	SAMSN1	SERPINB2	SCN9A	NUP62	ADAM19
	ATP8B2	TRIM6-TRIM34	ZCCHC24	NES	SUPT6H	FAM118A	RAB35	EMR2	TMEM194B	RRP1B	SMC1A
	MMP19	HNRNPA1L2	WDFY2	IDH1	CD300LB	PRPF38A	GBX1	DNAJB8	RNASE1	LAMC3	ZNF496
	CHD4	GRK5	DDR2	FBN1	C1S	EPC1	UROC1	FSTL3	CFLAR	SLC26A10	CXCR5
	FCAR	LOC348840	PRAMEF14	LUZP4	C13orf29	LRP1	LOC283663	FTO	HNRNPF	MKL1	C3AR1
	SLC16A1	SMARCAL1	SAMD8	GNB1	ITGB1BP2	MTF1	COL6A3	CHST11	MGC12916	SETD8	SF3B3
	GRM7	MTA2	TLN1	PABPC3	SENP1	LCP2	C2orf48	RASIP1	LAIR1	FES	GNA13
	NFE2	TRIM9	MAP3K8	CDC45	APLNR	ADAP2	PELO	LOC154761	INSIG2	FUT4	IPMK
	NRP1	CCDC19	MYOF	TCP11L1	UBN1	ELL2	GPATCH2	JUB	GABRP	ZNF643	C1orf55
	SST	PHLDB1	VNN 2.00	DACH2	LZTS1	KCNJ8	DCHS1	RHO	USP37	ARHGAP23	MTHFD2

	CHSY3	SFRS4	TNC	ADAMTS2	VSIG4	PCDHB3	DENND3	RAD51L3	YARS	COL12A1	MKI67
	DLX5	GALC	TMEM185B	JAG1	SIGLEC5	USP18	RASGRP2	CBFA2T3	PIK3R1	ERC1	GPR84
	COTL1	MPEG1	CCL23	TMEM49	C3orf64	PMP22	KLF2	PLEKHG1	CD163	PTRF	SLCO2B1
	SMOX	TGFB1	SMTN	C1R	H6PD	TRPV2	MGAT2	PPM1F	CCDC105	SLIT3	AGFG1
	PDIA5	KPNA2	ITPKB	GPR161	F9	SUN2	FAM38B	ATF7	MYH9	TRIM6	CASP10
	LILRB1	TRIP12	SH3PXD2B	DHH	CFHR2	CFHR5	CD276	LOC100302650	ARID3C	UGDH	LBXCOR1
	OR6C2	OR8G2	TMCO5A	GPLD1	RBM28	C4orf31	FAM55C	ZSWIM4	PCDH12	CD86	UBC
	ZNF281	MEGF10	MSL1	BHLHE41	C9orf47	SNX32	NCF2	SART3	AK3L1	C8orf86	TMCO7
	NPHS1	RBM8A	HSPA14	CLC	CSF1	BMP2K	FGD5	ETV6	RASGRP4	CD4	SLC24A4
	VCL	TERF2	MNDA	MYH2	KLK7	MYOCD	OR13J1	SIGLEC10	MTNR1B	LMCD1	C19orf59
	DNAH12	TBC1D10A	CATSPERB	MS4A4A	KIF23	MAPRE1	PDE2A	COL15A1	STAT4	PDCD1LG2	OR2W3
	LYN	ARHGEF19	MTERFD2	YEATS2	IFNAR2	ESPL1	DIAPH2	DRGX	TNFAIP2	FBXO42	TRAF1
	C1orf198	PARVA	IL24	GRXCR2	DRD3	POLE2	LDOC1L	CXorf21	SLC7A1	TBXAS1	PCDHGB6
	HNRNPA3	DHX38	CD80	ADCY3	IER2	TBC1D2B	SIRPB1	RASD1	RBM17	ZNF423	LMNB1
	ANKLE2	C13orf30	CCR1	C19orf38	LHFPL2	P704P	TANC2	STX11	NDST2	VGLL3	GLIPR2
	P2RY2	C17orf107	R3HDM1	UGGT1	CFP	DNAI2	MEF2D	MFGE8	PDGFRB	EPAS1	LOC257358
	DSE	CREM	CD53	WDR33	PUS7	RNASEH1	EIF2AK3	TECPR1	ACBD3	MATN1	KIAA1210
	ZBTB2	SIGLEC9	TEAD4	UBL4B	CDKN2B	TCF7L1	CHD8	TAS2R31	FBLIM1	CTXN2	SP100
	PIAS4	ORC1L	SPARC	BAZ2A	CDH11	NPPA	NCOR2	YWHAG	PSMD1	LOH3CR2A	SYNJ2
	ATP2B4	CASP1	GBGT1	ITGAM	TRY 6.00	FOXJ2	SH3PXD2A	BIRC3	OR1L6	FCHSD2	PDYN
	PAG1	SGK269	TYMS	EGLN3	PRC1	ARHGAP11B	PDK1	DISC1	DNAJC18	PASK	RRM2
	NNMT	ADAMTS7	HIST1H2BL	IFNA6	TNP1	GLI3	CPSF7	ABL1	NOS3	LILRA1	SLC2A6
	CBX6	GMEB2	PHF19	CD300C	ZNF641	TXLNG	HDLBP	MGC42105	NRBP1	OR4N4	SCD
	KIAA1462	IL4I1	HNRNPH1	ARHGDIB	BEST1	CD93	LRFN1	CHFR	DBH	EIF4H	TRIM5
	TOE1	TNFSF13B	TTLL4	CMKLR1	ARPC2	MC2R	TINF2	THOC5	SPRED3	FAM26E	MYO1F
	C1QC	OLFML2B	MPO	WDR47	RBM15	FAM70B	NYNRIN	MCM3AP	CIC	ATF2	IRF9
	EIF2C3	WDR26	LOC100134259	FCGR2C	PRIMA1	RBMXL1	RPS6KC1	LOC100131551	CCDC116	PLBD2	SERPINE2
	SDC3	KLHL5	FAM102B	SCARNA15	TPM4	ZBTB46	NCRNA00120	LAMA1	PAMR1	UCK2	MYLK

	LOC100130331	TMEM154	SLC1A4	LOC643837	SAMHD1	TRIM47	TACC3	PKD1L1	CYFIP1	DAXX	KIF18A
	CRLF3	FSCN1	ELK4	LGI2	ALS2	ADH1A	C9orf144	IFNA13	CCL11	GATA6	GPR182
	DCTN1	PML	PLD5	ITPRIPL1	PRDM13	F13B	SDS	LCP1	CPB2	THRAP3	ABCB6
	INPP5D	ATP10A	RAB39	LYPD5	COL4A2	HMGB1P1	TMEM109	ITLN2	HAL	KCMF1	AOC3
	RXFP2	FMO3	TMEM22	RXFP1	ZFX	AMICA1	CBLB	TMEM105	SAP30	MTHFR	CD33
	DYSF	FRZB	LDLRAD3	EML1	SNORD1C	FKBP10	PCDHGC3	NLRC4	MS4A6A	F2R	PLK2
	FYN	HULC	GPR124	PDE3A	MARVELD1	CDC42EP3	QSOX2	DAPP1	MYO1G	MMP14	CYBB
	APOL3	NOP56	STIL	LTB4R2	CDC6	SUPT7L	RUFY4	METAP2	PPBP	SEMA4B	KDM5B
	CCR8	GAL3ST4	PRKCB	HSPG2	HPS3	RBM12	TSNAX-DISC1	CLEC2B	CKAP4	OR13G1	GJD3
	FUS	GGCX	EML3	PRTN3	SHROOM4	NECAP2					
**Lost from S2**	JOSD1	PLK3	THBD	NDEL1	SNAI1	C5AR1	PADI4	IL1RN	OSM	TREML2	GPR183
	RNF122	MYADM	CD300E	IL8	FLNA	CXCR2	KCTD10	F13A1	ITPKC	APOBEC3A	DLC1
	UGT1A7	ABL2	CCL8	CSF3	PLAUR	PHF13	SKIL	TNFSF18	S100A9	LHX8	GMEB1
	C14orf165	CXCR1	DNAJB5	GPR97	WWTR1	PLEKHO2	C15orf39	MMP10	FCGR3B	CD209	CCL3
	TRAF3	CXCL2	SRF	CORO1C	FGF10	CFHR1	SELE	EIF4A1	HTR6	ETV3	SFPQ
	CRISPLD2	LOC84740	AMMECR1L	SGK1	FPR1	IRS4	FCRL1	EPHA2	KRT75	GNA12	KIRREL
	LIPN	PLEKHG5	CSGALNACT2	MMP8	FAM157B	JDP2	NRP2	ST3GAL2	ATOH1	CSTT	OPN1LW
	OR5V1	RNU4ATAC	SPZ1	TMEM2	LMNA	PHC2	TREML3	CYP2W1	SLC13A5	MX2	TFE3
	SLC6A5	RND1	CCNL1	PAGE4	CCL4L2	SH3D20	FAM53C	EGFLAM	C1orf216	MAP2K3	NFKBID
	CLEC4D	SCNN1B	FADS1	CCL3L1	CD83	MSN	S100A8	SERPINB8	BCL9L	ARID5A	CFHR4
	PDE4B	TDG	CDC42SE1	P2RY8	SH2B3	ZSWIM2	LILRA5	EHBP1L1	MS4A6E	AADACL4	LIPI
	S100A7	TAF5L	ZYX	MRC1	CEACAM4	IL2RA	HNRNPM	BCL3	ARL4C	EIF4G1	ACTN3
	SELL	CASP5	BCORL1	WDR76	PLXND1	CNR2	RASSF2	PER2	EGR4	GPR27	RQCD1
	DCLRE1B	FOSB	EMILIN2	TUBB6	DENND5A	MMP12	HMX3	RELT	RASSF3	C9orf30	FCGR2A
	BCL2A1	TAC4	ACSM4	DPYSL2	GFRAL	ODF3L1	RGS9	NIPAL4	GRRP1	HK3	NUP188
	TREM1	CSRNP2	SEMA4C	TNKS1BP1	SAP130	KIAA1949	PPP1R15A	YY2	PIP5K1A	PLEKHM2	ODF2
	IL13RA2	C10orf54	TNFRSF1B	OTUD3	SLC25A25	CLEC4C	CCL7	LY6G6C	TNFRSF1A	QTRTD1	C13orf33
	MAP7D1	IL10	DDX5	MTCP1	STK40	DUSP14	RGS2	BARD1	KCTD5	SERTAD2	LOC440563

	BGN	CEACAM3	EMR3	CR1	MS4A1	SSH1	SP110	CDK2	GABRG3	TNFSF11	REL
	ELF4	ASAP1	TET3	BICD1	FFAR3	ZNFX1	NFKBIZ	NLRP3	ZC3H12A	DNMT1	ULBP2
	RNF19B	ISG20L2	SEC23A	FILIP1L	LINGO1	RPH3A	MESDC1	MCTP1	TMPRSS4	C19orf75	VNN 3.00
	RELB	BTBD19	NOS2	MSH6	INS-IGF2	IL13	HSD3B1	GUCY1A2	CSF2RB	HCK	LYAR
	GTPBP4	OAS3	VEZT	POLD3	CD14	C17orf46	LILRB2	ADAM8	FAM110C	CEP170L	KIF3C
	NFKB1	LOC284441	SIGLEC12	PRPS1	CD97	MYD88	SLC15A4	ZNF597	CMIP	KCTD11	CAMK4
	CYP2A6	SHC1	ELMO1	KDM5C	CD69	FOLR2	AFAP1	RNASE2	PLA2G2A	MOBKL2C	C12orf68
	VSTM1	FRMD6	HTR7P1	CYP7A1	EHD3	MLXIP	LRRC33	SPEN	PPAN-P2RY11	DYNC1I1	SLC25A43
	EPHB1	TMEM173	C13orf18	BYSL	KIAA1644	JMJD6	SBNO2	DKC1	TRAM2	C11orf24	MLKL
	LOC595101	CCL21	CCL19	LOC100126784	VSIG8	TGM3	PLEKHM1	ACTA1	FBN2	RAI14	FAM20A
	STK10	ZBED4	IL7R	LRRFIP1	GYPE	TNFRSF10D	TRPC4	SLC11A1	RALGDS	ZFPM2	LRRC59
	MAP7D2	EHD4	FRMD8	CRKL	CREG2	ACVR1	GPR176	SIRPB2	IFFO2	TMEM184B	ACTN1
	BCL6B	PHACTR1	SFRS2	RIPK1	ASB10	ARHGEF6	IRAK3	MAML1	MMP25	HK1	TGFB3
	INSL4	CACNA2D1	P2RX1	CCL4	KIAA1244	ELK3	SULF1	ALOX15	RILPL2	PTPN1	ETF1
	SLC10A1	GNAI2	MSRB3	FCER2	DCUN1D3	CSNK1E	PRIC285	KRT32	MT1B	IGSF21	DCBLD1
	PREX1	IFNA1	POTEE	CHN1	SMAP2	GNA15	LOC284688	ACTB	MAPK6	CDC42EP2	KALRN
	PSMD11	PELI1	KCNK3	LOC644936	C9orf25	PLB1	CEBPB	CCR7	IL1R1	DUOXA1	GADD45B
	VSX2	FPR3	TAS2R40	LOC340017	MAP4K4	KCTD20	SPRY4	EHD2	NONO	CLSPN	MAT2A
	SCN3A	AOAH	MURC	ZEB2	SKI	ARRB1	ADPRH	CENPI	LRRC32	USP36	SEC24D
	KCNA6	TMEM84	TUBGCP3	C6orf150	CSF3R	C5orf58	FIGLA	IL17RA	MYCL2	CLEC4A	AMPD2
	UGT1A8	TUBA1A	TNFRSF13C	DDIT4	TSHZ3	KCNJ5	GORASP2	ARRDC3	HEATR1	ABCF1	CLASP1
	A2M	BLK	DAAM2	HCLS1	PIP4K2A	KHNYN	CBWD6	AHDC1	GBP1	EPB41L2	RNF217
	SERPINB9	HNRNPR	UIMC1	TTYH2	VIM	TBC1D25	SRL	KERA	FAM38A	CSF1R	TWIST2
	HIVEP1	PCDHAC2	FCN1	EMR1	MKNK1	PLEK	DLGAP5	GFOD2	APOLD1	CFH	ARID3B
	KDM3A	ZRANB3	CHRM3	RARA	CTGF	STAB1	KHSRP	CDV3	CASP7	NKX6-3	SLCO1B1
	CCL13	ARHGEF2	TP53BP2	ADORA2A	NFAM1	POLR3D	ISY1	SAMSN1	SERPINB2	SCN9A	NUP62
	ADAM19	ATP8B2	SOX21	TRIM6-TRIM34	ZCCHC24	ERF	TNFAIP1	NES	SUPT6H	FAM118A	RAB35
	EMR2	RRP1B	SMC1A	MMP19	NR4A1	HNRNPA1L2	WDFY2	IDH1	XIRP1	CD300LB	PRPF38A

	GBX1	DNAJB8	RNASE1	LAMC3	ZNF496	CHD4	GRK5	DDR2	FBN1	C1S	EPC1
	UROC1	FSTL3	CFLAR	SLC26A10	CXCR5	LOC348840	PRAMEF14	LUZP4	C13orf29	LRP1	LOC283663
	FTO	MKL1	C3AR1	SLC16A1	SMARCAL1	SAMD8	GNB1	ARHGAP17	ITGB1BP2	MTF1	COL6A3
	CHST11	MGC12916	SETD8	SF3B3	GRM7	MTA2	TLN1	SENP1	LCP2	C2orf48	RASIP1
	LAIR1	FES	NFE2	TRIM9	AKIRIN1	MAP3K8	CDC45	APLNR	ADAP2	PELO	LOC154761
	INSIG2	LIF	FUT4	IPMK	NRP1	CCDC19	MYOF	TCP11L1	UBN1	ELL2	GPATCH2
	GABRP	ZNF643	C1orf55	SST	PHLDB1	VNN 2.00	DACH2	LZTS1	DCHS1	ITGA5	RHO
	ARHGAP23	MTHFD2	CHSY3	SFRS4	TNC	TRA2B	PHLDA2	ADAMTS2	VSIG4	PCDHB3	DENND3
	RAD51L3	YARS	COL12A1	MKI67	DLX5	JAG1	SIGLEC5	ATXN7L3	USP18	RASGRP2	PTBP1
	IDI2	CBFA2T3	NFKB2	PIK3R1	ERC1	GPR84	COTL1	MPEG1	CCL23	C3orf64	PMP22
	KLF2	PLEKHG1	CD163	PTRF	SLCO2B1	SMOX	TGFB1	SMTN	RASSF1	C1R	H6PD
	TRPV2	MGAT2	PPM1F	CCDC105	SFRS15	SLIT3	AGFG1	PDIA5	KPNA2	PER1	ITPKB
	GPR161	F9	SUN2	FAM38B	ATF7	MYH9	TRIM6	CASP10	LILRB1	TRIP12	SH3PXD2B
	DHH	CFHR2	C6orf145	CFHR5	NFATC1	CD276	LOC100302650	ARID3C	UGDH	LBXCOR1	OR6C2
	OR8G2	TMCO5A	GPLD1	RBM28	C4orf31	FAM55C	ZSWIM4	PCDH12	CD86	UBC	ZNF281
	MEGF10	MSL1	BHLHE41	SNX32	NCF2	SART3	AK3L1	C8orf86	TMCO7	NPHS1	RBM8A
	HSPA14	BMP2K	FGD5	ETV6	RASGRP4	CD4	SLC24A4	VCL	TERF2	MNDA	MYH2
	KLK7	MYOCD	OR13J1	PLEKHG2	SIGLEC10	MTNR1B	LMCD1	C19orf59	DNAH12	TBC1D10A	CATSPERB
	MS4A4A	KIF23	MAPRE1	PDE2A	COL15A1	STAT4	MAFK	PDCD1LG2	OR2W3	LYN	ARHGEF19
	MTERFD2	YEATS2	IFNAR2	ESPL1	DIAPH2	DRGX	TNFAIP2	FBXO42	TRAF1	C1orf198	PARVA
	C1orf183	IL24	GRXCR2	DRD3	POLE2	LDOC1L	CXorf21	SLC7A1	TBXAS1	PCDHGB6	HNRNPA3
	DHX38	CD80	HDC	ADCY3	IER2	TBC1D2B	SIRPB1	RASD1	RBM17	ZNF423	LMNB1
	ANKLE2	C13orf30	CCR1	C19orf38	LHFPL2	P704P	TANC2	STX11	NDST2	VGLL3	GLIPR2
	P2RY2	C17orf107	R3HDM1	UGGT1	CFP	DNAI2	MEF2D	MFGE8	PDGFRB	EPAS1	LOC257358
	DSE	CD53	WDR33	RNASEH1	EIF2AK3	TECPR1	RRAD	ACBD3	MATN1	KIAA1210	ZBTB2
	SIGLEC9	TEAD4	UBL4B	CDKN2B	TCF7L1	CHD8	TAS2R31	FBLIM1	CTXN2	SP100	PIAS4
	ORC1L	SPARC	BAZ2A	CDH11	KLF16	NPPA	NCOR2	YWHAG	PSMD1	LOH3CR2A	SYNJ2
	ATP2B4	CASP1	GBGT1	ITGAM	PDGFB	TRY 6.00	FOXJ2	SH3PXD2A	BIRC3	OR1L6	FCHSD2

	PDYN	SGK269	TYMS	PRC1	ARHGAP11B	PDK1	DISC1	DNAJC18	PASK	RRM2	NNMT
	ADAMTS7	FAM43A	HIST1H2BL	IFNA6	TNP1	GLI3	CPSF7	ABL1	NOS3	LILRA1	SLC2A6
	CBX6	GMEB2	PHF19	DOT1L	CD300C	ZNF641	TXLNG	HDLBP	MGC42105	NRBP1	OR4N4
	SCD	KIAA1462	IL4I1	HNRNPH1	ARHGDIB	BEST1	CD93	LRFN1	CHFR	DBH	EIF4H
	TRIM5	TOE1	TNFSF13B	TTLL4	CMKLR1	ARPC2	MC2R	THOC5	SPRED3	FAM26E	MYO1F
	C1QC	OLFML2B	MPO	WDR47	FAM70B	NYNRIN	MCM3AP	CIC	IRF9	GPR3	EIF2C3
	WDR26	LOC100134259	FCGR2C	PRIMA1	RPS6KC1	CCDC116	PLBD2	SERPINE2	SDC3	C3orf59	KLHL5
	FAM102B	SCARNA15	TPM4	ZBTB46	LAMA1	PAMR1	UCK2	MYLK	LOC100130331	TMEM154	SLC1A4
	LOC643837	SAMHD1	TRIM47	TACC3	PKD1L1	CYFIP1	DAXX	KIF18A	CRLF3	FSCN1	ELK4
	LGI2	ALS2	ADH1A	C9orf144	IFNA13	CCL11	GATA6	GPR182	DCTN1	PML	PLD5
	ITPRIPL1	PRDM13	F13B	SDS	LCP1	CPB2	THRAP3	ABCB6	INPP5D	ATP10A	RAB39
	LYPD5	COL4A2	HMGB1P1	TMEM109	ITLN2	HAL	KCMF1	AOC3	RXFP2	FMO3	TMEM22
	RXFP1	ZFX	AMICA1	CBLB	TMEM105	SAP30	MTHFR	CD33	DYSF	FRZB	EML1
	SNORD1C	FKBP10	PCDHGC3	NLRC4	MS4A6A	FYN	HULC	GPR124	PDE3A	MARVELD1	CDC42EP3
	QSOX2	DAPP1	MYO1G	MMP14	CYBB	APOL3	NOP56	STIL	LTB4R2	CDC6	SUPT7L
	RUFY4	NAB2	LRRC42	METAP2	SEMA4B	KDM5B	CCR8	GAL3ST4	PRKCB	HSPG2	TSNAX-DISC1
	CLEC2B	CKAP4	OR13G1	GJD3	FUS	GGCX	EML3	PRTN3	SHROOM4		
**Conserved in S2 and S3**	NFIL3	ZFP36	FOSL2	SLC2A3	SLC2A14	TRIB1	KLF6	ADAMTS4	DUSP1	JUNB	ADAMTS1
	MCL1	RIOK1	ADM	PPRC1	FOSL1	SOCS3	FOS	CSRNP1	DDX21	ZFP36L1	KIAA0146
	CCRN4L	EGR1	WDR43	STEAP4	SIK1	PIM1	NFE2L2	NAMPT	TMEM185B	IRAK2	CEBPD
	LDLRAD3	TINF2	RHOB	EIF2C2	TNFAIP3	FLJ36031	NFKBIA	KBTBD2	EIF4A3	BHLHE40	TNFRSF10A
	KLF10	C9orf21	NUP98	CHSY1	SERTAD1	NR4A3	PLK2	NOLC1	PANX1	TMEM49	ICAM1
	SPRY1	DUSP5	CREM	GALC	AREG	ATF3	BCL6	BAZ1A	TMEM194B	ARL5B	ETS2
	PFKFB3	B3GNT5	PUS7	EGR3	ADAM17	S1PR3	ETS1	PHLDA1	RBMS1	STAT3	CLC
	CSF1	MIDN	STC1	HES1	FOXC2	CDKN1A	WTAP	DDX3X	RBM12	CYR61	LOC100131551
	IPO4	SULT1B1	KCNJ8	SLFN11	ELL	HNRNPF	POLR2D	HBEGF	TMEM217	CHIC2	FAM49A
	TMEM39A	JUN	HPS3	RELA	WEE1	AG2	C14orf43	GNA13	FAM107A	USP37	DUSP6
	ARID5B	MXD1	EMP1	CSDAP1	MT1X	ITPRIP	HIVEP2	CDC42EP4	GPR4	LONRF3	CCL2

	OSMR	PABPC3	KLF4	TSC22D2	PTPRE	SRGN	PPBP	RBM15	SPRY2	PPP1R15B	RND3
	PYGL	PRPF4	F2R	GLT1D1	SLC2A1	EGLN3	H3F3C	FHL2	THBS1	TFRC	RBMXL1
	C9orf47	JUB	NCRNA00120	SOX7	CXCL3	ATF2	KDM6B	C10orf10	IL4R	URB2	SCNN1G
	S100A12	SH3BP5	FCAR	HSP90B3P	CH25H	PAG1	SFRS7	IL1B	NUPL1	RLF	FPR2
	NCL	CHD1	LATS2	SF1	CLCF1	SSRP1	GPR135	MAFF			
**Acquired in S3**	PNRC1	NCOA7	UGCG	SFRS13B	EREG	SNAPC1	NUDCD1	ZNF460	EDIL3	ADAMTS9	WDYHV1
	CHMP1B	YRDC	PTP4A1	DKFZP434K028	C7orf68	SEH1L	PATL1	EIF3E	PAK1IP1	RNASE4	CLDND1
	P2RY14	SERPINE1	C10orf119	TAF1D	CALCR	EIF1B	RNF139	RPL7	IL1RAP	IL1RL2	UBIAD1
	MED30	MIPOL1	NUP153	TFPI	EIF3H	ORM1	RPS6KA5	NKX3-1	LRP12	NT5E	TAF4B
	MMRN1	TMED10	EIF2S1	CA1	C18orf20	PLSCR4	MIR17HG	ING1	CCL20	RBM7	ALG9
	C18orf19	ZNF143	RPS12	SLC16A2	PLAC8	YWHAZ	ZFAND1	LOC100133669	PCDHGB2	ERO1L	CCT2
	GPC6	HBD	CNN3	FTHL3	DKFZP434H168	HSPA5	SNORA56	GNL3	LDHAL6B	SEL1L	NUP35
	NMD3	RPS3A	SFRS3	RPS29	NETO2	DCP1A	XKR9	PNP	HIF1A	SLC43A3	COX8C
	NAF1	ARL6IP6	CATSPERG	TIMP4	GCH1	PDIA6	LOC100127888	PM20D2	DIO3OS	H3F3B	GEM
	ZHX2	TMEM43	CNIH	ACVR1C	ST8SIA6	VDAC2	TOX4	PPM1D	C14orf118	NIP7	TATDN1
	SYNCRIP	PCDHGB1	SAV1	NXT1	ACTL6A	MALT1	TIMM9	HRH1	PTPN12	POU4F2	EXOC6
	ZBTB25	TGFA	AHSP	MARS2	RPS13	MCFD2	C6orf25	HSPD1	PNO1	UBA2	SENP2
	HBB	SIRPD	EXT2	HBM	LAPTM4B	HSD17B7	RPL30	LOC729020	TMEM209	SUPT3H	TBPL1
	RRM2B	TAF2	GLIPR1L2	ASNSD1	HMGN4	TM9SF1	PCNP	SLC25A32	MTUS1	ALAS2	TMEM67
	SNX31	IER3	TTC35	PPP1R2P3	RPL10A	HIPK3	FYTTD1	IPO5	ZNF295	APEX1	CUL4B
	TMEM68	ATP13A3	SAMD4A	FAM99B	SIRT1	UAP1	STAG1	PPP2R1B	FMO2	TCEA1	SLC22A3
	TGM2	ZNF649	GATA1	ACTR10	UTP18	SPRED2	AKAP14	CD109	HNRNPK	SLC39A6	NSMAF
	MRPL33	ANG	NUP160	PSEN1	ARMC10	LOC221710	NHEDC2	RHAG	EIF3J	FMN2	HNRNPC
	ALKBH1	NIPAL1	RAD21	NUAK2	SMNDC1	ABCC9	CGRRF1	LMAN1	SNW1	PABPC1	NFYA
	WDR3	NR4A2	YY1	PALM2	GPD2	ARIH1	TCP1	MGC16025	CXCR7	KIAA0196	RAB2A
	TMEM50B	CSE1L	TBCCD1	PRMT5	GOLGA5	NARS2	CPE	DERL1	C2orf49	TMEM74	EBAG9
	ZNF331	E2F5	MED17	HEMGN	ZC3H15	CCNI	LDLR	TEX10	OBFC2A	MT1L	TES
	EPB42	ZNF830	CNBP	CCT4	GABPB1	NGDN	ZBTB11	HSP90B1	MSLNL	TRMT5	C2CD4B

	EXD2	OR2L13	JHDM1D	SLC39A9	IFITM2	INTS4L2	NIPAL2	GOLGA6A	TC2N	ZNF484	MID2
	TPP1	DICER1	ANKRD46	RCOR1	TXNDC16	EPHA8	JKAMP	ZNF706	BAG5	NGLY1	MTDH
	MT1DP	PSMC6	AKAP2	PUS3	NOG	EIF1	VAPA	LOC100130264	MAPK1IP1L	STT3B	EAF1
	ZCCHC2	ZC3H14	ZNF410	DAD1	C20orf199	WIT1	ABCE1	S1PR1	KIAA0020	TTC9C	DPAGT1
	INSIG1	C14orf135	PPIL4	CAV1	ERRFI1						
**Lost from S3**	PNRC1	UGCG	ADAMTS4	FOSL1	EREG	SNAPC1	ZNF460	EDIL3	ADAMTS9	KIAA0146	WDYHV1
	STEAP4	SIK1	CHMP1B	YRDC	PIM1	PTP4A1	NFE2L2	NAMPT	TMEM185B	DKFZP434K028	C7orf68
	SEH1L	LDLRAD3	TINF2	PATL1	EIF3E	TNFAIP3	FLJ36031	PAK1IP1	EIF4A3	RNASE4	CLDND1
	P2RY14	SERPINE1	TAF1D	CALCR	CHSY1	SERTAD1	PLK2	EIF1B	RNF139	IL1RAP	PANX1
	IL1RL2	ICAM1	UBIAD1	MED30	CREM	GALC	AREG	MIPOL1	TFPI	BCL6	EIF3H
	BAZ1A	TMEM194B	ORM1	NKX3-1	LRP12	ARL5B	NT5E	MMRN1	PUS7	TMED10	EGR3
	ADAM17	EIF2S1	S1PR3	CA1	C18orf20	PLSCR4	CCL20	RBM7	ALG9	RBMS1	C18orf19
	STAT3	RPS12	CLC	CSF1	SLC16A2	PLAC8	YWHAZ	LOC100133669	PCDHGB2	CDKN1A	ERO1L
	CCT2	GPC6	HBD	CNN3	FTHL3	DKFZP434H168	HSPA5	SNORA56	WTAP	GNL3	RBM12
	LDHAL6B	SEL1L	NUP35	NMD3	CYR61	LOC100131551	RPS3A	RPS29	NETO2	DCP1A	IPO4
	XKR9	PNP	HIF1A	SLC43A3	SULT1B1	KCNJ8	ELL	COX8C	HNRNPF	CATSPERG	TIMP4
	GCH1	PDIA6	POLR2D	LOC100127888	PM20D2	DIO3OS	GEM	ZHX2	TMEM43	TMEM217	CNIH
	ACVR1C	CHIC2	ST8SIA6	FAM49A	VDAC2	TOX4	PPM1D	C14orf118	NIP7	TMEM39A	TATDN1
	SYNCRIP	PCDHGB1	SAV1	NXT1	HPS3	ACTL6A	RELA	MALT1	TIMM9	HRH1	POU4F2
	EXOC6	ZBTB25	TGFA	AG2	AHSP	FAM107A	USP37	MARS2	RPS13	MCFD2	C6orf25
	HSPD1	PNO1	SENP2	HBB	SIRPD	EXT2	ARID5B	HBM	LAPTM4B	HSD17B7	MXD1
	RPL30	CSDAP1	LOC729020	TMEM209	MT1X	SUPT3H	TBPL1	RRM2B	TAF2	GLIPR1L2	HIVEP2
	ASNSD1	HMGN4	TM9SF1	PCNP	SLC25A32	MTUS1	ALAS2	OSMR	TMEM67	PABPC3	SNX31
	IER3	TTC35	PPP1R2P3	RPL10A	HIPK3	FYTTD1	IPO5	APEX1	CUL4B	TMEM68	ATP13A3
	SAMD4A	FAM99B	TSC22D2	UAP1	STAG1	PPP2R1B	FMO2	TCEA1	SRGN	PPBP	RBM15
	SLC22A3	TGM2	ZNF649	GATA1	ACTR10	UTP18	PPP1R15B	AKAP14	CD109	SLC39A6	NSMAF
	MRPL33	ANG	RND3	NUP160	PSEN1	ARMC10	LOC221710	NHEDC2	RHAG	EIF3J	PYGL
	PRPF4	FMN2	F2R	GLT1D1	HNRNPC	SLC2A1	ALKBH1	NIPAL1	RAD21	NUAK2	ABCC9

	EGLN3	CGRRF1	LMAN1	H3F3C	SNW1	PABPC1	FHL2	WDR3	THBS1	YY1	TFRC
	PALM2	GPD2	ARIH1	RBMXL1	JUB	TCP1	MGC16025	KIAA0196	RAB2A	TMEM50B	CXCL3
	ATF2	CSE1L	PRMT5	GOLGA5	NARS2	CPE	DERL1	C2orf49	TMEM74	EBAG9	ZNF331
	E2F5	MED17	SCNN1G	S100A12	HEMGN	ZC3H15	CCNI	LDLR	OBFC2A	SH3BP5	MT1L
	TES	EPB42	FCAR	HSP90B3P	CCT4	GABPB1	CH25H	NGDN	PAG1	ZBTB11	HSP90B1
	MSLNL	TRMT5	C2CD4B	EXD2	OR2L13	JHDM1D	SLC39A9	IFITM2	INTS4L2	IL1B	NIPAL2
	NUPL1	GOLGA6A	FPR2	TC2N	MID2	NCL	TPP1	DICER1	ANKRD46	TXNDC16	EPHA8
	JKAMP	ZNF706	BAG5	NGLY1	MTDH	MT1DP	PSMC6	PUS3	NOG	EIF1	VAPA
	LOC100130264	MAPK1IP1L	STT3B	EAF1	ZC3H14	ZNF410	CLCF1	DAD1	C20orf199	WIT1	SSRP1
	ABCE1	KIAA0020	TTC9C	DPAGT1	INSIG1	C14orf135	PPIL4	CAV1	ERRFI1		
**Conserved in S3 and S4**	KLF6	JUNB	KLF4	DUSP6	DUSP1	MCL1	PPRC1	MAFF	NFIL3	ZFP36	FOSL2
	WDR43	ITPRIP	DDX21	SF1	RIOK1	CHD1	SOCS3	HES1	HBEGF	C9orf21	STC1
	B3GNT5	SPRY1	ETS1	MIDN	SPRED2	CSRNP1	SOX7	BHLHE40	SLC2A14	SLC2A3	KLF10
	C10orf119	KDM6B	EMP1	NOLC1	DDX3X	SFRS3	PTPN12	NAF1	ADM	CCRN4L	KBTBD2
	ZFAND1	RCOR1	PFKFB3	DUSP5	URB2	NR4A2	FOXC2	TNFRSF10A	ATF3	S1PR1	EIF2C2
	C10orf10	H3F3B	RPS6KA5	ZNF143	SFRS7	GPR135	SFRS13B	LONRF3	SPRY2	TRIB1	GPR4
	NCRNA00120	AKAP2	TMEM49	ETS2	CNBP	ZNF295	TAF4B	RHOB	NFKBIA	WEE1	FOS
	PHLDA1	JUN	CEBPD	IL4R	ZCCHC2	MIR17HG	HNRNPK	UBA2	ADAMTS1	TEX10	NUP98
	ARL6IP6	ZFP36L1	CXCR7	SLFN11	RPL7	EGR1	NR4A3	ZNF484	SIRT1	TBCCD1	CCL2
	IRAK2	C9orf47	RLF	LATS2	CDC42EP4	ZNF830	NCOA7	GNA13	NUP153	ING1	PTPRE
	C14orf43	NUDCD1	SMNDC1	NFYA							
**Acquired in S4**	HNRPDL	HNRNPH3	PPP1R15A	TTLL11	DDX5	MTERFD2	LOC100302650	DDX42	C17orf46	OR56B4	CDK17
	NAB1	GTPBP4	CAMSAP1	JMJD6	SH2B3	RPGR	HNRNPA3	ARRDC3	RBMX	EFNB2	ELK3
	FOSB	EPAS1	PEAR1	MAT2A	PCDHA7	USP36	NOC3L	SCN4B	HNRNPH1	SFPQ	ZNF833
	GRIK3	TGFBR2	KCNE4	PIM3	LRIG2	SPRY4	EPN2	LRRC70	LOC440354	ZNF343	RPS6KB1
	EXOSC2	F2RL3	SNRK	EPC2	LOC641367	RARA	DLL4	HMGXB4	INTS2	KLF9	ECD
	NDEL1	GPR3	DNTTIP2	LOC286094	MLLT10	RAPGEF1	TMC7	PRDM10	C16orf52	SH3TC2	C11orf94
	RUFY2	ANKRD40	TMEM22	GRB10	ZNF462	GALNTL6	PCDHB4	NFX1	SEMA4C	KCNE3	HYMAI

	PPARGC1B	LMNA	TM4SF1	PPP2R2A	DEFB118	NEK5	TUBGCP3	STARD13	LOC344595	TAF1	ZNF263
	CCDC46	PACRGL	INVS	DHX15	CST11	ARAP3	TAF5L	CPSF6	ORC2L	VIPR1	RGS9
	MDGA2	TIAL1	AMMECR1L	GPATCH2	ABL2	SPAG9	NUP85	KLF7	VPS13B	APOLD1	PMS1
	DLC1	METAP2	AP3M2	AATK	IRF2BP2	ZBTB49	AGTR1	TCTE1	CWC22	OTUD4	THUMPD2
	SFRS1	HNRNPD	FGFR1OP2	RND1	CDNF	KIAA0355	ZNF507	MAK16	HTR7P1	OTUD3	LOC338758
	IL1RAPL1	B3GALT1	OR2H2	LYAR	RFX3	BDP1	RAPGEF5	LOC100129716	PAPL	FAM160A2	RABGAP1
	FRMD8	BAGE2	C9orf93	CHML	CEP72	DDX50	POLR3D	DGKD	POLR1B	UIMC1	PCDH17
	ZNF7	TXLNG	C3orf59	TAF5	JOSD1	SAMD8	ARHGAP29	ASAP1	MAP2K3	SLC35F1	SFRS2
	ABI2	C10orf78	BCL6B	LDB2	ZNF25	CEP68	PKD2L2	ZMYM5	LPL	FRZB	ZNF202
	TRA2B	SNORD1C	C1orf55	GGNBP2	EPC1	MAFG	SMC5	SLC26A10	CXorf36	PRPF38A	TMOD2
	PGLYRP1	MCM3APAS	OTUD6B	DUSP12	JMJD1C	CLEC1A	RFX8	ABCG2	RGS5	RAPH1	SDHAP2
	ISG20L2	DOCK9	NOP14	C9orf144B	WDR20	CDKN2B	PGAP1	ANKRD36B	ZNF496	NRP1	INTS6
	ANKAR	ATG12	DIAPH2	IRGC	GFOD2	SMURF2	MYADM	C20orf194	CSGALNACT2	ZEB1	THRAP3
	LOC100268168	PHF3	SHROOM4	GMEB1	NCRNA00169	SPRED3	CCDC55	ZNF333	RP1L1	SBF2	KIAA1328
	SWAP70	ANO2	DHX38	FLT1	TLK2	SUGT1P1	RFX2	HMGCLL1	SEC14L1	MYCT1	LOC652276
	FFAR2	LOC595101	THBD	MAP4K4	PDE9A	C1orf107	C18orf2	PIWIL2	SHE	MYO1B	EPGN
	BMX	TAF1L	MAP1D	MCF2L	SMC6	GADD45B	C9orf72	FBXO11	PDE4B	BBS7	PLEKHG1
	PIBF1	C2orf86	PLXNA2	SECISBP2	LILRB5	GDAP1	KCNAB1	PLK3	VEZF1	NOM1	C20orf112
	ARID4A	COIL	PNN	POLG2	HCFC2	FBXO48	FAM122A	ZCCHC6	CCNB3	C2orf16	SNORD89
	SRGAP2	PITPNC1	YES1	AHCTF1	KLHL10	SP3	WAC	STK40	SPIRE1	DIS3	ADAMTS5
	PRTFDC1	OR10K2	SLED1	ATAD2B	FRMD3	FUBP1	DCT	TMEM150C	LOC100271836	SF3B1	SMU1
	TACR1	C11orf20	HNRNPM	NAA25	SCARF1	EPHA2	PCDHGA1	HNRNPU	SPARCL1	STAT5B	FLT4
	PGM5P2	IER2	SPEN	NOP58	TMEM146	SNHG4	DENND3	ZNF500	TASP1	MYSM1	VEGFA
	PKD1L1	ANKRD31	TRPM6	INHBB	C9orf150	DOT1L	SPATA3	PSMD12	ATL2	QTRTD1	PHOSPHO2
	ATP6V1G2	ST6GALNAC1	EIF4A1	OR52J3	MAP3K13	SLC10A6	SGK1	UTP6	MTPAP	ERF	ATP1B2
	MORC3	AEBP2	CCDC83	OR51I2	REV1	ZNF207	GEMIN4	NOTCH4	SART3	JAG1	POLR3E
	GAGE2A	ACAP2	SMTN	APBB2	CSF3	LOC221122	RIC8B	FAM119B	C9orf25	TNFAIP8L1	TARDBP
	IRAK3	BMS1	PALMD	KDM4C	PGM2L1	OR8H1	C18orf25	GABPA	PLVAP	VENTXP7	POLR1E

	PCM1	SV2C	PRPF4B	DENND4C	LHFPL4	C12orf76	C15orf39	B4GALNT1	MSL1	HERC4	GIT2
	DAND5	GPCPD1	ZDHHC17	CFLAR	PPP3CC	KHDRBS1	SERPINB5	KPNA5	CSNK1E	LOC641298	RNF160
	CXCL2	TPO	CCDC9	HDX	SRL	DOCK6	PCDH12	ETV3	IGSF9B	C3orf50	HNRNPA3P1

D) List of lost, conserved and acquired genes across stages in KICH

	AATF	ATP8B2	B2M	KCTD4	KIAA1683	KLHL30	LAIR1	PAPLN	RNASE6	SKI
	KBTBD5	ITIH1	CRLF2	PTTG2	SOX21	FAM138B	ACER3	C1orf126	CD101	CTTN
	RALGDS	RAPGEF2	ZNF134	C6orf222	CASQ1	KIAA0922	LSM 12.00	NAPSA	NPDC1	PCYOX1L
	PROK2	PSTPIP1	PYGM	STK17A	UTF1	ABL1	ARRDC4	GPRC5A	IMPDH2	NOS2
	PARP9	TXLNB	UPK2	SYNPR	BEND3	BIK	CELF4	CHD7	CORO7	LRRC3
	SLC1A4	N4BP1	UTP6	FSD2	SCRT2	TAS2R60	BCAN	C1orf9	CWC25	ECEL1
	EMB	MAML1	PQLC2	WDR69	YWHAB	LOC153910	APOC1	CHML	CLN6	CSF1R
	GALNT7	GBP5	NCF1B	SAMD9L	SLC1A3	CSDAP1	CXCR2	GFRA2	HNRNPA3	HUNK
	KIF7	VASH2	FAM74A4	GRM2	MAN2B2	TREM2	ADRBK2	CELSR3	GRWD1	MBD6
	PTGIR	CFI	DPY19L1	LOC100128542	NKD2	TMEM43	TRIM21	ABCC3	BRCA1	BRD1
	CCRL2	GPR114	LRRC8B	MOV10	PMM2	USP38	POU4F3	PLAC1	BIN2	CBL
**Lost. From normal**	FEM1B	GDF5	MORC4	ZNF146	FCRL1	C7orf57	CCDC86	EMR1	IRF6	ISLR2
	JAK3	JPH1	PCSK1	PPDPF	PRKCSH	CSF2	PRAMEF8	FGF19	CD247	PSMD7
	RASSF6	SEPN1	SLC26A2	SLCO2B1	SOD2	WBP11	ZNF395	SNORA76	GKN1	CAPZB
	CARNS1	CENPA	GZMB	LIMK1	LOC100126784	PCDHB2	PSMB2	SEC16A	SORBS2	DKFZp566F0947
	TMEM14E	DOK3	FAM177A1	FZD8	HMGB3	KIF14	LRRN4	STX5	TCL1A	GJC2
	GPR56	IER5	RNF2	SLC35C1	TPSB2	SNTN	EEPD1	FAM110B	GCLM	IRF7
	KDM5A	SH2D3C	TRPM2	WFDC1	MS4A6E	CSNK1D	FAM72D	SLC30A2	TM4SF4	C12orf70
	APBB1	MKNK1	SIDT2	TMEM22	TOP1P1	ZNF469	C2orf39	C6orf154	DNAJB13	HLA-F
	RNF40	FAM177B	OR2H1	FEV	CCL17	BEX1	CD99	DMPK	FCGR1C	HPX
	IL6ST	LRRC41	NOTCH2	OMD	PA2G4	PNO1	SOBP	TMEM57	DFNB31	FAM159A
	FAM49A	HSPA14	LARP1B	PITPNM1	IRGM	ADCYAP1	CGA	COL6A3	FAM179A	PYCR1
	SLC22A17	TPSAB1	RPL23AP32	FKBP1A	GPC4	HAS2AS	PDZD8	PRPF38A	PRDM13	SNORD15B

	TUBA3E	LY6E	MORF4L2	MVP	OTUD7B	TMEM98	WIPF1	ATP6V0A1	CDC37L1	COL17A1
	JPH2	KRT80	MAP1LC3A	TXLNA	UBP1	ZAR1L	C19orf76	C6orf62	CCDC135	CKAP4
	FOXO3B	HMGA2	RNASEH1	C4orf6	IL1F7	EMILIN1	FLJ23867	IGFBP4	PM20D2	SELL
	STAT6	TBX15	ZCCHC6	OTOF	ADAP2	ARHGDIB	DDA1	IMPDH1	SF3A1	ZNF828
	ADORA3	PLXDC2	TRAPPC2P1	IL22RA2	BICD1	HNRNPA1	KLHL25	MAN2B1	PLA2G4C	TPM2
	GABRR3	NKX2-6	RNASE8	SNORA46	FMR1	LY86	NPTX2	SLC24A3	LIPN	EFEMP1
	HR	PDIA4	SNORA67	B4GALNT1	DYNC1LI1	GHRL	MX1	PIGR	XPNPEP1	TEX19
	PRSS21	ARMC3	ASGR1	DULLARD	IL10RA	MC1R	NRP1	PROCR	RLF	RNASE2
	RUNX3	UNC13A	FLJ43950	ORM2	ARRB2	FKBP1AP1	GLI2	ITGA7	LOC100134259	PPP2R2A
	PSMD11	CPLX3	FLJ39609	DTNA	ECE1	FNDC1	NCF4	NRXN2	PDGFA	POLR3C
	GPR142	CLEC4C	CEP55	EIF4G1	FBXO46	GLB1L	IL32	MFGE8	SCARNA16	CD37
	ETV4	GPR4	HAVCR1	LAMA3	PABPC4	PLXNB3	TLK2	UBAP2L	TREML4	KREMEN2
	C15orf48	CD8A	CDCP1	DYNLL1	NLGN1	PDE12	SDK1	SEC31A	ZC3H3	SLAMF9
	C6	ECM1	RC3H1	TREML1	DENND5A	ENTPD2	GBP2	KIAA0355	MGC16121	TECTA
	SIRPG	CEP170L	GNB1	IGSF21	IL27RA	KLHL18	SAMD14	SIPA1L2	TUBA8	HUS1B
	HTR6	ATP13A3	CXorf22	CYP21A2	LDLRAD3	PAPD7	TNKS2	VNN 3.00	AIM2	DEFB109P1B
	NRIP3	SGTB	ZFP28	APOC4	CPA2	CRIP1	DDX60L	EXT1	KLHDC7B	STAT5A
	MYO1A	EXOSC9	HLA-DOB	TAL1	KRT5	FFAR3	ALOX5AP	CRLF1	EMID1	TOE1
	KPNA7	RAX	GIT2	ITGA2	ITIH3	KATNA1	TEP1	BAHD1	C16orf52	CAPZA1
	GPR176	LST1	PRKD2	QSOX1	RGS4	SDC3	APPBP2	FAS	FIBIN	GPR172A
	ITM2C	KIF19	LDOC1L	MICB	ZNF516	ZNF800	CD207	ARHGAP10	C2CD4B	FCGR3B
	RBM15	TXNDC5	ZNF513	C3	FAM23A	HGFAC	HTR7	NOP56	NR4A1	PARVG
	RAB36	SIGLEC16	STC2	REG1A	MYL2	C2orf55	FRMD8	KIF18B	MPZL2	NHS
	PRAM1	SULF2	NRBP1	NUP188	SMPDL3B	ZHX2	DEFB103B	GMFG	MALT1	MAP3K11
	BMP1	CLEC4E	KLF3	PDZD4	RAC2	CXCL10	FAM105B	LILRA1	MNDA	PKDCC
	NKAIN1	PLA2G2D	ABHD11	CDK5R1	ELMO1	TTN	CD44	CDH3	DAZAP1	MANF
	TTC7A	ARHGAP31	LILRB1	MGAT5B	NRN1L	SIAH2	SLC15A3	KLF14	CASKIN2	GPR37L1
	IL13RA1	STAC3	TNKS1BP1	WDR1	HCK	RBPMS	ALAS1	MEGF11	MYO1E	PTPRG

	TSSC4	UHRF1	PI3	CDCP2	FGA	KIAA0182	PLOD3	RIMKLB	SPRED3	CSNK2A2
	LOC606724	SERTAD3	EFNA2	SP8	ADM	C19orf21	HCST	TGIF1	TYROBP	CTLA4
	ARHGEF38	PPP1R10	TMEM149	KDSR	KIFAP3	STIP1	LOC100129066	TREML2	FAM129C	HERPUD2
	KHDRBS1	MYO1G	ZNFX1	TRPM8	C9orf71	OTUD5	PDE4D	SLC6A20	SMARCD1	UBE2R2
	CA9	DACT1	ELOVL2	IQGAP3	SLC35C2	TNIK	FOXI3	CHST3	FCN1	FHAD1
	RPH3A	TNFRSF13C	ARL5B	CPSF7	RNF145	VWA1	ZNF263	ADD2	AFAP1	C3orf59
	KIAA1045	PCDH17	PTAFR	S1PR5	ADAMTS8	FAM26F	HLA-DMA	KCNMB1	SLC34A2	SWAP70
	BLK	CCDC88A	COL18A1	CRELD2	CTSG	MGC45800	MTNR1A	PARVB	YARS	GNAI2
	OS9	POLR2D	RBM12	RUNX1	ETV3L	EFEMP2	FHL1	LMBR1L	STAT1	STK35
	ZBTB10	CCR8	B4GALT4	FSCN1	NLRP12	EGR4	S100P	CLEC5A	FKBP10	IRF9
	PPP1CB	RASGRP4	TP53INP2	ZNF622	NKX6-1	FCRLA	C1QL2	AHR	CDH6	INHBE
	NT5DC3	SEMA4C	TLR9	TMEM86B	TSHZ3	SNORA74A	CTSD	GFAP	GOLGA7B	IFITM1
	MYO7B	SLC1A5	TNFRSF21	CDC5L	CTPS	PLEKHA1	SDPR	TMEM151A	TULP3	ADAM17
	NCKAP1L	TCEAL5	DKFZp686O24166	FAM72B	FLJ16779	GNG4	PHF20L1	SPAG6	UTP14A	WDR63
	ANO9	CYBA	FST	GNB2	ITGB1	MYD88	SCEL	HSF5	CCL8	CD276
	HLA-H	NDC80	ROR2	SIGLEC9	SLC3A2	TOX4	XBP1	ZBTB16	F3	FYTTD1
	MYO1F	PDE4B	SH3RF3	URGCP	CAPN11	NAPSB	NAV2	SOX4	SPOCD1	FAM189A2
	FAM46A	PLEKHN1	PRSS23	LOC100130386	CEACAM4	DGKD	GARS	NLRP3	NUDT11	SLC7A5P2
	SP2	C1QA	CYTH2	MAGED4B	RAP1B	GPSM3	NLGN2	TMEM155	C22orf31	GJB2
	KLF9	SLC39A14	STMN3	ZACN	HEMGN	FABP2	ALPK3	EDEM1	RPN2	TPM3
	CD83	CKS2	PRDM4	ZDHHC22	PTCRA	CSNK1A1	CXCL2	DPEP2	FCGR1A	HLA-DPB1
	HSPA8	PGBD1	SCUBE1	ADAM8	CD300LF	MFRP	MFSD2B	OSGIN1	UNC13D	BCL9
	IFNGR2	NCL	NFKBID	SLC27A3	TES	TMEM39A	IGFBPL1	BIRC7	C10orf4	C1QTNF6
	CXCR1	UCN	USP42	OR2B11	C2orf18	FBXO45	FOXO3	RAB8A	ARX	PABPN1
	SIRT1	TRIM67	WNT9A	CDK12	CHI3L2	CNIH2	EPSTI1	FFAR2	MOBKL2C	RHBDF1
	SP110	TAP2	TEAD4	DMRT1	CYP24A1	HLA-DRB6	SIDT1	SLCO2A1	SOCS6	ZNF365
	NLRC5	REL	PGLYRP1	FKBP11	LAMC2	SERPINB1	ADAM20	PRPF38B	SELPLG	SPIB
	CLDN9	FGD2	KPNB1	NCOA5	RASA4	TDG	ZBTB8A	EMR3	BATF	C13orf31

	EDN2	FAM5B	APOL1	CD300A	FNDC4	GMEB1	LPGAT1	RASEF	RETN	CTNND1
	MMP2	SIGLEC5	VANGL2	CDK2AP2	FAM107A	KCNK7	NOP16	PDP1	FCGR2A	G6PD
	HPSE	IFITM2	MAGED4	PIK3R5	PPP2R1A	TRIM39	YTHDF2	PSMD12	SF1	TMEM39B
	OSBPL10	ROR1	ARL 9.00	MS4A7	CPA5	APAF1	CST3	MYH9	TNFRSF11A	RPE65
	CXCL5	DOCK2	DYRK2	MICAL1	NIPAL4	PLCH1	DNAJC5B	KCTD20	UAP1	ZMYND15
	ABCB4	ELL	GPR153	HYOU1	ROPN1L	VASN	GPA33	C6orf174	KRT86	LILRA4
	CD63	CECR6	PER2	RAB32	RIPK1	FAM160A2	TCP11L1	ZNF653	VAX1	ATXN7
	FBXO28	MGEA5	OTUD4	RASA3	CCL23	LRP10	PRPF4	IL31RA	C22orf34	EPPK1
	PHLDA2	UNC5CL	COL6A1	DUS3L	HAS2	KALRN	ADAMDEC1	CCNL1	LTC4S	PHC2
	IER3	KRT17	SLC18A2	TAGLN2	ZFP57	BMP2K	C3orf36	HRH2	MYO5A	PLA2G7
	RAPGEF5	WTAP	OR1F2P	HLA-E	PLEKHG1	SH2D5	CSF2RA	PAPD5	SLC10A3	TLR4
	ARL13B	BZW1	C10orf46	HIVEP1	KIAA0247	LRRFIP2	MCM6	NFAM1	PHF17	RCE1
	SSC5D	C12orf5	DNAJA1	MAPK7	RSPH4A	SAR1A	SV2C	SLCO5A1	C21orf63	GPC1
	KITLG	WFDC2	BRPF1	DCUN1D5	DIAPH1	GATAD2A	GTF2E2	IWS1	KIAA0020	BMP15
	ASB11	ANKRD53	CSPG5	E2F6	SERPING1	YES1	PADI4	DMRT3	ALOX15B	E2F3
	FNDC3A	IL6R	KCTD11	LOC100302650	MDM2	NINJ2	RNF4	RPF2	S100A9	C9orf140
	LUZP1	SPRY2	DENND3	ENTPD7	SYTL3	MUC12	C1QB	KAAG1	KCNJ14	LAMA2
	LTF	RPS6KL1	ARPC5	CYB5R3	GNA15	HLA-DQB1	HNRNPD	ODF3B	ZFYVE27	ACTC1
	FCER1G	RAB31	DKFZP586I1420	HDC	INTS6	KIAA0802	RNF31	SLC16A3	TBXAS1	ORM1
	CHSY3	LYVE1	SPG20	ZFAND5	DONSON	EPN2	NCRNA00164	ANKRD50	INMT	TMSB10
	TRIM26	VPREB3	KRT75	C12orf77	AMOTL1	C6orf118	HKDC1	KCMF1	KIAA1199	NCF1
	S100A8	SNAPC2	LILRP2	PHLPP2	PTPN9	SERPINA3	SH3TC1	PARP8	CMTM2	DARC
	GEM	GGT5	ICOSLG	IPCEF1	PIP4K2A	COL4A2	LIPG	TMEM158	C4orf51	CCDC50
	HGSNAT	KLF5	KPNA4	NCF2	PAPPA	PDCD1	RLTPR	DEFA1B	ADNP2	CSNK1E
	MMP14	SNIP1	SUN2	TBCCD1	ATP2A2	MT1X	SHISA2	DNALI1	SH2B2	UBE2D1
	ZFP36L2	CCL20	CDR2L	CLIC1	ITGAM	MAPK6	RFX2	PPAN	REPS1	VASP
	YY1AP1	C11orf21	C13orf29	C17orf91	MAFK	NCEH1	OSBPL11	SEC23IP	TSPYL2	YPEL4
	BIRC3	C1orf106	CNN3	EIF1	IL7R	LIMD2	YTHDF1	KRT33B	CHD4	HS3ST1

	PHLDA1	MHG0057	SH3BP5	SHISA5	SPI1	ZNF597	DLX2	MKNK2	RAB21	SETD8
	AQP9	ETV5	MFHAS1	PIGT	TGFB1I1	IL18BP	TBC1D22B	TEX10	MAPRE1	NRAS
	RASL10A	HS3ST2	MSI1	SLC25A45	TRIM9	ZCCHC2	APOLD1	C1orf198	CTTNBP2NL	CD300LB
	IPPK	PPP3R1	SPON2	AURKAPS1	CSF1	SIGLEC10	WAPAL	FUT7	CAMK2N2	FBRS
	MYBL2	MADCAM1	C18orf8	RBBP8	TLR2	TOX2	MOBKL2A	ODF2	TUBB6	TPO
	DDAH2	ITPRIP	PPP2R1B	TMEM51	UBTD2	KLHL6	KRAS	LTB4R	RPIA	TNFSF9
	EMP3	FCGR1B	MEPCE	NKIRAS2	ADAMTSL4	MAP4K4	NAF1	NAMPT	TNFAIP8L3	ZBTB43
	DDHD1	IL18RAP	PTRH2	RND3	TUFT1	FAM110C	HSP90B1	PNRC1	VSTM2A	ADAM9
	IRF2BP2	ADPRHL2	SLC7A2	CEACAM3	ELF3	FOSB	RRP12	TRAF1	MGC16025	CFP
	IL4I1	RAB35	ZNF165	C22orf9	CD248	CHORDC1	DAPK3	IL1RL1	LSP1	VCL
	CPM	MID1	SULT4A1	CLEC17A	CD2BP2	HMGA1	HSPH1	NFATC1	ARG1	ACVR1
	ASPHD1	GADD45B	TAF5L	RBM38	SCXB	SFRS15	ULBP2	LRRC8A	SENP2	BCL9L
	RAB43	BCL2A1	GPR84	RGS3	AKIRIN2	C12orf68	SLAIN2	TMEM173	CRK	LOC100129550
	PLEKHA4	WDR43	C1orf162	ESYT1	S100A12	GAR1	MAT2A	PLD3	ZC3H12A	ACTR3
	C9orf25	MAP3K6	C1orf130	EMR2	GADD45A	KDM5B	BRD4	IFNGR1	IL18R1	MAP1LC3C
	UBAP1	C17orf96	CCNJ	U2AF1	C19orf35	CXCL16	HSPB8	LAPTM5	NID1	TUBB3
	USP36	AHNAK2	GTPBP4	LILRB4	LONRF3	REXO1	IRAK2	NUMBL	TUBB2A	FAM18A
	ZDHHC1	FCAR	MTF1	NUPL1	PICALM	CMTM7	PITPNC1	SH3RF1	IFFO2	MLKL
	MT1E	RAI14	C1QTNF1	C9orf109	CBFB	CTXN1	RASD2	WARS	MYBPC2	CCDC109B
	ITGB6	RCAN1	HSPA5	FJX1	OSMR	ATF5	CFL1	ITGB8	RAB20	EBI3
	WAS	ATG9B	FOXO1	MEFV	PDE3B	SH3BGRL3	SLC17A9	TP53	BACH2	CXorf49B
	INPP1	PANX1	PDIA3	PSEN1	GPR37	C19orf38	NGFR	RBM7	TCF7	PEA15
	S100A3	C9orf21	CDC42SE1	LOC541471	PAK3	OSGIN2	GPR132	WDR82	WWTR1	CTSZ
	GATA6	IRAK3	OPN5	AREG	CD3EAP	MSC	ATF4	BTN2A2	PCDHA4	RIN1
	SH3GL1	CLEC4D	NBL1	ANGPTL4	CHIC2	CST7	CYTH1	HLA-B	LRG1	TIPARP
	SMNDC1	A4GALT	BAG3	PPARD	PVRL2	EAF1	COL8A1	FMNL1	SLC10A6	TYMP
	B4GALT1	DUSP5	ERRFI1	RIPK2	RNF138	SMURF1	STXBP1	ANKRD13C	CENPN	CD7
	PSTPIP2	STK40	COL23A1	LOC338651	SULT1C4	UBC	URB2	C14orf80	HNRNPAB	ZNF672

	GPRIN1	LILRB3	CDC42EP4	NFE2	NR4A3	UBE2S	CD74	MYEOV	RNF149	C19orf59
	TAC4	C10orf108	SPRED1	CXCL1	EFHD2	TNFAIP6	SF3B4	YWHAH	YWHAZ	CMTM3
	FPR1	MNT	NFKB1	PCBP1	ITGAX	SECTM1	TRIM38	FKBP5	PLAGL2	SPRED2
	FAM72A	TUBA1C	CLDN23	MET	SAP30BP	SMAP2	TGFBR1	UBE2I	ARFGAP3	FAM131A
	S100A2	TNFAIP1	TRIM36	C22orf24	AFF4	F2RL3	NACAD	NFE2L2	CSGALNACT2	CSRP1
	DEGS1	FAIM3	GAB2	HAUS2	SFN	CYP19A1	LTA	GNAI3	NUFIP2	SEC24D
	SLC25A25	ANP32B	C9orf110	CEBPG	SEMA6A	SNX9	C2orf77	ENDOD1	SEL1L3	SLC2A14
	NMU	IFITM3	SENP5	PAQR4	VPS37C	DDX17	ERN 1.00	SYN1	JUND	PRIC285
	CNR1	RRS1	TLE1	TRAF4	COMP	ANKRD57	CLEC1A	KRT18	CHD1	MTHFD2
	RBBP6	SMOX	TNFRSF10A	PAQR9	PRDM1	TRIB1	CABLES1	ZNF367	BMP2	CWC22
	OASL	OSCAR	CHRNA10	FOXP4	HP	PVR	ZSWIM6	IDI2	PHF13	TNFRSF6B
	BHLHE40	GNA13	PIM1	ACBD3	DDX27	FAM59A	MKI67IP	NAA15	NEXN	PIM3
	RHPN2	SFPQ	CSF3R	FERMT2	KRTAP5-1	UGDH	C5orf58	CCDC96	DTX3L	GRK6
	PAF1	CITED4	PDLIM7	C11orf88	SLC32A1	CAMSAP1	OSM	DCLK1	PPP1R15B	SFRS13B
	BACH1	KIAA0226	TRIM47	TUBA1A	IL24	AEN	FAF2	MMP25	VPS37B	NFKBIZ
	SLC16A6	CEBPD	CSRNP2	MAP7D3	MMP9	ELK1	HCLS1	ADAMTS9	NCRNA00152	C19orf22
	GPR97	PGM2	RHOG	SERTAD1	SIK2	ZNF189	LSR	SPRY4	JOSD1	DCP1A
	SACS	CCL22	IL1B	MED26	PNN	ZFP36	GNL1	DYRK3	CPA4	GRRP1
	PURB	SMTN	SERPINH1	UBE2H	ATP6V0A2	ISG20	ROBO4	CD81	SYNC	DOT1L
	RNF103	FAM100A	TNFRSF10D	SAMD4B	CHRNE	MAFG	CRISPLD2	EGFLAM	FZD5	TEKT1
	BAZ1A	PPP1R15A	KRT16	MESDC1	SMARCA5	ZNF787	B4GALT5	EIF2C2	KLF7	MED17
	SPRY1	ARL8A	MEST	ACHE	ISG20L2	ZDHHC5	S100A16	UPP1	CCDC9	HSP90AB1
	NCOA7	TGFB3	GFPT2	JHY37681	NOLC1	NUDT10	POLR3D	DCUN1D3	DKFZp761E198	PER1
	SYNCRIP	APOBEC3B	UTP3	HSPA6	PLB1	C5AR1	RELL1	TIMP1	HIVEP2	LZTS1
	VGF	RBM24	TOB2	MAP7D1	AMMECR1L	DNAJB1	FADS3	BCAR1	MEX3D	GPR183
	SEH1L	TNFRSF18	ATP2B1	DUSP16	MT1M	C1orf38	TOP 1.00	MTA2	PRR24	STAT3
	STEAP4	ARL 14.00	TAP1	BCL10	PGM2L1	RND1	SAMD4A	STRN3	C19orf26	XPO6
	FGFR1	RELA	SRPR	MAP2K3	PSME4	CDKN1A	FAM83G	KIR2DL4	OBFC2A	LATS2

	NOP58	COL13A1	CCL2	EIF4A1	MAPKAPK2	ZNF295	EPHA2	NPC1	PRR7	ELL2
	SBNO2	ELF4	SHC1	MAPK1IP1L	MTMR9	PIWIL4	FBXL12	TEAD3	MT2A	MXD1
	RAP2B	C17orf107	RNF19B	CORO1C	TNFAIP3	SLC2A3	WDR44	MEF2D	LONRF1	SCG2
	SQSTM1	NPPB	CCNK	AGFG1	KCTD5	GRASP	NFIL3	PLK3	GGNBP2	SERPINB8
	TMEM233	ARHGDIA	INHBB	RHOB	TP53BP2	RYBP	LRRC8E	DDX5	FHL3	MSN
	GNL2	GJA1	MAK	MAP1S	PDLIM1	RBMS1	ZSWIM4	DDX21	IL1R1	JMJD6
	KCTD10	SEC24A	DUSP14	CCDC120	SF3A2	C2CD4A	ZC3H12C	BAG5	ETS1	SDC4
	SRF	RAPH1	TNFRSF10B	RHOU	TAC1	EIF2AK3	NFKB2	SOCS3	KBTBD2	BATF3
	C9orf150	TNFRSF12A	EZR	TFE3	TMEM217	YWHAG	HNRNPA0	NFKBIE	CSF3	CCRN4L
	KPNA2	TMEM2	RELB	BYSL	CSRNP1	SOX9	TNFRSF1A	GRAMD1A	KRT8	RRAD
	DLGAP4	MIDN	ARHGAP17	ERF	FAM53C	DNAJC2	LMNA	PLAUR	ADAMTS1	GPR3
	SPATA2L	CSDA	DUSP8	CNKSR3	C6orf145	MEX3C	SGMS2	KLF6	RASSF1	CREB5
	ODF3L1	SPHK1	UGCG	ARID5B	CDV3	SPSB1	MMP19	SERPINE1	C16orf72	DNAJB11
	KDM6B	LDLR	CNN2	CX3CL1	RELT	SNAI1	BCL3	MCL1	TSC22D2	ARID5A
	MAFF	FSTL3	ZYX	MACC1	MAMLD1	TICAM1	ADAMTS4	LRRC59	NAB2	MT1A
	KLF16	ITPKC	C11orf24	AG2	DNAJB5	CDC42EP1	PTPN1	CHSY1	ETS2	LIF
	CEBPB	RARA	ZNF474	MAP3K14	CLCF1	FOSL1				
**CONSERVED IN N/S1**	ADAMTS7	TTYH3	CD53	SLAMF6	TFPI	ART4	AGT	CNN1	CTGF	MGC29506
	TNIP3	NLRP7	GZMK	CCR1	CXorf21	PRNP	FOLR2	PLEK	PTPN22	S1PR4
	CCL13	CXCL3	NHEDC2	LYPD5	VSIG1	C1orf92	ADA	APOBEC3A	PXDN	AZIN1
	NFATC2	RFTN1	KCNA6	ARF4	PVT1	SPARC	ACVRL1	CLDN1	ZBP1	CFB
	C1S	MARCO	TRPA1	CALD1	CCL21	FPR3	CDKN2BAS	KIAA1324	MICALCL	EPB41L2
	MMP10	NKX3-1	NRG3	IL11	PLAC8	CD4	ITGA1	STOM	FBLIM1	ITK
	C15orf21	AEBP1	PTRF	ACTA2	IGDCC4	PCDHGA4	SERPINE2	PMP22	VCAN	COL5A2
	MPEG1	PTP4A1	GUCY1A2	TLR6	ARNTL2	CDR2	JAM3	MRC1	SKIL	TMEM200A
	ZNF259	STAT4	FSTL1	C2orf85	C6orf150	NUP107	CD1A	LOH3CR2A	C2orf89	EFCAB4B
	COL16A1	ZNF267	COL1A1	PMEPA1	WISP1	EDAR	CBLB	SLAMF1	SIGLEC1	ADAMTS2
	MCAM	PAG1	TIMP3	CD209	MYL9	FBLN5	COL5A1	MICAL2	PTX3	AMICA1

	EMP1	CSF2RB	C1R	COL15A1	HGF	PLCXD2	HNRNPA1L2	LOC401093	EHD4	CD226
	SLA2	CDK2	CD28	FAM65B	GPCPD1	CD68	TNFRSF9	LMNB1	DUSP4	FERMT3
	HIVEP3	FPR2	SIGLEC14	ATF3	GPR19	ADAM12	ARHGAP15	CD38	CD5	EIF2S1
	COL7A1	MARCKSL1	RPGR	CYR61	PAX9	GPR171	MIR155HG	BTBD19	PLTP	LCP1
	CD86	FLNC	COL6A2	LILRB5	ARHGAP30	CCR7	AKAP2	EMILIN2	THBS2	MS4A4A
	STAB1	C13orf18	HLA-DQA1	SLIT3	CD70	PDLIM4	HECA	SEC23A	CXCL13	WEE1
	ANXA1	HLA-DRA	PIM2	HK3	CD163	AOAH	CD300E	EGR3	CILP2	CHST11
	SH2B3	OXTR	PLEKHO1	ACTN1	TJP1	CCL7	PTHLH	IL2RA	DDX3X	GLIPR2
	IL10	CD97	IFI16	LILRA5	THOC4	PCDHGA12	FOXP3	RAB33A	CXCR5	ICOS
	TRA2B	GLIPR1	FOSL2	ACTG1	STK17B	RBMXL1	B3GNT5	TNFSF8	SERPINB2	ACTN4
	RASSF2	NFKBIA	HLA-DOA	PTPN12	KLF4	TAGLN	PLEKHG2	CDK17	ADAM19	ABL2
	INHBA	SLC25A32	DMP1	ARCN1	MALL	ELOVL5	SH2D2A	IRF8	TNFAIP2	TRPV2
	CCIN	IRF1	C14orf43	FILIP1L	MX2	NUP98	TRH	SEMA7A	PTPRE	B3GNT7
	PDGFB	MFSD2A	AXL	LILRB2	IL8	VCAM1	EREG	PALLD	IRF4	TREM1
	FLNA	KLF10	STX11	ITGA5	LILRA6	COL4A1	FGR	LMCD1	S1PR2	CD93
	RAET1L	ANKRD1	SGK1	SGK223	ACTB	HRH1	KIAA1949	NNMT	SOX7	IL6
	HEYL	HAPLN3	DLC1	NDEL1	FAM102B	SDS	IL4R	LBR	ARF6	FUT4
	MYADM	HBEGF	LCP2	RNF19A	HAS1	SERPINB9	PLEKHO2	XIRP1	CLDN6	ARL4C
	SELE	PPRC1	AKAP12	ICAM1	GBP1	THBS1	THBD			
	C1orf94	HMGB1P1	C3orf57	EEF1A1	LOC96610	TUBGCP4	FBXO39	BTLA	C14orf4	MAL2
	NLGN4X	PAX6	RPS12	SOX30	TMPO	TSEN15	ZNF521	DRD1	OPTC	BUD13
	FAM20A	IARS	LOC441208	RPS16	TRAM1	ZNF660	HTR2A	ARSF	IGFL1	TFF2
	BTG2	FASLG	HPD	LMO4	MRTO4	PALM2-AKAP2	PLCB1	ST6GALNAC3	PCDHA11	WISP2
**ACQUIRED IN S1**	C8orf80	CTNNAL1	NOS3	PLCE1	VGLL3	INSL4	CARD9	DDX18	ECSCR	FAM83D
	KIF18A	RPL41	TTC35	ZNF662	TSPO2	CYP8B1	FAM75C1	C4orf41	CNOT7	COL22A1
	FRMD4B	SYCE1L	TP63	ANXA2P1	C14orf149	FKBP14	OAS3	RPS27A	SNTB2	RPRML
	COL10A1	RP1	ARHGAP22	CALU	CTSK	FAM124B	FYB	PLCXD3	TBCEL	KHDC1
	FMO6P	ATRX	CLEC14A	ITCH	SP140	TCF12	ZNF542	ST8SIA6	GPR22	PAEP

	CCL3	DHX36	KIAA1467	PELI2	TNFRSF1B	GPR173	PI16	ATAD5	CDON	CXCL11
	DIO2	FNBP4	LY9	PCDHB13	SLITRK6	C4orf43	CD48	EIF5A2	MBTPS2	PNMA2
	RNF125	SNAPC5	SNORA53	TESK2	USP51	SCARA5	CD3D	CDS2	ID4	MANSC1
	MEF2A	RPL22	SLC4A1	OGN	HIF1A	HOXC10	KDR	LRIT3	SLAMF7	TMEM100
	WBP5	CLDN11	EHD2	RECK	SPTBN5	VEZT	PF4	ZBED2	FCRL3	HHIPL1
	OLFML3	CD14	LOXL2	MED13L	PARP11	SHE	SLC22A4	TMEM54	TNFSF13B	TRIM17
	ARHGAP9	CCR2	GIMAP4	MFI2	MID2	MYCL1	MYST3	NLRP2	ROBO1	SLFN5
	UNC45B	CXorf59	C1orf216	CD2	FANCB	ZNF560	GDAP1	MMP28	QSER1	SLC27A2
	PLA2G5	PLA2G2A	POU2AF1	IL17B	RTL1	C2	C5orf20	CTCFL	KCNN4	KLHL23
	PPP2R2B	VASH1	OR6F1	CACNA2D4	GBP4	GPR174	TMEM8C	ASB2	CEACAM19	FUT8
	LSM 11.00	RASGEF1B	RPL13AP6	SGCE	U2AF2	SYT16	C17orf102	CAV3	GUCA1C	LOC415056
	MS4A10	RNF17	TEX11	TRPV3	BACE1	CD79A	NFIA	SH2D1A	SLC5A4	SPATA5
	STAG1	TMEM106A	TRIM34	TRIP13	VIPR2	BHLHA15	GNASAS	PHOX2A	CCRL1	CLEC4A
	GNE	HOXC11	LAMA4	NUBPL	PLEKHG6	SLAMF8	STOX2	COL2A1	GBP7	RORB
	CASD1	KLHDC8A	SMARCA1	PTCHD3	CEP72	MYNN	NMT2	NUDCD1	PKN3	RGPD5
	SGK269	DDX60	FMOD	GVIN1	RCSD1	STEAP2	TMC7	TMSB15B	CWF19L2	HNRNPH2
	MEF2C	TMF1	OR52W1	C17orf53	C9orf167	PCDH19	PCDHGA9	QTRTD1	TMEM150C	EOMES
	FBXL7	GPRASP2	IGJ	MTAP	MYLK	XPC	BCL11B	CLECL1	LMOD1	MAFB
	SCCPDH	KIR3DL2	HMOX1	PRKAR2B	PRKDC	VRK1	PCDHGC4	ABCC13	IKZF3	MN1
	UTRN	CHST13	MYCT1	SRGN	NPHS1	AMZ1	KDM6A	LAX1	SGCG	ANXA2P3
	ATP10A	ELOVL3	HOXC4	LRRC15	MEX3B	SYNPO2	KIAA1239	GABRB1	NDST3	PITX1
	CDK14	KIAA1147	KIRREL	SGMS1	SUSD5	ALOXE3	SEL1L2	ADIG	RPL26	RRM2B
	TM2D2	UBE2V2	CD109	DSE	DSTN	LOC80154	C6orf147	IGSF5	NCAPG2	AKAP9
	ATP6V1C1	CAV1	APCDD1L	ASAP1	ENTPD3	H3F3B	LIG1	NDN	NCRNA00052	ARID4A
	CEP135	FAM198B	GPN3	MLL5	MYCBP	PLEKHF2	POLA1	BTNL2	C7	JAG1
	PHOSPHO2	SYT12	TCEAL1	EFCAB1	PLA2G1B	BANK1	ILDR1	LRRC55	BRCA2	C1QTNF2
	CRYM	CXCR7	FAM129A	TRDMT1	BAI1	DGKG	GIMAP6	IL33	LAG3	TRAT1
	AIF1	CEACAM21	ENPP2	HVCN1	OR4A47	HTR1D	FADS2	POLE2	PTPRT	SLC14A2

	LOC100240726	C2orf48	LOC641298	MAPT	PABPC1	POTEF	PUS7	ADAM28	CCR5	GNL3L
	PDZRN4	SEMA3G	LDHAL6B	LOC100188949	IGSF6	LYPD6	ABI3BP	LAYN	LY96	NAV1
	ADH1C	CAMK4	FIGF	IL21R	PARVA	RBM3	TARDBP	CRCT1	XCR1	GABRA5
	KRT34	CCDC3	CLIC2	SLC26A8	BCAR4	CTSS	MRGPRF	NID2	NTN4	TLL2
	ZNF831	FLJ41941	ALG1L	FAM49B	KCTD17	KLRC3	LOC400759	TM4SF1	AZU1	P2RY6
	CARD16	FANCM	KIAA1524	NCRNA00189	NLRP4	ANGPT1	DDX10	IGSF1	CCNA1	C20orf166
	MIR17HG	SCARA3	SFRS3	ZFHX4	COL11A1	SYNPO2L	CCND2	CTSL2	KIAA1429	PLEKHA2
	RFC3	RNF150	CPNE4	SLC5A11	KCNA1	PBRM1	SELP	CPSF6	IGSF9B	ZNF460
	SHC4	LOC100131551	CELF3	RHAG	CASP5	GPR15	NEDD4	RGL1	OIG39326	LOC201651
	DUSP5P	ANTXR1	CD27	CLEC10A	DLGAP2	ERG	P704P	PTK2	PNOC	KHDC1L
	NCRNA00112	OR10H1	ALDH3B2	ATP4B	C9orf125	CMKLR1	COL3A1	CTDSPL2	TERF1	USH1G
	CNRIP1	CXCR6	FES	NBN	NLRP1	PPP1R12A	THSD4	TIGIT	TTC17	AMOTL2
	EFNB2	OAS2	C12orf42	ARHGDIG	ABCE1	ELK3	CXorf30	BEST1	EIF3E	ITGA11
	LCK	SLC7A14	PTX4	CD1C	MAP3K1	CPNE7	ANK1	HELB	NCAPD3	ODAM
	ASB15	CCR4	PM20D1	CRYAB	GNA14	DCTN6	LOC644936	MIPOL1	ST6GALNAC1	ZCCHC5
	FAM43A	FAP	SNHG4	TPT1	SSTR3	CREBZF	GNG11	PCDHGB2	SSTR1	UBASH3B
	VNN 2.00	DAPP1	FYN	TGM2	FLT3	FCRL5	RDH12	SLC22A16	GLIPR1L1	ANKDD1A
	C4A	LRRN3	MCTP1	PCDHGA5	PTCHD2	CCL26	C11orf58	LYZ	PATL1	HSD11B1
	MTBP	PLSCR1	GPR12	CALHM2	CYP2S1	DHH	ELTD1	LOC389333	LYN	PTPN7
	TRIM29	FGD5	PLXND1	RDX	UTP23	VGLL1	ANGPTL2	LETM2	NUAK1	SLC8A1
	TNXB	VSIG4	NCAM2	CRTAM	GNL3	PCDHGB7	DKK 3.00	PTPLAD2	ENTHD1	FRMD6
	GRHL2	PLN	TASP1	TGFB2	SULT1E1	CAPN5	MAK16	RLIM	SLC7A7	C20orf103
	CAMK1G	EIF3H	KIF5C	NECAP1	KIF20B	LOC388152	METTL7B	NUP62CL	EMR4P	FAM138F
	DSCC1	MMRN1	RAET1E	CYBB	ITGA9	PIK3AP1	PLSCR4	SLC40A1	MMP1	TIE1
	GOSR2	RXFP1	PCDHA8	FAM198A	NEBL	RPL30	ST8SIA2	GPR1	KDELR3	SOX17
	CXCL9	PCDHB4	FN1	APLN	ARSB	C3orf64	RPS27	SMOC2	CUX2	CRYBB1
	PAWR	PLXNC1	FMN2	S100Z	FGFBP1	ZPBP	KCNT2	NES	PARM1	TRIM22
	PRELP	ACTA1	DOCK11	FAM26E	FHL2	ABCA4	TCF4	CNTN5	FAR2	SLC2A10

	OLFML2B	SIGLEC7	ST8SIA1	ABI3	MDFI	TNFAIP8L1	BAGE	GAL	ADORA2B	CXCR3
	RRN3P1	AFF2	H3F3C	PCDHB8	OR51B5	AKAP7	APOBEC3G	RRP15	TM4SF18	VNN 1.00
	SCML4	PCA3	FGFR1OP2	MYH7	PKHD1L1	FEZ1	KCNK6	ATM	HLA-DRB1	RPS6KA5
	WNT5A	ADAMTS12	LOC150786	TPM1	C14orf126	TSLP	CXorf56	GPR133	MRE11A	NT5E
	ALPK2	CD19	RCN1	GABRA4	TNFRSF11B	SYT9	WNT7A	CCK	DPYSL3	IL23A
	SLC16A14	DSC3	CD200R1	ING3	KCND3	DERL1	MFAP4	CCNYL1	IL2RG	RHOJ
	ZNF423	AP1S2	CD34	PCDHB7	DMBT1	SLC4A7	C13orf36	GCKR	TMEM64	PCSK5
	CHST8	AFF3	TCEAL7	TNC	CMAH	FABP5	PCDHB10	PTGER2	C12orf69	IKBIP
	MED30	GPR50	PCDHB6	C6orf97	HTR2B	BNIP2	CDH5	TMEM26	KIAA0125	TAS2R50
	LOC644538	PTPRH	LGALS2	ZCCHC18	C13orf33	HEG1	PTGER4	FLRT2	RPL7	CD80
	LOC654433	ZFAND1	C8orf34	C11orf75	SIRPA	LOC100133669	STRA6	EDN1	LOXL3	RARRES1
	TMEM45A	SLC9A9	ARHGAP23	ANKRD55	OLFM1	TGFBI	ARHGAP25	PDCD1LG2	FADS1	CD163L1
	F2R	S1PR1	PPP1R16B	SH3RF2	FLI1	NCRNA00120	UCN2	C10orf128	FOXL1	MYOCD
	POSTN	SAMD3	RHO	GPX8	MGC87042	TMEM71	IL1RAP	CD200	LRRC33	BET3L
	GRK5	LRRC8C	PANK2	MRC2	ITGA4	MSRB3	TUBB	DYNC1I1	MAGEH1	TSPAN2
	RMST	TFEC	A2M	SULF1	PTPRO	NXNL2	LRP1	CDH11	PROS1	GAP43
	NPL	PHYHIP	ZSCAN23	PCDHB16	LCTL	MGP	PCDHB3	LRRC4C	CASP1	DAB2
	PCDHAC2	CFH	DOK2	CENPJ	SAMSN1	FBN1	SLC25A21	KCNG2	TNNT2	HEPH
	PRUNE2	GRIA1	APOBEC3C	RUFY4	PDE8B	TNFSF11	MCART6	SLFN11	GNG2	PRKCB
	CNTNAP3	VIM	NLRP10	ME1	PDLIM3	SLFN12L	RORA	FOXC2	TMEM150B	LOX
	PCDHB9	SNAI2	SLC43A3	CR1	ZEB2	CDCA7	KLHL4	HPS5	STEAP1	TULP2
	NR5A2	GPR85	TNFSF18	GALNT6	LGI2	XYLT1	TFPI2	APBA1	FERMT1	
	C1orf94	HMGB1P1	C3orf57	EEF1A1	LOC96610	TUBGCP4	FBXO39	C14orf4	GUCY1A2	MAL2
	NLGN4X	PAX6	RPS12	SOX30	TMPO	TSEN15	ZNF521	DRD1	OPTC	CXCR5
**LOST FROM S1**	FAM20A	FPR2	HBEGF	LOC441208	RPS16	TRAM1	C1orf92	HTR2A	ARSF	IGFL1
	BTG2	FASLG	HPD	LMO4	MRTO4	PALM2-AKAP2	PLCB1	ST6GALNAC3	PCDHA11	WISP2
	C8orf80	NOS3	PLCE1	NLRP7	INSL4	CARD9	DDX18	ECSCR	ELOVL5	FAM83D
	KIF18A	LILRB2	RPL41	TTC35	ZNF662	TSPO2	CYP8B1	FAM75C1	C4orf41	COL22A1

	HEYL	PLAC8	SLA2	SYCE1L	TP63	CD300E	ANXA2P1	C14orf149	CD86	FKBP14
	OAS3	RPS27A	SNTB2	RPRML	RP1	ARHGAP22	CALU	CTSK	FAM124B	FYB
	PLCXD3	TBCEL	KHDC1	FMO6P	ATRX	CLEC14A	EMP1	HLA-DRA	ITCH	SP140
	TCF12	ZNF542	ST8SIA6	GPR22	PAEP	CCL3	HECA	KIAA1467	NFKBIA	PELI2
	S1PR4	GPR173	PI16	ATAD5	CDON	CXCL11	DIO2	FGR	FNBP4	GZMK
	LY9	NRG3	PCDHB13	SLITRK6	C4orf43	CD48	MBTPS2	PNMA2	RNF125	SNAPC5
	SNORA53	USP51	SCARA5	CD3D	CDS2	ID4	MANSC1	MEF2A	RPL22	SLC4A1
	OGN	HIF1A	HOXC10	KDR	LRIT3	SLAMF7	TMEM100	WBP5	EHD2	RECK
	STOM	VEZT	PF4	RAET1L	HHIPL1	OLFML3	CD14	MED13L	PARP11	SGK223
	SHE	SLC22A4	TNFSF13B	TRIM17	ARHGAP9	CCR2	GIMAP4	MID2	MYCL1	NLRP2
	NUP98	SLFN5	UNC45B	CXorf59	C1orf216	CD2	FAM65B	FANCB	CDKN2BAS	ZNF560
	ACTN4	FOSL2	GDAP1	QSER1	S1PR2	SLC27A2	PLA2G5	PLA2G2A	RTL1	ARF4
	C5orf20	CTCFL	EGR3	KCNN4	KLHL23	PPP2R2B	OR6F1	CACNA2D4	GBP4	GPR174
	TMEM8C	ASB2	CD4	CEACAM19	FUT8	ITGA1	LSM 11.00	RASGEF1B	RPL13AP6	SGCE
	U2AF2	ZNF267	SYT16	C17orf102	CAV3	GUCA1C	LOC415056	MS4A10	RNF17	TEX11
	BACE1	CD79A	CDK2	NFIA	PPRC1	SH2D1A	SLAMF6	SPATA5	TRIM34	TRIP13
	VIPR2	GNASAS	PHOX2A	CCRL1	CLEC4A	GNE	HNRNPA1L2	HOXC11	PLEKHG6	STOX2
	COL2A1	GBP7	RORB	AGT	CASD1	COL7A1	KLHDC8A	PTCHD3	CD226	CEP72
	NMT2	NUDCD1	PKN3	RGPD5	SGK269	ZBP1	ARCN1	DDX60	GVIN1	PTP4A1
	STEAP2	TMC7	TMSB15B	CWF19L2	HNRNPH2	MEF2C	OR52W1	HAS1	CXCL3	C17orf53
	C9orf167	PCDH19	PCDHGA9	PLEKHO1	TMEM150C	CCL7	FBXL7	GPRASP2	MYLK	XPC
	C14orf43	CLECL1	LBR	MAFB	PRNP	SCCPDH	KIR3DL2	CYR61	HMOX1	IL10
	PRKAR2B	VRK1	PCDHGC4	ABCC13	NKX3-1	UTRN	CHST13	HK3	SRGN	NPHS1
	AMICA1	KDM6A	LAX1	NDEL1	SGCG	ANXA2P3	ARF6	ELOVL3	HOXC4	LRRC15
	KIAA1239	GABRB1	NDST3	PITX1	CDK14	KIAA1147	SGMS1	SUSD5	ALOXE3	SEL1L2
	ADIG	RPL26	RRM2B	TM2D2	UBE2V2	DSE	DSTN	HIVEP3	LOC80154	TFPI
	C6orf147	IGSF5	GPCPD1	NCAPG2	ZNF259	AKAP9	CAV1	LILRA5	MS4A4A	APCDD1L
	ENTPD3	LIG1	NDN	NCRNA00052	MMP10	TRH	ARID4A	CCL21	CEP135	FAM198B

	GPN3	MLL5	MYCBP	PLEKHF2	POLA1	PAX9	BTNL2	C7	EIF2S1	JAG1
	MRC1	PHOSPHO2	TCEAL1	EFCAB1	PLA2G1B	BANK1	COL16A1	ILDR1	LRRC55	BRCA2
	C1QTNF2	CRYM	CXCR7	FAM129A	TRDMT1	DGKG	DUSP4	GIMAP6	HLA-DOA	IL33
	LAG3	LMNB1	TRAT1	AIF1	ENPP2	HVCN1	OR4A47	HTR1D	FADS2	POLE2
	PTPN12	PTPRT	SLC14A2	LOC100240726	C2orf48	FOXP3	MAPT	POTEF	PUS7	ADAM28
	CCR5	CD209	GNL3L	PDZRN4	SEMA3G	LDHAL6B	LOC100188949	IGSF6	LYPD6	PLCXD2
	ABI3BP	ANXA1	NAV1	ADH1C	CAMK4	FIGF	RBM3	TARDBP	CRCT1	XCR1
	GABRA5	DMP1	KRT34	CCDC3	CLIC2	VSIG1	SLC26A8	BCAR4	CTSS	FLNA
	GPR171	NTN4	TLL2	ZNF831	ALG1L	IRF4	KCTD17	KLRC3	LOC400759	TM4SF1
	AZU1	CARD16	FANCM	KIAA1524	MARCKSL1	SOX7	NCRNA00189	NLRP4	ANGPT1	DDX10
	SGK1	IGSF1	CCNA1	C20orf166	TNFRSF9	MIR17HG	SCARA3	SFRS3	ZFHX4	COL11A1
	SYNPO2L	ACVRL1	CCND2	CTSL2	PLEKHA2	RFC3	RNF150	CPNE4	SLC5A11	KCNA1
	NHEDC2	PBRM1	SELP	CPSF6	IGSF9B	ZNF460	AOAH	JAM3	PMEPA1	SHC4
	LOC100131551	CELF3	RHAG	CASP5	GPR15	XIRP1	RGL1	JUZ39326	LOC201651	DUSP5P
	ERG	KLF4	P704P	PTK2	KHDC1L	NCRNA00112	OR10H1	ALDH3B2	ATP4B	C9orf125
	CMKLR1	CTDSPL2	USH1G	SERPINB2	CNRIP1	CXCR6	FES	MIR155HG	NBN	PLEK
	PPP1R12A	THSD4	TIGIT	TTC17	CD5	AMOTL2	EFNB2	OAS2	C12orf42	ARHGDIG
	ABCE1	ELK3	PDGFB	TTYH3	CXorf30	BEST1	EIF3E	ITGA11	ITK	LCK
	SLC7A14	PTX4	MAP3K1	CPNE7	ANK1	CDK17	HELB	NCAPD3	ODAM	ASB15
	CCR4	PM20D1	CRYAB	GNA14	DCTN6	IL6	LOC644936	MIPOL1	ST6GALNAC1	CBLB
	FAM43A	SNHG4	TPT1	SSTR3	CREBZF	GNG11	HGF	PCDHGB2	SSTR1	STK17B
	UBASH3B	VNN 2.00	FYN	TGM2	FLT3	RDH12	SLC22A16	GLIPR1L1	LRRN3	MCTP1
	PCDHGA5	PTCHD2	SH2D2A	ABL2	C11orf58	MFSD2A	HSD11B1	MTBP	PLSCR1	GPR12
	CYP2S1	ELTD1	LCP2	LOC389333	LYN	PTPN7	TRIM29	FGD5	RDX	UTP23
	VGLL1	LETM2	NUAK1	SLC8A1	NCAM2	KCNA6	PCDHGB7	PTPLAD2	ENTHD1	FRMD6
	GRHL2	SH2B3	TASP1	TGFB2	SULT1E1	CAPN5	RLIM	C20orf103	CAMK1G	KIF5C
	NECAP1	KIF20B	LOC388152	METTL7B	NUP62CL	EMR4P	FAM138F	DSCC1	MMRN1	RAET1E
	CXorf21	CYBB	ITGA9	KLF10	PLSCR4	SLC40A1	MMP1	RBMXL1	TIE1	GOSR2

	RXFP1	PCDHA8	NEBL	RPL30	ST8SIA2	TLR6	GPR1	KDELR3	SOX17	CXCL9
	CDR2	ICOS	PCDHB4	ACTB	ITGA5	APLN	ARSB	C3orf64	RPS27	CUX2
	PAWR	PLXNC1	S100Z	FGFBP1	ZPBP	NUP107	PARM1	TRIM22	PTHLH	C2orf85
	ACTA1	DOCK11	EFCAB4B	PTX3	ABCA4	AXL	DDX3X	IRF1	TCF4	CNTN5
	ST8SIA1	ABI3	ARL4C	GPR19	MDFI	BAGE	CD38	GAL	ADORA2B	RRN3P1
	AFF2	PCDHB8	TRA2B	OR51B5	AKAP7	APOBEC3G	RRP15	TM4SF18	VNN 1.00	WISP1
	SCML4	PCA3	FGFR1OP2	MYH7	PKHD1L1	ATM	HLA-DRB1	RPS6KA5	WNT5A	LOC150786
	PTPRE	THOC4	TPM1	C14orf126	TSLP	EREG	CXorf56	GPR133	NT5E	RFTN1
	SELE	RCN1	SEC23A	GABRA4	TNFRSF11B	SYT9	CCK	DPYSL3	IL23A	SLC16A14
	DSC3	ING3	KCND3	MFAP4	CCNYL1	IL2RG	SERPINB9	ZNF423	AP1S2	CD34
	IGDCC4	PCDHB7	DMBT1	CD93	SLC4A7	IL11	C13orf36	GCKR	EPB41L2	TMEM64
	CHST8	AFF3	TCEAL7	CMAH	MARCO	PCDHB10	PTGER2	C12orf69	IKBIP	MED30
	PCDHB6	C6orf97	BNIP2	CDH5	KIAA0125	TAS2R50	PTPRH	C2orf89	LGALS2	ZCCHC18
	PTGER4	FLRT2	RPL7	TRPA1	CD80	LOC654433	ZFAND1	C8orf34	C11orf75	FLNC
	SIRPA	LOC100133669	CFB	EDN1	RARRES1	TMEM45A	C13orf18	ANKRD55	OLFM1	TIMP3
	ARHGAP25	FADS1	CD163L1	CD53	S1PR1	WEE1	CTGF	SH3RF2	CD97	FLI1
	LYPD5	PCDHGA12	PCDHGA4	C10orf128	FOXL1	SAMD3	RHO	GPX8	TMEM71	IL1RAP
	MX2	CD200	LRRC33	BET3L	HLA-DQA1	LRRC8C	PANK2	ITGA4	PAG1	MPEG1
	MSRB3	TUBB	DYNC1I1	MAGEH1	RMST	LCP1	PIM2	A2M	CLDN1	PTPRO
	EDAR	TNIP3	NXNL2	LRP1	PROS1	GAP43	STAT4	NPL	TJP1	IRF8
	ZSCAN23	PCDHB16	LCTL	MGP	LRRC4C	CASP1	DAB2	PCDHAC2	DOK2	CENPJ
	FBN1	SLC25A21	KCNG2	TNNT2	PRUNE2	GRIA1	RUFY4	ICAM1	PDE8B	APOBEC3A
	TNFSF11	GNG2	PRKCB	CCR1	CNTNAP3	FSTL1	B3GNT7	THBD	NLRP10	ANKRD1
	ME1	SLFN12L	RORA	SERPINE2	FOXC2	LOX	PCDHB9	CR1	ZEB2	CDCA7
	DLC1	HPS5	STEAP1	TULP2	TMEM200A	BTBD19	NR5A2	GPR85	TNFSF18	LGI2
	TFPI2	APBA1	FERMT1							
**CONSERVED IN S1/S2**	CXCL13	BTLA	CCL13	BUD13	IARS	IL2RA	SDS	ZNF660	TFF2	EHD4
	RAB33A	ADAMTS2	CTNNAL1	VGLL3	ADAMTS7	AZIN1	CNOT7	FRMD4B	FUT4	COL10A1

	COL15A1	DHX36	TNFRSF1B	ATF3	CD28	EIF5A2	TESK2	COL4A1	MYL9	CD68
	CLDN11	NNMT	SPTBN5	ZBED2	C1S	FCRL3	RPGR	TNFSF8	ARHGAP15	LOXL2
	TAGLN	TMEM54	MFI2	MYST3	ROBO1	LOH3CR2A	SPARC	MMP28	POU2AF1	IL17B
	C2	VASH1	ARNTL2	ARHGAP30	TRPV3	HRH1	SLC5A4	STAG1	TMEM106A	CD1A
	BHLHA15	LAMA4	NUBPL	SLAMF8	SMARCA1	CNN1	MYNN	FMOD	RCSD1	ADAM12
	COL6A2	TMF1	ADA	FILIP1L	QTRTD1	AKAP2	EOMES	IGJ	MTAP	SLAMF1
	BCL11B	LMOD1	PRKDC	IKZF3	LOC401093	MN1	MYCT1	AMZ1	THBS2	ATP10A
	MEX3B	SYNPO2	IL8	KIRREL	VCAN	CHST11	HAPLN3	CD109	COL5A2	LILRA6
	ATP6V1C1	ASAP1	C1R	CSF2RB	H3F3B	SYT12	PTPN22	FBLN5	BAI1	COL1A1
	CEACAM21	FERMT3	LOC641298	PABPC1	LAYN	LILRB5	LY96	NFATC2	PVT1	IL21R
	PARVA	MRGPRF	NID2	FLJ41941	FAM49B	P2RY6	MCAM	KIAA1429	CLDN6	NEDD4
	ANTXR1	CD27	CLEC10A	DLGAP2	PNOC	COL3A1	TERF1	CALD1	FBLIM1	NLRP1
	PTRF	CCIN	CD1C	SIGLEC1	TNFAIP2	MGC29506	ZCCHC5	FAP	DAPP1	TREM1
	FCRL5	ANKDD1A	C4A	CCL26	LYZ	PATL1	CALHM2	DHH	PLEKHO2	PLXND1
	SLIT3	ANGPTL2	RASSF2	SEMA7A	TNXB	VSIG4	CRTAM	GNL3	SIGLEC14	DKK 3.00
	PALLD	PLN	MAK16	SLC7A7	EIF3H	C6orf150	PIK3AP1	ACTG1	KIAA1949	FAM198A
	STAB1	COL5A1	FN1	SMOC2	CRYBB1	FMN2	KCNT2	NES	PDLIM4	PRELP
	FAM26E	FHL2	FAR2	SLC2A10	OLFML2B	SIGLEC7	TNFAIP8L1	AKAP12	CXCR3	PXDN
	H3F3C	MALL	FEZ1	KCNK6	ADAMTS12	ART4	MRE11A	ALPK2	CD19	CCR7
	WNT7A	VCAM1	CD200R1	SLC25A32	DERL1	FPR3	KIAA1324	RHOJ	B3GNT5	CD163
	PCSK5	ACTA2	TNC	FABP5	GPR50	HTR2B	TMEM26	LOC644538	C13orf33	HEG1
	FOLR2	STRA6	LOXL3	SLC9A9	TRPV2	ARHGAP23	TGFBI	PDCD1LG2	F2R	PPP1R16B
	EMILIN2	MICAL2	NCRNA00120	RNF19A	UCN2	MYOCD	POSTN	ACTN1	MGC87042	FAM102B
	IFI16	GRK5	MRC2	PMP22	TSPAN2	GLIPR2	TFEC	SULF1	OXTR	CDH11
	CILP2	PLEKHG2	IL4R	PHYHIP	PCDHB3	AEBP1	CFH	SAMSN1	C15orf21	HEPH
	APOBEC3C	GBP1	MCART6	SLFN11	GLIPR1	LMCD1	VIM	PDLIM3	CD70	TMEM150B
	SKIL	SNAI2	PLTP	SLC43A3	ADAM19	KLHL4	MYADM	MICALCL	INHBA	GALNT6
	XYLT1	THBS1	STX11							

	UGT2B4	FAM117A	HECW2	SNX16	SNX30	SV2A	TRIM59	DDI2	EFTUD2	SMTNL1
	TCP11L1	VPS25	OR4F21	C1orf173	CSDC2	GTPBP2	SIRPD	C3orf52	HAUS6	LOC728392
	PGM2	PRKD3	TTYH2	NKD1	AKAP5	DCP1A	HLA-DMA	MT3	PPM1J	STAT5A
	UBASH3A	B4GALT6	D4S234E	LOC100302401	LOC283314	SYTL2	CXCR2P1	CASQ2	FCGR2A	HLA-L
	NFX1	NID1	PGS1	VCPIP1	ASB11	APOC2	C1orf113	CD82	HLA-DMB	LILRA2
	PEAR1	SNORA67	ARAP3	PCDH17	RASA2	SSTR2	UBXN2B	TOLR	IZUMO1	FAM194A
	BZW2	C1QB	C3orf63	CANT1	CTSA	DOPEY2	MYO6	SLC6A1	TSC22D2	UBA3
	CATSPER1	ADAM10	GALNTL6	LPPR4	MEIS3	NPDC1	PSIP1	STEAP3	KSR2	SH2D6
	C1QTNF1	C3orf17	C7orf68	RAC3	ZNF813	NXF3	ARID1A	CLIC4	MOXD1	MUSTN1
	SETX	TMEM39A	VSX2	CCL11	LOC100192426	DDN	ARPP19	DCAF13	FBXO5	FMN1
	LRCH3	MTMR9	ARL13B	HAVCR2	HHATL	LAIR1	PDGFA	VMO1	WNT5B	EFHC2
	C7orf45	ADAMTS4	NLGN3	PDLIM1	PRPH2	RTN4RL2	ZFATAS	MSC	MTMR7	UGT2A1
	ADM2	ARL 10.00	ERI1	IL17RD	TYMP	IL1A	GUCY2E	LBX2	CES4	FBXO45
**ACQUIRED IN S2**	ZNF704	FIBCD1	DOK1	MRPL3	RNF32	TINF2	UHRF2	UMODL1	UNC13D	ZNF525
	C13orf39	VNN 3.00	PAGE2B	CARS	HCG18	RAD21	SNORA8	TNFRSF8	ZBTB10	ZNF91
	CASP14	DAD1L	NCRNA00164	BSPRY	FER1L4	HCK	LACTB2	LTC4S	NAA15	PLXNA2
	UBE2J1	ZNF121	ARHGEF3	C5AR1	C8orf58	FAM116A	PALM2	RNF168	ZNF623	HES2
	ADIPOQ	PSG6	BTK	GYPC	LBXCOR1	NCBP2	SBF2	TMEM176A	ZBTB26	ACER3
	COL5A3	FKBP10	GATA6	MYO1G	XPA	ACO1	C10orf116	FXYD5	HLA-G	LRRC37A
	ATP2A3	CMYA5	CTPS	ITGAM	MAP3K6	MRPS22	NRG2	OXR1	RABGAP1	CALY
	TM6SF2	CPA1	C6orf204	KIAA1432	SMC2	VAV1	ZC3HAV1L	ZDHHC23	GOLGA6A	DEFA1B
	PLK5P	CDSN	EDNRA	HLA-DOB	MAK	ZDHHC21	C3orf32	IKBKB	ABCG4	CEBPG
	EVI2A	AVPR2	ENTPD7	GLE1	GTF2E1	XKR9	ATP13A3	ATP8B1	EIF4G2	HRC
	IRAK3	MFSD1	PGRMC2	SET	ANPEP	GUCY1A3	EPHB2	ITIH3	PRPF38A	C8orf39
	HPGDS	TSPAN5	C14orf73	TMEM155	CAPZA1	MS4A6A	PLA2G16	SH3PXD2B	SLC37A2	TNFRSF19
	OIT3	ART1	FAM55D	ALDH3B1	DCUN1D1	DUSP7	IGF2	SLC22A3	SP1	TMEM132A
	XKRX	CTHRC1	GRIN1	MARVELD1	SLC43A1	SVIL	BEST4	C17orf72	FYTTD1	IGDCC3
	RGP1	TMCC1	TOPORS	ZDHHC13	CELP	MCOLN2	PRKCH	PTGIR	RAPGEF5	RARG

	SMC4	SMURF2	AFAP1L2	AKAP1	CHSY1	CORO1C	CRIP1	CRISPLD2	CTR9	PODNL1
	RPL22L1	TSKU	CC2D2B	AQP3	CCDC52	EPHX4	GPNMB	LRRCC1	NAGLU	PSMD11
	SLC29A1	SLAMF9	GJA8	GNG13	ASGR1	BNIP3L	CATSPERG	GBGT1	IL16	PDLIM7
	OPN4	DIO3OS	ANKRD34B	CHST1	HLA-DPB1	OLFML1	TMC8	ZNF883	MYL3	CHAT
	DEFB103B	BMX	C14orf139	CDC7	GHR	LAMC2	LRIG2	OLFML2A	PHLDA2	RYR2
	SH3GL2	SIGLEC5	CMTM3	KIF26A	LOC151534	MCF2L2	RPL23AP53	TUBA1A	SLA	ANKRD57
	DENND4C	DICER1	DTX3L	ENC1	LGI4	MFAP2	RASAL2	URB2	ZNF281	PRG2
	WNT7B	BATF3	C17orf76	DNAJA1	LFNG	SLC5A3	DCST1	FAM122A	LOC81691	NXN
	ELF5	APOC1	CD8A	FMO3	HCLS1	SLC27A3	CHAF1B	EXPH5	IL13RA1	IPO7
	MMP11	SYT11	UPF0639	FAIM	FPR1	RAB7A	IL1RN	MEF2D	PRPF38B	RRN3P2
	ZNF292	PGPEP1L	PFKFB1	CARTPT	GP1BA	RAB6B	TLR4	TRMT12	ZNF676	C14orf118
	PRDM1	RPS13	BTN2A2	KIAA1107	MAT2A	MDK	PARP9	PDE4B	C11orf88	HLA-DRB6
	NDRG4	RELT	40422	SLC2A14	FBXO32	KCNJ5	LAMP3	PIK3R5	PPAPDC2	ZFAT
	SCN10A	DAAM2	NUDT16	ZCCHC6	ZNF256	KIAA1529	LOC200030	LRRC37A2	NOL8	SOX5
	C19orf38	CCNB3	CXCL2	ITIH4	LRRC42	SPOCD1	ZNF572	CPA3	UBR5	TREML2
	OR2AE1	PCNP	PIK3C2A	TINAGL1	CRIP3	PADI1	ITPRIP	NSUN3	HSD17B6	ADRA1A
	F2	ADCY4	EIF2C2	FRMD4A	SLC2A3	C10orf55	CCKBR	PCM1	RALGDS	ZMYND15
	PSG2	GDF7	C16orf75	CYFIP1	GREB1	MTSS1	ARHGAP17	CKS2	DLX1	GDPD5
	KIF9	SLC22A18	LPHN3	LPL	TMEM167B	ZNF469	SAA4	RAB2A	RAB32	SLC39A11
	TTLL7	AVPR1B	ACER2	FCGR1C	PHC3	SYNCRIP	TMC6	TMEM176B	VPRBP	VIT
	ASTE1	FIG4	NFASC	RSPH9	YIPF1	PNMA5	PPFIBP1	WWTR1	ZNF643	CLDN7
	SEC14L1	SLC15A2	SNORA9	ZNF484	CNGB1	BCOR	KDM6B	MYO1C	ADAM15	ADAMTS15
	C1QA	CRK	PPP2R2A	ARSI	AOC3	COL13A1	C4orf51	ADAP2	COMMD2	GON4L
	TOMM70A	CCNL1	KIAA1462	C21orf125	FAM7A2	CD33	HMHA1	IL34	INVS	PPIL1
	DCLRE1B	PILRA	DQX1	SCPEP1	SORD	KLHL33	CP	AHDC1	RNF165	FUT7
	HAMP	MAGEL2	CCRL2	CDC42EP3	CXCL16	LOC651250	SIK1	SOCS3	ZNF436	CCL16
	IL5RA	C9orf44	ACSL6	KIAA1161	SNX4	CSPG4	FKTN	GRN	KIAA0368	KNDC1
	LOC145820	NCF4	SLC39A14	HTR1B	MYOZ3	DOC2B	IQCB1	PABPC3	ZW10	CTSG

	AFAP1	IL7R	KLC3	CHST6	CXorf49B	ACAD9	SLC1A7	DDR2	SLC11A1	ZNF385D
	DPPA4	C6orf227	C6orf138	STK3	TET3	IL10RA	PRND	PTPN2	SLC45A3	MFAP5
	IL17F	ZNF645	BAG4	MTCP1	PAK1IP1	PTGFRN	SFRP1	TMPRSS4	DCAF10	FBN2
	DEFA4	ELOVL4	FCGR1B	SLC2A5	SLC9A1	HTRA3	RYBP	SLC31A1	FHL5	H1F0
	LUM	SLC12A8	TMEM233	WNK2	NTRK2	TBXA2R	HGC6.3	RPL13AP17	CCDC48	TNKS1BP1
	POU6F2	KRT1	STRBP	CACNA1F	BMP2K	CYP1B1	EMILIN1	MOCS1	MYO9B	TNFRSF11A
	C10orf131	C1RL	FAM78A	RBMS3	RNF138P1	SERPIND1	SYTL1	NR5A1	APOLD1	MYO5C
	TJP2	C15orf39	GOLGB1	ZNF347	GAPVD1	IBSP	CCDC155	ARID1B	CXCR4	SOBP
	ZBTB11	CDC26	KIF7	TUBB2B	NT5C1B	PSG5	RPS10P7	SPTBN1	TMEM130	DKFZp686O24166
	HOOK1	PIGA	RBM12	TMEM44	PSG3	CYP4B1	ENTPD2	GNB4	LHFP	MXRA8
	KIF3C	LOC729467	CBX2	CNTD2	IGFALS	KIAA0196	BFSP2	TLX2	ITGA7	PLAC4
	RAB27A	TMOD2	ZNF470	RGS7BP	CHSY3	IMPG1	KIAA1755	SERPINH1	PLXDC1	LINGO3
	FAM49A	KIAA1407	CERCAM	SAA2	CSRP1	ECM1	HDGFRP3	SFRS2	TPSAB1	ELF4
	LHX6	IFNK	EGFL6	C9orf129	CD79B	CHRD	CTTNBP2NL	GGT5	CAPN14	HTRA4
	LTBP1	SRL	OR13J1	VLDLR	MYEOV	CHMP4C	NAA50	UGCG	WNT9A	C6orf26
	CHST15	FOXN2	LOC284837	LXN	TBC1D23	PKD2L1	CEL	MTMR4	RABL3	CDV3
	EPB41	GNAI2	RICH2	SIGLEC6	NFATC1	SERPINE1	OR11H4	CCBL2	MAP7D1	MYH3
	P2RX1	FCHSD2	GSN	NCRNA00095	PPP1R14A	RASSF3	TMEM119	CORO2A	LOC338651	PSIMCT-1
	SLC1A3	ATPBD4	PSG4	SPSB1	IL22RA1	AURKAPS1	GFI1	KLHL6	LOC100126784	LOC284100
	ARMC8	CCDC80	DGKA	MTF2	ATG3	PTPLB	TEX10	ARMC1	ASPN	MMP7
	ZNF471	MYBPC2	OBP2B	ADAMTS6	FAM131B	ZNF781	ANKRD26	SNAI1	TMEM65	PLA2G2D
	AGAP11	TYW1B	CNTNAP1	MYH9	NCF1C	CD300C	TADA2A	EMP2	GOLGA7	RNF20
	POM121L9P	ANO1	DNAH1	TAF2	TOPBP1	GLI3	MLF 1.00	ACRBP	ALDH1B1	CDH6
	MGAT3	PLAU	TMEM132E	RGS13	AATK	APPL1	IGFBP2	C10orf119	MTUS1	PHF20L1
	PTGIS	CAPN8	CCL8	ITIH1	PALMD	STAG3	C9orf64	GPRC5A	PRICKLE2	KRT5
	NEK4	TPM4	C6orf103	GPR160	ZNF229	COX6B2	DEFB1	GSR	DBX1	KDM4C
	SLC16A6	SULT1B1	CIITA	SH3RF3	SYNJ2	KCNMB1	FNDC1	MS4A3	ZNF782	C20orf112
	KPNA4	RHBDL2	RIN1	FCAR	SERPINA9	DAGLA	GPR183	FAM76B	PGAP2	CCL18

	CHMP5	ZNF880	AQP9	WHSC1L1	ZDHHC2	FCRL2	COL12A1	TPSB2	DLEC1	TGFB1I1
	TTF1	FXYD1	PTP4A3	FOXF2	CSTA	LRRK1	SRPX2	LTBP4	RPGRIP1	GNG10
	POU2F2	TIMP1	B4GALT4	EDIL3	NOTCH3	TCF7	BST1	C6orf27	LOC440461	MYOM1
	FAM180A	CLEC5A	JAG2	TREML4	CD44	OSBPL11	ZNF382	KCNQ4	RPLP0P2	FCRL4
	C1orf183	CNN3	LSAMP	ZNF681	PTCRA	C15orf48	SPAG1	GPR176	DCAF12	TSR1
	TNFRSF17	C10orf28	PADI2	S100B	ADAMTS10	TIPARP	COL6A3	ECM2	FOXO3	SH3PXD2A
	HSPG2	HTR1F	PLAA	TATDN1	CACNA1I	IGSF10	GATA1	DLX2	GMEB1	CYB5R2
	IL24	NCF1	GPR124	C1orf186	NLRP12	NLRP8	KRTAP1-5	CSF1	LOC729799	FAM120A
	SEC22A	AKNA	COL14A1	MYO7B	PRSS35	ECEL1	OSR2	SYT13	UBAP2	NAA35
	DLX6AS	NBPF3	SLC24A3	PRRT4	C9orf93	IL10RB	TNNT3	KRT17	LOC221442	C14orf49
	ACTG2	ELN	PDCD10	RAB42	DFNA5	HIP1	GJC1	PHLDB1	TNFAIP6	NAV2
	SARM1	ADAM6	MMP14	COL4A2	PRRX1	KRTAP5-4	CATSPER2P1	ISLR	CNTN2	GMPS
	MYH11	MESDC1	NFYA	NRXN2	PDE3A	GALNT5	TPM2	GPR161	SNRK	MS4A2
	PODN	ART5	CALB2	RHPN2	HDC	ACP5	CPXM1	ABTB1	SGK196	FOXO3B
	TRAM2	LEP	LDB3	SERPINF1	AGPAT4	TMEM200B	WFDC1	B4GALT1	PITPNC1	LOC100130581
	APOL4	FNDC3B	SMC5	HSPB6	RB1CC1	PKP1	ROBO2	TM6SF1	PIK3CD	GTF3C4
	ZNF135	PVRL4	MAP3K12	LOC284749	PRTFDC1	RAD23B	CCDC109B	DCLK3	MFSD11	CBWD6
	NOM1	PAPPA	COL1A2	MST1R	ERICH1	GPR143				
**LOST FROM S2**	UGT2B4	CD28	CTNNAL1	FAM117A	HECW2	SNX16	SNX30	SV2A	ACTG1	DDI2
	DLGAP2	EFTUD2	FAM198A	IFI16	SMTNL1	TCP11L1	VPS25	OR4F21	C1orf173	CSDC2
	GTPBP2	SIRPD	ARHGAP15	C3orf52	HAUS6	LOC728392	PCDHB3	PGM2	PRKD3	SPARC
	TTYH2	GPR50	NKD1	STRA6	DCP1A	HLA-DMA	MT3	PLXND1	PPM1J	STAT5A
	UBASH3A	B4GALT6	D4S234E	LOC100302401	LOC283314	SYTL2	CXCR2P1	CASQ2	FCGR2A	HLA-L
	NFX1	NID1	PGS1	VCPIP1	ASB11	APOC2	C1orf113	CD82	HLA-DMB	LILRA2
	PEAR1	PTPN22	SNORA67	ARAP3	EIF5A2	PCDH17	RASA2	ROBO1	SSTR2	UBXN2B
	TJKL	IZUMO1	FAM194A	BZW2	C1QB	C3orf63	CANT1	CTSA	DOPEY2	MYO6
	SLC6A1	TSC22D2	CATSPER1	ADAM10	ATF3	GALNTL6	LPPR4	MEIS3	MYOCD	NPDC1
	PSIP1	STEAP3	TFEC	KSR2	SH2D6	C1QTNF1	C3orf17	C7orf68	RAC3	ZNF813

	NXF3	ARID1A	ATP6V1C1	CLIC4	MOXD1	MUSTN1	NNMT	VSX2	CCL11	LOC100192426
	DDN	ARPP19	DCAF13	FBXO5	FMN1	LRCH3	MTMR9	ARL13B	C15orf21	HAVCR2
	HHATL	LAIR1	PDGFA	SLC9A9	VMO1	WNT5B	EFHC2	C7orf45	MGC87042	PDLIM1
	PRPH2	RTN4RL2	ZFATAS	IL8	MSC	MTMR7	ADM2	ARL 10.00	EIF3H	IL17RD
	TYMP	IL1A	GUCY2E	LBX2	CES4	FBXO45	ZNF704	FIBCD1	DOK1	KCNT2
	MRPL3	RNF32	TINF2	UMODL1	UNC13D	ZNF525	C13orf39	VNN 3.00	PAGE2B	HCG18
	RAD21	TNFRSF1B	TNFRSF8	ZBTB10	ZNF91	CASP14	DAD1L	NCRNA00164	BSPRY	FER1L4
	HCK	LACTB2	LTC4S	NAA15	PLXNA2	PRKDC	UBE2J1	ZNF121	ARHGEF3	C5AR1
	C8orf58	FAM116A	PALM2	RNF168	ZNF623	CD1C	HES2	ADIPOQ	PSG6	BTK
	GYPC	LBXCOR1	NCBP2	SBF2	SLC7A7	TMEM176A	ZBTB26	ACER3	COL5A3	GATA6
	MYO1G	NFATC2	XPA	C10orf116	FXYD5	HLA-G	LRRC37A	RCSD1	ATP2A3	CMYA5
	CTPS	ITGAM	MAK16	MAP3K6	MRPS22	NRG2	OXR1	RABGAP1	CALY	TM6SF2
	CPA1	C6orf204	SMC2	VAV1	ZC3HAV1L	ZDHHC23	GOLGA6A	DEFA1B	PLK5P	CDSN
	EDNRA	HLA-DOB	MYADM	ZDHHC21	C3orf32	ABCG4	DHH	EVI2A	AVPR2	GTF2E1
	IL4R	NUBPL	XKR9	PNOC	ATP13A3	EIF4G2	HRC	IRAK3	MFSD1	PGRMC2
	TERF1	CRTAM	ANPEP	GUCY1A3	VIM	EPHB2	ITIH3	NES	C8orf39	HPGDS
	TSPAN5	C14orf73	TMEM155	WNT7A	CAPZA1	MS4A6A	PLA2G16	SH3PXD2B	SLC37A2	TNFRSF19
	OIT3	ART1	FAM55D	ALDH3B1	DCUN1D1	DUSP7	IGF2	SLC22A3	SLC43A3	SP1
	TMEM132A	TMF1	XKRX	FUT4	GRIN1	MARVELD1	PLN	SLC43A1	SVIL	BEST4
	C17orf72	FYTTD1	IGDCC3	RGP1	TMCC1	TOPORS	ZDHHC13	CELP	MCOLN2	PRKCH
	PTGIR	RAPGEF5	RARG	SMC4	SMURF2	AFAP1L2	AKAP1	CHSY1	CRIP1	CRISPLD2
	CTR9	PODNL1	RPL22L1	TSKU	CC2D2B	AQP3	EPHX4	GPNMB	LRRCC1	NAGLU
	PSMD11	SLC29A1	SLAMF9	GJA8	GNG13	ASGR1	CATSPERG	GBGT1	IL16	PDLIM7
	OPN4	DIO3OS	ANKRD34B	HLA-DPB1	OLFML1	TMC8	ZNF883	MYL3	CHAT	DEFB103B
	BMX	C14orf139	CEACAM21	GHR	LRIG2	MYCT1	OLFML2A	PHLDA2	RYR2	SH3GL2
	SIGLEC5	CMTM3	CSF2RB	KIF26A	LOC151534	MCF2L2	SLFN11	TUBA1A	B3GNT5	CXCR3
	SLA	ANKRD57	DENND4C	DICER1	DTX3L	ENC1	FAR2	LGI4	RASAL2	SIGLEC7
	URB2	ZNF281	PRG2	BATF3	C17orf76	DNAJA1	LFNG	SLC5A3	DCST1	FAM122A

	LOC81691	NXN	TMEM106A	ELF5	APOC1	CD8A	FMO3	HCLS1	SLC27A3	CHAF1B
	EXPH5	IPO7	SYT11	UPF0639	UCN2	FAIM	FPR1	RAB7A	IL1RN	MEF2D
	PRPF38B	RRN3P2	TNFAIP8L1	ZNF292	PGPEP1L	CCL26	CARTPT	BAI1	GP1BA	RAB6B
	TLR4	ZNF676	C14orf118	PRDM1	RPS13	SLC5A4	BTN2A2	KIAA1107	MAT2A	PARP9
	PDE4B	RPGR	CD68	HLA-DRB6	NDRG4	RELT	40422	CD1A	FBXO32	KCNJ5
	LAMP3	PIK3R5	PPAPDC2	ZFAT	SCN10A	DAAM2	NUDT16	P2RY6	TREM1	PARVA
	ZCCHC6	ZNF256	INHBA	KIAA1529	LOC200030	LRRC37A2	NOL8	SOX5	C19orf38	CCNB3
	CXCL2	ITIH4	LRRC42	SPOCD1	ZNF572	CPA3	DHX36	TREML2	OR2AE1	CDH11
	PCNP	PIK3C2A	TINAGL1	TNXB	CRIP3	PADI1	ARNTL2	CRYBB1	MRE11A	NSUN3
	HSD17B6	ADRA1A	F2	ADCY4	CHST11	FRMD4A	QTRTD1	C10orf55	CCKBR	PCM1
	RAB33A	RALGDS	SLAMF8	SLIT3	ZMYND15	PSG2	GDF7	C16orf75	CYFIP1	GREB1
	MTSS1	ARHGAP17	CKS2	DLX1	GDPD5	KIF9	SLC22A18	LPHN3	LPL	TMEM167B
	ZNF469	MYNN	RAB2A	RAB32	SLC39A11	STAB1	TTLL7	AVPR1B	ACER2	FCGR1C
	PHC3	SYNCRIP	TMC6	TMEM176B	VPRBP	VIT	ASTE1	FIG4	NFASC	RSPH9
	TMEM26	YIPF1	PNMA5	KIAA1429	PPFIBP1	WWTR1	ZNF643	ZBED2	CLDN7	SEC14L1
	SLC15A2	SNORA9	ZNF484	BCOR	IKZF3	KDM6B	MYO1C	ADAM15	ADAMTS15	C1QA
	CRK	FHL2	ARSI	AOC3	COL13A1	NEDD4	C4orf51	FLJ41941	ADAP2	COMMD2
	GON4L	LY96	TOMM70A	CCNL1	KIAA1462	C21orf125	FAM7A2	CD33	HMHA1	IL34
	INVS	PPIL1	C1S	DCLRE1B	KIAA1949	LOH3CR2A	NLRP1	PILRA	DQX1	SCPEP1
	SORD	KLHL33	CP	AHDC1	LAYN	RNF165	FUT7	HAMP	MAGEL2	CDC42EP3
	CXCL16	LOC651250	LOXL3	SIK1	SOCS3	TMEM54	CCL16	IL5RA	C9orf44	ACSL6
	KIAA1161	SNX4	XYLT1	CSPG4	FKTN	GRN	KIAA0368	KNDC1	LOC145820	NCF4
	SLC39A14	HTR1B	MYOZ3	DOC2B	HRH1	IQCB1	ZW10	CTSG	AFAP1	IL7R
	KLC3	TNFSF8	IL17B	CXorf49B	ACAD9	PALLD	SLC1A7	C1R	DDR2	SLC11A1
	ZNF385D	DPPA4	C6orf227	TFF2	C6orf138	EHD4	TET3	IL10RA	PRND	PTPN2
	SLC45A3	MFAP5	IL17F	ZNF645	BAG4	FPR3	MTCP1	PAK1IP1	PTGFRN	SFRP1
	SIGLEC1	TMPRSS4	DCAF10	SLAMF1	DEFA4	ELOVL4	FCGR1B	SLC2A5	SLC9A1	HTRA3
	RYBP	SLC31A1	FHL5	H1F0	LUM	SLC12A8	TMEM233	ARHGAP30	CLDN11	WNK2

	BHLHA15	NTRK2	TBXA2R	HGC6.3	RPL13AP17	CCDC48	FBLIM1	KCNK6	TNKS1BP1	POU6F2
	KRT1	CALHM2	STRBP	CACNA1F	BMP2K	CYP1B1	EMILIN1	MOCS1	MYO9B	PLEKHO2
	TNFRSF11A	C10orf131	C1RL	FAM78A	RBMS3	SIGLEC14	AZIN1	RNF138P1	SERPIND1	SYTL1
	NR5A1	APOLD1	IARS	MYO5C	C15orf39	GOLGB1	ZNF347	CXCL13	GAPVD1	VCAM1
	IBSP	CCDC155	ANGPTL2	ARID1B	H3F3C	SOBP	ZBTB11	AMZ1	CFH	KIF7
	TUBB2B	MICALCL	ARHGAP23	NT5C1B	PSG5	RPS10P7	SPTBN1	TMEM130	DKFZp686O24166	HOOK1
	MRC2	PIGA	RBM12	SAMSN1	PSG3	CYP4B1	ENTPD2	LHFP	MXRA8	SDS
	KIF3C	LOC729467	CBX2	CNTD2	GBP1	IGFALS	KIAA0196	ART4	BFSP2	TLX2
	ITGA7	FRMD4B	PLAC4	RAB27A	TMOD2	TRPV2	ZNF470	RGS7BP	CHSY3	KIAA1755
	SERPINH1	NID2	LINGO3	FAM49A	KIAA1407	PVT1	CERCAM	CD200R1	CSRP1	ECM1
	HDGFRP3	SFRS2	SYT12	TPSAB1	ELF4	LHX6	IFNK	EGFL6	C9orf129	CD79B
	CHRD	CTTNBP2NL	GGT5	CAPN14	ASAP1	HEG1	HTRA4	LTBP1	SRL	ALPK2
	OR13J1	POU2AF1	VLDLR	MYEOV	CHMP4C	NAA50	VASH1	WNT9A	EMILIN2	FABP5
	ATP10A	C6orf26	CHST15	FOXN2	LXN	TBC1D23	TMEM150B	PKD2L1	CEL	MTMR4
	FMN2	RABL3	CD19	CDV3	EPB41	GNAI2	RICH2	SIGLEC6	NFATC1	PIK3AP1
	SERPINE1	OR11H4	CCBL2	CNOT7	FERMT3	MAP7D1	MYH3	P2RX1	FCHSD2	GSN
	POSTN	PPP1R14A	RASSF3	TMEM119	VSIG4	CORO2A	LOC338651	MN1	PSIMCT-1	SLC1A3
	ATPBD4	LYZ	PSG4	IL22RA1	LOC100126784	LOC284100	ARMC8	CCDC80	DGKA	ATG3
	PTPLB	ARMC1	ASPN	CD163	PDCD1LG2	ZNF471	OBP2B	ADAMTS6	FAM131B	FOLR2
	ZNF781	ANKRD26	SNAI1	TMEM65	F2R	PLA2G2D	AGAP11	TYW1B	CNTNAP1	IL21R
	MYH9	NCF1C	CD300C	HEPH	TADA2A	EMP2	GOLGA7	GRK5	RNF20	SEMA7A
	POM121L9P	ANO1	FILIP1L	TAF2	TOPBP1	GLI3	MLF 1.00	IL2RA	ACRBP	ALDH1B1
	CDH6	HTR2B	MGAT3	PLAU	TMEM132E	TNFAIP2	RGS13	AATK	APPL1	C10orf119
	MTUS1	PHF20L1	PTGIS	CAPN8	CCL8	CCR7	ITIH1	MCART6	PALMD	STAG3
	C9orf64	LAMA4	PRICKLE2	KRT5	NEK4	TPM4	C6orf103	GPR160	ZNF229	COX6B2
	DEFB1	GSR	DBX1	KDM4C	SLC16A6	SULT1B1	CIITA	SH3RF3	SYNJ2	FN1
	KCNMB1	FNDC1	MALL	MS4A3	BTLA	C20orf112	KPNA4	RHBDL2	RIN1	SERPINA9
	DAGLA	STAG1	ANTXR1	THBS1	ANKDD1A	PGAP2	SMARCA1	COL10A1	CCL18	ZNF880

	TRPV3	AQP9	SLC25A32	WHSC1L1	ZDHHC2	FCRL2	C2	COL12A1	COL6A2	FBLN5
	TPSB2	DERL1	DLEC1	TGFB1I1	TTF1	CCL13	FXYD1	AKAP2	CD27	FOXF2
	CSTA	IGJ	LRRK1	SRPX2	LTBP4	RPGRIP1	POU2F2	TIMP1	B4GALT4	EDIL3
	SPTBN5	ZNF660	NOTCH3	TCF7	VGLL3	BST1	C6orf27	LOC440461	MYOM1	FAM180A
	CLEC5A	FMOD	JAG2	TREML4	CD44	OSBPL11	ZNF382	KCNQ4	PCSK5	RPLP0P2
	FCRL4	C1orf183	SLC2A10	CNN3	LSAMP	MCAM	ZNF681	PTCRA	C15orf48	SPAG1
	GPR176	LMCD1	TSR1	TNFRSF17	C10orf28	FEZ1	PADI2	SNAI2	S100B	ADAMTS10
	LILRB5	TIPARP	COL6A3	ECM2	FOXO3	SH3PXD2A	HSPG2	HTR1F	LILRA6	PLAA
	TATDN1	CACNA1I	IGSF10	GATA1	DLX2	ACTA2	CYB5R2	IL24	NCF1	GPR124
	OLFML2B	C1orf186	NLRP12	OXTR	NLRP8	KRTAP1-5	CSF1	PTRF	FAM120A	SEC22A
	CLEC10A	PXDN	AKNA	COL14A1	MYO7B	PRSS35	OSR2	SYT13	CNN1	LOC641298
	DLX6AS	APOBEC3C	C6orf150	KLHL4	LOC401093	NBPF3	SLC24A3	KIRREL	MGC29506	PRRT4
	TNC	C9orf93	GALNT6	H3F3B	IL10RB	MICAL2	TNNT3	C14orf49	MMP28	ACTG2
	ELN	PDCD10	RAB42	DFNA5	HIP1	COL4A1	GJC1	PHLDB1	NAV2	SARM1
	ADAM6	MMP14	COL4A2	PRRX1	KRTAP5-4	CATSPER2P1	ISLR	PHYHIP	NCRNA00120	VCAN
	CNTN2	GMPS	LMOD1	MYH11	MESDC1	NFYA	NRXN2	PDE3A	GALNT5	TPM2
	GPR161	SNRK	MS4A2	PODN	ART5	SYNPO2	TAGLN	CALB2	RHPN2	HDC
	ACP5	ABTB1	DKK 3.00	GLIPR2	SGK196	FOXO3B	MRGPRF	TRAM2	LEP	LDB3
	ACTN1	PMP22	SERPINF1	AGPAT4	TMEM200B	WFDC1	B4GALT1	PRELP	PLTP	PITPNC1
	TGFBI	FAP	TSPAN2	LOC100130581	APOL4	FNDC3B	MYL9	CILP2	HSPB6	RB1CC1
	SULF1	CALD1	ROBO2	SMOC2	TM6SF1	MYST3	PIK3CD	GTF3C4	LOC644538	ZNF135
	THBS2	PVRL4	MAP3K12	ADAMTS12	COL1A1	LOC284749	PRTFDC1	FCRL5	FAM26E	PLEKHG2
	RAD23B	CCDC109B	MFSD11	COL3A1	CBWD6	NOM1	CD109	PDLIM3	PAPPA	COL1A2
	ADAM12	STX11	MST1R	AEBP1	ERICH1	GPR143				
	C13orf33	TESK2	TRIM59	ADAMTS7	AKAP5	CD70	BCL11B	UBA3	EOMES	KIAA1324
**CONSERVED IN S2/S3**	SETX	TMEM39A	ADAMTS4	NLGN3	UGT2A1	ERI1	UHRF2	CARS	SNORA8	FCRL3
	FKBP10	ACO1	GNL3	KIAA1432	SKIL	MAK	IKBKB	CEBPG	ENTPD7	GLE1
	ATP8B1	FAM102B	SET	PRPF38A	CTHRC1	PPP1R16B	CORO1C	CCDC52	BNIP3L	CHST1

	CDC7	LAMC2	RPL23AP53	MFAP2	WNT7B	BUD13	RNF19A	IL13RA1	MMP11	RHOJ
	PFKFB1	TRMT12	ADA	MDK	C11orf88	SLC2A14	UBR5	GLIPR1	ITPRIP	EIF2C2
	SLC2A3	SAA4	MEX3B	FAM49B	CNGB1	PPP2R2A	CCRL2	ZNF436	PABPC3	CHST6
	STK3	FBN2	PATL1	CLDN6	PABPC1	DAPP1	TJP2	CXCR4	CDC26	TMEM44
	GNB4	IMPG1	PLXDC1	SAA2	UGCG	LOC284837	NCRNA00095	SPSB1	AURKAPS1	GFI1
	KLHL6	MTF2	TEX10	MMP7	MYBPC2	AKAP12	DNAH1	C4A	ZCCHC5	IGFBP2
	GPRC5A	ZNF782	FCAR	GPR183	FAM76B	CHMP5	MFI2	MTAP	PTP4A3	GNG10
	PDLIM4	DCAF12	ADAM19	GMEB1	LOC729799	ECEL1	UBAP2	NAA35	HAPLN3	COL15A1
	KRT17	LOC221442	RASSF2	TNFAIP6	LOXL2	CCIN	CPXM1	COL5A1	SMC5	PKP1
	COL5A2	DCLK3	ADAMTS2							
	NPFFR2	AOX1	ESR2	GNB1	JMJD7-PLA2G4B	LOC613037	PARP6	POU2F3	PRDM10	PSD3
	RBM18	SSRP1	TIGIT	TKT	UPF3B	USP1	WDR5B	ZNF567	ZNF785	MYF6
	C12orf63	LGALS7B	KRT79	ZNF80	DLK2	MCHR1	ITLN1	AQP1	ATP2A1	BMP2
	C11orf58	CBLB	CCDC123	CTNND1	DARC	ELF2	GIPC2	LOC400657	MC1R	MPP6
	NCRNA00176	PGBD4	PHF8	PRDM8	RCE1	SGPL1	SIAH1	SLC28A1	TEAD2	TRPM6
	USP48	WBSCR27	GPR141	C1QL4	C4orf47	TSPAN19	AGER	APOL6	C6	CDCA4
	CELF1	EEF1A2	FAM129B	GDPD3	HAUS2	HNRNPC	HNRNPR	IER5	IGSF1	ISY1
	LIF	NXF1	PCDHGA3	PKIA	PLK3	SIK2	SNRNP40	TLE1	TRAF2	UBA6
**ACQUIRED IN S3**	ZNF586	MAB21L2	ZAR 1.00	GLOD5	C19orf55	CLCN6	CLDN14	GGH	GPR173	IRAK2
	LOC100125556	LOC100271836	LOC283663	LOC440354	MGST1	NCRNA00085	PDGFRL	POLE3	RAB41	SEC11C
	STAG3L3	VWA5B2	ZNF691	ADAM7	CYP4A22	SLURP1	SSX8	ZNF705A	RNF183	SLCO5A1
	SPATC1	ISLR2	DNAH3	ACAT2	ARF4	AUH	C21orf91	C6orf114	CST7	FAM82A2
	GPR109B	HN1L	LRP5	LRRC15	MCART2	NFKB1	PTPDC1	RDH5	RIMS3	RRAD
	SLAMF7	SLC7A5P2	ZNF658	CPN1	ADAM2	ZSCAN10	C1orf104	CELF5	CH25H	DDB2
	DSC2	DTX4	LOC100131434	MARCKSL1	MORC2	NNAT	PDXDC1	PPP1R1A	QSOX2	SPPL2A
	ZNF8	NPPA	TREH	ACSL5	ARHGAP31	ATM	BIRC3	C4orf46	CWF19L2	DLL4
	DPYSL3	FAM18A	FCGR2C	KLRK1	LOC440944	PRRT2	WHSC1	SLC7A3	PENK	CCT6P1
	CDHR1	FASLG	HHIPL1	HLA-DQB1	HPSE	KIAA0020	LOC441454	MORF4L1	RBMXL1	RINL

	TCEA1	TTC21A	ZWILCH	SLC17A3	C12orf42	ADCY7	BASP1	CASP3	CLEC18B	FAM192A
	FAM22F	FAM45B	FBXO25	FLVCR1	LILRA4	LOC100272228	JKH37681	POLR3D	PVRIG	RGS1
	RUNX2	SERINC2	SFRS7	TACC1	TARDBP	TF	TIA1	LOC729609	C2orf73	AOC2
	CELF6	EIF2C3	KLKP1	LOC144571	MFHAS1	MIER1	MMP2	MS4A14	NKX3-1	NPR 2.00
	PPP1R15B	SEC24D	SERINC4	SERPINA3	STC1	TRA2B	UBAP1	VBP1	YARS	ZNF789
	NEU4	ACSL1	C16orf74	CASC5	CD244	CD6	DNAJC5B	FAM115C	INO80	ISYNA1
	KCNJ13	REEP3	RTN3	SLC4A7	SOX7	TRANK1	UBE2R2	ZNF805	FAM123A	IRGM
	TAS2R4	FLT3	ALPK3	CD180	CD247	CLLU1	EEF1DP3	FAM118A	GBP5	KIAA1409
	SFRS11	SGIP1	STIP1	ZNF137	ZNF557	ZNF706	OR1J1	CNTN6	OR1J2	ANKRD20A3
	C1orf77	FAM22G	FNBP4	KAL1	KDELR3	KIAA1045	LOC653113	MFSD10	MRRF	MURC
	NFKBIZ	NPTX2	PCDHGA12	PYHIN1	SH2D1A	SNHG3	NANOG	FFAR3	C2orf84	CDK20
	CEACAM19	CLEC2D	CYR61	HINFP	HSBP1	LOC100128842	NRG1	RBM20	TNFRSF10B	WDR52
	XBP1	C3orf65	C1orf213	CCDC117	CDK17	FAM120AOS	GOLGA6L5	KDELR2	MASP2	NCRNA00167
	SFRS13A	SGCB	TSTD2	UBASH3B	UBD	VNN 1.00	TMEFF2	TDRD1	LPAR4	LY6H
	ATCAY	AKAP13	ANKRD27	CD22	CG030	ETV6	FAM114A2	FAM13C	HAUS3	HIVEP1
	LSM14A	NOP14	RIPK2	SLC8A3	SNORD1C	TM4SF20	APOC1P1	C10orf91	CGB8	HMX3
	OR51A7	KCNK9	C10orf81	CCL20	BREA2	C5orf58	GDF3	UGT3A2	CD80	DNM1
	HLA-DQA1	HSPA6	KIR2DL1	KLRB1	NAV3	NRBP2	PRPF39	PTDSS1	SCD	SIGLEC8
	STC2	THAP9	PCDHGC4	ARL 14.00	BEAN	C8ORFK29	CHD7	EP400NL	FANCC	FCRL6
	FZD10	HEY1	LSM 6.00	PPM1N	RBM5	RFTN2	RPL32P3	MKW42248	TMEM151B	UNC5A
	ZNF732	OR51S1	TTPA	GCKR	PITX3	UCA1	C9orf130	CLDN9	COL23A1	GCFC1
	GPR85	IGSF9	KRT80	PCDHGA6	PHYHIPL	PRF1	SP140	SPRY1	TAS2R14	GLP2R
	WDR65	PAEP	ACBD3	C11orf61	C2CD4A	ECE1	ITGB6	KIF21B	LOC100130557	LOC154761
	LOC401588	LOC728024	PISD	SLC38A5	YPEL4	ZNF326	GOLGA6B	AIMP1	FLJ10213	INTS4
	JUB	MSI1	PCDHGB1	RANBP1	SAMD3	ZNF526	ZNF841	ZSCAN22	ARL 9.00	HOXD13
	PCDHGB8P	SLC9A10	C12orf69	C15orf51	C4orf33	P2RY10	PHACTR4	POLD3	ZNF7	NCR1
	LRRC4C	ADAMTS14	CCDC39	CLEC18A	MEI1	NAALADL2	PDZRN3	SLC17A9	TRIM66	GPA33
	C11orf21	CAMSAP1	CHORDC1	CITED4	PPAPDC1B	RAB33B	RND3	S100PBP	TLE4	TMTC2

	WNT1	OR1Q1	BCMO1	C9orf40	KLHL29	LRP2BP	NKG7	PCDHGB3	PTPN3	RNF182
	TJP1	UBE2Q2P1	DNAH6	OR10AD1	GRM4	C16orf87	CDH2	CTSW	FGF14	GZMK
	HOXB2	KCND1	NOLC1	TGM2	ASFMR1	DPF1	IFNA2	ADAMTS9	GRAPL	GTF2E2
	NIPAL4	SLC1A5	TEAD4	TSC1	ZNF200	SUMO1P1	ARL6IP6	C19orf23	C2CD4B	CARD14
	CXCL9	GOLGA2	LDHAL6A	LOC100190986	NIP7	RUNDC2C	DSC1	TRPA1	DNAH2	ZCCHC12
	SLC4A10	DYNC1LI2	DYRK2	EIF4EBP1	FPR2	GPM6A	KIAA1797	PCDH18	PFKFB3	TMEM45A
	TRA2A	HIST1H1D	PAK3	CDKN2B	ISCA1P1	ITGB3BP	PAFAH1B2	ROBO3	PCDHGC5	LDLR
	NFAT5	RCN1	STT3A	ZBTB5	FAM166A	WT1	C21orf122	EBF2	ISCA1	LENG8
	LOC390595	SLC7A5	TNK2	C10orf25	CD72	DIAPH2	FRG1	IL2RB	LNP1	LOC653566
	SF1	MYCNOS	OR6W1P	GPR174	RNASE10	AGAP5	CPT1C	DERL3	KIAA0467	MEGF11
	PCDHGA11	RGS3	TTC9C	UNC5CL	ABCC2	FDFT1	LBH	MSL3	NONO	PRR16
	ZBTB49	GSG1L	CYTL1	FZD6	KLRA1	RBPMS	ST8SIA1	RHOXF1	ARL4C	CXorf42
	DUSP4	ETV1	TSNAX-DISC1	C12orf56	GJB7	TRPM8	CCDC111	C11orf94	GPR61	ADAM23
	ANKRD49	GARNL3	LOC100272146	RBM7	CHRNA3	DNAJC12	MIAT	SC4MOL	TPBG	RPE65
	TNFRSF9	SAE1	YWHAZ	CDK2	DRAM1	FSTL1	NKTR	SAT1	ZNF321	CBR4
	CD3EAP	SLC25A37	TSPYL6	CAV1	MED17	HIST1H3F	PRINS	BIRC2	CEACAM22P	RUNX1
	SPARCL1	ZDHHC19	LTA	DNAJC25	ZNF202	ZNF22	ZNF250	IL18	NIPSNAP3B	TLL2
	CST1	CASP7	IL17REL	LAX1	ARRDC3	C15orf28	SCLT1	P2RX5	ANKK1	PLEKHF2
	PTGDR	ZNF235	CYP2B7P1	NDST3	C8orf4	NEK10	NRN1	IL1F7	SURF4	CDKN2AIP
	SCN11A	ATP6V1G1	C9orf131	KLF10	LGSN	TAS2R5	AHNAK2	PIK3R6	PIWIL4	IL23R
	JPH3	VAMP1	ICOS	ZNF169	GOLGA1	PDE7A	C8orf42			
**LOST FROM S3**	NPFFR2	AOX1	COL5A2	ESR2	GNB1	JMJD7-PLA2G4B	LOC613037	PARP6	POU2F3	PRDM10
	PSD3	RBM18	TIGIT	TMEM44	UPF3B	USP1	WDR5B	ZNF567	ZNF785	ZCCHC5
	MYF6	C12orf63	LGALS7B	PKP1	KRT79	ZNF80	DLK2	MCHR1	ITLN1	AQP1
	ATP2A1	BMP2	C11orf58	CBLB	CCDC123	CTNND1	DARC	ELF2	FAM102B	GIPC2
	IGFBP2	LOC400657	MC1R	MPP6	NAA35	NCRNA00176	PGBD4	PHF8	PRDM8	RCE1
	SGPL1	SIAH1	SLC28A1	TEAD2	TRPM6	USP48	WBSCR27	GPR141	ECEL1	C1QL4
	C4orf47	TSPAN19	AGER	APOL6	C6	CDCA4	CELF1	EEF1A2	FAM129B	GDPD3

	HAUS2	HNRNPC	HNRNPR	IER5	IGSF1	ISY1	ITPRIP	LIF	LOC221442	MFAP2
	NXF1	PCDHGA3	PKIA	PLK3	SIK2	SNRNP40	TLE1	TMEM39A	TRAF2	UBA6
	ZNF586	MAB21L2	ZAR 1.00	GLOD5	CLCN6	CLDN14	COL5A1	GGH	GPR173	IRAK2
	LOC100125556	LOC100271836	LOC283663	LOC440354	MDK	MGST1	NCRNA00085	PDGFRL	POLE3	RAB41
	RASSF2	SEC11C	SNORA8	STAG3L3	VWA5B2	ZNF691	ADAM7	CYP4A22	SLURP1	SSX8
	ZNF705A	RNF183	SLCO5A1	SPATC1	ISLR2	DNAH3	ACAT2	ARF4	AUH	C21orf91
	C6orf114	CST7	FAM82A2	FKBP10	GPR109B	HN1L	IKBKB	LRP5	LRRC15	MAK
	MCART2	NFKB1	PTPDC1	RDH5	RIMS3	RRAD	SLAMF7	SLC7A5P2	ZNF658	CPN1
	ADAM2	ZSCAN10	C1orf104	CELF5	CH25H	DAPP1	DDB2	DSC2	DTX4	LOC100131434
	MARCKSL1	MORC2	NNAT	PDXDC1	PPP1R1A	QSOX2	SPPL2A	TESK2	ZNF8	NPPA
	SAA4	WNT7B	TREH	ADA	ARHGAP31	ATM	BIRC3	C4orf46	CCDC52	CWF19L2
	DLL4	DPYSL3	FAM18A	FCGR2C	KLRK1	LOC440944	PATL1	PRRT2	WHSC1	SLC7A3
	PENK	CCT6P1	CDHR1	FASLG	HHIPL1	HLA-DQB1	HPSE	KIAA0020	LOC441454	MORF4L1
	RBMXL1	RINL	TCEA1	TRIM59	TTC21A	ZWILCH	SLC17A3	C11orf88	C12orf42	ADCY7
	BASP1	CASP3	CLEC18B	FAM192A	FAM22F	FAM45B	FBXO25	FLVCR1	LILRA4	LOC100272228
	NHJ37681	POLR3D	PPP2R2A	PTP4A3	PVRIG	RGS1	RUNX2	SERINC2	SFRS7	SMC5
	TARDBP	TF	TIA1	LOC729609	C2orf73	AKAP12	AOC2	CELF6	EIF2C3	GLIPR1
	KLKP1	LOC144571	LOC284837	MFHAS1	MIER1	MMP2	MS4A14	NKX3-1	NPR 2.00	PPP1R15B
	SEC24D	SERINC4	SERPINA3	SPSB1	STC1	TRA2B	UBAP1	VBP1	YARS	ZNF789
	NEU4	ACSL1	C16orf74	C4A	CASC5	CD244	CD6	DNAJC5B	FAM115C	INO80
	ISYNA1	KCNJ13	KIAA1432	MEX3B	PLXDC1	REEP3	RTN3	SLC4A7	SOX7	TRANK1
	UBE2R2	ZNF805	FAM123A	IRGM	TAS2R4	FLT3	ADAMTS7	ALPK3	CD180	CD247
	CLLU1	EEF1DP3	FAM118A	GBP5	KIAA1409	NLGN3	SFRS11	SGIP1	STIP1	ZNF137
	ZNF557	ZNF706	OR1J1	CNTN6	OR1J2	ANKRD20A3	BNIP3L	C1orf77	COL15A1	FAM22G
	FBN2	FNBP4	KDELR3	KIAA1045	LOC653113	MFSD10	MRRF	MURC	NFKBIZ	NPTX2
	PABPC1	PCDHGA12	PYHIN1	SH2D1A	SNHG3	NANOG	MYBPC2	FFAR3	C2orf84	CDC26
	CDK20	CEACAM19	CLEC2D	CYR61	GNL3	GPR183	HINFP	HSBP1	LOC100128842	MMP7
	NRG1	PDLIM4	RBM20	SAA2	WDR52	XBP1	C3orf65	ACO1	C1orf213	CCDC117

	CDK17	FAM120AOS	GNB4	GOLGA6L5	KDELR2	MASP2	NCRNA00167	SFRS13A	SGCB	TSTD2
	UBASH3B	UBD	VNN 1.00	TMEFF2	TDRD1	LPAR4	LY6H	ATCAY	AKAP13	CD22
	CG030	CPXM1	ETV6	FAM114A2	FAM13C	HAUS3	HIVEP1	LSM14A	NCRNA00095	NOP14
	SLC8A3	TM4SF20	APOC1P1	C10orf91	CGB8	HMX3	OR51A7	KCNK9	C10orf81	CCL20
	BREA2	C5orf58	GDF3	UGT3A2	CD80	CORO1C	DNM1	HLA-DQA1	HSPA6	KIR2DL1
	KLRB1	NAV3	NRBP2	PRPF39	PTDSS1	SCD	SIGLEC8	STC2	THAP9	TNFAIP6
	PCDHGC4	ARL 14.00	BEAN	C8ORFK29	CHD7	DCAF12	EP400NL	FANCC	FCRL6	FZD10
	GMEB1	HEY1	LSM 6.00	PPM1N	RBM5	RFTN2	RPL32P3	42248	TMEM151B	UNC5A
	ZNF732	OR51S1	TTPA	GCKR	PITX3	UCA1	ADAMTS2	AURKAPS1	C9orf130	CLDN9
	COL23A1	GCFC1	GPR85	IGSF9	KRT80	LOXL2	PCDHGA6	PHYHIPL	PRF1	SP140
	SPRY1	STK3	TAS2R14	GLP2R	WDR65	PAEP	ACBD3	C11orf61	C2CD4A	ECE1
	GFI1	GLE1	ITGB6	KIF21B	LOC100130557	LOC154761	LOC401588	LOC728024	PISD	RPL23AP53
	SLC38A5	YPEL4	ZNF326	GOLGA6B	AIMP1	FLJ10213	INTS4	JUB	MSI1	PCDHGB1
	RANBP1	SAMD3	SKIL	UBR5	ZNF841	ZSCAN22	ARL 9.00	HOXD13	PCDHGB8P	SLC9A10
	C12orf69	C15orf51	C4orf33	CCRL2	CHST1	P2RY10	PHACTR4	POLD3	RNF19A	ZNF7
	LRRC4C	PFKFB1	ADAMTS14	CARS	CCDC39	CLEC18A	DCLK3	ENTPD7	IL13RA1	MEI1
	NAALADL2	PDZRN3	SETX	SLC17A9	TRIM66	GPA33	C11orf21	CAMSAP1	CEBPG	CHORDC1
	CITED4	KLHL6	PPAPDC1B	RAB33B	RND3	S100PBP	TLE4	TMTC2	WNT1	OR1Q1
	BCMO1	C9orf40	KLHL29	LRP2BP	MMP11	NKG7	PCDHGB3	PTPN3	RNF182	TJP1
	UBE2Q2P1	UGT2A1	UHRF2	DNAH6	OR10AD1	GRM4	ADAM19	BUD13	C16orf87	CDH2
	CTSW	FGF14	GZMK	HOXB2	KCND1	NOLC1	PPP1R16B	TGM2	UBA3	ASFMR1
	DPF1	IFNA2	ADAMTS9	CHMP5	GRAPL	GTF2E2	NIPAL4	SLC1A5	TEAD4	TSC1
	ZNF200	SUMO1P1	ARL6IP6	C19orf23	C2CD4B	CARD14	CXCL9	GOLGA2	HAPLN3	LDHAL6A
	LOC100190986	NIP7	RUNDC2C	DSC1	TRPA1	DNAH2	ZCCHC12	FCAR	SLC4A10	AKAP5
	DYNC1LI2	DYRK2	EIF4EBP1	FPR2	GPM6A	GPRC5A	KIAA1797	PCDH18	PFKFB3	TMEM45A
	TRA2A	UBAP2	CCIN	HIST1H1D	PAK3	CDKN2B	ISCA1P1	ITGB3BP	PAFAH1B2	ROBO3
	IMPG1	LDLR	NFAT5	RCN1	RHOJ	STT3A	ZBTB5	ZNF782	FAM166A	WT1
	C21orf122	DNAH1	EBF2	ISCA1	LAMC2	LENG8	LOC390595	SLC7A5	TNK2	UGCG

	C10orf25	CD72	FRG1	IL2RB	LNP1	LOC653566	MTAP	PABPC3	SF1	MYCNOS
	OR6W1P	GPR174	RNASE10	AGAP5	CPT1C	DERL3	KIAA0467	KRT17	MEGF11	PCDHGA11
	RGS3	TTC9C	UNC5CL	FDFT1	LBH	MSL3	NONO	PRR16	ZBTB49	GSG1L
	CD70	CDC7	CTHRC1	CYTL1	FZD6	KLRA1	RBPMS	SET	ST8SIA1	CLDN6
	RHOXF1	ARL4C	C13orf33	CXorf42	DUSP4	ETV1	LOC729799	TSNAX-DISC1	C12orf56	GJB7
	TRPM8	CCDC111	KIAA1324	C11orf94	GPR61	ADAM23	ANKRD49	EOMES	GARNL3	LOC100272146
	RBM7	TEX10	CHRNA3	BCL11B	DNAJC12	MIAT	SC4MOL	TPBG	RPE65	TNFRSF9
	MTF2	SAE1	SLC2A14	YWHAZ	CDK2	DRAM1	FSTL1	NKTR	SAT1	ZNF321
	CBR4	CD3EAP	CNGB1	SLC25A37	TSPYL6	CAV1	MED17	TJP2	HIST1H3F	PRINS
	BIRC2	CEACAM22P	RUNX1	SPARCL1	ZDHHC19	LTA	DNAJC25	ZNF202	ZNF22	ZNF250
	IL18	NIPSNAP3B	CST1	CASP7	IL17REL	GNG10	PRPF38A	ARRDC3	C15orf28	CXCR4
	SCLT1	ERI1	P2RX5	ANKK1	PLEKHF2	PTGDR	ZNF235	CYP2B7P1	NDST3	C8orf4
	NEK10	NRN1	IL1F7	EIF2C2	SURF4	ATP8B1	CDKN2AIP	SCN11A	ATP6V1G1	C9orf131
	ZNF436	KLF10	LGSN	FAM76B	SLC2A3	TAS2R5	AHNAK2	PIK3R6	PIWIL4	IL23R
	ADAMTS4	JPH3	CHST6	VAMP1	ICOS	ZNF169	GOLGA1	PDE7A		
**CONSERVED IN S3/S4**	SSRP1	TKT	C19orf55	ACSL5	TACC1	KAL1	TNFRSF10B	TRMT12	ANKRD27	RIPK2
	SNORD1C	ZNF526	NCR1	PCDHGC5	DIAPH2	FCRL3	ABCC2	TLL2	MFI2	LAX1
	FAM49B	C8orf42								
**ACQUIRED IN S4**	BEND4	DEFA1B	MYH16	TDGF3	VIPR2	GTSF1L	KCNB2	NKX2-5	NPY5R	ANKRD2
	ARHGAP29	AS3MT	ATP5G2	AVPR2	BACH1	BAI1	BZW2	C19orf2	C21orf7	C6orf108
	CAV2	CCDC7	CD300E	CD96	CSDA	CSRP1	CUTA	CXCL13	DDX42	DDX52
	DHX33	DIAPH3	DIP2A	DNAH14	DSCAM	DUSP1	ECHDC1	EEF1G	EPB41L3	ERF
	ESYT2	FDXR	FGFR4	FMO3	GCK	GPATCH8	HLA-DRB5	HNF4G	IFIT5	IKZF5
	IL7R	IQCB1	IRS1	KCNH4	LEPR	LIMS1	LIMS2	LPIN3	LRRC34	LTB4R2
	LTBP3	MCPH1	MINK1	MLL4	MOXD1	MRPL2	MRPL37	MRPS5	MYLK	MYO9A
	NACAP1	NACA	NBPF16	NLRC3	NME2	NPM1	NR2C1	NUDT16P1	PHB2	PIAS4
	PLEKHA6	PLSCR1	PPHLN1	PPP1R14A	PTP4A1	PTTG1IP	PUF60	RABGGTB	RIN2	RIOK1
	ROPN1L	RPL13AP6	RPL13A	RPL22	RPL3	RPL4	RPS18	RPS3	RPS9	SERPINB6

	SERPIND1	SNCG	SPDYE3	SPN	STARD3NL	STAT5A	SYT2	TLR10	TP53	TPT1
	TSPAN2	UBE2CBP	USP54	USPL1	ZNF37B	ZNF584	ZSCAN2	C20orf200	LOC90586	ADH1A
	APOC3	ASMT	C2orf61	CACNG6	CGB	CPA1	CTSG	GYPA	KCNQ2	KCTD4
	LY6G6F	MBL2	MIXL1	NCRNA00230B	UGT2B10	GPR26	C10orf99	AAMP	ABCB1	ACER2
	ACTN2	AMBP	BCAR3	BEST3	BIRC7	C6orf154	C9orf68	CDC42EP4	CDKN2A	CNN1
	DCTN2	DNAH5	EEF1A1P9	EEF1A1	ETHE1	FBXO22	FCHO2	FHOD3	GALNT9	GLTSCR2
	HSPD1	IFIT2	IGF2R	ITGB3	ITPR3	KCNIP3	KDELR1	MCAM	MLLT10	MORF4
	NCRNA00188	NKRF	NLN	NPY1R	NSMCE2	PAPD4	PAX8	PCBD1	PLDN	PODXL2
	PPP2CB	RARRES1	RASSF3	RPL6	RPS20	RPS26	RPS29	RPS2	RPS4X	RPS5
	S100A1	SLC16A6	SLC44A4	STEAP2	TAGLN3	TANK	TARS2	TATDN1	TBX18	TGFBR2
	TMEM14C	TMEM67	TRNP1	TRPC4	UACA	VSTM1	ZNF259	ZSCAN12	CBWD5	LOC100127888
	LOC723809	SIGLEC6	ACP1	ACTA2	AGBL5	ARSG	C10orf84	C13orf1	C19orf33	C1orf107
	CDC42BPA	CRIM1	CWC25	CYSLTR2	DCTD	EEF1B2	EIF3H	GNPAT	HIF3A	HIPK4
	HSPB2	IL1RL2	IPO4	LOC100190938	LOC728190	LRCH1	NEDD1	NEK3	NSMCE4A	PCBP2
	PCID2	PDZK1	POLRMT	PPWD1	PSMB5	RNF41	RPL10A	RPL13P5	RPL18A	RPL37A
	RPL41	RPL7	RPLP0	RPS12	RPS16	RPS25	RPS3A	SEC14L1	SIK1	SLA2
	SLC35D2	SMAGP	SNAPC1	SNHG6	SUPT5H	TAGLN	TBCA	TGDS	TGOLN2	UTP23
	VNN 2.00	VPS54	YOD1							

E) List of lost, conserved and acquired genes across stages in KIRP

	DCLK1	CCL2	IL1R1	SOCS3	THBS1	PLEKHO2	PLAUR	GBP1	IL24	GJA1
	SLC2A3	SLC2A14	ICAM1	DCUN1D3	SELE	AKAP12	RHOU	THBD	PPP1R15A	INHBA
	SNAI1	STX11	MYADM	CHIC2	ADAMTS1	TDG	SERPINB1	GEM	C6orf150	RAP2B
	MMP19	DTX3L	ADAMTS4	IFI16	CEBPB	SPRY1	MAP7D3	TNFRSF1A	CSF1	DSE
	SEC24D	INHBB	KCTD10	KBTBD2	ETS1	PANX1	OBFC2A	PGM2	SERPINH1	IL6
	IRF8	ISG20	AHR	TNFAIP3	HELB	BATF3	PLK3	ETS2	SERPINB8	CD300E
	THBS2	C19orf59	SERPINB9	JKR37681	MCL1	AGFG1	RELT	GPR183	PIM1	KLF4
	FAM102B	SPHK1	ZFP36	KIAA1199	ALDH1L2	LIF	KIAA1949	FGF7	NLRP12	FOSB
	PTPN1	MMP25	LMNB1	OLFML2B	S100A3	CLCF1	RARA	PRDM1	DDX21	FFAR2
	IRF1	TP53BP2	BACH1	SECTM1	GZMB	CSF3	ADAMTS2	NHEDC2	SLC2A10	BCL2A1
	C1S	SHC1	AG2	RASSF2	TFE3	BCL3	SLC26A2	SERPINE1	BAZ1A	CCL7
	REG3G	STEAP4	LILRA5	CD69	ELF1	CLEC12A	DPPA4	MBNL1	MAMLD1	SBNO2
	C1R	FSTL3	OPN1MW	CD93	HAS2	HP	CCL8	CNN2	TRIB1	NAV3
**LOST FROM NORMAL**	GLIPR1	GDF5	CD300LB	HIVEP1	TXLNB	HRH1	AQP9	AFF3	NAA15	ADAM12
	LBR	SIGLEC5	COL1A2	PALLD	FAM159A	TSHZ2	ARID5B	GRAMD1A	SLFN11	FSTL1
	LILRA4	LHFPL2	STAT3	TSHZ3	RBMS1	LCP2	PYHIN1	LILRA3	TCF4	LPAR1
	CCDC80	CLDN6	KLF6	ZCCHC12	NFATC2	EMR3	C1orf38	COL16A1	ZYX	MMP2
	EMR2	ARF6	SVEP1	CDC42EP1	PLEKHG1	RASSF5	FAM49A	FBN1	SULT1C4	CLEC5A
	CSDAP1	DLC1	IL13RA1	CSF2RB	C5AR1	IL8	FFAR3	EGR3	MEX3C	WDR63
	PARP9	STK17B	ADAMTS12	SMAP2	C9orf21	CP	MXD1	RNASE2	ITK	ZNF474
	PODNL1	OSMR	FHL2	SAMHD1	WNT2B	CTTNBP2NL	SOX7	RGS1	ACBD3	CEP170
	CNR1	RND1	TMEM2	COL4A1	CDC42SE1	PODN	LUM	MAK	COL10A1	GPR171
	KRT1	VNN 3.00	TRAT1	GZMH	LILRB2	DUSP10	ARL4C	SLAMF1	HIVEP3	P2RY10
	GNL3L	TICAM1	CHST11	NAMPT	TDO2	IL7R	CCDC141	SERPINB2	CD86	NCOA7
	CD96	MEFV	TNFRSF9	PIM2	CD28	HAPLN3	CSGALNACT2	AFAP1	SAMD4A	EFCAB4B
	LYZ	SELL	NINJ2	KIAA1045	LAMA4	ULBP2	OASL	COL3A1	P704P	TMEM71
	ACTR3	DACT1	NLRP3	AXL	SLIT3	PTPRE	HAVCR1	VNN 2.00	NUMBL	TMEM217

	COL6A3	ADD2	SPSB1	CCL18	LZTS1	CDKN3	ATP8B2	RHOH	TNFSF8	S100A9
	CLEC4C	HECA	PDE4B	CXCR4	TUBA1A	GATA6	MS4A7	ANKRD1	CFH	NCF2
	FCGR1B	PDLIM1	FCGR1C	TIMP2	CSF3R	CRISPLD2	NFE2	KLRD1	C9orf150	ARL5B
	CSRNP1	GUCY1A2	RUNX2	RUNX3	GPR65	CD300LF	ABL2	BEST1	OSM	GNA13
	RFTN1	SOX4	SLAMF6	PTPRC	DCN	AEBP1	PRRX1	PRIC285	C1orf198	TNMD
	BHLHE40	TNFSF9	RASGEF1B	EPB41L2	LDLRAD3	CFHR1	IL2RA	FRMD6	MUSK	PLEK
	CD209	MFSD2B	ELF3	CXCR6	CD300C	ENAH	CD97	LOC150786	MCART6	SH3PXD2B
	PPRC1	ANTXR1	ZSWIM4	ART4	SRF	HAS1	FPR1	IKZF3	LRG1	TAC1
	PTPN22	UBE2J1	TAGAP	ANXA1	LMNA	FAIM3	CRTAM	EMILIN2	MICAL2	ELK3
	ARFGAP3	FAM65B	GPR97	SIGLEC6	FGR	RET	BTK	LOC153910	DYRK3	COL15A1
	TCF7	SPRY2	CDK2	SOX9	NAALAD2	CD44	COL5A1	ALAS1	RNF122	LOC541471
	GPA33	TNFSF13B	TLR8	ADAMTS3	CD247	TNFRSF1B	TRPC4	TEC	OMD	COL8A1
	FKBP10	CBFB	GALNT5	AFF2	NALCN	CFP	PLTP	CD163	FCN1	LCP1
	APOBEC3C	GPR112	RAET1L	CDH11	PTENP1	GZMK	CHRDL2	COL1A1	CREB5	AOAH
	NKAIN1	RGS4	MPEG1	OGFRL1	PTGIR	RHPN2	ADA	CST7	PADI1	TREML2
	C13orf31	THEMIS	GRIN2A	LILRB4	GZMA	IL4R	XCR1	PGM2L1	PPYR1	C7orf58
	NPFFR2	CD3G	PLAC8	DOCK2	GBP2	TMEM119	ZFP36L2	PRF1	FUT4	TBX21
	ZBTB8A	CHSY1	IL13	RUNX1	SLC32A1	CDKN1A	IL7	SELPLG	VIM	PEA15
	ANUBL1	SEMA3A	KIF18A	KLHL6	FCGR3A	HSF5	LAPTM5	CELF2	APOL6	EVI2B
	ADAMDEC1	TEAD4	CNN3	LY9	SACS	CFHR3	APOBEC3B	RFX2	CD302	PCDHGC3
	FPR3	FOSL1	WDR82	JAK1	RGS16	CCL5	ITGB1	KIR2DL3	SMARCA5	CD7
	RNASE3	AEN	NCRNA00152	S100Z	FMNL1	DYRK2	CD84	XYLT1	TULP3	HCK
	CFHR4	CD53	NFKBIZ	APOBEC3F	CD8A	ADAM19	CAMSAP1	POU2F2	LRRC25	FCGR2A
	ODF3L1	CD48	FCGR1A	IL10RA	PVT1	ZFPM2	ANKRD50	CRLF3	MICALCL	GIT2
	LMCD1	ITGBL1	HTR7	SERTAD1	STK4	B3GNT5	MACC1	SH2D1A	ARL 11.00	TEAD3
	RAB8B	EPHA2	SPC25	RGS18	FYB	ITGA4	SH2B3	TIGIT	TMEM155	C17orf87
	JUNB	KLRB1	SP1	DDX5	TCL1A	TMEM149	C6orf145	SIRPB2	WDR44	CTSS
	LILRB3	TNC	ASAP1	CEACAM3	LAMP3	SEMA7A	TIMP1	HAS2AS	CYBB	CD8B

	GPR84	BICD1	GRRP1	CALU	IFNG	TGFB3	ST8SIA4	GBP5	CNR2	COL5A2
	DARC	CA9	C10orf10	LRP1	IFNGR2	SPARC	DCP1A	GGT5	PIWIL4	ITPKC
	IRF9	TPM3	CLEC17A	LAX1	TGFBR1	SIGLEC9	CHSY3	GXYLT2	TMEM59L	HEPH
	MDM2	TNFRSF12A	NID2	GNA15	KRT80	NCKAP1L	MYO1F	LAMA2	RARRES3	TUBA1C
	MDFI	FCER2	APOBEC3D	SFRP2	EMB	KLF16	MSN	EVI2A	ARHGAP17	TRIM22
	CD200R1	SFRS13B	FJX1	ARRDC5	LILRB5	CCDC88A	XRCC2	PAG1	CD5	SIX6
	MAP3K14	CNKSR3	MSC	MYD88	TNFRSF10B	B4GALT5	LILRB1	RBMXL1	FGF10	RRAD
	LHFP	COL11A1	CSDA	CD1A	SOCS5	PCOLCE	PHLDA1	GRAP2	HLA-DRA	JAK3
	LIMK2	C9orf91	NOD2	IKZF1	WIPF1	PHF20	ULBP1	CLECL1	CCL21	BASP1
	GALNT1	VCAM1	CCR3	CYBRD1	MAFF	MS4A3	SPN	ZNF831	CHRM2	TMEM156
	CD33	FGD2	ARHGAP15	CD180	CARD11	CD40LG	ARL 14.00	HLA-H	MAP3K7	ASIP
	AMICA1	PIP5K1A	HCLS1	C19orf38	ARID5A	NUP98	DUSP14	VWF	MTHFD2	ZCCHC6
	FLI1	CTSG	FCGR2B	GNAO1	C14orf43	PCDHB12	ITGB3	GAPT	RYBP	MT1A
	XIRP1	RAB32	CXCR2	TWIST1	NID1	CD4	KDM6B	CABP2	MS4A1	GADD45B
	KPNA2	IL2RB	ZHX2	FST	HMGA1	RCN3	CCDC120	FGFR1	PAPPA	ICOS
	TMEM39A	NRP1	C2orf77	HAS3	CCDC50	RAC2	FCRL6	CLEC4A	MLKL	GPRIN1
	RHOB	PDIA4	DISC1	VCL	CPXM1	ARHGAP30	FKBP7	IL18BP	CYTIP	FCRLA
	EMILIN1	C12orf35	HLA-DOA	KLF7	PIK3CG	RASA3	SFRP4	DNAJB1	RELB	COL29A1
	ZFP36L1	TLR4	HLA-DOB	NGFR	SIX3	SLA2	SMCHD1	SLFN5	LSP1	ALOX5AP
	ASPHD1	GNAI3	NKG7	STK10	COLEC12	GFRA1	IRF4	ETV6	BTLA	SERTAD2
	MMP11	MNDA	SLAMF7	TNFAIP1	SOD2	MALT1	FAM53C	ARHGEF6	CLDN4	TLR10
	DENND5A	SIGLEC7	WDFY4	MGP	ENTPD7	PPP1R12A	EMP1	PGK 2.00	CTLA4	CD3D
	IL34	TRIM38	RAP1B	TRAM2	NCF1B	VCAN	SYNCRIP	NRP2	SLC1A3	PXDN
	ACAP1	PLA2G2D	EDN2	CD3E	ICAM4	PRG2	KLRC2	ARPC5	SGTB	TXNDC5
	SCARF2	CXCR2P1	MFHAS1	ISLR	TTLL7	TSC22D2	TNFRSF13C	NFAM1	CSRNP2	TMEM45A
	CACNA1I	LILRA6	NLRP10	SEL1L3	IRF7	PAK3	GFRA2	FRZB	RBMS3	TMEM212
	FCRL1	REG1A	SIRPD	LTA	PARP15	SERPINF1	RELA	GRM4	TBCCD1	SASH3
	FIGF	CASP4	BAG5	HGF	DDX58	FLJ43390	GPR141	CILP	S100A2	ITGB2

	SLC9A9	FAM83G	RAB23	LIMD2	RCSD1	HSP90B3P	LY86	FCRL2	EEPD1	SCT
	TMEM173	GFI1	ALOX15	PRR11	USP6NL	HERPUD2	ITSN1	TNFSF11	KLRK1	SIGLEC8
	BMPER	KLF10	F13A1	IFITM1	MYOD1	ARNTL2	KIAA0355	MRC1	RGPD8	WISP2
	CLEC7A	PIK3R5	CD52	KIAA0802	ADCY7	C16orf72	SIRPB1	ITGAM	STXBP1	FCRL5
	FRMD4B	FAM129C	C4orf7	KIR3DL2	BMP1	GPR174	FMOD	IL18RAP	CD163L1	HLA-DQA2
	FCAR	C13orf18	FANCA	C1orf162	PRICKLE1	LOC652276	COL4A2	LAIR1	MMP23A	GLI1
	ZNF460	SIGLEC14	DTHD1	BMP2	IL27RA	DKK 3.00	CENPA	DOCK11	CCNB2	LIX1L
	KIAA0748	CTGF	YWHAG	CSTA	KLRC1	S100A16	UPP1	EBF1	LOC647121	DNAJB11
	TREM1	S1PR2	VAV1	SNX20	C2orf89	POU2AF1	CRP	SPTA1	PIF1	ADRBK2
	ABI3BP	CD22	HKDC1	RBM24	MC4R	ITGAD	SPON2	ABCA6	MYO1G	ARL13B
	CLC	DMP1	EOMES	HK3	CDKN2B	ACTRT2	ASZ1	KRT26	KRTAP4-11	OR2W5
	OR52E4	POU4F2	PRAMEF20	PRAMEF4	SP9	VGLL2	IL16	NFKBIE	MEF2D	ARHGAP11A
	CRHR1	ITGA5	BAT2L2	FAM129A	CD14	HSP90B1	SAMD3	IRAK3	ETV7	EMR1
	PCDHGA5	CD2	WEE1	MS4A2	C1QC	SIRT1	CPA3	TXLNA	MATN3	PMAIP1
	RIPK1	C17orf64	ACTB	MOXD1	ASCC3	SCN1B	OTUD4	BARD1	PRKCB	PRPF38A
	EREG	PARVG	IL10	UGDH	GLIPR2	CDK17	IL2RG	C9orf110	AGAP2	CXorf21
	SYN1	NCF1C	PCDHB15	BRCA2	RAPH1	TTC24	SIGLEC10	ERCC6L	IL17REL	LY96
	NFKB2	RNASE6	GNG2	RARRES1	PRAM1	TTC39A	WAS	ABHD5	CORO1A	NBPF14
	HCST	SYNC	PRELP	LPGAT1	P2RX5	GMIP	IRAK2	ITGAL	LYN	TAS2R43
	IFNGR1	CDH6	LAT2	NCR1	PYGL	RAI14	DDHD1	GPSM3	TFPI2	C12orf5
	MSR1	FOXN2	GPR55	SKIL	SP140	VSIG4	YWHAZ	IER3	TPSAB1	ZNF80
	MEX3A	CD6	PARP8	ABCC9	CTSW	PVR	CXCL11	NFYA	CDC42SE2	GGTA1
	FILIP1L	KDM5A	NLRC3	LRRC15	CPZ	MAPRE1	GPC4	CMA1	DGKD	IL21R
	TUBB6	CD38	SIGLECP3	KLHL29	S100B	CD74	FERMT3	HRK	TLR2	INPP5D
	LOC283856	CUX2	SLC8A3	POSTN	C1QB	GMFG	EFNA1	CLEC9A	TYROBP	MS4A6A
	HLA-E	C16orf54	APBB1IP	GAB2	TM4SF4	GPR161	STAT4	LOC727896	KCTD20	DEGS1
	PPFIBP1	AOC3	NOTCH2	ELF4	RAB42	FAM198B	CHRNA6	LY6H	FCER1G	ARHGDIB
	FOXO3	ELANE	OAS3	RTKN2	STAT1	HDX	BEND6	KDM5B	FAM114A1	DLGAP1

	ADCYAP1R1	SERPING1	UBC	C6orf204	C1orf200	HOPX	MKI67IP	CHRNE	FCGR2C	CMTM3
	DDR2	PDGFRA	TLR6	PRAME	TRH	KLF5	CLDN9	FNDC1	LOC641298	HRH4
	SPIB	PTGS2	KIF21B	ZNF365	REG1B	CXCR1	MAP4K1	SIGLEC1	FYTTD1	PPM1D
	FAR2	SIRPG	IL1B	OSR1	PTCRA	ITGAX	CDC27	SCUBE2	GPR114	PCDHB14
	SLAMF8	HLA-A	CD1C	KRT8	CEACAM4	NLGN2	CDC7	HLA-DPB1	CYTSB	CCR2
	CADM3	TOP2A	CCL13	FHL3	HSPA5	CCR5	GBGT1	CAPZA1	FAP	GLI2
	SDK1	TMEM196	ATP2B4	MAP7D1	PTEN	PLEKHO1	INMT	IFIT3	ZNF800	AREG
	CTHRC1	MEIS3	BATF	ANP32D	C12orf4	TGFBI	B3GNT7	CRISPLD1	CLIC2	CHD4
	IL9R	FPR2	LOC100130331	NFKB1	GMEB1	C2orf18	RASSF3	DUSP22	RLTPR	S100A12
	CNTLN	CALD1	UBASH3A	NUFIP2	FKBP11	CD1D	LPAR4	OLFML1	FCRL3	SLC24A4
	LATS2	CLEC10A	RBP1	KCNA3	SLC7A11	IL33	EXT1	GABPB1	HTR2B	NAPSB
	TAGLN2	SH2D2A	LDLR	DOCK10	ADAMTSL4	C2CD4B	ANGPTL7	ZNF217	A2M	ZNF749
	KCNF1	PDGFRL	NLRC5	CAP2	IFNAR2	HLA-DPA1	CDCA7	KIAA1210	NR2F1	ZNF101
	CD5L	PCDHB10	PTPN12	RNF217	CD72	DNAJC5B	PLA2G7	NCF1	TBC1D2B	SWAP70
	KAAG1	ACTN1	C1QTNF1	PDLIM5	TMC8	SRP72	UNC13D	KLHL25	SELP	CXCL6
	BTN2A2	ZNF643	CD37	DEPDC1	PNRC1	APOB48R	BLM	AKNA	NELL2	TLE1
	MELK	SIT 1.00	CXCR3	FGFBP2	NIN	DERL1	MOBP	MEI1	PLB1	PCDHB18
	C3AR1	JMJD1C	HLA-DPB2	GLT8D2	MED17	MRC2	KIF2A	SCGN	MAP4K4	PIK3R6
	BAI2	HYOU1	PLD2	MED6	TES	KCMF1	LILRA2	MORF4L2	VASH2	IFITM3
	CD300A	LRRN1	CLEC2B	TAP1	RGL4	C12orf68	LOC285205	CTSZ	SLC37A2	PTPN2
	GTF2E2	IL11	SNCB	CITED1	ABCA9	CASP5	CHD1	LST1	TLR7	PBRM1
	TNXB	CHORDC1	CDON	SLC46A2	C5orf58	GPR176	CFB	VASP	MS4A4A	B4GALT1
	MIDN	ZEB2	SLCO5A1	MIXL1	PAPLN	DDX60L	C17orf96	EZH2	CXCL10	MFAP4
	FILIP1	ZNF267	FAM7A3	ZNF683	TUBA1B	CSF1R	SMC6	CD27	LOXL1	PAX5
	C12orf61	RNF138	NUDT10	GPR15	TTYH2	ARAP2	CD200	CD177	SH3GL1	C13orf29
	ATP11C	GDF11	HPVC1	CDC42EP4	STATH	FKBP14	CCR6	C6orf174	FUT8	CYP19A1
	CBL	LOC644936	EFEMP1	BCL10	C17orf62	GPR3	PLEKHA4	C1QA	BTN3A2	LOXL3
	TGFB2	PSG1	ODF2	HNRNPF	BUB1	C1RL	MKI67	PGA4	ITPRIPL1	MTA2

	KBTBD8	LRRC8B	FAM126A	LRRC8E	C1QL2	MAP1LC3C	P2RY8	C20orf103	UTP14A	FAM116A
	NLRC4	MEGF11	CSF2RA	ZNF595	CCR4	PHACTR2	FSHR	SIDT1	MID1	C19orf75
	DAD1L	FAM181A	GRXCR1	GUCY2F	IL28A	KRTAP13-4	NKX6-3	OR52D1	PRG3	PRO1768
	RBMY1A3P	SDC4P	SNORA31	TARM1	TAS2R9	GZMM	PCDHGB2	DOK2	NCF4	FLNA
	CLEC4D	CASP8	FMO3	FCER1A	CASP3	LOC100133991	HIVEP2	EIF2AK3	SLA	RHOG
	MYLK	GRK6	FAM101B	NLGN4X	NCEH1	TRPC3	UNC5CL	TWIST2	C10orf105	NCR3
	ZNF35	CSRP3	PDCD1	FCHO1	ST8SIA1	CEACAM8	MAP3K5	NKAIN4	PTCH2	LOC284441
	XBP1	MMP17	CCR7	BCL6	ZNF469	PMEPA1	UGGT1	OR10A3	ANTXR2	CXorf22
	DLGAP4	MAP1LC3B	FEZ1	KIAA1324	CCDC146	ANXA2P3	SEMA4B	SIPA1L2	LTB	LILRA1
	WISP1	LOH3CR2A	SH3PXD2A	CCL19	PIK3CD	TINF2	AP4E1	ZNF410	ANKRD57	APAF1
	CSF2	LOC339524	ALMS1	IGSF6	B2M	NCK1	RAB20	C2	CDCA2	EPC2
	MICB	MAP3K12	C7	RNF182	PTGDR	LEF1	FNBP1	KIF3C	GATS	CXCR5
	PSME4	S100A8	IL17RA	S1PR4	C1QTNF6	GTDC1	ZNF878	UBE2H	HNRNPAB	CXCL1
	UCP1	LOC100128164	STK38L	C20orf141	SLC7A2	BLK	BCAT1	CYSLTR1	IWS1	CD19
	FAM72D	ZBP1	LYPD1	MLL5	DIRC2	ARHGAP9	BMP2K	LETM2	BRPF1	IDO1
	PTGER2	PNMA2	CILP2	NDC80	KCNQ3	ISLR2	CMTM2	IGLON5	GPR37	NEXN
	COL6A2	SCG2	LTBP2	EBI3	SPI1	C4orf11	CCDC140	DEFB103B	MUC7	SPAG11A
	UBTFL1	TNFRSF13B	HS3ST1	CYTH4	ERAP1	SLC1A7	PSD3	DTL	PLXDC1	NUPL1
	IKZF4	MACF1	CD80	TBX20	C4orf6	C22orf9	LOC285830	ENDOD1	TRPA1	NNMT
	IGJ	LYVE1	IER5	RELL1	TLR9	IMPDH2	PDE4D	CSNK1A1	FLJ16779	TEX19
	TRANK1	PLEKHG2	TRPV2	BCL11A	RTN1	HAUS2	PI15	OTOP3	FAM171B	TMEM185B
	GKN1	SDC3	RBM9	TNFAIP2	SH3BP1	GPR18	RFPL4A	C19orf22	TAP2	PLIN3
	ADH1A	ARL 9.00	CASP1	APOBEC3G	SLC16A6	CHI3L2	ST3GAL2	LRRK1	SH3GLB1	KRTAP1-5
	PPP4R1	VWCE	RASGRP4	NRAS	ALOX5	RASAL3	FHL1	IGDCC4	DOK3	BZW1
	TRA2B	RAB33A	HDC	LPXN	EXTL1	DTNA	FLRT2	C1orf150	CCDC9	PGLYRP1
	EVI5	RPGRIP1	COL14A1	RASD2	CIITA	STYX	CASS4	FCHSD2	PCDH17	NPC1
	WTAP	E2F2	EGR1	PPIG	DEF6	EMP3	TTC39C	ZNF532	MGC29506	GPR56
	RAB31	CDKN2BAS	SPAG1	RBBP6	RPL12	PAQR4	SEMA4C	CSNK1G3	TIAM1	D4S234E

	IL4	ITGA1	TIFAB	LOC152225	ACTR2	ZAP70	RGS22	ZPBP2	SVIL	KCTD11
	TNFRSF10A	RANBP17	INSL6	CNKSR2	GNB1	BRCA1	TBX5	CCL4	KIAA0226	CYLD
	PITPNM1	HIF1A	PLCXD2	TBXAS1	SDC4	LOC100188949	TUBB3	GRK5	HMHA1	CH25H
	BTBD19	DKK 2.00	DIAPH2	LOC283070	FOXG1	JLD37226	KCNK6	LRRC4C	LCK	HPR
	CCIN	ARRB2	ADRA2A	GPR142	SEZ6L	CHRD	KCNH8	PSTPIP1	FAM72B	PROCR
	PER2	MNT	OSCAR	ZAK	PPARD	TAF5L	CDC6	ITPRIP	C12orf63	C3orf64
	SPATA12	CCDC46	NEIL3	TRPM2	CLSPN	SLIT1	CD101	PDCD1LG2	MFAP2	DLX4
	MUC12	FBXW7	ATP2B1	ELAVL4	LRRC32	SLC38A8	SYT16	DCBLD2	BCL9	IQGAP3
	TXNDC3	CCND2	RSU1	SH3RF3	C6orf25	CCL11	SERPINA3	CLSTN2	UNC5A	ETV3
	RALGDS	EFHD2	IL18R1	OSBPL10	NT5DC3	PIP4K2A	SLC10A6	CHODL	PROK2	PPP3R1
	TRIM62	ZBTB32	CNTFR	CAPN11	RGS19	GVIN1	LOC96610	PSG9	CCL20	KCNA2
	NFIX	ATF7	PRKCH	NCOA3	ANKRD44	INTS8	KEL	HR	MTF1	NECAP2
	ZDHHC18	LOC100130386	ARMC3	FOLR2	TLE4	DGKH	HRCT1	SSC5D	DENND4A	ITGA9
	DLGAP5	MARCKSL1	MYBPC2	MYBL2	HMCN1	CCDC102B	RASSF1	ODZ3	FCGR3B	C14orf174
	ADCY3	CECR6	SERPINB11	KIF2C	HS3ST2	SOCS1	RIN1	TMCC1	CD79A	BTBD10
	ERI1	MAP1A	UBE2D1	TMSB10	RBM12	LOC442308	ZNF597	ZNF644	R3HDM1	SGMS2
	SLC35C1	MOBKL2A	TLR1	TNKS2	OR52N4	DCX	KRT24	FEM1B	MMP10	VSIG1
	VIPR2	RGS9	TRERF1	GINS4	HHIPL1	ADIPOQ	UBD	TEX11	RNF125	DNAJC2
	ADORA3	ISG20L2	TNIK	SP110	LAIR2	PHF20L1	BIN2	SLC41A2	DPY19L1	TSPAN18
	NMI	CENPF	RAPGEF5	MAN2B1	AZIN1	NUP160	PDGFRB	MEX3D	DONSON	ESYT1
	COL21A1	B3GALNT2	EIF5B	TEP1	C14orf145	KRT18	PRND	NAV1	PLXDC2	PRR7
	BTN3A3	RPL10	AMMECR1L	C10orf54	SHISA5	SYTL1	LONRF1	RGS14	DCAF12	XCL1
	KIF5A	FUT7	RAB35	CLDN1	LOXL2	NOL8	PSTPIP2	GALNT7	PABPC4	HNRNPA1L2
	LOC283731	DPEP2	IL31RA	HK2	RAB30	LOC100233209	TPM1	CCDC96	PREX1	KLF8
	HEYL	RPS2	CXCL9	PLBD2	EPSTI1	HNRNPA0	ADAM17	ACTL8	LGALS12	MAGED4B
	SGCD	CD276	ENC1	CDC37L1	HOOK3	KCTD4	SORBS2	MAPKAPK2	PLCB4	FAS
	GNB4	BCAR1	TMEM43	FYCO1	FERMT2	CXCL16	ANXA2P1	CXCL13	PITPNB	SYCP3
	ZCCHC24	TRIM40	FOSL2	PLSCR1	C10orf18	LOC145820	PTGER4	FBLIM1	MET	CLEC12B

	LOC400759	PCDHB6	TRAF3IP3	PLCH1	UAM40057	SOBP	ARHGAP20	NKX2-2	GLIS3	SMOC1
	LYG1	TBC1D10C	SHOC2	KLRG1	RRM2	TMEM179	FOXF2	RXRG	ZNF487	NBEA
	MIAT	LIPN	FBLL1	PUS3	MMD	PLD4	GRIA1	FAM26F	DKFZp761E198	XCL2
	LOC606724	LIMK1	CRLF1	CHST7	ROCK1	ARHGAP25	LGALS9	ZNF609	FOLR4	C11orf21
	KIF4A	PAK1IP1	SOX21	PLGLA	TRIM9	ADCYAP1	AKAP13	LAYN	HUS1B	XPO1
	RSPH4A	RNF41	CACNA1G	C10orf128	GPR124	SAA1	LRRC41	FAM135A	IL1A	PTPRCAP
	BAZ1B	NEGR1	ATP10D	FAM160A2	SETX	HCP5	NFE2L2	TCF12	ADRB3	C6orf114
	EDNRB	JUND	TNIP2	LGALS9B	C2orf85	SOAT1	SPON1	COLEC10	IGLL1	HHIP
	CBFA2T3	FAM49B	RGMA	STAT6	PIN1L	TUSC5	MPO	PCDH19	MAS1L	BMP5
	PIK3AP1	C11orf9	FAM46A	ACHE	DERL3	MYB	ACTN3	UHRF1	ITGB8	IL1F7
	COL8A2	CDC20	LYST	PTPN7	ADAM9	PURB	IL32	STAT2	HVCN1	ITGAV
	LOC100129550	ATF3	CD244	FASLG	IGFN1	GAL3ST4	COMP	KIF14	TRAF4	PCDHB13
	KIAA0408	SPRY4	REL	KIFC1	POLR3C	GNL2	PGM3	CD99	GPR132	POTEE
	SMURF1	RAB9B	ADAM28	KCNB2	C15orf42	FBLN7	ARHGAP10	FOXO3B	CDR2	CCDC109B
	ROPN1L	SLC18A2	POLR2D	RASSF6	ALPK3	HRASLS5	PML	ANKFN1	LECT2	SHROOM2
	TOPBP1	ZDHHC5	GM2A	GCNT1	SDS	CYP1B1	CLDND1	RPL31P11	ERN 1.00	CD1E
	P2RX7	CAMK2D	SETD7	MDK	COL12A1	UVRAG	GNLY	HLA-F	STK17A	CWC22
	PLCB2	SP7	MEF2C	TNFRSF17	TTC29	C11orf82	DNALI1	DCHS2	TREML4	ADAM8
	IL23A	KRT86	DDX18	CDC42EP3	MID2	PDE5A	BMPR2	ACAN	SMOX	KCNK17
	AMOTL1	ARID4B	NAB1	CORO7	FAM9B	PCDHB2	ACAP2	CRYAB	FAM78A	CD226
	PMM2	HSPB6	ERF	XKR3	ADAMTS16	FAM110B	TECTB	HJURP	CDK12	PRKAG3
	C4orf50	ACER3	PLIN4	C7orf57	NFE2L3	LTBP4	CCDC97	CYP2C9	OAS2	TPRG1
	ASPM	KIAA0922	TWF2	PHF19	PIGR	MYO3A	ASF1B	NCRNA00093	SRPX	ICAM3
	HLA-B	HSD3B1	CFL1	MX2	LRRC8C	SPAST	CCL24	GPR4	YWHAB	ENOX2
	SENP1	CA13	ARHGEF38	C17orf60	PLP1	C20orf186	KCNN4	WDR26	ITGA11	REPS1
	REEP1	PLAC8L1	MYO1D	PAPOLG	MCHR2	PDZD4	UBXN2A	MEOX1	FBXO41	APOL4
	HSD17B6	GNAI2	AKAP5	CCDC55	FAM71B	CNGB1	C17orf53	DKFZP434L187	ANLN	SPATA5
	PPBPL2	OSBPL11	DNAJA1	EPB41	SYT12	EID3	TCTEX1D4	TRPC2	COX6B2	NFIA

	TNFAIP8	SEZ6L2	KIAA0040	MEIS3P1	PPAP2C	MKX	C17orf67	KIAA1274	37135	DPYSL4
	AFF4	CEBPD	YTHDF1	LOC100126784	PTN	RIOK1	CCNA1	LRRN4	KIAA1211	RIT2
	FOXP3	PTRF	PSD2	KCNT2	FAM105B	AIM2	ODC1	STMN2	BIRC3	PRR24
	OPRK1	ALPK2	CLDN3	ZNF423	TIMD4	SYTL5	GABRA1	MIOS	FAM110C	FXYD5
	MSH6	FAM180B	S1PR3	SLCO1C1	ARHGAP26	HSPA6	ADAP2	CARS	B4GALNT4	RPS8
	RBBP8	SLFN12L	IPMK	PSG7	ASPN	HTR2A	P2RY13	MMP7	TFPI	ULBP3
	LOC284276	C1orf173	MGC45800	YPEL2	PPP1R1B	XRCC5	BCL11B	RHOQ	ADAM6	KIAA0125
	CRISP3	LOC645166	FOXD4L6	EZR	KIF7	INPP1	MMP23B	PTPRK	KIF20A	UBA6
	CD248	TNNI2	ODF3B	STK38	GAB3	NRBP1	C14orf106	SETBP1	TYMP	CHPF2
	IL12B	CEP55	JUN	LOC653653	POU4F3	REV3L	CHST15	CSNK1G1	WDR75	C10orf26
	CAP1	FOXP1	ZNF280B	SRGN	OGN	VANGL2	ROR1	SMNDC1	KRT17	CRY1
	MAP1LC3B2	PCDHB7	MEGF10	ADM	TP53	MXRA8	GABRR1	SEMG2	NEU1	LHX5
	SGK269	CCDC88B	ZRANB3	LRRN3	MAP3K2	TPX2	OR10V1	PF4	FAM113B	AKIRIN1
	EDN1	FAM111B	RBM38	OTOF	C13orf36	TAF1A	UBXN10	LEFTY2	LBH	FRMD5
	CRK	RIMKLB	PDGFA	FBLN1	TCN1	SPRED1	NIPAL4	SIRPA	DEPDC6	IL15RA
	HSPH1	PLEKHA2	TMEM163	LAMC3	MED13L	TMEM154	HLA-DQA1	LOC282997	CAMK1D	NPFFR1
	FAM55C	PLEKHA1	PRDM8	DIP2B	ARG1	YWHAH	DDX60	CCDC19	EPC1	SPP1
	WASF1	PACSIN1	AP1S2	NIP7	C12orf45	TSPYL6	LOC399959	PRICKLE2	RAB43	SNAI3
	TTC28	MPZL1	LIPE	PRSS21	CD79B	ECM2	RADIL	CFD	KDSR	C2orf39
	FRMD8	PSMB9	PHTF2	DYRK1A	ZDHHC20	HYDIN	SLC39A1	GPR34	SKI	STAC3
	MEIS1	TMEM151A	HAVCR2	FOXA1	AXIN2	JMJD6	PMP22	ZNF876P	NTF3	CD1B
	PLEKHM1	CDH22	THG1L	SLC17A2	CPM	NPTXR	YAP1	CBLB	MLC1	FOXC2
	RPL13AP20	ARMC4	SCN5A	P2RY14	SPOCD1	GUCY2D	PELI1	ARIH1	EIF2C4	PPIL4
	PDLIM7	SNTB2	KCNIP2	ZSWIM6	SHANK1	ZNF430	DAAM1	LOC723972	IFITM2	EGR4
	PPPDE1	SMAP1	ELMO1	HAR1B	HTR3A	NPTX2	BTN3A1	MYOF	TMSB15B	ZNF608
	TAF1D	TGIF1	SLC24A3	KCNQ5	EDNRA	SAMD4B	S1PR5	PPP1R3B	ENTHD1	IL12RB1
	HIC1	C1orf124	JQR36951	CLDN14	RCC1	RTP4	PAM	C11orf84	VPREB3	UGT2B15
	PHF16	RRP12	LYPD5	LRRC37A2	C14orf45	CCR8	EFEMP2	GUCY2E	LEPREL2	ARL4A

	RGS20	TMEM158	PTPN6	DNTTIP2	VPREB1	H6PD	FAM40B	MYO9B	DPYSL3	KIR3DL1
	FEV	MSRB3	ADNP2	KDELC1	SHD	ARHGAP23	SLC15A3	NRIP1	DCLRE1B	OXTR
	EMR4P	WDR69	HSPA1B	KLHL5	FGD6	C6orf115	MARCKS	UCN2	IQGAP1	DGKA
	ZNF165	EMID1	TTPAL	CDH19	ARF4	MED14	GATAD2A	IL12RB2	NCAPH	SGK223
	PSMB10	FGF13	DNAJC3	PRC1	CPT1C	TRPV5	EHD2	MAP3K8	RNASE1	BTN1A1
	C6	KRT78	APOL2	RPGR	PAMR1	PTX3	PKNOX1	NAP1L1	TADA2A	GAS2L3
	SOCS6	TNFAIP8L2	CLIC1	SYNPO2	SLC1A4	IKZF2	BGN	IL22RA2	WARS	PHEX
	C9orf25	KPNA4	NONO	DEM 1.00	OPN3	NUP188	HLA-DMA	NBPF15	RSPO1	GPR87
	KPNA3	CASP10	CLEC4E	CDH23	RECQL	DNAJA4	UBQLN2	PVRL2	GSC	CMKLR1
	RAX	BIRC7	KQO37500	MAGED4	CYFIP1	IFT57	EDA2R	SULF2	RHBDF2	MAPK7
	LRCH2	FAM60A	PTRH2	CALB2	CITED4	C1orf186	IRF2BP2	IKBKE	CD70	SLAMF9
	CCNJ	PKNOX2	CSNK1E	AHNAK2	CPNE5	STMN3	BCHE	GATC	LRRC42	JOSD1
	PLSCR3	CHRNA7	C10orf119	CDHR1	KAL1	TMEM130	IL6ST	BACH2	PCDHGB7	SEMA6B
	ATG12	ITGA2	OR10K2	TGM2	UBE2I	TMIGD2	SLC22A16	MARCO	IGFBP6	GPR143
	FAM23A	SULF1	C15orf39	GRIK4	EPHA7					
	CYR61	UGCG	RND3	EIF4A1	APBA2	CDV3	PELO	APOBEC3A	IFFO2	MMP14
	DNAJB5	RBM7	HSPB8	MAP1B	PPP1R15B	LRRC59	NOLC1	ACTG1	POLR3D	WDR43
**CONSERVED IN N/S1**	CORO1C	NFIL3	CNTNAP1	KIAA0020	TUFT1	TKT	UBAP1	RNF19B	RRS1	SLC25A25
	BYSL	RIPK2	PABPC1	TNFRSF10D	PLK2	PABPC3	TRIM16	HSP90AB1	TEX10	GTPBP4
	HBM	DGKG	GMPS	OSGIN2	ZNF367	ACVR1	EIF2S1	UCK2	ZNF259	SH2D5
	POLR1E	TRIM32	EIF4A3	TAF4B	SLC25A32	AVEN	NCRNA00120	GNL3	CCRN4L	
	HBB	NUAK2	HBA1	HBA2	RPL7	CCT4	EIF3H	COCH	GDI2	GRHPR
	HBG2	EPB42	HBG1	KCNK10	CCT2	EEF1A1P9	INHBE	GADD45A	RAN	TRIM35
	PRMT5	DUSP8	MAFG	HSPA8	C4orf14	CCT5	PPP2R2A	GALK2	ARHGDIG	KCNG2
**ACQUIRED IN S1**	LASS1	FXR1	MTFR1	MGST1	SLC17A1	SLC9A3R1	SLC13A1	METTL13	SLC47A2	SLC47A1
	SHMT2	GRPEL1	BAG2	GARNL3	PYCR1	TCP1	SORD	AFG3L2	GPATCH4	KIF1A
	EIF3E	UMPS	GPR89A	KPNB1	WDR12	EPS15L1	CGREF1	MGC14436	RSL1D1	MRPL45
	RPL10A	MAPK12	CHMP1B	NLRP6	TARS2	NPM1	C22orf28	IRAK1	EIF4B	ECHDC1

	ST13	MRPS27	ACOT4	EEF2	TMEM144	RGL1	MMADHC	ALDH1B1	CIRH1A	OPTN
	DCAF13	C2orf47	EEF1B2	PPA2	CCT3	C1orf43	C6orf106	NOP14	C1QBP	C11orf54
	CCR9	AGXT2	ATP6V1B2	COX15	DAP3	CRTAP	F12	MRPS30	TFB2M	ALDH1A1
	TOMM20	NCRNA00113	VCP	DPP3	PLEKHA5	RPP40	NIPSNAP1	TMEM177	AQP11	UBE2R2
	ATIC	MRPL3	GPN3	C1orf107	EEF1A1	SLC31A1	PANK1	NMD3	SNHG5	ETF1
	C1orf163	TFDP2	PKLR	AGMAT	PGK 1.00	NOB1	HADH	SLC15A4	RAD23B	TIMM8A
	TMEM106A	LOC285733	VRK3	DAB2	PSMD11	INHBC	LCN10	RABGGTB	RRAGA	ACSF2
	TAF9	CCNG1	MRPL44	GDF1	AK3L1	SLC22A13	FAM10A4	PTDSS1	CCT7	UTP3
	TDP2	LRPPRC	EFTUD1	RABEPK	XYLB	SPG20	C13orf38	TARS	GPD1	PARD3
	RMND1	MDH1	FAH	BRE	SLC5A10	PGAM1	SLC26A1	HINT1	PGAM5	FARSA
	EIF2A	ADH5	IARS2	NOX4	MSRA	C13orf1	PRPF18	TOR1A	MAD2L1BP	BTF3
	STAMBP	BECN1	BPHL	ZNF622	PEPD	GPR89B	PPA1	MTHFD1	ASH2L	PRDX3
	TMEM14B	HSPD1	IGBP1	BCL2L13	CRYL1	HPN	BAG3	LAP3	PECI	AKR7A3
	ACTR6	ANKS4B	NCRNA00188	RAB5C	C17orf71	SLC25A30	RPL7L1	ILF2	BHMT2	CALM3
	PRKAG1	SLC35D2	ANPEP	PSMD2	PGAM4	GEMIN4	ENPEP	HTATIP2	RARS	HSDL2
	KIAA0100	NGLY1	TNFAIP8L1	SLC39A14	CHMP5	MRPL42	SLC28A1	TRAP1	STOML2	CBX4
	SLC25A5	RARS2	FAM177A1	VDAC1	ECHS1	ETFA	SLC27A2	RBP5	SLC1A1	NACA
	PSMB5	CCL14-CCL15	SCO1	SLC7A9	DARS2	TM7SF3	C12orf11	LACTB2	BPNT1	RPE
	ACY1	MRFAP1	EEF1A2	EHHADH	DYNLL2	EIF3C	RPS3A	C21orf7	GPN1	CYB5A
	MCCC2	ACAT2	RNASEH1	RBKS	CES2	SLC39A11	EXOSC3	DHTKD1	GHITM	SLC16A4
	DRAM2	PCK2	LRP2	C6orf57	F7	TP53RK	SLC22A2	DCTN6	NEDD4	SQSTM1
	YARS2	GRINA	SLC23A1	IARS	KLHL12	STRAP	LONP1	ZDHHC9	TMEM206	NACAP1
	MAPKAP1	PRPS1	PCCB	PSMD10	ALDH6A1	SAMM50	ECE2	FXN	TM2D2	RIPPLY2
	RPUSD4	CISD2	YIPF4	AKR7A2	TTC35	RPL3	FTHL3	HSPA9	IPO4	AIFM1
	C5orf30	RNF5P1	MLX	CMBL	KHK	PCCA	ALDH9A1	CPPED1	SDHC	MRPL50
	SCFD2	ABCB7	RPS12	SSTR1	NUDT5	DPH3	MRPL37	SSR3	CLTC	MRPL46
	NDUFS4	RETSAT	CLP 1	RPL17	PIPOX	SENP2	ERICH1	GATM	ADSL	MAPT
	SETD3	NDUFS2	RPL26	C4orf3	MRPS35	TRIM16L	MMS19	KCNJ12	LDHB	ETNK2

	PLD1	XPR1	GART	IMMT	FKBP4	PCTP	ABCC2	FAM96A	MRPS23	F2
	ISM1	C22orf45	PPAT	GNPDA1	ATXN10	ALDH1L1	DCTPP1	SFXN1	EIF2S3	RNF152
	SEMA5A	C6orf120	SPAG5	C9orf30	WARS2	OAT	DAK	C1orf57	C6orf72	PBLD
	FDPS	DNAJC12	ACAT1	AKR1C3	AARS	NEFL	ACADM	VDAC2	ATF5	YRDC
	HAUS6	SOHLH2	PRODH2	RNF123	ESD	FTL	NR4A3	MRPS22	C20orf24	PRDM4
	MFI2	TSPYL1	RAB7A	HECW1	TMLHE	ABCF1	CYFIP2	GLYATL1	RG9MTD1	FAM82B
	INADL	TTC9C	C21orf33	PDZK1	ATP5F1	HNRNPH2	CDC123	CLSTN3	MARS2	ZNF697
	UBXN8									
	HBA1	RPL7	CCT4	EIF3H	HBM	COCH	GDI2	GRHPR	KCNK10	CCT2
	BYSL	EEF1A1P9	GADD45A	TRIM35	POLR3D	HSPA8	C4orf14	CCT5	PPP2R2A	GALK2
	ARHGDIG	KCNG2	LASS1	ZNF259	MTFR1	SLC17A1	SLC9A3R1	SLC13A1	SLC47A2	SLC47A1
	SHMT2	BAG2	GARNL3	PYCR1	TCP1	SORD	AFG3L2	GPATCH4	KIF1A	EIF3E
	UMPS	GPR89A	WDR12	EPS15L1	CGREF1	MGC14436	RSL1D1	GTPBP4	RPL10A	MAPK12
	NLRP6	TARS2	NPM1	C22orf28	HSP90AB1	UBAP1	IRAK1	ECHDC1	ST13	MRPS27
	ACOT4	EEF2	TMEM144	RGL1	MMADHC	ALDH1B1	OPTN	DCAF13	C2orf47	EEF1B2
	C1orf43	C6orf106	NOP14	C11orf54	CCR9	AGXT2	ATP6V1B2	CRTAP	F12	MRPS30
	TFB2M	ALDH1A1	TOMM20	VCP	PLEKHA5	RPP40	NIPSNAP1	TMEM177	AQP11	UBE2R2
**LOST FROM S1**	EIF2S1	NOLC1	ATIC	TRIM32	GPN3	EEF1A1	SLC31A1	TNFRSF10D	PANK1	NMD3
	SNHG5	ETF1	C1orf163	PKLR	AGMAT	PGK 1.00	NOB1	HADH	SLC15A4	TMEM106A
	LOC285733	ACVR1	VRK3	ACTG1	DAB2	LCN10	RABGGTB	RRAGA	ACSF2	TAF9
	CCNG1	MRPL44	GDF1	AK3L1	SLC22A13	FAM10A4	PTDSS1	CCT7	TDP2	LRPPRC
	RABEPK	XYLB	SPG20	APBA2	TARS	GPD1	PARD3	RMND1	MDH1	FAH
	BRE	SLC5A10	PGAM1	SLC26A1	HINT1	PGAM5	FARSA	EIF2A	ADH5	NOX4
	MSRA	C13orf1	TOR1A	MAD2L1BP	BTF3	STAMBP	BPHL	ZNF622	PEPD	GPR89B
	PPA1	MTHFD1	ASH2L	PRDX3	TMEM14B	HSPD1	IGBP1	BCL2L13	CRYL1	HPN
	BAG3	LAP3	PECI	AKR7A3	ACTR6	ANKS4B	NCRNA00188	RAB5C	C17orf71	SLC25A30
	ILF2	BHMT2	CALM3	POLR1E	PRKAG1	SLC35D2	ANPEP	PGAM4	GEMIN4	ENPEP
	HTATIP2	RARS	HSDL2	TNFAIP8L1	SLC39A14	CHMP5	MRPL42	SLC28A1	TRAP1	STOML2

	CBX4	RARS2	FAM177A1	VDAC1	ECHS1	ETFA	SLC27A2	RBP5	SLC1A1	NACA
	CCL14-CCL15	SCO1	HSPB8	SLC7A9	TM7SF3	C12orf11	LACTB2	RPE	ACY1	MRFAP1
	PABPC3	EEF1A2	EHHADH	DYNLL2	EIF3C	RPS3A	C21orf7	CYB5A	MCCC2	ACAT2
	RNASEH1	RBKS	CES2	SLC39A11	EXOSC3	RRS1	WDR43	DHTKD1	SLC16A4	DRAM2
	PCK2	LRP2	C6orf57	F7	TP53RK	TUFT1	SLC22A2	DCTN6	NEDD4	SQSTM1
	YARS2	GRINA	SLC23A1	TAF4B	STRAP	ZDHHC9	TMEM206	SLC25A32	NACAP1	MAPKAP1
	PRPS1	PSMD10	ALDH6A1	APOBEC3A	SAMM50	CNTNAP1	ECE2	FXN	TM2D2	RIPPLY2
	RPUSD4	CISD2	YIPF4	AKR7A2	TTC35	RPL3	SLC25A25	AIFM1	C5orf30	RNF5P1
	MLX	CMBL	KHK	PCCA	ALDH9A1	CPPED1	SDHC	MRPL50	SCFD2	RPS12
	SSTR1	DPH3	MRPL37	MRPL46	NDUFS4	RETSAT	CLP 1	RPL17	PIPOX	SENP2
	GATM	CORO1C	SETD3	C4orf3	MRPS35	TRIM16L	RBM7	KCNJ12	LDHB	ETNK2
	PLD1	GART	IMMT	FKBP4	PCTP	ABCC2	TRIM16	MRPS23	F2	ISM1
	C22orf45	PPAT	GNPDA1	SH2D5	ALDH1L1	SFXN1	RNF152	SEMA5A	SPAG5	OAT
	DAK	C1orf57	C6orf72	PBLD	FDPS	DNAJC12	PABPC1	ACAT1	AKR1C3	AARS
	NEFL	ACADM	ATF5	YRDC	PRODH2	RNF123	ESD	FTL	C20orf24	DGKG
	PRDM4	MFI2	TSPYL1	RAB7A	HECW1	TMLHE	CYFIP2	ZNF367	GLYATL1	FAM82B
	TTC9C	C21orf33	PDZK1	ATP5F1	HNRNPH2	GNL3	CLSTN3	MARS2	ZNF697	
**CONSERVED IN S1/S2**	HBB	NUAK2	HBA2	HBG2	EPB42	RNF19B	HBG1	INHBE	RAN	PRMT5
	DUSP8	MAFG	FXR1	MGST1	EIF4A1	METTL13	GRPEL1	PLK2	KPNB1	MRPL45
	CHMP1B	EIF4B	CYR61	CIRH1A	PPA2	CCT3	C1QBP	COX15	DAP3	NCRNA00113
	DPP3	TKT	MRPL3	CCRN4L	C1orf107	TEX10	TFDP2	RAD23B	TIMM8A	DNAJB5
	PSMD11	INHBC	UTP3	EFTUD1	C13orf38	KIAA0020	AVEN	IARS2	PRPF18	BECN1
	PELO	RPL7L1	PPP1R15B	UGCG	MAP1B	NCRNA00120	PSMD2	CDV3	KIAA0100	NGLY1
	IFFO2	SLC25A5	PSMB5	DARS2	BPNT1	GPN1	GHITM	RIPK2	IARS	KLHL12
	LONP1	PCCB	FTHL3	NFIL3	RND3	HSPA9	IPO4	ABCB7	NUDT5	SSR3
	CLTC	ERICH1	ADSL	MAPT	MMP14	NDUFS2	RPL26	MMS19	XPR1	FAM96A
	ATXN10	DCTPP1	EIF2S3	C6orf120	C9orf30	WARS2	GMPS	EIF4A3	VDAC2	HAUS6
	SOHLH2	NR4A3	MRPS22	ABCF1	LRRC59	RG9MTD1	UCK2	INADL	OSGIN2	CDC123

	UBXN8									
**ACQUIRED IN S2**	GDF15	ZFP36L1	IGFN1	SLC16A10	ZBTB25	BRS3	DIAPH3	TUBB2B	LOC144776	VTI1A
	PIM1	SERPINE3	KLF6	MPZL1	C1orf187	PPIF	RAP2B	HKDC1	KRTAP1-1	VGF
	MYPN	ADM	TMEM161B	MEP1B	ARHGAP28	PPP1R3B	TAS2R13	CSNK1A1P	SLC16A1	ASB3
	KLHL33	PIM3	TTC28	TP53BP2	NSUN3	NLN	XIST	BEND6	SNORA4	CRABP1
	NAMPT	AGTPBP1	SEMA3E	PIK3C2G	PTP4A1	PSMD7	LARS	HTR3C	LOC645676	PGM3
	PI4KA	PLAC4	BDKRB2	ATXN8OS	PSMB2	HIF1A	RNF112	C7orf52	EIF1B	PPM1D
	LSAMP	KIAA0368	CARD6	FAM71B	OR10K2	SLC22A25	ANXA8L1	AWAT1	C4orf17	C6orf10
	FGF16	FOXD3	GRXCR2	KRTAP12-1	OR10T2	OR4F21	OR4F5	SYCP1	TACR3	ROBO2
	CISH	KLHL1	DYRK3	PCDHA4	DNAJC5G	CEP170	NRAP	FAM153C	FOSL2	EPDR1
	FAM153A	QRSL1	JOSD1	ING2	FLJ41941	HMGB3L1	LOXL2	ARHGEF3	YBX1	PATL1
	FLG2	TMEM49	SAAL1	RAG2	C21orf130	BCL6	TGFBI	C4orf26	GXYLT2	FILIP1
	POU4F2	EXOC6	SNORA62	GPR135	C3orf27	SLC28A2	SNAPC1	CEP78	LOC100130148	SOCS3
	DCDC1	PCDHGA9	P4HA3	BDNF	KIAA0391	NEAT1	LCORL	DEFB103B	IMPDH2	EXOSC10
	STX17	ASFMR1	GAL	STK17A	VLDLR	PALMD	GPR12	CHP	PHF7	COL25A1
	PROS1	PGD	UBC	MGC16384	CD274	ZNF460	PPP1R15A	RPL23AP82	ATP12A	B3GNT5
	GABPB2	ZNF57	KIAA1919	DCUN1D3	ZC3H12A	GPR179	DKFZP434L187	XPNPEP1	INTS4L1	LOC727677
	OR2AE1	LIF	NKX2-4	MPHOSPH9	C14orf126	LOC283731	LOC285629	MNAT1	OR4F4	FAM119B
	PIR	DNMT3B	SEC14L4	RNF10	TTC39B	DNAJC25	TMEM136	TTLL7	JUN	FLJ14107
	ART5	KIAA1328	LOC729609	ABL2	MLLT11	GADL1	ZFP36	CLSTN2	LIPI	PIP4K2A
	HTR1B	PTENP1	DFFA	NCRNA00169	EXD2	PGM2L1	RNF122	CEP170L	RNF180	HTR1F
	HIST1H2AL	TCP11L1	PIPSL	ZCWPW2	C2orf57	GCLM	PRO1768	KRTAP5-1	HMBOX1	BMP2K
	OR51B6	PSG2	HNRNPC	KIAA1598	KIF2A	KALRN	CFH	NR6A1	RARB	THOC5
	NMT2	RFX6	GABARAPL3	KRT33B	SNORA6	GPRIN3	FAM123A	ISLR2	ZBTB38	C6orf174
	C11orf74	ANKRD26	TTPA	NPC1	MDGA2	KCTD4	TUBA1A	PLA1A	FGF3	LOC100302401
	AP4S1	SPATS1	SPSB1	LDHAL6B	C10orf68	KRT76	OR9Q1	ZNF669	PAPPA	TAS2R43
	METTL6	GCNT2	FHOD1	ANKRD36B	STAM	FAM66E	ZNF584	SLC4A1	GATA6	IL8
	LOC729603	PFKFB3	PHF21B	HIST1H2AK	PDHX	C3	MAP7D3	STRBP	AXIN2	FBXL20

	FAM41C	GYS2	SPATA1	NEU3	WDR88	TPTE2P1	LOC100132288	KRBA2	STT3A	C14orf109
	GNA13	ANKRD36	CXCL2	DHX32	LOC100128292	ZNF418	SMARCD3	RNF217	NUBPL	COQ2
	PGBD5	KIAA1632	DOCK3	C6orf59	TAS2R31	UTP6	ZNF70	NRP1	SMS	SRP54
	ATF3	PDZD8	RPL23AP64	C3orf26	MGC2889	DMRTC1	SLC26A5	RASEF	SYT16	ARL4D
	LMOD2	OLIG3	ZNF322B	C22orf43	ZSCAN20	CHD1L	OXSM	AKAP6	POLR3G	LOC100128640
	NRD1	NAV3	RPL23P8	ACTR10	IQCB1	LOC401052	SAV1	OPN3	SEC23A	RNF214
	PRICKLE2	SNORA13	ZNF208	VWA2	EIF2C3	NAV1	TAF13	SLC39A2	NBPF9	H6PD
	ZNF611	RAB31	TBC1D3P2	EXD1	MSH4	LOX	FLJ22536	DHDDS	MAPK8	POU2F3
	NME6	PSG8	WDR11	PTPDC1	LHFPL4	LOC154822	PWRN2	ADK	ZBTB37	TUBB2A
	ZNF449	HMOX1	SLC2A12	MMP17	CNN3	TAS2R30	ADORA1	C10orf62	C3orf16	CSH2
	KRTAP5-11	OR52H1	MAP1LC3C	TEX2	ZNF326	DNM3	NGDN	MYO1B	MID1	RBM17
	TSNAX-DISC1	C14orf101	NEUROD1	SET	C6orf164	MAP3K9	LOC220729	CCDC83	CSRNP1	LEPRE1
	GRIN2B	EHBP1	CCDC54	MAP4K4	C14orf166	PNRC1	LOC650623	DBI	YOD1	MGC12916
	LOC595101	C5orf47	PPP4R1L	C10orf90	PCA3	EEF1G	TAF12	C14orf135	RNPEP	PLCD4
	MRPS25	OSMR	STBD1	TCEB3C	MT1IP	RAD18	EIF2B3	ERC2	CCDC149	TNFSF18
	PPP1R2P1	TRDMT1	FAM153B	OR1K1	PPPDE2	GAA	JAC38412	GON4L	GLB1L3	SNORD116-28
	OR5B21	WDR41	FHOD3	CKAP5	C4orf19	SEC22B	GBA	LGALS8	OXNAD1	MRGPRE
	PCBD1	ADAM21	SLC2A3	RDH8	KIF4B	SPTLC3	ST6GALNAC3	LOC285780	FGFR2	NEIL3
	DENND5A	FLJ36031	MANBA	ATRNL1	CEP152	NDUFAF4	TUBB	GABRB2	COL27A1	PTPLA
	RBBP8	MYOF	FOXI1	DEPDC5	SLC39A7	LMAN1L	ALDH18A1	ME1	PVR	FAM190B
	PLEKHG3	HTR1A	BMS1P4	RASAL2	GNG5	IER3	C2orf61	SDCCAG8	NMNAT3	PDCD11
	KLHL10	GDAP1	TSC1	RNPC3	CTH	SULT4A1	CT62	FBXO48	IL13RA2	EXOG
	TAAR5	CREG2	C9orf25	DNASE1	LOC100302650	ARID3C	BHLHE40	ZNF382	C14orf119	VWA5A
	ZNF808	FEM1C	C3orf48	MCART2	SMAD2	RTN3	TSPYL6	FSTL1	LRRC36	PSEN1
	TSGA13	MAP3K13	OSBPL11	LOC91316	TRIB1	DNAH14	SLC22A15	FAM7A3	SPAG9	NXNL1
	SLC38A8	PRDX1	WDR19	LOC729799	CDH19	SIAH1	EXTL1	LOC126536	LAMA4	TBC1D10A
	ATP13A3	MCL1	GDAP2	SPATA8	C22orf15	DYNC1LI1	LOC729082	LGALS12	AMTN	LRRIQ3
	PIP5K1A	ETFDH	CPS1	CXCL1	EID2B	LONRF1	NKX2-2	SLC25A20	CACNG2	OBP2A

	ISX	HDX	FBXO18	C17orf58	TBC1D1	CDO1	RPP14	C20orf123	ZNF667	HIST1H4L
	PTPN20A	FGF23	ZNF563	WLS	NEXN	SEC23B	SLC38A7	RUFY3	MED20	C10orf46
	C4orf29	TTL	AGBL1	ATP6V1G3	C2orf51	EVX2	FKSG83	GPR101	LOC144742	LRIT1
	NXF4	WFDC6	MCF 2.00	EIF2AK3	C14orf43	FCHSD2	NCRNA00115	AHCYL1	ZNF248	TIMM17A
	PCDHA3	TOX4	SNW1	C13orf36	FAM160A2	TRIM38	WFDC8	HEXB	BACE2	GDPD1
	CALR3	C10orf12	NTAN1	CFL2	BET1	AIFM3	CTTNBP2NL	TRAF6	RNF212	DUSP14
	LOC100124692	ZSWIM6	RSRC2	NIN	FTH1	ZNF704	DDIT4	CCL2	TIPARP	SPINLW1
	PAR5	JPH1	EIF2S2	HAVCR2	OR13F1	CPD	ZFAND5	EAF1	EPRS	EIF4E
	RSPO4	KIAA1107	APPBP2	PTEN	FOXG1	GALNTL1	SMNDC1	FLJ44054	FBXW7	QZI38777
	CWC27	PRDM7	C14orf118	ABHD4	GPHN	MRPS16	UBE4B	TXNRD3IT1	LSM 3.00	FBXO25
	NRBF2	AKIRIN2	RAB9B	EIF3L	C3orf35	FER1L5	LOC100287227	MALT1	CSNK2A1	RPL36A
	CGB8	VAMP3	STAC	CCNL1	LDLRAD3	PNPLA5	STL	FAM20A	UBA3	OTUD6A
	ZNF717	PNRC2	YME1L1	C16orf87	SEC11A	KRTAP1-3	GAB2	DCP1A	ADAM22	ALS2CR8
	HACL1	TBC1D23	HYMAI	LOC284100	ABCC5	DNAH1	AAK1	AHSP	HNRNPA2B1	AMAC1
	PSG6	ZNF295	ULK4	HOXA9	ALKBH1	PDE4DIP	CYP51A1	HNRNPH3	CTSA	ZNF625
	MUC13	NAA35	IFNB1	DEFB109P1B	ZNF577	DUSP18	FLG	NDUFB5	DUSP5P	ADH4
	PFN2	GTPBP2	SLC25A40	INVS	RRAGD	DEFB1	TBC1D7	DDIT3	SEC22C	UHRF1BP1
	TAS2R19	ITSN1	NR2C2	RALYL	LETM2	EIF4A2	NHEDC1	LOC440896	PCDHA1	RNF151
	SLED1	TMEM100	ZFYVE20	RASA1	BRD7	LTA4H	LIX1L	RASSF6	C16orf68	PABPC4
	NPEPPS	ZIC3	NPFFR2	C14orf28	KIAA0831	C11orf9	JAKMIP3	RHOB	KLHL5	PPP2R5E
	PTCD2	GPR137B	TCTE1	SLC2A1	OR13C2	FEV	KCNJ8	CLIC6	MALAT1	YEATS2
	NEUROG3	GTF2H3	RAB9A	MLH1	HINT3	C18orf20	GBE1	PRDM13	FAM198A	DGKI
	WDR6	CHRFAM7A	S100B	DLX6	C17orf42	HEATR5A	PRG1	GCNT3	TSC22D2	ABCG4
	CLASP2	ZMYM4	GPI	NHLRC2	LOC144486	SPCS1	HDHD1A	FERMT2	SYT14	CYP26B1
	AATF	DNAJB4	FMNL2	F2RL2	KIR3DL3	FKBP7	MTERFD2	FAM188A	C7orf4	EBNA1BP2
	STRN3	MRAS	ZNF713	ENAH	KIAA1529	ZNF555	KLHL24	NPAS1	C10orf10	ATXN7
	TRMT11	ASB18	NCOA7	IFIT1B	SLC2A14	IL4	MTR	RWDD3	CXCL3	ENPP1
	PLIN5	HSN2	PHTF1	LITAF	KCTD6	PCDHA10	PLAGL1	ATG2B	TMX1	COL22A1

	NKIRAS1	HN1	DYM	TRMT5	LRIG2	MCHR1	DLEU2L	LOH12CR1	KRT222	ENO1
	PIK3C3	LRP8	PI4KAP2	CPNE2	HEXA	ISCA2	SLC25A24	TMX4	SATB1	FLJ35776
	LOC100130522	DIRC2	DUS2L	TMCO7	ZNF25	CLK3	PHF21A	KIAA1279	MLLT10	SEC31B
	STAC2	CYP1A2	MYLK	NARS	DNAH12	SAMD8	STXBP4	CRX	NME1	TRIM45
	CLCC1	C20orf141	C7orf46	HIF1AN	MRRF	SERPINE1	SOAT1	CEACAM22P	BRAF	UQCRHL
	C2orf56	NDUFV1	CHCHD4	BCL10	HRASLS	CLIP1	SEC13	IPO7	GCLC	PTGR2
	HABP2	MAN2B2	C9orf72	TRIM44	PI4K2B	IMPG2	SLC25A37	LEPROT	PCDHGA3	TMC5
	TAB2	ZNF271	KCNIP1	ASXL3	GLT25D1	FABP3	GK5	COPB2	FCF1	CASP6
	MMP7	TEC	FLJ40292	TAS2R5	HSF2	LOC347376	DGKH	RRP8	CATSPER4	KRTAP10-1
	OR13C4	OR5AU1	CDC26	CSMD2	PSMA1	ARHGEF38	KIAA1797	CHEK2	SERPINF1	PGGT1B
	HOXB9	APEX1	MYSM1	DPH5	RNF128	LOC728190	FAM151B	MRPL47	RNF141	RRN3
	CYTSB	MDH1B	RTN4IP1	EXT1	MANF	COPB1	ARHGAP29	WBP2NL	MYO19	ZFYVE26
	C14orf34	SV2B	MIR17HG	CSDA	LAMA1	ANP32B	PYGL	CPOX	KLF7	UQCRH
	UCHL1	NPM3	HELB	SNX32	KLKP1	IGLON5	EIF1	PTGFRN	MFN1	ARRDC3
	ADRB2	LOC728323	STIP1	NAA38	TLR5	MUC21	SUPV3L1	TTC14	CACNG1	SERHL2
	CSGALNACT2	NEFM	MRPL33	FAM45B	RET	DOCK7	SDHB	ZNF433	KIAA0319L	SCARB2
	ADAM17	TRUB1	FKBP3	FIG4	RPL23AP53	CRYBG3	IDO2	CHMP2B	GADD45B	RPL36AL
	PIK3CA	RPS25	LOC145783	KLHDC4	GBF1	INSM1	LOC220930	GLB1	NCBP1	TPM3
	FUT5	C18orf55	C3orf71	BLVRB	ANKRD34B	FUBP1	ZADH2	ALG10	NHLRC3	RCN1
	DSE	TIMM9	TUBB8	SRI	CDX2	NPPB	RPS4X	SLCO5A1	CDK5R2	LRRC40
	CDC27	SIP1	RALGAPA2	TMEM2	FAM45A	PNMA3	FAM178A	SLC19A2	VAPA	TAF15
	THRAP3	KIR2DS4	FAM21A	KCNT2	NTNG2	CACNB4	KIAA1958	HESX1	GMFB	UTS2
	EIF2C4	DNAJC13	CUEDC2	PSMA3	LOC116437	XIRP1	MTOR	RNF2	CECR5	LRP12
	GARS	ERO1L	ZNF442	AFAP1	ME2	LPHN2	CCDC21	LRG1	VIPAR	PCDH11Y
	ACAP2	ZNF135	UBQLN3	TPD52L1	YARS	RBM6	PPIAL4G	CCDC102B	ALG6	WBP5
	KCNA10	RNASE1	RGS2	IFITM2	ZCCHC11	CYP 20.00	ACBD3	RNF19A	ZNF354C	HCN4
	RFT1	MT2A	NHLH2	NBPF10	DPAGT1	HYAL4	SNORA72	WSB1	SERPINB7	CDK11A
	TCF25	LRRTM2	ECD	BBS7	LOC100128788	REN	DHRS9	SH3RF1	CLIP4	CBWD3

	HNRNPR	GRN	RUNDC2C	MTPAP	MAP3K7	DNAJC10	SNX6	TRNT1	JMY	SNORA56
	GNPAT	QARS	KRT74	EXOC5	ZEB1	SNX10	CCDC79	EDEM1	CXorf23	CSNK2A1P
	SYT11	CCIN	CCNH	CHUK	NRCAM	OR13D1	GZF1	VSTM1	LOC441046	TRIP13
	SULT1E1	GATSL1	POLR3A	TAC1	GNAI3	TMEM20	C5orf42	SEMA3B	SPDYE7P	C2CD4A
	IPO9	SPATA7	BTBD3	RHEB	C3orf59	SOS 2	RNF150	MSL1		
**LOST FROM S2**	IGFN1	SLC16A10	ZBTB25	TUBB2B	LOC144776	SERPINE3	KLF6	MPZL1	NFIL3	RAP2B
	CHMP1B	NGLY1	KRTAP1-1	VGF	MYPN	RND3	ADM	MEP1B	ARHGAP28	TAS2R13
	CSNK1A1P	SLC16A1	ASB3	KLHL33	PIM3	TTC28	TP53BP2	CDV3	NSUN3	NLN
	XIST	BEND6	SNORA4	CRABP1	AGTPBP1	SEMA3E	PIK3C2G	PTP4A1	PSMD7	LARS
	HTR3C	LOC645676	PGM3	PI4KA	PLAC4	BDKRB2	ATXN8OS	PSMB2	HIF1A	RNF112
	C7orf52	EIF1B	LSAMP	KIAA0368	CARD6	FAM71B	OR10K2	SLC22A25	ANXA8L1	AWAT1
	C4orf17	C6orf10	FGF16	FOXD3	GRXCR2	KRTAP12-1	OR10T2	OR4F5	SYCP1	TACR3
	ERICH1	ROBO2	CISH	KLHL1	DYRK3	PCDHA4	DNAJC5G	CEP170	NRAP	FAM153C
	FOSL2	EPDR1	FAM153A	QRSL1	JOSD1	ING2	FLJ41941	HMGB3L1	LOXL2	ARHGEF3
	YBX1	FLG2	TKT	TMEM49	SAAL1	RAG2	C21orf130	BCL6	TGFBI	C4orf26
	GXYLT2	FILIP1	POU4F2	GPR135	C3orf27	SLC28A2	LOC100130148	SOCS3	DCDC1	PCDHGA9
	P4HA3	CCRN4L	KIAA0391	NEAT1	LCORL	DEFB103B	IMPDH2	EXOSC10	ASFMR1	GAL
	STK17A	PALMD	GPR12	CHP	PHF7	COL25A1	PROS1	PGD	UBC	MGC16384
	CD274	ZNF460	PPP1R15B	PPP1R15A	RPL23AP82	ATP12A	B3GNT5	GABPB2	KIAA1919	DCUN1D3
	ZC3H12A	GPR179	PSMD11	DKFZP434L187	XPNPEP1	C9orf30	INTS4L1	LOC727677	OR2AE1	LIF
	NKX2-4	MPHOSPH9	LOC283731	LOC285629	HBG1	OR4F4	PIR	DNMT3B	SEC14L4	RNF10
	TTC39B	DNAJC25	TMEM136	EPB42	TTLL7	JUN	FLJ14107	ART5	KIAA1328	LOC729609
	ABL2	MLLT11	GADL1	ZFP36	BECN1	LONP1	CLSTN2	LIPI	PIP4K2A	HTR1B
	PTENP1	DFFA	NCRNA00169	EXD2	PGM2L1	RNF122	RNF180	HTR1F	HIST1H2AL	PIPSL
	ZCWPW2	PSMB5	C2orf57	GCLM	PRO1768	KRTAP5-1	HMBOX1	BMP2K	OR51B6	PSG2
	HNRNPC	KIF2A	KALRN	CFH	NR6A1	RARB	THOC5	RFX6	GABARAPL3	KRT33B
	SNORA6	GPRIN3	FAM123A	ISLR2	ZBTB38	C6orf174	MAP1B	C11orf74	ANKRD26	TTPA
	MDGA2	KCTD4	TUBA1A	PLA1A	FGF3	LOC100302401	AP4S1	SPATS1	SPSB1	LDHAL6B

	C10orf68	KRT76	OR9Q1	ZNF669	PAPPA	TAS2R43	METTL6	GCNT2	FHOD1	ZNF584
	SLC4A1	GATA6	IL8	LOC729603	PHF21B	HIST1H2AK	C3	MAP7D3	AXIN2	FBXL20
	FAM41C	GYS2	SPATA1	NEU3	WDR88	TPTE2P1	LOC100132288	KRBA2	STT3A	BPNT1
	C14orf109	GNA13	ANKRD36	CXCL2	DHX32	LOC100128292	ZNF418	SMARCD3	RNF217	NUBPL
	COQ2	PGBD5	KIAA1632	DOCK3	C6orf59	IFFO2	UTP6	ZNF70	NRP1	SMS
	SRP54	ATF3	PDZD8	RPL23AP64	C3orf26	MGC2889	DMRTC1	SLC26A5	MMP14	TIMM8A
	RASEF	SYT16	ARL4D	LMOD2	OLIG3	ZNF322B	INHBE	C13orf38	C22orf43	ZSCAN20
	CHD1L	OXSM	AKAP6	POLR3G	NRD1	NAV3	NDUFS2	RPL23P8	ACTR10	IQCB1
	LOC401052	SSR3	SAV1	OPN3	SEC23A	RNF214	PRICKLE2	SNORA13	ZNF208	VWA2
	EIF2C3	KLHL12	NAV1	TAF13	SLC39A2	NBPF9	EFTUD1	H6PD	ZNF611	RAB31
	TBC1D3P2	EXD1	WARS2	MSH4	LOX	FLJ22536	DHDDS	MAPK8	IPO4	POU2F3
	NME6	PSG8	RG9MTD1	PTPDC1	LHFPL4	PELO	LOC154822	PWRN2	ADK	ZBTB37
	TUBB2A	ZNF449	HMOX1	SLC2A12	MMP17	CNN3	TAS2R30	ADORA1	C10orf62	C3orf16
	CSH2	KRTAP5-11	OR52H1	MAP1LC3C	TEX2	ZNF326	DNM3	NGDN	MYO1B	MID1
	TSNAX-DISC1	C14orf101	NEUROD1	SET	C6orf164	MAP3K9	MGST1	XPR1	LOC220729	CCDC83
	CSRNP1	LEPRE1	GRIN2B	CCDC54	C14orf166	PNRC1	DBI	MGC12916	LOC595101	C5orf47
	PPP4R1L	C10orf90	PCA3	EEF1G	TAF12	C14orf135	RNPEP	PLCD4	MRPS25	OSMR
	STBD1	TCEB3C	MT1IP	EIF2B3	DPP3	ERC2	CCDC149	TNFSF18	PPP1R2P1	TRDMT1
	HBG2	FAM153B	OR1K1	PPPDE2	GAA	GON4L	GLB1L3	SNORD116-28	OR5B21	WDR41
	FHOD3	C4orf19	SEC22B	GBA	LGALS8	OXNAD1	MRGPRE	PCBD1	SLC2A3	RDH8
	KIF4B	SPTLC3	LOC285780	NEIL3	FLJ36031	MANBA	ATRNL1	CEP152	NDUFAF4	TUBB
	GABRB2	PTPLA	RBBP8	MYOF	FOXI1	DEPDC5	SLC39A7	COX15	LMAN1L	ME1
	PVR	FTHL3	FAM190B	HBB	PLEKHG3	METTL13	HTR1A	BMS1P4	RASAL2	GNG5
	IER3	RNF19B	C2orf61	NMNAT3	KLHL10	KIAA0100	GDAP1	TSC1	RNPC3	CTH
	SULT4A1	CT62	FBXO48	IL13RA2	EXOG	TAAR5	CREG2	DNASE1	LOC100302650	BHLHE40
	ZNF382	C14orf119	VWA5A	ZNF808	FEM1C	C3orf48	MCART2	SMAD2	RTN3	TSPYL6
	FSTL1	LRRC36	PSEN1	TSGA13	MAP3K13	OSBPL11	LOC91316	TRIB1	FAM7A3	SPAG9
	NXNL1	SLC38A8	PRDX1	WDR19	LOC729799	CDH19	SIAH1	EXTL1	LOC126536	LAMA4

	TBC1D10A	ATP13A3	MCL1	GDAP2	SPATA8	C22orf15	DYNC1LI1	LOC729082	INHBC	LGALS12
	AMTN	LRRIQ3	ETFDH	CXCL1	EID2B	LONRF1	RAD23B	NKX2-2	SLC25A20	CACNG2
	OBP2A	ISX	HDX	HBA2	C17orf58	TBC1D1	CDO1	RPP14	C20orf123	ZNF667
	HIST1H4L	MRPL45	PTPN20A	FGF23	ZNF563	WLS	NEXN	SEC23B	SLC38A7	RUFY3
	C10orf46	C4orf29	AVEN	ATP6V1G3	C2orf51	EVX2	FKSG83	GPR101	LOC144742	LRIT1
	NXF4	MCF 2.00	EIF2AK3	C14orf43	FCHSD2	NCRNA00115	AHCYL1	ZNF248	PCDHA3	TOX4
	SNW1	C13orf36	FAM160A2	TRIM38	WFDC8	HEXB	BACE2	CALR3	C10orf12	PPA2
	NTAN1	CFL2	BET1	AIFM3	CTTNBP2NL	TRAF6	RNF212	DUSP14	LOC100124692	ZSWIM6
	RSRC2	NIN	FTH1	ZNF704	DDIT4	CCL2	TIPARP	SPINLW1	PAR5	HAVCR2
	OR13F1	CPD	ZFAND5	EAF1	EIF4E	RSPO4	KIAA1107	PTEN	FOXG1	GALNTL1
	FLJ44054	FBXW7	JWC38777	CWC27	PRDM7	C14orf118	ABHD4	MRPS16	UBE4B	TXNRD3IT1
	LSM 3.00	FBXO25	NRBF2	AKIRIN2	RAB9B	EIF3L	C3orf35	FER1L5	UBXN8	LOC100287227
	MALT1	CSNK2A1	RPL36A	CGB8	VAMP3	CCNL1	PNPLA5	UTP3	STL	FAM20A
	UBA3	OTUD6A	ZNF717	PNRC2	MAPT	YME1L1	C16orf87	KRTAP1-3	GAB2	DCP1A
	ADAM22	ALS2CR8	HACL1	TBC1D23	HYMAI	LOC284100	ABCC5	DNAH1	AAK1	AHSP
	HNRNPA2B1	UCK2	AMAC1	PSG6	ZNF295	ULK4	HOXA9	ALKBH1	PDE4DIP	CYP51A1
	HNRNPH3	CTSA	ZNF625	MUC13	IFNB1	DEFB109P1B	ZNF577	DUSP18	CIRH1A	FLG
	NDUFB5	DUSP5P	ADH4	PFN2	SLC25A40	INVS	RRAGD	DEFB1	TBC1D7	SEC22C
	UHRF1BP1	TAS2R19	ITSN1	NR2C2	LRRC59	RALYL	LETM2	EIF4A2	NHEDC1	LOC440896
	PCDHA1	RNF151	TMEM100	ZFYVE20	RASA1	BRD7	LTA4H	LIX1L	RASSF6	C16orf68
	PABPC4	NPEPPS	ZIC3	NPFFR2	C14orf28	KIAA0831	C11orf9	JAKMIP3	RHOB	KLHL5
	PPP2R5E	PTCD2	C6orf120	GPR137B	SLC2A1	OR13C2	FEV	CLIC6	MALAT1	YEATS2
	NEUROG3	GHITM	RAB9A	MLH1	HINT3	C18orf20	GBE1	PRDM13	FAM198A	DGKI
	WDR6	CHRFAM7A	S100B	DLX6	C17orf42	ADSL	ABCF1	HEATR5A	PRG1	GCNT3
	TSC22D2	ABCG4	CLASP2	ZMYM4	GPI	NHLRC2	LOC144486	SPCS1	OSGIN2	HDHD1A
	FERMT2	SYT14	CYP26B1	AATF	DNAJB4	F2RL2	KIR3DL3	FKBP7	FAM188A	C7orf4
	EBNA1BP2	STRN3	MRAS	ZNF713	ENAH	KIAA1529	ZNF555	KLHL24	NPAS1	C10orf10
	ATXN7	TRMT11	ASB18	NCOA7	IFIT1B	SLC2A14	IL4	MTR	RWDD3	ENPP1

	PLIN5	HSN2	PHTF1	LITAF	KCTD6	PCDHA10	PLAGL1	ATG2B	TMX1	FAM96A
	INADL	COL22A1	NKIRAS1	HN1	DYM	LRIG2	MCHR1	DLEU2L	NR4A3	LOH12CR1
	KRT222	ENO1	PIK3C3	LRP8	PI4KAP2	CPNE2	MAFG	HEXA	ISCA2	SLC25A24
	TMX4	SATB1	FLJ35776	LOC100130522	DIRC2	GRPEL1	DUS2L	TMCO7	ZNF25	CLK3
	PHF21A	KIAA1279	SEC31B	STAC2	CYP1A2	NARS	DNAH12	SAMD8	STXBP4	CRX
	NME1	TRIM45	CLCC1	C7orf46	HIF1AN	MRRF	SERPINE1	SOAT1	CEACAM22P	BRAF
	UQCRHL	CCT3	C2orf56	NDUFV1	CHCHD4	BCL10	HRASLS	CLIP1	SEC13	PTGR2
	HABP2	MAN2B2	C9orf72	TRIM44	PI4K2B	IMPG2	SLC25A37	LEPROT	PCDHGA3	DUSP8
	TMC5	TAB2	ZNF271	KCNIP1	ASXL3	FABP3	GK5	COPB2	FCF1	CASP6
	MMP7	TEC	FLJ40292	TAS2R5	HSF2	LOC347376	EIF4A1	DGKH	RRP8	CATSPER4
	OR13C4	OR5AU1	CDC26	ATXN10	CSMD2	PSMA1	ARHGEF38	KIAA1797	CHEK2	SERPINF1
	PGGT1B	HOXB9	MYSM1	DPH5	RNF128	FAM151B	MRPL47	RRN3	CYTSB	MDH1B
	RTN4IP1	MANF	ARHGAP29	ZFYVE26	C14orf34	SV2B	MIR17HG	CSDA	LAMA1	ANP32B
	PYGL	CPOX	KLF7	UQCRH	UCHL1	HELB	SNX32	DNAJB5	PCCB	KLKP1
	IGLON5	EIF1	PTGFRN	MFN1	ARRDC3	ADRB2	LOC728323	STIP1	NAA38	TLR5
	MUC21	TTC14	CACNG1	SERHL2	NEFM	RIPK2	MRPL33	NCRNA00113	RET	DOCK7
	SDHB	ZNF433	KIAA0319L	C1QBP	SCARB2	ADAM17	FKBP3	FIG4	RPL23AP53	CRYBG3
	IDO2	CHMP2B	RPL36AL	PIK3CA	RPS25	KLHDC4	GBF1	INSM1	LOC220930	GLB1
	NCBP1	TPM3	PSMD2	FUT5	C18orf55	C3orf71	BLVRB	ANKRD34B	FUBP1	ZADH2
	ALG10	NHLRC3	RCN1	DSE	SOHLH2	TUBB8	SRI	CDX2	NPPB	RPS4X
	SLCO5A1	CDK5R2	LRRC40	TFDP2	RALGAPA2	TMEM2	PNMA3	SLC19A2	VAPA	UGCG
	TAF15	THRAP3	KIR2DS4	KCNT2	NTNG2	CACNB4	KIAA1958	HESX1	GMFB	UTS2
	CLTC	EIF2C4	DNAJC13	CUEDC2	PSMA3	LOC116437	XIRP1	MTOR	CECR5	DCTPP1
	LRP12	ERO1L	ZNF442	AFAP1	ME2	LPHN2	CCDC21	LRG1	VIPAR	PCDH11Y
	ACAP2	ZNF135	UBQLN3	TPD52L1	SLC25A5	YARS	MMS19	RBM6	PPIAL4G	CCDC102B
	ALG6	HSPA9	WBP5	KCNA10	RNASE1	RGS2	IFITM2	ZCCHC11	CYP 20.00	ACBD3
	MRPS22	RNF19A	ZNF354C	HCN4	RFT1	MT2A	NHLH2	NBPF10	DPAGT1	HYAL4
	WSB1	SERPINB7	CDK11A	TCF25	LRRTM2	BBS7	LOC100128788	REN	DHRS9	EIF4A3

	SH3RF1	CBWD3	HNRNPR	GRN	RUNDC2C	TRNT1	JMY	SNORA56	GNPAT	QARS
	KRT74	EXOC5	SNX10	CCDC79	EDEM1	CXorf23	CSNK2A1P	SYT11	CCIN	CCNH
	NRCAM	OR13D1	GZF1	VSTM1	LOC441046	SULT1E1	GATSL1	POLR3A	TAC1	GNAI3
	C5orf42	SEMA3B	SPDYE7P	C2CD4A	SPATA7	BTBD3	RHEB	C3orf59	SOS 2	MSL1
	GDF15	ZFP36L1	BRS3	DIAPH3	PRPF18	VTI1A	PIM1	C1orf187	PPIF	HKDC1
	TMEM161B	PPP1R3B	NCRNA00120	NAMPT	PPM1D	OR4F21	PATL1	PLK2	EXOC6	NUAK2
	SNORA62	DARS2	FXR1	SNAPC1	CEP78	DAP3	BDNF	STX17	VLDLR	ZNF57
	C1orf107	C14orf126	MNAT1	FAM119B	KIAA0020	CEP170L	TCP11L1	KIAA1598	NMT2	NPC1
	ANKRD36B	STAM	FAM66E	PFKFB3	PDHX	STRBP	IARS2	TAS2R31	RPL26	LOC100128640
	RAN	WDR11	RBM17	EHBP1	MAP4K4	LOC650623	YOD1	RAD18	IARS	ILD38412
	CKAP5	ADAM21	ST6GALNAC3	FGFR2	DENND5A	COL27A1	ALDH18A1	SDCCAG8	PDCD11	TEX10
**CONSERVED IN S2/S3**	C9orf25	ARID3C	DNAH14	SLC22A15	PIP5K1A	CPS1	FBXO18	MED20	TTL	AGBL1
	WFDC6	TIMM17A	GDPD1	NUDT5	JPH1	EIF2S2	EPRS	APPBP2	SMNDC1	GPHN
	STAC	LDLRAD3	SEC11A	NAA35	VDAC2	ABCB7	GTPBP2	DDIT3	SLED1	GPN1
	TCTE1	KCNJ8	GTF2H3	FMNL2	MTERFD2	PRMT5	CXCL3	RPL7L1	KPNB1	CDC123
	TRMT5	MLLT10	MYLK	C20orf141	IPO7	GCLC	CYR61	EIF4B	GLT25D1	KRTAP10-1
	APEX1	LOC728190	RNF141	EXT1	COPB1	WBP2NL	MYO19	NPM3	SUPV3L1	CSGALNACT2
	FAM45B	TRUB1	GADD45B	LOC145783	TIMM9	CDC27	SIP1	FAM45A	FAM178A	FAM21A
	RNF2	GARS	EIF2S3	SNORA72	ECD	CLIP4	MTPAP	MAP3K7	DNAJC10	SNX6
	ZEB1	CHUK	TRIP13	HAUS6	TMEM20	GMPS	MRPL3	IPO9	RNF150	
	UNC50	PHOSPHO2	POLR1B	ZC3H8	USP49	PRMT3	GTF3C3	ZNF318	TUBGCP5	ZC3H15
	FASTKD2	CDC5L	EEF1B2	ANO4	MCM3APAS	POLH	TJAP1	HSP90AB1	GAD1	C11orf58
	MAD2L1BP	HSP90AB2P	SLC29A1	CD2AP	SUV39H2	TFAM	KIN	ELOVL5	E2F5	ORC4L
**ACQUIRED IN S3**	NIPA2	XPO5	NFYA	TTTY4C	MTX2	ATAD1	FAM163B	C10orf119	ENPP4	OTUD1
	PCGF6	GNMT	NIF3L1	AFF3	AARS2	NCKAP1	PRPS1	DDX50	RPE	TMEM185B
	RPL7	LRPPRC	SLC35B2	TOMM20	C2orf58	KIF6	TUBA4B	ERCC3	C10orf137	SLC25A16
	SLC35F5	YIPF3	SLC25A27	MINPP1	WDR12	AGFG1	C5orf30	LONRF2	C16orf81	NCL
	PPP2R5D	ORC2L	VPS26A	GAS2L3	XPOT	CEBPZ	MUT	LOC730101	VEGFA	TAF3

	ENPP5	KLHDC3	WDR43	SGTB	UBR2	39142	TIAL1	PRICKLE4	CYorf15B	HCN2
	BPY2	TAS1R3	POLR1C	HSPA14	SNORD1C	VEPH1	TARS	KLHL23	MIPOL1	PROX2
	FAM66C	KCNN2	MOAP1	RPP38	GCC2	DIRAS1	GABRB3	PRPF40A	OR2L1P	ABCC10
	C10orf88	EEF2	WNT8B	ATP1B4	RPS4Y1	GOLGA7	TCHH	GLRX3	MARS2	TMEM14A
	C2orf89	SOX3	CASP5	HAT1	NFE2L2	DDX18	GPATCH2	MAP4K3	COL21A1	CENPQ
	PYCR1	NOLC1	TTC21B	NCRNA00188	MMADHC	USP6NL	NOC3L	FNDC8	FRMD4A	C20orf160
	NOP58	IWS1	LRRC1	DCAF16	ASAP1	TTLL6	FAM175B	TMEM182	PPAT	PHYH
	ZFAND1	SEC61A2	FOXP4	FLJ10357	ZNF207	ACCN2	MAP3K15	GLO1	USP47	AZIN1
	SERPINA7	TCEA1	PAICS	CSGALNACT1	LOC100132354	RCL1	DDX3Y	TMEM63B	C2orf3	INTS7
	SPHKAP	SNAR-B2	SNAR-C3	ASNSD1	ARL5A	C6orf153	CHRM3	COL4A4	PNO1	TLK2
	SPIRE1	MKX	SEPHS1	LPA	AP3M1	PNPT1	UTP14A	GTF2H1	CUL7	LOC100287718
	COL4A3	SLC25A32	ZDBF2	KIAA0240	CWC22	ZFAND3	RGS11	EIF3A	C6orf138	AMMECR1L
	MRPS10	GPR162	MCM10	RSRC1	MWD7500	TTC17	PLS3	WT1	OLA1	PABPC3
	HPS3	CHRNA4	PEX6	IRX2	SNHG4	R3HDM1	RNU6ATAC	EEF1A1	DCAF12L2	SLC25A12
	AP3M2	TRAM2	NUP35	IBTK	WIF1	MEA1	CALCOCO2	CCNB1IP1	ABI2	TRERF1
	TRAM1	MTHFD2	GCA	FZD5	GTF2H2C	BEND7	PPP2R2A	ANAPC1	FAM86B2	THAP1
	FARSB	C1orf43	UBE2A	EIF5A2	B3GAT1	UTP20	PRKAG1	MRPL30	C12orf24	DLX1
	TAF8	UNC13C	PTPLAD1	CALCRL	ICA1L	NUP98	RNASE12	SCARNA22	TTTY7	TDRD6
	ABCE1	FAM168B	CDK7	WBP11P1	GDI2	FAM69B	ZC3H12C	UBLCP1	GDF6	PPRC1
	APOBEC2	UBE3A	TGFBR1	ACTR3	GART	ANKRD16	HEATR1	ICAM5	UBA2	LANCL1
	KCNS3	TMEM59L	CACNA1H	UCHL5	LOC729020	TRMT61B	TM2D2	TMEM151B	FAM66A	TSNAX
	OR2L3	SLC6A17	C16orf52	DDX1	FETUB	ACBD6	ATIC	METAP2	SLC7A13	ICK
	PDE3B	TTTY9B	PLEKHA2	DNAJB3	DIMT1L	RNF41	SYT4	RGPD8	WDR35	FGF8
	PCSK2	ERGIC2	TBC1D15	ACVR1	EEF1A1P9	SLC39A10	SP3	DOCK1	KLK2	PCGEM1
	EBF3	COL9A3	CYP1A1	SUMO1P3	WDSUB1	PCDHGB3	UBE2D1	SPAG16	HNRNPF	FAM126A
	ZNF317	BRMS1L	ZRANB3	SLC43A1	RAB3GAP1	TRIML2	PLCB4	KCTD15	HDLBP	FRMPD4
	TMSB4Y	C9orf4	C6orf154	SPIN3	NUDCD1	PPP4R4	TADA1	APIP	CYP7A1	ANKRD40
	GRPEL2	AMOTL2	DHX36	LCE2D	DYNC1I2	PRKY	TDRG1	NLGN4Y	IL1F8	TNFRSF21

	FTSJD2	GOLGA8E	RHBG	TMPRSS9	EIF1AY	PRDX3	IL20RB	RUFY2	UTF1	RNF8
	PPP2R5C	UTP18	ARG2	KRTAP5-2	WDR67	NPY	C1orf27	C15orf41	UPF2	GABRA5
	LOC84856	RABGGTB	NVL	FLRT1	CYorf15A	ABCA17P	TCFL5	STARD7	LOC202181	INSIG2
	IFITM5	CAPRIN1	ST3GAL5	CIAO1	SENP2	PNMA1	HSPC072	NAP1L1	LPPR1	VPS37A
	C5orf25	KRT32	KIAA2022	LHX6	PKNOX1	SIRT1	SMC6	CNOT7	C11orf46	KCNK13
	HOXD10	ARMCX5	DEGS1	TSEN15	C6orf130	SMARCAL1	TRIM59	ANKAR	TBCC	KLK3
	C2orf49	RSL1D1	TSG101	SMC3	OGT	PPP2R5A	HCRTR2	ZSWIM2	ANKRD13B	TFB2M
	IL7	SRF	EPC2	FAM117B	MORF4L2	ZDHHC13	CES8	EIF3J	KCNQ5	RPS3A
	CPSF6	SMPX	TOM1L1	NCRNA00176	CAPSL	FAM134A	LOC646214	ZRANB1	DCLRE1A	LSM14A
	BRIP1	C19orf2	SLC39A14	RGPD3	C2orf29	SNTB1	MGC34034	C12orf50	BMS1	IL12A
	GGNBP2	UBXN4	LIN7A	SDCBP	ADORA2B	IFIT3	PUS7	C2orf69	PTPLAD2	SSB
	GNG10	MPP3	MELK	TMEM5	C8orf71	C10orf2	MRPS18A	BZW1	MCPH1	LOC286467
	MANEAL	API5	LOC100130093	CASP7	C2orf60	RGPD4	CAMSAP1L1	PRKRA	KATNA1	MORF4
	SNX16	DUSP12	SEC23IP	C1D	MAP1D	DNAJC25-GNG10	MIA3	PLA2R1	UNCX	MAK16
	CCNJ	GORASP2	C10orf84	UXS1	EIF2AK4	ELSPBP1	MOBKL3	OR5B12	C14orf33	CCDC138
	JUB	GPR161	USP28	GTPBP4	ESM1	PIGC	SIM2	CBWD2	RAB27A	P2RX6
	OR5V1	PSG1	CHML	IVNS1ABP	XK	OR6C65	RPS4Y2	TOPBP1	BCCIP	TRIM58
	PROX1	RRAGB	PPP1R3C	SOX8	ALG13	CBS	POTED	FAM78B	RAB3GAP2	MCM6
	DNAJC24	SUMO1	ACP1	GAGE2B	TRIM53	OTUD6B	KHSRP	C5	PPIG	TFEB
	XRCC5	C2orf47	KRTAP4-12	ATF2	CYP26A1	NUP133	KIAA0125	PGBD3	ZFY	TWIST2
	RBM41	UGT1A7	EIF2S1	ITPKA	TERF1	KRTAP6-3	KLRC1	EDDM3A	RGS5	DCAF17
	TRAM1L1	SLC25A25	MYO1E	TGFBRAP1	ITM2A	ATP5C1	BYSL	GLS	TUBGCP4	FASTKD1
	ZNF716	SCN8A	C10orf18	AAMP	LDB1	PIK3C2A	IRX1	BUB3	RRP15	RYR2
	TXLNG	TBC1D22B	PHF16	UBE2V2	GIGYF2	NBPF14	BUB1	PRKAR2B	MORC4	RPS6KC1
	CCDC33	RCAN2	CCT4	C20orf199	DSCR8	ACTR6	OSGEPL1	WIT1	FRS3	JAM3
	HECTD2	BRD4	USP17	TAF1A	YEATS4	BLOC1S2	GPR27	WWP1	GRAMD1A	TMEM131
	MATN2	CSTF2T	WDR33	FNTA	ETF1	LRCH2	CNNM4	MST4	TAF9B	ADCY3
	LRRC4C	ZAK	IMP4	RANBP2	TMEM217	STAT1	ARID3A	POGK	SNAP47	NMD3

	IL6	ZNF143	PALB2	CBX4	MCM3	LEUTX	UBAP2L	HSPA12A	PFKM	BAGE2
	IRAK1BP1	PLD6	CKM	USP13	TMEM74	EIF4G2	SELV	ODZ2	OR5B2	FAM171A2
	FAM101B	GPX8	GSX2	PARP2	KLRC3	EIF2A	C6orf72	C8orf45	CDC73	CARS
	LMAN2L	SCRN3	CTSL3	MAGEB18	C12orf4	FBXO9	SHOX2	LOC284232	SHOC2	ALS2CR4
	RNF139	NLRP4	MC3R	TOR1AIP1	NFYB	METTL10	EYA4	SRP72	C1orf105	PLEKHM3
	TRIM37	C6orf155	RRM1	REPS1	GUCA1B	PSG11	RB1CC1	UNC5CL	RPAP1	HEATR2
	PRTG	MSH6	SEMA6D	SCML2	PMAIP1	OAT	THOC2	FOLR3	OR5AN1	PCDHAC2
	C6orf218	SIGMAR1	SENP1	USP16	PDZK1P1	ACCSL	SRFBP1	VSX1	TCEAL1	XRCC2
	FLJ35024	TTC27	STIM1	KCTD18	HOXA11AS	GRIN2A	SEMA6B	PGC	CAMLG	GPATCH4
	PIGW	YY1	OPA1	MCART6	ATP6V1C1	TAS2R3	CKAP2L	PABPC1	DCAF10	HPRT1
	DDX26B	NIPA1	DCUN1D5	NPM1	ARMC1	SLC33A1	PPIL5	POLR3D	NONO	TOR1A
	KDELC1	SGK223	UAP1	NAT10	TEX19	LYSMD1	AHCTF1	C10orf67	SF3B1	TRAK2
	HOXD8	RGPD6	PAIP1	LRAT	INPP4A	IKBKAP	TRAF5	CXorf56	C11orf82	C7orf45
	BPI	TLK1	DNA2	KCNK12	RGAG1	RCOR3	RQCD1	MID2	R3HDML	ANKRD27
	KRT85	OR2W3	CCT2	PCM1	DCTN6	GOLT1A	FOXA2	GOLGA8C	VGLL2	ZNF792
	DIAPH2	CCDC121	LOC255167	GSTTP2	LIN52	HLCS	OPTN	C12orf48	SNRNP27	FOXD1
	LOC285401	LRFN2	DNAJC27	DDX52	CHAC2	SNORA76	SLC25A30	OR51E2	ISM2	LOC153910
	PGAM5	CHURC1	TGS1	BRSK2	APOC3	PGAP1	PISRT1	CDX4	LOC728276	OR4C11
	OR52M1	OR5H2	OR7G3	OR8K5	PRM1	SAA3P	SPZ1	TMEM95	ADAMTS8	MARS
	ZNF280C	C8orf86	KRT38	ERCC6	TRIM3	DAGLA	PANK1	RBM7	TXNDC9	SLC4A5
	GABRR2	ZNF479	MRPS35	GPR20	DNM1L	MRPS9	FLVCR1	POLR3C	DDX42	RGS9BP
	FKTN	FAM184A	EFHA2	PLEKHA3	PEX19	RAD50	EIF3C	ADAMTS15	DCAF6	LASS1
	KRT35	C6orf64	CUL9	NR5A2	DNMT1	USP32	DDX21	FXC1	MDGA1	TSN
	TMEM38A	WDYHV1	VPS54	SLC23A2	ALS2	IGFL4	HNRPLL	ZNF333	LOC392196	ABTB2
	PTK7	SPINK6	LOC643955	C8orf75	UMPS	AASDHPPT	TMEM55A	GLT25D2	SLC4A1AP	CXorf51
	DEFB109P1	KRTAP20-2	NCRNA00112	OR4A16	RNASE9	MOCS1	SP9	KIAA1737	GALR1	ZNF679
	ITIH2	SMN1	MRPL42	TMPRSS15	MYBBP1A	COCH	ESF1	ZCCHC7	GNPNAT1	PAPPA2
	SAP130	PRR5L	SLC16A9	SPOPL	GPATCH1	C5orf44	RNF115	PHGDH	GPR63	KL

	TUBA3E	C1QTNF8	LDB2	C6orf129	SCN1B	MFF	FAM86B1	HOXD11	QSER1	PGM5P2
	FANCM	LRRC39	MC5R	RBM12	MBTPS2	COIL	METT5D1	SUCLG1	GABPB1	SSX6
	HOXA11	LIN9	MLL4	RNF149	RPAP3	KIF18A	ABCB10	SRD5A1	SRGAP1	KCNJ11
	C10orf131	CHM	RABGAP1	IL1F10	SUPT3H	PYGO1	MGC14436	FNIP2	CEBPG	AKT3
	IZUMO1	MTHFD1L	TTC9C	TAF9	TBCE	EDDM3B	C12orf11	NOL11	PTPRS	PIKFYVE
	40422	COX11	ZNF181	SFXN4	OR51B5	SGPP1	NEK7	ZBTB32	C1QL4	PGLYRP2
	PEX5	LOC100133545	FGFR1OP2	RPS23	LGR4	PDGFRL	MUC6	RBMX	CLIC5	SLC17A2
	CAPZA2	RAI14	GAPDHS	B3GALNT2	CYP2C18	ABHD15	SLC16A14	SSRP1	KDM5D	BTNL9
	TSR1	HSPB3	CYP39A1	C20orf144	ZNF618	DKFZp686A1627	HNRNPA3	C1orf25	ACVR1C	DGKD
	C14orf142	STARD4	SPC24	BEAN	CUL3	HPS5	PREX2	PWP1	ERO1LB	GLYATL2
	ANKIB1	PLAC1	MAGEC2	WNT3	TAF4B	ARL17B	NDUFS1	LOC407835	NLRP1	NOL10
	TTC30B	CNNM2	RDH14	ZNF507	LRP6	ESCO2	PNN	CWF19L1	ROCK2	ZNF98
	UBE2G1	YWHAQ	KCNIP4	KAZALD1	PATE4	TBX22	ADIPOR1	CPEB2	ZNF627	ASPM
	BCOR	MAGED4B	C3orf52	SLC35E3	TIPRL	TXNDC16	SLC6A15	EPB41L4B	FATE1	RTN4RL2
	GPR1	LOC100133957	SMARCE1	TYSND1	CYYR1	MKI67IP	ITIH5	WDFY1	SLFN12	LOC285627
	CPNE6	RPS10P7	ATF6	CASP12	FAM19A5	C19orf57	DNAJA3	NTRK2	METTL2A	RAB4A
	KCNH3	HTR3A	CDH17	HOXD3	UEVLD	FARSA	CECR7	TMEM147	ZNF343	MRAP2
	AKAP2	C17orf71	PRIM1	TNFSF14	QSOX2	GAGE2C	PVRL3	KIAA0892	CAMP	KLHL29
	TYRP1	SRPK1	FEZ2	GUCA1C	CCNI	STRADB	ADRA1B	BMPR1A		
	UNC50	PHOSPHO2	GTPBP2	POLR1B	USP49	ZNF318	EEF1B2	ANO4	MCM3APAS	POLH
	TJAP1	MAD2L1BP	KIN	CDC123	ORC4L	XPO5	NFYA	TTTY4C	MTX2	ENPP4
	PCGF6	GNMT	NIF3L1	AARS2	C1orf187	DDX50	RPL7	TTL	SLC35B2	TOMM20
	C2orf58	MED20	KIF6	TUBA4B	ERCC3	PDCD11	C10orf137	SLC25A16	VLDLR	YIPF3
**LOST FROM S3**	SLC25A27	C16orf81	PPP2R5D	ORC2L	MUT	SLC22A15	LOC730101	ENPP5	KLHDC3	UBR2
	LOI39142	TIAL1	PRICKLE4	CYorf15B	BPY2	TAS1R3	POLR1C	SNORD1C	BRS3	VEPH1
	KLHL23	MIPOL1	PROX2	FAM66C	KCNN2	RPP38	ABCC10	C10orf88	EEF2	WNT8B
	ATP1B4	RPS4Y1	TCHH	PRPF18	C2orf89	SOX3	CASP5	NFE2L2	DDX18	GPATCH2
	CENPQ	NCRNA00188	FNDC8	C20orf160	FBXO18	TTLL6	PHYH	SEC61A2	FOXP4	FLJ10357

	ZNF207	MAP3K15	SERPINA7	LOC100132354	DDX3Y	C2orf3	SPHKAP	OR4F21	SNAR-B2	SNAR-C3
	ASNSD1	TRMT5	C6orf153	CHRM3	TLK2	SEPHS1	LPA	PNPT1	UTP14A	CUL7
	LOC100287718	COL4A3	ZDBF2	KIAA0240	CWC22	ZFAND3	FAM178A	RGS11	C6orf138	MRPS10
	GPR162	WT1	OLA1	CHRNA4	PEX6	IRX2	SNHG4	RNU6ATAC	EEF1A1	SLC25A12
	AP3M2	NUP35	WIF1	MEA1	CCNB1IP1	TRERF1	GCA	ABCB7	GTF2H2C	BEND7
	FAM86B2	FARSB	B3GAT1	UTP20	PRKAG1	MRPL30	C12orf24	DLX1	TAF8	UNC13C
	ICA1L	RNASE12	SCARNA22	TTTY7	TDRD6	CDK7	RBM17	WBP11P1	FAM69B	ZC3H12C
	UBLCP1	APOBEC2	UBE3A	ST6GALNAC3	NAMPT	ANKRD16	ICAM5	PPP1R3B	UBA2	NUDT5
	COL27A1	KCNS3	TMEM59L	TRMT61B	TMEM151B	FAM66A	TSNAX	OR2L3	C16orf52	DDX1
	ACBD6	SLC7A13	ICK	TTTY9B	FMNL2	DIMT1L	RNF41	SYT4	RGPD8	WDR35
	FGF8	PCSK2	TBC1D15	EEF1A1P9	SLC39A10	KLK2	PCGEM1	COL9A3	CYP1A1	SUMO1P3
	WDSUB1	PCDHGB3	SPAG16	FAM126A	ZNF317	MLLT10	SDCCAG8	BRMS1L	SLC43A1	FRMPD4
	TMSB4Y	C9orf4	C6orf154	WFDC6	SPIN3	PPP4R4	TADA1	APIP	CYP7A1	DAP3
	GRPEL2	AMOTL2	LCE2D	PRKY	NLGN4Y	TNFRSF21	FTSJD2	TMPRSS9	EIF1AY	IL20RB
	EIF4B	UTF1	RNF8	PPP2R5C	KRTAP5-2	C1orf27	GABRA5	LOC84856	RABGGTB	NVL
	FLRT1	CYorf15A	TCFL5	STARD7	LOC202181	IFITM5	ST3GAL5	CIAO1	HSPC072	NAP1L1
	LPPR1	C5orf25	SMC6	CNOT7	C11orf46	KCNK13	TSEN15	C6orf130	SMARCAL1	PIP5K1A
	ANKAR	TBCC	KLK3	TSG101	OGT	PPP2R5A	HCRTR2	ZSWIM2	ANKRD13B	SRF
	FAM117B	CES8	KCNQ5	RPS3A	CPSF6	SMPX	NCRNA00176	CAPSL	FAM134A	LOC646214
	DCLRE1A	LSM14A	BRIP1	C12orf50	BMS1	IL12A	GGNBP2	PUS7	C2orf69	SSB
	MELK	TMEM5	C8orf71	C10orf2	MRPS18A	MCPH1	LOC286467	MANEAL	LOC100130093	C2orf60
	CAMSAP1L1	PRKRA	KATNA1	SNX16	DUSP12	MAP1D	DNAJC25-GNG10	PLA2R1	UNCX	MAK16
	CCNJ	GPHN	C10orf84	ELSPBP1	OR5B12	C14orf33	YOD1	CCDC138	JUB	PIGC
	SIM2	CBWD2	OR5V1	CHML	OR6C65	BCCIP	RRAGB	PPP1R3C	ALG13	CBS
	POTED	RAB3GAP2	DNAJC24	APEX1	GAGE2B	TRIM53	KHSRP	C5	TFEB	XRCC5
	KRTAP4-12	CYP26A1	NUP133	KIAA0125	ZFY	ANKRD36B	RBM41	ZEB1	UGT1A7	ITPKA
	KRTAP6-3	EDDM3A	RGS5	DCAF17	TRAM1L1	SLC25A25	ITM2A	ATP5C1	FASTKD1	ZNF716
	SCN8A	PIK3C2A	IRX1	RRP15	RYR2	TBC1D22B	SUPV3L1	AGBL1	PRKAR2B	RCAN2

	C20orf199	DSCR8	ACTR6	OSGEPL1	WIT1	FRS3	HECTD2	USP17	YEATS4	GPR27
	GRAMD1A	WDR33	FNTA	LRCH2	TMEM161B	TAF9B	IMP4	PIM1	TMEM217	STAT1
	ARID3A	POGK	SNAP47	IL6	ZNF143	PALB2	MCM3	LEUTX	PFKM	BAGE2
	IRAK1BP1	PLD6	CKM	SELV	ODZ2	PARP2	KLRC3	EIF2A	SCRN3	CTSL3
	MAGEB18	C12orf4	FBXO9	PPM1D	LOC284232	ALS2CR4	NLRP4	MYO19	MC3R	NFYB
	METTL10	EYA4	C6orf155	REPS1	GUCA1B	PSG11	UNC5CL	HEATR2	PRTG	SEMA6D
	SIP1	SCML2	FOLR3	OR5AN1	C6orf218	MYLK	SIGMAR1	SENP1	USP16	PDZK1P1
	SRFBP1	VSX1	MAP3K7	TCEAL1	XRCC2	FLJ35024	KCTD18	GRIN2A	CAMLG	GPATCH4
	PIGW	MCART6	TAS2R3	DCAF10	HPRT1	DDX26B	NPM1	PPIL5	NONO	KDELC1
	SGK223	NAT10	TRIP13	TEX19	LYSMD1	AHCTF1	C10orf67	SF3B1	TRAK2	RGPD6
	PAIP1	LRAT	IKBKAP	TRAF5	C11orf82	DNAH14	CYR61	BPI	DNA2	PRMT5
	RGAG1	RCOR3	R3HDML	KRT85	DCTN6	FOXA2	VGLL2	ZNF792	CCDC121	LOC255167
	GSTTP2	KCNJ8	FOXD1	LRFN2	DNAJC27	SNORA76	SLC25A30	OR51E2	ISM2	LOC153910
	CHURC1	APOC3	PGAP1	CEP78	PISRT1	CDX4	KRTAP10-1	LOC728276	OR4C11	OR52M1
	OR5H2	OR7G3	OR8K5	PRM1	SAA3P	SPZ1	ADAMTS8	MARS	NPM3	ZNF280C
	C8orf86	TRIM3	DAGLA	PANK1	TXNDC9	GABRR2	RNF150	ZNF479	MRPS35	GPR20
	MRPS9	FLVCR1	POLR3C	DDX42	FAM184A	EFHA2	ADAMTS15	SNORA62	TIMM9	C6orf64
	CUL9	FXC1	MDGA1	ZNF333	C20orf141	PTK7	SPINK6	LOC643955	UMPS	CXorf51
	DEFB109P1	KRTAP20-2	NCRNA00112	OR4A16	RNASE9	MOCS1	SP9	KIAA1737	GALR1	ZNF57
	ZNF679	ITIH2	TMPRSS15	MYBBP1A	COCH	ESF1	ZCCHC7	SNORA72	SLC16A9	ADAM21
	TAS2R31	GPATCH1	C5orf44	GPR63	KL	TUBA3E	C1QTNF8	C6orf129	SCN1B	MFF
	FAM86B1	QSER1	PGM5P2	FANCM	LRRC39	MC5R	MBTPS2	COIL	METT5D1	SUCLG1
	LIN9	MLL4	ABCB10	APPBP2	C10orf131	WDR11	RABGAP1	IL1F10	SUPT3H	IZUMO1
	TAF9	LOC100128640	TBCE	EDDM3B	NOL11	PIKFYVE	GADD45B	ZNF181	WBP2NL	SFXN4
	OR51B5	NEK7	ZBTB32	C14orf126	C1QL4	PGLYRP2	PEX5	LOC100133545	RPS23	RBMX
	CLIC5	SLC17A2	CAPZA2	RAI14	GAPDHS	B3GALNT2	CYP2C18	SLC16A14	KDM5D	BTNL9
	CXCL3	TSR1	HSPB3	C20orf144	DKFZp686A1627	HNRNPA3	C1orf25	ACVR1C	DGKD	C14orf142
	STARD4	BEAN	CUL3	PWP1	ERO1LB	GLYATL2	ANKIB1	MAGEC2	WNT3	RPL26

	ARL17B	MTPAP	LOC407835	NLRP1	NOL10	TTC30B	RDH14	ZNF507	PNN	CWF19L1
	ZNF98	UBE2G1	KCNIP4	PATE4	TBX22	CPEB2	ZNF627	MAGED4B	SLC35E3	TIPRL
	SLC6A15	FATE1	GPR1	LOC100133957	SMARCE1	TYSND1	MKI67IP	SEC11A	ITIH5	LOC285627
	CPNE6	RPS10P7	CASP12	FAM19A5	CEP170L	NTRK2	EHBP1	METTL2A	KCNH3	CDH17
	FARSA	TMEM147	ZNF343	FAM66E	PRIM1	TNFSF14	GAGE2C	KIAA0892	TYRP1	STRADB
	ADRA1B	BMPR1A								
**CONSERVED IN S3/S4**	ZC3H8	PRMT3	GTF3C3	TUBGCP5	ZC3H15	FASTKD2	CDC5L	HSP90AB1	GAD1	C11orf58
	HSP90AB2P	SLC29A1	CD2AP	SUV39H2	TFAM	ELOVL5	E2F5	NIPA2	ATAD1	FAM163B
	C10orf119	RPL7L1	OTUD1	AFF3	NCKAP1	PRPS1	FAM45A	RPE	TEX10	TMEM185B
	LRPPRC	LOC728190	SLC35F5	MINPP1	WDR12	AGFG1	C5orf30	LONRF2	NCL	PATL1
	VPS26A	GAS2L3	XPOT	CEBPZ	SMNDC1	IPO7	VEGFA	TAF3	HKDC1	WDR43
	SGTB	HCN2	HSPA14	NUAK2	SNAPC1	TARS	MOAP1	GCC2	DIRAS1	GABRB3
	PRPF40A	DNAJC10	OR2L1P	EIF2S3	NMT2	GOLGA7	GLRX3	MARS2	TMEM14A	HAT1
	MAP4K3	COL21A1	PYCR1	NOLC1	TTC21B	MMADHC	USP6NL	NOC3L	FRMD4A	NOP58
	IWS1	LRRC1	DCAF16	ASAP1	FAM175B	TMEM182	RNF141	TCTE1	PPAT	ZFAND1
	ACCN2	GLO1	CPS1	USP47	AZIN1	TCEA1	PAICS	CSGALNACT1	RCL1	TMEM63B
	TMEM20	INTS7	ARL5A	COL4A4	PNO1	SPIRE1	MKX	GMPS	AP3M1	NPC1
	CKAP5	FAM45B	GDF15	GTF2H1	SLC25A32	EIF3A	AMMECR1L	MCM10	RSRC1	POL37500
	HAUS6	TTC17	CHUK	PLS3	PABPC3	HPS3	TRUB1	R3HDM1	DCAF12L2	TRAM2
	FGFR2	IBTK	KIAA1598	CALCOCO2	ABI2	TRAM1	MTHFD2	FZD5	PPP2R2A	ANAPC1
	THAP1	C1orf43	UBE2A	EIF5A2	GPN1	PTPLAD1	CALCRL	NUP98	ABCE1	FAM168B
	GDI2	GDF6	PPRC1	TGFBR1	ACTR3	MAP4K4	GART	HEATR1	EPRS	LANCL1
	CACNA1H	UCHL5	LOC729020	TM2D2	SLC6A17	CDC27	FETUB	ATIC	METAP2	PDE3B
	VTI1A	PLEKHA2	DNAJB3	RAN	MTERFD2	ERGIC2	KPNB1	ACVR1	TIMM17A	SP3
	DOCK1	EBF3	DARS2	STAC	UBE2D1	GDPD1	HNRNPF	MRPL3	ZRANB3	RAB3GAP1
	TRIML2	GCLC	PLCB4	KCTD15	HDLBP	NUDCD1	ANKRD40	IARS2	DHX36	DYNC1I2
	EXT1	TDRG1	IL1F8	GOLGA8E	RHBG	PRDX3	RUFY2	UTP18	ARG2	WDR67
	NPY	C15orf41	UPF2	COPB1	ABCA17P	INSIG2	KIAA0020	CAPRIN1	SENP2	PNMA1

	VPS37A	KRT32	KIAA2022	LHX6	PKNOX1	SIRT1	HOXD10	ARMCX5	DEGS1	TRIM59
	C2orf49	RSL1D1	SMC3	TFB2M	IL7	EPC2	MORF4L2	PDHX	ZDHHC13	EIF3J
	CLIP4	TOM1L1	MNAT1	ZRANB1	C19orf2	SLC39A14	RGPD3	C2orf29	SNTB1	MGC34034
	UBXN4	LIN7A	SDCBP	ADORA2B	DIAPH3	IFIT3	PTPLAD2	GNG10	MPP3	BZW1
	API5	VDAC2	CASP7	RGPD4	MORF4	TCP11L1	SEC23IP	C1D	MIA3	ECD
	GORASP2	STAM	UXS1	EIF2AK4	MOBKL3	GPR161	USP28	GTPBP4	ESM1	IARS
	RAB27A	P2RX6	PSG1	IVNS1ABP	XK	ARID3C	RPS4Y2	TOPBP1	TRIM58	PROX1
	SOX8	FAM78B	MCM6	SUMO1	ACP1	OTUD6B	PPIG	C2orf47	ATF2	PGBD3
	TWIST2	EIF2S1	TERF1	C1orf107	BDNF	KLRC1	IPO9	EIF2S2	MYO1E	TGFBRAP1
	NCRNA00120	BYSL	GLS	TUBGCP4	C10orf18	AAMP	LDB1	FAM21A	BUB3	TXLNG
	PHF16	UBE2V2	GIGYF2	NBPF14	BUB1	MORC4	RPS6KC1	CCDC33	CCT4	JAM3
	BRD4	TAF1A	BLOC1S2	WWP1	TMEM131	MATN2	CSTF2T	ETF1	CNNM4	MST4
	ADCY3	LRRC4C	ZAK	RANBP2	PLK2	NMD3	JPH1	CBX4	UBAP2L	HSPA12A
	USP13	TMEM74	EIF4G2	OR5B2	FAM171A2	FAM101B	GPX8	GSX2	C6orf72	C8orf45
	CDC73	CARS	LMAN2L	FAM119B	SHOX2	LRT38412	SHOC2	RNF139	TOR1AIP1	EXOC6
	SRP72	C1orf105	PLEKHM3	TRIM37	RRM1	RB1CC1	RPAP1	MSH6	GLT25D1	PMAIP1
	OAT	THOC2	PCDHAC2	ACCSL	TTC27	STIM1	HOXA11AS	SEMA6B	PGC	YY1
	OPA1	ATP6V1C1	PFKFB3	CKAP2L	PABPC1	NIPA1	DCUN1D5	LOC650623	ARMC1	SLC33A1
	POLR3D	TOR1A	UAP1	HOXD8	INPP4A	CXorf56	C7orf45	TLK1	KCNK12	RQCD1
	MID2	ANKRD27	SLED1	OR2W3	CCT2	PCM1	GOLT1A	GOLGA8C	DIAPH2	LIN52
	HLCS	OPTN	C12orf48	SNRNP27	LOC285401	CSGALNACT2	DDX52	CHAC2	GARS	PGAM5
	TGS1	BRSK2	C9orf25	TMEM95	FXR1	KRT38	ERCC6	RBM7	SLC4A5	DNM1L
	RGS9BP	FKTN	PLEKHA3	PEX19	ZFP36L1	RAD50	EIF3C	DCAF6	LASS1	KRT35
	NR5A2	DENND5A	DNMT1	USP32	DDX21	RNF2	TSN	TMEM38A	WDYHV1	VPS54
	SLC23A2	ALS2	IGFL4	HNRPLL	LOC392196	ABTB2	RAD18	C8orf75	AASDHPPT	TMEM55A
	GLT25D2	SLC4A1AP	SMN1	MRPL42	GNPNAT1	PAPPA2	SAP130	PRR5L	NAA35	PPIF
	SPOPL	RNF115	PHGDH	LDB2	HOXD11	RBM12	GABPB1	SSX6	HOXA11	RNF149
	RPAP3	KIF18A	SRD5A1	SRGAP1	KCNJ11	ALDH18A1	CHM	PYGO1	MGC14436	SNX6

	FNIP2	CEBPG	AKT3	MTHFD1L	TTC9C	C12orf11	PTPRS	40422	COX11	SGPP1
	FGFR1OP2	LGR4	PDGFRL	MUC6	STRBP	LDLRAD3	ABHD15	LOC145783	SSRP1	CYP39A1
	ZNF618	GTF2H3	SPC24	HPS5	PREX2	PLAC1	TAF4B	NDUFS1	CNNM2	LRP6
	ESCO2	ROCK2	YWHAQ	KAZALD1	DDIT3	ADIPOR1	ASPM	BCOR	C3orf52	TXNDC16
	EPB41L4B	RTN4RL2	CYYR1	WDFY1	SLFN12	ATF6	C19orf57	DNAJA3	RAB4A	HTR3A
	HOXD3	UEVLD	CECR7	MRAP2	AKAP2	C17orf71	STX17	QSOX2	PVRL3	CAMP
	KLHL29	SRPK1	FEZ2	GUCA1C	CCNI					
**ACQUIRED IN S4**	HSPA4L	PTGES3	UBE2H	PSMD11	STIP1	KIAA0368	CDV3	PAQR5	FBXO22	IL28RA
	REEP3	PTK2	PANX1	RNF19B	C19orf55	EIF4A3	ARL 15.00	SLC7A1	YWHAG	YARS
	HSPA5	UBQLN1	ANKRD57	RSC1A1	HNRNPR	CHRNA3	C10orf46	ALOXE3	INSR	ACER2
	YME1L1	MAPK8	CAT	RAB2A	NEU3	SLC16A4	CCNDBP1	SORD	UGT8	PARD3
	RAD23B	EEF2K	SLC31A1	SLC20A1	TGM4	LOC387647	EIF2C3	ERLIN1	TGFA	NAA50
	MOCOS	MYO5A	ZSCAN20	CPD	RASEF	HIST2H2BA	RNASEH1	GRLF1	DFFA	UBN1
	UBFD1	NDRG1	DNMBP	PTP4A2	CTH	TDRD7	UNC5A	RTN3	PTGFR	RAB9A
	CAB39	INSM2	TMED5	ZDHHC9	ARFGEF1	SPAG1	KRT80	TMEM184C	GFPT1	ANKRD56
	MRPL19	ARF4	ATP13A3	HIST1H2BE	MAP2K1	UGCG	PSMB2	STXBP1	G3BP2	COL4A6
	MEGF9	HIST1H2BC	NMT1	EEF1DP3	RRM2B	ITPR3	MGLL	KLHL8	KCTD8	SYAP1
	FAM110C	L1CAM	HERC3	SARS	SEL1L3	TRPS1	EIF4G3	KJT40057	LRRC57	NRBF2
	ERO1L	PAFAH1B2	BICD1	KHDRBS3	LITAF	HSP90AA1	TEX2	GCLM	UGT1A1	DOCK5
	EDEM1	C10orf12	RAP1GDS1	PLIN3	WLS	UGT1A6	AMFR	SLC10A6	AHCYL1	FDFT1
	MFSD9	SGPP2	LEPROT	EIF2C2	RBBP7	GABARAPL3	ORAI1	SLC2A1	ARL13A	EIF1AX
	SLC35F2	USP12	IQGAP1	EDA2R	SLC7A11	UHRF1BP1	DOCK7	UBE4B	RPA4	JMY
	PRRG4	ZBTB38	DDX3X	FLJ45983	KPNA6	ASCC1	STRAP	SDC1	RBBP5	CLTC
	TRIM44	WWTR1	PCDH1	LAPTM4B	ANXA5	SLITRK2	OSBP	PTPLB	TRIB3	LRP1B
	CAMK2N1	ZDHHC18	OR2AE1	GATA3	MOBKL1B	PDXDC1	GXYLT1	RBBP4	APC2	FAM163A
	SYTL5	KGFLP1	ZFYVE9	TSPAN5	ZNF526	TGFB1	KCNMA1	CAPN2	CSDE1	PTENP1
	PDE1A	RALB	UBC	PCSK5	FAS	SPTY2D1	PHEX	DYNLL2	RPS6KA5	SNORA23
	FIBCD1	PCYOX1	GMCL1	TUBA4A	SPPL2A	GSPT1	KTN1	MAFG	USP31	SRPX

	PEX2	NT5DC3	KIAA0090	DDI2	AVPR2	SIPA1L2	DCTPP1	HECW2	IGF2R	VWA5B2
	ZNF365	JAK1	VEZT	LRRC58	DPP10	RBBP8	MTDH	VWF	CNBP	KDM6A
	GRIN2B	RBM18	SPINT1	IDI1	TM9SF3	TRIP12	FAM199X	ANXA2P2	RSF1	VAMP7
	PPP2R2C	PTGR1	FAM38B	RTN4	SFN	LOC283856	HIST1H1C	RAD21	RRAGC	LMO1
	METAP1	RAB30	IDE	RGP1	CCRN4L	ELAVL2	MSN	SLBP	ALCAM	BTBD10
	ZNF516	B3GALT1	GATC	PSAP	CPNE3	CAMK2N2	LIMS1	MAGI3	SLC25A24	CUL2
	SNX25	C3orf36	EAF1	RCC1	RDH10	PDZD4	FOSL2	RNF213	LIMCH1	PSMD10
	C14orf43	TMOD1	TMEM189-UBE2V1	PPP2R5E	APLP1	PTPN14	ARHGAP11B	IGF1R	LGALS3	C9orf40
	NOMO2	CSNK2A1P	KIF2A	KLHL13	HOMER1	MBOAT2	FAM171B	GSR	C16orf5	LRRFIP1
	NEURL1B	HOXA6	STT3B	FAM102A	CACNA1G	SLC38A1	LDHA	XPO6	CORO2A	LOC100131193
	SURF4	ASCC3	COPB2	AGAP1	PVR	ZNF146	HIST2H2BE	PARP1	MLLT11	POLR3A
	C1orf161	LEMD1	FERMT2	C9orf5	SLMAP	MAN2A1	NRP1	VAV3	PPP4R2	GDE1
	ACSL3	PCDH17	TRNP1	NXPH1	NOP14	PSTPIP2	TMEM56	SMARCA5	TMEM159	DUSP5
	CNTN3	STK39	TMSB15A	SLC44A1	FIP1L1	DDB1	EIF4G1	CHRNA5	SIX4	AK3L1
	ITGB3	SLC35D1	LMAN1	COL22A1	CCDC6	EYA3	FCN2	GRID1	YIPF4	UBR5
	BRAF	RNF125	PANX2	UST	GRN	POP1	FAM160A1	TGOLN2	SLCO3A1	ARHGEF35
	RALGAPA2	HBQ1	C10orf99	RNF24	SPTBN1	LRRC3	THRAP3	POLR1A	MGC27382	GCNT3
	FTH1	YEATS2	RAB27B	PRKCA	NFIL3	DEFB132	P4HA1	SEMA4B	MLXIP	TEAD1
	YAP1	GABARAPL1	LARP1B	RAB6A	CA5B	ZFHX3	PTCHD1	YY2	CACHD1	VAMP3
	PTDSS1	GPR157	ADAM17	B3GNT8	ATP1B3	AQR	ZNF697	CA12	TMEM132B	CTNNAL1
	CPNE9	H2AFZ	SLITRK6	GBE1	G6PD	RASL11A	KIAA0196	NIPAL1	KDM4DL	CHMP4C
	GPR52	MTF1	P2RY1	PLEKHB2	SAMD4B	INSC	FZD6	ADCY1	WNT9A	GRIK5
	FAM81A	CHP	BMPR1B	ANXA6	NCRNA00235	ARL5B	PTPRZ1	FAM108C1	DENND4A	EIF2AK2
	BRI3BP	HEG1	STAMBP	CDKN2B	KIAA1279	UCHL1	LRRC59	ARHGEF17	TMEM45B	ZC3HAV1L
	ADM	LZIC	C1orf183	VCL	FTHL3	GRIK1	SPARCL1	FUT11	LRP12	DDIT4
	RNF103	EXOC6B	RAB40B	SLC16A5	TANC2	SLC25A41	IL6R	NHLRC4	MMD	MMP16
	GPR56	LPHN2	YPEL4	ATP11B	CD82	LASS6	ITGA3	EFTUD2	RCAN3	VPS24
	SERP1	PRKAR1A	SRCRB4D	HOXB6	FAM174B	STAG1	GPRC5A	KCNG1	HN1	SLC5A3

	FRMPD2	KIF3C	GPC3	ETFA	ATP6V1A	CMTM8	PARM1	TMBIM1	ZFR2	RMND5A
	SLC9A2	ZNF584	RAB10	SCCPDH	REXO2	CALM3	LRRC42	MTSS1L	NOL9	KIAA1244
	C9orf30	PRG1	RETSAT	RALBP1	OSGIN2	B4GALNT1	PFKP	PGRMC2	TBC1D16	SLC30A1
	RNPEP	TMOD3	SRD5A2	ZDHHC5	DHX9	ZNF460	INPP5F	PTP4A1	MAP7D2	CBR1
	DNAJA1	CTNNA1	LOC595101	C10orf28	TCF23	DIAPH1	RICH2	LRIG1	STARD8	TYMS
	ZNF609	HSF1	C6orf25	ARIH1	CHST15	EXT2	HPSE	PPM1B	ARRDC3	C4orf19
	IPMK	C9orf129	ISCA1P1	PRR19	ACOT7	GDF7	ABCC3	ZBTB10	SPRED2	USP7
	BCL9	CCND1	SLFN5	PPP3R1	CBX2	FBXO45	FAM3C	TNPO1	CDIPT	FOXF2
	EIF1B	DUSP13	BCL10	MORF4L1	EPB41	SNURF	MAST4	SH2D4A	EIF2AK3	PPP1R2P1
	SLC22A23	ABP1	RAP2B	EPDR1	SNRPA1	DDIT4L	SERTAD2	TARDBP	NCK1	PPP1R8
	ULBP2	AFF1	HSPG2	UBE2L6	HIST1H1E	SESTD1	ADAM22	PRRG1	NLN	SHISA4
	C4orf49	FMN1	NIPAL3	ANGPT4	ERRFI1	PRKCE	VWDE	INHBB	LEPROTL1	GLYR1
	HSPD1	CCT8L2	ESPL1	PRR11	MCFD2	KPNA1	RASSF8	KCND3	FUBP1	ATG4A
	MOBP	ABCB6	BAIAP3	RHOC	TPBG	KAT2B	EFNA2	HNRNPH3	C4orf34	CDK15
	TRIAP1	SC4MOL	NOTCH1	ADD3	AP3S1	OR2L2	CANT1	VHL	TSPAN3	SCARB2
	DHCR7	BTC	CPA6	BAG4	C16orf72	PEAR1	CDCP1	MAN2B2	C4orf37	SOX13
	LOC653653	PLOD1	NSF	GOLGA8DP	NPTX2	TUFT1	HOXD1	CNKSR2	SAE1	TG
	ZNF281	BMP3	MGAT5	SEC23A	PTEN	KIF1A	CD47	CTNNB1	SPATS1	SERINC3
	SLC39A6	ZW10	PRNP	SYT16	ITGA6	DENND4C	RGAG4	ITGB5	LOC344967	SOD1
	SLC30A6	EFEMP1	ARCN1	NKIRAS1	MAP3K9	SEC24D	CSPG5	MAN1A2	MCF2L2	LOC728323
	FAM59A	FGFRL1	BCAR3	UCK2	ITGA1	PTPN3	TPO	ODF3	OR52W1	SLC38A8
	PPTC7	C9orf41	NOS1AP	STK32A	EPHA2	COX15	PSMD2	AGRN	DYM	C3orf39
	B4GALNT3	RFPL2	TMF1	NAA15	TBL1XR1	GOLGA8G	KIRREL	NETO2	DR1	CTNND1
	MED28	ITGAV	PPM1L	SWAP70	ECT2	EPHA5	ADAM23	SCYL2	TMED3	NAV2
	CXorf22	SNORD115-26	TMCO3	LOC441208	SPTA1	LYPD6	CDKN1A	DTX3L	HIST1H1T	PRC1
	CTNNA2	ZNF366	ITGA2	CCT7	NPDC1	MXD1	CUEDC1	LRRC3B	PLA2G4E	CMYA5
	CGN	WAC	ABI1	ADI1	DCUN1D3	MAFA	POU3F2	SPRED3	ITPR1	STC1
	ACTN1	OLIG1	MEST	PLCXD2	MECOM	UBE2W	PGM3	ATCAY	ACER1	MCOLN2

	GRPR	PFN2	DLL3	FOLR4	UBR4	FKBP4	HECTD1	TNIK	FOLH1	C20orf54
	EPHA6	XIST	GBP2	CEPT1	SUSD4	TSC22D2	SLCO2A1	ST3GAL1	NLGN4X	HEY1
	HN1L	NTSR2	CPOX	C1orf103	OTOP2	GNL3L	C6orf106	EHD4	AMHR2	SNRNP40
	LRRC28	CREG1	DNM2	EPAS1	PSMD1	PDZD8	C1orf9	PKM2	FLT1	SMAD3
	CA10	ERMP1	GABRA1	ITGB1	RNF4	FECH	NEDD4	ITSN2	C9orf53	GATSL2
	SLC41A2	MRPL49	IRS2	ARHGEF12	AEN	UBE2O	GOLGA7B	SLCO4A1	PINK1	DDX60
	RAB43	PAK2	CXorf1	MAST2	ITGB6	MGAT4B	GTF3C4	NAV1	SV2C	RAB1A
	C10orf118	KCTD5	PCGF5	SLIT2	JMJD6	SHISA9	LRRC37A4	EFR3A	IL10RB	HSD3B2
	RPS6KA6	CDKN2AIP	IGFBPL1	TNKS1BP1	AADAC	FNDC3B	DCUN1D1	EXD1	CTBP2	FBXL16
	SGMS2	ZIC2	TMEM63C	RNF182	TBC1D2	MYBL1	DSTN	AVPI1	NCS1	AK5
	IGFBP3	RNF11	STYK1	UBAP1	DPY19L1	SUCNR1	IL13	MGAT5B	VBP1	FLNA
	WBSCR17	UGT1A4	ATP1B1	TJP2	JAKMIP3	RAET1K	GALNT13	METTL11A	WWC3	KIAA1324
	FAM69C	FAM63A	PDK4	MRPS6	TMPRSS7	PCDH19	DEPDC1B	ERN 1.00	CERCAM	NME6
	C1orf58	FAM114A1	DNAJC3	CD44	ATE1	TRIM36	BAG3	NUP210	EI24	POLR3E
	ABL1	DNAJB14	IPO11	FAM108B1	GLB1	ARHGAP36	MDM2	DOC2B	UGP2	CYP11B1
	PPM1K	CIZ1	CCNF	MRPL13	C3orf49	CDH7	CHRNB3	CLVS2	CYP11B2	DYTN
	FAM25B	FGF10	FRMPD2L1	GAST	GPR6	GRXCR1	GSTA3	GUCY2GP	HEATR7B2	IL1F6
	LOC100130274	LOC144742	LOC255025	LOC283332	MC2R	MRAP	MSGN1	NPVF	OR10Q1	OR1J4
	OR1N1	OTOP3	PIWIL3	PPBPL2	PPY2	PRAMEF2	PSG10	SNORA71B	SSTR4	HCN4
	UBE2K	PGM2L1	SQLE	ZDHHC20	UCP2	TP53BP1	ZNF500	MAPK6	SCNN1G	MFSD6
	PNP	RAPGEF5	CAP1	CDH18	RRP1B	CREM	ODAM	CDYL2	PRSS23	ROR1
	WFS1	PARP14	DMRT1	HIST3H2BB	GJC1	RASA2	MYO1B	SAMD8	STAR	DRT39508
	KAL1	RGMB	ABCA12	P4HA2	PODXL2	TAF13	ASPH	TPP1	SFMBT2	SLC26A7
	HAND2	ZNF263	ACSL4	MBOAT7	BIRC6	LOC729082	ARL6IP1	YPEL5	YWHAZ	NR5A1
	SBNO1	NRG4	NGRN	STMN4	CIB1	KIAA1217	FAM120A	ETV3L	DAZAP2	SCNN1B
	BBX	IDH1	KIAA1429	C4orf32	SLC35A2	STAT3	GATSL1	CSRP2	TTC7B	UBQLN2
	PDE12	PARP15	PTAFR	GPSM2	HSN2	MCTP1	QSOX1	CLCN3	ZCCHC2	TMX4
	SPNS1	TXNRD1	ACAN	ZNF295	PDPK1	PRKX	ZNF827	PPAPDC1B	NLRP10	ZDHHC22

	ALDOA	ASAP2	HIST3H3	NID1	IRAK1	ERGIC1	ACTG1	C3orf58	MBNL3	ADCYAP1
	FAM107B	CD93	C9orf140	CLDND1	CHRM4	PDE5A	CELSR2	CT62	HYAL3	ARL4D
	PLEC	CHMP2B	PIR	TSPAN14	ALG11	MYOF	MRPL33	OSMR	CPEB1	HPCAL1
	HIF1AN	QPCTL	MED14	ATP2B1	TSPAN15	TEX9	ZNF774	TXNDC11	ANXA4	TMEM206
	TNKS2	ZNF623	BAALC	HOXB8	GPBAR1	GHRHR	GRAMD3	GPR116	SYCP2	C12orf53
	CALB1	GNL3	MACF1	KIAA1958	PGD	APP	TFAP2A	CXorf38	CTBS	LOC641298
	TMEM109	FAM168A	TMEM47	RNF10	USO1	ARHGAP21	AADACL4	NPTXR	RBM15	PELO
	DERL1	TNFRSF11B	TAF2	MBP	LOC100129550	ROD1	PTCD2	SULT2A1	SLC48A1	ATAD2
	KDR	INA	PCTP	ZNF813	PCDHGA12	AFAP1L2	SPRY1	CHST8	LHFPL1	PLXND1
	ECE1	LDOC1	LHX1	PRIMA1	USP8	HIST1H2BK	RABGAP1L	ETV3	GMNN	YIPF6
	GNB1	STBD1	CSNK2A1	LOC652276	GPD2	TMC7	MESDC2	GNAI3	DLK1	ICMT
	TMEM66	TMED7-TICAM2	GPR87	HIST1H2BJ	HAPLN1	KLHDC10	ANO6	ZNHIT6	ACER3	FBXW11
	CA7	ACTR2	PITRM1	MPZL3	C1orf163	CRLF1	CHMP1B	LOC284441	ROBO1	ZNF488
	STRN	MAPRE3	ZNF616	CSDA	RXFP1	GCOM1	UBIAD1	TUSC3	PGAM4	KPNA7
	PPP1R12A	RAB38	VWC2	ZC3HAV1	MED12L	ZMAT3	BEX5	C11orf86	TLE1	PSME4
	DIRAS2	TMPRSS11A	ZFP64	IMPA1	CCT3	CAMK1G	USP38	CD276	TPRG1L	PCDHA13
	COPS4	GNA11	ILDR2	TNFAIP8L1	PEF1	KIAA0319	GAB2	ZNF473	UBXN2A	CKS1B
	NBPF10	NR1D1	CEACAM19	LNPEP	PTPN13	LOC283267	PSMD8	SESN1	KCNK1	ECE2
	HMBOX1	ADCYAP1R1	SHANK2	EPB42	CLIC2	SH3YL1	ANXA2P1	PUS7L	MCART2	TBC1D8B
	TLE6	DIRC2	RNF128	ASPHD2	AAK1	PLA2G5	RNF168	C3orf38	TH	EBF1
	C12orf23	TMEM8A	DPP3	ALG1	SERINC5	IL1RAP	SNORD94	GBF1	HSPH1	GABRE
	NADK	TMTC1	DKC1	NKX3-2	CHST2	B4GALT6	WNK3	RC3H2	PDIA5	GNPTAB
	SSFA2	TMEM48	SFTA3	ZFX	SIK1	SLC5A7	MMP13	GRPEL1	TMEM68	RAB18
	NT5C2	MAD2L1	FASN	HUWE1	PEG10	GPKOW	ROCK1	GPNMB	TFE3	KIAA0100
	C1orf226	NOTCH2	CXorf59	LRP3	WDR1	PPP1CB	FOXO4	PI4K2B	B4GALT3	C2orf18
	GHITM	ISG20L2	HIST3H2A	LAMB3	GMEB1	USP14	RCVRN	SEMA4F	KIAA1524	NPTN
	PDIA3P	YTHDF3	C3orf26	C17orf28	ZNF689	NOTCH2NL	GOLGA4	LIN54	ME2	TTBK2
	CUTC	APOOL	ABHD2	FAM98B	C17orf67	SYT14	FAM49B	MMGT1	SON	HYOU1

	F11R	NSMAF	CPT1A	TIMM8A	DENND2C	RBCK1	RAX	ANO7	PHF3	NFIC
	FAM84A	C15orf50	PAPSS2	PRKAR2A	RAB5C	MAP3K2	GNG12	SASH1	EBF2	RIF1
	COPG	CLPX	ATP2A2	TMEM59	GNAO1	HSPA8	ARPP19	GAPVD1	DNAJC5B	WDR3
	LRRC31	FUCA1	ENOPH1	ITGA5	ENY2	DCLK3	ZHX1	KITLG	LXN	HIST2H2AA3
	VPS13C	C19orf38	ALKBH8	HOXB9	SLC30A3	SEC16A	RTF1	LLPH	HSPA1B	PIK3CA
	MTFR1	DYNLT3	ATP2C1	PGBD5	RHPN2	DNAJB1	ANXA2	IDS	SLC29A2	THRB
	MAPK3	NCOA2	HIST2H3D	ATG16L1	PSG5	ADAR	PKIA	TMED2	PSMD14	DOCK6
	FICD	WDR44	SNAI2	KIAA0513	ABCC5	ARMCX3	GUCY1A2	ZNF430	ATG9A	RNF111
	C8orf76	ZNF705A	CELF1	TFDP2	PTPRM	MKNK2	IPP	PXDNL	DMRTA1	KIAA1211
	HIST1H2AG	PDE10A	GFI1B	FAM69A	CHMP5	ELK3	CMIP	LGMN	NBEA	SEC62
	HIST2H4A	MAB21L2	RPRML	DNAJC6	ABL2	BRWD3	MFHAS1	TMLHE	LIPG	RRM2
	C3orf64	KDM3A	CDH5	LARP4B	PCNP	C2CD4C	MAP6D1	BPNT1	KCNC4	PSMA7
	FKBP14	A2M	SP100	SP2	MAMSTR	REEP2	CLSPN	CTR9	GUCY2F	OR1Q1
	RXFP2	SGMS1	SH3RF1	SRPX2	UBE2D3	CMTM4	IMPAD1	PRAMEF1	WDR45L	LYN
	EGLN3	ABCC1	SNX12	CDS2	NOMO1	GPR172A	AKIRIN1	TPD52	IQCB1	PITPNM1
	SLC9A7	STC2	NPAS1	PLVAP	CYTSB	NOV	LOC151162	PPP3CC	ZFP106	CTDSPL2
	KDELC2	ZC3H7A	MCM4	ZNFX1	SLC39A7	ANO2	FZD7	GPR107	CCPG1	KRT16
	TMEM164	UNC13A	GRINA	HNRNPH2	HBG1	FAM83B	ZCRB1	DEDD2	ATP1B2	RCN1
	C15orf58	BOD1L	HELB	REPS2	CEP68	NPEPPS	TMEM110	SLC35E1	TBC1D20	KCTD9
	EPR1	PCMT1	EIF4E	MANSC1	GCET2	MCM2	XKR4	NOTCH4	ELF4	MAP1LC3B2
	TRIM5	EVI5	C21orf67	WNK1	SIAH2	RFK	PHLDA1	ATF3	POLR3K	ADAMTS5
	CPA4	ZIC5	CXorf36	ARMC7	AFF2	FAM57A	PSMA1	CRIPT	ARFGEF2	TM9SF4
	LOC402377	GRIA1	GALNTL2	UBR1	LRPAP1	JKAMP	CSDAP1	FAM71A	GLUD2	PRKDC
	F2RL1	GTF2E1	SCN3B	C4orf12	ASAH1	KDM5C	SEC22B	ATP11A	FRK	CLPTM1
	KCMF1	GPBP1	POLE4	TMEM87B	SLC20A2	RAET1G	AMBN	SNORD116-4	HIPK3	SKIL
	PWP2	PPP1R14C	REST	CHMP7	ARPC2	CYP2U1	KLF14	BRCC3	UJA38777	CCDC75
	OR2B6	KBTBD4	PPP1R2P3	AOC2	VANGL1	CDC42BPA	HDGFRP3	C12orf36	LAMA2	LOC285830
	LRRTM2	CLRN1	PHOX2A	NHSL1	LSM 10.00	GABPB2	STK40	HNRNPK	SH3GLB1	STXBP5

	NFKBIL2	C10orf47	MRE11A	HSPA4	HTA	TMEM79	ANKRD46	CCDC7	FKBP1AP1	ZFP91
	PHTF1	RELA	TCTN3	ING2	TMEM39A	KIF4A	RND3	INVS	SLC36A1	SLC35A3
	MDGA2	DCAF8L2	UHMK1	NEFL	DUSP7	LILRB5	SLC18A2	ATP8B1	DCAF12	STAU1
	OIP5	PRDX6	CLCC1	MAPRE1	POU3F3	TOP 1.00	PDIA6	SSTR5	C1orf55	COL8A1
	HAUS2	CDYL	SERTAD1	COL18A1	ATXN8OS	OR10A3	OR1G1	TRIM40	AK2	C14orf147
	MSI2	STX12	PPAP2B	GPR180	NCALD	ARNTL	GCNT1	ADAM15	CD209	RNF39
	CBFA2T3	UBR3	CNTNAP2	CNIH	NUDT4	PPME1	RDX	CD46	ZNF664	ZFAND6
	WDR26	KIAA1199	MAD2L2	LOC84989	C1orf150	TAS1R2	BHLHE40	VCPIP1	CXorf64	RLBP1
	SNORA2A	HMG20A	C12orf66	DUSP4	CORO1C	DSCR3	BCL2L11	KIAA0494	PPP2R2B	SETD7
	BLVRB	TULP1	HOXC13	FBXO2	HELLS	KIAA1522	FBXO8	NEO1	PRDM5	STAMBPL1
	LPCAT2	DMPK	OLFM3	ADAM11	ZNF259	HIST1H2AC	PBK	TTC39B	CD177	BAZ2A
	EXOSC2	C8orf39	EPT1	RNF152	ARHGAP17	ENTPD4	NOP10	SULT1A1	HADHB	C4orf41
	HMCN1	PEA15	CXorf57	REG4	HIAT1	TIMP2	HSPA13	UBE2R2	C12orf49	DLL4
	NUSAP1	USP39	RTN4RL1	CBX1	TLX1	TBL1X	SMARCD2	ZNF37A	MYH16	TMEM108
	PRPS2	CASC5	ANKRD36BP1	COL5A3	GNAT2	ZNF542	ARHGAP11A	SH3BP2	AVL9	RBMS1
	KCTD21	KCNIP1	ATRN	CCR10	LONP2	HIST2H2AB	DHFR	KDELR1	KBTBD5	PCSK6
	TNFSF9	TGM5	AHSA1	NRCAM	GPRC5C	GANAB	AGPAT9	GOLT1B	PTPN9	LRP11
	MPP1	MGAT4A	EDA	ITGA8	CAPN6	STS	GSDMA	PIGN	PRKCI	AP3D1
	PRICKLE3	GOLIM4	CD34	MAP3K5	RBM8A	STX5	KCNK3	PPP3CA	GADD45A	LIMA1
	GSTM3	NUP155	OLIG2	ACBD5	CCDC80	EPHX1	RHOD	C11orf17	PNRC2	THSD4
	C3orf37	PIP4K2A	KLHL20	SKA2	RHBDD1	OR13J1	TSEN34	RFX3	SIGLEC6	RASSF9
	IER5	TTC35	CNGA1	KIAA0754	DGAT2	NR6A1	RNASE7	CYFIP1	PIK3C2B	GSN
	LOC100144604	PDE1C	PQLC3	RSPO3	DTX4	FAM57B	SNORA74A	PRKAG2	ATRNL1	FBLN2
	DBI	SPESP1	IFIH1	LIPA	LEO1	DACH1	LRP1	PRO0611	TEX15	CEP97
	SNX2	AMBRA1	MRPS14	TAF5L	CCDC43	SLC12A2	TPSD1	C15orf44	PREPL	TSPAN17
	RHAG	WBP2	DUSP14	RAB7L1	TAB3	CSNK1A1	PSG7	HIST1H3H	CDR2L	OVCH2
	WIPF2	STARD13	ATP11C	GALNT12	GPR110	TWSG1	SGK196	ADAM7	RAB32	FAM83G
	KIAA1715	FSD1	NUDT21	C19orf40	EMR2	RNASE10	EARS2	MAL2	PAPOLG	C9orf150

	TIE1	ADAM9	HHIPL2	CMTM1	TMEM2	AQP8	XRRA1	GDPD5	SLCO5A1	RGS7BP
	SSR3	SCUBE3	C18orf26	MRFAP1	TOR1AIP2	RTN2	KIAA1161	STEAP3	DSEL	ATG2A
	RFX7	SPTLC2	AACS	HOXB2	B3GNT5	ETHE1	CCDC93	PLXNA3	COX7A2L	FAM25A
	TMEM65	LOC146336	BACH1	LASP1	CHAF1B	FCN3	ATP9A	FRG2B	ZNF576	SLURP1
	GGCX	PID1	LAMC1	CCNY	MLL	LAMC2	HMGCR	SRP68	UGDH	DKFZP434K028
	ZNF845	RCN2	PLXNA1	ALAS1	FYTTD1	UHRF1BP1L	SEC14L1	PLEKHM1	MPP6	TKT
	PJA1	NOVA2	URB2	SPOCK1	GABRD	FAM164A	SLC6A6	LAMP1	TAB2	INADL
	ARHGEF5	ANTXR1	C1orf21	ADAM10	NR0B2	MYO5C	TMEM62	C9orf91	SHROOM2	SRMS
	EPHB1	NF1	PHF8	ATP4A	LEPR	PARP8	GPR137B	GRK5	C9orf102	ABCG2
	SMC2	WASF3	ZBTB2	IDH2	VCP	TMEM132A	NHLRC2	FOXO1	EGLN1	ZBTB7C
	ACSL1	HYAL4	FAM72A	HMGA1	TEX12	C8orf55	MLLT3	SEC31A	GRIK3	GOLPH3L
	LARP6	CDC37L1	S1PR1	IMMT	FCF1	TANC1	HNRNPC	ADAMTS4	NCAPD2	GNB4
	COQ2	FLJ43860	IFNAR1	BMI1	PLCD4	RASGRF2	SPNS2	MOV10	HBD	RIPK2
	IRGC	TNKS	RAI1	CYP51A1	B4GALNT4	CSNK1G1	KIF11	MAN1A1	ADK	TCEB3
	DOLK	KCNS2	CEACAM1	SPINT2	DAAM2	RNF20	TESK2	MYCBP2	MAP7D1	CBR3
	CXXC5	TMEM127	LOC399815	SLC9A11	PSAT1	YIPF5	MYH15	MAP2	ANGPT2	CXCR7
	ISCA1	PTGER2	ZNF687	CPSF4L	BSDC1	UGT1A5	CD274	FUNDC2P2	DHRS7	UBXN7
	BTBD3	SERBP1	RNF7	WBP11	USP36	SNX3	NCRNA00087	HADHA	PLBD2	C9orf21
	FHL5	NRAS	MGST1	H3F3C	TTC9	SEC63	CNOT1	BZW2	FOXR1	TTYH2
	RCE1	CTDSPL	HEATR5B	WASF2	GPR179	TMEM72	CACNA2D1	CES7	SPAST	SDC2
	MYST2	SFT2D2	RNF144B	NEUROG3	STX6	LYSMD2	FUT5	ATP6V0E1	GRM1	RACGAP1
	KCTD20	FUT10	SIGLEC1	CHSY3	LCE1E	HNRNPU	CD55	BST2	UBE2Z	MANF
	KLHL15	CDC42	CCDC136	RNGTT	RNF187	UTP15	CHCHD6	FAM46A	GPR160	DIO3
	SERPINB8	MED30	SEMA4C	OXSR1	TP53I11	LTBP3	F2R	STT3A	GRIN2C	MINA
	LOC100126784	TP53BP2	MSH2	PPT1	POLR2B	FURIN	UBE2I	UGGT1	TTF2	GNG4
	TNFAIP1	FAM53C	CTSD	SESN2	MED21	C1QL1	CACYBP	PTGER3	ERCC4	DYSF
	RAD54L2	SNORA34	C12orf52	TSPAN9	PRDX1	POC1A	NBLA00301	MPZL2	STK32C	EGFR
	PRR15	BUB1B	SEC14L4	FBXO22OS	TOB1	AXIN1	SMAD5OS	ENDOD1	SIGLECP3	ACBD3

	SRPRB	PCNX	RAB14	SLC10A3	ALAS2	POLQ	KIAA0922	DEK	ELTD1	PIAS1
	GPATCH8	MUCL1	PLD5	SLC28A3	KCNH1	STK3	RRAGA	SOD3	TRIM63	LRRC52
	PIGB	SIRPB1	ANXA9	ST6GALNAC4	SMYD3	FBXW2	FAM190B	BAT2L1	KLF7	RAB9B
	BMP6	NDE1	MAN1B1	FBXO42	SPTLC1	FAM155A	ZNF174	GSG2	TMCO7	WDR47
	KLHL5	RPS27L	ZNF778	UBA1	KCNC1	KIF5C	ANKRD31	APBB2	SRP54	FAM63B
	HS2ST1	DLG5	ALDH9A1	PCNT	SLC45A4	COL15A1	C20orf177	LGI4	INMT	CCDC21
	PAPOLA	IFITM1	SLC17A3	DHRS3	STAU2	KLC1	KCNN3	DPP8	SEL1L	CPB2
	ARTN	LRCH3	SESN3	FBXO48	MFSD11	GABRQ	YWHAB	SEC24A	GPR88	TRAF6
	SPRY4	XRN1	FOXA1	AARS	RG9MTD2	TGFBR2	GLCE	C9orf3	TMEM192	SNX7
	SLC16A3	PLDN	ACAP2	MEIS1	ZNF828	LYVE1	KCTD3	INCENP	ZNF670	SLC7A14
	ARID5B	LAMA5	MTOR	GPRIN1	LMO4	FAM104A	KCNC3	DHDDS	RRAS2	KIAA0319L
	PLXDC2	CSPG4	ABO	PEX13	KIF20B	DRAM2	PHKB	TMTC2	CHST3	SNORD116-20
	CNST	RERE	PTPRU	OAZ2	PCDH12	CDK1	LOC653566	BAHD1	LMNA	TMCC1
	CDH13	SGOL1	LRRC10	PPPDE1	VN1R5	LRBA	TOR1B	METTL14	ZNF652	LYST
	GRHPR	E2F7	NCBP1	HOXD4	NOMO3	UNC45A	BMPR2	DERA	BLNK	CREG2
	RAB11FIP5	KCNH4	BRD2	ATP10D	DNAJB4	SCRIB	TRIM2	USP48	MC4R	SEMA4G
	SLK	ATP6V1H	PDXK	NDFIP1	TAOK1	OPALIN	FBXO28	RAB5A	LONP1	SMS
	C1orf190	BCKDK	LOC100133612	FANCD2	MOSPD2	GSK3B	ASAH2B	GKN1	ANKRD17	TFDP1
	PTPN4	HIST1H2BN	NUDT11	PLK1S1	PDCL	MAP1B	LYPLA1	INSIG1	SCRG1	DGKE
	MYRIP	KRT15	TNFRSF10B	HIATL1	SATL1	COL14A1	USP24	SLC26A10	ENDOU	COL4A5
	METTL13	FAM157A	SNAPC3	PLAC2	LEPREL2	RFFL	CAPN14	LUZP6	IQCF5	CENPI
	GTF3C1	PDGFC	ARAP2	ETV5	MAPK14	GCC1	SMC1A	SDF4	RSPRY1	HK2
	SH3KBP1	SBSN	ZNF667	NODAL	CNN3	C7orf65	MTUS1	IFI16	PPAP2A	APOLD1
	PARP9	LINGO2	LAMB2	KPNA4	INPP1	THBS1	LOC221710	AP4E1	LCORL	BANK1
	HNRNPA3P1	ZNF367	CTDSP2	C20orf3	UTP23	GRID2	TAAR9	RRN3	ESYT1	STEAP4
	COL6A6	SIRPD	PTPRF	MUC2	TMX1	SYNJ2	SNRPN	CYB5RL	PRKRIR	FAM155B
	NUMBL	LARGE	CDK2	KIAA0146	CLOCK	NAF1	NEK1	LOR	B3GNT2	AZI2
	GALNT7	USP37	PSMD3	EPN3	EPHB2	C12orf35	VPS33A	DAG1	ZNF462	PIK3C3

	NANS	RNF26	BCAS2	LOC100170939	RAPGEF6	SMARCA2	LOC148189	LOC400696	RPA2	PIP4K2C
	KPNA2	PTPRG	HOMER2	PKMYT1	TAS2R50	BCL6B	POLR2D	IL2RA	PRKD3	NUP62CL
	SAMD9	TBC1D2B	HOXB5	DCDC1	SAMD4A	HIPK2	LOC344595	BRPF3	ACADSB	ZNF79
	ZDHHC2	HS3ST4	PRDM10	ARSK	PHACTR4	MMRN2	RNF133	DNAJC25	SP140L	C10orf128
	NPAT	KIF13A	LAMP2	GPATCH3	UQCRC2	GRSF1	MS4A2	LQE39692	LRRC17	MIB1
	VPS13B	ELOVL1	XDH	MTMR9	LOC723972	MYNN	FRMD8	GPR4	SSH1	TMEM64
	EXOC5	RXRA	GLS2	CYP4Z1	DDA1	HK1	ABCA2	TNPO3	SUCLG2	ZNF598
	ASXL2	TMPPE	HERC2	TMEM189	LOC348840	FAM49A	DCAF13	IL1R2	CALU	OSBPL3
	MERTK	C8orf37	SMG1	ADARB1	EN2	RAD54B	LYG2	TNFRSF10D	SHISA5	BTBD1
	POC1B	CAST	P4HB	RGS17	UGT1A3	OTUD4	KCNT1	ASB9	PIAS2	DHX8
	NBPF3	MED13	CTTNBP2NL	EFTUD1	LOC550643	MTAP	PCDH11X	SLC25A46	FLJ23867	CALR3
	VRK2	LOC201651	PRCP	SLC26A11	WDR31	MAGEE1	PPP2CB	PDCD4	ATXN7L3B	DNMT3B
	LMBR1	G3BP1	GALNT2	UBE2D2	NAP1L2	ELK4	FRAS1	SNIP1	RHOJ	PALM2
	IGSF9B	KCNJ6	EGFL7	PDLIM2	CCDC47	HLTF	CEP55	FLJ43390	RAPGEF1	EFNA5
	SMEK2	MAP4	GBA	GALNTL6	KIAA1012	LCE1B	CALB2	KIF23	LOC90246	BAX
	NBEAL1	NQO1	CLN3	KLHL33	LCOR	TRIM62	C20orf94	SPTAN1	SLC25A40	AGTPBP1
	GJA4	LRP8	SLC3A2	PIK3R4	OTUD7B	RAB3D	NOS3	LIG3	CDK8	TTLL7
	PIPSL	TNK2	LOC100128788	HSPA9	DNAJC16	LOC285419	SLC39A11	ANKRD34C	LOC728640	ZNF699
	GPC1	VEZF1	VDR	RAPGEF4	MYH10	PLP2	GNPDA2	TPX2	TMEM111	UVRAG
	AGPAT3	DNAJC22	SEC61A1	PRDM4	VPS4B	LOC441089	OGFOD1	SLIT3	OCRL	SYTL3
	PPL	NAT8L	KCNA2	SORT1	PCDHGA9	SYNM	NME1-NME2	DSE	C16orf87	SOCS7
	DCP1A	LRRTM3	FAM102B	SRXN1	MFN2	CAND1	BRE	WDHD1	MMRN1	GPBP1L1
	BARD1	TMEM38B	ENTPD7	FAM129B	WAPAL	ULBP3	STOX1	SMC4	C12orf34	TIRAP
	UBE2E1	TRIO	SPATA5L1	SCAPER	TREML3	TFRC	SLC17A5	POLD3	PRKD2	PDE4DIP
	HIGD1A	FAM64A	PRSS8	SNAPC5	ZNF434	TRAPPC10	CBL	SQSTM1	MGC87042	KDSR
	SPEN	LPCAT4	ABCA3	CRK	ADCY5	GSTA1	PSORS1C3	IGF2BP3	TMOD2	CMTM6
	RNF169	AOC3	PROCR	EPS15L1	PCDHGA7	KIAA1704	LIMD1	EXPH5	SCIN	TTLL4
	EXOSC6	PRPS1L1	AHRR	CCNB2	ACCN4	GLP2R	CADM2	C19orf21	SYCE2	FILIP1

	DSCAM	PLK1	CUL5	TRAF3	PPAPDC1A	PBX3	C16orf75	PIGV	SHC3	KIAA1644
	MGC12982	FAM82B	DAB1	SLC30A5	HIST4H4	PLSCR4	NACC2	ELOVL6	DLC1	CTSO
	NCRNA00161	ADH5	SH3BP4	ISY1	TMEM97	FOXN2	FAM60A	C9orf172	SPAG9	CLSTN1
	ZNF518B	MGAT2	LOC285033	SAR1A	PIAS4	SPINK7	ERAP2	KDM5B	GDF1	ZNF619
	KAAG1	PRRT4	LOC388428	IL4R	RAD51	SLC29A3	KPRP	PLXNB3	TCF12	UTP3
	CRTC3	CD3EAP	PDZD7	RGPD5	PIGK	MAMDC2	FAM83D	NTNG2	RPS6KA2	CCNE2
	KDM1A	CNOT8	PSIP1	SERPINH1	OR13C5	LAMC3	HEXA	LOC400759	NTN4	ATL3
	VAPA	SMCR8	GULP1	PANK3	C15orf38	SOCS5	SEC24B	STX1A	PIAS3	LTBP1
	MAN2A2	GRB2	IFITM3	AKR1C3	TSSC1	GGA2	PIGA	ACTN4	THBS4	VPS13D
	POSTN	ENOX2	PCBP1	TRIM68	ZNF436	SERINC1	MMP3	PHAX	FBXO30	ZNF749
	SNW1	HBG2	MDH1	GLDC	MAGT1	CDK6	SPTBN2	ZNF791	VAV2	OSBPL8
	ALG3	FOXK2	KRT4	FER	KIAA1919	EEPD1	DLEU2	PCDHA4	C17orf46	C15orf39
	CHSY1	ARRB1	MYT1	HIST1H4H	PRCC	ZNF646	IGF2BP2	SET	LOC643837	MCART1
	ITCH	CLEC14A	NUDT16P1	ETS1	FAM18B	IREB2	SLC25A33	B4GALT4	MCC	SVIL
	RFC1	CHPT1	DHRSX	LCLAT1	PHC3	BEND2	OGFRL1	MFGE8	GOSR1	ELMOD1
	THEG	SFRS15	CFHR5	FCHSD2	HSDL2	FAM73A	C12orf62	KIF18B	KLHL3	RPN2
	ERLEC1	SYK	PSG4	CA11	RPL13P5	WDR76	SLC38A10	UNC5B	MLPH	ZNF420
	KIAA0101	NDST2	MAOA	ATXN3L	BARHL2	C11orf36	C12orf12	C14orf70	C1orf68	C21orf131
	C5orf52	CSRP3	CYMP	DEFB129	FAM181A	FKSG73	FOXB1	GABRA4	GNRH2	GOLGA6C
	GPR12	HSFYL1	HTR5A	IFNA7	IFNK	IL25	KCNK18	KLKP1	KRTAP4-4	KRTAP4-7
	LOC285194	LOC286135	LOC29034	LOC340094	ODF4	OPRD1	OR10G4	OR10H1	OR11H4	OR1A2
	OR1D4	OR1N2	OR4A15	OR4C3	OR4K17	OR51A4	OR51I2	OR51S1	OR52R1	OR56A4
	OR5AR1	OR5K4	OR5L1	OR6C68	OR6N2	OR7A5	OR7G1	OR8D2	OR8D4	OR8H1
	OR8J3	OR9I1	OR9K2	PRAMEF13	PRMT8	PROL1	PTPN20A	SERPINB10	SMEK3P	SNORA41
	SNORA54	TAS2R16	TAS2R9	TMEM89	TMPRSS11BNL	USP26	USP46	C3orf70	C8orf33	NRBP1
	LOC150527	PRUNE	ASB1	HSP90B3P	C10orf26	CCNO	TAF1B	KALRN	FMR1	LRP10
	IFIT2	DENND5B	GCN1L1	DOPEY2	CDK12	CHST11	CASK	PRRC1	TMEM37	PLA2G2C
	UBE2T	SOS 1	EIF1AD	PLD1	CYBASC3	REEP5	XKR6	ZNF785	JAZF1	CDKL5

	NLGN1	HIPK1	ZFR	AURKA	NUP205	CPA3	SPCS1	RLIM	CNIH4	AP2A1
	DPF3	NEIL2	PLEKHG1	FOLR2	SH3BP5	PAK7	DEFB126	IFLTD1	MAPKAP1	PLP1
	CACNB4	MIA2	CDCA8	ZWINT	LAP3	DNM3	ADAM18	KLF5	DLGAP5	GPX5
	SLC19A2	SMAD2	LHX3	CLDN16	CASP3	SNAPC2	BCL9L	GNG8	C7orf70	CENPA
	MFSD2B	SCNN1A	IGSF3	NUDT16L1	NBPF1	C14orf48	SEC23B	KLF10	C9orf69	ARID1A
	BCL6	FBXL4	USP34	PAQR4	CPSF2	WARS	KLHL2	ANKRD34A	CHRNA1	COL4A1
	SOX2	ELAVL3	PCDH7	PMP2	CCT8	CCNK	TK1	CDC42SE1	ATP1A1	CCDC146
	MAP3K14	MBOAT1	VKORC1L1	GEMIN4	MAT1A	AIFM2	KCNV1	DTL	RUSC1	ANXA7
	DNTTIP2	NUP62	ZNF134	HDAC3	TRAPPC9	DNAH3	C2orf86	MITF	MRPL37	KIF13B
	GMPR	PSMB5	ATL2	ELN	KLHDC8A	CADPS	ZCCHC17	TSKU	ATP6AP1	IQGAP3
	ADSS	PPP1R3D	ILF2	BTG2	EFHB	PLG	INTS8	KLHL21	WNK4	RPS6KA3
	NRG3	TBC1D14	ALPP	SLC30A9	HEXIM1	SLC1A2	IKZF5	EXOC3L2	CNTN5	GAN
	MPHOSPH9	SMG5	TMEM84	SLC5A12	RBL1	EMX2OS	MGC15885	CLASP1	SLC35A5	POLR2A
	SPTLC3	NCEH1	GPN2	RABL3	LOC90586	ENPP2	C8orf83	KIF2C	C9orf79	KIF1B
	ZNF543	ZNF704	UBASH3B	C16orf63	MFSD1	ERCC6L	ZNF496	APOH	CYB5R1	IGSF21
	FOXD2	EPC1	MRPL15	ANGPT1	NCRNA00152	VPS35	RAB35	CLPB	MND1	MAP4K5
	RAPH1	GRIA2	TNXB	AMPH	PCNA	VPS41	FLNB	DPYD	CTNND2	FAF1
	RECK	KLF6	ACLY	SYVN1	RAB6B	ADAT1	DOCK9	ACAT2	NOTCH3	HDAC2
	PAQR3	TSNAX-DISC1	SLC38A7	DLL1	SH3TC2	C1orf177	DDX47	FAT4	ZNF766	PIGF
	KCNQ1	CCNE1	GAA	NPBWR1	LPIN2	PRKG1	CCDC155	SAMD12	OLS37681	NRG2
	FAM72D	SYNGR2	SPATA6	FZD10	PPA1	REM1	FKBP15	SYTL4	SHPRH	FMR1NB
	YBX1									

Figure S6: Differentially expressed genes in stage 1 of KIPAN,[KIPAN=Pan-kidney cohort (KICH+KIRC+KIRP)]

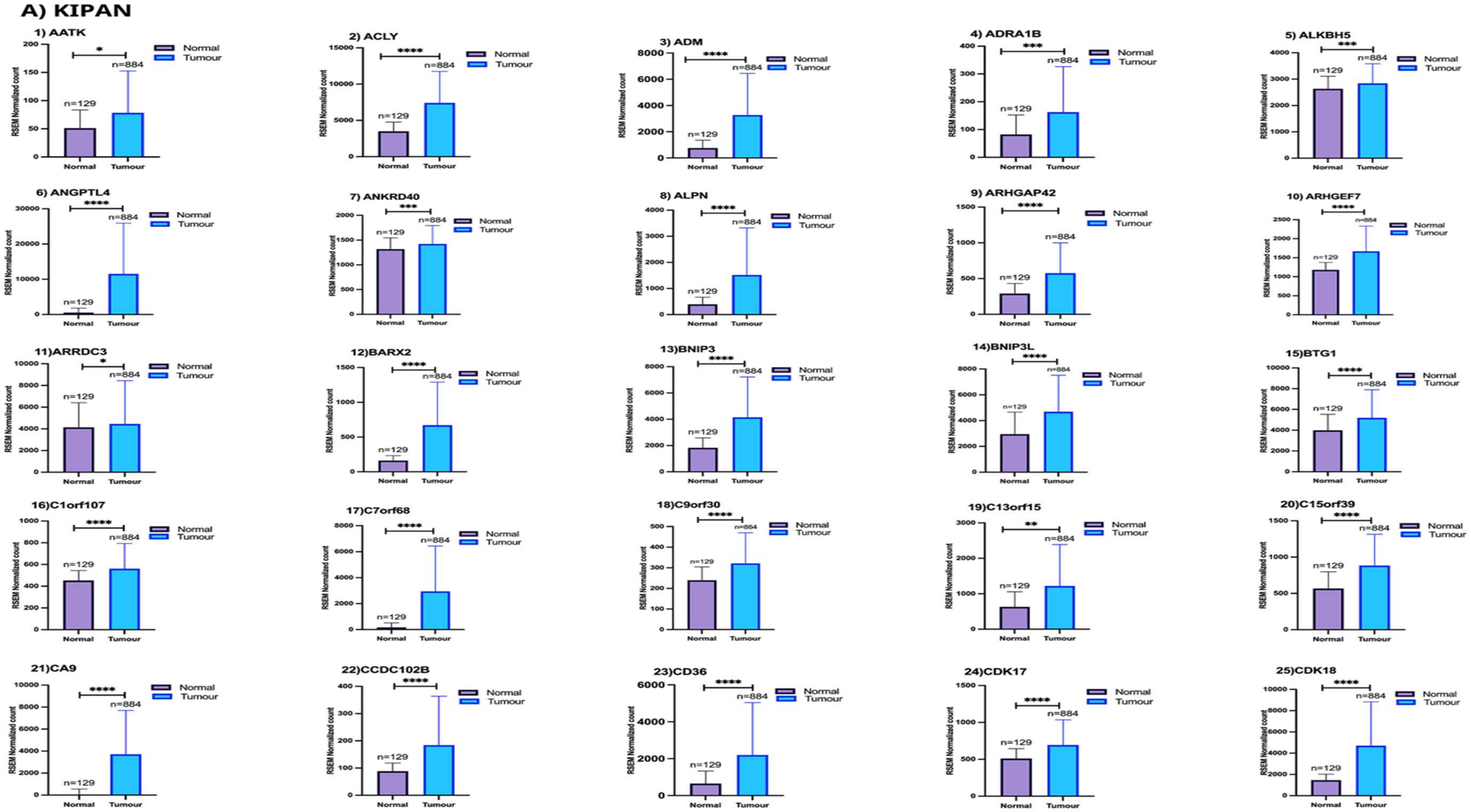

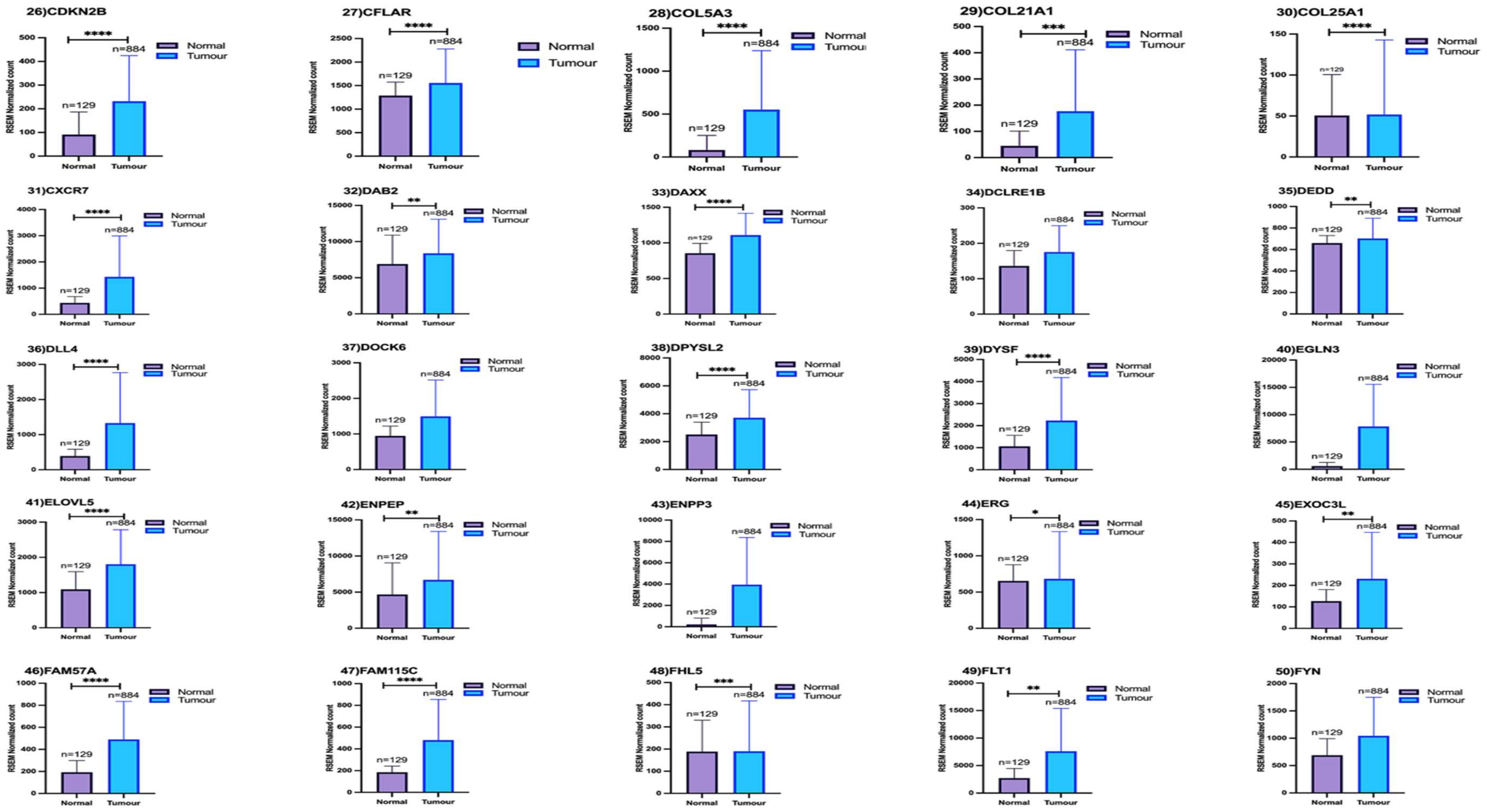
26)CDKN2B
27)CFLAR
28)COL5A3
29)COL21A1
30)COL25A1
31)CXCR7
32)DAB2
33)DAXX
34)DCLRE1B
35)DEDD
36)DLL4
37)DOCK6
38)DPYSL2
39)DYSF
40)EGLN3
41)ELOVL5
42)ENPEP
43)ENPP3
44)ERG
45)EXOC3L
46)FAM57A
47)FAM115C
48)FHL5
49)FLT1
50)FYN
Normal
Tumour
n=129
n=884
RSEM Normalized count

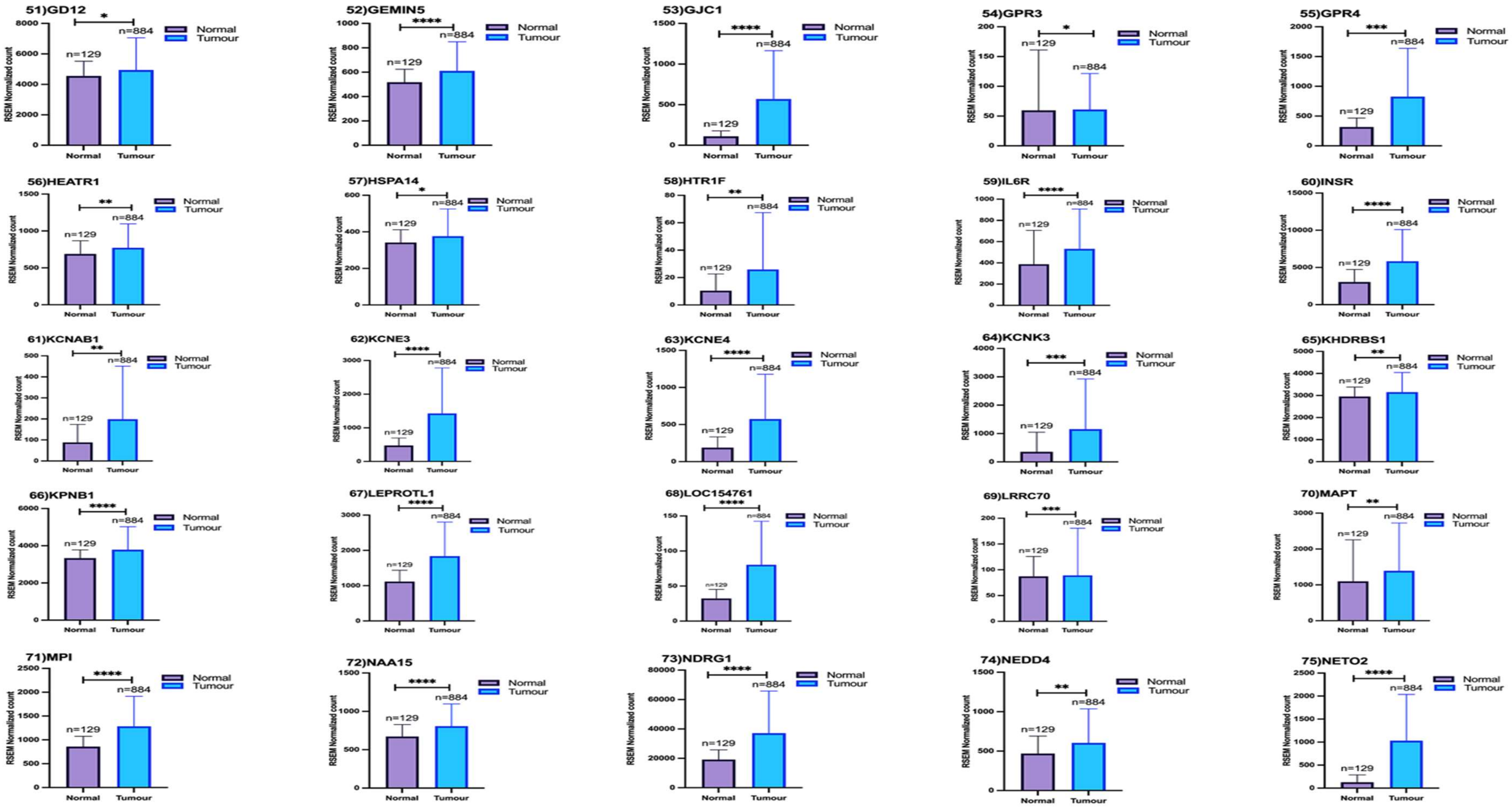
51)GD12
52)GEMIN5
53)GJC1
54)GPR3
55)GPR4
56)HEATR1
57)HSPA14
58)HTR1F
59)IL6R
60)INSR
61)KCNAB1
62)KCNE3
63)KCNE4
64)KCNK3
65)KHDRBS1
66)KPNB1
67)LEPROTL1
68)LOC154761
69)LRRC70
70)MAPT
71)MPI
72)NAA15
73)NDRG1
74)NEDD4
75)NETO2
Normal
Tumour
RSEM Normalized count
n=129
n=884

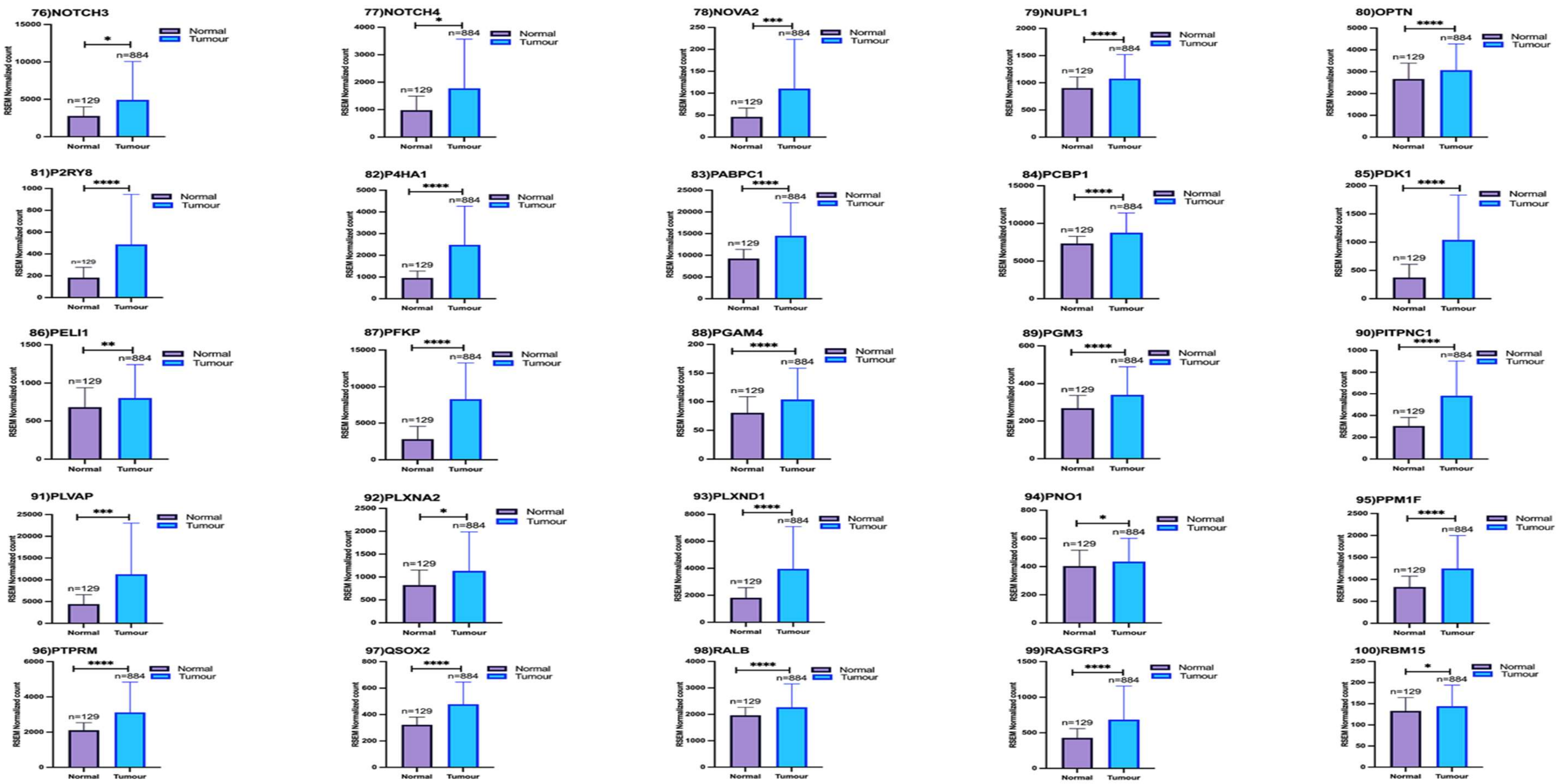
76)NOTCH3
*
n=129
n=884
Normal
Tumour
RSEM Normalized count
77)NOTCH4
*
n=129
n=884
78)NOVA2
***
n=129
n=884
79)NUPL1
****
n=129
n=884
80)OPTN
****
n=129
n=884
81)P2RY8
****
n=129
82)P4HA1
****
n=129
n=884
83)PABPC1
****
n=129
n=884
84)PCBP1
****
n=129
n=884
85)PDK1
****
n=129
86)PELI1
**
n=129
n=884
87)PFKP
****
n=129
n=884
88)PGAM4
****
n=129
n=884
89)PGM3
****
n=129
n=884
90)PITPNC1
****
n=129
n=884
91)PLVAP
***
n=129
92)PLXNA2
*
n=129
n=884
93)PLXND1
****
n=129
n=884
94)PNO1
*
n=129
n=884
95)PPM1F
****
n=129
n=884
96)PTPRM
****
n=129
n=884
97)QSOX2
****
n=129
n=884
98)RALB
****
n=129
n=884
99)RASGRP3
****
n=129
n=884
100)RBM15
*
n=129
n=884

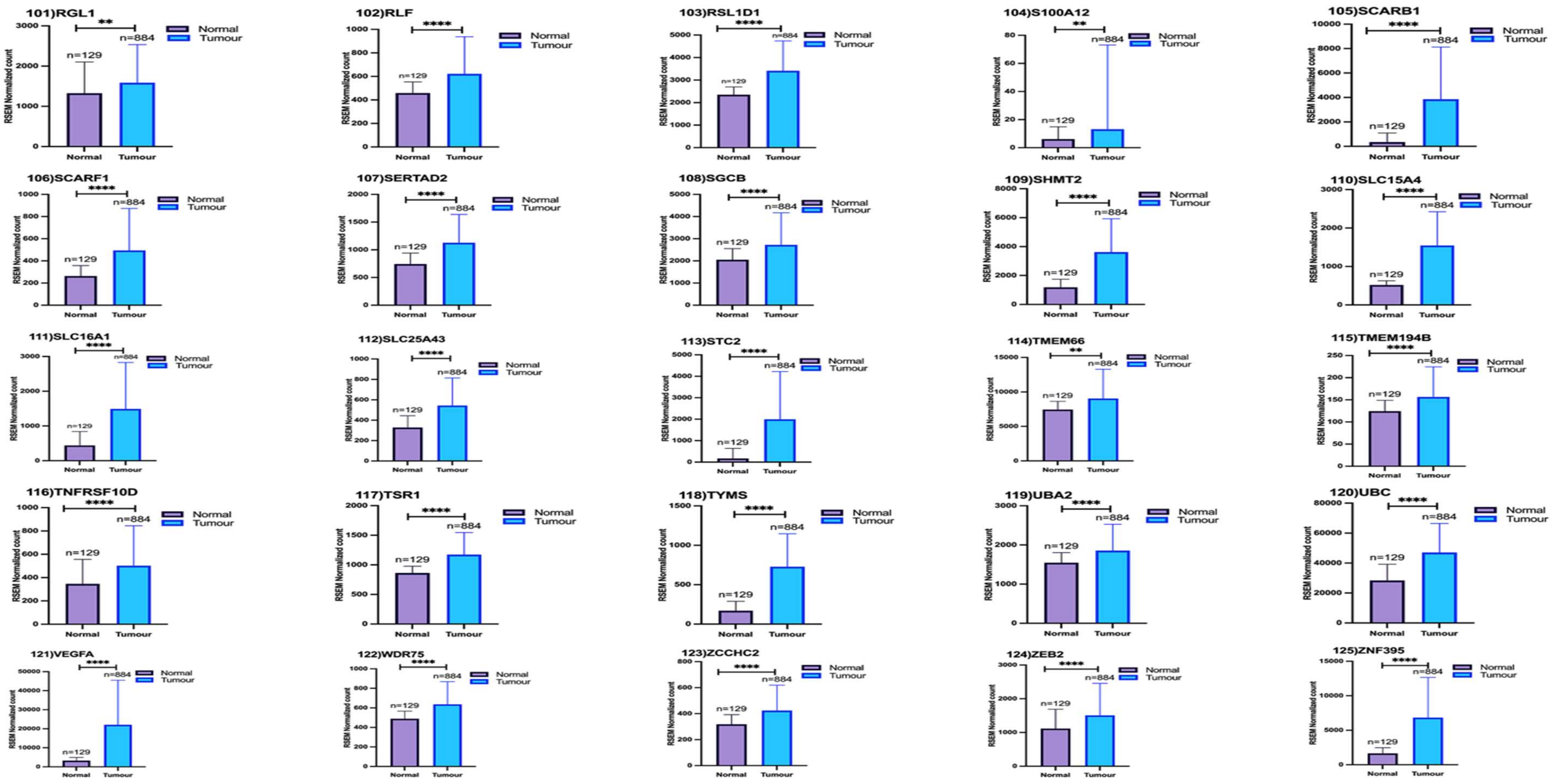
101)RGL1
102)RLF
103)RSL1D1
104)S100A12
105)SCARB1
106)SCARF1
107)SERTAD2
108)SGCB
109)SHMT2
110)SLC15A4
111)SLC16A1
112)SLC25A43
113)STC2
114)TMEM66
115)TMEM194B
116)TNFRSF10D
117)TSR1
118)TYMS
119)UBA2
120)UBC
121)VEGFA
122)WDR75
123)ZCCHC2
124)ZEB2
125)ZNF395
RSEM Normalized count
Normal
Tumour
n=129
n=884

Figure S7: Differentially expressed genes in stage 1 of KIRC, [KIRC=Kidney renal clear cell carcinoma]

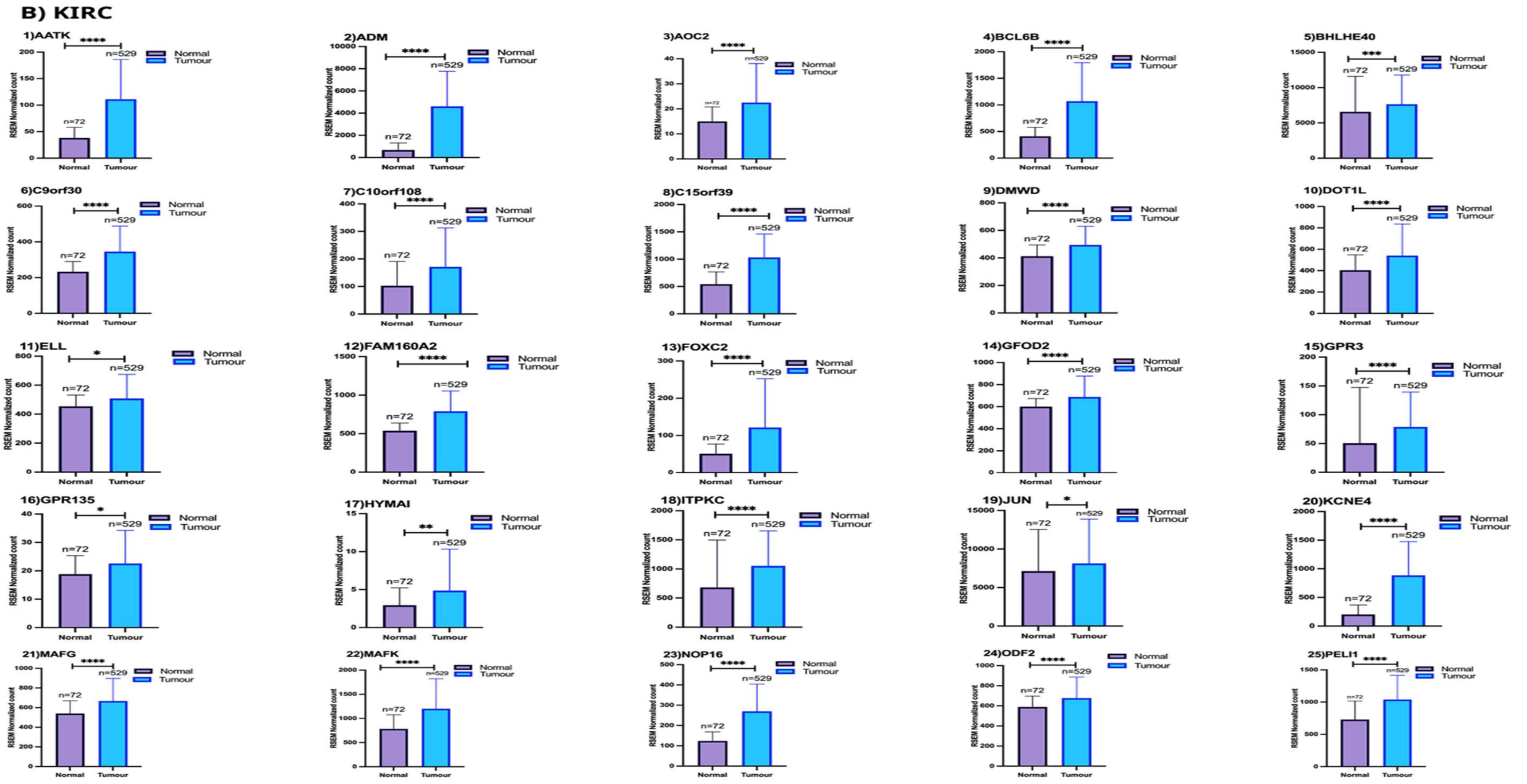

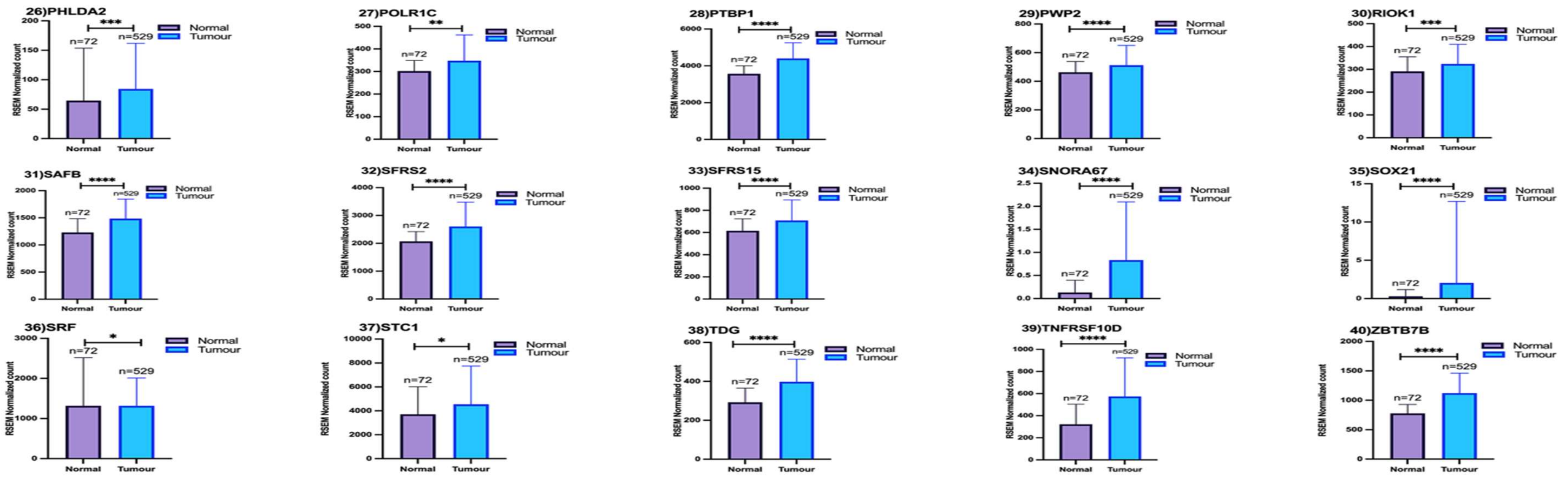

Figure S8: Differentially expressed genes in stage 1 of KICH, [KICH= Kidney chromophobe]

C) KICH

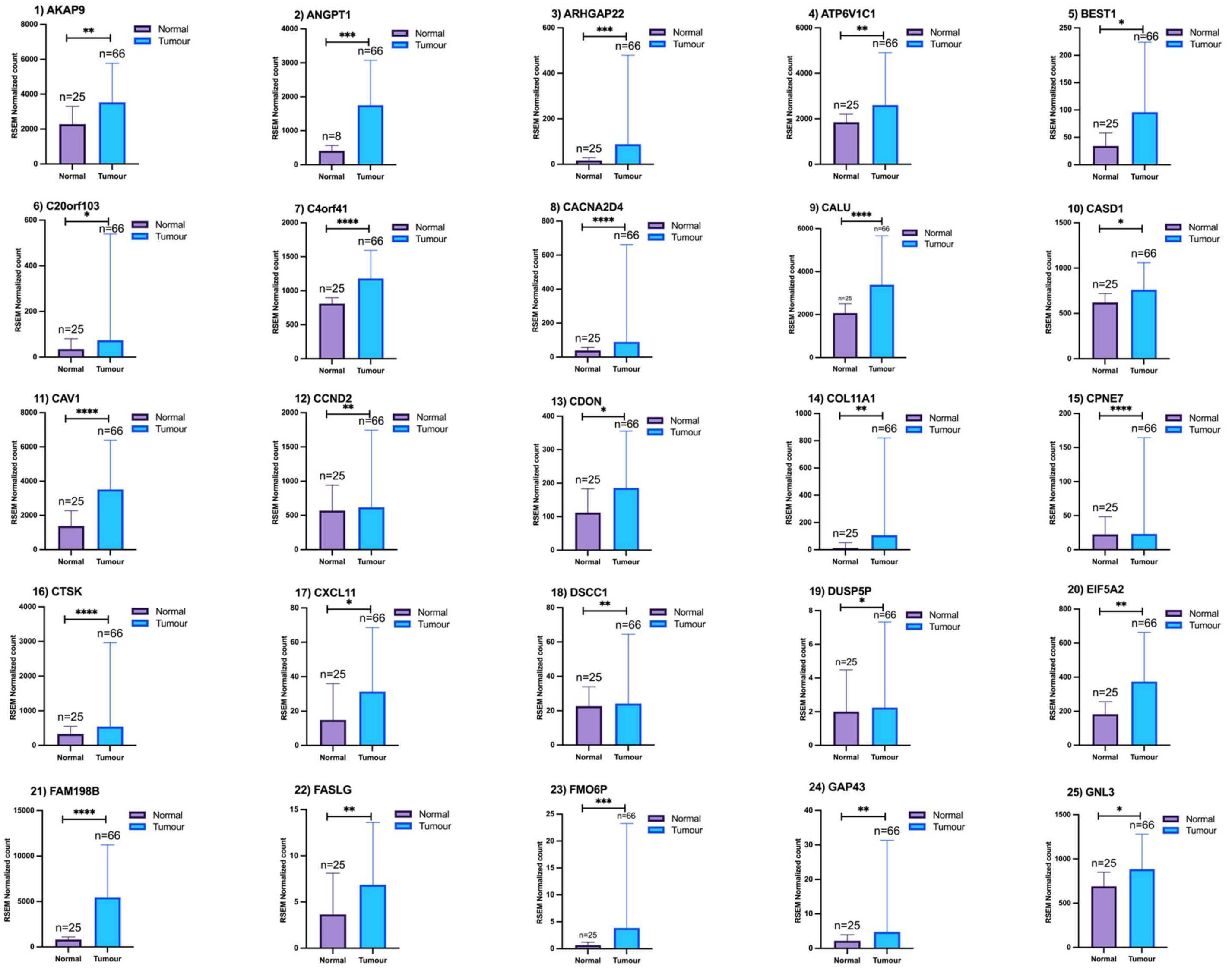

1) AKAP9
2) ANGPT1
3) ARHGAP22
4) ATP6V1C1
5) BEST1
6) C20orf103
7) C4orf41
8) CACNA2D4
9) CALU
10) CASD1
11) CAV1
12) CCND2
13) CDON
14) COL11A1
15) CPNE7
16) CTSK
17) CXCL11
18) DSCC1
19) DUSP5P
20) EIF5A2
21) FAM198B
22) FASLG
23) FMO6P
24) GAP43
25) GNL3
Normal
Tumour
RSEM Normalized count
n=25
n=66
n=8

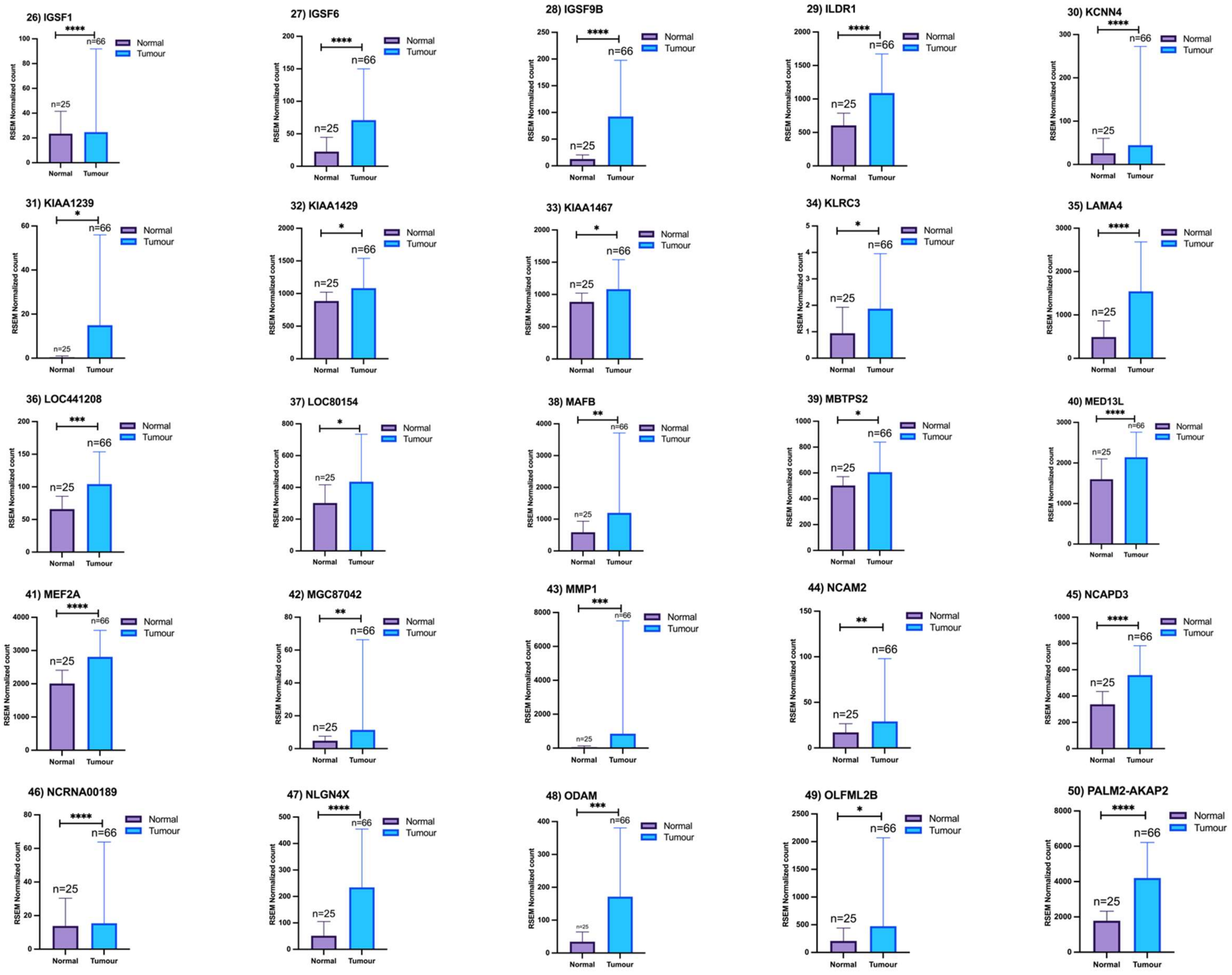
26) IGSF1
27) IGSF6
28) IGSF9B
29) ILDR1
30) KCNN4
31) KIAA1239
32) KIAA1429
33) KIAA1467
34) KLRC3
35) LAMA4
36) LOC441208
37) LOC80154
38) MAFB
39) MBTPS2
40) MED13L
41) MEF2A
42) MGC87042
43) MMP1
44) NCAM2
45) NCAPD3
46) NCRNA00189
47) NLGN4X
48) ODAM
49) OLFML2B
50) PALM2-AKAP2
Normal
Tumour
n=25
n=66
RSEM Normalized count

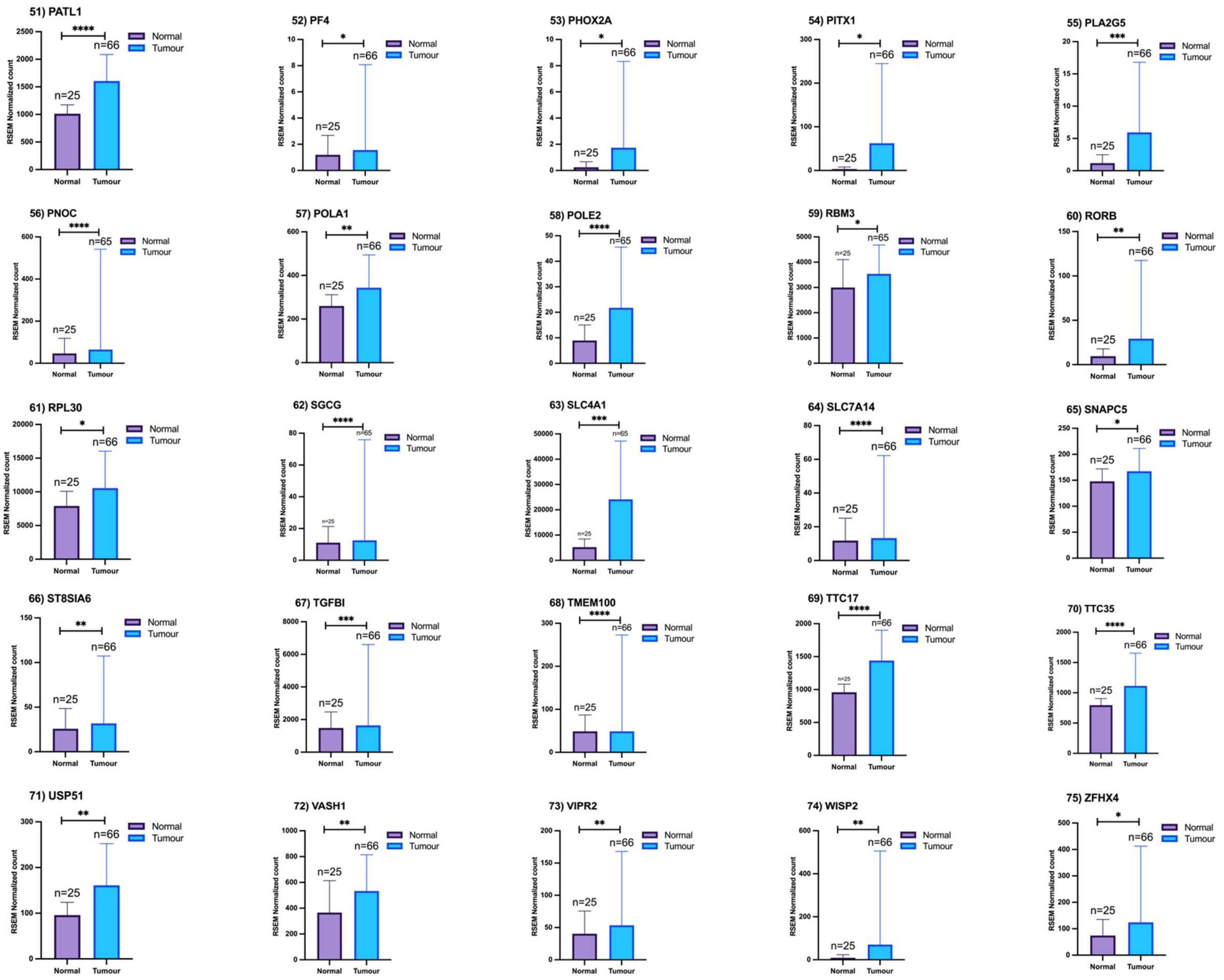
51) PATL1
****
n=25
n=66
52) PF4
*
n=25
n=66
53) PHOX2A
*
n=25
n=66
54) PITX1
*
n=25
n=66
55) PLA2G5
***
n=25
n=66
56) PNOC
****
n=25
n=65
57) POLA1
**
n=25
n=66
58) POLE2
****
n=25
n=65
59) RBM3
*
n=25
n=65
60) RORB
**
n=25
n=66
61) RPL30
*
n=25
n=66
62) SGCG
****
n=25
n=65
63) SLC4A1
***
n=25
n=65
64) SLC7A14
****
n=25
n=66
65) SNAPC5
*
n=25
n=66
66) ST8SIA6
**
n=25
n=66
67) TGFBI
***
n=25
n=66
68) TMEM100
****
n=25
n=66
69) TTC17
****
n=25
n=66
70) TTC35
****
n=25
n=66
71) USP51
**
n=25
n=66
72) VASH1
**
n=25
n=66
73) VIPR2
**
n=25
n=66
74) WISP2
**
n=25
n=66
75) ZFHX4
*
n=25
n=66
Normal
Tumour
RSEM Normalized count

76) ZNF560

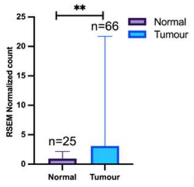

**
n=66
n=25
Normal
Tumour
RSEM Normalized count
25
20
15
10
5
0
Normal
Tumour

Figure S9: Differentially expressed genes in stage 1 of KIRP, [KIRP= Kidney renal papillary cell carcinoma]

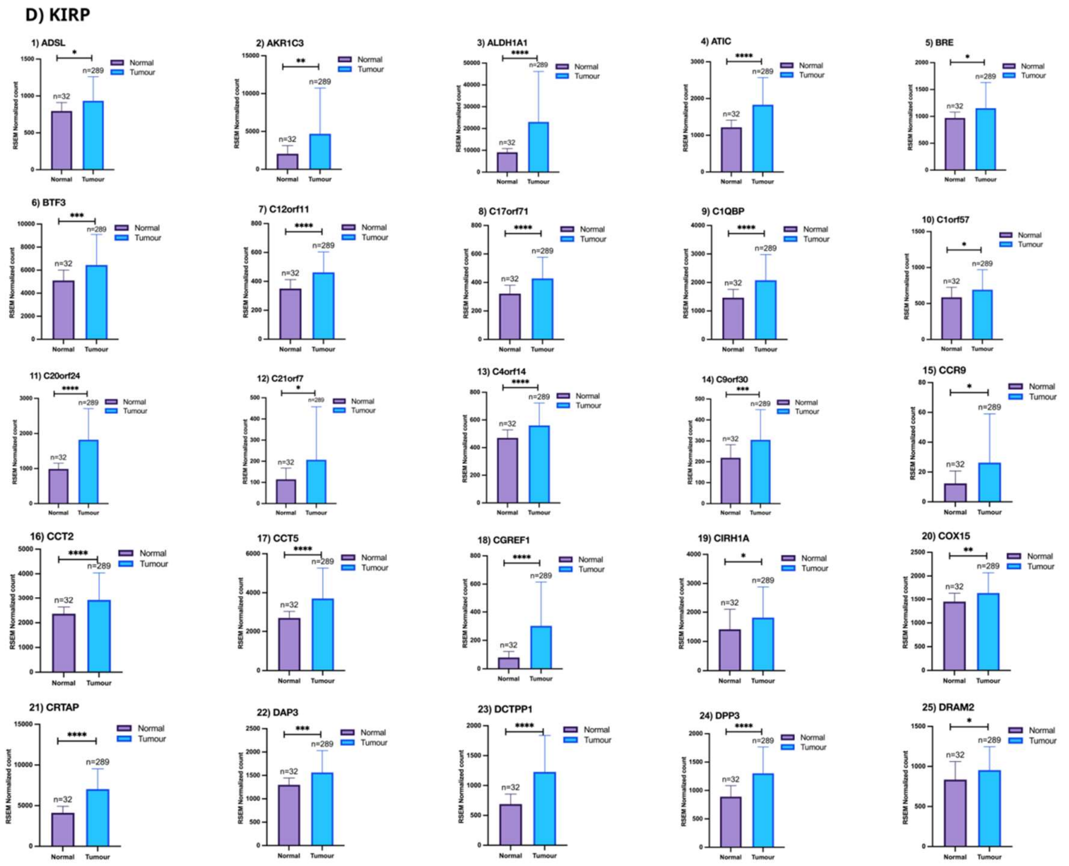

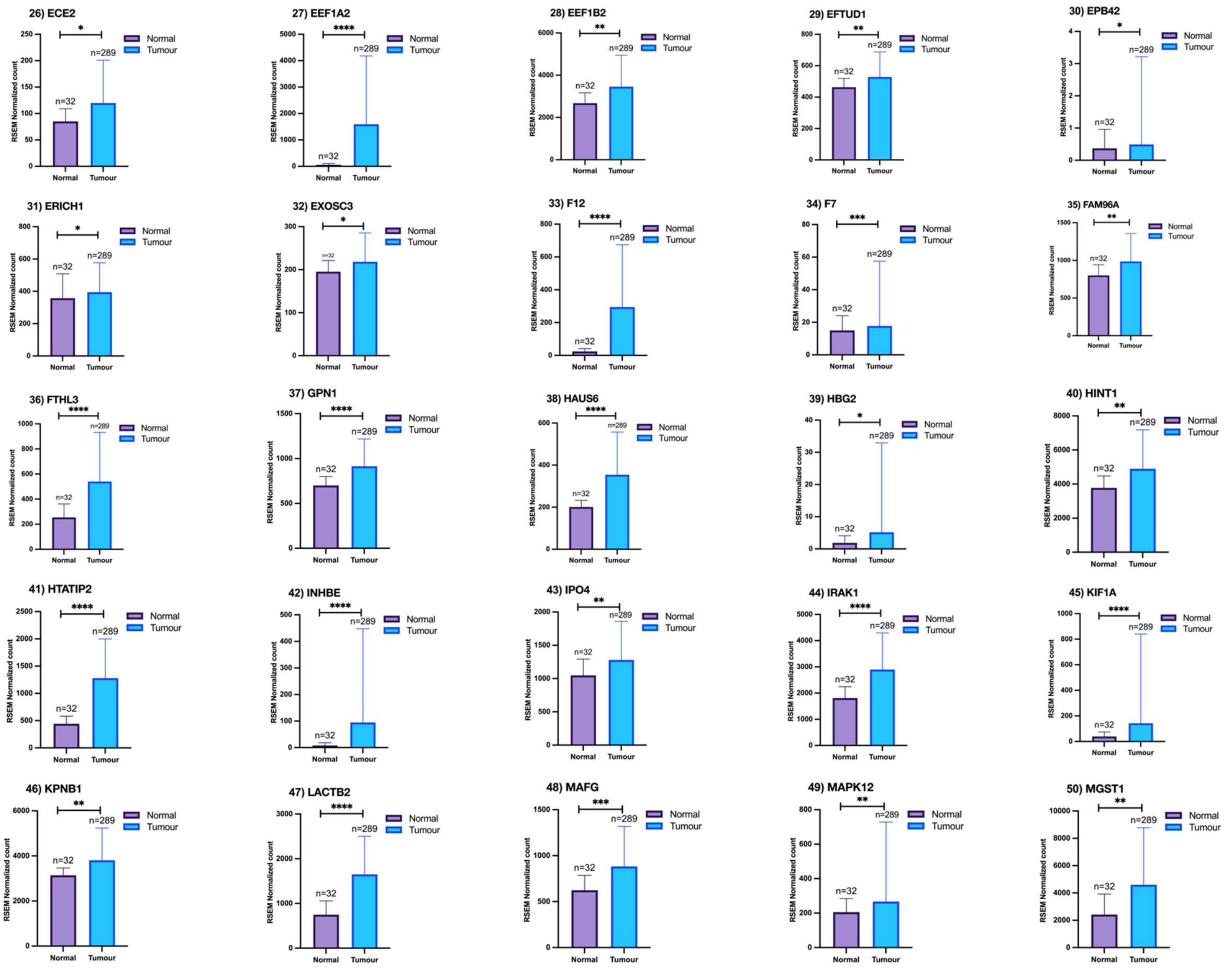

26) ECE2
*
27) EEF1A2
****
28) EEF1B2
**
29) EFTUD1
**
30) EPB42
*
31) ERICH1
*
32) EXOSC3
*
33) F12
****
34) F7
***
35) FAM96A
**
36) FTHL3
****
37) GPN1
****
38) HAUS6
****
39) HBG2
*
40) HINT1
**
41) HTATIP2
****
42) INHBE
****
43) IPO4
**
44) IRAK1
****
45) KIF1A
****
46) KPNB1
**
47) LACTB2
****
48) MAFG
***
49) MAPK12
**
50) MGST1
**
Normal
Tumour
n=32
n=289
RSEM Normalized count

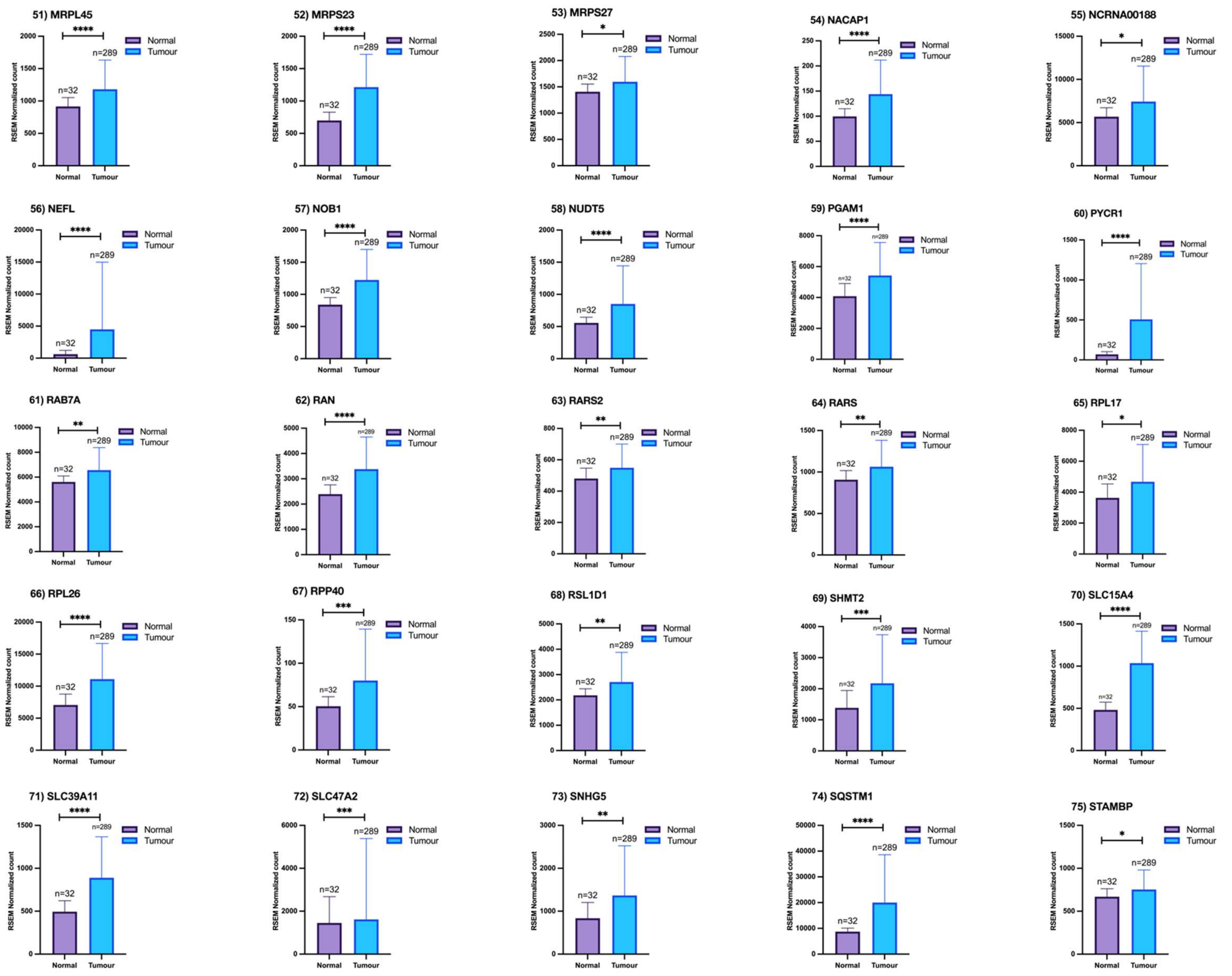
51) MRPL45
****
n=32
n=289
52) MRPS23
****
n=32
n=289
53) MRPS27
*
n=32
n=289
54) NACAP1
****
n=32
n=289
55) NCRNA00188
*
n=32
n=289
56) NEFL
****
n=32
n=289
57) NOB1
****
n=32
n=289
58) NUDT5
****
n=32
n=289
59) PGAM1
****
n=32
n=289
60) PYCR1
****
n=32
n=289
61) RAB7A
**
n=32
n=289
62) RAN
****
n=32
n=289
63) RARS2
**
n=32
n=289
64) RARS
**
n=32
n=289
65) RPL17
*
n=32
n=289
66) RPL26
****
n=32
n=289
67) RPP40
***
n=32
n=289
68) RSL1D1
**
n=32
n=289
69) SHMT2
***
n=32
n=289
70) SLC15A4
****
n=32
n=289
71) SLC39A11
****
n=32
n=289
72) SLC47A2
***
n=32
n=289
73) SNHG5
**
n=32
n=289
74) SQSTM1
****
n=32
n=289
75) STAMBP
*
n=32
n=289
RSEM Normalized count
Normal
Tumour

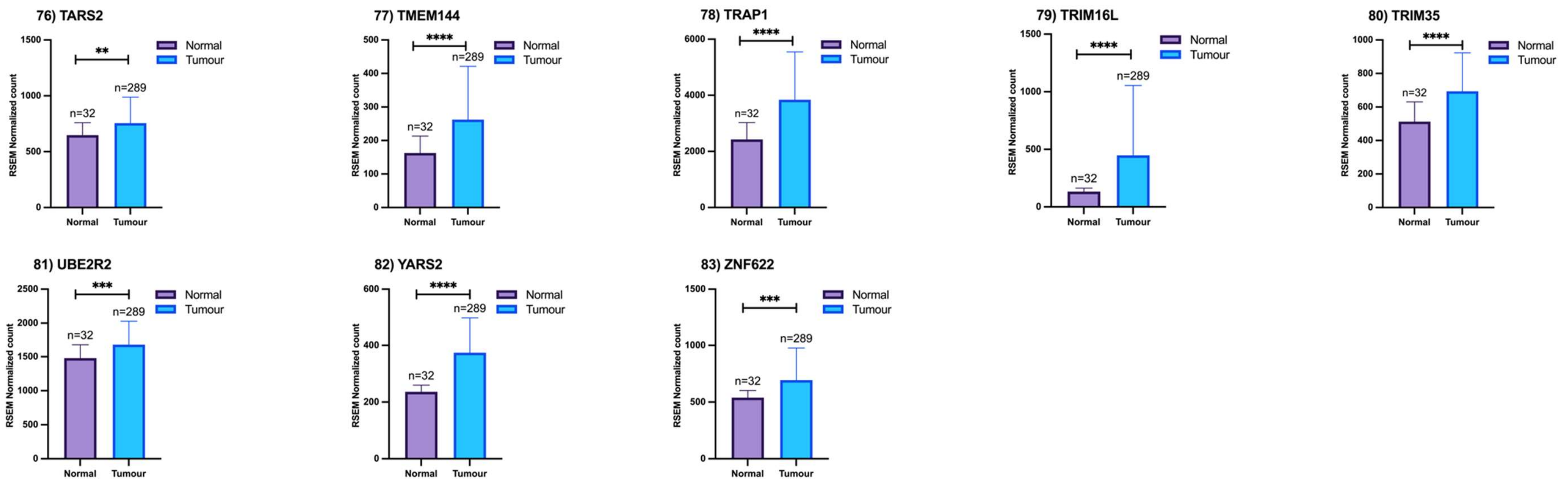

Table S4: List of coexpressed genes with Myc in KIPAN , [KIPAN=[Pan-kidney cohort (KICH+KIRC+KIRP)]

**Coexpressed genes with Myc in KIPAN (Normal)**										
EHD2	CREB5	C6orf150	PTCH2	EPN2	CCL22	IL10	CTSG	MSRB3	TCEAL5	APAF1
CEBPB	LGALS9	CD86	CSPG5	SULF1	S100A9	NCOA5	TRPC4	GPA33	ASB11	TTC17
SNAI1	GRAMD1A	MMP17	ADAMTS7	SLAMF8	VASH2	SNX20	EMB	SQRDL	IRF2BP2	SHKBP1
TUBA1A	CDV3	GJA1	CNKSR3	SORBS2	HLA-F	PCDHGB7	LRRC14B	STAMBPL1	PRR16	SFXN3
RHOB	RGS1	AKIRIN2	PITPNB	ITGAL	JDP2	FMOD	TRPV5	SRGN	TMEM51	MEI1
SERPINH1	TP53BP2	ADAM12	TCF7	MSN	BTN2A1	RNF217	IL27RA	GPR65	SIPA1	FAM189A1
FSTL3	GPR183	VSTM1	UTP14A	HERPUD2	PLEKHN1	MAP3K14	ARPC1B	MFAP4	PDLIM5	P2RX7
TMEM217	NID2	PLK2	GLI2	SIPA1L2	MARVELD1	TTC9	ZBP1	RSPO3	NANS	TNF
ZSWIM4	NPW	LILRB3	NBL1	GLDN	DCP1A	LMNB2	GMEB1	SLC39A1	EXOSC9	NFATC2

GDF5	TAGLN2	RBMS1	GAB2	GNL3	BAG5	LOC728392	NT5E	PPP1R14A	PLSCR1	HRCT1
HAPLN3	BAZ1A	CSDA	HLA-B	KCTD11	PMEPA1	PRND	ZNF532	ANXA6	GAP43	CLGN
CXCL1	CLCF1	RNF122	KRTAP2-1	WISP2	FIGF	ANXA5	CMA1	LDLR	SPATS2L	RRM2
CDC42EP1	ASPHD1	DUSP14	PFN1	CYP19A1	OAS2	CNN3	KCTD17	PACS1	LOC728855	UTP3
CITED4	SCXB	HCK	COL18A1	LIX1L	PTTG1	WDR43	CSDAP1	CHRDL1	RBBP8	PCDHGA6
SPHK1	BMP2	CTSZ	THBS3	FFAR2	MS4A7	LONRF1	ABI3BP	CDC20	ARHGEF6	RCSD1
TM4SF4	MAP3K8	FERMT3	PMP22	HIVEP1	QRFPR	MRAS	RNF112	CD180	AP2A1	HSPA7
COL8A1	MLKL	CRYGD	MFGE8	RRP12	LOC541471	SH2B2	C1orf130	ITGB6	KIF20A	RNF166
COL16A1	PDGFB	RASSF5	GPSM3	TMEM39B	AFF3	C11orf87	NOP58	GPR124	FAM159A	CYBB
IL6	TICAM1	SPI1	GRK6	FKBP10	ZC3H12A	PILRA	NDN	FYB	TAX1BP3	NXN
ARL4C	FGFR1	PRRX2	MC1R	HLA-A	PITPNM1	C17orf96	PRG2	DPF1	SAMD4B	CBR3
TUBB3	GATA6	C9orf21	HIVEP3	CCNK	LY9	CDH11	ARVCF	FGF13	MAML1	FAM109B
TNFAIP3	SCGN	CD80	TMEM119	VAV1	ACTR3	UTP11L	TEX10	CTPS	KDM5B	ZNF263
CXCL2	SERPINF1	MYO1F	CFL1	DENND5A	CCL20	GGN	DNAJA4	ZBTB47	SPEG	C7orf26
PLAUR	NFKBIE	CX3CL1	RAI14	MYH9	IER3	ACHE	CHN1	ARHGAP10	ELL	LOC399959
C6orf145	AEBP1	SFRS13B	JAK3	IMPDH2	DOK3	GZMH	MS4A4A	SP110	SNX22	SIGLECP3
THBD	RIN1	NR0B1	DPEP2	SAMHD1	ARHGAP9	BIN2	POLR1E	TTC28	SOX8	RCAN1
COL1A1	KDELC1	PYCR1	CDCA4	ANXA2	ADAMTS16	BRPF1	ATF5	GUCY1B3	MYLK2	ST8SIA4
IGFN1	ARL 9.00	FCGR1C	MMP16	BEST1	HLA-H	RNF175	IRAK3	CCDC85B	ABL1	MICAL3
ADAMTS4	RXFP2	IFNAR2	PSTPIP1	RARRES3	CRTAM	CNPY3	CTSW	FAM18A	OPN3	SEPN1
SPSB1	UPP1	LGALS1	MKI67IP	NLGN4X	IGDCC4	TCN1	FAM46A	CADPS	FAM70B	MKI67
NLRP3	MAMLD1	PVRL2	SIX3	C5orf58	PGBD1	MKL1	NCEH1	ARMC5	PTMS	PGM2L1
FJX1	DARC	ARF6	FGD2	PPP1R15B	CSNK1D	ATG7	GAPT	CCNL1	IRAK1	CYBRD1
MEX3D	PRR7	SH3BGRL3	ANTXR1	HAS2	IL23A	RNF19A	MTHFD1L	IL2RB	ASB2	OASL
SPON2	GDPD5	IFNGR2	SCGB1D4	CNGB1	RHOA	EBF2	MARCKS	P2RY10	MYL9	LPAR1
ICAM1	IRF8	RAB35	ARHGAP15	PCDHGA5	CLCC1	ITGB8	GPR37L1	RGS3	GDF10	ENG
COL5A1	RIPK2	C2orf89	DYRK2	CD3D	FATE1	JPH2	TTYH2	KRT81	TMEM200A	TRIM11
KRT86	MKX	IFITM1	HAVCR1	NAALADL1	ATP1B4	HIC1	MACC1	TIFAB	CYP11A1	SPRED3

LMCD1	ADAMTS1	KRT8	HTRA3	GLT25D1	FOLR2	HSPB6	HLX	ART4	SAMD11	HTR2A
ICAM4	UBE2S	KIF7	RRAS	FBXL12	TFPI2	EPR1	PLCH1	MAPKAPK2	IL15RA	TRPC2
PDLIM1	MAP3K12	CTGF	C3orf59	KAAG1	CACNA1I	ST3GAL2	ZNF259	NLRC4	B3GNT9	UGDH
CCL2	BTBD19	SLC15A3	C19orf38	C22orf9	NMI	CST2	ZCCHC12	ADCY3	PPP1R12B	FAM71D
MARCH3	NKAIN4	HCLS1	CFTR	LEFTY2	EBI3	FAM65B	SF3A2	FBXL7	MAPRE1	TJP1
PLEKHO2	GPR84	HOMER3	SRM	GRID1	UNC13D	ZNF787	RAB8B	CASP5	G6PD	CDT1
WNT2B	IER5	MT1A	MIDN	DAPL1	LHX6	MAP1B	TMEM132B	BRSK1	RLTPR	MAFF
ANGPTL7	SDS	ITK	MFRP	ARNTL2	PDE4B	WNT7A	TNIP3	KLRB1	FKBP7	APOB48R
PDLIM7	TUBB6	LILRA4	ALOX5	TES	ADAM15	DAGLB	IFI30	OSM	CDH7	OPALIN
CCIN	SULT1C4	PCDHB14	STXBP1	CTLA4	CRLF1	GNB1L	VANGL2	CMKLR1	TM4SF1	AATF
CSF1	C1orf198	ETS1	KLF7	SLAMF1	C1QL2	CRLF3	CLEC4F	FES	PER2	ZNF597
THBS2	ADAM19	RIMS4	SLIT3	WIPF1	CD247	TRPM8	TULP3	IL17B	DGCR11	CXorf26
TNFRSF1A	UBE2I	NCF2	FAM83G	RGL4	IL31RA	SMTN	ADAMTS9	STAG3	CD1D	CBFA2T3
COL1A2	MMP19	C1orf162	RHOH	GXYLT2	C17orf107	C12orf44	GPR161	LPXN	SCARA5	RASL12
CMTM3	IFI16	RAC2	RSPO1	CHSY3	CAPZB	SAP30	SLFN12L	NUTF2	LOC647121	ARRDC5
DNAJB5	GPR37	MCL1	FAM180B	PATE4	SH3BP1	C21orf63	MOBKL2A	ZNF423	CSF2	LRIG1
C3orf36	NFKB1	HLA-DPB1	CD6	TSHZ3	CSF1R	LTA	CCND2	CACNA1G	IWS1	NFE2L2
NUMBL	PROCR	MXRA8	LRFN5	SLAMF6	C9orf150	GEM	CD8A	HGSNAT	TSPAN4	RD3
CXCL6	SH2D5	BEAN	ELF4	CBFB	COPZ2	MARCO	SLC25A32	KIAA0922	MMP15	TTYH3
SFRP4	CAMK2N2	GLIPR1	DUSP8	ARL8A	MS4A6A	STX1A	FCHO1	EVI2A	KIAA1598	NAA38
SELE	OBFC2A	C13orf36	PTGFRN	ARL4A	ZNF831	VCL	TEX13B	RAPGEF5	SIX1	SIAH1
SYNC	RHOU	SOX7	ARID5B	ARHGDIA	ZNF622	U2AF1	CXorf49B	PDP1	MSR1	MICB
ASCL1	MESDC1	LDLRAD3	HNRNPA0	CACNB3	DDX60L	FAM160A2	NPR 2.00	FRMD5	FAM134B	IGSF21
ELN	STAC3	CILP2	MYBL1	MMP25	SKI	CCDC96	AFF2	SMARCD1	RCC1	TNC
BAI2	PRAM1	C12orf68	ADCY7	FAM26F	RPL13AP17	KRTAP1-3	MFSD2B	WDR62	SPC24	NOLC1
COL11A1	NFKB2	PIWIL4	HLA-DQB2	BMP15	CCNJ	EMR2	FCGR2B	TLR4	PCDHB13	NRXN3
MMP14	CD74	C1QB	CD5	FAM64A	C1QTNF4	TMEM132A	MAGEB18	CATSPER1	CYB5R2	TEP1
EMILIN2	SLC38A8	IL4I1	HM13	SETD8	TAP2	TRIP10	AIM2	CASP3	PON2	IL11

DCUN1D3	IRF7	PDIA4	C3AR1	IL1B	ITGB1	ANXA2P2	TLN1	ZNF385A	ROBO1	PCDHGC5
PCOLCE	CTXN1	HCST	ITGBL1	SEMA4C	ACAP1	PCDHGB1	COL7A1	PLP2	POLR3C	TUBB
PODNL1	LCP2	DPPA2	C10orf105	TRIM47	IMPDH1	C1orf216	BTN3A3	CYSLTR1	GNA13	LY6E
FLJ16779	FGR	AFAP1	NCF1	CD96	GAL3ST4	C2CD4A	C16orf72	B4GALT1	PPAN	ADAMDEC1
FSCN1	TNFAIP8L3	C20orf103	AMMECR1L	CA5BP	PCDHB18	STIM1	LILRA3	EGLN2	FLT3LG	CAMK1
ZYX	VCAM1	DOK1	CDON	CXCL11	PLOD3	C17orf87	MARCH4	TREM2	KCNK7	RTN1
HS3ST1	CLEC12A	NAV3	LRRC8E	NME1-NME2	CSRP1	TREML2	ZAP70	TBC1D2B	FERMT2	CXCL9
AKAP12	EFHD2	ITPRIP	SHANK1	PRC1	CDK2AP2	RGS9	FOSB	WFDC2	SKA1	CMTM5
CXCL3	TAP1	FBLIM1	MEF2D	UCN2	C4A	CYTH1	STX4	KCNH8	SCHIP1	ZNF250
TIMP1	SLC35C1	ARMC4	ADAM8	DEF6	CDC42EP4	DYRK3	TRA2B	C2orf27A	PAPD7	ATP2B1
UGCG	CRP	LTBP2	FUT7	B3GNT7	SIRPB2	SYN1	RRN3P2	OSBPL10	GNB1	PCDHB11
TRH	ADAMTSL4	BCL3	SMOX	HAMP	TXNDC3	CEP170	RBM15B	DEM 1.00	ANKLE2	SEL1L2
THBS1	ADAMTS12	LST1	GBGT1	PYHIN1	RNASE1	SLCO2A1	FNDC4	CNRIP1	COL12A1	AXIN1
IFFO2	SFRP2	NFIL3	STK10	NFATC1	CDKN1A	C17orf49	LOC153910	ANXA2P1	CCDC140	ZNF35
KRTAP1-1	GPC4	CDKN3	NMT2	ABL2	WDR82	SNAI3	ARHGAP20	ANK1	IBSP	JPH3
NFKBIA	LRRN1	RETN	PSMB9	CDHR1	CD163	BTLA	CYTSB	SIAH2	ZNF428	MELK
C6orf174	MARCKSL1	PLTP	NRP2	DOCK2	ANKRD35	ESYT1	FFAR3	PPP1R12A	P4HA3	GBX2
CCDC80	SEMA7A	ULBP2	HSPA5	VEGFC	RAB20	TXNDC5	C7orf57	EZR	COLQ	ZNF496
GPRIN1	TLE1	CFH	PALLD	PHC2	HTRA4	DCBLD2	FCRL6	PKIB	OR10A2	CLIC2
RCN3	FCAR	KLF2	LAIR1	GAB3	KLHL6	MET	TDG	E2F1	C19orf50	FOXP3
DLGAP1	ZNF365	BBC3	PIK3R5	GTPBP2	SYT16	PCDHB2	MYLK	WDR44	TMEM185B	NKG7
HK3	GZMB	MAN2B1	HLA-E	MED26	PPIC	C19orf59	PLEKHM2	TAL1	RASGRP2	ADAM9
MDK	LZTS1	CSF3R	UBAP1	EFCAB4B	AVEN	FAM129B	SLC35C2	PPP1R14B	IKBKG	PIGZ
PRIC285	CHIC2	NAMPT	WTAP	PRG4	CD2	RUNDC3A	ASF1B	NETO1	EIF1	EREG
KIAA1949	WISP1	PTCHD2	CNR1	BEND6	DEFB103B	DDX27	CAV1	CD163L1	SULT4A1	ADCYAP1
TYMP	DAPK3	ADAMTS3	GFPT2	KIAA1199	RNASE6	KLK5	SIRPB1	CD1E	EMID1	TNKS1BP1
RELB	MEIS3	PTPRE	TREML3	FPR3	RAB33A	ATF4	PYGM	ALK	TRAF4	MATK
TNFSF9	MMP2	CDH6	TAF5L	CSTA	ACVRL1	PIK3CD	DEC1	RNF182	CD84	TMED9

HCN1	C9orf25	GPR97	GRRP1	ADA	KIAA0748	NUDT1	MAP3K6	THEMIS	ABCA8	RHBDD3
GBP1	NFIX	PRRX1	NKIRAS2	CSF3	BIRC7	PTPRCAP	DDX3X	SEPT1	CHRM4	CIB2
PTPN1	OXTR	CEACAM21	CHPF2	F2RL3	EDN1	PDGFRB	ZHX2	RAB34	TCF23	STK17A
WIF1	RASSF2	KIAA0020	STS	EEPD1	IL2RG	TNFSF18	PLBD2	RNASE2	RAMP3	ZFP36L1
RRAD	NFKBIZ	PIGR	C19orf22	SMURF1	DUSP5	SLC18A1	CHST11	LOC100134259	RAB43	ITGA9
AG2	C11orf9	TUBA1B	GOLGA7B	AGAP2	FHAD1	YWHAZ	C19orf30	C1QTNF3	CD38	MED15
ADAMTS2	HSPB8	SELPLG	SGK1	TRANK1	LRG1	C11orf21	ITPRIPL1	BHLHE40	DENND3	C3orf64
MAP7D1	FADS3	GPR171	SDC4	LOC100270710	UNC93B1	CXorf21	SIGLEC10	SELP	DUSP1	XCL1
BCL2A1	TMEM173	IGFBP6	LY96	WASF1	TNFRSF10A	BTN3A2	DONSON	RBM7	RNF149	PUS1
FNDC1	CNKSR2	SPRY4	LRP1	S1PR4	GZMK	TBC1D10C	C16orf54	CLIC3	KIF4A	CD55
SERPINE1	GNAI2	EPSTI1	TEAD3	LYPD1	CCL21	SYT4	PDPN	CHST7	KCNMB1	CSPG4
ATP1A3	CD300LB	PHF17	LIMK1	CRYBB1	CRELD2	BACH2	EVI2B	MOBP	CRLF2	MEST
TWIST2	COL6A3	SDC3	TXLNB	ADCY2	ECE1	MPZL1	CASS4	ADARB1	TOE1	EEF1D
COL3A1	NNMT	PSTPIP2	PANX1	SVIL	FOSL2	SPOCD1	RSU1	MFAP2	NAB1	PCDHA4
LRRC59	PDGFRL	MSC	TTC39C	NECAP2	CCDC141	EVL	LYAR	AMOTL2	CASQ1	DCX
HAS1	KRT80	DDX5	LAT2	SOX4	CCDC9	ITIH3	NLRP1	RFC2	OPN5	TLX2
CD276	CDC42SE1	MT2A	TNFRSF9	ALPK2	MICALL2	HAS2AS	LILRB5	PRPF38B	BIK	YTHDF2
PEA15	ARHGAP17	GMIP	PMS2L11	IL12RB1	CLEC12B	SEMA3A	GSN	RTKN2	CHEK2	TPSAB1
COMP	GBP2	GLI1	LRRC41	CEACAM3	TBCCD1	CCR5	ATP2B4	AKNA	TAC4	P2RY6
GLIPR2	KCNT2	MAP4K4	LAPTM5	FBLN7	FLJ10357	EID3	EOMES	CKS2	GDF11	CKLF
DLGAP4	ARID5A	PLEKHG2	C13orf39	HAVCR2	CCDC81	PCDHB12	CYBA	LAMA2	LMNB1	ITPKA
C1QTNF6	IL1R1	PARVG	ITGB2	RAB42	CLEC4E	HMHA1	TMEM156	ARRDC2	TPBG	DYNLL1
CCL4	THBS4	FMNL1	ARHGAP30	CDKN2BAS	NPFFR2	PIP4K2A	DNMT3B	UBE2Z	LASP1	PLCB2
SYCE1L	OR2B11	MEX3C	EFEMP1	STAB1	LOC283070	CHODL	KIAA0247	CPXM1	SDPR	RPGRIP1
PVR	NGFR	C1QTNF2	TRAT1	NMNAT2	TMEM71	CALR	MNT	GLT8D2	TP53RK	GFRA2
MFAP5	AHNAK2	GDF1	DDAH2	SPRY2	INPP5D	KIFC1	C19orf35	CBLN4	TET3	CRIP1
MAGED4B	CSNK1E	MYD88	LEPREL2	PLA2G7	C2CD4B	CD52	ANKRD50	JUB	SSBP4	CCDC137
RELT	CD72	MTHFD2	ARL 14.00	YPEL4	GFI1	RIPK1	GABPB1	TRIM27	FAM78A	RYBP

TNFAIP2	FCN1	F12	IFI35	TGFB2	FXYD5	CACNA1A	CCL3L1	GYPC	VSIG1	SPC25
BYSL	TMSB10	LRAT	TLR2	SH3RF3	IQGAP3	DUSP23	LAMP3	PBK	CRY1	JAM3
C1R	LRRN4	CENPW	TNFRSF12A	MGC87042	IL2RA	OAS3	ILK	TRIM16	MYO1D	NCR3
IFITM3	RHBDF2	CORO7	KRT23	EPHA7	KLF8	TMEM59L	LEF1	FAR2	APBA1	SLC43A3
PLEKHA4	TNFRSF1B	PTRF	CASP1	IFNGR1	DDX21	IL7	AIPL1	IRF4	GNB2	TSSC4
LOH3CR2A	TWF2	MANF	KRT75	C10orf54	BID	PHLDB1	FOXC2	GFRA1	SPRED2	PTPN6
RHOG	SIGLEC1	GMFG	APBB1IP	SMARCD3	FAM113B	MX1	CEP55	LOC283050	DCDC2	PDLIM3
GHRH	GNL2	MAP1LC3C	OMD	FGF7	CD33	ITSN1	GTPBP4	ZNF668	KCNA10	MYL12A
HTN3	PIM1	FEZ1	CCL5	KCNB2	SDK1	ROM1	NPC1	FCGR3A	NOD1	FAM110C
KRTAP9-9	RBM46	CSRNP1	SEL1L3	WDR46	DCUN1D5	FAIM3	DNAJC5B	WDFY4	GSG2	CUX2
LOC255025	CD300LF	MICAL1	ICOS	PDZD4	CRISPLD1	TRAF1	CECR6	HAS3	ZNF608	FAM177B
TRIM43	HLA-DRB6	CCRL2	RAB31	LAMA4	B3GNT5	HEPH	GALNT1	CAMK1D	IFI44L	RPS6KL1
TRIM48	C1QA	NLRP12	CYP2C9	GPR132	UBA7	SLC11A1	IL18RAP	MICALL1	RAB23	AOC3
PLA2G2A	IGF1	FBN1	CD28	TNFRSF4	LDLRAD2	C3	NRAS	MACF1	CD2BP2	SHOX2
SOCS3	PML	C1RL	C12orf70	RALGDS	IL34	PTPN2	PDCD1	STIP1	RFX8	IFFO1
KLF16	LGALS9C	FCGR1A	LRIT2	A4GALT	STAT6	C17orf62	PABPC4	VASN	DDX58	GAS7
B4GALT5	C17orf60	PRDM1	KRT33A	AADACL2	SLC32A1	RGS10	RHPN2	TMEM151A	TAF1D	RRP1
EMP3	ANKRD13B	MEGF11	NUP62	KLF10	FAM118A	ASAP1	NUDT10	RPE65	IGFL2	TLE4
EIF4A1	FILIP1L	FOSL1	DPYSL3	PIK3R6	C2orf18	ADAM33	NAB2	FAM176A	TGM2	TPM4
TGFB3	RASA3	MESP2	NCF4	GZMA	ACTG1	NKX6-1	PLEKHG4	CCT5	CD40	TPX2
KLF4	TTLL7	HTR3A	WNT6	SH3GL1	IKZF1	ODF2	CXorf36	PYGL	TRIO	LAX1
PLK3	CNIH2	TUBA1C	CSGALNACT2	CD53	PPP4R1	RUNX2	UCK2	RND1	FGD1	GATS
PXDN	CPA4	FCER1G	RGS4	RPS2	TAGAP	ZBTB8A	PRKCH	CST3	PRKD2	GPR141
DLC1	SAMD14	SH3PXD2B	KIAA1274	DISC1	PCDHB15	RGMA	NOP16	PAK1IP1	HJURP	CD200R1
NINJ2	DTX3L	CDH23	STEAP4	GRK5	PSMB2	CADM4	C8orf47	IDUA	PCDHGB2	CCR8
SAMD4A	CARS	ALDH1L2	HLA-DMA	FRMD8	APOBEC3C	APOBEC3D	LRRC32	FGF16	CEBPG	CAV2
OSCAR	SPARC	NLRC5	KCTD5	CXCL10	PCDHB9	EIF2AK3	TNFRSF18	TNNI2	FAM135B	FAIM2
XIRP1	PRICKLE1	UTF1	SLC24A4	ARMC3	MCAM	ACTA2	TGFB1	MEG3	SPATA12	DNTTIP1

C5AR1	HEYL	COL6A1	NPDC1	SVEP1	VWA1	RNF145	DGKD	GRM2	TMEM54	MS4A14
BATF3	RAB32	BMPER	ALOX5AP	GGTA1	MAP4K1	NPFFR1	TRIB1	S100A2	CD8B	SHC2
LOXL1	KIF3C	CD69	IL21R	HIST1H4J	LOC285548	GRN	FAM110B	NAGK	NELL2	KRT34
RND3	MATN3	FBLN1	ENAH	FAM167B	COL8A2	FOLR4	ARHGEF1	C1orf129	DDX39	APOBEC3H
MAGED4	SHISA5	CD40LG	CLDN1	PHF13	TCEAL7	CLEC1A	F10	PARP8	KCNQ5	STK38
C13orf33	S1PR2	INMT	APOBEC3F	CSRNP2	HOPX	C12orf45	DULLARD	SPATA2L	ZNF217	RPS19
C1orf38	CD97	DEGS1	NAPSB	EML3	OR10K2	BIRC5	DCHS1	SIGLEC14	TNIP2	IGFBPL1
KLK8	CXCR2P1	DACT1	GPR172A	WNT10B	CALD1	FLI1	HECA	ADAMTS18	ITGAD	CAD
C8orf71	CD248	PYCARD	LY86	CD93	ETV7	SH3PXD2A	RCE1	OTOF	CHRD	VENTX
ANXA1	TRPV2	ISG20	PARP9	ISG15	FST	C1orf68	CCNB2	SNAPC2	PCDH19	RBMXL1
KCNA6	NHEDC2	SERPINA3	RELL1	SLC17A2	ISG20L2	LYPD5	GPR19	GRAP2	BRD9	SDF2L1
SLC2A3	MED17	FGF21	MIR155HG	BST2	DSE	SRF	RBBP6	ARHGAP25	STK38L	FNBP1
MEGF6	SH2B3	PAPLN	SEC24D	LIPN	NCF1C	MED6	KLHDC7B	ITGA11	RBMX2	SLMO1
CCKBR	NDEL1	LILRA2	LYZ	RUFY4	SH2D1A	VPS37C	IL17D	RNF19B	FXYD1	SCMH1
RARA	ABCB4	CD14	CD4	GNAI3	FAM110A	GADD45B	GDAP1L1	STOM	HLA-DOB	ARHGAP11B
SCARF2	SOD2	CASP4	CD7	HLA-DPA1	CDC42EP5	NID1	RPS6KA4	NUSAP1	TNFRSF6B	SCN5A
LOC100126784	PAQR4	SLC2A10	HSP90B1	MAK	CALU	CENPM	EMR1	INHBB	TCF3	LOC100129550
CXCL16	SGK223	FCGR1B	TRIM38	IFI27	C2orf85	ADAM2	BCL10	GPR55	EIF1AD	ANO1
COL6A2	TNFRSF10B	HKDC1	ACTN4	C2	H2AFX	OGN	PELO	PLA2G5	SLC10A3	AMH
CNTNAP1	NT5DC3	TNFSF8	AOAH	STMN3	FAM53C	COL10A1	ZSWIM6	GNL1	LPPR2	CRABP2
HNRNPCL1	UNC5A	SRPX	CD200	CLEC2B	CCDC120	FSHR	CSK	AKAP2	OPRL1	SH2D3C
LILRA6	ISLR	DPYSL4	SH2D2A	COL13A1	PPARD	SNAI2	PCDHGA12	PLXDC2	TRIOBP	NDP
TPM1	PTRH2	MX2	BIRC3	CYTIP	CLEC7A	SLC37A1	CIITA	ENPP1	KCTD20	DPP9
PTX3	LOC606724	VIPR2	NRP1	CLEC10A	CYTH4	ERF	CRYAB	ZCCHC24	LRP10	ABCA6
EXT1	ZNF469	BASP1	POU2F2	CLEC4A	SMPDL3B	OLFML1	CELSR3	POLR2D	SEC23A	MAZ
CD177	C8orf84	PTP4A3	RIN3	EZH2	NCRNA00093	CD3G	ACAN	FBXL19	RALA	
MAP7D3	MYOZ3	ZMYND15	RASSF1	SCN1B	CD48	FAM198B	LRRFIP2	S1PR5	PDIA5	
TFE3	SERPING1	CCDC50	RCVRN	MAP1S	CXCR3	MAFB	EBF1	LOC285205	CLDN3	

CFP	KLF6	CD44	EDN2	SLA2	ITGA3	PI16	CLEC4C	NKX3-1	ECM2	
PPRC1	GUCY1A2	CTTNBP2NL	HUS1B	ANKRD53	PKMYT1	SPN	NALCN	TNMD	PF4V1	
ANKRD1	PLB1	CCL3	TPM3	UBE2C	ARHGAP1	MYO3A	MVP	KCND1	FAM114A1	
CORO1C	C1orf127	SFN	RBP1	TMC8	SEMA6A	CCR6	RPGR	ZAK	CCDC3	
RBM24	IFITM2	AMICA1	CDKN2A	SULF2	VAT1	PTPRC	NXT1	CLSTN2	MRVI1	
FPR1	EFEMP2	ITGB3	ETV6	IGFBP7	SERTAD1	CALHM2	CENPA	PCDHGA10	TM4SF19	
SERPINB8	ZDHHC18	MATN4	LIF	CEACAM4	CYTH2	TACC3	ZCCHC5	RUNX1	IL12B	
IL4R	SACS	CD300E	GZMM	NEXN	UNC5CL	SLC37A2	HSPB1P1	TTC39A	ZFP36L2	
DNAJB11	ZFP36	EMILIN1	COL15A1	CTSS	C6	FLJ41941	PLEKHG1	ELL2	SHISA2	
COL5A2	GPR176	KCTD4	JUND	SIGLEC7	SIRPG	KIAA0226	SGTB	CHORDC1	WDR1	
TWIST1	PRR24	DTX2	CORO1A	SEZ6L2	TMEM39A	C19orf10	PDCD1LG2	APOBEC3G	CCDC123	
SLC2A14	PLAC8	INPP1	LILRA1	LTB	GTSE1	PREX1	AREG	ZNF830	CSMD2	
SHC1	HNRNPAB	NOP2	IL10RA	DBN1	NR2F1	MNDA	TRIP13	SPARCL1	FIBIN	
PLEKHO1	CNN2	PPFIBP1	PMM2	HLA-DRA	RHBDF1	RALY	KEL	C11orf88	LMOD1	
TSKS	ATP8B2	IL7R	JOSD1	SLC10A6	PTGDR	CCM2	SLC1A3	PSMD7	IFI6	
BMP1	VASP	SERPINE2	MYO9B	ARHGDIB	CCDC97	EMP1	SLC6A20	XCL2	TBC1D10B	
LILRB2	KRT33B	C1QC	FAS	BTN2A2	STAT4	ADAM17	MALT1	DACT3	WBP4	
HLA-DQA2	NLGN2	DKK 2.00	CSF2RB	COL29A1	VSTM2L	SIGLEC16	GPR25	IKBKE	SGCD	
OSR1	C11orf24	PTCRA	IL32	PGM2	REEP2	CLDN6	ADRA1D	FZD8	FAM195B	
TNXB	NRBP1	AGFG1	TRPM2	FAM49A	DKK 3.00	TNIK	SIGLEC5	DSCC1	SAE1	
FSTL1	TPRG1	FAM102B	CARHSP1	CELF2	WARS	LRRC8A	FCHSD2	STAT2	LOXL2	
PLD2	RFTN1	LBR	BTK	SAP30BP	BCAR1	SQSTM1	ART5	KIAA0355	C12orf35	
TAC1	CEBPD	YWHAG	TSC22D2	OGFRL1	XAF1	DUSP4	RGS20	Sep-09	SRP19	
IL18BP	BATF	DCLK1	LSR	BOC	ROR1	LRRC4C	IL16	XYLT1	MYBPC2	
SOX9	APOBEC3A	KCNQ3	C14orf80	ITGAM	NRG3	YTHDF1	TRIM22	OAS1	GTDC1	
TMPRSS11D	TYROBP	FLNA	KCTD10	AMOTL1	CXCR6	VASH1	SLC26A2	ACVR1	SIT 1.00	
ETS2	DOK2	PVT1	CMTM7	BCL9L	TKT	LTBP4	ITGA4	RAPH1	SGCE	
LRRC25	CKAP4	GPR56	IRF1	TNFAIP1	TMEM149	RET	PPM1M	CCR7	TLR9	

AEN	TNFSF13B	TMEM233	GTF2E2	STK17B	TNFAIP8L2	NGF	C22orf24	LOC152225	NBPF15	
MTA2	C13orf18	KRT18	RPIA	LRRC42	FAM129A	VCAN	CD1C	ANP32B	DEFB109P1B	
OLFML2B	PLEK	HLA-DOA	TP53	TMEM200B	SECTM1	CLEC5A	SYDE1	LRRN4CL	MEOX1	
CYP21A2	LOC100130776	CRISPLD2	DTNA	FHOD1	ADAMTS10	C21orf7	IFI44	PSORS1C1	CRYBB2	
PPP1R2P9	PRKCDBP	MAPK7	CHI3L2	ARPC5	CST5	PCDHB10	TDO2	ZNF267	DUSP16	
HRH1	ITGA5	KCNK6	FCGR2A	NOD2	LTB4R	PTPN22	NPTXR	BCL2L12	TAPBP	
VIM	CFB	ZFPM2	FAM109A	SAMD3	PLD4	C17orf53	CLDND1	TBX21	TMEM214	
KIAA0802	GNA15	SIGLEC9	TEAD4	CCDC88B	C11orf84	RCN1	C2orf77	RPF2	CD1A	
MEX3A	ODF3L1	HABP2	MUC12	PNRC1	VSIG4	CD37	C12orf77	LUM	KANK2	
STX11	KIAA1045	FKBP1A	WNT5B	NCKAP1L	ACOT9	GNG4	SLC9A9	CD209	MGC45800	
FUT4	CLEC11A	MMP23B	TRIM21	SASH3	TMEM98	AHR	KLHL25	GIMAP5	LMBR1L	
COL4A2	CD300C	CD151	OSMR	FHL2	PECAM1	RPL28	NPPB	LYVE1	RPL39L	
CADM3	MGP	EPHA2	CCDC46	CHRNB2	UCN	EXOSC3	SLAMF7	CCL14	ANKDD1A	
GALNT5	SLC27A3	RASAL3	GRIA1	PTPN12	CAP2	FOXM1	B2M	EIF2S2	LGI1	
S100A16	WAS	LILRA5	HP	ARHGAP22	NACAD	CCDC86	ZDHHC5	ANXA4	SERTAD3	
PODN	YWHAH	PSG1	ELK3	TAGLN	FAM150B	LTBR	NMB	RNF213	KCNH2	
C10orf10	CYR61	DUSP10	MYO1G	HTR7	FLNC	THOC6	CWC22	TSTA3	PIM2	
RAP2B	LMNA	SHF	AXL	FKBP11	APOLD1	CDR2L	FCGR2C	DMP1	LRRC15	
TMEM2	CCL18	EPB41L2	PRSS23	IL8	IPO4	TMEM22	NIP7	E2F6	ZNF536	
CHRM1	ITGAX	LHFP	KLHL29	SCRG1	REEP4	SV2A	HMGA1	RASSF3	FN1	
ODF3B	ROPN1L	OLFM4	SERPINB1	POSTN	PPP1R9B	ITGA7	LRRC20	SYNCRIP	SCT	
SCG2	LEPRE1	WDR69	NUDT11	KIF21B	NFKBID	PLIN3	IL18	ACBD3	REXO1	
SLCO5A1	DNM1	FZD1	SMAP2	PLXDC1	KIF19	SELL	PDE5A	RAD51	LENG9	
C1S	COL4A1	CD3E	PCDHGC3	MMP7	LTC4S	RIMKLB	SEMA6B	SYNPO2	AURKB	
SPRY1	S100A3	IRF9	PSMB10	WSCD2	FADS2	SF3B4	GNB4	PLCB3	ENDOD1	
RRS1	PHF19	BAX	DNAJC2	DBNDD1	NCRNA00152	PAQR9	HDAC7	REG1A	EBF3	
C1QTNF1	IRAK2	LOC148145	ITPKC	CDR2	UTP6	TMSB15B	CHD1	IL10RB	NOTCH2	
ARSI	CD300A	CDH24	PLAC9	THOC4	IL27	IL13RA1	TEKT3	INTS8	HIVEP2	

SERPINB9	LOXL3	CSF2RA	LOC100233209	SLFN11	PCDH17	LRRN3	AXIN2	NR4A3	CLDN4	
POLR3D	TMEM43	NOP56	GGT5	RUSC2	CDK2	TRIM67	PLXNB3	LOC727896	SH3BP5	
CABYR	CCL4L2	CD63	LPGAT1	RASGRP4	RFX2	DDHD1	SFRP5	C6orf141	KLF5	
CDH22	CLIC1	FPR2	CD101	CCRN4L	CD81	TCTEX1D4	SFPQ	PI15	ACTN3	
DMRT1	LIMD2	SLA	SSTR4	CARD16	NEDD9	HYOU1	KREMEN2	GEFT	CCL7	
CHSY1	MN1	C10orf26	TNNT3	HBEGF	TMEM196	HAUS2	URB2	ELMO1	MAP1A	
ACTN1	GRASP	PPP1R15A	FHL1	FLRT2	ENTHD1	NDC80	TPM2	BACH1	SPNS1	
TGFB1I1	LATS2	TIMP2	TIGIT	GBP5	CHFR	CACNA1C	ATG9B	CCDC88A	PCSK5	
MDFI	LSP1	PTGIR	CAMSAP1	CFHR3	MGC12982	LHX9	DPY19L1	CCR4	C10orf128	
MICAL2	PHLDA1	RELA	PPIB	PPAP2C	GRIN2D	ARHGAP23	KCNJ14	JUNB	GPR126	
MMP11	TSPAN18	RGS19	ARRB2	DGKA	LAT	SLC1A4	IKZF3	CD3EAP	BGN	
TREM1	NFAM1	TBXAS1	KPNA2	CXCR4	RUNX3	ROBO4	LASS1	ADAP2	CD68	
MYADM	KDM6B	FAM5B	ADPRHL2	ACTB	C9orf91	CLEC4D	FAM126A	A2M	SMNDC1	
FHL3	INHBA	EHBP1L1	LCP1	GAR1	LOC338651	KIAA1210	HSPB1	WNT9A	TINF2	
SBNO2	CCDC109B	TNFSF10	SYT11	MPEG1	S100A8	PDLIM4	IGSF6	P4HB	CPM	
LY6H	LILRB4	LILRB1	SEPT5	JMJD6	UBASH3A	XPO6	STAT3	ZNF193	MORF4L2	
PLSCR3	KBTBD2	CST7	PKDCC	SLC24A3	MRC2	PPM1D	TMEM158	PHLDA3	PLCG1	
**Coexpressed genes with Myc in KIPAN (Stage1)**										
NFIL3	ZFP36	CCRN4L	CYR61	BYSL	MYADM	ADAMTS4	JUNB	MCL1	FOSB	KLF10
C6orf145	ITPRIP	PPP1R15B	RIOK1	NOLC1	DDX21	POLR3D	PPRC1	DCUN1D3	CSRNP1	KLF6
EIF4A1	LYAR	WDR43	DUSP1	DNAJB5	SOX7	SLC2A14	ISG20L2	SRF	JOSD1	JDP2
RHOB	SLC2A3	XIRP1	F2RL3	SERTAD1	SNAI1	SOCS3	CCL2	THBS1	C9orf21	ETS2
TNFAIP3	EMP1	ERF	FOSL1	PNRC1	B3GNT5	LATS2	APOLD1	GTPBP4	CSGALNACT2	AATK
JUN	STC1	KDM6B	NDEL1	SEMA4C	GEM	URB2	GPR4	SPRY1	DDX3X	WTAP
ARID5A	ETS1	C1orf183	TNFRSF10D	TNFRSF10A	PLK2	PDGFB	AG2	PPP1R15A	RNF19B	HBEGF
ICAM1	C1orf107	RGL1	BCL6B	PELI1	SEC23A	SERPINE1	ETF1	ITGA5	KCNE4	DENND5A
FOS	NFKBIA	SH3BP5	TAF5L	PFKFB3	SPRY4	TAL1	SRGN	PLK3	DLC1	RNF122
BAZ1A	MAFF	RAI14	SERPINB8	MESDC1	AREG	KLF4	C10orf108	CHSY1	PLEKHG2	ASAP1

SPG20	MAT2A	ADM	LRRC8A	PPP2R2A	PTPRE	NFKB1	ABCE1	NUP153	SLC25A32	LMNA
PELO	SEH1L	ACLY	SHANK3	CFLAR	SH2B3	YRDC	TRA2B	C10orf119	ABL2	FOXC2
EIF2S1	S1PR1	NCL	HBB	STARD13	DNTTIP2	KCTD15	MSN	CALD1	TAF4B	GRK5
LIF	NEDD4	JMJD6	POLR2D	BMP2	SPRY2	SLFN11	NUPL1	LRRC70	DLL4	EGLN3
PHLDA1	CDK17	ZNF295	CXCR7	DGKD	SLC35F1	CA9	PYGL	TEX10	SERTAD2	PCBP1
C9orf30	C1orf55	UBC	NOP14	RBM8A	RBMXL1	NOTCH4	ATF3	GNA13	LDB2	KCTD20
FRMD8	ANGPTL4	P2RY8	EPAS1	VEGFA	STC2	AK3L1	EPHA2	HNRNPK	EGR3	FAM38A
PDLIM2	ELOVL5	LMCD1	NRAS	ANKRD40	UCK2	NEDD9	PLEKHG1	COL5A3	TSR1	BNIP3L
MKI67IP	NID1	CD36	EIF2C2	CDKN2B	ENPP3	RIPK1	DDX50	RLF	NOS3	COL25A1
DUSP14	PEA15	FLT1	PLXND1	FILIP1L	PHF13	PANX1	RALB	INHBB	KIAA1949	BARX2
KCNAB1	HIVEP2	CWC22	CORO1C	HSPA14	AMMECR1L	FOSL2	CNST	S1PR3	HDX	C15orf39
ARRDC3	EPC1	QSOX2	THBD	CD97	ZCCHC2	PDE4B	PRPS1	P4HA1	RBM12	SLC16A1
EIF3J	SCARF1	USP13	GEMIN5	DYRK3	PRPF4	ACTN1	SLC10A6	PITPNB	WAC	KPNB1
DPYSL2	KCNK3	TCP1	FLT4	DNAJA1	NONO	HTR1F	C13orf33	ITPKC	SMTN	TNFAIP8L1
SHC1	TIE1	ADAM17	SH2D3C	YME1L1	CXorf36	C7orf68	SKIL	ADCY4	NRP1	RSL1D1
KIAA0020	HNRNPU	CD93	KBTBD2	MAP3K14	RPS6KA5	RBM15	RRAGA	CD34	LRRC32	PTPN12
GDI2	CDH5	TMEM2	NFATC1	CDK18	NAA15	HIC1	GJA4	DOCK6	WWTR1	MPI
HYAL2	PRR16	ERG	APOBEC3A	CACNA1C	SEC14L1	STEAP4	EHBP1	RBMS1	BATF3	PNO1
KCNE3	APLN	GJC1	SHROOM4	GNB1	SIK1	GPR176	STAT3	DCLRE1B	NOVA2	C6orf150
CNN3	SLC15A4	NDRG1	PITPNC1	ZNF395	CLEC1A	F2R	SFRS3	PLEKHA2	LOC154761	SCARB1
C10orf10	SHMT2	OPTN	PGM3	CHD1	FAM115C	FAM57A	PLVAP	PFKP	FAM102B	ARAP3
MAPT	PEAR1	CSPG4	ROBO4	AFAP1	PLXNA2	RELB	TYMS	PTPRM	SAP30	TMEM194B
ZNF281	BTG1	SFRS7	NUP98	TMIGD1	KLF9	FYN	RHOJ	STK10	C13orf15	GPR3
DYSF	GRRP1	PGK 1.00	DAXX	FAM70B	C22orf45	ELTD1	SGCB	TMEM66	KLHL20	MSH6
PPAP2B	EGR1	UBA2	STX11	CCDC102B	PRDM1	MAP4K4	RAPGEF5	VIM	CALCOCO2	FDPS
TIMP3	ERRFI1	ZNF267	ENPEP	PRPF38A	RASGRP3	SOX17	SIRT1	SDPR	NFATC2	NAF1
INTS6	PER2	MMRN2	MCAM	AFARP1	RAP2A	KIAA0146	IL6R	PWP2	FRMD3	GEMIN4
TBCCD1	PDGFRB	BTN2A1	GNAI3	PCDH12	HNRNPA3	SPRED2	NETO2	C3orf59	LONRF3	TNFRSF4

GIMAP5	FAM110C	ZBTB2	PIP4K2A	HAT1	CDC42SE1	SWAP70	MYCT1	ARID5B	ADAMTS1	ARHGAP42
C9orf25	ADRA1B	GPR135	POLR1E	ALKBH5	ESAM	HEATR1	LHFP	CYB5A	S100A12	FAM43A
NUAK2	SPARC	BNIP3	PVT1	GPR183	EGOT	FHL5	CHIC2	PAIP2	PRMT5	PAK1IP1
ZNF496	LZTS1	ZEB1	ETV3	RNF19A	SLC25A43	DEDD	SYNCRIP	PABPC1	BCL9L	TCF4
COL21A1	COL6A3	SASH1	SGK1	UBE2J1	BHLHE40	NR4A3	ELL2	RERGL	FCHSD2	FLNA
NUP62	EXOC3L	WDR75	PGAM4	SYDE1	NOTCH3	CEP170	ARHGEF7	BHMT2	PDK1	PTP4A1
CASP7	GPATCH4	SLC35D2	RNF145	EBF1	GART	INSR	CAMK4	HEPH	PPM1F	GALC
TRPC4	CXCL2	LEPROTL1	DAB2	ZEB2	HNRNPF	JAM3	RALGDS	KHDRBS1	RBM7	TMEM22
WDR3	ACBD3	RSU1	ADA	MAPK12						
**Coexpressed genes with Myc in KIPAN (stage2)**										
ZFP36	SOCS3	MCL1	FOSL2	CYR61	JOSD1	NFIL3	ICAM1	THBS1	ADAMTS1	HBEGF
STAT3	KLF6	BCL6	ABL2	EIF4A1	PLK3	JUNB	EGR3	IL4R	DNAJB5	FLNA
CORO1C	RND3	PFKFB3	PYGL	PHF13	SGK1	C9orf30	IL8	MYADM	KIRREL	WTAP
MSN	NDEL1	F13A1	SNAI1	SRGN	WWTR1	DPYSL2	RHOB	CSGALNACT2	ZFP36L1	SERPINB8
FAM49A	SERTAD2	CXCL2	CD300E	JUN	FPR2	GPR135	RNF19B	AFAP1	CCL2	DCLRE1B
TMEM49	DUSP1	DUSP14	KIAA1949	PADI4	TNFSF18	CD209	MLKL	LATS2	ADAM17	NUPL1
IFFO2	POLD3	S100A12	S100A9	EHBP1L1	SLC15A4	SMARCAL1	FPR1	ACTN1	ARID5B	EPB41L2
PPP1R15B	AMPD2	SAP130	RIOK1	EMP1	UTP6	SEC23A	OSMR	ARID5A	RASSF3	CHSY1
MYD88	DLC1	MAP4K4	C5AR1	ADAMTS4	SEMA4C	LMNA	PRPF38A	POLR2D	PSMD11	SPRY1
NRP1	VIM	PLEKHO2	ZEB2	GPR183	KCTD20	RASSF2	ASAP1	AMMECR1L	WDR47	MSH6
GNA13	PTPRE	MYOF	CFLAR	EPHA2	TNFAIP3	APOBEC3A	C1orf216	GNB1	IFNAR2	TCP11L1
RAD51L3	KLF10	LRRC59	BAZ1A	SP110	PHLDB1	JDP2	CAP1	NR4A3	MRC1	PLAUR
FILIP1L	NRP2	CEP170L	KLF4	CD97	BYSL	MPZL1	S100A8	ARNT	HIVEP2	STEAP4
RNF217	ISG20L2	GNAI3	EGFLAM	ADPRH	CRISPLD2	DCUN1D3	PPM1D	IL1B	CEP170	HEATR1
ETS1	STK10	RAI14	FHL2	KALRN	TPM3	PDE4B	MTERFD2	CHST11	CRLF3	PER2
PHACTR1	CTTNBP2NL	ZRANB3	MCTP1	TRAM2	NFKBID	PELI1	TUBA1A	COTL1	PLXND1	P2RY8
ATP8B2	SLFN11	ARL4C	GPATCH2	CASP10	PLEKHG5	RNASE2	IL2RA	C10orf54	STAT4	TET3
FAM110C	DDIT4	C10orf10	GMEB1	CCDC46	PIP4K2A	TNFRSF1A	PIK3R1	FOS	CAMK4	C6orf145

PRPS1	USP37	SRF	RILPL2	CFH	HTR6	SLC2A14	PGM3	BCL3	NCL	NR1H4
GON4L	FCGR2A	RLF	FTO	SFRS7	EIF2C1	LOC284441	PMP22	SFPQ	SH2B3	PIP5K1A
C1orf107	LILRB2	TNFRSF10A	NFKB2	GNAI2	ELMO1	IRAK3	PRICKLE2	CASP5	ETV3	STARD13
BCL9L	PPRC1	SLC2A3	CCDC88A	ODF2	LDLRAD3	TNFRSF1B	OSM	LHX8	IL7R	TP53BP2
UCK2	LMCD1	SLC2A1	LILRA5	KPNB1	CSF2RB	CSDAP1	SIRPB1	SUPT7L	CMIP	LIPN
ABCB6	USP18	SHC1	NLRP3	PLK2	PDIA6	KCMF1	SERPINB9	RALB	CEBPD	MAT2A
WDR43	SELL	EHD2	SLC25A43	DYNC1I1	IFI16	CCL4L2	TUBGCP3	CLEC4D	AK3L1	DISC1
PROS1	RGS9	CCRN4L	ZNF281	BTBD19	TRIP12	MSL1	MTCP1	PANX1	DENND5A	C1S
FOSB	RBM8A	PHC2	C6orf150	ARRB1	ATF7	BGN	RGL1	AOAH	ITPRIP	EMR1
FCGR3B	SH3BP5	TRPC4	H6PD	TMEM194B	TRIM9	ARPC2	IL10	ENO1	SF3A3	ARRDC3
S100PBP	SKIL	S1PR3	C3orf59	C9orf21	LYAR	HNRNPA3	WDFY2	EIF4A3	HCLS1	JUB
CLEC4C	ZNF643	C1R	SUPT6H	SLC13A5	TTC27	FAM38A	TUBB	CACNA2D1	BHLHE41	WDR26
SHROOM4	TAF12	DDX21	SAMSN1	TRIM5	CASP7	GPR176	INVS	CBLB	FAM102B	ALDH18A1
MMP14	A2M	EGR1	ARHGAP29	VSTM1	NRBP1	SLC16A3	WEE1	C13orf18	AGTPBP1	PCBP1
DUSP12	DYRK3	YEATS2	SMAP2	C10orf119	IPO9	ACBD3	ULBP2	OTUD3	ITGB1	CALD1
CEACAM3	APOBEC3C	SH3PXD2B	ADAP2	MTF1	SRGAP2	GLIPR1	LOC643837	CASP1	LYN	EXOSC10
TNFRSF10D	DDX3X	KCNK3	DAXX	HEATR5B	AG2	LIF	GTPBP4	LCP2	UGGT1	LRRC42
TSR1	ELK3	ZC3H12A	PDK1	LRP1	DNAJC10	TAF5L	CASP4	EPC1	FBXO42	FPR3
CXorf21	GLIPR2	NOTCH2	SULT1B1	HNRNPU	RBMS1	NFKBIA	RPS6KC1	YY1AP1	ZCCHC24	RIPK1
ATP2B4	PDIA5	CHIC2	CXCL3	DEDD	MAP7D2	HSPA14	IPMK	IL13RA2	RNASE1	MKNK1
SLC11A1	NNMT	GLT25D1	SFRS4	CLEC4A	MESDC1	FCHSD2	WDFY1	PPT1	TEAD4	TTLL4
FAM55C	PTP4A1	ARHGDIB	SIRPA	ELL2	TMCO7	SIRPB2	EMILIN2	CXCR2	JMJD6	COL12A1
ZNF267	KCTD11	CSF1R	NBPF10	MID1	PLEK	MALT1	LRRC37B2	STC1	TFPI	FLJ36031
NONO	ANXA5	ZNF496	FEB21	IRF2BP2	MS4A6E	C1orf183	TTC28	CCNYL1	CD86	RFX3
GBP1	CLASP1	HK3	PCDHGC3	SAMHD1	PDLIM1	C5orf58	PBX2	LBR	UBC	FUT11
C2orf86	SF3B1	CD53	ATP13A3	GPLD1	ETS2	IRS4	MNDA	FGD5	MSRB3	TPM4
NOLC1	FOLR2	SFXN3	SYDE1	ZHX2	KIF3C	GALC	EHD3	THRAP3	EML4	LINGO1
HNRNPH1	ADCY3	PPP1R3B	FSTL3	C19orf38	STAB1	B3GNT5	LRRC32	ACLY	KRT75	MFSD11

TLR6	NECAP2	TTLL7	ENTPD7	SSR3	FADS1	BAT2L2	REL	MAP3K14	ITGA5	ADAM19
GPR161	CFHR1	MMP8	ZNF462	SLC6A5	VEZT	FCN1	FADS3	OAS3	CCNL1	LCP1
TYMP	GPR4	SP100	PUS7	SPEN	RSU1	TTC13	AATF	NOS2	LIX1L	CCL8
BICD1	CD33	SLC24A4	TRPV2	LILRB1	EMR2	TRNAU1AP	RAD51L1	AXL	EMR3	RIMKLA
ABCF1	CD14	RASGRP2	TAF11	EIF2C3	SGK269	SAP30	MYO1B	ZYX	MYLK	HELB
FAM20A	CSTT	CD300C	UGDH	LEPRE1	KCTD3	ZNF597	TRAF1	ZNF830	S100A7	ELK4
LIPI	PLEKHA2	EIF2S3	FOXC2	CASP8	SKI	THOC5	KLRG1	RFFL	CD226	KLHL33
HDGF	LAIR1	MAP1D	NES	FAM119B	IL6R	CEACAM4	SACS	BTN2A1	DCBLD1	KDM6B
MPEG1	C3orf64	PREX1	CD69	CCDC102B	GFRAL	DAAM2	TNKS1BP1	WDR75	KCNJ8	ACTR3
YBX1	PTRF	PNRC1	PLEKHG2	TMEM2	PDCD1LG2	MAP2K3	URB2	C13orf33	PLEKHM3	SLC30A1
MAF	MS4A6A	FAM129A	WDR33	SPRY4	IFNGR2	SLC16A1	NRAS	NAMPT	BARD1	TRIM38
CD68	TMEM217	GUCY1A2	APLF	ZSWIM2	HAT1	HNRNPC	ZBTB2	BMP2K	VSIG4	CCL7
C13orf29	CD200	DENND3	GPR84	PLXDC2	NFATC1	GIT2	ACTN3	EHBP1	NAV1	BLMH
SLC43A3	CREM	FUT4	TRDMT1	SEL1L3	P2RX1	C6orf114	IL4I1	CXCR4	KIF2A	IL1R1
BBS9	TNFSF11	SOX7	FOSL1	HIP1	CALCOCO2	DHX9	FYN	LOC100134259	F2R	MT1X
SPSB1	IRAK2	KDM3A	AGAP1	NLRC4	IL24	MYO1G	TNFAIP1	SLCO2B1	COL27A1	CD4
TTC4	CHD1	USP31	HNRNPR	ITPRIPL1	KIAA0090	SRBD1	FAM26E	CDH11	CHSY3	ST3GAL2
MDC 1.00	GPN1	PARP1	DAB2	C1RL	KDM5B	APOLD1	CAPZA1	PAG1	GRK5	DHX57
SDCCAG8	SPARC	SLC1A4	SIGLEC5	FHL3	ATF2	R3HDM1	CR1	RUNX2	LOC283663	NIN
PGM2L1	RASGRP4	PRKCB	LOC100131551	WASF2	SMC6	DYNC1I2	DSCC1	STK17B	LPGAT1	GRRP1
DDX5	RELB	IRF9	SLC25A37	LY6G6C	GTPBP2	FSTL1	EIF2B4	TRA2B	CLEC7A	NRIP1
ARL5B	HMOX1	C15orf39	ADAMTS2	ENAH	RNF19A	TNFSF13B	CSDA	BCL6B	MBD5	DDX50
HDX	RNF24	YME1L1	C12orf68	TMEM106A	TMEM22	JAG1	RPH3A	AVEN	PLEKHM2	OLFML2B
TXNDC12	COL4A2	WIPF2	YWHAG	TTC21B	UBE2Z	CDC42EP3	PDE3A	UBTD2	BMP1	EPAS1
NCKAP1L	GUCY1B3	INSIG2	SLC4A1AP	RFTN1	CD5	OSBPL11	RASAL2	PPP4R1L	LOH3CR2A	LILRA1
RGS2	TARDBP	GPCPD1	LONRF3	RBMXL1	CASC3	PLEKHG1	C3	GYPC	HCK	CDC42EP4
TYMS	FZD5	LOC282997	SIGLEC9	POLR3C	FKBP7	TLR1	MAP3K2	CXCR1	TGFB1	PCDHAC2
CEP135	IDH1	APOL3	ZMYM4	DYSF	VANGL1	SWAP70	TAF13	MCAM	IER3	EGLN3

MS4A4A	FNDC3B	ARHGEF10	BCORL1	STAMBP	CDK12	PDCD11	NUP85	ARHGAP22	RPS6KB1	CCL19
CDK2	LRRC33	STX11	SNRNP40	RNASE4	C14orf149	WDR35	FHOD1	CHST15	PDGFB	RASSF5
KATNAL1	STK38	GYPE	ALS2	XPNPEP1	PPP3R1	RASAL3	LHFPL2	BNIP2	TULP3	TMEM173
MAML2	IER5	SIGLEC10	VAMP3	NOL3	SOCS5	BCL2A1	MAP3K12	CCR1	LZTS1	RCSD1
DOCK2	ZNF318	KHDRBS1	CHEK2	MTHFR	AIF1	ZNF207	FRMD4B	RPIA	AMDHD1	CSDE1
EHD4	P4HA2	ZCCHC2	TTYH3	CYBB	PARP14	SERTAD1	DHX8	SYNJ2	PDLIM3	SOD2
ARHGAP30	XRCC5	PRDM1	UBE2J1	PANK2	IL1RAP	RQCD1	HNRNPA3P1	KIAA0146	KLF2	CD300LB
KRT32	C17orf107	C2orf48	SIGLEC12	NBPF9	PNMA2	SAMD8	CD1D	CYP7A1	C3AR1	PML
GAS2L3	ETV6	NFYA	C6orf204	RECQL	OGFRL1	SLIT3	SP140L	TTYH2	RNF145	VCAM1
FBXL7	MCTP2	ARNTL2	ARHGEF2	KSR1	KLF8	CEP164	TRIM34	SQRDL	CD93	ECD
TMEM67	CGREF1	ENTPD1	FAM57A	ZNF438	FAM38B	ERCC3	PTPRM	CD163	SLFN5	POLR1B
AGFG1	NFAM1	HPS3	FKBP1A	P2RY6	FFAR3	NBPF14	ITPKC	CLEC2B	TMEM218	MYO1F
ANTXR2	ARL13B	C1orf198	RPA1	PEA15	TRIM6	FEZ1	CSF3R	UIMC1	RAP2A	DCHS1
SEPT43	APAF1	RPGRIP1L	IL13	DKK 3.00	DLX5	ZNHIT6	C1orf55	FOXP3	FKBP10	KCNE4
PUS10	THADA	LAPTM5	GIMAP5	RHO	ACTR2	LAMC1	EFTUD2	TBCCD1	GRAP2	NEK6
THG1L	DNAJC18	BNIP3L	METTL13	TLR8	SMOX	ZC3HAV1L	AP4B1	PTPN18	ENPEP	DIP2B
ME1	CNRIP1	SSTR2	FMNL1	HEG1	KBTBD11	TNFSF8	PLXNC1	MSH2	LDHAL6B	ARHGAP10
C2orf29	IRF1	PDGFRB	PALMD	KIAA1462	NUP153	COL6A3	VASH1	PARVA	DUSP11	SP3
PLOD1	DBH	RXFP1	GNL1	NPHP3	C10orf128	NID1	METAP2	RNF128	APBB1IP	INPP5B
FLI1	CENPJ	FCGR1C	INPP5D	HPCAL1	ERRFI1	C1QC	OBFC2A	MED8	SEC14L1	MYOCD
DOK2	ABLIM3	KIF21B	ORC2L	KLHL20	HES1	SH3KBP1	EDEM1	GEN1	RPGR	GAB3
C21orf63	RAB9B	C9orf47	POLR1E	C1QB	TICAM1	ZCCHC11	CDKN1A	MGAT2	DAPP1	BIRC3
ADRBK2	PTPN12	HK2	PLOD2	CWC25	CRK	CXCR7	PPP1R16B	SH3GL1	ACSM4	CLIC2
TUBB6	GEMIN4	RBM17	RASA3	LYVE1	LOC145783	LDHA	DOCK1	IWS1	CDC27	DDX26B
C1orf38	C1orf112	HECW2	VAV1	NUFIP2	DZIP1L	FES	NBPF15	HSPB8	UGCG	MS4A1
SPOP	FAM70B	RSC1A1	PPP1R9B	RAB3IL1	ARSB	RNASE6	TMEM71	IGSF21	NUP62	LRRFIP1
APOBEC3F	NAP1L1	NFE2	CTGF	ANKRD6	SEMA5B	CSF1	FCGR1B	CEBPB	COL4A1	FUBP1
CD72	SDC3	HOXA4	DSE	RCN1	FAM104A	C17orf87	RAB20	TMEM200A	EBI3	TNFAIP8L1

ZNF608	GGT5	EEF2K	TWIST2	ILF2	RARA	FCGR3A	PDLIM2	CCL23	YWHAQ	CXCL12
BRE	CACNA2D4	RNFT1	CEP110	EPHA3	RRM2	GBGT1	GGCX	FCAR	CD6	APLNR
AFARP1	LOC84856	PAK2	RBM15	IL18R1	RNF112	C1QA	RAB39	PIK3CD	IL21R	CNR2
MAP7D3	PAPOLG	NFKB1	RAB23	MSTO2P	DHX33	TMEM26	GPR132	PPPDE1	NFATC3	PHC1
KPNA2	SFT2D2	COL5A2	PRKD3	PMS1	KCNA6	C19orf59	IFITM3	AP3S1	SLC39A10	CREG1
PHACTR4	IKZF1	HVCN1	CYFIP1	C2orf49	ATXN1	CDC42SE1	PEX13	NOL9	CALHM2	OR5V1
FRMD4A	HEYL	COL15A1	DUSP22	ARHGAP25	PSMB2	HELZ	P2RX7	ADORA2A	HDAC3	SLAMF1
PLSCR1	CDH2	AFAP1L2	LOC644538	NTF3	FN1	DYRK2	ZNF641	TAL1	C9orf96	KCNJ5
CSRNP2	CCDC88C	RNF125	GPR141	ELOVL5	FCGR1A	PIK3CG	NAIP	STX2	PEAR1	IL10RB
CD80	TESK2	KERA	WSB1	FMO3	GIMAP4	GRASP	AHDC1	IFI44	SLC26A10	MIDN
ZNF395	IL10RA	DAPK1	MAPK14	C4orf47	PMEPA1	SPRY2	PTENP1	KBTBD2	TSPAN18	C7orf68
EVI2B	VRK2	FAIM3	HPS5	C1orf162	SLC7A5	C17orf85	SHMT2	RHOJ	LAMC3	CLSTN3
ALPK2	RABGAP1L	USP48	WAS	CMKLR1	ASCC1	LTA4H	HNRNPH3	THUMPD2	ITGAM	SLAMF8
MDM2	PIK3C2B	TGFB3	ANKRD40	SETDB1	C1orf113	PLSCR4	MTF2	FCER1G	SLFN12	ANKZF1
PHKA2	HEPH	LIG3	GAL3ST4	COPA	CTNNB1	SYNE1	ABI3	TNFAIP8	SFRS3	NID2
CSRNP1	BCL10	FGD6	PRIMA1	IL6ST	STX4	MCM6	DDB2	TLN1	ERG	HLA-DRA
WDR46	ANTXR1	CD28	NEDD9	WARS	ENPP3	ENG	CEP68	S100A16	SERBP1	MFGE8
RHBDF2	DCDC1	PCDHB3	TMEM14E	AMICA1	PKD1L1	KIAA0802	PCDH12	GPR124	MOV10	MSTO1
SNRNP200	CDC5L	SLCO3A1	TGFA	XIRP1	SYNPO2	PSTPIP2	CHML	RPL23A	CCDC18	BATF3
LMNB1	TDG	RNF149	TXLNG	DDX42	ALPL	SPAST	RRAGA	SYT11	PLAGL1	GNA15
HACE1	TPCN2	VEGFA	GGTA1	MTRR	SFRS2	LOC257358	C1QTNF6	OASL	RNF115	STEAP1
SH3GLB1	NEDD4	GMFG	RAC2	VNN 3.00	ACAP1	CELSR1	ARHGAP15	RNF122	PPM1F	ZNF25
MPP3	C18orf1	HLA-DMA	MICAL2	IKBIP	YTHDF2	GNG2	SLA	ZNF487	SELPLG	JAK3
RNF2	SSRP1	SERPINE1	CYTH4	MAP3K13	PER1	ZNF143	ALDH9A1	SHANK3	TNFRSF8	NOTCH3
GEMIN5	RASIP1	MAP3K8	INF2	RND1	FERMT3	CKLF	GCLM	FBXO45	LAMA4	MICAL3
NLRP1	NOP56	ABHD12B	LMLN	TRIM14	PARD3	SLC41A1	PLIN3	IL18BP	MARVELD1	PFKP
FBLIM1	PNO1	PIK3R5	SLC5A4	CDKN2C	MSR1	MT1B	FRRS1	TFE3	GJC1	SLC39A1
PIP4K2B	GLIS3	ADARB1	DOK1	CCL4	IFITM1	FOXL1	PLCG1	ANGPTL4	PXDN	PRUNE2

IL16	DPRXP4	MED1	IPO4	PCSK6	PRCC	RUFY4	OTUD7B	MAOB	CBL	SOX13
FRMD8	DOK3	S100Z	SDCBP	GLI3	DHX16	ZNFX1	NSMAF	CD84	HSPG2	TMEM131
ADAR	GFOD1	NFKBIZ	TM4SF1	P4HB	PDE2A	RRAD	ECM2	DNTTIP2	LTV1	DOK6
TBX21	CCR8	PITPNC1	TAF15	TCF21	SERPINB2	IL7	SIGLEC14	C10orf25	LRRK1	ANG
NME7	SNX32	KCTD17	VCL	HAPLN3	RNPEP	FAM43A	CAPZB	CBFA2T3	HSPA5	SNX33
CECR6	EML3	ARGFXP2	PLD2	ABCA1	RRN3P2	PLSCR3	C14orf49	PPBP	AP2A1	IFNGR1
KLRAQ1	ACAD11	LHFP	FARSB	SH3PXD2A	OAS2	SSR1	AQP9	LOC100129034	PON2	POLR3GL
SEC24D	FAM89A	CCDC47	CLTC	LIPA	HERC4	ARL6IP6	CD163L1	LYZ	DIAPH2	CASS4
FUT7	TXNDC5	IL15RA	ITGB1BP2	TLR2	ARMC2	BASP1	SPIB	PCDHB16	GDI2	C17orf71
AATK	GFOD2	MMP19	PLCB2	CCL16	CTSL1	RTTN	TEX10	HAL	TMEM133	CLK1
FAF2	CWC22	ERCC5	TRIP10	SCML4	ROBO1	CXCR5	C5orf13	LHX6	CFHR4	STAT2
ADAMTS7	TRIM55	FSCN1	SELP	C1orf77	ATF3	GPR21	DEF6	GIMAP8	VKORC1	CCDC55
UBE4B	CYSLTR1	UTP11L	P4HA1	ZFP36L2	ADAM8	XPR1	P2RY14	FLJ10213	SLCO1C1	TIAM1
PASK	APOBEC3D	CP110	CSPG4	RNU4ATAC	GMIP	SFRS13B	HNRNPM	QSER1	DDX20	SLC41A2
ROBO4	BZW1	ARL17A	CNST	ORC1L	NPL	SULF1	ZNF638	NKIRAS2	PTAFR	ATOH1
CXorf57	BRCA2	TANC2	APPBP2	THBS3	PLBD2	CDON	LILRB3	ARHGEF1	ITGB2	GJB2
ACPL2	HDLBP	MLC1	MNR54	ACTA2	ISG20	DARS	MMP25	NFE2L3	NMI	ADAM28
WDR19	RPS27A	ADAM15	IFT172	TIAL1	MXI1	RCC2	GPSM3	MGST1	DDI2	EIF4E2
COL23A1	F2RL3	ING1	S1PR1	BST1	RBMS2	SLC12A4	CRKL	PRR16	WDR12	MCM5
MT1A	GALNT6	NCRNA00201	NHEJ1	IGFBP4	LOC654433	CCIN	SLC16A6	FAT4	LEPROT	LOC100233209
MASTL	WDFY4	FXYD5	POPDC2	IQSEC3	ITK	TCF4	C2orf77	ST7L	HAVCR2	FAP
RUNX1	CD109	SEC61A2	EIF2AK2	TAS2R40	TMC8	ARL 10.00	GNL2	FAR2	ATAD2B	C1orf200
KLRB1	IFT81	PSRC1	LOC154761	LASP1	LGALS9	SLC10A6	PLA2R1	CD300A	DDOST	SORBS3
ZFPM2	MPI	NLRC3	RIN3	SRP68	KTELC1	CDKAL1	SLC16A2	C6orf167	KATNA1	PRIC285
SH3RF3	MYO9B	STAG1	DDR2	OR51B6	TCN2	NASP	RGS1	C20orf141	SPAG9	BTBD10
CTSS	P2RY1	SDS	PTEN	CARD16	PCGF6	VNN 2.00	FAM107A	ZBTB11	LOC595101	C17orf46
HIVEP3	NCK1	GNGT2	ENO2	EDA2R	TAF1A	DGKH	CHD1L	KIAA1274	ANKRD53	HLA-E
GBA	MIA3	DOCK7	C17orf68	JAK1	AOC3	OLFML2A	USP13	CLC	EPSTI1	EPRS

PLEKHA1	LUZP4	LOC729467	ZC3HAV1	CACNA1C	SNX20	CLK2	EVI2A	C18orf45	MMADHC	ARPC5
HLA-DMB	CDCA2	ZNF239	GNB4	GLB1L	GALNT2	HMX3	TRAF3IP2	HFE	PROCR	SLC28A1
IRF8	RIF1	ITSN1	LTB4R2	DOCK9	RARG	PNP	KIF18A	RNASE10	ALDOC	GTF2B
PLCB1	LOC84740	NUP107	GJA4	FXR1	C14orf64	ITGA8	ANAPC1	NCF4	GIMAP1	HNRNPA1L2
TCP1	TGM2	RAPGEF5	CNPY4	CORO1A	PAK1IP1	ZCCHC6	NCSTN	RALGDS	TLR5	CDK19
TIPRL	C2CD2	LOC145837	PTPRC	TMCC1	GALNT12	TIPARP	SNHG3-RCC1	BRCA1	TMEM18	GJA1
NUAK1	NOTCH1	ABCC3	CBWD3	KLHL6	IFITM2	HTR2B	ITGAL	ARHGEF15	GART	BTAF1
GTF2H1	LRRC70	ASNSD1	CDCA7	C12orf24	FCGR2C	SIGLEC7	PGBD3	SEC24C	C11orf9	CD40LG
SERINC3	TGM3	GPX8	FBLN5	IGFBP3	TAP2	POLG2	TAGLN2	COL5A1	FIGN	HNRNPK
CDH13	SASH1	DDX18	LILRA6	CDK18	KDM1B	MMRN2	DNAJC22	GP5	ANKRD36	KIFC3
DGKD	CLIC1	FAIM2	GRM7	CNTNAP1	ANKLE2	DONSON	SCN3A	MATN1	DCTN1	PRPF18
TTC31	TPM1	SYNPO	DOCK11	PITPNM2	S100A10	FJX1	CD302	MCM9	CIDEB	ZC3H11A
PDE1B	PFKFB4	SIPA1	ADCY4	MAP4K1	RAB8B	C15orf51	CPB2	TM4SF18	TUBD1	CERKL
WDR54	KIAA1919	NHEDC2	SNAPC1	PIK3R6	COL3A1	CHFR	PARVG	HDAC2	STOX2	METTL8
GGNBP2	LOC283314	SMARCE1	MCFD2	IL18RAP	PRDX4	ANXA2P2	RPL23AP82	USP34	PPP2R5D	S100B
CLCF1	CHRM3	SMURF2	SLC30A6	MEIS2	THBS2	ERN 1.00	NOC3L	ADRA1B	TRIT1	ABCC9
SYNCRIP	ETF1	ZEB1	ODF2L	E2F6	NUP188	CCNH	MALAT1	CNTNAP3	PRR5L	EPHB1
SEC63	PLEKHO1	NDST2	MLL5	KLHL5	CCL3L1	POGZ	OLR1	EPHB4	LANCL1	HLA-DRB1
ASPH	EPC2	RBBP4	RNASE3	PCDHGC5	IFT52	FBXO28	SLC35D2	BID	FGD2	C4orf31
ERMAP	FMNL3	STS	FAM178A	NOD2	ARAP3	ZBED1	RFX8	HSP90B1	TBXAS1	TMEM50A
ACVRL1	NHLRC3	NUP98	RBM26	BMS1	ALPK1	PCDHGB7	SNORA8	PRPF38B	PRPF40A	GIPC3
HKDC1	LY6G5B	PLD1	QSOX2	CARD9	CFP	S1PR4	OR2W3	MORN1	TRIP13	SLC22A16
FRZB	TRIM22	FAM46A	CLEC5A	MEX3B	SYTL2	SLC7A1	C3orf58	HECTD2	CCDC52	FBXO5
C1orf115	GPR180	MAP3K7	IL12RB1	PIAS3	ATM	ARHGAP20	C17orf60	CD99	TM4SF4	RASGRP3
GEM	STAT1	FBLN2	C7orf58	PCA3	ARHGAP42	AKAP12	OCLM	TCEB3	PCDHB10	FCRL1
ABCC1	SASH3	RBBP9	TP53	LOC729603	CMAH	PHF20	LRRC8C	CIB4	PLIN2	SOX17
INHBB	EIF2C4	NBAS	RBMS3	C1orf91	AGAP2	AIM1	PRTN3	VASP	C6orf186	MCM10
DYNC1LI2	TEK	TEX9	RHBDL2	TNFSF9	PAAF1	WASF1	CSTF2T	INO80D	GALNTL4	KIAA0240

HTR7	HLA-DQA1	ZNF286A	ARL 11.00	MKI67IP	ADIPOR1	SSTR3	STARD3NL	TLR7	MPO	SFMBT2
AVPR1B	CIRH1A	BMS1P4	CLEC18B	GMCL1	NDRG1	DOCK5	GORAB	SPTA1	ZNF142	EMID1
PCGF1	POLR3A	C22orf45	CYP11B1	BTG2	XYLB	BEND4	C1orf170	AGAP5	PLK1S1	BNIP3
ATP11C	CMTM5	RHBDD1	HAS2	GLT1D1	FGR	CDH24	CCL11	GTF2H4	NFASC	LRFN1
NOTCH4	DNAJC7	RAP2B	HLA-DPA1	C14orf174	C3orf70	LOC400804	PTPN22	FAM115C	LY9	BLK
SLC1A3	SCN9A	H3F3C	NCF2	IKBKE	MGAT3	GPR133	ABCA12	CEBPZ	HLA-DPB1	TSHZ1
SLC36A1	RPL22	EGOT	MYG31	SMC3	CDC7	PPFIBP1	MC2R	HK1	C11orf21	CNPY3
CCL20	ATP11A	MGAT4A	MARCKS	GM2A	TNFRSF25	CCDC99	C6orf170	DUSP7	ANXA2	NFATC2
NR2E1	HNRNPF	ZNF512	MYO3A	ZBTB25	TBCE	ACR	XIST	CLEC14A	CCDC75	LDOC1L
BTG1	FAM78A	LPXN	ATG12	CCR4	TAF5	ZMYM2	C14orf139	ILDR2	CD34	PLAU
SLC5A1	NR3C1	SIRPD	LOC541471	ZWINT	CPNE5	TRIM47	NUDT5	C1orf124	LOC283267	CFDP1
FAM131B	LPHN2	CARD8	GRIN2B	FHL5	TNKS2	WARS2	ATP6V1B2	PSMD1	UPB1	C4A
CLEC1A	TIE1	DNMT1	PCDHGA9	CCDC104	SSBP2	STX6	ROCK2	FTSJ3	PKNOX2	DHH
SMYD3	CXorf36	ARHGAP17	LAYN	DEM 1.00	PLEKHA5	TAC4	TSPAN2	FBN1	VAT1	ARHGAP19
QKI	FAM160A2	LUM	MAP7D1	BDNF	SPATA6	FAM136A	SLC45A1	ATF6	RGS19	CDH5
ABI2	SPATA1	GLI2	CDK11A	BTF3L4	STARD3	PODXL	GIMAP7	RAB31	LOC400927	DNAJB4
GCA	PTGER4	ANXA6	FOLH1	AKR1C3	PTGDR	ENC1	RAB3D	KHNYN	OR2B11	HS2ST1
SGOL2	DZIP1	ZSWIM5	F9	DHX40	LSM 11.00	RLTPR	BCKDK	HSD17B7	THSD1	THBD
SLC9A9	MSC	RNF144B	ARRDC4	FAM21A	ITGA4	SIGLEC1	OLFML1	GABRP	EEF1B2	TNFSF14
SPG20	ATAD5	CREB1	LTB4R	GIMAP6	IQGAP1	SNIP1	PARP15	PRELID2	ZNF202	LILRB5
SAMD3	NIP7									
**Coexpressed genes with Myc in KIPAN (stage3)**										
NFIL3	PNRC1	ZFP36	WDR43	PPRC1	KLF6	C10orf119	SLC2A14	SNAPC1	NUDCD1	SLC2A3
DDX21	MCL1	UGCG	SOCS3	ADAMTS4	NAMPT	POLR1B	RIOK1	PLK2	KIAA0146	NOLC1
PTP4A1	PUS7	JUNB	ZFP36L1	NUP35	EDIL3	RNF139	FLJ36031	FOS	SMNDC1	LDLRAD3
EGR1	RPL7	TMEM185B	PPM1D	EIF3E	PFKFB3	NCL	BAZ1A	ELOVL5	DUSP1	ZNF143
ZC3H15	MIPOL1	TAF4B	IL1RAP	STEAP4	NUP98	NFYA	PNO1	RHOB	PRPS1	ASNSD1
ADM	HNRNPF	CCRN4L	DDX18	YRDC	C9orf21	RBMS1	EREG	JUB	EEF1B2	TEX10

ARL6IP6	ZNF207	HAT1	TMEM49	TCEA1	EIF3J	MED30	USP37	NOC3L	DKFZP434K028	ZFAND1
STAT3	CALCR	PANX1	WDYHV1	WTAP	PATL1	C7orf68	GNA13	PDIA6	RNASE4	EIF4A3
SERPINE1	C1orf107	TAF1D	TRIB1	HSPA14	POLR2D	APEX1	CEBPD	PABPC3	GTF3C3	PPP1R15B
SIRT1	RPS12	MSH6	PABPC1	EIF2S3	DDX50	ARL5B	NCRNA00188	TMEM67	SEH1L	ZRANB3
RNF149	ADAM17	ICAM1	TIAL1	KCNJ8	SNORD1C	KLF10	PCDHGB2	SLC16A2	ADAMTS1	CYR61
AMMECR1L	NT5E	E2F5	NFKBIA	RBM12	TGFA	HNRNPA3	SFRS3	CSDAP1	WDR75	EIF2C2
UTP18	UBA2	LOC100133669	SFRS13B	B3GNT5	NFE2L2	MMADHC	MALT1	ATF2	MIR17HG	SPRY1
HSPA5	CCT4	ANKRD40	TNFRSF10A	GPN1	NCOA7	ATP13A3	TIMM9	SYNCRIP	LOC100127888	NOP58
TNFAIP3	ZHX2	RPS13	TTC27	OSMR	EIF3H	ORM1	FOSL1	LDHAL6B	C2orf49	SF3B1
URB2	YWHAQ	XRCC5	GALC	PAK1IP1	FOSL2	RND3	NETO2	NSMAF	RBM7	SEPT12
WDR12	CDC5L	PTPN12	DYNC1I2	CEBPZ	EIF4A1	YWHAZ	CCL20	C20orf199	METAP2	ABCE1
FASTKD2	ATF3	TCP11L1	FOXC2	SLC35A1	ADAMTS9	ANG	TMEM209	CCT2	TXLNG	TFPI
OLA1	PRPF40A	RPL10A	HNRNPC	NUP153	GTPBP2	ASAP1	SSRP1	EGR3	ZNF318	SP3
EIF2S1	WEE1	CA1	TMEM194B	LOC100131551	STC1	HMGN4	S1PR3	SLFN11	GDI2	HPS3
GTPBP4	CXCL3	FAM49A	ZBTB2	SLC22A3	EPC2	UBXN4	BCL6	DUSP11	TSR1	TTC9C
RPL7L1	PLAC8	WIT1	ABCC9	RBM15	SLC39A10	RPS3A	HSD17B7	ORC2L	SUV39H2	FARSB
HES1	PM20D2	CREM	STAG1	AZIN1	ERO1L	KIN	PLSCR4	RBMX	MTDH	GEM
HSP90AB1	SLC25A32	ARMC10	HEATR1	SERTAD1	CSRNP1	LATS2	RBMXL1	ETS2	EXT2	CSE1L
TIMP4	BZW1	ALS2CR4	NMD3	SFRS7	LONRF3	USP49	ARID5B	MYCTB	SRGN	FHL2
LOC728190	YY1	CNIH	GEMIN4	MORF4L2	PPP1R3B	ZNHIT6	FAM119B	MLLT10	C1orf55	MMRN1
SLC39A6	OBFC2A	C9orf30	THBS1	ABI2	GART	EMP1	CLDND1	MKI67IP	PDCD11	ABL2
EEF1A1P9	HNRNPK	DIO3OS	DARS	RPL31	ACTL6A	C12orf11	P2RY14	PKNOX1	MID2	NUAK2
MAP4K4	C6orf72	MTHFD2	H3F3B	POLH	BTF3	HSP90AB2P	TMEM39A	RPS6KA5	MAD2L1BP	ZNF410
NKX3-1	IL7	C10orf18	PRMT5	CWC22	EXOC6	MTERFD2	MT1X	ETS1	SULT1B1	SDCBP
DKFZP434H168	SH3BP5	IL1B	HNRNPR	SNORA8	RPS6KB1	UNC50	NXT1	NHEDC2	ZNF830	PGM3
PPBP	DERL1	CHSY1	MTPAP	TOMM20	C2orf86	GTF2H1	IFITM5	CD2AP	HBEGF	ZNF281
DPAGT1	ECD	SLC43A3	SLC35F5							
**Coexpressed genes with Myc in KIPAN (stage4)**										

KLF6	NFIL3	KLF4	HNRNPH3	FOSL2	JUNB	MCL1	DUSP6	PFKFB3	WDR43	MTERFD2
C10orf119	DUSP1	ELK3	DDX21	NOLC1	ZFP36	TAF4B	ANKRD40	PPRC1	ARRDC3	HNRNPA3
SPRED2	CAMSAP1	NOC3L	TMC7	B3GALT1	B3GNT5	SEMA4C	CCRN4L	ZNF462	ITPRIP	ECD
INVS	PEAR1	EPAS1	AMMECR1L	STARD13	EIF4A3	RPS6KA5	ABI2	EMP1	STC1	PTP4A1
DUSP5	EPC2	SPRED3	ISG20L2	SMNDC1	TUBGCP3	C9orf21	SPAG9	EPHA2	ABL2	FAM110C
SPRY1	EXOSC2	C1orf107	JMJD6	RIOK1	NAB1	HNRPDL	RNF19B	PHF3	NPDC1	GTPBP4
PTPN12	LRRFIP1	MYO1B	DOCK5	RFX3	C16orf72	HNRNPU	ADCY3	SOCS3	WAC	ETS1
POLR1B	SH2B3	CNBP	ORC2L	TIAL1	URB2	TXLNG	GPR135	FOSB	RUFY2	MLLT10
LDLRAD3	EFNB2	ERLIN1	WDR47	CDC27	ZNF496	SIRT1	ZRANB3	ZFP36L1	ARHGAP29	GEMIN4
PPP3R1	SP3	TM4SF1	CALCOCO2	RBMX	UGCG	LOC100302650	SOX7	HNRNPK	SPRY4	LOC344595
FUBP1	CEP68	GPATCH2	TGFBR2	SLC2A14	NDEL1	METAP2	BHLHE40	DNTTIP2	DDX3X	CHD1
ARL6IP6	UPF2	DIAPH2	TNFAIP1	USP31	HBEGF	SERTAD2	RPS6KB1	CWC22	NRP1	ZCCHC2
SCN4B	SAMD8	ZEB1	EIF2C2	LOC440354	LRRC42	OTUD4	CPSF6	DDX42	TTLL11	C1orf55
CSGALNACT2	SLFN5	JOSD1	ZNF25	FAM119B	YME1L1	MFSD11	GRIK3	ASPH	TAF5L	NUP153
SMC3	ZNF143	RAPGEF5	CCDC46	KIAA0355	FOXC2	F2RL3	THRAP3	DDX5	SLCO2A1	PSMD11
THUMPD2	CDKN1A	SRGAP2	COIL	DYNC1I2	PCDHAC2	IL1RAP	FAM38B	UTP18	CDK17	NCRNA00120
C2orf86	SLC2A3	LATS2	EBF3	MBD5	HNRNPA3P1	CDNF	NEK5	SMURF2	LMNA	LOC641367
JMJD1C	BYSL	EPC1	DDX18	NAA25	GRB10	PGM2L1	ASAP1	ADAM17	RALB	HTR7P1
PNP	CRK	RGS9	SPRY2	KCNE4	DDX50	NCL	PGAP1	KLF10	BRSK2	HSPG2
DGKD	OR56B4	ZNF343	ATM32	TNFRSF10A	YEATS2	ROCK2	USP13	DHX33	SEC14L1	S1PR1
KHDRBS1	HEATR5B	CREB1	HERC4	KLF7	DEFB118	CNST	TARDBP	NOTCH1	SHROOM4	SLC26A10
GPN1	ANKRD27	C20orf112	GPATCH8	SWAP70	MCFD2	NR4A2	HNRNPF	VPS24	ZNF584	RPGR
CCDC55	LONRF3	H3F3B	SPEN	TCTE1	STAM	MAPK3	LIG3	LRIG2	MPP3	IL7
PCDH17	LOC286094	ETV3	SERINC3	CLIC2	CDKN2B	PCSK5	RAB3GAP1	PMS1	MCTP1	WDFY1
ZC3H15	PRPF38A	HEATR1	CST11	C17orf46	PRPF40A	OTUD3	RSL1D1	CREM	AGTPBP1	KCTD20
C9orf150	ACLY	HIVEP2	ALS2	TMEM22	CEBPZ	APOLD1	DOCK9	STAT3	CHMP1B	PRDM10
ZNF146	TGFA	ITGB1	PLEKHG1	CSRNP1	VPS26A	WWTR1	MAPK8	DCUN1D3	SFN	NUP98
TASP1	BMS1	SMC6	FBXO11	TCF23	ANO2	PAPL	SRP68	UBA2	TRY12	MAFG

MAP1D	JAG1	UTP6	ADAMTS1	LRRC70	RAPH1	IRF2BP2	RND1	NCK1	DLL4	VEZF1
PELI1	PPP2R2A	BZW1	CSDAP1	IL4R	ARHGAP17	MDGA2	PIBF1	ATF3	GPR161	LPHN2
THBS1	ANKMY1	RASA2	SNRK	DDIT4	FRZB	NOP58	RHOB	TEX10	TNFAIP8L1	DOCK1
C3orf64	GPR4	SKIL	MYADM	MOBP	PPIG	RARA	KCNE3	IL1RAPL1	C20orf117	FBXO45
PNO1	WDR3	C10orf10	ADM	ADAM15	USP37	RBM8A	GMCL1	TAF5	ZC3HAV1L	UBC
CCDC88A	RIF1	B4GALNT1	CHML	FBXO48	ABCG2	APLF	NFYA	SV2C	PLXND1	ARAP3
FEZ2	PPP2R2C	PEG10	AKT3	AP3M1	MSH6	PPARGC1B	ZNF507	DLC1	H3F3C	WAPAL
HNRNPR	GABRB3	PFKP	USP36	CDC73	THBD	BMX	MASTL	MAP4K4	SGK1	QSOX2
FOXD2	GMEB1	TMCO7	HES1	ZNHIT6	PCDHA7	LDB2	PLEKHA1	WEE1	ADAMTS5	ATP11B
EIF4A1	ANKAR	TACR1	RGS5	EGR1	MTAP	SFRS3	CCNYL1	EFTUD2	ATAD2B	KBTBD2
SNORD1C	ING1	C3orf36	UBE2Z	GDAP1	TTC27	TRDMT1	CDK12	STAMBP	LOC399815	DNMBP
OR2W3	CEP170	JUN	HOXD10	IL6R	FOS	CXCR7	FNDC3B	TNKS2	DOCK6	RPE
40057	HNRNPM	FLT1	PITPNC1	PHOSPHO2	ATG12	GRK5	SLC10A6	MSL1	ZNF500	HNM78
RQCD1	VEZT	GJC1	RBMS3	C10orf78	BCL6B	TUBD1	TGFB1	UIMC1	PHLDA1	ANAPC1
C18orf54	CLEC1A	PIK3C2B								

Table S5: List of coexpressed genes with Myc in KIRC , [KIRC=Kidney renal clear cell carcinoma]

**Coexpressed genes with Myc in KIRC (Normal)**										
CEBPB	RHOB	DCUN1D3	SPHK1	AHNAK2	NUMBL	TUBA1A	CDC42EP1	CITED4	KIAA1949	CXCL6
ANXA1	CSF1	BLK	ARL4C	PDLIM1	MEX3D	PODNL1	SYNC	RAC2	C1QTNF1	PEA15
FSTL3	AKAP12	RBM24	SERPINH1	PLEKHO2	AG2	SERPINE1	C6orf145	RAC3	HRH1	THBD
COL8A1	MMP14	DNAJB5	PLEKHO1	DARC	ZYX	BAI2	SPRY1	MTHFD2	HAPLN3	POU2F2
TM4SF4	CREB5	ZSWIM4	MAP3K12	CXCL2	SPSB1	CXCL1	MMP19	CD276	NLGN2	DLGAP1
PTPN1	MAGED4	PLD2	MMP2	LOXL1	TMEM217	TGFB1I1	SLCO5A1	FUT4	IER5	TNFRSF1A
SNAI1	C1R	TXLNB	IL4R	LOC100126784	MAGED4B	EXT1	DLGAP4	HEYL	PDLIM7	GPR37
WNT2B	RND3	MICAL1	FILIP1L	TTLL7	CHSY1	BMP2	KCNT2	SERPINB9	NFKBIA	ICAM1
ADAMTS4	PHF17	TIMP1	DTNA	MAP7D1	SYT11	SGK223	CPA4	C1S	KDELC1	BIRC3

GRASP	SPON2	UGCG	MICAL2	LIMD2	B4GALT5	NBL1	EHBP1L1	C6orf174	COL16A1	SLC27A3
IFFO2	CNTNAP1	ADAM8	NDEL1	C1orf38	CD40LG	CORO1C	LAT	SOX9	THBS1	ARID5A
NFIX	CMTM3	CRLF2	EIF4A1	TNFAIP2	PLK2	TLE1	RIN1	CCIN	RRAS	PPRC1
SCARF2	CHI3L2	BTN2A1	MEIS3	BMPER	CD97	CYP19A1	PXDN	LZTS1	ZDHHC18	CD44
VIM	TNFAIP3	BATF3	C9orf21	CFH	IGFBP6	RHOG	KRT86	MAMLD1	FADS3	RASA3
TMEM43	GDF5	CD69	IGFN1	TMC8	TGFB3	CDV3	TUBB3	CNR1	COL6A2	RELT
LOXL3	RCN3	CCL2	PIGR	AIM2	PLEKHA4	THBS3	TMEM233	TNFAIP8L3	LSP1	LMCD1
CSNK1E	MAP1LC3C	CACNA1I	FSTL1	VCAM1	ITGAX	CCL21	SRPX	IFI16	MICALL2	S1PR2
DOK1	RNF175	RELB	CXCL16	GNAI2	FSCN1	SH2B3	ARHGAP17	THBS2	TFE3	MEI1
DUSP10	ATP8B2	NGFR	GATA6	KIF7	SORBS2	GAB3	RHBDF2	RASGRP2	CDC42SE1	PLXNB3
LY6H	LOC100270710	RGS1	LRRN1	MYADM	C1QTNF6	SMAP2	PIK3R6	CD3E	SERPINF1	MAPK7
LIX1L	TWIST1	GPR183	FEZ1	SLC35C1	STEAP4	C11orf24	NLRP3	KLF16	GOLGA7B	ITK
EMILIN2	GLIPR2	AEBP1	STAC3	KLF2	IFITM3	GALNT5	C19orf30	C17orf60	FLJ41941	PVR
LRRC59	CD72	CCDC80	WAS	SBNO2	BATF	KIAA1045	CLEC4F	ICAM4	FAIM3	SDK1
MAP4K1	MTA2	CHIC2	COLQ	MAP7D3	IFITM2	TTC39C	PSTPIP1	DNAJB11	SFRP2	C6
NFKB2	RNF112	ARHGAP22	EPB41L2	FLJ16779	TSKS	CD37	KLF4	SLAMF6	ETS1	YWHAH
HSPB8	WNT1	PCOLCE	YPEL4	LY9	ETS2	C1orf162	IL7R	PLSCR3	C10orf10	HSPA7
C6orf150	CADPS	CXCR4	MLKL	LHFP	ACAP1	CTGF	FJX1	KBTBD2	CD6	RAB31
PRR24	ARHGAP1	FLNC	C9orf25	BTBD19	MIA	BEAN	ELN	KCTD4	LDLRAD3	PCDHB14
BTN2A2	DGKA	C13orf18	SHC1	GLDN	GDPD5	SEMA7A	ITGB3	RFTN1	C2CD4A	TUBB6
DOK2	HCST	PSTPIP2	TNFRSF1B	STX11	LGALS1	OSR1	FHL3	ANKRD35	CDH22	NINJ2
DLC1	SACS	PTRF	VSTM2A	CD74	KRTAP5-1	PTGIR	RASSF1	CNKSR2	LOC100130776	LATS2
BCL2A1	NFKBID	SDC3	SLA	TCF7	MC1R	SERPING1	LOC338651	PIWIL4	CD248	MED17
PHLDA1	FAM129A	SLC2A3	CORO6	HCLS1	EMP3	RALGDS	RHOU	MARVELD1	GRAMD1A	TMEM156
ACTN1	TMEM173	PRAM1	C3orf36	CADM3	CTSG	MAP4K4	SERPINB8	TMEM2	FUT7	SV2A
MDK	ADAM19	VENTX	ENAH	RXFP2	FERMT3	CARS	ISG20	CCR7	PARVG	ARHGAP15
RASSF2	EFEMP1	MXRA8	LILRB3	NFKB1	RARA	HSPB6	SAMD4A	GGTA1	RELL1	C11orf9
HP	OXTR	IL1R1	TPM1	OLFML2B	CCDC50	ADAMTS1	NLGN4X	NKX6-1	PDIA4	C1RL

CD5	ANKRD13B	PLAUR	OBFC2A	GFI1	REEP2	LCP2	SH3PXD2B	CNTN6	C2orf18	NFAM1
CXCL3	RASSF5	DAPK3	UNC5A	FLNA	MEX3C	DEF6	C1QA	LRRN4	SEMA4C	CCL4
ZAK	MT1A	ANGPTL7	FAM113B	COMP	CCDC88B	C2orf89	CCL14	COL1A1	PAPLN	C21orf7
DUSP14	GPC4	C8orf84	LBR	SLAMF1	DYRK2	NHEDC2	NLRP1	IL18BP	CYP21A2	NRP1
ITGA7	CD207	SLC2A14	P2RX6	OSCAR	ZNF831	INMT	TWF2	TRAT1	MEX3A	LRRC25
ENTHD1	NT5DC3	RHOH	POU2AF1	COL6A1	CACNB1	BDNF	FSHR	DTX3L	HTR7	B3GNT7
CYR61	CCL4L2	SH3BGRL3	RFX2	COL1A2	ITGA5	SLITRK5	FGR	GPR55	DTX2	GFPT2
AGAP2	EDN1	CD27	SULT1C4	TAGLN2	TREM1	PML	GPR171	KIF3C	GMIP	NEXN
IFNAR2	KCNH8	SELPLG	NPDC1	ZIC4	CSPG4	C1QTNF2	C1orf198	FAM198B	CCDC97	CFP
MAP1A	ARHGAP30	CORO7	AKIRIN2	FZD1	GRK5	CMA1	MMP11	SAMD14	CAP2	SLC24A4
ARMC4	TNXB	ALDH1L2	CCDC109B	KIAA0802	CORO1A	MYN65	PLAC9	MEF2D	FBLN7	BTK
CDH6	KIAA0748	PYGM	SEMA3A	UBE2I	HKDC1	PODN	BEST1	LAX1	TNIP3	SH2D5
IL1B	GPSM3	FIBIN	ANKRD53	RGS19	MFGE8	KLF7	CLEC10A	TBC1D10C	PTRH2	TNFSF13B
DACT1	CCL22	ATP2B4	KIAA1274	AMICA1	TIGIT	DPYSL3	DPEP2	SPRY4	PLK3	PLB1
ODF3B	BAZ1A	EBF1	IGFBP7	HAND2	NFKBIE	MCAM	HSP90B1	GPR176	RASAL3	BRSK1
KCNK7	PCDHGA5	PRICKLE1	C19orf35	JAK3	MAP1B	ITGAL	PALLD	SAMD3	ACAN	TIMP2
IL1F10	APOBEC3D	GPRIN1	PDZD4	MAP3K8	LOH3CR2A	TNFSF8	GNG8	CNRIP1	FMNL1	PLTP
SVIL	IKZF1	POLR3D	LRG1	TYMP	RIPK2	APBB1IP	IRAK2	TRPV2	TXNDC5	ETV6
DUSP8	FAM92B	FPR1	C2orf85	PRIC285	SCGN	ADAMTSL4	NELL2	NAV3	SETD8	SNAI3
CD3G	CLEC1A	ABCB4	ADAMTS2	SCG2	LAIR1	CCRL2	PITPNB	EFHD2	KLF6	UTP6
FAM102B	HSPB3	DCBLD2	FAM46A	ISLR	FNDC4	GRRP1	IFNGR1	WNT5B	CAMK2N2	CKAP4
SMARCD3	IL31RA	PTP4A3	SLC18A1	NT5E	TNFRSF13C	CYTIP	COL18A1	NFKBIZ	SHOX2	FAM150B
CELF2	SPEG	LILRB2	C16orf54	TAL1	IL21R	KCTD10	CLEC2B	MAN2B1	PTX3	PRRX1
ITPRIP	ASPHD1	TXNDC3	MSN	COL5A1	CSTA	NAPSB	SIRPG	CEACAM4	KCTD5	SFRP4
ZCCHC5	SPI1	CDH24	LEPRE1	HAVCR1	LRRN3	NAALADL1	IL10RA	TSPAN18	SAP30	WNT10B
PCDHGB7	ZBP1	BTLA	KCNA5	PTCH2	NID2	RBMS1	CMTM7	CLIC1	IFITM1	ARL4A
IDUA	FBXL12	STK10	FCHO1	MYBL1	SASH3	GAP43	PTPRCAP	CD300LF	RNASE6	IL34
PLEKHG2	MDFI	DOK3	DNM1	GNA15	CABYR	CLDND1	ZNF365	FHL1	CENPW	TAGAP

CACNA1A	CCR6	CTTNBP2NL	STXBP1	MSC	SMTN	PTPN12	TLN1	FHOD1	BIRC7	PHF19
PMP22	PLEKHN1	SOD2	MYO9B	RAB33A	APOBEC3F	YWHAG	ANTXR1	ARSI	PIK3R5	VAV1
LGALS9C	MESDC1	LAT2	STAT4	TCEAL7	C20orf103	MMP23B	CCDC140	AXL	KLF8	PLAC8
LONRF1	TNFSF9	CAV1	FXYD5	CHST7	LOC541471	JPH2	PTPRE	ZFPM2	RAB35	LEPREL2
CSF2RA	CD3D	GBP2	LILRA4	CDH23	KIF21B	TAGLN	PDGFRB	GMFG	STS	PCDHB18
BEND6	HDC	SPARC	ADCY7	TLR9	ZNF193	MYH9	NLRC5	CEACAM21	S100A16	CD300LB
IRF8	SGTB	SH2D2A	CEBPD	RRS1	ASB2	GPR84	KLHL6	GFRA1	LOC283050	GLIPR1
MEGF11	MIR155HG	FGFR1	OSMR	MED26	CD2	BBC3	CSGALNACT2	ARL 14.00	COL4A2	C12orf70
SEMA6A	ARHGEF1	PRKCDBP	NCF1	PLEK	CD151	NNMT	NRP2	ELK3	MGP	RRN3P2
EPN2	C10orf105	WIPF1	SMURF1	WDR82	CCL23	TNFSF18	SEL1L3	TP53BP2	C10orf54	GRK6
RASSF3	ARRDC5	MYOZ3	PTGFRN	KIAA1199	BCL9L	UBASH3A	PLCH1	TAF5L	MYO1F	KCNA6
FCRLA	MN1	PGBD1	DCLK1	C5AR1	ARID5B	CD7	TREML2	RGL4	RD3	SAMHD1
CALU	FCHSD2	CHFR	C12orf68	LIMK1	HTRA3	GBP1	DGKG	PRDM1	CCKBR	ITGB8
SLC24A3	PTGDR	GLI1	RGS20	PPP1R14A	KRTAP1-1	EIF1AD	CST2	STK17B	COL6A3	ITGA3
MSRB3	DISC1	CSF2RB	ROBO1	CCDC141	CLEC11A	UTP14A	CCDC46	LTBP2	FAIM2	ROM1
LOC400891	CD300A	GRAP2	NRG3	CALD1	RNF145	C10orf128	CD96	LRRC15	VASP	TNFRSF10B
KLHDC7B	FCRL2	TRPC4	FAM126A	SPIB	TYROBP	NALCN	AFAP1	ARHGDIB	CXCR3	HABP2
PRRX2	CHSY3	SDC4	CLEC4E	TPSB2	CMTM5	HECA	SHISA5	GRID1	HLA-E	LOC283070
LTA	CD1E	CFHR3	IL16	PROCR	LRIG1	SFRS13B	FIGF	NPR 2.00	AMMECR1L	NUDT11
SHF	SLC1A4	FAS	IL17D	PLA2G5	FLT3LG	UBE2S	MYLK	LPXN	TMEM119	LILRA6
WTAP	ARVCF	KIAA0922	RBM46	TMEM158	GUCY1B3	FBLIM1	NRBP1	KCNK6	IRAK3	CCDC81
SLIT3	CRISPLD1	SLAMF8	CLEC12A	FAM5B	NAMPT	BYSL	CCL5	LOC100134259	OMD	FRMD5
CNIH2	SLC2A10	ACTA2	TMEM196	NCF4	TPM2	CALHM2	ARRDC2	TTYH2	CSRP1	CD63
TMEM39B	KAZALD1	RAB32	PRR16	CACNA1C	AGFG1	TBX1	PECAM1	IL6	DDX5	STX1A
NTRK3	TLR2	C9orf91	PIP4K2A	IGDCC4	HUS1B	MMP17	STIM1	SYNPO2	SIGLECP3	MAP3K6
FKBP11	CD4	PACS1	INPP5D	WISP1	CRTAM	IL17B	LOC283663	C3orf59	TMSB10	SIGLEC1
TPSAB1	PRND	F2RL3	LGALS9	GAB2	TNFAIP8L2	ADCY2	CSMD2	HLA-DPB1	DACT3	TPRG1
MAP1S	LY86	FOXG1	TICAM1	CST7	CFB	LOC401093	CRLF3	GTPBP2	EBF2	SPARCL1

SIAH1	DCP1A	SH2D1A	BMP1	SLFN11	A2M	MEGF6	LPGAT1	RLTPR	PAQR4	KRT75
ADAMTS12	GJA1	PTCRA	GNL2	OLFM4	ARF6	TEAD3	FLI1	WISP2	CD48	ITGBL1
FXYD1	FGD2	DEGS1	LOC100233209	LTBP4	CX3CL1	FLRT2	RCSD1	JPH3	NLRP12	GIMAP5
MCL1	GAPT	IL12RB1	SGCE	MATN3	SIX3	ADAM33	ADARB1	BASP1	AMOTL1	GRM7
ROR1	APOBEC3C	LY96	C11orf87	GZMM	FCGR1B	HS3ST1	LST1	C4orf7	HAS1	CD52
CYTH4	PSG1	KCNA3	CD86	VSTM2L	FGF13	LOC158696	MGC87042	EML3	RHOA	LYL1
INPP1	GPR37L1	NCF2	CCNJ	TAP1	S1PR4	RIN3	RP1-177G6.2	DDX53	SIT 1.00	HSPA5
SLC26A2	ARHGAP9	IL32	HK3	PLBD2	FCGR2B	BTN3A2	RTBDN	SYDE1	FHAD1	DKK 2.00
SRM	SERPINA3	LDLRAD2	BRSK2	ANXA6	FCER1G	PYHIN1	LOC606724	IKZF3	PDE5A	GGT5
TBXAS1	NKX3-1	CTSW	HCK	GRIA1	PDGFRL	PVRIG	GNB4	TNF	MYL9	CD81
MFAP4	SELE	IMPDH1	NDP	TAC1	PCDHB15	ITGB1	KCTD11	PCDHB9	ANP32B	NCF1C
MUC17	ZP1	KCNMB1	TMEM59L	FCGR2A	ALOX5AP	UNC13D	ASAP1	C9orf150	IGFBPL1	RUNX2
LRP1	TMEM71	LMOD1	CLCF1	HMHA1	LCP1	CSF2	LMNA	CD247	C3AR1	MS4A6A
FCGR1A	CLEC4A	PDLIM3	EREG	HLA-F	TRIM38	THEMIS	WASF1	PREX1	CASQ1	BIN2
ENPP1	LGI4	CWC22	MYD88	KIAA0226	ALOX5	SFRP5	NDN	AEN	IL13RA1	COL11A1
SOCS3	ATF4	AKNA	DOCK2	GLT25D1	FAM109A	C10orf26	IL7	CXorf21	PMEPA1	DERL3
F10	CASP1	PCDHGC5	KCNAB1	ECE1	SGK1	GLI2	CACNB3	COPZ2	DUSP7	PMS2L11
CHN1	SPOCD1	CD1C	HLA-DOB	CD79A	EID3	SLAMF7	CBFA2T3	NFIL3	RAP2B	RAB8B
COL3A1	PLA2G2A	TES	SIRPB2	JAM3	SNX20	SEC24D	SGCA	RAI14	BAG5	NUP62
BST2	RCE1	ARHGEF6	PPAP2C	ACTC1	PPP1R15B	KIAA0125	ZFP36	TNFRSF17	TMEM132B	C1QB
ADAMTS10	CXCR5	PARP15	TUBA1B	LILRA1	ELF4	SH3RF3	SLC9A5	FAM49A	ANKDD1A	CD93
CSDA	LILRB4	TSHZ3	TNFSF10	ALK	RASL12	DNAJA4	TCL1A	KCNMA1	FAM78A	SLC1A3
PCDHGC3	DPF1	COL5A2	CCDC136	CSK	UPP1	PLN	CSF3	ADAMTS16	PPP1R12A	SLC5A5
ARHGAP25	A4GALT	DPY19L1	TMPRSS15	FHL5	C1QC	ITPRIPL1	LAPTM5	ADRA1D	KRT33B	TIAM1
RUSC2	CTSZ	FAM177B	SLC9A9	IRF4	PPFIBP1	CCR5	FGF7	LMBR1L	ELFN2	LRFN5
MRVI1	MKI67IP	ZIC1	FAM18A	NMT2	ICAM5	HEPH	AOAH	MYO1D	MFAP5	PTPN22
CCL20	SNAI2	PRSS23	TNFRSF18	EFEMP2	ZPBP2	NRBF2	DENND5A	KIAA0408	MUSTN1	CNN1
FBLN1	HIVEP1	GXYLT2	C3orf70	MMP3	PDLIM4	SCRG1	ZMYND15	CXCR6	C13orf26	GNAI3

TBCCD1	CCL19	LILRB1	CD53	LRRN4CL	EVI2B	CLCC1	LOC728392	ILK	CLEC7A	PTPRN
CYTH2	TNNT3	MARCKSL1	SERTAD2	TNFRSF9	HIVEP3	LRRC8E	RFX8	KEL	RGS10	ADAM17
HHIPL2	37135	TRIP10	DKK 3.00	TRAF5	CCR4	KCNN4	SERPINE2	CRIP1	FAM65B	IRF7
CD101	PPP1R9B	C1orf216	SAP30BP	CDCA4	C1orf94	GRIN2A	CD33	ABL2	PDCD1	COL13A1
SULF1	SIX1	PHF13	VEGFC	SBSN	SPN	MICB	RELA	LOC285205	TMEM39A	NGF
P2RX1	GSN	PDGFB	MUC7	KRT80	ARRB2	GEFT	FCGR1C	HLA-DPA1	CARD16	DEM 1.00
PCDHGB1	IGFL3	SLA2	EEPD1	ZNF532	COL4A1	PI3	AMOTL2	HDAC9	AOC3	GPC6
CD28	PSMB9	PNLIPRP3	CNN2	FOSL1	P2RY6	KCNQ4	TRANK1	IL1F8	CDON	IL13
C8orf74	CACNG2	DMRTC2	FGF21	GPR32	KCNU1	KRT76	KRTAP10-10	KRTAP11-1	KRTAP21-2	KRTAP4-2
LEUTX	LRRC30	LYPD4	NLRP13	OCM2	OR10P1	OR4M1	PRR23B	PRR23C	TEX13A	IL2RG
IL1F5	GPR65	MPP4	RIMKLB	NFATC4	C5orf58	TNFRSF12A	CSRNP2	C7orf58	ULBP2	GPR25
FXYD7	CPA3	CCND2	PRR23A	IL8	EVL	FCRL3	CEP170	B3GNT9	GPR56	LGI1
AVEN	WFDC1	ADCYAP1R1	PVT1	ACOT9	CSRNP1	ZNF536	ITIH3	AKR1B15	ARHGAP10	TAPBP
FAM189A1	MMP16	CASP4	OSM	FILIP1	VGLL2	FLJ10357	SIGLEC14	MGC29506	PCDHB13	TCTEX1D4
SCML4	FAR2	PCDHB10	ACTG2	TSGA10IP	S100B	RNF19A	CHRNA9	ASCL1	RAPGEF5	NUDT10
TRIM21	CYSLTR1	PID1	BCL3	SHANK1	UCN2	G6PD	OGN	SIGLEC6	CRISPLD2	WFDC2
NECAP2	MYO3A	TNFRSF13B	TRPV5	COL15A1	ARHGAP20	PLXDC1	ART3	PAPL	NOD2	MFRP
ITGB2	KRT27	DDHD1	PTN	IBSP	GPR172A	FPR3	RAPH1	CRLF1	PRKCH	FAM180B
PPP1R12B	TNNI2	ACTR3	HM13	CST5	MRAS	JMJD6	MYO1G	DDX26B	LOC388796	KRT79
TBC1D2B	CD14	LASS1	NAB2	MKL1	C12orf35	DAGLB	EDNRA	REG1A	SYNM	NOP2
CCDC88A	CA5BP	GPR174	CMKLR1	38047	MANF	FST	C4A	DAPL1	HAUS2	POPDC3
GDF10	CHODL	FYB	DYRK3	ADCYAP1	CNKSR3	SORBS1	TLX2	CILP2	TMEM200B	TWIST2
CDK17	NCKAP1L	NTF3	HTR2A	ZNF267	PRG4	EML1	NR0B1	GDF1	MBNL1	HNRNPAB
PAX5	PYCARD	MKX	RGS13	RCN1	EVI2A	BMP15	NXPH3	KLHL38	LRRC70	HOPX
RSU1	PHACTR3	ZAP70	NMNAT2	CTLA4	CSF1R	CLEC17A	SULF2	RTN1	SUSD5	CLEC5A
VPREB3	ODF3L1	CCNL1	ZNF423	RIMBP3C	CDH3	NCRNA00093	LTB	CD80	XCL1	ART4
PTPRC	TPSD1	IFNGR2	CXorf36	GPR18	FAM129C	DPYSL4	TMEM35	HLA-DRA	JOSD1	DCDC2
SEC23A	IFI44	REEP1	SGCD	FAM155A	AP1G2	C10orf90	RUFY4	ACTN4	IL23A	LTB4R

MYH11	LOC150622	FADS2	PDE4B	ADPRHL2	DUXA	MIDN	EBF3	ITGAM	ANXA2	XCR1
ACVRL1	CBFB	PPP1R15A	UBA7	NOTCH2	ENDOD1	LOC283867	STARD3	ZNF80	LAMA4	ZNF469
EEF2K	ZNF394	WDR69	PKDCC	FCER2	CFTR	GZMB	LILRA2	TPM3	ADAM12	PRDM6
FCN1	PARP9	ZNF830	PIWIL3	HLA-DOA	KLRG1	PTAFR	C4BPA	PIGT	TMOD1	GPR126
ZNF250	ANKRD50	WDR43	SOX7	ZNF608	RPGRIP1	GTF2E2	MS4A2	APOLD1	LRRC17	GNL3
TMSB15B	FCRL1	ROBO4	FKBP10	CATSPER1	TMEM22	CTSS	RPSAP52	FKBP1A	FBN1	FRMD8
MS4A1	STAMBPL1	KRT1	P2RY10	ADCY8	SEZ6L2	GZMA	SERPINB1	OR10V1	LDB3	KRT34
PLD4	FBXL7	TTC17	DAB1	GALNT1	SKI	TP53	KIAA0020	LEFTY2	UNC45B	AFF3
EMILIN1	GPR124	LPPR2	CCNK	ACD	UTP11L	DDAH2	RIPK1	MSL1	GAR1	GZMK
NCOA5	THBS4	CLEC9A	KANK2	PLP1	CLIP3	CEBPG	FCRL5	IL33	TRIM47	VANGL2
RAB9B	ADAMTS7	TRAF3IP3	GALNT13	FKBP7	SLCO2A1	ARHGDIA	GTDC1	NEDD9	ICOS	FAM135B
WDFY4	CNN3	SYNPO2L	SNIP1	XCL2	RAB20	UBE2C	KDM6B	ANK1	KIAA0247	PI16
PCDHAC2	PLOD3	TRAF1	MEOX1	MVP	C20orf200	SIGLEC9	ZCCHC12	VIPR2	KIAA1324L	MYOCD
C13orf33	NCRNA00181	ARPC5	MPEG1	PRPF38B	CRTAP	CASP3	S1PR1	GPR112	SFTPA1	CD200
VSIG4	STAB2	RUNX3	LRRC8A	NID1	RBMS3	TBX5	SFTPC	TMEM149	KLRB1	LPPR4
SPEM1	HRCT1	VASH2	HERPUD2	RASGRP4	MIAT	SYT16	PGM2	ZNF597	PTCHD1	MET
ELAVL4	CXorf49B	PAX3	KRT78	HMGA2	SYNCRIP	HBEGF	TTC28	KCNH2	SCARA5	HPR
LOC100129550	SLMO1	KCNQ5	UNC5CL	SH2D3C	FNDC1	APOBEC3A	CCDC3	FCGR2C	PDLIM5	NLRP4
IFFO1	PPIB	LOXL2	PPM1D	SRPK3	RBMX2	PDP1	REEP4	BAX	POLR2D	C1orf130
BRDT	SF3A2	PPP2R2C	FAM70B	MMP9	DUSP5	SFN	SDPR	HYOU1	VSIG10L	CD163
SFTPA2	FAM26F	NOP56	DSCC1	PGBD2	JAK2	B2M	C16orf72	JHN8	ZNF35	PPM1M
C2	INHBE	SQSTM1	ELMO1	HLA-B	HGFAC	CHRDL1	IRF9	SLC16A8	NCF1B	DDX3X
PFN1	C3	RPGR	CECR6	DCUN1D5	PIK3CD	TNIK	ZNF71	TMEM132A	CASQ2	FAM118A
DES	HOMER3	ARL 9.00	TEKT3	PANX1	XYLT1	TAP2	BRPF1	TNFRSF10A	RAC1	CYBRD1
OLFML1	PIGZ	SLC17A2	AMAC1	PCDHGA6	SRGN	IGSF21	CXCR2P1	HNRNPA0	TRPC1	PLCG1
C19orf38	DCX	TLR10	FAM114A1	OR4D6	OR4X2	PRB1	SNORA1	TREML2P1	ALPK2	NPTXR
SIPA1L2	DAND5	KPNA2	MS4A4A	JAZF1	MESP2	CD19	VAT1	TOX2	MT2A	DENND3
HCN1	TMEM98	CASS4	CAPZB	CD22	C11orf84	IFI35	CNTFR	SLC15A3	PDGFC	TSPYL2

C17orf53	SCN1B	PIM2	HLA-A	CRYBB1	TAC4	VIT	ROCK1	CHPF2	NRIP3	NRGN
ZCCHC24	LOC284688	ITSN1	GUCY1A2	NMI	LOC96610	FAM116A	NCK1	SH3BP1	PMP2	FAM110A
FAM167B	KCNA2	C20orf197	RHBDF1	C14orf34	MPZ	LMNB2	FADS1	FNBP1	ADRA1A	MNDA
GNA13	RGS9	C22orf9	MUC12	ACVR1	HSPB1P1	HRC	HLA-DRB6	FBXO45	MED6	C1orf54
MGC12982	UNC93B1	CSDAP1	RNASE1	GGN	DOCK10	P2RY12	EMB	C11orf21	SLC30A3	TRPM2
C19orf10	IFI27	FOXL1	PHKG1	ROPN1L	SLC25A37	RIMS4	GPR132	GPA33	CSRP3	C6orf167
EIF2AK3	CCRN4L	ACTB	GJC2	MOBKL2A	CDKN3	GDF11	IL4I1	RPIA	SNAP91	MED15
ACTA1	OGFRL1	CLEC12B	KIAA1324	STAB1	NRAS	LOC283174	GPR19	IL18	MS4A7	GPR114
BMX	NPC1	CLIC2	MAFB	C19orf22	GHRL	KRTAP10-4	GLT8D2	POM121L8P	CD226	ZEB2
UBAP1	WDR44	SLFN5	MPZL1	ATP2B1	CNR2	HHLA2	TMEM214	AHR	FN1	EGLN2
NFIA	SUN2	EDN2	KBTBD8	KALRN	SLC7A2	HSD17B6	ARNTL2	NRXN2	PELO	FCAR
NACAD	DTHD1	SLC17A7	ANKRD1	CST3	POLR1E	JDP2	ACBD3	SMPDL3B	CCDC9	COL7A1
KRTAP26-1	ADCY3	TCIRG1	TM4SF19	RNF217	POSTN	KCNB2	OR11A1	SLC10A6	CNGB1	TLR4
SH3RF2	THOC4	KAAG1	COL10A1	LCE3D	POTEC	NCRNA00152	MKNK1	C2orf53	CELSR3	GBGT1
GJC1	TMEFF2	CHST8	CPT1C	NKIRAS2	HLA-DQB2	ADAMTSL5	MT1B	CSF3R	AMPD1	REG3G
C1QTNF3	CLGN	CDH19	NRN1L	KCND1	ADAMTS3	CD99	YWHAZ	AFF2	KRT82	JSRP1
ABI3BP	GSTM5	GAS7	PTPN7	LOC148145	C21orf63	TRIOBP	OR4A47	LEF1	THOC1	MIER2
ST6GAL2	EXOSC3	UCN	KCNT1	DMD	TRPM8	F2R	CD300C	LOC26102	ABCC8	SRP19
TREML1	PCSK5	NXT1	FCHSD1	RBM7	ACTL8	INHBA	ITGB1BP2	STK4	SNED1	PSMB2
NMUR1	MEF2C	PCDHB19P	LAMA2	RET	OR5C1	SPRY2	GBP5	TCEAL5	CD38	KLHL29
ANGPTL5	RNF34	BTN3A3	DDX60L	CD79B	ZC3H12C	LOC643763	TRIO	LTF	CARNS1	FOLR2
CSPG5	EFCAB4B	NUDT1	APOBEC3G	CNPY4	NUP85	SIGLEC7	GRIN2D	KIF17	SYTL1	IFI6
ARHGAP23	PLCXD2	C3orf58	FAM76B	CNPY3	TSC22D2	XAF1	TUBA1C	MARCKS	WBP4	JUB
ITGA8	LOC100188949	CDR2	AXIN2	CAMSAP1	BCL11A	GZMH	PVRL2	TPRXL	PDE3A	CBR3
COL6A6	KLK5	RBP1	BID	AIF1	TBXA2R	BCAT1	DBNDD1	HSPB1	PPIC	SAA1
RHOQ	ANXA4	CDK9	RRAD	MX2	FGF14	PCDHGB2	NFATC1	CD8A	STARD5	NECAB1
CD180	C1orf200	GIPC3	COL5A3	SH3BP5	MOBP	LUM	EPC2	ACHE	PPP4R1	CCM2
RBM15	EOMES	C17orf87	CDKN2BAS	CSNK1D	FBLL1	RPE65	SEPN1	MMP7	PILRA	FAM64A

RPL28	PCDHGA10	KCTD13	EPHA2	MC4R	CHORDC1	RAB33B	TGFB2	LSR	TRIM11	REG1B
STMN3	APOL3	TNFRSF4	GCG	GABPB1	C12orf42	CTXN1	SCARF1	UBTD2	MYBPC2	SELS
CD1D	GLRA3	SPC24	TAF1D	CAV2	OSBPL5	OR1F2P	ADAM6	LAMA3	JUND	U2AF1
CYTH1	PHC2	RGS11	GSG2	RYR3	SFXN3	TSPAN4	SPP2	ISG15	SLC4A5	C1orf186
ZNF668	STAG3	KIAA1598	PPARD	MMD2	SLITRK3	HGSNAT	LPAR1	EPHA3	DYNC1LI2	ANXA2P2
LRRC8C	IL2RA	NETO1	ABCA8	WDR46	TEX10	LOC399959	TNNT1	LRFN1	NCKAP5L	PLP2
CASP5	C3orf64	C3orf45	SFTPB	TMIGD2	RFPL4A	NLRC4	NOTCH3	SLC15A4	EPAS1	PKIB
ADAM9	CD209	BNC1	ATP1B4	EBI3	STOM	ZEB1	PSMB10	UNC5B	ZSWIM6	HLA-DQA1
HPCA	RNASE2	SLC43A3	DEFB124	HLA-DMA	CHST11	SVEP1	RALA	SIRT1	TMEM200A	GYPC
PLA2G4C	CRELD1	PPP3CA	DNAJC5B	STBD1	PAQR6	CIB2	COL27A1	RHEBL1	C12orf53	ADCY4
KDELR2	SQRDL	NRAP	KLHL30	PER1	CD302	TKT	P2RX5	EMR2	SERTAD1	OR51E2
TNK2	CYTL1	PARP8	WNT6	ZNF189	MTHFD1L	LRRC33	ZNF263	FAM134B	RXRG	WNK2
MYOM1	KRT8	IL15RA	EPSTI1	FBXW7	LPAR4	INF2	NKX2-1	ADORA3	LYAR	PCDHB2
NRN1	SSPN	FZD8	ITGA4	DUSP22	TBX18	DDX58	SLC35C2	SHC2	PHTF2	RBM9
TSPAN32	C19orf50	CAMK2D	ATP1A2	PAPD7	C9orf172	GDPD2	IFI44L	LY6G6C	NFE2L3	INHBB
PLXDC2	ZNF140	KRT16	MRC2	CRELD2	PDGFD	WNT9A	SIGLEC15	COL21A1	KCNIP1	CRYGN
CDC42EP5	FBXL19	NRXN1	C8orf58	MAML1	LILRA5	SOX8	RECK	PCDH17	PMM2	GRN
SOX4	OTOF	C8orf47	NPFFR2	IL10RB	STX4	F12	CLDN1	CAD	KRT81	PRR7
CCL18	GPR1	EZH2	SERPINB7	RPS2	MACC1	ADAMTS8	SNX22	CDSN	VASN	MEX3B
SPDYE4	SNAP25	C1orf14	SLFN12L	CACNA1G	ECSCR	EHD2	SIN3B	VCAN	SLC8A2	TSC22D3
ADAMTS9	SMARCD1	ADAMTSL3	BCL10	STK38L	NCR3	ADH7	PTGIS	SHC4	CHD1	LIMS2
CRYAB	NAB1	KLK14	GCC1	XKR4	TMEM47	RARRES3	HLA-L	KIN	FAM53C	HAUS8
CTNNB1	HAVCR2	SYCE1L	RBBP8	C7	BEGAIN	ARHGAP31	CFL1	DLX1	IL18R1	SPATS2L
IRF3	CCDC137	OPRL1	BNC2	ST8SIA4	PCP4L1	ZNF805	ZNF430	C1orf173	SERPINB2	SIGLEC10
OSBPL10	TMEM151A	PRAF2	TSHZ2	MAP3K14	TMEM54	ZBTB8A	OAS2	ENG	INTS8	PLEKHM2
TDO2	C1orf150	NBLA00301	PI15	ATHL1	NDC80	SNAPC2	OPN3	CDH17	PTPN2	MOBKL2C
FCER1A	CAMK1D	ALOX15B	RNF125	KCNQ3	RNF182	DIAPH2	GTSE1	TRIM22	ARMC3	SPRED2
C20orf106	DNAJC2	CD47	CGB	SP140	HTR3A	LYVE1	TPD52L2	RRM2	LAMP3	NAA38

FER1L4	POM121L9P	TNMD	FOXD1	PDCD1LG2	ARHGDIG	CARD17	DDX60	HLA-DQA2	EMILIN3	MSL3
BPIL2	TBC1D10B	FUT8	RGS18	RNF122	PTPN6	SAMD11	KRT28	FOSL2	CADM4	ARX
FAM13C	SYT14	RAB23	HLA-H	TGFBR2	PYCR1	PDPN	PSMD7	ARHGEF2	GPR141	LAMB2
ESYT1	TREML3	ZNF131	SIPA1	MGC45800	ZNF200	ST3GAL2	CCDC102B	SF3B4	CRK	HSPG2
DNAJB4	P4HB	STAT6	ISG20L2	BGN	ZNF222	TCEAL4	TRPC2	LARGE	RADIL	PPWD1
COL8A2	ADA	GEM	FNDC3B	RGS17	CDH13	AP1S2	ADAM15	GFRA3	CIITA	ZNF121
LOC100101266	LINS1	CKLF	ATG9B	SLC39A1	C1orf110	B3GNTL1	ITPKA	RCVRN	MNT	LAPTM4A
LCTL	TRIM35	WSCD2	AP3S1	TINF2	SELL	BACH2	KDSR	ZBTB32	MAP4	MYCT1
DGKD	IRAK1	NRM	ZC3H12A	MACF1	TMEM188	ARHGEF10	NME1-NME2	VWA1	ANO9	C12orf45
RPTN	S1PR5	CCR10	CD160	HGF	PIM1	ZBTB47	GPR161	SPRED1	AP4M1	C13ORF56
GNA12	CCL3	HIVEP2	NIP7	RNF103	DIP2B	CDK2	KCTD17	RBMXL1	TTC9	ITGB4
PHLDA3	RAMP1	RCC2	CDC42EP4	APOL6	NOD1	HSP90AB4P	CPNE5	VASH1	CLIC3	CPXM2
ZNF746	CGB7	FOXN2	CALR	PRSS30P	ITM2A	CYP4Z2P	PPP1R10	THSD1	CD68	GABBR1
TBX15	ITGA1	ARL6IP6	H6PD	ANXA2P1	SEZ6L	VGF	SEMA6C	REM1	EPR1	CNTLN
RPS4X	FZD7	ZCCHC10	ODZ3	RGMA	MATK	PCDHGA12	tAKR	E2F1	LOC153910	CDR2L
HDAC7	KIAA1644	PAPOLG	QRFPR	C7orf26	SCG5	VPS37C	NIN	TP53RK	SIGLEC5	ANKRD34A
TGFB1	NOM1	DCLRE1B	ZBTB49	HLA-DPB2	CCDC120	C9orf100	SERTAD3	ARPC1B	NAGK	TACC3
KIF19	PCDHB12	ZNF598	KCNF1	TRAF2	BCL11B	PLCB3	FLJ43390	TBL2	TOE1	OAS3
BUD13	FAM131A	CYTSB	GAL3ST4	SP110	SPATA12	C17orf49	SMNDC1	LOC727896	NOVA1	FCGR3A
NKAIN3	DMPK	P2RX7	SIAH2	FAM55C	SLC6A20	AADACL2	MEOX2	PRRT2	MPPED1	TULP3
PPP1R1B	S100A2	DGKQ	FLJ42393	APOD	C1orf127	RECQL	PARVA	B3GNT5	SH3PXD2A	ZNF473
MMP25	THOC6	RIMS3	DLG4	DCHS1	GNB1L	TBX21	DCAF15	ENTPD2	CCL17	RFTN2
SNCA	PSORS1C2	IMPDH2	BIRC2	ZNF622	CHEK2	KLF10	GALNTL6	HJURP	FHL2	NBPF15
NUSAP1	DSE	ECM1	OXCT2	LDLR	SBF1P1	IL27	FMNL3	SH3GL1	TLE4	NLGN3
KCTD20	NDRG4	STAT2	NOV	RNF166	TRH	OSTN	VCL	LAMA5	ERF	MCM6
LAMC1	C17orf76	CCDC107	ELL2	SCXB	ITGB6	IKBKG	TPO	ADPRH	EMR4P	CHML
POFUT2	PRC1	FAM83G	ADRBK2	MLLT11	ZNF259	ELOVL5	XIRP1	GPR173	DONSON	C2orf27A
NEGR1	RRP12	GIMAP4	IL2RB	FGD1	MMP15	CD99L2	PDIA3	FOXO3	ITGAD	CEP164

PSG8	TEAD4	IGSF6	FIGN	TMEFF1	SNHG12	CRX	GNLY	CEP55	PTTG1	TSSC4
TLR6	LOC285548	C2CD4B	ARMC5	TACR2	CNTN2	ACTN3	GCKR	RBBP6	DDX21	PLEKHG1
CARHSP1	PRKG1	TGM2	MSR1	ABCA6	LMF2	RNF213	DCAF4L2	SLC25A32	TRIM17	SFRS17A
RPS6KA4	LENG9	CDK2AP2	TRIP13	PSORS1C1	KLHL4	SIGLEC16	MORF4L2	PPP1CB	C1orf68	TUBA8
DNAJC1	EIF1	FEM1C	OSTM1	IL27RA	IGF1	CCDC112	ARL8A	MT1M	CXCL13	BIRC5
C14orf80	AATF	IQGAP3	PLXNA3	ABCB5	APBA1	UBE2Z	HDX	CNTNAP3	OR10K2	TTC13
HOXD13	NUTF2	KIRREL	IRF1	VARS	FES	BCAR1	C5orf20	PCDHB11	SSX1	KRT18
MRC1	RSPO3	C10orf50	CLEC4D	RPL13AP20	SLC37A2	KLF17	PAPOLB	TSPAN1	TNFAIP1	BMP2K
MEG3	HTATIP2	INSM2	CCL3L1	SPRED3	KPNA4	KIAA1462	MS4A14	NCK2	SND1	LOC100128554
BRD1	ZNF280B	FAM159A	RGS22	INPP4A	TMED9	NRXN3	EPB42	IL2	TPX2	PGLYRP3
PTPRK	TCF4	CD84	BACH1	CCR2	AKAP13	TNFRSF8	SGCG	MYO1A	KRT222	NKX2-8
PYGB	GNB1	CCDC59	DDX17	FAM129B	DLX5	ACTR5	ZRSR2	ATG16L2	CLSTN2	DLK1
DDX11	PAM	IL12B	HSP90AB1	EMID1	MFSD2A	ATP1A3	KCNV1	RAMP3	BTN3A1	XPO6
CLVS1	LTBP3	HLA-J	GPR97	SPNS1	JAKMIP1	TIFAB	SKINTL	AREG	PTGDS	FOXJ2
CENPI	ANXA5	PEAR1	FERMT2	PAG1	MMRN1	AK5	LRRC20	SNX33	MATN4	LOC645166
NKD1	PCSK1	OR52N5	BICD1	LTBR	TMC2	P2RY8	ERMN	TCF3	CD3EAP	CD244
RNASE4	ANO1	TJP1	NOP58	LOC401387	E2F6	KDM5B	S100A3	CFD	F3	SIGMAR1
PLEKHG4	DPP9	C14orf184	DNTTIP1	DDX39	LYZ	IKBIP	FBXO30	P2RY14	LPAR5	IFNA5
LOC728855	SPANXN3	EZR	KC6	PAQR9	AGTR2	BOC	URB2	OR8B12	TLL1	FCRL6
ARHGAP26	ZCCHC6	QRFP	GPR34	TESK1	SMOX	FAM169B	STAT3	IL10	CD40	DNAJB13
SH2B2	MAK	NKAIN4	ITGA9	FBXL6	EPDR1	FAM71C	GPR156	PARP14	TAC3	FAM72B
GNG4	SELP	LAYN	MALT1	HAPLN2	ABR	STAT5A	DSC2	KLHL25	NKX3-2	NUCB2
TP53I13	SERPINA9	EMR1	MEIS1	SOAT1	GIT2	ARFGAP1	KLRD1	OPN1LW	C17orf67	ZSCAN22
CCR1	RIMS1	LGALS3BP	LOC400804	TRAM2	REP15	SKA1	SWAP70	C13orf31	CDKN1A	IPO4
PAK1IP1	CAMKK1	CMAH	RILPL1	RAB13	LOC647121	CDX1	RNF219	SYT12	AMZ1	CEP250
TTC39A	EPHA7	OS9	ADAP2	RFC2	TM4SF1	C14orf180	WTIP	GALNTL2	TET3	MSI1
ASF1B	SUV420H2	CD8B	TMEM185B	FLJ45079	TFPI2	TLR8	MICALL1	NFE2L2	PLCB2	SETD7
RABIF	CMTM1	CFHR4	SPATA2L	KREMEN2	SDS	ACSL4	IFIT1B	CD163L1	PCGF3	HPGDS

AXIN1	PCDHB16	TRIM27	PCDHA6	LOC653653	TMEM159	NPR 1.00	ZNF295	AGRN	MMP24	ZNF642
MGAT2	NEIL3	IGSF1	KLK8	XIST	FAM71D	EIF2S2	RPL22L1	PGM2L1	PYGL	GNG2
NHS	RNF149	UBA6	CRYBB2	LPCAT4	ECM2	PDZRN4	PRICKLE2	BARHL2	EVC2	DCN
NASP	LMX1A	ZMIZ2	GEMIN8	ACTG1	LIPC	ZNF548	UBE2D2	STOML1	PLD5	LOC644538
PRKD2	FGF20	HSPA6	CYLD	BTBD10	LHX6	C15orf41	CCDC14	CDH5	RGS3	LY6E
SEMA6B	RALY	MAPRE1	PCDH19	LPAR6	FOXF2	HYAL2	CTPS	EXOSC9	DUSP27	MELK
ITGA11	FAM89A	FAM171B	MTA1	C9orf110	RTKN2	GPR4	ACTL7B	SEL1L2	PDE6B	DDOST
FAM48B2	KRT23	TMEM44	ZC3H4	ABRA	DVL2	LLGL1	DNAJC3	CLK1	CDH11	GLT25D2
FAM69A	LOC728264	MAP3K5	MTMR9	LTC4S	KRTCAP2	SORBS3	C2orf55	ULBP1	PPAP2A	HS3ST3A1
ZHX2	PNN	RIMBP3	STRN3	TNFSF11	SOX10	SFRS4	VWF	CCR8	TMEM100	RPP38
SPC25	PNMAL2	MRGPRF	DKFZP434L187	WNT7A	PNRC1	DDX20	LIF	TXNL4B	DUSP4	GPR82
ARL 11.00	CLEC2D	CAMK1	RAET1L	BHLHE22	TNP1	RPF2	CENPA	ZNF217	LDB2	TNNI3
NPAS4	LRRC42	SLC11A1	APAF1	BLMH	LZTR1	MORC2	CARD10	AP2A1	NR2F1	LAIR2
CLECL1	AKAP2	H2AFX	PREX2	EIF2S1	DUSP1	CDC20	PCDHB6	DULLARD	C12orf44	EEF1D
TMEM155	RPS19	HOXC8	DIMT1L	BNIP2	UBE2B	VCX	UTP3	SMC6	NDE1	VAMP5
RAP1B	C6orf118	OSR2	MFNG	C19orf59	CHRNB2	TRIM16	KDELC2	SEC61A1	GPR20	KLHL28
RBM22	SULT4A1									
**Coexpressed genes with Myc in KIRC (Stage1)**										
NFIL3	KDM6B	ZFP36	MIDN	PLK3	FOSL1	CCRN4L	JUNB	NDEL1	MYADM	PPRC1
SOCS3	EPHA2	FOSB	RARA	KLF4	MCL1	C6orf145	ADAMTS4	JMJD6	CSRNP1	ERF
PPP1R15A	BYSL	MAFF	ITPRIP	PHF13	KLF10	DUSP5	SERTAD1	JOSD1	XIRP1	MAP2K3
TICAM1	CYR61	RIOK1	ADAMTS1	ZC3H12A	AREG	EIF4A1	DNAJB5	JDP2	KLF16	CLCF1
HBEGF	CDKN1A	ITPKC	POLR3D	SFPQ	BCL3	NFKB1	LMNA	KLF6	SNAI1	DUSP6
DDX5	AG2	BHLHE40	PFKFB3	CCL2	CXCL2	SOX7	SRF	EMP1	DDX21	IRAK2
NOLC1	SLC2A14	ARID5A	HES1	DCUN1D3	URB2	TAF5L	BAZ1A	ATF3	SPRY2	PPP1R15B
LYAR	SFRS15	FOSL2	PHLDA2	TRIB1	ETS2	SLC2A3	GTPBP4	YRDC	JUN	STK40
DUSP1	F2RL3	LIF	AMMECR1L	NAB2	ZYX	NFATC1	BMP2	FOS	DDX3X	IER3
MAT2A	PNP	GEM	TNFAIP3	PWP2	FRMD8	ISG20L2	B3GNT5	IL1RL1	WDR43	SFRS7

PHLDA1	RELB	TNFRSF10A	KBTBD2	RHOB	RELA	CSNK1E	MESDC1	MAFK	SIK1	SOX9
MAPK7	SEMA4C	GPR3	SPATA2L	ZNF295	RND3	SF1	RIPK1	CHSY1	PHC2	SERPINE1
RALGDS	APOLD1	TRA2B	TMEM217	KCTD11	C12orf68	NFKBID	ZBED4	THBS1	STC1	RFX2
SPRY1	NR4A1	AEN	IDI2	RRAD	CSGALNACT2	NR4A3	AATK	CHIC2	FERMT2	GPR4
ING1	NFKB2	EIF4A3	LATS2	STAT3	ODF2	CHD1	TNFRSF1A	PIM1	NIP7	DOT1L
ZFP36L1	ADM	SNORA67	CDC42EP4	DUSP14	ELL	TUBB6	NFKBIA	LRRC8A	THBD	TSC22D2
CYTH2	NFE2L2	BRD1	HIVEP2	HDC	EIF2C2	PRKD2	MEX3D	GPR135	EGR3	FAM107A
CDC42SE1	PER1	BCL6B	MAFG	TAL1	FAM160A2	EIF2S1	WTAP	MAP3K14	PELI1	PNRC1
SPEN	LONRF3	PRPF4	TAF13	TNFRSF10B	C9orf21	C10orf108	ICAM1	SPRY4	HIVEP1	PLEKHG2
TGIF2	CDC42EP1	PDGFB	ETS1	ZNF830	ATXN7L3	RCE1	TRIM40	CCNL1	TDG	C15orf39
RASSF1	BCL9L	INTS6	GNL2	AOC2	SFRS3	RNF122	SLC25A32	FOXC2	SNORA1	ETF1
POLR1C	IPO4	HNRNPU	LRRC42	RRP12	ADAMTS9	NUP98	SERTAD3	C9orf30	GADD45B	SAFB
SERPINB8	NUP153	DMWD	SOX21	TCEB3	USP42	NUAK2	TFRC	HYMAI	DNAJA1	C5AR1
ARHGAP17	RGS2	ZBTB7B	LRCH1	C1orf183	MAK16	TNFRSF10D	DLC1	FAM43A	GFOD2	NOP16
GTPBP2	SFRS2	KBTBD5	CCDC9	ITGA5	KCNE4	NCL	PIP5K1A	TNFAIP1	C3orf59	AKIRIN1
PTBP1	ARC	RAB20	RBMS1	C17orf85						

Coexpressed genes with Myc in KIRC (Stage2)

FOSL2	JOSD1	JUNB	SOCS3	PLK3	ZFP36	ADAMTS1	HBEGF	ADAMTS4	THBD	CYR61
PPRC1	EGR3	NDEL1	THBS1	SNAI1	C5AR1	PADI4	ICAM1	IL1RN	OSM	TNFAIP3
TREML2	MCL1	GPR183	RNF122	IL1B	FPR2	MYADM	CH25H	CD300E	IL8	NR4A3
FLNA	CXCR2	STAT3	KCTD10	KLF6	JUN	F13A1	ITPKC	APOBEC3A	S100A12	SPRY1
IRAK2	DLC1	UGT1A7	ABL2	CCL8	CSF3	EMP1	PLAUR	PHF13	NFIL3	BHLHE40
SKIL	TNFSF18	S100A9	BCL6	LHX8	GMEB1	C14orf165	SOX7	CXCR1	DNAJB5	GPR97
DUSP1	WWTR1	PLEKHO2	NUPL1	CSDAP1	C15orf39	MMP10	FCGR3B	CD209	CCRN4L	CCL3
TRAF3	CXCL2	SRF	CSRNP1	IL4R	CORO1C	GLT1D1	FGF10	CFHR1	SELE	SRGN
ELL	EIF4A1	HTR6	RND3	CCL2	ETV3	KLF10	CXCL3	SFPQ	TSC22D2	CRISPLD2
STEAP4	KDM6B	LOC84740	DUSP6	AMMECR1L	LATS2	SGK1	FPR1	IRS4	FCRL1	EPHA2
CHSY1	KRT75	GNA12	PFKFB3	FAM49A	RELA	KIRREL	LIPN	PLEKHG5	CSGALNACT2	PTPRE

MMP8	FAM157B	JDP2	WEE1	MIDN	NRP2	ST3GAL2	ATOH1	CSTT	OPN1LW	OR5V1
RNU4ATAC	SPZ1	TMEM2	WTAP	LMNA	PHC2	MAFF	TREML3	CYP2W1	SLC13A5	MX2
TFE3	C14orf43	SLC6A5	RND1	CCNL1	PAGE4	CCL4L2	SH3D20	FAM53C	EGFLAM	ZFP36L1
C1orf216	MAP2K3	FOS	NFKBID	CLEC4D	SSRP1	SCNN1B	ATF3	MXD1	NAMPT	FADS1
ITPRIP	KLF4	CCL3L1	CD83	MSN	S100A8	SERPINB8	BCL9L	ARID5A	NCL	CFHR4
PDE4B	TDG	CDC42SE1	AG2	P2RY8	SH2B3	ZSWIM2	LILRA5	EHBP1L1	MS4A6E	AADACL4
LIPI	S100A7	TAF5L	ZYX	MRC1	CEACAM4	IL2RA	HNRNPM	BCL3	NFE2L2	ADAM17
ARL4C	EIF4G1	ACTN3	URB2	SLC2A14	SELL	CASP5	BCORL1	WDR76	PLXND1	CNR2
RASSF2	PER2	EGR4	C10orf10	GPR27	RQCD1	DCLRE1B	FOSB	EMILIN2	TUBB6	DENND5A
MMP12	HMX3	BAZ1A	RELT	RASSF3	C9orf30	TMEM39A	PANX1	FCGR2A	ETS1	PPP1R15B
BCL2A1	TAC4	ACSM4	DPYSL2	GFRAL	ODF3L1	RGS9	NIPAL4	GRRP1	HK3	NUP188
FHL2	SLC2A3	TREM1	CSRNP2	PIM1	PYGL	POLR2D	SCNN1G	SEMA4C	TNKS1BP1	SAP130
DDX3X	KIAA1949	PPP1R15A	YY2	PIP5K1A	PLEKHM2	ODF2	RIOK1	IL13RA2	C10orf54	HIVEP2
TNFRSF1B	OTUD3	SLC25A25	CLEC4C	CCL7	LY6G6C	TNFRSF1A	QTRTD1	SFRS7	C13orf33	MAP7D1
IL10	DDX5	MTCP1	SF1	STK40	DUSP14	RGS2	BARD1	KCTD5	SERTAD2	LOC440563
BGN	CEACAM3	EMR3	FOXC2	CR1	RHOB	MS4A1	SSH1	SP110	CDK2	GABRG3
TNFSF11	REL	ELF4	ASAP1	TET3	BICD1	FFAR3	ZNFX1	NFKBIZ	NLRP3	ZC3H12A
DNMT1	ULBP2	RNF19B	ISG20L2	SEC23A	FILIP1L	LINGO1	RPH3A	MESDC1	MCTP1	TMPRSS4
C19orf75	VNN 3.00	RELB	BTBD19	NOS2	GPR4	MSH6	INS-IGF2	IL13	HSD3B1	GUCY1A2
CSF2RB	HCK	LYAR	GTPBP4	OAS3	VEZT	POLD3	CD14	GPR135	C17orf46	S1PR3
LILRB2	ADAM8	FAM110C	CEP170L	KIF3C	NFKB1	LOC284441	SIGLEC12	PRPS1	CD97	MYD88
SLFN11	SLC15A4	SERTAD1	ZNF597	CEBPD	CMIP	KCTD11	CAMK4	EGR1	CYP2A6	SHC1
ELMO1	KDM5C	CD69	FOLR2	AFAP1	RNASE2	PLA2G2A	MOBKL2C	C12orf68	VSTM1	FRMD6
HTR7P1	CYP7A1	EHD3	MLXIP	LRRC33	SPEN	ARID5B	PPAN-P2RY11	DYNC1I1	NUP98	SLC25A43
EPHB1	TMEM173	C13orf18	BYSL	EIF2C2	KIAA1644	JMJD6	SBNO2	DKC1	TRAM2	C11orf24
NOLC1	MLKL	PRPF4	LOC595101	CCL21	CCL19	LOC100126784	VSIG8	TGM3	PLEKHM1	ACTA1
FBN2	RAI14	FAM20A	STK10	ZBED4	IL7R	LRRFIP1	GYPE	TNFRSF10D	TRPC4	SLC11A1
RALGDS	ZFPM2	LRRC59	MAP7D2	EHD4	FRMD8	CRKL	FLJ36031	CREG2	ACVR1	FOSL1

KBTBD2	GPR176	SIRPB2	IFFO2	TMEM184B	ACTN1	BCL6B	PHACTR1	SFRS2	RIPK1	ASB10
ARHGEF6	IRAK3	MAML1	MMP25	HK1	TGFB3	INSL4	CACNA2D1	P2RX1	CCL4	KIAA1244
ELK3	SULF1	RLF	ALOX15	CLCF1	RILPL2	PTPN1	ETF1	SLC10A1	SIK1	GNAI2
MSRB3	FCER2	DCUN1D3	CSNK1E	OSMR	PRIC285	KRT32	MT1B	IGSF21	DCBLD1	PREX1
DDX21	SULT1B1	IFNA1	POTEE	CHN1	SMAP2	GNA15	STC1	SLC2A1	IPO4	LOC284688
ACTB	MAPK6	CDC42EP2	ADM	KIAA0146	KALRN	PSMD11	PELI1	KCNK3	LOC644936	C9orf25
PLB1	CEBPB	ARL5B	CCR7	IL1R1	DUOXA1	GADD45B	VSX2	FPR3	MT1X	TAS2R40
LOC340017	MAP4K4	KCTD20	SPRY4	EHD2	NONO	CLSPN	MAT2A	SCN3A	AOAH	WDR43
MURC	ZEB2	SKI	ARRB1	ADPRH	CENPI	LRRC32	USP36	SEC24D	KCNA6	TMEM84
TUBGCP3	C6orf150	CSF3R	C5orf58	FIGLA	IL17RA	41153	CLEC4A	AMPD2	UGT1A8	TUBA1A
TNFRSF13C	DDIT4	H3F3C	TSHZ3	KCNJ5	GORASP2	ARRDC3	HEATR1	ABCF1	CLASP1	A2M
BLK	HSP90B3P	NFKBIA	DAAM2	HCLS1	PIP4K2A	KHNYN	CBWD6	AHDC1	GBP1	EPB41L2
RNF217	SERPINB9	HNRNPR	UIMC1	TTYH2	VIM	TBC1D25	SH3BP5	ETS2	SRL	KERA
FAM38A	CSF1R	TWIST2	HIVEP1	PCDHAC2	FCN1	EMR1	MKNK1	PLEK	DLGAP5	GFOD2
APOLD1	CFH	ARID3B	KDM3A	PHLDA1	ZRANB3	CHRM3	RARA	CTGF	STAB1	KHSRP
CDV3	CASP7	NKX6-3	DUSP5	SLCO1B1	CCL13	ARHGEF2	TP53BP2	ADORA2A	NFAM1	POLR3D
ISY1	SAMSN1	SERPINB2	SCN9A	NUP62	ADAM19	ATP8B2	SOX21	TRIM6-TRIM34	ZCCHC24	ERF
TNFAIP1	SPRY2	NES	SUPT6H	FAM118A	RAB35	EMR2	TMEM194B	RRP1B	SMC1A	MMP19
NR4A1	HNRNPA1L2	WDFY2	IDH1	XIRP1	CD300LB	PRPF38A	GBX1	DNAJB8	RNASE1	LAMC3
ZNF496	CHD4	GRK5	DDR2	FBN1	C1S	EPC1	HES1	UROC1	FSTL3	CFLAR
RBMS1	SLC26A10	CXCR5	FCAR	LOC348840	PRAMEF14	LUZP4	C13orf29	LRP1	CHD1	LOC283663
FTO	HNRNPF	MKL1	C3AR1	SLC16A1	SMARCAL1	SAMD8	GNB1	ARHGAP17	ITGB1BP2	MTF1
COL6A3	CHST11	MGC12916	SETD8	SF3B3	GRM7	MTA2	TLN1	PABPC3	SENP1	LCP2
C2orf48	RASIP1	CDC42EP4	LAIR1	FES	GNA13	NFE2	TRIM9	AKIRIN1	MAP3K8	CDC45
APLNR	ADAP2	PELO	LOC154761	INSIG2	LIF	FUT4	IPMK	NRP1	CCDC19	MYOF
TCP11L1	UBN1	ELL2	GPATCH2	JUB	GABRP	ZNF643	C1orf55	SST	PHLDB1	VNN 2.00
DACH2	LZTS1	KCNJ8	DCHS1	ITGA5	RHO	USP37	ARHGAP23	MTHFD2	CHSY3	SFRS4
TNC	TRA2B	PHLDA2	ADAMTS2	VSIG4	PCDHB3	DENND3	RAD51L3	YARS	COL12A1	MKI67

DLX5	GALC	TMEM185B	JAG1	SIGLEC5	ATXN7L3	USP18	RASGRP2	PTBP1	IDI2	CBFA2T3
NFKB2	PIK3R1	ERC1	GPR84	COTL1	MPEG1	CCL23	TMEM49	C3orf64	PMP22	KLF2
PLEKHG1	CD163	PTRF	SLCO2B1	SMOX	TGFB1	SMTN	RASSF1	C1R	H6PD	TRPV2
MGAT2	PPM1F	CCDC105	SFRS15	SLIT3	AGFG1	PDIA5	KPNA2	PER1	ITPKB	GPR161
F9	SUN2	FAM38B	ATF7	MYH9	TRIM6	CASP10	LILRB1	TRIP12	SH3PXD2B	DHH
TNFRSF10A	CDKN1A	CFHR2	C6orf145	CFHR5	NFATC1	CD276	LOC100302650	ARID3C	UGDH	LBXCOR1
OR6C2	OR8G2	TMCO5A	GPLD1	RBM28	C4orf31	FAM55C	ZSWIM4	PCDH12	CD86	UBC
TRIB1	ZNF281	MEGF10	MSL1	BHLHE41	C9orf47	SNX32	NCF2	SART3	AK3L1	C8orf86
TMCO7	NPHS1	C9orf21	RBM8A	HSPA14	CLC	CSF1	BMP2K	FGD5	ETV6	RASGRP4
CD4	SLC24A4	VCL	TERF2	MNDA	MYH2	KLK7	MYOCD	OR13J1	PLEKHG2	SIGLEC10
MTNR1B	LMCD1	C19orf59	DNAH12	TBC1D10A	CATSPERB	MS4A4A	KIF23	MAPRE1	PDE2A	COL15A1
STAT4	MAFK	PDCD1LG2	OR2W3	LYN	ARHGEF19	MTERFD2	YEATS2	IFNAR2	ESPL1	DIAPH2
DRGX	TNFAIP2	FBXO42	TRAF1	C1orf198	PARVA	C1orf183	IL24	GRXCR2	DRD3	POLE2
LDOC1L	CXorf21	SLC7A1	TBXAS1	PCDHGB6	HNRNPA3	DHX38	CD80	HDC	ADCY3	IER2
TBC1D2B	SIRPB1	RASD1	RBM17	ZNF423	LMNB1	ANKLE2	C13orf30	CCR1	C19orf38	LHFPL2
P704P	TANC2	STX11	NDST2	VGLL3	GLIPR2	P2RY2	C17orf107	R3HDM1	UGGT1	CFP
DNAI2	MEF2D	MFGE8	PDGFRB	EPAS1	LOC257358	DSE	CREM	CD53	WDR33	PUS7
RNASEH1	EIF2AK3	TECPR1	CHIC2	RRAD	ACBD3	MATN1	KIAA1210	ZBTB2	SIGLEC9	TEAD4
UBL4B	CDKN2B	TCF7L1	CHD8	TAS2R31	FBLIM1	CTXN2	SP100	PIAS4	ORC1L	SPARC
TFRC	BAZ2A	CDH11	KLF16	NPPA	NCOR2	YWHAG	PSMD1	LOH3CR2A	SYNJ2	ATP2B4
TMEM217	CASP1	GBGT1	ITGAM	PDGFB	TRY 6.00	FOXJ2	SH3PXD2A	BIRC3	OR1L6	FCHSD2
PDYN	PAG1	SGK269	TYMS	EGLN3	PRC1	ARHGAP11B	PDK1	DISC1	DNAJC18	PASK
RRM2	NNMT	ADAMTS7	FAM43A	HIST1H2BL	LONRF3	IFNA6	TNP1	GLI3	CPSF7	ABL1
NOS3	LILRA1	SLC2A6	CBX6	GMEB2	PHF19	DOT1L	CD300C	ZNF641	TXLNG	HDLBP
MGC42105	NRBP1	OR4N4	SCD	B3GNT5	KIAA1462	IL4I1	HNRNPH1	ARHGDIB	BEST1	CD93
LRFN1	CHFR	DBH	EIF4H	TRIM5	TOE1	TNFSF13B	TTLL4	CMKLR1	ARPC2	MC2R
TINF2	THOC5	SPRED3	FAM26E	MYO1F	C1QC	OLFML2B	MPO	WDR47	RBM15	FAM70B
EIF4A3	NYNRIN	MCM3AP	CIC	ATF2	IRF9	GPR3	EIF2C3	WDR26	LOC100134259	FCGR2C

PRIMA1	RBMXL1	RPS6KC1	LOC100131551	CCDC116	PLBD2	SERPINE2	SDC3	C3orf59	KLHL5	FAM102B
SCARNA15	TPM4	ZBTB46	NCRNA00120	LAMA1	PAMR1	UCK2	MYLK	LOC100130331	TMEM154	SLC1A4
LOC643837	SAMHD1	TRIM47	TACC3	PKD1L1	CYFIP1	DAXX	KIF18A	CRLF3	FSCN1	ELK4
LGI2	ALS2	ADH1A	C9orf144	IFNA13	CCL11	GATA6	GPR182	DCTN1	PML	PLD5
ITPRIPL1	PRDM13	F13B	SDS	LCP1	CPB2	THRAP3	ABCB6	INPP5D	ATP10A	RAB39
FAM107A	LYPD5	COL4A2	HMGB1P1	TMEM109	ITLN2	HAL	KCMF1	AOC3	RXFP2	FMO3
TMEM22	RXFP1	ZFX	AMICA1	CBLB	TMEM105	SAP30	MTHFR	CD33	DYSF	FRZB
LDLRAD3	EML1	SNORD1C	FKBP10	PCDHGC3	NLRC4	MS4A6A	F2R	PLK2	FYN	HULC
GPR124	PDE3A	MARVELD1	CDC42EP3	QSOX2	DAPP1	MYO1G	MMP14	CYBB	APOL3	NOP56
STIL	LTB4R2	CDC6	SUPT7L	RUFY4	NAB2	LRRC42	METAP2	PPBP	SEMA4B	KDM5B
CCR8	GAL3ST4	PRKCB	HSPG2	HPS3	AREG	RBM12	TSNAX-DISC1	CLEC2B	CKAP4	OR13G1
GJD3	FUS	GGCX	EML3	PRTN3	SHROOM4	NECAP2				
**Coexpressed genes with Myc in KIRC (Stage3)**										
PNRC1	NFIL3	ZFP36	FOSL2	NCOA7	SLC2A3	UGCG	SLC2A14	TRIB1	KLF6	ADAMTS4
DUSP1	JUNB	ADAMTS1	MCL1	RIOK1	ADM	PPRC1	SFRS13B	FOSL1	SOCS3	EREG
FOS	CSRNP1	DDX21	SNAPC1	ZFP36L1	NUDCD1	ZNF460	EDIL3	ADAMTS9	KIAA0146	CCRN4L
EGR1	WDR43	WDYHV1	STEAP4	SIK1	CHMP1B	YRDC	PIM1	PTP4A1	NFE2L2	NAMPT
TMEM185B	DKFZP434K028	C7orf68	IRAK2	SEH1L	CEBPD	LDLRAD3	TINF2	PATL1	RHOB	EIF3E
EIF2C2	TNFAIP3	FLJ36031	PAK1IP1	NFKBIA	KBTBD2	EIF4A3	RNASE4	CLDND1	BHLHE40	P2RY14
TNFRSF10A	SERPINE1	C10orf119	KLF10	C9orf21	TAF1D	CALCR	NUP98	CHSY1	SERTAD1	NR4A3
PLK2	EIF1B	NOLC1	RNF139	RPL7	IL1RAP	PANX1	IL1RL2	TMEM49	ICAM1	SPRY1
UBIAD1	DUSP5	MED30	CREM	GALC	AREG	MIPOL1	ATF3	NUP153	TFPI	BCL6
EIF3H	BAZ1A	TMEM194B	ORM1	RPS6KA5	NKX3-1	LRP12	ARL5B	ETS2	PFKFB3	NT5E
B3GNT5	TAF4B	MMRN1	PUS7	TMED10	EGR3	ADAM17	EIF2S1	S1PR3	CA1	C18orf20
ETS1	PLSCR4	PHLDA1	MIR17HG	ING1	CCL20	RBM7	ALG9	RBMS1	C18orf19	ZNF143
STAT3	RPS12	CLC	CSF1	MIDN	SLC16A2	STC1	PLAC8	HES1	YWHAZ	ZFAND1
LOC100133669	PCDHGB2	FOXC2	CDKN1A	ERO1L	CCT2	GPC6	HBD	CNN3	FTHL3	DKFZP434H168
HSPA5	SNORA56	WTAP	DDX3X	GNL3	RBM12	LDHAL6B	SEL1L	NUP35	NMD3	CYR61

LOC100131551	RPS3A	SFRS3	RPS29	NETO2	DCP1A	IPO4	XKR9	PNP	HIF1A	SLC43A3
SULT1B1	KCNJ8	SLFN11	ELL	COX8C	NAF1	HNRNPF	ARL6IP6	CATSPERG	TIMP4	GCH1
PDIA6	POLR2D	LOC100127888	PM20D2	DIO3OS	HBEGF	H3F3B	GEM	ZHX2	TMEM43	TMEM217
CNIH	ACVR1C	CHIC2	ST8SIA6	FAM49A	VDAC2	TOX4	PPM1D	C14orf118	NIP7	TMEM39A
TATDN1	SYNCRIP	PCDHGB1	JUN	SAV1	NXT1	HPS3	ACTL6A	RELA	MALT1	TIMM9
HRH1	PTPN12	POU4F2	EXOC6	ZBTB25	TGFA	WEE1	AG2	C14orf43	GNA13	AHSP
FAM107A	USP37	MARS2	RPS13	MCFD2	C6orf25	HSPD1	DUSP6	PNO1	UBA2	SENP2
HBB	SIRPD	EXT2	ARID5B	HBM	LAPTM4B	HSD17B7	MXD1	EMP1	RPL30	CSDAP1
LOC729020	TMEM209	MT1X	SUPT3H	TBPL1	ITPRIP	RRM2B	TAF2	GLIPR1L2	HIVEP2	ASNSD1
HMGN4	TM9SF1	PCNP	CDC42EP4	SLC25A32	MTUS1	ALAS2	GPR4	LONRF3	CCL2	OSMR
TMEM67	PABPC3	SNX31	IER3	TTC35	PPP1R2P3	RPL10A	HIPK3	FYTTD1	IPO5	ZNF295
APEX1	CUL4B	KLF4	TMEM68	ATP13A3	SAMD4A	FAM99B	TSC22D2	SIRT1	UAP1	STAG1
PPP2R1B	FMO2	TCEA1	PTPRE	SRGN	PPBP	RBM15	SLC22A3	TGM2	ZNF649	GATA1
ACTR10	SPRY2	UTP18	PPP1R15B	SPRED2	AKAP14	CD109	HNRNPK	SLC39A6	NSMAF	MRPL33
ANG	RND3	NUP160	PSEN1	ARMC10	LOC221710	NHEDC2	RHAG	EIF3J	PYGL	PRPF4
FMN2	F2R	GLT1D1	HNRNPC	SLC2A1	ALKBH1	NIPAL1	RAD21	NUAK2	SMNDC1	ABCC9
EGLN3	CGRRF1	LMAN1	H3F3C	SNW1	PABPC1	NFYA	FHL2	WDR3	NR4A2	THBS1
YY1	TFRC	PALM2	GPD2	ARIH1	RBMXL1	C9orf47	JUB	TCP1	MGC16025	NCRNA00120
CXCR7	KIAA0196	SOX7	RAB2A	TMEM50B	CXCL3	ATF2	CSE1L	KDM6B	TBCCD1	C10orf10
PRMT5	GOLGA5	IL4R	NARS2	URB2	CPE	DERL1	C2orf49	TMEM74	EBAG9	ZNF331
E2F5	MED17	SCNN1G	S100A12	HEMGN	ZC3H15	CCNI	LDLR	TEX10	OBFC2A	SH3BP5
MT1L	TES	EPB42	ZNF830	FCAR	CNBP	HSP90B3P	CCT4	GABPB1	CH25H	NGDN
PAG1	ZBTB11	SFRS7	HSP90B1	MSLNL	TRMT5	C2CD4B	EXD2	OR2L13	JHDM1D	SLC39A9
IFITM2	INTS4L2	IL1B	NIPAL2	NUPL1	RLF	GOLGA6A	FPR2	TC2N	ZNF484	MID2
NCL	TPP1	DICER1	ANKRD46	RCOR1	TXNDC16	CHD1	EPHA8	JKAMP	LATS2	ZNF706
BAG5	SF1	NGLY1	MTDH	MT1DP	PSMC6	AKAP2	PUS3	NOG	EIF1	VAPA
LOC100130264	MAPK1IP1L	STT3B	EAF1	ZCCHC2	ZC3H14	ZNF410	CLCF1	DAD1	C20orf199	WIT1
SSRP1	ABCE1	S1PR1	GPR135	KIAA0020	TTC9C	DPAGT1	INSIG1	C14orf135	PPIL4	CAV1

ERRFI1	MAFF									
**Coexpressed genes with Myc in KIRC (Stage4)**										
KLF6	JUNB	KLF4	DUSP6	DUSP1	MCL1	PPRC1	MAFF	HNRPDL	NFIL3	ZFP36
FOSL2	HNRNPH3	WDR43	PPP1R15A	ITPRIP	TTLL11	DDX21	SF1	RIOK1	DDX5	CHD1
MTERFD2	LOC100302650	DDX42	SOCS3	C17orf46	HES1	HBEGF	OR56B4	C9orf21	CDK17	STC1
B3GNT5	SPRY1	NAB1	GTPBP4	ETS1	CAMSAP1	JMJD6	SH2B3	RPGR	HNRNPA3	ARRDC3
MIDN	RBMX	EFNB2	ELK3	FOSB	SPRED2	CSRNP1	EPAS1	SOX7	PEAR1	MAT2A
BHLHE40	PCDHA7	SLC2A14	USP36	NOC3L	SCN4B	HNRNPH1	SLC2A3	SFPQ	ZNF833	GRIK3
KLF10	TGFBR2	C10orf119	KCNE4	KDM6B	EMP1	NOLC1	PIM3	LRIG2	SPRY4	EPN2
LRRC70	LOC440354	ZNF343	RPS6KB1	EXOSC2	F2RL3	DDX3X	SNRK	SFRS3	EPC2	LOC641367
RARA	PTPN12	DLL4	HMGXB4	NAF1	INTS2	KLF9	ECD	ADM	CCRN4L	NDEL1
GPR3	DNTTIP2	LOC286094	MLLT10	RAPGEF1	TMC7	KBTBD2	ZFAND1	PRDM10	C16orf52	RCOR1
PFKFB3	DUSP5	SH3TC2	C11orf94	RUFY2	ANKRD40	TMEM22	GRB10	ZNF462	GALNTL6	URB2
PCDHB4	NFX1	SEMA4C	KCNE3	HYMAI	NR4A2	FOXC2	PPARGC1B	LMNA	TNFRSF10A	TM4SF1
PPP2R2A	DEFB118	NEK5	TUBGCP3	STARD13	LOC344595	TAF1	ATF3	ZNF263	S1PR1	CCDC46
EIF2C2	PACRGL	C10orf10	INVS	DHX15	H3F3B	CST11	ARAP3	TAF5L	CPSF6	RPS6KA5
ORC2L	ZNF143	VIPR1	RGS9	MDGA2	SFRS7	TIAL1	AMMECR1L	GPATCH2	GPR135	ABL2
SPAG9	NUP85	KLF7	VPS13B	APOLD1	PMS1	DLC1	METAP2	AP3M2	AATK	IRF2BP2
ZBTB49	AGTR1	TCTE1	CWC22	OTUD4	SFRS13B	THUMPD2	SFRS1	HNRNPD	FGFR1OP2	RND1
LONRF3	CDNF	KIAA0355	ZNF507	SPRY2	MAK16	HTR7P1	OTUD3	LOC338758	IL1RAPL1	B3GALT1
TRIB1	OR2H2	LYAR	RFX3	BDP1	RAPGEF5	GPR4	LOC100129716	PAPL	FAM160A2	NCRNA00120
RABGAP1	FRMD8	BAGE2	C9orf93	CHML	CEP72	DDX50	POLR3D	DGKD	POLR1B	AKAP2
UIMC1	PCDH17	ZNF7	TXLNG	TMEM49	C3orf59	TAF5	JOSD1	SAMD8	ARHGAP29	ASAP1
MAP2K3	SLC35F1	SFRS2	ABI2	C10orf78	BCL6B	LDB2	ETS2	ZNF25	CNBP	CEP68
PKD2L2	ZMYM5	ZNF295	LPL	FRZB	ZNF202	TRA2B	SNORD1C	C1orf55	TAF4B	GGNBP2
EPC1	MAFG	SMC5	SLC26A10	CXorf36	RHOB	PRPF38A	NFKBIA	TMOD2	PGLYRP1	MCM3APAS
OTUD6B	DUSP12	JMJD1C	CLEC1A	WEE1	RFX8	ABCG2	RGS5	RAPH1	SDHAP2	FOS
ISG20L2	DOCK9	NOP14	C9orf144B	WDR20	CDKN2B	PGAP1	ANKRD36B	ZNF496	PHLDA1	NRP1

INTS6	ANKAR	ATG12	JUN	DIAPH2	IRGC	GFOD2	SMURF2	CEBPD	MYADM	C20orf194
IL4R	CSGALNACT2	ZEB1	THRAP3	LOC100268168	PHF3	SHROOM4	GMEB1	NCRNA00169	ZCCHC2	SPRED3
CCDC55	ZNF333	RP1L1	SBF2	KIAA1328	SWAP70	ANO2	MIR17HG	DHX38	FLT1	TLK2
SUGT1P1	RFX2	HMGCLL1	SEC14L1	MYCT1	LOC652276	FFAR2	LOC595101	THBD	MAP4K4	HNRNPK
PDE9A	C1orf107	C18orf2	PIWIL2	SHE	MYO1B	EPGN	BMX	TAF1L	MAP1D	MCF2L
SMC6	UBA2	GADD45B	C9orf72	FBXO11	PDE4B	BBS7	ADAMTS1	PLEKHG1	PIBF1	C2orf86
PLXNA2	SECISBP2	TEX10	LILRB5	GDAP1	NUP98	KCNAB1	PLK3	VEZF1	ARL6IP6	NOM1
C20orf112	ARID4A	ZFP36L1	COIL	PNN	CXCR7	POLG2	HCFC2	FBXO48	FAM122A	SLFN11
ZCCHC6	CCNB3	ABL23	SNORD89	SRGAP2	RPL7	PITPNC1	YES1	AHCTF1	KLHL10	EGR1
SP3	WAC	NR4A3	STK40	ZNF484	SPIRE1	DIS3	SIRT1	ADAMTS5	PRTFDC1	OR10K2
SLED1	TBCCD1	ATAD2B	FRMD3	FUBP1	CCL2	IRAK2	C9orf47	DCT	TMEM150C	LOC100271836
SF3B1	SMU1	TACR1	C11orf20	HNRNPM	NAA25	SCARF1	EPHA2	PCDHGA1	RLF	HNRNPU
SPARCL1	STAT5B	FLT4	PGM5P2	IER2	LATS2	SPEN	NOP58	TMEM146	SNHG4	DENND3
ZNF500	TASP1	MYSM1	VEGFA	PKD1L1	ANKRD31	CDC42EP4	TRPM6	INHBB	C9orf150	DOT1L
SPATA3	PSMD12	ATL2	ZNF830	QTRTD1	PHOSPHO2	ATP6V1G2	ST6GALNAC1	EIF4A1	NCOA7	OR52J3
GNA13	MAP3K13	SLC10A6	SGK1	UTP6	MTPAP	ERF	NUP153	ING1	ATP1B2	MORC3
AEBP2	PTPRE	CCDC83	OR51I2	REV1	ZNF207	GEMIN4	NOTCH4	SART3	JAG1	POLR3E
GAGE2A	ACAP2	SMTN	APBB2	CSF3	LOC221122	C14orf43	RIC8B	FAM119B	C9orf25	TNFAIP8L1
TARDBP	IRAK3	BMS1	PALMD	KDM4C	PGM2L1	OR8H1	C18orf25	GABPA	PLVAP	VENTXP7
POLR1E	PCM1	SV2C	PRPF4B	DENND4C	LHFPL4	NUDCD1	C12orf76	C15orf39	B4GALNT1	MSL1
HERC4	GIT2	DAND5	GPCPD1	ZDHHC17	CFLAR	PPP3CC	KHDRBS1	SERPINB5	KPNA5	CSNK1E
LOC641298	RNF160	CXCL2	TPO	SMNDC1	CCDC9	HDX	NFYA	SRL	DOCK6	PCDH12
ETV3	IGSF9B	C3orf50	HNRNPA3P1							

Table: S6: List of coexpressed genes with Myc in KICH, [KICH=Kidney chromophobe]

**Coexpressed genes with Myc in KICH (Normal)**										
AATF	ADAMTS7	ATP8B2	B2M	KCTD4	KIAA1683	KLHL30	LAIR1	PAPLN	RNASE6	SKI
TTYH3	KBTBD5	ITIH1	CRLF2	PTTG2	SOX21	FAM138B	ACER3	C1orf126	CD101	CTTN
RALGDS	RAPGEF2	ZNF134	C6orf222	CASQ1	CD53	KIAA0922	LSM 12.00	NAPSA	NPDC1	PCYOX1L
PROK2	PSTPIP1	PYGM	SLAMF6	STK17A	TFPI	UTF1	ABL1	ARRDC4	ART4	GPRC5A
IMPDH2	NOS2	PARP9	TXLNB	UPK2	SYNPR	AGT	BEND3	BIK	CELF4	CHD7
CORO7	LRRC3	SLC1A4	CNN1	CTGF	MGC29506	N4BP1	UTP6	FSD2	SCRT2	TAS2R60
BCAN	C1orf9	CWC25	ECEL1	EMB	MAML1	PQLC2	WDR69	YWHAB	TNIP3	LOC153910
APOC1	CHML	CLN6	CSF1R	GALNT7	GBP5	NCF1B	NLRP7	SAMD9L	SLC1A3	CSDAP1
CXCR2	GFRA2	GZMK	HNRNPA3	HUNK	KIF7	VASH2	FAM74A4	CCR1	CXorf21	GRM2
MAN2B2	PRNP	TREM2	ADRBK2	CELSR3	FOLR2	GRWD1	MBD6	PLEK	PTGIR	PTPN22
CFI	DPY19L1	LOC100128542	NKD2	S1PR4	TMEM43	TRIM21	ABCC3	BRCA1	BRD1	CCL13
CCRL2	CXCL3	GPR114	LRRC8B	MOV10	NHEDC2	PMM2	USP38	POU4F3	PLAC1	BIN2
CBL	FEM1B	GDF5	LYPD5	MORC4	VSIG1	ZNF146	C1orf92	FCRL1	ADA	C7orf57
CCDC86	EMR1	IRF6	ISLR2	JAK3	JPH1	PCSK1	PPDPF	PRKCSH	CSF2	PRAMEF8
FGF19	APOBEC3A	CD247	PSMD7	PXDN	RASSF6	SEPN1	SLC26A2	SLCO2B1	SOD2	WBP11
ZNF395	SNORA76	GKN1	CAPZB	CARNS1	CENPA	GZMB	LIMK1	LOC100126784	PCDHB2	PSMB2
SEC16A	SORBS2	DKFZp566F0947	TMEM14E	AZIN1	DOK3	FAM177A1	FZD8	HMGB3	KIF14	LRRN4
NFATC2	RFTN1	STX5	TCL1A	GJC2	GPR56	IER5	KCNA6	RNF2	SLC35C1	TPSB2
SNTN	ARF4	EEPD1	FAM110B	GCLM	IRF7	KDM5A	SH2D3C	TRPM2	WFDC1	MS4A6E
CSNK1D	FAM72D	SLC30A2	TM4SF4	C12orf70	APBB1	MKNK1	PVT1	SIDT2	SPARC	TMEM22
TOP1P1	ZNF469	ACVRL1	C2orf39	C6orf154	DNAJB13	HLA-F	RNF40	FAM177B	OR2H1	FEV
CCL17	BEX1	CD99	CLDN1	DMPK	FCGR1C	HPX	IL6ST	LRRC41	NOTCH2	OMD
PA2G4	PNO1	SOBP	TMEM57	DFNB31	FAM159A	FAM49A	HSPA14	LARP1B	PITPNM1	ZBP1
IRGM	ADCYAP1	CGA	COL6A3	FAM179A	PYCR1	SLC22A17	TPSAB1	RPL23AP32	CFB	FKBP1A
GPC4	HAS2AS	PDZD8	PRPF38A	PRDM13	SNORD15B	TUBA3E	C1S	LY6E	MARCO	MORF4L2
MVP	OTUD7B	TMEM98	TRPA1	WIPF1	ATP6V0A1	CALD1	CCL21	CDC37L1	COL17A1	FPR3
JPH2	KRT80	MAP1LC3A	TXLNA	UBP1	CDKN2BAS	ZAR1L	C19orf76	C6orf62	CCDC135	CKAP4

FOXO3B	HMGA2	KIAA1324	RNASEH1	C4orf6	IL1F7	EMILIN1	FLJ23867	IGFBP4	PM20D2	SELL
STAT6	TBX15	ZCCHC6	OTOF	ADAP2	ARHGDIB	DDA1	IMPDH1	SF3A1	ZNF828	ADORA3
MICALCL	PLXDC2	TRAPPC2P1	IL22RA2	BICD1	EPB41L2	HNRNPA1	KLHL25	MAN2B1	PLA2G4C	TPM2
MMP10	GABRR3	NKX2-6	RNASE8	SNORA46	FMR1	LY86	NPTX2	SLC24A3	LIPN	EFEMP1
HR	NKX3-1	NRG3	PDIA4	SNORA67	B4GALNT1	DYNC1LI1	GHRL	MX1	PIGR	XPNPEP1
TEX19	PRSS21	ARMC3	ASGR1	DULLARD	IL10RA	MC1R	NRP1	PROCR	RLF	RNASE2
RUNX3	UNC13A	FLJ43950	ORM2	ARRB2	FKBP1AP1	GLI2	IL11	ITGA7	LOC100134259	PLAC8
PPP2R2A	PSMD11	CPLX3	FLJ39609	CD4	DTNA	ECE1	FNDC1	ITGA1	NCF4	NRXN2
PDGFA	POLR3C	STOM	GPR142	CLEC4C	CEP55	EIF4G1	FBLIM1	FBXO46	GLB1L	IL32
MFGE8	SCARNA16	CD37	ETV4	GPR4	HAVCR1	LAMA3	PABPC4	PLXNB3	TLK2	UBAP2L
TREML4	KREMEN2	C15orf48	CD8A	CDCP1	DYNLL1	NLGN1	PDE12	SDK1	SEC31A	ZC3H3
SLAMF9	C6	ECM1	ITK	RC3H1	TREML1	DENND5A	ENTPD2	GBP2	KIAA0355	MGC16121
TECTA	SIRPG	CEP170L	GNB1	IGSF21	IL27RA	KLHL18	SAMD14	SIPA1L2	TUBA8	HUS1B
HTR6	ATP13A3	C15orf21	CXorf22	CYP21A2	LDLRAD3	PAPD7	TNKS2	VNN 3.00	AIM2	DEFB109P1B
NRIP3	SGTB	ZFP28	APOC4	CPA2	AEBP1	CRIP1	DDX60L	EXT1	KLHDC7B	PTRF
STAT5A	MYO1A	ACTA2	EXOSC9	HLA-DOB	IGDCC4	TAL1	KRT5	FFAR3	ALOX5AP	CRLF1
EMID1	PCDHGA4	SERPINE2	TOE1	KPNA7	RAX	GIT2	ITGA2	ITIH3	KATNA1	TEP1
BAHD1	C16orf52	CAPZA1	GPR176	LST1	PMP22	PRKD2	QSOX1	RGS4	SDC3	APPBP2
FAS	FIBIN	GPR172A	ITM2C	KIF19	LDOC1L	MICB	ZNF516	ZNF800	CD207	ARHGAP10
C2CD4B	FCGR3B	RBM15	TXNDC5	VCAN	ZNF513	C3	COL5A2	FAM23A	HGFAC	HTR7
MPEG1	NOP56	NR4A1	PARVG	PTP4A1	RAB36	SIGLEC16	STC2	REG1A	MYL2	C2orf55
FRMD8	KIF18B	MPZL2	NHS	PRAM1	SULF2	GUCY1A2	NRBP1	NUP188	SMPDL3B	TLR6
ZHX2	DEFB103B	GMFG	MALT1	MAP3K11	ARNTL2	BMP1	CLEC4E	KLF3	PDZD4	RAC2
CDR2	CXCL10	FAM105B	JAM3	LILRA1	MNDA	MRC1	PKDCC	SKIL	TMEM200A	ZNF259
NKAIN1	PLA2G2D	ABHD11	CDK5R1	ELMO1	TTN	CD44	CDH3	DAZAP1	MANF	TTC7A
ARHGAP31	LILRB1	MGAT5B	NRN1L	SIAH2	SLC15A3	STAT4	KLF14	CASKIN2	FSTL1	GPR37L1
IL13RA1	STAC3	TNKS1BP1	WDR1	C2orf85	C6orf150	HCK	RBPMS	ALAS1	MEGF11	MYO1E
NUP107	PTPRG	TSSC4	UHRF1	CD1A	PI3	CDCP2	FGA	KIAA0182	LOH3CR2A	PLOD3

RIMKLB	SPRED3	CSNK2A2	LOC606724	SERTAD3	EFNA2	SP8	ADM	C19orf21	HCST	TGIF1
TYROBP	CTLA4	ARHGEF38	C2orf89	EFCAB4B	PPP1R10	TMEM149	COL16A1	KDSR	KIFAP3	STIP1
LOC100129066	TREML2	FAM129C	HERPUD2	KHDRBS1	MYO1G	ZNF267	ZNFX1	TRPM8	C9orf71	OTUD5
PDE4D	SLC6A20	SMARCD1	UBE2R2	CA9	COL1A1	DACT1	ELOVL2	IQGAP3	PMEPA1	SLC35C2
TNIK	WISP1	FOXI3	CHST3	FCN1	FHAD1	RPH3A	TNFRSF13C	ARL5B	CPSF7	EDAR
RNF145	VWA1	ZNF263	ADD2	AFAP1	C3orf59	CBLB	KIAA1045	PCDH17	PTAFR	S1PR5
SLAMF1	ADAMTS8	FAM26F	HLA-DMA	KCNMB1	SLC34A2	SWAP70	BLK	CCDC88A	COL18A1	CRELD2
CTSG	MGC45800	MTNR1A	PARVB	SIGLEC1	YARS	ADAMTS2	GNAI2	OS9	POLR2D	RBM12
RUNX1	ETV3L	MCAM	EFEMP2	FHL1	LMBR1L	PAG1	STAT1	STK35	ZBTB10	CCR8
B4GALT4	FSCN1	NLRP12	TIMP3	EGR4	S100P	CD209	CLEC5A	FKBP10	IRF9	MYL9
PPP1CB	RASGRP4	TP53INP2	ZNF622	NKX6-1	FCRLA	C1QL2	AHR	CDH6	FBLN5	INHBE
NT5DC3	SEMA4C	TLR9	TMEM86B	TSHZ3	SNORA74A	COL5A1	CTSD	GFAP	GOLGA7B	IFITM1
MICAL2	MYO7B	PTX3	SLC1A5	TNFRSF21	AMICA1	CDC5L	CTPS	PLEKHA1	SDPR	TMEM151A
TULP3	ADAM17	EMP1	NCKAP1L	TCEAL5	DKFZp686O24166	FAM72B	FLJ16779	GNG4	PHF20L1	SPAG6
UTP14A	WDR63	ANO9	CSF2RB	CYBA	FST	GNB2	ITGB1	MYD88	SCEL	HSF5
CCL8	CD276	HLA-H	C1R	NDC80	ROR2	SIGLEC9	SLC3A2	TOX4	XBP1	ZBTB16
F3	FYTTD1	MYO1F	PDE4B	SH3RF3	URGCP	CAPN11	COL15A1	NAPSB	NAV2	SOX4
SPOCD1	FAM189A2	FAM46A	PLEKHN1	PRSS23	LOC100130386	CEACAM4	DGKD	GARS	HGF	NLRP3
NUDT11	PLCXD2	SLC7A5P2	SP2	C1QA	CYTH2	HNRNPA1L2	LOC401093	MAGED4B	RAP1B	EHD4
GPSM3	NLGN2	TMEM155	C22orf31	CD226	GJB2	KLF9	SLC39A14	STMN3	ZACN	HEMGN
FABP2	ALPK3	EDEM1	RPN2	SLA2	TPM3	CD83	CDK2	CKS2	PRDM4	ZDHHC22
PTCRA	CSNK1A1	CXCL2	DPEP2	FCGR1A	HLA-DPB1	HSPA8	PGBD1	SCUBE1	ADAM8	CD28
CD300LF	FAM65B	MFRP	MFSD2B	OSGIN1	UNC13D	BCL9	IFNGR2	NCL	NFKBID	SLC27A3
TES	TMEM39A	IGFBPL1	BIRC7	C10orf4	C1QTNF6	CXCR1	GPCPD1	UCN	USP42	OR2B11
C2orf18	FBXO45	FOXO3	RAB8A	ARX	CD68	PABPN1	SIRT1	TRIM67	WNT9A	TNFRSF9
CDK12	CHI3L2	CNIH2	EPSTI1	FFAR2	LMNB1	MOBKL2C	RHBDF1	SP110	TAP2	TEAD4
DMRT1	CYP24A1	HLA-DRB6	SIDT1	SLCO2A1	SOCS6	ZNF365	DUSP4	FERMT3	HIVEP3	NLRC5
REL	PGLYRP1	FKBP11	FPR2	LAMC2	SERPINB1	SIGLEC14	ADAM20	PRPF38B	SELPLG	SPIB

ATF3	CLDN9	FGD2	KPNB1	NCOA5	RASA4	TDG	ZBTB8A	EMR3	BATF	C13orf31
EDN2	GPR19	FAM5B	ADAM12	APOL1	CD300A	ARHGAP15	FNDC4	GMEB1	LPGAT1	RASEF
RETN	CTNND1	MMP2	SIGLEC5	VANGL2	CD38	CDK2AP2	FAM107A	KCNK7	NOP16	PDP1
CD5	EIF2S1	FCGR2A	G6PD	HPSE	IFITM2	MAGED4	PIK3R5	PPP2R1A	TRIM39	YTHDF2
PSMD12	SF1	TMEM39B	COL7A1	MARCKSL1	OSBPL10	ROR1	RPGR	ARL 9.00	CYR61	MS4A7
PAX9	CPA5	APAF1	CST3	GPR171	MIR155HG	MYH9	TNFRSF11A	RPE65	CXCL5	DOCK2
DYRK2	MICAL1	NIPAL4	PLCH1	DNAJC5B	KCTD20	UAP1	ZMYND15	ABCB4	BTBD19	ELL
GPR153	HYOU1	ROPN1L	VASN	GPA33	C6orf174	KRT86	LILRA4	CD63	CECR6	PER2
PLTP	RAB32	RIPK1	FAM160A2	TCP11L1	ZNF653	VAX1	ATXN7	FBXO28	MGEA5	OTUD4
RASA3	CCL23	LCP1	LRP10	PRPF4	IL31RA	C22orf34	EPPK1	PHLDA2	UNC5CL	COL6A1
DUS3L	HAS2	KALRN	ADAMDEC1	CCNL1	LTC4S	PHC2	IER3	KRT17	SLC18A2	TAGLN2
ZFP57	BMP2K	C3orf36	CD86	HRH2	MYO5A	PLA2G7	RAPGEF5	WTAP	OR1F2P	HLA-E
PLEKHG1	SH2D5	CSF2RA	FLNC	PAPD5	SLC10A3	TLR4	ARL13B	BZW1	C10orf46	COL6A2
HIVEP1	KIAA0247	LRRFIP2	MCM6	NFAM1	PHF17	RCE1	SSC5D	C12orf5	DNAJA1	LILRB5
MAPK7	RSPH4A	SAR1A	SV2C	SLCO5A1	ARHGAP30	C21orf63	GPC1	KITLG	WFDC2	BRPF1
CCR7	DCUN1D5	DIAPH1	GATAD2A	GTF2E2	IWS1	KIAA0020	BMP15	ASB11	AKAP2	ANKRD53
CSPG5	E2F6	EMILIN2	SERPING1	YES1	PADI4	DMRT3	ALOX15B	E2F3	FNDC3A	IL6R
KCTD11	LOC100302650	MDM2	NINJ2	RNF4	RPF2	S100A9	C9orf140	LUZP1	SPRY2	DENND3
ENTPD7	SYTL3	THBS2	MUC12	C1QB	KAAG1	KCNJ14	LAMA2	LTF	RPS6KL1	ARPC5
CYB5R3	GNA15	HLA-DQB1	HNRNPD	MS4A4A	ODF3B	ZFYVE27	ACTC1	FCER1G	RAB31	STAB1
DKFZP586I1420	HDC	INTS6	KIAA0802	RNF31	SLC16A3	TBXAS1	ORM1	C13orf18	CHSY3	LYVE1
SPG20	ZFAND5	DONSON	EPN2	HLA-DQA1	SLIT3	NCRNA00164	CD70	ANKRD50	INMT	PDLIM4
TMSB10	TRIM26	VPREB3	KRT75	C12orf77	AMOTL1	C6orf118	HECA	HKDC1	KCMF1	KIAA1199
NCF1	S100A8	SEC23A	SNAPC2	CXCL13	LILRP2	PHLPP2	PTPN9	SERPINA3	SH3TC1	PARP8
WEE1	CMTM2	DARC	GEM	GGT5	ICOSLG	IPCEF1	PIP4K2A	ANXA1	COL4A2	LIPG
TMEM158	C4orf51	CCDC50	HGSNAT	KLF5	KPNA4	NCF2	PAPPA	PDCD1	RLTPR	DEFA1B
ADNP2	CSNK1E	HLA-DRA	MMP14	PIM2	SNIP1	SUN2	TBCCD1	ATP2A2	HK3	MT1X
SHISA2	CD163	DNALI1	SH2B2	UBE2D1	ZFP36L2	CCL20	CDR2L	CLIC1	ITGAM	MAPK6

RFX2	PPAN	REPS1	VASP	YY1AP1	C11orf21	C13orf29	C17orf91	MAFK	NCEH1	OSBPL11
SEC23IP	TSPYL2	YPEL4	BIRC3	C1orf106	CNN3	EIF1	IL7R	LIMD2	YTHDF1	KRT33B
AOAH	CD300E	CHD4	EGR3	HS3ST1	PHLDA1	TRE40057	SH3BP5	SHISA5	SPI1	ZNF597
DLX2	MKNK2	RAB21	SETD8	AQP9	ETV5	MFHAS1	PIGT	TGFB1I1	IL18BP	TBC1D22B
TEX10	MAPRE1	NRAS	RASL10A	CILP2	HS3ST2	MSI1	SLC25A45	TRIM9	ZCCHC2	APOLD1
C1orf198	CTTNBP2NL	CD300LB	CHST11	IPPK	PPP3R1	SPON2	AURKAPS1	CSF1	SIGLEC10	WAPAL
FUT7	CAMK2N2	FBRS	MYBL2	MADCAM1	C18orf8	RBBP8	TLR2	TOX2	MOBKL2A	ODF2
SH2B3	TUBB6	TPO	DDAH2	ITPRIP	OXTR	PPP2R1B	TMEM51	UBTD2	KLHL6	KRAS
LTB4R	PLEKHO1	RPIA	ACTN1	TJP1	TNFSF9	CCL7	EMP3	FCGR1B	MEPCE	NKIRAS2
PTHLH	ADAMTSL4	IL2RA	MAP4K4	NAF1	NAMPT	TNFAIP8L3	ZBTB43	DDHD1	DDX3X	IL18RAP
PTRH2	GLIPR2	RND3	TUFT1	FAM110C	HSP90B1	IL10	PNRC1	VSTM2A	ADAM9	CD97
IRF2BP2	ADPRHL2	IFI16	LILRA5	SLC7A2	CEACAM3	ELF3	FOSB	RRP12	THOC4	TRAF1
MGC16025	CFP	IL4I1	RAB35	ZNF165	C22orf9	CD248	CHORDC1	DAPK3	IL1RL1	LSP1
VCL	CPM	MID1	SULT4A1	CLEC17A	CD2BP2	HMGA1	HSPH1	NFATC1	PCDHGA12	ARG1
ACVR1	ASPHD1	GADD45B	TAF5L	FOXP3	RBM38	SCXB	SFRS15	ULBP2	LRRC8A	RAB33A
SENP2	BCL9L	RAB43	BCL2A1	CXCR5	GPR84	RGS3	AKIRIN2	C12orf68	SLAIN2	TMEM173
CRK	ICOS	LOC100129550	PLEKHA4	WDR43	C1orf162	ESYT1	S100A12	GAR1	MAT2A	TRA2B
GLIPR1	PLD3	ZC3H12A	ACTR3	C9orf25	MAP3K6	C1orf130	EMR2	GADD45A	KDM5B	BRD4
IFNGR1	IL18R1	MAP1LC3C	UBAP1	C17orf96	CCNJ	U2AF1	C19orf35	CXCL16	HSPB8	LAPTM5
NID1	TUBB3	USP36	AHNAK2	FOSL2	GTPBP4	LILRB4	LONRF3	REXO1	ACTG1	IRAK2
NUMBL	STK17B	TUBB2A	FAM18A	ZDHHC1	FCAR	MTF1	NUPL1	PICALM	RBMXL1	CMTM7
PITPNC1	SH3RF1	IFFO2	MLKL	MT1E	RAI14	C1QTNF1	C9orf109	CBFB	CTXN1	RASD2
WARS	MYBPC2	CCDC109B	ITGB6	RCAN1	B3GNT5	HSPA5	TNFSF8	FJX1	OSMR	ATF5
CFL1	ITGB8	RAB20	EBI3	WAS	SERPINB2	ATG9B	FOXO1	MEFV	PDE3B	SH3BGRL3
SLC17A9	TP53	ACTN4	BACH2	CXorf49B	INPP1	PANX1	PDIA3	PSEN1	RASSF2	GPR37
NFKBIA	C19orf38	NGFR	RBM7	TCF7	HLA-DOA	PTPN12	PEA15	S100A3	C9orf21	CDC42SE1
LOC541471	PAK3	KLF4	OSGIN2	TAGLN	GPR132	PLEKHG2	WDR82	WWTR1	CDK17	CTSZ
GATA6	IRAK3	OPN5	ADAM19	AREG	CD3EAP	MSC	ATF4	BTN2A2	PCDHA4	RIN1

SH3GL1	CLEC4D	NBL1	ANGPTL4	CHIC2	CST7	CYTH1	HLA-B	LRG1	TIPARP	SMNDC1
A4GALT	BAG3	PPARD	PVRL2	EAF1	COL8A1	FMNL1	SLC10A6	TYMP	B4GALT1	DUSP5
ERRFI1	ABL2	RIPK2	RNF138	SMURF1	STXBP1	ANKRD13C	CENPN	CD7	INHBA	PSTPIP2
STK40	COL23A1	LOC338651	SULT1C4	UBC	URB2	C14orf80	HNRNPAB	ZNF672	GPRIN1	LILRB3
SLC25A32	DMP1	CDC42EP4	NFE2	NR4A3	UBE2S	ARCN1	CD74	MYEOV	RNF149	C19orf59
TAC4	C10orf108	MALL	SPRED1	CXCL1	EFHD2	TNFAIP6	SF3B4	YWHAH	YWHAZ	CMTM3
ELOVL5	FPR1	MNT	NFKB1	PCBP1	ITGAX	SECTM1	TRIM38	FKBP5	PLAGL2	SPRED2
FAM72A	TUBA1C	CLDN23	MET	SAP30BP	SH2D2A	SMAP2	TGFBR1	UBE2I	ARFGAP3	FAM131A
S100A2	TNFAIP1	TRIM36	C22orf24	AFF4	F2RL3	NACAD	NFE2L2	CSGALNACT2	CSRP1	DEGS1
FAIM3	GAB2	HAUS2	IRF8	SFN	CYP19A1	LTA	GNAI3	NUFIP2	SEC24D	SLC25A25
ANP32B	C9orf110	CEBPG	SEMA6A	SNX9	C2orf77	ENDOD1	SEL1L3	SLC2A14	TNFAIP2	TRPV2
NMU	CCIN	IFITM3	IRF1	SENP5	PAQR4	VPS37C	DDX17	ERN 1.00	SYN1	JUND
PRIC285	C14orf43	CNR1	RRS1	TLE1	TRAF4	COMP	FILIP1L	MX2	NUP98	ANKRD57
CLEC1A	KRT18	TRH	CHD1	MTHFD2	SEMA7A	PTPRE	RBBP6	SMOX	TNFRSF10A	PAQR9
PRDM1	TRIB1	CABLES1	ZNF367	BMP2	CWC22	OASL	OSCAR	CHRNA10	FOXP4	HP
PVR	ZSWIM6	B3GNT7	IDI2	PDGFB	PHF13	TNFRSF6B	BHLHE40	GNA13	PIM1	ACBD3
DDX27	FAM59A	MKI67IP	NAA15	NEXN	PIM3	RHPN2	SFPQ	CSF3R	FERMT2	KRTAP5-1
MFSD2A	UGDH	C5orf58	CCDC96	DTX3L	GRK6	PAF1	AXL	CITED4	PDLIM7	C11orf88
SLC32A1	CAMSAP1	OSM	DCLK1	PPP1R15B	SFRS13B	LILRB2	BACH1	IL8	KIAA0226	TRIM47
TUBA1A	VCAM1	IL24	AEN	EREG	FAF2	MMP25	PALLD	VPS37B	NFKBIZ	SLC16A6
CEBPD	CSRNP2	MAP7D3	MMP9	ELK1	HCLS1	IRF4	TREM1	FLNA	KLF10	ADAMTS9
NCRNA00152	C19orf22	GPR97	PGM2	RHOG	SERTAD1	SIK2	ZNF189	LSR	SPRY4	JOSD1
DCP1A	SACS	CCL22	IL1B	MED26	PNN	STX11	ZFP36	GNL1	DYRK3	CPA4
GRRP1	PURB	SMTN	SERPINH1	UBE2H	ITGA5	ATP6V0A2	ISG20	ROBO4	CD81	SYNC
DOT1L	RNF103	FAM100A	LILRA6	TNFRSF10D	COL4A1	SAMD4B	CHRNE	MAFG	CRISPLD2	EGFLAM
FGR	FZD5	LMCD1	S1PR2	TEKT1	BAZ1A	CD93	PPP1R15A	RAET1L	KRT16	MESDC1
SMARCA5	ANKRD1	ZNF787	B4GALT5	EIF2C2	KLF7	MED17	SPRY1	ARL8A	MEST	ACHE
ISG20L2	ZDHHC5	S100A16	UPP1	CCDC9	HSP90AB1	NCOA7	TGFB3	GFPT2	37681	NOLC1

SGK1	NUDT10	POLR3D	DCUN1D3	SGK223	DKFZp761E198	PER1	SYNCRIP	APOBEC3B	UTP3	HSPA6
PLB1	C5AR1	RELL1	ACTB	HRH1	TIMP1	HIVEP2	LZTS1	VGF	RBM24	TOB2
MAP7D1	AMMECR1L	DNAJB1	FADS3	BCAR1	MEX3D	GPR183	KIAA1949	SEH1L	TNFRSF18	ATP2B1
DUSP16	NNMT	MT1M	C1orf38	TOP 1.00	MTA2	PRR24	STAT3	STEAP4	ARL 14.00	SOX7
TAP1	BCL10	PGM2L1	IL6	RND1	HEYL	SAMD4A	STRN3	C19orf26	XPO6	FGFR1
RELA	SRPR	MAP2K3	PSME4	CDKN1A	FAM83G	KIR2DL4	OBFC2A	LATS2	NOP58	COL13A1
CCL2	EIF4A1	MAPKAPK2	ZNF295	EPHA2	HAPLN3	NPC1	PRR7	ELL2	SBNO2	ELF4
SHC1	MAPK1IP1L	MTMR9	PIWIL4	FBXL12	TEAD3	MT2A	MXD1	RAP2B	C17orf107	DLC1
RNF19B	CORO1C	TNFAIP3	NDEL1	SLC2A3	WDR44	MEF2D	FAM102B	LONRF1	SCG2	SQSTM1
NPPB	CCNK	AGFG1	KCTD5	GRASP	NFIL3	PLK3	GGNBP2	SERPINB8	TMEM233	ARHGDIA
INHBB	RHOB	SDS	TP53BP2	RYBP	LRRC8E	DDX5	FHL3	IL4R	MSN	GNL2
GJA1	MAK	MAP1S	PDLIM1	RBMS1	ZSWIM4	DDX21	IL1R1	JMJD6	KCTD10	SEC24A
DUSP14	CCDC120	SF3A2	C2CD4A	LBR	ZC3H12C	BAG5	ETS1	SDC4	SRF	RAPH1
TNFRSF10B	ARF6	FUT4	MYADM	RHOU	TAC1	EIF2AK3	NFKB2	SOCS3	HBEGF	KBTBD2
BATF3	C9orf150	LCP2	TNFRSF12A	EZR	TFE3	TMEM217	YWHAG	HNRNPA0	NFKBIE	CSF3
CCRN4L	KPNA2	RNF19A	TMEM2	RELB	BYSL	CSRNP1	SOX9	HAS1	TNFRSF1A	GRAMD1A
KRT8	RRAD	DLGAP4	MIDN	ARHGAP17	ERF	FAM53C	DNAJC2	LMNA	PLAUR	SERPINB9
ADAMTS1	GPR3	SPATA2L	PLEKHO2	XIRP1	CSDA	DUSP8	CNKSR3	C6orf145	MEX3C	SGMS2
KLF6	RASSF1	CREB5	ODF3L1	SPHK1	CLDN6	ARL4C	UGCG	ARID5B	CDV3	SPSB1
SELE	MMP19	SERPINE1	C16orf72	DNAJB11	KDM6B	LDLR	CNN2	CX3CL1	RELT	SNAI1
BCL3	MCL1	TSC22D2	ARID5A	MAFF	FSTL3	ZYX	MACC1	MAMLD1	PPRC1	TICAM1
AKAP12	ADAMTS4	ICAM1	GBP1	LRRC59	NAB2	MT1A	THBS1	KLF16	ITPKC	C11orf24
AG2	DNAJB5	CDC42EP1	PTPN1	CHSY1	ETS2	LIF	CEBPB	RARA	ZNF474	MAP3K14
CLCF1	THBD	FOSL1								
Coexpressed genes with Myc in KICH (Stage 1)										
C1orf94	HMGB1P1	C3orf57	EEF1A1	LOC96610	TUBGCP4	FBXO39	CXCL13	BTLA	C14orf4	GUCY1A2
MAL2	NLGN4X	PAX6	RPS12	SOX30	TMPO	TSEN15	ZNF521	CCL13	DRD1	OPTC

BUD13	CXCR5	FAM20A	FPR2	HBEGF	IARS	IL2RA	LOC441208	RPS16	SDS	TRAM1
ZNF660	C1orf92	HTR2A	ARSF	IGFL1	TFF2	BTG2	EHD4	FASLG	HPD	LMO4
MRTO4	PALM2-AKAP2	PLCB1	RAB33A	ST6GALNAC3	PCDHA11	WISP2	ADAMTS2	C8orf80	CTNNAL1	NOS3
PLCE1	VGLL3	NLRP7	INSL4	ADAMTS7	CARD9	DDX18	ECSCR	ELOVL5	FAM83D	KIF18A
LILRB2	RPL41	TTC35	ZNF662	TSPO2	CYP8B1	FAM75C1	AZIN1	C4orf41	CNOT7	COL22A1
FRMD4B	FUT4	HEYL	PLAC8	SLA2	SYCE1L	TP63	CD300E	ANXA2P1	C14orf149	CD86
FKBP14	OAS3	RPS27A	SNTB2	RPRML	COL10A1	RP1	ARHGAP22	CALU	COL15A1	CTSK
FAM124B	FYB	PLCXD3	TBCEL	KHDC1	FMO6P	ATRX	CLEC14A	EMP1	HLA-DRA	ITCH
SP140	TCF12	ZNF542	ST8SIA6	GPR22	PAEP	CCL3	DHX36	HECA	KIAA1467	NFKBIA
PELI2	S1PR4	TNFRSF1B	GPR173	PI16	ATAD5	ATF3	CD28	CDON	CXCL11	DIO2
FGR	FNBP4	GZMK	LY9	NRG3	PCDHB13	SLITRK6	C4orf43	CD48	EIF5A2	MBTPS2
PNMA2	RNF125	SNAPC5	SNORA53	TESK2	USP51	SCARA5	CD3D	CDS2	COL4A1	ID4
MANSC1	MEF2A	RPL22	SLC4A1	OGN	HIF1A	HOXC10	KDR	LRIT3	MYL9	SLAMF7
TMEM100	WBP5	CD68	CLDN11	EHD2	NNMT	RECK	SPTBN5	STOM	VEZT	PF4
ZBED2	RAET1L	C1S	FCRL3	HHIPL1	OLFML3	RPGR	TNFSF8	ARHGAP15	CD14	LOXL2
MED13L	PARP11	SGK223	SHE	SLC22A4	TAGLN	TMEM54	TNFSF13B	TRIM17	ARHGAP9	CCR2
GIMAP4	MFI2	MID2	MYCL1	MYST3	NLRP2	NUP98	ROBO1	SLFN5	UNC45B	CXorf59
C1orf216	CD2	FAM65B	FANCB	LOH3CR2A	SPARC	CDKN2BAS	ZNF560	ACTN4	FOSL2	GDAP1
MMP28	QSER1	S1PR2	SLC27A2	PLA2G5	PLA2G2A	POU2AF1	IL17B	RTL1	ARF4	C2
C5orf20	CTCFL	EGR3	KCNN4	KLHL23	PPP2R2B	VASH1	OR6F1	ARNTL2	CACNA2D4	GBP4
GPR174	TMEM8C	ARHGAP30	ASB2	CD4	CEACAM19	FUT8	ITGA1	LSM 11.00	RASGEF1B	RPL13AP6
SGCE	U2AF2	ZNF267	SYT16	C17orf102	CAV3	GUCA1C	LOC415056	MS4A10	RNF17	TEX11
TRPV3	BACE1	CD79A	CDK2	HRH1	NFIA	PPRC1	SH2D1A	SLAMF6	SLC5A4	SPATA5
STAG1	TMEM106A	TRIM34	TRIP13	VIPR2	CD1A	BHLHA15	GNASAS	PHOX2A	CCRL1	CLEC4A
GNE	HNRNPA1L2	HOXC11	LAMA4	NUBPL	PLEKHG6	SLAMF8	STOX2	COL2A1	GBP7	RORB
AGT	CASD1	COL7A1	KLHDC8A	SMARCA1	PTCHD3	CD226	CEP72	CNN1	MYNN	NMT2
NUDCD1	PKN3	RGPD5	SGK269	ZBP1	ARCN1	DDX60	FMOD	GVIN1	PTP4A1	RCSD1
STEAP2	TMC7	TMSB15B	ADAM12	COL6A2	CWF19L2	HNRNPH2	MEF2C	TMF1	OR52W1	HAS1

CXCL3	ADA	C17orf53	C9orf167	FILIP1L	PCDH19	PCDHGA9	PLEKHO1	QTRTD1	TMEM150C	CCL7
AKAP2	EOMES	FBXL7	GPRASP2	IGJ	MTAP	MYLK	XPC	SLAMF1	BCL11B	C14orf43
CLECL1	LBR	LMOD1	MAFB	PRNP	SCCPDH	KIR3DL2	CYR61	HMOX1	IL10	PRKAR2B
PRKDC	VRK1	PCDHGC4	ABCC13	IKZF3	LOC401093	MN1	NKX3-1	UTRN	CHST13	HK3
MYCT1	SRGN	NPHS1	AMZ1	AMICA1	KDM6A	LAX1	NDEL1	THBS2	SGCG	ANXA2P3
ARF6	ATP10A	ELOVL3	HOXC4	LRRC15	MEX3B	SYNPO2	KIAA1239	GABRB1	NDST3	PITX1
CDK14	IL8	KIAA1147	KIRREL	SGMS1	SUSD5	VCAN	ALOXE3	SEL1L2	ADIG	CHST11
HAPLN3	RPL26	RRM2B	TM2D2	UBE2V2	CD109	COL5A2	DSE	DSTN	HIVEP3	LILRA6
LOC80154	TFPI	C6orf147	IGSF5	GPCPD1	NCAPG2	ZNF259	AKAP9	ATP6V1C1	CAV1	LILRA5
MS4A4A	APCDD1L	ASAP1	C1R	CSF2RB	ENTPD3	H3F3B	LIG1	NDN	NCRNA00052	MMP10
TRH	ARID4A	CCL21	CEP135	FAM198B	GPN3	MLL5	MYCBP	PLEKHF2	POLA1	PAX9
BTNL2	C7	EIF2S1	JAG1	MRC1	PHOSPHO2	SYT12	TCEAL1	EFCAB1	PLA2G1B	BANK1
COL16A1	ILDR1	LRRC55	PTPN22	BRCA2	C1QTNF2	CRYM	CXCR7	FAM129A	FBLN5	TRDMT1
BAI1	COL1A1	DGKG	DUSP4	GIMAP6	HLA-DOA	IL33	LAG3	LMNB1	TRAT1	AIF1
CEACAM21	ENPP2	HVCN1	OR4A47	HTR1D	FADS2	POLE2	PTPN12	PTPRT	SLC14A2	LOC100240726
C2orf48	FERMT3	FOXP3	LOC641298	MAPT	PABPC1	POTEF	PUS7	ADAM28	CCR5	CD209
GNL3L	PDZRN4	SEMA3G	LDHAL6B	LOC100188949	IGSF6	LYPD6	PLCXD2	ABI3BP	ANXA1	LAYN
LILRB5	LY96	NAV1	NFATC2	PVT1	ADH1C	CAMK4	FIGF	IL21R	PARVA	RBM3
TARDBP	CRCT1	XCR1	GABRA5	DMP1	KRT34	CCDC3	CLIC2	VSIG1	SLC26A8	BCAR4
CTSS	FLNA	GPR171	MRGPRF	NID2	NTN4	TLL2	ZNF831	FLJ41941	ALG1L	FAM49B
IRF4	KCTD17	KLRC3	LOC400759	TM4SF1	AZU1	P2RY6	CARD16	FANCM	KIAA1524	MARCKSL1
SOX7	NCRNA00189	NLRP4	ANGPT1	DDX10	SGK1	IGSF1	CCNA1	C20orf166	TNFRSF9	MCAM
MIR17HG	SCARA3	SFRS3	ZFHX4	COL11A1	SYNPO2L	ACVRL1	CCND2	CTSL2	KIAA1429	PLEKHA2
RFC3	RNF150	CPNE4	SLC5A11	KCNA1	NHEDC2	PBRM1	SELP	CPSF6	IGSF9B	ZNF460
CLDN6	AOAH	JAM3	PMEPA1	SHC4	LOC100131551	CELF3	RHAG	CASP5	GPR15	XIRP1
NEDD4	RGL1	MJH26	LOC201651	DUSP5P	ANTXR1	CD27	CLEC10A	DLGAP2	ERG	KLF4
P704P	PTK2	PNOC	KHDC1L	NCRNA00112	OR10H1	ALDH3B2	ATP4B	C9orf125	CMKLR1	COL3A1
CTDSPL2	TERF1	USH1G	SERPINB2	CALD1	CNRIP1	CXCR6	FBLIM1	FES	MIR155HG	NBN

NLRP1	PLEK	PPP1R12A	THSD4	TIGIT	TTC17	CD5	AMOTL2	EFNB2	OAS2	PTRF
C12orf42	ARHGDIG	ABCE1	ELK3	PDGFB	TTYH3	CXorf30	CCIN	BEST1	EIF3E	ITGA11
ITK	LCK	SLC7A14	PTX4	CD1C	MAP3K1	SIGLEC1	CPNE7	ANK1	CDK17	HELB
NCAPD3	ODAM	ASB15	CCR4	PM20D1	CRYAB	GNA14	DCTN6	IL6	LOC644936	MIPOL1
TNFAIP2	MGC29506	ST6GALNAC1	ZCCHC5	CBLB	FAM43A	FAP	SNHG4	TPT1	SSTR3	CREBZF
GNG11	HGF	PCDHGB2	SSTR1	STK17B	UBASH3B	VNN 2.00	DAPP1	FYN	TGM2	TREM1
FLT3	FCRL5	RDH12	SLC22A16	GLIPR1L1	ANKDD1A	C4A	LRRN3	MCTP1	PCDHGA5	PTCHD2
SH2D2A	CCL26	ABL2	C11orf58	LYZ	MFSD2A	PATL1	HSD11B1	MTBP	PLSCR1	GPR12
CALHM2	CYP2S1	DHH	ELTD1	LCP2	LOC389333	LYN	PTPN7	TRIM29	FGD5	PLEKHO2
PLXND1	RDX	SLIT3	UTP23	VGLL1	ANGPTL2	LETM2	NUAK1	RASSF2	SEMA7A	SLC8A1
TNXB	VSIG4	NCAM2	CRTAM	GNL3	KCNA6	PCDHGB7	SIGLEC14	DKK 3.00	PALLD	PTPLAD2
ENTHD1	FRMD6	GRHL2	PLN	SH2B3	TASP1	TGFB2	SULT1E1	CAPN5	MAK16	RLIM
SLC7A7	C20orf103	CAMK1G	EIF3H	KIF5C	NECAP1	KIF20B	LOC388152	METTL7B	NUP62CL	EMR4P
FAM138F	DSCC1	MMRN1	RAET1E	C6orf150	CXorf21	CYBB	ITGA9	KLF10	PIK3AP1	PLSCR4
SLC40A1	MMP1	ACTG1	KIAA1949	RBMXL1	TIE1	GOSR2	RXFP1	PCDHA8	FAM198A	NEBL
RPL30	ST8SIA2	TLR6	GPR1	KDELR3	SOX17	STAB1	CXCL9	CDR2	COL5A1	ICOS
PCDHB4	ACTB	FN1	ITGA5	APLN	ARSB	C3orf64	RPS27	SMOC2	CUX2	CRYBB1
PAWR	PLXNC1	FMN2	S100Z	FGFBP1	ZPBP	KCNT2	NES	NUP107	PARM1	TRIM22
PDLIM4	PRELP	PTHLH	C2orf85	ACTA1	DOCK11	EFCAB4B	FAM26E	FHL2	PTX3	ABCA4
AXL	DDX3X	IRF1	TCF4	CNTN5	FAR2	SLC2A10	OLFML2B	SIGLEC7	ST8SIA1	ABI3
ARL4C	GPR19	MDFI	TNFAIP8L1	BAGE	CD38	GAL	ADORA2B	AKAP12	CXCR3	PXDN
RRN3P1	AFF2	H3F3C	MALL	PCDHB8	TRA2B	OR51B5	AKAP7	APOBEC3G	RRP15	TM4SF18
VNN 1.00	WISP1	SCML4	PCA3	FGFR1OP2	MYH7	PKHD1L1	FEZ1	KCNK6	ATM	HLA-DRB1
RPS6KA5	WNT5A	ADAMTS12	LOC150786	PTPRE	THOC4	TPM1	C14orf126	TSLP	EREG	ART4
CXorf56	GPR133	MRE11A	NT5E	RFTN1	SELE	ALPK2	CD19	CCR7	RCN1	SEC23A
GABRA4	TNFRSF11B	SYT9	WNT7A	CCK	DPYSL3	IL23A	SLC16A14	VCAM1	DSC3	CD200R1
ING3	SLC25A32	KCND3	DERL1	FPR3	MFAP4	CCNYL1	IL2RG	KIAA1324	RHOJ	SERPINB9
ZNF423	AP1S2	B3GNT5	CD163	CD34	IGDCC4	PCDHB7	DMBT1	CD93	SLC4A7	IL11

C13orf36	GCKR	EPB41L2	TMEM64	PCSK5	CHST8	ACTA2	AFF3	TCEAL7	TNC	CMAH
FABP5	MARCO	PCDHB10	PTGER2	C12orf69	IKBIP	MED30	GPR50	PCDHB6	C6orf97	HTR2B
BNIP2	CDH5	TMEM26	KIAA0125	TAS2R50	LOC644538	PTPRH	C2orf89	LGALS2	ZCCHC18	C13orf33
HEG1	PTGER4	FLRT2	FOLR2	RPL7	TRPA1	CD80	LOC654433	ZFAND1	C8orf34	C11orf75
FLNC	SIRPA	LOC100133669	STRA6	CFB	EDN1	LOXL3	RARRES1	TMEM45A	C13orf18	SLC9A9
TRPV2	ARHGAP23	ANKRD55	OLFM1	TGFBI	TIMP3	ARHGAP25	PDCD1LG2	FADS1	CD163L1	CD53
F2R	S1PR1	WEE1	CTGF	PPP1R16B	SH3RF2	CD97	EMILIN2	FLI1	LYPD5	MICAL2
NCRNA00120	PCDHGA12	PCDHGA4	RNF19A	UCN2	C10orf128	FOXL1	MYOCD	POSTN	SAMD3	RHO
ACTN1	GPX8	MGC87042	TMEM71	FAM102B	IFI16	IL1RAP	MX2	CD200	LRRC33	BET3L
GRK5	HLA-DQA1	LRRC8C	PANK2	MRC2	PMP22	ITGA4	PAG1	MPEG1	MSRB3	TUBB
DYNC1I1	MAGEH1	TSPAN2	RMST	GLIPR2	LCP1	PIM2	TFEC	A2M	CLDN1	SULF1
PTPRO	EDAR	TNIP3	NXNL2	OXTR	LRP1	CDH11	CILP2	PROS1	GAP43	PLEKHG2
STAT4	IL4R	NPL	TJP1	IRF8	PHYHIP	ZSCAN23	PCDHB16	LCTL	MGP	PCDHB3
LRRC4C	CASP1	DAB2	PCDHAC2	AEBP1	CFH	DOK2	CENPJ	SAMSN1	FBN1	SLC25A21
KCNG2	TNNT2	C15orf21	HEPH	PRUNE2	GRIA1	APOBEC3C	RUFY4	GBP1	ICAM1	PDE8B
APOBEC3A	TNFSF11	MCART6	SLFN11	GNG2	PRKCB	GLIPR1	CCR1	CNTNAP3	FSTL1	B3GNT7
THBD	LMCD1	VIM	NLRP10	ANKRD1	ME1	PDLIM3	SLFN12L	CD70	RORA	SERPINE2
FOXC2	TMEM150B	SKIL	LOX	PCDHB9	SNAI2	PLTP	SLC43A3	ADAM19	CR1	ZEB2
CDCA7	KLHL4	DLC1	HPS5	MYADM	STEAP1	TULP2	MICALCL	TMEM200A	BTBD19	NR5A2
GPR85	TNFSF18	INHBA	GALNT6	LGI2	XYLT1	THBS1	TFPI2	STX11	APBA1	FERMT1
Coexpressed genes with Myc in KICH (Stage 2)										
UGT2B4	C13orf33	CD28	CTNNAL1	FAM117A	HECW2	SNX16	SNX30	SV2A	TESK2	TRIM59
ACTG1	DDI2	DLGAP2	EFTUD2	FAM198A	IFI16	SMTNL1	TCP11L1	VPS25	OR4F21	C1orf173
CSDC2	GTPBP2	SIRPD	ARHGAP15	C3orf52	HAUS6	LOC728392	PCDHB3	PGM2	PRKD3	SPARC
TTYH2	GPR50	NKD1	STRA6	ADAMTS7	AKAP5	DCP1A	HLA-DMA	MT3	PLXND1	PPM1J
STAT5A	UBASH3A	B4GALT6	D4S234E	LOC100302401	LOC283314	SYTL2	CXCR2P1	CASQ2	FCGR2A	HLA-L
NFX1	NID1	PGS1	VCPIP1	ASB11	APOC2	C1orf113	CD82	HLA-DMB	LILRA2	PEAR1

PTPN22	SNORA67	ARAP3	EIF5A2	PCDH17	RASA2	ROBO1	SSTR2	UBXN2B	THTY6	IZUMO1
FAM194A	CD70	BCL11B	BZW2	C1QB	C3orf63	CANT1	CTSA	DOPEY2	MYO6	SLC6A1
TSC22D2	UBA3	CATSPER1	ADAM10	ATF3	GALNTL6	LPPR4	MEIS3	MYOCD	NPDC1	PSIP1
STEAP3	TFEC	KSR2	SH2D6	C1QTNF1	C3orf17	C7orf68	RAC3	ZNF813	NXF3	ARID1A
ATP6V1C1	CLIC4	EOMES	KIAA1324	MOXD1	MUSTN1	NNMT	SETX	TMEM39A	VSX2	CCL11
LOC100192426	DDN	ARPP19	DCAF13	FBXO5	FMN1	LRCH3	MTMR9	ARL13B	C15orf21	HAVCR2
HHATL	LAIR1	PDGFA	SLC9A9	VMO1	WNT5B	EFHC2	C7orf45	ADAMTS4	MGC87042	NLGN3
PDLIM1	PRPH2	RTN4RL2	ZFATAS	IL8	MSC	MTMR7	UGT2A1	ADM2	ARL 10.00	EIF3H
ERI1	IL17RD	TYMP	IL1A	GUCY2E	LBX2	CES4	FBXO45	ZNF704	FIBCD1	DOK1
KCNT2	MRPL3	RNF32	TINF2	UHRF2	UMODL1	UNC13D	ZNF525	C13orf39	VNN 3.00	PAGE2B
CARS	HCG18	RAD21	SNORA8	TNFRSF1B	TNFRSF8	ZBTB10	ZNF91	CASP14	DAD1L	NCRNA00164
BSPRY	FER1L4	HCK	LACTB2	LTC4S	NAA15	PLXNA2	PRKDC	UBE2J1	ZNF121	FCRL3
ARHGEF3	C5AR1	C8orf58	FAM116A	PALM2	RNF168	ZNF623	CD1C	HES2	ADIPOQ	PSG6
BTK	GYPC	LBXCOR1	NCBP2	SBF2	SLC7A7	TMEM176A	ZBTB26	ACER3	COL5A3	FKBP10
GATA6	MYO1G	NFATC2	XPA	ACO1	C10orf116	FXYD5	HLA-G	LRRC37A	RCSD1	ATP2A3
CMYA5	CTPS	ITGAM	MAK16	MAP3K6	MRPS22	NRG2	OXR1	RABGAP1	CALY	TM6SF2
CPA1	C6orf204	GNL3	KIAA1432	SKIL	SMC2	VAV1	ZC3HAV1L	ZDHHC23	GOLGA6A	DEFA1B
PLK5P	CDSN	EDNRA	HLA-DOB	MAK	MYADM	ZDHHC21	C3orf32	IKBKB	ABCG4	CEBPG
DHH	EVI2A	AVPR2	ENTPD7	GLE1	GTF2E1	IL4R	NUBPL	XKR9	PNOC	ATP13A3
ATP8B1	EIF4G2	FAM102B	HRC	IRAK3	MFSD1	PGRMC2	SET	TERF1	CRTAM	ANPEP
GUCY1A3	VIM	EPHB2	ITIH3	NES	PRPF38A	C8orf39	HPGDS	TSPAN5	C14orf73	TMEM155
WNT7A	CAPZA1	MS4A6A	PLA2G16	SH3PXD2B	SLC37A2	TNFRSF19	OIT3	ART1	FAM55D	ALDH3B1
DCUN1D1	DUSP7	IGF2	SLC22A3	SLC43A3	SP1	TMEM132A	TMF1	XKRX	CTHRC1	FUT4
GRIN1	MARVELD1	PLN	SLC43A1	SVIL	BEST4	C17orf72	FYTTD1	IGDCC3	RGP1	TMCC1
TOPORS	ZDHHC13	CELP	MCOLN2	PPP1R16B	PRKCH	PTGIR	RAPGEF5	RARG	SMC4	SMURF2
AFAP1L2	AKAP1	CHSY1	CORO1C	CRIP1	CRISPLD2	CTR9	PODNL1	RPL22L1	TSKU	CC2D2B
AQP3	CCDC52	EPHX4	GPNMB	LRRCC1	NAGLU	PSMD11	SLC29A1	SLAMF9	GJA8	GNG13
ASGR1	BNIP3L	CATSPERG	GBGT1	IL16	PDLIM7	OPN4	DIO3OS	ANKRD34B	CHST1	HLA-DPB1

OLFML1	TMC8	ZNF883	MYL3	CHAT	DEFB103B	BMX	C14orf139	CDC7	CEACAM21	GHR
LAMC2	LRIG2	MYCT1	OLFML2A	PHLDA2	RYR2	SH3GL2	SIGLEC5	CMTM3	CSF2RB	KIF26A
LOC151534	MCF2L2	RPL23AP53	SLFN11	TUBA1A	B3GNT5	CXCR3	SLA	ANKRD57	DENND4C	DICER1
DTX3L	ENC1	FAR2	LGI4	MFAP2	RASAL2	SIGLEC7	URB2	ZNF281	PRG2	WNT7B
BATF3	C17orf76	DNAJA1	LFNG	SLC5A3	DCST1	FAM122A	LOC81691	NXN	TMEM106A	ELF5
APOC1	BUD13	CD8A	FMO3	HCLS1	RNF19A	SLC27A3	CHAF1B	EXPH5	IL13RA1	IPO7
MMP11	RHOJ	SYT11	UPF0639	UCN2	FAIM	FPR1	RAB7A	IL1RN	MEF2D	PRPF38B
RRN3P2	TNFAIP8L1	ZNF292	PGPEP1L	PFKFB1	CCL26	CARTPT	BAI1	GP1BA	RAB6B	TLR4
TRMT12	ZNF676	ADA	C14orf118	PRDM1	RPS13	SLC5A4	BTN2A2	KIAA1107	MAT2A	MDK
PARP9	PDE4B	RPGR	C11orf88	CD68	HLA-DRB6	NDRG4	RELT	KUYN	SLC2A14	CD1A
FBXO32	KCNJ5	LAMP3	PIK3R5	PPAPDC2	ZFAT	SCN10A	DAAM2	NUDT16	P2RY6	TREM1
PARVA	ZCCHC6	ZNF256	INHBA	KIAA1529	LOC200030	LRRC37A2	NOL8	SOX5	C19orf38	CCNB3
CXCL2	ITIH4	LRRC42	SPOCD1	ZNF572	CPA3	DHX36	UBR5	TREML2	OR2AE1	CDH11
PCNP	PIK3C2A	TINAGL1	TNXB	CRIP3	PADI1	ARNTL2	CRYBB1	GLIPR1	ITPRIP	MRE11A
NSUN3	HSD17B6	ADRA1A	F2	ADCY4	CHST11	EIF2C2	FRMD4A	QTRTD1	SLC2A3	C10orf55
CCKBR	PCM1	RAB33A	RALGDS	SLAMF8	SLIT3	ZMYND15	PSG2	GDF7	C16orf75	CYFIP1
GREB1	MTSS1	ARHGAP17	CKS2	DLX1	GDPD5	KIF9	SLC22A18	LPHN3	LPL	TMEM167B
ZNF469	SAA4	MYNN	RAB2A	RAB32	SLC39A11	STAB1	TTLL7	AVPR1B	ACER2	FCGR1C
PHC3	SYNCRIP	TMC6	TMEM176B	VPRBP	VIT	ASTE1	FIG4	MEX3B	NFASC	RSPH9
TMEM26	YIPF1	PNMA5	KIAA1429	PPFIBP1	WWTR1	ZNF643	ZBED2	CLDN7	FAM49B	SEC14L1
SLC15A2	SNORA9	ZNF484	CNGB1	BCOR	IKZF3	KDM6B	MYO1C	ADAM15	ADAMTS15	C1QA
CRK	FHL2	PPP2R2A	ARSI	AOC3	COL13A1	NEDD4	C4orf51	FLJ41941	ADAP2	COMMD2
GON4L	LY96	TOMM70A	CCNL1	KIAA1462	C21orf125	FAM7A2	CD33	HMHA1	IL34	INVS
PPIL1	C1S	DCLRE1B	KIAA1949	LOH3CR2A	NLRP1	PILRA	DQX1	SCPEP1	SORD	KLHL33
CP	AHDC1	LAYN	RNF165	FUT7	HAMP	MAGEL2	CCRL2	CDC42EP3	CXCL16	LOC651250
LOXL3	SIK1	SOCS3	TMEM54	ZNF436	CCL16	IL5RA	C9orf44	ACSL6	KIAA1161	SNX4
XYLT1	CSPG4	FKTN	GRN	KIAA0368	KNDC1	LOC145820	NCF4	SLC39A14	HTR1B	MYOZ3
DOC2B	HRH1	IQCB1	PABPC3	ZW10	CTSG	AFAP1	IL7R	KLC3	TNFSF8	CHST6

IL17B	CXorf49B	ACAD9	PALLD	SLC1A7	C1R	DDR2	SLC11A1	ZNF385D	DPPA4	C6orf227
TFF2	C6orf138	EHD4	STK3	TET3	IL10RA	PRND	PTPN2	SLC45A3	MFAP5	IL17F
ZNF645	BAG4	FPR3	MTCP1	PAK1IP1	PTGFRN	SFRP1	SIGLEC1	TMPRSS4	DCAF10	FBN2
SLAMF1	DEFA4	ELOVL4	FCGR1B	SLC2A5	SLC9A1	HTRA3	RYBP	SLC31A1	FHL5	H1F0
LUM	PATL1	SLC12A8	TMEM233	ARHGAP30	CLDN11	WNK2	BHLHA15	NTRK2	TBXA2R	HGC6.3
RPL13AP17	CCDC48	FBLIM1	KCNK6	TNKS1BP1	CLDN6	POU6F2	PABPC1	KRT1	CALHM2	STRBP
CACNA1F	BMP2K	CYP1B1	EMILIN1	MOCS1	MYO9B	PLEKHO2	TNFRSF11A	C10orf131	C1RL	DAPP1
FAM78A	RBMS3	SIGLEC14	AZIN1	RNF138P1	SERPIND1	SYTL1	NR5A1	APOLD1	IARS	MYO5C
TJP2	C15orf39	GOLGB1	ZNF347	CXCL13	GAPVD1	VCAM1	IBSP	CCDC155	ANGPTL2	ARID1B
CXCR4	H3F3C	SOBP	ZBTB11	AMZ1	CDC26	CFH	KIF7	TUBB2B	MICALCL	ARHGAP23
NT5C1B	PSG5	RPS10P7	SPTBN1	TMEM130	DKFZp686O24166	HOOK1	MRC2	PIGA	RBM12	SAMSN1
TMEM44	PSG3	CYP4B1	ENTPD2	GNB4	LHFP	MXRA8	SDS	KIF3C	LOC729467	CBX2
CNTD2	GBP1	IGFALS	KIAA0196	ART4	BFSP2	TLX2	ITGA7	FRMD4B	PLAC4	RAB27A
TMOD2	TRPV2	ZNF470	RGS7BP	CHSY3	IMPG1	KIAA1755	SERPINH1	NID2	PLXDC1	LINGO3
FAM49A	KIAA1407	PVT1	CERCAM	SAA2	CD200R1	CSRP1	ECM1	HDGFRP3	SFRS2	SYT12
TPSAB1	ELF4	LHX6	IFNK	EGFL6	C9orf129	CD79B	CHRD	CTTNBP2NL	GGT5	CAPN14
ASAP1	HEG1	HTRA4	LTBP1	SRL	ALPK2	OR13J1	POU2AF1	VLDLR	MYEOV	CHMP4C
NAA50	UGCG	VASH1	WNT9A	EMILIN2	FABP5	ATP10A	C6orf26	CHST15	FOXN2	LOC284837
LXN	TBC1D23	TMEM150B	PKD2L1	CEL	MTMR4	FMN2	RABL3	CD19	CDV3	EPB41
GNAI2	RICH2	SIGLEC6	NFATC1	PIK3AP1	SERPINE1	OR11H4	CCBL2	CNOT7	FERMT3	MAP7D1
MYH3	P2RX1	FCHSD2	GSN	NCRNA00095	POSTN	PPP1R14A	RASSF3	TMEM119	VSIG4	CORO2A
LOC338651	MN1	PSIMCT-1	SLC1A3	ATPBD4	LYZ	PSG4	SPSB1	IL22RA1	AURKAPS1	GFI1
KLHL6	LOC100126784	LOC284100	ARMC8	CCDC80	DGKA	MTF2	ATG3	PTPLB	TEX10	ARMC1
ASPN	CD163	MMP7	PDCD1LG2	ZNF471	MYBPC2	OBP2B	ADAMTS6	FAM131B	FOLR2	ZNF781
AKAP12	ANKRD26	SNAI1	TMEM65	F2R	PLA2G2D	AGAP11	TYW1B	CNTNAP1	IL21R	MYH9
NCF1C	CD300C	HEPH	TADA2A	EMP2	GOLGA7	GRK5	RNF20	SEMA7A	POM121L9P	ANO1
DNAH1	FILIP1L	TAF2	TOPBP1	C4A	GLI3	MLF 1.00	IL2RA	ZCCHC5	ACRBP	ALDH1B1
CDH6	HTR2B	MGAT3	PLAU	TMEM132E	TNFAIP2	RGS13	AATK	APPL1	IGFBP2	C10orf119

MTUS1	PHF20L1	PTGIS	CAPN8	CCL8	CCR7	ITIH1	MCART6	PALMD	STAG3	C9orf64
GPRC5A	LAMA4	PRICKLE2	KRT5	NEK4	TPM4	C6orf103	GPR160	ZNF229	COX6B2	DEFB1
GSR	DBX1	KDM4C	SLC16A6	SULT1B1	CIITA	SH3RF3	SYNJ2	FN1	KCNMB1	FNDC1
MALL	MS4A3	BTLA	ZNF782	C20orf112	KPNA4	RHBDL2	RIN1	FCAR	SERPINA9	DAGLA
STAG1	ANTXR1	GPR183	THBS1	ANKDD1A	FAM76B	PGAP2	SMARCA1	COL10A1	CCL18	CHMP5
ZNF880	TRPV3	AQP9	MFI2	SLC25A32	WHSC1L1	ZDHHC2	FCRL2	C2	COL12A1	COL6A2
FBLN5	TPSB2	DERL1	DLEC1	TGFB1I1	TTF1	CCL13	FXYD1	AKAP2	CD27	MTAP
PTP4A3	FOXF2	CSTA	IGJ	LRRK1	SRPX2	LTBP4	RPGRIP1	GNG10	POU2F2	TIMP1
B4GALT4	EDIL3	SPTBN5	ZNF660	NOTCH3	TCF7	VGLL3	BST1	C6orf27	LOC440461	MYOM1
FAM180A	CLEC5A	FMOD	JAG2	TREML4	CD44	OSBPL11	ZNF382	KCNQ4	PCSK5	RPLP0P2
FCRL4	C1orf183	SLC2A10	CNN3	LSAMP	MCAM	ZNF681	PTCRA	C15orf48	PDLIM4	SPAG1
GPR176	DCAF12	LMCD1	TSR1	TNFRSF17	C10orf28	FEZ1	PADI2	SNAI2	S100B	ADAMTS10
LILRB5	TIPARP	ADAM19	COL6A3	ECM2	FOXO3	SH3PXD2A	HSPG2	HTR1F	LILRA6	PLAA
TATDN1	CACNA1I	IGSF10	GATA1	DLX2	ACTA2	GMEB1	CYB5R2	IL24	NCF1	GPR124
OLFML2B	C1orf186	NLRP12	OXTR	NLRP8	KRTAP1-5	CSF1	LOC729799	PTRF	FAM120A	SEC22A
CLEC10A	PXDN	AKNA	COL14A1	MYO7B	PRSS35	ECEL1	OSR2	SYT13	CNN1	LOC641298
UBAP2	NAA35	DLX6AS	APOBEC3C	C6orf150	HAPLN3	KLHL4	LOC401093	NBPF3	SLC24A3	KIRREL
MGC29506	PRRT4	TNC	C9orf93	GALNT6	H3F3B	IL10RB	COL15A1	MICAL2	TNNT3	KRT17
LOC221442	C14orf49	MMP28	RASSF2	ACTG2	ELN	PDCD10	RAB42	DFNA5	HIP1	COL4A1
GJC1	PHLDB1	TNFAIP6	NAV2	SARM1	ADAM6	MMP14	COL4A2	PRRX1	KRTAP5-4	CATSPER2P1
ISLR	PHYHIP	NCRNA00120	VCAN	CNTN2	GMPS	LMOD1	MYH11	MESDC1	NFYA	NRXN2
PDE3A	GALNT5	LOXL2	TPM2	GPR161	SNRK	MS4A2	PODN	ART5	SYNPO2	CCIN
TAGLN	CALB2	RHPN2	HDC	ACP5	CPXM1	ABTB1	DKK 3.00	GLIPR2	SGK196	FOXO3B
MRGPRF	TRAM2	COL5A1	LEP	LDB3	ACTN1	PMP22	SERPINF1	AGPAT4	TMEM200B	WFDC1
B4GALT1	PRELP	PLTP	PITPNC1	TGFBI	FAP	TSPAN2	LOC100130581	APOL4	FNDC3B	MYL9
SMC5	CILP2	HSPB6	RB1CC1	SULF1	PKP1	CALD1	ROBO2	SMOC2	TM6SF1	MYST3
PIK3CD	GTF3C4	COL5A2	LOC644538	ZNF135	THBS2	PVRL4	MAP3K12	ADAMTS12	COL1A1	LOC284749
PRTFDC1	FCRL5	FAM26E	PLEKHG2	RAD23B	CCDC109B	DCLK3	MFSD11	COL3A1	CBWD6	NOM1

CD109	PDLIM3	PAPPA	COL1A2	ADAM12	STX11	MST1R	AEBP1	ADAMTS2	ERICH1	GPR143
Coexpressed genes with Myc in KICH (Stage 3)										
NPFFR2	AOX1	COL5A2	ESR2	GNB1	JMJD7-PLA2G4B	LOC613037	PARP6	POU2F3	PRDM10	PSD3
RBM18	SSRP1	TIGIT	TKT	TMEM44	UPF3B	USP1	WDR5B	ZNF567	ZNF785	ZCCHC5
MYF6	C12orf63	LGALS7B	PKP1	KRT79	ZNF80	DLK2	MCHR1	ITLN1	AQP1	ATP2A1
BMP2	C11orf58	CBLB	CCDC123	CTNND1	DARC	ELF2	FAM102B	GIPC2	IGFBP2	LOC400657
MC1R	MPP6	NAA35	NCRNA00176	PGBD4	PHF8	PRDM8	RCE1	SGPL1	SIAH1	SLC28A1
TEAD2	TRPM6	USP48	WBSCR27	GPR141	ECEL1	C1QL4	C4orf47	TSPAN19	AGER	APOL6
C6	CDCA4	CELF1	EEF1A2	FAM129B	GDPD3	HAUS2	HNRNPC	HNRNPR	IER5	IGSF1
ISY1	ITPRIP	LIF	LOC221442	MFAP2	NXF1	PCDHGA3	PKIA	PLK3	SIK2	SNRNP40
TLE1	TMEM39A	TRAF2	UBA6	ZNF586	MAB21L2	ZAR 1.00	GLOD5	C19orf55	CLCN6	CLDN14
COL5A1	GGH	GPR173	IRAK2	LOC100125556	LOC100271836	LOC283663	LOC440354	MDK	MGST1	NCRNA00085
PDGFRL	POLE3	RAB41	RASSF2	SEC11C	SNORA8	STAG3L3	VWA5B2	ZNF691	ADAM7	CYP4A22
SLURP1	SSX8	ZNF705A	RNF183	SLCO5A1	SPATC1	ISLR2	DNAH3	ACAT2	ARF4	AUH
C21orf91	C6orf114	CST7	FAM82A2	FKBP10	GPR109B	HN1L	IKBKB	LRP5	LRRC15	MAK
MCART2	NFKB1	PTPDC1	RDH5	RIMS3	RRAD	SLAMF7	SLC7A5P2	ZNF658	CPN1	ADAM2
ZSCAN10	C1orf104	CELF5	CH25H	DAPP1	DDB2	DSC2	DTX4	LOC100131434	MARCKSL1	MORC2
NNAT	PDXDC1	PPP1R1A	QSOX2	SPPL2A	TESK2	ZNF8	NPPA	SAA4	WNT7B	TREH
ACSL5	ADA	ARHGAP31	ATM	BIRC3	C4orf46	CCDC52	CWF19L2	DLL4	DPYSL3	FAM18A
FCGR2C	KLRK1	LOC440944	PATL1	PRRT2	WHSC1	SLC7A3	PENK	CCT6P1	CDHR1	FASLG
HHIPL1	HLA-DQB1	HPSE	KIAA0020	LOC441454	MORF4L1	RBMXL1	RINL	TCEA1	TRIM59	TTC21A
ZWILCH	SLC17A3	C11orf88	C12orf42	ADCY7	BASP1	CASP3	CLEC18B	FAM192A	FAM22F	FAM45B
FBXO25	FLVCR1	LILRA4	LOC100272228	HGFR1	POLR3D	PPP2R2A	PTP4A3	PVRIG	RGS1	RUNX2
SERINC2	SFRS7	SMC5	TACC1	TARDBP	TF	TIA1	LOC729609	C2orf73	AKAP12	AOC2
CELF6	EIF2C3	GLIPR1	KLKP1	LOC144571	LOC284837	MFHAS1	MIER1	MMP2	MS4A14	NKX3-1
NPR 2.00	PPP1R15B	SEC24D	SERINC4	SERPINA3	SPSB1	STC1	TRA2B	UBAP1	VBP1	YARS
ZNF789	NEU4	ACSL1	C16orf74	C4A	CASC5	CD244	CD6	DNAJC5B	FAM115C	INO80

ISYNA1	KCNJ13	KIAA1432	MEX3B	PLXDC1	REEP3	RTN3	SLC4A7	SOX7	TRANK1	UBE2R2
ZNF805	FAM123A	IRGM	TAS2R4	FLT3	ADAMTS7	ALPK3	CD180	CD247	CLLU1	EEF1DP3
FAM118A	GBP5	KIAA1409	NLGN3	SFRS11	SGIP1	STIP1	ZNF137	ZNF557	ZNF706	OR1J1
CNTN6	OR1J2	ANKRD20A3	BNIP3L	C1orf77	COL15A1	FAM22G	FBN2	FNBP4	KAL1	KDELR3
KIAA1045	LOC653113	MFSD10	MRRF	MURC	NFKBIZ	NPTX2	PABPC1	PCDHGA12	PYHIN1	SH2D1A
SNHG3	NANOG	MYBPC2	FFAR3	C2orf84	CDC26	CDK20	CEACAM19	CLEC2D	CYR61	GNL3
GPR183	HINFP	HSBP1	LOC100128842	MMP7	NRG1	PDLIM4	RBM20	SAA2	TNFRSF10B	TRMT12
WDR52	XBP1	C3orf65	ACO1	C1orf213	CCDC117	CDK17	FAM120AOS	GNB4	GOLGA6L5	KDELR2
MASP2	NCRNA00167	SFRS13A	SGCB	TSTD2	UBASH3B	UBD	VNN 1.00	TMEFF2	TDRD1	LPAR4
LY6H	ATCAY	AKAP13	ANKRD27	CD22	CG030	CPXM1	ETV6	FAM114A2	FAM13C	HAUS3
HIVEP1	LSM14A	NCRNA00095	NOP14	RIPK2	SLC8A3	SNORD1C	TM4SF20	APOC1P1	C10orf91	CGB8
HMX3	OR51A7	KCNK9	C10orf81	CCL20	BREA2	C5orf58	GDF3	UGT3A2	CD80	CORO1C
DNM1	HLA-DQA1	HSPA6	KIR2DL1	KLRB1	NAV3	NRBP2	PRPF39	PTDSS1	SCD	SIGLEC8
STC2	THAP9	TNFAIP6	PCDHGC4	ARL 14.00	BEAN	C8ORFK29	CHD7	DCAF12	EP400NL	FANCC
FCRL6	FZD10	GMEB1	HEY1	LSM 6.00	PPM1N	RBM5	RFTN2	RPL32P3	ADT42248	TMEM151B
UNC5A	ZNF732	OR51S1	TTPA	GCKR	PITX3	UCA1	ADAMTS2	AURKAPS1	C9orf130	CLDN9
COL23A1	GCFC1	GPR85	IGSF9	KRT80	LOXL2	PCDHGA6	PHYHIPL	PRF1	SP140	SPRY1
STK3	TAS2R14	GLP2R	WDR65	PAEP	ACBD3	C11orf61	C2CD4A	ECE1	GFI1	GLE1
ITGB6	KIF21B	LOC100130557	LOC154761	LOC401588	LOC728024	PISD	RPL23AP53	SLC38A5	YPEL4	ZNF326
GOLGA6B	AIMP1	FLJ10213	INTS4	JUB	MSI1	PCDHGB1	RANBP1	SAMD3	SKIL	UBR5
ZNF526	ZNF841	ZSCAN22	ARL 9.00	HOXD13	PCDHGB8P	SLC9A10	C12orf69	C15orf51	C4orf33	CCRL2
CHST1	P2RY10	PHACTR4	POLD3	RNF19A	ZNF7	NCR1	LRRC4C	PFKFB1	ADAMTS14	CARS
CCDC39	CLEC18A	DCLK3	ENTPD7	IL13RA1	MEI1	NAALADL2	PDZRN3	SETX	SLC17A9	TRIM66
GPA33	C11orf21	CAMSAP1	CEBPG	CHORDC1	CITED4	KLHL6	PPAPDC1B	RAB33B	RND3	S100PBP
TLE4	TMTC2	WNT1	OR1Q1	BCMO1	C9orf40	KLHL29	LRP2BP	MMP11	NKG7	PCDHGB3
PTPN3	RNF182	TJP1	UBE2Q2P1	UGT2A1	UHRF2	DNAH6	OR10AD1	GRM4	ADAM19	BUD13
C16orf87	CDH2	CTSW	FGF14	GZMK	HOXB2	KCND1	NOLC1	PPP1R16B	TGM2	UBA3
ASFMR1	DPF1	IFNA2	ADAMTS9	CHMP5	GRAPL	GTF2E2	NIPAL4	SLC1A5	TEAD4	TSC1

ZNF200	SUMO1P1	ARL6IP6	C19orf23	C2CD4B	CARD14	CXCL9	GOLGA2	HAPLN3	LDHAL6A	LOC100190986
NIP7	RUNDC2C	DSC1	TRPA1	DNAH2	ZCCHC12	FCAR	SLC4A10	AKAP5	DYNC1LI2	DYRK2
EIF4EBP1	FPR2	GPM6A	GPRC5A	KIAA1797	PCDH18	PFKFB3	TMEM45A	TRA2A	UBAP2	CCIN
HIST1H1D	PAK3	CDKN2B	ISCA1P1	ITGB3BP	PAFAH1B2	ROBO3	PCDHGC5	IMPG1	LDLR	NFAT5
RCN1	RHOJ	STT3A	ZBTB5	ZNF782	FAM166A	WT1	C21orf122	DNAH1	EBF2	ISCA1
LAMC2	LENG8	LOC390595	SLC7A5	TNK2	UGCG	C10orf25	CD72	DIAPH2	FRG1	IL2RB
LNP1	LOC653566	MTAP	PABPC3	SF1	MYCNOS	OR6W1P	GPR174	RNASE10	AGAP5	CPT1C
DERL3	KIAA0467	KRT17	MEGF11	PCDHGA11	RGS3	TTC9C	UNC5CL	FCRL3	ABCC2	FDFT1
LBH	MSL3	NONO	PRR16	ZBTB49	GSG1L	CD70	CDC7	CTHRC1	CYTL1	FZD6
KLRA1	RBPMS	SET	ST8SIA1	CLDN6	RHOXF1	ARL4C	C13orf33	CXorf42	DUSP4	ETV1
LOC729799	TSNAX-DISC1	C12orf56	GJB7	TRPM8	CCDC111	KIAA1324	C11orf94	GPR61	ADAM23	ANKRD49
EOMES	GARNL3	LOC100272146	RBM7	TEX10	CHRNA3	BCL11B	DNAJC12	MIAT	SC4MOL	TPBG
RPE65	TNFRSF9	MTF2	SAE1	SLC2A14	YWHAZ	CDK2	DRAM1	FSTL1	NKTR	SAT1
ZNF321	CBR4	CD3EAP	CNGB1	SLC25A37	TSPYL6	CAV1	MED17	TJP2	HIST1H3F	PRINS
BIRC2	CEACAM22P	RUNX1	SPARCL1	ZDHHC19	LTA	DNAJC25	ZNF202	ZNF22	ZNF250	IL18
NIPSNAP3B	TLL2	CST1	CASP7	MFI2	IL17REL	GNG10	LAX1	PRPF38A	ARRDC3	C15orf28
CXCR4	SCLT1	ERI1	P2RX5	ANKK1	PLEKHF2	PTGDR	ZNF235	CYP2B7P1	NDST3	C8orf4
NEK10	NRN1	IL1F7	EIF2C2	SURF4	ATP8B1	CDKN2AIP	SCN11A	ATP6V1G1	C9orf131	ZNF436
KLF10	LGSN	FAM76B	SLC2A3	TAS2R5	AHNAK2	PIK3R6	PIWIL4	FAM49B	IL23R	ADAMTS4
JPH3	CHST6	VAMP1	ICOS	ZNF169	GOLGA1	PDE7A	C8orf42			
Coexpressed genes with Myc in KICH (Stage 4)										
BEND4	DEFA1B	LAX1	MYH16	TDGF3	VIPR2	GTSF1L	KCNB2	NKX2-5	NPY5R	ABCC2
ANKRD2	ARHGAP29	AS3MT	ATP5G2	AVPR2	BACH1	BAI1	BZW2	C19orf2	C19orf55	C21orf7
C6orf108	CAV2	CCDC7	CD300E	CD96	CSDA	CSRP1	CUTA	CXCL13	DDX42	DDX52
DHX33	DIAPH3	DIP2A	DNAH14	DSCAM	DUSP1	ECHDC1	EEF1G	EPB41L3	ERF	ESYT2
FDXR	FGFR4	FMO3	GCK	GPATCH8	HLA-DRB5	HNF4G	IFIT5	IKZF5	IL7R	IQCB1
IRS1	KAL1	KCNH4	LEPR	LIMS1	LIMS2	LPIN3	LRRC34	LTB4R2	LTBP3	MCPH1

MFI2	MINK1	MLL4	MOXD1	MRPL2	MRPL37	MRPS5	MYLK	MYO9A	NACAP1	NACA
NBPF16	NLRC3	NME2	NPM1	NR2C1	NUDT16P1	PCDHGC5	PHB2	PIAS4	PLEKHA6	PLSCR1
PPHLN1	PPP1R14A	PTP4A1	PTTG1IP	PUF60	RABGGTB	RIN2	RIOK1	RIPK2	ROPN1L	RPL13AP6
RPL13A	RPL22	RPL3	RPL4	RPS18	RPS3	RPS9	SERPINB6	SERPIND1	SNCG	SPDYE3
SPN	SSRP1	STARD3NL	STAT5A	SYT2	TLL2	TLR10	TNFRSF10B	TP53	TPT1	TSPAN2
UBE2CBP	USP54	USPL1	ZNF37B	ZNF584	ZSCAN2	C20orf200	LOC90586	ADH1A	APOC3	ASMT
C2orf61	CACNG6	CGB	CPA1	CTSG	GYPA	KCNQ2	KCTD4	LY6G6F	MBL2	MIXL1
NCRNA00230B	UGT2B10	GPR26	C10orf99	AAMP	ABCB1	ACER2	ACSL5	ACTN2	AMBP	BCAR3
BEST3	BIRC7	C6orf154	C8orf42	C9orf68	CDC42EP4	CDKN2A	CNN1	DCTN2	DNAH5	EEF1A1P9
EEF1A1	ETHE1	FBXO22	FCHO2	FHOD3	GALNT9	GLTSCR2	HSPD1	IFIT2	IGF2R	ITGB3
ITPR3	KCNIP3	KDELR1	MCAM	MLLT10	MORF4	NCR1	NCRNA00188	NKRF	NLN	NPY1R
NSMCE2	PAPD4	PAX8	PCBD1	PLDN	PODXL2	PPP2CB	RARRES1	RASSF3	RPL6	RPS20
RPS26	RPS29	RPS2	RPS4X	RPS5	S100A1	SLC16A6	SLC44A4	STEAP2	TACC1	TAGLN3
TANK	TARS2	TATDN1	TBX18	TGFBR2	TKT	TMEM14C	TMEM67	TRMT12	TRNP1	TRPC4
UACA	VSTM1	ZNF259	ZSCAN12	CBWD5	LOC100127888	LOC723809	SIGLEC6	ACP1	ACTA2	AGBL5
ANKRD27	ARSG	C10orf84	C13orf1	C19orf33	C1orf107	CDC42BPA	CRIM1	CWC25	CYSLTR2	DCTD
DIAPH2	EEF1B2	EIF3H	FAM49B	FCRL3	GNPAT	HIF3A	HIPK4	HSPB2	IL1RL2	IPO4
LOC100190938	LOC728190	LRCH1	NEDD1	NEK3	NSMCE4A	PCBP2	PCID2	PDZK1	POLRMT	PPWD1
PSMB5	RNF41	RPL10A	RPL13P5	RPL18A	RPL37A	RPL41	RPL7	RPLP0	RPS12	RPS16
RPS25	RPS3A	SEC14L1	SIK1	SLA2	SLC35D2	SMAGP	SNAPC1	SNHG6	SNORD1C	SUPT5H
TAGLN	TBCA	TGDS	TGOLN2	UTP23	VNN 2.00	VPS54	YOD1	ZNF526		

Table S7: List of coexpressed genes with Myc in KIRP, [KIRP=Kidney renal papillary cell carcinoma]

Coexpressed genes with Myc in KIRP (Normal)										
DCLK1	CCL2	IL1R1	SOCS3	THBS1	PLEKHO2	PLAUR	GBP1	IL24	GJA1	SLC2A3
SLC2A14	ICAM1	DCUN1D3	SELE	AKAP12	RHOU	THBD	PPP1R15A	INHBA	SNAI1	STX11
MYADM	CHIC2	ADAMTS1	TDG	SERPINB1	GEM	C6orf150	RAP2B	MMP19	DTX3L	ADAMTS4
IFI16	CYR61	CEBPB	SPRY1	MAP7D3	TNFRSF1A	CSF1	DSE	SEC24D	INHBB	KCTD10
KBTBD2	ETS1	PANX1	OBFC2A	PGM2	SERPINH1	IL6	IRF8	ISG20	AHR	UGCG
TNFAIP3	HELB	BATF3	PLK3	ETS2	SERPINB8	CD300E	THBS2	C19orf59	SERPINB9	37681
MCL1	AGFG1	RELT	GPR183	PIM1	KLF4	RND3	FAM102B	SPHK1	ZFP36	KIAA1199
ALDH1L2	LIF	KIAA1949	FGF7	NLRP12	FOSB	PTPN1	MMP25	LMNB1	OLFML2B	S100A3
CLCF1	RARA	PRDM1	DDX21	FFAR2	IRF1	TP53BP2	BACH1	SECTM1	GZMB	CSF3
ADAMTS2	NHEDC2	SLC2A10	BCL2A1	C1S	SHC1	AG2	RASSF2	TFE3	BCL3	SLC26A2
SERPINE1	BAZ1A	CCL7	REG3G	STEAP4	LILRA5	CD69	ELF1	CLEC12A	DPPA4	MBNL1
MAMLD1	SBNO2	C1R	FSTL3	OPN1MW	CD93	HAS2	HP	CCL8	CNN2	TRIB1
NAV3	GLIPR1	GDF5	CD300LB	HIVEP1	TXLNB	HRH1	AQP9	AFF3	EIF4A1	NAA15
ADAM12	LBR	SIGLEC5	COL1A2	PALLD	FAM159A	TSHZ2	ARID5B	GRAMD1A	SLFN11	FSTL1
LILRA4	APBA2	LHFPL2	CDV3	STAT3	TSHZ3	RBMS1	LCP2	PYHIN1	LILRA3	TCF4
LPAR1	CCDC80	CLDN6	KLF6	ZCCHC12	NFATC2	EMR3	C1orf38	COL16A1	ZYX	MMP2
EMR2	ARF6	SVEP1	CDC42EP1	PLEKHG1	RASSF5	FAM49A	FBN1	SULT1C4	CLEC5A	CSDAP1
DLC1	IL13RA1	CSF2RB	C5AR1	IL8	FFAR3	EGR3	PELO	MEX3C	WDR63	PARP9
STK17B	ADAMTS12	APOBEC3A	SMAP2	C9orf21	CP	MXD1	RNASE2	ITK	ZNF474	PODNL1
OSMR	FHL2	SAMHD1	WNT2B	CTTNBP2NL	SOX7	RGS1	ACBD3	CEP170	CNR1	RND1
TMEM2	COL4A1	CDC42SE1	PODN	LUM	MAK	COL10A1	GPR171	KRT1	VNN 3.00	TRAT1
GZMH	LILRB2	DUSP10	ARL4C	SLAMF1	HIVEP3	P2RY10	GNL3L	TICAM1	CHST11	NAMPT
TDO2	IL7R	CCDC141	SERPINB2	CD86	NCOA7	CD96	MEFV	TNFRSF9	PIM2	CD28
HAPLN3	CSGALNACT2	AFAP1	SAMD4A	EFCAB4B	LYZ	SELL	NINJ2	KIAA1045	LAMA4	ULBP2
OASL	COL3A1	P704P	TMEM71	ACTR3	DACT1	NLRP3	AXL	SLIT3	IFFO2	PTPRE
HAVCR1	VNN 2.00	NUMBL	TMEM217	COL6A3	ADD2	SPSB1	CCL18	LZTS1	CDKN3	ATP8B2
RHOH	TNFSF8	S100A9	CLEC4C	HECA	PDE4B	CXCR4	TUBA1A	GATA6	MS4A7	ANKRD1

CFH	NCF2	FCGR1B	PDLIM1	FCGR1C	TIMP2	CSF3R	MMP14	CRISPLD2	NFE2	KLRD1
DNAJB5	C9orf150	ARL5B	CSRNP1	GUCY1A2	RUNX2	RUNX3	GPR65	CD300LF	ABL2	BEST1
OSM	GNA13	RFTN1	SOX4	SLAMF6	PTPRC	DCN	AEBP1	PRRX1	PRIC285	C1orf198
RBM7	TNMD	BHLHE40	TNFSF9	RASGEF1B	EPB41L2	HSPB8	LDLRAD3	CFHR1	IL2RA	FRMD6
MUSK	PLEK	CD209	MFSD2B	ELF3	CXCR6	CD300C	MAP1B	ENAH	CD97	LOC150786
MCART6	SH3PXD2B	PPRC1	ANTXR1	ZSWIM4	ART4	SRF	HAS1	PPP1R15B	FPR1	IKZF3
LRG1	TAC1	PTPN22	UBE2J1	TAGAP	ANXA1	LMNA	FAIM3	CRTAM	EMILIN2	MICAL2
ELK3	ARFGAP3	FAM65B	GPR97	LRRC59	SIGLEC6	FGR	RET	BTK	LOC153910	DYRK3
COL15A1	TCF7	SPRY2	CDK2	SOX9	NAALAD2	CD44	COL5A1	ALAS1	RNF122	LOC541471
GPA33	TNFSF13B	TLR8	ADAMTS3	CD247	TNFRSF1B	TRPC4	TEC	OMD	COL8A1	FKBP10
CBFB	GALNT5	AFF2	NALCN	CFP	PLTP	CD163	FCN1	LCP1	APOBEC3C	GPR112
NOLC1	RAET1L	CDH11	PTENP1	GZMK	CHRDL2	COL1A1	CREB5	AOAH	NKAIN1	RGS4
MPEG1	OGFRL1	PTGIR	RHPN2	ADA	CST7	PADI1	TREML2	C13orf31	THEMIS	GRIN2A
LILRB4	GZMA	IL4R	XCR1	PGM2L1	PPYR1	C7orf58	NPFFR2	CD3G	PLAC8	DOCK2
GBP2	TMEM119	ZFP36L2	PRF1	FUT4	TBX21	ZBTB8A	CHSY1	IL13	RUNX1	SLC32A1
CDKN1A	IL7	SELPLG	VIM	PEA15	ANUBL1	SEMA3A	KIF18A	KLHL6	FCGR3A	HSF5
LAPTM5	CELF2	APOL6	ACTG1	EVI2B	ADAMDEC1	TEAD4	CNN3	LY9	SACS	CFHR3
APOBEC3B	RFX2	CD302	PCDHGC3	FPR3	FOSL1	WDR82	JAK1	RGS16	CCL5	ITGB1
KIR2DL3	SMARCA5	CD7	RNASE3	AEN	NCRNA00152	S100Z	FMNL1	DYRK2	CD84	XYLT1
TULP3	HCK	CFHR4	CD53	NFKBIZ	APOBEC3F	CD8A	ADAM19	CAMSAP1	POU2F2	LRRC25
FCGR2A	ODF3L1	CD48	FCGR1A	IL10RA	PVT1	ZFPM2	ANKRD50	CRLF3	MICALCL	GIT2
POLR3D	LMCD1	ITGBL1	WDR43	HTR7	SERTAD1	STK4	B3GNT5	MACC1	SH2D1A	ARL 11.00
TEAD3	RAB8B	EPHA2	CORO1C	SPC25	RGS18	FYB	NFIL3	ITGA4	SH2B3	TIGIT
TMEM155	C17orf87	JUNB	KLRB1	SP1	DDX5	TCL1A	CNTNAP1	TMEM149	C6orf145	SIRPB2
WDR44	CTSS	LILRB3	TNC	ASAP1	CEACAM3	LAMP3	SEMA7A	TIMP1	HAS2AS	CYBB
CD8B	GPR84	BICD1	GRRP1	CALU	IFNG	TGFB3	ST8SIA4	GBP5	CNR2	COL5A2
DARC	CA9	C10orf10	LRP1	IFNGR2	SPARC	DCP1A	GGT5	PIWIL4	ITPKC	IRF9
TPM3	CLEC17A	LAX1	TGFBR1	SIGLEC9	CHSY3	GXYLT2	TMEM59L	HEPH	MDM2	TNFRSF12A

NID2	GNA15	KRT80	NCKAP1L	MYO1F	LAMA2	RARRES3	TUBA1C	MDFI	FCER2	APOBEC3D
SFRP2	EMB	KIAA0020	KLF16	MSN	EVI2A	ARHGAP17	TRIM22	CD200R1	SFRS13B	FJX1
ARRDC5	LILRB5	CCDC88A	XRCC2	PAG1	CD5	SIX6	MAP3K14	CNKSR3	MSC	MYD88
TNFRSF10B	B4GALT5	LILRB1	RBMXL1	FGF10	RRAD	LHFP	COL11A1	CSDA	CD1A	SOCS5
PCOLCE	PHLDA1	GRAP2	HLA-DRA	JAK3	LIMK2	C9orf91	NOD2	IKZF1	WIPF1	PHF20
ULBP1	CLECL1	CCL21	BASP1	GALNT1	VCAM1	CCR3	CYBRD1	MAFF	MS4A3	SPN
ZNF831	CHRM2	TMEM156	CD33	FGD2	ARHGAP15	CD180	CARD11	CD40LG	ARL 14.00	HLA-H
MAP3K7	ASIP	AMICA1	PIP5K1A	HCLS1	C19orf38	ARID5A	NUP98	DUSP14	VWF	MTHFD2
ZCCHC6	FLI1	CTSG	FCGR2B	GNAO1	C14orf43	PCDHB12	ITGB3	GAPT	RYBP	MT1A
XIRP1	RAB32	CXCR2	TWIST1	NID1	CD4	KDM6B	CABP2	MS4A1	GADD45B	KPNA2
IL2RB	ZHX2	FST	HMGA1	RCN3	CCDC120	FGFR1	PAPPA	ICOS	TMEM39A	NRP1
C2orf77	HAS3	CCDC50	RAC2	FCRL6	CLEC4A	MLKL	GPRIN1	RHOB	PDIA4	DISC1
VCL	CPXM1	ARHGAP30	FKBP7	IL18BP	CYTIP	FCRLA	EMILIN1	C12orf35	HLA-DOA	KLF7
PIK3CG	RASA3	SFRP4	DNAJB1	RELB	COL29A1	ZFP36L1	TLR4	HLA-DOB	NGFR	SIX3
SLA2	SMCHD1	SLFN5	LSP1	ALOX5AP	ASPHD1	GNAI3	NKG7	STK10	COLEC12	GFRA1
IRF4	ETV6	BTLA	SERTAD2	MMP11	MNDA	SLAMF7	TNFAIP1	SOD2	MALT1	FAM53C
ARHGEF6	CLDN4	TLR10	DENND5A	SIGLEC7	WDFY4	MGP	ENTPD7	PPP1R12A	EMP1	PGK 2.00
CTLA4	CD3D	IL34	TRIM38	RAP1B	TRAM2	NCF1B	VCAN	SYNCRIP	NRP2	SLC1A3
PXDN	ACAP1	PLA2G2D	EDN2	CD3E	ICAM4	PRG2	KLRC2	ARPC5	SGTB	TXNDC5
SCARF2	CXCR2P1	MFHAS1	ISLR	TTLL7	TSC22D2	TNFRSF13C	NFAM1	CSRNP2	TMEM45A	CACNA1I
LILRA6	NLRP10	SEL1L3	IRF7	PAK3	GFRA2	FRZB	RBMS3	TMEM212	FCRL1	REG1A
SIRPD	LTA	PARP15	SERPINF1	RELA	GRM4	TBCCD1	SASH3	FIGF	CASP4	BAG5
HGF	DDX58	FLJ43390	GPR141	CILP	S100A2	ITGB2	SLC9A9	FAM83G	RAB23	LIMD2
RCSD1	HSP90B3P	TUFT1	LY86	FCRL2	EEPD1	SCT	TMEM173	GFI1	ALOX15	PRR11
USP6NL	HERPUD2	ITSN1	TNFSF11	KLRK1	SIGLEC8	BMPER	KLF10	F13A1	IFITM1	MYOD1
ARNTL2	KIAA0355	MRC1	RGPD8	WISP2	CLEC7A	PIK3R5	CD52	KIAA0802	ADCY7	C16orf72
SIRPB1	ITGAM	STXBP1	FCRL5	FRMD4B	FAM129C	C4orf7	KIR3DL2	BMP1	GPR174	FMOD
IL18RAP	CD163L1	HLA-DQA2	FCAR	C13orf18	FANCA	C1orf162	PRICKLE1	LOC652276	COL4A2	LAIR1

MMP23A	GLI1	ZNF460	SIGLEC14	DTHD1	BMP2	IL27RA	DKK 3.00	CENPA	DOCK11	CCNB2
LIX1L	KIAA0748	CTGF	YWHAG	CSTA	KLRC1	S100A16	UPP1	EBF1	LOC647121	DNAJB11
TREM1	S1PR2	VAV1	SNX20	C2orf89	POU2AF1	CRP	SPTA1	PIF1	ADRBK2	ABI3BP
CD22	HKDC1	RBM24	MC4R	ITGAD	SPON2	ABCA6	MYO1G	ARL13B	CLC	DMP1
EOMES	HK3	CDKN2B	ACTRT2	ASZ1	KRT26	KRTAP4-11	OR2W5	OR52E4	POU4F2	PRAMEF20
PRAMEF4	SP9	VGLL2	IL16	NFKBIE	MEF2D	ARHGAP11A	CRHR1	ITGA5	BAT2L2	FAM129A
CD14	HSP90B1	SAMD3	IRAK3	ETV7	EMR1	PCDHGA5	CD2	WEE1	MS4A2	C1QC
SIRT1	CPA3	TXLNA	MATN3	PMAIP1	RIPK1	C17orf64	ACTB	MOXD1	ASCC3	SCN1B
OTUD4	BARD1	PRKCB	PRPF38A	EREG	PARVG	IL10	UGDH	GLIPR2	CDK17	IL2RG
C9orf110	AGAP2	CXorf21	SYN1	NCF1C	PCDHB15	BRCA2	RAPH1	TTC24	TKT	SIGLEC10
ERCC6L	IL17REL	LY96	NFKB2	RNASE6	GNG2	RARRES1	PRAM1	TTC39A	WAS	ABHD5
CORO1A	NBPF14	HCST	SYNC	PRELP	LPGAT1	P2RX5	GMIP	IRAK2	ITGAL	LYN
TAS2R43	IFNGR1	UBAP1	CDH6	LAT2	NCR1	PYGL	RAI14	DDHD1	GPSM3	TFPI2
C12orf5	MSR1	FOXN2	GPR55	SKIL	SP140	VSIG4	YWHAZ	IER3	TPSAB1	ZNF80
MEX3A	CD6	PARP8	ABCC9	CTSW	PVR	CXCL11	NFYA	CDC42SE2	GGTA1	FILIP1L
KDM5A	NLRC3	LRRC15	CPZ	MAPRE1	GPC4	CMA1	DGKD	IL21R	TUBB6	CD38
SIGLECP3	KLHL29	S100B	CD74	FERMT3	HRK	TLR2	INPP5D	LOC283856	CUX2	SLC8A3
POSTN	C1QB	GMFG	EFNA1	CLEC9A	TYROBP	MS4A6A	HLA-E	C16orf54	APBB1IP	GAB2
TM4SF4	GPR161	STAT4	LOC727896	KCTD20	DEGS1	PPFIBP1	AOC3	NOTCH2	ELF4	RAB42
FAM198B	CHRNA6	LY6H	FCER1G	ARHGDIB	FOXO3	ELANE	OAS3	RTKN2	STAT1	HDX
BEND6	KDM5B	FAM114A1	DLGAP1	ADCYAP1R1	SERPING1	UBC	C6orf204	C1orf200	HOPX	MKI67IP
CHRNE	FCGR2C	CMTM3	DDR2	PDGFRA	TLR6	PRAME	TRH	KLF5	CLDN9	FNDC1
LOC641298	HRH4	SPIB	PTGS2	KIF21B	ZNF365	REG1B	CXCR1	MAP4K1	SIGLEC1	FYTTD1
PPM1D	FAR2	SIRPG	IL1B	OSR1	PTCRA	ITGAX	CDC27	SCUBE2	GPR114	PCDHB14
SLAMF8	HLA-A	CD1C	KRT8	CEACAM4	NLGN2	CDC7	HLA-DPB1	CYTSB	CCR2	CADM3
TOP2A	CCL13	RNF19B	FHL3	HSPA5	CCR5	GBGT1	CAPZA1	FAP	GLI2	SDK1
TMEM196	ATP2B4	MAP7D1	PTEN	PLEKHO1	INMT	IFIT3	ZNF800	AREG	CTHRC1	MEIS3
BATF	ANP32D	C12orf4	TGFBI	B3GNT7	CRISPLD1	RRS1	CLIC2	CHD4	IL9R	FPR2

LOC100130331	NFKB1	GMEB1	C2orf18	RASSF3	DUSP22	RLTPR	S100A12	CNTLN	CALD1	UBASH3A
NUFIP2	FKBP11	CD1D	LPAR4	OLFML1	FCRL3	SLC24A4	LATS2	CLEC10A	RBP1	KCNA3
SLC7A11	IL33	EXT1	GABPB1	HTR2B	NAPSB	TAGLN2	SH2D2A	LDLR	DOCK10	ADAMTSL4
C2CD4B	ANGPTL7	ZNF217	A2M	ZNF749	KCNF1	PDGFRL	NLRC5	CAP2	IFNAR2	SLC25A25
HLA-DPA1	CDCA7	KIAA1210	NR2F1	ZNF101	CD5L	PCDHB10	PTPN12	RNF217	CD72	DNAJC5B
PLA2G7	NCF1	TBC1D2B	SWAP70	KAAG1	ACTN1	C1QTNF1	PDLIM5	TMC8	SRP72	UNC13D
KLHL25	SELP	CXCL6	BTN2A2	ZNF643	CD37	DEPDC1	PNRC1	APOB48R	BLM	AKNA
NELL2	TLE1	MELK	SIT 1.00	CXCR3	FGFBP2	NIN	DERL1	MOBP	MEI1	PLB1
PCDHB18	C3AR1	JMJD1C	HLA-DPB2	GLT8D2	MED17	MRC2	KIF2A	SCGN	MAP4K4	PIK3R6
BAI2	HYOU1	PLD2	MED6	TES	KCMF1	LILRA2	MORF4L2	VASH2	IFITM3	CD300A
LRRN1	CLEC2B	TAP1	RGL4	C12orf68	LOC285205	CTSZ	SLC37A2	PTPN2	GTF2E2	IL11
SNCB	CITED1	ABCA9	CASP5	CHD1	LST1	TLR7	PBRM1	TNXB	CHORDC1	CDON
SLC46A2	C5orf58	GPR176	CFB	VASP	MS4A4A	B4GALT1	MIDN	ZEB2	SLCO5A1	MIXL1
PAPLN	DDX60L	C17orf96	EZH2	CXCL10	MFAP4	FILIP1	ZNF267	FAM7A3	ZNF683	TUBA1B
CSF1R	SMC6	CD27	LOXL1	PAX5	C12orf61	RNF138	NUDT10	GPR15	TTYH2	ARAP2
CD200	CD177	SH3GL1	C13orf29	ATP11C	GDF11	HPVC1	CDC42EP4	STATH	FKBP14	CCR6
C6orf174	FUT8	CYP19A1	CBL	LOC644936	EFEMP1	BCL10	C17orf62	GPR3	PLEKHA4	C1QA
BTN3A2	LOXL3	TGFB2	PSG1	ODF2	HNRNPF	BYSL	RIPK2	BUB1	C1RL	MKI67
PGA4	ITPRIPL1	MTA2	KBTBD8	LRRC8B	FAM126A	LRRC8E	C1QL2	MAP1LC3C	P2RY8	C20orf103
UTP14A	FAM116A	NLRC4	MEGF11	CSF2RA	ZNF595	CCR4	PHACTR2	FSHR	SIDT1	MID1
C19orf75	DAD1L	FAM181A	GRXCR1	GUCY2F	IL28A	KRTAP13-4	NKX6-3	OR52D1	PRG3	PRO1768
RBMY1A3P	SDC4P	SNORA31	TARM1	TAS2R9	GZMM	PCDHGB2	DOK2	PABPC1	NCF4	FLNA
CLEC4D	CASP8	FMO3	FCER1A	CASP3	LOC100133991	HIVEP2	EIF2AK3	SLA	RHOG	MYLK
GRK6	FAM101B	NLGN4X	NCEH1	TRPC3	UNC5CL	TNFRSF10D	TWIST2	C10orf105	NCR3	ZNF35
CSRP3	PDCD1	FCHO1	ST8SIA1	CEACAM8	MAP3K5	NKAIN4	PTCH2	LOC284441	XBP1	MMP17
CCR7	BCL6	ZNF469	PMEPA1	UGGT1	OR10A3	ANTXR2	CXorf22	DLGAP4	MAP1LC3B	FEZ1
KIAA1324	CCDC146	ANXA2P3	SEMA4B	SIPA1L2	LTB	LILRA1	WISP1	LOH3CR2A	SH3PXD2A	CCL19
PIK3CD	TINF2	AP4E1	ZNF410	ANKRD57	APAF1	CSF2	LOC339524	ALMS1	IGSF6	B2M

NCK1	RAB20	C2	CDCA2	EPC2	MICB	MAP3K12	C7	RNF182	PTGDR	LEF1
FNBP1	KIF3C	GATS	CXCR5	PSME4	S100A8	IL17RA	S1PR4	C1QTNF6	GTDC1	ZNF878
UBE2H	HNRNPAB	CXCL1	UCP1	LOC100128164	STK38L	C20orf141	SLC7A2	BLK	BCAT1	CYSLTR1
IWS1	CD19	FAM72D	ZBP1	LYPD1	MLL5	DIRC2	ARHGAP9	BMP2K	LETM2	BRPF1
IDO1	PTGER2	PNMA2	CILP2	NDC80	KCNQ3	ISLR2	CMTM2	IGLON5	GPR37	NEXN
COL6A2	SCG2	LTBP2	EBI3	SPI1	C4orf11	CCDC140	DEFB103B	MUC7	SPAG11A	UBTFL1
TNFRSF13B	HS3ST1	CYTH4	ERAP1	SLC1A7	PSD3	DTL	PLXDC1	NUPL1	IKZF4	MACF1
CD80	TBX20	C4orf6	C22orf9	LOC285830	ENDOD1	TRPA1	NNMT	PLK2	IGJ	LYVE1
IER5	RELL1	TLR9	IMPDH2	PDE4D	CSNK1A1	FLJ16779	TEX19	TRANK1	PLEKHG2	TRPV2
BCL11A	RTN1	HAUS2	PI15	OTOP3	FAM171B	TMEM185B	GKN1	SDC3	RBM9	TNFAIP2
SH3BP1	GPR18	RFPL4A	C19orf22	TAP2	PLIN3	ADH1A	ARL 9.00	CASP1	APOBEC3G	SLC16A6
CHI3L2	ST3GAL2	LRRK1	SH3GLB1	KRTAP1-5	PPP4R1	VWCE	RASGRP4	NRAS	ALOX5	RASAL3
FHL1	IGDCC4	DOK3	BZW1	TRA2B	RAB33A	HDC	LPXN	EXTL1	DTNA	FLRT2
C1orf150	CCDC9	PGLYRP1	EVI5	PABPC3	RPGRIP1	COL14A1	RASD2	CIITA	STYX	CASS4
FCHSD2	PCDH17	NPC1	WTAP	E2F2	EGR1	PPIG	DEF6	EMP3	TTC39C	ZNF532
MGC29506	GPR56	RAB31	CDKN2BAS	SPAG1	RBBP6	RPL12	PAQR4	SEMA4C	CSNK1G3	TIAM1
D4S234E	IL4	ITGA1	TIFAB	LOC152225	ACTR2	ZAP70	RGS22	ZPBP2	SVIL	KCTD11
TNFRSF10A	RANBP17	INSL6	CNKSR2	GNB1	BRCA1	TBX5	CCL4	KIAA0226	CYLD	PITPNM1
HIF1A	PLCXD2	TBXAS1	SDC4	LOC100188949	TUBB3	GRK5	HMHA1	CH25H	BTBD19	DKK 2.00
DIAPH2	LOC283070	FOXG1	Myd90	KCNK6	LRRC4C	LCK	HPR	CCIN	ARRB2	ADRA2A
TRIM16	GPR142	SEZ6L	CHRD	KCNH8	PSTPIP1	FAM72B	PROCR	PER2	MNT	OSCAR
ZAK	PPARD	TAF5L	CDC6	ITPRIP	C12orf63	C3orf64	SPATA12	CCDC46	NEIL3	TRPM2
CLSPN	SLIT1	CD101	PDCD1LG2	MFAP2	DLX4	MUC12	FBXW7	ATP2B1	ELAVL4	LRRC32
SLC38A8	SYT16	DCBLD2	BCL9	IQGAP3	TXNDC3	CCND2	RSU1	SH3RF3	C6orf25	CCL11
SERPINA3	CLSTN2	UNC5A	ETV3	RALGDS	EFHD2	IL18R1	OSBPL10	NT5DC3	PIP4K2A	SLC10A6
CHODL	PROK2	PPP3R1	TRIM62	ZBTB32	CNTFR	CAPN11	RGS19	GVIN1	LOC96610	PSG9
CCL20	KCNA2	NFIX	ATF7	PRKCH	NCOA3	ANKRD44	INTS8	KEL	HR	MTF1
NECAP2	ZDHHC18	HSP90AB1	LOC100130386	ARMC3	FOLR2	TLE4	DGKH	HRCT1	SSC5D	DENND4A

ITGA9	DLGAP5	MARCKSL1	MYBPC2	MYBL2	HMCN1	CCDC102B	RASSF1	ODZ3	FCGR3B	C14orf174
ADCY3	CECR6	SERPINB11	KIF2C	HS3ST2	SOCS1	RIN1	TMCC1	CD79A	BTBD10	ERI1
MAP1A	UBE2D1	TMSB10	RBM12	LOC442308	ZNF597	ZNF644	R3HDM1	SGMS2	SLC35C1	MOBKL2A
TLR1	TNKS2	OR52N4	DCX	KRT24	FEM1B	MMP10	VSIG1	VIPR2	RGS9	TRERF1
GINS4	HHIPL1	ADIPOQ	UBD	TEX11	RNF125	DNAJC2	ADORA3	ISG20L2	TNIK	SP110
LAIR2	PHF20L1	BIN2	SLC41A2	DPY19L1	TSPAN18	NMI	CENPF	RAPGEF5	MAN2B1	AZIN1
NUP160	PDGFRB	MEX3D	DONSON	ESYT1	COL21A1	B3GALNT2	EIF5B	TEP1	C14orf145	KRT18
PRND	NAV1	PLXDC2	PRR7	BTN3A3	RPL10	AMMECR1L	C10orf54	SHISA5	SYTL1	LONRF1
RGS14	DCAF12	XCL1	KIF5A	FUT7	RAB35	CLDN1	LOXL2	NOL8	PSTPIP2	GALNT7
PABPC4	HNRNPA1L2	LOC283731	DPEP2	IL31RA	HK2	RAB30	LOC100233209	TPM1	CCDC96	PREX1
KLF8	HEYL	RPS2	CXCL9	PLBD2	EPSTI1	HNRNPA0	ADAM17	ACTL8	LGALS12	MAGED4B
SGCD	CD276	ENC1	CDC37L1	HOOK3	KCTD4	SORBS2	MAPKAPK2	PLCB4	FAS	GNB4
BCAR1	TMEM43	FYCO1	FERMT2	CXCL16	ANXA2P1	CXCL13	PITPNB	SYCP3	ZCCHC24	TRIM40
FOSL2	TEX10	PLSCR1	C10orf18	LOC145820	PTGER4	FBLIM1	GTPBP4	MET	CLEC12B	LOC400759
PCDHB6	TRAF3IP3	PLCH1	40057	SOBP	ARHGAP20	NKX2-2	GLIS3	SMOC1	LYG1	TBC1D10C
SHOC2	HBM	KLRG1	RRM2	TMEM179	FOXF2	RXRG	ZNF487	NBEA	MIAT	LIPN
FBLL1	PUS3	MMD	PLD4	GRIA1	FAM26F	DKFZp761E198	XCL2	LOC606724	LIMK1	CRLF1
CHST7	ROCK1	ARHGAP25	LGALS9	ZNF609	FOLR4	C11orf21	KIF4A	PAK1IP1	SOX21	PLGLA
TRIM9	DGKG	ADCYAP1	AKAP13	LAYN	HUS1B	XPO1	RSPH4A	RNF41	CACNA1G	C10orf128
GPR124	SAA1	LRRC41	FAM135A	IL1A	PTPRCAP	BAZ1B	NEGR1	ATP10D	FAM160A2	SETX
HCP5	NFE2L2	TCF12	ADRB3	C6orf114	EDNRB	JUND	TNIP2	LGALS9B	C2orf85	SOAT1
SPON1	COLEC10	IGLL1	HHIP	CBFA2T3	FAM49B	RGMA	STAT6	PIN1L	TUSC5	MPO
PCDH19	MAS1L	BMP5	PIK3AP1	C11orf9	FAM46A	ACHE	DERL3	MYB	ACTN3	UHRF1
ITGB8	IL1F7	COL8A2	CDC20	LYST	PTPN7	ADAM9	PURB	IL32	STAT2	HVCN1
ITGAV	LOC100129550	ATF3	CD244	FASLG	IGFN1	GAL3ST4	COMP	KIF14	TRAF4	PCDHB13
KIAA0408	SPRY4	REL	KIFC1	POLR3C	GNL2	PGM3	CD99	GPR132	POTEE	SMURF1
RAB9B	ADAM28	KCNB2	C15orf42	FBLN7	ARHGAP10	FOXO3B	CDR2	CCDC109B	ROPN1L	SLC18A2
GMPS	POLR2D	RASSF6	ALPK3	HRASLS5	PML	OSGIN2	ANKFN1	LECT2	SHROOM2	TOPBP1

ZDHHC5	GM2A	GCNT1	ZNF367	SDS	CYP1B1	CLDND1	RPL31P11	ERN 1.00	CD1E	P2RX7
CAMK2D	SETD7	MDK	COL12A1	UVRAG	GNLY	HLA-F	STK17A	CWC22	PLCB2	SP7
MEF2C	TNFRSF17	TTC29	C11orf82	DNALI1	DCHS2	TREML4	ADAM8	IL23A	KRT86	DDX18
CDC42EP3	MID2	PDE5A	BMPR2	ACAN	SMOX	KCNK17	AMOTL1	ARID4B	NAB1	CORO7
FAM9B	PCDHB2	ACAP2	CRYAB	FAM78A	CD226	PMM2	HSPB6	ERF	XKR3	ADAMTS16
FAM110B	TECTB	HJURP	CDK12	PRKAG3	C4orf50	ACER3	PLIN4	C7orf57	NFE2L3	LTBP4
CCDC97	CYP2C9	OAS2	TPRG1	ASPM	KIAA0922	TWF2	PHF19	PIGR	MYO3A	ASF1B
NCRNA00093	SRPX	ICAM3	HLA-B	HSD3B1	CFL1	MX2	LRRC8C	SPAST	ACVR1	CCL24
GPR4	EIF2S1	YWHAB	ENOX2	SENP1	CA13	ARHGEF38	C17orf60	PLP1	C20orf186	KCNN4
WDR26	ITGA11	REPS1	REEP1	PLAC8L1	MYO1D	PAPOLG	MCHR2	PDZD4	UBXN2A	MEOX1
FBXO41	APOL4	HSD17B6	GNAI2	AKAP5	CCDC55	FAM71B	CNGB1	C17orf53	DKFZP434L187	ANLN
SPATA5	PPBPL2	OSBPL11	DNAJA1	EPB41	SYT12	EID3	TCTEX1D4	TRPC2	COX6B2	NFIA
TNFAIP8	SEZ6L2	KIAA0040	MEIS3P1	PPAP2C	MKX	C17orf67	KIAA1274	37135	DPYSL4	AFF4
CEBPD	YTHDF1	LOC100126784	PTN	RIOK1	CCNA1	LRRN4	KIAA1211	RIT2	FOXP3	PTRF
PSD2	KCNT2	FAM105B	AIM2	ODC1	STMN2	BIRC3	PRR24	OPRK1	ALPK2	CLDN3
ZNF423	TIMD4	SYTL5	GABRA1	UCK2	MIOS	FAM110C	ZNF259	FXYD5	MSH6	FAM180B
S1PR3	SLCO1C1	ARHGAP26	HSPA6	ADAP2	CARS	B4GALNT4	RPS8	RBBP8	SLFN12L	IPMK
PSG7	ASPN	HTR2A	P2RY13	MMP7	TFPI	ULBP3	LOC284276	C1orf173	SH2D5	MGC45800
YPEL2	PPP1R1B	XRCC5	BCL11B	RHOQ	ADAM6	KIAA0125	CRISP3	LOC645166	FOXD4L6	EZR
KIF7	INPP1	MMP23B	PTPRK	KIF20A	UBA6	CD248	TNNI2	ODF3B	STK38	GAB3
NRBP1	C14orf106	SETBP1	TYMP	CHPF2	IL12B	CEP55	JUN	POLR1E	LOC653653	POU4F3
REV3L	CHST15	CSNK1G1	WDR75	C10orf26	CAP1	FOXP1	ZNF280B	SRGN	OGN	VANGL2
ROR1	SMNDC1	KRT17	CRY1	MAP1LC3B2	PCDHB7	MEGF10	ADM	TP53	MXRA8	GABRR1
SEMG2	NEU1	LHX5	SGK269	CCDC88B	ZRANB3	LRRN3	MAP3K2	TPX2	OR10V1	PF4
FAM113B	AKIRIN1	EDN1	FAM111B	RBM38	OTOF	C13orf36	TAF1A	UBXN10	LEFTY2	LBH
TRIM32	FRMD5	CRK	RIMKLB	PDGFA	FBLN1	TCN1	SPRED1	NIPAL4	SIRPA	DEPDC6
IL15RA	HSPH1	PLEKHA2	TMEM163	LAMC3	MED13L	TMEM154	HLA-DQA1	LOC282997	CAMK1D	NPFFR1
FAM55C	PLEKHA1	PRDM8	DIP2B	ARG1	YWHAH	DDX60	CCDC19	EPC1	SPP1	WASF1

EIF4A3	PACSIN1	AP1S2	NIP7	C12orf45	TSPYL6	LOC399959	PRICKLE2	RAB43	SNAI3	TTC28
MPZL1	LIPE	PRSS21	CD79B	ECM2	RADIL	CFD	KDSR	C2orf39	FRMD8	PSMB9
PHTF2	DYRK1A	ZDHHC20	HYDIN	SLC39A1	GPR34	SKI	STAC3	MEIS1	TMEM151A	HAVCR2
FOXA1	AXIN2	JMJD6	PMP22	ZNF876P	NTF3	CD1B	PLEKHM1	CDH22	THG1L	SLC17A2
TAF4B	CPM	NPTXR	YAP1	CBLB	MLC1	FOXC2	RPL13AP20	ARMC4	SCN5A	P2RY14
SPOCD1	GUCY2D	PELI1	ARIH1	EIF2C4	PPIL4	PDLIM7	SNTB2	SLC25A32	KCNIP2	ZSWIM6
SHANK1	ZNF430	DAAM1	LOC723972	IFITM2	EGR4	PPPDE1	SMAP1	ELMO1	HAR1B	HTR3A
NPTX2	BTN3A1	MYOF	TMSB15B	ZNF608	TAF1D	TGIF1	SLC24A3	KCNQ5	EDNRA	SAMD4B
AVEN	S1PR5	PPP1R3B	ENTHD1	NCRNA00120	IL12RB1	HIC1	C1orf124	36951	CLDN14	RCC1
RTP4	PAM	C11orf84	VPREB3	UGT2B15	PHF16	RRP12	LYPD5	LRRC37A2	C14orf45	CCR8
EFEMP2	GUCY2E	LEPREL2	ARL4A	RGS20	TMEM158	PTPN6	DNTTIP2	VPREB1	H6PD	GNL3
FAM40B	MYO9B	DPYSL3	KIR3DL1	FEV	MSRB3	ADNP2	KDELC1	SHD	ARHGAP23	SLC15A3
NRIP1	DCLRE1B	OXTR	EMR4P	WDR69	HSPA1B	KLHL5	FGD6	C6orf115	MARCKS	UCN2
IQGAP1	DGKA	ZNF165	EMID1	TTPAL	CDH19	ARF4	MED14	GATAD2A	IL12RB2	NCAPH
SGK223	PSMB10	FGF13	DNAJC3	PRC1	CPT1C	TRPV5	EHD2	MAP3K8	RNASE1	BTN1A1
C6	KRT78	APOL2	RPGR	PAMR1	PTX3	PKNOX1	NAP1L1	TADA2A	GAS2L3	SOCS6
TNFAIP8L2	CLIC1	SYNPO2	SLC1A4	IKZF2	BGN	IL22RA2	WARS	PHEX	C9orf25	KPNA4
NONO	DEM 1.00	CCRN4L	OPN3	NUP188	HLA-DMA	NBPF15	RSPO1	GPR87	KPNA3	CASP10
CLEC4E	CDH23	RECQL	DNAJA4	UBQLN2	PVRL2	GSC	CMKLR1	RAX	BIRC7	37500
MAGED4	CYFIP1	IFT57	EDA2R	SULF2	RHBDF2	MAPK7	LRCH2	FAM60A	PTRH2	CALB2
CITED4	C1orf186	IRF2BP2	IKBKE	CD70	SLAMF9	CCNJ	PKNOX2	CSNK1E	AHNAK2	CPNE5
STMN3	BCHE	GATC	LRRC42	JOSD1	PLSCR3	CHRNA7	C10orf119	CDHR1	KAL1	TMEM130
IL6ST	BACH2	PCDHGB7	SEMA6B	ATG12	ITGA2	OR10K2	TGM2	UBE2I	TMIGD2	SLC22A16
MARCO	IGFBP6	GPR143	FAM23A	SULF1	C15orf39	GRIK4	EPHA7			
**Coexpressed genes with Myc in KIRP (Stage 1)**										
HBB	NUAK2	HBA1	HBA2	RPL7	CCT4	EIF3H	HBM	COCH	GDI2	GRHPR
HBG2	EPB42	RNF19B	HBG1	KCNK10	CCT2	BYSL	EEF1A1P9	INHBE	GADD45A	RAN

TRIM35	PRMT5	DUSP8	POLR3D	MAFG	HSPA8	C4orf14	CCT5	PPP2R2A	GALK2	ARHGDIG
KCNG2	LASS1	ZNF259	FXR1	MTFR1	MGST1	EIF4A1	SLC17A1	SLC9A3R1	SLC13A1	METTL13
SLC47A2	SLC47A1	SHMT2	GRPEL1	BAG2	GARNL3	PYCR1	TCP1	PLK2	SORD	AFG3L2
GPATCH4	KIF1A	EIF3E	UMPS	GPR89A	KPNB1	WDR12	EPS15L1	CGREF1	MGC14436	RSL1D1
MRPL45	GTPBP4	RPL10A	MAPK12	CHMP1B	NLRP6	TARS2	NPM1	C22orf28	HSP90AB1	UBAP1
IRAK1	EIF4B	ECHDC1	CYR61	ST13	MRPS27	ACOT4	EEF2	TMEM144	RGL1	MMADHC
ALDH1B1	CIRH1A	OPTN	DCAF13	C2orf47	EEF1B2	PPA2	CCT3	C1orf43	C6orf106	NOP14
C1QBP	C11orf54	CCR9	AGXT2	ATP6V1B2	COX15	DAP3	CRTAP	F12	MRPS30	TFB2M
ALDH1A1	TOMM20	NCRNA00113	VCP	DPP3	PLEKHA5	TKT	RPP40	NIPSNAP1	TMEM177	AQP11
UBE2R2	EIF2S1	NOLC1	ATIC	TRIM32	MRPL3	CCRN4L	GPN3	C1orf107	EEF1A1	TEX10
SLC31A1	TNFRSF10D	PANK1	NMD3	SNHG5	ETF1	C1orf163	TFDP2	PKLR	AGMAT	PGK 1.00
NOB1	HADH	SLC15A4	RAD23B	TIMM8A	TMEM106A	LOC285733	ACVR1	VRK3	ACTG1	DAB2
DNAJB5	PSMD11	INHBC	LCN10	RABGGTB	RRAGA	ACSF2	TAF9	CCNG1	MRPL44	GDF1
AK3L1	SLC22A13	FAM10A4	PTDSS1	CCT7	UTP3	TDP2	LRPPRC	EFTUD1	RABEPK	XYLB
SPG20	C13orf38	APBA2	TARS	GPD1	PARD3	RMND1	MDH1	KIAA0020	FAH	BRE
SLC5A10	AVEN	PGAM1	SLC26A1	HINT1	PGAM5	FARSA	EIF2A	ADH5	IARS2	NOX4
MSRA	C13orf1	PRPF18	TOR1A	MAD2L1BP	BTF3	STAMBP	BECN1	BPHL	ZNF622	PEPD
GPR89B	PPA1	PELO	MTHFD1	ASH2L	PRDX3	TMEM14B	HSPD1	IGBP1	BCL2L13	CRYL1
HPN	BAG3	LAP3	PECI	AKR7A3	ACTR6	ANKS4B	NCRNA00188	RAB5C	C17orf71	SLC25A30
RPL7L1	PPP1R15B	ILF2	BHMT2	UGCG	CALM3	POLR1E	MAP1B	PRKAG1	SLC35D2	NCRNA00120
ANPEP	PSMD2	PGAM4	GEMIN4	ENPEP	HTATIP2	RARS	HSDL2	CDV3	KIAA0100	NGLY1
TNFAIP8L1	SLC39A14	CHMP5	MRPL42	SLC28A1	TRAP1	IFFO2	STOML2	CBX4	SLC25A5	RARS2
FAM177A1	VDAC1	ECHS1	ETFA	SLC27A2	RBP5	SLC1A1	NACA	PSMB5	CCL14-CCL15	SCO1
HSPB8	SLC7A9	DARS2	TM7SF3	C12orf11	LACTB2	BPNT1	RPE	ACY1	MRFAP1	PABPC3
EEF1A2	EHHADH	DYNLL2	EIF3C	RPS3A	C21orf7	GPN1	CYB5A	MCCC2	ACAT2	RNASEH1
RBKS	CES2	SLC39A11	EXOSC3	RRS1	WDR43	DHTKD1	GHITM	SLC16A4	DRAM2	PCK2
LRP2	C6orf57	F7	TP53RK	TUFT1	SLC22A2	DCTN6	NEDD4	SQSTM1	RIPK2	YARS2
GRINA	SLC23A1	TAF4B	IARS	KLHL12	STRAP	LONP1	ZDHHC9	TMEM206	SLC25A32	NACAP1

MAPKAP1	PRPS1	PCCB	PSMD10	ALDH6A1	APOBEC3A	SAMM50	CNTNAP1	ECE2	FXN	TM2D2
RIPPLY2	RPUSD4	CISD2	YIPF4	AKR7A2	TTC35	RPL3	FTHL3	NFIL3	RND3	SLC25A25
HSPA9	IPO4	AIFM1	C5orf30	RNF5P1	MLX	CMBL	KHK	PCCA	ALDH9A1	CPPED1
SDHC	MRPL50	SCFD2	ABCB7	RPS12	SSTR1	NUDT5	DPH3	MRPL37	SSR3	CLTC
MRPL46	NDUFS4	RETSAT	CLP 1	RPL17	PIPOX	SENP2	ERICH1	GATM	ADSL	CORO1C
MAPT	SETD3	MMP14	NDUFS2	RPL26	C4orf3	MRPS35	TRIM16L	RBM7	MMS19	KCNJ12
LDHB	ETNK2	PLD1	XPR1	GART	IMMT	FKBP4	PCTP	ABCC2	FAM96A	TRIM16
MRPS23	F2	ISM1	C22orf45	PPAT	GNPDA1	SH2D5	ATXN10	ALDH1L1	DCTPP1	SFXN1
EIF2S3	RNF152	SEMA5A	C6orf120	SPAG5	C9orf30	WARS2	OAT	DAK	C1orf57	C6orf72
PBLD	FDPS	GMPS	DNAJC12	PABPC1	ACAT1	AKR1C3	AARS	EIF4A3	NEFL	ACADM
VDAC2	ATF5	YRDC	HAUS6	SOHLH2	PRODH2	RNF123	ESD	FTL	NR4A3	MRPS22
C20orf24	DGKG	PRDM4	MFI2	TSPYL1	RAB7A	HECW1	TMLHE	ABCF1	CYFIP2	ZNF367
GLYATL1	LRRC59	RG9MTD1	FAM82B	UCK2	INADL	TTC9C	C21orf33	PDZK1	OSGIN2	ATP5F1
HNRNPH2	CDC123	GNL3	CLSTN3	MARS2	ZNF697	UBXN8				

Coexpressed genes with Myc in KIRP (Stage 2)

GDF15	ZFP36L1	IGFN1	SLC16A10	ZBTB25	BRS3	DIAPH3	PRPF18	TUBB2B	LOC144776	VTI1A
PIM1	SERPINE3	KLF6	MPZL1	NFIL3	C1orf187	PPIF	RAP2B	HKDC1	CHMP1B	NGLY1
KRTAP1-1	VGF	MYPN	RND3	ADM	TMEM161B	MEP1B	ARHGAP28	PPP1R3B	TAS2R13	CSNK1A1P
SLC16A1	ASB3	KLHL33	PIM3	TTC28	TP53BP2	CDV3	NSUN3	NCRNA00120	NLN	XIST
BEND6	SNORA4	CRABP1	NAMPT	AGTPBP1	SEMA3E	PIK3C2G	PTP4A1	PSMD7	LARS	HTR3C
LOC645676	PGM3	PI4KA	PLAC4	BDKRB2	ATXN8OS	PSMB2	HIF1A	RNF112	C7orf52	EIF1B
PPM1D	LSAMP	KIAA0368	CARD6	FAM71B	OR10K2	SLC22A25	ANXA8L1	AWAT1	C4orf17	C6orf10
FGF16	FOXD3	GRXCR2	KRTAP12-1	OR10T2	OR4F21	OR4F5	SYCP1	TACR3	ERICH1	ROBO2
CISH	KLHL1	DYRK3	PCDHA4	DNAJC5G	CEP170	NRAP	FAM153C	FOSL2	EPDR1	FAM153A
QRSL1	JOSD1	ING2	FLJ41941	HMGB3L1	LOXL2	ARHGEF3	YBX1	PATL1	FLG2	PLK2
TKT	TMEM49	SAAL1	RAG2	C21orf130	BCL6	TGFBI	C4orf26	GXYLT2	FILIP1	POU4F2
EXOC6	NUAK2	SNORA62	GPR135	C3orf27	DARS2	FXR1	SLC28A2	SNAPC1	CEP78	DAP3

LOC100130148	SOCS3	DCDC1	PCDHGA9	P4HA3	BDNF	CCRN4L	KIAA0391	NEAT1	LCORL	DEFB103B
IMPDH2	EXOSC10	STX17	ASFMR1	GAL	STK17A	VLDLR	PALMD	GPR12	CHP	PHF7
COL25A1	PROS1	PGD	UBC	MGC16384	CD274	ZNF460	PPP1R15B	PPP1R15A	RPL23AP82	ATP12A
B3GNT5	GABPB2	ZNF57	KIAA1919	DCUN1D3	ZC3H12A	GPR179	PSMD11	DKFZP434L187	XPNPEP1	C9orf30
INTS4L1	C1orf107	LOC727677	OR2AE1	LIF	NKX2-4	MPHOSPH9	C14orf126	LOC283731	LOC285629	MNAT1
HBG1	OR4F4	FAM119B	PIR	DNMT3B	SEC14L4	RNF10	TTC39B	DNAJC25	TMEM136	EPB42
TTLL7	JUN	FLJ14107	ART5	KIAA1328	KIAA0020	LOC729609	ABL2	MLLT11	GADL1	ZFP36
BECN1	LONP1	CLSTN2	LIPI	PIP4K2A	HTR1B	PTENP1	DFFA	NCRNA00169	EXD2	PGM2L1
RNF122	CEP170L	RNF180	HTR1F	HIST1H2AL	TCP11L1	PIPSL	ZCWPW2	PSMB5	C2orf57	GCLM
PRO1768	KRTAP5-1	HMBOX1	BMP2K	OR51B6	PSG2	HNRNPC	KIAA1598	KIF2A	KALRN	CFH
NR6A1	RARB	THOC5	NMT2	RFX6	GABARAPL3	KRT33B	SNORA6	GPRIN3	FAM123A	ISLR2
ZBTB38	C6orf174	MAP1B	C11orf74	ANKRD26	TTPA	NPC1	MDGA2	KCTD4	TUBA1A	PLA1A
FGF3	LOC100302401	AP4S1	SPATS1	SPSB1	LDHAL6B	C10orf68	KRT76	OR9Q1	ZNF669	PAPPA
TAS2R43	METTL6	GCNT2	FHOD1	ANKRD36B	STAM	FAM66E	ZNF584	SLC4A1	GATA6	IL8
LOC729603	PFKFB3	PHF21B	HIST1H2AK	PDHX	C3	MAP7D3	STRBP	AXIN2	FBXL20	FAM41C
GYS2	SPATA1	NEU3	WDR88	TPTE2P1	IARS2	LOC100132288	KRBA2	STT3A	BPNT1	C14orf109
GNA13	ANKRD36	CXCL2	DHX32	LOC100128292	ZNF418	SMARCD3	RNF217	NUBPL	COQ2	PGBD5
KIAA1632	DOCK3	C6orf59	IFFO2	TAS2R31	UTP6	ZNF70	NRP1	SMS	SRP54	ATF3
PDZD8	RPL23AP64	C3orf26	MGC2889	DMRTC1	SLC26A5	MMP14	TIMM8A	RASEF	SYT16	ARL4D
LMOD2	OLIG3	ZNF322B	INHBE	C13orf38	RPL26	C22orf43	ZSCAN20	CHD1L	OXSM	AKAP6
POLR3G	LOC100128640	NRD1	NAV3	NDUFS2	RPL23P8	ACTR10	IQCB1	LOC401052	SSR3	SAV1
OPN3	SEC23A	RNF214	PRICKLE2	SNORA13	ZNF208	VWA2	EIF2C3	KLHL12	NAV1	TAF13
SLC39A2	NBPF9	EFTUD1	H6PD	ZNF611	RAB31	TBC1D3P2	EXD1	WARS2	MSH4	LOX
FLJ22536	DHDDS	MAPK8	IPO4	POU2F3	NME6	RAN	PSG8	RG9MTD1	WDR11	PTPDC1
LHFPL4	PELO	LOC154822	PWRN2	ADK	ZBTB37	TUBB2A	ZNF449	HMOX1	SLC2A12	MMP17
CNN3	TAS2R30	ADORA1	C10orf62	C3orf16	CSH2	KRTAP5-11	OR52H1	MAP1LC3C	TEX2	ZNF326
DNM3	NGDN	MYO1B	MID1	RBM17	TSNAX-DISC1	C14orf101	NEUROD1	SET	C6orf164	MAP3K9
MGST1	XPR1	LOC220729	CCDC83	CSRNP1	LEPRE1	GRIN2B	EHBP1	CCDC54	MAP4K4	C14orf166

PNRC1	LOC650623	DBI	YOD1	MGC12916	LOC595101	C5orf47	PPP4R1L	C10orf90	PCA3	EEF1G
TAF12	C14orf135	RNPEP	PLCD4	MRPS25	OSMR	STBD1	TCEB3C	MT1IP	RAD18	EIF2B3
DPP3	ERC2	CCDC149	TNFSF18	PPP1R2P1	TRDMT1	HBG2	FAM153B	IARS	OR1K1	PPPDE2
GAA	38412	GON4L	GLB1L3	SNORD116-28	OR5B21	WDR41	FHOD3	CKAP5	C4orf19	SEC22B
GBA	LGALS8	OXNAD1	MRGPRE	PCBD1	ADAM21	SLC2A3	RDH8	KIF4B	SPTLC3	ST6GALNAC3
LOC285780	FGFR2	NEIL3	DENND5A	FLJ36031	MANBA	ATRNL1	CEP152	NDUFAF4	TUBB	GABRB2
COL27A1	PTPLA	RBBP8	MYOF	FOXI1	DEPDC5	SLC39A7	COX15	LMAN1L	ALDH18A1	ME1
PVR	FTHL3	FAM190B	HBB	PLEKHG3	METTL13	HTR1A	BMS1P4	RASAL2	GNG5	IER3
RNF19B	C2orf61	SDCCAG8	NMNAT3	PDCD11	TEX10	KLHL10	KIAA0100	GDAP1	TSC1	RNPC3
CTH	SULT4A1	CT62	FBXO48	IL13RA2	EXOG	TAAR5	CREG2	C9orf25	DNASE1	LOC100302650
ARID3C	BHLHE40	ZNF382	C14orf119	VWA5A	ZNF808	FEM1C	C3orf48	MCART2	SMAD2	RTN3
TSPYL6	FSTL1	LRRC36	PSEN1	TSGA13	MAP3K13	OSBPL11	LOC91316	TRIB1	DNAH14	SLC22A15
FAM7A3	SPAG9	NXNL1	SLC38A8	PRDX1	WDR19	LOC729799	CDH19	SIAH1	EXTL1	LOC126536
LAMA4	TBC1D10A	ATP13A3	MCL1	GDAP2	SPATA8	C22orf15	DYNC1LI1	LOC729082	INHBC	LGALS12
AMTN	LRRIQ3	PIP5K1A	ETFDH	CPS1	CXCL1	EID2B	LONRF1	RAD23B	NKX2-2	SLC25A20
CACNG2	OBP2A	ISX	HDX	FBXO18	HBA2	C17orf58	TBC1D1	CDO1	RPP14	C20orf123
ZNF667	HIST1H4L	MRPL45	PTPN20A	FGF23	ZNF563	WLS	NEXN	SEC23B	SLC38A7	RUFY3
MED20	C10orf46	C4orf29	AVEN	TTL	AGBL1	ATP6V1G3	C2orf51	EVX2	FKSG83	GPR101
LOC144742	LRIT1	NXF4	WFDC6	MCF 2.00	EIF2AK3	C14orf43	FCHSD2	NCRNA00115	AHCYL1	ZNF248
TIMM17A	PCDHA3	TOX4	SNW1	C13orf36	FAM160A2	TRIM38	WFDC8	HEXB	BACE2	GDPD1
CALR3	C10orf12	PPA2	NTAN1	CFL2	BET1	AIFM3	CTTNBP2NL	TRAF6	RNF212	DUSP14
LOC100124692	ZSWIM6	RSRC2	NIN	FTH1	ZNF704	NUDT5	DDIT4	CCL2	TIPARP	SPINLW1
PAR5	JPH1	EIF2S2	HAVCR2	OR13F1	CPD	ZFAND5	EAF1	EPRS	EIF4E	RSPO4
KIAA1107	APPBP2	PTEN	FOXG1	GALNTL1	SMNDC1	FLJ44054	FBXW7	OPE38777	CWC27	PRDM7
C14orf118	ABHD4	GPHN	MRPS16	UBE4B	TXNRD3IT1	LSM 3.00	FBXO25	NRBF2	AKIRIN2	RAB9B
EIF3L	C3orf35	FER1L5	UBXN8	LOC100287227	MALT1	CSNK2A1	RPL36A	CGB8	VAMP3	STAC
CCNL1	LDLRAD3	PNPLA5	UTP3	STL	FAM20A	UBA3	OTUD6A	ZNF717	PNRC2	MAPT
YME1L1	C16orf87	SEC11A	KRTAP1-3	GAB2	DCP1A	ADAM22	ALS2CR8	HACL1	TBC1D23	HYMAI

LOC284100	ABCC5	DNAH1	AAK1	AHSP	HNRNPA2B1	UCK2	AMAC1	PSG6	ZNF295	ULK4
HOXA9	ALKBH1	PDE4DIP	CYP51A1	HNRNPH3	CTSA	ZNF625	MUC13	NAA35	IFNB1	DEFB109P1B
ZNF577	DUSP18	VDAC2	CIRH1A	FLG	NDUFB5	ABCB7	DUSP5P	ADH4	PFN2	GTPBP2
SLC25A40	INVS	RRAGD	DEFB1	TBC1D7	DDIT3	SEC22C	UHRF1BP1	TAS2R19	ITSN1	NR2C2
LRRC59	RALYL	LETM2	EIF4A2	NHEDC1	LOC440896	PCDHA1	RNF151	SLED1	TMEM100	ZFYVE20
RASA1	BRD7	LTA4H	LIX1L	RASSF6	C16orf68	PABPC4	NPEPPS	ZIC3	NPFFR2	C14orf28
KIAA0831	C11orf9	JAKMIP3	RHOB	KLHL5	PPP2R5E	PTCD2	GPN1	C6orf120	GPR137B	TCTE1
SLC2A1	OR13C2	FEV	KCNJ8	CLIC6	MALAT1	YEATS2	NEUROG3	GTF2H3	GHITM	RAB9A
MLH1	HINT3	C18orf20	GBE1	PRDM13	FAM198A	DGKI	WDR6	CHRFAM7A	S100B	DLX6
C17orf42	ADSL	ABCF1	HEATR5A	PRG1	GCNT3	TSC22D2	ABCG4	CLASP2	ZMYM4	GPI
NHLRC2	LOC144486	SPCS1	OSGIN2	HDHD1A	FERMT2	SYT14	CYP26B1	AATF	DNAJB4	FMNL2
F2RL2	KIR3DL3	FKBP7	MTERFD2	FAM188A	C7orf4	EBNA1BP2	STRN3	MRAS	ZNF713	ENAH
KIAA1529	ZNF555	KLHL24	NPAS1	C10orf10	ATXN7	TRMT11	PRMT5	ASB18	NCOA7	IFIT1B
SLC2A14	IL4	MTR	RWDD3	CXCL3	ENPP1	RPL7L1	PLIN5	KPNB1	HSN2	PHTF1
LITAF	KCTD6	PCDHA10	PLAGL1	CDC123	ATG2B	TMX1	FAM96A	INADL	COL22A1	NKIRAS1
HN1	DYM	TRMT5	LRIG2	MCHR1	DLEU2L	NR4A3	LOH12CR1	KRT222	ENO1	PIK3C3
LRP8	PI4KAP2	CPNE2	MAFG	HEXA	ISCA2	SLC25A24	TMX4	SATB1	FLJ35776	LOC100130522
DIRC2	GRPEL1	DUS2L	TMCO7	ZNF25	CLK3	PHF21A	KIAA1279	MLLT10	SEC31B	STAC2
CYP1A2	MYLK	NARS	DNAH12	SAMD8	STXBP4	CRX	NME1	TRIM45	CLCC1	C20orf141
C7orf46	HIF1AN	MRRF	SERPINE1	SOAT1	CEACAM22P	BRAF	UQCRHL	CCT3	C2orf56	NDUFV1
CHCHD4	BCL10	HRASLS	CLIP1	SEC13	IPO7	GCLC	PTGR2	HABP2	MAN2B2	C9orf72
TRIM44	PI4K2B	IMPG2	SLC25A37	LEPROT	CYR61	PCDHGA3	DUSP8	TMC5	TAB2	EIF4B
ZNF271	KCNIP1	ASXL3	GLT25D1	FABP3	GK5	COPB2	FCF1	CASP6	MMP7	TEC
FLJ40292	TAS2R5	HSF2	LOC347376	EIF4A1	DGKH	RRP8	CATSPER4	KRTAP10-1	OR13C4	OR5AU1
CDC26	ATXN10	CSMD2	PSMA1	ARHGEF38	KIAA1797	CHEK2	SERPINF1	PGGT1B	HOXB9	APEX1
MYSM1	DPH5	RNF128	LOC728190	FAM151B	MRPL47	RNF141	RRN3	CYTSB	MDH1B	RTN4IP1
EXT1	MANF	COPB1	ARHGAP29	WBP2NL	MYO19	ZFYVE26	C14orf34	SV2B	MIR17HG	CSDA
LAMA1	ANP32B	PYGL	CPOX	KLF7	UQCRH	UCHL1	NPM3	HELB	SNX32	DNAJB5

PCCB	KLKP1	IGLON5	EIF1	PTGFRN	MFN1	ARRDC3	ADRB2	LOC728323	STIP1	NAA38
TLR5	MUC21	SUPV3L1	TTC14	CACNG1	SERHL2	CSGALNACT2	NEFM	RIPK2	MRPL33	NCRNA00113
FAM45B	RET	DOCK7	SDHB	ZNF433	KIAA0319L	C1QBP	SCARB2	ADAM17	TRUB1	FKBP3
FIG4	RPL23AP53	CRYBG3	IDO2	CHMP2B	GADD45B	RPL36AL	PIK3CA	RPS25	LOC145783	KLHDC4
GBF1	INSM1	LOC220930	GLB1	NCBP1	TPM3	PSMD2	FUT5	C18orf55	C3orf71	BLVRB
ANKRD34B	FUBP1	ZADH2	ALG10	NHLRC3	RCN1	DSE	TIMM9	SOHLH2	TUBB8	SRI
CDX2	NPPB	RPS4X	SLCO5A1	CDK5R2	LRRC40	TFDP2	CDC27	SIP1	RALGAPA2	TMEM2
FAM45A	PNMA3	FAM178A	SLC19A2	VAPA	UGCG	TAF15	THRAP3	KIR2DS4	FAM21A	KCNT2
NTNG2	CACNB4	KIAA1958	HESX1	GMFB	UTS2	CLTC	EIF2C4	DNAJC13	CUEDC2	PSMA3
LOC116437	XIRP1	MTOR	RNF2	CECR5	DCTPP1	LRP12	GARS	ERO1L	ZNF442	AFAP1
ME2	LPHN2	CCDC21	LRG1	VIPAR	PCDH11Y	ACAP2	ZNF135	UBQLN3	TPD52L1	SLC25A5
YARS	MMS19	RBM6	PPIAL4G	CCDC102B	ALG6	HSPA9	EIF2S3	WBP5	KCNA10	RNASE1
RGS2	IFITM2	ZCCHC11	CYP 20.00	ACBD3	MRPS22	RNF19A	ZNF354C	HCN4	RFT1	MT2A
NHLH2	NBPF10	DPAGT1	HYAL4	SNORA72	WSB1	SERPINB7	CDK11A	TCF25	LRRTM2	ECD
BBS7	LOC100128788	REN	DHRS9	EIF4A3	SH3RF1	CLIP4	CBWD3	HNRNPR	GRN	RUNDC2C
MTPAP	MAP3K7	DNAJC10	SNX6	TRNT1	JMY	SNORA56	GNPAT	QARS	KRT74	EXOC5
ZEB1	SNX10	CCDC79	EDEM1	CXorf23	CSNK2A1P	SYT11	CCIN	CCNH	CHUK	NRCAM
OR13D1	GZF1	VSTM1	LOC441046	TRIP13	SULT1E1	GATSL1	POLR3A	TAC1	GNAI3	HAUS6
TMEM20	GMPS	C5orf42	MRPL3	SEMA3B	SPDYE7P	C2CD4A	IPO9	SPATA7	BTBD3	RHEB
C3orf59	SOS 2	RNF150	MSL1							

Coexpressed genes with Myc in KIRP (Stage 3)

UNC50	PHOSPHO2	GTPBP2	POLR1B	ZC3H8	USP49	PRMT3	GTF3C3	ZNF318	TUBGCP5	ZC3H15
FASTKD2	CDC5L	EEF1B2	ANO4	MCM3APAS	POLH	TJAP1	HSP90AB1	GAD1	C11orf58	MAD2L1BP
HSP90AB2P	SLC29A1	CD2AP	SUV39H2	TFAM	KIN	ELOVL5	E2F5	CDC123	ORC4L	NIPA2
XPO5	NFYA	TTTY4C	MTX2	ATAD1	FAM163B	C10orf119	RPL7L1	ENPP4	OTUD1	PCGF6
GNMT	NIF3L1	AFF3	AARS2	NCKAP1	C1orf187	PRPS1	DDX50	FAM45A	RPE	TEX10
TMEM185B	RPL7	TTL	LRPPRC	SLC35B2	TOMM20	C2orf58	MED20	LOC728190	KIF6	TUBA4B

ERCC3	PDCD11	C10orf137	SLC25A16	SLC35F5	VLDLR	YIPF3	SLC25A27	MINPP1	WDR12	AGFG1
C5orf30	LONRF2	C16orf81	NCL	PPP2R5D	PATL1	ORC2L	VPS26A	GAS2L3	XPOT	CEBPZ
MUT	SLC22A15	SMNDC1	LOC730101	IPO7	VEGFA	TAF3	ENPP5	KLHDC3	HKDC1	WDR43
SGTB	UBR2	THY39142	TIAL1	PRICKLE4	CYorf15B	HCN2	BPY2	TAS1R3	POLR1C	HSPA14
NUAK2	SNORD1C	BRS3	SNAPC1	VEPH1	TARS	KLHL23	MIPOL1	PROX2	FAM66C	KCNN2
MOAP1	RPP38	GCC2	DIRAS1	GABRB3	PRPF40A	DNAJC10	OR2L1P	ABCC10	C10orf88	EEF2
WNT8B	ATP1B4	EIF2S3	RPS4Y1	NMT2	GOLGA7	TCHH	GLRX3	MARS2	PRPF18	TMEM14A
C2orf89	SOX3	CASP5	HAT1	NFE2L2	DDX18	GPATCH2	MAP4K3	COL21A1	CENPQ	PYCR1
NOLC1	TTC21B	NCRNA00188	MMADHC	USP6NL	NOC3L	FNDC8	FRMD4A	C20orf160	FBXO18	NOP58
IWS1	LRRC1	DCAF16	ASAP1	TTLL6	FAM175B	TMEM182	RNF141	TCTE1	PPAT	PHYH
ZFAND1	SEC61A2	FOXP4	FLJ10357	ZNF207	ACCN2	MAP3K15	GLO1	CPS1	USP47	AZIN1
SERPINA7	TCEA1	PAICS	CSGALNACT1	LOC100132354	RCL1	DDX3Y	TMEM63B	TMEM20	C2orf3	INTS7
SPHKAP	OR4F21	SNAR-B2	SNAR-C3	ASNSD1	TRMT5	ARL5A	C6orf153	CHRM3	COL4A4	PNO1
TLK2	SPIRE1	MKX	GMPS	SEPHS1	LPA	AP3M1	NPC1	CKAP5	FAM45B	GDF15
PNPT1	UTP14A	GTF2H1	CUL7	LOC100287718	COL4A3	SLC25A32	ZDBF2	KIAA0240	CWC22	ZFAND3
FAM178A	RGS11	EIF3A	C6orf138	AMMECR1L	MRPS10	GPR162	MCM10	RSRC1	AYF37500	HAUS6
TTC17	CHUK	PLS3	WT1	OLA1	PABPC3	HPS3	CHRNA4	PEX6	IRX2	TRUB1
SNHG4	R3HDM1	RNU6ATAC	EEF1A1	DCAF12L2	SLC25A12	AP3M2	TRAM2	FGFR2	NUP35	IBTK
KIAA1598	WIF1	MEA1	CALCOCO2	CCNB1IP1	ABI2	TRERF1	TRAM1	MTHFD2	GCA	ABCB7
FZD5	GTF2H2C	BEND7	PPP2R2A	ANAPC1	FAM86B2	THAP1	FARSB	C1orf43	UBE2A	EIF5A2
GPN1	B3GAT1	UTP20	PRKAG1	MRPL30	C12orf24	DLX1	TAF8	UNC13C	PTPLAD1	CALCRL
ICA1L	NUP98	RNASE12	SCARNA22	TTTY7	TDRD6	ABCE1	FAM168B	CDK7	RBM17	WBP11P1
GDI2	FAM69B	ZC3H12C	UBLCP1	GDF6	PPRC1	APOBEC2	UBE3A	TGFBR1	ACTR3	ST6GALNAC3
MAP4K4	NAMPT	GART	ANKRD16	HEATR1	ICAM5	PPP1R3B	EPRS	UBA2	NUDT5	COL27A1
LANCL1	KCNS3	TMEM59L	CACNA1H	UCHL5	LOC729020	TRMT61B	TM2D2	TMEM151B	FAM66A	TSNAX
OR2L3	SLC6A17	C16orf52	CDC27	DDX1	FETUB	ACBD6	ATIC	METAP2	SLC7A13	ICK
PDE3B	TTTY9B	FMNL2	VTI1A	PLEKHA2	DNAJB3	DIMT1L	RNF41	RAN	SYT4	RGPD8
WDR35	MTERFD2	FGF8	PCSK2	ERGIC2	TBC1D15	KPNB1	ACVR1	EEF1A1P9	TIMM17A	SLC39A10

SP3	DOCK1	KLK2	PCGEM1	EBF3	COL9A3	CYP1A1	DARS2	SUMO1P3	WDSUB1	STAC
PCDHGB3	UBE2D1	SPAG16	GDPD1	HNRNPF	MRPL3	FAM126A	ZNF317	MLLT10	SDCCAG8	BRMS1L
ZRANB3	SLC43A1	RAB3GAP1	TRIML2	GCLC	PLCB4	KCTD15	HDLBP	FRMPD4	TMSB4Y	C9orf4
C6orf154	WFDC6	SPIN3	NUDCD1	PPP4R4	TADA1	APIP	CYP7A1	DAP3	ANKRD40	IARS2
GRPEL2	AMOTL2	DHX36	LCE2D	DYNC1I2	PRKY	EXT1	TDRG1	NLGN4Y	IL1F8	TNFRSF21
FTSJD2	GOLGA8E	RHBG	TMPRSS9	EIF1AY	PRDX3	IL20RB	RUFY2	EIF4B	UTF1	RNF8
PPP2R5C	UTP18	ARG2	KRTAP5-2	WDR67	NPY	C1orf27	C15orf41	UPF2	COPB1	GABRA5
LOC84856	RABGGTB	NVL	FLRT1	CYorf15A	ABCA17P	TCFL5	STARD7	LOC202181	INSIG2	KIAA0020
IFITM5	CAPRIN1	ST3GAL5	CIAO1	SENP2	PNMA1	HSPC072	NAP1L1	LPPR1	VPS37A	C5orf25
KRT32	KIAA2022	LHX6	PKNOX1	SIRT1	SMC6	CNOT7	C11orf46	KCNK13	HOXD10	ARMCX5
DEGS1	TSEN15	C6orf130	SMARCAL1	TRIM59	PIP5K1A	ANKAR	TBCC	KLK3	C2orf49	RSL1D1
TSG101	SMC3	OGT	PPP2R5A	HCRTR2	ZSWIM2	ANKRD13B	TFB2M	IL7	SRF	EPC2
FAM117B	MORF4L2	PDHX	ZDHHC13	CES8	EIF3J	KCNQ5	RPS3A	CLIP4	CPSF6	SMPX
TOM1L1	NCRNA00176	CAPSL	FAM134A	MNAT1	LOC646214	ZRANB1	DCLRE1A	LSM14A	BRIP1	C19orf2
SLC39A14	RGPD3	C2orf29	SNTB1	MGC34034	C12orf50	BMS1	IL12A	GGNBP2	UBXN4	LIN7A
SDCBP	ADORA2B	DIAPH3	IFIT3	PUS7	C2orf69	PTPLAD2	SSB	GNG10	MPP3	MELK
TMEM5	C8orf71	C10orf2	MRPS18A	BZW1	MCPH1	LOC286467	MANEAL	API5	LOC100130093	VDAC2
CASP7	C2orf60	RGPD4	CAMSAP1L1	PRKRA	KATNA1	MORF4	SNX16	TCP11L1	DUSP12	SEC23IP
C1D	MAP1D	DNAJC25-GNG10	MIA3	PLA2R1	ECD	UNCX	MAK16	CCNJ	GORASP2	STAM
GPHN	C10orf84	UXS1	EIF2AK4	ELSPBP1	MOBKL3	OR5B12	C14orf33	YOD1	CCDC138	JUB
GPR161	USP28	GTPBP4	ESM1	PIGC	SIM2	CBWD2	IARS	RAB27A	P2RX6	OR5V1
PSG1	CHML	IVNS1ABP	XK	ARID3C	OR6C65	RPS4Y2	TOPBP1	BCCIP	TRIM58	PROX1
RRAGB	PPP1R3C	SOX8	ALG13	CBS	POTED	FAM78B	RAB3GAP2	MCM6	DNAJC24	SUMO1
ACP1	APEX1	GAGE2B	TRIM53	OTUD6B	KHSRP	C5	PPIG	TFEB	XRCC5	C2orf47
KRTAP4-12	ATF2	CYP26A1	NUP133	KIAA0125	PGBD3	ZFY	ANKRD36B	TWIST2	RBM41	ZEB1
UGT1A7	EIF2S1	ITPKA	TERF1	C1orf107	BDNF	KRTAP6-3	KLRC1	EDDM3A	IPO9	RGS5
DCAF17	EIF2S2	TRAM1L1	SLC25A25	MYO1E	TGFBRAP1	ITM2A	NCRNA00120	ATP5C1	BYSL	GLS
TUBGCP4	FASTKD1	ZNF716	SCN8A	C10orf18	AAMP	LDB1	PIK3C2A	FAM21A	IRX1	BUB3

RRP15	RYR2	TXLNG	TBC1D22B	SUPV3L1	PHF16	UBE2V2	AGBL1	GIGYF2	NBPF14	BUB1
PRKAR2B	MORC4	RPS6KC1	CCDC33	RCAN2	CCT4	C20orf199	DSCR8	ACTR6	OSGEPL1	WIT1
FRS3	JAM3	HECTD2	BRD4	USP17	TAF1A	YEATS4	BLOC1S2	GPR27	WWP1	GRAMD1A
TMEM131	MATN2	CSTF2T	WDR33	FNTA	ETF1	LRCH2	CNNM4	TMEM161B	MST4	TAF9B
ADCY3	LRRC4C	ZAK	IMP4	RANBP2	PIM1	TMEM217	STAT1	ARID3A	PLK2	POGK
SNAP47	NMD3	IL6	JPH1	ZNF143	PALB2	CBX4	MCM3	LEUTX	UBAP2L	HSPA12A
PFKM	BAGE2	IRAK1BP1	PLD6	CKM	USP13	TMEM74	EIF4G2	SELV	ODZ2	OR5B2
FAM171A2	FAM101B	GPX8	GSX2	PARP2	KLRC3	EIF2A	C6orf72	C8orf45	CDC73	CARS
LMAN2L	SCRN3	CTSL3	MAGEB18	C12orf4	FBXO9	FAM119B	SHOX2	KRE38412	PPM1D	LOC284232
SHOC2	ALS2CR4	RNF139	NLRP4	MYO19	MC3R	TOR1AIP1	EXOC6	NFYB	METTL10	EYA4
SRP72	C1orf105	PLEKHM3	TRIM37	C6orf155	RRM1	REPS1	GUCA1B	PSG11	RB1CC1	UNC5CL
RPAP1	HEATR2	PRTG	MSH6	SEMA6D	GLT25D1	SIP1	SCML2	PMAIP1	OAT	THOC2
FOLR3	OR5AN1	PCDHAC2	C6orf218	MYLK	SIGMAR1	SENP1	USP16	PDZK1P1	ACCSL	SRFBP1
VSX1	MAP3K7	TCEAL1	XRCC2	FLJ35024	TTC27	STIM1	KCTD18	HOXA11AS	GRIN2A	SEMA6B
PGC	CAMLG	GPATCH4	PIGW	YY1	OPA1	MCART6	ATP6V1C1	TAS2R3	PFKFB3	CKAP2L
PABPC1	DCAF10	HPRT1	DDX26B	NIPA1	DCUN1D5	NPM1	LOC650623	ARMC1	SLC33A1	PPIL5
POLR3D	NONO	TOR1A	KDELC1	SGK223	UAP1	NAT10	TRIP13	TEX19	LYSMD1	AHCTF1
C10orf67	SF3B1	TRAK2	HOXD8	RGPD6	PAIP1	LRAT	INPP4A	IKBKAP	TRAF5	CXorf56
C11orf82	DNAH14	CYR61	C7orf45	BPI	TLK1	DNA2	PRMT5	KCNK12	RGAG1	RCOR3
RQCD1	MID2	R3HDML	ANKRD27	KRT85	SLED1	OR2W3	CCT2	PCM1	DCTN6	GOLT1A
FOXA2	GOLGA8C	VGLL2	ZNF792	DIAPH2	CCDC121	LOC255167	GSTTP2	LIN52	HLCS	KCNJ8
OPTN	C12orf48	SNRNP27	FOXD1	LOC285401	LRFN2	CSGALNACT2	DNAJC27	DDX52	CHAC2	SNORA76
GARS	SLC25A30	OR51E2	ISM2	LOC153910	PGAM5	CHURC1	TGS1	BRSK2	APOC3	C9orf25
PGAP1	CEP78	PISRT1	CDX4	KRTAP10-1	LOC728276	OR4C11	OR52M1	OR5H2	OR7G3	OR8K5
PRM1	SAA3P	SPZ1	TMEM95	ADAMTS8	MARS	NPM3	ZNF280C	FXR1	C8orf86	KRT38
ERCC6	TRIM3	DAGLA	PANK1	RBM7	TXNDC9	SLC4A5	GABRR2	RNF150	ZNF479	MRPS35
GPR20	DNM1L	MRPS9	FLVCR1	POLR3C	DDX42	RGS9BP	FKTN	FAM184A	EFHA2	PLEKHA3
PEX19	ZFP36L1	RAD50	EIF3C	ADAMTS15	DCAF6	LASS1	SNORA62	KRT35	TIMM9	C6orf64

CUL9	NR5A2	DENND5A	DNMT1	USP32	DDX21	FXC1	MDGA1	RNF2	TSN	TMEM38A
WDYHV1	VPS54	SLC23A2	ALS2	IGFL4	HNRPLL	ZNF333	LOC392196	ABTB2	RAD18	C20orf141
PTK7	SPINK6	LOC643955	C8orf75	UMPS	AASDHPPT	TMEM55A	GLT25D2	SLC4A1AP	CXorf51	DEFB109P1
KRTAP20-2	NCRNA00112	OR4A16	RNASE9	MOCS1	SP9	KIAA1737	GALR1	ZNF57	ZNF679	ITIH2
SMN1	MRPL42	TMPRSS15	MYBBP1A	COCH	ESF1	ZCCHC7	GNPNAT1	PAPPA2	SNORA72	SAP130
PRR5L	SLC16A9	ADAM21	NAA35	PPIF	SPOPL	TAS2R31	GPATCH1	C5orf44	RNF115	PHGDH
GPR63	KL	TUBA3E	C1QTNF8	LDB2	C6orf129	SCN1B	MFF	FAM86B1	HOXD11	QSER1
PGM5P2	FANCM	LRRC39	MC5R	RBM12	MBTPS2	COIL	METT5D1	SUCLG1	GABPB1	SSX6
HOXA11	LIN9	MLL4	RNF149	RPAP3	KIF18A	ABCB10	APPBP2	SRD5A1	SRGAP1	KCNJ11
ALDH18A1	C10orf131	CHM	WDR11	RABGAP1	IL1F10	SUPT3H	PYGO1	MGC14436	SNX6	FNIP2
CEBPG	AKT3	IZUMO1	MTHFD1L	TTC9C	TAF9	LOC100128640	TBCE	EDDM3B	C12orf11	NOL11
PTPRS	PIKFYVE	40422	COX11	GADD45B	ZNF181	WBP2NL	SFXN4	OR51B5	SGPP1	NEK7
ZBTB32	C14orf126	C1QL4	PGLYRP2	PEX5	LOC100133545	FGFR1OP2	RPS23	LGR4	PDGFRL	MUC6
RBMX	CLIC5	SLC17A2	STRBP	CAPZA2	RAI14	GAPDHS	LDLRAD3	B3GALNT2	CYP2C18	ABHD15
SLC16A14	LOC145783	SSRP1	KDM5D	BTNL9	CXCL3	TSR1	HSPB3	CYP39A1	C20orf144	ZNF618
DKFZp686A1627	HNRNPA3	GTF2H3	C1orf25	ACVR1C	DGKD	C14orf142	STARD4	SPC24	BEAN	CUL3
HPS5	PREX2	PWP1	ERO1LB	GLYATL2	ANKIB1	PLAC1	MAGEC2	WNT3	RPL26	TAF4B
ARL17B	MTPAP	NDUFS1	LOC407835	NLRP1	NOL10	TTC30B	CNNM2	RDH14	ZNF507	LRP6
ESCO2	PNN	CWF19L1	ROCK2	ZNF98	UBE2G1	YWHAQ	KCNIP4	KAZALD1	PATE4	DDIT3
TBX22	ADIPOR1	CPEB2	ZNF627	ASPM	BCOR	MAGED4B	C3orf52	SLC35E3	TIPRL	TXNDC16
SLC6A15	EPB41L4B	FATE1	RTN4RL2	GPR1	LOC100133957	SMARCE1	TYSND1	CYYR1	MKI67IP	SEC11A
ITIH5	WDFY1	SLFN12	LOC285627	CPNE6	RPS10P7	ATF6	CASP12	FAM19A5	C19orf57	DNAJA3
CEP170L	NTRK2	EHBP1	METTL2A	RAB4A	KCNH3	HTR3A	CDH17	HOXD3	UEVLD	FARSA
CECR7	TMEM147	ZNF343	FAM66E	MRAP2	AKAP2	C17orf71	PRIM1	STX17	TNFSF14	QSOX2
GAGE2C	PVRL3	KIAA0892	CAMP	KLHL29	TYRP1	SRPK1	FEZ2	GUCA1C	CCNI	STRADB
ADRA1B	BMPR1A									

Coexpressed genes with Myc in KIRP (Stage 4)

HSPA4L	AZIN1	PTGES3	COPB1	UBE2H	PSMD11	TCP11L1	STIP1	KIAA0368	EIF4G2	CDV3
IARS	PAQR5	FBXO22	IL28RA	ABTB2	LOC145783	REEP3	PTK2	PANX1	RNF19B	C19orf55
STAM	EIF4A3	ARL 15.00	SLC7A1	YWHAG	YARS	HSPA5	TTC9C	UBQLN1	ANKRD57	RSC1A1
HNRNPR	CHRNA3	VPS37A	PTPLAD1	WWP1	C10orf46	ALOXE3	INSR	TERF1	ACER2	YME1L1
MAPK8	CAT	RAB2A	NEU3	SLC16A4	CCNDBP1	SORD	UGT8	PARD3	RAD23B	EPRS
EEF2K	NCKAP1	SLC31A1	UEVLD	SLC20A1	TGM4	LOC387647	EIF2C3	ERLIN1	MORC4	TGFA
NAA50	TUBGCP4	MOCOS	MYO5A	ZSCAN20	CPD	RASEF	HIST2H2BA	RNASEH1	EPB41L4B	GRLF1
DFFA	IL7	GAS2L3	UBN1	LOC729020	UBFD1	PLCB4	NDRG1	DNMBP	PTP4A2	CTH
TDRD7	UNC5A	RTN3	PTGFR	RAB9A	CAB39	INSM2	TMED5	RTN4RL2	ZDHHC9	PYCR1
ARFGEF1	SPAG1	KRT80	TMEM184C	RPE	GFPT1	NOLC1	ANKRD56	SHOX2	MRPL19	ARF4
CASP7	GDF15	PLS3	ATP13A3	HIST1H2BE	MAP2K1	SLC39A14	UGCG	PSMB2	STXBP1	CALCOCO2
G3BP2	COL4A6	MEGF9	HIST1H2BC	NMT1	EEF1DP3	RRM2B	ITPR3	MGLL	KLHL8	KCTD8
SYAP1	FAM110C	L1CAM	UBE2A	HERC3	LGR4	USP47	SARS	EBF3	SEL1L3	TRPS1
EIF4G3	HYU40057	DDX21	LRRC57	NRBF2	ERO1L	PAFAH1B2	BICD1	KHDRBS3	LITAF	HSP90AA1
HCN2	TEX2	GCLM	NPC1	CKAP5	UGT1A1	DOCK5	EDEM1	C10orf12	RAP1GDS1	NMT2
PLIN3	WLS	UGT1A6	AMFR	SLC10A6	AHCYL1	PHF16	FDFT1	MFSD9	TRAM1	SGPP2
LEPROT	EIF2C2	RBBP7	PREX2	MYO1E	GABARAPL3	CXorf56	ORAI1	SLC2A1	UXS1	ARL13A
EIF1AX	SLC35F2	USP12	IQGAP1	EDA2R	SLC7A11	UHRF1BP1	DOCK7	UBE4B	PFKFB3	RPA4
JMY	CBX4	PRRG4	PRPS1	ZBTB38	DDX3X	FLJ45983	KPNA6	ASCC1	STIM1	STRAP
SDC1	NUP98	RBBP5	CLTC	TRIM44	ARID3C	WWTR1	PCDH1	PPAT	LAPTM4B	BRSK2
ANXA5	SLITRK2	OSBP	PTPLB	MTHFD2	TRIB3	LRP1B	CAMK2N1	ZDHHC18	OR2AE1	GATA3
MOBKL1B	PDXDC1	GXYLT1	RBBP4	APC2	FAM163A	SYTL5	KGFLP1	ZFYVE9	TSPAN5	VPS26A
RNF139	ZNF526	TGFB1	KCNMA1	CAPN2	GPN1	CSDE1	PTENP1	PDE1A	RALB	COX11
UBC	PCSK5	FAS	RBM7	SPTY2D1	PHEX	DYNLL2	RPS6KA5	SNORA23	FIBCD1	PCYOX1
TMEM20	GMCL1	TUBA4A	SPPL2A	GSPT1	KTN1	MAFG	OAT	USP31	ANAPC1	SRPX
PEX2	NT5DC3	PMAIP1	KIAA0090	DDI2	AVPR2	SIPA1L2	DCTPP1	HECW2	IGF2R	KIAA2022
RHBG	VWA5B2	ZNF365	JAK1	VEZT	LRRC58	DPP10	RBBP8	MTDH	VWF	CNBP
KDM6A	GRIN2B	RBM18	SPINT1	IDI1	TM9SF3	TRIP12	FAM199X	ANXA2P2	RSF1	ALDH18A1

VAMP7	PGAM5	PPP2R2C	PTGR1	FAM38B	RTN4	NDUFS1	TMEM38A	SFN	LOC283856	HIST1H1C
RAD21	RRAGC	LMO1	METAP1	RAB30	IDE	RGP1	CCRN4L	ELAVL2	MSN	SLBP
ALCAM	BTBD10	TM2D2	ZNF516	B3GALT1	GATC	PSAP	CPNE3	CAMK2N2	LIMS1	MAGI3
RNF115	SLC25A24	LRRC4C	CUL2	FAM45B	SNX25	C3orf36	EAF1	RCC1	LDLRAD3	RDH10
PDZD4	FOSL2	RNF213	LIMCH1	PSMD10	FAM171A2	C14orf43	TMOD1	TMEM189-UBE2V1	TMEM185B	PPP2R5E
APLP1	PTPN14	ZDHHC13	ARHGAP11B	IGF1R	LGALS3	C9orf40	NOMO2	CSNK2A1P	KIF2A	KLHL13
HOMER1	MBOAT2	FEZ2	FAM171B	GSR	C16orf5	HOXA11	LRRFIP1	NEURL1B	HOXA6	STT3B
FAM102A	CACNA1G	SLC38A1	LDHA	XPO6	CORO2A	TGS1	UPF2	LOC100131193	SURF4	COL21A1
TXLNG	ASCC3	COPB2	AGAP1	PVR	ZNF146	HIST2H2BE	PARP1	MLLT11	POLR3A	C1orf161
LEMD1	FERMT2	C9orf5	SLMAP	MAN2A1	NRP1	PPP2R2A	VAV3	CPS1	ADCY3	PPP4R2
GDE1	ACSL3	PCDH17	DYNC1I2	TRNP1	NXPH1	NOP14	PSTPIP2	TMEM56	TOM1L1	SMARCA5
TMEM159	DUSP5	CNTN3	ALS2	STK39	TMEM55A	TMEM131	TMSB15A	SLC44A1	FIP1L1	DDB1
EIF4G1	CHRNA5	SIX4	AK3L1	ITGB3	SLC35D1	LMAN1	COL22A1	CCDC6	EYA3	FCN2
GRID1	YIPF4	UBR5	BRAF	RNF125	HOXD11	PANX2	XPOT	UST	GRN	POP1
FAM160A1	TGOLN2	SLCO3A1	ARHGEF35	TTC27	RALGAPA2	HBQ1	C10orf99	RNF24	SPTBN1	LRRC3
THRAP3	POLR1A	C3orf52	MGC27382	TGFBRAP1	C10orf119	GCNT3	FTH1	YEATS2	RAB27B	PRKCA
NFIL3	DEFB132	P4HA1	SEMA4B	MLXIP	TEAD1	YAP1	GABARAPL1	LARP1B	MIA3	RRM1
AP3M1	RAB6A	CA5B	OTUD6B	ZFHX3	PTCHD1	YY2	CACHD1	VAMP3	PTDSS1	GPR157
ADAM17	B3GNT8	ATP1B3	AQR	MST4	TAF3	ZNF697	CA12	TMEM132B	ZRANB1	ATF2
CTNNAL1	NIPA2	CPNE9	H2AFZ	SLITRK6	GBE1	G6PD	SLC6A17	RASL11A	KIAA0196	NIPAL1
CNNM4	KDM4DL	CHMP4C	GPR52	MTF1	P2RY1	HOXD10	PLEKHB2	SAMD4B	INSC	FZD6
ADCY1	WNT9A	GRIK5	FAM81A	NAA35	CHP	BMPR1B	LANCL1	ANXA6	NCRNA00235	ARL5B
PTPRZ1	FAM108C1	DENND4A	EIF2AK2	BRI3BP	STAC	HEG1	STAMBP	CDKN2B	KIAA1279	UCHL1
LRRC59	PLEKHA2	ARHGEF17	TMEM45B	ZC3HAV1L	ADM	LZIC	C1orf183	VCL	FTHL3	GOLGA8C
GRIK1	SPARCL1	FUT11	PDHX	LRP12	PHGDH	DDIT4	RNF103	GMPS	EXOC6B	RAB40B
SLC16A5	COL4A4	TANC2	SLC25A41	CHAC2	MAP4K3	IL6R	NHLRC4	MMD	MMP16	GPR56
LPHN2	YPEL4	CDC27	ATP11B	CD82	LASS6	RAB3GAP1	KCNJ11	ITGA3	EFTUD2	RCAN3
VPS24	SERP1	PRKAR1A	SRCRB4D	HOXB6	FAM174B	STAG1	GPRC5A	KCNG1	HN1	GART

SLC5A3	SMC3	FRMPD2	KIF3C	GPC3	ETFA	ATP6V1A	CMTM8	PARM1	TMBIM1	ZFR2
RMND5A	SLC9A2	SMNDC1	NIPA1	ZNF584	RAB10	SCCPDH	REXO2	JPH1	WDR43	CALM3
LRRC42	MTSS1L	SHOC2	ARG2	NOL9	KIAA1244	C9orf30	PRG1	RETSAT	RALBP1	OSGIN2
B4GALNT1	TGFBR1	PFKP	PGRMC2	TBC1D16	SLC30A1	RNPEP	TMOD3	PCDHAC2	SRD5A2	ZDHHC5
DHX9	MORF4L2	ZNF460	INPP5F	RQCD1	PTP4A1	IPO7	MAP7D2	CBR1	DNAJA1	CTNNA1
LOC595101	DEGS1	C10orf28	TCF23	DIAPH1	RICH2	LRIG1	STARD8	TYMS	ZNF609	HSF1
C6orf25	ARIH1	DENND5A	CHST15	EXT2	HPSE	PPM1B	ARRDC3	C4orf19	IPMK	ABCA17P
OR2W3	C9orf129	ISCA1P1	PRR19	ACOT7	GDF7	ABCC3	ZBTB10	SPRED2	USP7	BCL9
CCND1	SLFN5	PPP3R1	CBX2	FBXO45	FAM3C	OTUD1	TNPO1	CDIPT	FOXF2	PVRL3
EIF1B	DUSP13	BCL10	MORF4L1	EPB41	SNURF	MAST4	SH2D4A	EIF2AK3	PPP1R2P1	SLC22A23
ABP1	RAP2B	EPDR1	SNRPA1	DDIT4L	GPR161	SERTAD2	TARDBP	SRP72	NCK1	PPP1R8
ULBP2	SGTB	AFF1	HSPG2	UBE2L6	HIST1H1E	SESTD1	ADAM22	PRRG1	PNMA1	NLN
SHISA4	ANKRD27	C4orf49	FMN1	NIPAL3	ANGPT4	ERRFI1	PRKCE	VWDE	INHBB	LEPROTL1
GLYR1	HSPD1	CCT8L2	ESPL1	ZC3H8	PRR11	MCFD2	KPNA1	RASSF8	KCND3	FUBP1
ATG4A	MOBP	ABCB6	BAIAP3	NBPF14	RHOC	TPBG	KAT2B	EFNA2	HNRNPH3	RANBP2
C4orf34	CDK15	TRIAP1	SC4MOL	NOTCH1	SLC25A32	ADD3	AP3S1	OR2L2	YUJ40422	CANT1
EIF2AK4	VHL	TSPAN3	SCARB2	DHCR7	BTC	CPA6	ACVR1	BAG4	C16orf72	PEAR1
CDCP1	MAN2B2	BRD4	ABCE1	RAN	TRIM58	C4orf37	SOX13	FZD5	LOC653653	PLOD1
NSF	GOLGA8DP	NPTX2	TUFT1	HOXD1	CNKSR2	SAE1	TG	ZNF281	BMP3	MGAT5
EIF3A	DOCK1	SEC23A	PTEN	KIF1A	CD47	GTF3C3	CTNNB1	CCT4	SPATS1	SERINC3
SLC39A6	ZW10	PRNP	SYT16	ITGA6	DENND4C	RGAG4	ITGB5	LOC344967	SOD1	SLC30A6
EFEMP1	ARCN1	NKIRAS1	MAP3K9	SEC24D	CSPG5	MAN1A2	MCF2L2	LOC728323	GORASP2	FAM59A
FGFRL1	BCAR3	UCK2	ITGA1	PTPN3	TPO	GSX2	ODF3	OR52W1	SLC38A8	TMEM95
PPTC7	TSN	C9orf41	NOS1AP	STK32A	EPHA2	COX15	RBM12	PSMD2	AGRN	DYM
GARS	C3orf39	B4GALNT3	RFPL2	GLS	TMF1	NAA15	TBL1XR1	GOLGA8G	KIRREL	NETO2
DR1	CLIP4	CTNND1	MED28	ITGAV	PPM1L	SWAP70	CSGALNACT1	ECT2	EPHA5	ADAM23
SCYL2	TMED3	NAV2	CXorf22	SNORD115-26	TMCO3	LOC441208	SPTA1	LYPD6	SPIRE1	CDKN1A
DTX3L	HIST1H1T	PRC1	CTNNA2	ZNF366	ITGA2	CCT7	NPDC1	MXD1	CUEDC1	LRRC3B

PLA2G4E	CMYA5	CGN	WAC	MKX	HKDC1	ABI1	NPY	ADI1	DCUN1D3	MAFA
POU3F2	SPRED3	ITPR1	HNRNPF	STC1	ACTN1	OLIG1	MEST	PLCXD2	MECOM	UBE2W
PGM3	ATCAY	ACER1	MCOLN2	GRPR	PFN2	DLL3	FOLR4	SNRNP27	WDYHV1	UBR4
KLHL29	FKBP4	HECTD1	MID2	TNIK	DNAJA3	FOLH1	C20orf54	EPHA6	XIST	GBP2
CEPT1	PTPRS	SUSD4	TSC22D2	SLCO2A1	ST3GAL1	NLGN4X	HEY1	HN1L	ATAD1	NTSR2
CPOX	C1orf103	OTOP2	GNL3L	C6orf106	EHD4	PRR5L	AMHR2	SNRNP40	NCRNA00120	LRRC28
CREG1	DNM2	EPAS1	PSMD1	PDZD8	C1orf9	PKM2	FLT1	SMAD3	CA10	ERMP1
GABRA1	ITGB1	RNF4	FECH	ROCK2	NEDD4	ITSN2	C9orf53	GATSL2	SLC41A2	MRPL49
IRS2	ARHGEF12	AEN	UBE2O	GOLGA7B	SLCO4A1	PINK1	DDX60	DCAF12L2	RAB43	PAK2
CXorf1	MAST2	HPS3	PDGFRL	ITGB6	MGAT4B	GTF3C4	NAV1	SV2C	DHX36	MINPP1
RAB1A	C10orf118	KCTD5	PCGF5	SLIT2	TAF4B	JMJD6	SHISA9	LRRC37A4	EFR3A	IL10RB
VTI1A	HSD3B2	RPS6KA6	RPAP3	CDKN2AIP	IGFBPL1	TNKS1BP1	AADAC	FNDC3B	RSRC1	DCUN1D1
EXD1	CTBP2	FBXL16	SPOPL	SGMS2	ZIC2	TMEM63C	RNF182	C11orf58	KCNK12	TBC1D2
MYBL1	DSTN	AVPI1	NCS1	AK5	IGFBP3	RNF11	STYK1	UBAP1	DPY19L1	ELOVL5
SUCNR1	IL13	MGAT5B	VBP1	FLNA	IL1F8	WBSCR17	PABPC1	UGT1A4	ATP1B1	TJP2
JAKMIP3	RAET1K	GALNT13	ZC3H15	METTL11A	WWC3	KIAA1324	PNO1	FAM69C	GTPBP4	FAM63A
PDK4	MRPS6	TMPRSS7	PCDH19	DEPDC1B	ERN 1.00	CERCAM	NME6	C1orf58	FAM114A1	DNAJC3
CD44	ATE1	TRIM36	BAG3	NUP210	EI24	POLR3E	ABL1	DNAJB14	IPO11	ACCN2
FAM108B1	ARMCX5	GLB1	ARHGAP36	MDM2	DOC2B	UGP2	CYP11B1	PPM1K	CIZ1	CCNF
MRPL13	ACCSL	C3orf49	CDH7	CHRNB3	CLVS2	CYP11B2	DYTN	FAM25B	FGF10	FRMPD2L1
GAST	GPR6	GRXCR1	GSTA3	GUCY2GP	HEATR7B2	IL1F6	LOC100130274	LOC144742	LOC255025	LOC283332
LOC285401	MC2R	MRAP	MSGN1	NPVF	OR10Q1	OR1J4	OR1N1	OTOP3	PIWIL3	PPBPL2
PPY2	PRAMEF2	PSG10	SNORA71B	SSTR4	HCN4	UBE2K	PGM2L1	SQLE	ZDHHC20	UCP2
TP53BP1	ZNF500	MAPK6	XK	SCNN1G	MFSD6	PNP	RAPGEF5	CAP1	IWK38412	CDH18
RRP1B	CREM	ODAM	CDYL2	PRSS23	ROR1	WFS1	PARP14	DMRT1	PATL1	HIST3H2BB
GJC1	RASA2	MYO1B	SAMD8	STAR	39508	KAL1	RGMB	ABCA12	P4HA2	PODXL2
WDFY1	TAF13	GUCA1C	KCTD15	ASPH	TPP1	SFMBT2	SLC26A7	HAND2	ZNF263	ACSL4
MBOAT7	BIRC6	LOC729082	USP13	ARL6IP1	YPEL5	YWHAZ	NR5A1	SBNO1	NRG4	KRT32

NGRN	STMN4	CIB1	KIAA1217	FAM120A	ETV3L	DAZAP2	SCNN1B	BBX	IDH1	KIAA1429
C4orf32	SLC35A2	DIAPH2	STAT3	GATSL1	GCC2	CSRP2	TTC7B	UBQLN2	PDE12	PARP15
HOXA11AS	PTAFR	TCTE1	LIN7A	GPSM2	HSN2	MCTP1	QSOX1	CLCN3	ZCCHC2	TMX4
SPNS1	TXNRD1	ACAN	ZNF295	PDPK1	SENP2	PRKX	ZNF827	PPAPDC1B	NLRP10	LOC728190
ZDHHC22	FAM168B	ALDOA	ASAP2	HIST3H3	NID1	IRAK1	ERGIC1	ACTG1	C3orf58	MBNL3
ADCYAP1	FAM107B	CD93	C9orf140	CLDND1	CHRM4	PDE5A	CELSR2	CT62	HYAL3	SLC4A1AP
ARL4D	PLEC	CHMP2B	AGFG1	PIR	TSPAN14	IARS2	ALG11	MYOF	MRPL33	OSMR
TCEA1	EIF3J	CPEB1	HPCAL1	HIF1AN	QPCTL	MED14	ATP2B1	TSPAN15	TEX9	ZNF774
TXNDC11	GABRB3	ANXA4	RNF141	TMEM206	ATF6	TNKS2	IFIT3	ZNF623	BAALC	HOXB8
SEC23IP	GPBAR1	GHRHR	MUC6	GRAMD3	GPR116	SYCP2	MOAP1	PYGO1	C12orf53	CALB1
SIRT1	GNL3	MACF1	KIAA1958	PGD	APP	TFAP2A	CXorf38	CTBS	SLC33A1	LOC641298
TMEM109	FAM168A	TMEM47	SSRP1	RNF10	USO1	ARHGAP21	AADACL4	NPTXR	RBM15	PELO
DERL1	TNFRSF11B	GOLT1A	TAF2	MBP	LOC100129550	ROD1	PTCD2	SULT2A1	ARMC1	SLC48A1
RGPD3	ATAD2	KDR	INA	PCTP	ZNF813	CCDC33	PCDHGA12	AFAP1L2	SPRY1	CHST8
LHFPL1	PLXND1	ECE1	LDOC1	SRGAP1	LHX1	PRIMA1	USP8	HSPA12A	HIST1H2BK	RABGAP1L
ETV3	GMNN	YIPF6	GNB1	SLC29A1	STBD1	CSNK2A1	LOC652276	OPTN	GPD2	TMC7
MTHFD1L	MESDC2	KPNB1	GNAI3	DLK1	ICMT	TMEM66	TMED7-TICAM2	GPR87	MMADHC	HIST1H2BJ
HAPLN1	ABHD15	KLHDC10	ANO6	CACNA1H	ZNHIT6	ACER3	FBXW11	CA7	ACTR2	PITRM1
MPZL3	C1orf163	CRLF1	CHMP1B	LOC284441	ADORA2B	ROBO1	ZNF488	STRN	MAPRE3	ZNF616
CSDA	RXFP1	GCOM1	UBIAD1	MSH6	TUSC3	PGAM4	KPNA7	PPP1R12A	RAB38	VWC2
ZC3HAV1	MED12L	ZMAT3	HDLBP	BEX5	C11orf86	TLE1	PSME4	DIRAS2	TMPRSS11A	ZFP64
IMPA1	CCT3	CAMK1G	USP38	CD276	TPRG1L	PCDHA13	COPS4	GNA11	ILDR2	TAF1A
TNFAIP8L1	PEF1	KIAA0319	GAB2	ZNF473	UBXN2A	CKS1B	NBPF10	NR1D1	CEACAM19	LNPEP
BLOC1S2	PTPN13	LOC283267	PSMD8	SESN1	KCNK1	ECE2	HMBOX1	ADCYAP1R1	SHANK2	EPB42
CLIC2	SH3YL1	ANXA2P1	PUS7L	MCART2	TBC1D8B	TLE6	TARS	DIRC2	RNF128	HOXD3
ASPHD2	PRPF40A	HNRPLL	AAK1	PLA2G5	RNF168	GPX8	C3orf38	THY	TRUB1	EBF1
C12orf23	YWHAQ	TMEM8A	DPP3	ALG1	SERINC5	IL1RAP	GLT25D1	SNORD94	GBF1	EIF2S2
HSPH1	GABRE	NADK	TMTC1	DKC1	NKX3-2	CHST2	B4GALT6	WNK3	PPRC1	GNG10

RC3H2	PDIA5	GNPTAB	SSFA2	TMEM48	SFTA3	ZFX	SIK1	QSOX2	SLC5A7	MMP13
GRPEL1	GCLC	TMEM68	RAB18	NT5C2	MAD2L1	FASN	HUWE1	PEG10	GPKOW	PPIG
ROCK1	GPNMB	TFE3	KIAA0100	C1orf226	GDI2	NOTCH2	CXorf59	LRP3	WDR1	PPP1CB
IWS1	C19orf57	FOXO4	PI4K2B	B4GALT3	C2orf18	TMEM63B	GHITM	ISG20L2	VEGFA	HIST3H2A
LAMB3	GMEB1	USP14	RCVRN	SEMA4F	PABPC3	KIAA1524	NPTN	PDIA3P	YTHDF3	C3orf26
C17orf28	ZNF689	NOTCH2NL	GOLGA4	FKTN	LIN54	ME2	TTBK2	CUTC	APOOL	ABHD2
FAM98B	C17orf67	SYT14	FAM49B	MMGT1	SON	HYOU1	F11R	TMEM182	NSMAF	CPT1A
TIMM8A	DENND2C	RBCK1	RAX	RAD18	ANO7	PHF3	NFIC	FAM84A	C15orf50	PAPSS2
PRKAR2A	RAB5C	ZNF618	MAP3K2	GNG12	VPS54	SASH1	EBF2	NUDCD1	RIF1	COPG
CLPX	ATP2A2	TMEM59	GNAO1	HSPA8	TTC17	ARPP19	GAPVD1	PLK2	DNAJC5B	SLED1
WDR3	LRRC31	FUCA1	ENOPH1	ITGA5	ENY2	DCLK3	ZHX1	KITLG	LXN	HIST2H2AA3
VPS13C	C19orf38	ALKBH8	HOXB9	SLC30A3	SEC16A	RTF1	LLPH	HSPA1B	PIK3CA	CHM
YY1	PGBD3	MTFR1	DYNLT3	ATP2C1	PGBD5	RHPN2	DNAJB1	ANXA2	IDS	SLC29A2
THRB	MAPK3	NCOA2	HIST2H3D	ATG16L1	PSG5	RB1CC1	GLRX3	ADAR	PKIA	TMED2
BUB1	PSMD14	DOCK6	FICD	WDR44	SNAI2	KIAA0513	ABCC5	ARMCX3	NUAK2	GUCY1A2
ZNF430	ATG9A	RNF111	C8orf76	ZNF705A	CELF1	TFDP2	PTPRM	MKNK2	IPP	PXDNL
DMRTA1	KIAA1211	HIST1H2AG	PDE10A	GFI1B	FAM69A	CHMP5	FNIP2	ELK3	CMIP	LGMN
NBEA	LONRF2	SEC62	HIST2H4A	MAB21L2	RPRML	DNAJC6	ABL2	BRWD3	MFHAS1	TMLHE
LIPG	DIRAS1	C1orf107	RRM2	C3orf64	KDM3A	CDH5	RPAP1	LARP4B	PCNP	C2CD4C
BZW1	MAP6D1	BPNT1	KCNC4	PEX19	PSMA7	FKBP14	A2M	SP100	SP2	USP6NL
MAMSTR	LDB1	REEP2	CLSPN	CTR9	GUCY2F	OR1Q1	RXFP2	SGMS1	SH3RF1	SRPX2
PTPLAD2	UBE2D3	CMTM4	IMPAD1	PRAMEF1	INTS7	WDR45L	LYN	EGLN3	ABCC1	SNX12
CDS2	ATP6V1C1	NOMO1	GPR172A	AKIRIN1	SLC35F5	TPD52	IQCB1	PITPNM1	SLC9A7	STC2
NPAS1	PLVAP	CYTSB	NOV	LOC151162	PPP3CC	ZFP106	CTDSPL2	C15orf41	KDELC2	ZC3H7A
MCM4	ZNFX1	SLC39A7	SRPK1	GAD1	ANO2	FZD7	GPR107	CCPG1	KRT16	TMEM164
UNC13A	GRINA	HNRNPH2	HBG1	FAM83B	ZCRB1	LHX6	DEDD2	ATP1B2	C19orf2	RCN1
C15orf58	BOD1L	HELB	ACTR3	REPS2	CEP68	NPEPPS	TMEM110	SLC35E1	TBC1D20	PKNOX1
FAM175B	KCTD9	EPR1	PCMT1	EIF4E	MANSC1	C17orf71	GCET2	ASAP1	MCM2	XKR4

NOTCH4	ELF4	MAP1LC3B2	DDIT3	TRIM5	EVI5	C21orf67	WNK1	SIAH2	RFK	PHLDA1
ATF3	POLR3K	ADAMTS5	CPA4	DARS2	ZIC5	CXorf36	ARMC7	AFF2	CALCRL	FAM57A
PSMA1	CRIPT	CDC5L	ARFGEF2	TM9SF4	LOC402377	GRIA1	GALNTL2	UBR1	LRPAP1	JKAMP
CSDAP1	FAM71A	GLUD2	PRKDC	SNTB1	F2RL1	GTF2E1	SCN3B	C4orf12	ERCC6	ASAH1
KDM5C	SEC22B	ATP11A	FRK	CLPTM1	KCMF1	CAPRIN1	GPBP1	POLE4	TMEM87B	SLC20A2
RAET1G	AMBN	SNORD116-4	HIPK3	SKIL	PWP2	PPP1R14C	REST	CHMP7	ARPC2	CYP2U1
HSPA14	KLF14	BRCC3	YUJ38777	CCDC75	OR2B6	KBTBD4	PPP1R2P3	AOC2	VANGL1	CDC42BPA
HDGFRP3	TRIM37	LRPPRC	C12orf36	LAMA2	LOC285830	SPC24	FXR1	LRRTM2	CLRN1	PHOX2A
NHSL1	LSM 10.00	SLFN12	GABPB2	STK40	HNRNPK	SH3GLB1	STXBP5	KAZALD1	SNX6	NFKBIL2
API5	C10orf47	MRE11A	HSPA4	HTA	TMEM79	ANKRD46	CCDC7	FKBP1AP1	ZFP91	ETF1
PHTF1	RELA	TCTN3	ING2	TMEM39A	KIF4A	RND3	INVS	SLC36A1	SLC35A3	MDGA2
DCAF8L2	UHMK1	NEFL	DUSP7	LILRB5	SLC18A2	ATP8B1	DCAF12	STAU1	OIP5	PRDX6
CLCC1	MAPRE1	POU3F3	TOP 1.00	PDIA6	SSTR5	C1orf55	COL8A1	HAUS2	CDYL	SERTAD1
C1orf43	COL18A1	ATXN8OS	OR10A3	OR1G1	TRIM40	AK2	C14orf147	MSI2	STX12	PPAP2B
GPR180	NCALD	ARNTL	GCNT1	ADAM15	ZFP36L1	CD209	RNF39	C12orf11	CBFA2T3	SAP130
UBR3	CNTNAP2	CNIH	NUDT4	PPME1	RDX	CD46	ZNF664	ZFAND6	WDR26	KIAA1199
MAD2L2	LOC84989	C1orf150	TAS1R2	BHLHE40	VCPIP1	CXorf64	RLBP1	SNORA2A	HMG20A	C12orf66
DUSP4	CORO1C	DSCR3	BCL2L11	KIAA0494	SEMA6B	PPP2R2B	SETD7	BLVRB	TULP1	HOXC13
FBXO2	HELLS	KIAA1522	FBXO8	UTP18	CSTF2T	NEO1	PRDM5	LIN52	STAMBPL1	LPCAT2
EXT1	TRIM59	DMPK	OLFM3	BDNF	ADAM11	GDPD1	ZNF259	HIST1H2AC	PBK	TTC39B
CD177	BAZ2A	EXOSC2	C8orf39	EPT1	RNF152	ARHGAP17	ENTPD4	NOP10	DIAPH3	SULT1A1
RAB27A	HADHB	AKAP2	C4orf41	HMCN1	FAM101B	PEA15	CXorf57	REG4	HIAT1	TIMP2
HSPA13	UBE2R2	C12orf49	DLL4	NUSAP1	USP39	RTN4RL1	CBX1	TLX1	TBL1X	SMARCD2
ZNF37A	CD2AP	MYH16	TMEM108	PRPS2	CASC5	ANKRD36BP1	COL5A3	GNAT2	ZNF542	NR5A2
ARHGAP11A	SH3BP2	AVL9	RBMS1	KCTD21	KCNIP1	DNAJC10	ATRN	CCR10	NCL	LONP2
MNAT1	HIST2H2AB	UBXN4	DHFR	KDELR1	ANKRD40	KBTBD5	PCSK6	TNFSF9	TGM5	AHSA1
NRCAM	GPRC5C	LMAN2L	C10orf18	GANAB	AGPAT9	GOLT1B	PTPN9	LRP11	MPP1	GNPNAT1
MGAT4A	ESM1	MGC14436	EDA	ITGA8	CAPN6	STS	GSDMA	PIGN	PRKCI	AP3D1

PRICKLE3	GOLIM4	CD34	MAP3K5	RBM8A	STX5	KCNK3	PPP3CA	GADD45A	LIMA1	GSTM3
NUP155	OLIG2	ACBD5	CCDC80	MAP4K4	NOC3L	EPHX1	RHOD	C11orf17	PNRC2	THSD4
C3orf37	PIP4K2A	KLHL20	WDR67	SKA2	RHBDD1	OR13J1	TEX10	MTERFD2	TSEN34	RFX3
SIGLEC6	RASSF9	PLEKHM3	IER5	TTC35	HSP90AB2P	CNGA1	MORF4	KIAA0754	DGAT2	NR6A1
RNASE7	CYFIP1	PIK3C2B	GSN	LOC100144604	PDE1C	CSGALNACT2	PQLC3	RSPO3	FGFR1OP2	DTX4
FAM57B	SNORA74A	PRKAG2	ATRNL1	FBLN2	DBI	SPESP1	RGPD4	IFIH1	E2F5	LIPA
LEO1	DACH1	LRP1	PRO0611	TEX15	CEP97	SLC4A5	SNX2	AMBRA1	MRPS14	TAF5L
CCDC43	SLC12A2	TPSD1	PSG1	C15orf44	PREPL	TSPAN17	RHAG	WBP2	DUSP14	RAB7L1
TAB3	CSNK1A1	PSG7	HIST1H3H	CDR2L	OVCH2	WIPF2	STARD13	ATP11C	GALNT12	GPR110
HPS5	TWSG1	SGK196	FAM45A	ADAM7	RAB32	FAM83G	KIAA1715	FSD1	NUDT21	C19orf40
EMR2	RNASE10	DNM1L	EARS2	MAL2	PAPOLG	C9orf150	TIE1	SMN1	ADAM9	HHIPL2
CMTM1	TMEM2	AQP8	XRRA1	HSP90AB1	GDPD5	SLCO5A1	RGS7BP	SSR3	TOR1A	SCUBE3
C18orf26	MRFAP1	PAPPA2	TOR1AIP2	RTN2	KIAA1161	STEAP3	DSEL	ATG2A	INSIG2	RFX7
TFAM	SPTLC2	AACS	HOXB2	B3GNT5	ETHE1	CCDC93	PLXNA3	COX7A2L	HOXD8	FAM25A
TMEM65	LOC146336	BACH1	LASP1	FAM78B	CHAF1B	FCN3	ATP9A	FRG2B	ZNF576	SLURP1
GGCX	GLT25D2	EIF2S3	PID1	LAMC1	CCNY	MLL	LAMC2	SDCBP	HMGCR	SRP68
PGC	UGDH	DKFZP434K028	ZNF845	RCN2	PLXNA1	ALAS1	FYTTD1	UHRF1BP1L	KRT35	SEC14L1
PLEKHM1	MPP6	TKT	PJA1	NOVA2	URB2	SPOCK1	GABRD	FAM164A	SLC6A6	LAMP1
TAB2	INADL	ARHGEF5	ANTXR1	C1orf21	ADAM10	ACP1	NR0B2	MYO5C	TMEM62	C9orf91
SHROOM2	SRMS	EPHB1	NF1	PHF8	ATP4A	LEPR	PARP8	GPR137B	GRK5	C9orf102
ABCG2	SMC2	WASF3	ZBTB2	IDH2	VCP	TMEM132A	NHLRC2	FOXO1	TUBGCP5	EGLN1
ZBTB7C	ACSL1	HYAL4	FAM72A	HMGA1	TEX12	C8orf55	MLLT3	SEC31A	SLC23A2	GRIK3
GOLPH3L	AMMECR1L	LARP6	CDC37L1	S1PR1	IMMT	FCF1	TANC1	HNRNPC	ZRANB3	ADAMTS4
NCAPD2	GNB4	COQ2	FLJ43860	IFNAR1	BMI1	PLCD4	RASGRF2	SPNS2	MOV10	HBD
RIPK2	IRGC	TNKS	RAI1	TTC21B	USP32	CYP51A1	B4GALNT4	CSNK1G1	LRP6	KIF11
C2orf47	MAN1A1	ADK	DCUN1D5	TCEB3	DOLK	KCNS2	CEACAM1	SPINT2	DAAM2	RNF20
TESK2	MYCBP2	MAP7D1	CBR3	P2RX6	CXXC5	CAMP	TMEM127	LOC399815	SLC9A11	PSAT1
PAICS	YIPF5	KLRC1	MYH15	MAP2	ANGPT2	CXCR7	ISCA1	PTGER2	ZNF687	CPSF4L

BSDC1	UGT1A5	CD274	FUNDC2P2	DHRS7	UBXN7	BTBD3	DCAF6	SERBP1	RNF7	SGPP1
WBP11	USP36	ATIC	CARS	DNMT1	DDX52	SNX3	VDAC2	NCRNA00087	HADHA	PLBD2
C9orf21	FHL5	NRAS	PDE3B	MGST1	H3F3C	TTC9	SEC63	CNOT1	CDC73	BZW2
FOXR1	ERGIC2	MRPL42	TTYH2	RCE1	CTDSPL	HEATR5B	WASF2	GPR179	TMEM72	CACNA2D1
CES7	GLO1	SPAST	SDC2	MYST2	SFT2D2	PRMT3	RNF144B	NEUROG3	STX6	LYSMD2
FUT5	ATP6V0E1	GRM1	RACGAP1	KCTD20	FUT10	SIGLEC1	CHSY3	LCE1E	C8orf45	HNRNPU
CD55	BST2	UBE2Z	MANF	KLHL15	CDC42	CCDC136	RNGTT	RNF187	UTP15	RAD50
CHCHD6	FAM46A	GPR160	EIF3C	DIO3	UBE2V2	SERPINB8	MED30	SEMA4C	OXSR1	TP53I11
LTBP3	F2R	STT3A	GRIN2C	MINA	LOC100126784	TP53BP2	MSH2	PPT1	POLR2B	FURIN
UBE2I	UGGT1	TTF2	GNG4	TNFAIP1	FAM53C	CTSD	SESN2	MED21	C1QL1	CACYBP
PTGER3	ERCC4	DYSF	RAD54L2	SNORA34	C12orf52	TSPAN9	PRDX1	POC1A	NBLA00301	MPZL2
STK32C	EGFR	CNNM2	PRR15	FETUB	BUB1B	SEC14L4	C1D	FBXO22OS	TOB1	AXIN1
SMAD5OS	ENDOD1	SIGLECP3	ACBD3	SRPRB	PCNX	RAB14	SLC10A3	ALAS2	POLQ	KIAA0922
DEK	ELTD1	PIAS1	UAP1	GPATCH8	MUCL1	GOLGA7	PLD5	SLC28A3	KCNH1	STK3
RRAGA	GTF2H3	SOD3	TRIM63	PROX1	LRRC52	PIGB	SIRPB1	ANXA9	ST6GALNAC4	SMYD3
FBXW2	FAM190B	BAT2L1	JAM3	KLF7	RAB9B	USP28	BMP6	NDE1	MAN1B1	FBXO42
SPTLC1	FAM155A	ZNF174	GSG2	TMCO7	WDR47	KLHL5	RPS27L	ZNF778	UBA1	GABPB1
KCNC1	KIF5C	ANKRD31	RGS9BP	APBB2	SRP54	FAM63B	HS2ST1	DLG5	ALDH9A1	PCNT
SLC45A4	COL15A1	C20orf177	LGI4	INMT	DCAF16	CCDC21	PAPOLA	IFITM1	SLC17A3	DHRS3
STAU2	KLC1	ESCO2	OPA1	KCNN3	DPP8	SEL1L	CPB2	ARTN	CEBPG	LRCH3
SESN3	FBXO48	MFSD11	GTF2H1	GABRQ	STRBP	YWHAB	MCM10	SEC24A	GPR88	TRAF6
SPRY4	XRN1	FOXA1	AARS	RG9MTD2	TGFBR2	GLCE	C9orf3	TMEM192	SNX7	SLC16A3
PLDN	ACAP2	MEIS1	ZNF828	LYVE1	KCTD3	KIAA1598	FASTKD2	INCENP	ZNF670	SLC7A14
ARID5B	LAMA5	MTOR	GPRIN1	LMO4	FAM104A	KCNC3	DHDDS	RRAS2	GDF6	KIAA0319L
PLXDC2	CSPG4	ABO	PEX13	KIF20B	DRAM2	PHKB	TMTC2	CHST3	SNORD116-20	CNST
RERE	PTPRU	OAZ2	PCDH12	CDK1	LOC653566	BAHD1	LMNA	TMCC1	CDH13	SGOL1
RSL1D1	LRRC10	PPPDE1	VN1R5	LRBA	TOR1B	METTL14	ZNF652	LYST	GRHPR	E2F7
NCBP1	HOXD4	NOMO3	UNC45A	BMPR2	DERA	BLNK	CREG2	RAB11FIP5	KCNH4	BRD2

ATP10D	LASS1	DNAJB4	SCRIB	AFF3	TRIM2	USP48	MC4R	SEMA4G	SLK	ATP6V1H
PDXK	NDFIP1	TAOK1	OPALIN	FBXO28	RAB5A	LONP1	SMS	C1orf190	AKT3	BCKDK
ASPM	LOC100133612	FANCD2	MOSPD2	EIF2S1	GSK3B	C5orf30	ASAH2B	CYYR1	GKN1	ANKRD17
TFDP1	PTPN4	HIST1H2BN	NUDT11	PLK1S1	PDCL	MAP1B	LYPLA1	INSIG1	SCRG1	DGKE
MYRIP	KRT15	TNFRSF10B	AAMP	HIATL1	SATL1	COL14A1	USP24	SLC26A10	ENDOU	COL4A5
METTL13	IVNS1ABP	FAM157A	SNAPC3	PLAC2	LEPREL2	RFFL	CAPN14	LUZP6	IQCF5	CENPI
GTF3C1	PDGFC	ARAP2	C9orf25	ETV5	MAPK14	GCC1	SMC1A	SDF4	RSPRY1	HK2
SH3KBP1	SBSN	ZNF667	NODAL	CNN3	C7orf65	MTUS1	IFI16	TRIML2	PPAP2A	APOLD1
PARP9	LOC392196	LINGO2	LAMB2	KPNA4	HEATR1	INPP1	CCNI	THBS1	LOC221710	AP4E1
LCORL	BANK1	HNRNPA3P1	ZNF367	CTDSP2	C20orf3	UTP23	GRID2	TAAR9	RRN3	ESYT1
PPIF	STEAP4	COL6A6	SIRPD	PTPRF	TYNB37500	MUC2	TMX1	SYNJ2	SNRPN	CYB5RL
LRRC1	PRKRIR	FAM155B	NUMBL	LARGE	CDK2	KIAA0146	CLOCK	NAF1	NEK1	LOR
B3GNT2	AZI2	GALNT7	USP37	PSMD3	KRT38	EPN3	EPHB2	C12orf35	VPS33A	ABI2
DAG1	ZNF462	PIK3C3	NANS	RNF26	BCAS2	LOC100170939	RAPGEF6	SMARCA2	LOC148189	LOC400696
RPA2	PIP4K2C	POLR3D	KPNA2	STX17	PTPRG	HOMER2	PKMYT1	TAS2R50	MARS2	BCL6B
POLR2D	IL2RA	PRKD3	NUP62CL	RNF149	SAMD9	TBC1D2B	HOXB5	DCDC1	SAMD4A	HIPK2
LOC344595	BRPF3	ACADSB	ZNF79	ZDHHC2	HS3ST4	PRDM10	HAUS6	ARSK	PHACTR4	MMRN2
RNF133	DNAJC25	IGFL4	SP140L	C10orf128	NPAT	KIF13A	LAMP2	RCL1	UBE2D1	MPP3
GPATCH3	UQCRC2	GRSF1	MS4A2	KYVL39692	EPC2	LRRC17	MIB1	TMEM74	VPS13B	BCOR
ELOVL1	XDH	MTMR9	THOC2	LOC723972	MYNN	FRMD8	GPR4	SSH1	TMEM64	EXOC5
RXRA	GLS2	CYP4Z1	DDA1	HK1	ABCA2	TNPO3	SUCLG2	ZNF598	ASXL2	TMPPE
HERC2	TMEM189	LOC348840	FAM49A	DCAF13	IL1R2	CALU	OSBPL3	MERTK	C8orf37	SMG1
ADARB1	EN2	RAD54B	LYG2	TNFRSF10D	SHISA5	BTBD1	POC1B	CAST	P4HB	RGS17
PRDX3	UGT1A3	OTUD4	KCNT1	ASB9	PIAS2	DHX8	NBPF3	MED13	CTTNBP2NL	DNAJB3
EFTUD1	LOC550643	FGFR2	MTAP	PCDH11X	SLC25A46	FLJ23867	CALR3	VRK2	LOC201651	PRCP
SLC26A11	WDR31	MAGEE1	PPP2CB	PDCD4	ATXN7L3B	DNMT3B	LMBR1	G3BP1	GALNT2	UBE2D2
NAP1L2	ELK4	FRAS1	SNIP1	RHOJ	PALM2	IGSF9B	KCNJ6	EGFL7	EXOC6	PDLIM2
CCDC47	HLTF	CEP55	FLJ43390	RAPGEF1	EFNA5	SMEK2	MAP4	GBA	GALNTL6	KIAA1012

LCE1B	CALB2	KIF23	LOC90246	BAX	NBEAL1	NQO1	CLN3	GOLGA8E	WDR12	KLHL33
LCOR	TRIM62	C20orf94	SPTAN1	SLC25A40	AGTPBP1	GJA4	LRP8	SLC3A2	CEBPZ	PIK3R4
OTUD7B	RAB3D	NOS3	LIG3	CDK8	TTLL7	PIPSL	TNK2	PLEKHA3	LOC100128788	HSPA9
DNAJC16	LOC285419	SLC39A11	ANKRD34C	LOC728640	ZNF699	GPC1	VEZF1	VDR	RAPGEF4	MYH10
PLP2	GNPDA2	TPX2	ADIPOR1	FRMD4A	TMEM111	UVRAG	AGPAT3	DNAJC22	SEC61A1	IBTK
PRDM4	VPS4B	RNF2	LOC441089	OGFOD1	SLIT3	OCRL	SYTL3	PPL	NAT8L	KCNA2
OR2L1P	SORT1	SSX6	ECD	PCDHGA9	SYNM	NME1-NME2	DSE	C16orf87	SOCS7	DCP1A
LRRTM3	FAM102B	SRXN1	ZAK	IPO9	FAM119B	MFN2	CAND1	BRE	WDHD1	MMRN1
GPBP1L1	BARD1	TMEM38B	ENTPD7	FAM129B	WAPAL	C2orf29	ULBP3	STOX1	SMC4	C12orf34
TIRAP	UBE2E1	UBAP2L	TRIO	SPATA5L1	SCAPER	TREML3	TFRC	SLC17A5	POLD3	PRKD2
PDE4DIP	HIGD1A	FAM64A	PRSS8	C8orf75	SNAPC5	METAP2	ZNF434	FAM21A	TRAPPC10	CBL
SQSTM1	MGC87042	KDSR	SPEN	LPCAT4	ABCA3	RAB4A	CRK	TRAM2	ADCY5	GSTA1
HLCS	PSORS1C3	IGF2BP3	TMOD2	CMTM6	RNF169	C2orf49	AOC3	PROCR	TMEM14A	RUFY2
EPS15L1	PCDHGA7	TOR1AIP1	KIAA1704	LIMD1	EXPH5	SCIN	TTLL4	EXOSC6	PRPS1L1	AHRR
CCNB2	ACCN4	GLP2R	CADM2	C19orf21	SYCE2	FILIP1	DSCAM	PLK1	CUL5	NMD3
TRAF3	PPAPDC1A	HAT1	PBX3	C16orf75	PIGV	AASDHPPT	CHUK	SHC3	KIAA1644	MGC12982
EIF5A2	FAM82B	DAB1	SLC30A5	HIST4H4	PLSCR4	NACC2	ELOVL6	DLC1	CTSO	NCRNA00161
ADH5	SH3BP4	ISY1	TMEM97	FOXN2	FAM60A	C9orf172	SPAG9	CLSTN1	LOC650623	C6orf72
ZNF518B	MGAT2	CYP39A1	LOC285033	SAR1A	TLK1	PIAS4	SPINK7	ERAP2	KDM5B	GDF1
SUV39H2	ZNF619	BUB3	KAAG1	PRRT4	LOC388428	IL4R	RAD51	SLC29A3	KPRP	PLXNB3
TCF12	UTP3	CRTC3	CD3EAP	PDZD7	RGPD5	PIGK	MAMDC2	FAM83D	NTNG2	RPS6KA2
CCNE2	KDM1A	CNOT8	PSIP1	TOPBP1	SERPINH1	OR13C5	LAMC3	RPS4Y2	RPL7L1	HEXA
LOC400759	NTN4	ATL3	VAPA	SMCR8	GULP1	PANK3	C12orf48	C15orf38	SOCS5	SEC24B
STX1A	SNAPC1	SRD5A1	PIAS3	MOBKL3	LTBP1	KIF18A	MAN2A2	GRB2	IFITM3	AKR1C3
TSSC1	GGA2	PIGA	ACTN4	THBS4	GIGYF2	VPS13D	POSTN	ENOX2	PCBP1	TRIM68
ZNF436	SERINC1	MMP3	PHAX	FBXO30	ZNF749	SNW1	HBG2	MDH1	GLDC	MAGT1
CDK6	SPTBN2	ZNF791	VAV2	OSBPL8	ALG3	FOXK2	KRT4	FER	KIAA1919	EEPD1
DLEU2	PCDHA4	C17orf46	C15orf39	CHSY1	ARRB1	MYT1	HIST1H4H	PRCC	ZNF646	IGF2BP2

SET	LOC643837	MCART1	ITCH	CLEC14A	NUDT16P1	MRAP2	ETS1	FAM18B	IREB2	SLC25A33
B4GALT4	MCC	MCM6	SVIL	RFC1	CHPT1	DHRSX	LCLAT1	C1orf105	PHC3	BEND2
RPS6KC1	OGFRL1	MFGE8	GOSR1	ELMOD1	THEG	SFRS15	CFHR5	FCHSD2	HSDL2	FAM73A
C12orf62	KIF18B	KLHL3	RPN2	ERLEC1	SP3	SYK	PSG4	CA11	RPL13P5	WDR76
SLC38A10	UNC5B	MLPH	HTR3A	ZNF420	KIAA0101	NDST2	MAOA	ATXN3L	BARHL2	C11orf36
C12orf12	C14orf70	C1orf68	C21orf131	C5orf52	CSRP3	CYMP	DEFB129	FAM181A	FKSG73	FOXB1
GABRA4	GNRH2	GOLGA6C	GPR12	HSFYL1	HTR5A	IFNA7	IFNK	IL25	KCNK18	KLKP1
KRTAP4-4	KRTAP4-7	LOC285194	LOC286135	LOC29034	LOC340094	MGC34034	ODF4	OPRD1	OR10G4	OR10H1
OR11H4	OR1A2	OR1D4	OR1N2	OR4A15	OR4C3	OR4K17	OR51A4	OR51I2	OR51S1	OR52R1
OR56A4	OR5AR1	OR5B2	OR5K4	OR5L1	OR6C68	OR6N2	OR7A5	OR7G1	OR8D2	OR8D4
OR8H1	OR8J3	OR9I1	OR9K2	PRAMEF13	PRMT8	PROL1	PTPN20A	SERPINB10	SMEK3P	SNORA41
SNORA54	TAS2R16	TAS2R9	TMEM89	TMPRSS11BNL	USP26	USP46	C3orf70	C8orf33	NRBP1	LOC150527
PRUNE	ASB1	MATN2	HSP90B3P	C10orf26	CCNO	R3HDM1	TAF1B	KALRN	FMR1	LRP10
IFIT2	FAM163B	DENND5B	GCN1L1	DOPEY2	CDK12	CHST11	CASK	PRRC1	TMEM37	PLA2G2C
UBE2T	SOS 1	EIF1AD	PLD1	CYBASC3	REEP5	XKR6	ZNF785	JAZF1	CDKL5	NLGN1
ARL5A	HIPK1	ZFR	AURKA	PCM1	NUP205	CPA3	SPCS1	RLIM	CNIH4	AP2A1
DPF3	NEIL2	CCT2	PLEKHG1	FOLR2	SH3BP5	PAK7	C7orf45	DEFB126	IFLTD1	MAPKAP1
PLP1	CACNB4	MIA2	CDCA8	ZWINT	LAP3	DNM3	ADAM18	KLF5	DLGAP5	GPX5
SLC19A2	SMAD2	LHX3	CLDN16	CASP3	SNAPC2	BCL9L	GNG8	C7orf70	CENPA	MFSD2B
SCNN1A	IGSF3	NUDT16L1	THAP1	CKAP2L	NBPF1	C14orf48	SEC23B	KLF10	C9orf69	ARID1A
BCL6	FBXL4	USP34	PAQR4	CPSF2	WARS	KLHL2	ANKRD34A	CHRNA1	COL4A1	SOX2
ELAVL3	PCDH7	PMP2	CCT8	CECR7	CCNK	LDB2	NOP58	TK1	CDC42SE1	ATP1A1
CCDC146	MAP3K14	MBOAT1	ZFAND1	VKORC1L1	GEMIN4	MAT1A	AIFM2	KCNV1	DTL	RUSC1
ANXA7	DNTTIP2	NUP62	ZNF134	HDAC3	TRAPPC9	DNAH3	C2orf86	MITF	MRPL37	KIF13B
INPP4A	GMPR	PSMB5	ATL2	ELN	KLHDC8A	CADPS	ZCCHC17	TSKU	ATP6AP1	IQGAP3
ADSS	PPP1R3D	ILF2	BTG2	EFHB	TIMM17A	BYSL	PLG	INTS8	KLHL21	WNK4
RPS6KA3	NRG3	TBC1D14	ALPP	SLC30A9	HEXIM1	SLC1A2	IKZF5	EXOC3L2	CNTN5	TWIST2
GAN	MPHOSPH9	SMG5	TMEM84	SLC5A12	RBL1	EMX2OS	MGC15885	CLASP1	SLC35A5	POLR2A

SPTLC3	NCEH1	GPN2	RABL3	LOC90586	ENPP2	C8orf83	TDRG1	KIF2C	SUMO1	SOX8
C9orf79	KIF1B	ZNF543	ZNF704	UBASH3B	C16orf63	MFSD1	ERCC6L	ZNF496	APOH	CYB5R1
IGSF21	FOXD2	EPC1	MRPL15	ANGPT1	NCRNA00152	VPS35	UCHL5	KIAA0020	RAB35	CLPB
MND1	MAP4K5	RAPH1	GRIA2	TNXB	AMPH	PCNA	VPS41	FLNB	DPYD	CTNND2
FAF1	RECK	KLF6	ACLY	SYVN1	RAB6B	ADAT1	DOCK9	ACAT2	NOTCH3	PLAC1
TXNDC16	HDAC2	PAQR3	TSNAX-DISC1	SLC38A7	DLL1	SH3TC2	C1orf177	DDX47	FAT4	MRPL3
ZNF766	PIGF	KCNQ1	CCNE1	GAA	NPBWR1	LPIN2	PRKG1	TFB2M	CCDC155	SAMD12
37681	NRG2	FAM72D	SYNGR2	SPATA6	FZD10	PPA1	REM1	FKBP15	SYTL4	SHPRH
FMR1NB	YBX1									

Table S8: a)Functional enrichment analysis data of lost genes in KIPAN, [KIPAN= Pan-kidney cohort (KICH+KIRC+KIRP)

	ID	Description	GeneRatio	BgRatio	pvalue	p.adjust	qvalue	geneID	Count
R-HSA-1474244	R-HSA-1474244	Extracellular matrix organization	88/1288	301/10619	5.70E-16	7.22E-13	6.43E-13	SERPINH1/GDF5/COL8A1/COL16A1/COL1A1/COL5A1/ICAM4/COL1A2/ELN/COL11A1/MMP14/EMILIN2/PCOLCE/TIMP1/ADAMTS2/COL3A1/COMP/MFAP5/TGFB3/PXDN/LOXL1/COL6A2/COL5A2/BMP1/TNXB/COL4A2/MMP11/NID2/ADAM19/MMP19/VCAM1/MMP2/MATN3/EFEMP2/ITGAX/COL4A1/LOXL3/MMP17/ADAM12/LTBP2/ADAMTS3/SDC3/FBN1/COL6A1/FBLN1/CD44/ITGB3/MATN4/EMILIN1/CD151/TIMP2/COL18A1/MMP16/ADAM8/SDC4/ITGB2/EFEMP1/COL15A1/PPIB/ITGAL/MMP25/TGFB2/BMP2/COL5A3/COL25A1/COL21A1/JAM3/ACTN1/BGN/A2M/ITGB1/COL12A1/ITGA5/MMP8/COL27A1/SPARC/TGFB1/PDGFB/P4HA2/LAMC1/COL6A3/PLOD1/NID1/PLOD2/ADAMTS4/SERPINE1/ICAM1/ADAMTS9	88
R-HSA-449147	R-HSA-449147	Signaling by Interleukins	117/1288	463/10619	1.40E-15	8.90E-13	7.92E-13	CXCL1/IL6/CSF1/TNFRSF1A/COL1A2/FSCN1/TIMP1/CCL4/ANXA1/FPR1/IL4R/TWIST1/IL18BP/LGALS9/CLCF1/MAP3K8/RIPK2/RHOU/NFKB2/VCAM1/MMP2/IL1R1/TNFRSF1B/PIM1/SOD2/CNN2/CEBPD/BATF/ITGAX/IRAK2/CD86/HCK/CD80/CSF3R/MYD88/CCL3/IL7R/MAPK7/CSF2RA/RELA/GAB2/CFL1/JAK3/ALOX5/PSMB9/NKIRAS2/ITGB2/CASP1/CCL5/IL21R/CD4/HSP90B1/IL10RA/CSF2RB/IL32/TP53/OSMR/PSMB10/LCP1/VAV1/ANXA2/IL1B/CSF3/IL12RB1/HAVCR2/CD36/RPS6KA5/MSN/F13A1/CXCL2/JUN/CCL2/S100A12/PSMD11/VIM/PELI1/IL2RA/STAT4/PIK3R1/IRAK3/OSM/SHC1/IL10/ITGB1/LYN/LIF/IL13RA2/CSF1R/UBC/NOS2/IL6R/MAP2K3/FYN/IL24/CAPZA1/HMOX1/TGFB1/CCL19/SOCS5/CCR1/IL13/PTPN18/INPP5D/CDKN1A/CRK/EBI3/PAK2/IL18R1/PIK3CD/NFKB1/ICAM1/NFKBIA/ATF2/YWHAZ/CCL20/CA1/BCL6	117
R-HSA-6798695	R-HSA-6798695	Neutrophil degranulation	119/1288	480/10619	3.59E-15	1.52E-12	1.35E-12	CXCL1/PLAUR/PLEKHO2/HK3/RHOG/OSCAR/C5AR1/PTX3/CD177/CFP/FPR1/RAP2B/GPR84/FGR/CLEC12A/FCAR/TMEM173/FCN1/TNFRSF1B/PLAC8/CNN2/TYROBP/CKAP4/ITGAX/CD300A/NFAM1/CTSZ/GLIPR1/RETN/MAN2B1/PIGR/GMFG/FCER1G/DEGS1/PYCARD/SERPINA3/CD14/CD44/CRISPLD2/SIGLEC9/OLFM4/CD63/FPR2/TIMP2/HLA-B/ALOX5/C3AR1/ADAM8/LAIR1/ITGB2/TLR2/RAB31/LYZ/TRPM2/FCGR2A/HP/SERPINB1/ITGAL/IMPDH2/ANXA2/ARL8A/MMP25/NME1-NME2/DOCK2/CEACAM3/HBB/CD36/PYGL/S100A12/S100A9/SLC15A4/PSMD11/CAP1/S100A8/STK10/COTL1/RNASE2/KPNB1/SIRPB1/KCMF1/SELL/CLEC4D/FCGR3B/CLEC4C/TUBB/DDX3X/SLC11A1/SIRPA/CXCR2/CD53/MNDA/ACLY/MMP8/CD33/S100A7/NRAS/CD68/P2RX1/SIGLEC5/CR1/NCKAP1L/C3/CXCR1/IDH1/CYBB/CD93/APAF1/ACTR2/RAB9B/ARSB/ILF2/NFKB1/ORM1/XRCC5/CCT2/GDI2/HSP90AB1/SDCBP/PPBP	119

R-HSA-198933	R-HSA-198933	Immunoregulatory interactions between a Lymphoid and a non-Lymphoid cell	51/1288	132/10619	4.90E-15	1.55E-12	1.38E-12	COL1A1/ICAM4/COL1A2/COL3A1/PVR/OSCAR/TREM1/VCAM1/CD300LB/SIGLEC1/CD300LF/TYROBP/CD300C/CD300A/LILRB4/IFITM1/LILRA4/HCST/FCGR1A/CD40LG/LILRA2/CD300E/SIGLEC9/LILRA5/CD3E/LILRB1/HLA-B/LAIR1/HLA-E/ITGB2/NPDC1/CD200/LILRA1/ITGAL/HLA-A/CD3D/SLAMF6/CD96/SELL/ITGB1/CD33/KLRG1/CD226/SIGLEC5/C3/SIGLEC10/SIGLEC12/CD1D/CLEC2B/FCGR3A/ICAM1	51
R-HSA-6785807	R-HSA-6785807	Interleukin-4 and Interleukin-13 signaling	40/1288	108/10619	2.11E-11	5.35E-09	4.76E-09	IL6/COL1A2/FSCN1/TIMP1/ANXA1/IL4R/TWIST1/RHOU/VCAM1/MMP2/TNFRSF1B/PIM1/CEBPD/BATF/ITGAX/JAK3/ALOX5/ITGB2/HSP90B1/TP53/IL1B/CD36/F13A1/CCL2/VIM/PIK3R1/OSM/IL10/ITGB1/LIF/IL13RA2/NOS2/IL6R/HMOX1/TGFB1/SOCS5/IL13/CDKN1A/ICAM1/BCL6	40
R-HSA-6783783	R-HSA-6783783	Interleukin-10 signaling	24/1288	47/10619	8.39E-11	1.77E-08	1.58E-08	CXCL1/IL6/CSF1/TNFRSF1A/TIMP1/CCL4/FPR1/IL1R1/TNFRSF1B/CD86/CD80/CCL3/CCL5/IL10RA/IL1B/CSF3/CXCL2/CCL2/IL10/LIF/CCL19/CCR1/ICAM1/CCL20	24
R-HSA-1650814	R-HSA-1650814	Collagen biosynthesis and modifying enzymes	29/1288	67/10619	1.57E-10	2.84E-08	2.52E-08	SERPINH1/COL8A1/COL16A1/COL1A1/COL5A1/COL1A2/COL11A1/PCOLCE/ADAMTS2/COL3A1/COL6A2/COL5A2/BMP1/COL4A2/COL4A1/ADAMTS3/COL6A1/COL18A1/COL15A1/PPIB/COL5A3/COL25A1/COL21A1/COL12A1/COL27A1/P4HA2/COL6A3/PLOD1/PLOD2	29
R-HSA-1474290	R-HSA-1474290	Collagen formation	33/1288	90/10619	1.63E-09	2.58E-07	2.30E-07	SERPINH1/COL8A1/COL16A1/COL1A1/COL5A1/COL1A2/COL11A1/PCOLCE/ADAMTS2/COL3A1/PXDN/LOXL1/COL6A2/COL5A2/BMP1/COL4A2/COL4A1/LOXL3/ADAMTS3/COL6A1/CD151/COL18A1/COL15A1/PPIB/COL5A3/COL25A1/COL21A1/COL12A1/COL27A1/P4HA2/COL6A3/PLOD1/PLOD2	33
R-HSA-76002	R-HSA-76002	Platelet activation, signaling and aggregation	65/1288	262/10619	7.90E-09	1.11E-06	9.90E-07	COL1A1/COL1A2/TIMP1/PTPN1/RHOG/TGFB3/TAGLN2/LCP2/GNAI2/IGF1/ISLR/SERPING1/PLEK/GNA15/FERMT3/RAC2/MANF/FCER1G/SERPINA3/ITGB3/CD63/PFN1/CFL1/HSPA5/PIK3R5/APBB1IP/ACTN4/ARRB2/VAV1/LEFTY2/VEGFC/GTPBP2/TGFB2/CD36/TIMP3/FLNA/F13A1/ACTN1/GNB1/CAP1/GNAI3/PIK3R1/SHC1/PROS1/ARRB1/A2M/LYN/ANXA5/RASGRP2/FYN/F2R/SPARC/PRKCB/TGFB1/PDGFB/LHFPL2/CRK/GNA13/SERPINE1/ORM1/YWHAZ/OLA1/SRGN/MMRN1/PPBP	65
R-HSA-76005	R-HSA-76005	Response to elevated platelet cytosolic Ca2+	40/1288	134/10619	3.02E-08	3.52E-06	3.13E-06	TIMP1/TGFB3/TAGLN2/IGF1/ISLR/SERPING1/PLEK/FERMT3/MANF/SERPINA3/ITGB3/CD63/PFN1/CFL1/HSPA5/ACTN4/LEFTY2/VEGFC/GTPBP2/TGFB2/CD36/TIMP3/FLNA/F13A1/ACTN1/CAP1/PROS1/A2M/ANXA5/SPARC/PRKCB/TGFB1/PDGFB/LHFPL2/SERPINE1/ORM1/OLA1/SRGN/MMRN1/PPBP	40

b) Functional enrichment analysis of conserved genes in KIPAN

	ID	Description	GeneRatio	BgRatio	pvalue	p.adjust	qvalue	geneID	Count
R-HSA-6785807	R-HSA-6785807	Interleukin-4 and Interleukin-13 signaling	16/339	108/10619	2.89E-07	0.000288074	0.000257356	JUNB/MCL1/SOCS3/CCL2/ICAM1/LIF/STAT3/VIM/FOS/IL6R/VEGFA/S1PR1/ZEB1/CEBPD/BCL6/IL1B	16
R-HSA-449147	R-HSA-449147	Signaling by Interleukins	35/339	463/10619	1.78E-06	0.000868538	0.000775922	JUNB/MCL1/SOCS3/CCL2/ICAM1/NFKBIA/NFKB1/MSN/LIF/SHC1/PTPN12/STAT3/VIM/CXCL2/JUN/S100A12/PELI1/FOS/UBC/IL6R/FYN/VEGFA/S1PR1/TCP1/ZEB1/HNRNPF/IL1RAP/CEBPD/ATF2/OSMR/CCL20/BCL6/IL7/IL1B/RPS6KA5	35
R-HSA-177929	R-HSA-177929	Signaling by EGFR	10/339	49/10619	2.61E-06	0.000868538	0.000775922	SPRY1/HBEGF/AREG/SPRY2/NRAS/SHC1/ADAM17/PTPN12/UBC/TGFA	10

R-HSA-72203	R-HSA-72203	Processing of Capped Intron-Containing Pre-mRNA	22/339	244/10619	1.10E-05	0.002734546	0.00244295	WTAP/TRA2B/POLR2D/CWC22/NUP62/PRPF38A/RBM8A/HNRNPA3/PCBP1/HNRNPU/NUP153/HNRNPK/NUP98/HNRNPF/EIF4A3/SF3B1/CDC5L/PRPF40A/HNRNPC/HNRNPR/SMNDC1/RBMX	22
R-HSA-72163	R-HSA-72163	mRNA Splicing - Major Pathway	18/339	183/10619	2.18E-05	0.004358388	0.003893634	TRA2B/POLR2D/CWC22/PRPF38A/RBM8A/HNRNPA3/PCBP1/HNRNPU/HNRNPK/HNRNPF/EIF4A3/SF3B1/CDC5L/PRPF40A/HNRNPC/HNRNPR/SMNDC1/RBMX	18
R-HSA-72172	R-HSA-72172	mRNA Splicing	18/339	191/10619	3.88E-05	0.006144573	0.00548935	TRA2B/POLR2D/CWC22/PRPF38A/RBM8A/HNRNPA3/PCBP1/HNRNPU/HNRNPK/HNRNPF/EIF4A3/SF3B1/CDC5L/PRPF40A/HNRNPC/HNRNPR/SMNDC1/RBMX	18
R-HSA-182971	R-HSA-182971	EGFR downregulation	7/339	31/10619	4.31E-05	0.006144573	0.00548935	SPRY1/HBEGF/AREG/SPRY2/PTPN12/UBC/TGFA	7
R-HSA-180336	R-HSA-180336	SHC1 events in EGFR signaling	5/339	14/10619	5.08E-05	0.006339502	0.005663493	HBEGF/AREG/NRAS/SHC1/TGFA	5
R-HSA-1643713	R-HSA-1643713	Signaling by EGFR in Cancer	6/339	25/10619	0.000107226	0.011890142	0.010622244	HBEGF/AREG/NRAS/SHC1/UBC/TGFA	6
R-HSA-9009391	R-HSA-9009391	Non-genomic estrogen signaling	10/339	77/10619	0.000157737	0.015742117	0.014063467	SRF/HBEGF/AREG/NRAS/SHC1/GNB1/GNAI3/FOS/S1PR3/TGFA	10

c) Functional enrichment analysis of acquired genes in KIPAN

	ID	Description	GeneRatio	BgRatio	pvalue	p.adjust	qvalue	geneID	Count
R-HSA-449147	R-HSA-449147	Signaling by Interleukins	88/943	463/10619	2.62E-12	3.19E-09	2.87E-09	JUN/PELI1/FOS/S1PR1/UBC/VEGFA/CD36/TCP1/RPS6KA5/FYN/IL6R/S100A12/ZEB1/HNRNPF/BCL6/IL4R/F13A1/FPR1/OSMR/MYD88/PSMD11/IL1B/IL2RA/STAT4/TNFRSF1A/PIK3R1/NFKB2/IRAK3/TNFRSF1B/OSM/IL7R/CSF2RB/CEBPD/IL10/ITGB1/CASP1/LYN/IL13RA2/CSF1R/CD86/LCP1/NOS2/MAP2K3/IL1R1/IRAK2/IL24/CD4/CAPZA1/ATF2/HMOX1/HCK/TGFB1/CCL19/SOCS5/CCR1/SOD2/IL1RAP/VCAM1/CSF3R/IL13/PTPN18/INPP5D/CDKN1A/CRK/VAV1/CSF1/EBI3/PAK2/IL18R1/PIK3CD/IL21R/PSMB2/FN1/IL10RB/CD80/IL10RA/MAPK14/ITGAM/IL6ST/STX4/YWHAZ/CA1/DUSP6/CREB1/MAPK3/MAPK8/IL1RAPL1/MTAP	88
R-HSA-6798695	R-HSA-6798695	Neutrophil degranulation	78/943	480/10619	8.03E-08	4.89E-05	4.39E-05	ACLY/HBB/CD36/KPNB1/GDI2/SLC15A4/S100A12/FPR2/S100A9/FPR1/C5AR1/PSMD11/PLEKHO2/CAP1/PLAUR/S100A8/CRISPLD2/COTL1/RNASE2/FCGR2A/TNFRSF1B/SIRPB1/KCMF1/SELL/CLEC4D/FCGR3B/CLEC4C/TUBB/CEACAM3/GLIPR1/SLC11A1/SIRPA/CXCR2/HK3/CD53/MNDA/MMP8/FCN1/CD33/CD14/S100A7/LAIR1/CD68/GPR84/P2RX1/SIGLEC5/CR1/NCKAP1L/C3/SIGLEC9/CXCR1/IDH1/TMEM173/DOCK2/CYBB/XRCC5/C3AR1/NFAM1/APAF1/ACTR2/RAB9B/ARSB/ILF2/FCAR/CREG1/HVCN1/CYFIP1/MAPK14/LTA4H/ITGAM/FCER1G/ORM1/CCT2/PLAC8/HSP90AB1/DDX3X/PNP/ATP11B	78
R-HSA-194138	R-HSA-194138	Signaling by VEGF	26/943	107/10619	1.54E-06	0.000565707	0.000507968	VEGFA/NOS3/FLT1/FLT4/CDH5/FYN/MAPK12/NRP2/PIK3R1/ELMO1/AXL/PRKCB/WASF2/NCKAP1L/CYBB/CRK/DOCK1/VAV1/PAK2/CYFIP1/MAPK14/CTNNB1/NRP1/ROCK2/NCK1/AKT3	26
R-HSA-6785807	R-HSA-6785807	Interleukin-4 and Interleukin-13 signaling	26/943	108/10619	1.86E-06	0.000565707	0.000507968	FOS/S1PR1/VEGFA/CD36/IL6R/ZEB1/BCL6/IL4R/F13A1/IL1B/PIK3R1/TNFRSF1B/OSM/CEBPD/IL10/ITGB1/IL13RA2/NOS2/HMOX1/TGFB1/SOCS5/VCAM1/IL13/CDKN1A/FN1/ITGAM	26

R-HSA-2029482	R-HSA-2029482	Regulation of actin dynamics for phagocytic cup formation	18/943	61/10619	3.32E-06	0.000797642	0.000716231	FCGR2A/ELMO1/ARPC2/ACTR3/WASF2/WIPF2/NCKAP1L/ACTR2/CRK/DOCK1/VAV1/FCGR3A/CYFIP1/FCGR1A/WAS/HSP90AB1/MAPK3/NCK1	18
R-HSA-2029480	R-HSA-2029480	Fcgamma receptor (FCGR) dependent phagocytosis	22/943	86/10619	3.93E-06	0.000797642	0.000716231	FYN/PIK3R1/FCGR2A/ELMO1/ARPC2/LYN/ACTR3/WASF2/WIPF2/NCKAP1L/HCK/ACTR2/CRK/DOCK1/VAV1/FCGR3A/CYFIP1/FCGR1A/WAS/HSP90AB1/MAPK3/NCK1	22
R-HSA-4420097	R-HSA-4420097	VEGFA-VEGFR2 Pathway	22/943	99/10619	4.33E-05	0.007453872	0.006693088	VEGFA/NOS3/CDH5/FYN/MAPK12/PIK3R1/ELMO1/AXL/PRKCB/WASF2/NCKAP1L/CYBB/CRK/DOCK1/VAV1/PAK2/CYFIP1/MAPK14/CTNNB1/ROCK2/NCK1/AKT3	22
R-HSA-9006934	R-HSA-9006934	Signaling by Receptor Tyrosine Kinases	66/943	458/10619	4.90E-05	0.007453872	0.006693088	NEDD4/UBC/VEGFA/COL5A3/NOS3/FLT1/RALB/FLT4/RPS6KA5/CDH5/FYN/ARHGEF7/INSR/HNRNPF/MAPK12/NRP2/PIK3R1/ELMO1/ITGB1/LYN/MKNK1/IRS4/HNRNPH1/AXL/COL27A1/PAG1/PRKCB/WASF2/COL4A2/NCKAP1L/CYBB/LAMC1/GRAP2/PTPN18/SH3KBP1/CRK/SH3GL1/DOCK1/VAV1/FES/COL4A1/CXCL12/PAK2/COL5A2/CYFIP1/ADORA2A/NTF3/FN1/MAPK14/LAMC3/CTNNB1/EREG/DUSP6/SPRED2/SH2B3/NRP1/GRB10/SPRY2/ROCK2/CREB1/STAM/MAPK3/PCSK5/NCK1/AKT3/HNRNPM	66
R-HSA-166058	R-HSA-166058	MyD88:MAL (TIRAP) cascade initiated on plasma membrane	21/943	95/10619	6.97E-05	0.008210744	0.007372711	JUN/PELI1/FOS/UBC/CD36/RPS6KA5/S100A12/MYD88/NFKB2/IRAK3/TLR6/CD14/MAP2K3/IRAK2/ATF2/TLR1/MAPK14/DUSP6/CREB1/MAPK3/MAPK8	21
R-HSA-168188	R-HSA-168188	Toll Like Receptor TLR6:TLR2 Cascade	21/943	95/10619	6.97E-05	0.008210744	0.007372711	JUN/PELI1/FOS/UBC/CD36/RPS6KA5/S100A12/MYD88/NFKB2/IRAK3/TLR6/CD14/MAP2K3/IRAK2/ATF2/TLR1/MAPK14/DUSP6/CREB1/MAPK3/MAPK8	21

Table S9 a)Functional enrichment analysis data of lost genes in KIRC, [KIRC=Kidney Renal Clear Cell Carcinoma]

	ID	Description	GeneRatio	BgRatio	pvalue	p.adjust	qvalue	geneID	Count
R-HSA-449147	R-HSA-449147	Signaling by Interleukins	120/1259	463/10619	1.22E-17	1.56E-14	1.42E-14	ANXA1/CSF1/CXCL1/MMP2/IL4R/TIMP1/CRLF2/VIM/VCAM1/ITGAX/FSCN1/TWIST1/BATF/IL7R/SHC1/TNFRSF1B/RHOU/CTSG/IL1R1/CCL4/IL18BP/COL1A2/IL1B/CCL22/HSP90B1/JAK3/IL1F10/MAP3K8/RIPK2/FPR1/IL31RA/IL21R/MSN/IL10RA/IL34/PTPN12/SOD2/VAV1/STAT4/CSF2RA/CEBPD/OSMR/CSF2RB/IL16/FLT3LG/IRAK3/CCL5/IL6/STX1A/CD4/INPP5D/LGALS9/GAB2/IL12RB1/CD86/IL1RL1/MAPK7/IL1RN/OSM/F13A1/CSF3/CCL3/CXCL2/MAP2K3/CCL3L1/IL2RA/IL13RA2/TNFRSF1A/IL10/NOS2/IL13/HCK/NFKB1/MYD88/CCL19/CRKL/ALOX15/FCER2/PSMD11/PELI1/CSF3R/IL17RA/CSF1R/SERPINB2/LIF/NFKB2/PIK3R1/TGFB1/UBC/LYN/IL24/CD80/LMNB1/CCR1/PSMD1/CASP1/ITGAM/CCL11/LCP1/FYN/PRTN3/PIM1/IL1RAP/IL1RL2/ICAM1/BCL6/CA1/CCL20/STAT3/YWHAZ/CDKN1A/HIF1A/HNRNPF/RELA/PPP2R1B/TCP1/ATF2/S100A12/PSMC6/CLCF1	120

R-HSA-1474244	R-HSA-1474244	Extracellular matrix organization	86/1259	301/10619	1.42E-15	9.07E-13	8.26E-13	SERPINH1/COL8A1/MMP14/MMP19/MMP2/LOXL1/TIMP1/COL16A1/ADAM8/PXDN/CD44/GDF5/TGFB3/COL6A2/LOXL3/VCAM1/ITGAX/EMILIN2/ICAM4/PCOLCE/ELN/ITGB3/SDC3/ACTN1/CTSG/ADAM19/EFEMP1/COMP/COL1A1/ITGA7/COL6A1/COL1A2/CMA1/MMP11/TNXB/ITGAL/ACAN/TIMP2/ADAMTS2/COL18A1/COL5A1/NID2/SPARC/COL4A2/CD151/ITGB8/COL6A3/ITGA3/LTBP2/SDC4/PECAM1/MMP17/TPSAB1/BMP1/A2M/LTBP4/MATN3/BMP2/SERPINE1/ADAMTS9/MMP10/MMP8/MMP12/BGN/FBN2/MMP25/LAMC3/DDR2/FBN1/ITGA5/TNC/COL12A1/TGFB1/KLK7/COL15A1/MATN1/ITGAM/PDGFB/SH3PXD2A/LAMA1/HSPG2/ADAMTS4/ICAM1/ADAM17/PSEN1/THBS1	86
R-HSA-6783783	R-HSA-6783783	Interleukin-10 signaling	28/1259	47/10619	6.55E-15	2.80E-12	2.55E-12	CSF1/CXCL1/TIMP1/TNFRSF1B/IL1R1/CCL4/IL1B/CCL22/FPR1/IL10RA/CCL5/IL6/CD86/IL1RN/CSF3/CCL3/CXCL2/CCL3L1/TNFRSF1A/IL10/CCL19/FCER2/LIF/CD80/CCR1/ICAM1/CCL20/STAT3	28
R-HSA-6785807	R-HSA-6785807	Interleukin-4 and Interleukin-13 signaling	38/1259	108/10619	1.97E-10	6.30E-08	5.73E-08	ANXA1/MMP2/IL4R/TIMP1/VIM/VCAM1/ITGAX/FSCN1/TWIST1/BATF/TNFRSF1B/RHOU/COL1A2/IL1B/CCL22/HSP90B1/JAK3/CEBPD/IL6/OSM/F13A1/IL13RA2/IL10/NOS2/IL13/ALOX15/FCER2/LIF/PIK3R1/TGFB1/ITGAM/CCL11/PIM1/ICAM1/BCL6/STAT3/CDKN1A/HIF1A	38
R-HSA-198933	R-HSA-198933	Immunoregulatory interactions between a Lymphoid and a non-Lymphoid cell	41/1259	132/10619	2.88E-09	7.37E-07	6.71E-07	CD40LG/VCAM1/CD3E/PVR/ICAM4/SLAMF6/HCST/COL1A1/OSCAR/COL1A2/TREM1/NPDC1/ITGAL/CD3G/LAIR1/CLEC2B/IFITM1/CD300LF/CD3D/LILRA4/CD300LB/TREML2/CD300A/CD96/TYROBP/HLA-E/CRTAM/SIGLEC1/SH2D1A/CD300E/LILRA5/SELL/SIGLEC12/SIGLEC5/LILRB1/SIGLEC10/SIGLEC9/LILRA1/CD300C/CD33/ICAM1	41
R-HSA-380108	R-HSA-380108	Chemokine receptors bind chemokines	22/1259	48/10619	4.57E-09	9.75E-07	8.88E-07	CXCL6/CXCL1/CCL21/CXCL16/CXCR4/CCR7/CXCL3/CCRL2/CCR6/CXCR3/CCL5/CX3CL1/CXCR2/CXCR1/CXCL2/CCL19/CCL13/CXCR5/CCR1/CCR8/CCL20/PPBP	22
R-HSA-6798695	R-HSA-6798695	Neutrophil degranulation	97/1259	480/10619	5.43E-08	9.80E-06	8.92E-06	PLEKHO2/CXCL1/ADAM8/CD44/RHOG/PIGR/ITGAX/RAB31/TNFRSF1B/TMEM173/CTSG/HP/PLAUR/NFAM1/OSCAR/FGR/CFP/TBC1D10C/ITGAL/TIMP2/LRG1/TXNDC5/FPR1/LAIR1/CKAP4/MAN2B1/PTX3/STK10/DOK3/PLAC8/GMFG/GPR84/GLIPR1/CD300A/TYROBP/CLEC12A/CD63/PECAM1/TLR2/OLFM4/DEGS1/RHOA/PNP/C5AR1/CXCR2/S100A9/CXCR1/FCGR3B/CRISPLD2/MMP8/CLEC4D/S100A8/S100A7/SELL/FCGR2A/HK3/CLEC4C/CEACAM3/CR1/CD14/NFKB1/SLC15A4/RNASE2/SLC11A1/MMP25/P2RX1/PSMD11/FCN1/IDH1/C3AR1/SIGLEC5/COTL1/VCL/MNDA/SIRPB1/CD53/SIGLEC9/PSMD1/ITGAM/CD93/MPO/CYFIP1/KCMF1/CD33/CYBB/PRTN3/ORM1/CCT2/HBB/PPBP/ACTR10/PSEN1/PYGL/S100A12/FCAR/FPR2/VAPA	97
R-HSA-1474228	R-HSA-1474228	Degradation of the extracellular matrix	40/1259	140/10619	6.12E-08	9.80E-06	8.92E-06	COL8A1/MMP14/MMP19/MMP2/TIMP1/COL16A1/ADAM8/CD44/COL6A2/ELN/CTSG/COL1A1/COL6A1/COL1A2/CMA1/MMP11/ACAN/TIMP2/COL18A1/COL5A1/COL4A2/COL6A3/MMP17/TPSAB1/BMP1/A2M/ADAMTS9/MMP10/MMP8/MMP12/FBN2/MMP25/FBN1/COL12A1/KLK7/COL15A1/HSPG2/ADAMTS4/ADAM17/PSEN1	40
R-HSA-216083	R-HSA-216083	Integrin cell surface interactions	28/1259	85/10619	2.36E-07	3.36E-05	3.06E-05	COL8A1/COL16A1/CD44/COL6A2/VCAM1/ITGAX/ICAM4/ITGB3/COMP/COL1A1/ITGA7/COL6A1/COL1A2/ITGAL/COL18A1/COL5A1/COL4A2/ITGB8/COL6A3/ITGA3/PECAM1/FBN1/ITGA5/TNC/ITGAM/HSPG2/ICAM1/THBS1	28
R-HSA-76002	R-HSA-76002	Platelet activation, signaling and aggregation	59/1259	262/10619	6.32E-07	8.10E-05	7.37E-05	RAC2/PTPN1/TIMP1/LAT/RHOG/TGFB3/GNAI2/RASGRP2/PIK3R6/DGKA/SHC1/ITGB3/SERPING1/ACTN1/FERMT3/LCP2/FLNA/COL1A1/COL1A2/TAGLN2/GNG8/APBB1IP/ISLR/GNA15/TLN1/PIK3R5/VAV1/SPARC/PLEK/CALU/DGKG/CD63/PECAM1/A2M/RHOA/F2RL3/SERPINE1/GTPBP2/F13A1/GNA12/ARRB1/GNB1/PIK3R1/TGFB1/VCL/LYN/LHFPL2/PDGFB/FYN/PRKCB/ORM1/MMRN1/YWHAZ/HSPA5/SRGN/PPBP/CD109/F2R/THBS1	59

b)Functional enrichment analysis data of conserved genes in KIRC

	ID	Description	GeneRatio	BgRatio	pvalue	p.adjust	qvalue	geneID	Count
R-HSA-449147	R-HSA-449147	Signaling by Interleukins	35/206	463/10619	3.03E-12	2.05E-09	1.72E-09	SOCS3/MCL1/CLCF1/CDKN1A/NFKB1/CCL2/CXCL2/IRAK2/LIF/RELA/MAPK7/NFKB2/STAT3/TNFRSF1A/PIM1/NFKBIA/ICAM1/JUNB/JUN/DUSP6/MAP2K3/FOS/PELI1/CEBPD/BCL6/CSF1/HNRNPF/OSMR/ATF2/IL4R/S100A12/IL1B/PTPN12/S1PR1/RPS6KA5	35
R-HSA-975138	R-HSA-975138	TRAF6 mediated induction of NFkB and MAP kinases upon TLR7/8 or 9 activation	15/206	92/10619	2.19E-10	4.07E-08	3.40E-08	TICAM1/NFKB1/IRAK2/RELA/MAPK7/NFKB2/NFKBIA/JUN/DUSP6/MAP2K3/FOS/PELI1/ATF2/S100A12/RPS6KA5	15
R-HSA-168181	R-HSA-168181	Toll Like Receptor 7/8 (TLR7/8) Cascade	15/206	93/10619	2.57E-10	4.07E-08	3.40E-08	TICAM1/NFKB1/IRAK2/RELA/MAPK7/NFKB2/NFKBIA/JUN/DUSP6/MAP2K3/FOS/PELI1/ATF2/S100A12/RPS6KA5	15
R-HSA-975155	R-HSA-975155	MyD88 dependent cascade initiated on endosome	15/206	93/10619	2.57E-10	4.07E-08	3.40E-08	TICAM1/NFKB1/IRAK2/RELA/MAPK7/NFKB2/NFKBIA/JUN/DUSP6/MAP2K3/FOS/PELI1/ATF2/S100A12/RPS6KA5	15
R-HSA-168164	R-HSA-168164	Toll Like Receptor 3 (TLR3) Cascade	15/206	94/10619	3.00E-10	4.07E-08	3.40E-08	TICAM1/NFKB1/IRAK2/RELA/MAPK7/RIPK1/NFKB2/NFKBIA/JUN/DUSP6/MAP2K3/FOS/ATF2/S100A12/RPS6KA5	15
R-HSA-168138	R-HSA-168138	Toll Like Receptor 9 (TLR9) Cascade	15/206	97/10619	4.75E-10	4.79E-08	4.00E-08	TICAM1/NFKB1/IRAK2/RELA/MAPK7/NFKB2/NFKBIA/JUN/DUSP6/MAP2K3/FOS/PELI1/ATF2/S100A12/RPS6KA5	15
R-HSA-166166	R-HSA-166166	MyD88-independent TLR4 cascade	15/206	99/10619	6.39E-10	4.79E-08	4.00E-08	TICAM1/NFKB1/IRAK2/RELA/MAPK7/RIPK1/NFKB2/NFKBIA/JUN/DUSP6/MAP2K3/FOS/ATF2/S100A12/RPS6KA5	15
R-HSA-937061	R-HSA-937061	TRIF(TICAM1)-mediated TLR4 signaling	15/206	99/10619	6.39E-10	4.79E-08	4.00E-08	TICAM1/NFKB1/IRAK2/RELA/MAPK7/RIPK1/NFKB2/NFKBIA/JUN/DUSP6/MAP2K3/FOS/ATF2/S100A12/RPS6KA5	15
R-HSA-168142	R-HSA-168142	Toll Like Receptor 10 (TLR10) Cascade	14/206	85/10619	7.77E-10	4.79E-08	4.00E-08	NFKB1/IRAK2/RELA/MAPK7/NFKB2/NFKBIA/JUN/DUSP6/MAP2K3/FOS/PELI1/ATF2/S100A12/RPS6KA5	14
R-HSA-168176	R-HSA-168176	Toll Like Receptor 5 (TLR5) Cascade	14/206	85/10619	7.77E-10	4.79E-08	4.00E-08	NFKB1/IRAK2/RELA/MAPK7/NFKB2/NFKBIA/JUN/DUSP6/MAP2K3/FOS/PELI1/ATF2/S100A12/RPS6KA5	14

c)Functional enrichment analysis data of acquired genes in KIRC

	ID	Description	GeneRatio	BgRatio	pvalue	p.adjust	qvalue	geneID	Count
R-HSA-449147	R-HSA-449147	Signaling by Interleukins	90/964	463/10619	1.35E-12	1.70E-09	1.58E-09	JUNB/MAP2K3/DUSP6/JUN/FOS/IL1RL1/PELI1/IL1RN/OSM/IL1B/F13A1/S100A12/CSF3/BCL6/CCL3/IL4R/FPR1/CCL3L1/MSN/IL2RA/IL13RA2/TNFRSF1B/IL10/NOS2/IL13/CSF2RB/HCK/MYD88/CEBPD/SHC1/CCL19/IL7R/CRKL/IRAK3/CCL4/ALOX15/FCER2/OSMR/PSMD11/IL1R1/CSF3R/IL17RA/VIM/CSF1R/SERPINB2/HNRNPF/MAP3K8/PIK3R1/TGFB1/CD86/UBC/CSF1/CD4/STAT4/LYN/IL24/CD80/LMNB1/CCR1/PSMD1/CASP1/ITGAM/ATF2/FSCN1/CCL11/LCP1/INPP5D/FYN/PRTN3/IL1RAP/IL1RL2/RPS6KA5/CA1/CCL20/YWHAZ/HIF1A/PTPN12/PPP2R1B/TCP1/HSP90B1/PSMC6/S1PR1/RAPGEF1/IL1RAPL1/ZEB1/YES1/STAT5B/VEGFA/PSMD12/CXCL2	90
R-HSA-6783783	R-HSA-6783783	Interleukin-10 signaling	18/964	47/10619	5.39E-08	3.37E-05	3.15E-05	IL1RN/IL1B/CSF3/CCL3/FPR1/CCL3L1/TNFRSF1B/IL10/CCL19/CCL4/FCER2/IL1R1/CD86/CSF1/CD80/CCR1/CCL20/CXCL2	18
R-HSA-6785807	R-HSA-6785807	Interleukin-4 and Interleukin-13 signaling	26/964	108/10619	2.80E-06	9.17E-04	8.57E-04	JUNB/FOS/OSM/IL1B/F13A1/BCL6/IL4R/IL13RA2/TNFRSF1B/IL10/NOS2/IL13/CEBPD/ALOX15/FCER2/VIM/PIK3R1/TGFB1/ITGAM/FSCN1/CCL11/HIF1A/HSP90B1/S1PR1/ZEB1/VEGFA	26
R-HSA-72203	R-HSA-72203	Processing of Capped Intron-Containing Pre-mRNA	45/964	244/10619	2.93E-06	9.17E-04	8.57E-04	SF1/TRA2B/EIF4A3/PRPF4/HNRNPU/NUP98/NUP153/PTBP1/HNRNPM/NUP188/POLR2D/HNRNPR/ISY1/NUP62/PRPF38A/LUZP4/HNRNPF/SF3B3/RBM8A/HNRNPA3/DHX38/RBM17/WDR33/CPSF7/HNRNPH1/THOC5/FUS/SEH1L/NUP35/NXT1/FYTTD1/HNRNPK/NUP160/HNRNPC/SMNDC1/SNW1/PPIL4/DDX5/DDX42/RBMX/DHX15/NUP85/CWC22/HNRNPD/SF3B1	45
R-HSA-1169410	R-HSA-1169410	Antiviral mechanism by IFN-stimulated genes	20/964	80/10619	2.15E-05	5.37E-03	5.02E-03	EIF4A3/NUP98/NUP153/FLNA/MX2/EIF4G1/NUP188/OAS3/NUP62/USP18/KPNA2/UBC/SEH1L/NUP35/NUP160/ARIH1/ABCE1/NUP85/EIF4A1/KPNA5	20
R-HSA-512988	R-HSA-512988	Interleukin-3, Interleukin-5 and GM-CSF signaling	14/964	48/10619	5.98E-05	1.25E-02	1.17E-02	IL2RA/CSF2RB/HCK/SHC1/CRKL/PIK3R1/UBC/LYN/INPP5D/FYN/YWHAZ/RAPGEF1/YES1/STAT5B	14
R-HSA-72163	R-HSA-72163	mRNA Splicing - Major Pathway	33/964	183/10619	9.52E-05	1.70E-02	1.59E-02	SF1/TRA2B/EIF4A3/PRPF4/HNRNPU/PTBP1/HNRNPM/POLR2D/HNRNPR/ISY1/PRPF38A/HNRNPF/SF3B3/RBM8A/HNRNPA3/DHX38/RBM17/WDR33/CPSF7/HNRNPH1/FUS/HNRNPK/HNRNPC/SMNDC1/SNW1/PPIL4/DDX5/DDX42/RBMX/DHX15/CWC22/HNRNPD/SF3B1	33
R-HSA-6798695	R-HSA-6798695	Neutrophil degranulation	68/964	480/10619	1.26E-04	1.98E-02	1.85E-02	PNP/FPR2/CXCR2/S100A12/PLAUR/S100A9/CXCR1/PLEKHO2/FCGR3B/CRISPLD2/FPR1/MMP8/CLEC4D/S100A8/S100A7/SELL/FCGR2A/HK3/PYGL/TNFRSF1B/CLEC4C/CEACAM3/CR1/CD14/ADAM8/SLC15A4/RNASE2/TMEM173/STK10/SLC11A1/MMP25/P2RX1/PSMD11/FCN1/NFAM1/IDH1/FCAR/C3AR1/LAIR1/SIGLEC5/GPR84/COTL1/VCL/MNDA/SIRPB1/CFP/CD53/SIGLEC9/PSMD1/ITGAM/CD93/MPO/CYFIP1/KCMF1/CD33/CYBB/PPBP/CKAP4/PRTN3/ORM1/PLAC8/CCT2/HBB/ACTR10/PSEN1/VAPA/PGLYRP1/PSMD12	68

R-HSA-1169408	R-HSA-1169408	ISG15 antiviral mechanism	17/964	72/10619	1.87E-04	2.52E-02	2.36E-02	EIF4A3/NUP98/NUP153/MX2/EIF4G1/NUP188/NUP62/USP18/KPNA2/UBC/SEH1L/NUP35/NUP160/ARIH1/NUP85/EIF4A1/KPNA5	17
R-HSA-72172	R-HSA-72172	mRNA Splicing	33/964	191/10619	2.20E-04	2.52E-02	2.36E-02	SF1/TRA2B/EIF4A3/PRPF4/HNRNPU/PTBP1/HNRNPM/POLR2D/HNRNPR/ISY1/PRPF38A/HNRNPF/SF3B3/RBM8A/HNRNPA3/DHX38/RBM17/WDR33/CPSF7/HNRNPH1/FUS/HNRNPK/HNRNPC/SMNDC1/SNW1/PPIL4/DDX5/DDX42/RBMX/DHX15/CWC22/HNRNPD/SF3B1	33

Table S10 a)Functional enrichment analysis data of lost genes in KICH, [KICH=Kidney chromophobe]

	ID	Description	GeneRatio	BgRatio	pvalue	p.adjust	qvalue	geneID	Count
R-HSA-1474244	R-HSA-1474244	Extracellular matrix organization	135/2260	301/10619	1.41E-20	1.92E-17	1.65E-17	BCAN/GDF5/COL6A3/TPSAB1/COL17A1/EMILIN1/EFEMP1/ITGA7/PDGFA/LAMA3/ITGA2/SDC3/BMP1/CD44/FGA/PLOD3/ADAMTS8/COL18A1/CTSG/EFEMP2/CTSD/ADAM17/ITGB1/CAPN11/SCUBE1/ADAM8/LAMC2/MMP2/COL6A1/LAMA2/COL4A2/MMP14/ITGAM/MADCAM1/ADAM9/NID1/ITGB6/ITGB8/PSEN1/COL8A1/COL23A1/ITGAX/COMP/BMP2/MMP25/MMP9/ADAMTS9/SERPINH1/TGFB3/TIMP1/COL13A1/SDC4/ADAMTS1/MMP19/SERPINE1/ADAMTS4/OPTC/COL22A1/CTSK/KDR/ITGA1/COL2A1/COL7A1/MMP10/COL16A1/DMP1/CTSS/NTN4/TLL2/COL11A1/JAM3/PDGFB/ITGA11/TGFB2/CAPN5/ITGA9/MMP1/ITGA5/MFAP4/ITGA4/A2M/FBN1/ICAM1/LOX/SPARC/ADAM10/COL5A3/TNXB/ADAM15/LOXL3/DDR2/MFAP5/LUM/VCAM1/IBSP/NID2/CAPN14/LTBP1/EMILIN2/ASPN/CAPN8/LAMA4/FN1/THBS1/COL10A1/COL12A1/COL6A2/FBLN5/LTBP4/FMOD/SH3PXD2A/HSPG2/PXDN/COL14A1/TNC/ELN/COL4A1/VCAN/ACTN1/COL1A1/COL3A1/COL1A2/ADAM12/COL5A2/MFAP2/COL5A1/CASP3/COL15A1/FBN2/MMP7/ADAMTS2/LOXL2/ADAMTS14/MMP11/ADAM19	135
R-HSA-198933	R-HSA-198933	Immunoregulatory interactions between a Lymphoid and a non-Lymphoid cell	76/2260	132/10619	6.98E-20	4.75E-17	4.09E-17	B2M/LAIR1/NPDC1/TREM2/CD247/HLA-F/CD99/COL17A1/SELL/TREML4/CD8A/TREML1/MICB/C3/LILRA1/LILRB1/HCST/TYROBP/TREML2/IFITM1/ITGB1/SIGLEC9/FCGR1A/CD300LF/CD300A/SIGLEC5/LILRA4/HLA-E/CD300LB/SIGLEC10/MADCAM1/LILRB4/HLA-B/OSCAR/PVR/CD81/KIR2DL4/CD300E/CD3D/SLAMF7/SH2D1A/SLAMF6/COL2A1/CD226/KIR3DL2/LILRA5/RAET1E/CD34/CD200/ITGA4/ICAM1/LILRA2/CD1C/HLA-G/CRTAM/SIGLEC7/CD1A/TREM1/CD33/PILRA/SIGLEC1/VCAM1/CD200R1/CD19/SIGLEC6/CD300C/LILRB5/COL1A1/COL3A1/COL1A2/KLRK1/CLEC2D/CD22/KIR2DL1/KLRB1/SIGLEC8	76

R-HSA-449147	R-HSA-449147	Signaling by Interleukins	178/2260	463/10619	5.47E-18	2.48E-15	2.14E-15	CRLF2/NOS2/CSF1R/CBL/JAK3/CSF2/PSMD7/SOD2/PSMB2/IL6ST/TXLNA/STAT6/IL22RA2/IL10RA/PSMD11/IL32/IL27RA/STAT5A/CRLF1/CAPZA1/CXCL10/IL13RA1/HCK/PTAFR/CTSG/STAT1/FSCN1/ITGB1/MYD88/RAP1B/CXCL2/HSPA8/FOXO3/BATF/MMP2/PPP2R1A/PSMD12/IL31RA/CSF2RA/MAPK7/YES1/IL6R/PTPN9/CCL20/ITGAM/IL7R/IL18BP/CSF1/PPP2R1B/NKIRAS2/IL18RAP/HSP90B1/IL1RL1/CRK/S100A12/IL18R1/IRAK2/OSMR/CFL1/EBI3/FOXO1/TP53/IRAK3/RIPK2/UBC/CXCL1/YWHAZ/FPR1/NFKB1/ITGAX/GAB2/PIM1/CSF3R/OSM/IL24/CEBPD/MMP9/ELK1/CCL22/IL1B/TIMP1/STAT3/RELA/MAP2K3/PSME4/CDKN1A/CCL2/MAPKAPK2/SHC1/SQSTM1/MSN/IL1R1/RHOU/NFKB2/SOCS3/CSF3/TNFRSF1A/CNN2/MCL1/LIF/CLCF1/FASLG/CD86/RPS27A/CCL3/NFKBIA/PELI2/MEF2A/HIF1A/CCR2/CD4/MEF2C/HMOX1/IL10/NDN/DUSP4/IL33/LMNB1/PTPN12/CCR5/ANXA1/IRF4/SERPINB2/LCK/IL6/HGF/FYN/FLT3/LYN/PTPN7/MMP1/RPS6KA5/TSLP/IL23A/IL2RG/IL11/CD80/S1PR1/IL1RAP/LCP1/STAT4/CASP1/ICAM1/CCR1/RORA/CCL11/HAVCR2/IL1A/TNFRSF1B/VAV1/IL4R/VIM/DUSP7/IL16/CSF2RB/IL1RN/IL34/IL5RA/PTPN2/IL17F/VCAM1/IL22RA1/IL21R/IL2RA/FN1/S100B/IL10RB/PIK3CD/COL1A2/AGER/IKBKB/CASP3/NANOG/IL2RB/MTAP/HIST1H3F/IL18/IL23R	178
R-HSA-6785807	R-HSA-6785807	Interleukin-4 and Interleukin-13 signaling	58/2260	108/10619	1.15E-13	3.90E-11	3.36E-11	NOS2/JAK3/STAT6/IL13RA1/STAT1/FSCN1/ITGB1/HSPA8/FOXO3/BATF/MMP2/IL6R/ITGAM/HSP90B1/FOXO1/TP53/ITGAX/PIM1/OSM/CEBPD/MMP9/CCL22/IL1B/TIMP1/STAT3/CDKN1A/CCL2/RHOU/SOCS3/MCL1/LIF/FASLG/HIF1A/HMOX1/IL10/NDN/ANXA1/IRF4/IL6/HGF/MMP1/IL23A/IL2RG/S1PR1/ICAM1/RORA/CCL11/IL1A/TNFRSF1B/IL4R/VIM/IL17F/VCAM1/FN1/COL1A2/NANOG/IL18/IL23R	58
R-HSA-1474228	R-HSA-1474228	Degradation of the extracellular matrix	69/2260	140/10619	1.48E-13	4.03E-11	3.47E-11	BCAN/COL6A3/TPSAB1/COL17A1/LAMA3/BMP1/CD44/ADAMTS8/COL18A1/CTSG/CTSD/ADAM17/CAPN11/SCUBE1/ADAM8/LAMC2/MMP2/COL6A1/COL4A2/MMP14/ADAM9/NID1/PSEN1/COL8A1/COL23A1/MMP25/MMP9/ADAMTS9/TIMP1/COL13A1/ADAMTS1/MMP19/ADAMTS4/OPTC/CTSK/COL2A1/COL7A1/MMP10/COL16A1/CTSS/TLL2/COL11A1/CAPN5/MMP1/A2M/FBN1/ADAM10/COL5A3/ADAM15/CAPN14/CAPN8/FN1/COL10A1/COL12A1/COL6A2/HSPG2/COL14A1/ELN/COL4A1/COL1A1/COL3A1/COL1A2/COL5A2/COL5A1/CASP3/COL15A1/FBN2/MMP7/MMP11	69
R-HSA-6798695	R-HSA-6798695	Neutrophil degranulation	169/2260	480/10619	4.42E-13	1.00E-10	8.65E-11	B2M/LAIR1/IMPDH2/CXCR2/BIN2/PSMD7/DOK3/TRPM2/PA2G4/MVP/ATP6V0A1/CKAP4/SELL/IMPDH1/MAN2B1/DYNC1LI1/PIGR/RNASE2/ORM2/PSMD11/CLEC4C/DYNLL1/QSOX1/FCGR3B/TXNDC5/C3/GMFG/MNDA/CD44/TYROBP/FCN1/PTAFR/CTSG/S100P/CLEC5A/CTSD/NCKAP1L/CYBA/SIGLEC9/RAP1B/HSPA8/ADAM8/UNC13D/CXCR1/PGLYRP1/SERPINB1/KPNB1/CD300A/RETN/SIGLEC5/FCGR2A/HPSE/PSMD12/APAF1/CST3/DOCK2/CD63/NFAM1/DIAPH1/S100A9/LTF/ARPC5/CYB5R3/FCER1G/RAB31/ORM1/KCMF1/S100A8/SERPINA3/HGSNAT/DEFA1B/ITGAM/NRAS/TLR2/CEACAM3/CFP/VCL/ARG1/GPR84/TMEM173/S100A12/FCAR/PSEN1/CTSZ/CLEC4D/HLA-B/LRG1/B4GALT1/CXCL1/TNFAIP6/FPR1/NFKB1/ITGAX/DEGS1/OSCAR/HP/FAF2/MMP25/MMP9/PGM2/RHOG/CRISPLD2/ARL8A/HSP90AB1/HSPA6/C5AR1/RAP2B/SLC2A3/PLAUR/CNN2/EEF1A1/FPR2/PLAC8/FGR/STOM/CD14/ARHGAP9/SLC27A2/HK3/HVCN1/CTSS/AZU1/CYBB/ARSB/PTX3/DDX3X/CD93/SIRPA/CD53/TUBB/CR1/CANT1/CTSA/ADAM10/TNFRSF1B/ANPEP/ALDH3B1/PRG2/RAB7A/CD68/CYFIP1/TMC6/NFASC/CD33/GRN/SLC11A1/DEFA4/SLC2A5/KRT1/PLEKHO2/SIGLEC14/RAB27A/FABP5/P2RX1/GSN/LYZ/ARMC8/GOLGA7/PLAU/MS4A3/BST1/PADI2/PKP1/GGH/MGST1/GLIPR1/DSC1/PAFAH1B2/SURF4	169
R-HSA-6783783	R-HSA-6783783	Interleukin-10 signaling	32/2260	47/10619	6.49E-12	1.26E-09	1.09E-09	CSF2/IL10RA/CXCL10/PTAFR/CXCL2/CCL20/CSF1/CXCL1/FPR1/CCL22/IL1B/TIMP1/STAT3/CCL2/IL1R1/CSF3/TNFRSF1A/LIF/CD86/CCL3/CCR2/IL10/CCR5/IL6/CD80/ICAM1/CCR1/IL1A/TNFRSF1B/IL1RN/IL10RB/IL18	32

R-HSA-216083	R-HSA-216083	Integrin cell surface interactions	46/2260	85/10619	2.82E-11	4.80E-09	4.13E-09	COL6A3/ITGA7/ITGA2/CD44/FGA/COL18A1/ITGB1/COL6A1/COL4A2/ITGAM/MADCAM1/ITGB6/ITGB8/COL8A1/COL23A1/ITGAX/COMP/COL13A1/KDR/ITGA1/COL2A1/COL7A1/COL16A1/JAM3/ITGA11/ITGA9/ITGA5/ITGA4/FBN1/ICAM1/COL5A3/LUM/VCAM1/IBSP/FN1/THBS1/COL10A1/COL6A2/HSPG2/TNC/COL4A1/COL1A1/COL3A1/COL1A2/COL5A2/COL5A1	46
R-HSA-1442490	R-HSA-1442490	Collagen degradation	37/2260	64/10619	1.92E-10	2.91E-08	2.51E-08	COL6A3/COL17A1/COL18A1/CTSD/ADAM17/MMP2/COL6A1/COL4A2/MMP14/ADAM9/COL8A1/COL23A1/MMP9/COL13A1/MMP19/CTSK/COL2A1/COL7A1/MMP10/COL16A1/COL11A1/MMP1/ADAM10/COL5A3/COL10A1/COL12A1/COL6A2/COL14A1/COL4A1/COL1A1/COL3A1/COL1A2/COL5A2/COL5A1/COL15A1/MMP7/MMP11	37
R-HSA-877300	R-HSA-877300	Interferon gamma signaling	46/2260	92/10619	9.30E-10	1.27E-07	1.09E-07	B2M/GBP5/TRIM21/IRF6/IRF7/HLA-F/GBP2/CD44/PTAFR/STAT1/IRF9/FCGR1A/HLA-DPB1/IFNGR2/HLA-E/HLA-DQB1/TRIM26/FCGR1B/MID1/IFNGR1/HLA-B/TRIM38/OASL/MT2A/SOCS3/PTPN1/OAS3/HLA-DRA/TRIM17/GBP4/TRIM34/GBP7/IRF4/OAS2/TRIM29/TRIM22/IRF1/HLA-DRB1/HLA-DQA1/IRF8/ICAM1/HLA-G/PTPN2/VCAM1/GBP1/CIITA	46

b)Functional enrichment analysis data of conserved genes in KICH

	ID	Description	GeneRatio	BgRatio	pvalue	p.adjust	qvalue	geneID	Count
R-HSA-1474244	R-HSA-1474244	Extracellular matrix organization	43/364	301/10619	8.7524E-16	6.9144E-13	6.34E-13	PXDN/SPARC/MMP10/ITGA1/VCAN/COL5A2/JAM3/COL16A1/COL1A1/ADAMTS2/FBLN5/COL5A1/COL15A1/ADAM12/COL7A1/COL6A2/EMILIN2/ACTN1/ADAM19/DMP1/PDGFB/VCAM1/ITGA5/COL4A1/ICAM1/THBS1/COL10A1/LOXL2/LAMA4/FMOD/NID2/COL3A1/TNXB/FN1/TNC/LOXL3/ADAMTS4/LAMC2/MFAP2/MMP11/FBN2/MMP7/TLL2	43
R-HSA-2022090	R-HSA-2022090	Assembly of collagen fibrils and other multimeric structures	15/364	61/10619	1.3351E-09	5.2738E-07	4.83E-07	PXDN/COL5A2/COL1A1/COL5A1/COL15A1/COL7A1/COL6A2/COL4A1/COL10A1/LOXL2/COL3A1/LOXL3/LAMC2/MMP7/TLL2	15
R-HSA-216083	R-HSA-216083	Integrin cell surface interactions	17/364	85/10619	3.2632E-09	8.593E-07	7.88E-07	ITGA1/COL5A2/JAM3/COL16A1/COL1A1/COL5A1/COL7A1/COL6A2/VCAM1/ITGA5/COL4A1/ICAM1/THBS1/COL10A1/COL3A1/FN1/TNC	17
R-HSA-1474290	R-HSA-1474290	Collagen formation	17/364	90/10619	8.1627E-09	1.6121E-06	1.48E-06	PXDN/COL5A2/COL16A1/COL1A1/ADAMTS2/COL5A1/COL15A1/COL7A1/COL6A2/COL4A1/COL10A1/LOXL2/COL3A1/LOXL3/LAMC2/MMP7/TLL2	17
R-HSA-3000171	R-HSA-3000171	Non-integrin membrane-ECM interactions	13/364	59/10619	7.0649E-08	1.1163E-05	1.02E-05	COL5A2/COL1A1/COL5A1/ACTN1/PDGFB/COL4A1/THBS1/COL10A1/LAMA4/COL3A1/FN1/TNC/LAMC2	13
R-HSA-1442490	R-HSA-1442490	Collagen degradation	13/364	64/10619	1.9631E-07	2.5848E-05	2.37E-05	MMP10/COL5A2/COL16A1/COL1A1/COL5A1/COL15A1/COL7A1/COL6A2/COL4A1/COL10A1/COL3A1/MMP11/MMP7	13

R-HSA-3000178	R-HSA-3000178	ECM proteoglycans	14/364	76/10619	2.4011E-07	2.7098E-05	2.48E-05	SPARC/VCAN/COL5A2/COL1A1/COL5A1/COL6A2/DMP1/COL4A1/LAMA4/FMOD/COL3A1/TNXB/FN1/TNC	14
R-HSA-198933	R-HSA-198933	Immunoregulatory interactions between a Lymphoid and a non-Lymphoid cell	18/364	132/10619	5.417E-07	5.3493E-05	4.9E-05	SLAMF6/CD1A/COL1A1/SIGLEC1/CD226/LILRB5/CD300E/LILRA5/VCAM1/TREM1/ICAM1/COL3A1/CD1C/CRTAM/SIGLEC7/CD19/CD200R1/NCR1	18
R-HSA-446353	R-HSA-446353	Cell-extracellular matrix interactions	7/364	18/10619	1.2053E-06	0.00010327	9.47E-05	FBLIM1/FLNC/ACTN1/ACTG1/FLNA/ACTB/PARVA	7
R-HSA-1474228	R-HSA-1474228	Degradation of the extracellular matrix	18/364	140/10619	1.3072E-06	0.00010327	9.47E-05	MMP10/COL5A2/COL16A1/COL1A1/COL5A1/COL15A1/COL7A1/COL6A2/COL4A1/COL10A1/COL3A1/FN1/ADAMTS4/LAMC2/MMP11/FBN2/MMP7/TLL2	18

c)Functional enrichment analysis data of acquired genes in KICH

	ID	Description	GeneRatio	BgRatio	pvalue	p.adjust	qvalue	geneID	Count
R-HSA-1474244	R-HSA-1474244	Extracellular matrix organization	77/1388	301/10619	2.2963E-09	2.9943E-06	2.8E-06	OPTC/COL22A1/COL10A1/CTSK/KDR/LOXL2/LAMA4/COL2A1/FMOD/CTSS/NID2/NTN4/TLL2/COL11A1/COL3A1/ITGA11/TNXB/TGFB2/CAPN5/ITGA9/MMP1/FN1/MFAP4/TNC/LOXL3/ITGA4/A2M/FBN1/LOX/NID1/ADAM10/PDGFA/ADAMTS4/COL5A3/ITGAM/LAMC2/MFAP2/MMP11/ADAM15/COL13A1/CTSG/DDR2/MFAP5/FBN2/LUM/EMILIN1/IBSP/ITGA7/SERPINH1/TPSAB1/CAPN14/LTBP1/SERPINE1/ASPN/MMP7/CAPN8/COL12A1/LTBP4/TIMP1/CD44/COL6A3/SH3PXD2A/HSPG2/COL14A1/ELN/MMP14/COL4A2/COL1A2/BMP2/CASP3/MMP2/COL23A1/ITGB6/ADAMTS14/ADAMTS9/LTBP3/ITGB3	77
R-HSA-156842	R-HSA-156842	Eukaryotic Translation Elongation	34/1388	93/10619	7.1824E-09	4.6829E-06	4.39E-06	EEF1A1/RPS12/RPS16/RPL41/RPS27A/RPL22/RPL26/RPL30/RPS27/RPL7/RPL22L1/RPS13/EEF1A2/EEF1G/RPL13A/RPL3/RPL4/RPS18/RPS3/RPS9/RPL6/RPS20/RPS26/RPS29/RPS2/RPS4X/RPS5/EEF1B2/RPL10A/RPL18A/RPL37A/RPLP0/RPS25/RPS3A	34

R-HSA-156902	R-HSA-156902	Peptide chain elongation	31/1388	89/10619	1.1956E-07	5.197E-05	4.87E-05	EEF1A1/RPS12/RPS16/RPL41/RPS27A/RPL22/RPL26/RPL30/RPS27/RPL7/RPL22L1/RPS13/RPL13A/RPL3/RPL4/RPS18/RPS3/RPS9/RPL6/RPS20/RPS26/RPS29/RPS2/RPS4X/RPS5/RPL10A/RPL18A/RPL37A/RPLP0/RPS25/RPS3A	31
R-HSA-975956	R-HSA-975956	Nonsense Mediated Decay (NMD) independent of the Exon Junction Complex (EJC)	32/1388	95/10619	1.8272E-07	5.9565E-05	5.58E-05	RPS12/RPS16/RPL41/RPS27A/RPL22/RPL26/PABPC1/RPL30/RPS27/RPL7/NCBP2/RPL22L1/RPS13/RPL13A/RPL3/RPL4/RPS18/RPS3/RPS9/RPL6/RPS20/RPS26/RPS29/RPS2/RPS4X/RPS5/RPL10A/RPL18A/RPL37A/RPLP0/RPS25/RPS3A	32
R-HSA-1474228	R-HSA-1474228	Degradation of the extracellular matrix	41/1388	140/10619	2.9768E-07	7.7634E-05	7.27E-05	OPTC/COL10A1/CTSK/COL2A1/CTSS/TLL2/COL11A1/COL3A1/CAPN5/MMP1/FN1/A2M/FBN1/NID1/ADAM10/ADAMTS4/COL5A3/LAMC2/MMP11/ADAM15/COL13A1/CTSG/FBN2/TPSAB1/CAPN14/MMP7/CAPN8/COL12A1/TIMP1/CD44/COL6A3/HSPG2/COL14A1/ELN/MMP14/COL4A2/COL1A2/CASP3/MMP2/COL23A1/ADAMTS9	41
R-HSA-192823	R-HSA-192823	Viral mRNA Translation	30/1388	89/10619	4.3201E-07	8.3544E-05	7.82E-05	RPS12/RPS16/RPL41/RPS27A/RPL22/RPL26/RPL30/RPS27/RPL7/RPL22L1/RPS13/RPL13A/RPL3/RPL4/RPS18/RPS3/RPS9/RPL6/RPS20/RPS26/RPS29/RPS2/RPS4X/RPS5/RPL10A/RPL18A/RPL37A/RPLP0/RPS25/RPS3A	30
R-HSA-198933	R-HSA-198933	Immunoregulatory interactions between a Lymphoid and a non-Lymphoid cell	39/1388	132/10619	4.4847E-07	8.3544E-05	7.82E-05	CD3D/SLAMF7/SH2D1A/COL2A1/KIR3DL2/COL3A1/CD1C/CRTAM/RAET1E/SIGLEC7/CD19/CD200R1/CD34/CD200/ITGA4/LILRA2/NPDC1/LAIR1/HLA-G/SIGLEC5/CD8A/TREML2/CD33/PILRA/SIGLEC6/CD300C/TREML4/COL1A2/KLRK1/LILRA4/CD247/CLEC2D/CD22/KIR2DL1/KLRB1/SIGLEC8/NCR1/CD300E/CD96	39
R-HSA-927802	R-HSA-927802	Nonsense-Mediated Decay (NMD)	35/1388	115/10619	7.9882E-07	0.0001142	0.000107	RPS12/RPS16/RPL41/RPS27A/RPL22/RPL26/PABPC1/RPL30/RPS27/RPL7/DCP1A/NCBP2/RPL22L1/RPS13/PPP2R2A/UPF3B/RPL13A/RPL3/RPL4/RPS18/RPS3/RPS9/RPL6/RPS20/RPS26/RPS29/RPS2/RPS4X/RPS5/RPL10A/RPL18A/RPL37A/RPLP0/RPS25/RPS3A	35
R-HSA-975957	R-HSA-975957	Nonsense Mediated Decay (NMD) enhanced by the Exon Junction Complex (EJC)	35/1388	115/10619	7.9882E-07	0.0001142	0.000107	RPS12/RPS16/RPL41/RPS27A/RPL22/RPL26/PABPC1/RPL30/RPS27/RPL7/DCP1A/NCBP2/RPL22L1/RPS13/PPP2R2A/UPF3B/RPL13A/RPL3/RPL4/RPS18/RPS3/RPS9/RPL6/RPS20/RPS26/RPS29/RPS2/RPS4X/RPS5/RPL10A/RPL18A/RPL37A/RPLP0/RPS25/RPS3A	35
R-HSA-72689	R-HSA-72689	Formation of a pool of free 40S subunits	32/1388	101/10619	8.758E-07	0.0001142	0.000107	RPS12/RPS16/RPL41/RPS27A/RPL22/RPL26/EIF3E/EIF3H/RPL30/RPS27/RPL7/RPL22L1/RPS13/RPL13A/RPL3/RPL4/RPS18/RPS3/RPS9/RPL6/RPS20/RPS26/RPS29/RPS2/RPS4X/RPS5/RPL10A/RPL18A/RPL37A/RPLP0/RPS25/RPS3A	32

Table S11 a) Functional enrichment analysis data of lost genes in KIRP, [KIRP= Kidney renal papillary cell carcinoma]

	ID	Description	GeneRatio	BgRatio	pvalue	p.adjust	qvalue	geneID	Count
R-HSA-198933	R-HSA-198933	Immunoregulatory interactions between a Lymphoid and a non-Lymphoid cell	90/2540	132/10619	3.24E-27	4.48E-24	4.04E-24	ICAM1/CD300E/LILRA5/CD300LB/SIGLEC5/COL1A2/LILRA4/LILRA3/CD96/SELL/COL3A1/KLRD1/CD300LF/SLAMF6/CD300C/CRTAM/SIGLEC6/CD247/COL1A1/TREML2/LILRB4/CD3G/FCGR3A/ITGB1/KIR2DL3/CD8A/FCGR1A/SH2D1A/ITGA4/KLRB1/CD8B/SIGLEC9/CD200R1/LILRB5/LILRB1/CD1A/ULBP1/VCAM1/CD33/CD40LG/FCGR2B/COLEC12/SLAMF7/SIGLEC7/CD3D/CD3E/ICAM4/ITGB2/KLRK1/SIGLEC8/IFITM1/KIR3DL2/LAIR1/KLRC1/TREM1/CD22/SIGLEC10/HCST/ITGAL/NCR1/PVR/TYROBP/HLA-E/SIGLEC1/HLA-A/CD1C/CD1D/LILRA2/CD300A/CLEC2B/CD200/NCR3/LILRA1/B2M/MICB/CD19/OSCAR/LAIR2/KLRG1/CD99/HLA-F/TREML4/CD226/ICAM3/HLA-B/ULBP3/CD1B/KIR3DL1/C3/ICAM5	90
R-HSA-449147	R-HSA-449147	Signaling by Interleukins	194/2540	463/10619	1.85E-18	1.28E-15	1.15E-15	CCL2/IL1R1/SOCS3/IL24/ICAM1/RHOU/TNFRSF1A/CSF1/IL6/MCL1/PIM1/LIF/LMNB1/CLCF1/CSF3/SHC1/CNN2/COL1A2/STAT3/MMP2/IL13RA1/CSF2RB/OSMR/IL7R/SERPINB2/CD86/CSF3R/OSM/IL2RA/FPR1/ANXA1/TNFRSF1B/TEC/LCP1/IL4R/IL13/CDKN1A/IL7/VIM/JAK1/CCL5/ITGB1/HCK/IL10RA/JUNB/TIMP1/IFNG/FCER2/MSN/MYD88/SOCS5/JAK3/NOD2/VCAM1/MAP3K7/CTSG/TWIST1/CD4/IL2RB/IL18BP/IRF4/SOD2/IL34/RAP1B/RELA/HGF/ITGB2/ALOX15/F13A1/ITGAM/IL18RAP/IL27RA/VAV1/IL16/HSP90B1/IRAK3/TXLNA/IL10/IL2RG/NFKB2/IRAK2/LYN/YWHAZ/IL21R/S100B/INPP5D/GAB2/STAT4/FOXO3/STAT1/UBC/PTGS2/IL1B/ITGAX/CCR2/CCR5/CAPZA1/BATF/IL9R/NFKB1/S100A12/IL33/PTPN12/PTPN2/IL11/CXCL10/CSF1R/CBL/HNRNPF/CSF2RA/CASP3/BCL6/CCL19/PIK3CD/CSF2/PSME4/IL17RA/CXCL1/EBI3/CD80/CASP1/ALOX5/IL4/CCL4/HIF1A/LCK/CCL11/IL18R1/CNTFR/CCL20/SOCS1/IL31RA/MAPKAPK2/CRLF1/LGALS9/SAA1/IL1A/TNIP2/STAT6/PTPN7/IL32/STAT2/FASLG/MEF2C/IL23A/CFL1/CEBPD/IL12B/JUN/TP53/CRK/IL15RA/PSMB9/HAVCR2/PELI1/IL12RB1/PTPN6/IL12RB2/PSMB10/MAP3K8/IL22RA2/MAPK7/IL6ST/HSPA8/TCP1/IRAK1/VRK3/SQSTM1/PSMD10/PSMD7/PSMB2/CISH/RAG2/PSMD11/PSMB5/CXCL2/MAPK8/HMOX1/IL13RA2/TRAF6/HNRNPA2B1/NKIRAS1/TAB2/PSMA1/RIPK2/PIK3CA/PSMD2/PSMA3/HSPA9/PPP2R5D/IL20RB/IL12A/ZEB1/IL1F10	194
R-HSA-1474244	R-HSA-1474244	Extracellular matrix organization	130/2540	301/10619	6.79E-14	2.56E-11	2.30E-11	THBS1/ICAM1/ADAMTS1/MMP19/ADAMTS4/SERPINH1/MMP25/ADAMTS2/SERPINE1/GDF5/ADAM12/COL1A2/COL16A1/MMP2/FBN1/COL4A1/LUM/COL10A1/LAMA4/COL3A1/COL6A3/TIMP2/DCN/MUSK/EMILIN2/COL15A1/CD44/COL5A1/ADAMTS3/COL8A1/COL1A1/ITGB1/ADAM19/ITGA4/CTSS/TNC/TIMP1/TGFB3/COL5A2/SPARC/NID2/LAMA2/COL11A1/PCOLCE/VCAM1/VWF/CTSG/ITGB3/NID1/EMILIN1/MMP11/VCAN/PXDN/ICAM4/ITGB2/ITGAM/BMP1/FMOD/COL4A2/BMP2/ITGAD/DMP1/ITGA5/MATN3/ITGAL/TPSAB1/CMA1/ELANE/DDR2/ITGAX/A2M/ACTN1/TNXB/MFAP4/LOXL1/EFEMP1/LOXL3/TGFB2/CASP3/CEACAM8/MMP17/SH3PXD2A/COL6A2/LTBP2/SDC3/COL14A1/ITGA1/SDC4/MFAP2/CAPN11/ITGA9/MMP10/COL21A1/LOXL2/ADAM17/ITGB8/COL8A2/ADAM9/ITGAV/COMP/COL12A1/ADAM8/ACAN/ADAMTS16/LTBP4/ICAM3/ITGA11/ASPN/MMP7/PDGFA/FBLN1/LAMC3/SPP1/EFEMP2/BGN/ITGA2/CRTAP/P4HA3/COL25A1/MMP14/LOX/PSEN1/COL22A1/LAMA1/COL4A3/ICAM5/COL27A1/KLK2/COL9A3/ADAMTS8	130

R-HSA-6785807	R-HSA-6785807	Interleukin-4 and Interleukin-13 signaling	62/2540	108/10619	7.41E-14	2.56E-11	2.30E-11	CCL2/SOCS3/ICAM1/RHOU/IL6/MCL1/PIM1/LIF/COL1A2/STAT3/MMP2/IL13RA1/OSM/ANXA1/TNFRSF1B/IL4R/IL13/CDKN1A/VIM/JAK1/ITGB1/JUNB/TIMP1/FCER2/SOCS5/JAK3/VCAM1/TWIST1/IRF4/HGF/ITGB2/ALOX15/F13A1/ITGAM/HSP90B1/IL10/IL2RG/FOXO3/STAT1/PTGS2/IL1B/ITGAX/BATF/BCL6/ALOX5/IL4/HIF1A/CCL11/SOCS1/SAA1/IL1A/STAT6/FASLG/IL23A/CEBPD/IL12B/TP53/HSPA8/HMOX1/IL13RA2/IL12A/ZEB1	62
R-HSA-6783783	R-HSA-6783783	Interleukin-10 signaling	33/2540	47/10619	2.38E-11	6.43E-09	5.79E-09	CCL2/IL1R1/ICAM1/TNFRSF1A/CSF1/IL6/LIF/CSF3/STAT3/CD86/FPR1/TNFRSF1B/JAK1/CCL5/IL10RA/TIMP1/FCER2/IL10/PTGS2/IL1B/CCR2/CCR5/CXCL10/CCL19/CSF2/CXCL1/CD80/CCL4/CCL20/IL1A/IL12B/CXCL2/IL12A	33
R-HSA-6798695	R-HSA-6798695	Neutrophil degranulation	178/2540	480/10619	2.79E-11	6.43E-09	5.79E-09	PLEKHO2/PLAUR/SLC2A3/SERPINB1/RAP2B/PGM2/MMP25/CLEC12A/CD93/HP/CNN2/GLIPR1/SIGLEC5/LILRA3/CLEC5A/C5AR1/RNASE2/KRT1/LYZ/SELL/S100A9/CLEC4C/TIMP2/CRISPLD2/PTPRC/FPR1/LRG1/FGR/CD44/TNFRSF1B/CFP/FCN1/PLAC8/DOCK2/RNASE3/CD53/FCGR2A/CTSS/CEACAM3/CYBB/GPR84/SIGLEC9/NCKAP1L/MS4A3/CD33/CTSG/CXCR2/VCL/STK10/MNDA/RAP1B/PRG2/ARPC5/TXNDC5/NFAM1/ITGB2/TMEM173/SIRPB1/ITGAM/FCAR/LAIR1/SIGLEC14/HK3/CD14/ITGAL/PYGL/TLR2/GMFG/TYROBP/DEGS1/FCER1G/ELANE/CXCR1/ITGAX/FPR2/NFKB1/S100A12/UNC13D/C3AR1/KCMF1/CD300A/CTSZ/B4GALT1/CD177/PRG3/TARM1/CLEC4D/RHOG/CEACAM8/APAF1/B2M/S100A8/CXCL1/ARHGAP9/IMPDH2/NRAS/ALOX5/DOK3/PGLYRP1/RAB31/ACTR2/OSCAR/TRPM2/SERPINA3/FCGR3B/BIN2/MAN2B1/TBC1D10C/ROCK1/MPO/HVCN1/ITGAV/RAB9B/GM2A/ADAM8/PIGR/HLA-B/HSPA6/XRCC5/CRISP3/CAP1/NEU1/TCN1/SIRPA/ARG1/CFD/PTPN6/HSPA1B/IQGAP1/DNAJC3/PTX3/CYFIP1/GDI2/CCT2/HSPA8/HSP90AB1/EEF2/VCP/EEF1A1/SLC15A4/PGAM1/RAB5C/ILF2/ANPEP/SLC27A2/CPPED1/PLD1/FTL/RAB7A/PSMD7/FLG2/PSMD11/C3/ACTR10/MGST1/STBD1/GAA/MANBA/TUBB/HBB/PSEN1/DYNC1LI1/HEXB/FTH1/CTSA/LTA4H/C6orf120/GPI/GLB1/PSMD2/NHLRC3/VAPA/DNAJC13/GRN/ENPP4/GCA/FOLR3/BPI	178
R-HSA-380108	R-HSA-380108	Chemokine receptors bind chemokines	32/2540	48/10619	3.96E-10	7.82E-08	7.04E-08	CXCR4/CXCR6/XCR1/CCL5/CCL21/CCR3/CXCR2/CXCL11/CXCR1/CCR2/CCL13/CCR5/CXCL6/CXCR3/CXCL10/CCR6/CCR4/CCR7/CCL19/CXCR5/CXCL1/CCL20/XCL1/CXCL9/CXCL16/CXCL13/XCL2/PF4/CCR8/CCR9/CXCL2/CXCL3	32
R-HSA-877300	R-HSA-877300	Interferon gamma signaling	47/2540	92/10619	1.39E-08	2.39E-06	2.15E-06	SOCS3/GBP1/ICAM1/IRF8/PTPN1/IRF1/OASL/FCGR1B/CD44/GBP2/JAK1/FCGR1A/IFNG/GBP5/IFNGR2/IRF9/TRIM22/HLA-DRA/VCAM1/IRF4/TRIM38/IRF7/HLA-DQA2/IFNGR1/HLA-E/OAS3/STAT1/HLA-A/HLA-DPB1/HLA-DPA1/PTPN2/MID1/B2M/CIITA/TRIM62/SOCS1/PML/CAMK2D/HLA-F/OAS2/HLA-B/HLA-DQA1/PTPN6/TRIM35/TRIM45/MT2A/TRIM3	47
R-HSA-216083	R-HSA-216083	Integrin cell surface interactions	43/2540	85/10619	8.22E-08	1.14E-05	1.03E-05	THBS1/ICAM1/COL1A2/COL16A1/FBN1/COL4A1/LUM/COL10A1/COL3A1/COL6A3/CD44/COL5A1/COL8A1/COL1A1/ITGB1/ITGA4/TNC/COL5A2/VCAM1/VWF/ITGB3/ICAM4/ITGB2/ITGAM/COL4A2/ITGAD/ITGA5/ITGAL/ITGAX/COL6A2/ITGA1/ITGA9/ITGB8/COL8A2/ITGAV/COMP/ICAM3/ITGA11/SPP1/ITGA2/COL4A3/ICAM5/COL9A3	43

R-HSA-202733	R-HSA-202733	Cell surface interactions at the vascular wall	61/2540	137/10619	8.25E-08	1.14E-05	1.03E-05	SELE/THBD/SHC1/COL1A2/SELL/CD44/COL1A1/SELPLG/ITGB1/CD84/CD48/ITGA4/CEACAM3/TNFRSF10B/SPN/ITGB3/ITGB2/ITGAM/TREM1/ITGA5/CD2/ITGAL/LYN/CD74/INPP5D/FCER1G/SIRPG/ITGAX/SLC7A11/SELP/CD177/PSG1/DOK2/CEACAM8/SDC3/NRAS/TNFRSF10A/SDC4/LCK/PROCR/PSG9/IGLL1/ITGAV/CD244/CD99/PSG7/PF4/SIRPA/VPREB3/PTPN6/VPREB1/TNFRSF10D/SLC7A9/F2/SLC16A1/PROS1/PSG2/PSG8/PSG6/PIK3CA/PSG11	61

b)Functional enrichment analysis data of conserved genes in KIRP

	ID	Description	GeneRatio	BgRatio	pvalue	p.adjust	qvalue	geneID	Count
R-HSA-72766	R-HSA-72766	Translation	30/410	291/10619	8.66E-07	0.0008144	0.000752	EIF4A1/PABPC1/EIF2S1/EIF4B/PPA2/DAP3/MRPL3/IARS2/DARS2/IARS/SSR3/RPL26/EIF2S3/WARS2/MRPS22/EIF2S2/EPRS/SEC11A/GARS/TARS/MARS2/EIF3A/TRAM1/EIF3J/RPS4Y2/ETF1/CARS/SRP72/EIF3C/MRPL42	30
R-HSA-73817	R-HSA-73817	Purine ribonucleoside monophosphate biosynthesis	6/410	12/10619	2.42E-06	0.0010047	0.000927	GMPS/ADSL/PPAT/PAICS/GART/ATIC	6
R-HSA-379724	R-HSA-379724	tRNA Aminoacylation	10/410	42/10619	3.20E-06	0.0010047	0.000927	PPA2/IARS2/DARS2/IARS/WARS2/EPRS/GARS/TARS/MARS2/CARS	10
R-HSA-8956320	R-HSA-8956320	Nucleobase biosynthesis	6/410	15/10619	1.19E-05	0.0027955	0.00258	GMPS/ADSL/PPAT/PAICS/GART/ATIC	6
R-HSA-72649	R-HSA-72649	Translation initiation complex formation	10/410	58/10619	6.54E-05	0.0119254	0.011006	EIF4A1/PABPC1/EIF2S1/EIF4B/EIF2S3/EIF2S2/EIF3A/EIF3J/RPS4Y2/EIF3C	10
R-HSA-72662	R-HSA-72662	Activation of the mRNA upon binding of the cap-binding complex and eIFs, and subsequent binding to 43S	10/410	59/10619	7.60E-05	0.0119254	0.011006	EIF4A1/PABPC1/EIF2S1/EIF4B/EIF2S3/EIF2S2/EIF3A/EIF3J/RPS4Y2/EIF3C	10
R-HSA-379726	R-HSA-379726	Mitochondrial tRNA aminoacylation	6/410	21/10619	0.000106	0.0142149	0.013119	PPA2/IARS2/DARS2/WARS2/GARS/MARS2	6
R-HSA-6791226	R-HSA-6791226	Major pathway of rRNA processing in the nucleolus and cytosol	18/410	185/10619	0.000286	0.0335944	0.031003	WDR43/BYSL/TEX10/GNL3/UTP3/RPL26/PDCD11/WDR12/NCL/NOP58/RCL1/PNO1/HEATR1/UTP18/C1D/RPS4Y2/DDX52/DDX21	18
R-HSA-72702	R-HSA-72702	Ribosomal scanning and start codon recognition	9/410	58/10619	0.000346	0.0362278	0.033434	EIF4A1/EIF2S1/EIF4B/EIF2S3/EIF2S2/EIF3A/EIF3J/RPS4Y2/EIF3C	9
R-HSA-8868773	R-HSA-8868773	rRNA processing in the nucleus and cytosol	18/410	195/10619	0.000541	0.0492815	0.04548	WDR43/BYSL/TEX10/GNL3/UTP3/RPL26/PDCD11/WDR12/NCL/NOP58/RCL1/PNO1/HEATR1/UTP18/C1D/RPS4Y2/DDX52/DDX21	18

c)Functional enrichment analysis data of acquired genes in KIRP

	ID	Description	GeneRatio	BgRatio	pvalue	p.adjust	qvalue	geneID	Count
R-HSA-3000157	R-HSA-3000157	Laminin interactions	23/2929	30/10619	3.10E-08	4.30E-05	3.91E-05	LAMA4/LAMA1/COL4A4/COL4A3/COL4A6/ITGA3/HSPG2/ITGA6/ITGA1/ITGAV/ITGA2/ITGB1/NID1/LAMB3/LAMA2/COL18A1/LAMC1/LAMC2/LAMA5/COL4A5/LAMB2/LAMC3/COL4A1	23
R-HSA-379724	R-HSA-379724	tRNA Aminoacylation	27/2929	42/10619	7.27E-07	0.0005052	0.000459	TARS2/PPA2/TARS/FARSA/IARS2/PPA1/RARS/RARS2/DARS2/YARS2/IARS/WARS2/AARS/MARS2/LARS/EPRS/NARS/GARS/YARS/QARS/AARS2/FARSB/CARS/MARS/SARS/EARS2/WARS	27
R-HSA-9006934	R-HSA-9006934	Signaling by Receptor Tyrosine Kinases	170/2929	458/10619	3.54E-06	0.0016398	0.00149	EPS15L1/MAPK12/ATP6V1B2/VRK3/HPN/CALM3/NEDD4/MAPKAP1/CLTC/CYFIP2/FGF16/BDNF/UBC/FGF3/STAM/DOCK3/NRP1/DNM3/GRIN2B/FGFR2/COL27A1/PSEN1/LAMA4/FGF23/ATP6V1G3/AHCYL1/GAB2/RASA1/S100B/PIK3C3/BRAF/TAB2/TEC/LAMA1/DOCK7/ADAM17/PIK3CA/NCBP1/MTOR/NCKAP1/PPP2R5D/VEGFA/TIAL1/COL4A4/COL4A3/ABI2/PDE3B/RNF41/FGF8/DOCK1/COL9A3/HNRNPF/FLRT1/FRS3/WWP1/STAT1/ATP6V1C1/KL/AKT3/PTPRS/ROCK2/NTRK2/RAB4A/PTK2/INSR/TGFA/ITPR3/HSP90AA1/TRIB3/RALB/PCSK5/RPS6KA5/SPINT1/IGF1R/VAV3/ITGB3/PRKCA/YAP1/ITGA3/ATP6V1A/CTNNA1/DIAPH1/LRIG1/SPRED2/NCK1/PRKCE/BTC/CTNNB1/FGFRL1/PTPN3/CTNND1/ITGAV/ITGA2/ABI1/ITPR1/DNM2/FLT1/ITGB1/IRS2/PAK2/FGF10/NRG4/STAT3/PDPK1/ACTG1/ADCYAP1/KDR/SPRY1/USP8/ADCYAP1R1/ROCK1/LAMB3/KITLG/MAPK3/CDH5/LYN/LAMA2/DUSP7/DUSP4/COL5A3/PCSK6/CYFIP1/LAMC1/LAMC2/ADAM10/WASF3/SPINT2/NRAS/WASF2/ATP6V0E1/CDC42/POLR2B/FURIN/EGFR/YWHAB/LAMA5/PTPRU/ATP6V1H/COL4A5/PDGFC/MAPK14/SH3KBP1/LAMB2/THBS1/COL6A6/POLR2D/PPP2CB/RAPGEF1/BAX/PIK3R4/NOS3/CBL/CRK/CUL5/SHC3/RPS6KA2/LAMC3/GRB2/THBS4/VAV2/FER/ITCH/AP2A1/COL4A1/ATP6AP1/PLG/RPS6KA3/NRG3/POLR2A/NRG2	170
R-HSA-8852135	R-HSA-8852135	Protein ubiquitination	40/2929	79/10619	1.11E-05	0.003144	0.002857	VCP/UBE2R2/RRAGA/RNF152/UBC/RAD18/BCL10/UBE2A/UBE2D1/UBE2V2/CDC73/PEX5/UBE2G1/UBE2H/HIST1H2BE/HIST1H2BC/PEX2/USP7/WAC/UBE2W/UBE2K/DERL1/HIST1H2BK/HIST1H2BJ/RTF1/CTR9/UBE2D3/PRKDC/LEO1/RNF20/UBE2Z/UBA1/PEX13/HIST1H2BN/UBE2D2/HLTF/UBE2E1/UBE2T/PCNA/SHPRH	40
R-HSA-379716	R-HSA-379716	Cytosolic tRNA aminoacylation	17/2929	24/10619	1.28E-05	0.003144	0.002857	TARS/FARSA/PPA1/RARS/IARS/AARS/LARS/EPRS/NARS/GARS/YARS/QARS/FARSB/CARS/MARS/SARS/WARS	17
R-HSA-3000171	R-HSA-3000171	Non-integrin membrane-ECM interactions	32/2929	59/10619	1.36E-05	0.003144	0.002857	LAMA4/LAMA1/COL4A4/COL4A3/COL4A6/SDC1/TGFB1/ITGB3/PRKCA/HSPG2/ITGA6/ITGB5/AGRN/ITGAV/ITGA2/ACTN1/ITGB1/LAMB3/LAMA2/COL5A3/LAMC1/LAMC2/SDC2/LAMA5/COL4A5/LAMB2/THBS1/DAG1/LAMC3/NTN4/CASK/COL4A1	32
R-HSA-8866654	R-HSA-8866654	E3 ubiquitin ligases ubiquitinate target proteins	31/2929	59/10619	4.23E-05	0.0083875	0.007621	VCP/RRAGA/RNF152/UBC/RAD18/BCL10/UBE2A/UBE2D1/UBE2V2/CDC73/PEX5/HIST1H2BE/HIST1H2BC/PEX2/WAC/DERL1/HIST1H2BK/HIST1H2BJ/RTF1/CTR9/UBE2D3/PRKDC/LEO1/RNF20/PEX13/HIST1H2BN/UBE2D2/HLTF/UBE2E1/PCNA/SHPRH	31

R-HSA-6811442	R-HSA-6811442	Intra-Golgi and retrograde Golgi-to-ER traffic	79/2929	202/10619	0.000224	0.0388597	0.03531	KIF1A/RABEPK/DYNLL2/DCTN6/TUBB2B/VTI1A/KIF2A/TUBA1A/ACTR10/TUBB2A/SEC22B/KIF4B/DYNC1LI1/RAB9B/VAMP3/NAA35/RAB9A/COPB2/COPB1/NAA38/GBF1/TUBB8/KIF6/GCC2/USP6NL/RAB3GAP1/DYNC1I2/RAB3GAP2/VPS54/TUBA3E/KIF18A/CAPZA2/ARF4/PAFAH1B2/BICD1/PLIN3/TUBA4A/IGF2R/RAB30/RGP1/SURF4/MAN2A1/TGOLN2/RAB6A/KIF3C/NSF/ZW10/ARCN1/MAN1A2/TMF1/TMED3/RAB43/RAB1A/TMED7-TICAM2/RAB18/GOLGA4/TMED2/KIF4A/KDELR1/GOLIM4/STX5/KIF11/MAN1A1/STX6/RACGAP1/KLC1/KIF20B/GCC1/GALNT2/KIF23/AGPAT3/MAN2A2/GOSR1/KIF18B/KIF13B/ALPP/KIF2C/KIF1B/RAB6B	79
R-HSA-195258	R-HSA-195258	RHO GTPase Effectors	119/2929	327/10619	0.000259	0.0400213	0.036365	CALM3/DYNLL2/CYFIP2/DIAPH3/TUBB2B/KIF2A/TUBA1A/TUBB2A/CKAP5/DYNC1LI1/HIST1H4L/PFN2/RHOB/PPP2R5E/CLASP2/FMNL2/PIK3C3/MYLK/CLIP1/SEC13/TUBB8/NCKAP1/PPP2R5D/CENPQ/ABI2/NUP98/ACTR3/KLK2/DYNC1I2/PPP2R5C/KLK3/PPP2R5A/SRF/NUP133/BUB3/BUB1/RANBP2/AHCTF1/DIAPH2/TUBA3E/KIF18A/SPC24/ROCK2/YWHAQ/PTK2/YWHAG/HIST1H2BE/HIST1H2BC/IQGAP1/TUBA4A/KTN1/SFN/HIST2H2BE/PRKCA/H2AFZ/CTNNA1/DIAPH1/NCK1/RHOC/CTNNB1/ZW10/PRC1/ABI1/ITGB1/PAK2/FLNA/ABL1/HIST3H2BB/YWHAZ/PDPK1/ACTG1/HIST1H2BK/HIST1H2BJ/ACTR2/PPP1R12A/MAD2L1/ROCK1/PPP1CB/HIST2H2AA3/RHPN2/MAPK3/NCOA2/HIST2H3D/HIST2H4A/ARPC2/MAPRE1/HIST1H2AC/RHOD/SKA2/CYFIP1/HIST1H3H/WIPF2/WASF3/WASF2/CDC42/BUB1B/NDE1/KLC1/YWHAB/INCENP/TAOK1/HIST1H2BN/CENPI/MAPK14/PPP2CB/PIK3R4/MYH10/PLK1/HIST4H4/KDM1A/GRB2/HIST1H4H/CDCA8/ZWINT/CENPA/IQGAP3/CLASP1/KIF2C/ERCC6L	119
